# Supplementary material for: Systematic Modeling of Risk-Associated Copy Number Alterations in Cancer
Source: Int J Mol Sci. 2024 Sep 27;25(19):10455. doi: 10.3390/ijms251910455 (PMC11477427; doi:10.3390/ijms251910455)
Supplement: Supplementary file 1 [file ijms-25-10455-s001.zip › Supp File 1 - Model-Signatures.docx]

# Prognostic cancer signatures from copy number data

Alejandra Guardado, Raúl Aguirre-Gamboa, and Victor Treviño

## Supplementary File 1

Content

- Supplementary Table 1
- Supplementary Figure 1
- Supplementary Model Files

**Supplementary Table S1.** Cancer types and samples used.

| CANCER | CANCER | Initial Samples | Used Samples | Censoring (%) |
| --- | --- | --- | --- | --- |
| ACC | Adrenocortical carcinoma | 92 | 89 | 67 |
| BLCA | Bladder Urothelial Carcinoma | 412 | 398 | 73 |
| BRCA | Breast invasive carcinoma | 1097 | 1025 | 90 |
| CESC | Cervical squamous cell carcinoma and endocervical adenocarcinoma | 307 | 277 | 80 |
| CHOL | Cholangiocarcinoma | 45 | 35 | 54 |
| COAD | Colon adenocarcinoma | 458 | 345 | 85 |
| COADREAD | Colon adenocarcinoma & Rectum adenocarcinoma | 629 | 469 | 87 |
| DLBC | Lymphoid Neoplasm Diffuse Large B-cell Lymphoma | 48 | 46 | 89 |
| ESCA | Esophageal carcinoma | 185 | 162 | 65 |
| GBM | Glioblastoma multiforme | 595 | 572 | 25 |
| GBMLGG | Glioblastoma multiforme & Brain Lower Grade Glioma | 1110 | 1080 | 52 |
| HNSC | Head and Neck squamous cell carcinoma | 528 | 517 | 68 |
| KICH | Kidney Chromophobe | 113 | 65 | 88 |
| KIPAN | Kidney carcinomas (KICH, KIRC, KIRP) | 941 | 853 | 77 |
| KIRC | Kidney renal clear cell carcinoma | 537 | 520 | 69 |
| KIRP | Kidney renal papillary cell carcinoma | 291 | 268 | 88 |
| LAML | Acute Myeloid Leukemia | 200 | 168 | 38 |
| LGG | Brain Lower Grade Glioma | 515 | 508 | 82 |
| LIHC | Liver hepatocellular carcinoma | 377 | 338 | 73 |
| LUAD | Lung adenocarcinoma | 522 | 478 | 74 |
| LUSC | Lung squamous cell carcinoma | 504 | 478 | 67 |
| MESO | Mesothelioma | 87 | 86 | 34 |
| OV | Ovarian serous cystadenocarcinoma | 591 | 559 | 49 |
| PAAD | Pancreatic adenocarcinoma | 185 | 180 | 63 |
| PCPG | Pheochromocytoma and Paraganglioma | 179 | 162 | 96 |
| PRAD | Prostate adenocarcinoma | 499 | 492 | 98 |
| READ | Rectum adenocarcinoma | 171 | 124 | 93 |
| SARC | Sarcoma | 261 | 254 | 70 |
| SKCM | Skin Cutaneous Melanoma | 470 | 358 | 60 |
| STAD | Stomach adenocarcinoma | 443 | 394 | 79 |
| STES | Stomach adenocarcinoma & Esophageal carcinoma | 628 | 556 | 75 |
| TGCT | Testicular Germ Cell Tumors | 134 | 133 | 98 |
| THCA | Thyroid carcinoma | 503 | 495 | 97 |
| THYM | Thymoma | 124 | 121 | 95 |
| UCEC | Uterine Corpus Endometrial Carcinoma | 548 | 535 | 92 |
| UCS | Uterine Carcinosarcoma | 57 | 55 | 44 |
| UVM | Uveal Melanoma | 80 | 80 | 84 |
|  | **Total** | **14466** | **13275** |  |
|  | Unique | 11158 | 10317 |  |

**Supplementary Figure S1.** Comparison of the number of soft and deep alterations associated to survival. Each symbol represents a chromosome in a cancer type. The left panel shows the absolute number of alterations. The center panel shows the number of alterations+1 in logarithm base 10 scale. The right panel shows the fraction of the alterations relative to all alterations associated to survival.

**Supplementary Model Files**

As described in the main manuscript, our methodology generates up to 8 copy-number prognostic models per cancer type. These signatures correspond to: (1) soft amplifications (named as “all amplifications”), (2) soft deletions (named as “all deletions”), (3) Max-Sum of soft deletions and soft amplifications, (4) Combinations of soft deletions and soft amplifications, (5) deep amplification, (6) deep deletions, (7) Max-Sum of deep deletions and deep amplifications, and (8) Combinations of deep deletions and deep amplifications.

We systematically generated these 8 signatures and generated a file per cancer type to provide further details. An example is shown below for LUSC for the Max-Sum model of deep data. These files contain, a simplified view of the input data for each signature (only 1 representative gene-region per cytoband) followed by the Kaplan-Meier curves of the prognostic model together a multivariate-cox-model evaluation of the risk groups.

We archived all files in a compressed file submitted as “Cancer-Signatures_v12.zip”, which contains the following files.

| Cancer | Filename |
| --- | --- |
| ACC | ACCSignatureV12-sinSombreado.pdf |
| BLCA | BLCASignatureV12-sinSombreado.pdf |
| BRCA | BRCASignatureV12-sinSombreado.pdf |
| CESC | CESCSignatureV12-sinSombreado.pdf |
| CHOL | CHOLSignatureV12-sinSombreado.pdf |
| COADREAD | COADREADSignatureV12-sinSombreado.pdf |
| COAD | COADSignatureV12-sinSombreado.pdf |
| DLBC | DLBCSignatureV12-sinSombreado.pdf |
| ESCA | ESCASignatureV12-sinSombreado.pdf |
| GBMLGG | GBMLGGSignatureV12-sinSombreado.pdf |
| GBM | GBMSignatureV12-sinSombreado.pdf |
| HNSC | HNSCSignatureV12-sinSombreado.pdf |
| KICH | KICHSignatureV12-sinSombreado.pdf |
| KIPAN | KIPANSignatureV12-sinSombreado.pdf |
| KIRC | KIRCSignatureV12-sinSombreado.pdf |
| KIRP | KIRPSignatureV12-sinSombreado.pdf |
| LAML | LAMLSignatureV12-sinSombreado.pdf |
| LGG | LGGSignatureV12-sinSombreado.pdf |
| LIHC | LIHCSignatureV12-sinSombreado.pdf |
| LUAD | LUADSignatureV12-sinSombreado.pdf |
| LUSC | LUSCSignatureV12-sinSombreado.pdf |
| MESO | MESOSignatureV12-sinSombreado.pdf |
| OV | OVSignatureV12-sinSombreado.pdf |
| PAAD | PAADSignatureV12-sinSombreado.pdf |
| PCPG | PCPGSignatureV12-sinSombreado.pdf |
| PRAD | PRADSignatureV12-sinSombreado.pdf |
| READ | READSignatureV12-sinSombreado.pdf |
| SARC | SARCSignatureV12-sinSombreado.pdf |
| SKCM | SKCMSignatureV12-sinSombreado.pdf |
| STAD | STADSignatureV12-sinSombreado.pdf |
| STES | STESSignatureV12-sinSombreado.pdf |
| TGCT | TGCTSignatureV12-sinSombreado.pdf |
| THCA | THCASignatureV12-sinSombreado.pdf |
| THYM | THYMSignatureV12-sinSombreado.pdf |
| UCEC | UCECSignatureV12-sinSombreado.pdf |
| UCS | UCSSignatureV12-sinSombreado.pdf |
| UVM | UVMSignatureV12-sinSombreado.pdf |
