# Supplementary material for: Systematic Modeling of Risk-Associated Copy Number Alterations in Cancer
Source: Int J Mol Sci. 2024 Sep 27;25(19):10455. doi: 10.3390/ijms251910455 (PMC11477427; doi:10.3390/ijms251910455)
Supplement: Supplementary file 1 [file ijms-25-10455-s001.zip › LUSCSignatureV12-sinSombreado.pdf]

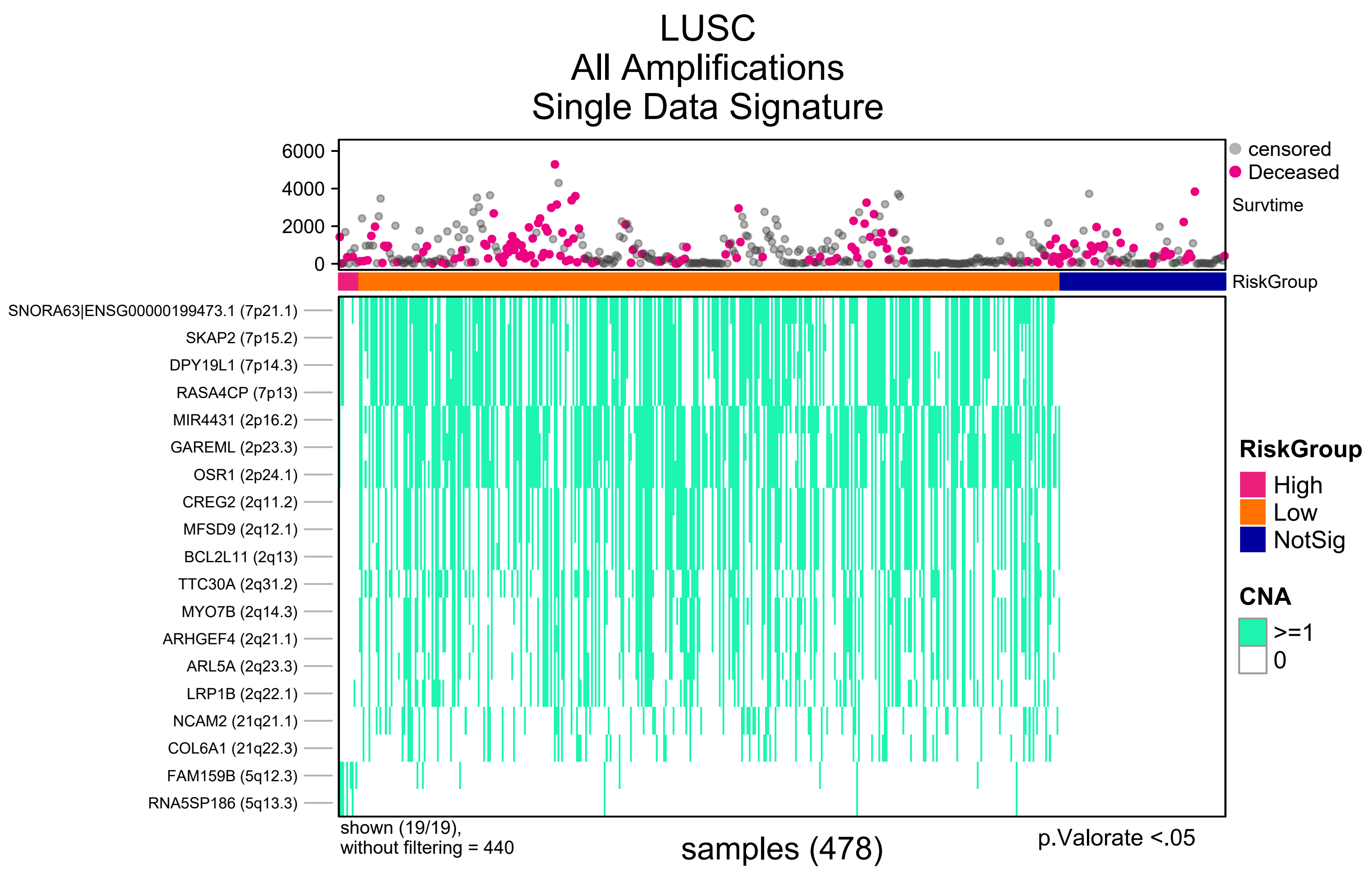
LUSC
All Amplifications
Single Data Signature
6000
4000
2000
0
censored
Deceased
Survtime
RiskGroup
SNORA63|ENSG00000199473.1 (7p21.1)
SKAP2 (7p15.2)
DPY19L1 (7p14.3)
RASA4CP (7p13)
MIR4431 (2p16.2)
GAREML (2p23.3)
OSR1 (2p24.1)
CREG2 (2q11.2)
MFSD9 (2q12.1)
BCL2L11 (2q13)
TTC30A (2q31.2)
MYO7B (2q14.3)
ARHGEF4 (2q21.1)
ARL5A (2q23.3)
LRP1B (2q22.1)
NCAM2 (21q21.1)
COL6A1 (21q22.3)
FAM159B (5q12.3)
RNA5SP186 (5q13.3)
RiskGroup
High
Low
NotSig
CNA
>=1
0
shown (19/19),
without filtering = 440
samples (478)
p.Valorate <.05

LUSC
All Amplifications
Single Data Signature

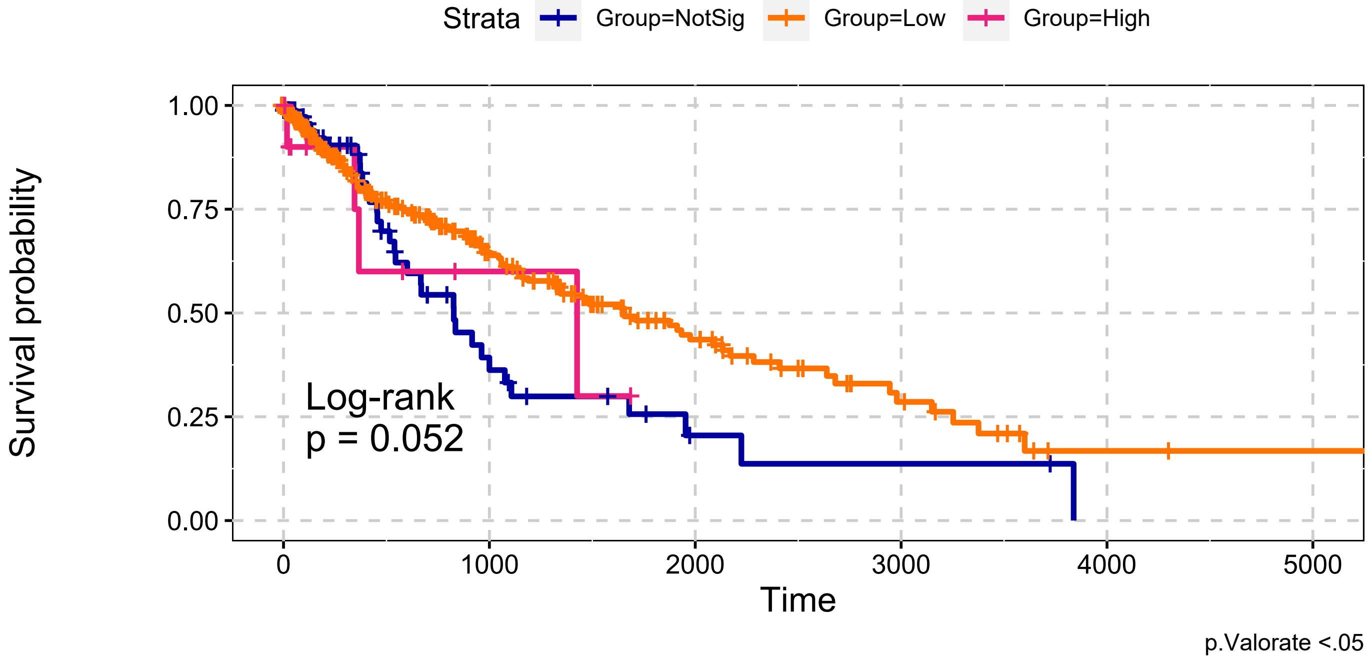

explanatory	beta	HR	L95	U95	p
Low	-0.46	0.63	0.43	0.93	0.02
High	0.01	1.01	0.36	2.86	0.98

n= 478, number of events =156
Score(logrank) test = 0.052

Number at risk

	0	1000	2000	3000	4000	5000
Group=NotSig	88	13	3	2	0	0
Group=Low	375	96	38	13	2	1
Group=High	11	2	0	0	0	0

p.Valorate <.05

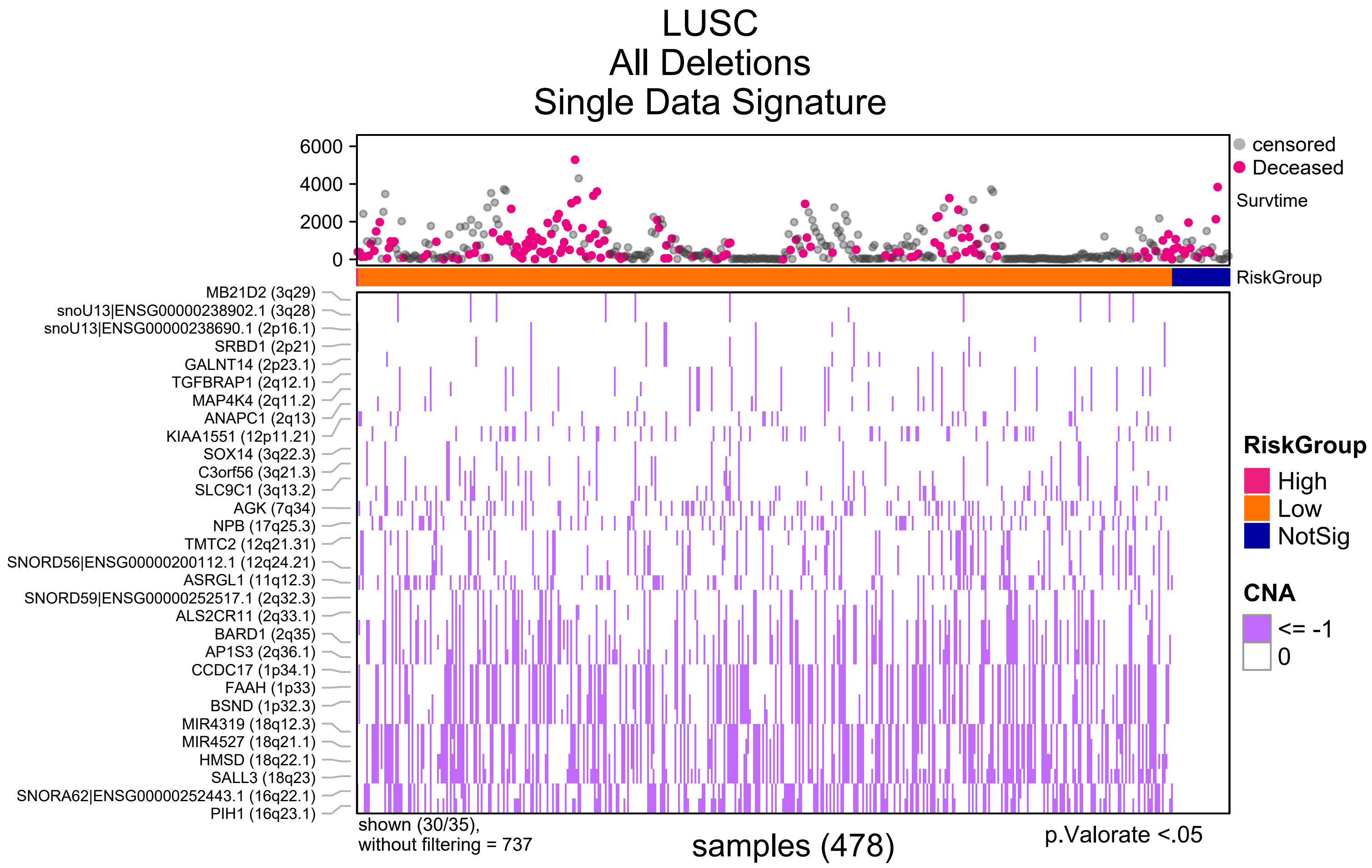
LUSC
All Deletions
Single Data Signature
6000
4000
2000
0
censored
Deceased
Survtime
RiskGroup
MB21D2 (3q29)
snoU13|ENSG00000238902.1 (3q28)
snoU13|ENSG00000238690.1 (2p16.1)
SRBD1 (2p21)
GALNT14 (2p23.1)
TGFBRAP1 (2q12.1)
MAP4K4 (2q11.2)
ANAPC1 (2q13)
KIAA1551 (12p11.21)
SOX14 (3q22.3)
C3orf56 (3q21.3)
SLC9C1 (3q13.2)
AGK (7q34)
NPB (17q25.3)
TMTC2 (12q21.31)
SNORD56|ENSG00000200112.1 (12q24.21)
ASRGL1 (11q12.3)
SNORD59|ENSG00000252517.1 (2q32.3)
ALS2CR11 (2q33.1)
BARD1 (2q35)
AP1S3 (2q36.1)
CCDC17 (1p34.1)
FAAH (1p33)
BSND (1p32.3)
MIR4319 (18q12.3)
MIR4527 (18q21.1)
HMSD (18q22.1)
SALL3 (18q23)
SNORA62|ENSG00000252443.1 (16q22.1)
PIH1 (16q23.1)
RiskGroup
High
Low
NotSig
CNA
<= -1
0
shown (30/35),
without filtering = 737
samples (478)
p.Valorate <.05

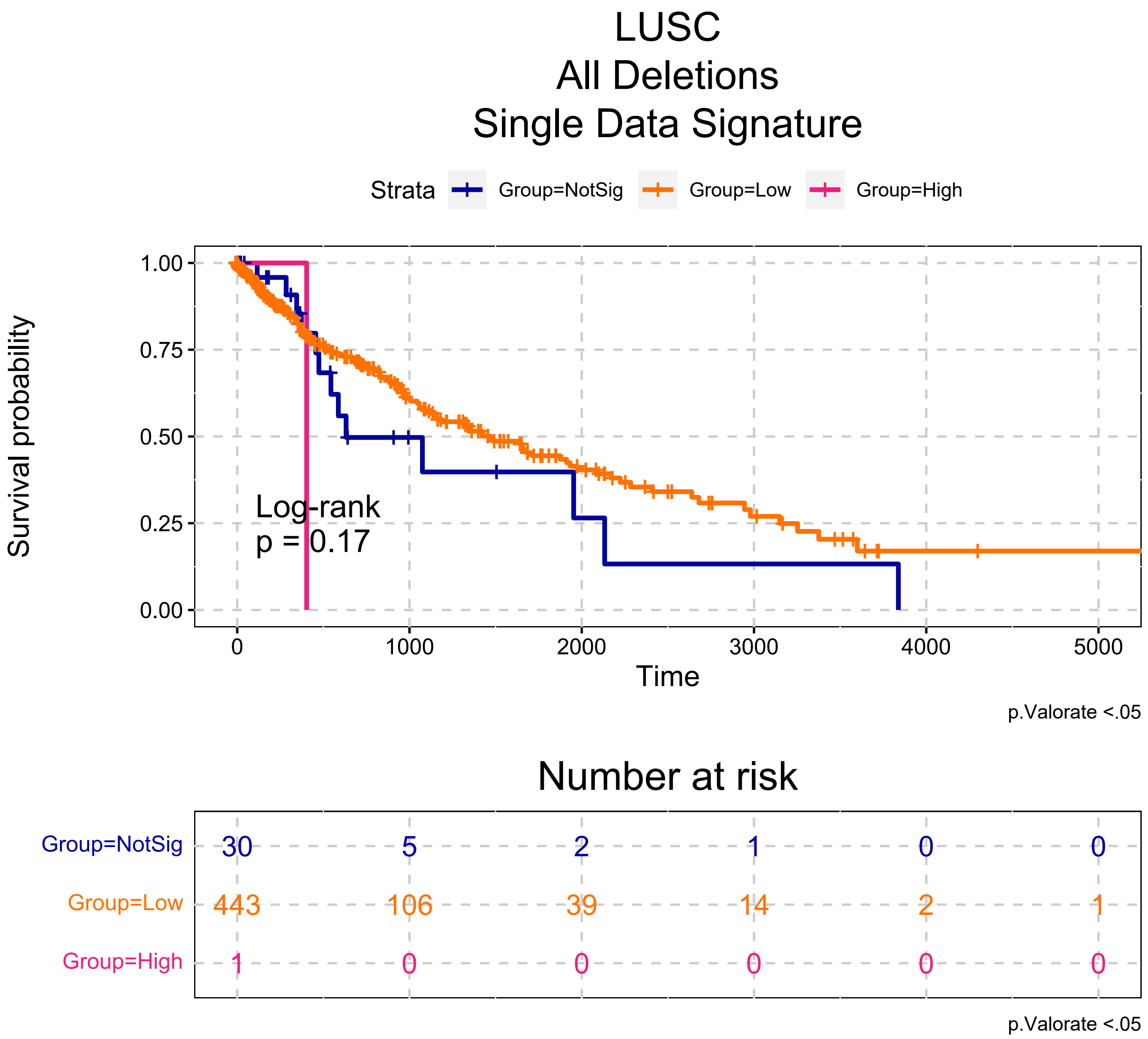

explanatory	beta	HR	L95	U95	p
Low	-0.31	0.73	0.41	1.30	0.28
High	1.15	3.17	0.41	24.39	0.27

n= 478, number of events =156
Score(logrank) test = 0.167

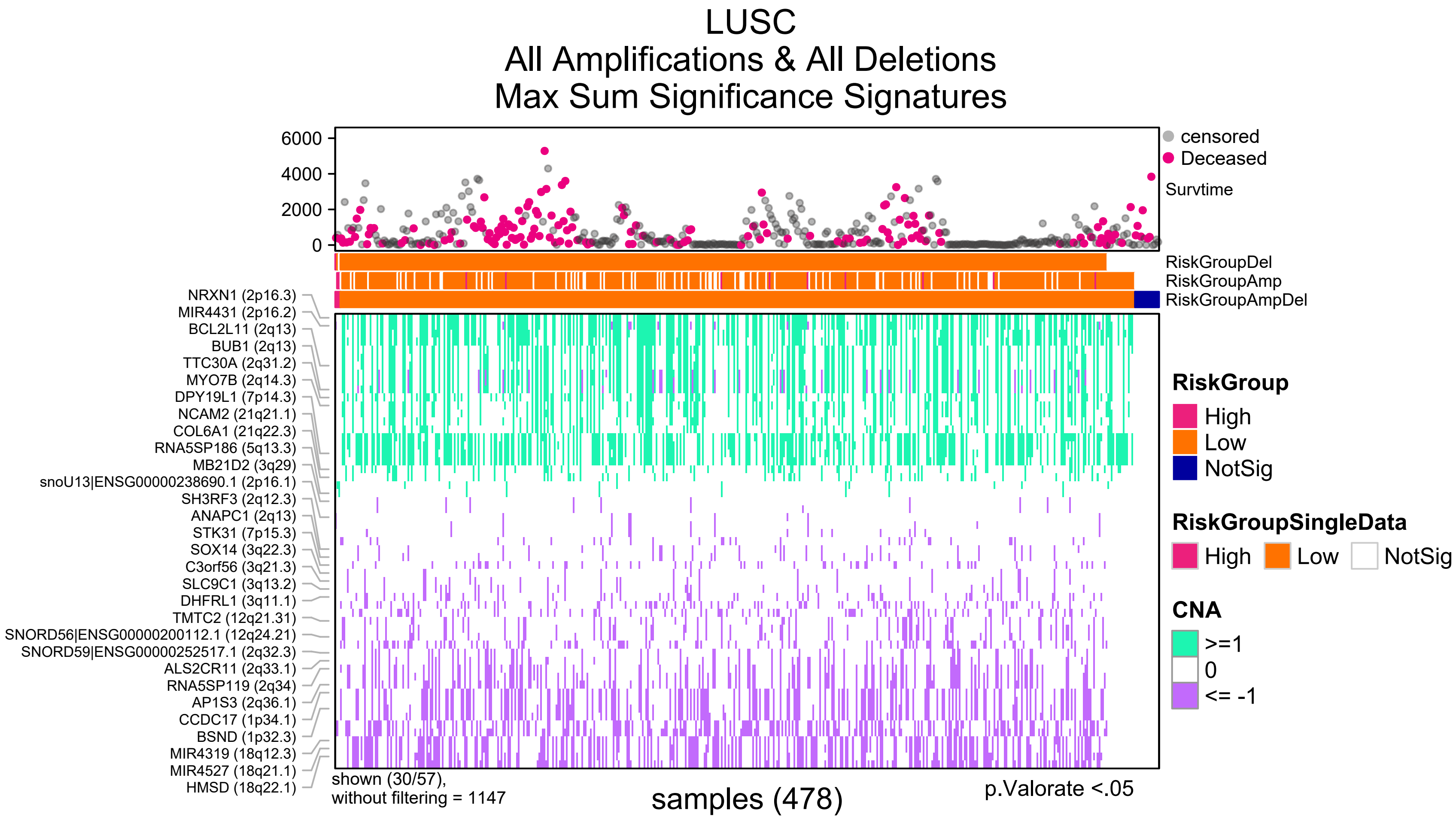

LUSC
All Amplifications & All Deletions
Max Sum Significance Signatures
6000
4000
2000
0
censored
Deceased
Survtime
RiskGroupDel
RiskGroupAmp
RiskGroupAmpDel
NRXN1 (2p16.3)
MIR4431 (2p16.2)
BCL2L11 (2q13)
BUB1 (2q13)
TTC30A (2q31.2)
MYO7B (2q14.3)
DPY19L1 (7p14.3)
NCAM2 (21q21.1)
COL6A1 (21q22.3)
RNA5SP186 (5q13.3)
MB21D2 (3q29)
snoU13|ENSG00000238690.1 (2p16.1)
SH3RF3 (2q12.3)
ANAPC1 (2q13)
STK31 (7p15.3)
SOX14 (3q22.3)
C3orf56 (3q21.3)
SLC9C1 (3q13.2)
DHFRL1 (3q11.1)
TMTC2 (12q21.31)
SNORD56|ENSG00000200112.1 (12q24.21)
SNORD59|ENSG00000252517.1 (2q32.3)
ALS2CR11 (2q33.1)
RNA5SP119 (2q34)
AP1S3 (2q36.1)
CCDC17 (1p34.1)
BSND (1p32.3)
MIR4319 (18q12.3)
MIR4527 (18q21.1)
HMSD (18q22.1)
shown (30/57),
without filtering = 1147
samples (478)
p.Valorate <.05
RiskGroup
High
Low
NotSig
RiskGroupSingleData
High
Low
NotSig
CNA
>=1
0
<= -1

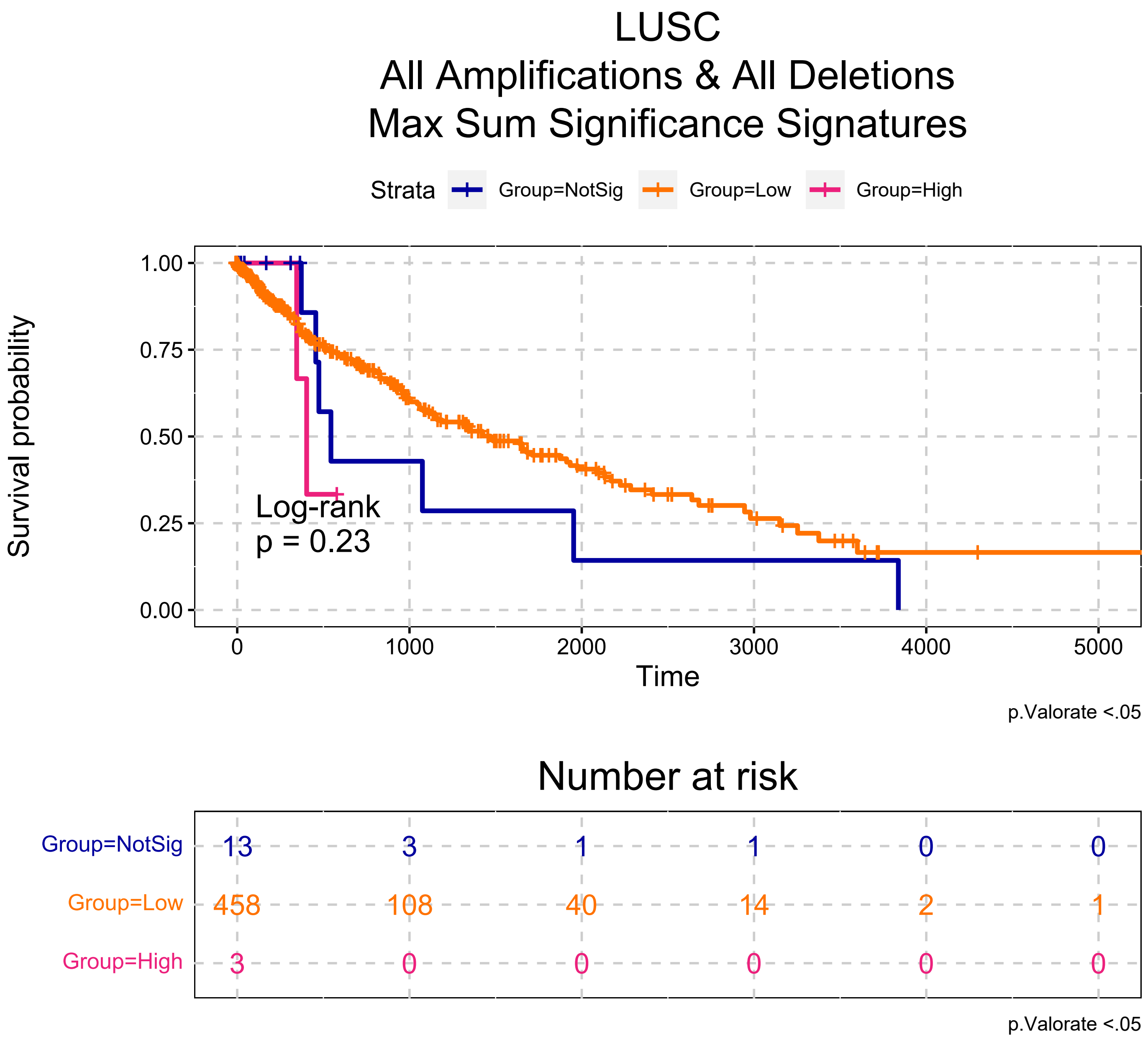

explanatory	beta	HR	L95	U95	p
Low	-0.34	0.71	0.33	1.54	0.39
High	0.69	1.98	0.41	9.68	0.40

n= 478, number of events =156
Score(logrank) test = 0.233

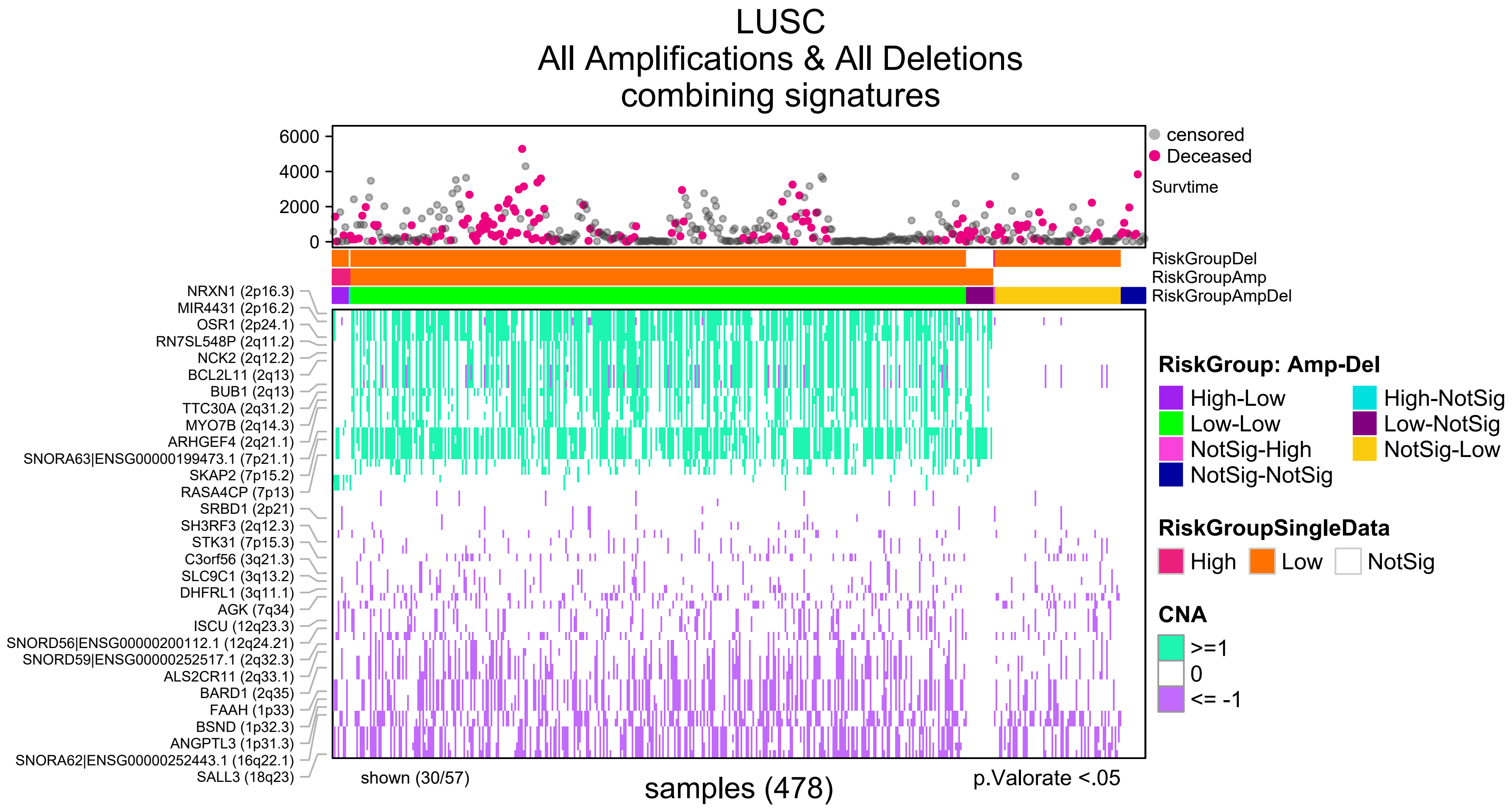

LUSC
All Amplifications & All Deletions
combining signatures
6000
4000
2000
0
censored
Deceased
Survtime
RiskGroupDel
RiskGroupAmp
RiskGroupAmpDel
NRXN1 (2p16.3)
MIR4431 (2p16.2)
OSR1 (2p24.1)
RN7SL548P (2q11.2)
NCK2 (2q12.2)
BCL2L11 (2q13)
BUB1 (2q13)
TTC30A (2q31.2)
MYO7B (2q14.3)
ARHGEF4 (2q21.1)
SNORA63|ENSG00000199473.1 (7p21.1)
SKAP2 (7p15.2)
RASA4CP (7p13)
SRBD1 (2p21)
SH3RF3 (2q12.3)
STK31 (7p15.3)
C3orf56 (3q21.3)
SLC9C1 (3q13.2)
DHFRL1 (3q11.1)
AGK (7q34)
ISCU (12q23.3)
SNORD56|ENSG00000200112.1 (12q24.21)
SNORD59|ENSG00000252517.1 (2q32.3)
ALS2CR11 (2q33.1)
BARD1 (2q35)
FAAH (1p33)
BSND (1p32.3)
ANGPTL3 (1p31.3)
SNORA62|ENSG00000252443.1 (16q22.1)
SALL3 (18q23)
shown (30/57)
samples (478)
p.Valorate <.05
RiskGroup: Amp-Del
High-Low
Low-Low
NotSig-High
NotSig-NotSig
High-NotSig
Low-NotSig
NotSig-Low
RiskGroupSingleData
High
Low
NotSig
CNA
>=1
0
<= -1

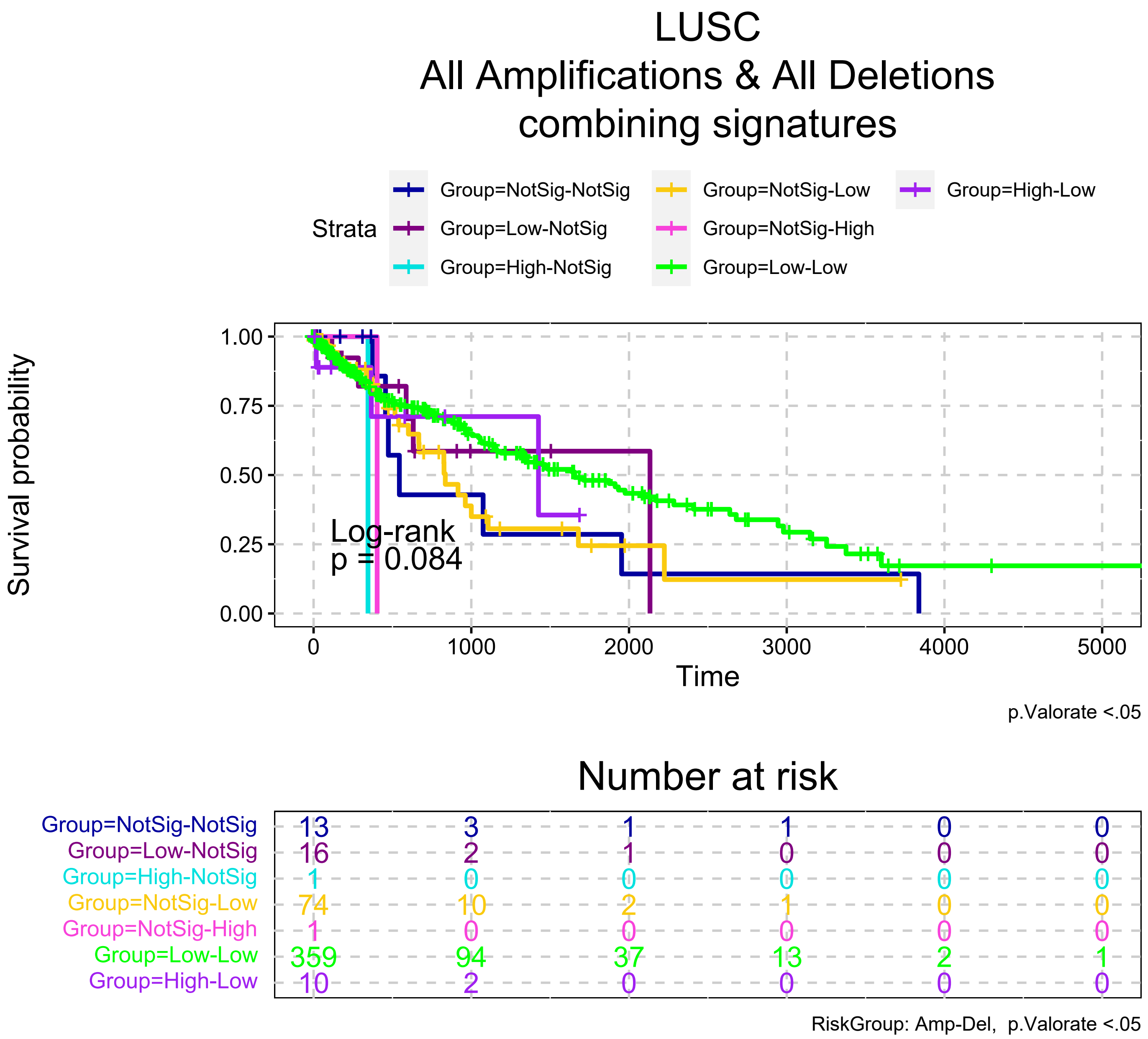

**explanatory**	**beta**	**HR**	**L95**	**U95**	**p**
Low-NotSig	-0.20	0.82	0.26	2.61	0.74
High-NotSig	1.42	4.15	0.50	34.24	0.19
NotSig-Low	0.04	1.04	0.45	2.44	0.92
NotSig-High	1.15	3.14	0.38	25.88	0.29
Low-Low	-0.41	0.66	0.30	1.43	0.29
High-Low	-0.16	0.85	0.22	3.34	0.82

n= 478, number of events =156
Score(logrank) test = 0.083

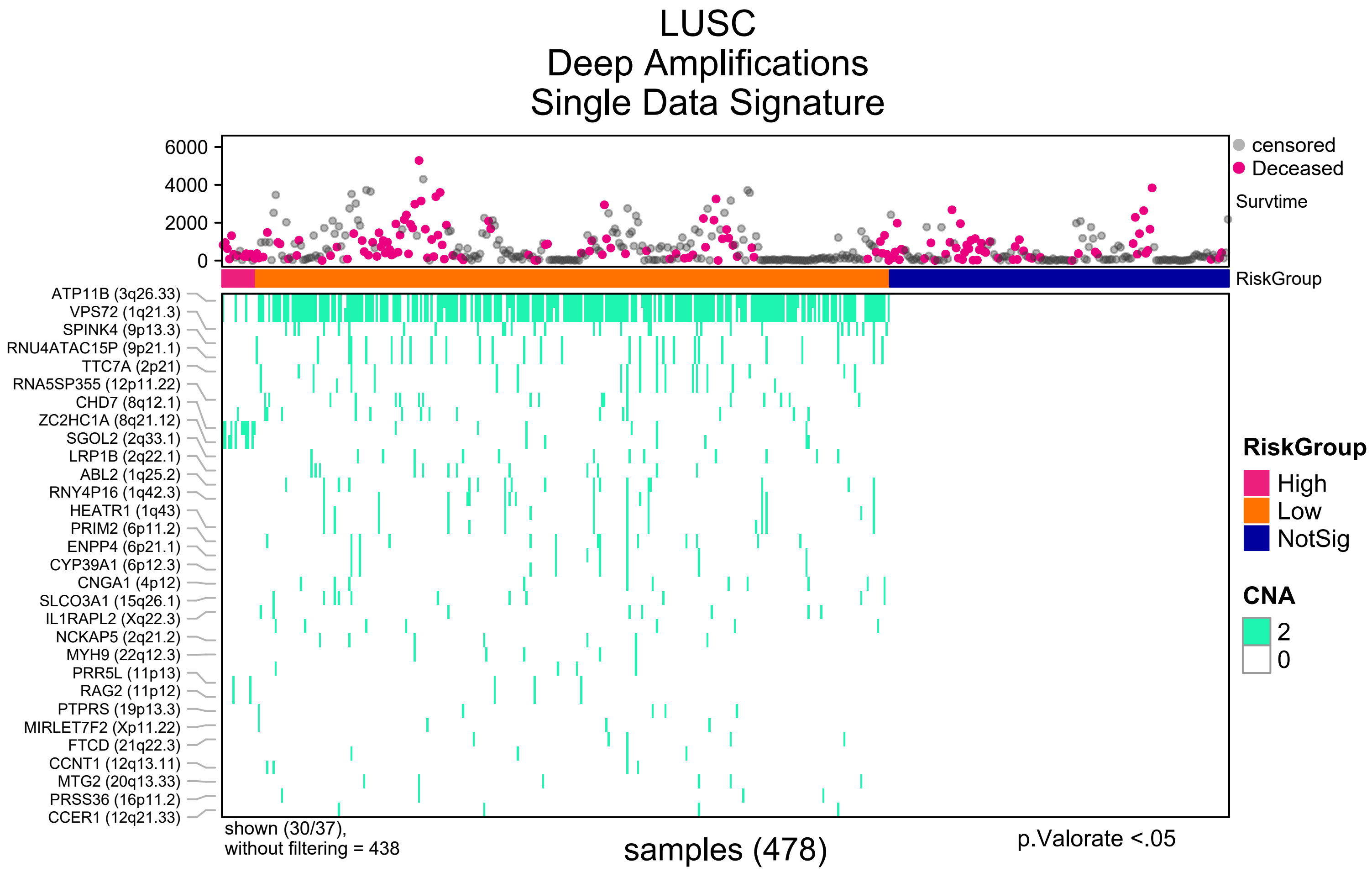
LUSC
Deep Amplifications
Single Data Signature
6000
4000
2000
0
censored
Deceased
Survtime
RiskGroup
ATP11B (3q26.33)
VPS72 (1q21.3)
SPINK4 (9p13.3)
RNU4ATAC15P (9p21.1)
TTC7A (2p21)
RNA5SP355 (12p11.22)
CHD7 (8q12.1)
ZC2HC1A (8q21.12)
SGOL2 (2q33.1)
LRP1B (2q22.1)
ABL2 (1q25.2)
RNY4P16 (1q42.3)
HEATR1 (1q43)
PRIM2 (6p11.2)
ENPP4 (6p21.1)
CYP39A1 (6p12.3)
CNGA1 (4p12)
SLCO3A1 (15q26.1)
IL1RAPL2 (Xq22.3)
NCKAP5 (2q21.2)
MYH9 (22q12.3)
PRR5L (11p13)
RAG2 (11p12)
PTPRS (19p13.3)
MIRLET7F2 (Xp11.22)
FTCD (21q22.3)
CCNT1 (12q13.11)
MTG2 (20q13.33)
PRSS36 (16p11.2)
CCER1 (12q21.33)
RiskGroup
High
Low
NotSig
CNA
2
0
shown (30/37),
without filtering = 438
samples (478)
p.Valorate <.05

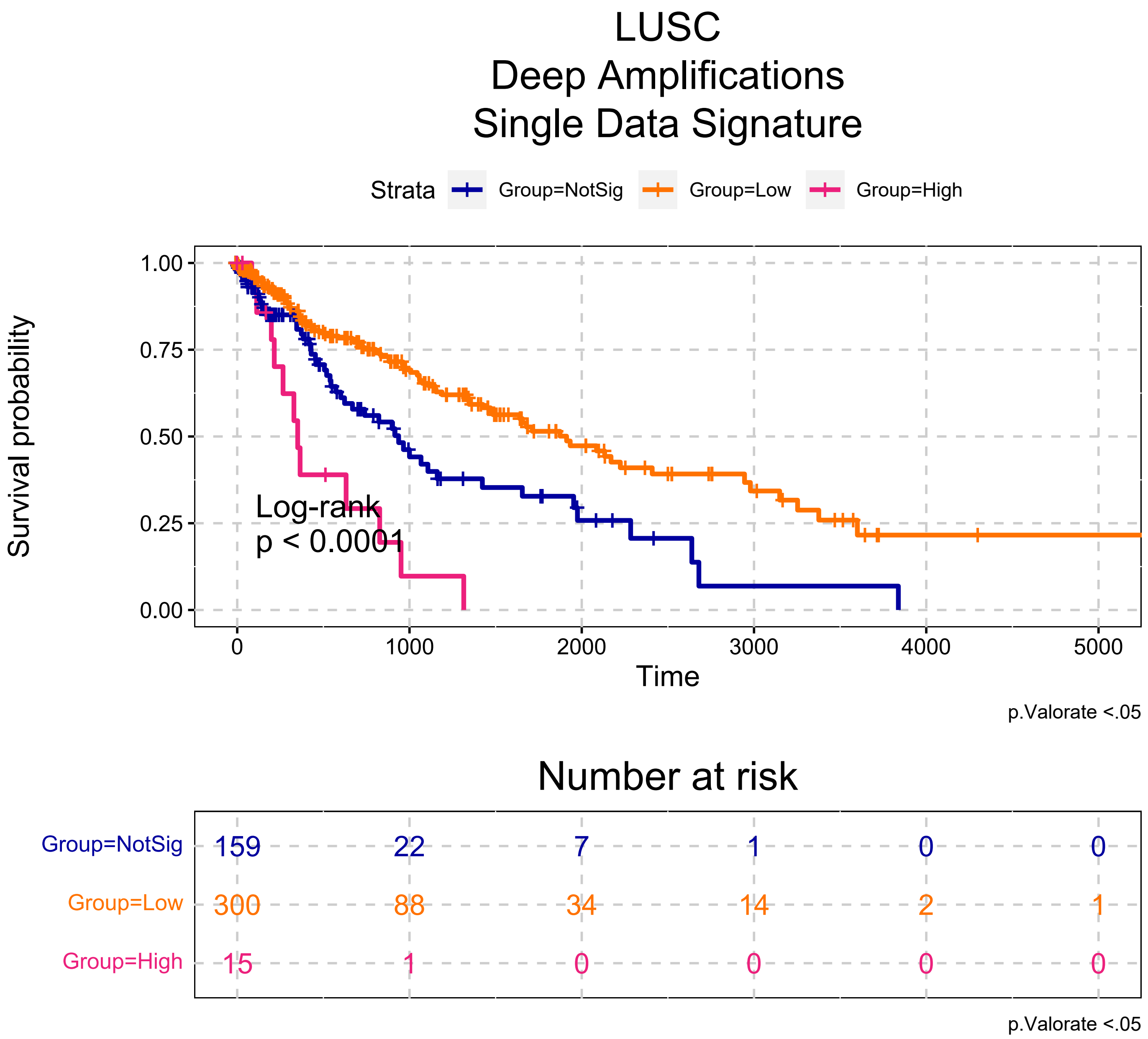

explanatory	beta	HR	L95	U95	p
Low	-0.63	0.53	0.38	0.75	0.00
High	0.96	2.62	1.39	4.93	0.00

n= 478, number of events =156
Score(logrank) test = p <.0001

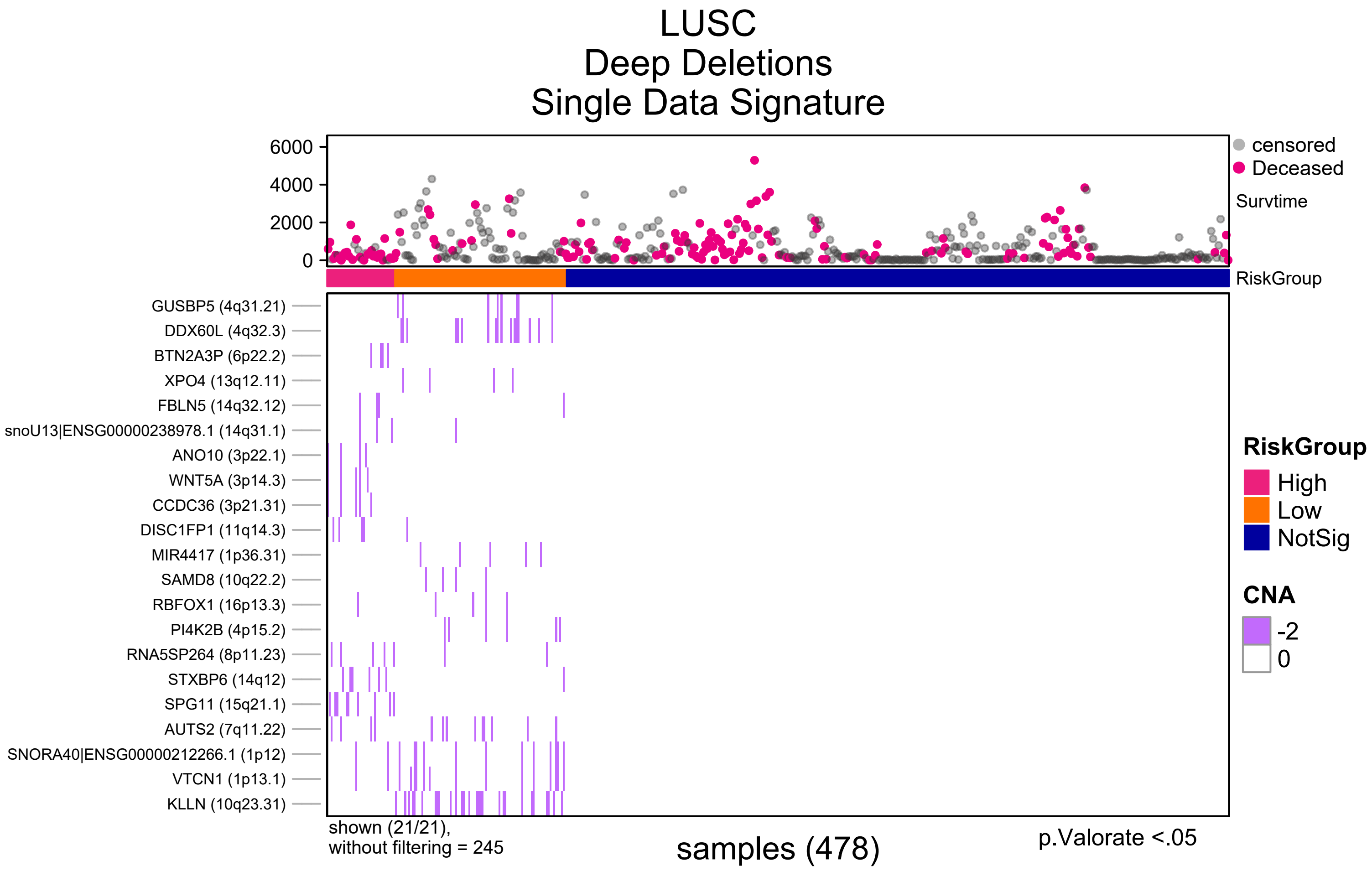

LUSC
Deep Deletions
Single Data Signature
6000
4000
2000
0
censored
Deceased
Survtime
RiskGroup
GUSBP5 (4q31.21)
DDX60L (4q32.3)
BTN2A3P (6p22.2)
XPO4 (13q12.11)
FBLN5 (14q32.12)
snoU13|ENSG00000238978.1 (14q31.1)
ANO10 (3p22.1)
WNT5A (3p14.3)
CCDC36 (3p21.31)
DISC1FP1 (11q14.3)
MIR4417 (1p36.31)
SAMD8 (10q22.2)
RBFOX1 (16p13.3)
PI4K2B (4p15.2)
RNA5SP264 (8p11.23)
STXBP6 (14q12)
SPG11 (15q21.1)
AUTS2 (7q11.22)
SNORA40|ENSG00000212266.1 (1p12)
VTCN1 (1p13.1)
KLLN (10q23.31)
RiskGroup
High
Low
NotSig
CNA
-2
0
shown (21/21),
without filtering = 245
samples (478)
p.Valorate <.05

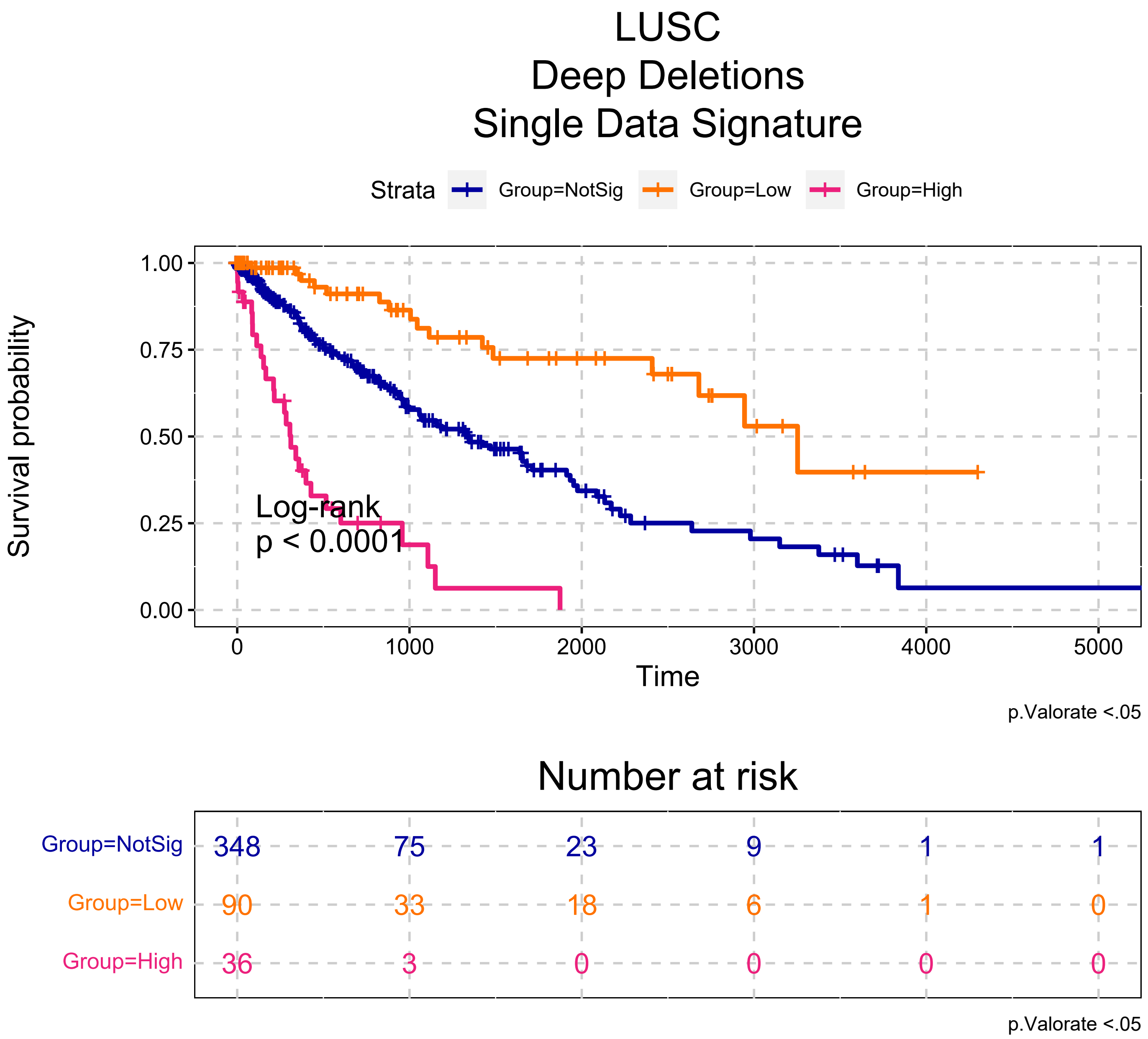

explanatory	beta	HR	L95	U95	p
Low	-1.10	0.33	0.20	0.57	0.00
High	1.39	4.01	2.60	6.18	0.00

n= 478, number of events =156
Score(logrank) test = p <.0001

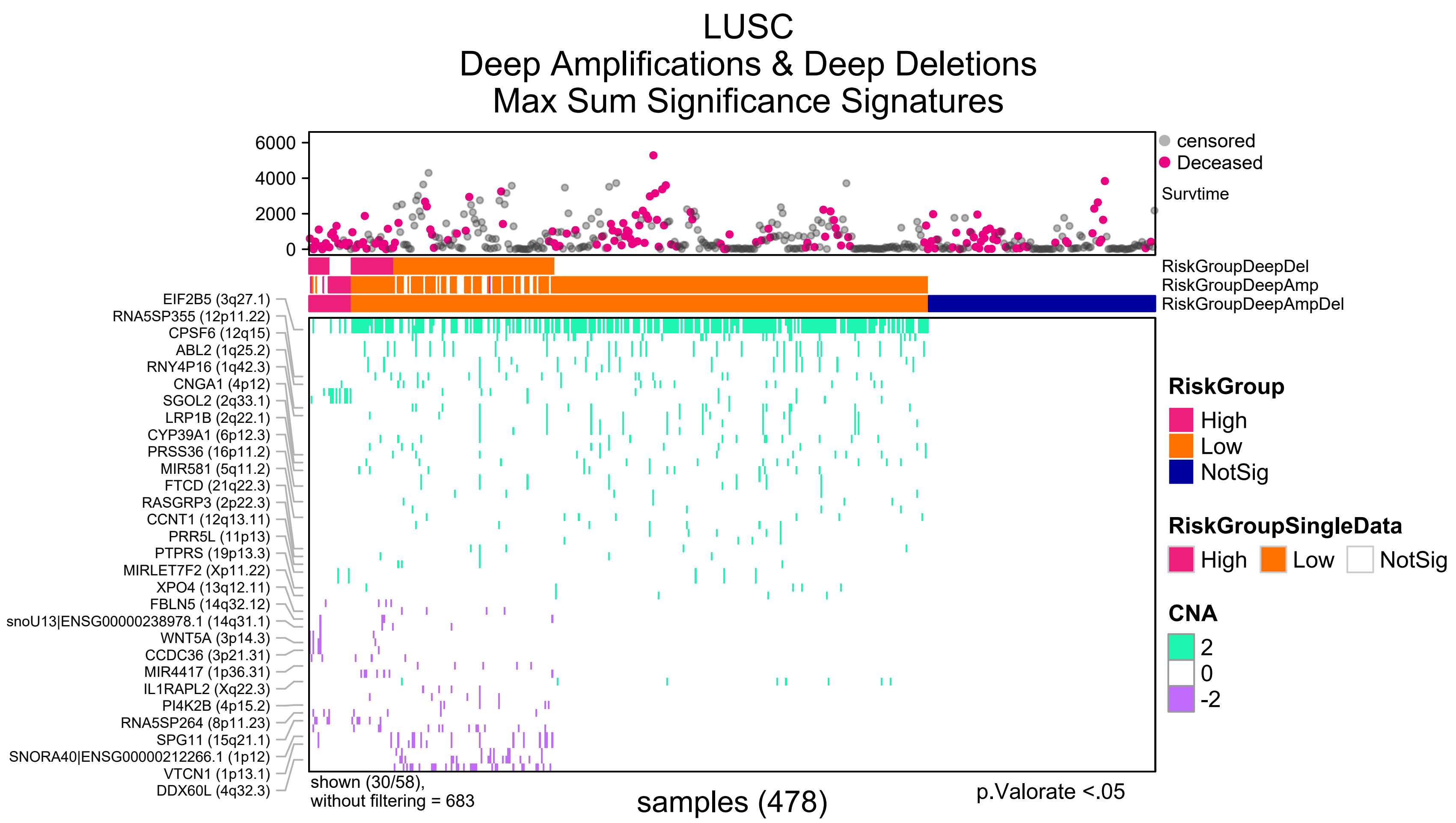
LUSC
Deep Amplifications & Deep Deletions
Max Sum Significance Signatures
6000
4000
2000
0
censored
Deceased
Survtime
RiskGroupDeepDel
RiskGroupDeepAmp
RiskGroupDeepAmpDel
EIF2B5 (3q27.1)
RNA5SP355 (12p11.22)
CPSF6 (12q15)
ABL2 (1q25.2)
RNY4P16 (1q42.3)
CNGA1 (4p12)
SGOL2 (2q33.1)
LRP1B (2q22.1)
CYP39A1 (6p12.3)
PRSS36 (16p11.2)
MIR581 (5q11.2)
FTCD (21q22.3)
RASGRP3 (2p22.3)
CCNT1 (12q13.11)
PRR5L (11p13)
PTPRS (19p13.3)
MIRLET7F2 (Xp11.22)
XPO4 (13q12.11)
FBLN5 (14q32.12)
snoU13|ENSG00000238978.1 (14q31.1)
WNT5A (3p14.3)
CCDC36 (3p21.31)
MIR4417 (1p36.31)
IL1RAPL2 (Xq22.3)
PI4K2B (4p15.2)
RNA5SP264 (8p11.23)
SPG11 (15q21.1)
SNORA40|ENSG00000212266.1 (1p12)
VTCN1 (1p13.1)
DDX60L (4q32.3)
RiskGroup
High
Low
NotSig
RiskGroupSingleData
High
Low
NotSig
CNA
2
0
-2
shown (30/58),
without filtering = 683
samples (478)
p.Valorate <.05

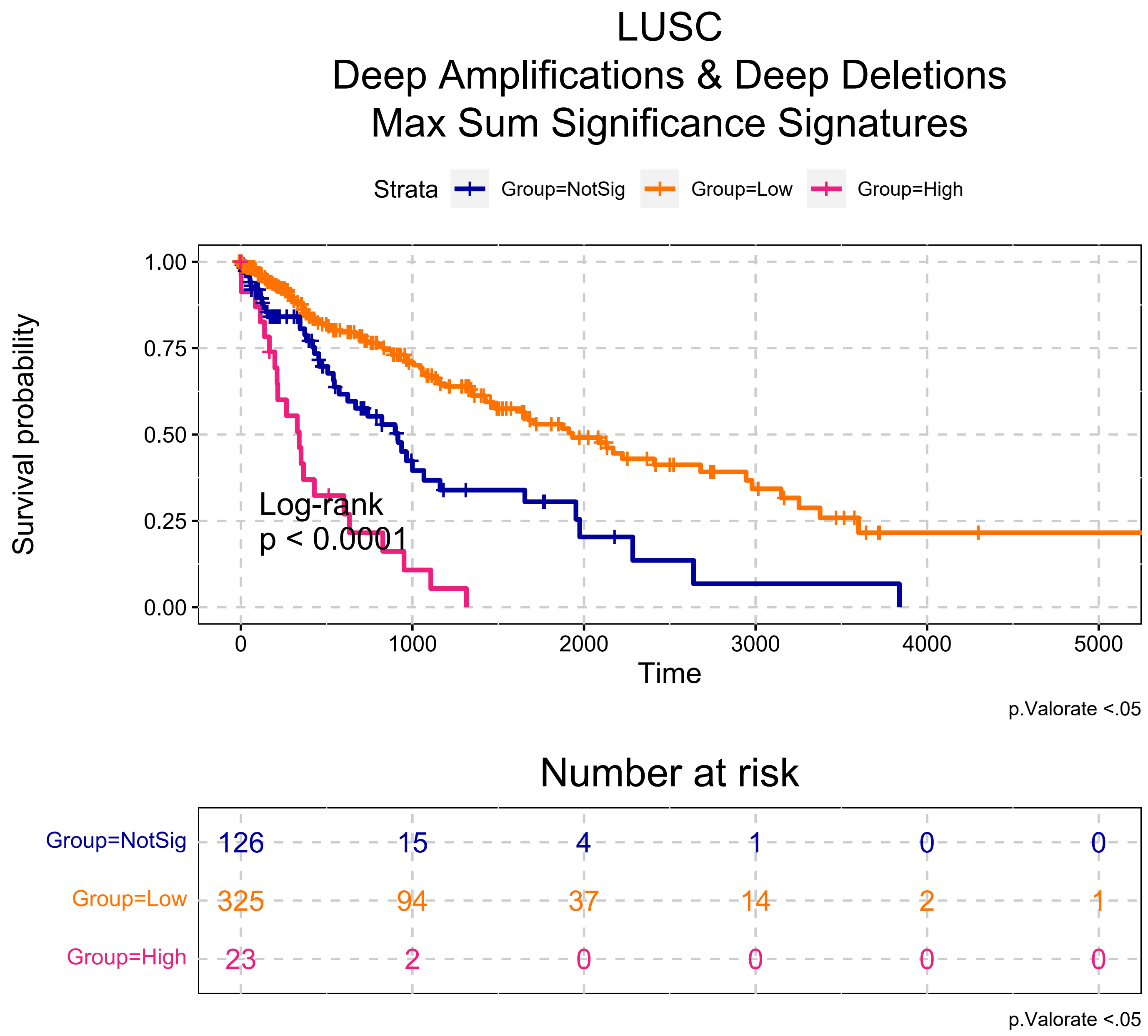

explanatory	beta	HR	L95	U95	p
Low	-0.77	0.46	0.32	0.67	0.00
High	1.07	2.92	1.72	4.96	0.00

n= 478, number of events =156
Score(logrank) test = p <.0001

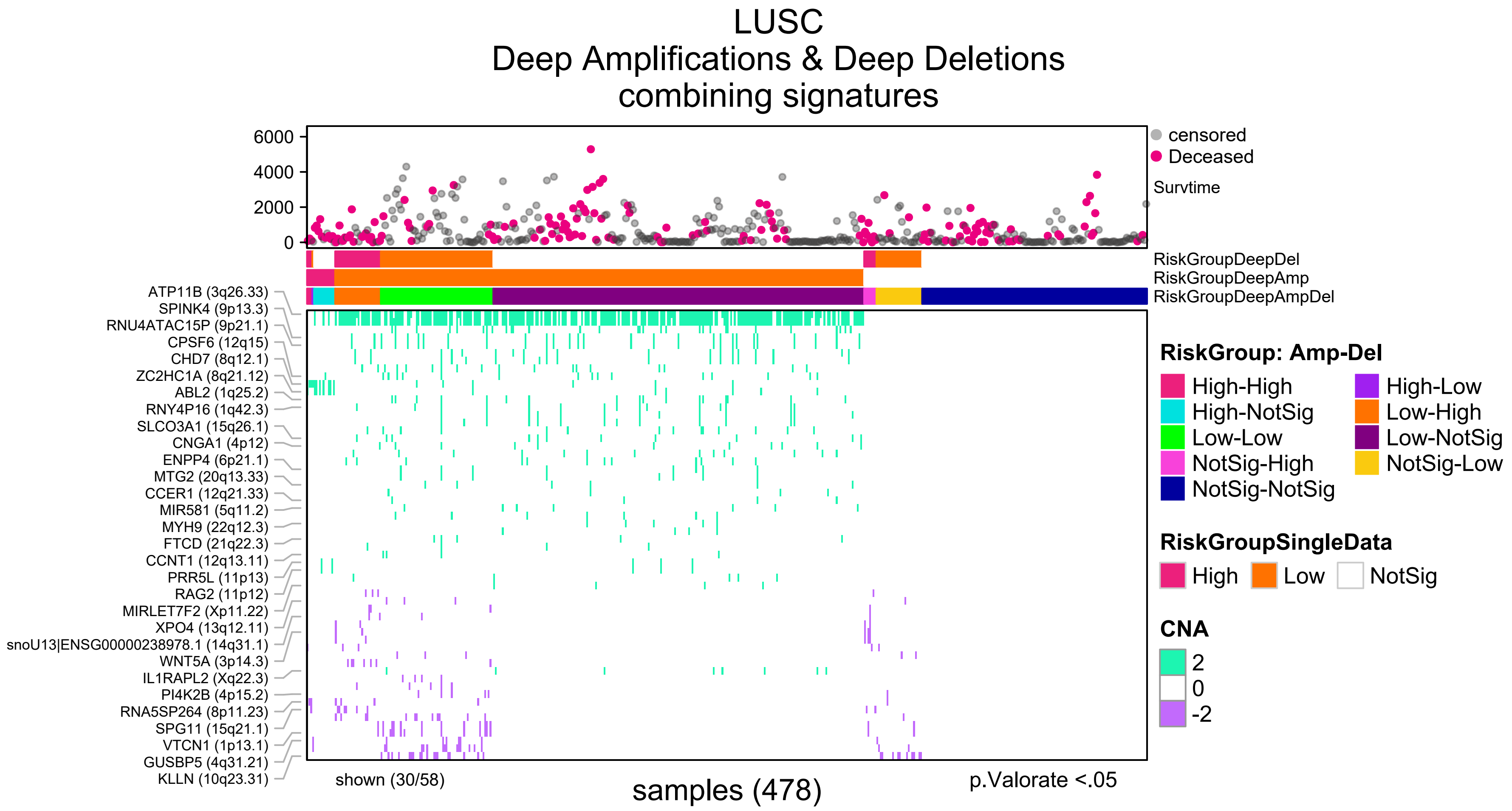

LUSC
Deep Amplifications & Deep Deletions
combining signatures
6000
4000
2000
0
censored
Deceased
Survtime
RiskGroupDeepDel
RiskGroupDeepAmp
RiskGroupDeepAmpDel
ATP11B (3q26.33)
SPINK4 (9p13.3)
RNU4ATAC15P (9p21.1)
CPSF6 (12q15)
CHD7 (8q12.1)
ZC2HC1A (8q21.12)
ABL2 (1q25.2)
RNY4P16 (1q42.3)
SLCO3A1 (15q26.1)
CNGA1 (4p12)
ENPP4 (6p21.1)
MTG2 (20q13.33)
CCER1 (12q21.33)
MIR581 (5q11.2)
MYH9 (22q12.3)
FTCD (21q22.3)
CCNT1 (12q13.11)
PRR5L (11p13)
RAG2 (11p12)
MIRLET7F2 (Xp11.22)
XPO4 (13q12.11)
snoU13|ENSG00000238978.1 (14q31.1)
WNT5A (3p14.3)
IL1RAPL2 (Xq22.3)
PI4K2B (4p15.2)
RNA5SP264 (8p11.23)
SPG11 (15q21.1)
VTCN1 (1p13.1)
GUSBP5 (4q31.21)
KLLN (10q23.31)
RiskGroup: Amp-Del
High-High
High-NotSig
Low-Low
NotSig-High
NotSig-NotSig
High-Low
Low-High
Low-NotSig
NotSig-Low
RiskGroupSingleData
High
Low
NotSig
CNA
2
0
-2
shown (30/58)
samples (478)
p.Valorate <.05

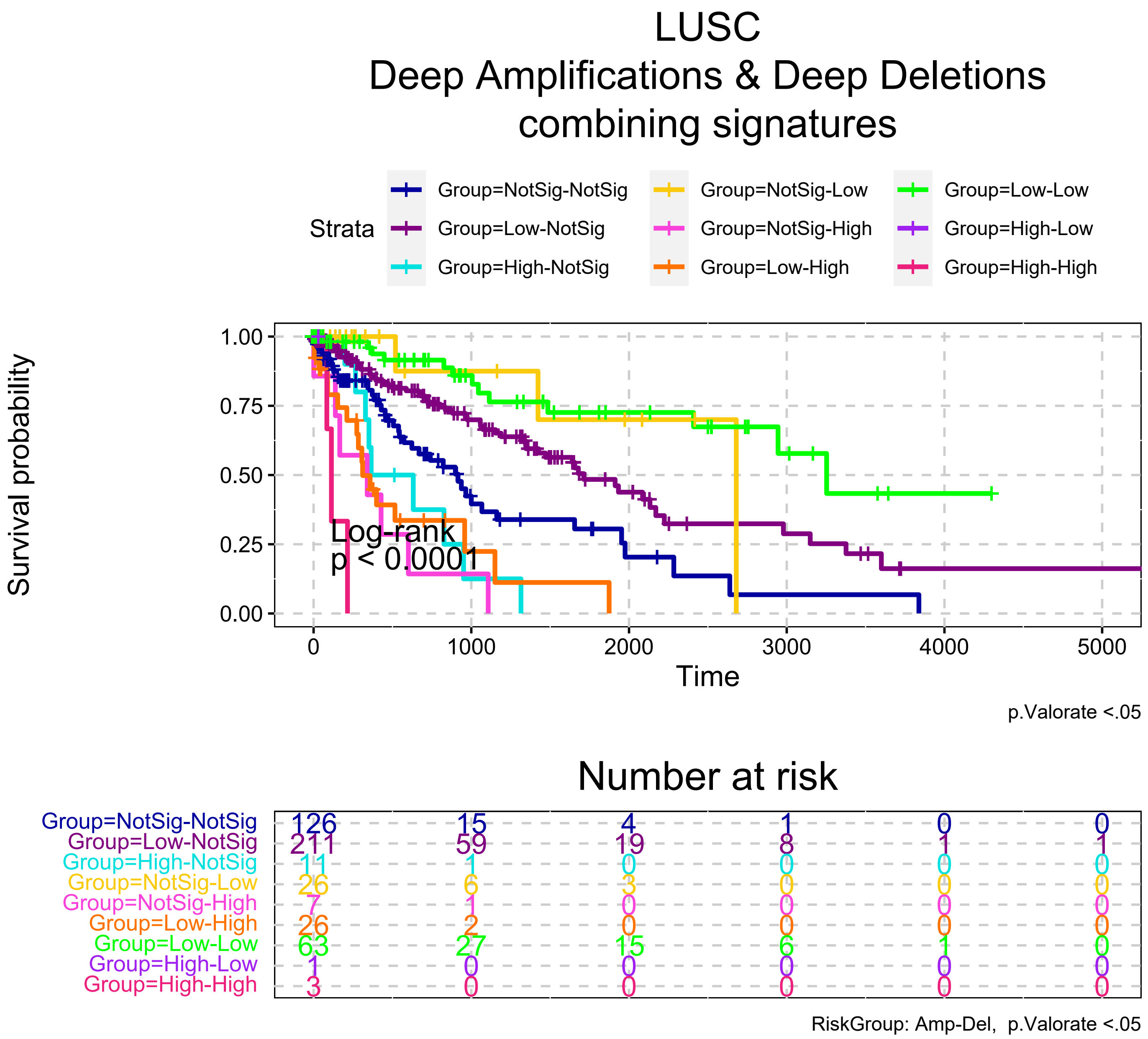

explanatory	beta	HR	L95	U95	p
Low-NotSig	-0.71	0.49	0.33	0.73	0.00
High-NotSig	0.73	2.08	1.01	4.29	0.05
NotSig-Low	-1.41	0.24	0.08	0.79	0.02
NotSig-High	1.19	3.30	1.48	7.38	0.00
Low-High	0.85	2.33	1.32	4.11	0.00
Low-Low	-1.56	0.21	0.11	0.40	0.00
High-Low	-11.65	0.00	0.00	Inf	1.00
High-High	2.39	10.92	3.27	36.48	0.00

n= 478, number of events =156
Score(logrank) test = p <.0001
