# Supplementary material for: Systematic Modeling of Risk-Associated Copy Number Alterations in Cancer
Source: Int J Mol Sci. 2024 Sep 27;25(19):10455. doi: 10.3390/ijms251910455 (PMC11477427; doi:10.3390/ijms251910455)
Supplement: Supplementary file 1 [file ijms-25-10455-s001.zip › MESOSignatureV12-sinSombreado.pdf]

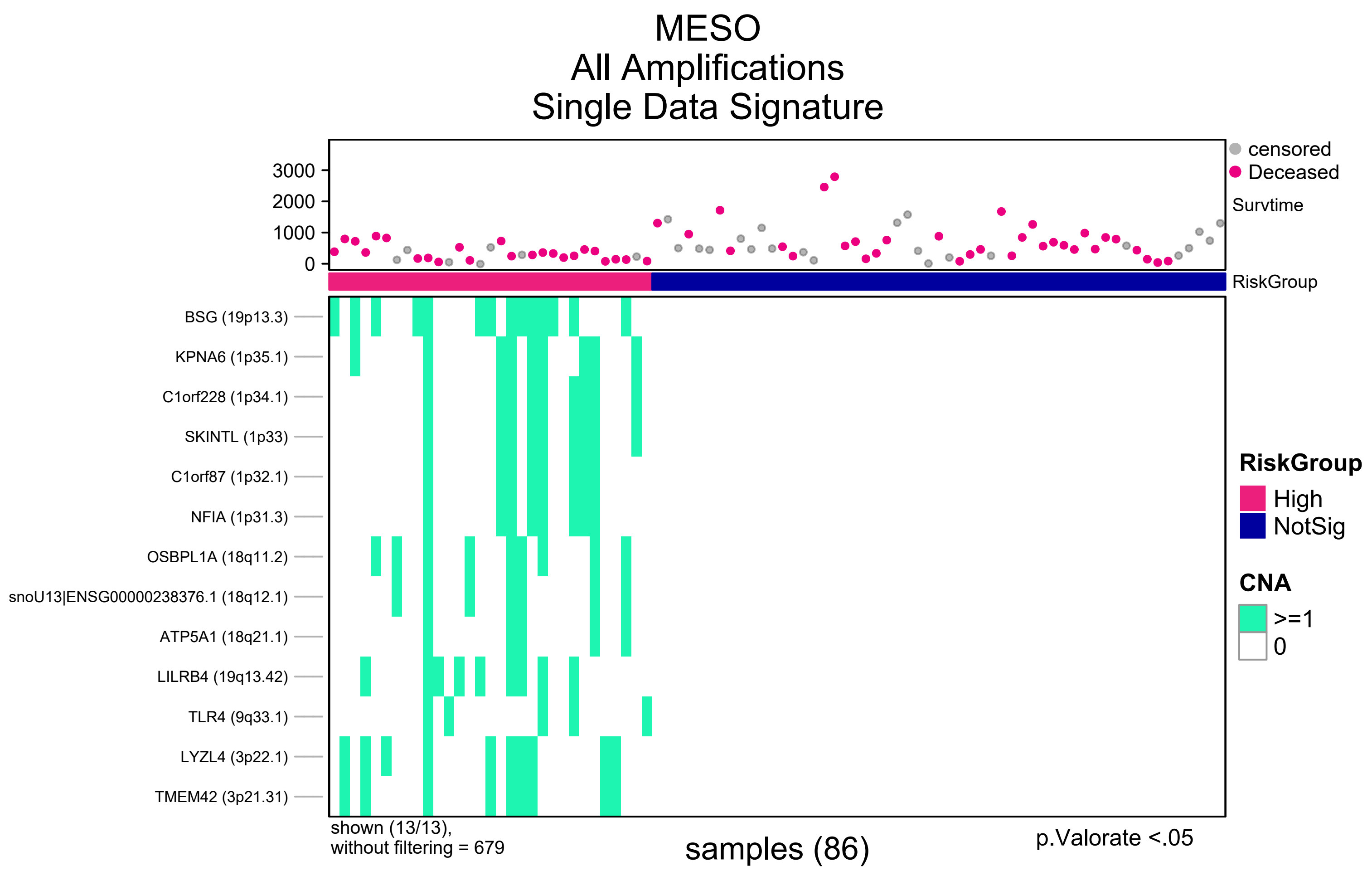

MESO
All Amplifications
Single Data Signature
3000
2000
1000
0
censored
Deceased
Survtime
RiskGroup
BSG (19p13.3)
KPNA6 (1p35.1)
C1orf228 (1p34.1)
SKINTL (1p33)
C1orf87 (1p32.1)
NFIA (1p31.3)
OSBPL1A (18q11.2)
snoU13|ENSG00000238376.1 (18q12.1)
ATP5A1 (18q21.1)
LILRB4 (19q13.42)
TLR4 (9q33.1)
LYZL4 (3p22.1)
TMEM42 (3p21.31)
RiskGroup
High
NotSig
CNA
>=1
0
shown (13/13),
without filtering = 679
samples (86)
p.Valorate <.05

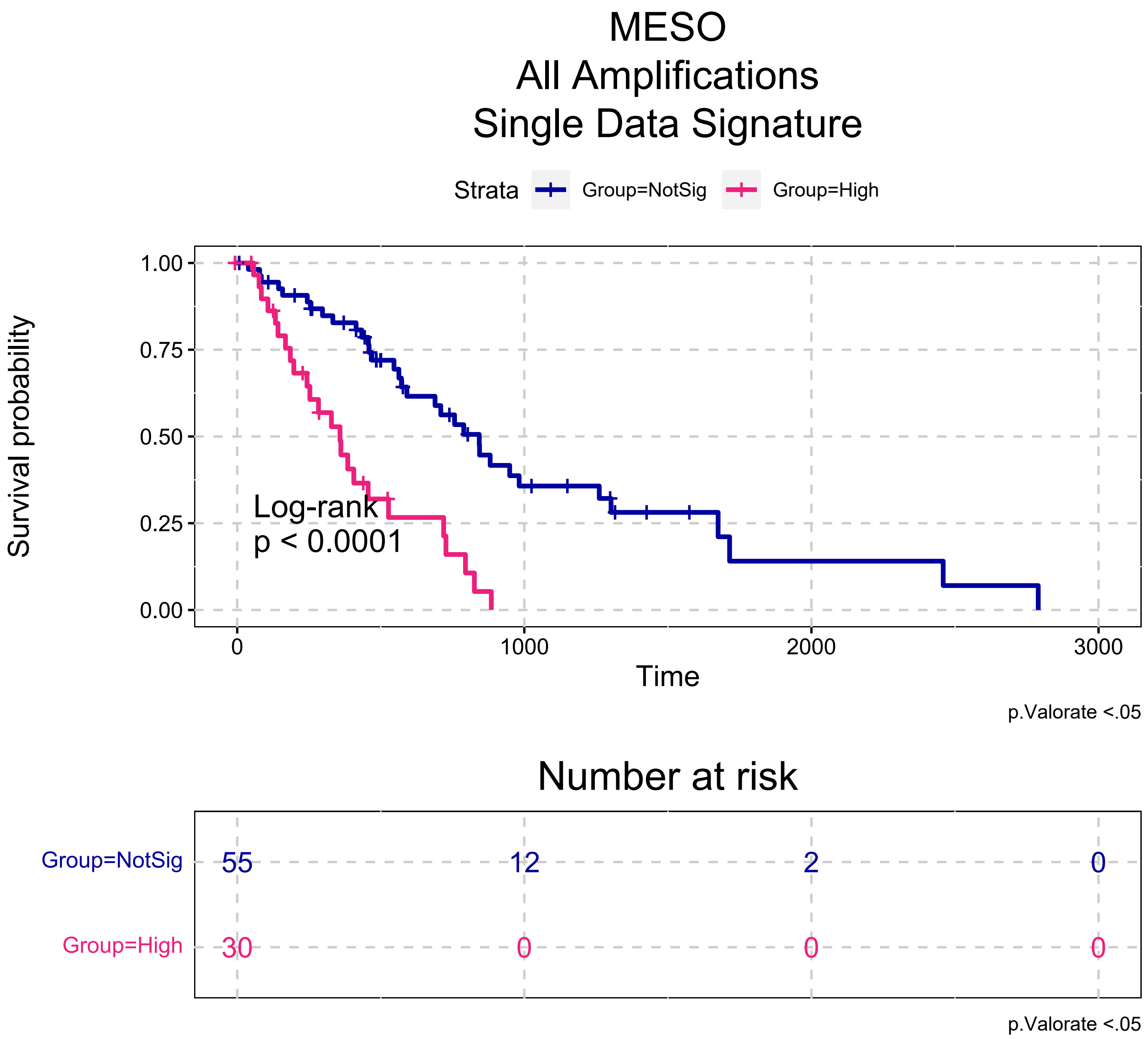

explanatory	beta	HR	L95	U95	p
High	1.28	3.61	2.02	6.46	0.00

n= 86, number of events =57
Score(logrank) test = p <.0001

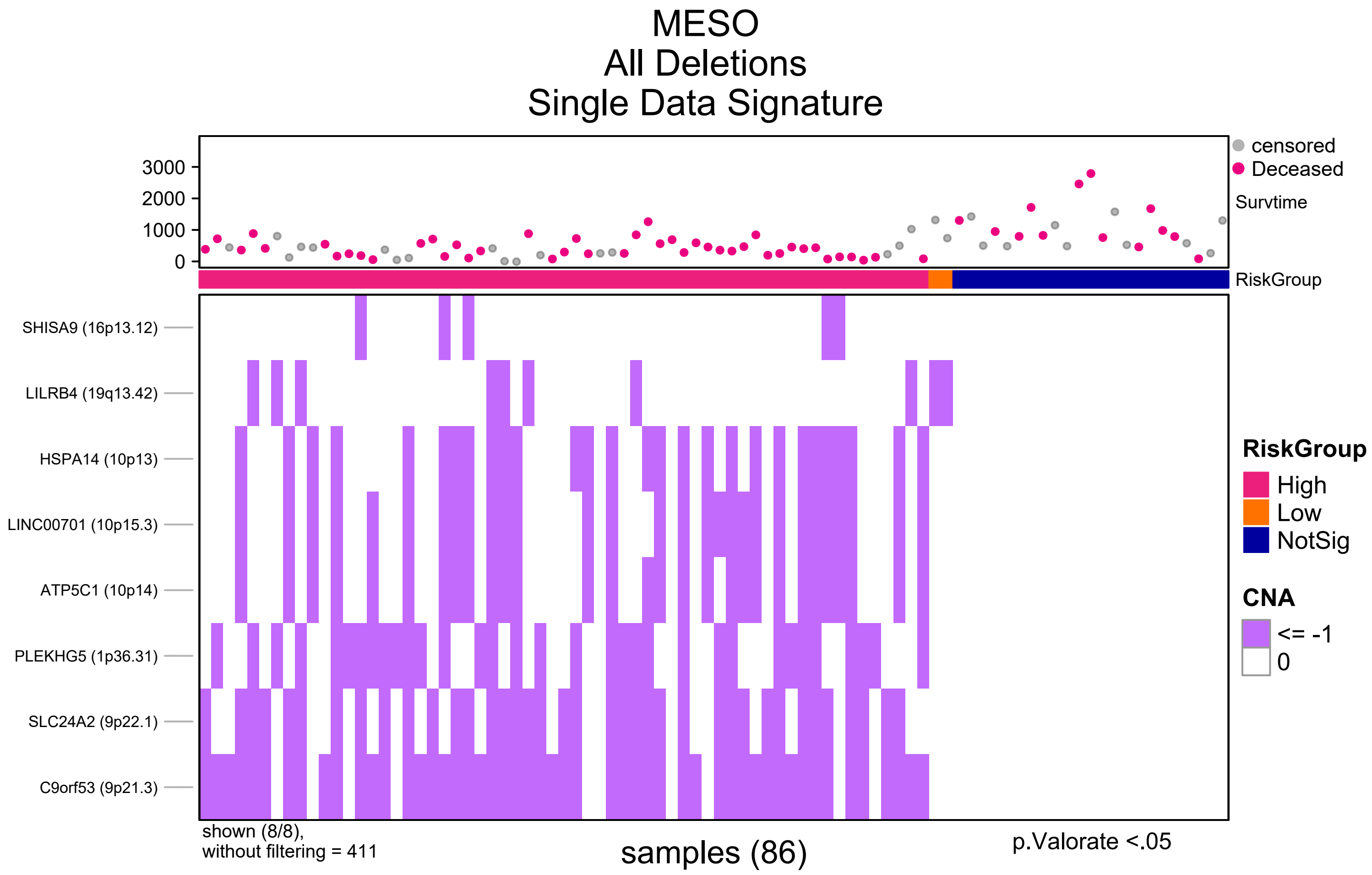

MESO
All Deletions
Single Data Signature
3000
2000
1000
0
censored
Deceased
Survtime
RiskGroup
SHISA9 (16p13.12)
LILRB4 (19q13.42)
HSPA14 (10p13)
LINC00701 (10p15.3)
ATP5C1 (10p14)
PLEKHG5 (1p36.31)
SLC24A2 (9p22.1)
C9orf53 (9p21.3)
RiskGroup
High
Low
NotSig
CNA
<= -1
0
shown (8/8),
without filtering = 411
samples (86)
p.Valorate <.05

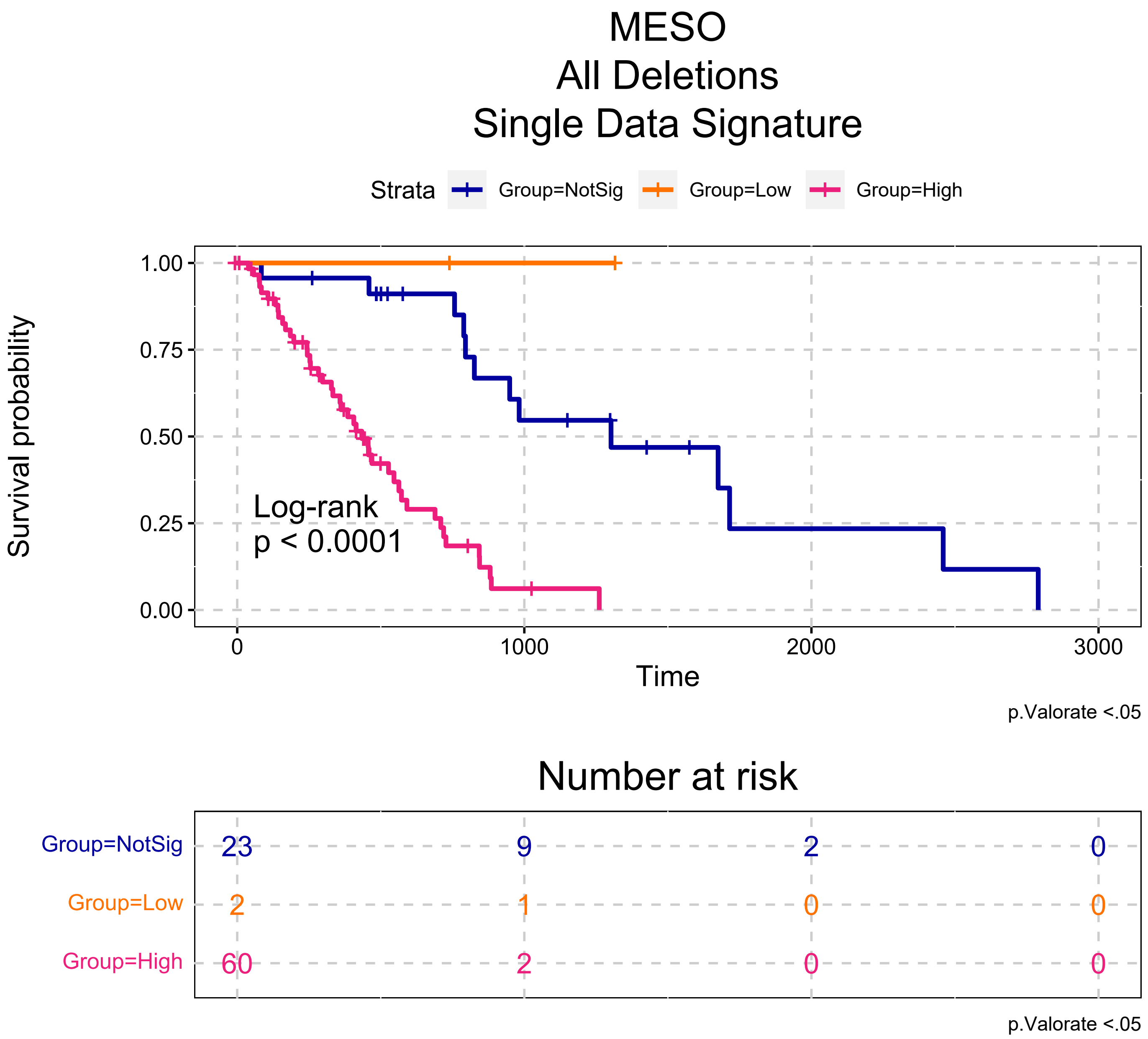

explanatory	beta	HR	L95	U95	p
Low	-16.24	0.00	0.00	Inf	1.00
High	1.84	6.29	2.85	13.87	0.00

n= 86, number of events =57
Score(logrank) test = p <.0001

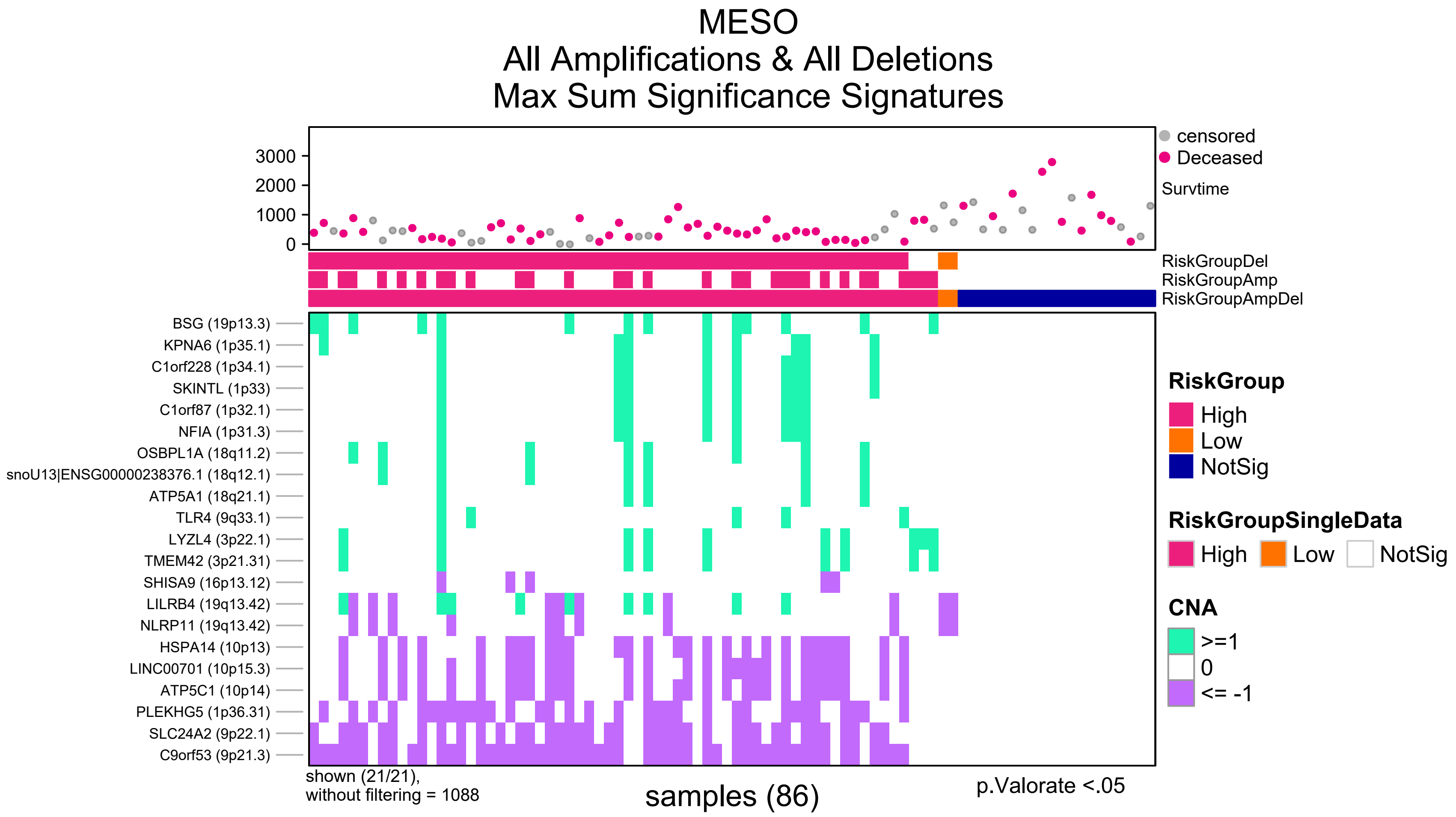
MESO
All Amplifications & All Deletions
Max Sum Significance Signatures
3000
2000
1000
0
censored
Deceased
Survtime
RiskGroupDel
RiskGroupAmp
RiskGroupAmpDel
BSG (19p13.3)
KPNA6 (1p35.1)
C1orf228 (1p34.1)
SKINTL (1p33)
C1orf87 (1p32.1)
NFIA (1p31.3)
OSBPL1A (18q11.2)
snoU13|ENSG00000238376.1 (18q12.1)
ATP5A1 (18q21.1)
TLR4 (9q33.1)
LYZL4 (3p22.1)
TMEM42 (3p21.31)
SHISA9 (16p13.12)
LILRB4 (19q13.42)
NLRP11 (19q13.42)
HSPA14 (10p13)
LINC00701 (10p15.3)
ATP5C1 (10p14)
PLEKHG5 (1p36.31)
SLC24A2 (9p22.1)
C9orf53 (9p21.3)
RiskGroup
High
Low
NotSig
RiskGroupSingleData
High
Low
NotSig
CNA
>=1
0
<= -1
shown (21/21),
without filtering = 1088
samples (86)
p.Valorate <.05

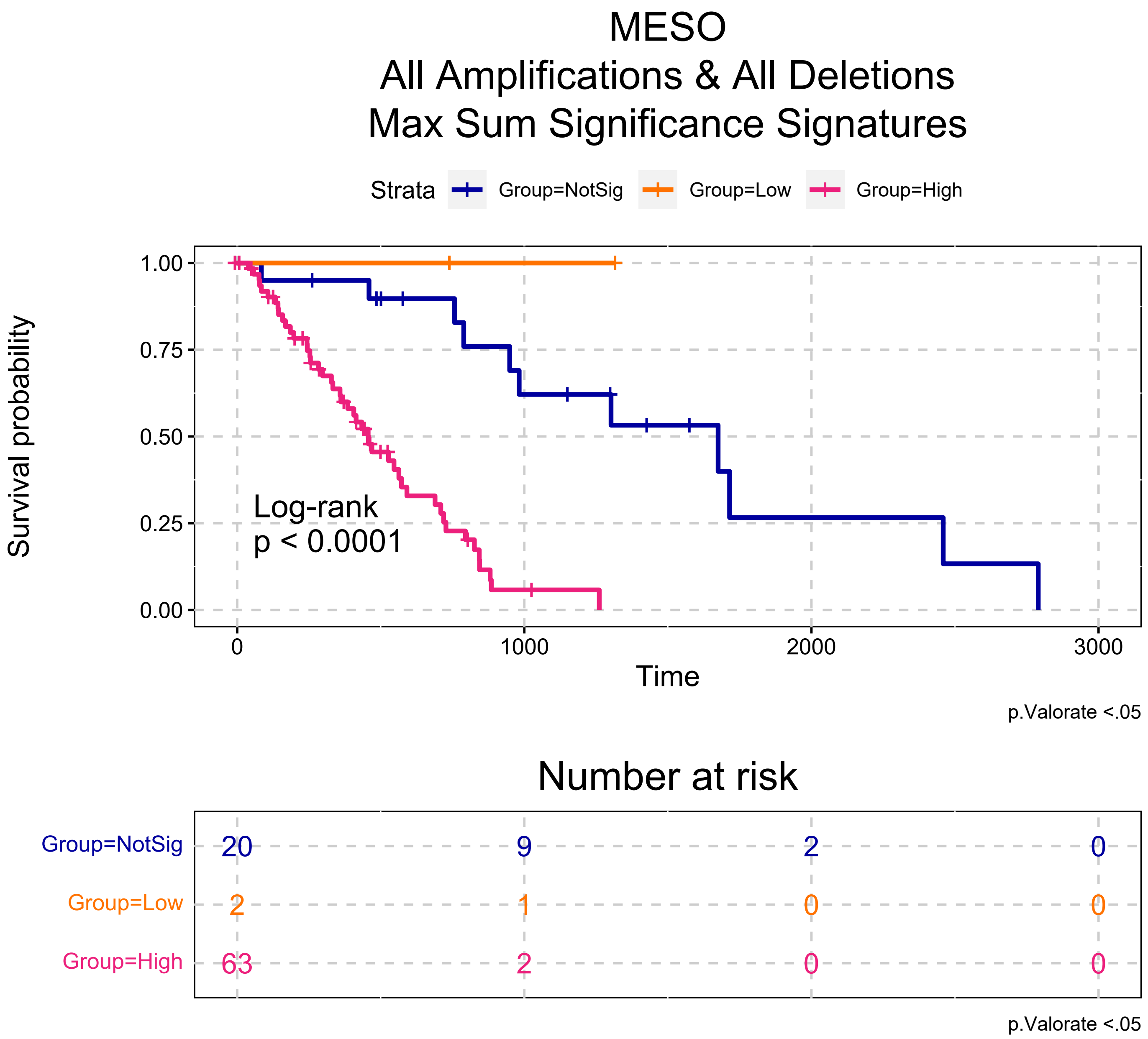

explanatory	beta	HR	L95	U95	p
Low	-16.05	0.00	0.00	Inf	1.00
High	2.01	7.44	3.03	18.26	0.00

n= 86, number of events =57
Score(logrank) test = p <.0001

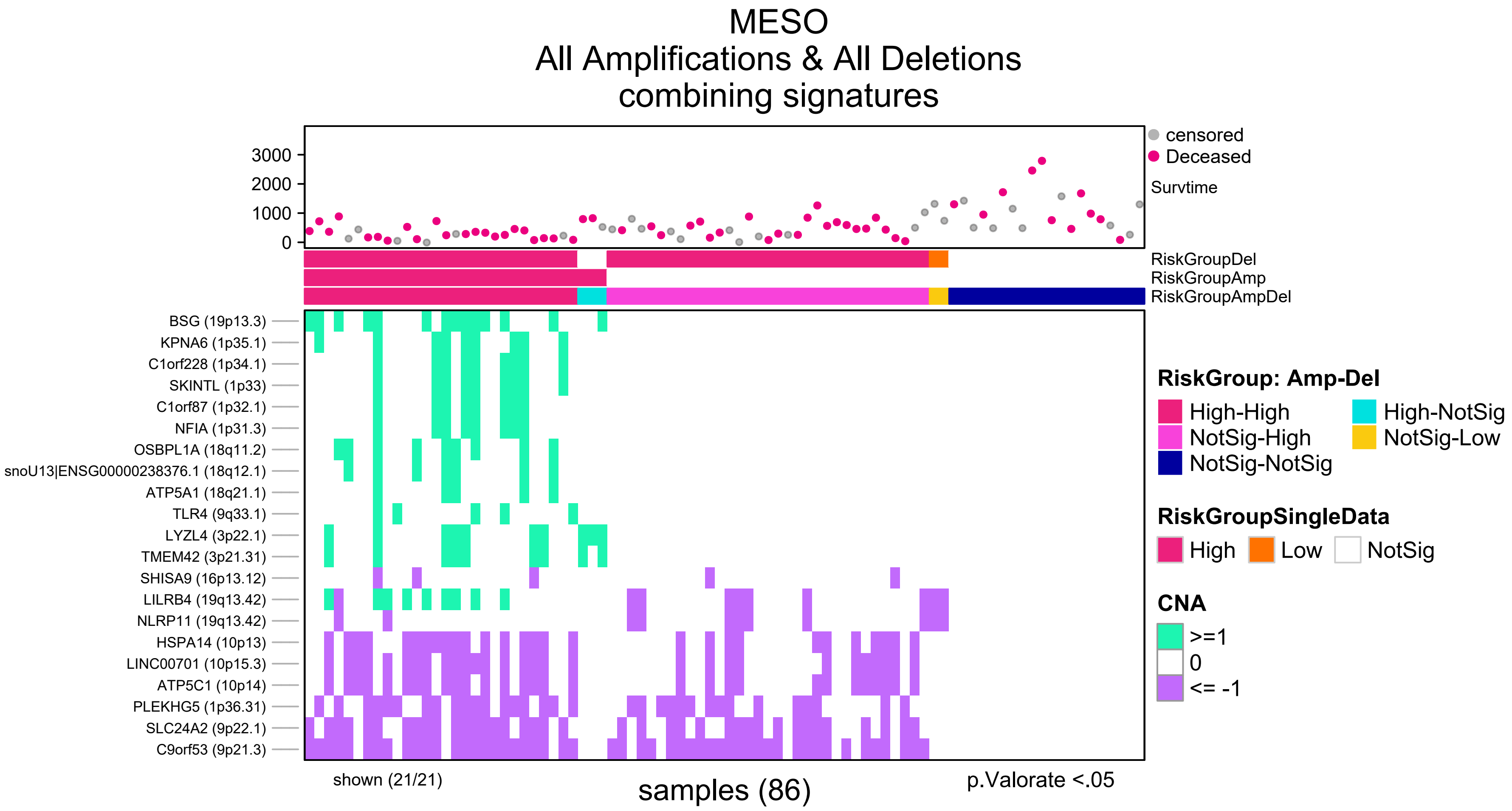
MESO
All Amplifications & All Deletions
combining signatures
3000
2000
1000
0
censored
Deceased
Survtime
RiskGroupDel
RiskGroupAmp
RiskGroupAmpDel
BSG (19p13.3)
KPNA6 (1p35.1)
C1orf228 (1p34.1)
SKINTL (1p33)
C1orf87 (1p32.1)
NFIA (1p31.3)
OSBPL1A (18q11.2)
snoU13|ENSG00000238376.1 (18q12.1)
ATP5A1 (18q21.1)
TLR4 (9q33.1)
LYZL4 (3p22.1)
TMEM42 (3p21.31)
SHISA9 (16p13.12)
LILRB4 (19q13.42)
NLRP11 (19q13.42)
HSPA14 (10p13)
LINC00701 (10p15.3)
ATP5C1 (10p14)
PLEKHG5 (1p36.31)
SLC24A2 (9p22.1)
C9orf53 (9p21.3)
RiskGroup: Amp-Del
High-High
High-NotSig
NotSig-High
NotSig-Low
NotSig-NotSig
RiskGroupSingleData
High
Low
NotSig
CNA
>=1
0
<= -1
shown (21/21)
samples (86)
p.Valorate <.05

MESO
All Amplifications & All Deletions
combining signatures

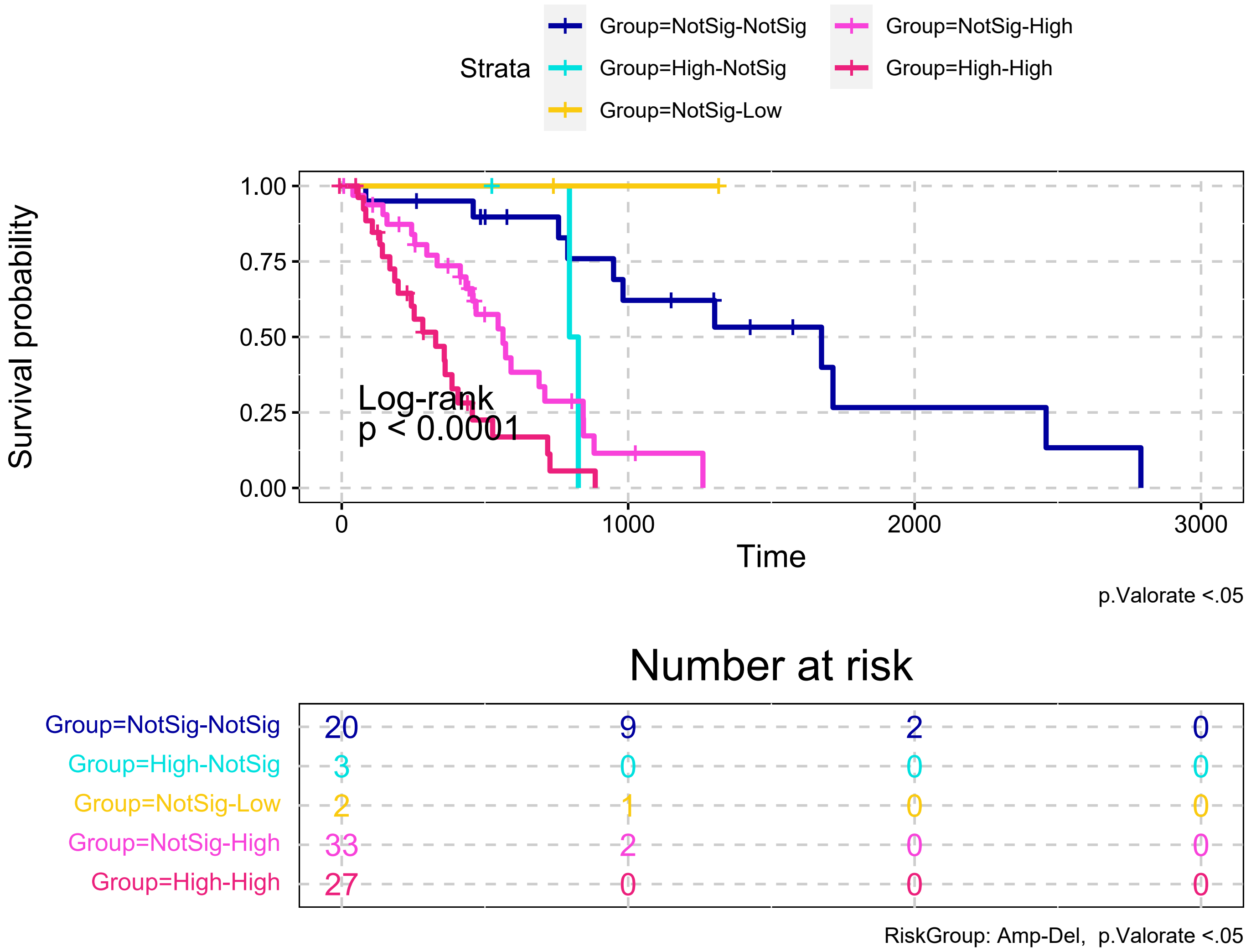

explanatory	beta	HR	L95	U95	p
High-NotSig	1.30	3.66	0.71	18.94	0.12
NotSig-Low	-16.19	0.00	0.00	Inf	1.00
NotSig-High	1.78	5.93	2.31	15.24	0.00
High-High	2.67	14.51	5.41	38.91	0.00

n= 86, number of events =57
Score(logrank) test = p <.0001

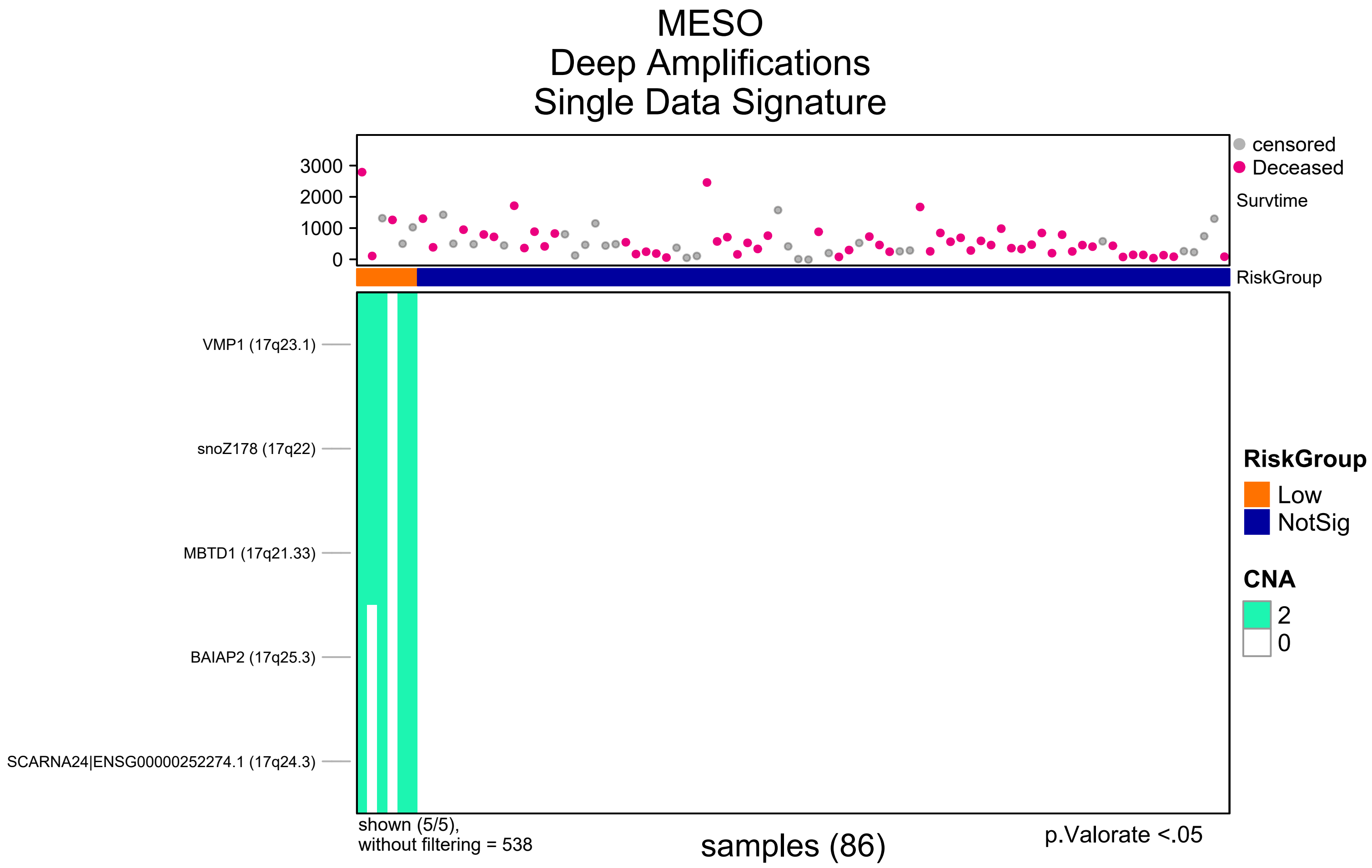

MESO
Deep Amplifications
Single Data Signature
3000
2000
1000
0
censored
Deceased
Survtime
RiskGroup
VMP1 (17q23.1)
snoZ178 (17q22)
MBTD1 (17q21.33)
BAIAP2 (17q25.3)
SCARNA24|ENSG00000252274.1 (17q24.3)
RiskGroup
Low
NotSig
CNA
2
0
shown (5/5),
without filtering = 538
samples (86)
p.Valorate <.05

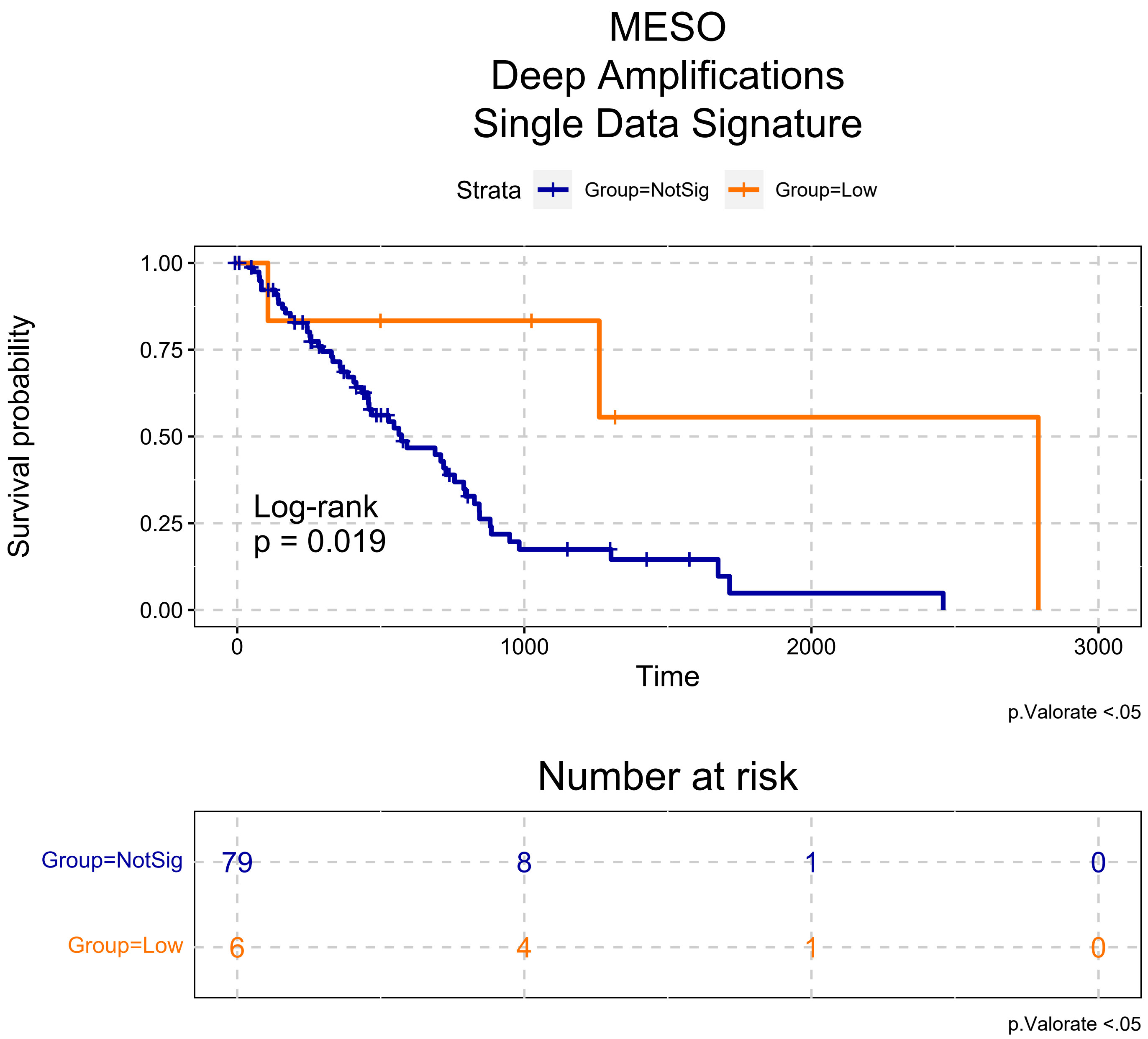

explanatory	beta	HR	L95	U95	p
Low	-1.56	0.21	0.05	0.88	0.03

n= 86, number of events =57
Score(logrank) test = 0.019

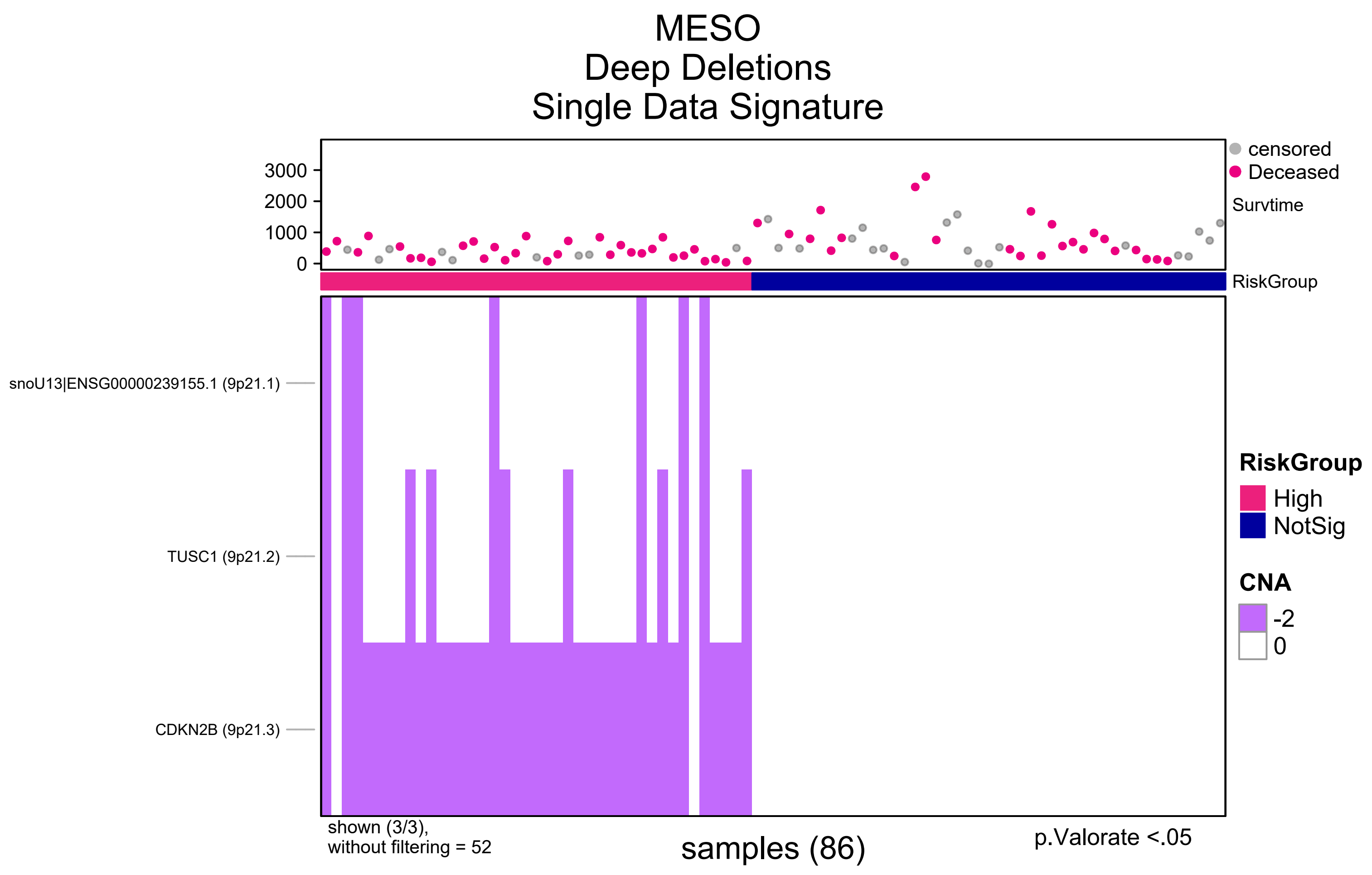
MESO
Deep Deletions
Single Data Signature
3000
2000
1000
0
censored
Deceased
Survtime
RiskGroup
snoU13|ENSG00000239155.1 (9p21.1)
TUSC1 (9p21.2)
CDKN2B (9p21.3)
RiskGroup
High
NotSig
CNA
-2
0
shown (3/3),
without filtering = 52
samples (86)
p.Valorate <.05

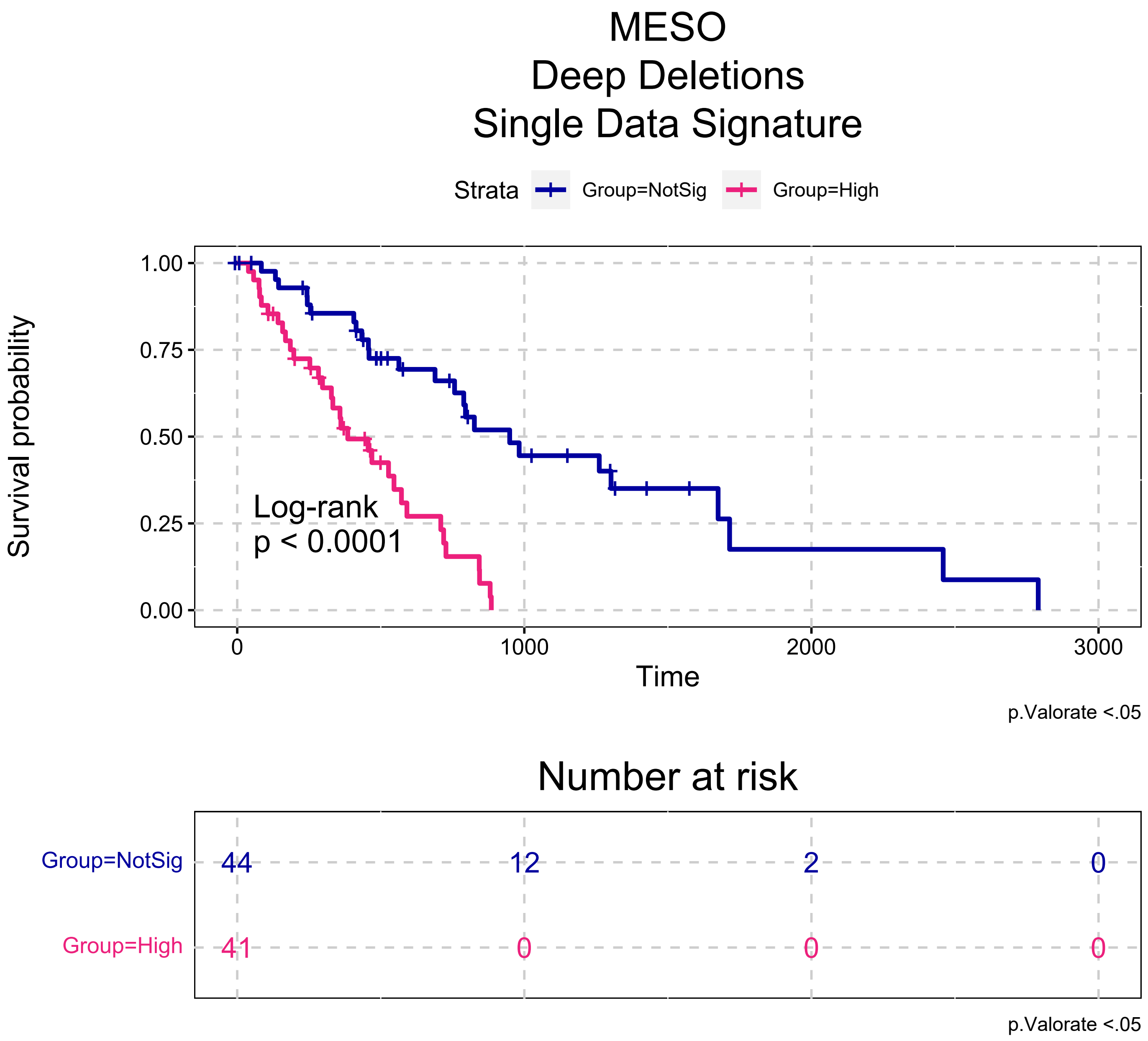

explanatory	beta	HR	L95	U95	p
High	1.36	3.91	2.12	7.19	0.00

n= 86, number of events =57
Score(logrank) test = p <.0001

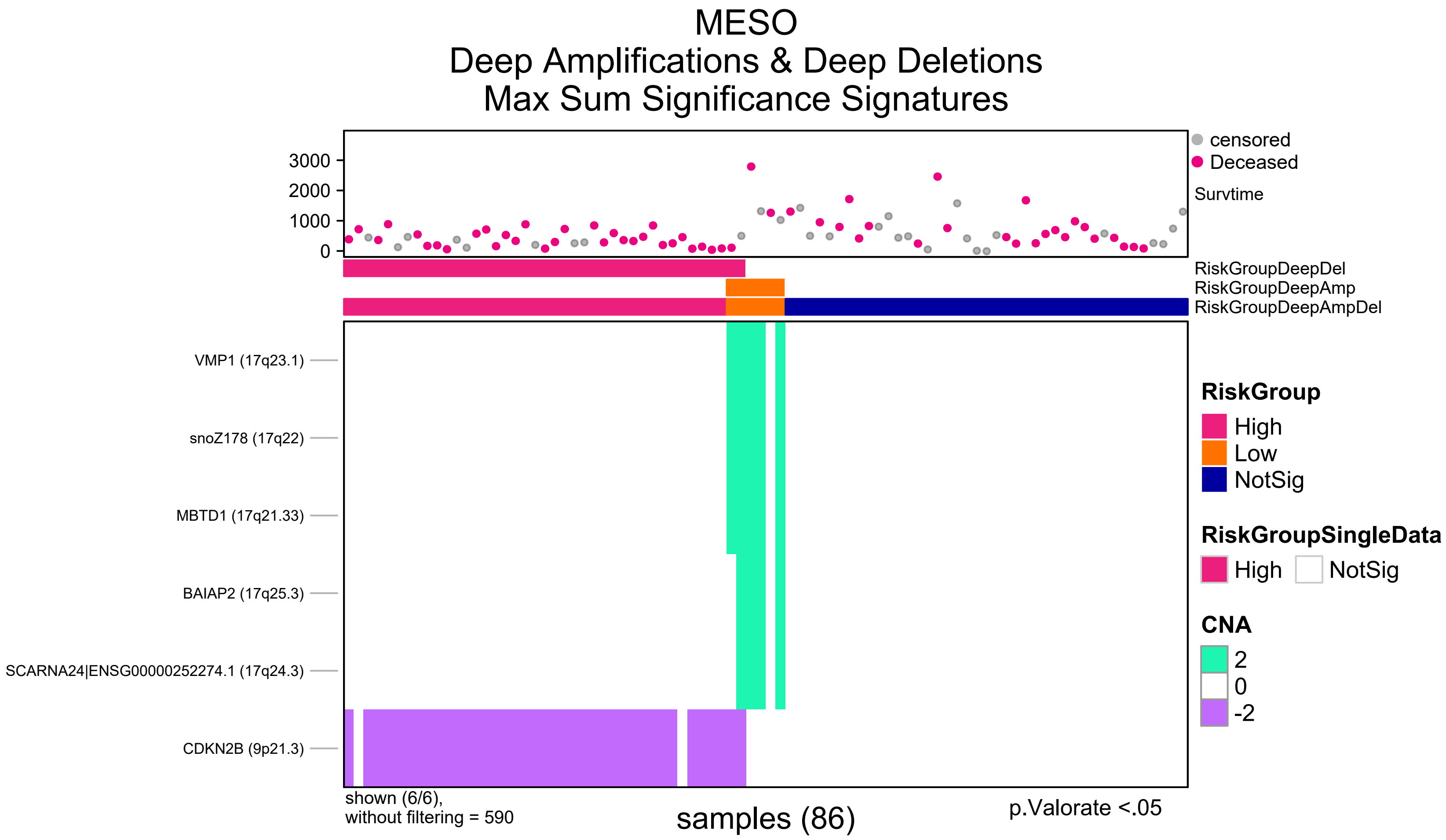

MESO
Deep Amplifications & Deep Deletions
Max Sum Significance Signatures
3000
2000
1000
0
censored
Deceased
Survtime
RiskGroupDeepDel
RiskGroupDeepAmp
RiskGroupDeepAmpDel
VMP1 (17q23.1)
snoZ178 (17q22)
MBTD1 (17q21.33)
BAIAP2 (17q25.3)
SCARNA24|ENSG00000252274.1 (17q24.3)
CDKN2B (9p21.3)
RiskGroup
High
Low
NotSig
RiskGroupSingleData
High
NotSig
CNA
2
0
-2
shown (6/6),
without filtering = 590
samples (86)
p.Valorate <.05

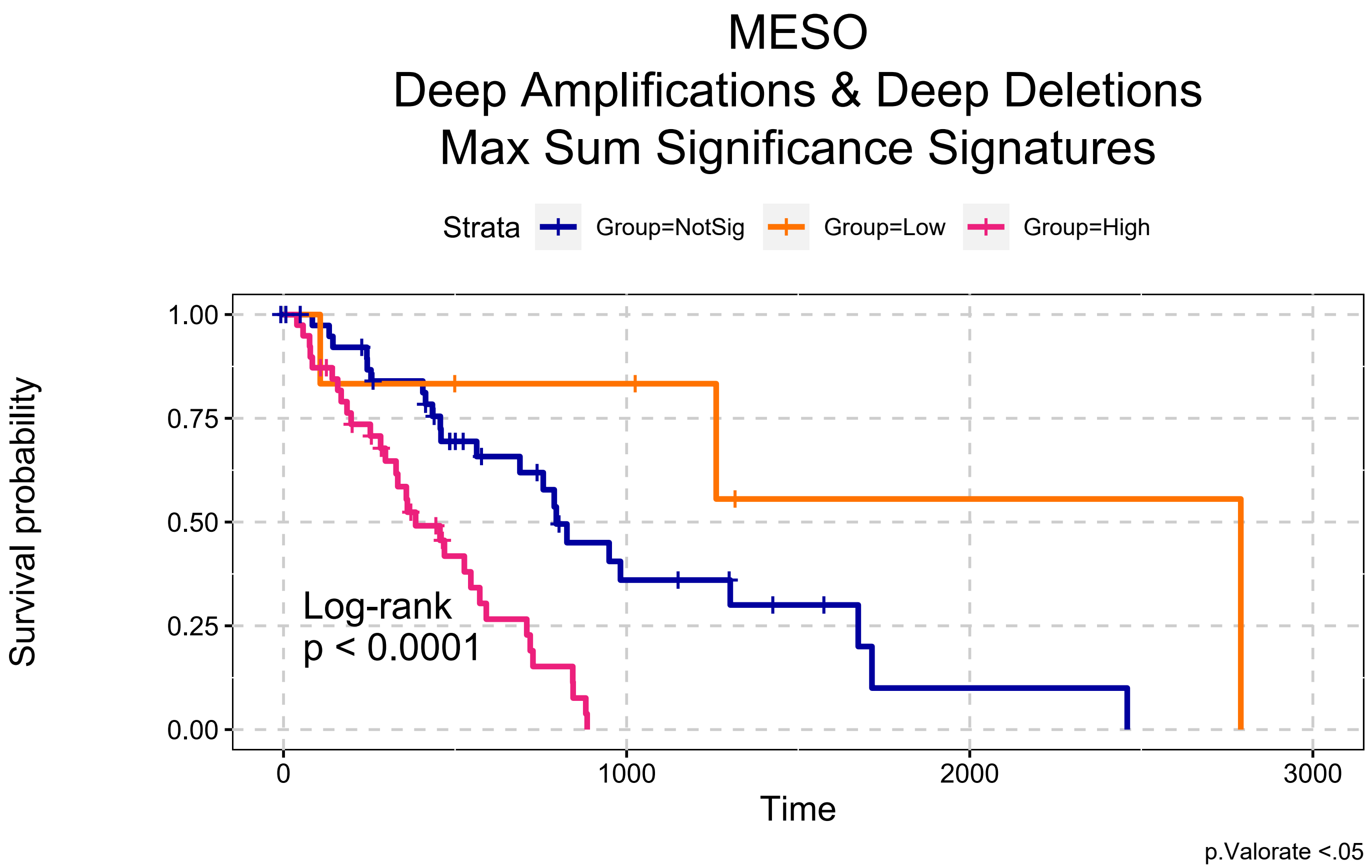

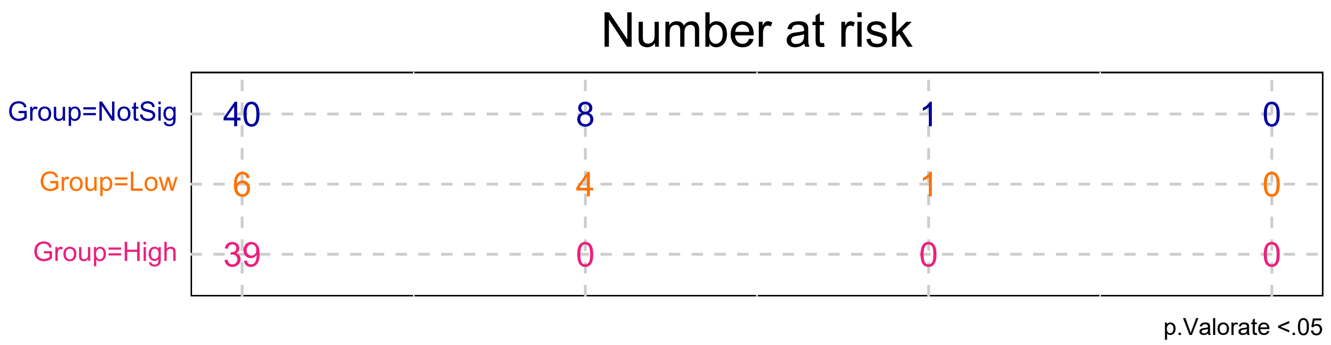

explanatory	beta	HR	L95	U95	p
Low	-1.21	0.30	0.07	1.28	0.10
High	1.19	3.29	1.80	6.01	0.00

n= 86, number of events =57
Score(logrank) test = p <.0001

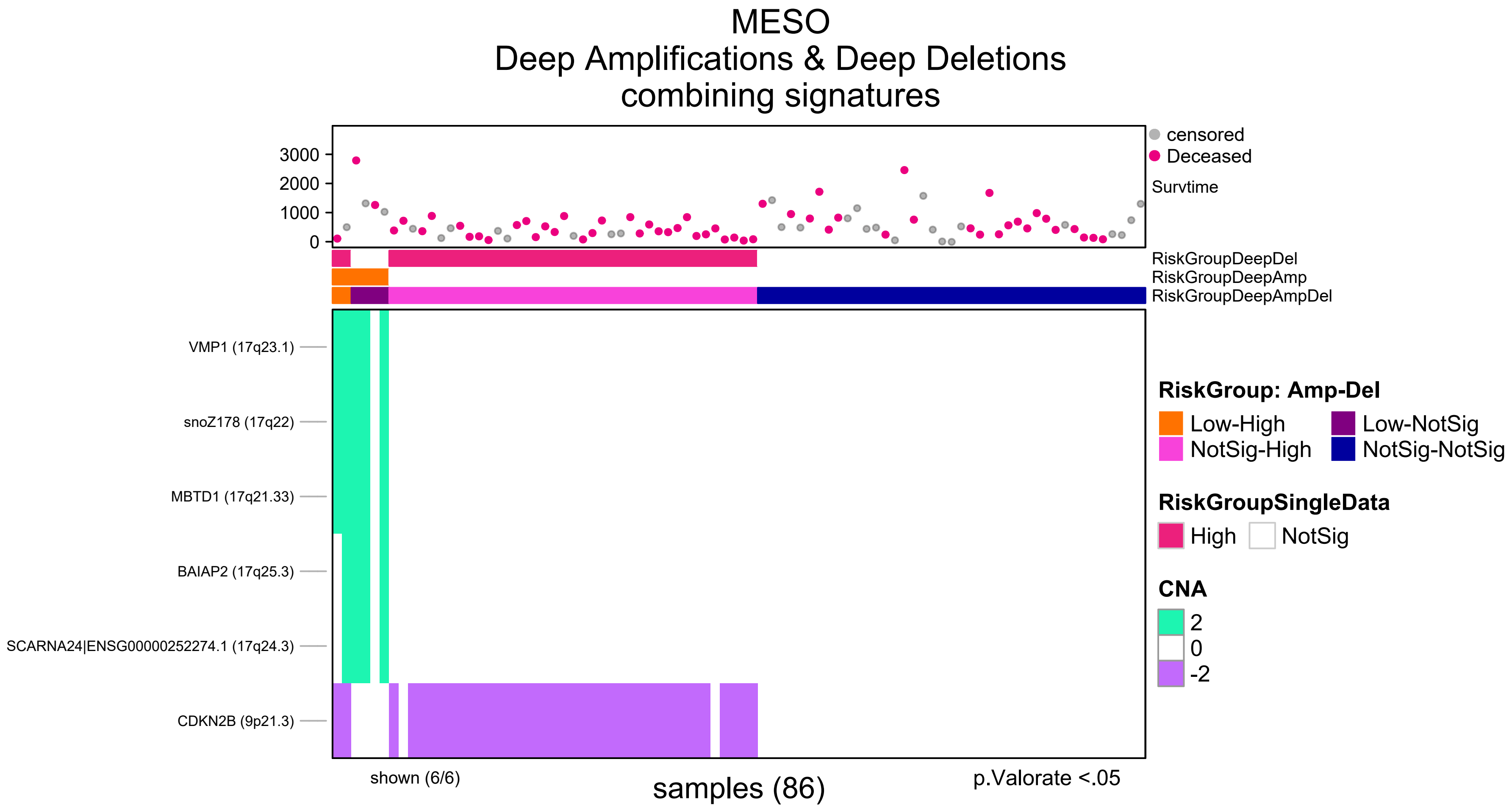

MESO
Deep Amplifications & Deep Deletions
combining signatures
3000
2000
1000
0
censored
Deceased
Survtime
RiskGroupDeepDel
RiskGroupDeepAmp
RiskGroupDeepAmpDel
VMP1 (17q23.1)
snoZ178 (17q22)
MBTD1 (17q21.33)
BAIAP2 (17q25.3)
SCARNA24|ENSG00000252274.1 (17q24.3)
CDKN2B (9p21.3)
RiskGroup: Amp-Del
Low-High
Low-NotSig
NotSig-High
NotSig-NotSig
RiskGroupSingleData
High
NotSig
CNA
2
0
-2
shown (6/6)
samples (86)
p.Valorate <.05

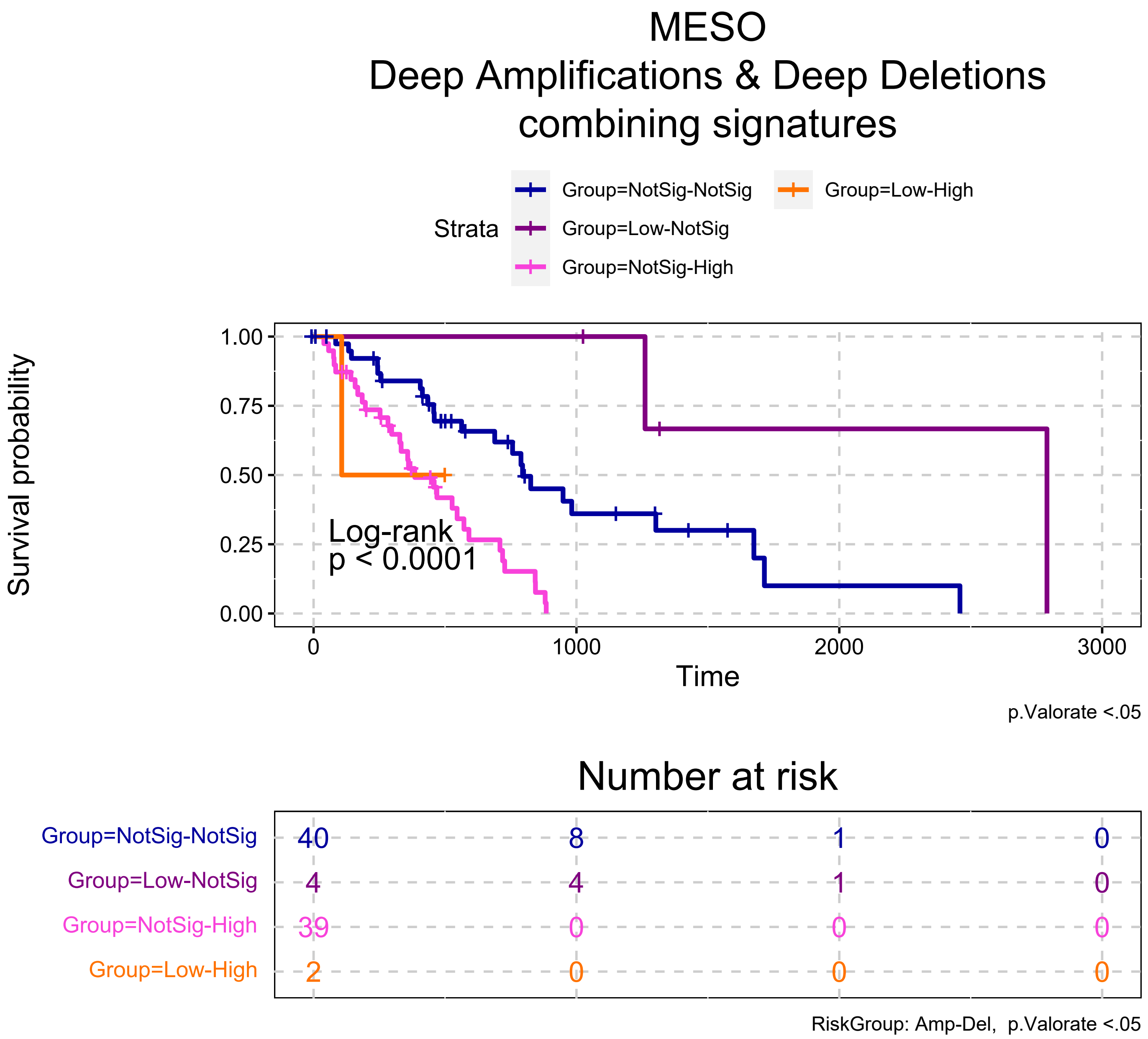

explanatory	beta	HR	L95	U95	p
Low-NotSig	-1.88	0.15	0.02	1.14	0.07
NotSig-High	1.22	3.40	1.85	6.26	0.00
Low-High	1.12	3.07	0.40	23.57	0.28

n= 86, number of events =57
Score(logrank) test = p <.0001
