# Supplementary material for: Systematic Modeling of Risk-Associated Copy Number Alterations in Cancer
Source: Int J Mol Sci. 2024 Sep 27;25(19):10455. doi: 10.3390/ijms251910455 (PMC11477427; doi:10.3390/ijms251910455)
Supplement: Supplementary file 1 [file ijms-25-10455-s001.zip › OVSignatureV12-sinSombreado.pdf]

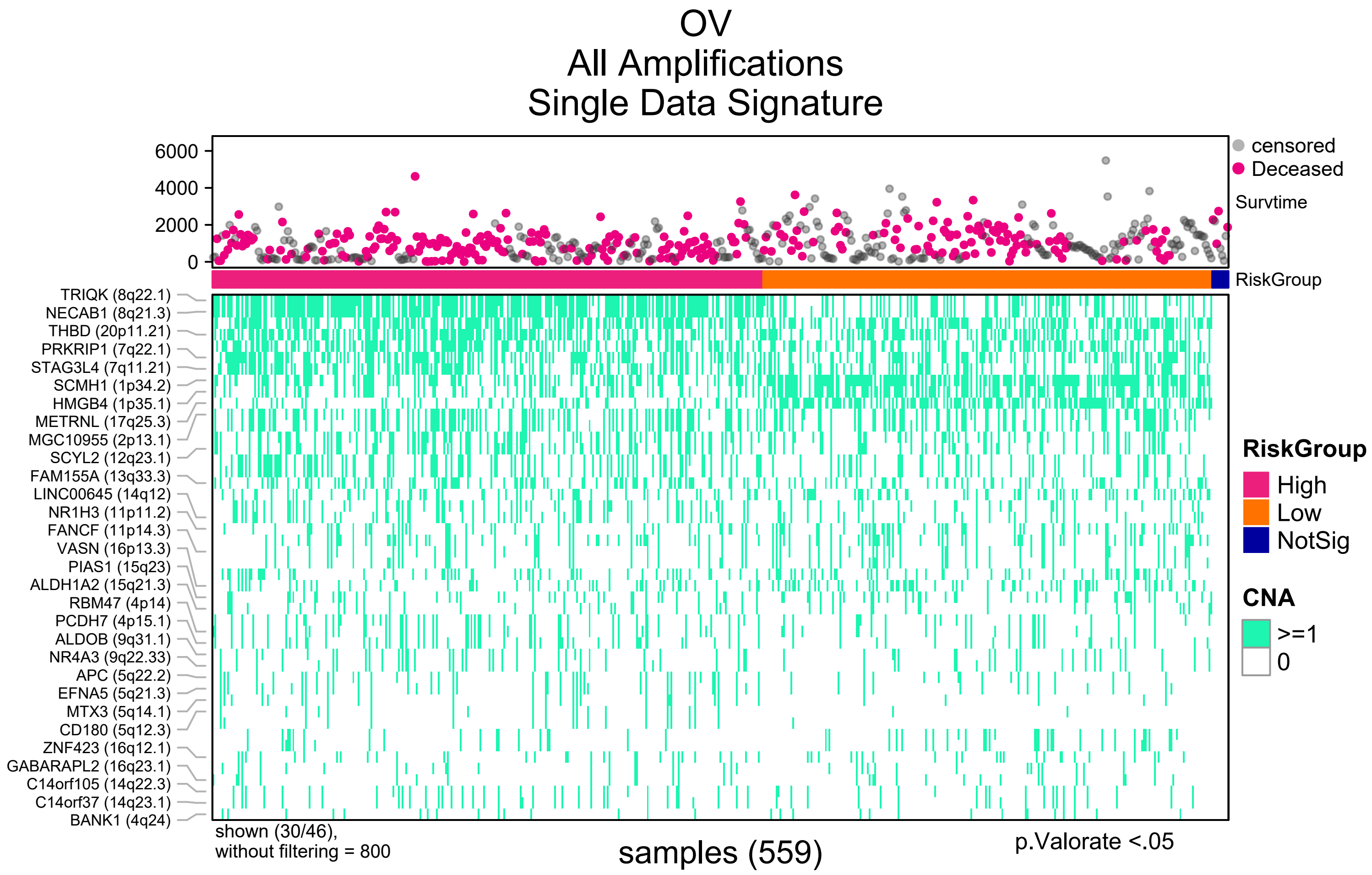

OV
All Amplifications
Single Data Signature
6000
4000
2000
0
censored
Deceased
Survtime
RiskGroup
TRIQK (8q22.1)
NECAB1 (8q21.3)
THBD (20p11.21)
PRKRIP1 (7q22.1)
STAG3L4 (7q11.21)
SCMH1 (1p34.2)
HMGB4 (1p35.1)
METRNL (17q25.3)
MGC10955 (2p13.1)
SCYL2 (12q23.1)
FAM155A (13q33.3)
LINC00645 (14q12)
NR1H3 (11p11.2)
FANCF (11p14.3)
VASN (16p13.3)
PIAS1 (15q23)
ALDH1A2 (15q21.3)
RBM47 (4p14)
PCDH7 (4p15.1)
ALDOB (9q31.1)
NR4A3 (9q22.33)
APC (5q22.2)
EFNA5 (5q21.3)
MTX3 (5q14.1)
CD180 (5q12.3)
ZNF423 (16q12.1)
GABARAPL2 (16q23.1)
C14orf105 (14q22.3)
C14orf37 (14q23.1)
BANK1 (4q24)
RiskGroup
High
Low
NotSig
CNA
>=1
0
shown (30/46),
without filtering = 800
samples (559)
p.Valorate <.05

OV
All Amplifications
Single Data Signature

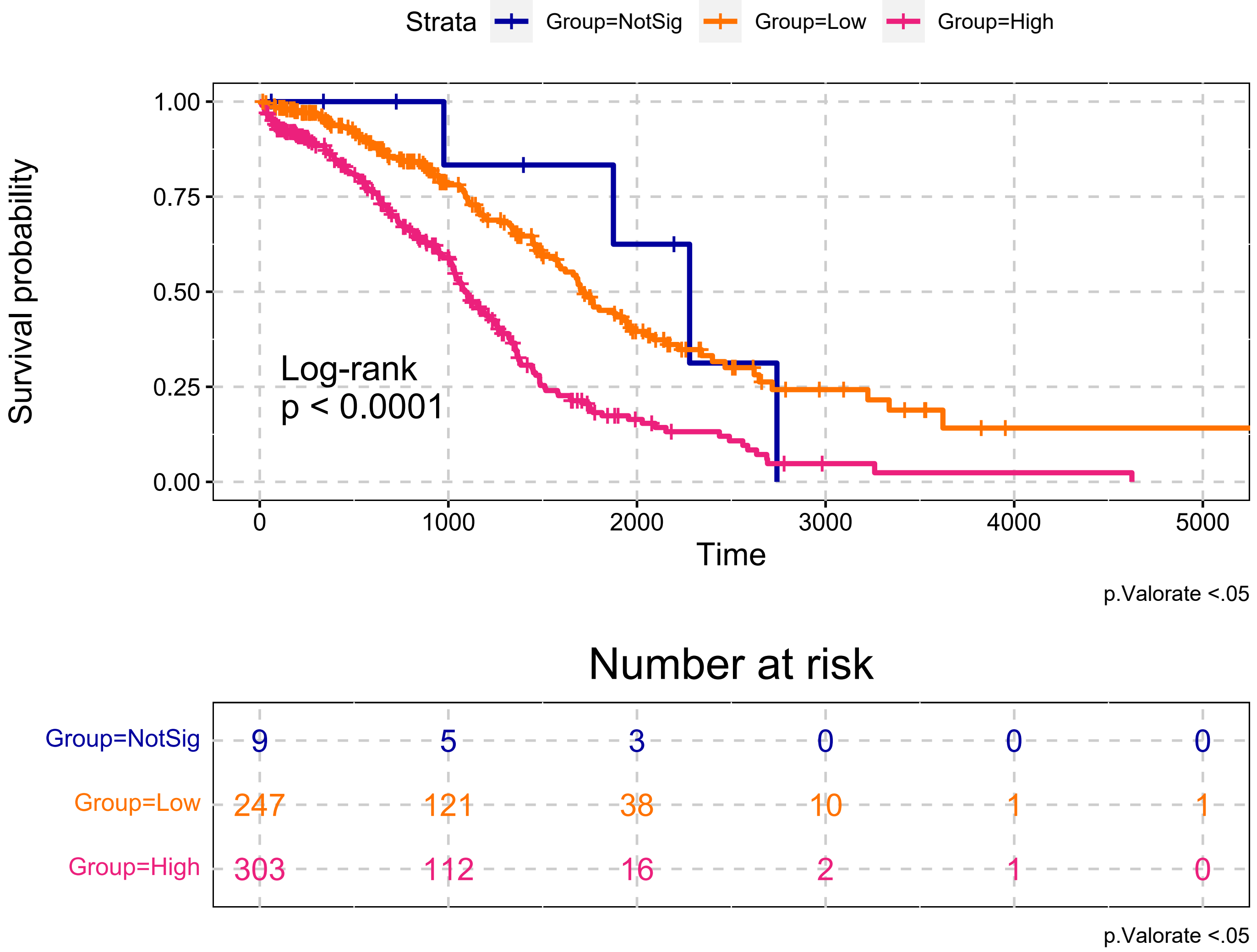

explanatory	beta	HR	L95	U95	p
Low	0.24	1.27	0.47	3.46	0.64
High	1.06	2.89	1.07	7.82	0.04

n= 559, number of events =287
Score(logrank) test = p <.0001

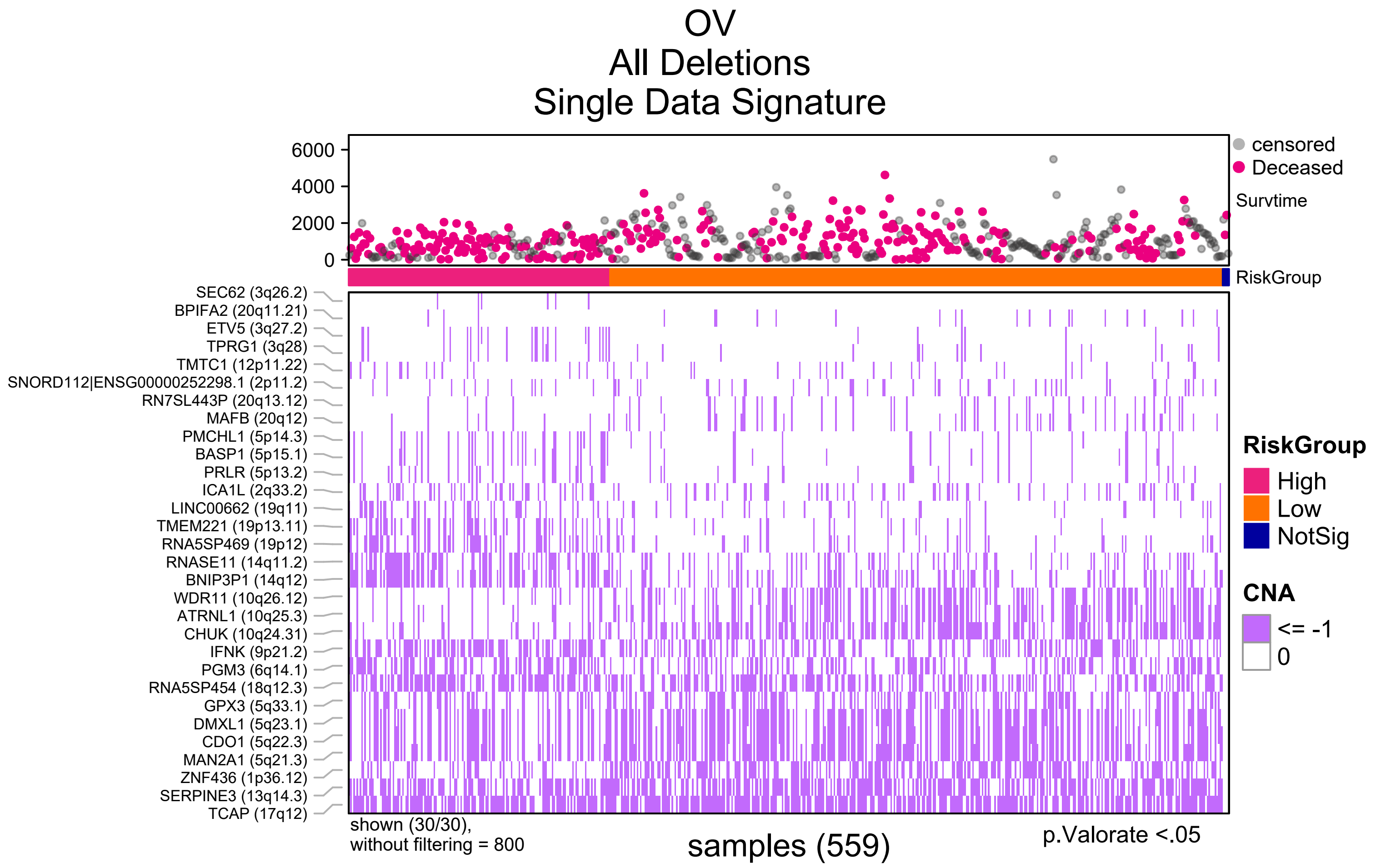
OV
All Deletions
Single Data Signature
6000
4000
2000
0
censored
Deceased
Survtime
RiskGroup
SEC62 (3q26.2)
BPIFA2 (20q11.21)
ETV5 (3q27.2)
TPRG1 (3q28)
TMTC1 (12p11.22)
SNORD112|ENSG00000252298.1 (2p11.2)
RN7SL443P (20q13.12)
MAFB (20q12)
PMCHL1 (5p14.3)
BASP1 (5p15.1)
PRLR (5p13.2)
ICA1L (2q33.2)
LINC00662 (19q11)
TMEM221 (19p13.11)
RNA5SP469 (19p12)
RNASE11 (14q11.2)
BNIP3P1 (14q12)
WDR11 (10q26.12)
ATRNL1 (10q25.3)
CHUK (10q24.31)
IFNK (9p21.2)
PGM3 (6q14.1)
RNA5SP454 (18q12.3)
GPX3 (5q33.1)
DMXL1 (5q23.1)
CDO1 (5q22.3)
MAN2A1 (5q21.3)
ZNF436 (1p36.12)
SERPINE3 (13q14.3)
TCAP (17q12)
RiskGroup
High
Low
NotSig
CNA
<= -1
0
shown (30/30),
without filtering = 800
samples (559)
p.Valorate <.05

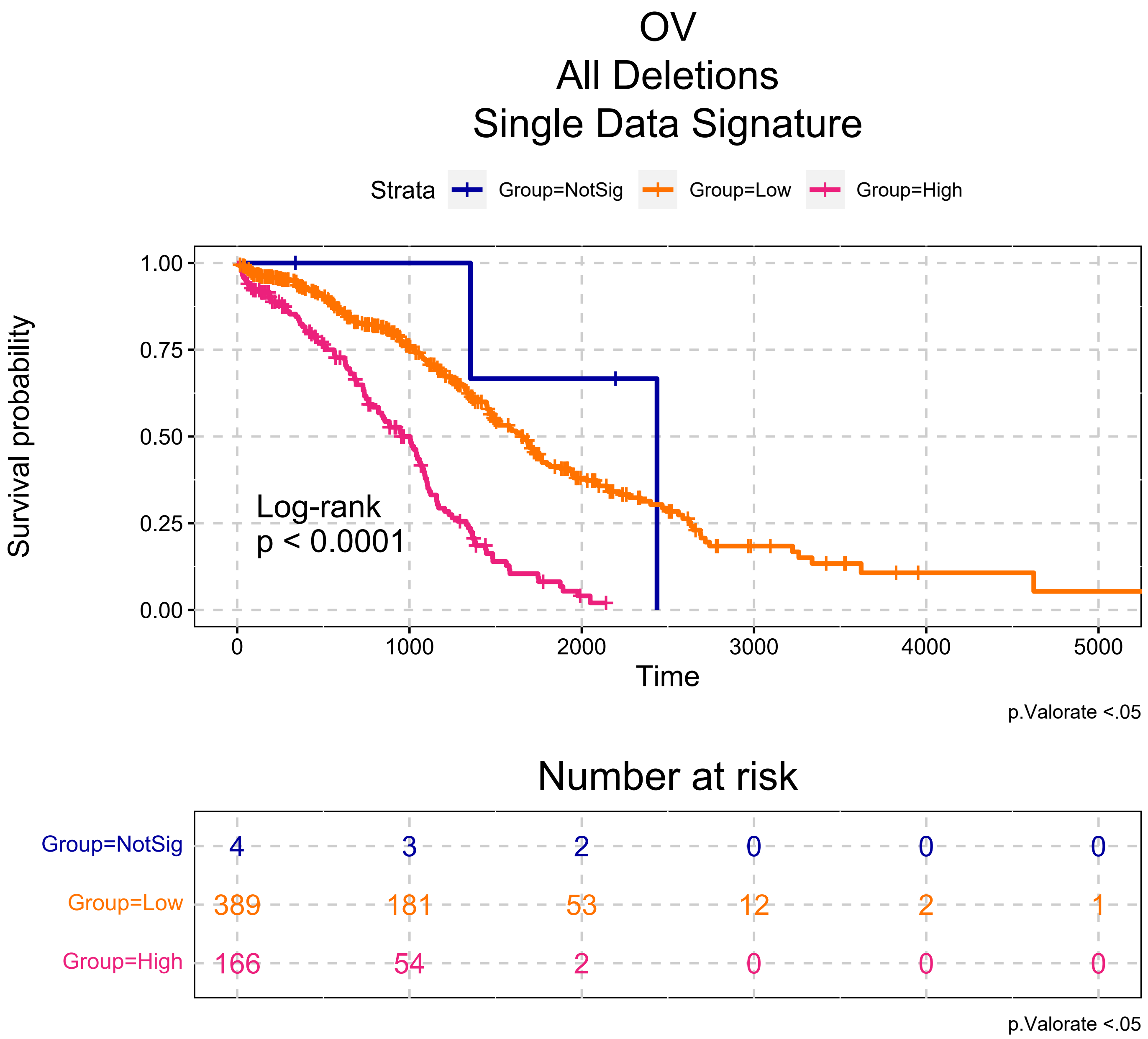

explanatory	beta	HR	L95	U95	p
Low	0.35	1.43	0.35	5.76	0.62
High	1.47	4.34	1.07	17.67	0.04

n= 559, number of events =287
Score(logrank) test = p <.0001

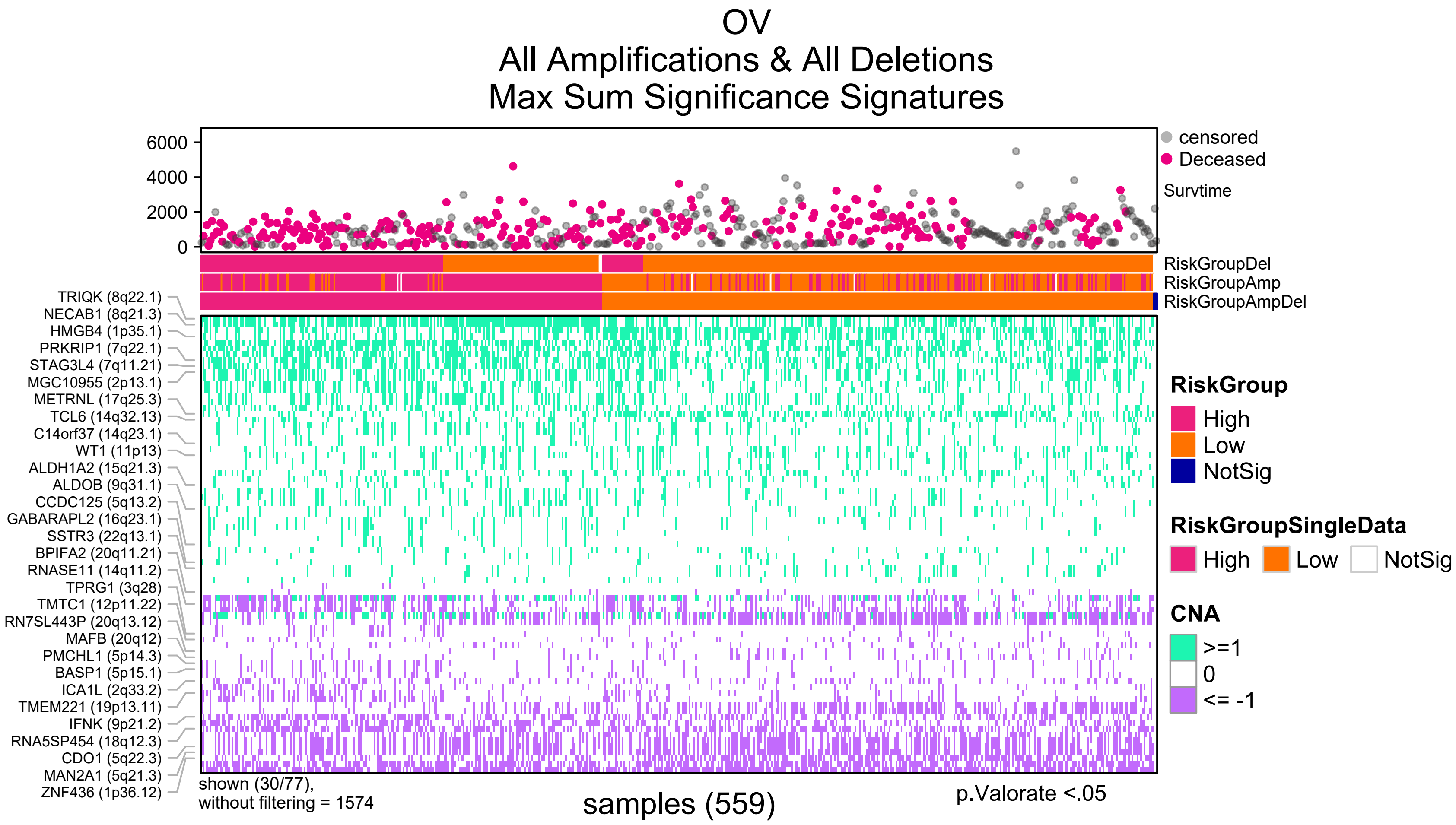
OV
All Amplifications & All Deletions
Max Sum Significance Signatures
6000
4000
2000
0
censored
Deceased
Survtime
RiskGroupDel
RiskGroupAmp
RiskGroupAmpDel
TRIQK (8q22.1)
NECAB1 (8q21.3)
HMGB4 (1p35.1)
PRKRIP1 (7q22.1)
STAG3L4 (7q11.21)
MGC10955 (2p13.1)
METRNL (17q25.3)
TCL6 (14q32.13)
C14orf37 (14q23.1)
WT1 (11p13)
ALDH1A2 (15q21.3)
ALDOB (9q31.1)
CCDC125 (5q13.2)
GABARAPL2 (16q23.1)
SSTR3 (22q13.1)
BPIFA2 (20q11.21)
RNASE11 (14q11.2)
TPRG1 (3q28)
TMTC1 (12p11.22)
RN7SL443P (20q13.12)
MAFB (20q12)
PMCHL1 (5p14.3)
BASP1 (5p15.1)
ICA1L (2q33.2)
TMEM221 (19p13.11)
IFNK (9p21.2)
RNA5SP454 (18q12.3)
CDO1 (5q22.3)
MAN2A1 (5q21.3)
ZNF436 (1p36.12)
shown (30/77),
without filtering = 1574
samples (559)
p.Valorate <.05
RiskGroup
High
Low
NotSig
RiskGroupSingleData
High
Low
NotSig
CNA
>=1
0
<= -1

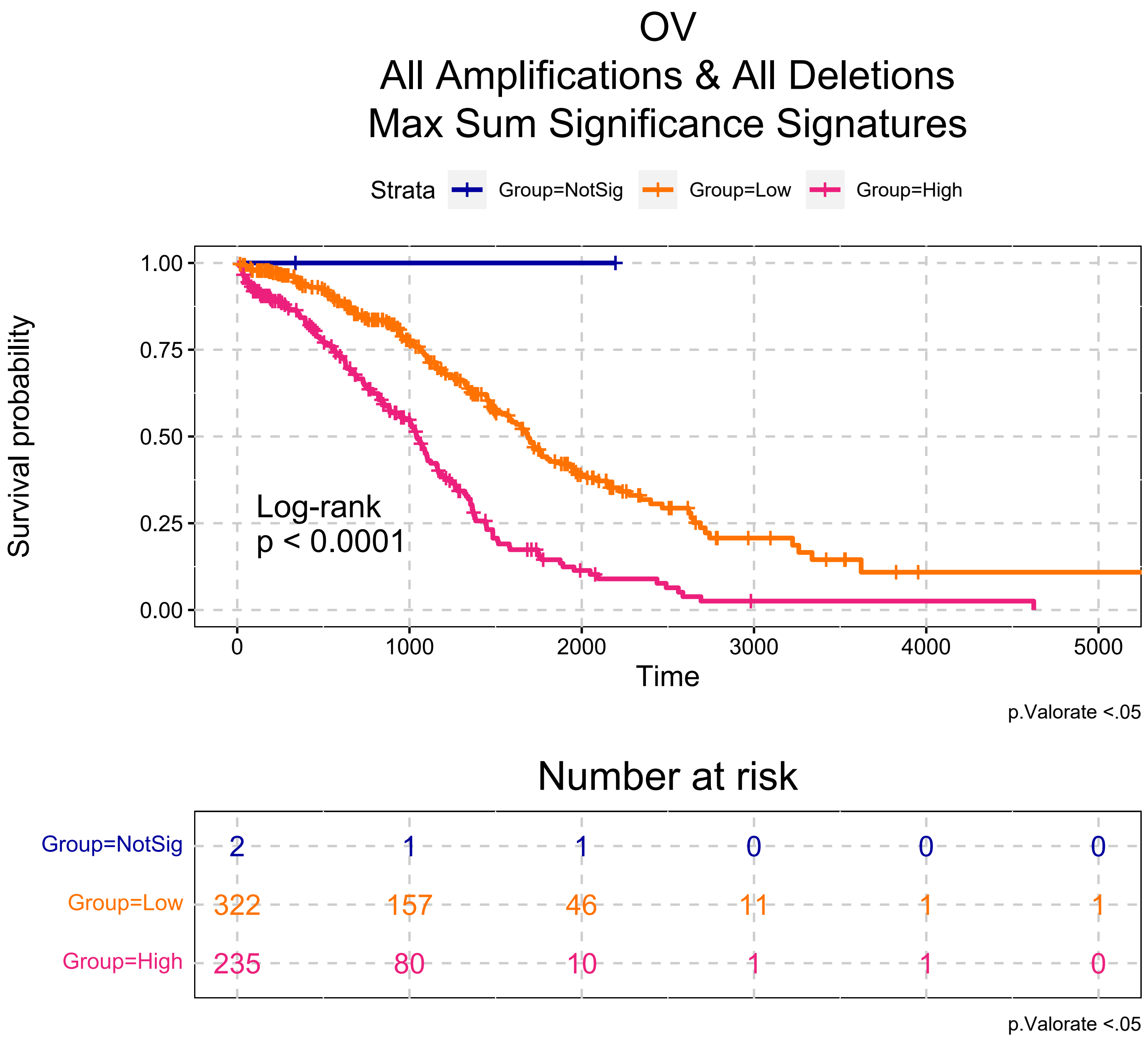

explanatory	beta	HR	L95	U95	p
Low	14.78	2632484.68	0.00	Inf	0.99
High	15.70	6565749.06	0.00	Inf	0.99

n= 559, number of events =287
Score(logrank) test = p <.0001

OV
All Amplifications & All Deletions
combining signatures

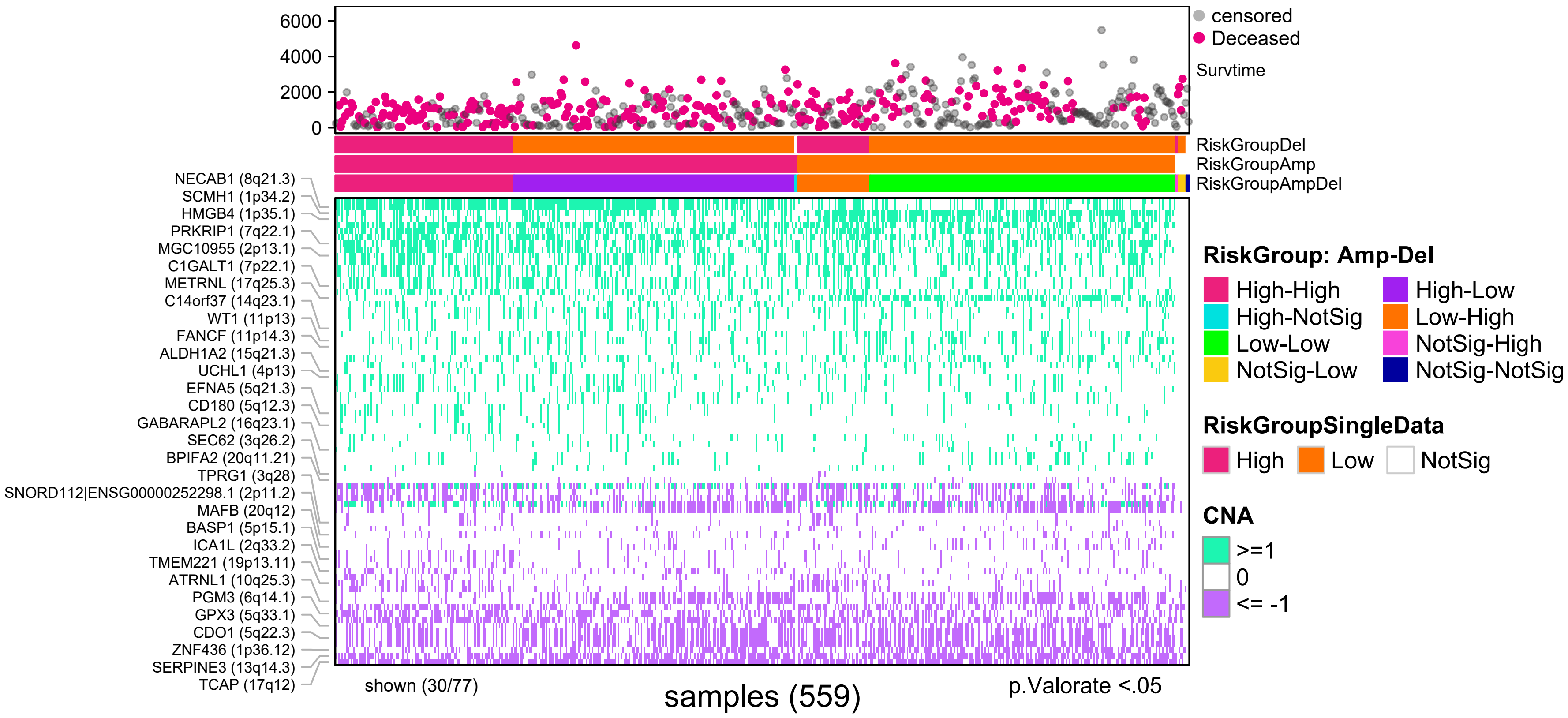

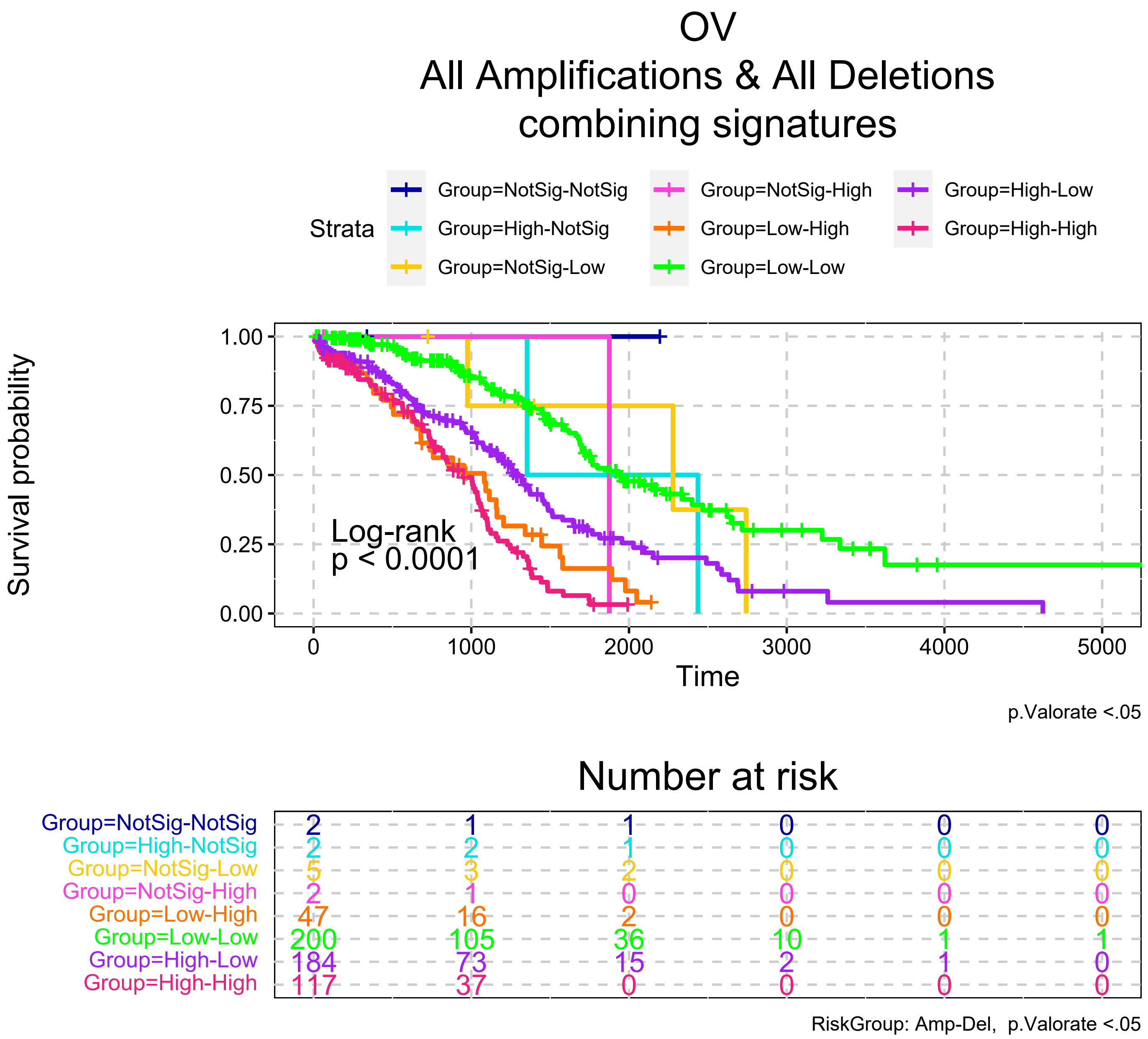

explanatory	beta	HR	L95	U95	p
High-NotSig	15.09	3585149.77	0.00	Inf	0.99
NotSig-Low	14.69	2408391.28	0.00	Inf	0.99
NotSig-High	15.05	3423795.55	0.00	Inf	0.99
Low-High	15.94	8337222.84	0.00	Inf	0.99
Low-Low	14.54	2072744.15	0.00	Inf	0.99
High-Low	15.41	4919901.60	0.00	Inf	0.99
High-High	16.19	10710093.35	0.00	Inf	0.99

n= 559, number of events =287
Score(logrank) test = p <.0001

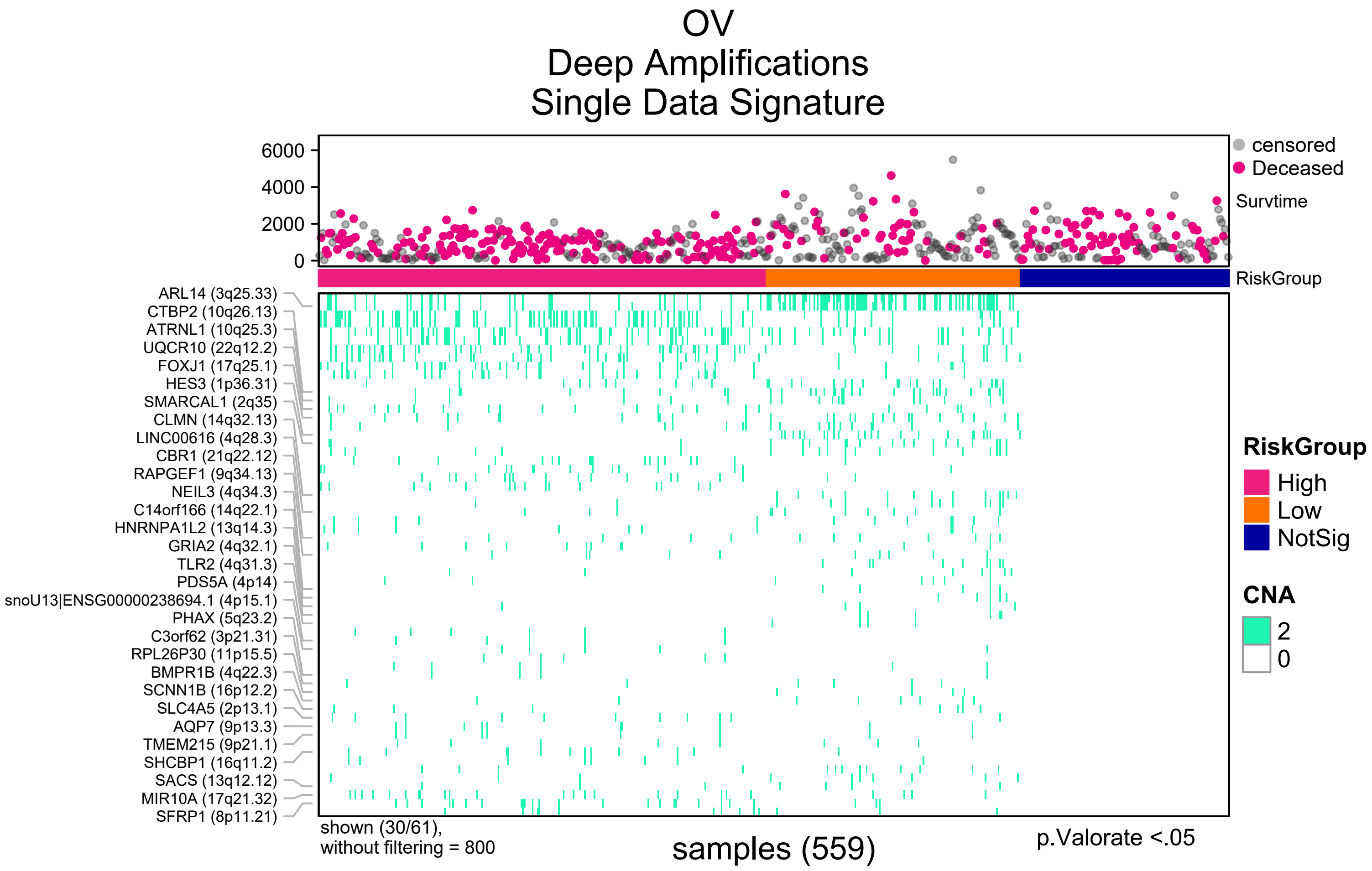

OV
Deep Amplifications
Single Data Signature
6000
4000
2000
0
censored
Deceased
Survtime
RiskGroup
ARL14 (3q25.33)
CTBP2 (10q26.13)
ATRNL1 (10q25.3)
UQCR10 (22q12.2)
FOXJ1 (17q25.1)
HES3 (1p36.31)
SMARCAL1 (2q35)
CLMN (14q32.13)
LINC00616 (4q28.3)
CBR1 (21q22.12)
RAPGEF1 (9q34.13)
NEIL3 (4q34.3)
C14orf166 (14q22.1)
HNRNPA1L2 (13q14.3)
GRIA2 (4q32.1)
TLR2 (4q31.3)
PDS5A (4p14)
snoU13|ENSG00000238694.1 (4p15.1)
PHAX (5q23.2)
C3orf62 (3p21.31)
RPL26P30 (11p15.5)
BMPR1B (4q22.3)
SCNN1B (16p12.2)
SLC4A5 (2p13.1)
AQP7 (9p13.3)
TMEM215 (9p21.1)
SHCBP1 (16q11.2)
SACS (13q12.12)
MIR10A (17q21.32)
SFRP1 (8p11.21)
RiskGroup
High
Low
NotSig
CNA
2
0
shown (30/61),
without filtering = 800
samples (559)
p.Valorate <.05

OV
Deep Amplifications
Single Data Signature

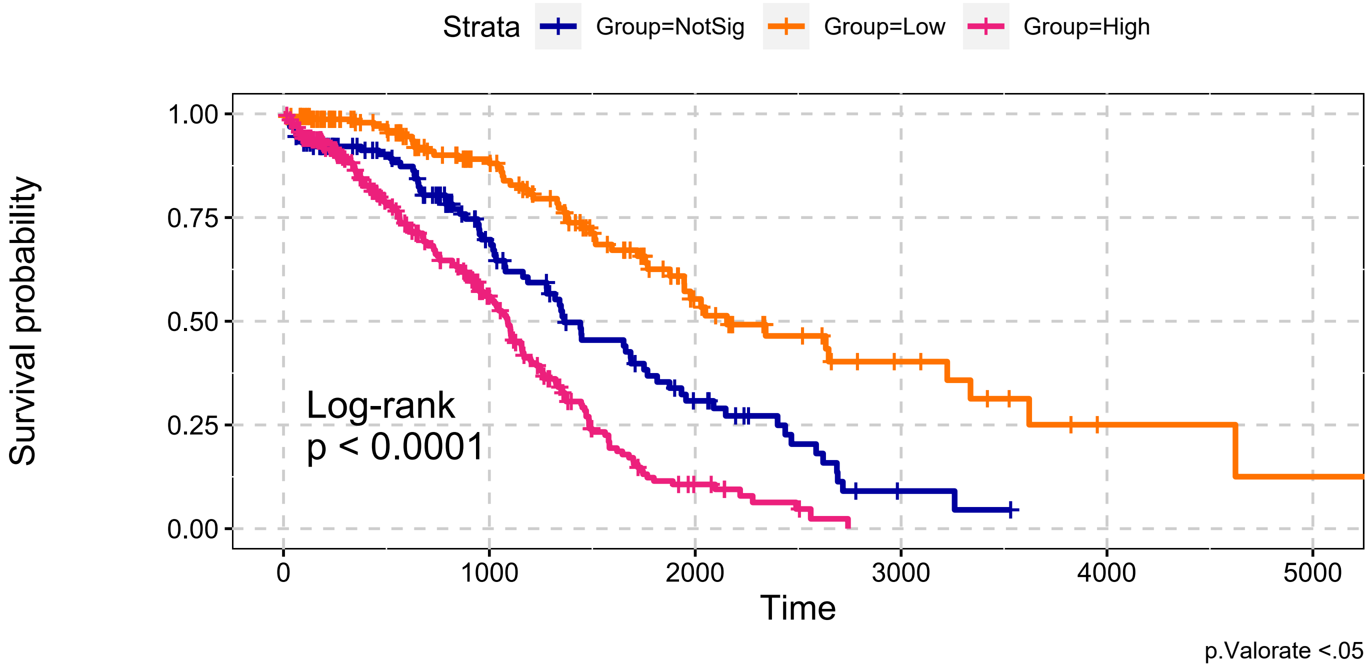

Number at risk

	0	1000	2000	3000	4000	5000
Group=NotSig	128	55	19	2	0	0
Group=Low	156	87	28	10	2	1
Group=High	275	96	10	0	0	0

p.Valorate <.05

explanatory	beta	HR	L95	U95	p
Low	-0.85	0.43	0.30	0.62	0.00
High	0.60	1.82	1.37	2.43	0.00

n= 559, number of events =287
Score(logrank) test = p <.0001

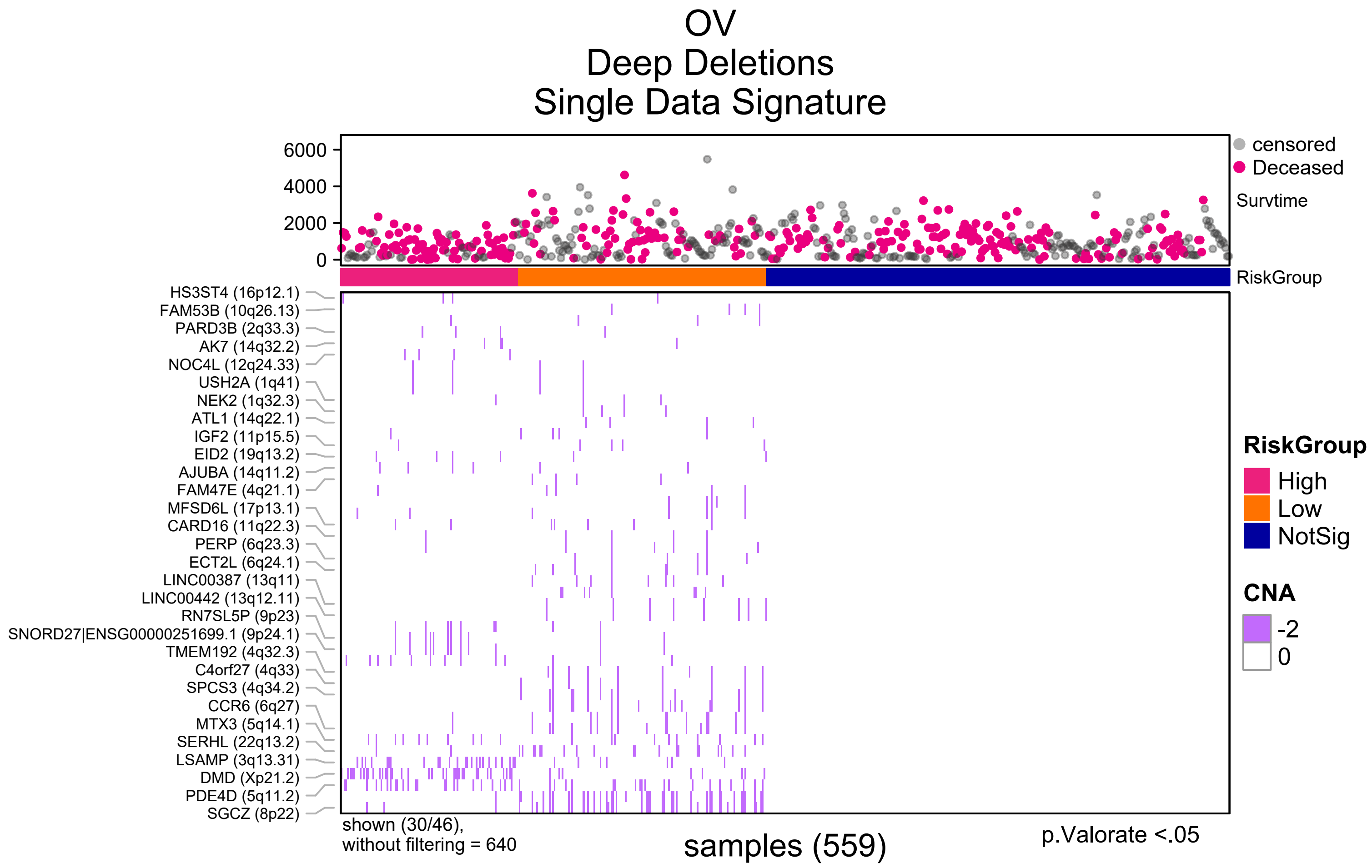
OV
Deep Deletions
Single Data Signature
6000
4000
2000
0
censored
Deceased
Survtime
RiskGroup
HS3ST4 (16p12.1)
FAM53B (10q26.13)
PARD3B (2q33.3)
AK7 (14q32.2)
NOC4L (12q24.33)
USH2A (1q41)
NEK2 (1q32.3)
ATL1 (14q22.1)
IGF2 (11p15.5)
EID2 (19q13.2)
AJUBA (14q11.2)
FAM47E (4q21.1)
MFSD6L (17p13.1)
CARD16 (11q22.3)
PERP (6q23.3)
ECT2L (6q24.1)
LINC00387 (13q11)
LINC00442 (13q12.11)
RN7SL5P (9p23)
SNORD27|ENSG00000251699.1 (9p24.1)
TMEM192 (4q32.3)
C4orf27 (4q33)
SPCS3 (4q34.2)
CCR6 (6q27)
MTX3 (5q14.1)
SERHL (22q13.2)
LSAMP (3q13.31)
DMD (Xp21.2)
PDE4D (5q11.2)
SGCZ (8p22)
RiskGroup
High
Low
NotSig
CNA
-2
0
shown (30/46),
without filtering = 640
samples (559)
p.Valorate <.05

OV
Deep Deletions
Single Data Signature

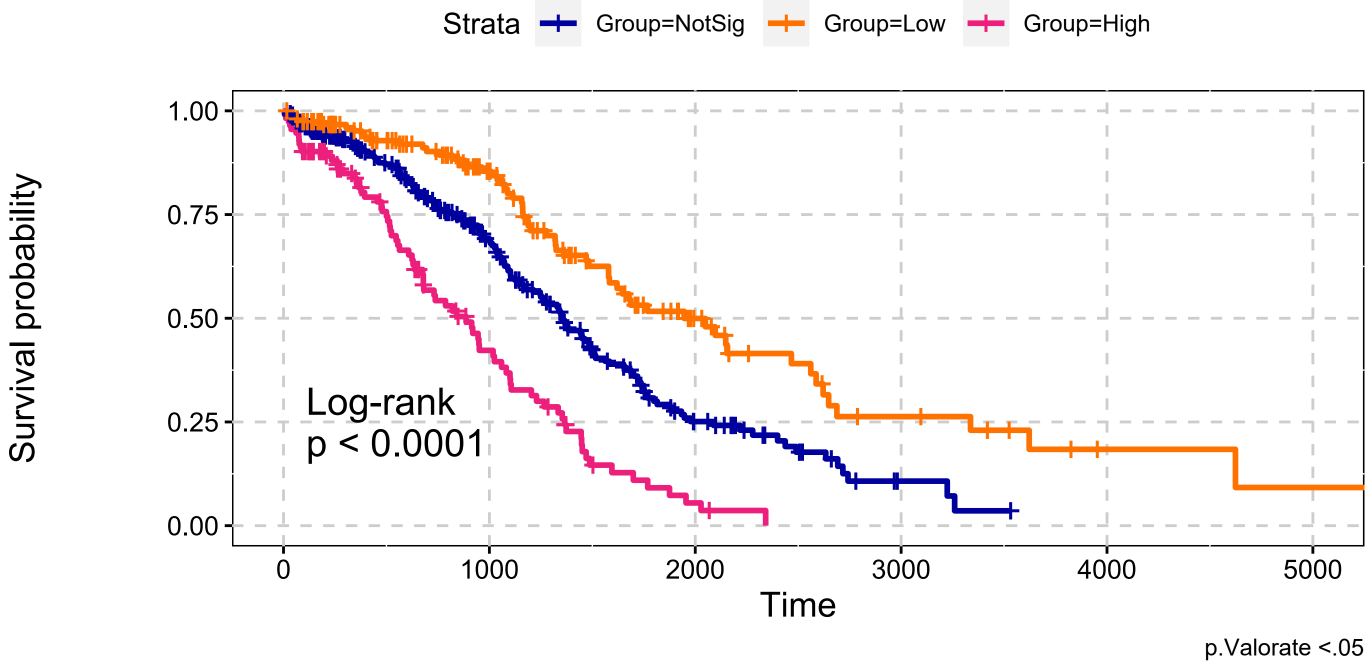

Number at risk

	0	1000	2000	3000	4000	5000
Group=NotSig	291	126	28	3	0	0
Group=Low	156	81	26	9	2	1
Group=High	112	31	3	0	0	0

p.Valorate <.05

explanatory	beta	HR	L95	U95	p
Low	-0.61	0.54	0.40	0.74	0.00
High	0.78	2.19	1.65	2.89	0.00

n= 559, number of events =287
Score(logrank) test = p <.0001

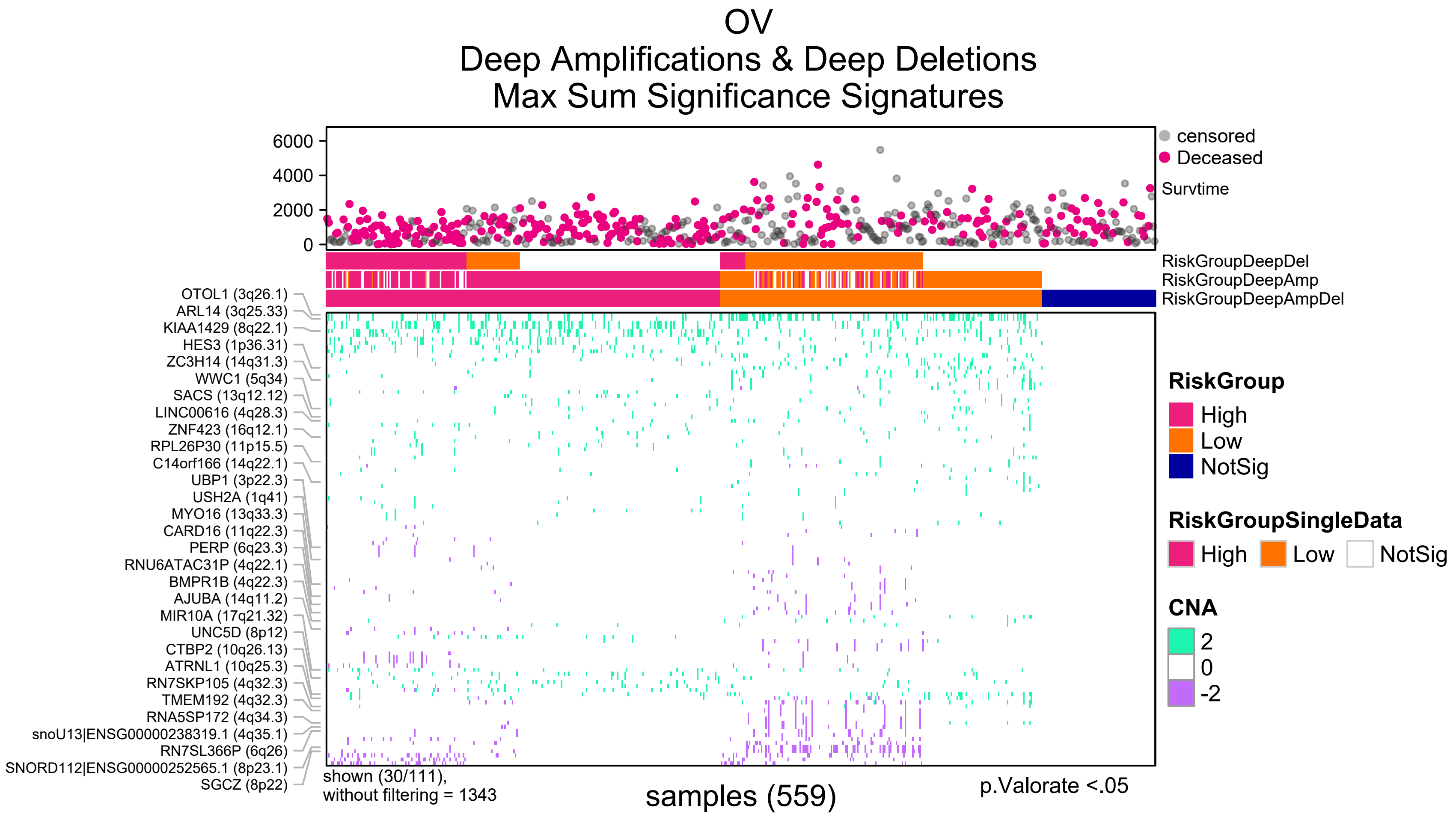

OV
Deep Amplifications & Deep Deletions
Max Sum Significance Signatures
6000
4000
2000
0
censored
Deceased
Survtime
RiskGroupDeepDel
RiskGroupDeepAmp
RiskGroupDeepAmpDel
OTOL1 (3q26.1)
ARL14 (3q25.33)
KIAA1429 (8q22.1)
HES3 (1p36.31)
ZC3H14 (14q31.3)
WWC1 (5q34)
SACS (13q12.12)
LINC00616 (4q28.3)
ZNF423 (16q12.1)
RPL26P30 (11p15.5)
C14orf166 (14q22.1)
UBP1 (3p22.3)
USH2A (1q41)
MYO16 (13q33.3)
CARD16 (11q22.3)
PERP (6q23.3)
RNU6ATAC31P (4q22.1)
BMPR1B (4q22.3)
AJUBA (14q11.2)
MIR10A (17q21.32)
UNC5D (8p12)
CTBP2 (10q26.13)
ATRNL1 (10q25.3)
RN7SKP105 (4q32.3)
TMEM192 (4q32.3)
RNA5SP172 (4q34.3)
snoU13|ENSG00000238319.1 (4q35.1)
RN7SL366P (6q26)
SNORD112|ENSG00000252565.1 (8p23.1)
SGCZ (8p22)
RiskGroup
High
Low
NotSig
RiskGroupSingleData
High
Low
NotSig
CNA
2
0
-2
shown (30/111),
without filtering = 1343
samples (559)
p.Valorate <.05

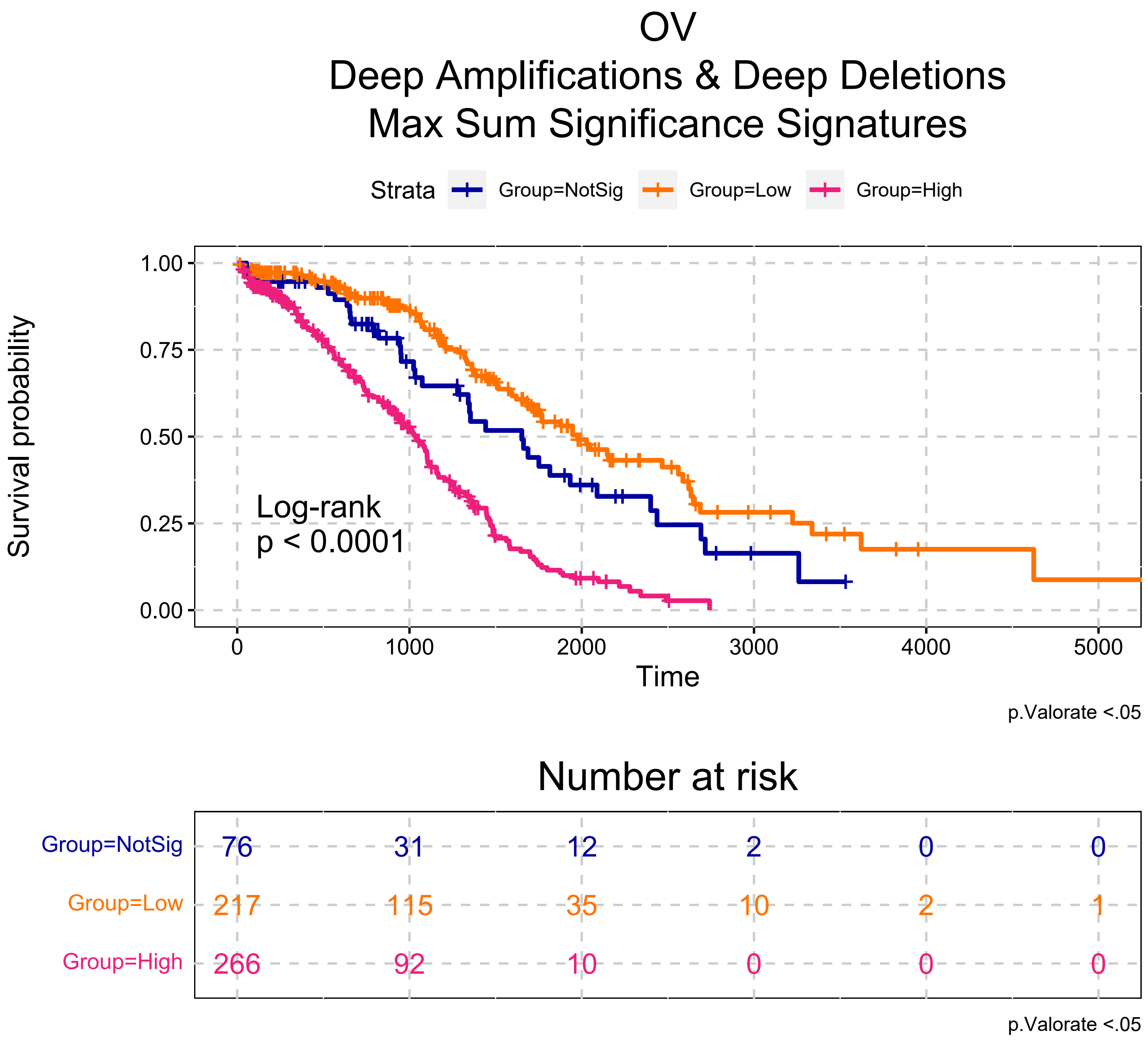

explanatory	beta	HR	L95	U95	p
Low	-0.42	0.66	0.44	0.97	0.04
High	0.85	2.33	1.62	3.37	0.00

n= 559, number of events =287
Score(logrank) test = p <.0001

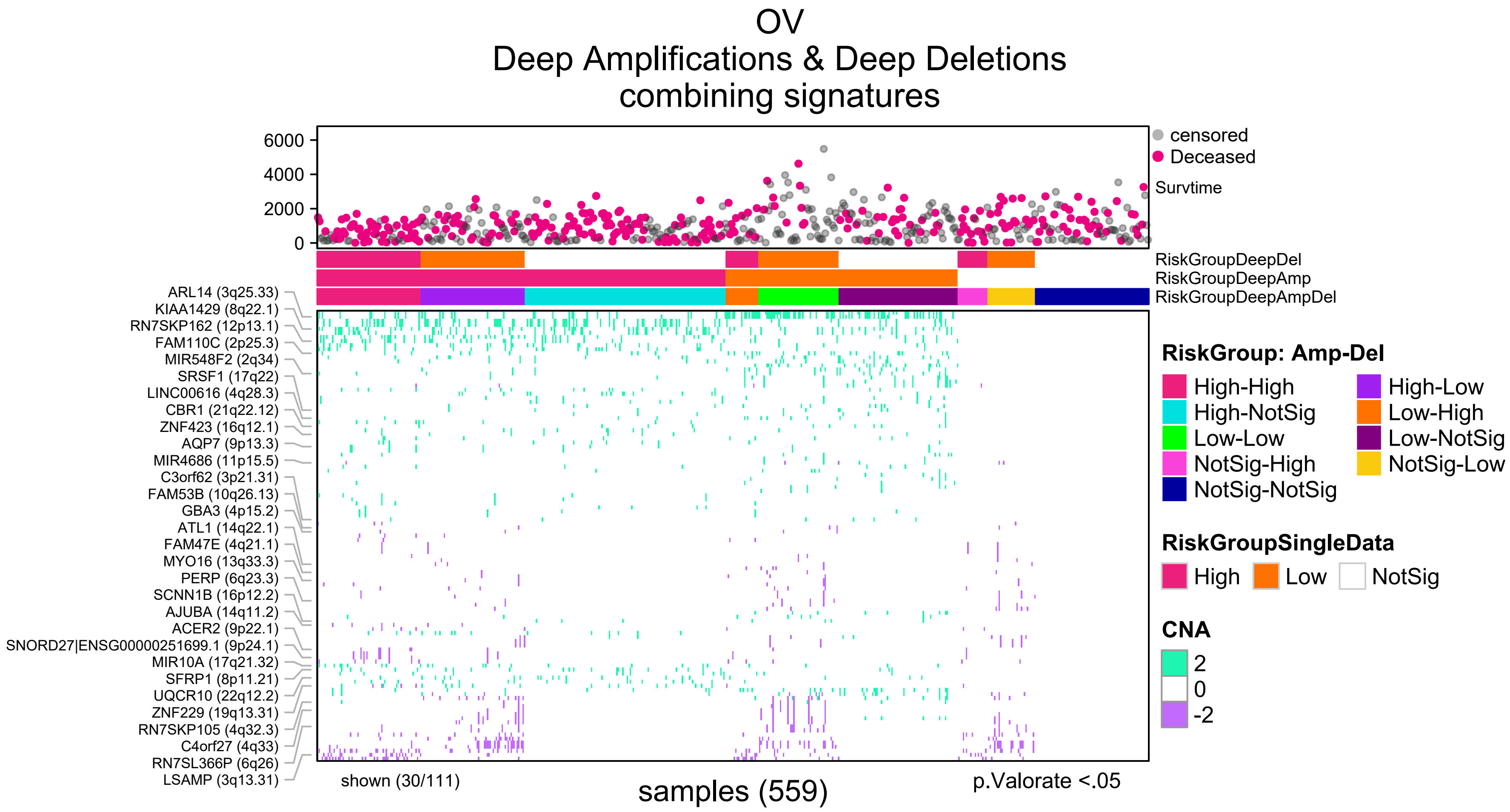

OV
Deep Amplifications & Deep Deletions
combining signatures
6000
4000
2000
0
censored
Deceased
Survtime
RiskGroupDeepDel
RiskGroupDeepAmp
RiskGroupDeepAmpDel
ARL14 (3q25.33)
KIAA1429 (8q22.1)
RN7SKP162 (12p13.1)
FAM110C (2p25.3)
MIR548F2 (2q34)
SRSF1 (17q22)
LINC00616 (4q28.3)
CBR1 (21q22.12)
ZNF423 (16q12.1)
AQP7 (9p13.3)
MIR4686 (11p15.5)
C3orf62 (3p21.31)
FAM53B (10q26.13)
GBA3 (4p15.2)
ATL1 (14q22.1)
FAM47E (4q21.1)
MYO16 (13q33.3)
PERP (6q23.3)
SCNN1B (16p12.2)
AJUBA (14q11.2)
ACER2 (9p22.1)
SNORD27|ENSG00000251699.1 (9p24.1)
MIR10A (17q21.32)
SFRP1 (8p11.21)
UQCR10 (22q12.2)
ZNF229 (19q13.31)
RN7SKP105 (4q32.3)
C4orf27 (4q33)
RN7SL366P (6q26)
LSAMP (3q13.31)
shown (30/111)
samples (559)
p.Valorate <.05
RiskGroup: Amp-Del
High-High
High-Low
High-NotSig
Low-High
Low-Low
Low-NotSig
NotSig-High
NotSig-Low
NotSig-NotSig
RiskGroupSingleData
High
Low
NotSig
CNA
2
0
-2

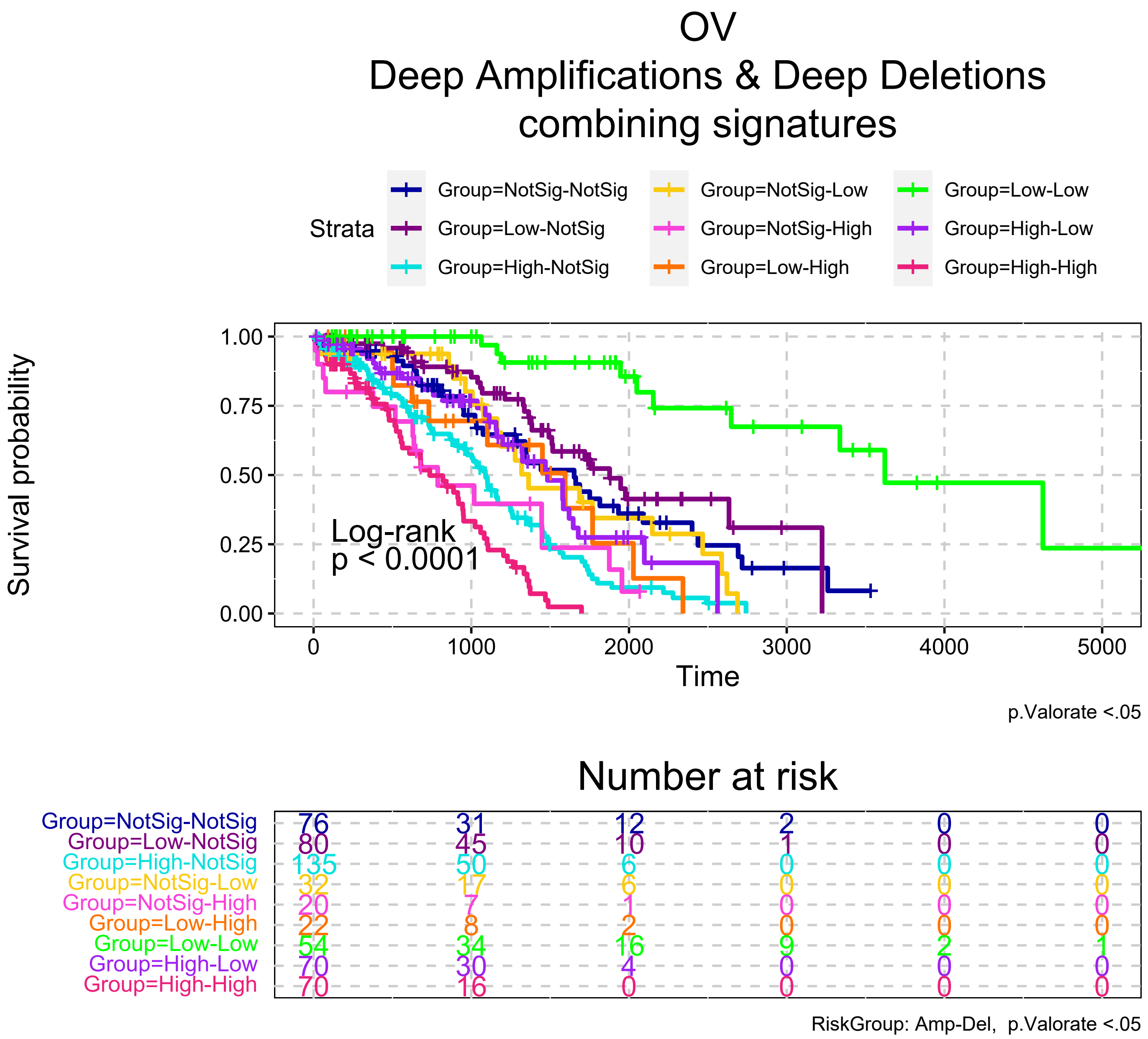

explanatory	beta	HR	L95	U95	p
Low-NotSig	-0.31	0.73	0.45	1.21	0.22
High-NotSig	0.86	2.35	1.57	3.52	0.00
NotSig-Low	0.21	1.24	0.71	2.17	0.46
NotSig-High	0.95	2.59	1.41	4.79	0.00
Low-High	0.45	1.57	0.79	3.11	0.20
Low-Low	-1.80	0.16	0.08	0.36	0.00
High-Low	0.32	1.38	0.84	2.26	0.20
High-High	1.52	4.57	2.91	7.18	0.00

n= 559, number of events =287
Score(logrank) test = p <.0001
