# Supplementary material for: Systematic Modeling of Risk-Associated Copy Number Alterations in Cancer
Source: Int J Mol Sci. 2024 Sep 27;25(19):10455. doi: 10.3390/ijms251910455 (PMC11477427; doi:10.3390/ijms251910455)
Supplement: Supplementary file 1 [file ijms-25-10455-s001.zip › PAADSignatureV12-sinSombreado.pdf]

PAAD
All Amplifications
Single Data Signature

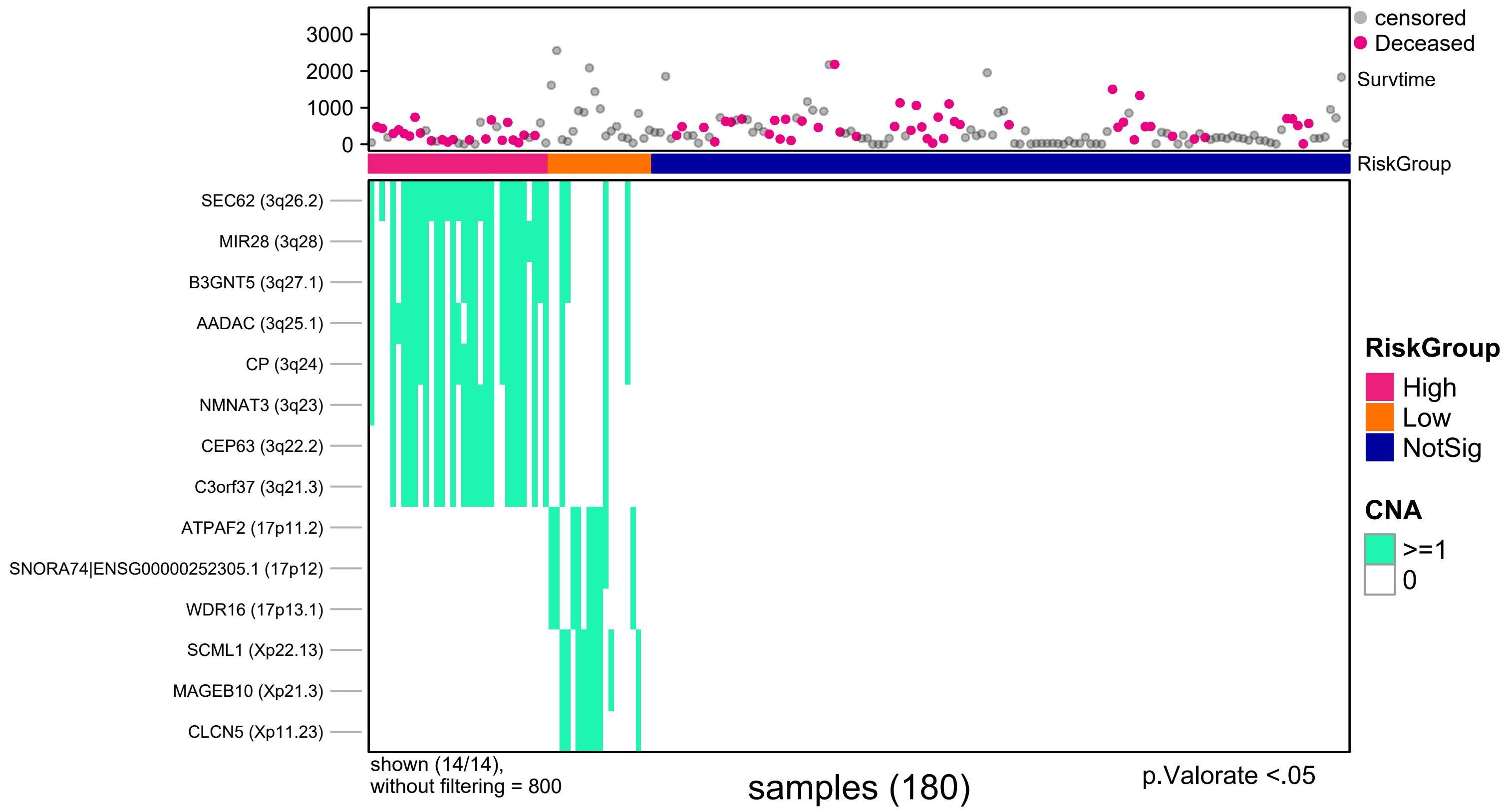

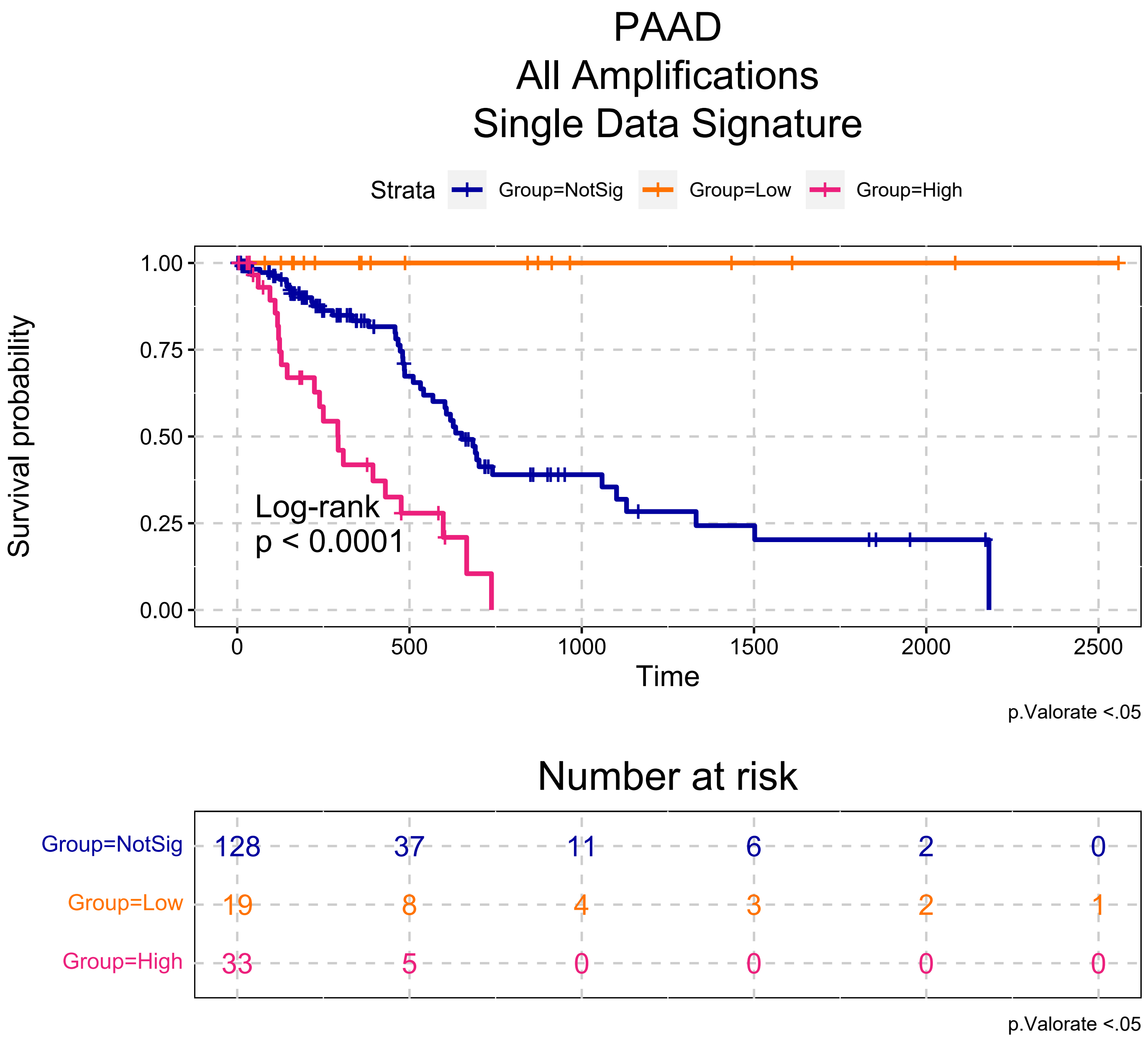

explanatory	beta	HR	L95	U95	p
Low	-18.44	0.00	0.00	Inf	0.99
High	1.25	3.49	2.02	6.03	0.00

n= 180, number of events =66
Score(logrank) test = p <.0001

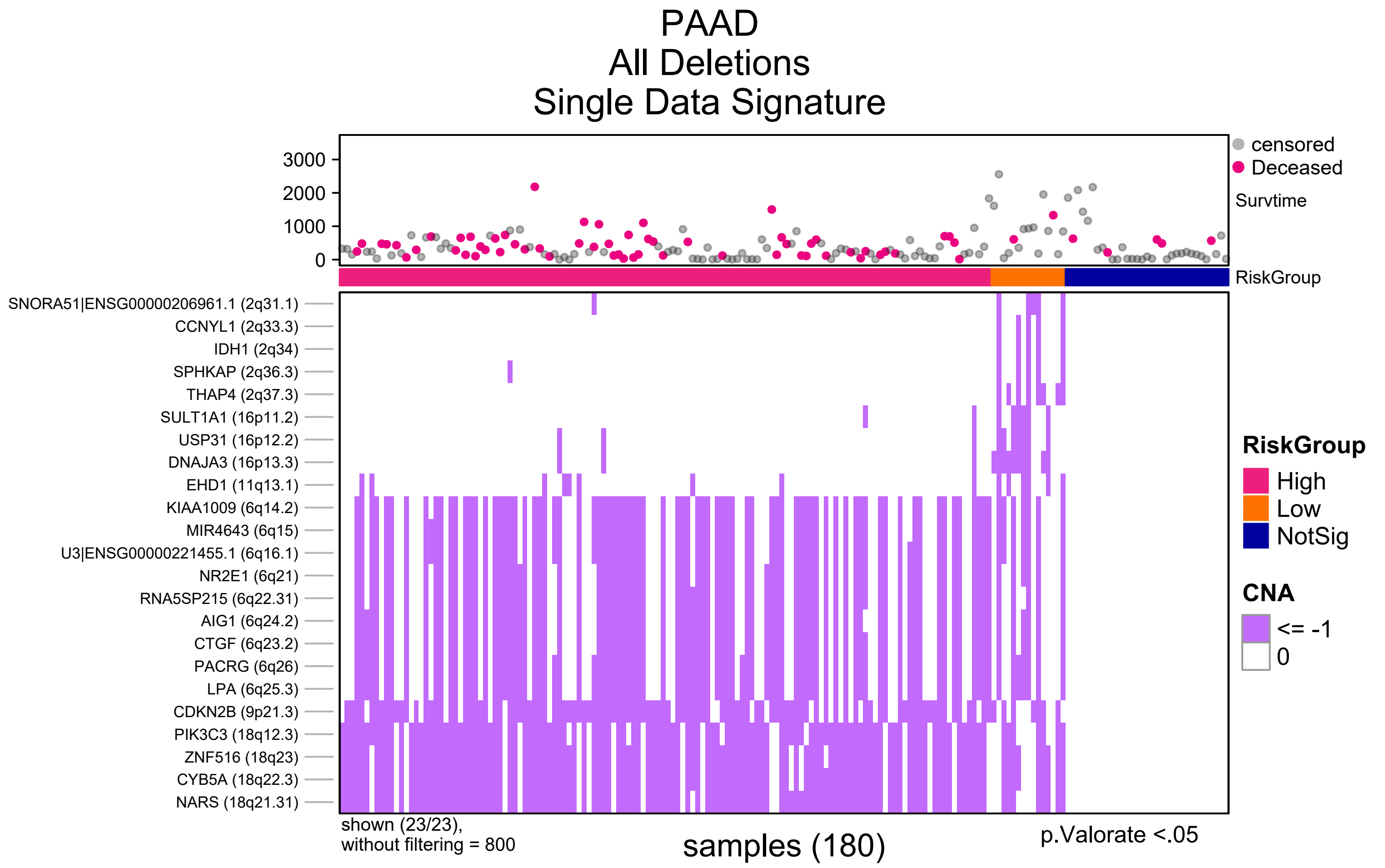

PAAD
All Deletions
Single Data Signature
3000
2000
1000
0
censored
Deceased
Survtime
RiskGroup
SNORA51|ENSG00000206961.1 (2q31.1)
CCNYL1 (2q33.3)
IDH1 (2q34)
SPHKAP (2q36.3)
THAP4 (2q37.3)
SULT1A1 (16p11.2)
USP31 (16p12.2)
DNAJA3 (16p13.3)
EHD1 (11q13.1)
KIAA1009 (6q14.2)
MIR4643 (6q15)
U3|ENSG00000221455.1 (6q16.1)
NR2E1 (6q21)
RNA5SP215 (6q22.31)
AIG1 (6q24.2)
CTGF (6q23.2)
PACRG (6q26)
LPA (6q25.3)
CDKN2B (9p21.3)
PIK3C3 (18q12.3)
ZNF516 (18q23)
CYB5A (18q22.3)
NARS (18q21.31)
RiskGroup
High
Low
NotSig
CNA
<= -1
0
shown (23/23),
without filtering = 800
samples (180)
p.Valorate <.05

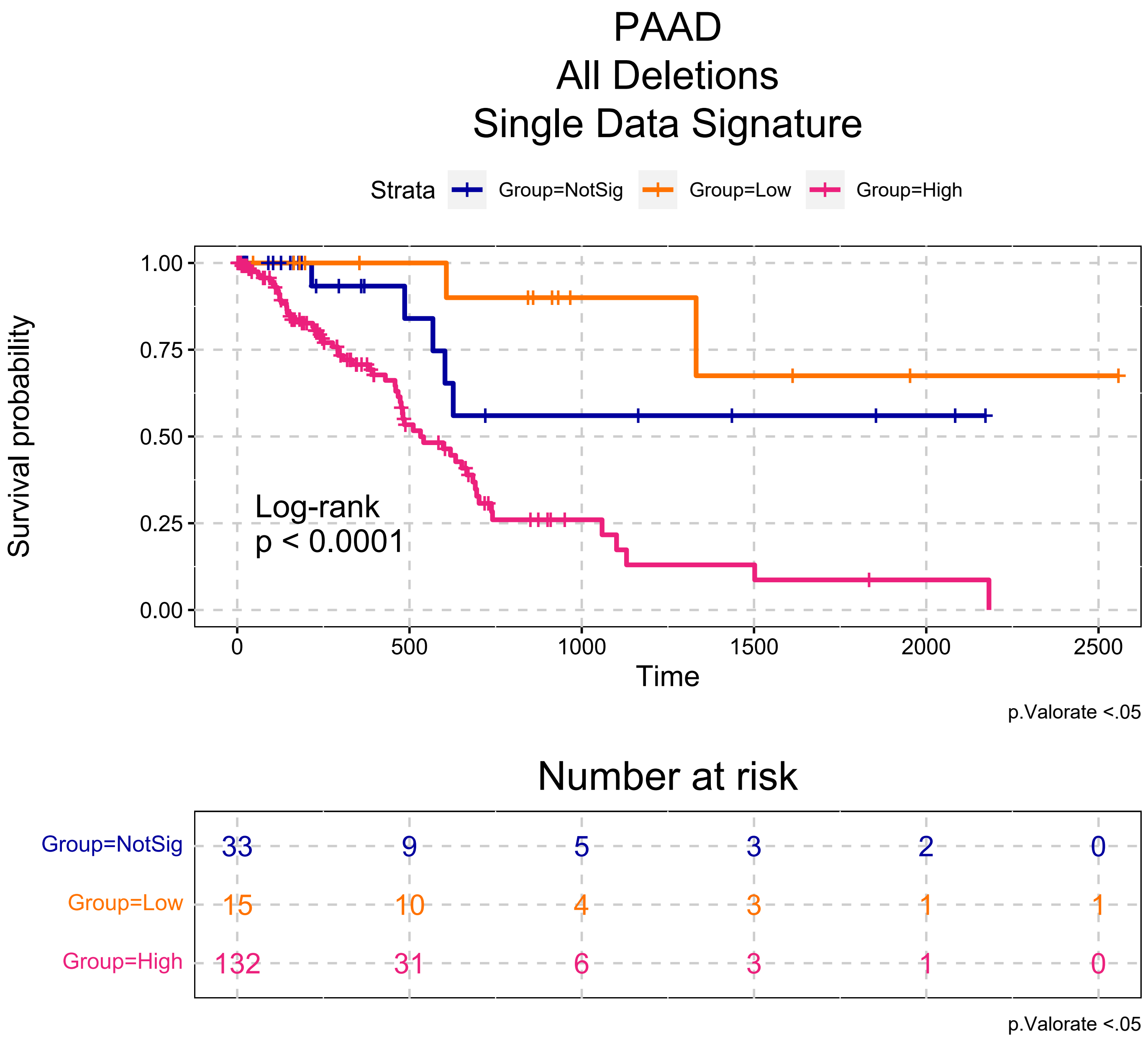

explanatory	beta	HR	L95	U95	p
Low	-0.96	0.38	0.07	1.97	0.25
High	1.24	3.46	1.37	8.74	0.01

n= 180, number of events =66
Score(logrank) test = p <.0001

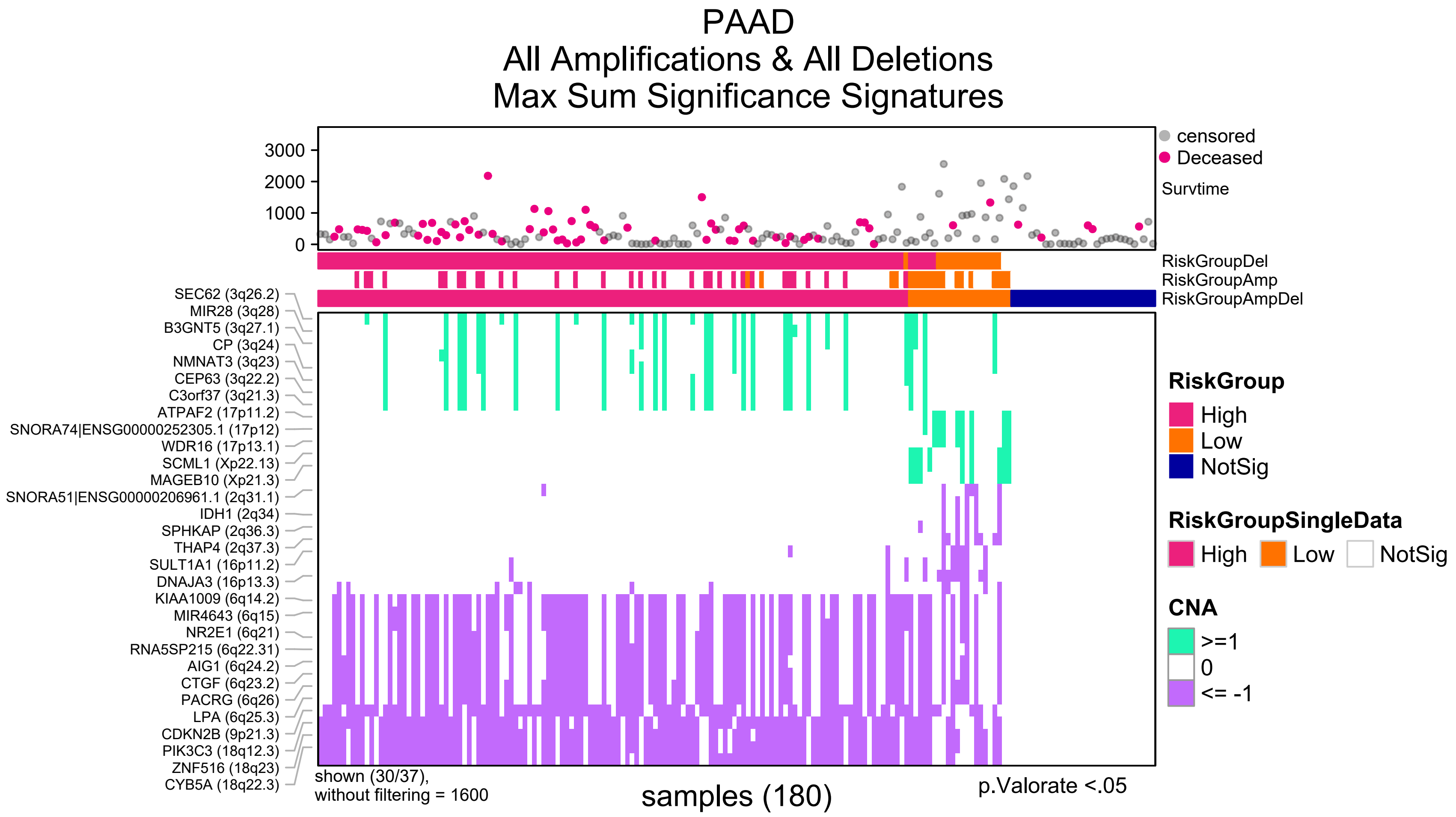

PAAD
All Amplifications & All Deletions
Max Sum Significance Signatures
3000
2000
1000
0
censored
Deceased
Survtime
RiskGroupDel
RiskGroupAmp
RiskGroupAmpDel
SEC62 (3q26.2)
MIR28 (3q28)
B3GNT5 (3q27.1)
CP (3q24)
NMNAT3 (3q23)
CEP63 (3q22.2)
C3orf37 (3q21.3)
ATPAF2 (17p11.2)
SNORA74|ENSG00000252305.1 (17p12)
WDR16 (17p13.1)
SCML1 (Xp22.13)
MAGEB10 (Xp21.3)
SNORA51|ENSG00000206961.1 (2q31.1)
IDH1 (2q34)
SPHKAP (2q36.3)
THAP4 (2q37.3)
SULT1A1 (16p11.2)
DNAJA3 (16p13.3)
KIAA1009 (6q14.2)
MIR4643 (6q15)
NR2E1 (6q21)
RNA5SP215 (6q22.31)
AIG1 (6q24.2)
CTGF (6q23.2)
PACRG (6q26)
LPA (6q25.3)
CDKN2B (9p21.3)
PIK3C3 (18q12.3)
ZNF516 (18q23)
CYB5A (18q22.3)
RiskGroup
High
Low
NotSig
RiskGroupSingleData
High
Low
NotSig
CNA
>=1
0
<= -1
shown (30/37),
without filtering = 1600
samples (180)
p.Valorate <.05

PAAD
All Amplifications & All Deletions
Max Sum Significance Signatures

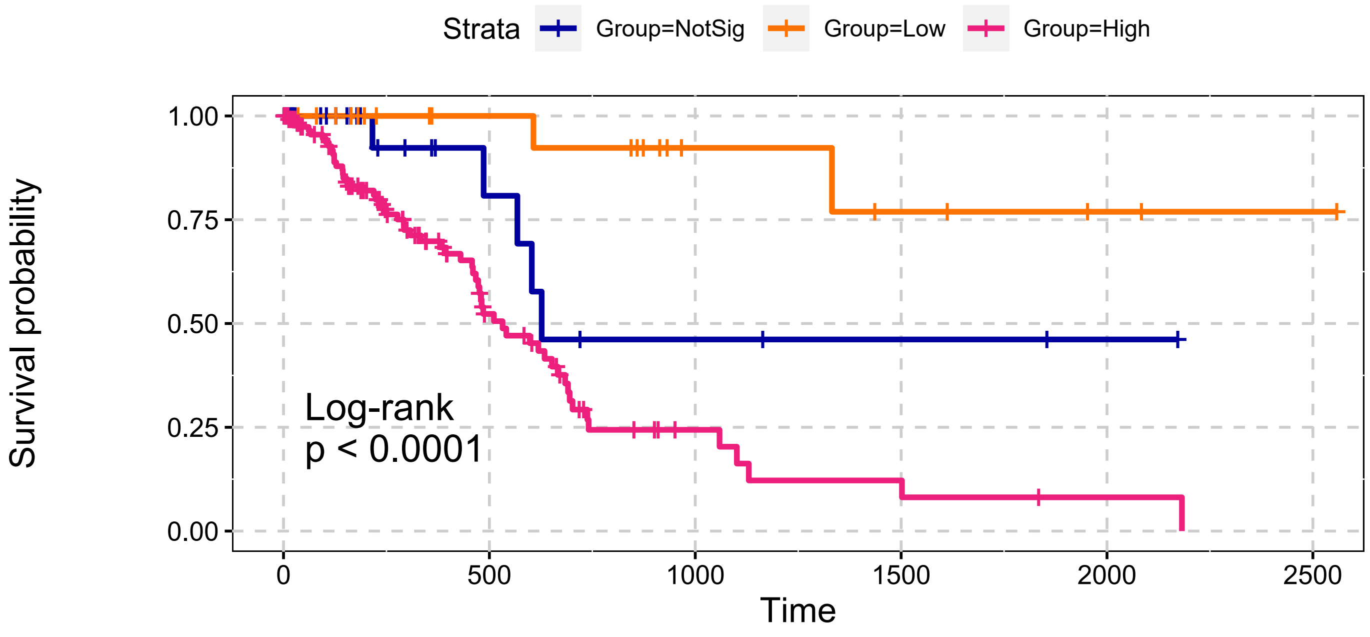

p.Valorate <.05

Number at risk

	0	500	1000	1500	2000	2500
Group=NotSig	31	7	3	2	1	0
Group=Low	22	13	6	4	2	1
Group=High	127	30	6	3	1	0

p.Valorate <.05

explanatory	beta	HR	L95	U95	p
Low	-1.55	0.21	0.04	1.10	0.06
High	1.00	2.71	1.08	6.80	0.03

n= 180, number of events =66
Score(logrank) test = p <.0001

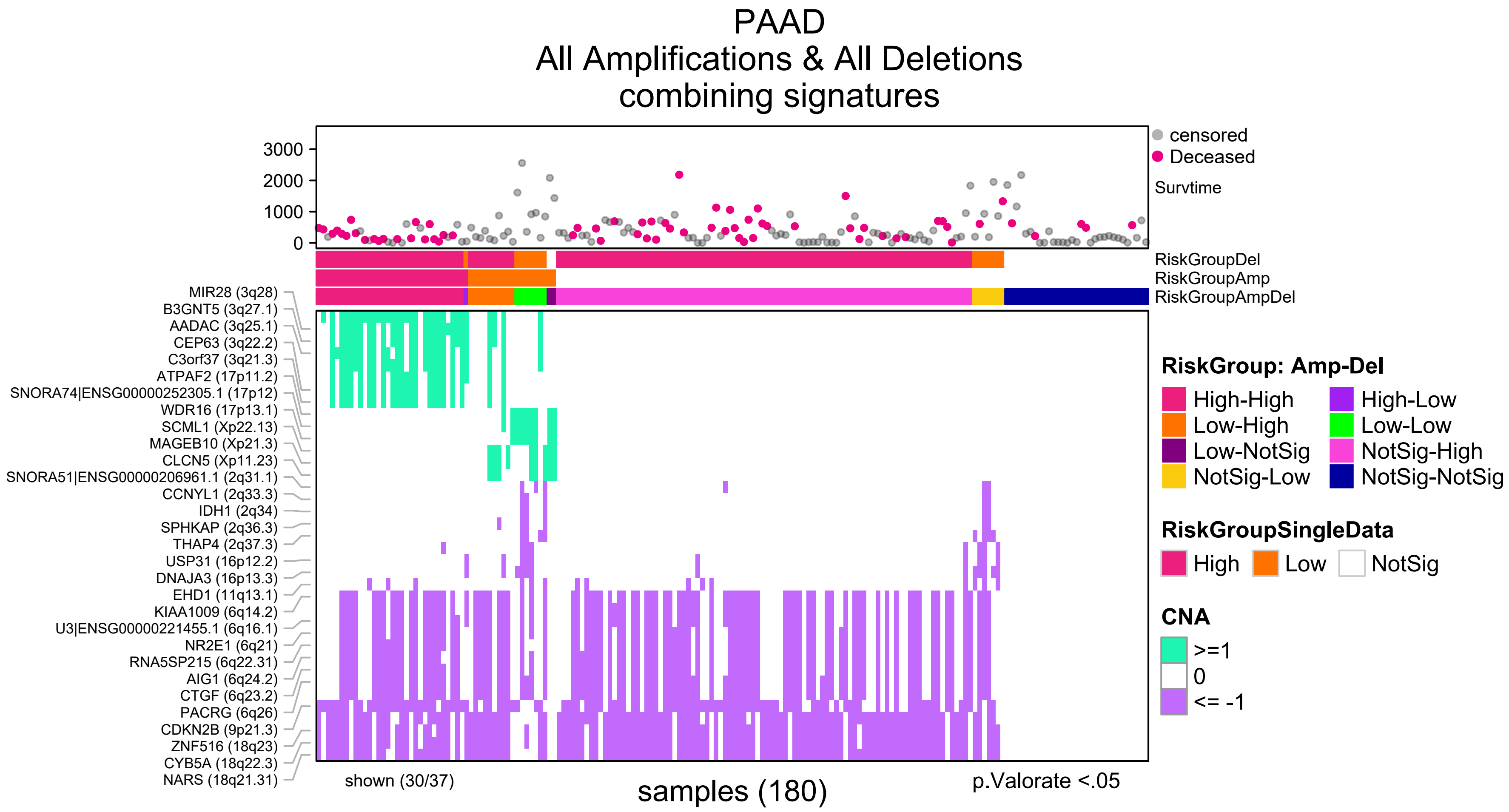
PAAD
All Amplifications & All Deletions
combining signatures
3000
2000
1000
0
censored
Deceased
Survtime
RiskGroupDel
RiskGroupAmp
RiskGroupAmpDel
MIR28 (3q28)
B3GNT5 (3q27.1)
AADAC (3q25.1)
CEP63 (3q22.2)
C3orf37 (3q21.3)
ATPAF2 (17p11.2)
SNORA74|ENSG00000252305.1 (17p12)
WDR16 (17p13.1)
SCML1 (Xp22.13)
MAGEB10 (Xp21.3)
CLCN5 (Xp11.23)
SNORA51|ENSG00000206961.1 (2q31.1)
CCNYL1 (2q33.3)
IDH1 (2q34)
SPHKAP (2q36.3)
THAP4 (2q37.3)
USP31 (16p12.2)
DNAJA3 (16p13.3)
EHD1 (11q13.1)
KIAA1009 (6q14.2)
U3|ENSG00000221455.1 (6q16.1)
NR2E1 (6q21)
RNA5SP215 (6q22.31)
AIG1 (6q24.2)
CTGF (6q23.2)
PACRG (6q26)
CDKN2B (9p21.3)
ZNF516 (18q23)
CYB5A (18q22.3)
NARS (18q21.31)
RiskGroup: Amp-Del
High-High
High-Low
Low-High
Low-Low
Low-NotSig
NotSig-High
NotSig-Low
NotSig-NotSig
RiskGroupSingleData
High
Low
NotSig
CNA
>=1
0
<= -1
shown (30/37)
samples (180)
p.Valorate <.05

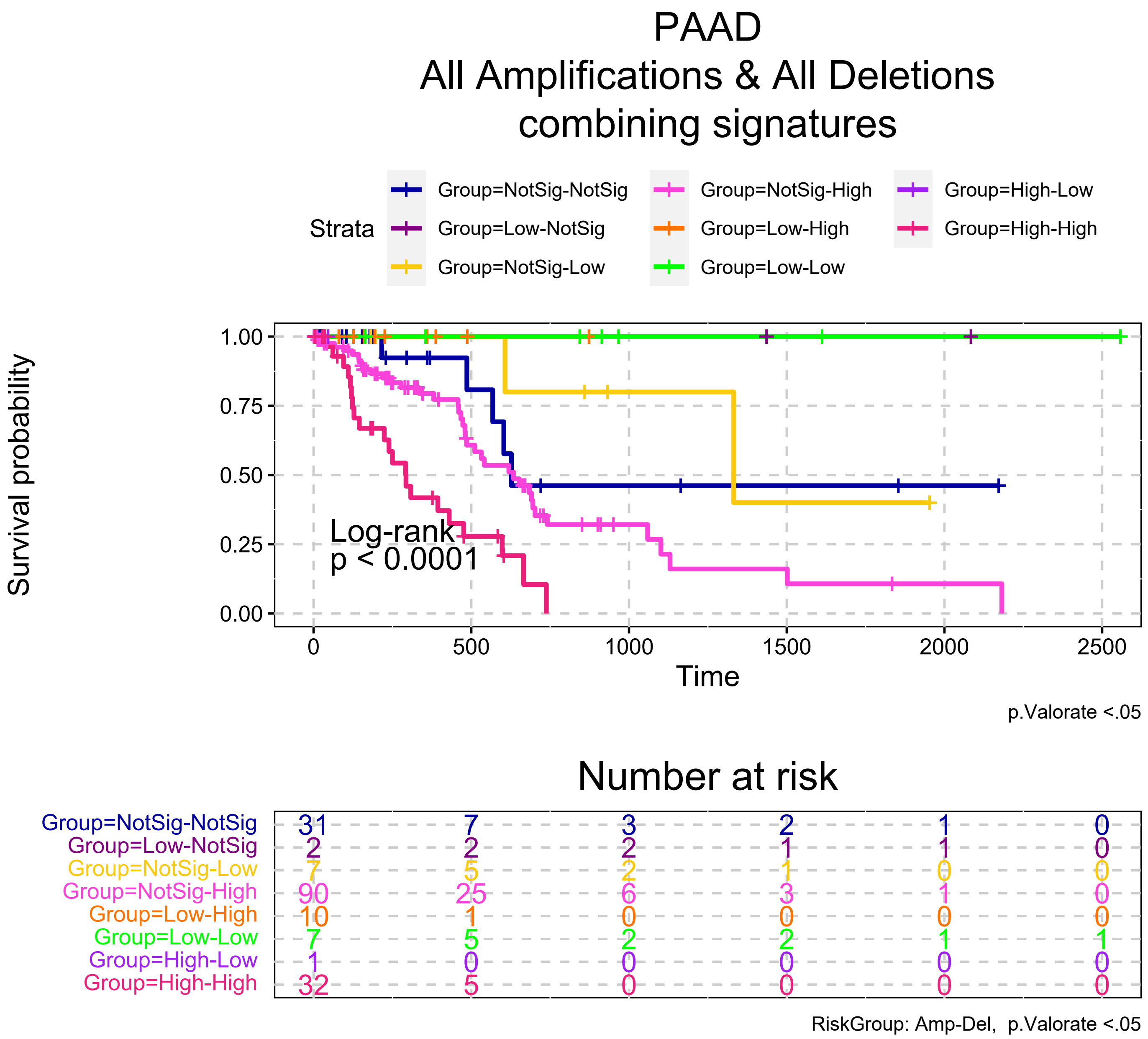

explanatory	beta	HR	L95	U95	p
Low-NotSig	-17.97	0.00	0.00	Inf	1.00
NotSig-Low	-0.48	0.62	0.12	3.19	0.57
NotSig-High	0.78	2.17	0.85	5.57	0.11
Low-High	-17.82	0.00	0.00	Inf	1.00
Low-Low	-17.94	0.00	0.00	Inf	1.00
High-Low	-17.75	0.00	0.00	Inf	1.00
High-High	1.84	6.30	2.31	17.15	0.00

n= 180, number of events =66
Score(logrank) test = p <.0001

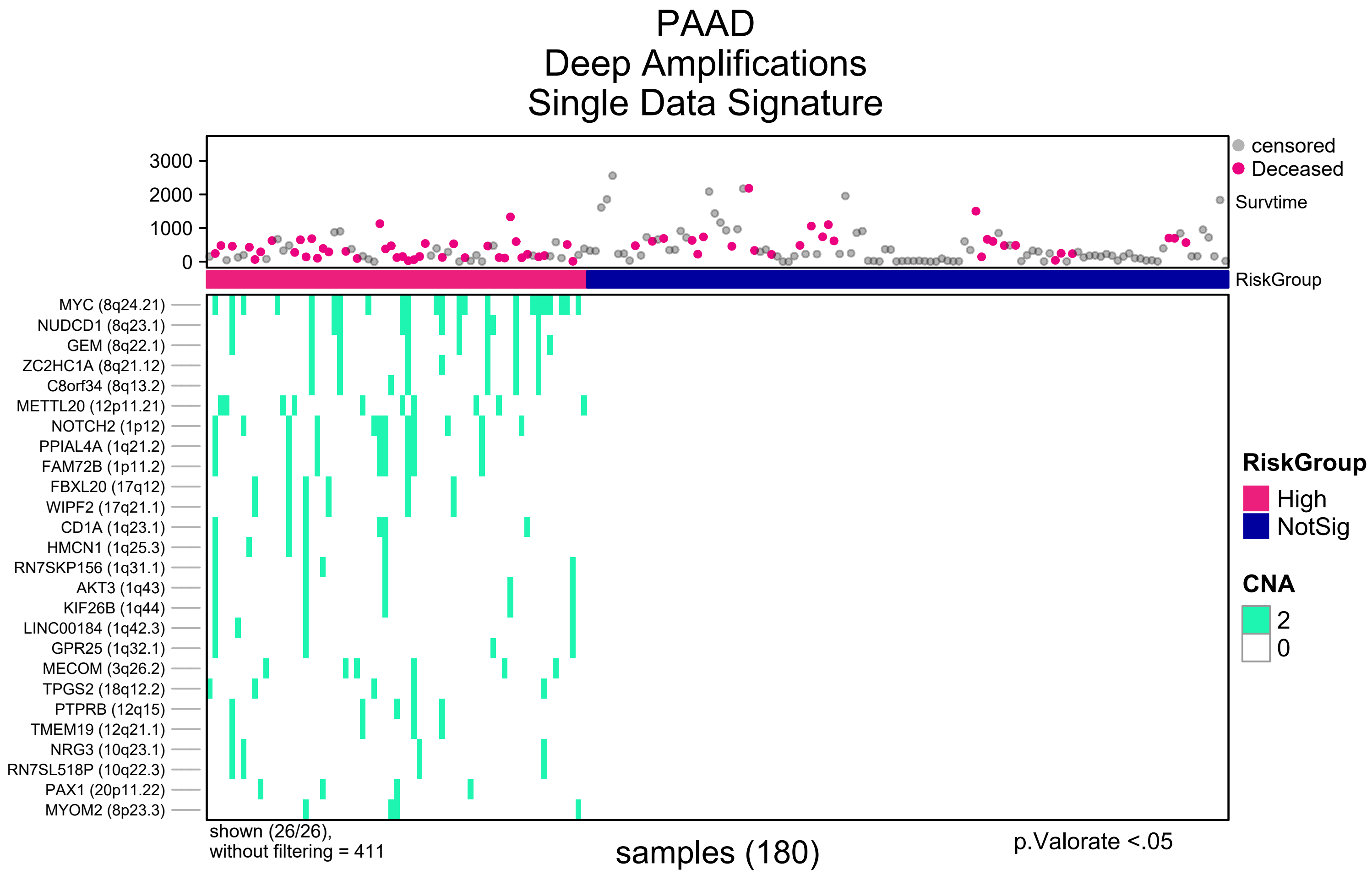
PAAD
Deep Amplifications
Single Data Signature
3000
2000
1000
0
censored
Deceased
Survtime
RiskGroup
MYC (8q24.21)
NUDCD1 (8q23.1)
GEM (8q22.1)
ZC2HC1A (8q21.12)
C8orf34 (8q13.2)
METTL20 (12p11.21)
NOTCH2 (1p12)
PPIAL4A (1q21.2)
FAM72B (1p11.2)
FBXL20 (17q12)
WIPF2 (17q21.1)
CD1A (1q23.1)
HMCN1 (1q25.3)
RN7SKP156 (1q31.1)
AKT3 (1q43)
KIF26B (1q44)
LINC00184 (1q42.3)
GPR25 (1q32.1)
MECOM (3q26.2)
TPGS2 (18q12.2)
PTPRB (12q15)
TMEM19 (12q21.1)
NRG3 (10q23.1)
RN7SL518P (10q22.3)
PAX1 (20p11.22)
MYOM2 (8p23.3)
RiskGroup
High
NotSig
CNA
2
0
shown (26/26),
without filtering = 411
samples (180)
p.Valorate <.05

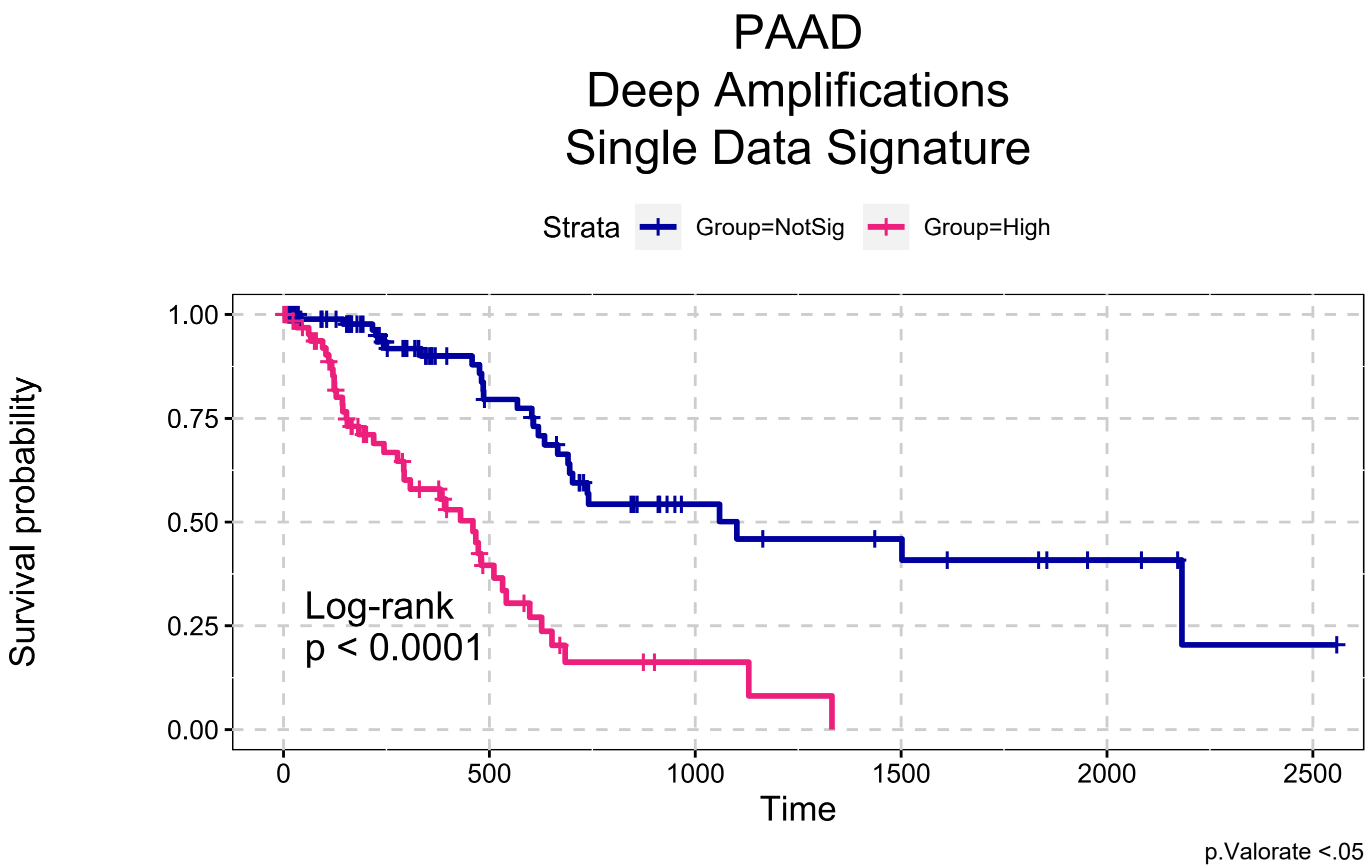

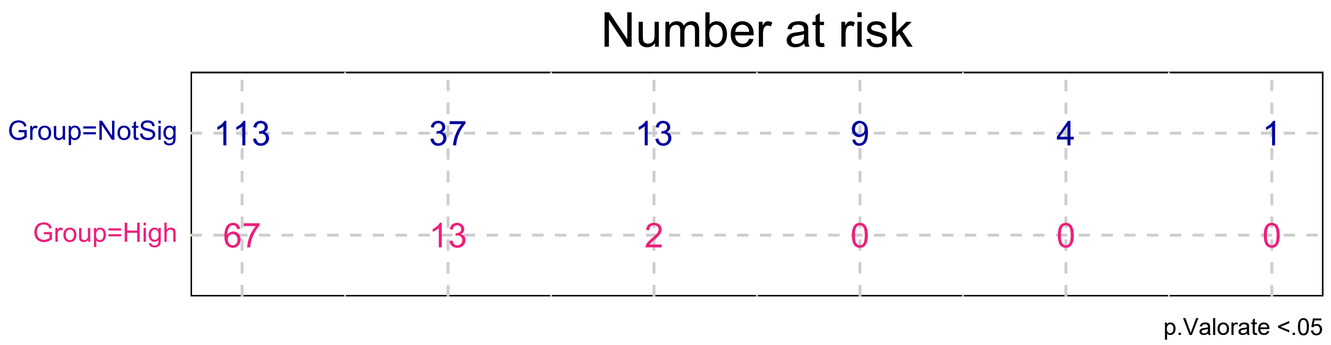

explanatory	beta	HR	L95	U95	p
High	1.45	4.27	2.55	7.17	0.00

n= 180, number of events =66
Score(logrank) test = p <.0001

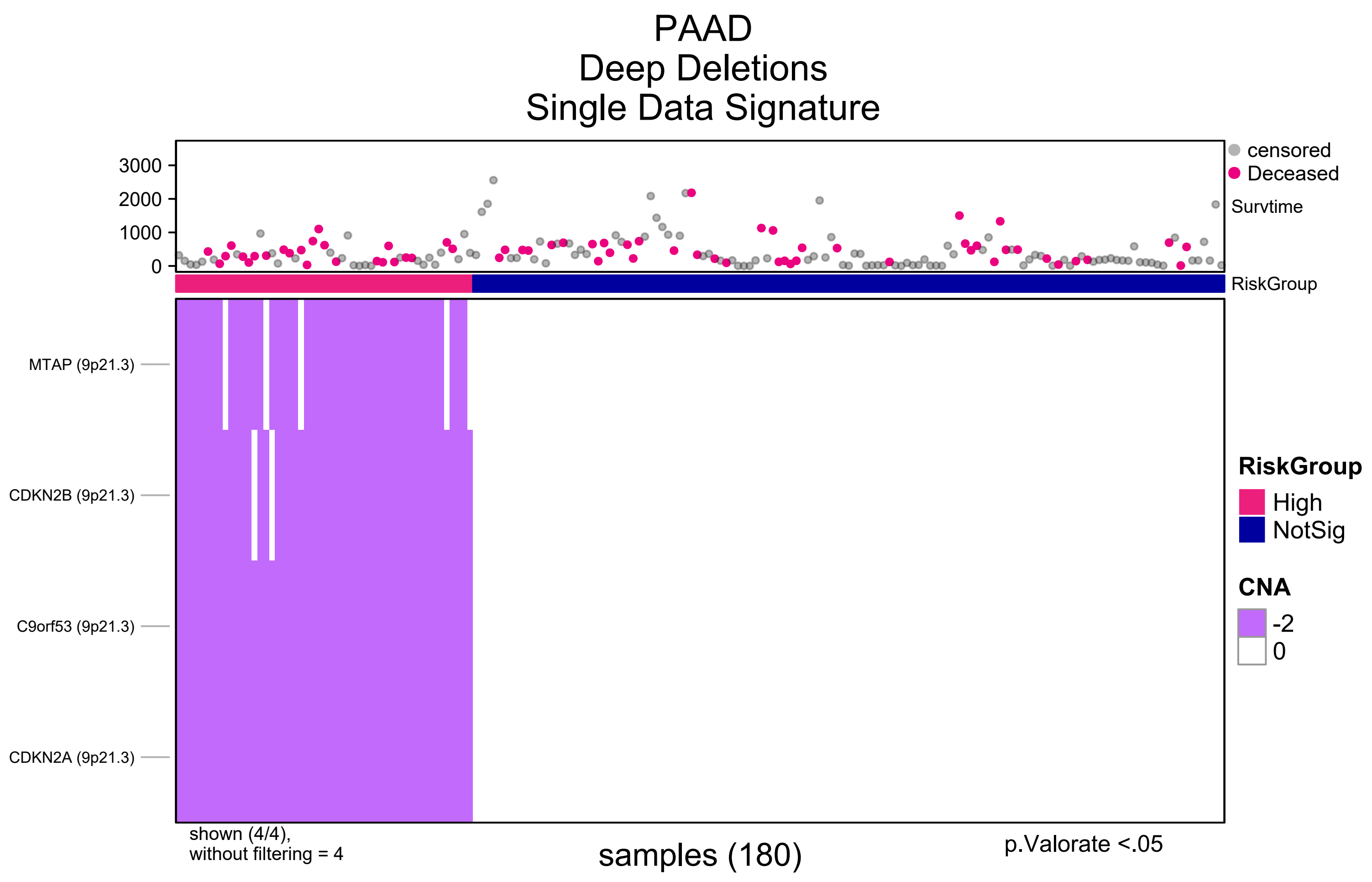
PAAD
Deep Deletions
Single Data Signature
3000
2000
1000
0
censored
Deceased
Survtime
RiskGroup
MTAP (9p21.3)
CDKN2B (9p21.3)
C9orf53 (9p21.3)
CDKN2A (9p21.3)
RiskGroup
High
NotSig
CNA
-2
0
shown (4/4),
without filtering = 4
samples (180)
p.Valorate <.05

PAAD
Deep Deletions
Single Data Signature

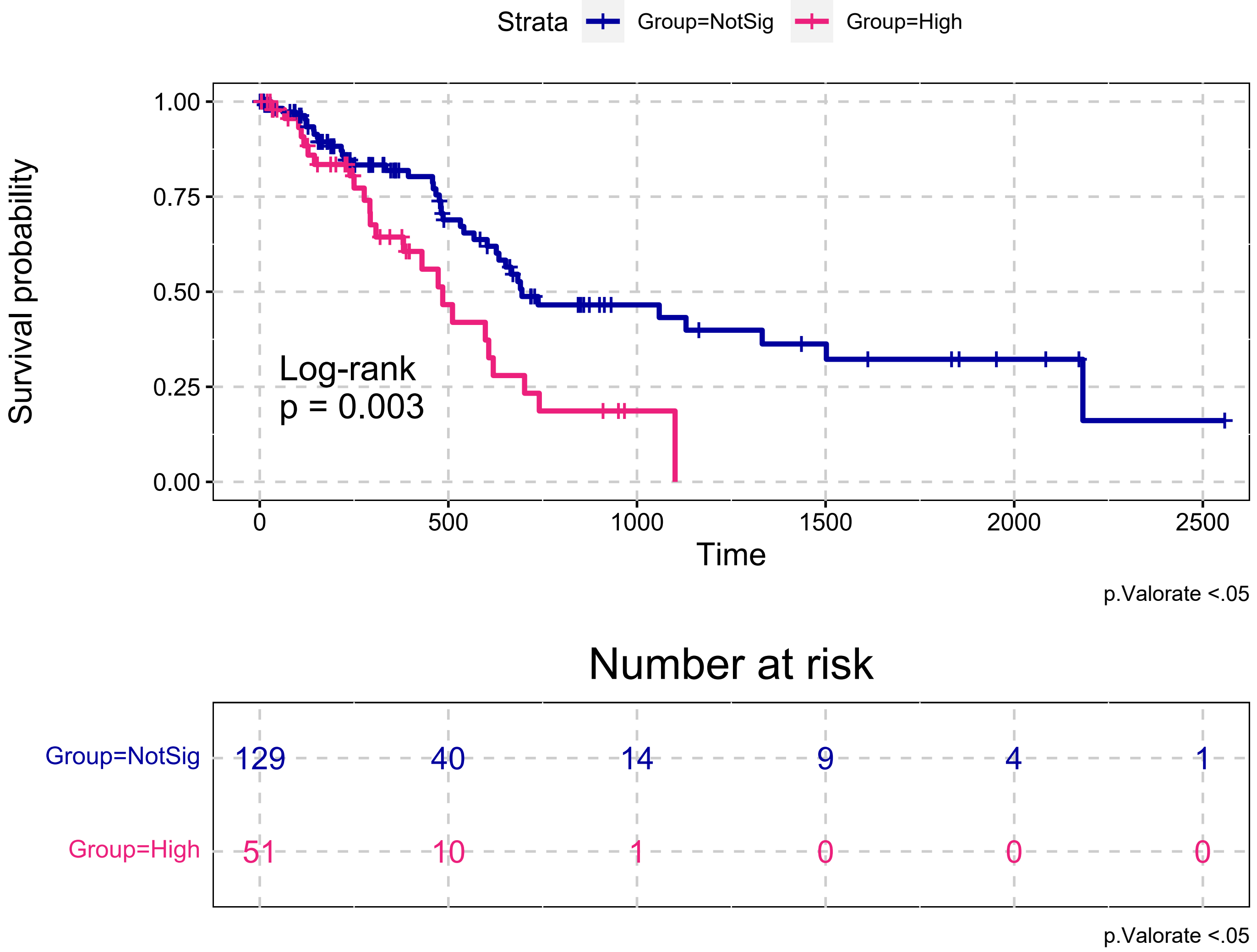

explanatory	beta	HR	L95	U95	p
High	0.77	2.15	1.28	3.61	0.00

n= 180, number of events =66
Score(logrank) test = 0.003

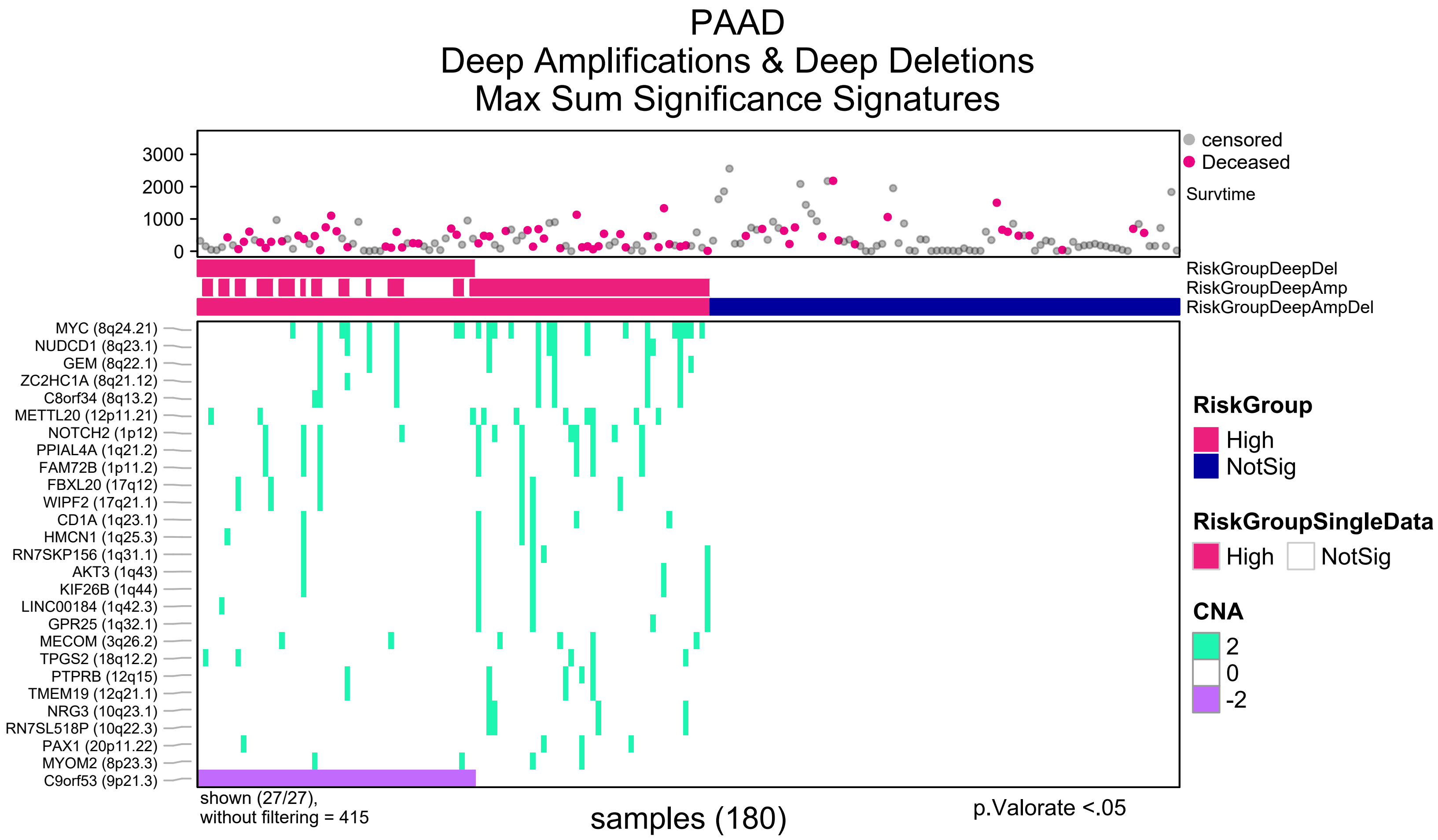
PAAD
Deep Amplifications & Deep Deletions
Max Sum Significance Signatures
3000
2000
1000
0
censored
Deceased
Survtime
RiskGroupDeepDel
RiskGroupDeepAmp
RiskGroupDeepAmpDel
MYC (8q24.21)
NUDCD1 (8q23.1)
GEM (8q22.1)
ZC2HC1A (8q21.12)
C8orf34 (8q13.2)
METTL20 (12p11.21)
NOTCH2 (1p12)
PPIAL4A (1q21.2)
FAM72B (1p11.2)
FBXL20 (17q12)
WIPF2 (17q21.1)
CD1A (1q23.1)
HMCN1 (1q25.3)
RN7SKP156 (1q31.1)
AKT3 (1q43)
KIF26B (1q44)
LINC00184 (1q42.3)
GPR25 (1q32.1)
MECOM (3q26.2)
TPGS2 (18q12.2)
PTPRB (12q15)
TMEM19 (12q21.1)
NRG3 (10q23.1)
RN7SL518P (10q22.3)
PAX1 (20p11.22)
MYOM2 (8p23.3)
C9orf53 (9p21.3)
RiskGroup
High
NotSig
RiskGroupSingleData
High
NotSig
CNA
2
0
-2
shown (27/27),
without filtering = 415
samples (180)
p.Valorate <.05

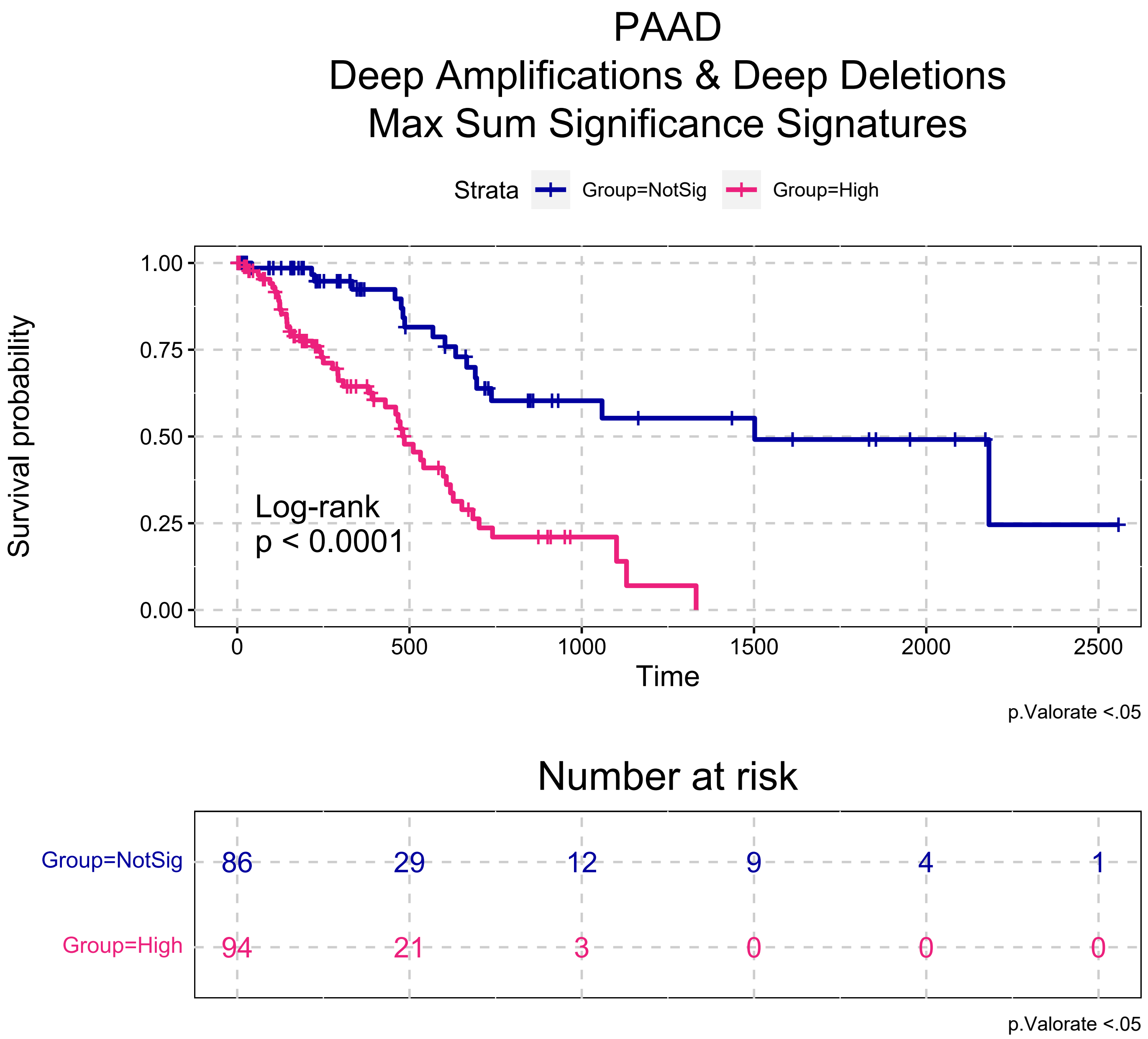

explanatory	beta	HR	L95	U95	p
High	1.43	4.17	2.33	7.46	0.00

n= 180, number of events =66
Score(logrank) test = p <.0001

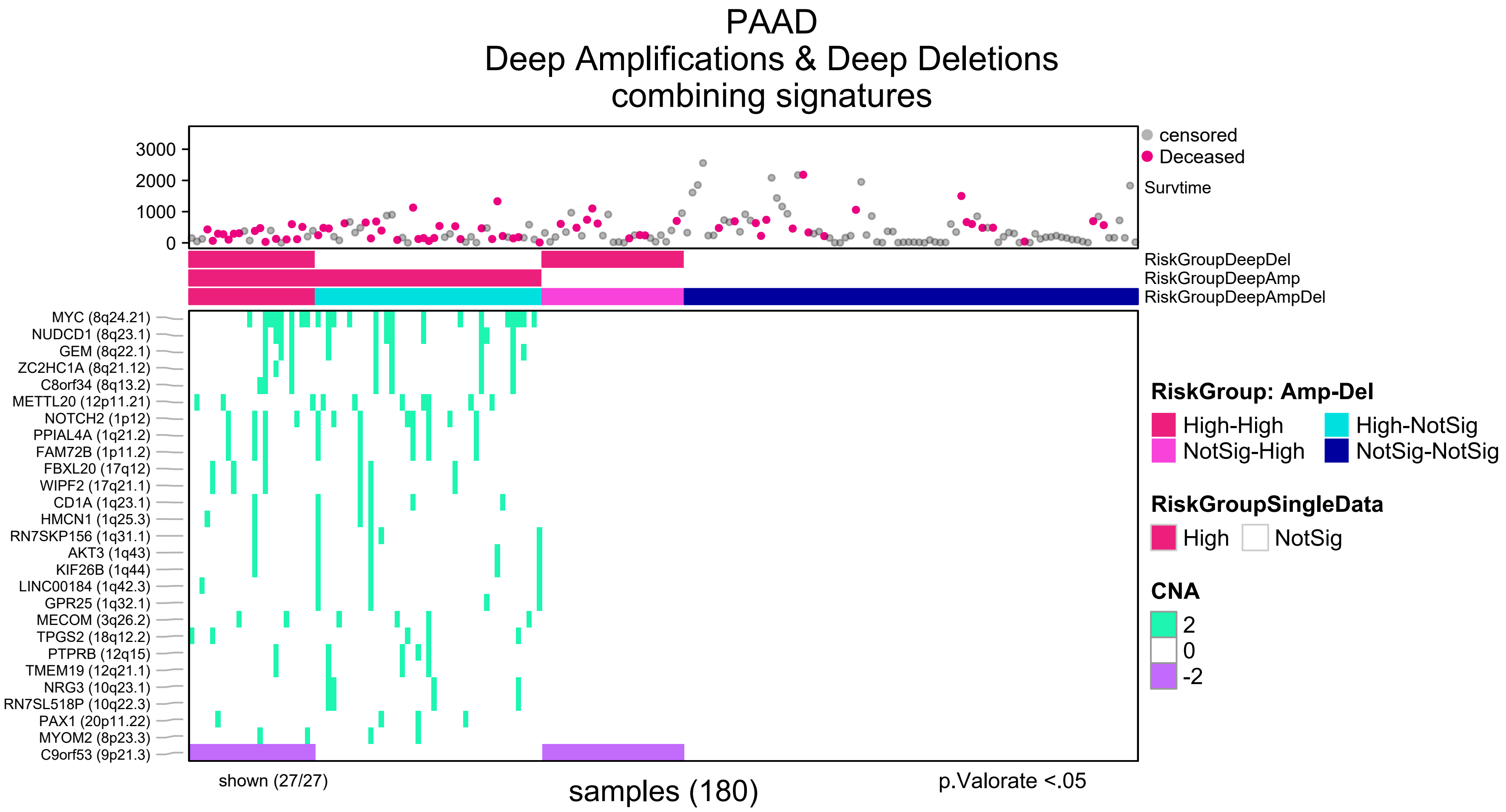
PAAD
Deep Amplifications & Deep Deletions
combining signatures
3000
2000
1000
0
censored
Deceased
Survtime
RiskGroupDeepDel
RiskGroupDeepAmp
RiskGroupDeepAmpDel
MYC (8q24.21)
NUDCD1 (8q23.1)
GEM (8q22.1)
ZC2HC1A (8q21.12)
C8orf34 (8q13.2)
METTL20 (12p11.21)
NOTCH2 (1p12)
PPIAL4A (1q21.2)
FAM72B (1p11.2)
FBXL20 (17q12)
WIPF2 (17q21.1)
CD1A (1q23.1)
HMCN1 (1q25.3)
RN7SKP156 (1q31.1)
AKT3 (1q43)
KIF26B (1q44)
LINC00184 (1q42.3)
GPR25 (1q32.1)
MECOM (3q26.2)
TPGS2 (18q12.2)
PTPRB (12q15)
TMEM19 (12q21.1)
NRG3 (10q23.1)
RN7SL518P (10q22.3)
PAX1 (20p11.22)
MYOM2 (8p23.3)
C9orf53 (9p21.3)
RiskGroup: Amp-Del
High-High
High-NotSig
NotSig-High
NotSig-NotSig
RiskGroupSingleData
High
NotSig
CNA
2
0
-2
shown (27/27)
samples (180)
p.Valorate <.05

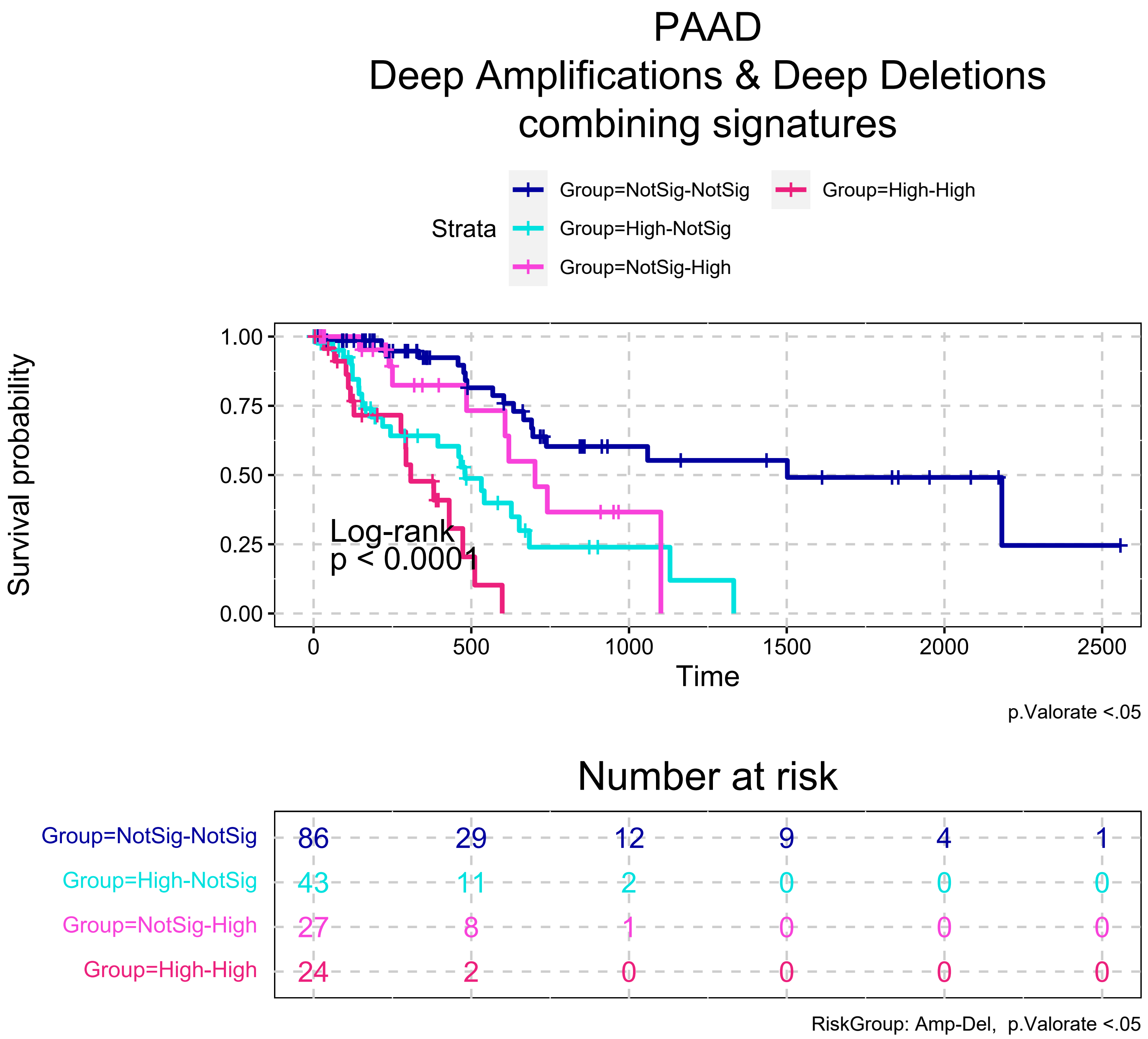

**explanatory**	**beta**	**HR**	**L95**	**U95**	**p**
High-NotSig	1.48	4.39	2.30	8.35	0.00
NotSig-High	0.80	2.22	0.97	5.09	0.06
High-High	2.30	9.98	4.60	21.63	0.00

n= 180, number of events =66
Score(logrank) test = p <.0001
