# Supplementary material for: Systematic Modeling of Risk-Associated Copy Number Alterations in Cancer
Source: Int J Mol Sci. 2024 Sep 27;25(19):10455. doi: 10.3390/ijms251910455 (PMC11477427; doi:10.3390/ijms251910455)
Supplement: Supplementary file 1 [file ijms-25-10455-s001.zip › PCPGSignatureV12-sinSombreado.pdf]

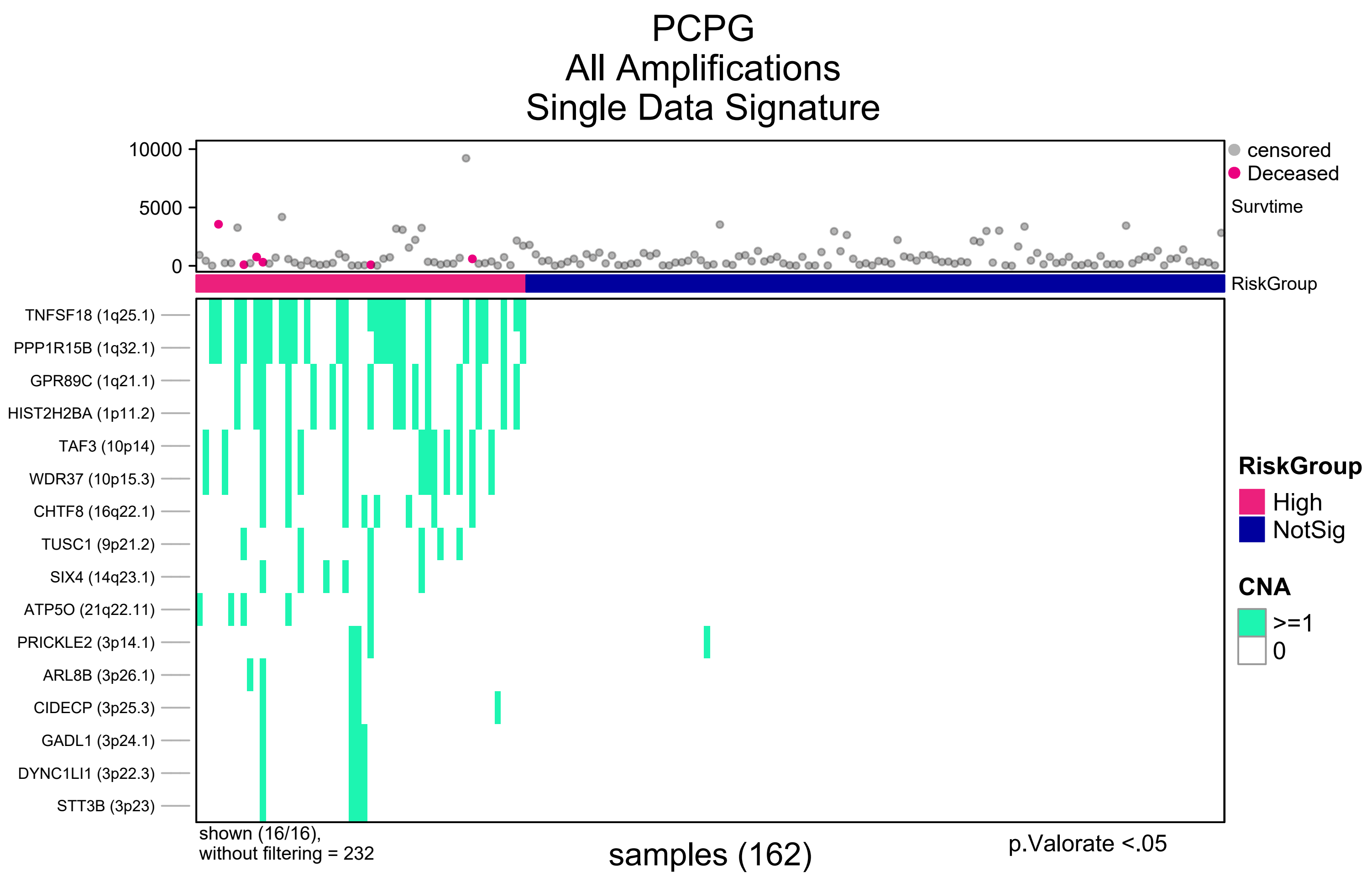
PCPG
All Amplifications
Single Data Signature
10000
5000
0
censored
Deceased
Survtime
RiskGroup
TNFSF18 (1q25.1)
PPP1R15B (1q32.1)
GPR89C (1q21.1)
HIST2H2BA (1p11.2)
TAF3 (10p14)
WDR37 (10p15.3)
CHTF8 (16q22.1)
TUSC1 (9p21.2)
SIX4 (14q23.1)
ATP5O (21q22.11)
PRICKLE2 (3p14.1)
ARL8B (3p26.1)
CIDECP (3p25.3)
GADL1 (3p24.1)
DYNC1LI1 (3p22.3)
STT3B (3p23)
RiskGroup
High
NotSig
CNA
>=1
0
shown (16/16),
without filtering = 232
samples (162)
p.Valorate <.05

PCPG
All Amplifications
Single Data Signature

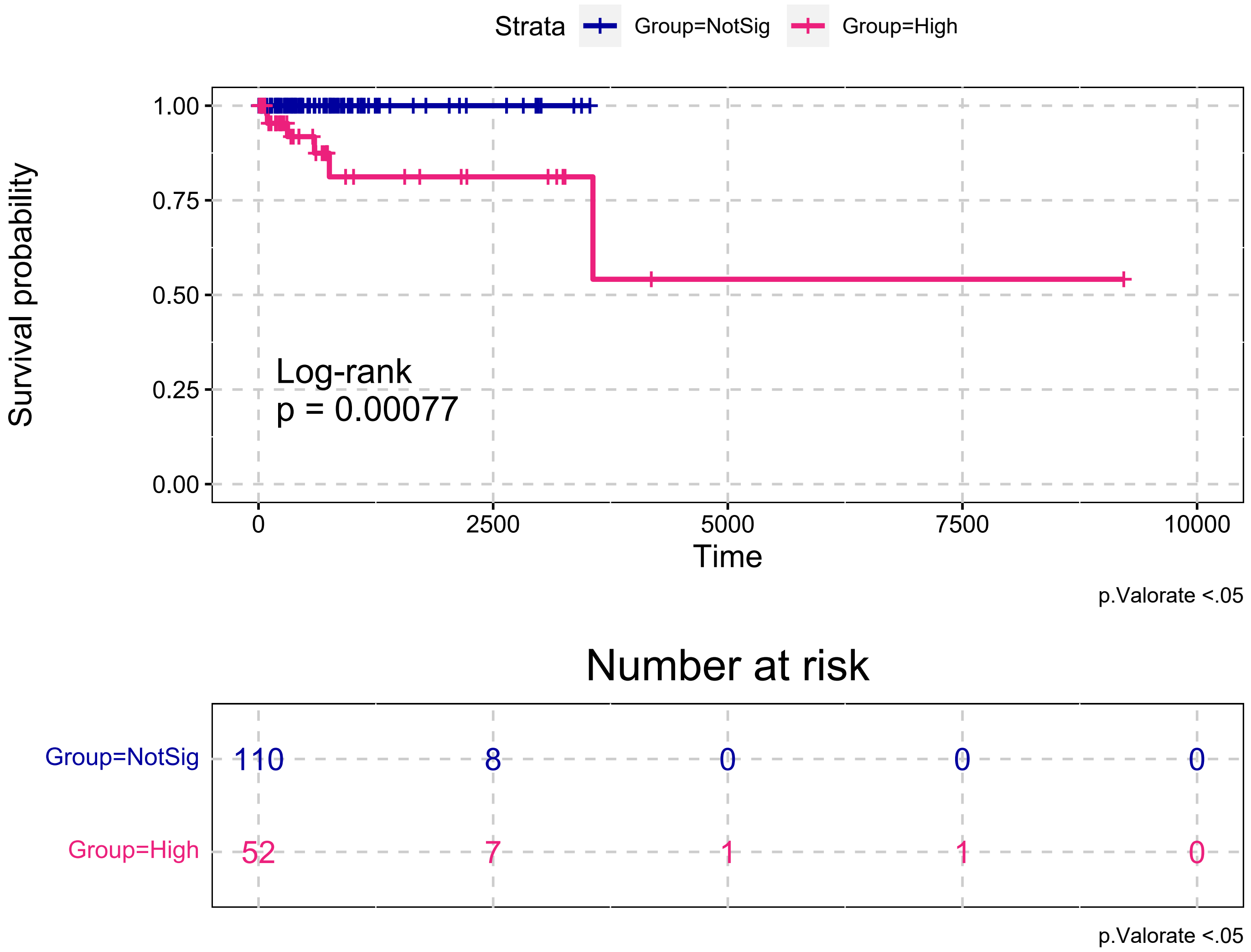

explanatory	beta	HR	L95	U95	p
High	21.40	1967034176.18	0.00	Inf	1.00

n= 162, number of events =6
Score(logrank) test = 0.001

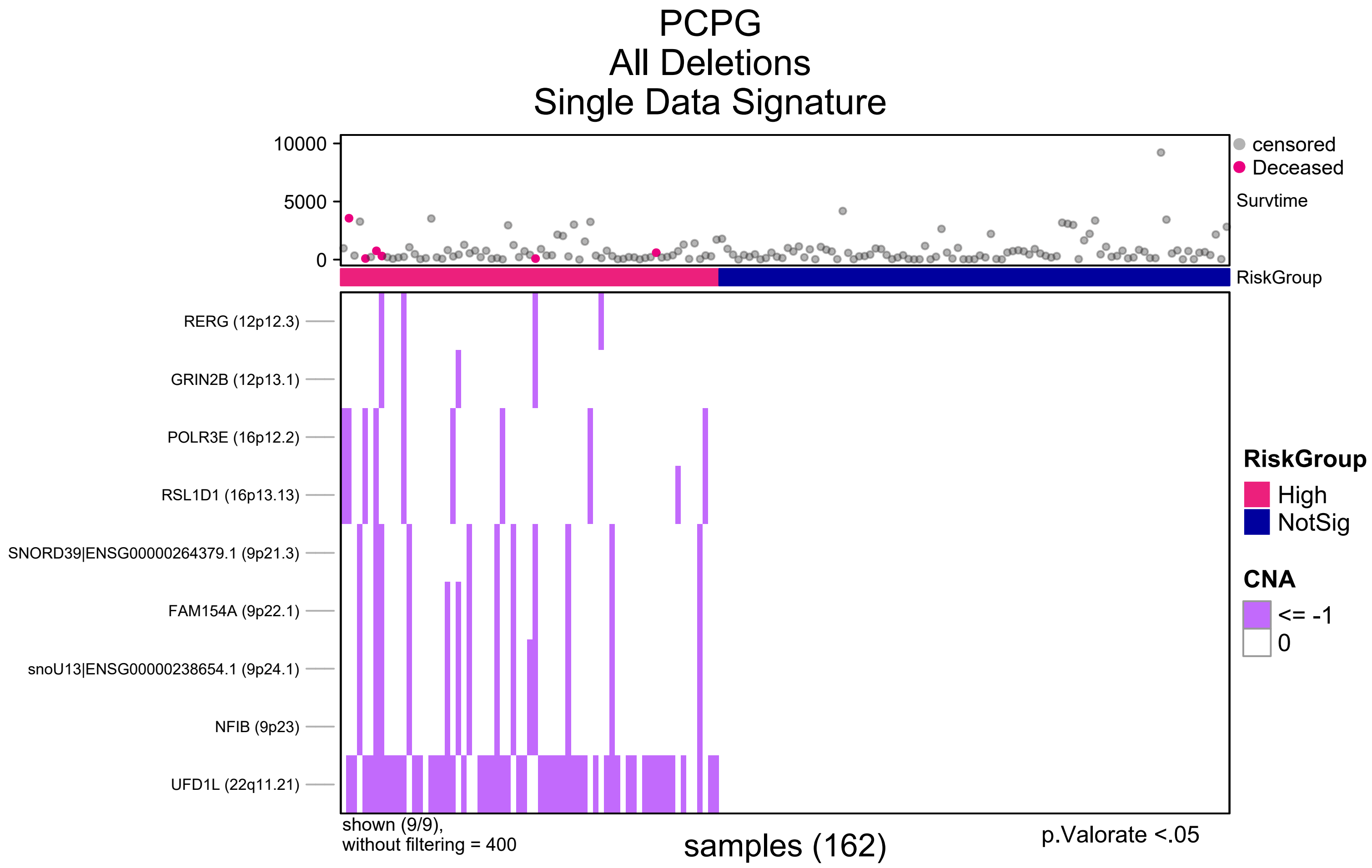
PCPG
All Deletions
Single Data Signature
10000
5000
0
censored
Deceased
Survtime
RiskGroup
RERG (12p12.3)
GRIN2B (12p13.1)
POLR3E (16p12.2)
RSL1D1 (16p13.13)
SNORD39|ENSG00000264379.1 (9p21.3)
FAM154A (9p22.1)
snoU13|ENSG00000238654.1 (9p24.1)
NFIB (9p23)
UFD1L (22q11.21)
RiskGroup
High
NotSig
CNA
<= -1
0
shown (9/9),
without filtering = 400
samples (162)
p.Valorate <.05

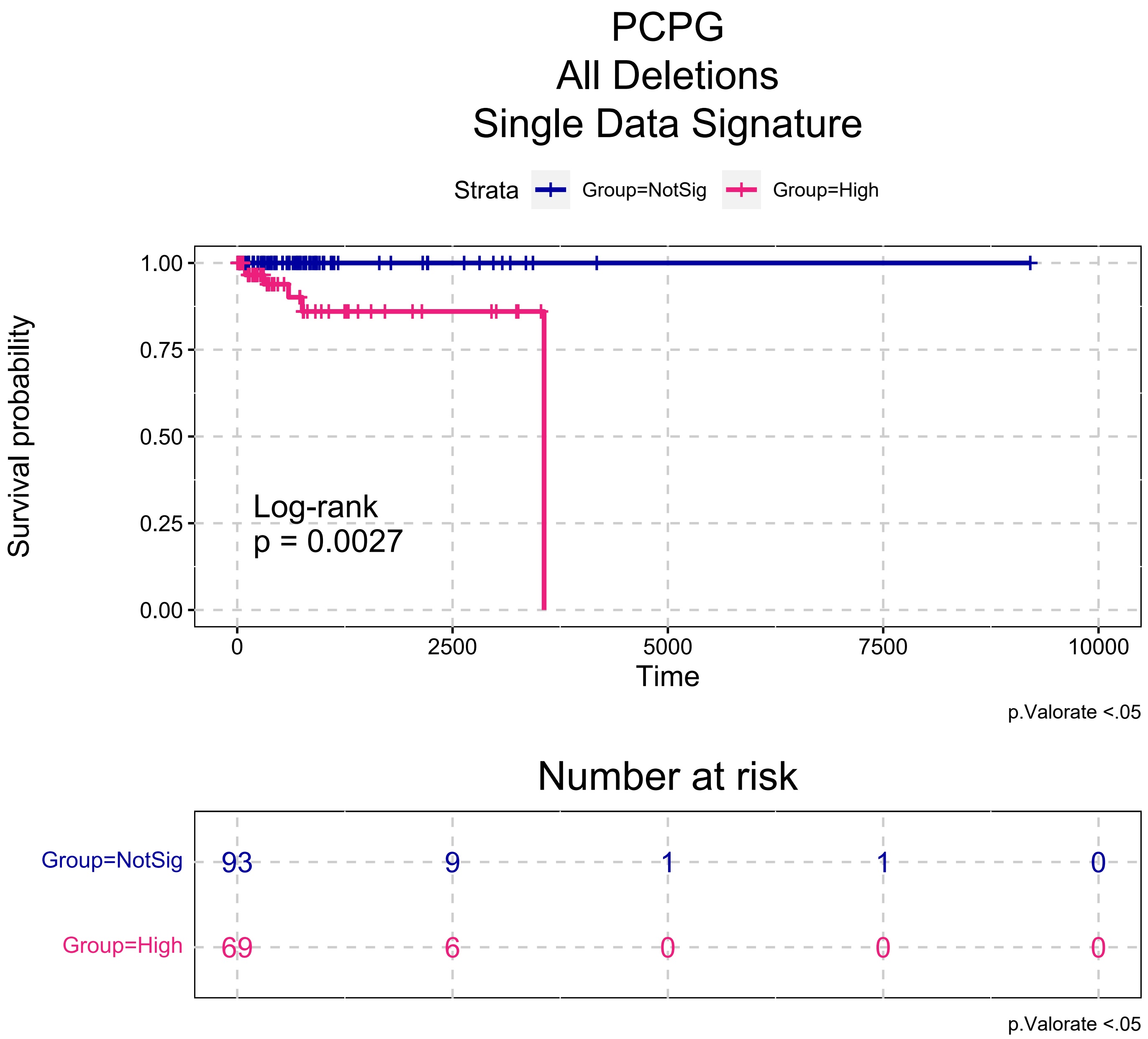

explanatory	beta	HR	L95	U95	p
High	20.69	971260644.90	0.00	Inf	1.00

n= 162, number of events =6
Score(logrank) test = 0.003

PCPG
All Amplifications & All Deletions
Max Sum Significance Signatures

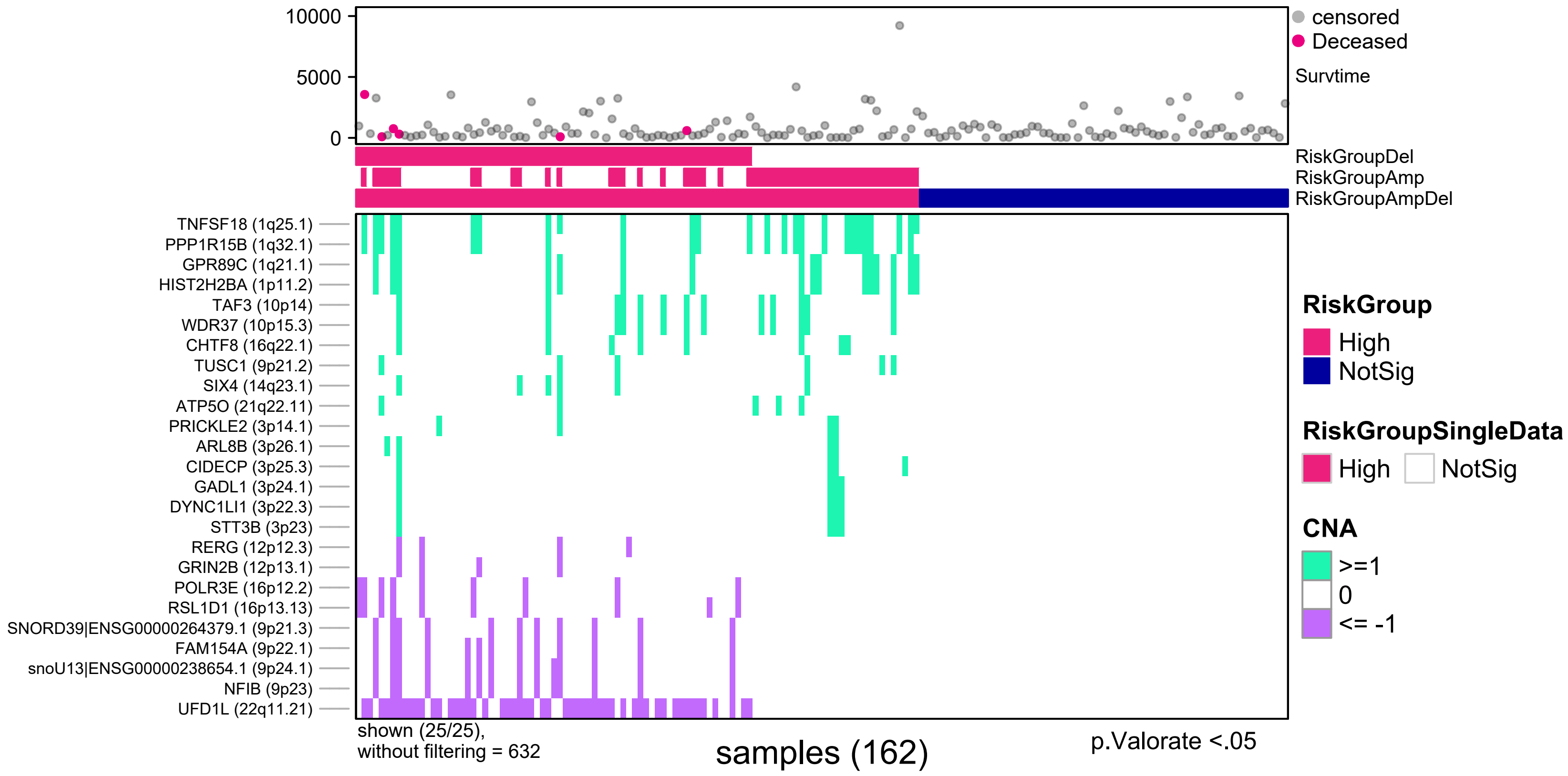

PCPG
All Amplifications & All Deletions
Max Sum Significance Signatures

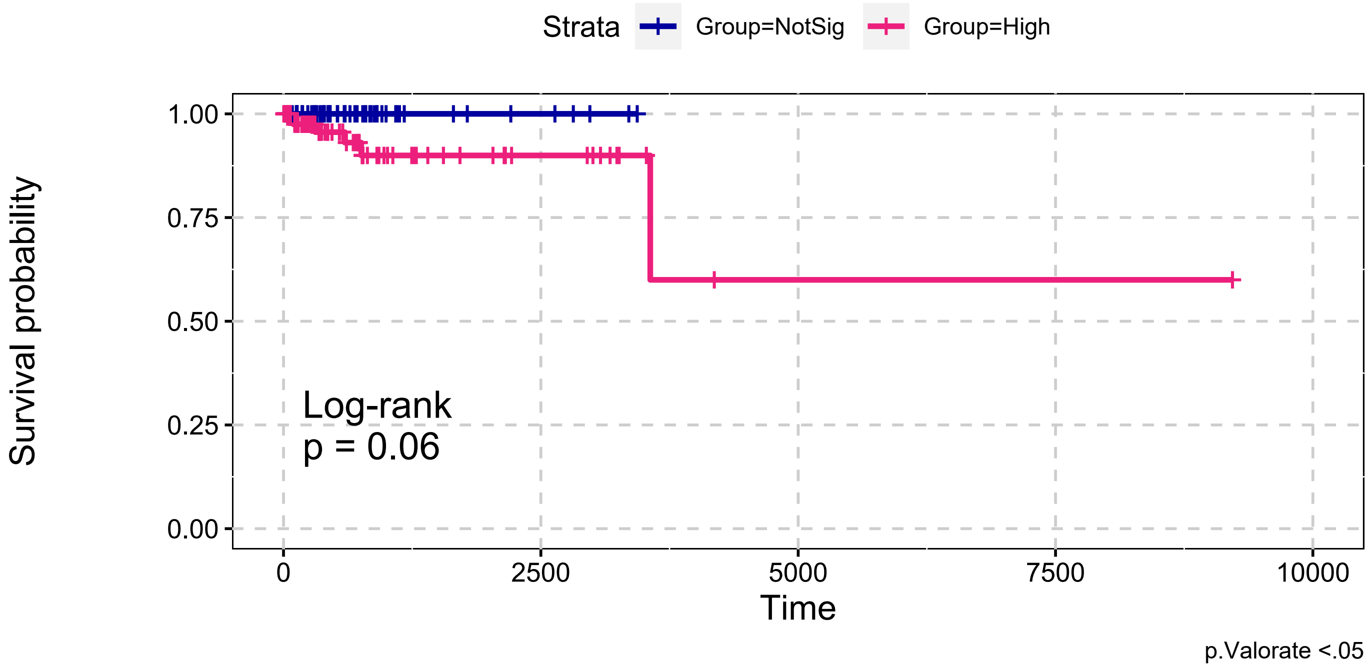

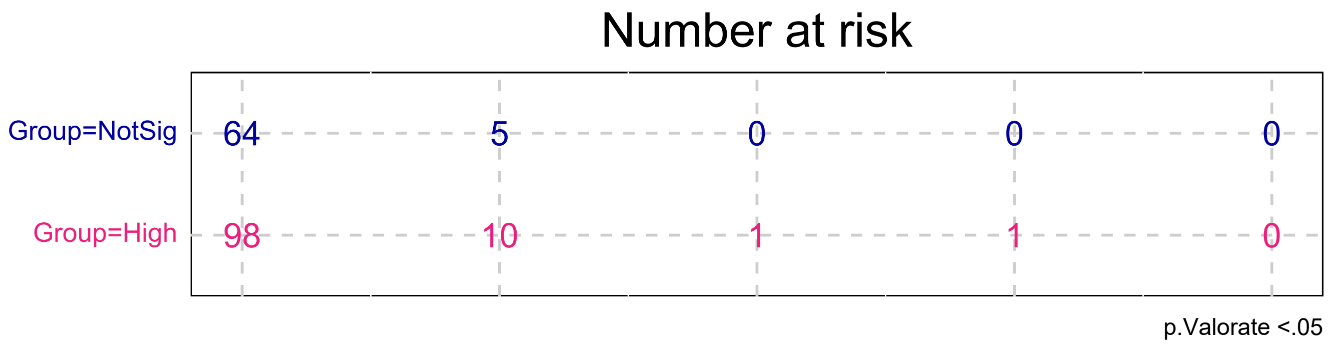

explanatory	beta	HR	L95	U95	p
High	19.90	440429404.30	0.00	Inf	1.00

n= 162, number of events =6
Score(logrank) test = 0.06

PCPG
All Amplifications & All Deletions
combining signatures

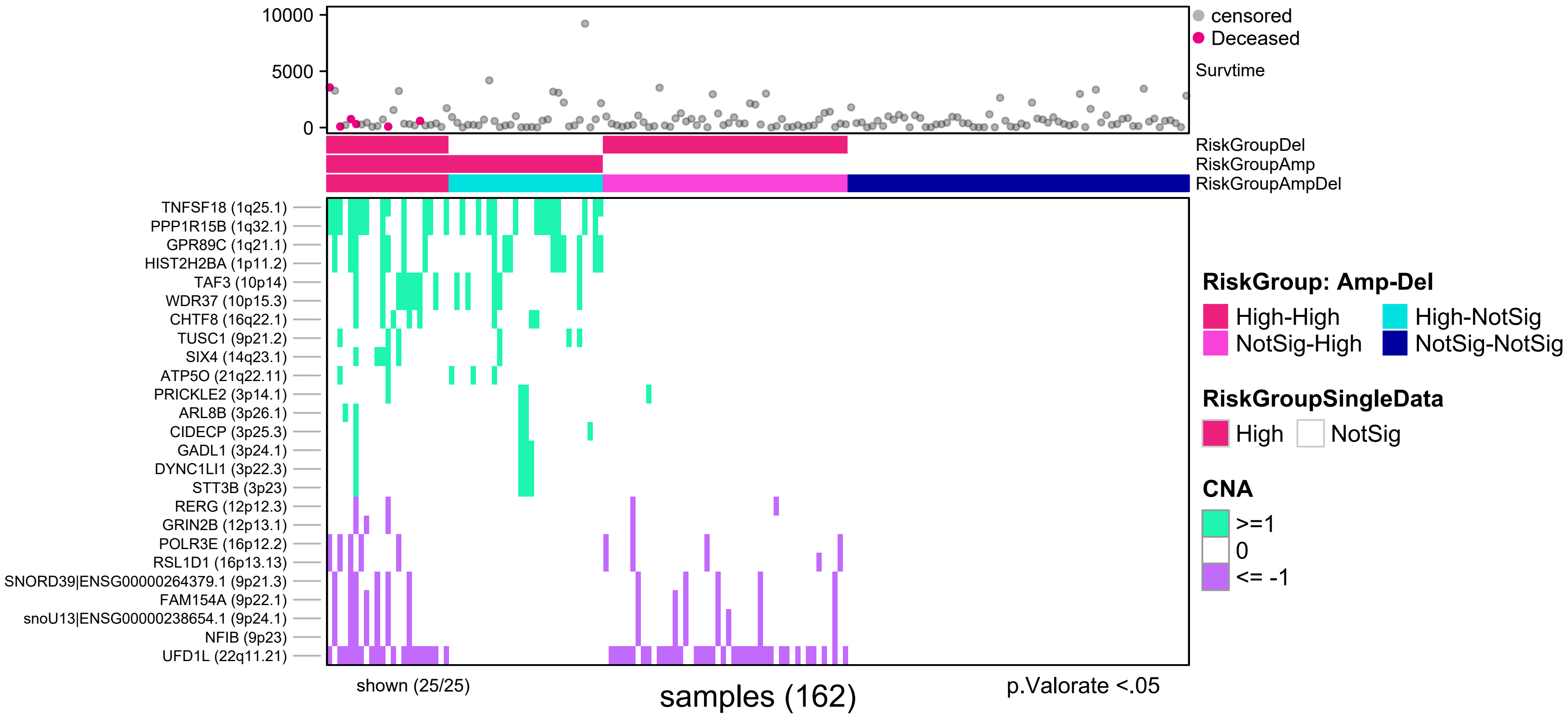

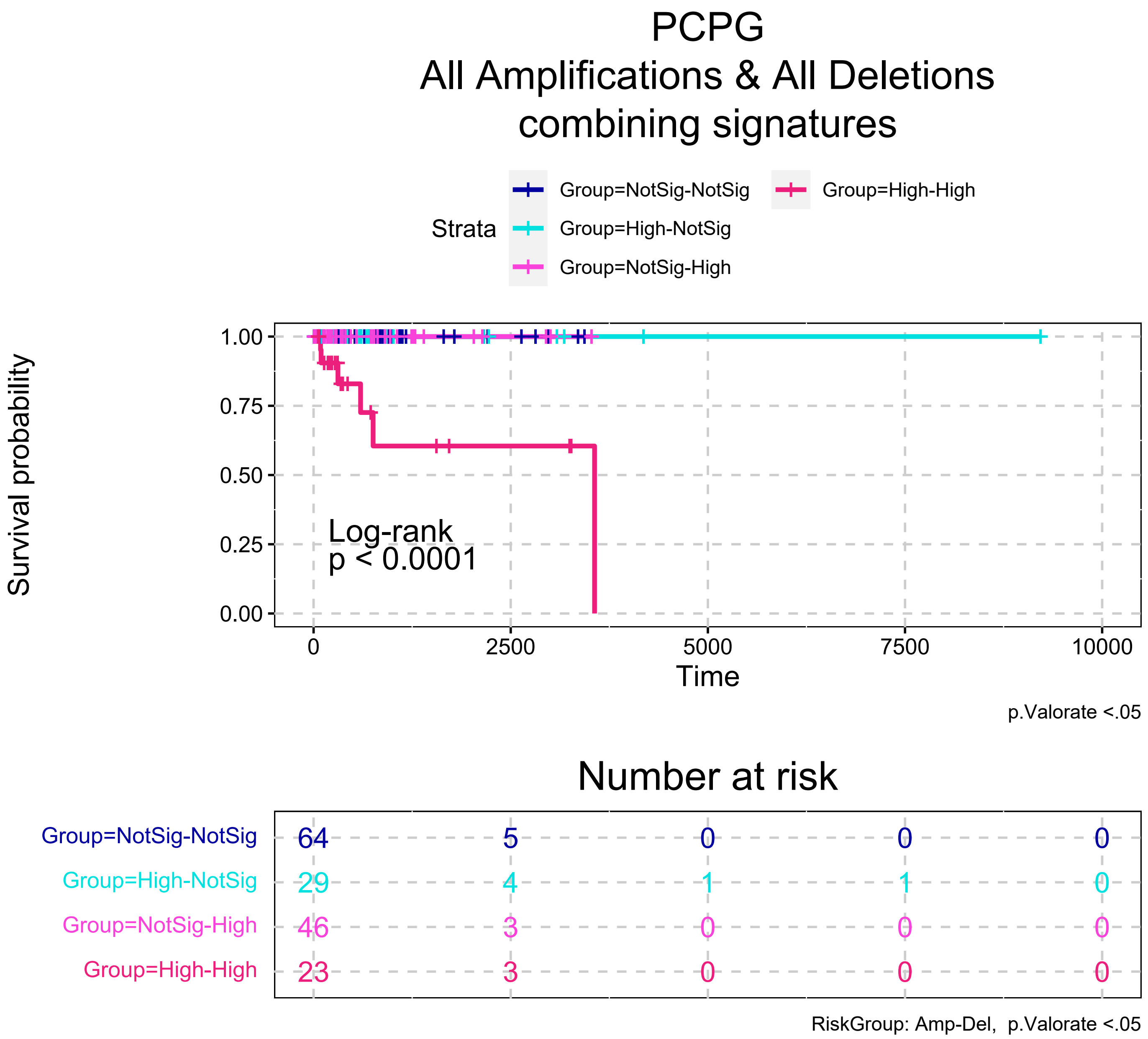

explanatory	beta	HR	L95	U95	p
High-NotSig	0.72	2.06	0.00	Inf	1.00
NotSig-High	0.00	1.00	0.00	Inf	1.00
High-High	23.48	15710435369.28	0.00	Inf	1.00

n= 162, number of events =6
Score(logrank) test = p <.0001

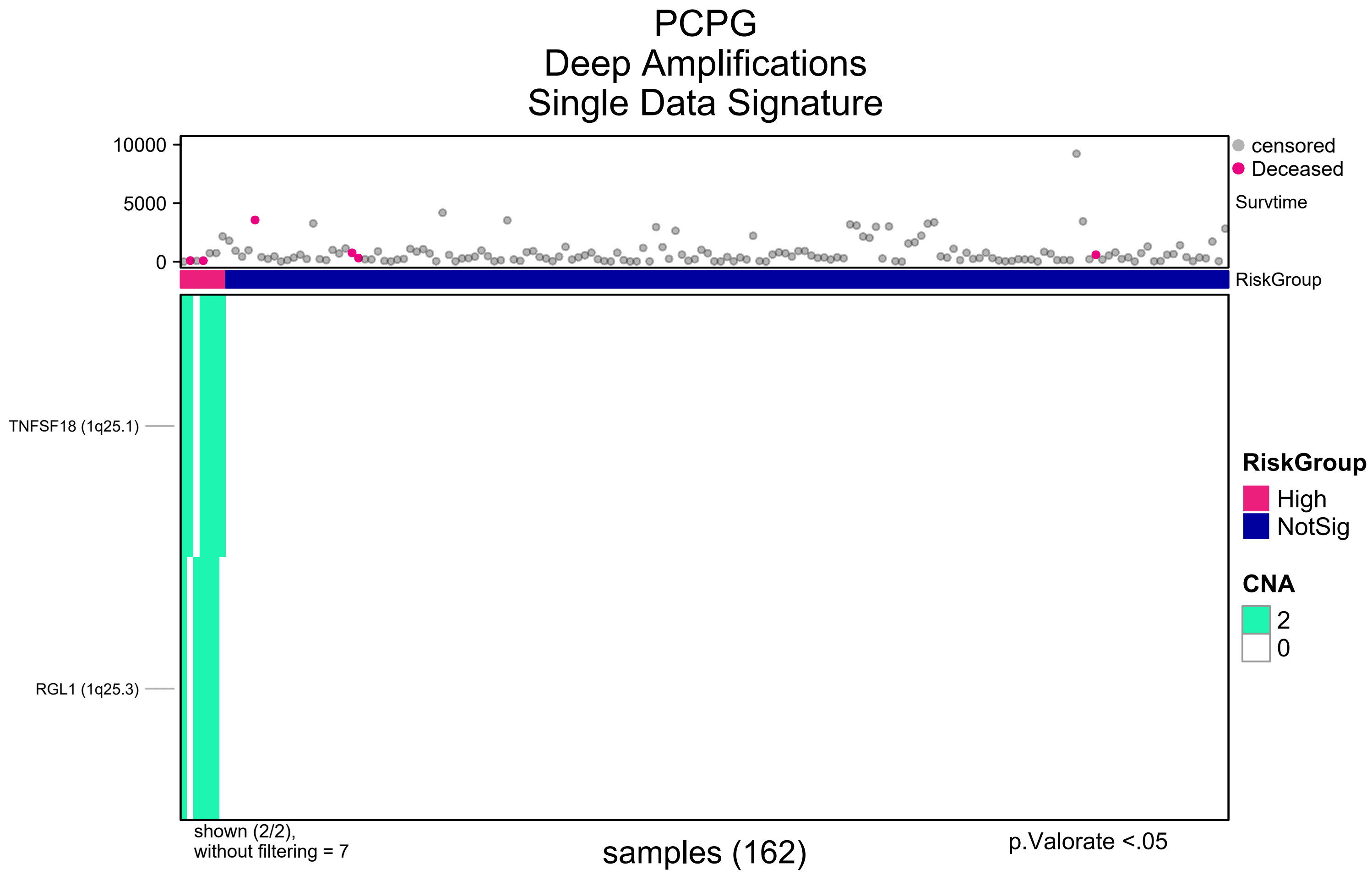

PCPG
Deep Amplifications
Single Data Signature
10000
5000
0
censored
Deceased
Survtime
RiskGroup
TNFSF18 (1q25.1)
RGL1 (1q25.3)
RiskGroup
High
NotSig
CNA
2
0
shown (2/2),
without filtering = 7
samples (162)
p.Valorate <.05

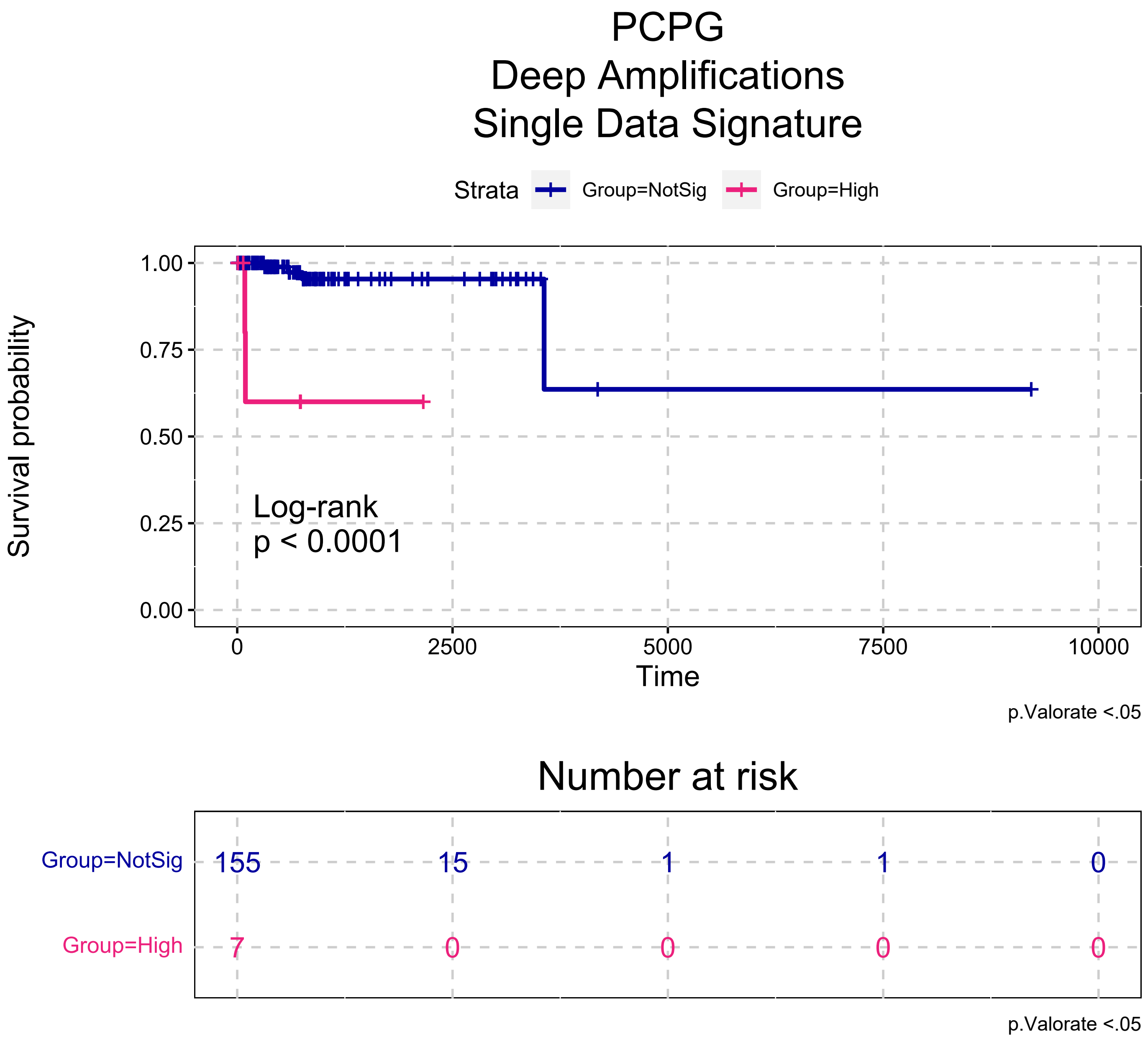

explanatory	beta	HR	L95	U95	p
High	2.99	19.91	3.27	121.38	0.00

n= 162, number of events =6
Score(logrank) test = p <.0001
