# Supplementary material for: Systematic Modeling of Risk-Associated Copy Number Alterations in Cancer
Source: Int J Mol Sci. 2024 Sep 27;25(19):10455. doi: 10.3390/ijms251910455 (PMC11477427; doi:10.3390/ijms251910455)
Supplement: Supplementary file 1 [file ijms-25-10455-s001.zip › PRADSignatureV12-sinSombreado.pdf]

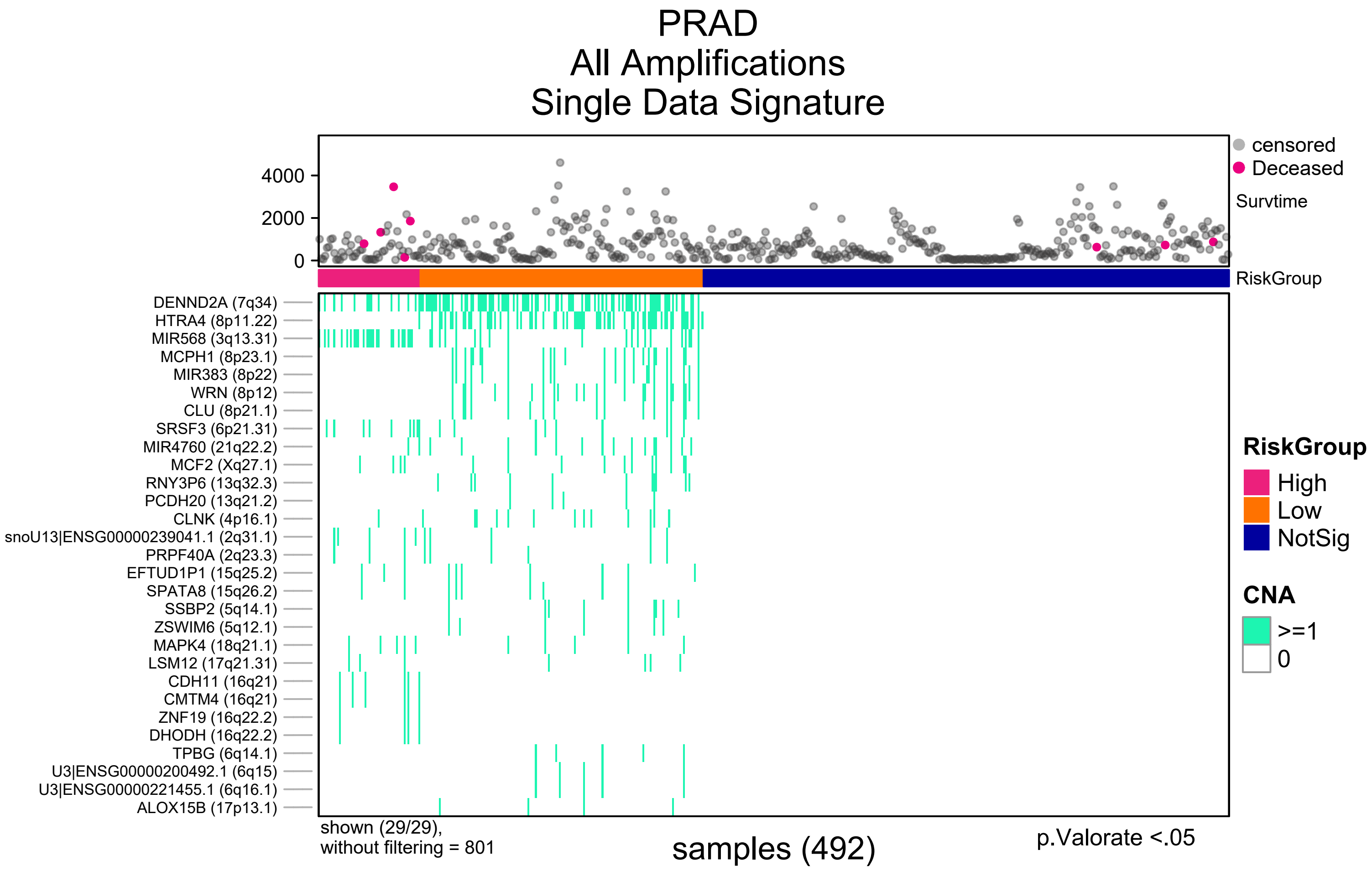

PRAD
All Amplifications
Single Data Signature
4000
2000
0
censored
Deceased
Survtime
RiskGroup
DENND2A (7q34)
HTRA4 (8p11.22)
MIR568 (3q13.31)
MCPH1 (8p23.1)
MIR383 (8p22)
WRN (8p12)
CLU (8p21.1)
SRSF3 (6p21.31)
MIR4760 (21q22.2)
MCF2 (Xq27.1)
RNY3P6 (13q32.3)
PCDH20 (13q21.2)
CLNK (4p16.1)
snoU13|ENSG00000239041.1 (2q31.1)
PRPF40A (2q23.3)
EFTUD1P1 (15q25.2)
SPATA8 (15q26.2)
SSBP2 (5q14.1)
ZSWIM6 (5q12.1)
MAPK4 (18q21.1)
LSM12 (17q21.31)
CDH11 (16q21)
CMTM4 (16q21)
ZNF19 (16q22.2)
DHODH (16q22.2)
TPBG (6q14.1)
U3|ENSG00000200492.1 (6q15)
U3|ENSG00000221455.1 (6q16.1)
ALOX15B (17p13.1)
RiskGroup
High
Low
NotSig
CNA
>=1
0
shown (29/29),
without filtering = 801
samples (492)
p.Valorate <.05

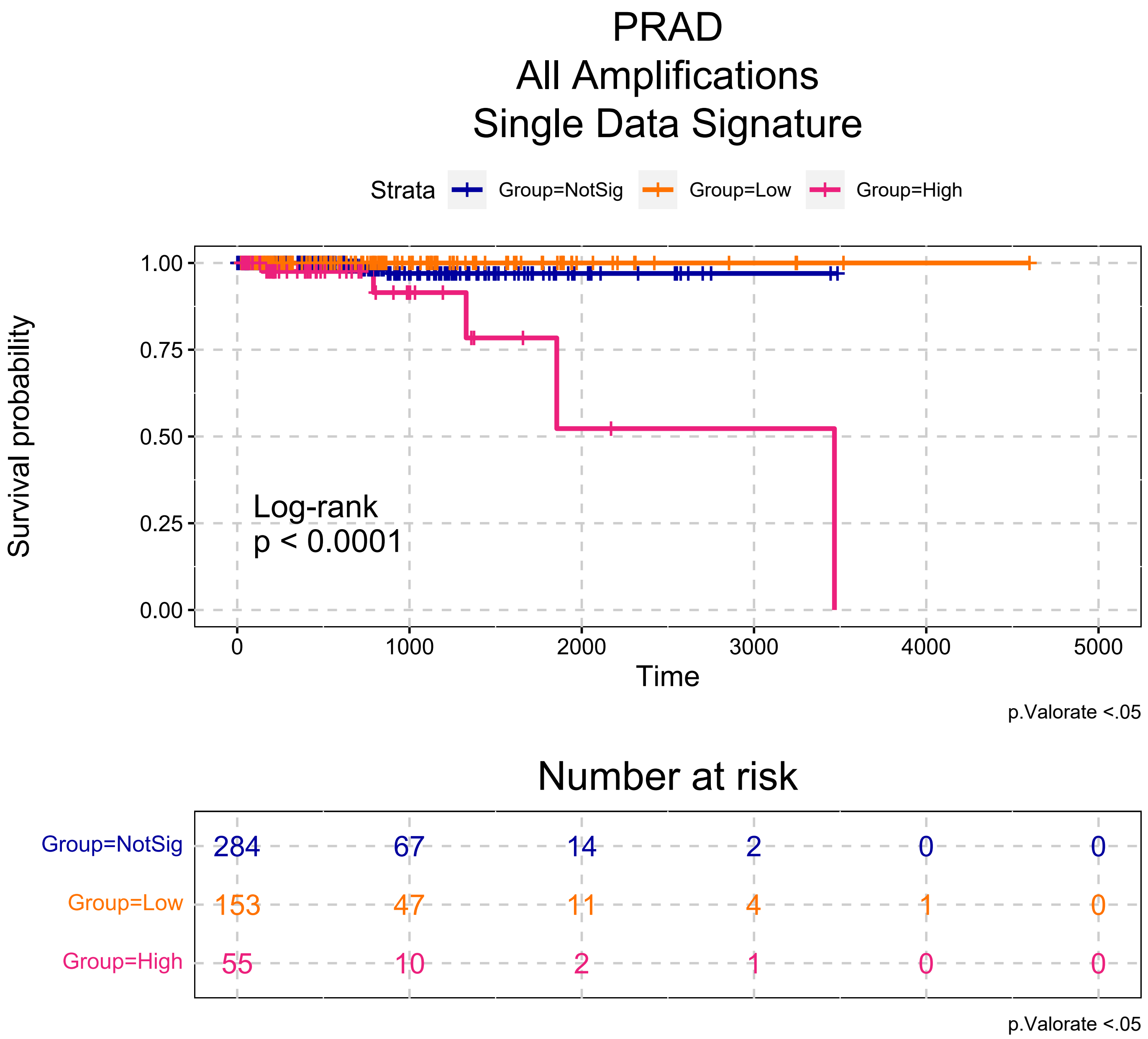

explanatory	beta	HR	L95	U95	p
Low	-19.66	0.00	0.00	Inf	1.00
High	2.12	8.37	1.93	36.19	0.00

n= 492, number of events =8
Score(logrank) test = p <.0001

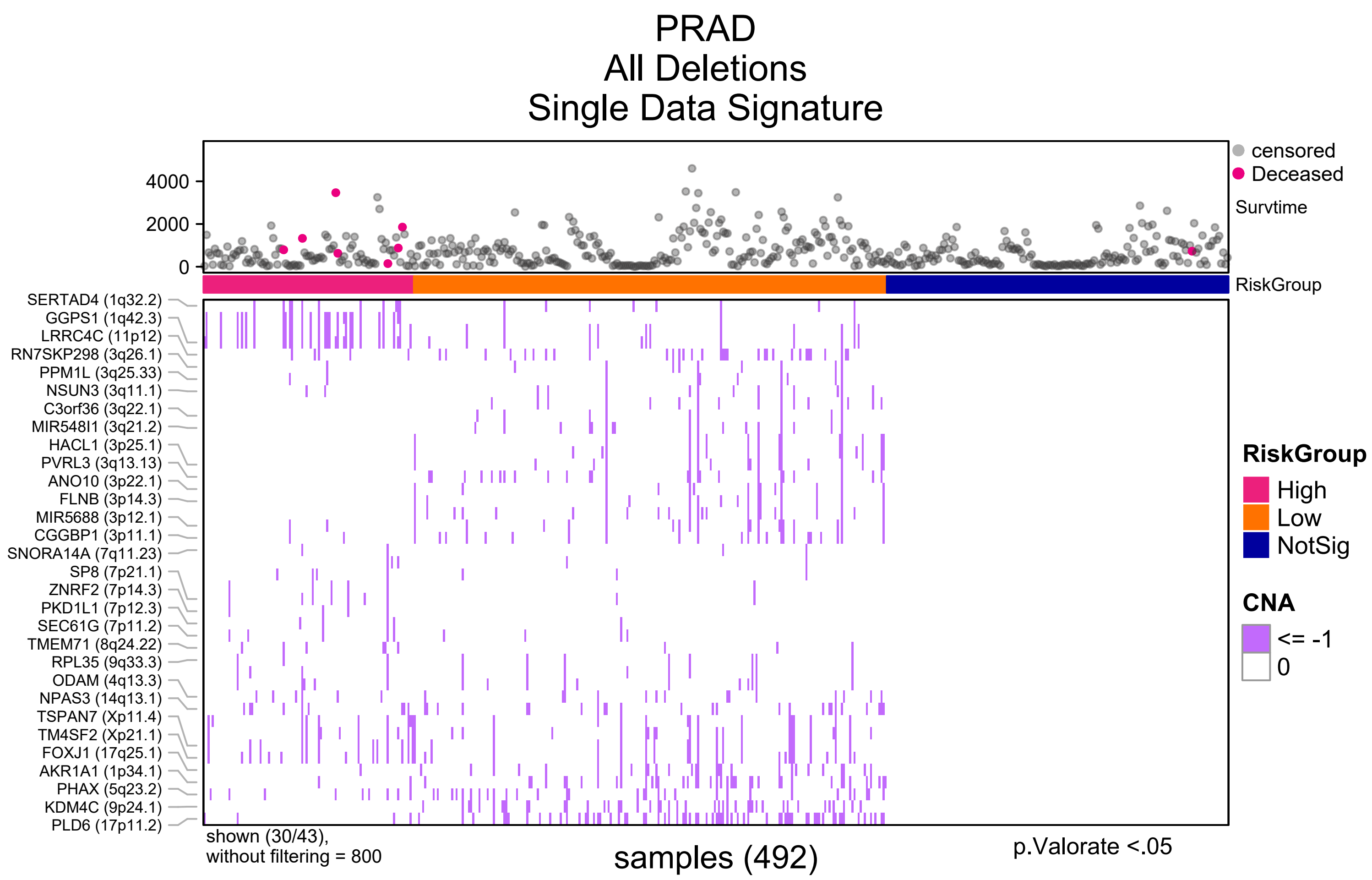

PRAD
All Deletions
Single Data Signature
censored
Deceased
Survtime
4000
2000
0
RiskGroup
SERTAD4 (1q32.2)
GGPS1 (1q42.3)
LRRC4C (11p12)
RN7SKP298 (3q26.1)
PPM1L (3q25.33)
NSUN3 (3q11.1)
C3orf36 (3q22.1)
MIR548I1 (3q21.2)
HACL1 (3p25.1)
PVRL3 (3q13.13)
ANO10 (3p22.1)
FLNB (3p14.3)
MIR5688 (3p12.1)
CGGBP1 (3p11.1)
SNORA14A (7q11.23)
SP8 (7p21.1)
ZNRF2 (7p14.3)
PKD1L1 (7p12.3)
SEC61G (7p11.2)
TMEM71 (8q24.22)
RPL35 (9q33.3)
ODAM (4q13.3)
NPAS3 (14q13.1)
TSPAN7 (Xp11.4)
TM4SF2 (Xp21.1)
FOXJ1 (17q25.1)
AKR1A1 (1p34.1)
PHAX (5q23.2)
KDM4C (9p24.1)
PLD6 (17p11.2)
RiskGroup
High
Low
NotSig
CNA
<= -1
0
shown (30/43),
without filtering = 800
samples (492)
p.Valorate <.05

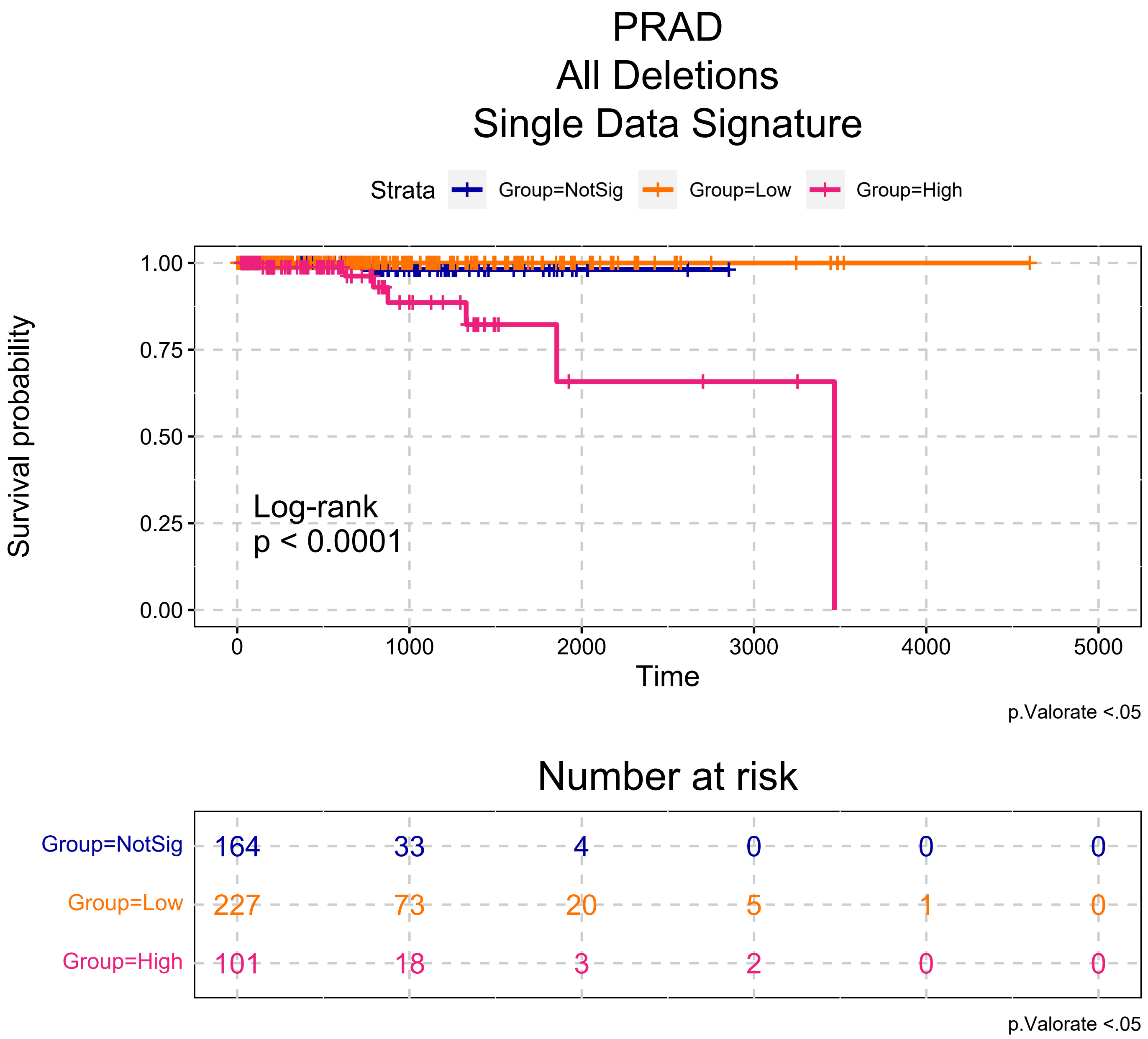

explanatory	beta	HR	L95	U95	p
Low	-19.47	0.00	0.00	Inf	1.00
High	2.24	9.38	1.13	78.07	0.04

n= 492, number of events =8
Score(logrank) test = p <.0001

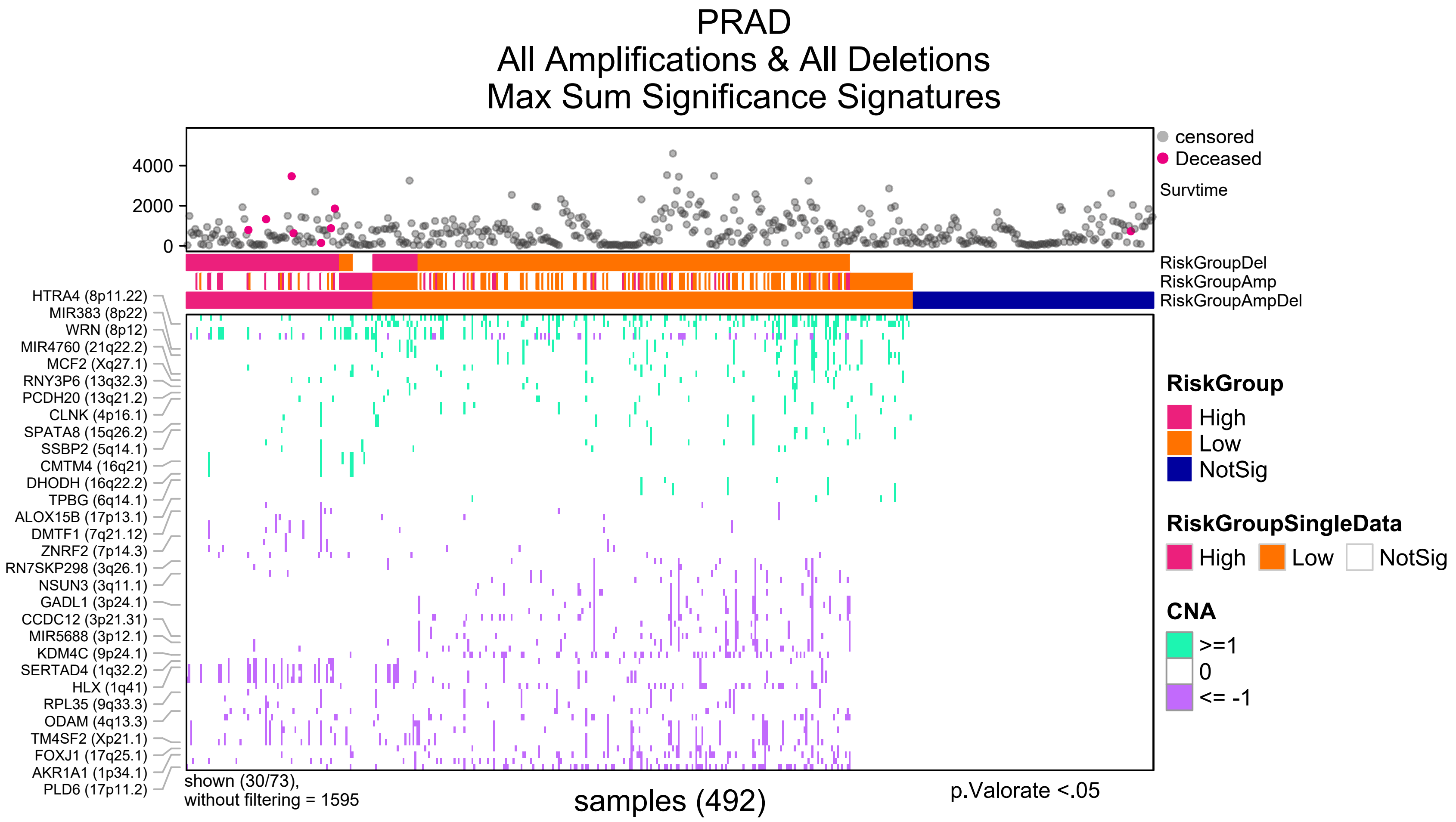

PRAD
All Amplifications & All Deletions
Max Sum Significance Signatures
4000
2000
0
censored
Deceased
Survtime
RiskGroupDel
RiskGroupAmp
RiskGroupAmpDel
HTRA4 (8p11.22)
MIR383 (8p22)
WRN (8p12)
MIR4760 (21q22.2)
MCF2 (Xq27.1)
RNY3P6 (13q32.3)
PCDH20 (13q21.2)
CLNK (4p16.1)
SPATA8 (15q26.2)
SSBP2 (5q14.1)
CMTM4 (16q21)
DHODH (16q22.2)
TPBG (6q14.1)
ALOX15B (17p13.1)
DMTF1 (7q21.12)
ZNRF2 (7p14.3)
RN7SKP298 (3q26.1)
NSUN3 (3q11.1)
GADL1 (3p24.1)
CCDC12 (3p21.31)
MIR5688 (3p12.1)
KDM4C (9p24.1)
SERTAD4 (1q32.2)
HLX (1q41)
RPL35 (9q33.3)
ODAM (4q13.3)
TM4SF2 (Xp21.1)
FOXJ1 (17q25.1)
AKR1A1 (1p34.1)
PLD6 (17p11.2)
RiskGroup
High
Low
NotSig
RiskGroupSingleData
High
Low
NotSig
CNA
>=1
0
<= -1
shown (30/73),
without filtering = 1595
samples (492)
p.Valorate <.05

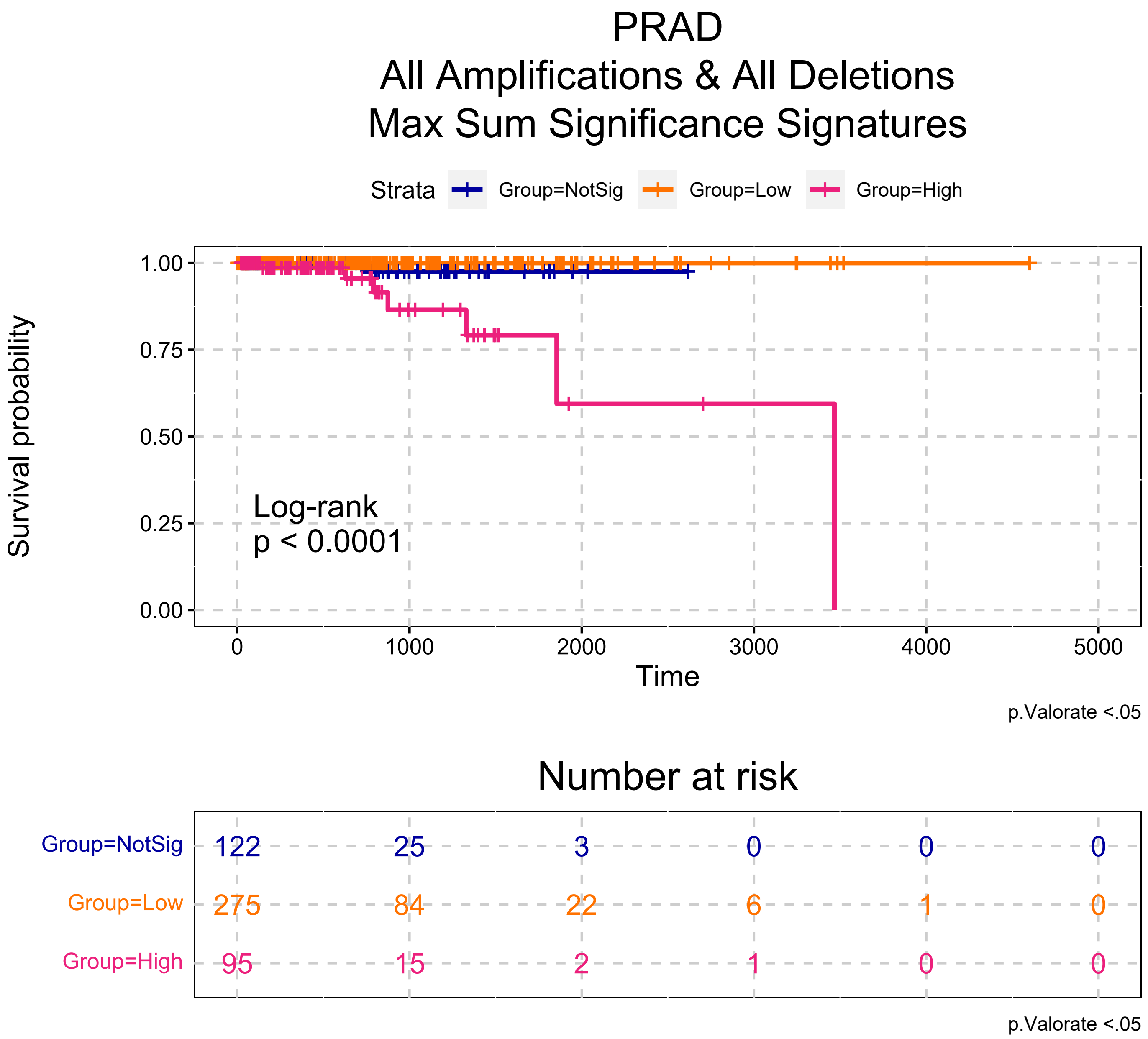

explanatory	beta	HR	L95	U95	p
Low	-19.75	0.00	0.00	Inf	1.00
High	2.11	8.29	0.99	69.17	0.05

n= 492, number of events =8
Score(logrank) test = p <.0001

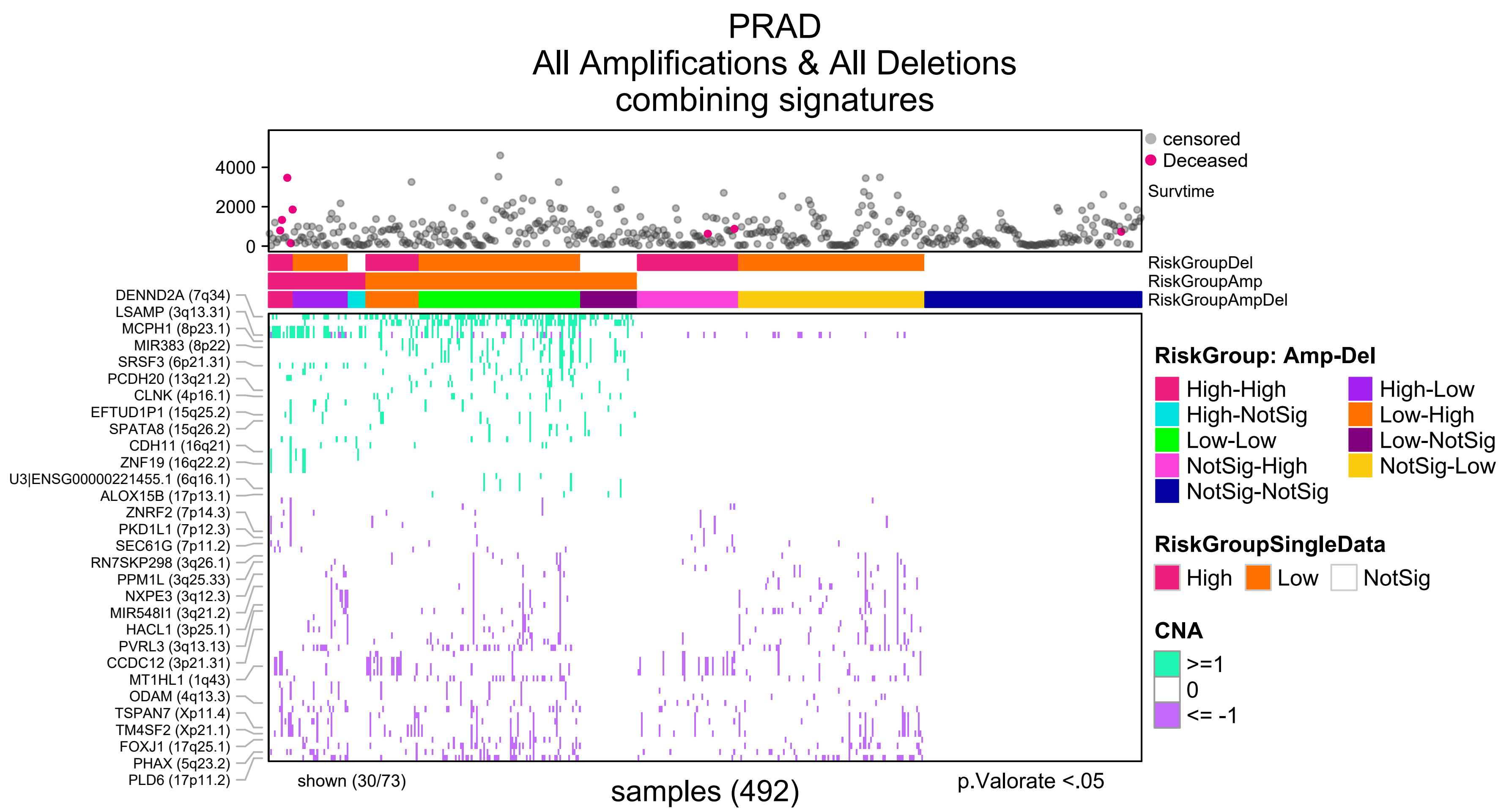
PRAD
All Amplifications & All Deletions
combining signatures
4000
2000
0
censored
Deceased
Survtime
RiskGroupDel
RiskGroupAmp
RiskGroupAmpDel
DENND2A (7q34)
LSAMP (3q13.31)
MCPH1 (8p23.1)
MIR383 (8p22)
SRSF3 (6p21.31)
PCDH20 (13q21.2)
CLNK (4p16.1)
EFTUD1P1 (15q25.2)
SPATA8 (15q26.2)
CDH11 (16q21)
ZNF19 (16q22.2)
U3|ENSG00000221455.1 (6q16.1)
ALOX15B (17p13.1)
ZNRF2 (7p14.3)
PKD1L1 (7p12.3)
SEC61G (7p11.2)
RN7SKP298 (3q26.1)
PPM1L (3q25.33)
NXPE3 (3q12.3)
MIR548I1 (3q21.2)
HACL1 (3p25.1)
PVRL3 (3q13.13)
CCDC12 (3p21.31)
MT1HL1 (1q43)
ODAM (4q13.3)
TSPAN7 (Xp11.4)
TM4SF2 (Xp21.1)
FOXJ1 (17q25.1)
PHAX (5q23.2)
PLD6 (17p11.2)
RiskGroup: Amp-Del
High-High
High-NotSig
Low-Low
NotSig-High
NotSig-NotSig
High-Low
Low-High
Low-NotSig
NotSig-Low
RiskGroupSingleData
High
Low
NotSig
CNA
>=1
0
<= -1
shown (30/73)
samples (492)
p.Valorate <.05

PRAD
All Amplifications & All Deletions
combining signatures

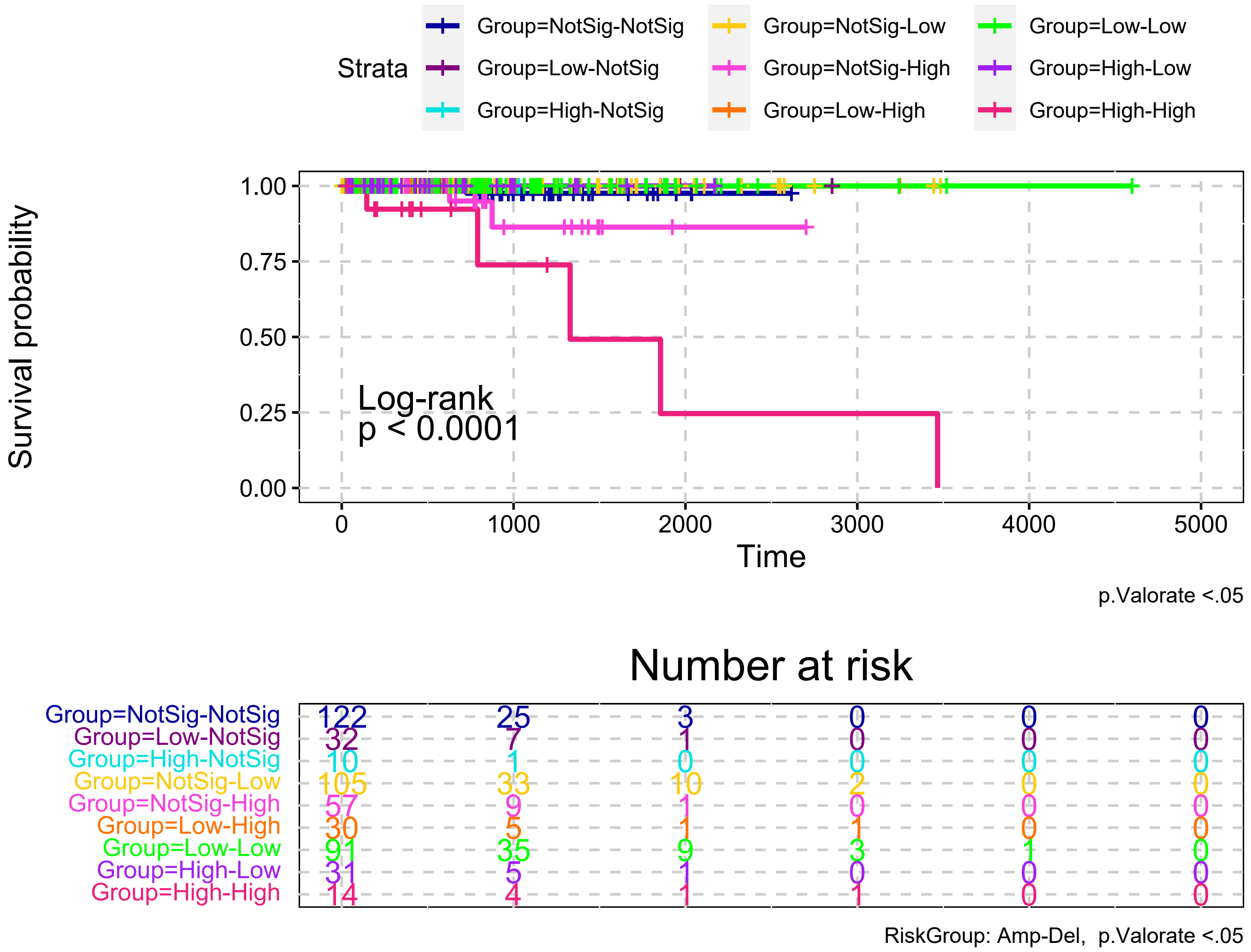

explanatory	beta	HR	L95	U95	p
Low-NotSig	-19.33	0.00	0.00	Inf	1.00
High-NotSig	-19.37	0.00	0.00	Inf	1.00
NotSig-Low	-19.19	0.00	0.00	Inf	1.00
NotSig-High	1.53	4.64	0.89	24.23	0.07
Low-High	-19.35	0.00	0.00	Inf	1.00
Low-Low	-19.08	0.00	0.00	Inf	1.00
High-Low	-19.35	0.00	0.00	Inf	1.00
High-High	3.19	24.39	5.32	111.87	0.00

n= 492, number of events =8
Score(logrank) test = p <.0001

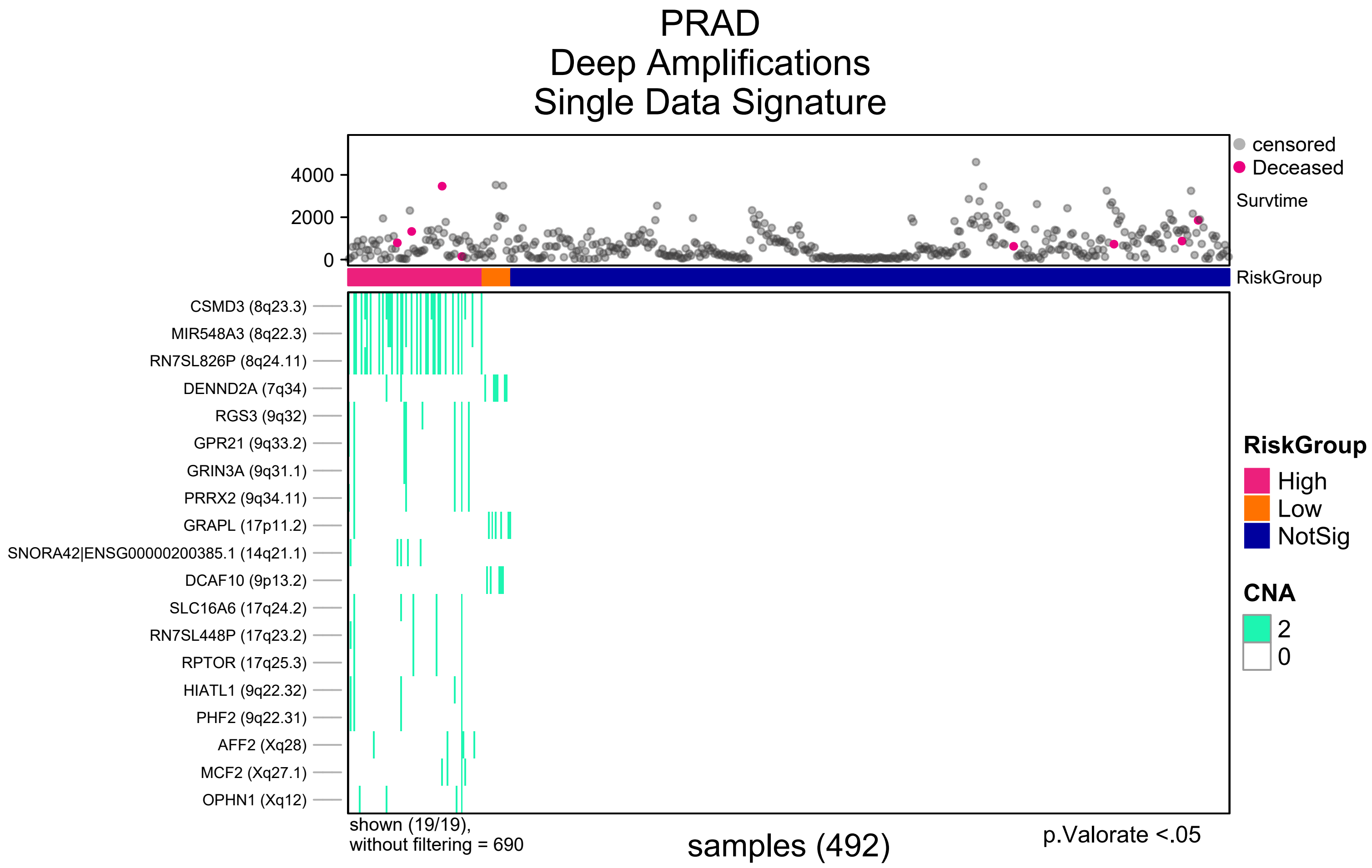
PRAD
Deep Amplifications
Single Data Signature
4000
2000
0
censored
Deceased
Survtime
RiskGroup
CSMD3 (8q23.3)
MIR548A3 (8q22.3)
RN7SL826P (8q24.11)
DENND2A (7q34)
RGS3 (9q32)
GPR21 (9q33.2)
GRIN3A (9q31.1)
PRRX2 (9q34.11)
GRAPL (17p11.2)
SNORA42|ENSG00000200385.1 (14q21.1)
DCAF10 (9p13.2)
SLC16A6 (17q24.2)
RN7SL448P (17q23.2)
RPTOR (17q25.3)
HIATL1 (9q22.32)
PHF2 (9q22.31)
AFF2 (Xq28)
MCF2 (Xq27.1)
OPHN1 (Xq12)
RiskGroup
High
Low
NotSig
CNA
2
0
shown (19/19),
without filtering = 690
samples (492)
p.Valorate <.05

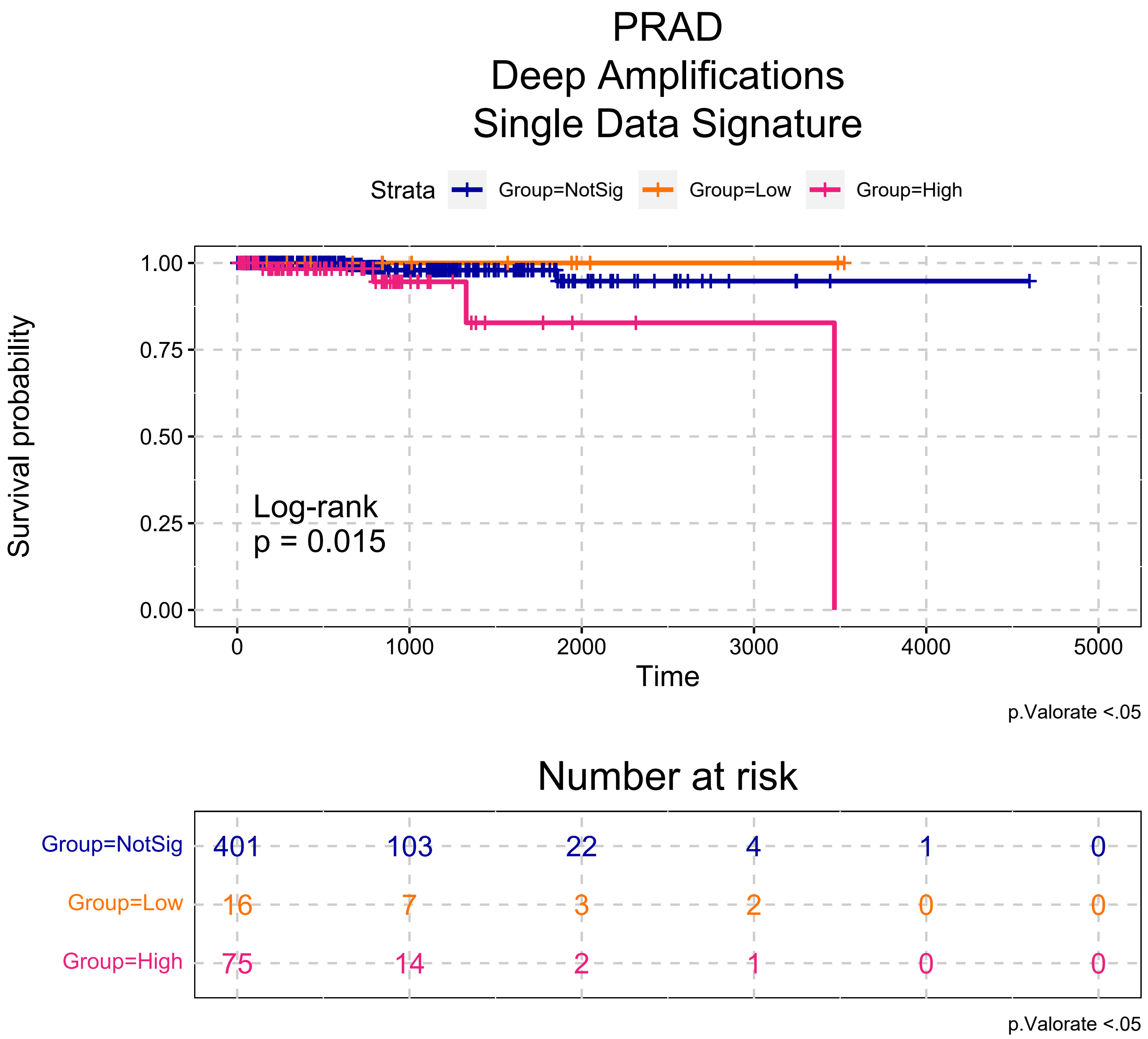

explanatory	beta	HR	L95	U95	p
Low	-17.65	0.00	0.00	Inf	1.00
High	1.64	5.16	1.22	21.88	0.03

n= 492, number of events =8
Score(logrank) test = 0.015

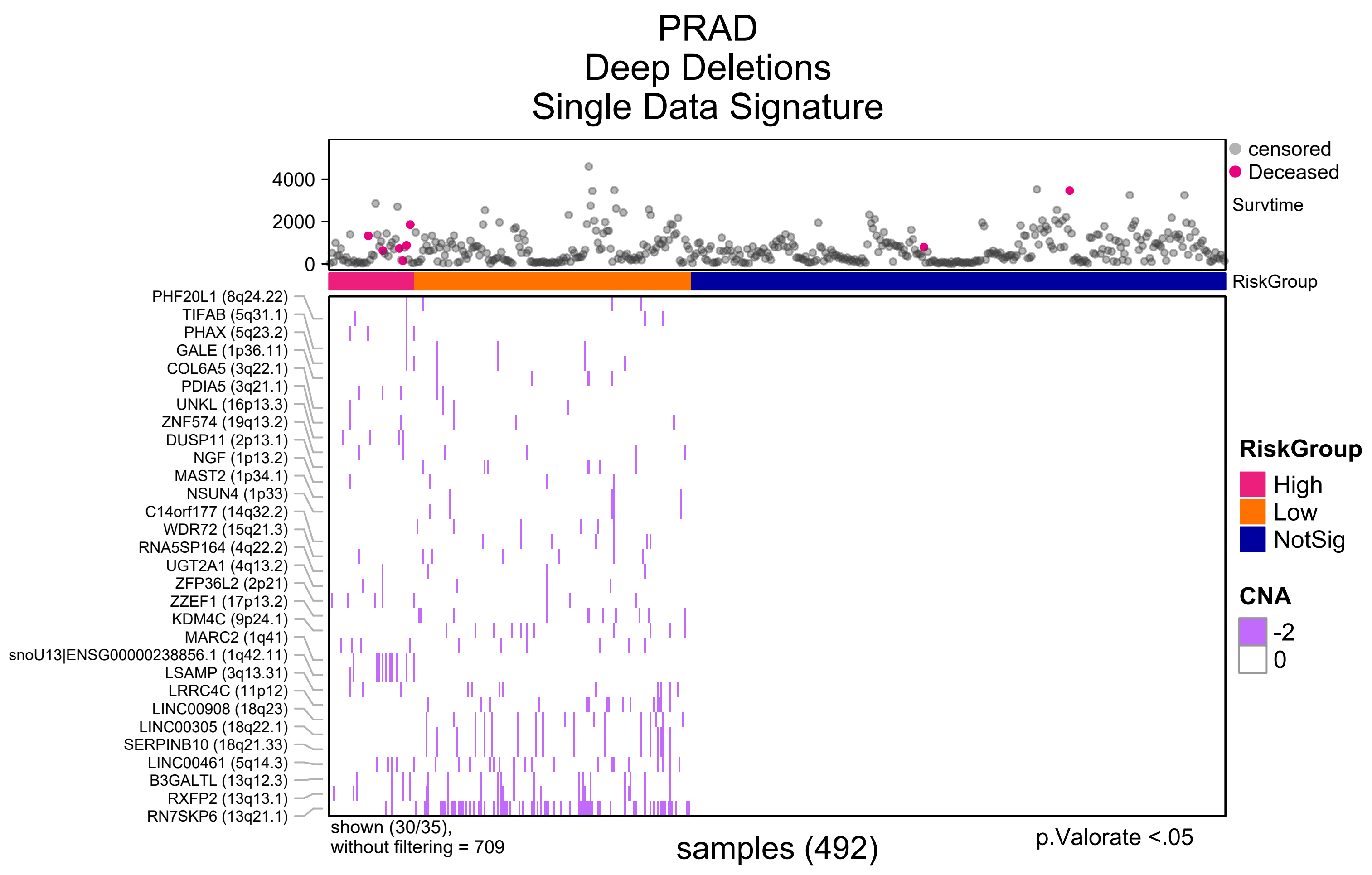

PRAD
Deep Deletions
Single Data Signature
4000
2000
0
censored
Deceased
Survtime
RiskGroup
PHF20L1 (8q24.22)
TIFAB (5q31.1)
PHAX (5q23.2)
GALE (1p36.11)
COL6A5 (3q22.1)
PDIA5 (3q21.1)
UNKL (16p13.3)
ZNF574 (19q13.2)
DUSP11 (2p13.1)
NGF (1p13.2)
MAST2 (1p34.1)
NSUN4 (1p33)
C14orf177 (14q32.2)
WDR72 (15q21.3)
RNA5SP164 (4q22.2)
UGT2A1 (4q13.2)
ZFP36L2 (2p21)
ZZEF1 (17p13.2)
KDM4C (9p24.1)
MARC2 (1q41)
snoU13|ENSG00000238856.1 (1q42.11)
LSAMP (3q13.31)
LRRC4C (11p12)
LINC00908 (18q23)
LINC00305 (18q22.1)
SERPINB10 (18q21.33)
LINC00461 (5q14.3)
B3GALTL (13q12.3)
RXFP2 (13q13.1)
RN7SKP6 (13q21.1)
RiskGroup
High
Low
NotSig
CNA
-2
0
shown (30/35),
without filtering = 709
samples (492)
p.Valorate <.05

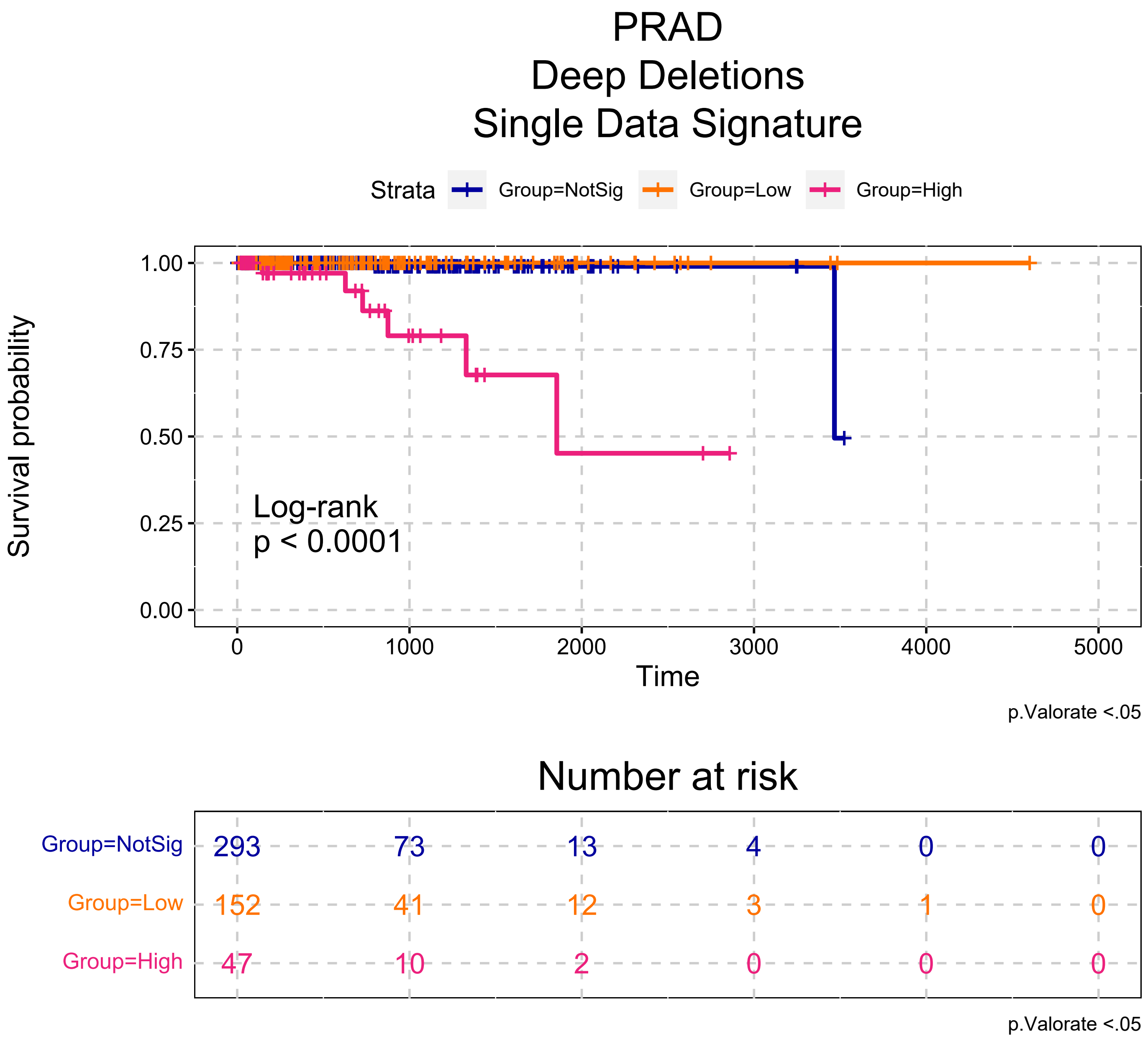

explanatory	beta	HR	L95	U95
Low	-12.08	0.00	0.00	352104869762747101162[illegible]
High	3.71	40.83	4.91	339.30

n= 492, number of events =8
Score(logrank) test = p <.0001

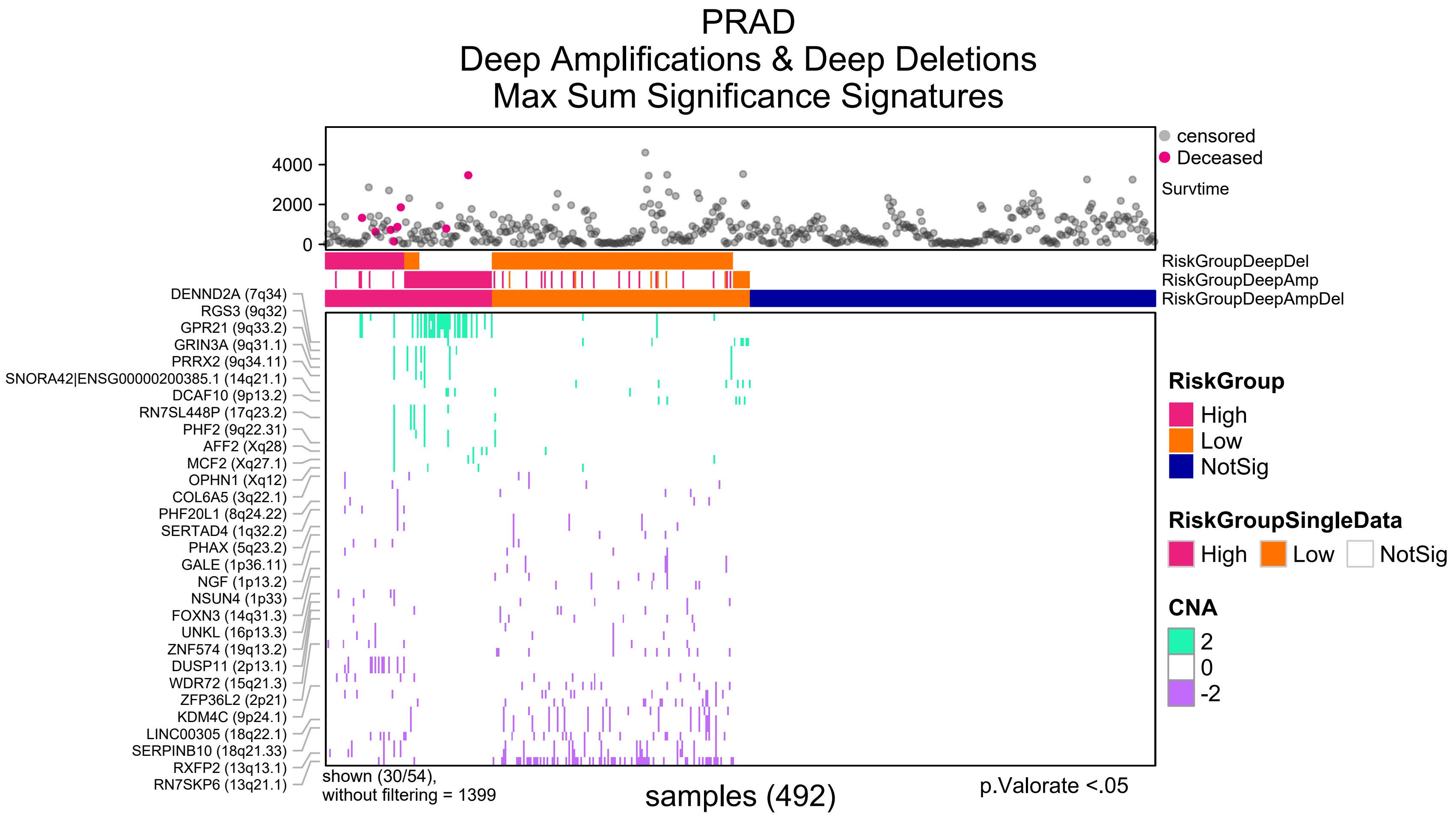

PRAD
Deep Amplifications & Deep Deletions
Max Sum Significance Signatures
censored
Deceased
Survtime
4000
2000
0
RiskGroupDeepDel
RiskGroupDeepAmp
RiskGroupDeepAmpDel
DENND2A (7q34)
RGS3 (9q32)
GPR21 (9q33.2)
GRIN3A (9q31.1)
PRRX2 (9q34.11)
SNORA42|ENSG00000200385.1 (14q21.1)
DCAF10 (9p13.2)
RN7SL448P (17q23.2)
PHF2 (9q22.31)
AFF2 (Xq28)
MCF2 (Xq27.1)
OPHN1 (Xq12)
COL6A5 (3q22.1)
PHF20L1 (8q24.22)
SERTAD4 (1q32.2)
PHAX (5q23.2)
GALE (1p36.11)
NGF (1p13.2)
NSUN4 (1p33)
FOXN3 (14q31.3)
UNKL (16p13.3)
ZNF574 (19q13.2)
DUSP11 (2p13.1)
WDR72 (15q21.3)
ZFP36L2 (2p21)
KDM4C (9p24.1)
LINC00305 (18q22.1)
SERPINB10 (18q21.33)
RXFP2 (13q13.1)
RN7SKP6 (13q21.1)
RiskGroup
High
Low
NotSig
RiskGroupSingleData
High
Low
NotSig
CNA
2
0
-2
shown (30/54),
without filtering = 1399
samples (492)
p.Valorate <.05

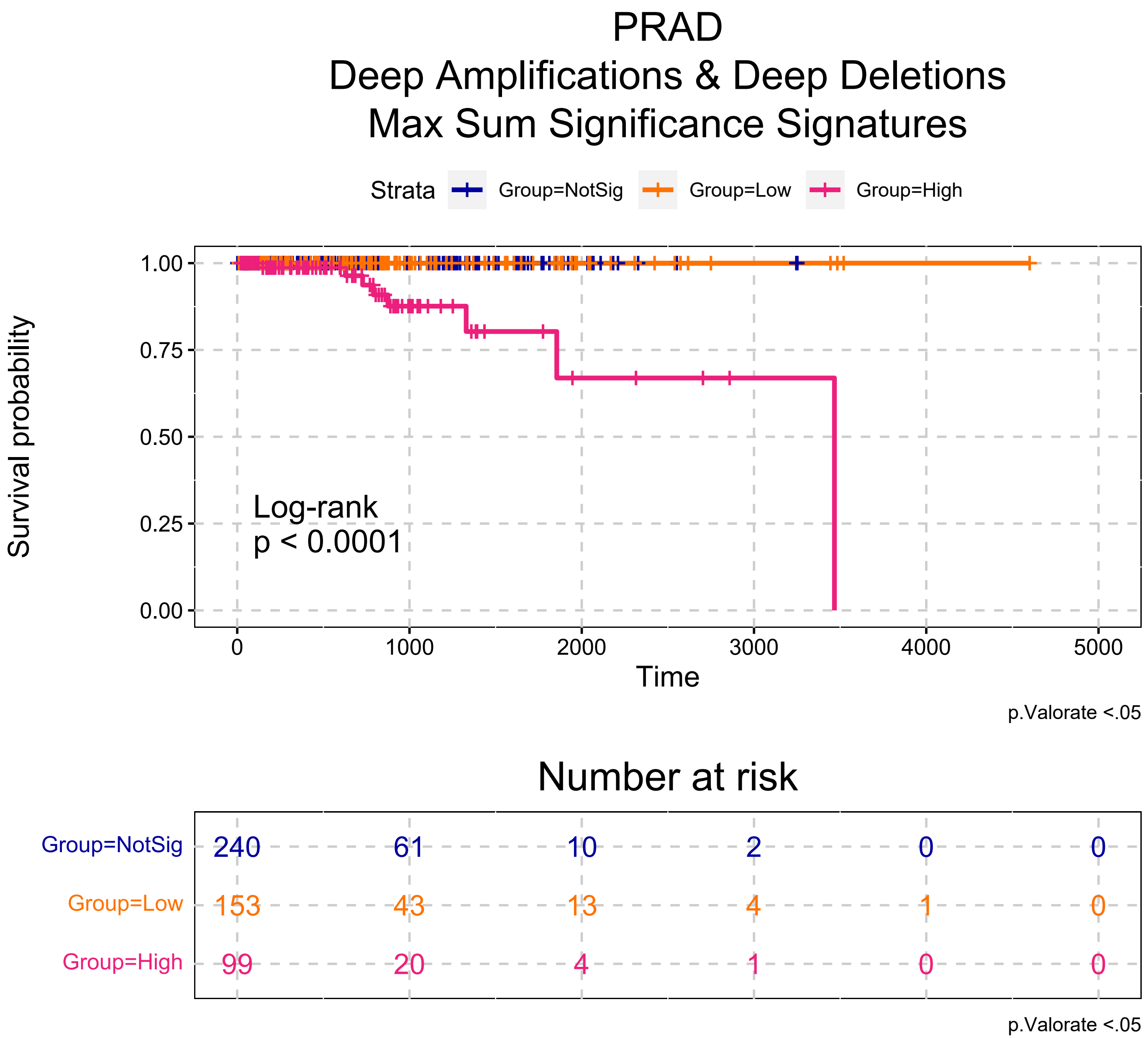

explanatory	beta	HR	L95	U95	p
Low	0.07	1.08	0.00	Inf	1.00
High	22.36	5160098526.87	0.00	Inf	1.00

n= 492, number of events =8
Score(logrank) test = p <.0001

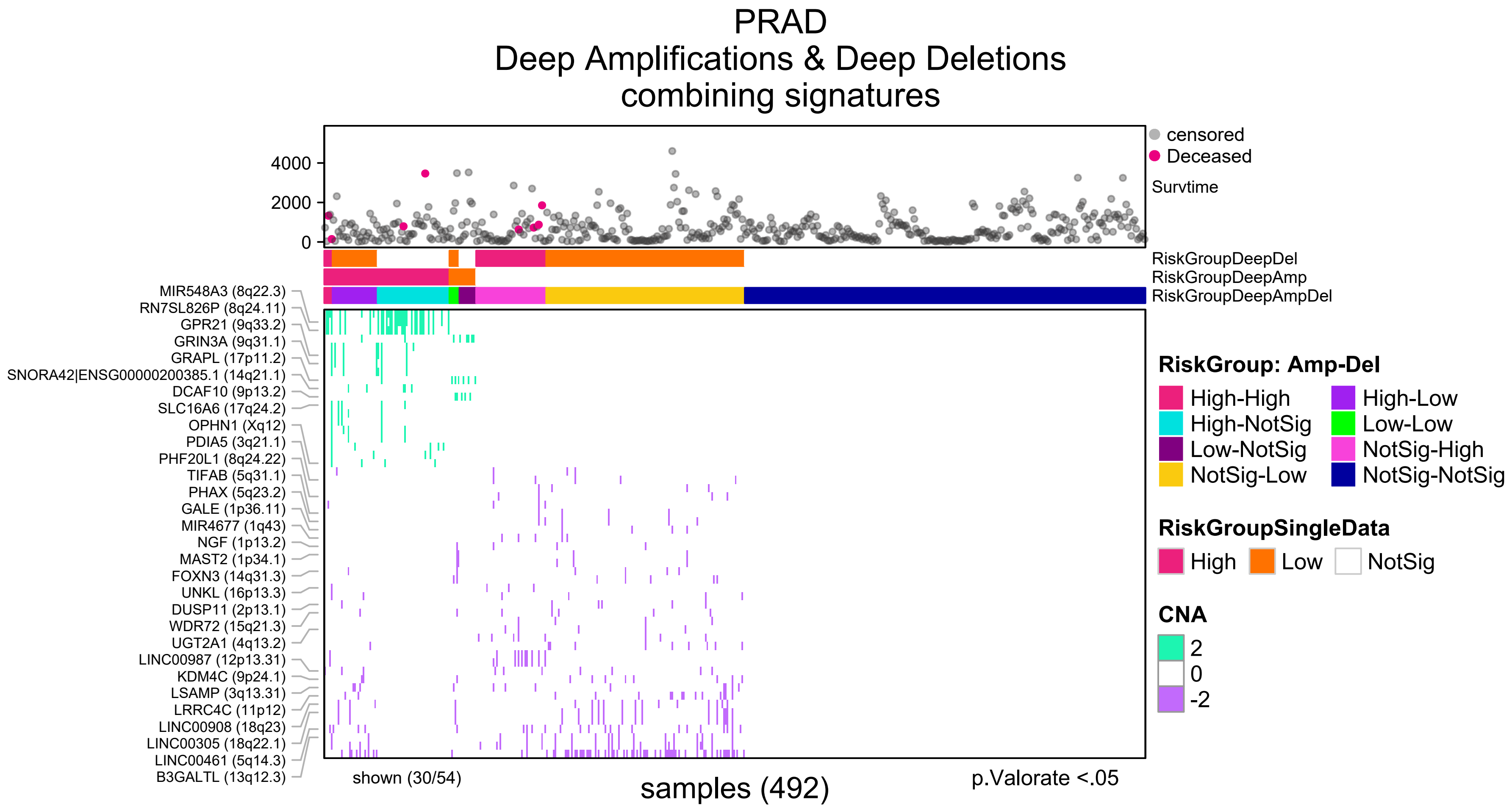
PRAD
Deep Amplifications & Deep Deletions
combining signatures
censored
Deceased
Survtime
4000
2000
0
RiskGroupDeepDel
RiskGroupDeepAmp
RiskGroupDeepAmpDel
MIR548A3 (8q22.3)
RN7SL826P (8q24.11)
GPR21 (9q33.2)
GRIN3A (9q31.1)
GRAPL (17p11.2)
SNORA42|ENSG00000200385.1 (14q21.1)
DCAF10 (9p13.2)
SLC16A6 (17q24.2)
OPHN1 (Xq12)
PDIA5 (3q21.1)
PHF20L1 (8q24.22)
TIFAB (5q31.1)
PHAX (5q23.2)
GALE (1p36.11)
MIR4677 (1q43)
NGF (1p13.2)
MAST2 (1p34.1)
FOXN3 (14q31.3)
UNKL (16p13.3)
DUSP11 (2p13.1)
WDR72 (15q21.3)
UGT2A1 (4q13.2)
LINC00987 (12p13.31)
KDM4C (9p24.1)
LSAMP (3q13.31)
LRRC4C (11p12)
LINC00908 (18q23)
LINC00305 (18q22.1)
LINC00461 (5q14.3)
B3GALTL (13q12.3)
RiskGroup: Amp-Del
High-High
High-Low
High-NotSig
Low-Low
Low-NotSig
NotSig-High
NotSig-Low
NotSig-NotSig
RiskGroupSingleData
High
Low
NotSig
CNA
2
0
-2
shown (30/54)
samples (492)
p.Valorate <.05

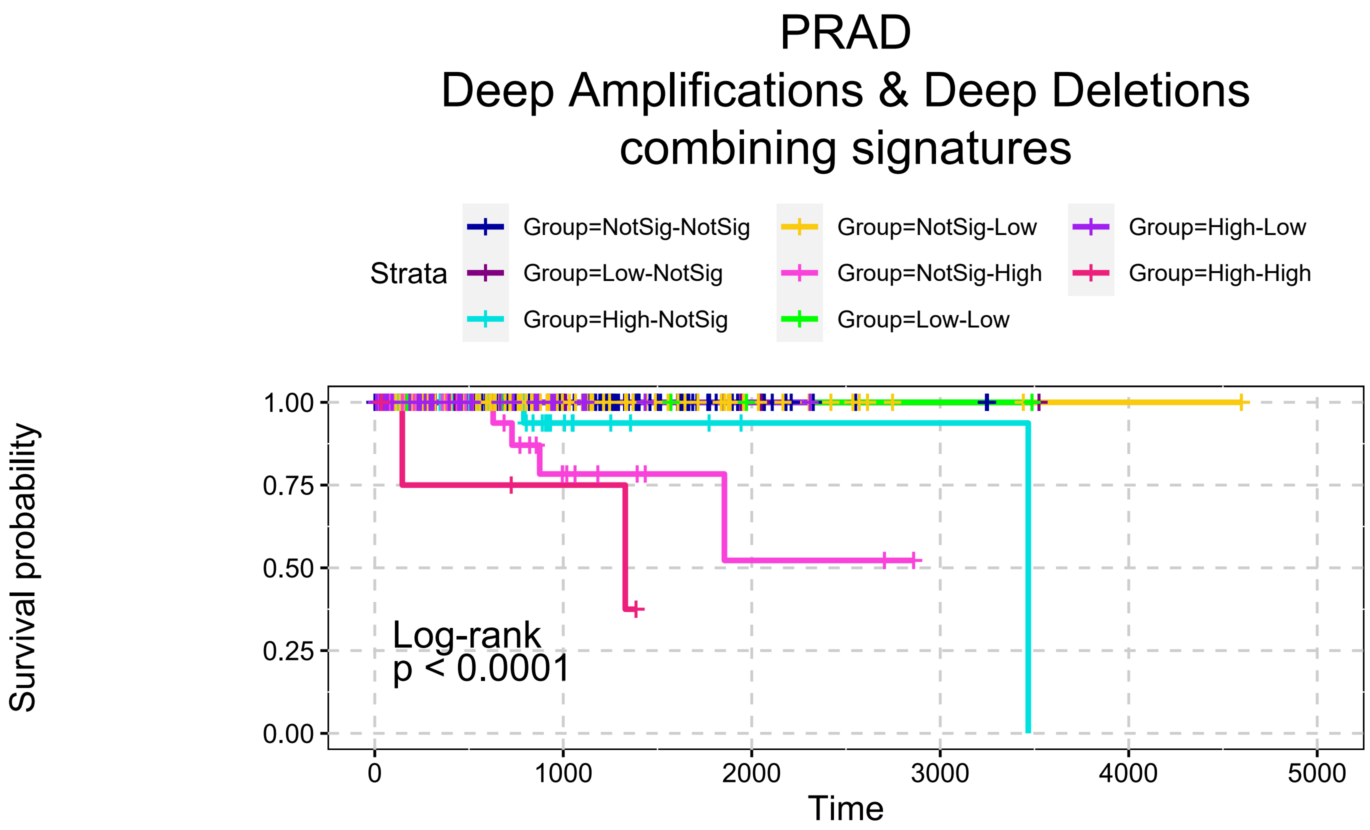

p.Valorate <.05

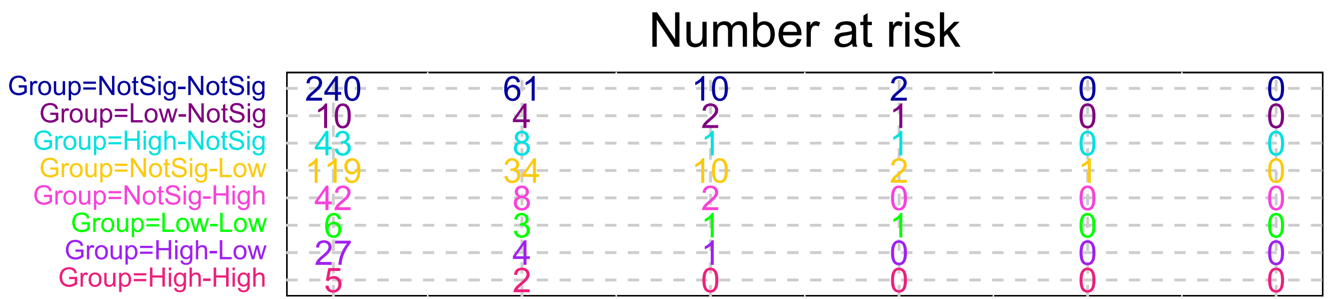

RiskGroup: Amp-Del, p.Valorate <.05

explanatory	beta	HR	L95	U95
Low-NotSig	0.23	1.26	0.00	Inf
High-NotSig	19.65	342850159.54	40959709.00	286980
NotSig-Low	0.11	1.12	0.00	Inf
NotSig-High	21.11	1475091700.51	312005257.14	697390
Low-Low	0.24	1.27	0.00	Inf
High-Low	-0.00	1.00	0.00	Inf
High-High	22.30	4829392886.49	856730266.29	272233

n= 492, number of events =8
Score(logrank) test = p <.0001
