# Supplementary material for: Systematic Modeling of Risk-Associated Copy Number Alterations in Cancer
Source: Int J Mol Sci. 2024 Sep 27;25(19):10455. doi: 10.3390/ijms251910455 (PMC11477427; doi:10.3390/ijms251910455)
Supplement: Supplementary file 1 [file ijms-25-10455-s001.zip › READSignatureV12-sinSombreado.pdf]

READ
All Amplifications
Single Data Signature

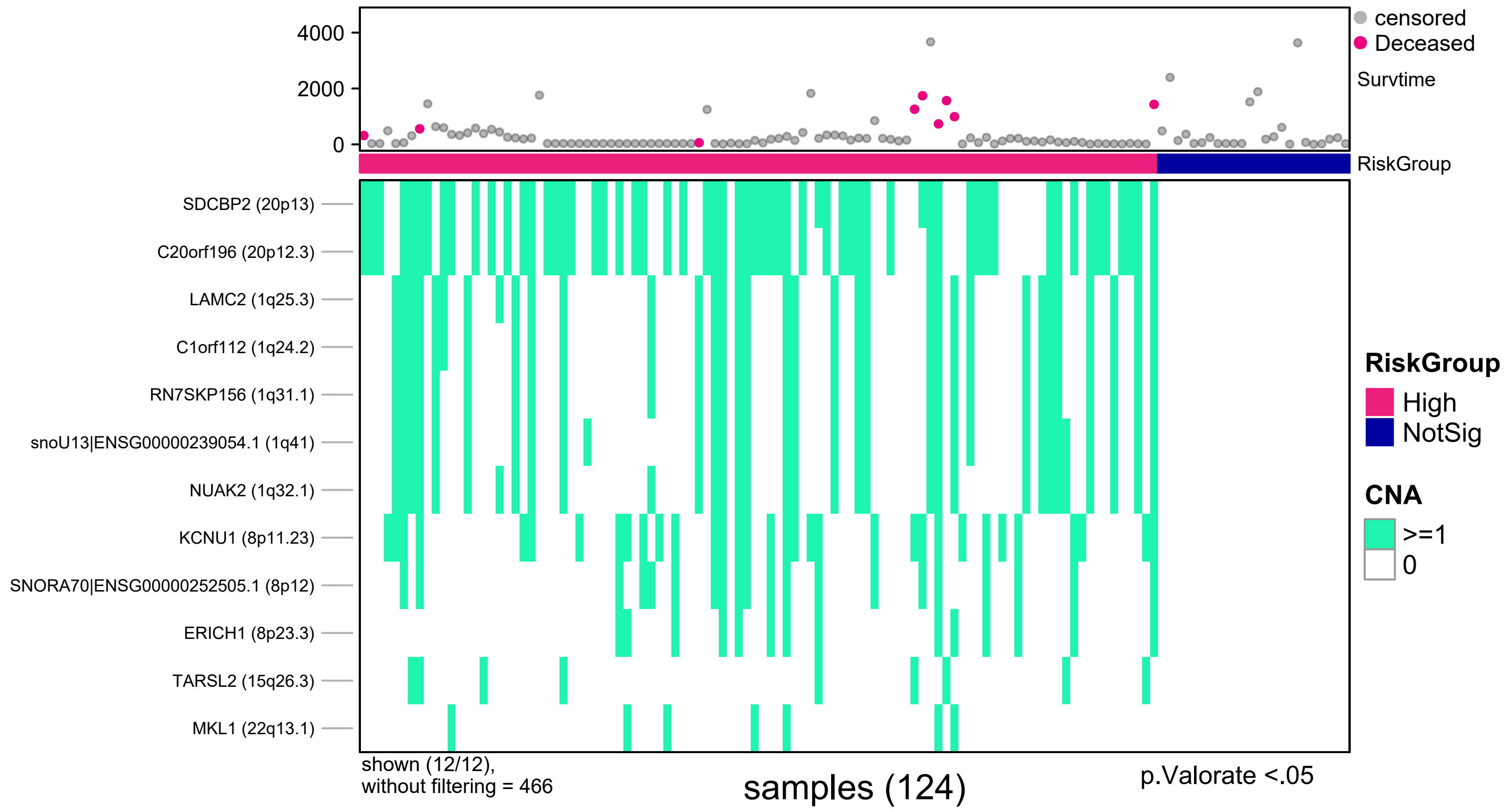

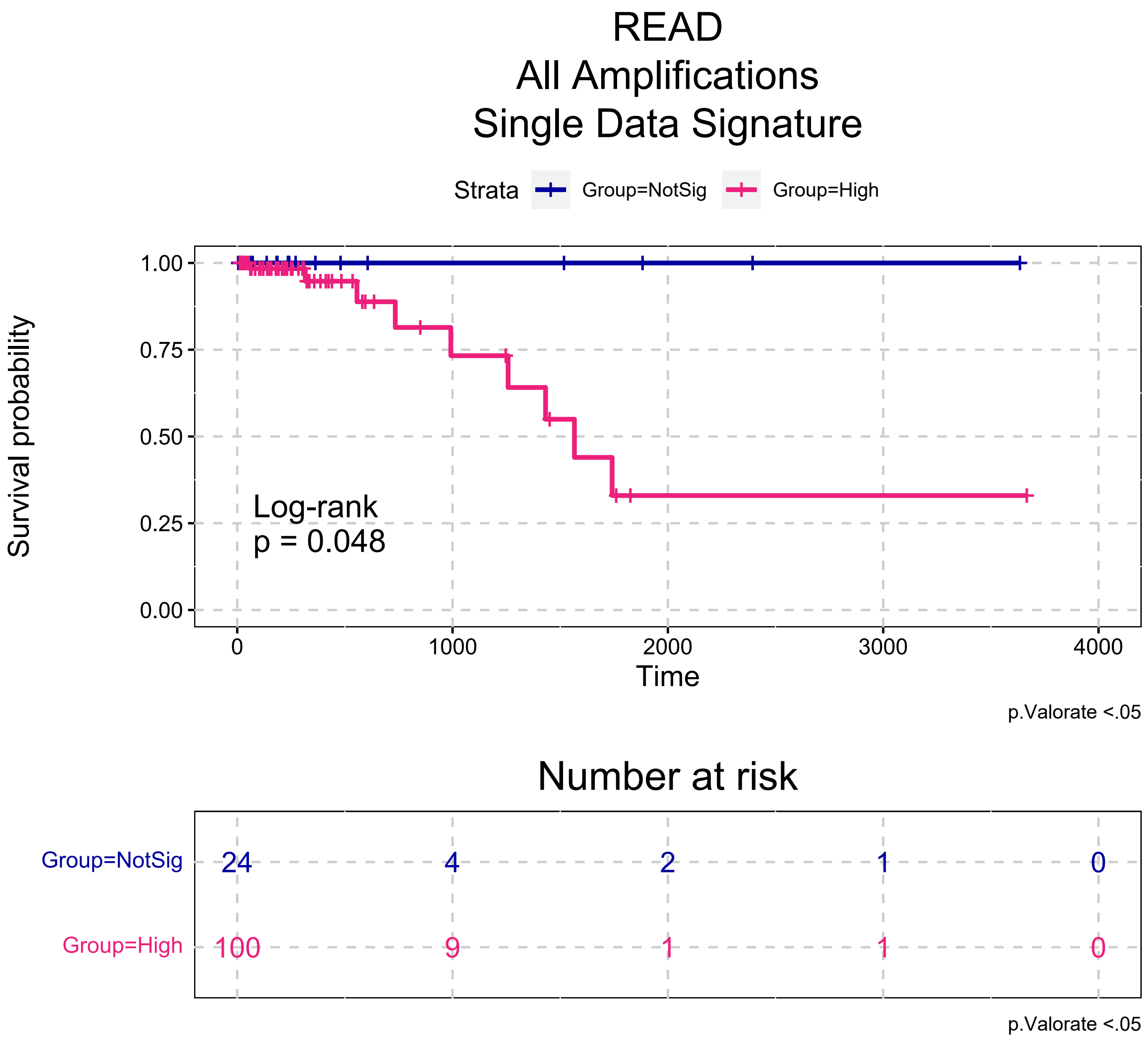

explanatory	beta	HR	L95	U95	p
High	19.64	337551252.13	0.00	Inf	1.00

n= 124, number of events =9
Score(logrank) test = 0.048

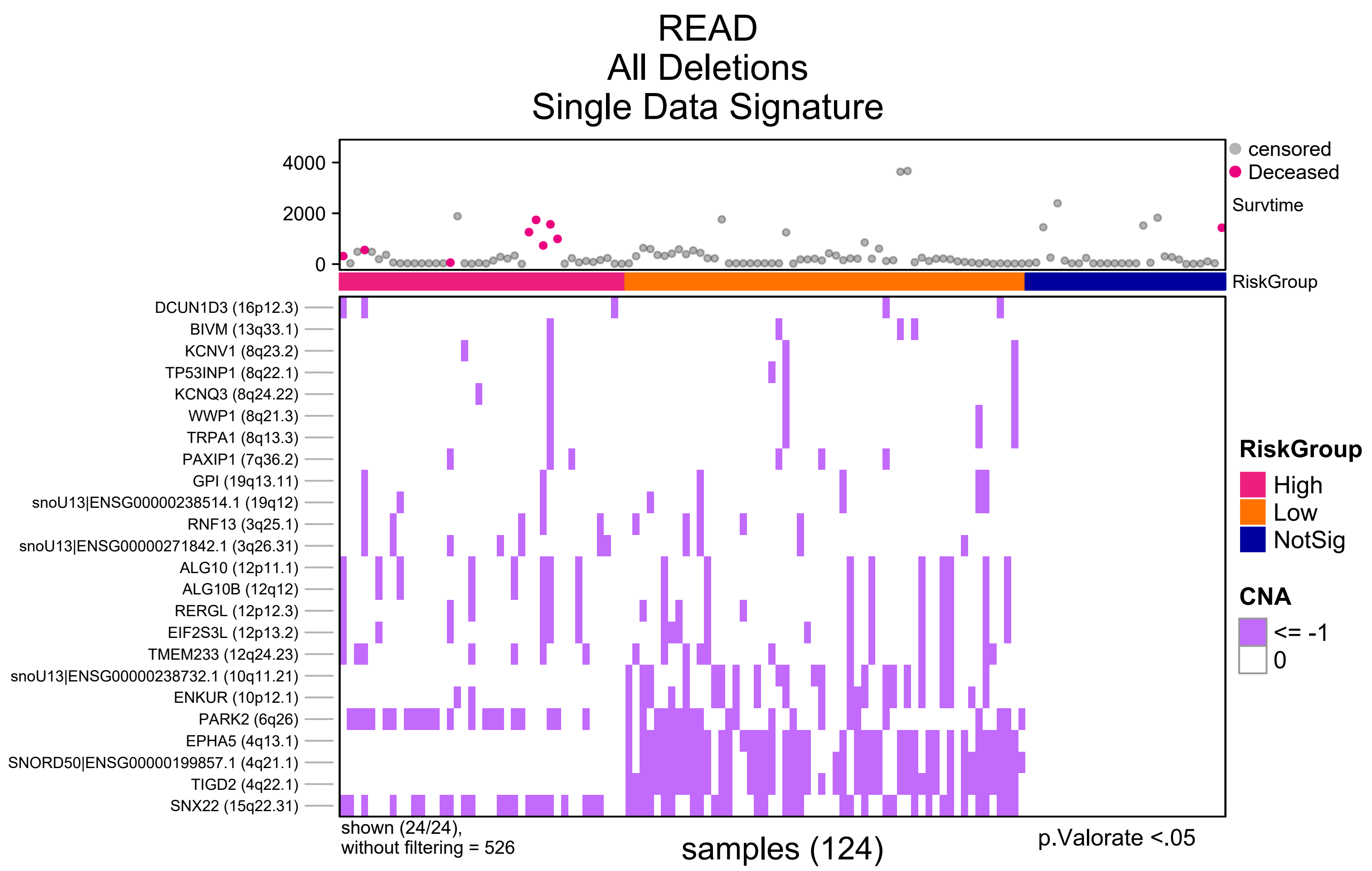
READ
All Deletions
Single Data Signature
4000
2000
0
censored
Deceased
Survtime
RiskGroup
DCUN1D3 (16p12.3)
BIVM (13q33.1)
KCNV1 (8q23.2)
TP53INP1 (8q22.1)
KCNQ3 (8q24.22)
WWP1 (8q21.3)
TRPA1 (8q13.3)
PAXIP1 (7q36.2)
GPI (19q13.11)
snoU13|ENSG00000238514.1 (19q12)
RNF13 (3q25.1)
snoU13|ENSG00000271842.1 (3q26.31)
ALG10 (12p11.1)
ALG10B (12q12)
RERGL (12p12.3)
EIF2S3L (12p13.2)
TMEM233 (12q24.23)
snoU13|ENSG00000238732.1 (10q11.21)
ENKUR (10p12.1)
PARK2 (6q26)
EPHA5 (4q13.1)
SNORD50|ENSG00000199857.1 (4q21.1)
TIGD2 (4q22.1)
SNX22 (15q22.31)
RiskGroup
High
Low
NotSig
CNA
<= -1
0
shown (24/24),
without filtering = 526
samples (124)
p.Valorate <.05

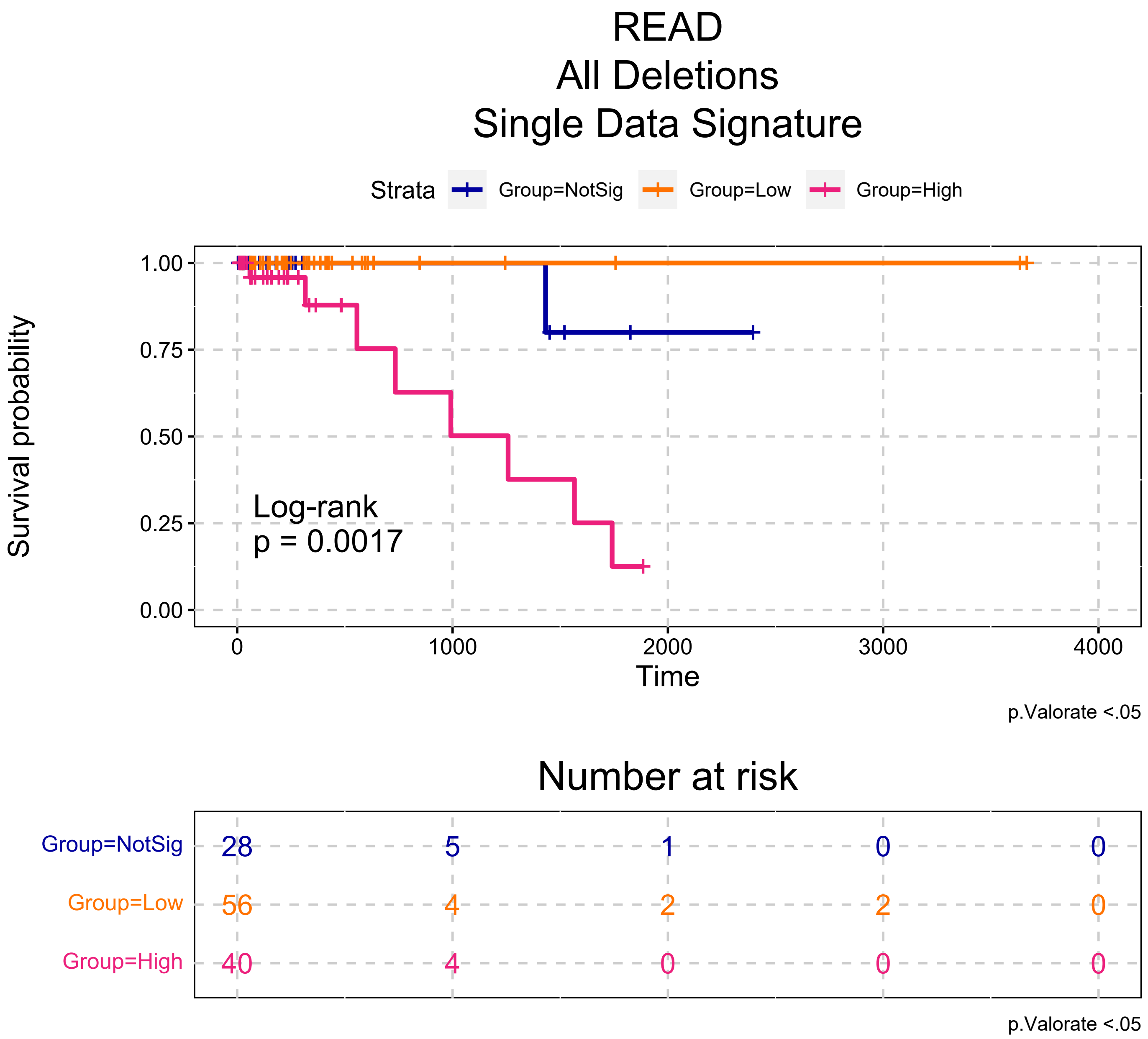

explanatory	beta	HR	L95	U95	p
Low	-18.83	0.00	0.00	Inf	1.00
High	1.95	7.06	0.87	57.40	0.07

n= 124, number of events =9
Score(logrank) test = 0.002

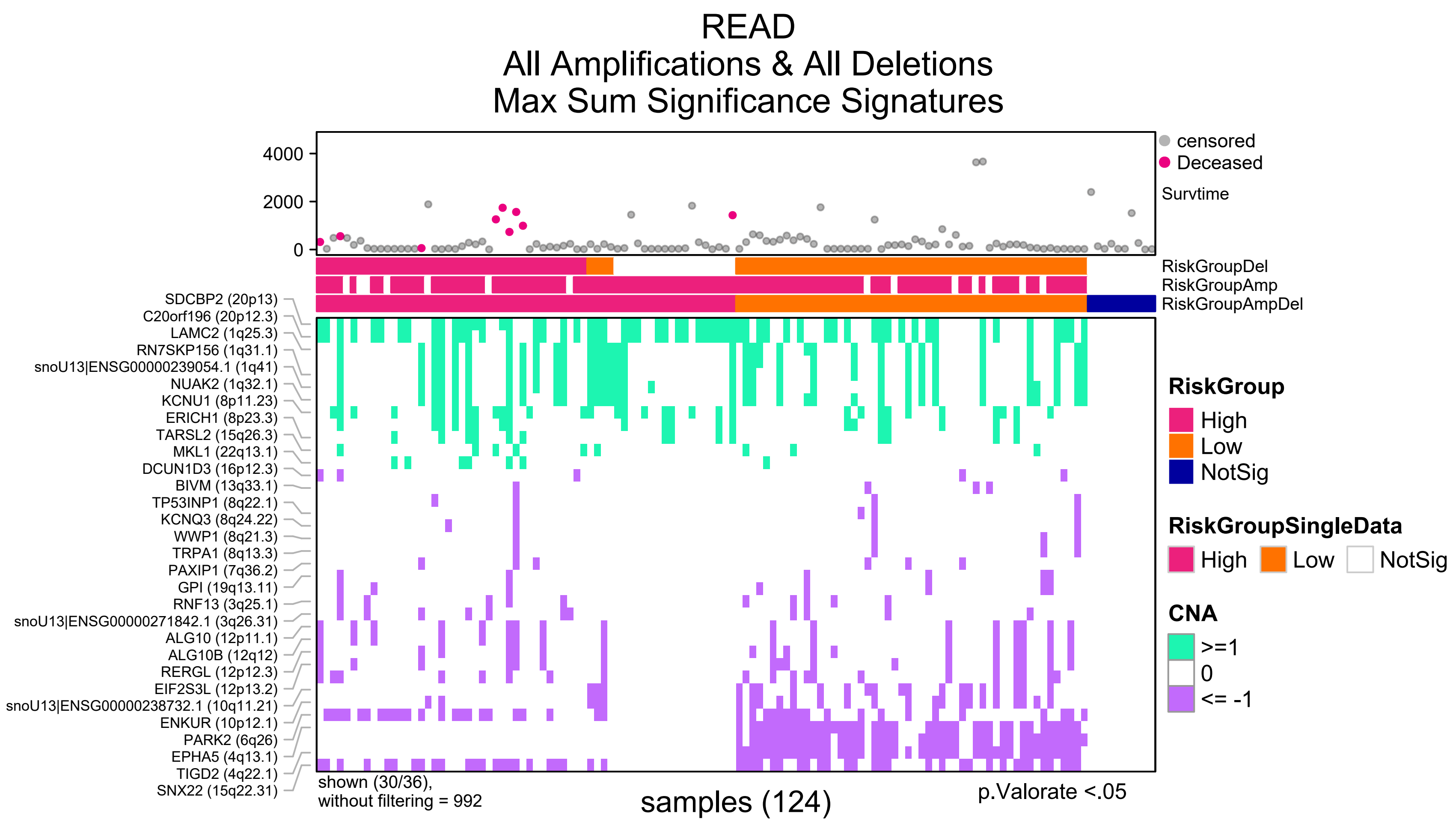

READ
All Amplifications & All Deletions
Max Sum Significance Signatures
4000
2000
0
censored
Deceased
Survtime
RiskGroupDel
RiskGroupAmp
RiskGroupAmpDel
SDCBP2 (20p13)
C20orf196 (20p12.3)
LAMC2 (1q25.3)
RN7SKP156 (1q31.1)
snoU13|ENSG00000239054.1 (1q41)
NUAK2 (1q32.1)
KCNU1 (8p11.23)
ERICH1 (8p23.3)
TARSL2 (15q26.3)
MKL1 (22q13.1)
DCUN1D3 (16p12.3)
BIVM (13q33.1)
TP53INP1 (8q22.1)
KCNQ3 (8q24.22)
WWP1 (8q21.3)
TRPA1 (8q13.3)
PAXIP1 (7q36.2)
GPI (19q13.11)
RNF13 (3q25.1)
snoU13|ENSG00000271842.1 (3q26.31)
ALG10 (12p11.1)
ALG10B (12q12)
RERGL (12p12.3)
EIF2S3L (12p13.2)
snoU13|ENSG00000238732.1 (10q11.21)
ENKUR (10p12.1)
PARK2 (6q26)
EPHA5 (4q13.1)
TIGD2 (4q22.1)
SNX22 (15q22.31)
RiskGroup
High
Low
NotSig
RiskGroupSingleData
High
Low
NotSig
CNA
>=1
0
<= -1
shown (30/36),
without filtering = 992
samples (124)
p.Valorate <.05

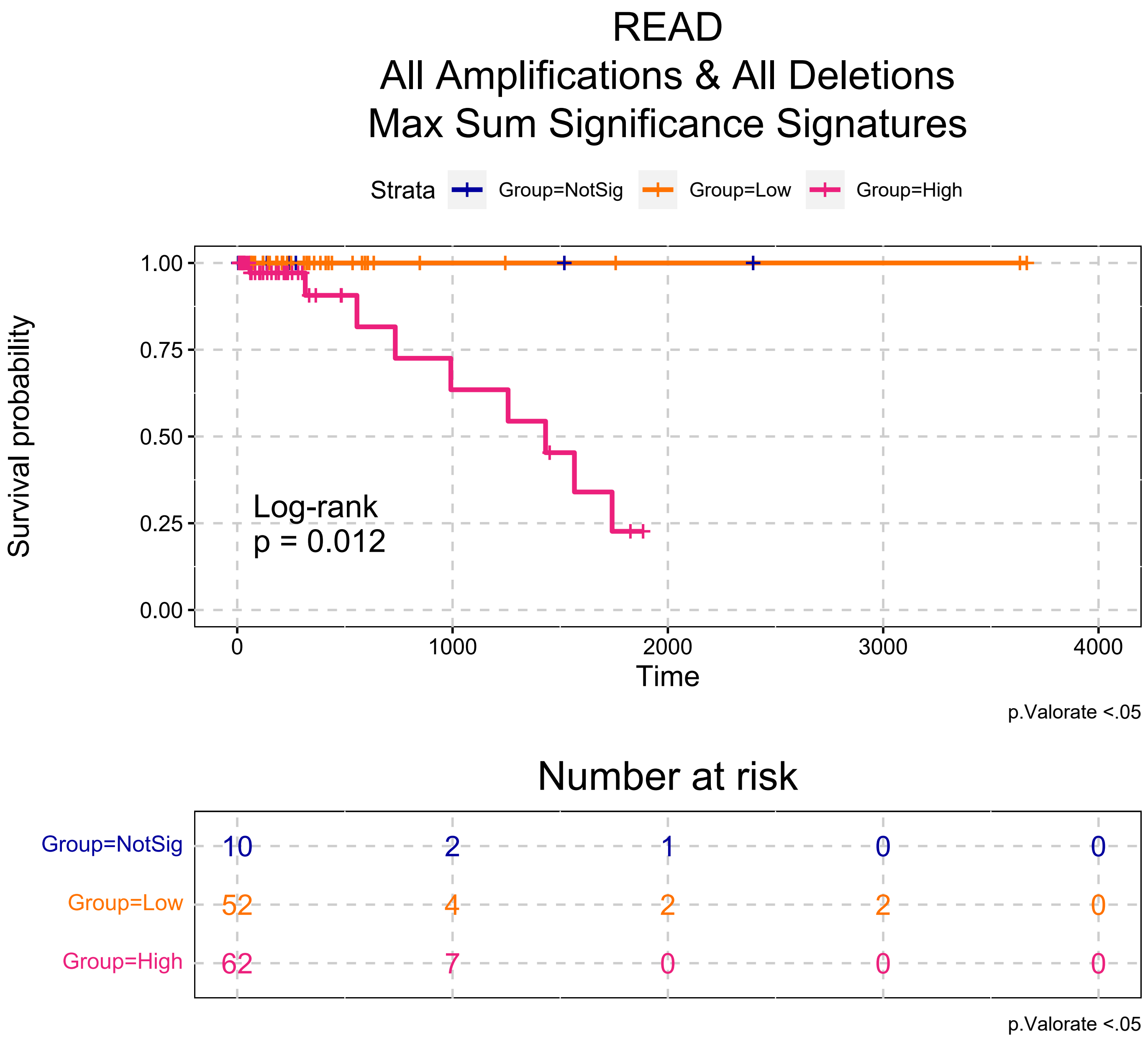

explanatory	beta	HR	L95	U95	p
Low	-0.07	0.93	0.00	Inf	1.00
High	21.16	1543285774.78	0.00	Inf	1.00

n= 124, number of events =9
Score(logrank) test = 0.012

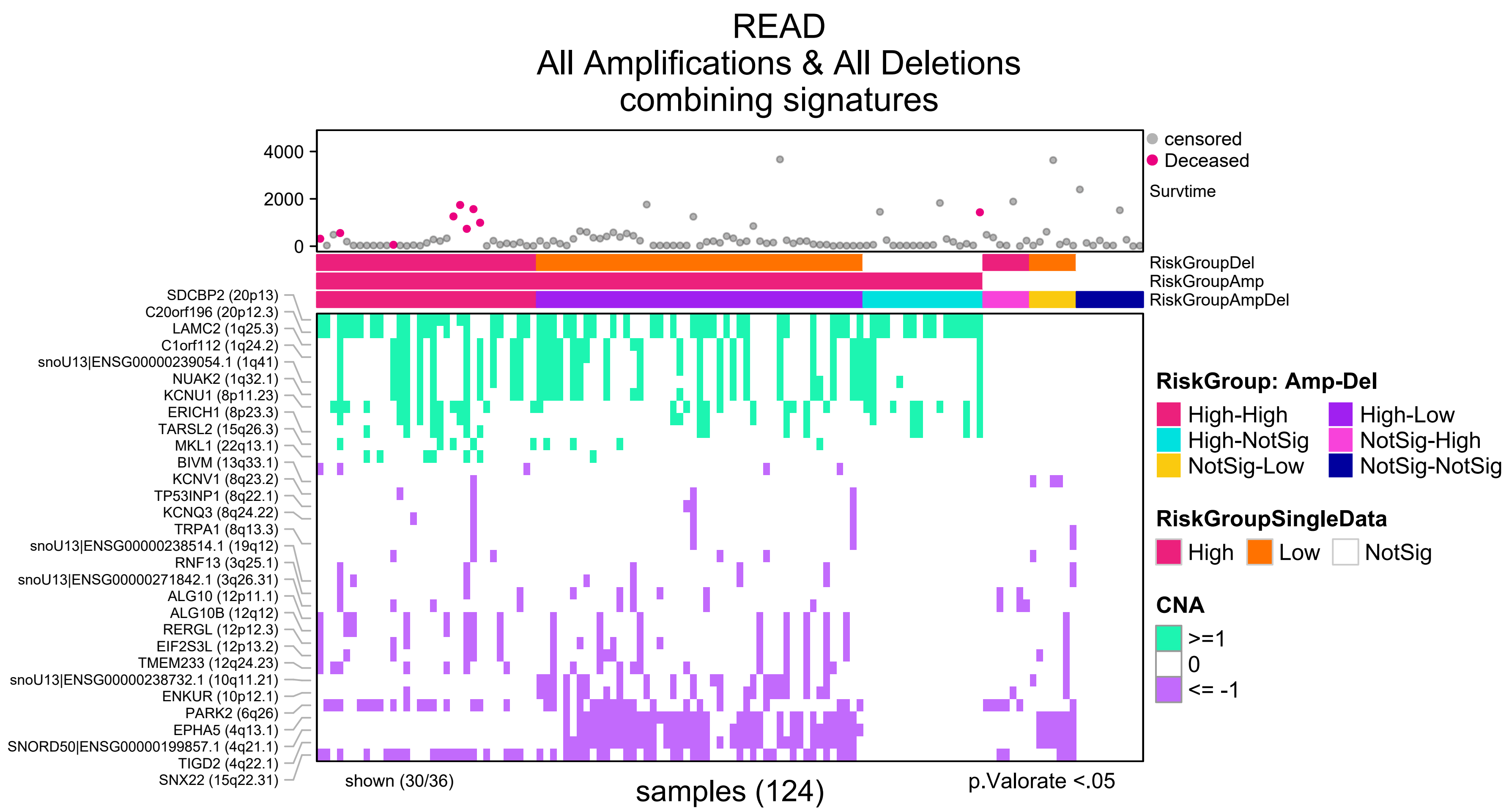

READ
All Amplifications & All Deletions
combining signatures
4000
2000
0
censored
Deceased
Survtime
RiskGroupDel
RiskGroupAmp
RiskGroupAmpDel
SDCBP2 (20p13)
C20orf196 (20p12.3)
LAMC2 (1q25.3)
C1orf112 (1q24.2)
snoU13|ENSG00000239054.1 (1q41)
NUAK2 (1q32.1)
KCNU1 (8p11.23)
ERICH1 (8p23.3)
TARSL2 (15q26.3)
MKL1 (22q13.1)
BIVM (13q33.1)
KCNV1 (8q23.2)
TP53INP1 (8q22.1)
KCNQ3 (8q24.22)
TRPA1 (8q13.3)
snoU13|ENSG00000238514.1 (19q12)
RNF13 (3q25.1)
snoU13|ENSG00000271842.1 (3q26.31)
ALG10 (12p11.1)
ALG10B (12q12)
RERGL (12p12.3)
EIF2S3L (12p13.2)
TMEM233 (12q24.23)
snoU13|ENSG00000238732.1 (10q11.21)
ENKUR (10p12.1)
PARK2 (6q26)
EPHA5 (4q13.1)
SNORD50|ENSG00000199857.1 (4q21.1)
TIGD2 (4q22.1)
SNX22 (15q22.31)
shown (30/36)
samples (124)
p.Valorate <.05
RiskGroup: Amp-Del
High-High
High-NotSig
NotSig-Low
High-Low
NotSig-High
NotSig-NotSig
RiskGroupSingleData
High
Low
NotSig
CNA
>=1
0
<= -1

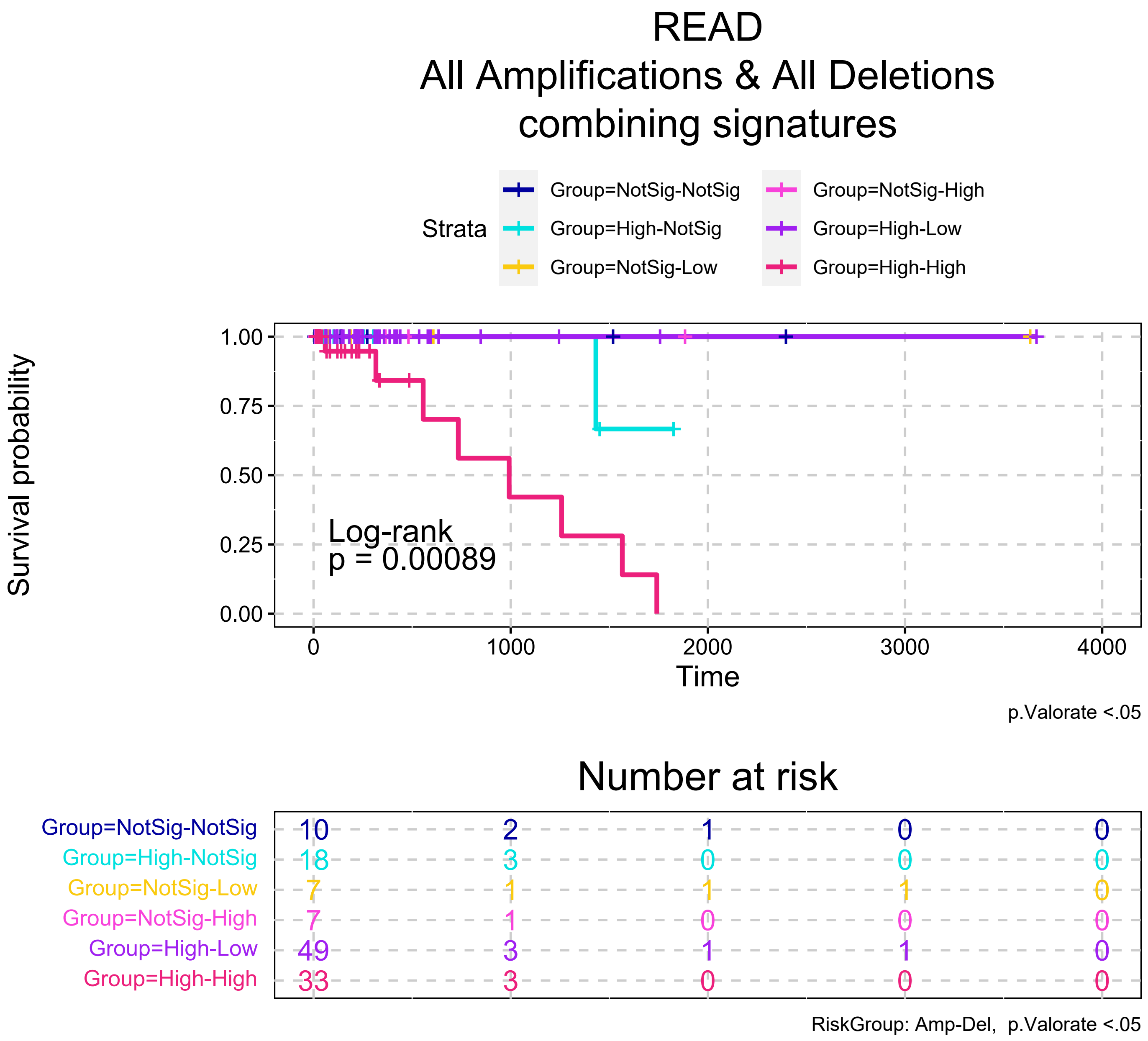

explanatory	beta	HR	L95	U95	p
High-NotSig	20.20	594252762.25	0.00	Inf	1.00
NotSig-Low	-0.03	0.97	0.00	Inf	1.00
NotSig-High	-0.03	0.97	0.00	Inf	1.00
High-Low	0.04	1.04	0.00	Inf	1.00
High-High	21.95	3401600294.23	0.00	Inf	1.00

n= 124, number of events =9
Score(logrank) test = 0.001

READ
Deep Amplifications
Single Data Signature

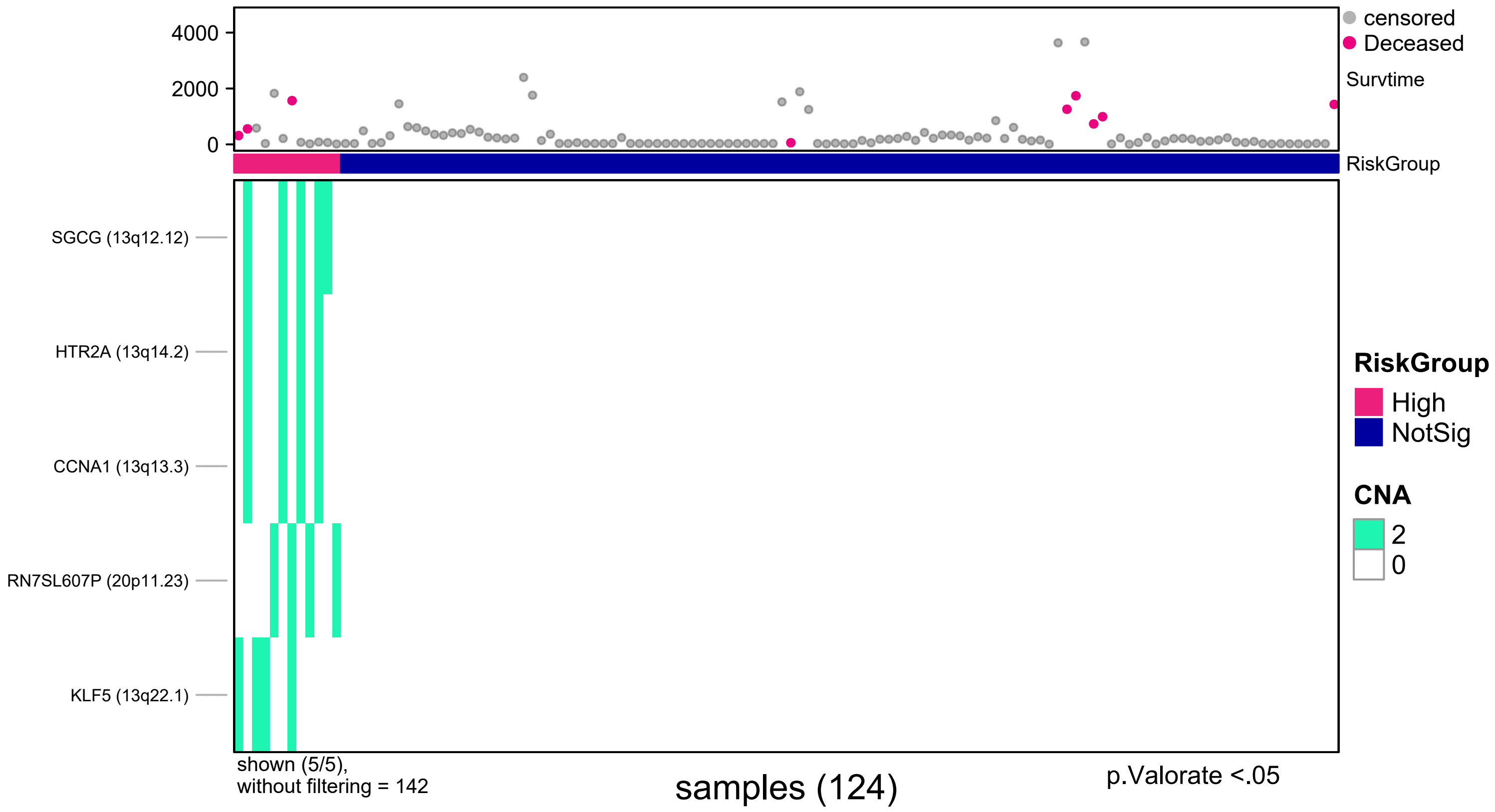

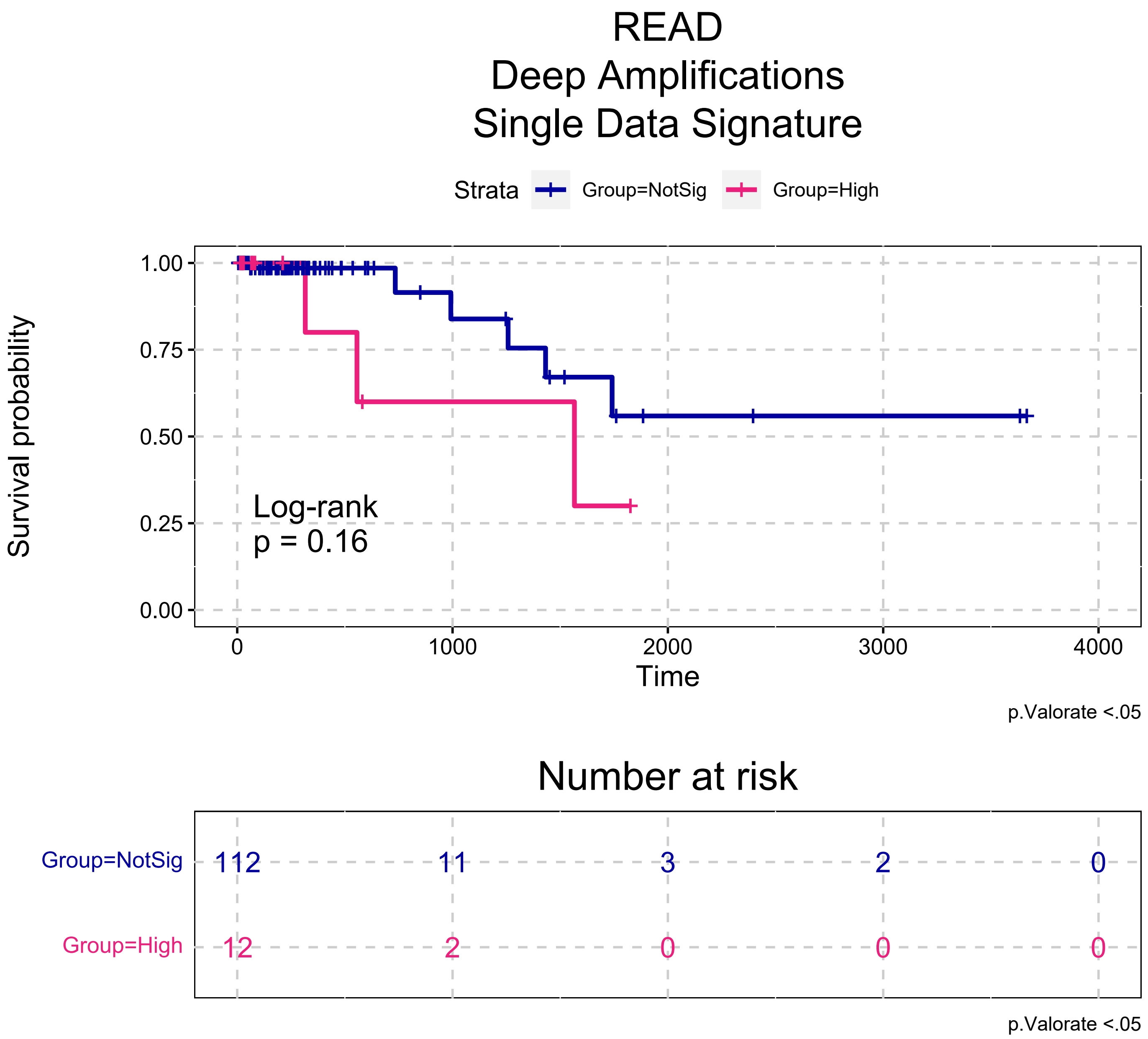

explanatory	beta	HR	L95	U95	p
High	0.96	2.61	0.65	10.55	0.18

n= 124, number of events =9
Score(logrank) test = 0.163
