# Supplementary material for: Systematic Modeling of Risk-Associated Copy Number Alterations in Cancer
Source: Int J Mol Sci. 2024 Sep 27;25(19):10455. doi: 10.3390/ijms251910455 (PMC11477427; doi:10.3390/ijms251910455)
Supplement: Supplementary file 1 [file ijms-25-10455-s001.zip › SARCSignatureV12-sinSombreado.pdf]

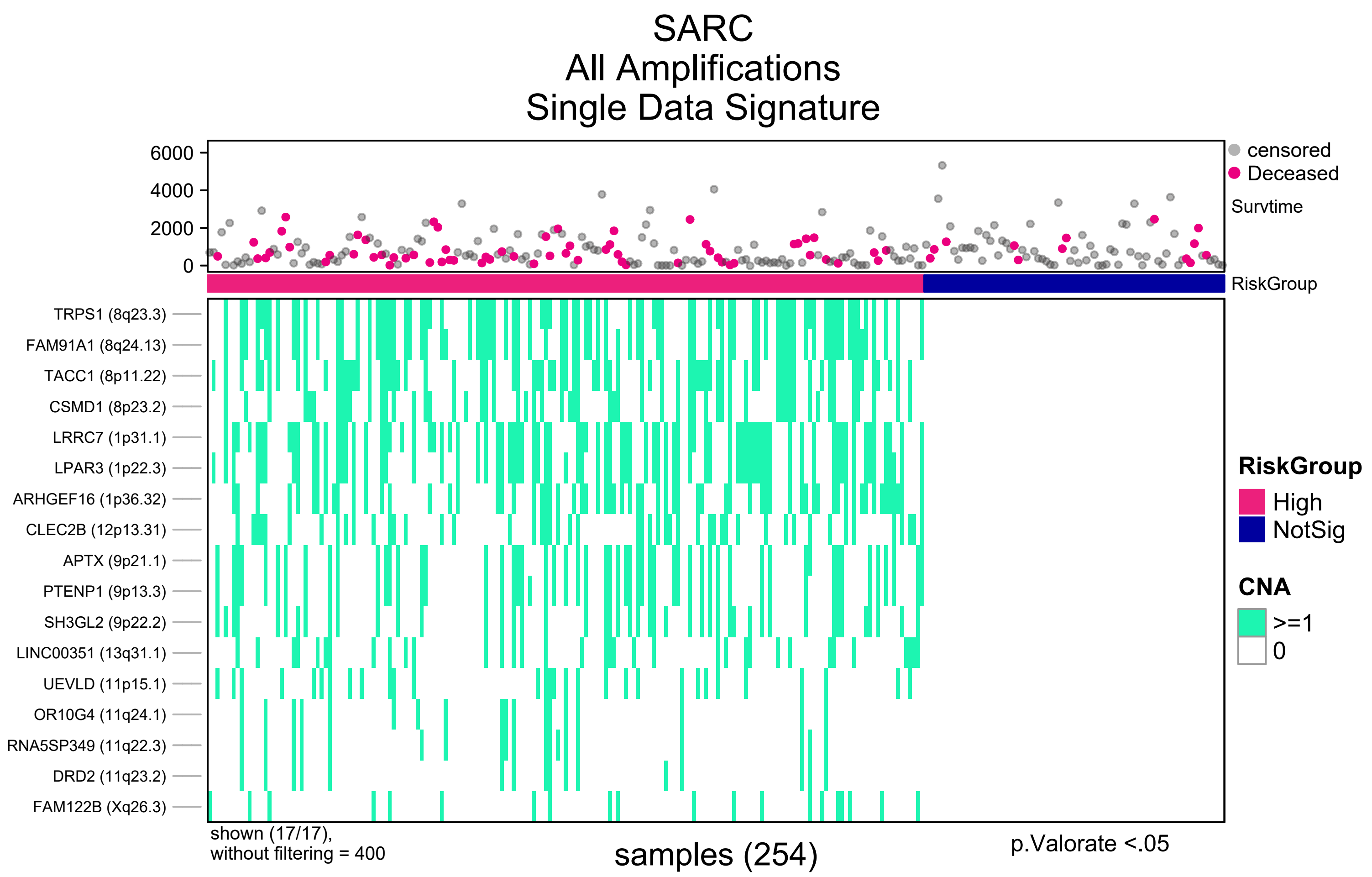
SARC
All Amplifications
Single Data Signature
6000
4000
2000
0
censored
Deceased
Survtime
RiskGroup
TRPS1 (8q23.3)
FAM91A1 (8q24.13)
TACC1 (8p11.22)
CSMD1 (8p23.2)
LRRC7 (1p31.1)
LPAR3 (1p22.3)
ARHGEF16 (1p36.32)
CLEC2B (12p13.31)
APTX (9p21.1)
PTENP1 (9p13.3)
SH3GL2 (9p22.2)
LINC00351 (13q31.1)
UEVLD (11p15.1)
OR10G4 (11q24.1)
RNA5SP349 (11q22.3)
DRD2 (11q23.2)
FAM122B (Xq26.3)
RiskGroup
High
NotSig
CNA
>=1
0
shown (17/17),
without filtering = 400
samples (254)
p.Valorate <.05

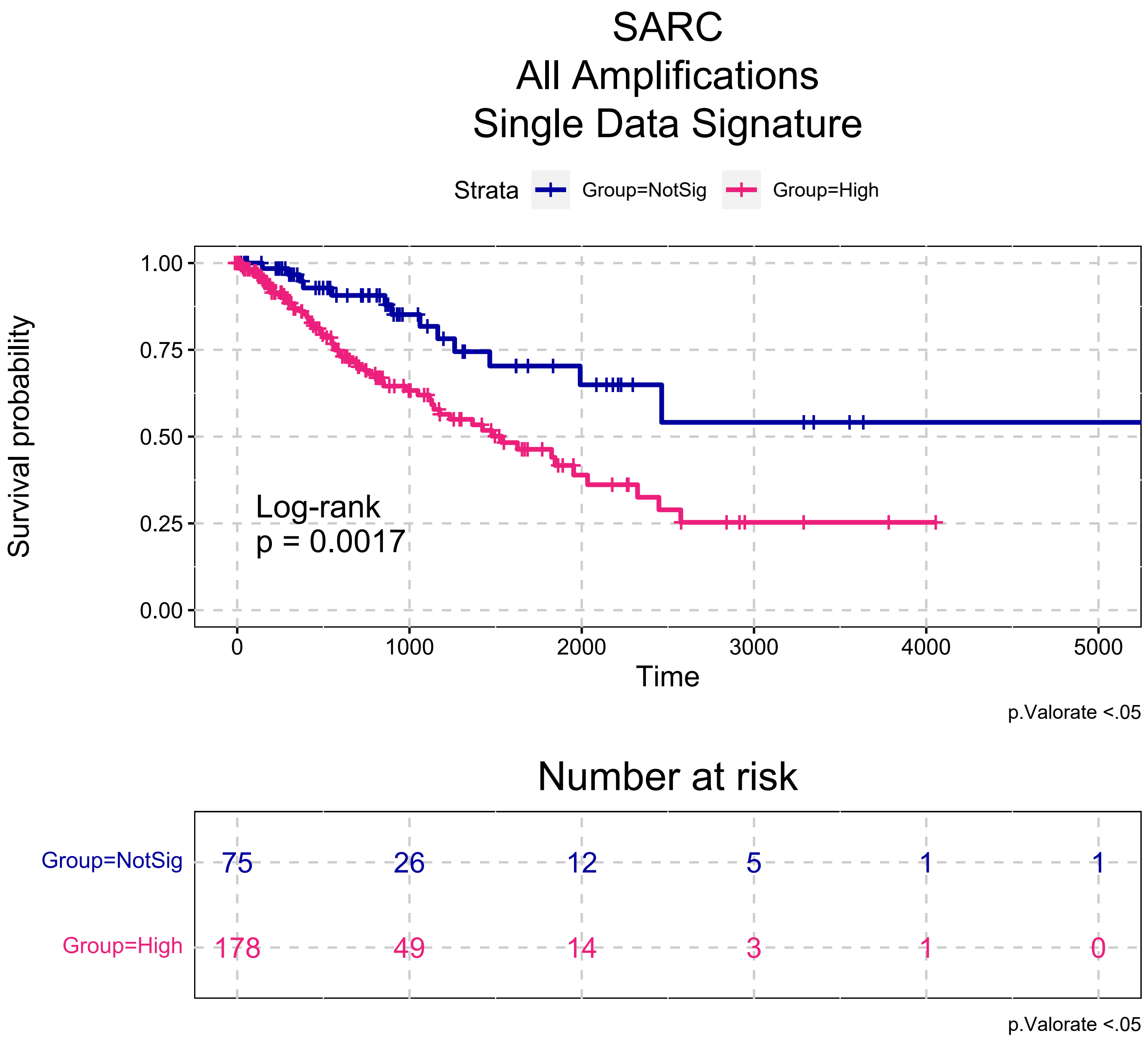

explanatory	beta	HR	L95	U95	p
High	0.92	2.52	1.38	4.59	0.00

n= 254, number of events =75
Score(logrank) test = 0.002

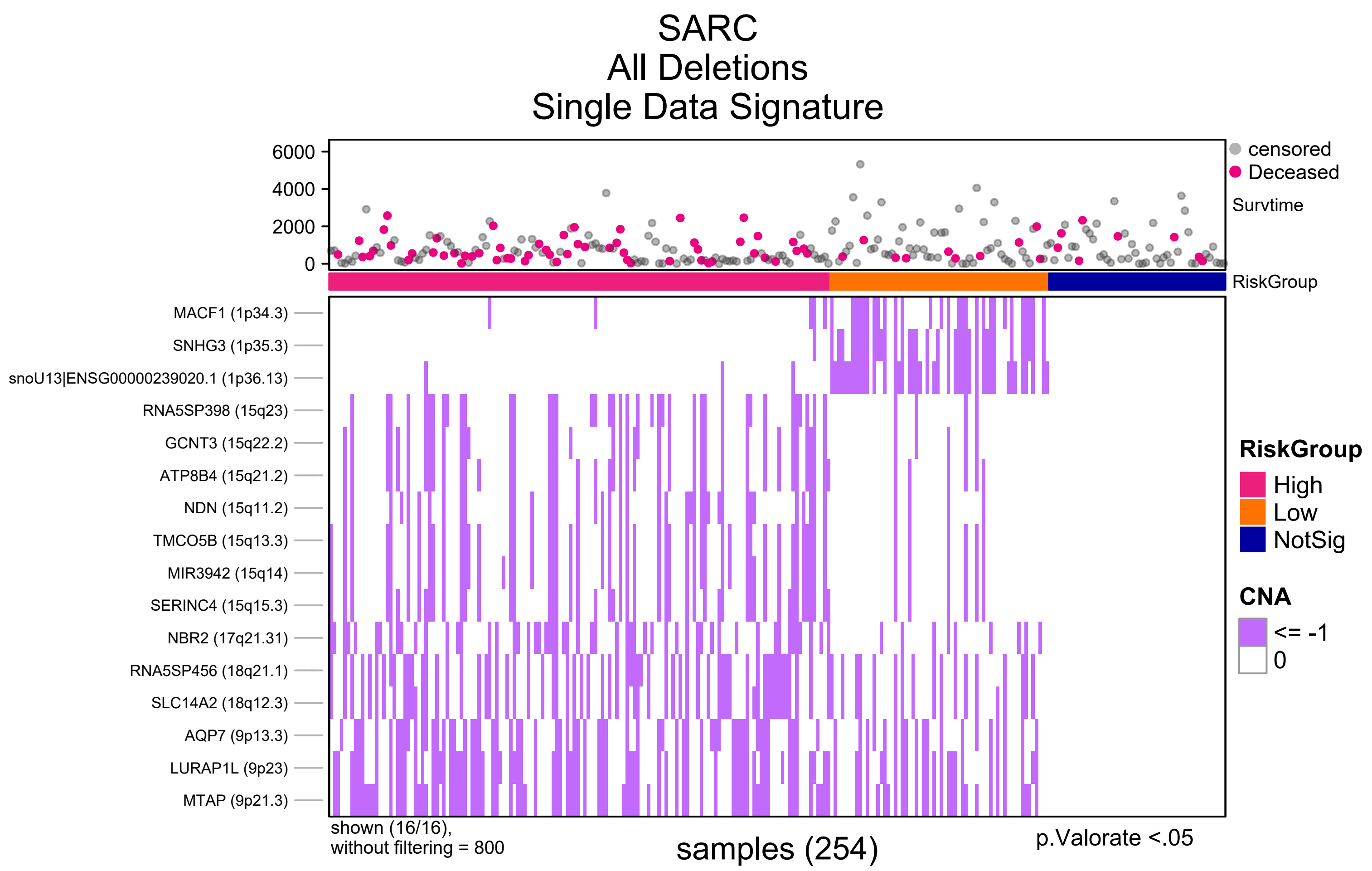

SARC
All Deletions
Single Data Signature
6000
4000
2000
0
censored
Deceased
Survtime
RiskGroup
MACF1 (1p34.3)
SNHG3 (1p35.3)
snoU13|ENSG00000239020.1 (1p36.13)
RNA5SP398 (15q23)
GCNT3 (15q22.2)
ATP8B4 (15q21.2)
NDN (15q11.2)
TMCO5B (15q13.3)
MIR3942 (15q14)
SERINC4 (15q15.3)
NBR2 (17q21.31)
RNA5SP456 (18q21.1)
SLC14A2 (18q12.3)
AQP7 (9p13.3)
LURAP1L (9p23)
MTAP (9p21.3)
RiskGroup
High
Low
NotSig
CNA
<= -1
0
shown (16/16),
without filtering = 800
samples (254)
p.Valorate <.05

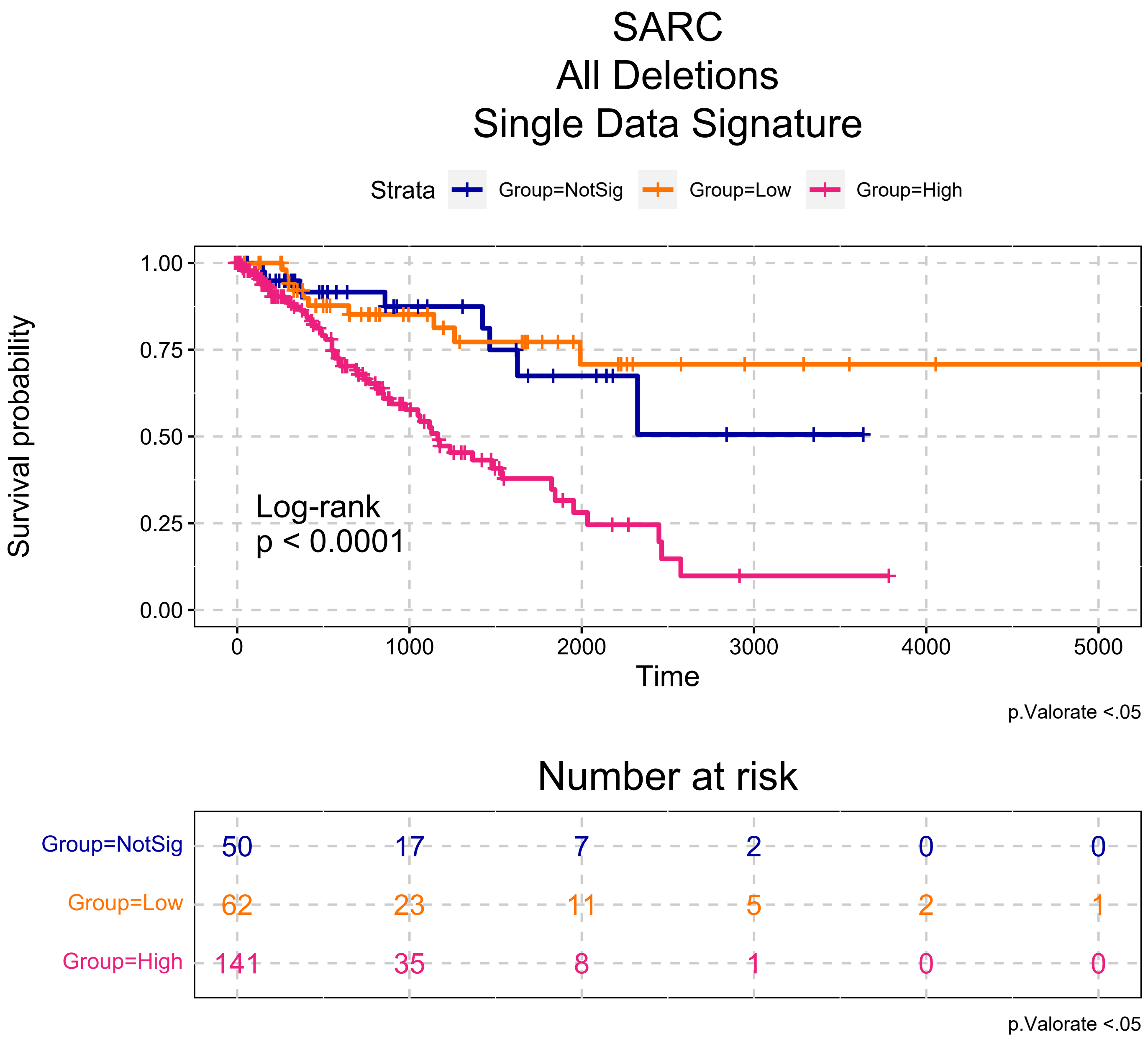

explanatory	beta	HR	L95	U95	p
Low	-0.19	0.83	0.33	2.10	0.69
High	1.16	3.19	1.52	6.72	0.00

n= 254, number of events =75
Score(logrank) test = p <.0001

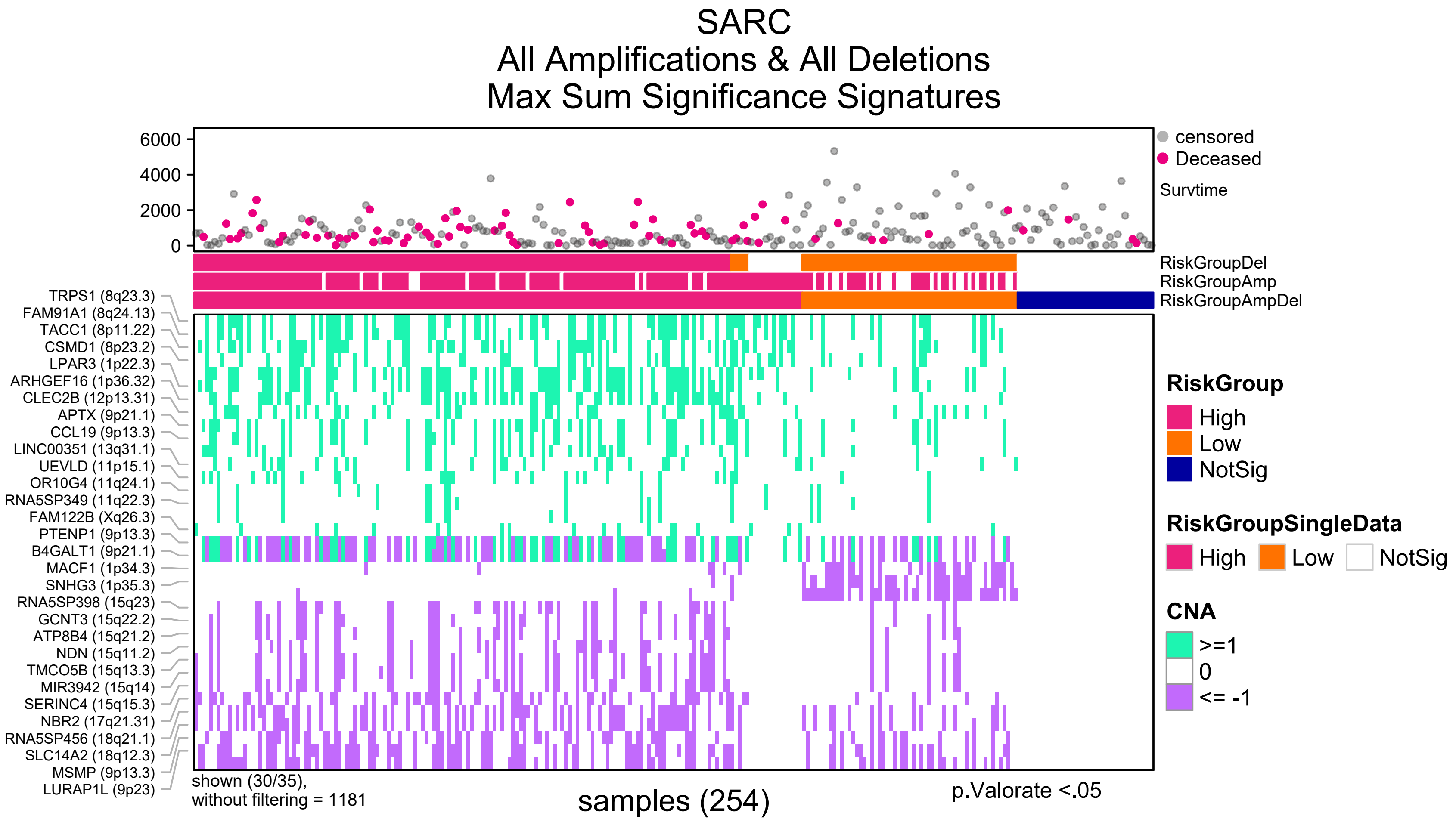

SARC
All Amplifications & All Deletions
Max Sum Significance Signatures
6000
4000
2000
0
censored
Deceased
Survtime
RiskGroupDel
RiskGroupAmp
RiskGroupAmpDel
TRPS1 (8q23.3)
FAM91A1 (8q24.13)
TACC1 (8p11.22)
CSMD1 (8p23.2)
LPAR3 (1p22.3)
ARHGEF16 (1p36.32)
CLEC2B (12p13.31)
APTX (9p21.1)
CCL19 (9p13.3)
LINC00351 (13q31.1)
UEVLD (11p15.1)
OR10G4 (11q24.1)
RNA5SP349 (11q22.3)
FAM122B (Xq26.3)
PTENP1 (9p13.3)
B4GALT1 (9p21.1)
MACF1 (1p34.3)
SNHG3 (1p35.3)
RNA5SP398 (15q23)
GCNT3 (15q22.2)
ATP8B4 (15q21.2)
NDN (15q11.2)
TMCO5B (15q13.3)
MIR3942 (15q14)
SERINC4 (15q15.3)
NBR2 (17q21.31)
RNA5SP456 (18q21.1)
SLC14A2 (18q12.3)
MSMP (9p13.3)
LURAP1L (9p23)
RiskGroup
High
Low
NotSig
RiskGroupSingleData
High
Low
NotSig
CNA
>=1
0
<= -1
shown (30/35),
without filtering = 1181
samples (254)
p.Valorate <.05

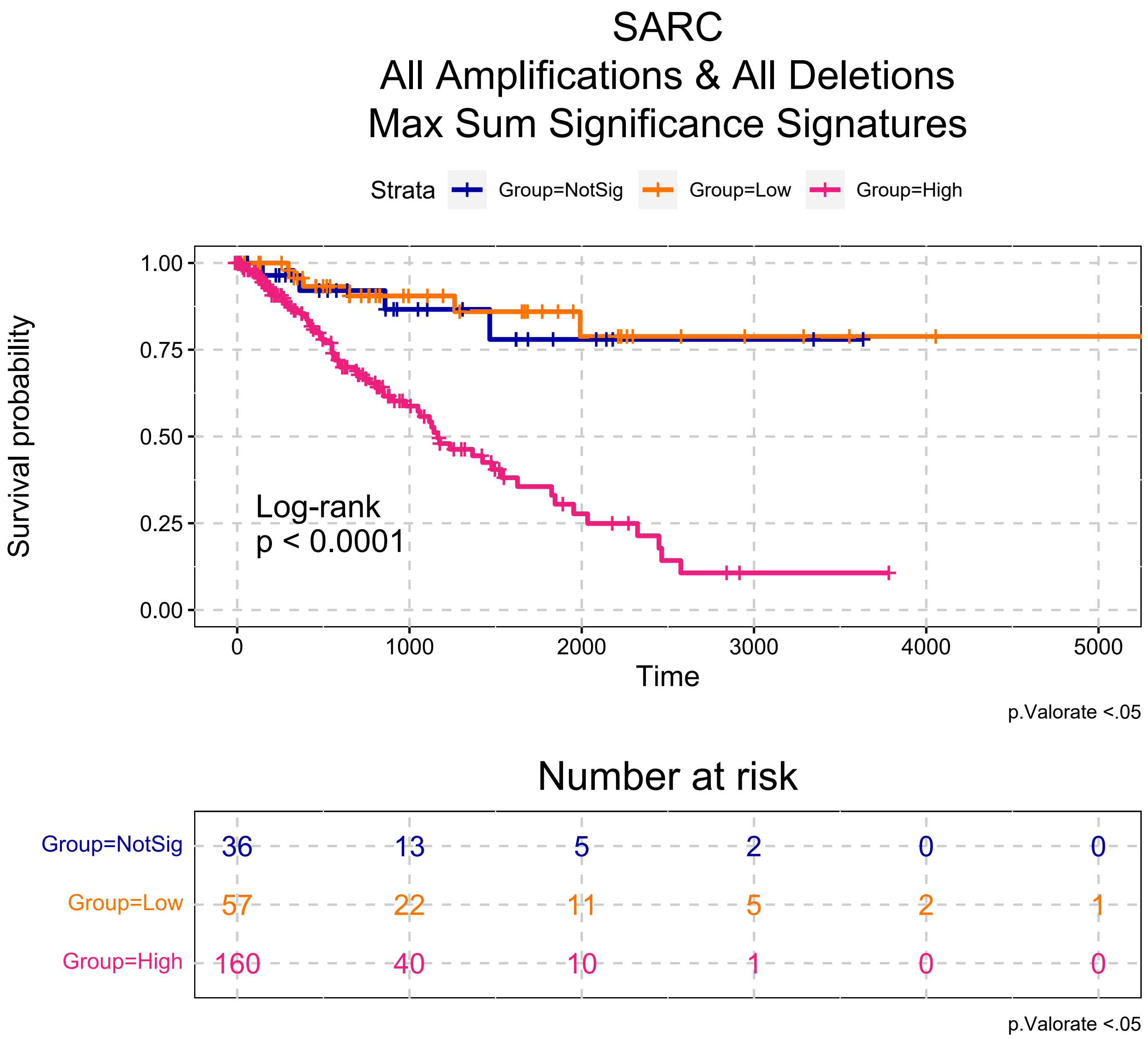

explanatory	beta	HR	L95	U95	p
Low	-0.28	0.76	0.21	2.69	0.67
High	1.56	4.75	1.73	13.07	0.00

n= 254, number of events =75
Score(logrank) test = p <.0001

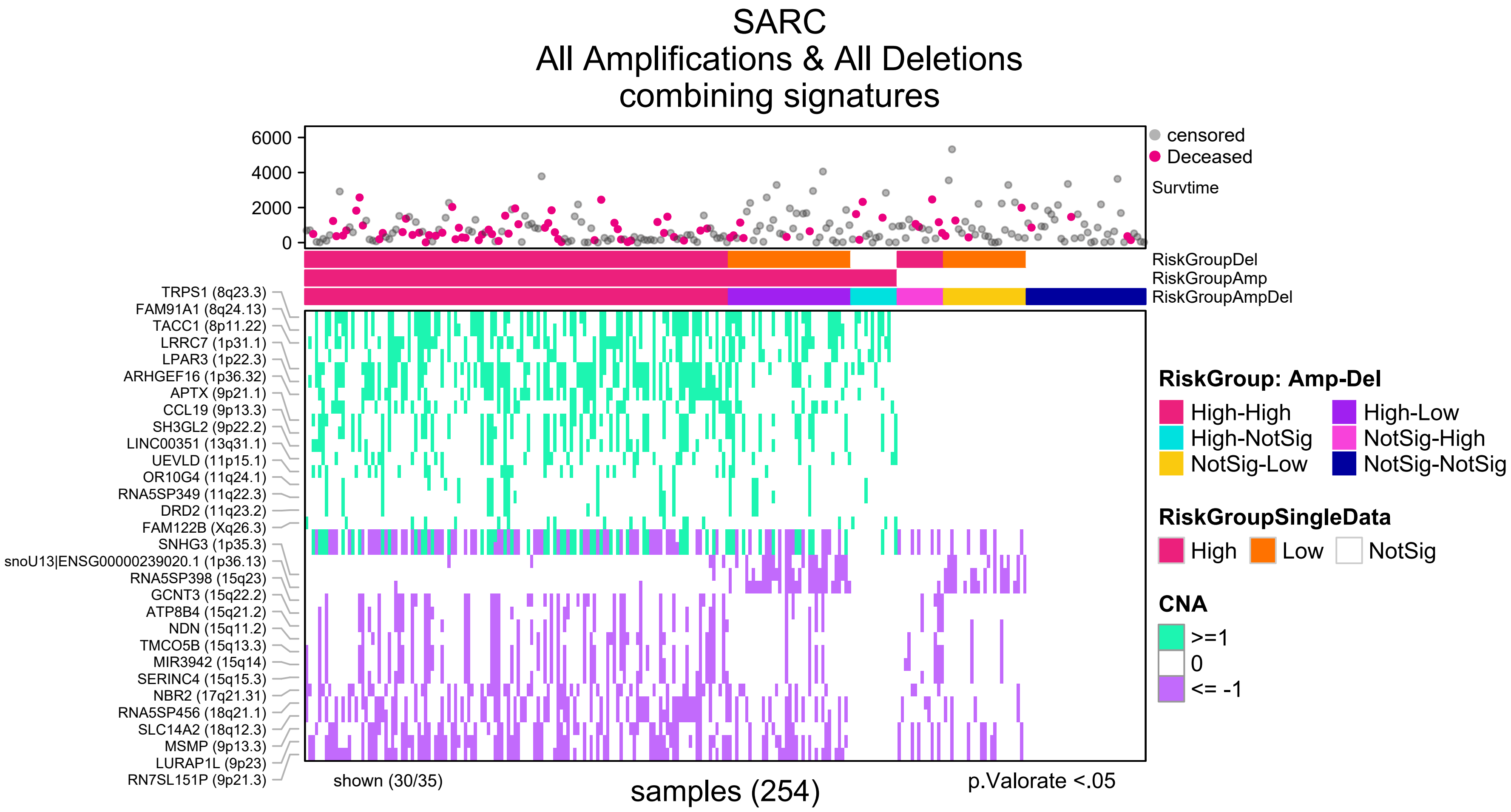

SARC
All Amplifications & All Deletions
combining signatures
6000
4000
2000
0
censored
Deceased
Survtime
RiskGroupDel
RiskGroupAmp
RiskGroupAmpDel
TRPS1 (8q23.3)
FAM91A1 (8q24.13)
TACC1 (8p11.22)
LRRC7 (1p31.1)
LPAR3 (1p22.3)
ARHGEF16 (1p36.32)
APTX (9p21.1)
CCL19 (9p13.3)
SH3GL2 (9p22.2)
LINC00351 (13q31.1)
UEVLD (11p15.1)
OR10G4 (11q24.1)
RNA5SP349 (11q22.3)
DRD2 (11q23.2)
FAM122B (Xq26.3)
SNHG3 (1p35.3)
snoU13|ENSG00000239020.1 (1p36.13)
RNA5SP398 (15q23)
GCNT3 (15q22.2)
ATP8B4 (15q21.2)
NDN (15q11.2)
TMCO5B (15q13.3)
MIR3942 (15q14)
SERINC4 (15q15.3)
NBR2 (17q21.31)
RNA5SP456 (18q21.1)
SLC14A2 (18q12.3)
MSMP (9p13.3)
LURAP1L (9p23)
RN7SL151P (9p21.3)
RiskGroup: Amp-Del
High-High
High-Low
High-NotSig
NotSig-High
NotSig-Low
NotSig-NotSig
RiskGroupSingleData
High
Low
NotSig
CNA
>=1
0
<= -1
shown (30/35)
samples (254)
p.Valorate <.05

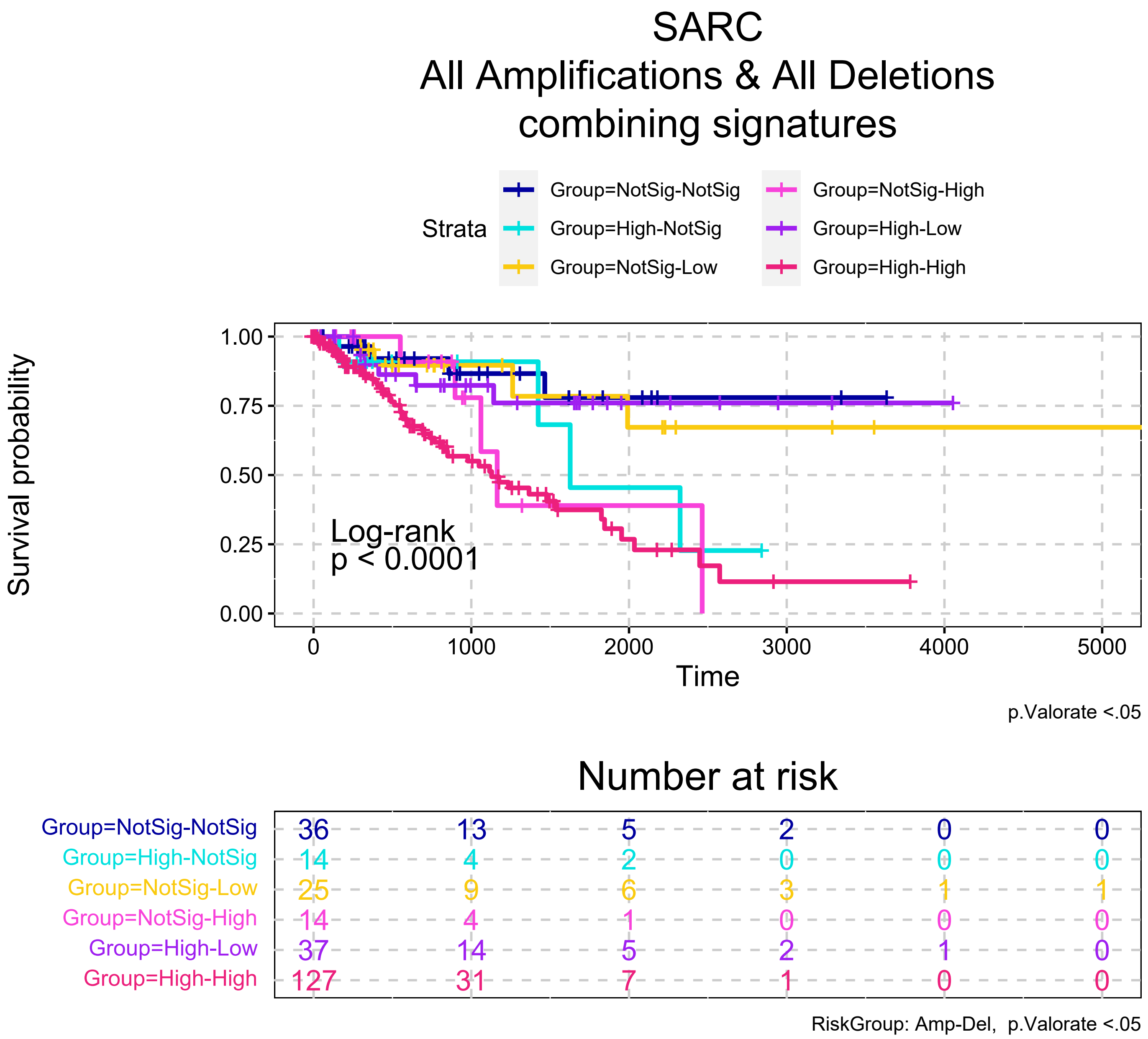

explanatory	beta	HR	L95	U95	p
High-NotSig	1.05	2.85	0.71	11.43	0.14
NotSig-Low	0.17	1.18	0.30	4.74	0.81
NotSig-High	1.17	3.23	0.86	12.08	0.08
High-Low	0.23	1.26	0.35	4.47	0.72
High-High	1.60	4.94	1.78	13.68	0.00

n= 254, number of events =75
Score(logrank) test = p <.0001

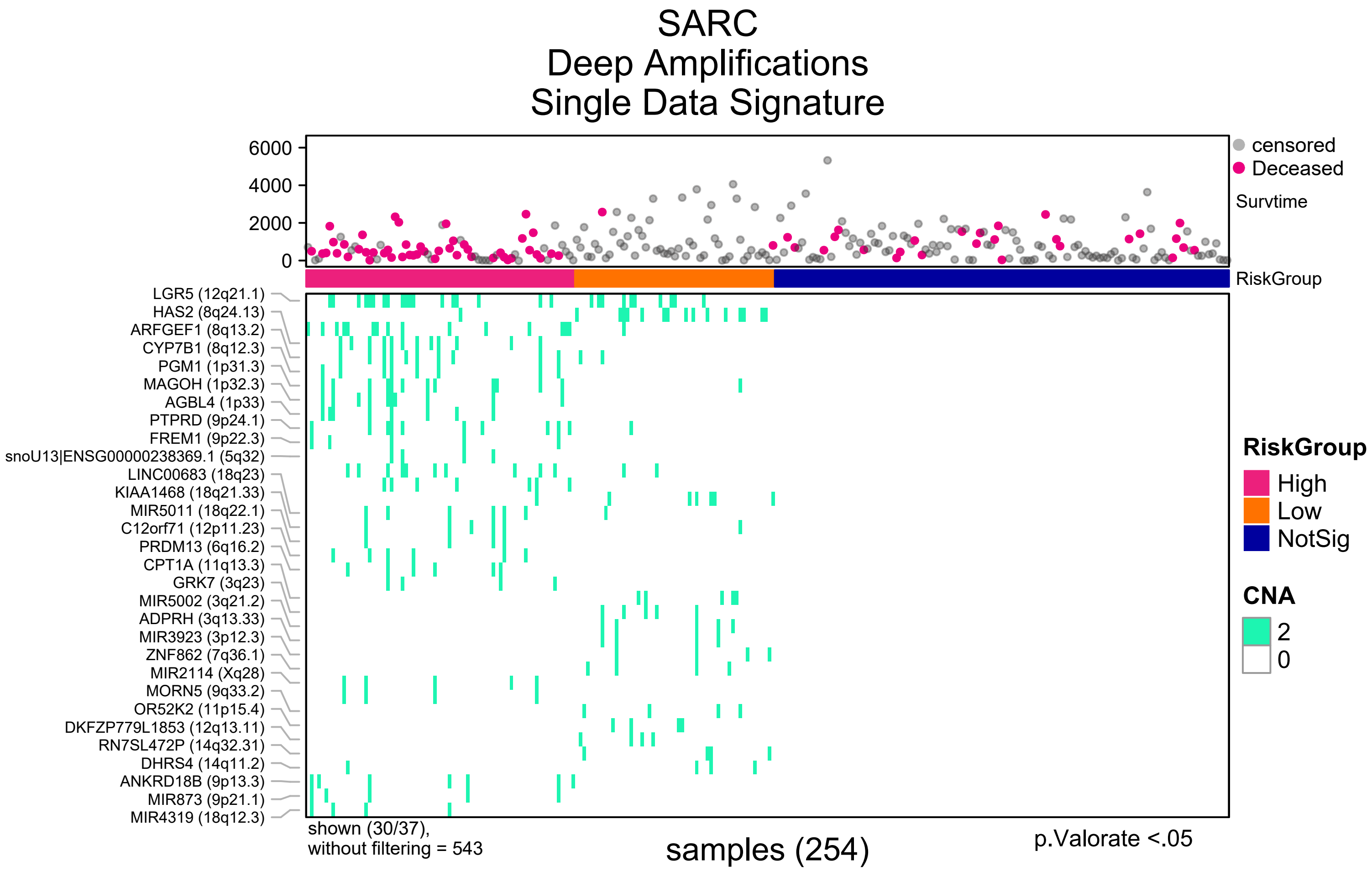

SARC
Deep Amplifications
Single Data Signature
6000
4000
2000
0
censored
Deceased
Survtime
RiskGroup
LGR5 (12q21.1)
HAS2 (8q24.13)
ARFGEF1 (8q13.2)
CYP7B1 (8q12.3)
PGM1 (1p31.3)
MAGOH (1p32.3)
AGBL4 (1p33)
PTPRD (9p24.1)
FREM1 (9p22.3)
snoU13|ENSG00000238369.1 (5q32)
LINC00683 (18q23)
KIAA1468 (18q21.33)
MIR5011 (18q22.1)
C12orf71 (12p11.23)
PRDM13 (6q16.2)
CPT1A (11q13.3)
GRK7 (3q23)
MIR5002 (3q21.2)
ADPRH (3q13.33)
MIR3923 (3p12.3)
ZNF862 (7q36.1)
MIR2114 (Xq28)
MORN5 (9q33.2)
OR52K2 (11p15.4)
DKFZP779L1853 (12q13.11)
RN7SL472P (14q32.31)
DHRS4 (14q11.2)
ANKRD18B (9p13.3)
MIR873 (9p21.1)
MIR4319 (18q12.3)
RiskGroup
High
Low
NotSig
CNA
2
0
shown (30/37),
without filtering = 543
samples (254)
p.Valorate <.05

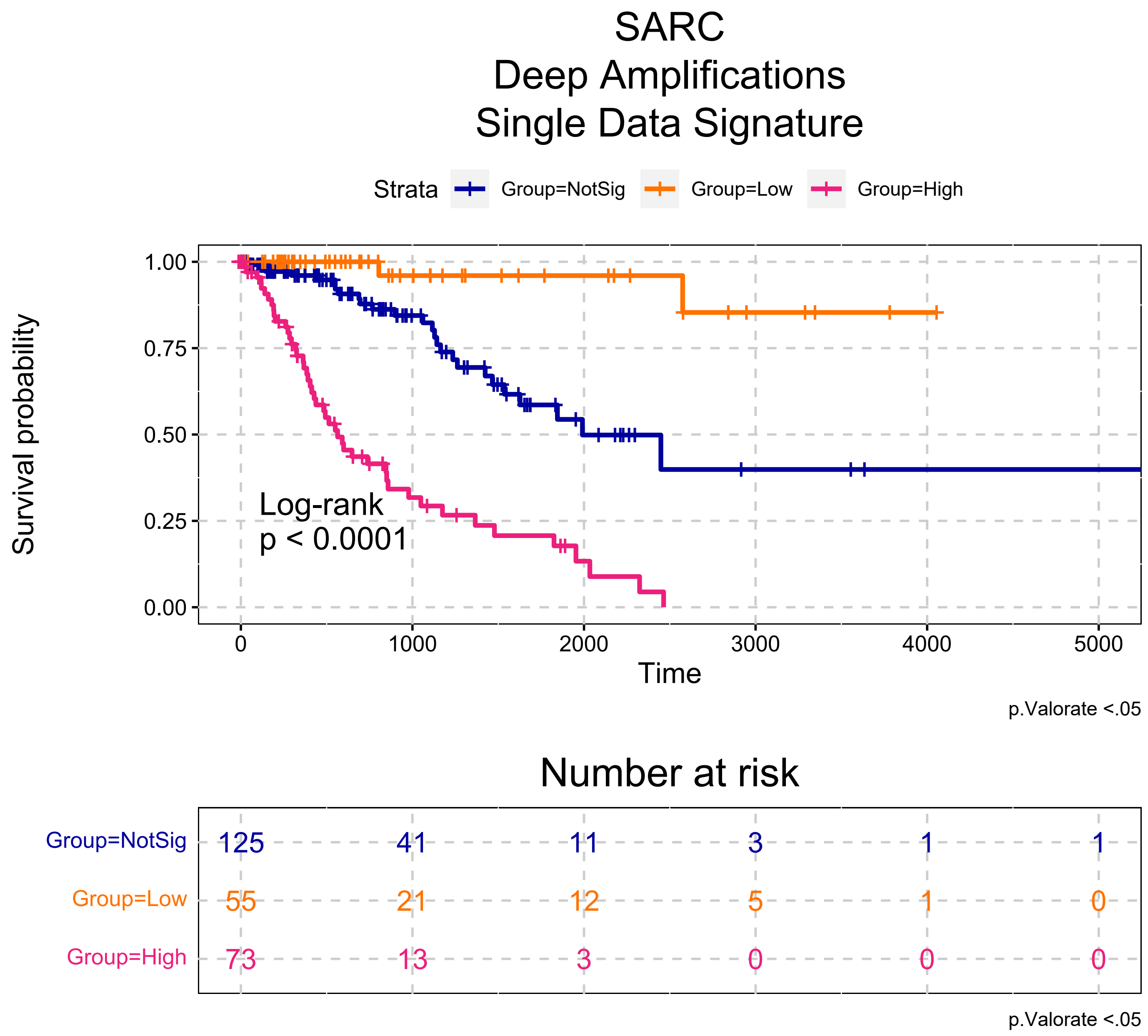

explanatory	beta	HR	L95	U95	p
Low	-2.13	0.12	0.03	0.51	0.00
High	1.53	4.64	2.85	7.54	0.00

n= 254, number of events =75
Score(logrank) test = p <.0001

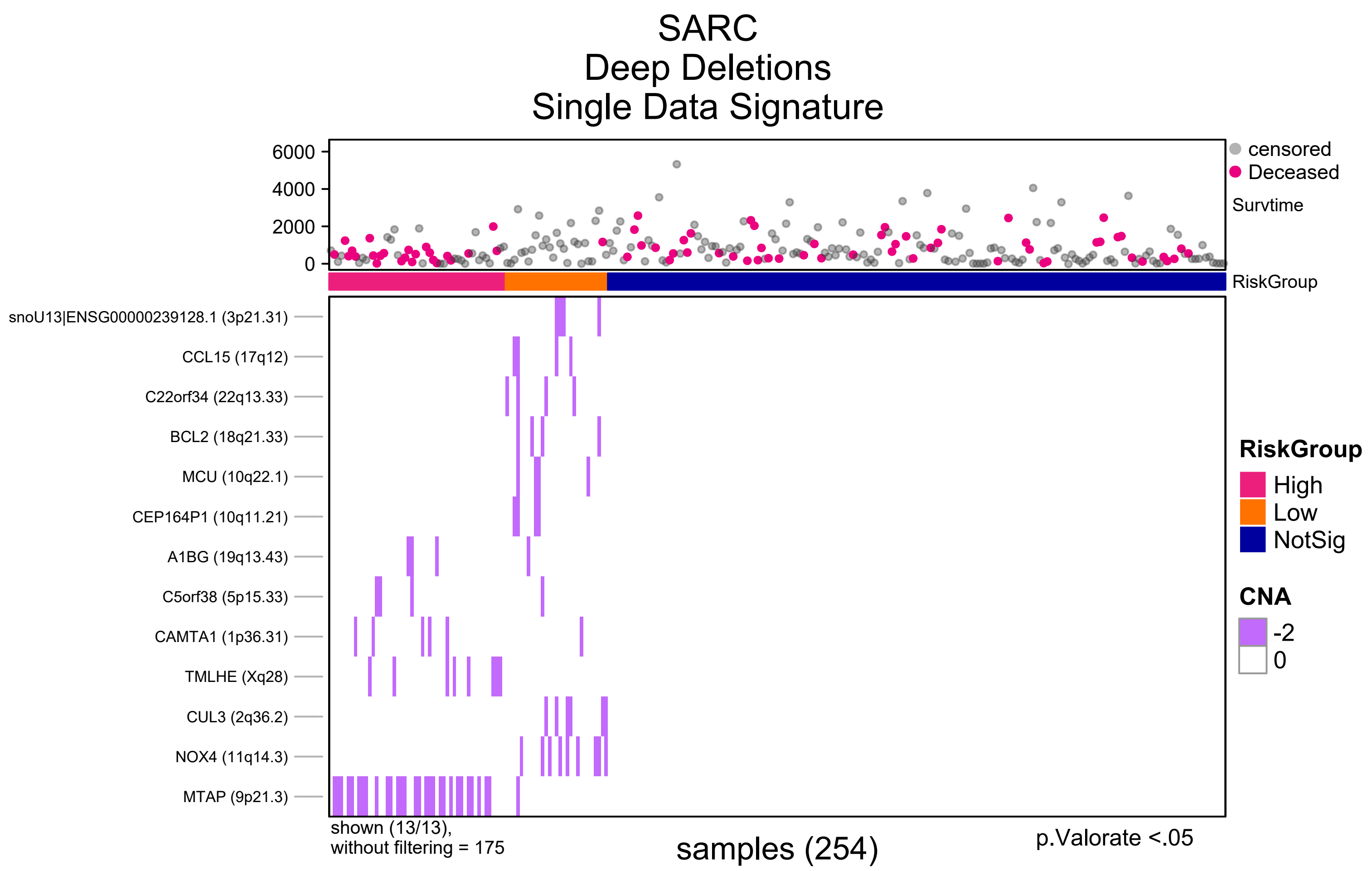
SARC
Deep Deletions
Single Data Signature
6000
4000
2000
0
censored
Deceased
Survtime
RiskGroup
snoU13|ENSG00000239128.1 (3p21.31)
CCL15 (17q12)
C22orf34 (22q13.33)
BCL2 (18q21.33)
MCU (10q22.1)
CEP164P1 (10q11.21)
A1BG (19q13.43)
C5orf38 (5p15.33)
CAMTA1 (1p36.31)
TMLHE (Xq28)
CUL3 (2q36.2)
NOX4 (11q14.3)
MTAP (9p21.3)
RiskGroup
High
Low
NotSig
CNA
-2
0
shown (13/13),
without filtering = 175
samples (254)
p.Valorate <.05

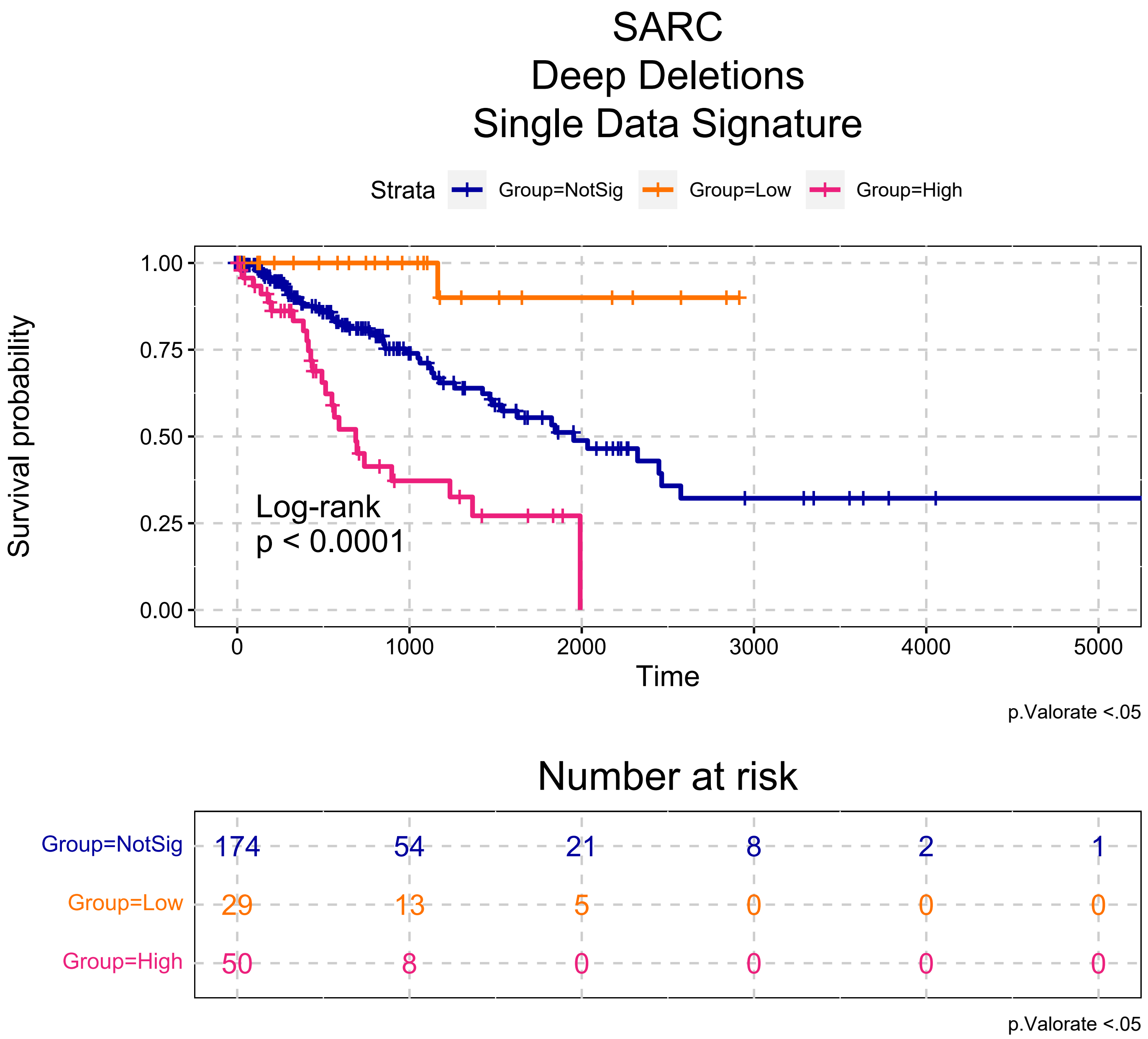

explanatory	beta	HR	L95	U95	p
Low	-2.30	0.10	0.01	0.72	0.02
High	1.00	2.73	1.65	4.51	0.00

n= 254, number of events =75
Score(logrank) test = p <.0001

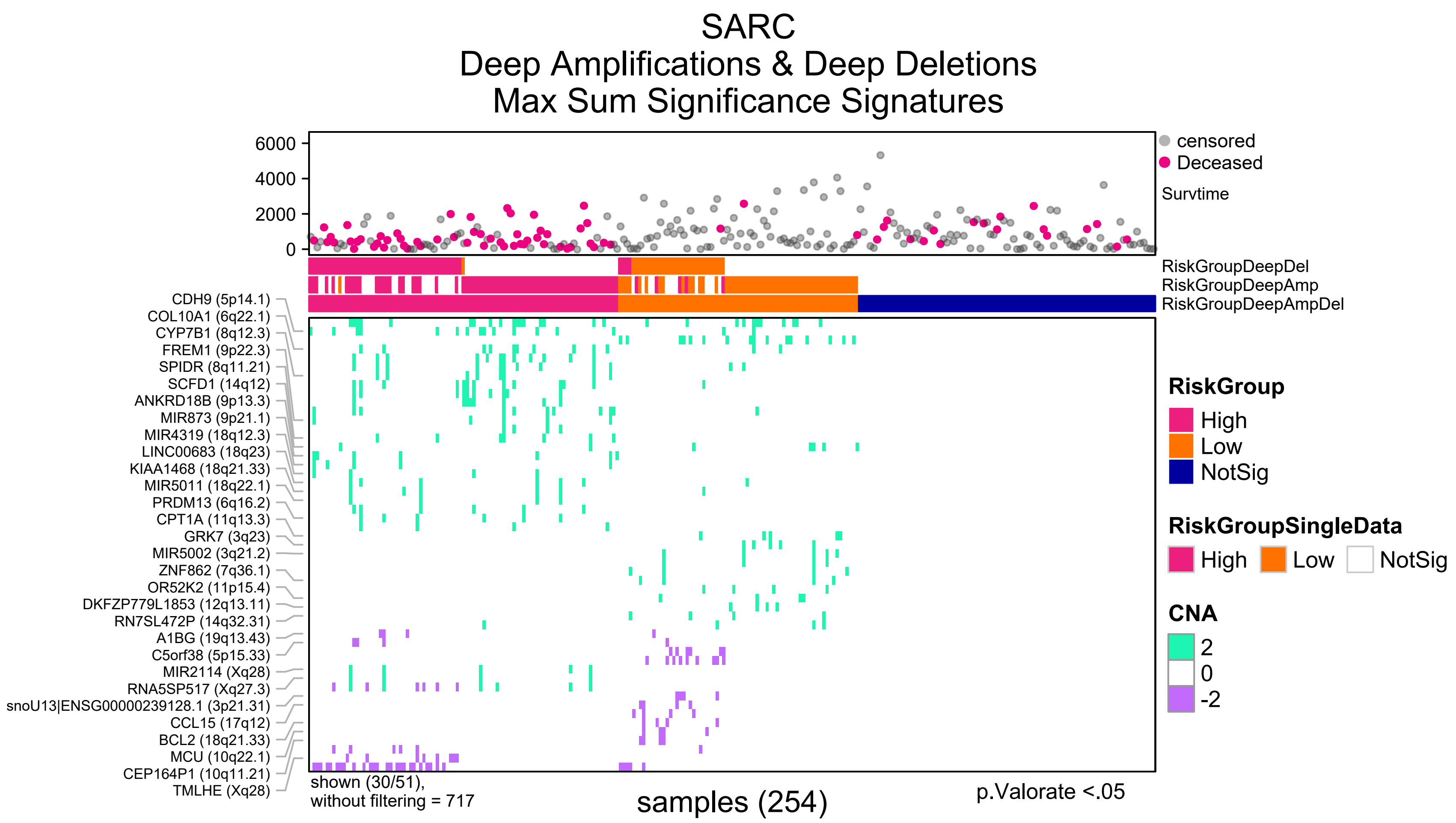

SARC
Deep Amplifications & Deep Deletions
Max Sum Significance Signatures
6000
4000
2000
0
censored
Deceased
Survtime
RiskGroupDeepDel
RiskGroupDeepAmp
RiskGroupDeepAmpDel
CDH9 (5p14.1)
COL10A1 (6q22.1)
CYP7B1 (8q12.3)
FREM1 (9p22.3)
SPIDR (8q11.21)
SCFD1 (14q12)
ANKRD18B (9p13.3)
MIR873 (9p21.1)
MIR4319 (18q12.3)
LINC00683 (18q23)
KIAA1468 (18q21.33)
MIR5011 (18q22.1)
PRDM13 (6q16.2)
CPT1A (11q13.3)
GRK7 (3q23)
MIR5002 (3q21.2)
ZNF862 (7q36.1)
OR52K2 (11p15.4)
DKFZP779L1853 (12q13.11)
RN7SL472P (14q32.31)
A1BG (19q13.43)
C5orf38 (5p15.33)
MIR2114 (Xq28)
RNA5SP517 (Xq27.3)
snoU13|ENSG00000239128.1 (3p21.31)
CCL15 (17q12)
BCL2 (18q21.33)
MCU (10q22.1)
CEP164P1 (10q11.21)
TMLHE (Xq28)
RiskGroup
High
Low
NotSig
RiskGroupSingleData
High
Low
NotSig
CNA
2
0
-2
shown (30/51),
without filtering = 717
samples (254)
p.Valorate <.05

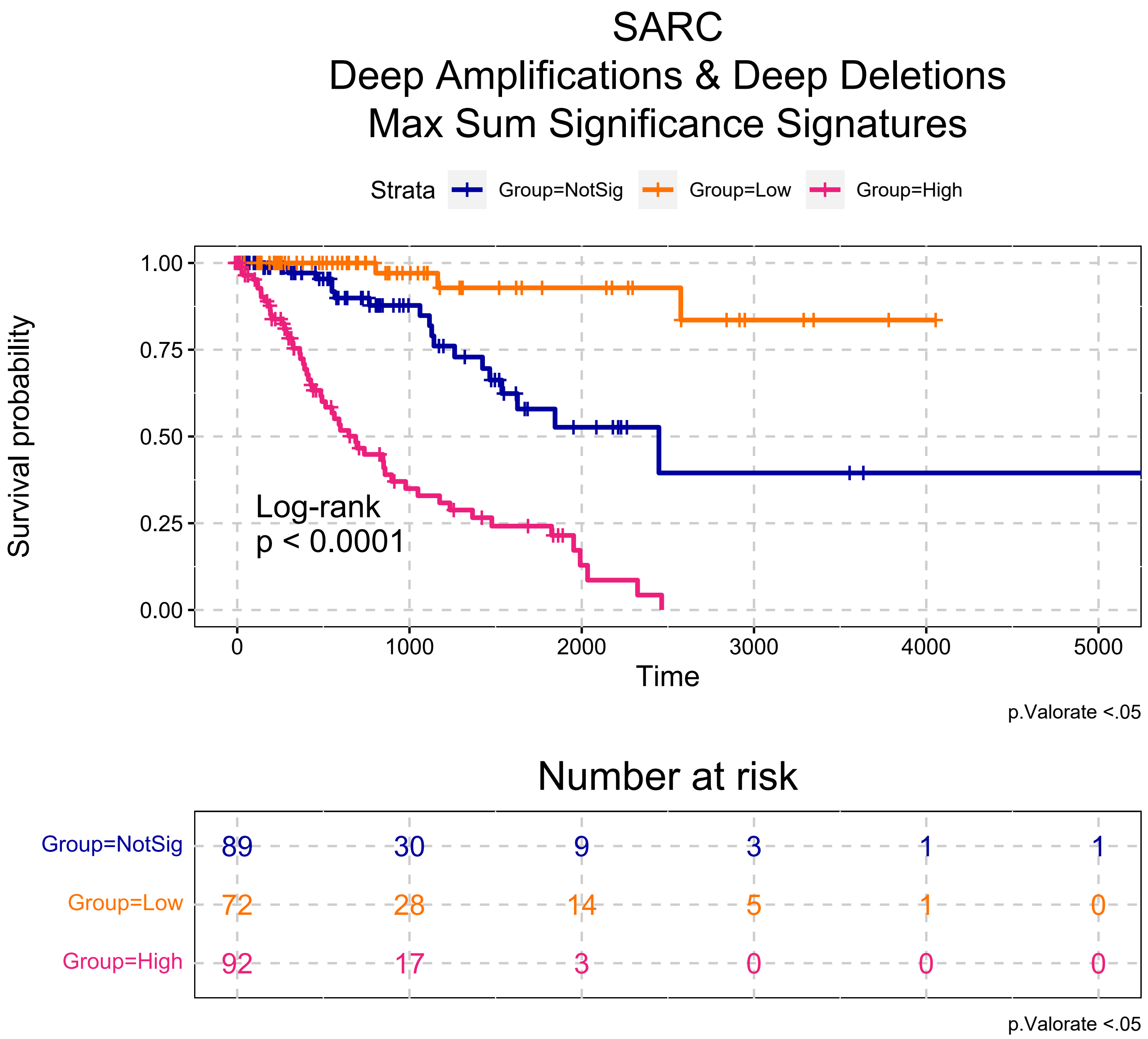

explanatory	beta	HR	L95	U95	p
Low	-1.89	0.15	0.04	0.52	0.00
High	1.51	4.51	2.62	7.77	0.00

n= 254, number of events =75
Score(logrank) test = p <.0001

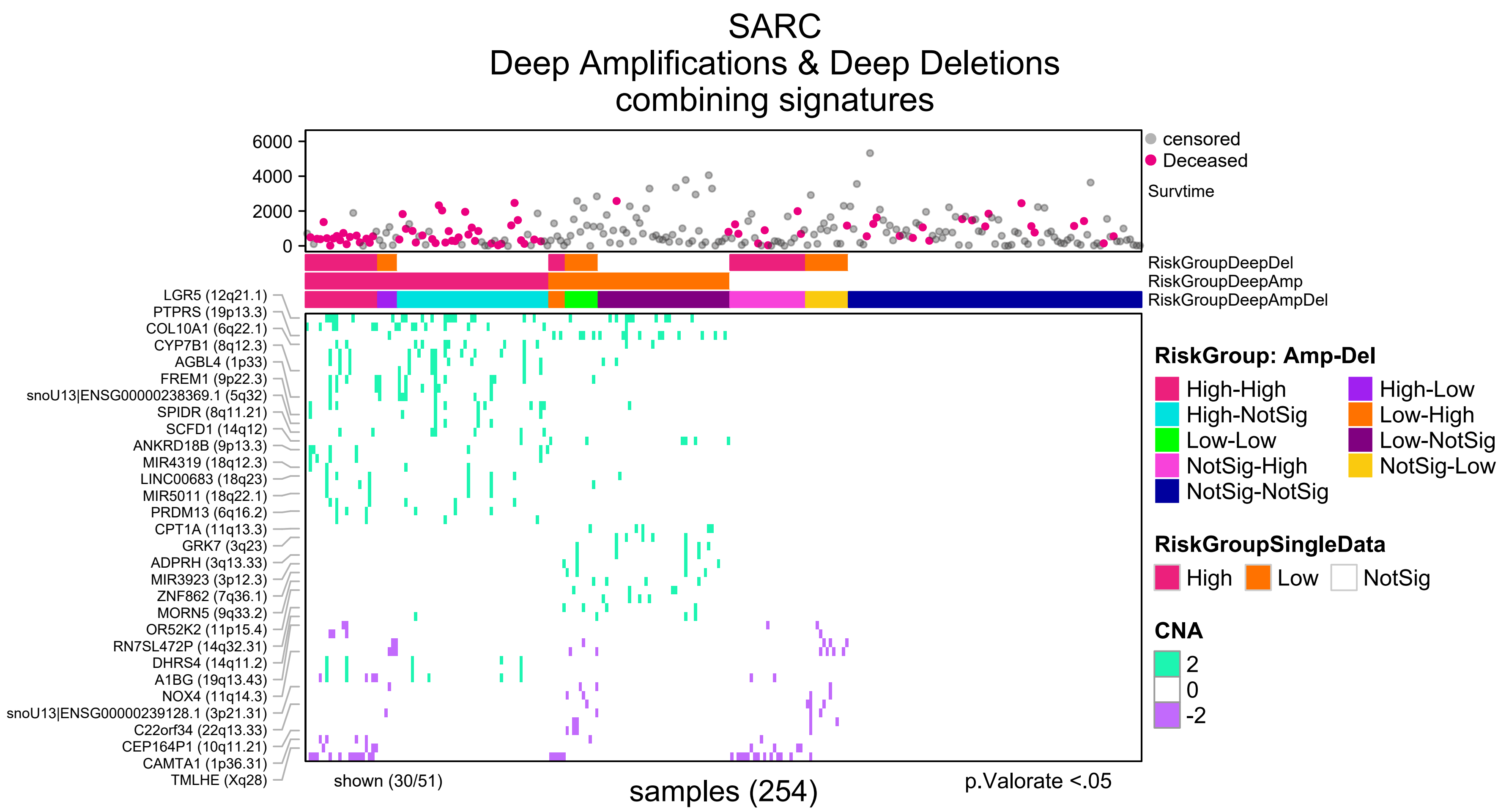

SARC
Deep Amplifications & Deep Deletions
combining signatures
6000
4000
2000
0
censored
Deceased
Survtime
RiskGroupDeepDel
RiskGroupDeepAmp
RiskGroupDeepAmpDel
LGR5 (12q21.1)
PTPRS (19p13.3)
COL10A1 (6q22.1)
CYP7B1 (8q12.3)
AGBL4 (1p33)
FREM1 (9p22.3)
snoU13|ENSG00000238369.1 (5q32)
SPIDR (8q11.21)
SCFD1 (14q12)
ANKRD18B (9p13.3)
MIR4319 (18q12.3)
LINC00683 (18q23)
MIR5011 (18q22.1)
PRDM13 (6q16.2)
CPT1A (11q13.3)
GRK7 (3q23)
ADPRH (3q13.33)
MIR3923 (3p12.3)
ZNF862 (7q36.1)
MORN5 (9q33.2)
OR52K2 (11p15.4)
RN7SL472P (14q32.31)
DHRS4 (14q11.2)
A1BG (19q13.43)
NOX4 (11q14.3)
snoU13|ENSG00000239128.1 (3p21.31)
C22orf34 (22q13.33)
CEP164P1 (10q11.21)
CAMTA1 (1p36.31)
TMLHE (Xq28)
shown (30/51)
samples (254)
p.Valorate <.05
RiskGroup: Amp-Del
High-High
High-NotSig
Low-Low
NotSig-High
NotSig-NotSig
High-Low
Low-High
Low-NotSig
NotSig-Low
RiskGroupSingleData
High
Low
NotSig
CNA
2
0
-2

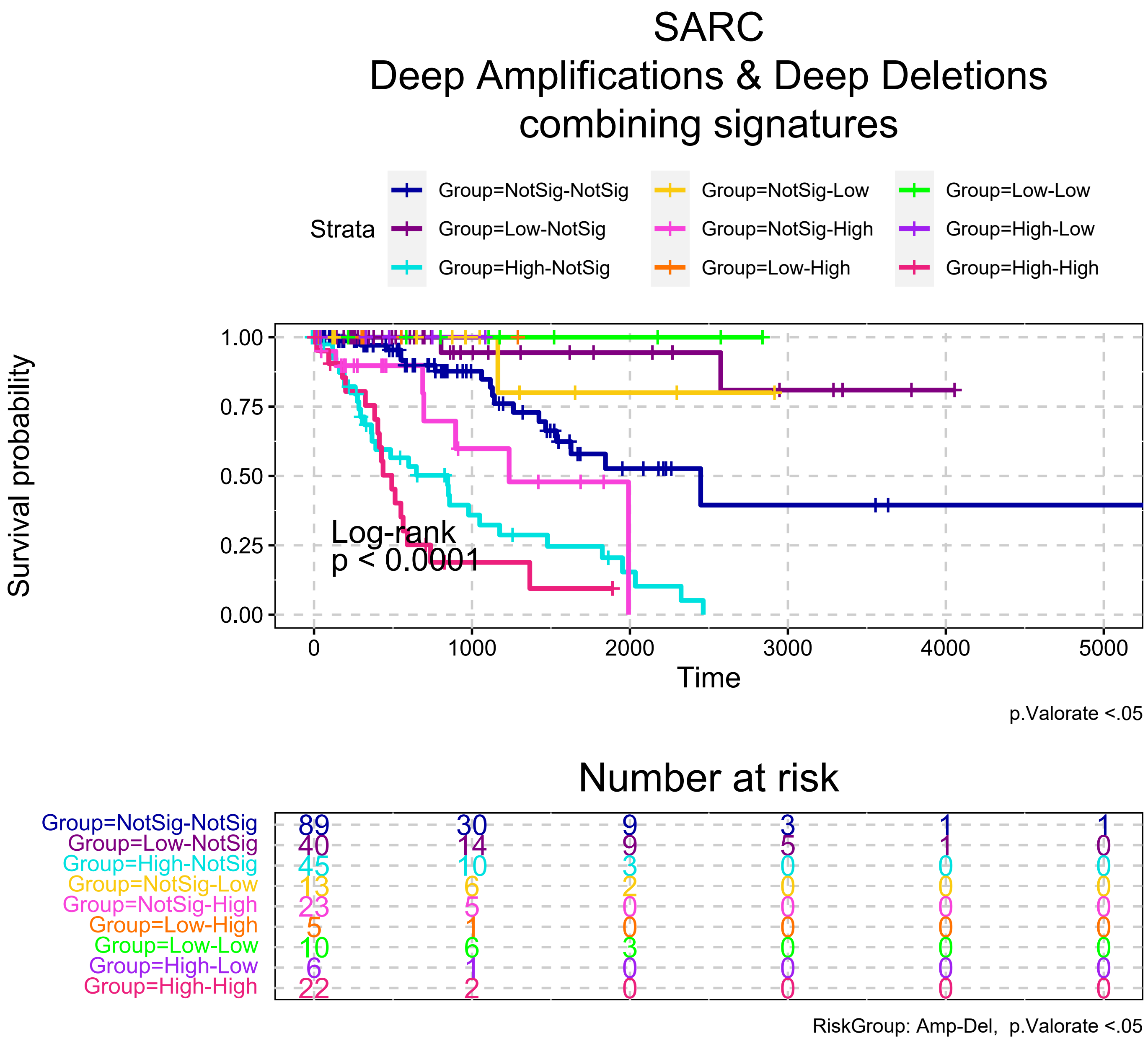

explanatory	beta	HR	L95	U95	p
Low-NotSig	-1.79	0.17	0.04	0.74	0.02
High-NotSig	1.52	4.56	2.53	8.24	0.00
NotSig-Low	-1.19	0.30	0.04	2.27	0.25
NotSig-High	0.85	2.34	0.97	5.66	0.06
Low-High	-17.73	0.00	0.00	Inf	1.00
Low-Low	-17.74	0.00	0.00	Inf	1.00
High-Low	-17.74	0.00	0.00	Inf	1.00
High-High	2.03	7.64	3.84	15.18	0.00

n= 254, number of events =75
Score(logrank) test = p <.0001
