# Supplementary material for: Systematic Modeling of Risk-Associated Copy Number Alterations in Cancer
Source: Int J Mol Sci. 2024 Sep 27;25(19):10455. doi: 10.3390/ijms251910455 (PMC11477427; doi:10.3390/ijms251910455)
Supplement: Supplementary file 1 [file ijms-25-10455-s001.zip › SKCMSignatureV12-sinSombreado.pdf]

SKCM
All Amplifications
Single Data Signature

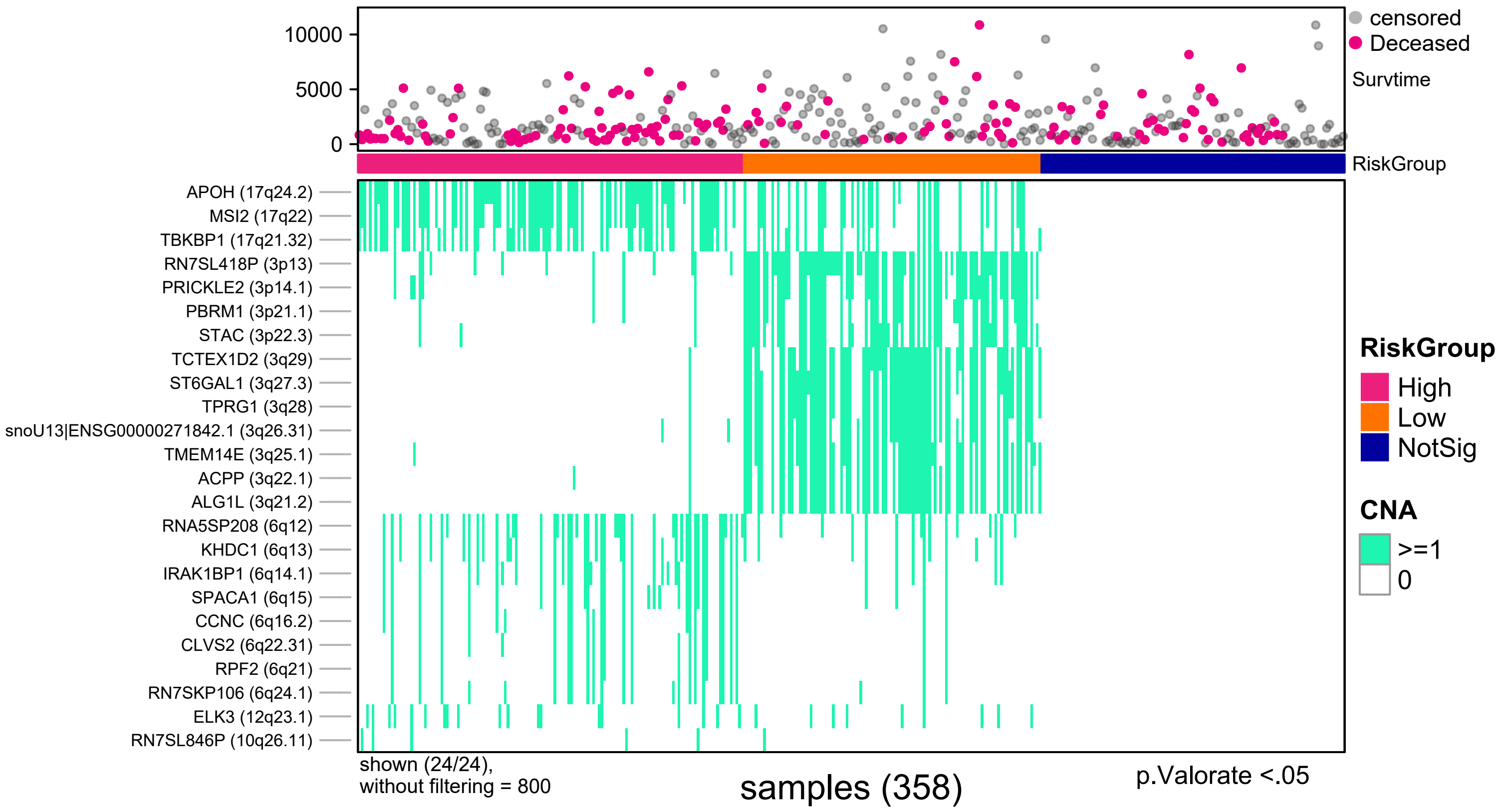

SKCM
All Amplifications
Single Data Signature

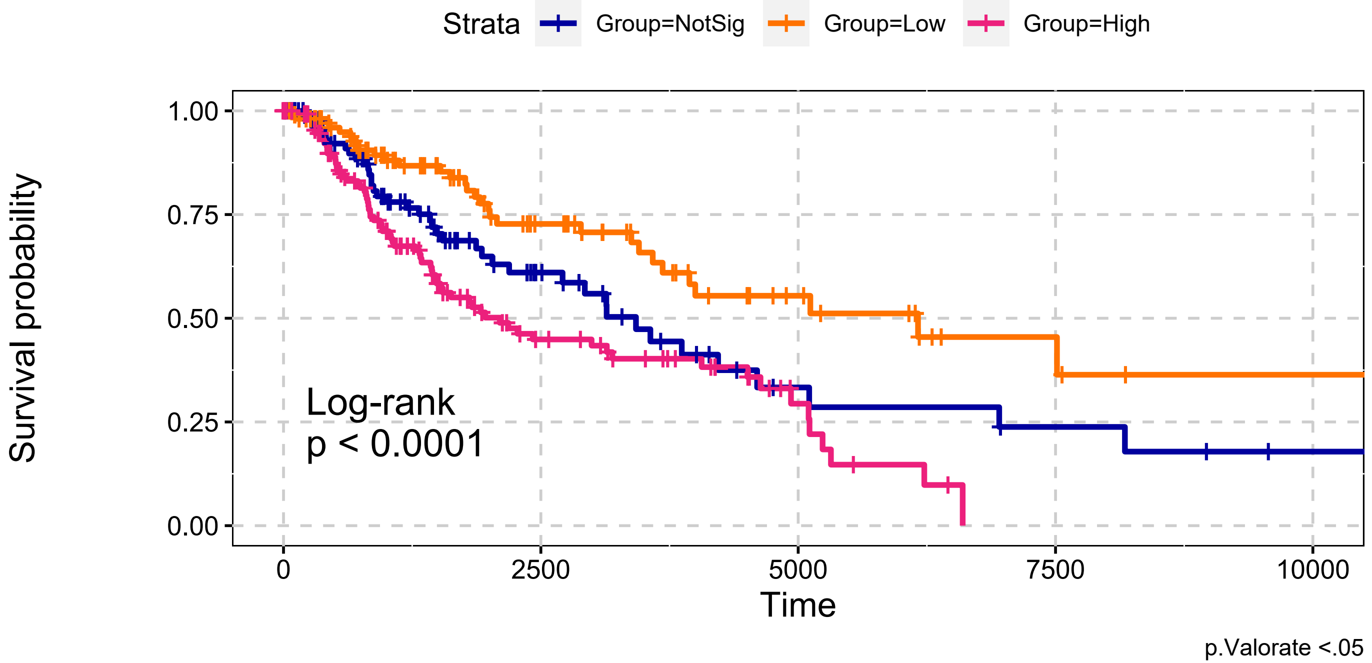

Number at risk

	0	2500	5000	7500	10000
Group=NotSig	110	26	7	4	1
Group=Low	108	40	14	5	2
Group=High	139	32	8	0	0

p.Valorate <.05

explanatory	beta	HR	L95	U95	p
Low	-0.53	0.59	0.37	0.95	0.03
High	0.38	1.46	0.98	2.15	0.06

n= 358, number of events =144
Score(logrank) test = p <.0001

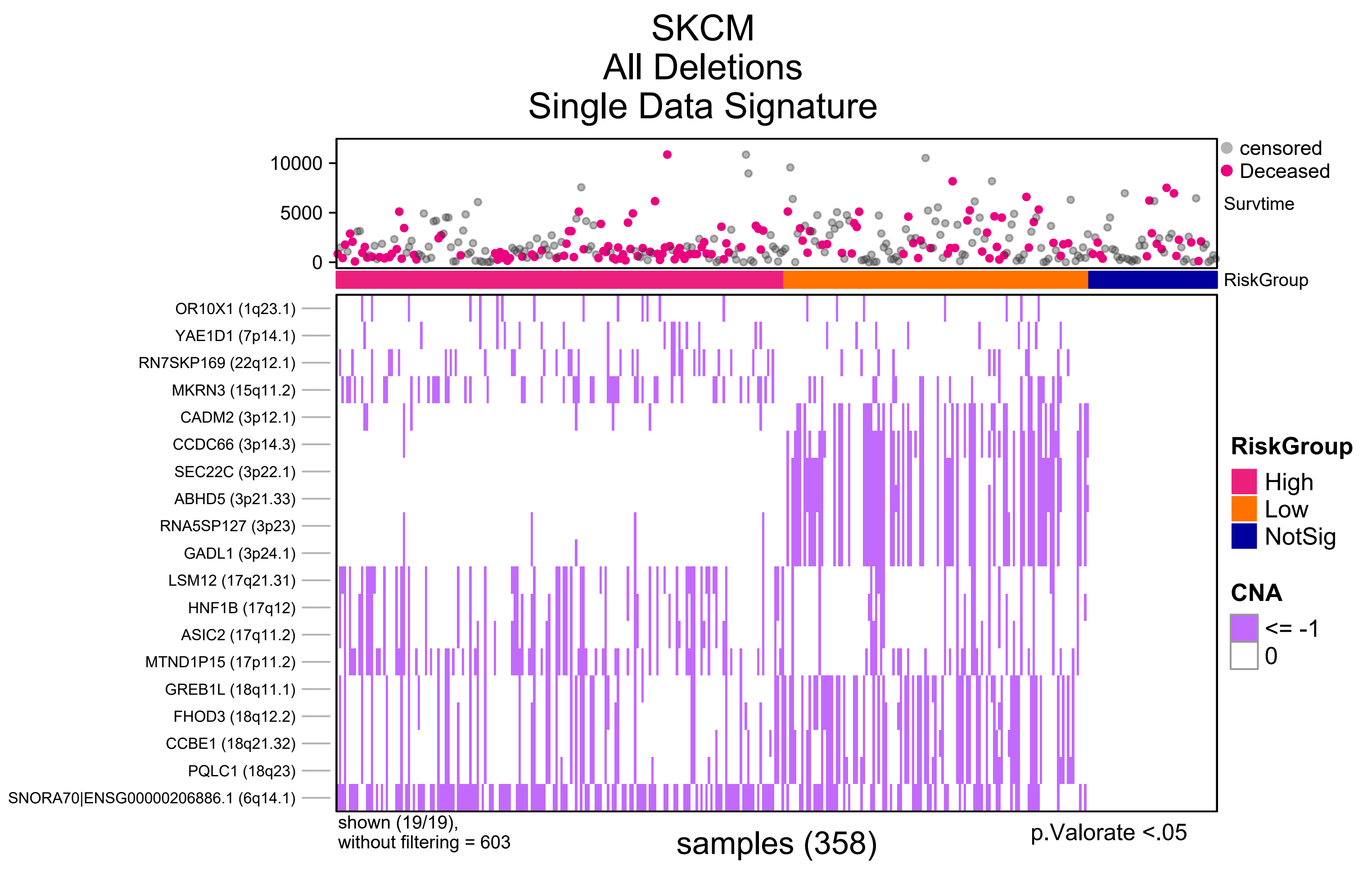
SKCM
All Deletions
Single Data Signature
10000
5000
0
censored
Deceased
Survtime
RiskGroup
OR10X1 (1q23.1)
YAE1D1 (7p14.1)
RN7SKP169 (22q12.1)
MKRN3 (15q11.2)
CADM2 (3p12.1)
CCDC66 (3p14.3)
SEC22C (3p22.1)
ABHD5 (3p21.33)
RNA5SP127 (3p23)
GADL1 (3p24.1)
LSM12 (17q21.31)
HNF1B (17q12)
ASIC2 (17q11.2)
MTND1P15 (17p11.2)
GREB1L (18q11.1)
FHOD3 (18q12.2)
CCBE1 (18q21.32)
PQLC1 (18q23)
SNORA70|ENSG00000206886.1 (6q14.1)
RiskGroup
High
Low
NotSig
CNA
<= -1
0
shown (19/19),
without filtering = 603
samples (358)
p.Valorate <.05

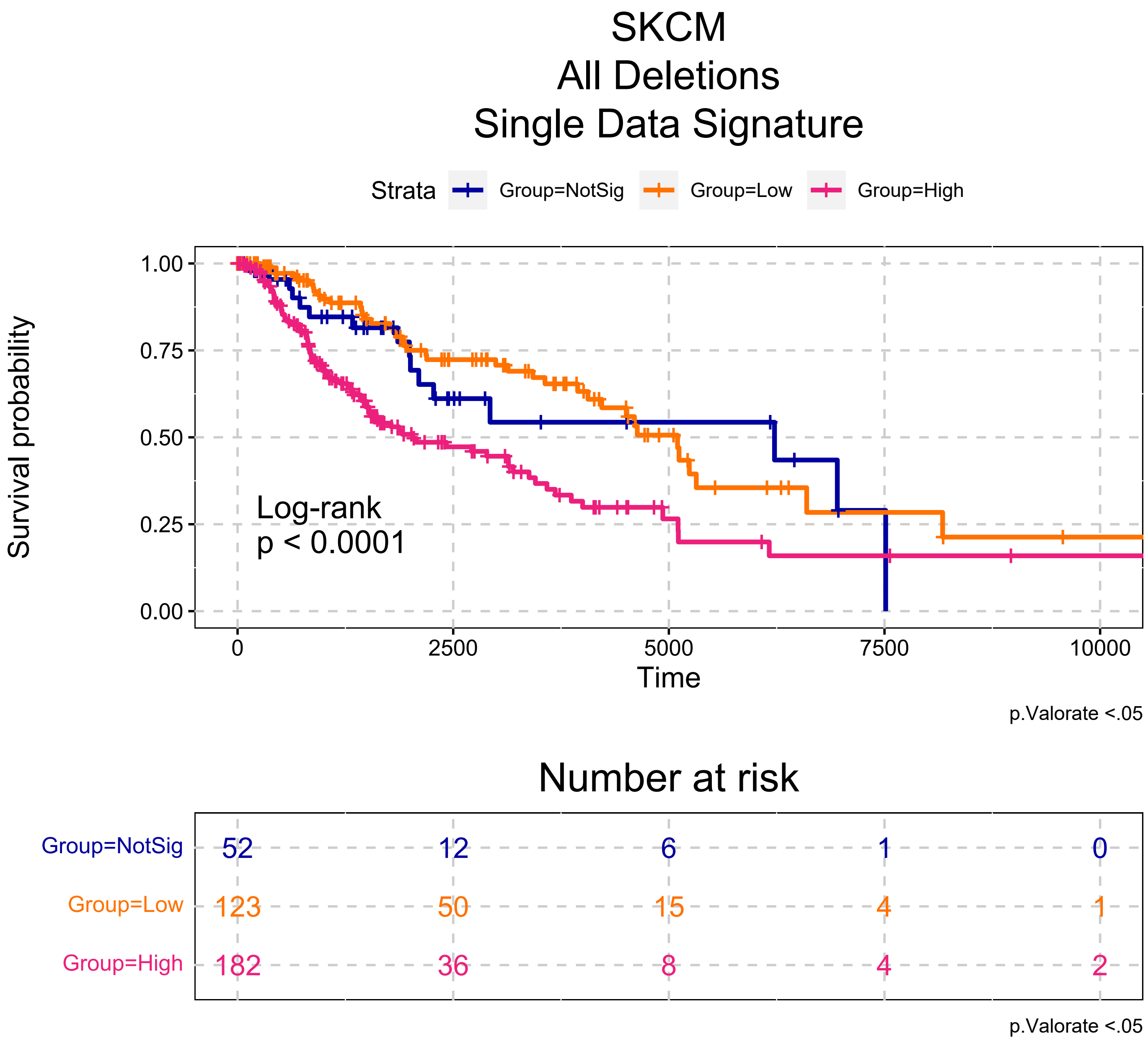

explanatory	beta	HR	L95	U95	p
Low	-0.17	0.85	0.47	1.52	0.58
High	0.63	1.88	1.10	3.22	0.02

n= 358, number of events =144
Score(logrank) test = p <.0001

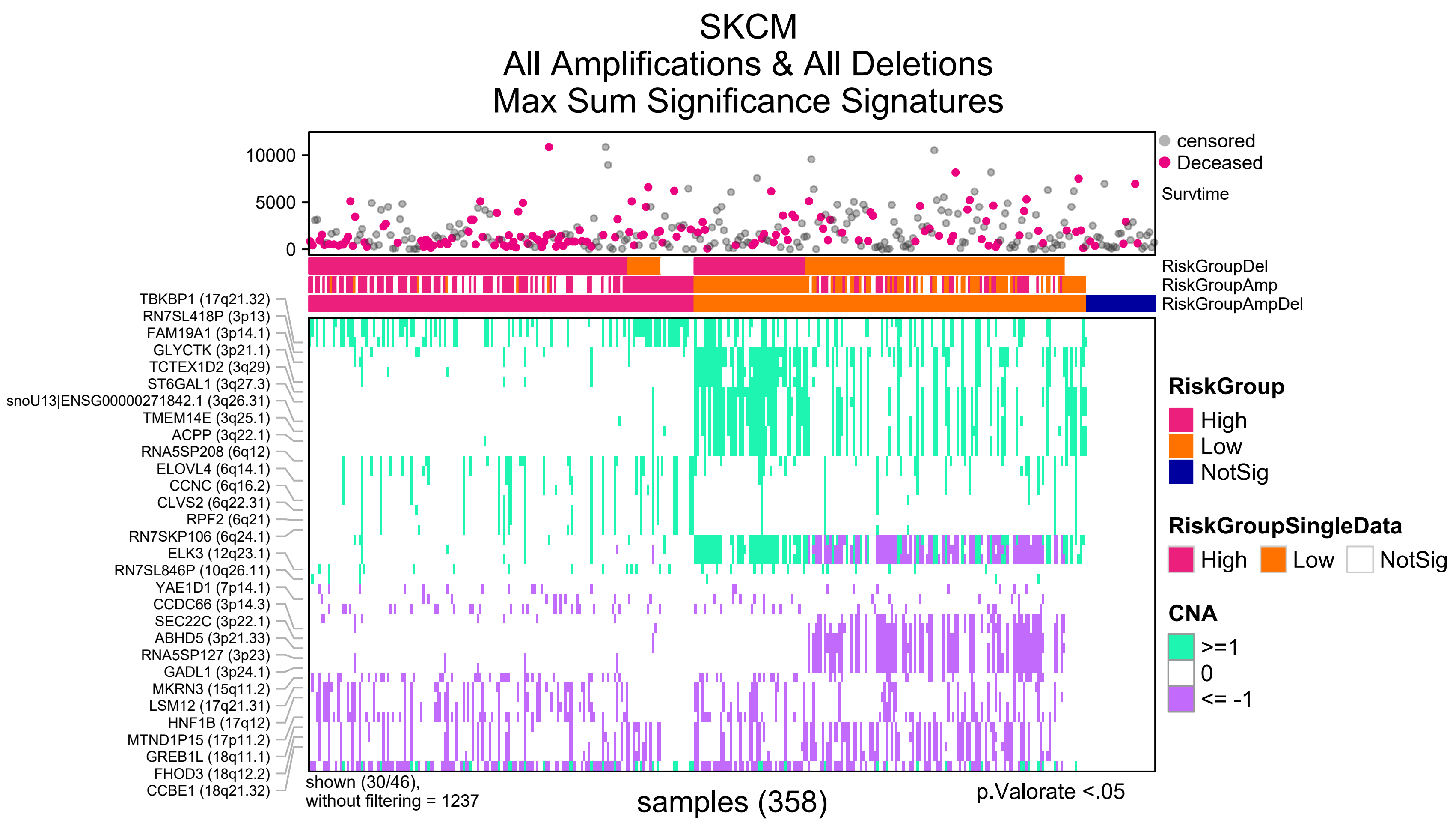
SKCM
All Amplifications & All Deletions
Max Sum Significance Signatures
10000
5000
0
censored
Deceased
Survtime
RiskGroupDel
RiskGroupAmp
RiskGroupAmpDel
TBKBP1 (17q21.32)
RN7SL418P (3p13)
FAM19A1 (3p14.1)
GLYCTK (3p21.1)
TCTEX1D2 (3q29)
ST6GAL1 (3q27.3)
snoU13|ENSG00000271842.1 (3q26.31)
TMEM14E (3q25.1)
ACPP (3q22.1)
RNA5SP208 (6q12)
ELOVL4 (6q14.1)
CCNC (6q16.2)
CLVS2 (6q22.31)
RPF2 (6q21)
RN7SKP106 (6q24.1)
ELK3 (12q23.1)
RN7SL846P (10q26.11)
YAE1D1 (7p14.1)
CCDC66 (3p14.3)
SEC22C (3p22.1)
ABHD5 (3p21.33)
RNA5SP127 (3p23)
GADL1 (3p24.1)
MKRN3 (15q11.2)
LSM12 (17q21.31)
HNF1B (17q12)
MTND1P15 (17p11.2)
GREB1L (18q11.1)
FHOD3 (18q12.2)
CCBE1 (18q21.32)
RiskGroup
High
Low
NotSig
RiskGroupSingleData
High
Low
NotSig
CNA
>=1
0
<= -1
shown (30/46),
without filtering = 1237
samples (358)
p.Valorate <.05

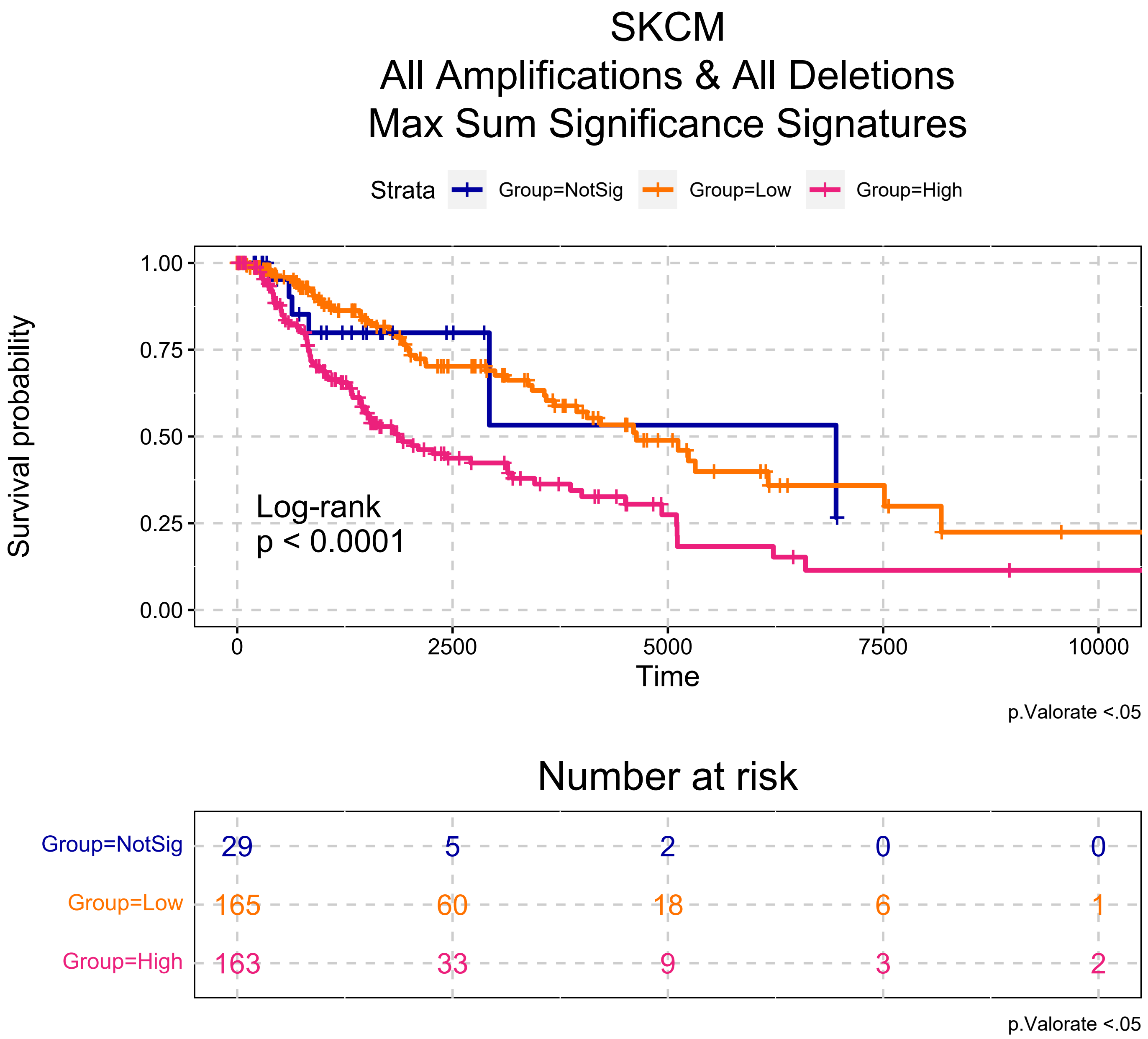

explanatory	beta	HR	L95	U95	p
Low	0.01	1.01	0.43	2.36	0.98
High	0.79	2.20	0.96	5.04	0.06

n= 358, number of events =144
Score(logrank) test = p <.0001

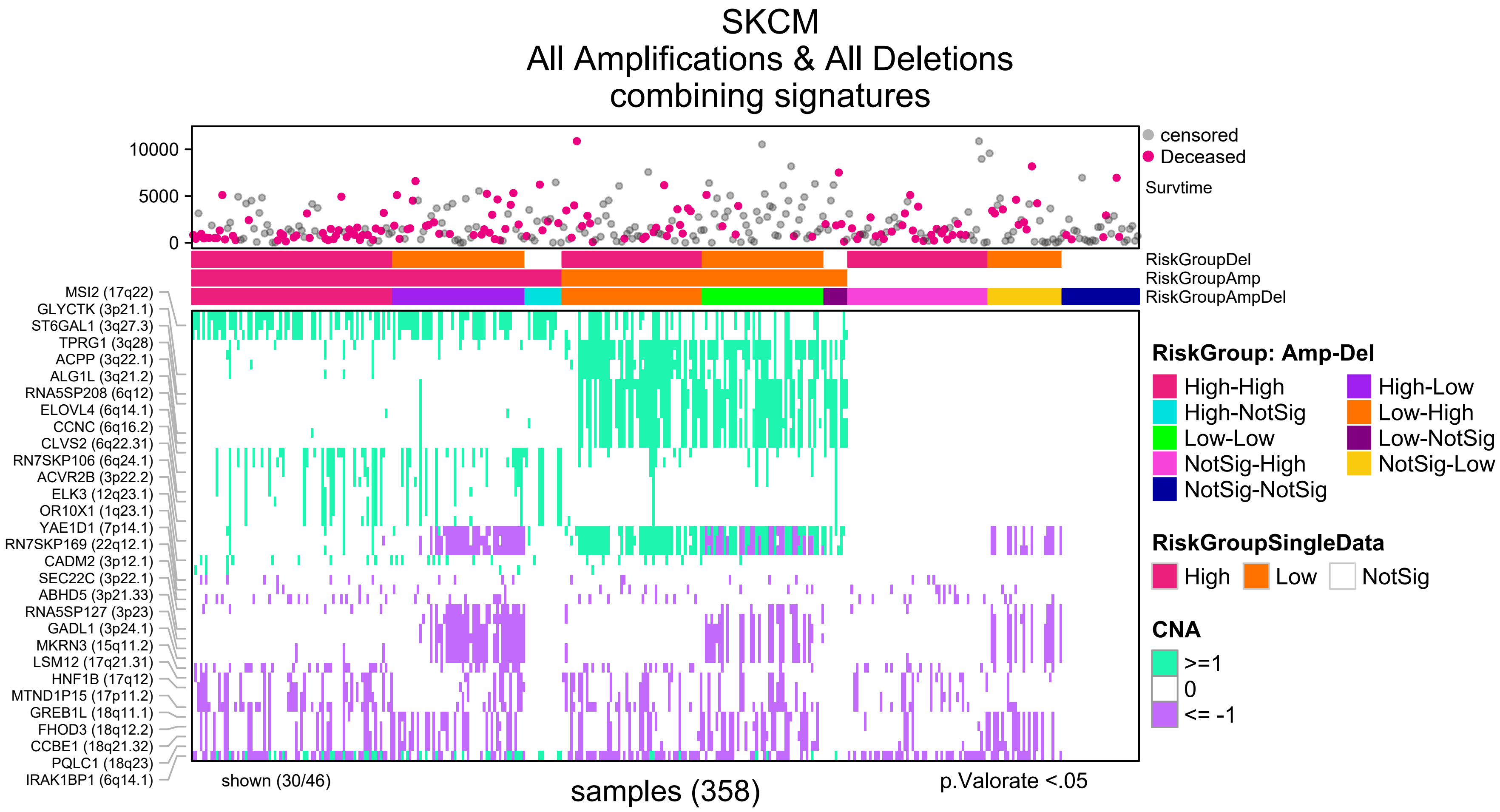

SKCM
All Amplifications & All Deletions
combining signatures
10000
5000
0
censored
Deceased
Survtime
RiskGroupDel
RiskGroupAmp
RiskGroupAmpDel
MSI2 (17q22)
GLYCTK (3p21.1)
ST6GAL1 (3q27.3)
TPRG1 (3q28)
ACPP (3q22.1)
ALG1L (3q21.2)
RNA5SP208 (6q12)
ELOVL4 (6q14.1)
CCNC (6q16.2)
CLVS2 (6q22.31)
RN7SKP106 (6q24.1)
ACVR2B (3p22.2)
ELK3 (12q23.1)
OR10X1 (1q23.1)
YAE1D1 (7p14.1)
RN7SKP169 (22q12.1)
CADM2 (3p12.1)
SEC22C (3p22.1)
ABHD5 (3p21.33)
RNA5SP127 (3p23)
GADL1 (3p24.1)
MKRN3 (15q11.2)
LSM12 (17q21.31)
HNF1B (17q12)
MTND1P15 (17p11.2)
GREB1L (18q11.1)
FHOD3 (18q12.2)
CCBE1 (18q21.32)
PQLC1 (18q23)
IRAK1BP1 (6q14.1)
shown (30/46)
samples (358)
p.Valorate <.05
RiskGroup: Amp-Del
High-High
High-NotSig
Low-Low
NotSig-High
NotSig-NotSig
High-Low
Low-High
Low-NotSig
NotSig-Low
RiskGroupSingleData
High
Low
NotSig
CNA
>=1
0
<= -1

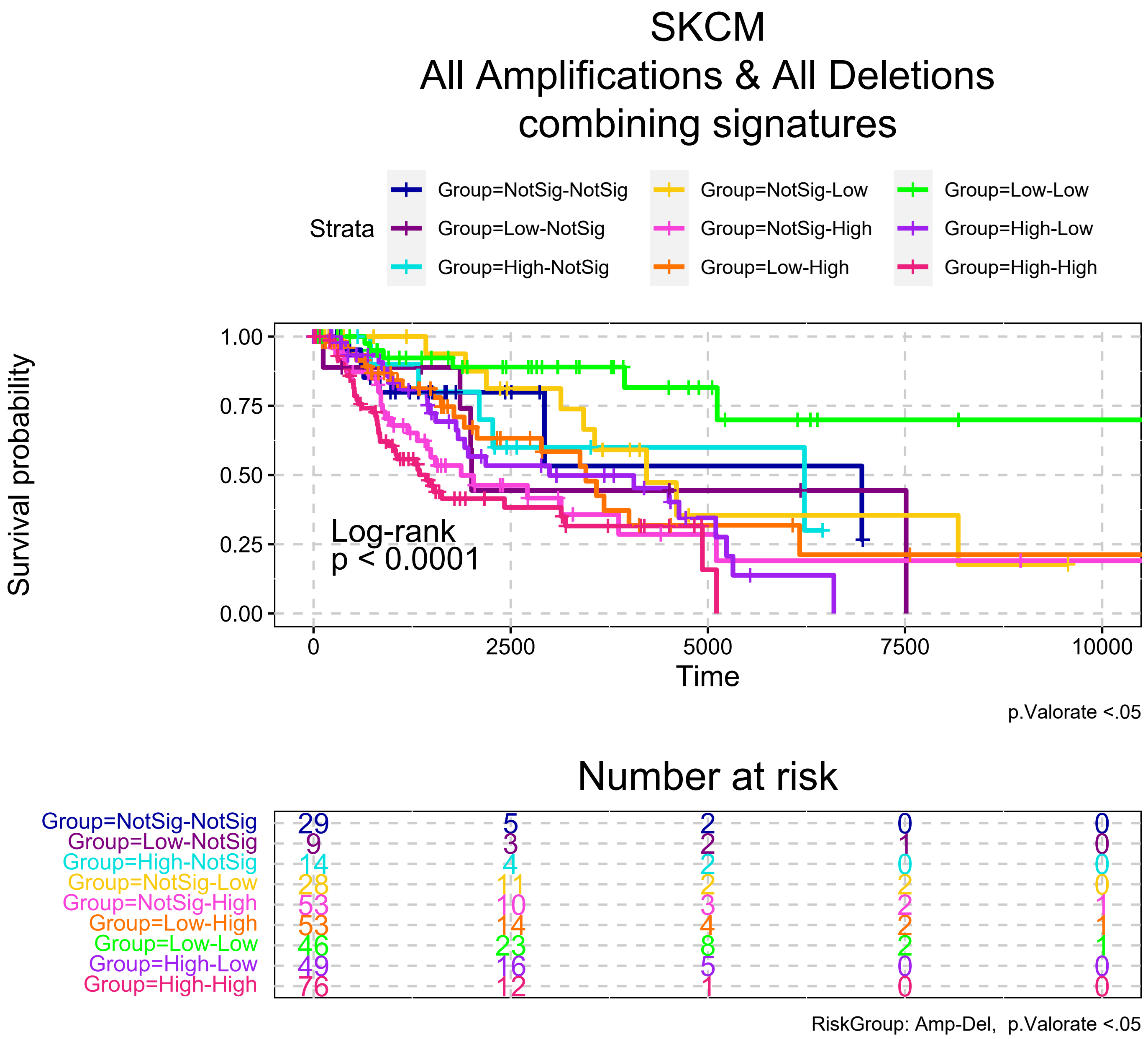

explanatory	beta	HR	L95	U95	p
Low-NotSig	0.28	1.32	0.40	4.34	0.65
High-NotSig	0.11	1.12	0.34	3.68	0.85
NotSig-Low	-0.02	0.98	0.35	2.76	0.97
NotSig-High	0.73	2.08	0.85	5.10	0.11
Low-High	0.33	1.39	0.56	3.48	0.48
Low-Low	-1.09	0.34	0.11	1.05	0.06
High-Low	0.53	1.69	0.69	4.15	0.25
High-High	1.08	2.96	1.25	7.00	0.01

n= 358, number of events =144
Score(logrank) test = p <.0001

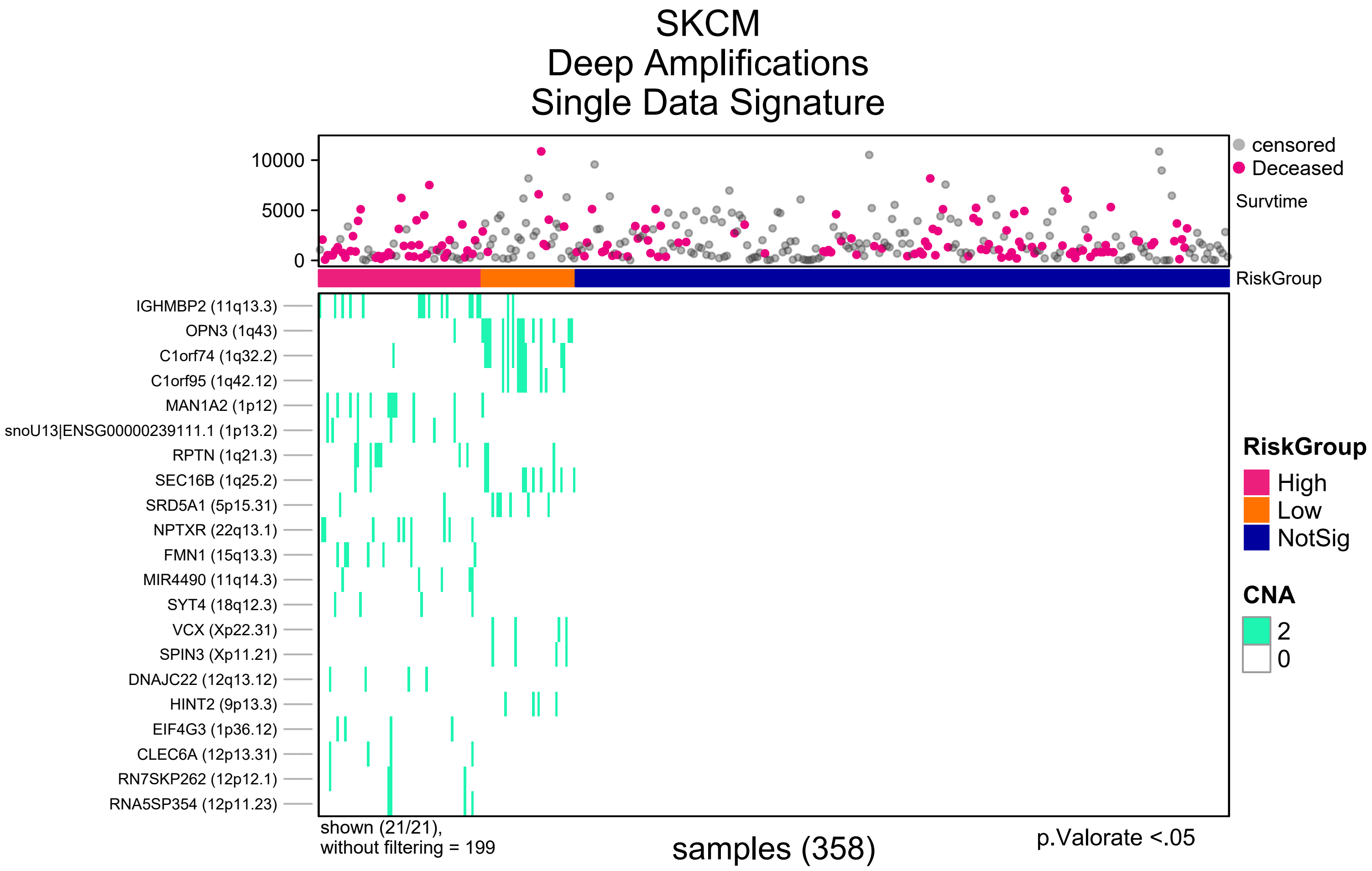
SKCM
Deep Amplifications
Single Data Signature
10000
5000
0
censored
Deceased
Survtime
RiskGroup
IGHMBP2 (11q13.3)
OPN3 (1q43)
C1orf74 (1q32.2)
C1orf95 (1q42.12)
MAN1A2 (1p12)
snoU13|ENSG00000239111.1 (1p13.2)
RPTN (1q21.3)
SEC16B (1q25.2)
SRD5A1 (5p15.31)
NPTXR (22q13.1)
FMN1 (15q13.3)
MIR4490 (11q14.3)
SYT4 (18q12.3)
VCX (Xp22.31)
SPIN3 (Xp11.21)
DNAJC22 (12q13.12)
HINT2 (9p13.3)
EIF4G3 (1p36.12)
CLEC6A (12p13.31)
RN7SKP262 (12p12.1)
RNA5SP354 (12p11.23)
RiskGroup
High
Low
NotSig
CNA
2
0
shown (21/21),
without filtering = 199
samples (358)
p.Valorate <.05

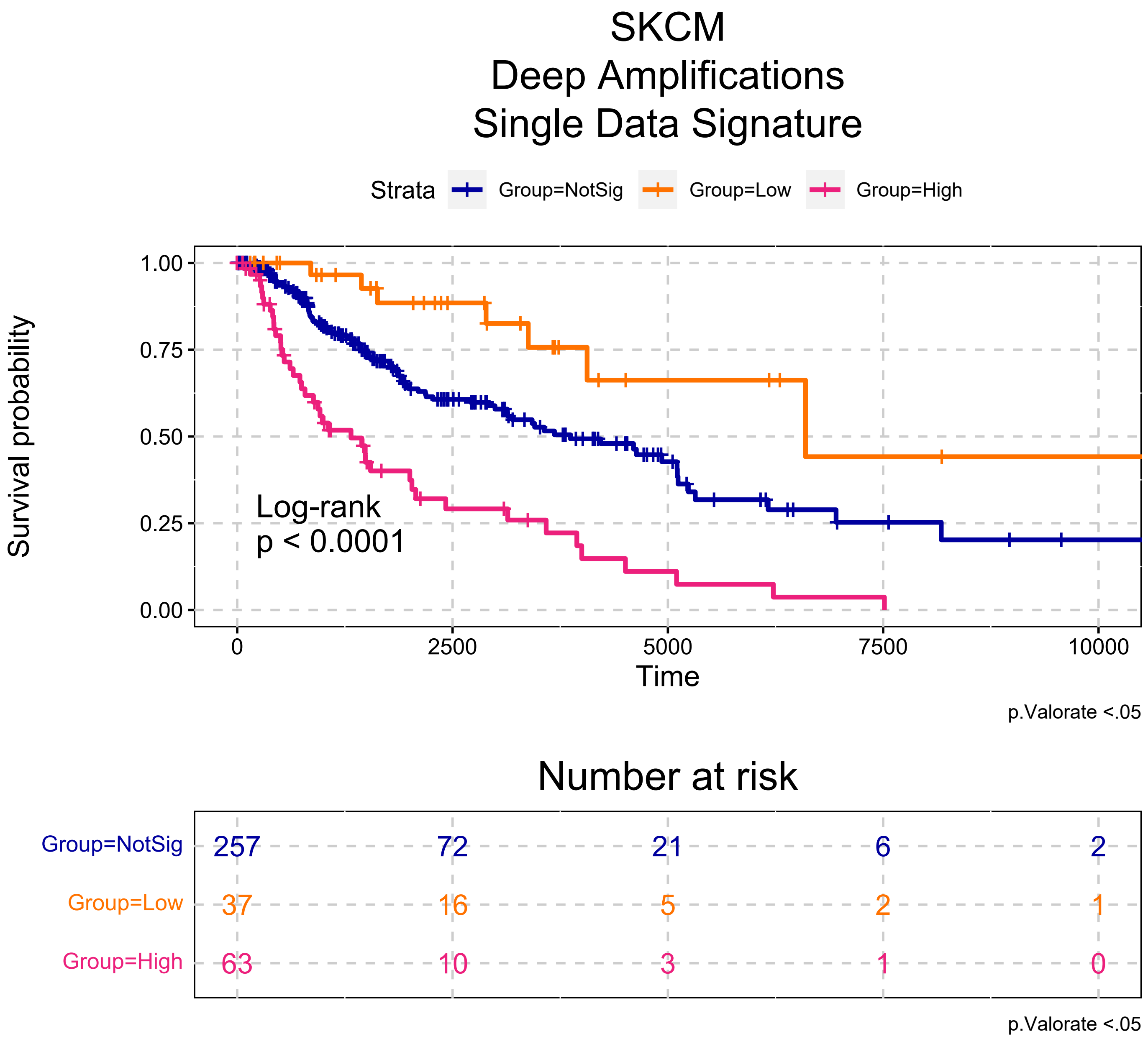

explanatory	beta	HR	L95	U95	p
Low	-0.96	0.38	0.18	0.83	0.01
High	0.99	2.69	1.87	3.87	0.00

n= 358, number of events =144
Score(logrank) test = p <.0001

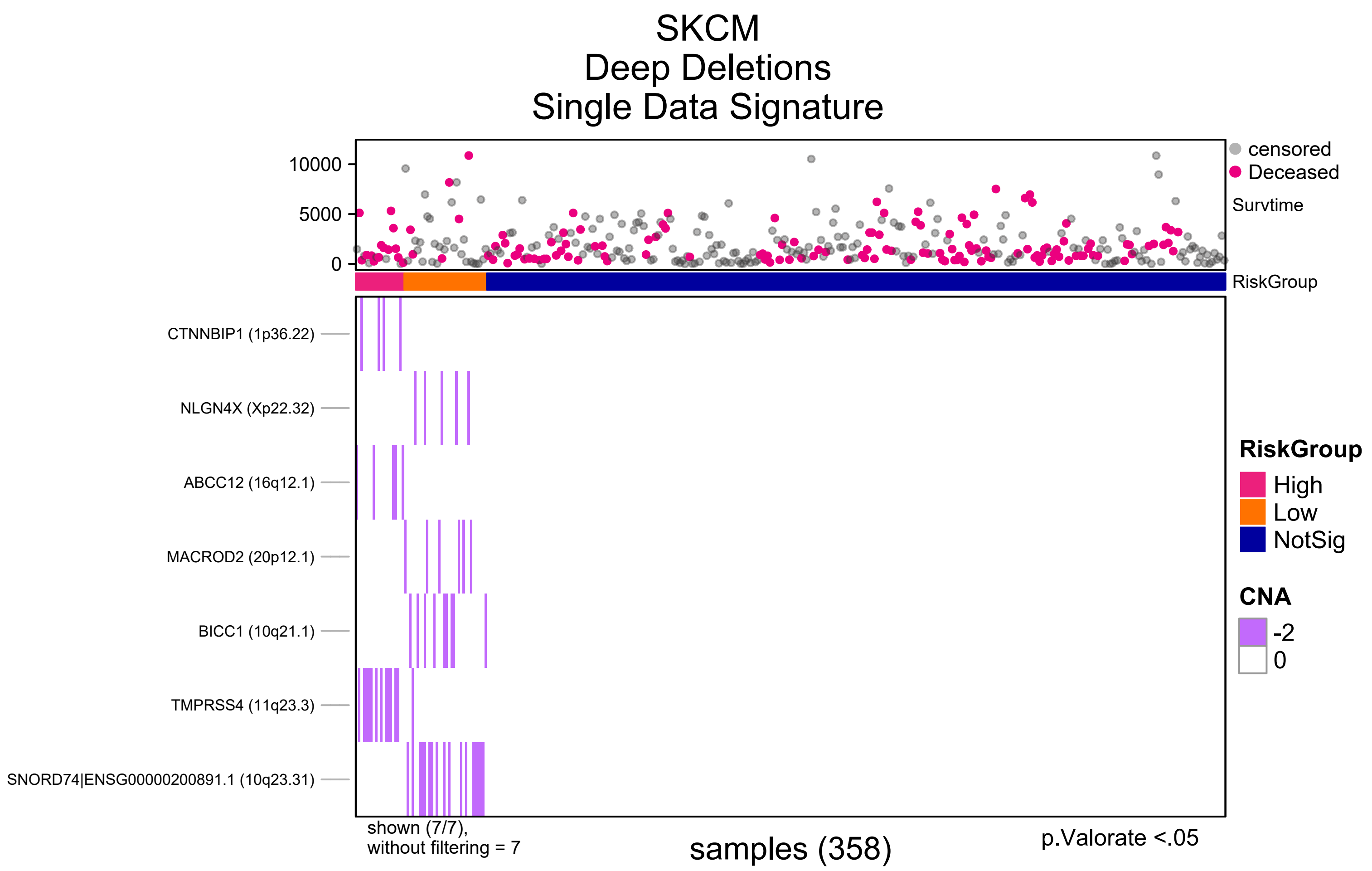
SKCM
Deep Deletions
Single Data Signature
10000
5000
0
censored
Deceased
Survtime
RiskGroup
CTNNBIP1 (1p36.22)
NLGN4X (Xp22.32)
ABCC12 (16q12.1)
MACROD2 (20p12.1)
BICC1 (10q21.1)
TMPRSS4 (11q23.3)
SNORD74|ENSG00000200891.1 (10q23.31)
RiskGroup
High
Low
NotSig
CNA
-2
0
shown (7/7),
without filtering = 7
samples (358)
p.Valorate <.05

SKCM
Deep Deletions
Single Data Signature

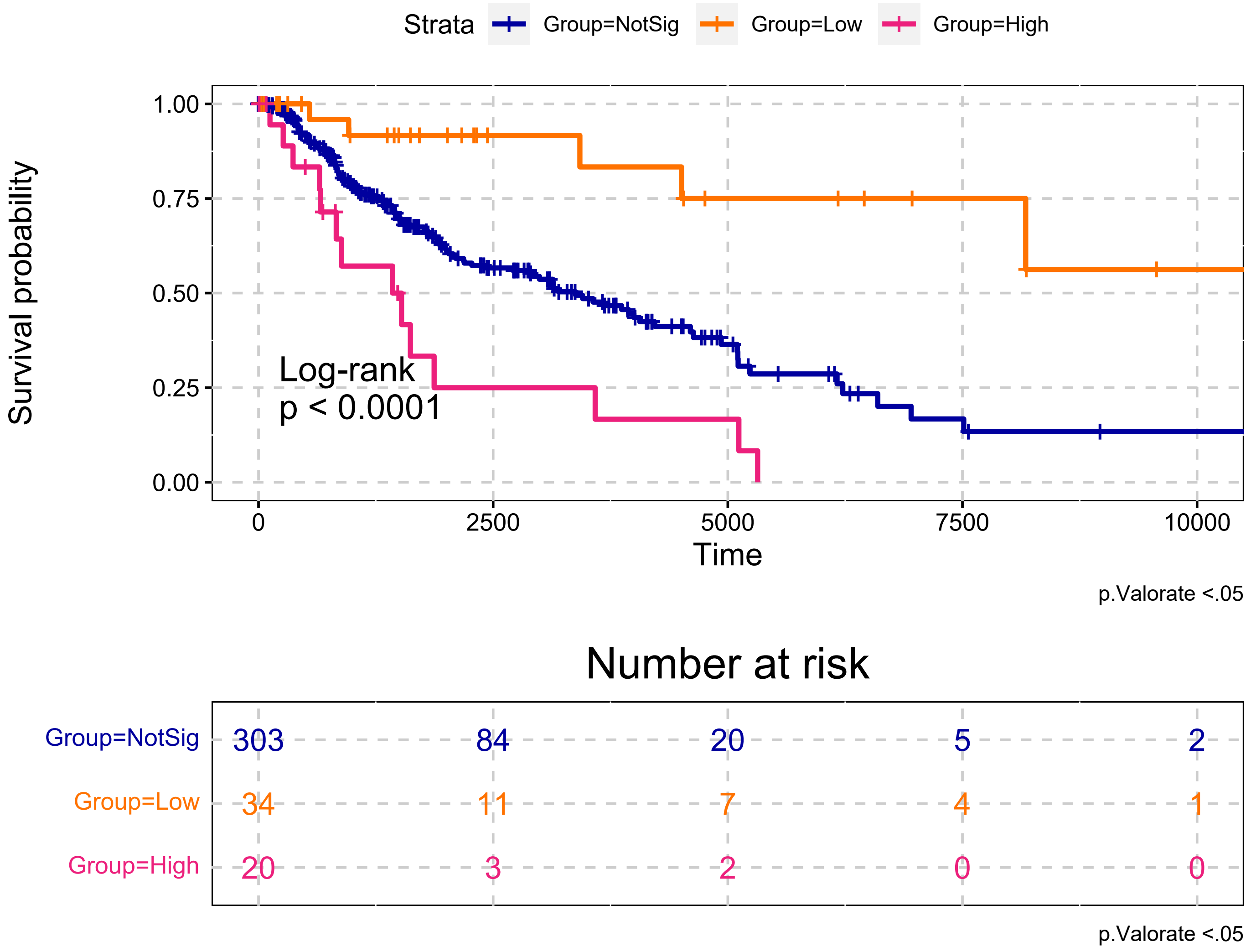

explanatory	beta	HR	L95	U95	p
Low	-1.46	0.23	0.09	0.57	0.00
High	0.82	2.28	1.31	3.98	0.00

n= 358, number of events =144
Score(logrank) test = p <.0001

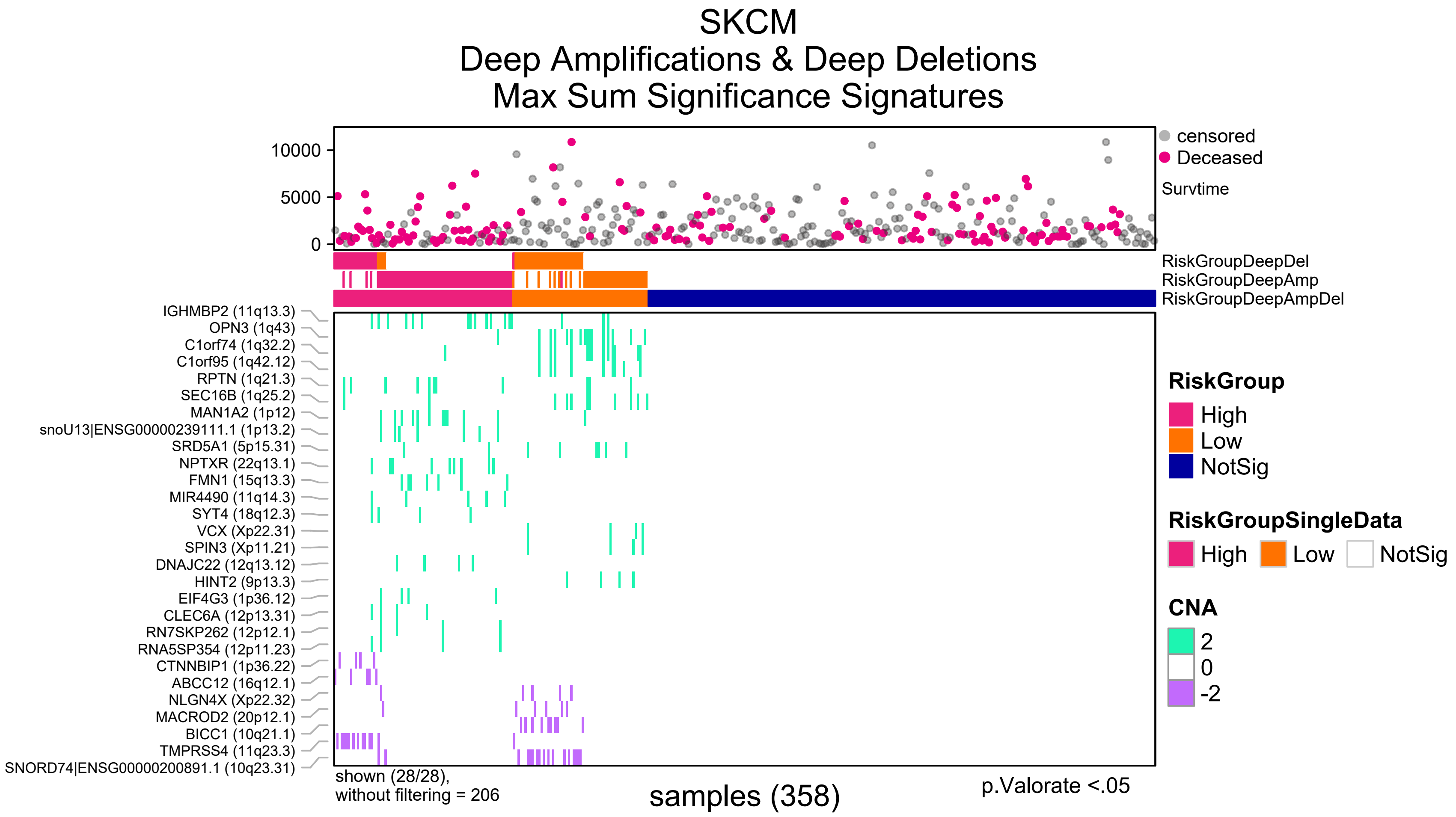

SKCM
Deep Amplifications & Deep Deletions
Max Sum Significance Signatures
10000
5000
0
censored
Deceased
Survtime
RiskGroupDeepDel
RiskGroupDeepAmp
RiskGroupDeepAmpDel
IGHMBP2 (11q13.3)
OPN3 (1q43)
C1orf74 (1q32.2)
C1orf95 (1q42.12)
RPTN (1q21.3)
SEC16B (1q25.2)
MAN1A2 (1p12)
snoU13|ENSG00000239111.1 (1p13.2)
SRD5A1 (5p15.31)
NPTXR (22q13.1)
FMN1 (15q13.3)
MIR4490 (11q14.3)
SYT4 (18q12.3)
VCX (Xp22.31)
SPIN3 (Xp11.21)
DNAJC22 (12q13.12)
HINT2 (9p13.3)
EIF4G3 (1p36.12)
CLEC6A (12p13.31)
RN7SKP262 (12p12.1)
RNA5SP354 (12p11.23)
CTNNBIP1 (1p36.22)
ABCC12 (16q12.1)
NLGN4X (Xp22.32)
MACROD2 (20p12.1)
BICC1 (10q21.1)
TMPRSS4 (11q23.3)
SNORD74|ENSG00000200891.1 (10q23.31)
RiskGroup
High
Low
NotSig
RiskGroupSingleData
High
Low
NotSig
CNA
2
0
-2
shown (28/28),
without filtering = 206
samples (358)
p.Valorate <.05

SKCM
Deep Amplifications & Deep Deletions
Max Sum Significance Signatures

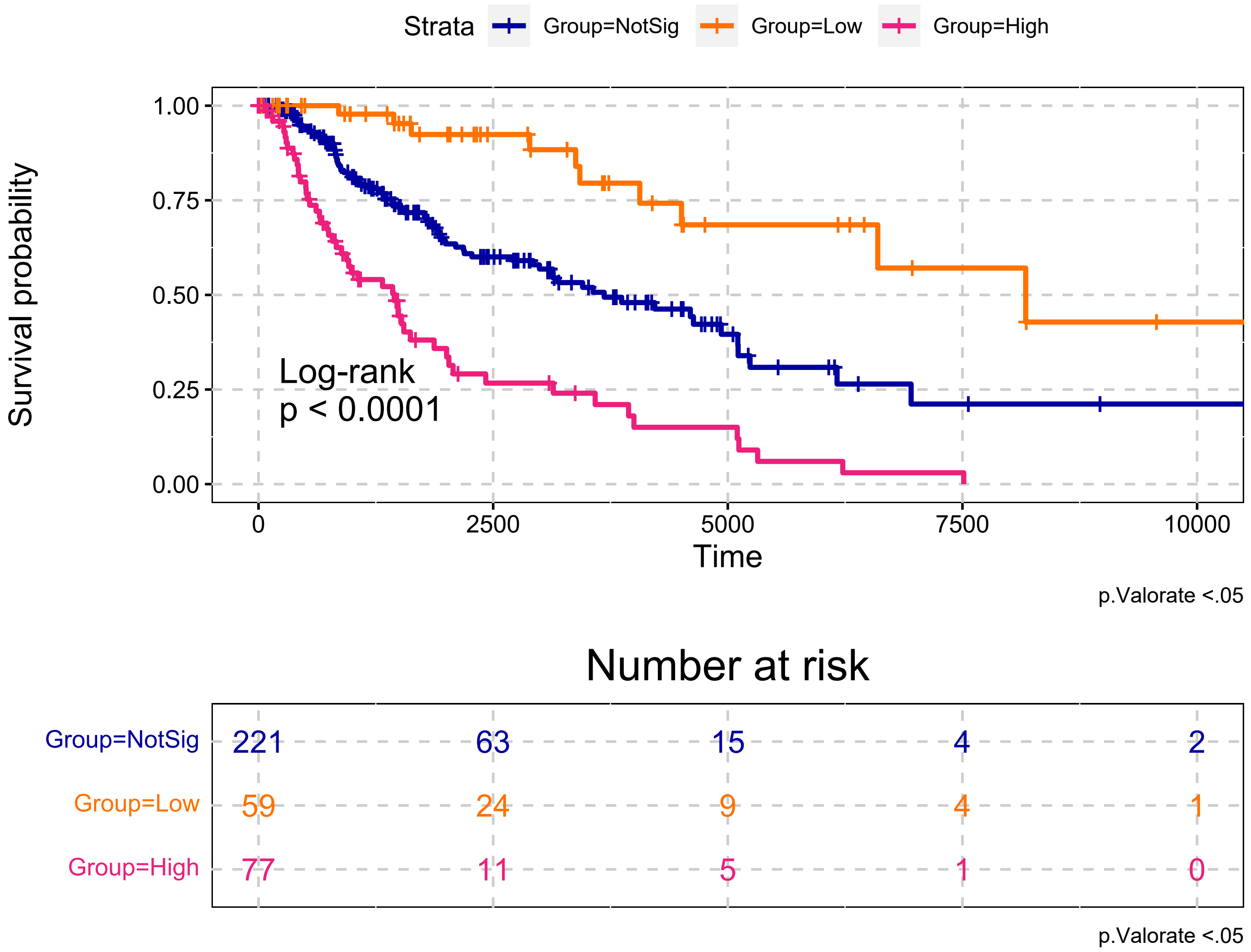

explanatory	beta	HR	L95	U95	p
Low	-1.14	0.32	0.16	0.62	0.00
High	0.96	2.60	1.83	3.69	0.00

n= 358, number of events =144
Score(logrank) test = p <.0001

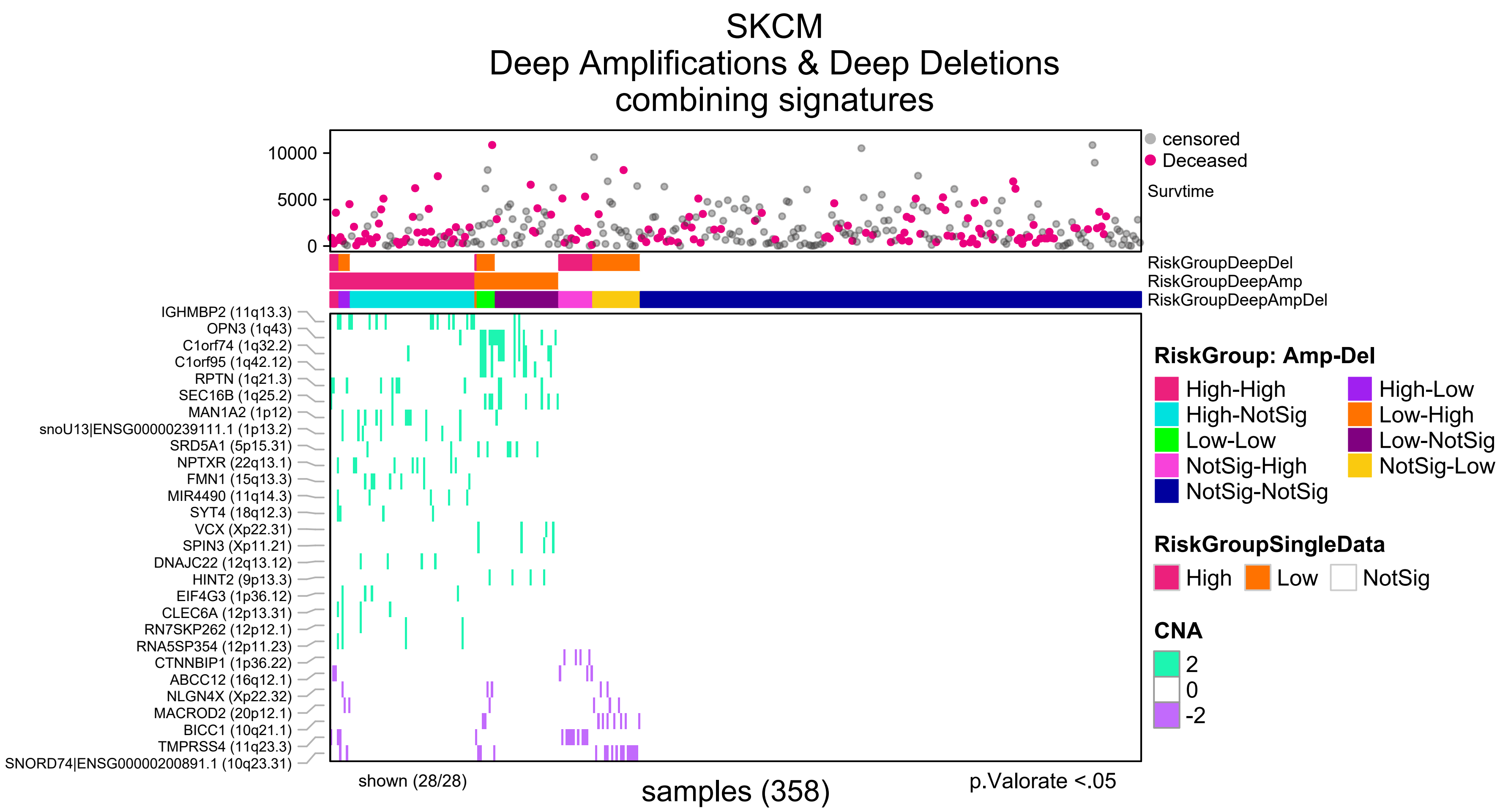

SKCM
Deep Amplifications & Deep Deletions
combining signatures
10000
5000
0
censored
Deceased
Survtime
RiskGroupDeepDel
RiskGroupDeepAmp
RiskGroupDeepAmpDel
IGHMBP2 (11q13.3)
OPN3 (1q43)
C1orf74 (1q32.2)
C1orf95 (1q42.12)
RPTN (1q21.3)
SEC16B (1q25.2)
MAN1A2 (1p12)
snoU13|ENSG00000239111.1 (1p13.2)
SRD5A1 (5p15.31)
NPTXR (22q13.1)
FMN1 (15q13.3)
MIR4490 (11q14.3)
SYT4 (18q12.3)
VCX (Xp22.31)
SPIN3 (Xp11.21)
DNAJC22 (12q13.12)
HINT2 (9p13.3)
EIF4G3 (1p36.12)
CLEC6A (12p13.31)
RN7SKP262 (12p12.1)
RNA5SP354 (12p11.23)
CTNNBIP1 (1p36.22)
ABCC12 (16q12.1)
NLGN4X (Xp22.32)
MACROD2 (20p12.1)
BICC1 (10q21.1)
TMPRSS4 (11q23.3)
SNORD74|ENSG00000200891.1 (10q23.31)
RiskGroup: Amp-Del
High-High
High-NotSig
Low-Low
NotSig-High
NotSig-NotSig
High-Low
Low-High
Low-NotSig
NotSig-Low
RiskGroupSingleData
High
Low
NotSig
CNA
2
0
-2
shown (28/28)
samples (358)
p.Valorate <.05

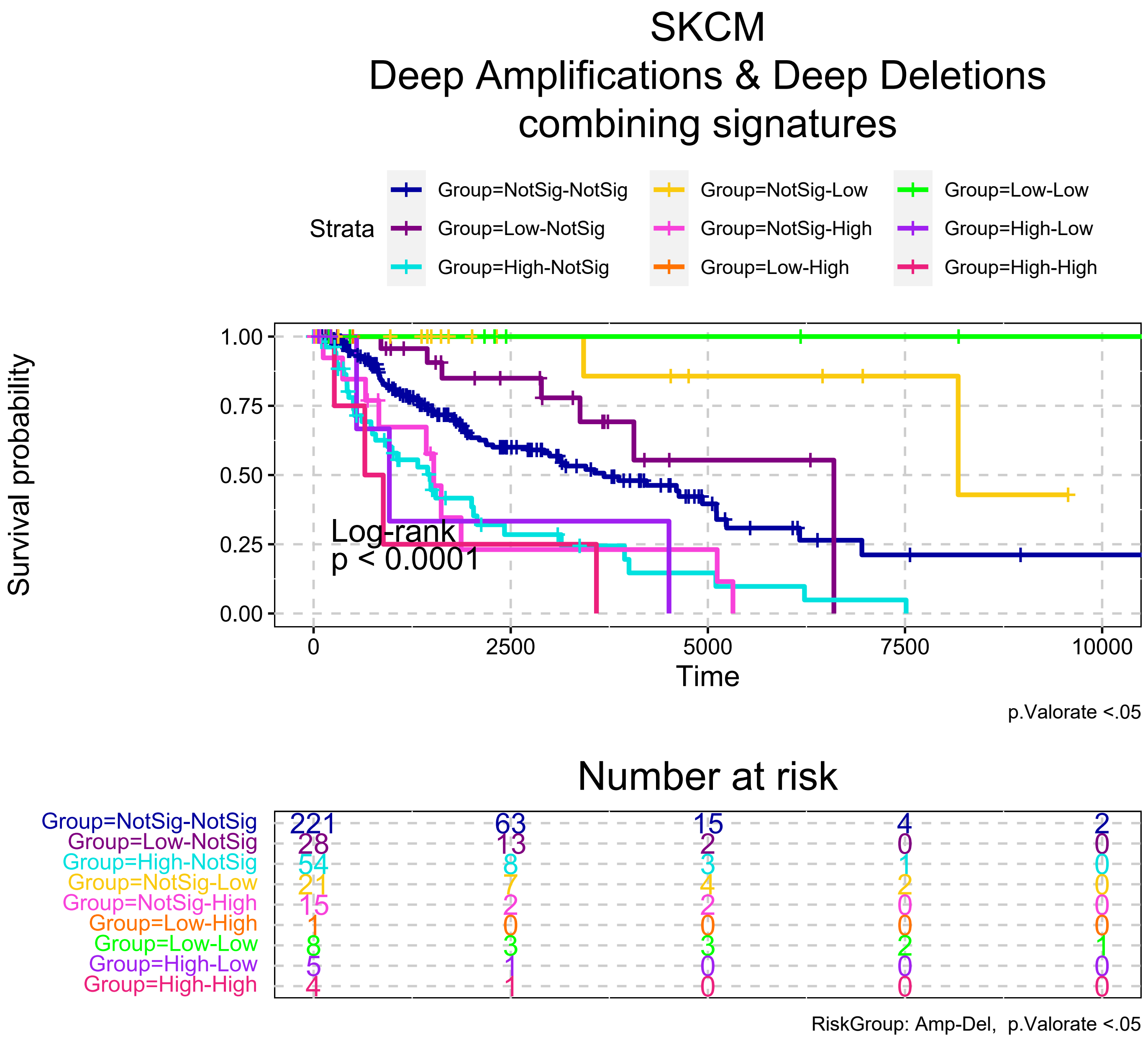

explanatory	beta	HR	L95	U95	p
Low-NotSig	-0.59	0.55	0.25	1.20	0.13
High-NotSig	0.91	2.49	1.68	3.70	0.00
NotSig-Low	-1.76	0.17	0.04	0.70	0.01
NotSig-High	0.84	2.32	1.20	4.50	0.01
Low-High	-17.17	0.00	0.00	Inf	1.00
Low-Low	-17.36	0.00	0.00	Inf	0.99
High-Low	1.02	2.78	0.88	8.84	0.08
High-High	1.43	4.20	1.53	11.50	0.01

n= 358, number of events =144
Score(logrank) test = p <.0001
