# Supplementary material for: Systematic Modeling of Risk-Associated Copy Number Alterations in Cancer
Source: Int J Mol Sci. 2024 Sep 27;25(19):10455. doi: 10.3390/ijms251910455 (PMC11477427; doi:10.3390/ijms251910455)
Supplement: Supplementary file 1 [file ijms-25-10455-s001.zip › STADSignatureV12-sinSombreado.pdf]

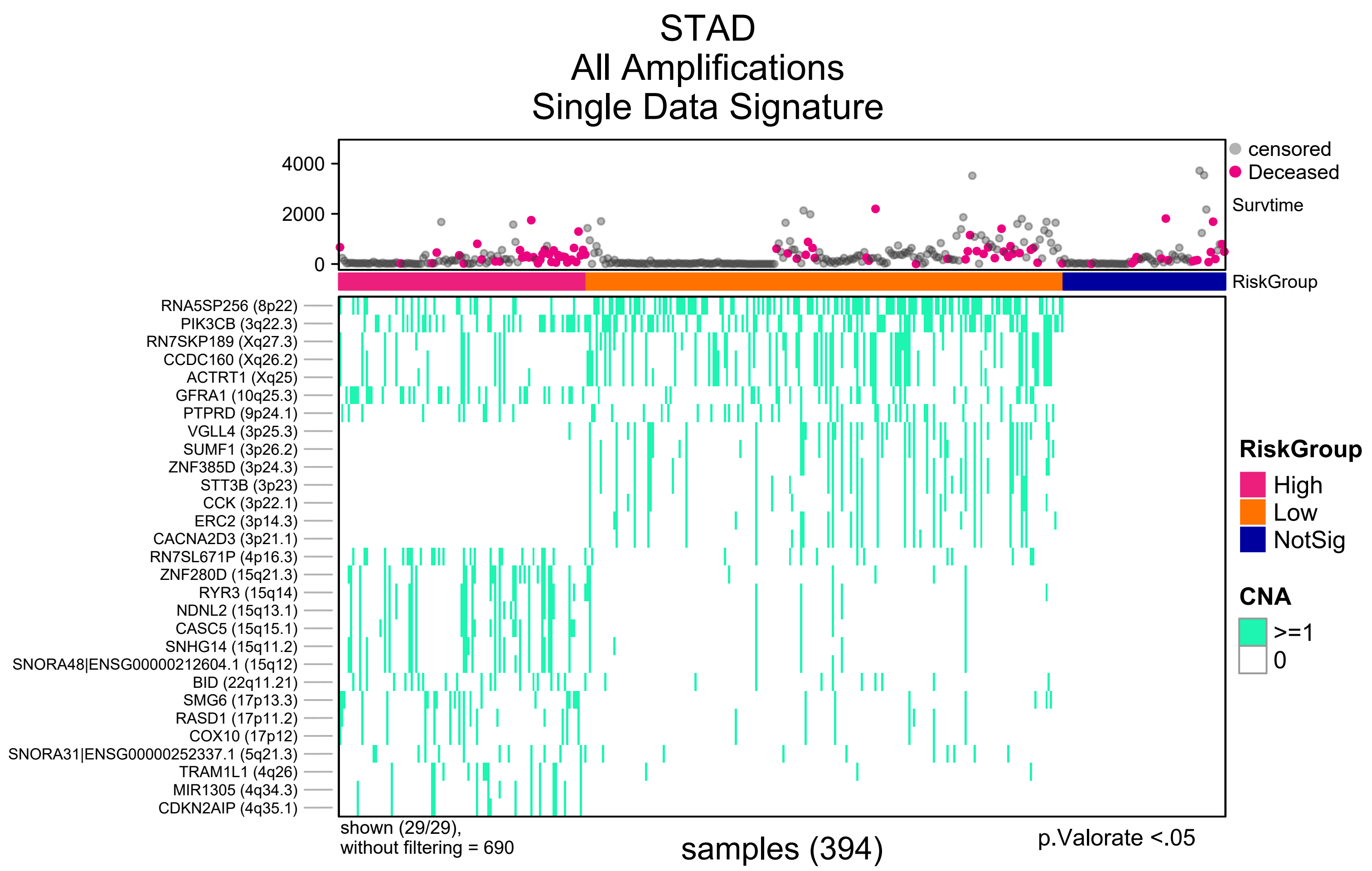

STAD
All Amplifications
Single Data Signature
4000
2000
0
censored
Deceased
Survtime
RiskGroup
RNA5SP256 (8p22)
PIK3CB (3q22.3)
RN7SKP189 (Xq27.3)
CCDC160 (Xq26.2)
ACTRT1 (Xq25)
GFRA1 (10q25.3)
PTPRD (9p24.1)
VGLL4 (3p25.3)
SUMF1 (3p26.2)
ZNF385D (3p24.3)
STT3B (3p23)
CCK (3p22.1)
ERC2 (3p14.3)
CACNA2D3 (3p21.1)
RN7SL671P (4p16.3)
ZNF280D (15q21.3)
RYR3 (15q14)
NDNL2 (15q13.1)
CASC5 (15q15.1)
SNHG14 (15q11.2)
SNORA48|ENSG00000212604.1 (15q12)
BID (22q11.21)
SMG6 (17p13.3)
RASD1 (17p11.2)
COX10 (17p12)
SNORA31|ENSG00000252337.1 (5q21.3)
TRAM1L1 (4q26)
MIR1305 (4q34.3)
CDKN2AIP (4q35.1)
RiskGroup
High
Low
NotSig
CNA
>=1
0
shown (29/29),
without filtering = 690
samples (394)
p.Valorate <.05

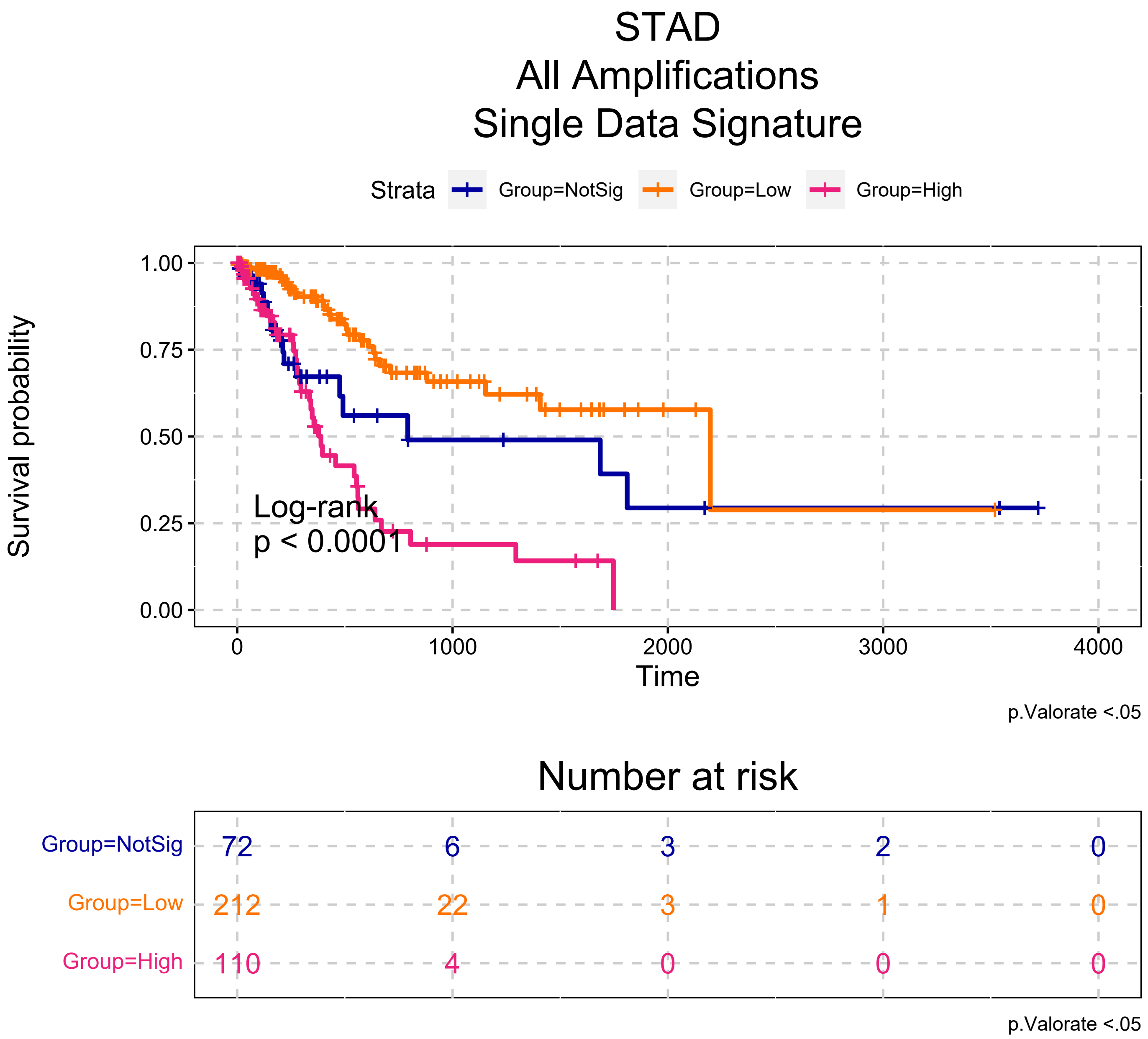

explanatory	beta	HR	L95	U95	p
Low	-0.81	0.45	0.24	0.82	0.01
High	0.61	1.85	1.02	3.33	0.04

n= 394, number of events =84
Score(logrank) test = p <.0001

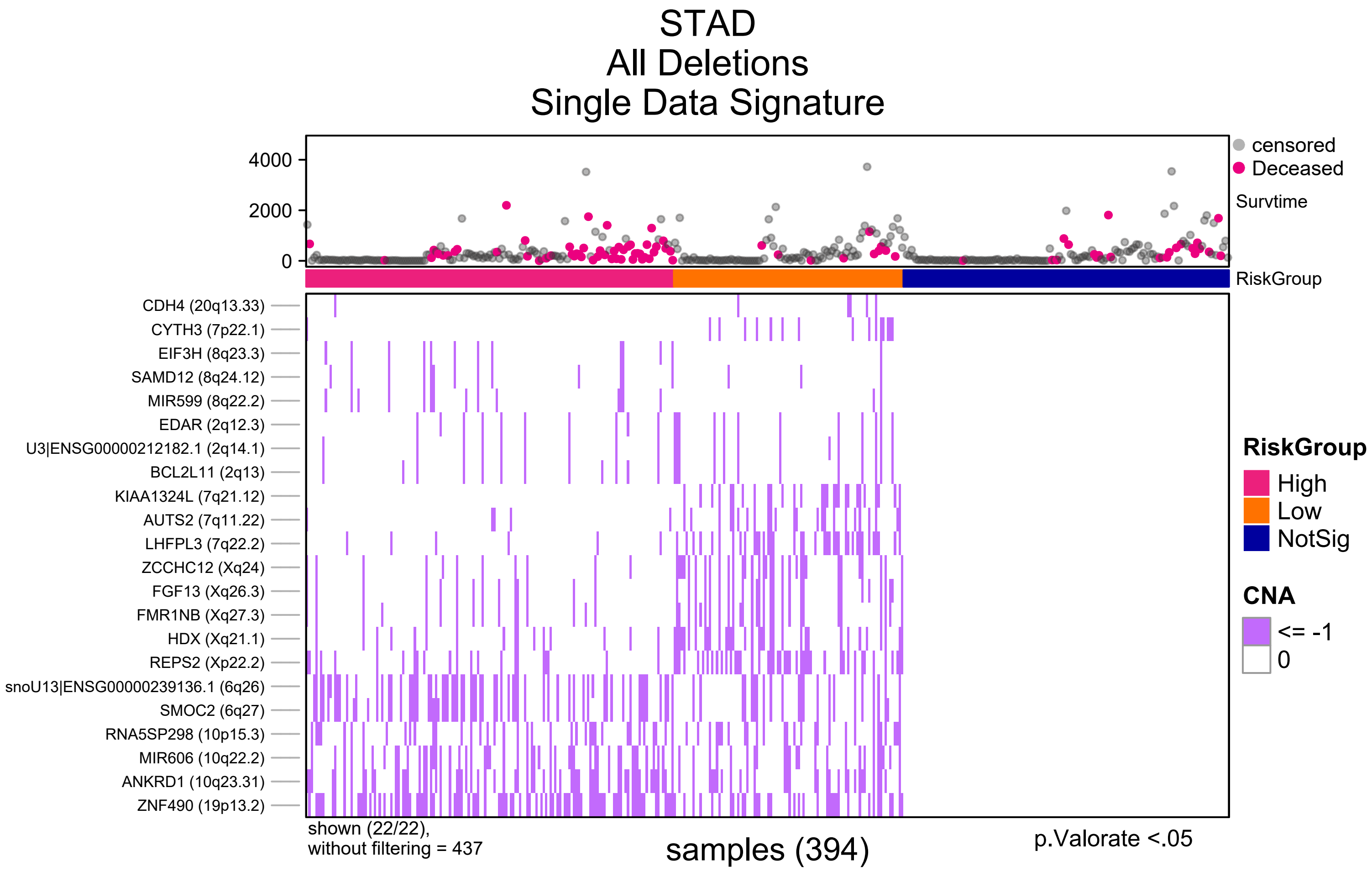

STAD
All Deletions
Single Data Signature
4000
2000
0
censored
Deceased
Survtime
RiskGroup
CDH4 (20q13.33)
CYTH3 (7p22.1)
EIF3H (8q23.3)
SAMD12 (8q24.12)
MIR599 (8q22.2)
EDAR (2q12.3)
U3|ENSG00000212182.1 (2q14.1)
BCL2L11 (2q13)
KIAA1324L (7q21.12)
AUTS2 (7q11.22)
LHFPL3 (7q22.2)
ZCCHC12 (Xq24)
FGF13 (Xq26.3)
FMR1NB (Xq27.3)
HDX (Xq21.1)
REPS2 (Xp22.2)
snoU13|ENSG00000239136.1 (6q26)
SMOC2 (6q27)
RNA5SP298 (10p15.3)
MIR606 (10q22.2)
ANKRD1 (10q23.31)
ZNF490 (19p13.2)
RiskGroup
High
Low
NotSig
CNA
<= -1
0
shown (22/22),
without filtering = 437
samples (394)
p.Valorate <.05

STAD
All Deletions
Single Data Signature

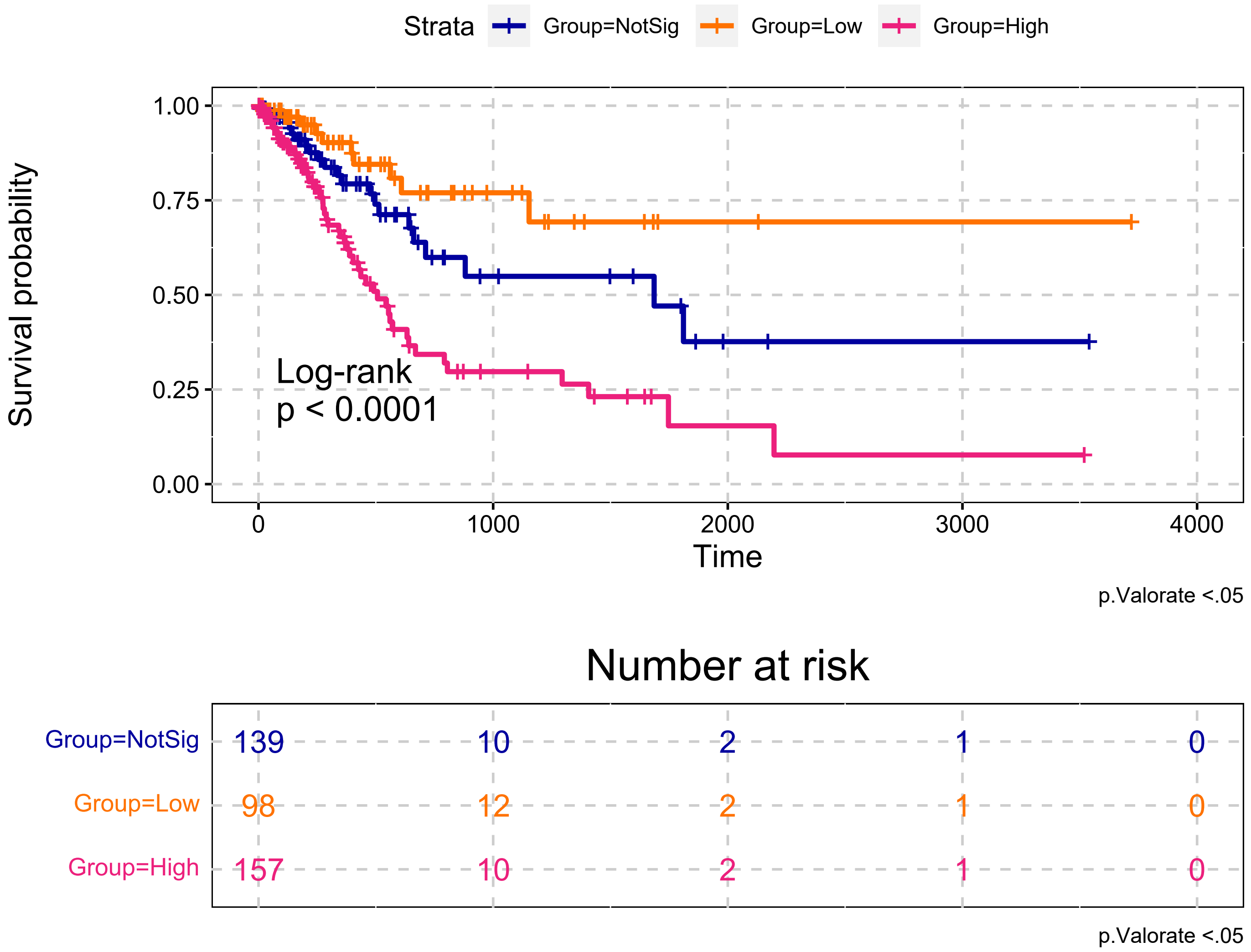

explanatory	beta	HR	L95	U95	p
Low	-0.64	0.52	0.25	1.11	0.09
High	0.77	2.16	1.31	3.57	0.00

n= 394, number of events =84
Score(logrank) test = p <.0001

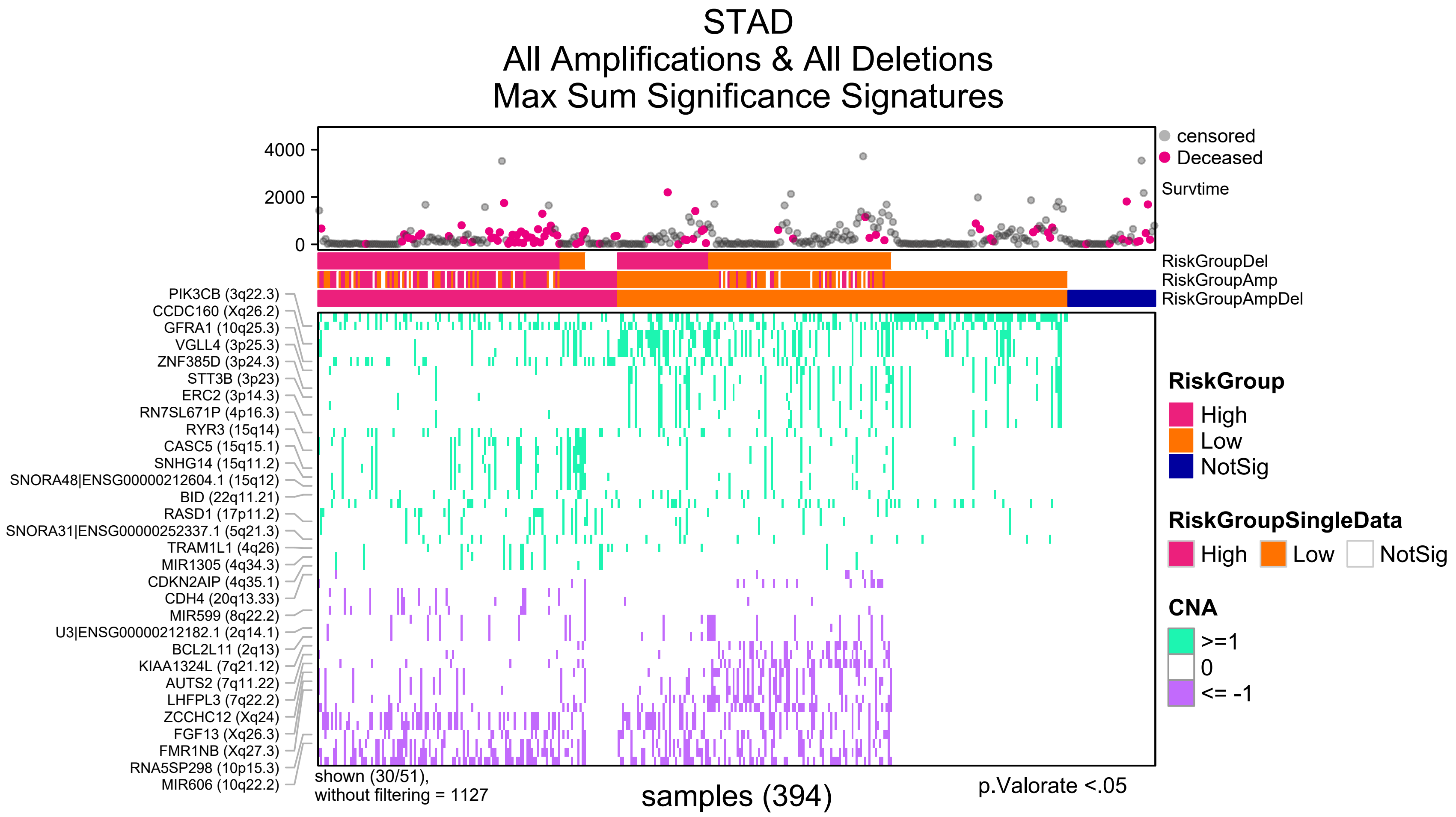

STAD
All Amplifications & All Deletions
Max Sum Significance Signatures
4000
2000
0
censored
Deceased
Survtime
RiskGroupDel
RiskGroupAmp
RiskGroupAmpDel
PIK3CB (3q22.3)
CCDC160 (Xq26.2)
GFRA1 (10q25.3)
VGLL4 (3p25.3)
ZNF385D (3p24.3)
STT3B (3p23)
ERC2 (3p14.3)
RN7SL671P (4p16.3)
RYR3 (15q14)
CASC5 (15q15.1)
SNHG14 (15q11.2)
SNORA48|ENSG00000212604.1 (15q12)
BID (22q11.21)
RASD1 (17p11.2)
SNORA31|ENSG00000252337.1 (5q21.3)
TRAM1L1 (4q26)
MIR1305 (4q34.3)
CDKN2AIP (4q35.1)
CDH4 (20q13.33)
MIR599 (8q22.2)
U3|ENSG00000212182.1 (2q14.1)
BCL2L11 (2q13)
KIAA1324L (7q21.12)
AUTS2 (7q11.22)
LHFPL3 (7q22.2)
ZCCHC12 (Xq24)
FGF13 (Xq26.3)
FMR1NB (Xq27.3)
RNA5SP298 (10p15.3)
MIR606 (10q22.2)
RiskGroup
High
Low
NotSig
RiskGroupSingleData
High
Low
NotSig
CNA
>=1
0
<= -1
shown (30/51),
without filtering = 1127
samples (394)
p.Valorate <.05

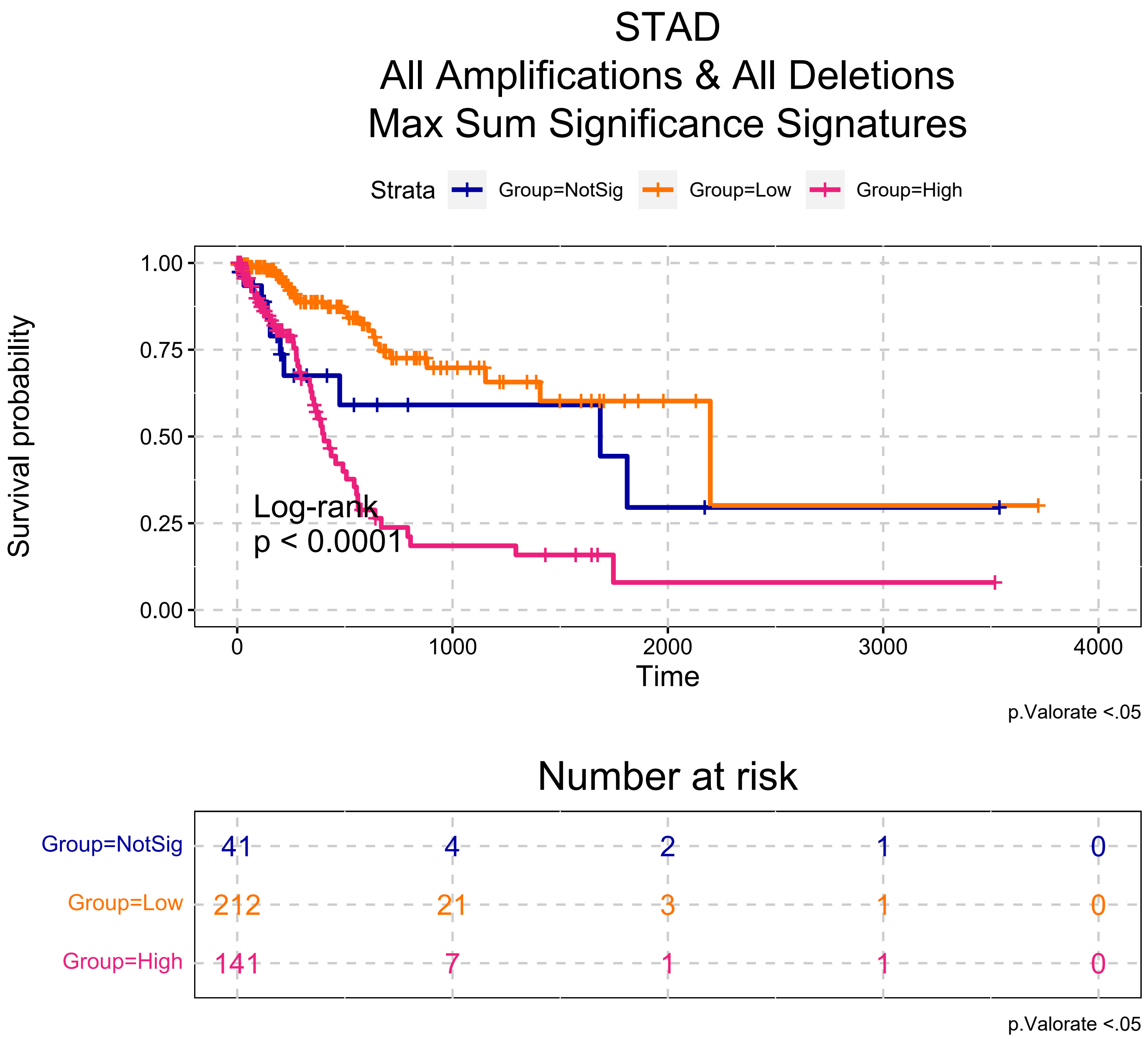

explanatory	beta	HR	L95	U95	p
Low	-0.90	0.41	0.19	0.86	0.02
High	0.58	1.79	0.90	3.58	0.10

n= 394, number of events =84
Score(logrank) test = p <.0001

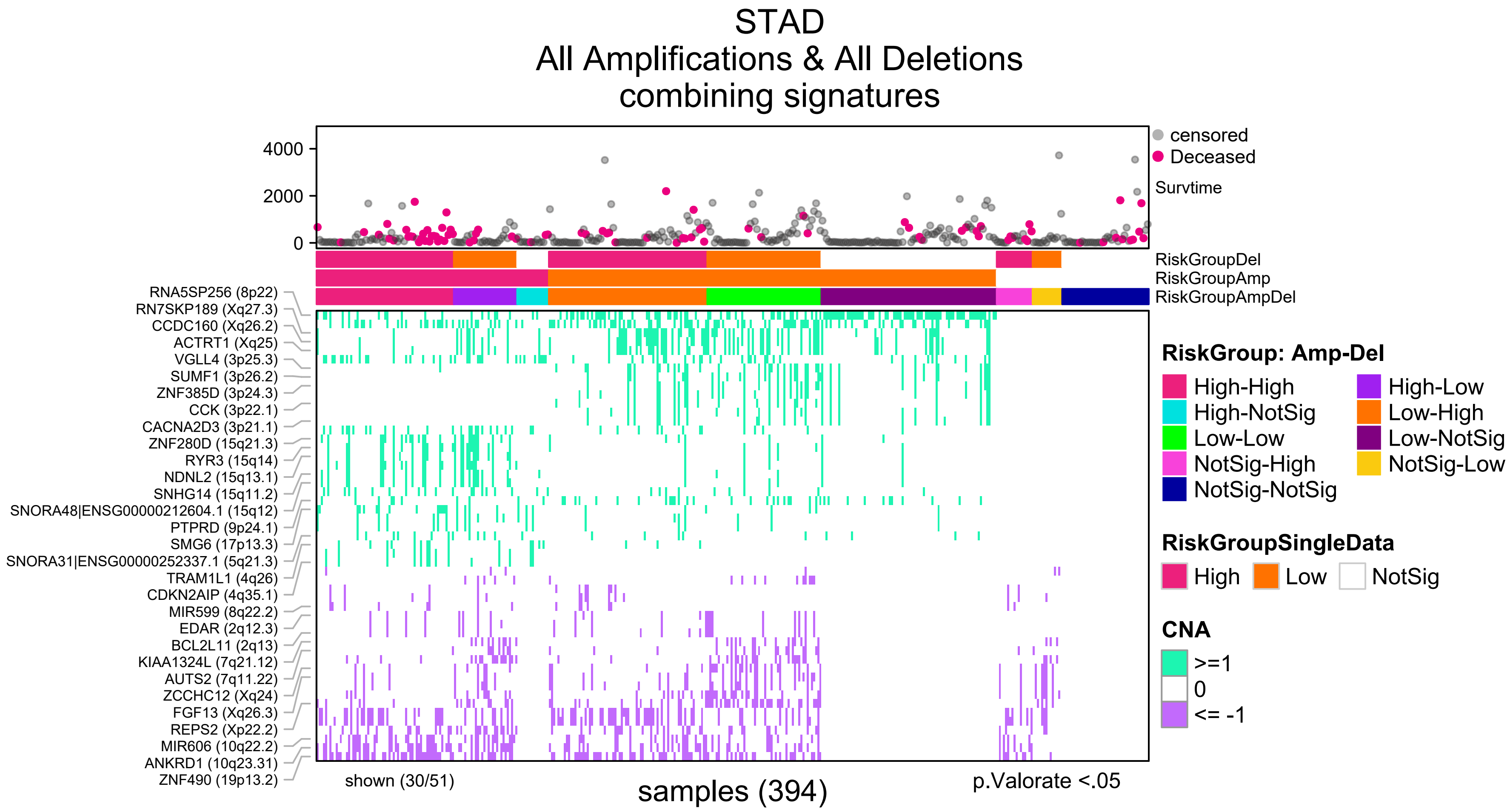
STAD
All Amplifications & All Deletions
combining signatures
4000
2000
0
censored
Deceased
Survtime
RiskGroupDel
RiskGroupAmp
RiskGroupAmpDel
RNA5SP256 (8p22)
RN7SKP189 (Xq27.3)
CCDC160 (Xq26.2)
ACTRT1 (Xq25)
VGLL4 (3p25.3)
SUMF1 (3p26.2)
ZNF385D (3p24.3)
CCK (3p22.1)
CACNA2D3 (3p21.1)
ZNF280D (15q21.3)
RYR3 (15q14)
NDNL2 (15q13.1)
SNHG14 (15q11.2)
SNORA48|ENSG00000212604.1 (15q12)
PTPRD (9p24.1)
SMG6 (17p13.3)
SNORA31|ENSG00000252337.1 (5q21.3)
TRAM1L1 (4q26)
CDKN2AIP (4q35.1)
MIR599 (8q22.2)
EDAR (2q12.3)
BCL2L11 (2q13)
KIAA1324L (7q21.12)
AUTS2 (7q11.22)
ZCCHC12 (Xq24)
FGF13 (Xq26.3)
REPS2 (Xp22.2)
MIR606 (10q22.2)
ANKRD1 (10q23.31)
ZNF490 (19p13.2)
RiskGroup: Amp-Del
High-High
High-NotSig
Low-Low
NotSig-High
NotSig-NotSig
High-Low
Low-High
Low-NotSig
NotSig-Low
RiskGroupSingleData
High
Low
NotSig
CNA
>=1
0
<= -1
shown (30/51)
samples (394)
p.Valorate <.05

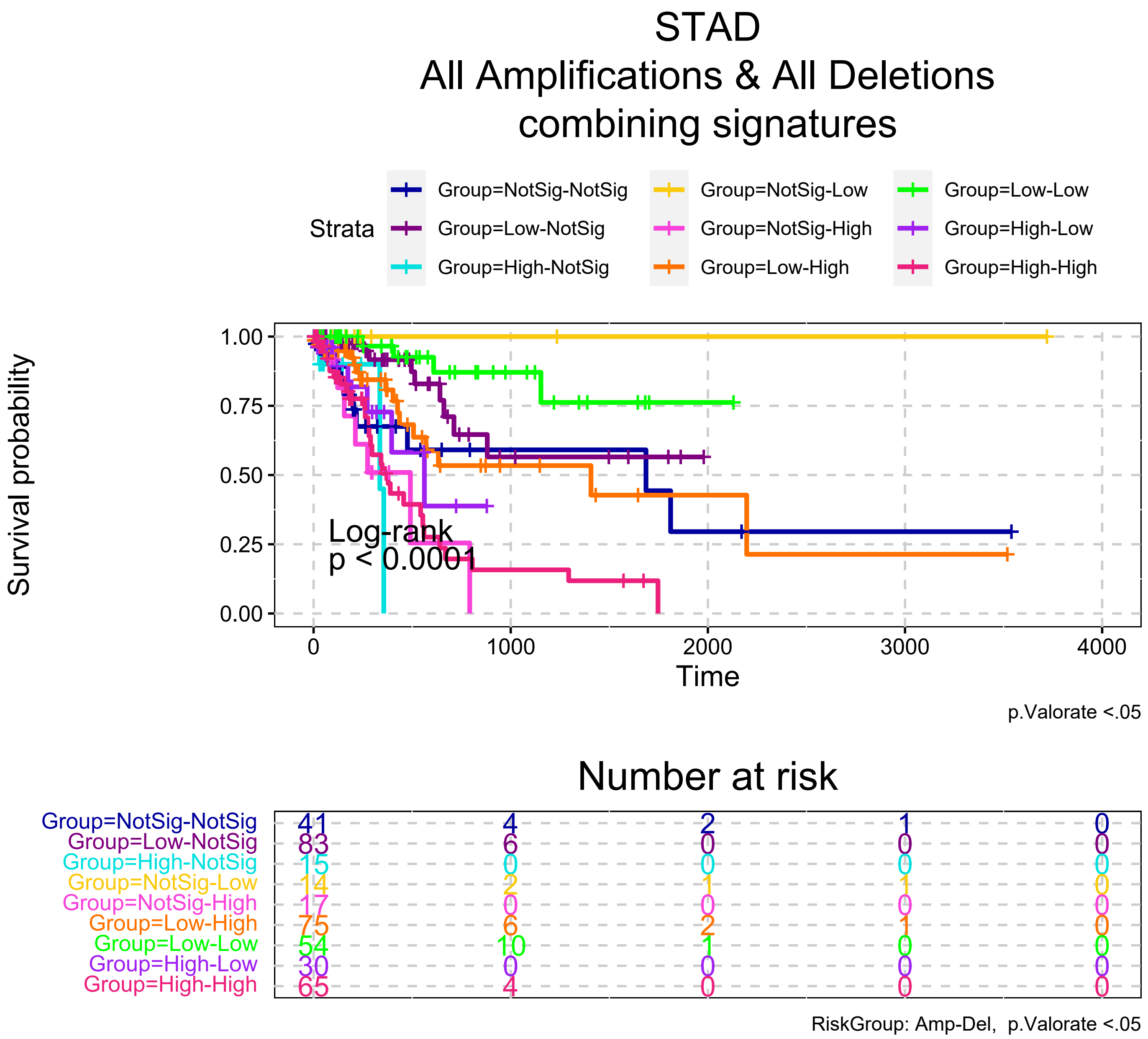

explanatory	beta	HR	L95	U95	p
Low-NotSig	-0.88	0.41	0.17	1.03	0.06
High-NotSig	1.15	3.14	0.83	11.85	0.09
NotSig-Low	-17.55	0.00	0.00	Inf	1.00
NotSig-High	0.97	2.63	0.98	7.09	0.06
Low-High	-0.18	0.83	0.38	1.85	0.66
Low-Low	-1.63	0.20	0.06	0.63	0.01
High-Low	0.26	1.30	0.46	3.66	0.62
High-High	0.77	2.16	1.03	4.53	0.04

n= 394, number of events =84
Score(logrank) test = p <.0001

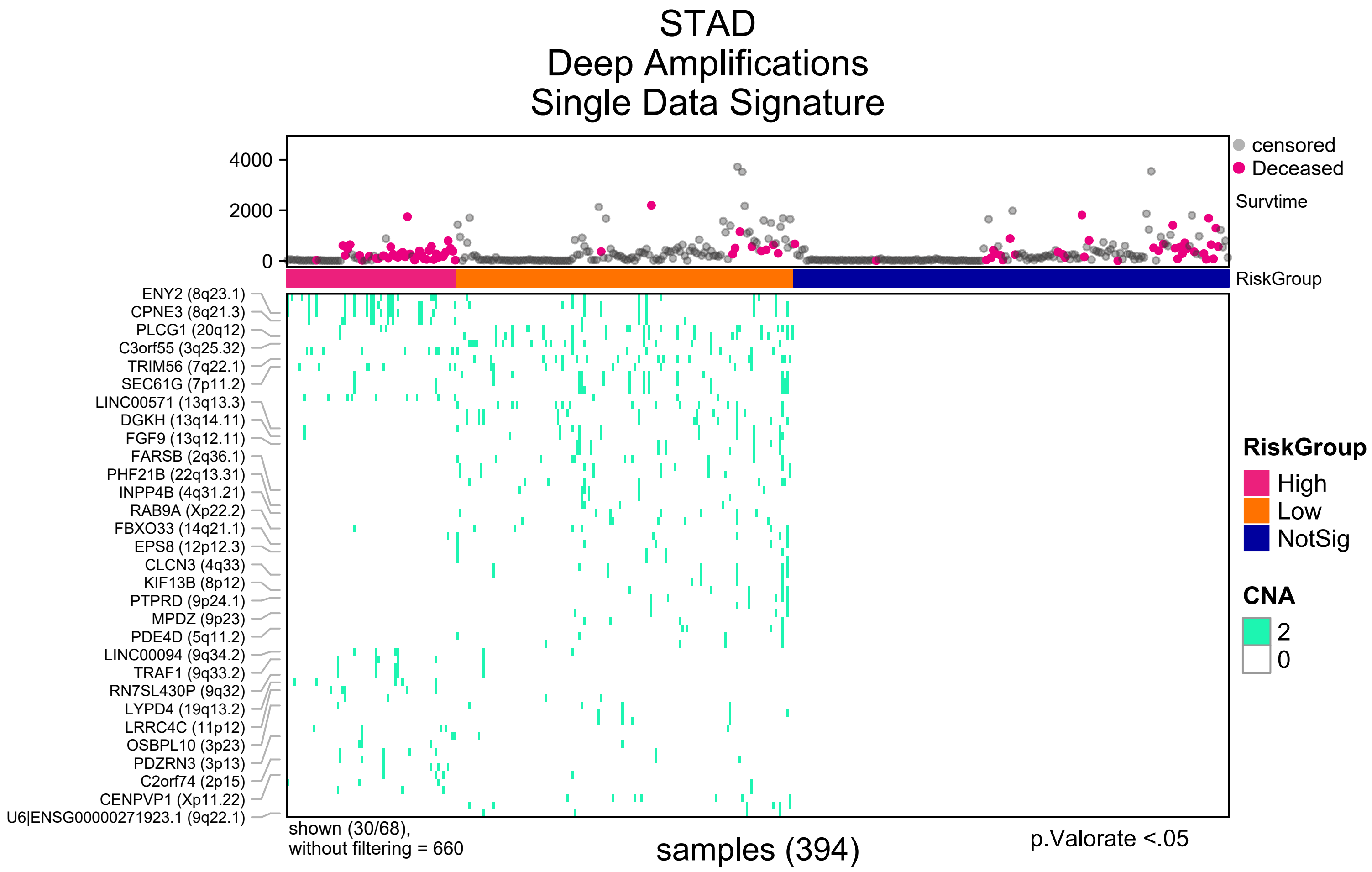

STAD
Deep Amplifications
Single Data Signature
4000
2000
0
censored
Deceased
Survtime
RiskGroup
ENY2 (8q23.1)
CPNE3 (8q21.3)
PLCG1 (20q12)
C3orf55 (3q25.32)
TRIM56 (7q22.1)
SEC61G (7p11.2)
LINC00571 (13q13.3)
DGKH (13q14.11)
FGF9 (13q12.11)
FARSB (2q36.1)
PHF21B (22q13.31)
INPP4B (4q31.21)
RAB9A (Xp22.2)
FBXO33 (14q21.1)
EPS8 (12p12.3)
CLCN3 (4q33)
KIF13B (8p12)
PTPRD (9p24.1)
MPDZ (9p23)
PDE4D (5q11.2)
LINC00094 (9q34.2)
TRAF1 (9q33.2)
RN7SL430P (9q32)
LYPD4 (19q13.2)
LRRC4C (11p12)
OSBPL10 (3p23)
PDZRN3 (3p13)
C2orf74 (2p15)
CENPVP1 (Xp11.22)
U6|ENSG00000271923.1 (9q22.1)
RiskGroup
High
Low
NotSig
CNA
2
0
shown (30/68),
without filtering = 660
samples (394)
p.Valorate <.05

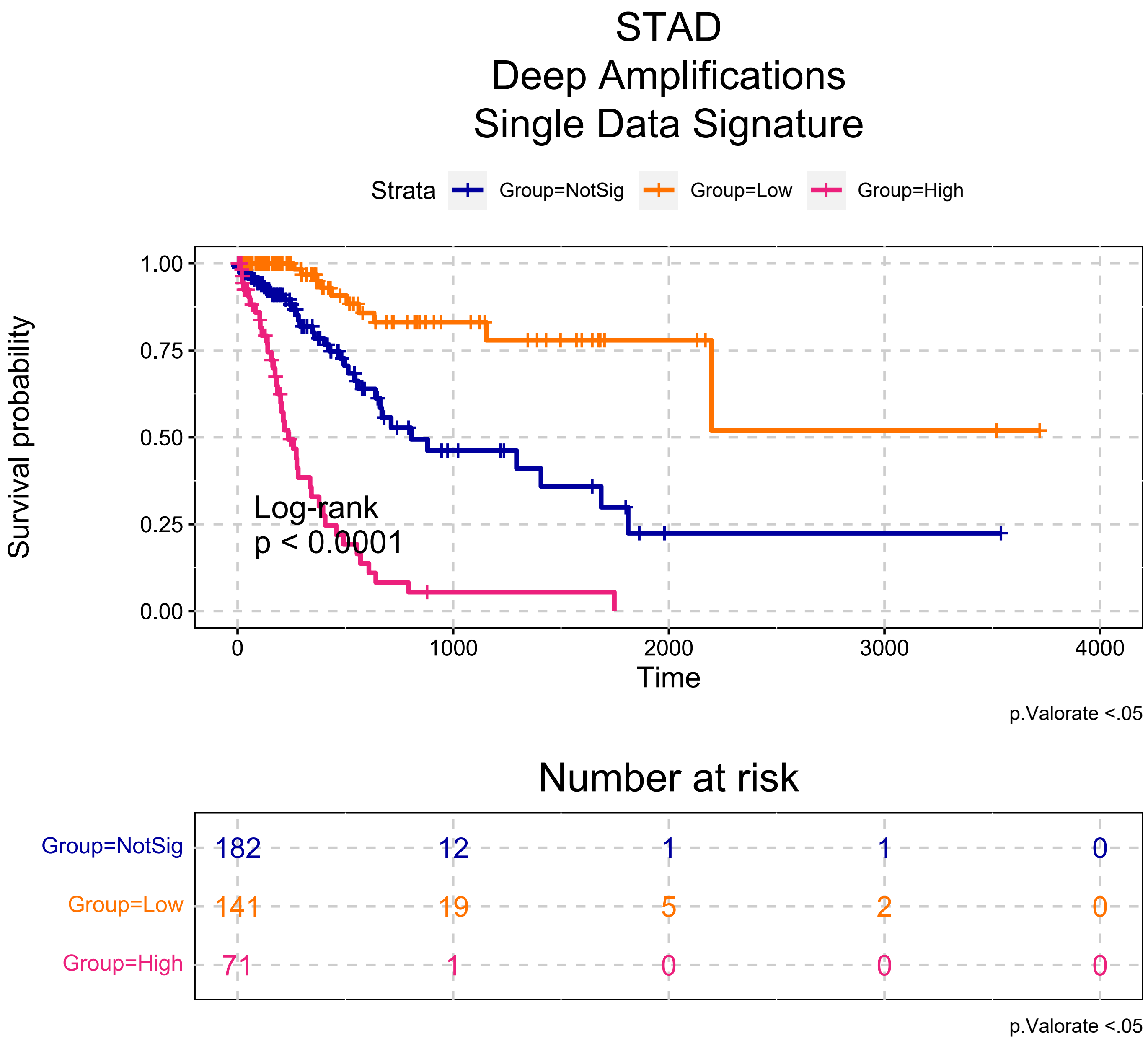

explanatory	beta	HR	L95	U95	p
Low	-1.40	0.25	0.12	0.50	0.00
High	1.45	4.26	2.67	6.79	0.00

n= 394, number of events =84
Score(logrank) test = p <.0001

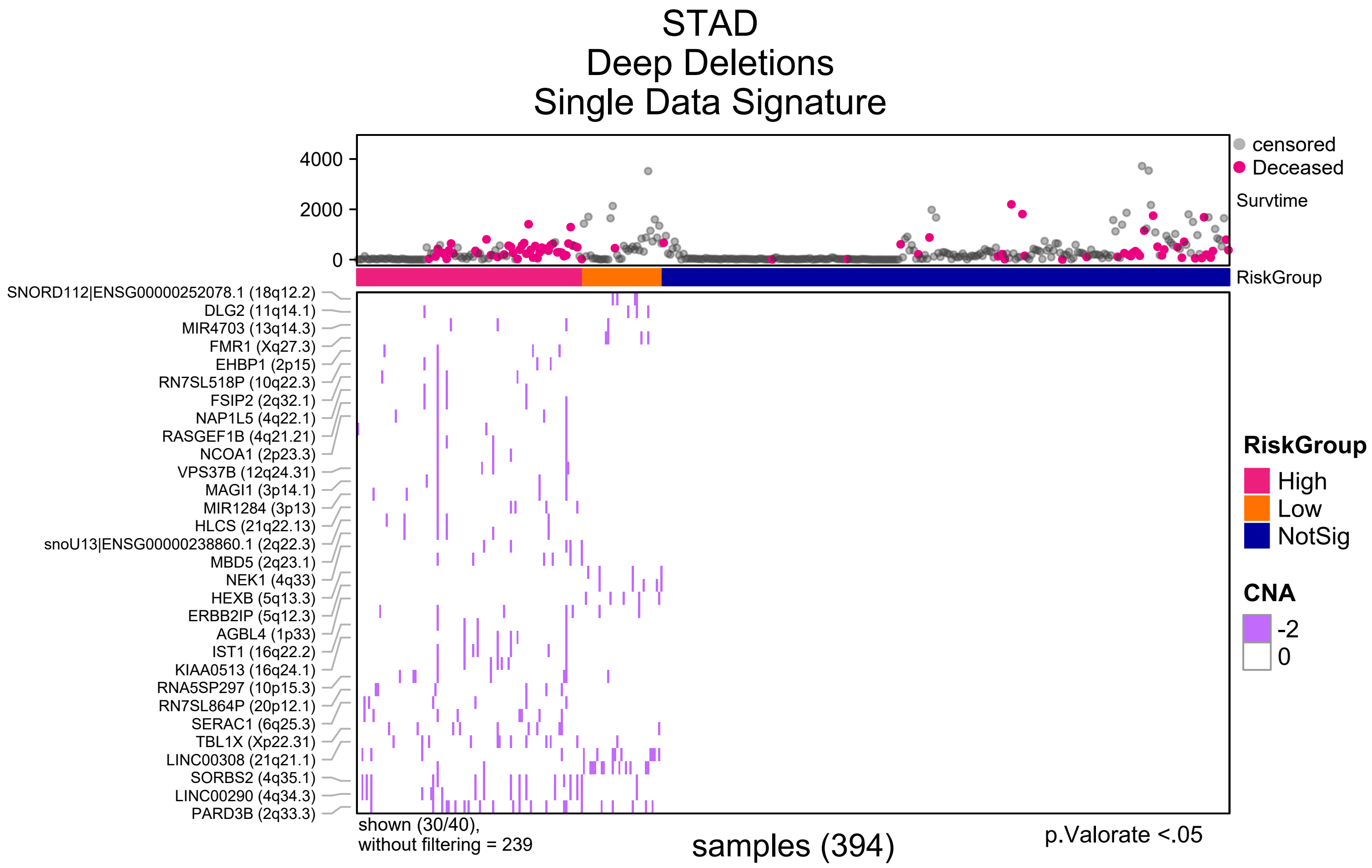
STAD
Deep Deletions
Single Data Signature
4000
2000
0
censored
Deceased
Survtime
RiskGroup
SNORD112|ENSG00000252078.1 (18q12.2)
DLG2 (11q14.1)
MIR4703 (13q14.3)
FMR1 (Xq27.3)
EHBP1 (2p15)
RN7SL518P (10q22.3)
FSIP2 (2q32.1)
NAP1L5 (4q22.1)
RASGEF1B (4q21.21)
NCOA1 (2p23.3)
VPS37B (12q24.31)
MAGI1 (3p14.1)
MIR1284 (3p13)
HLCS (21q22.13)
snoU13|ENSG00000238860.1 (2q22.3)
MBD5 (2q23.1)
NEK1 (4q33)
HEXB (5q13.3)
ERBB2IP (5q12.3)
AGBL4 (1p33)
IST1 (16q22.2)
KIAA0513 (16q24.1)
RNA5SP297 (10p15.3)
RN7SL864P (20p12.1)
SERAC1 (6q25.3)
TBL1X (Xp22.31)
LINC00308 (21q21.1)
SORBS2 (4q35.1)
LINC00290 (4q34.3)
PARD3B (2q33.3)
RiskGroup
High
Low
NotSig
CNA
-2
0
shown (30/40),
without filtering = 239
samples (394)
p.Valorate <.05

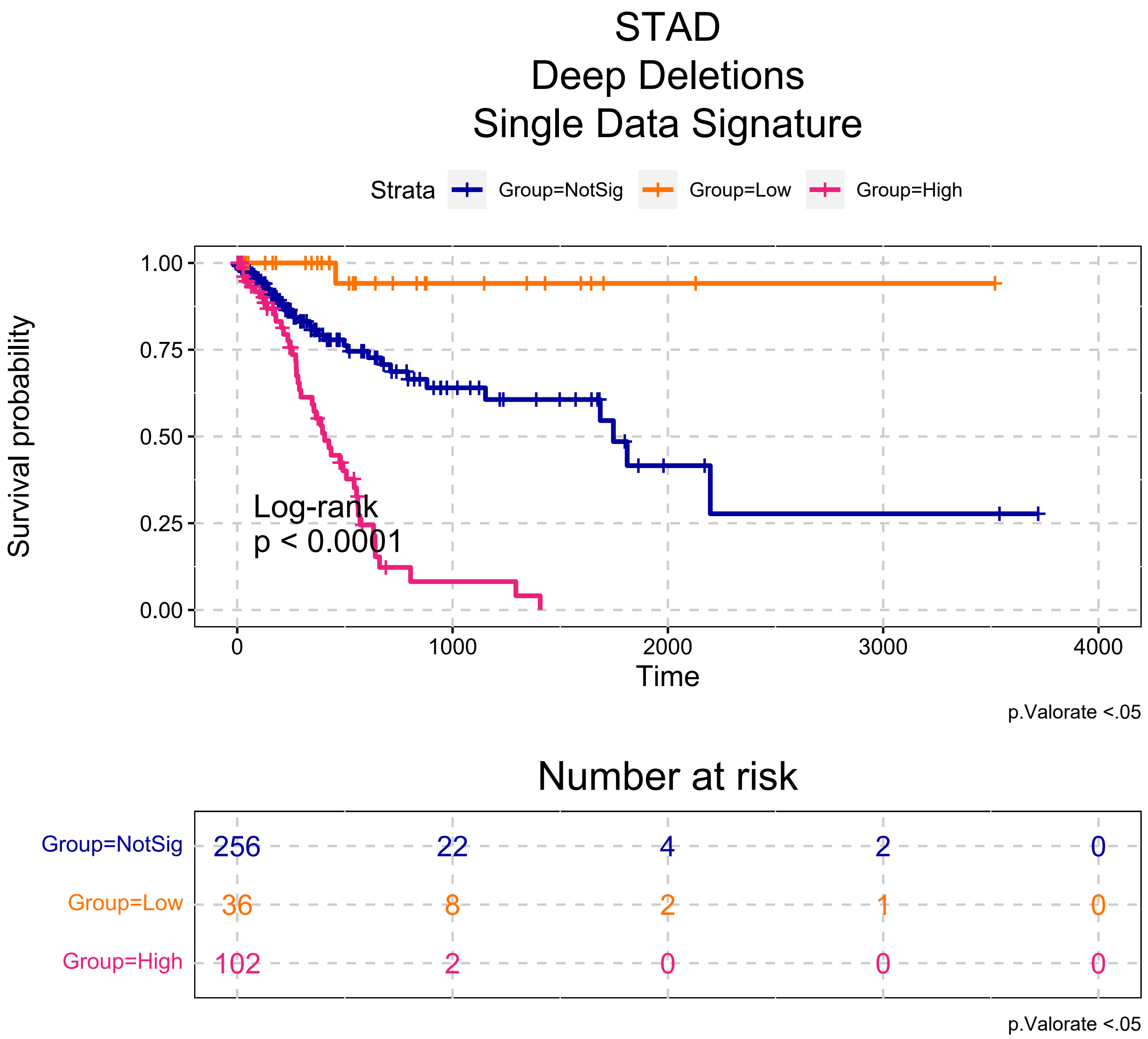

explanatory	beta	HR	L95	U95	p
Low	-2.38	0.09	0.01	0.67	0.02
High	1.34	3.84	2.43	6.06	0.00

n= 394, number of events =84
Score(logrank) test = p <.0001

STAD
Deep Amplifications & Deep Deletions
Max Sum Significance Signatures

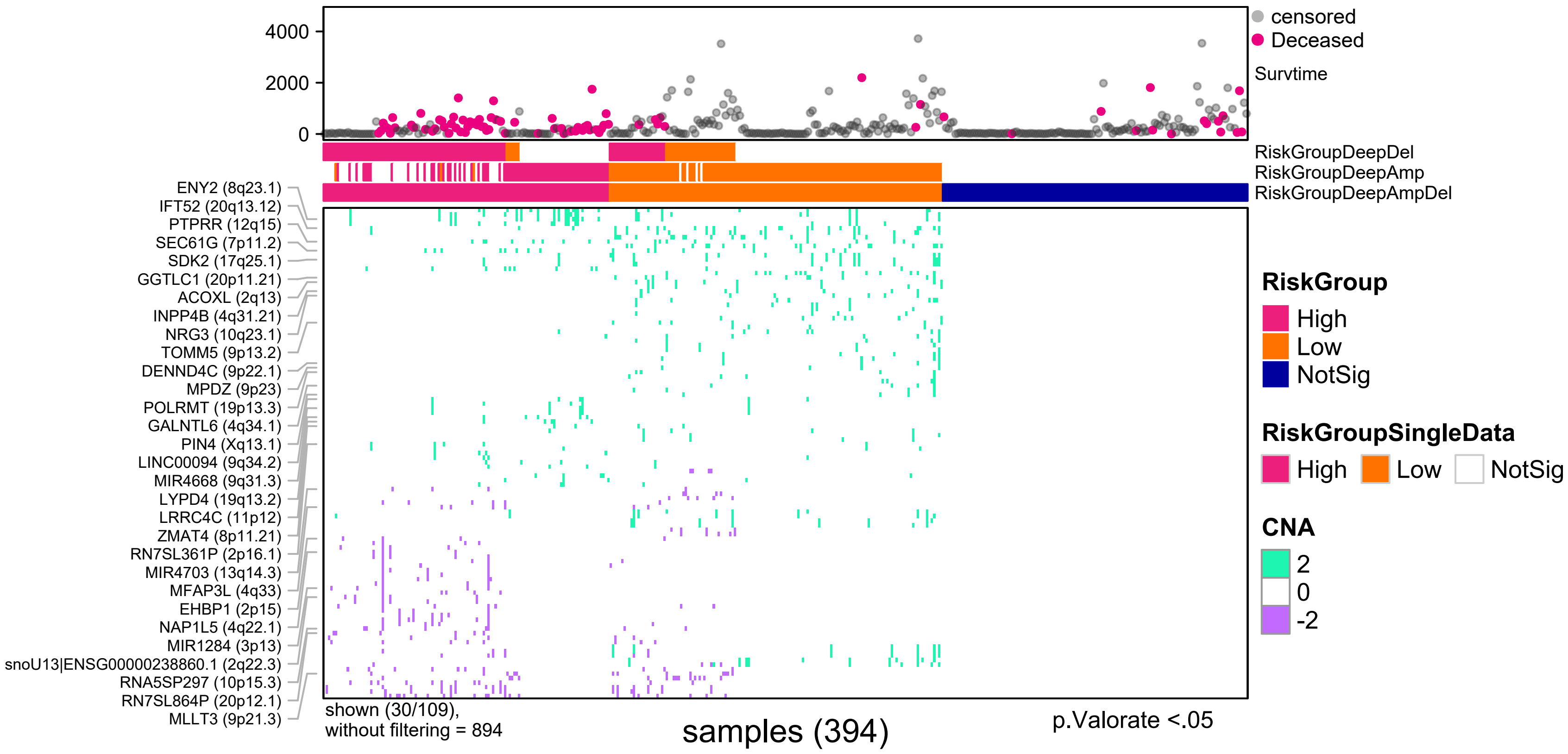

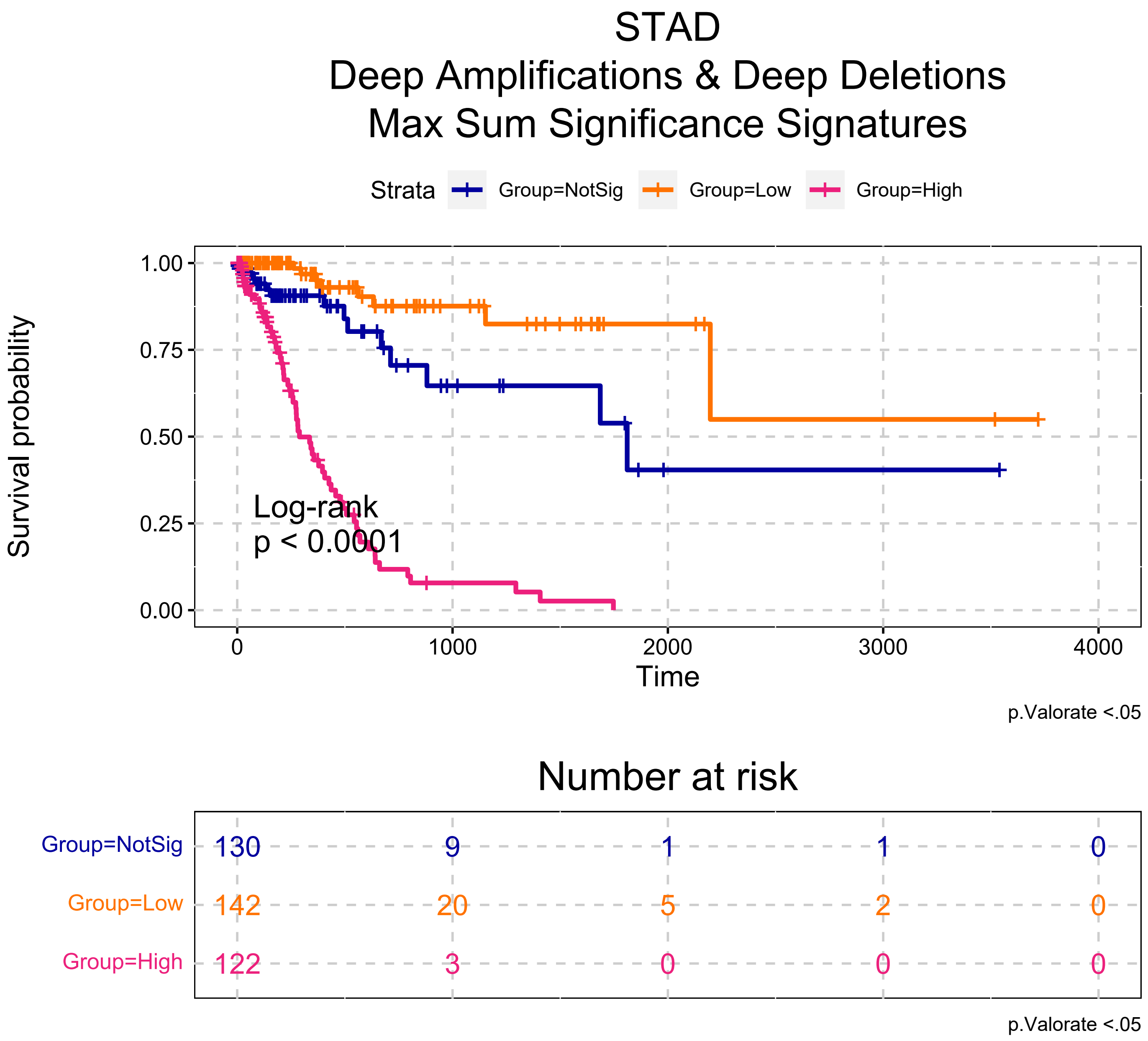

explanatory	beta	HR	L95	U95	p
Low	-1.13	0.32	0.14	0.76	0.01
High	1.78	5.94	3.30	10.67	0.00

n= 394, number of events =84
Score(logrank) test = p <.0001

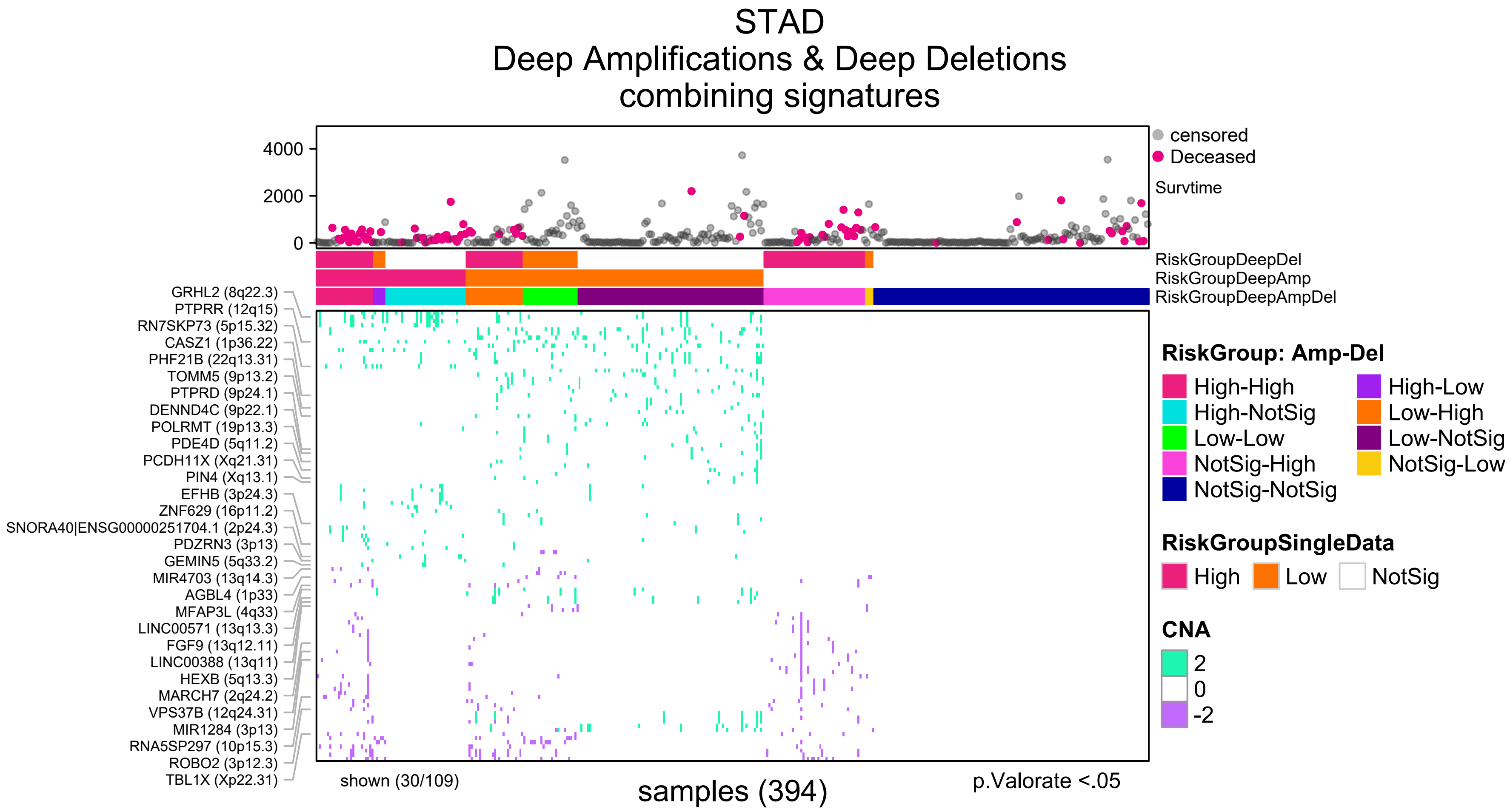
STAD
Deep Amplifications & Deep Deletions
combining signatures
4000
2000
0
censored
Deceased
Survtime
RiskGroupDeepDel
RiskGroupDeepAmp
RiskGroupDeepAmpDel
GRHL2 (8q22.3)
PTPRR (12q15)
RN7SKP73 (5p15.32)
CASZ1 (1p36.22)
PHF21B (22q13.31)
TOMM5 (9p13.2)
PTPRD (9p24.1)
DENND4C (9p22.1)
POLRMT (19p13.3)
PDE4D (5q11.2)
PCDH11X (Xq21.31)
PIN4 (Xq13.1)
EFHB (3p24.3)
ZNF629 (16p11.2)
SNORA40|ENSG00000251704.1 (2p24.3)
PDZRN3 (3p13)
GEMIN5 (5q33.2)
MIR4703 (13q14.3)
AGBL4 (1p33)
MFAP3L (4q33)
LINC00571 (13q13.3)
FGF9 (13q12.11)
LINC00388 (13q11)
HEXB (5q13.3)
MARCH7 (2q24.2)
VPS37B (12q24.31)
MIR1284 (3p13)
RNA5SP297 (10p15.3)
ROBO2 (3p12.3)
TBL1X (Xp22.31)
RiskGroup: Amp-Del
High-High
High-Low
High-NotSig
Low-High
Low-Low
Low-NotSig
NotSig-High
NotSig-Low
NotSig-NotSig
RiskGroupSingleData
High
Low
NotSig
CNA
2
0
-2
shown (30/109)
samples (394)
p.Valorate <.05

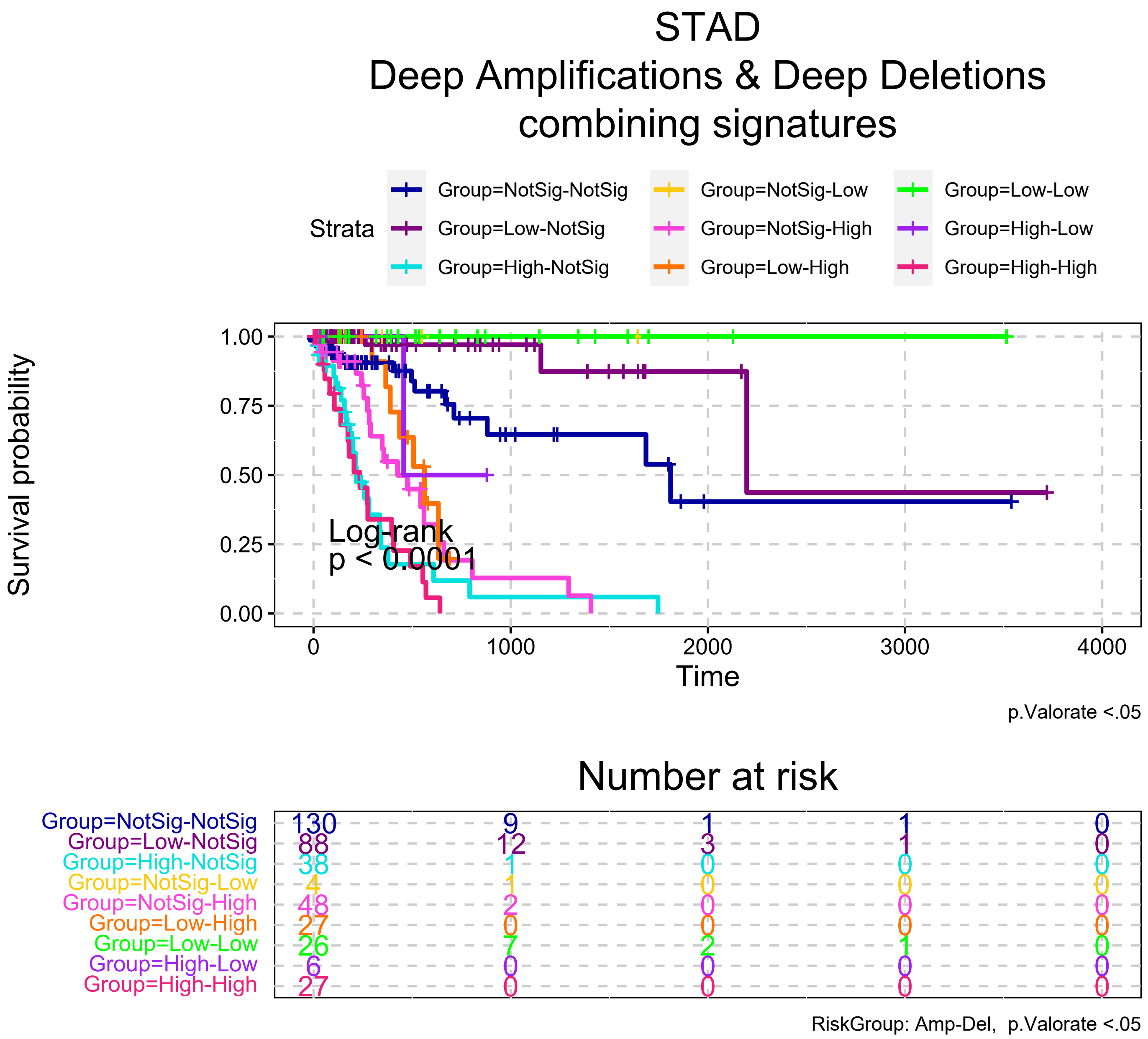

explanatory	beta	HR	L95	U95	p
Low-NotSig	-1.57	0.21	0.06	0.72	0.01
High-NotSig	2.07	7.95	3.97	15.91	0.00
NotSig-Low	-18.37	0.00	0.00	Inf	1.00
NotSig-High	1.49	4.45	2.22	8.93	0.00
Low-High	1.03	2.80	1.11	7.09	0.03
Low-Low	-18.26	0.00	0.00	Inf	1.00
High-Low	0.52	1.67	0.22	12.82	0.62
High-High	2.33	10.26	4.96	21.24	0.00

n= 394, number of events =84
Score(logrank) test = p <.0001
