# Supplementary material for: Systematic Modeling of Risk-Associated Copy Number Alterations in Cancer
Source: Int J Mol Sci. 2024 Sep 27;25(19):10455. doi: 10.3390/ijms251910455 (PMC11477427; doi:10.3390/ijms251910455)
Supplement: Supplementary file 1 [file ijms-25-10455-s001.zip › STESSignatureV12-sinSombreado.pdf]

STES
All Amplifications
Single Data Signature

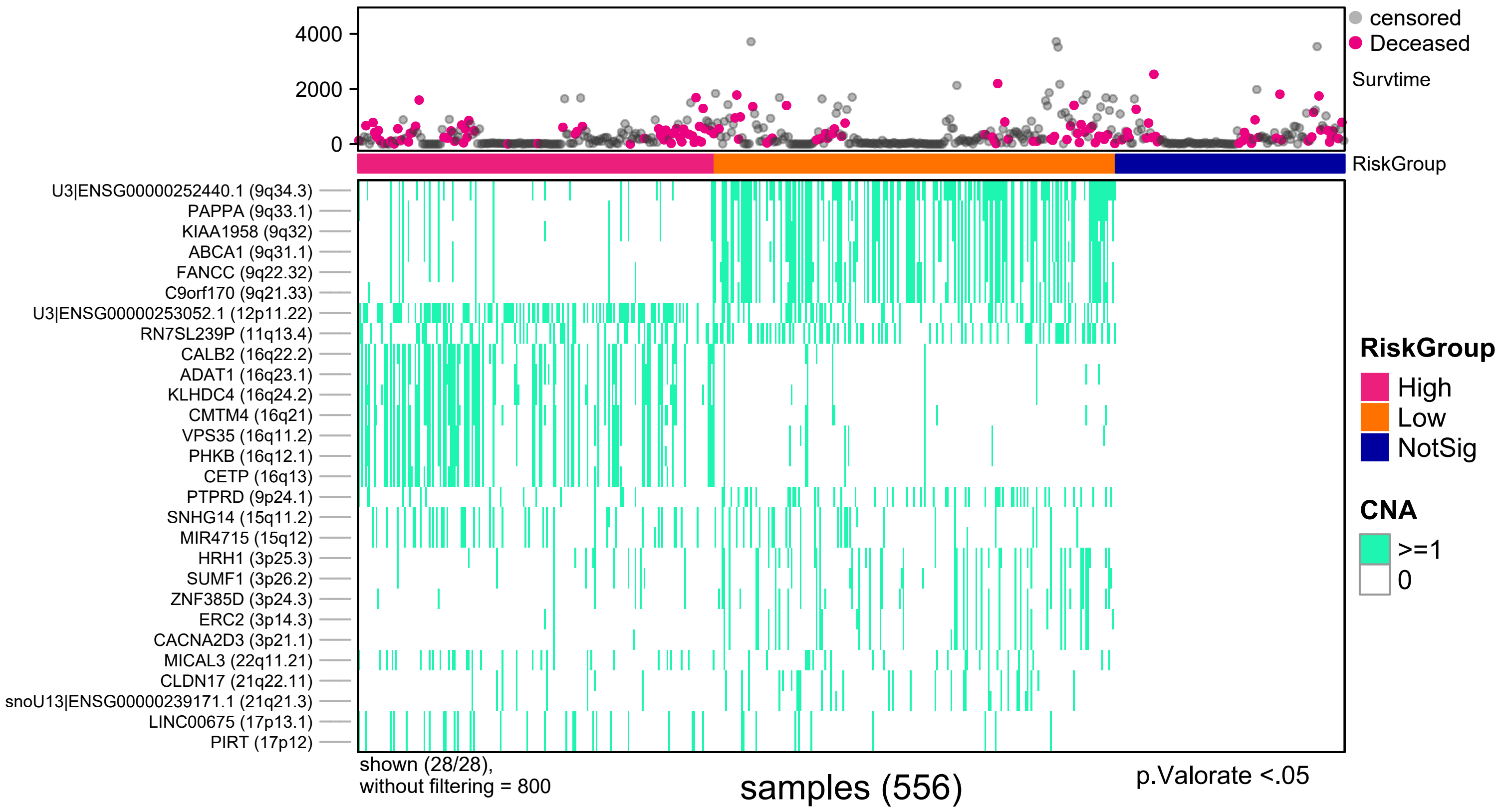

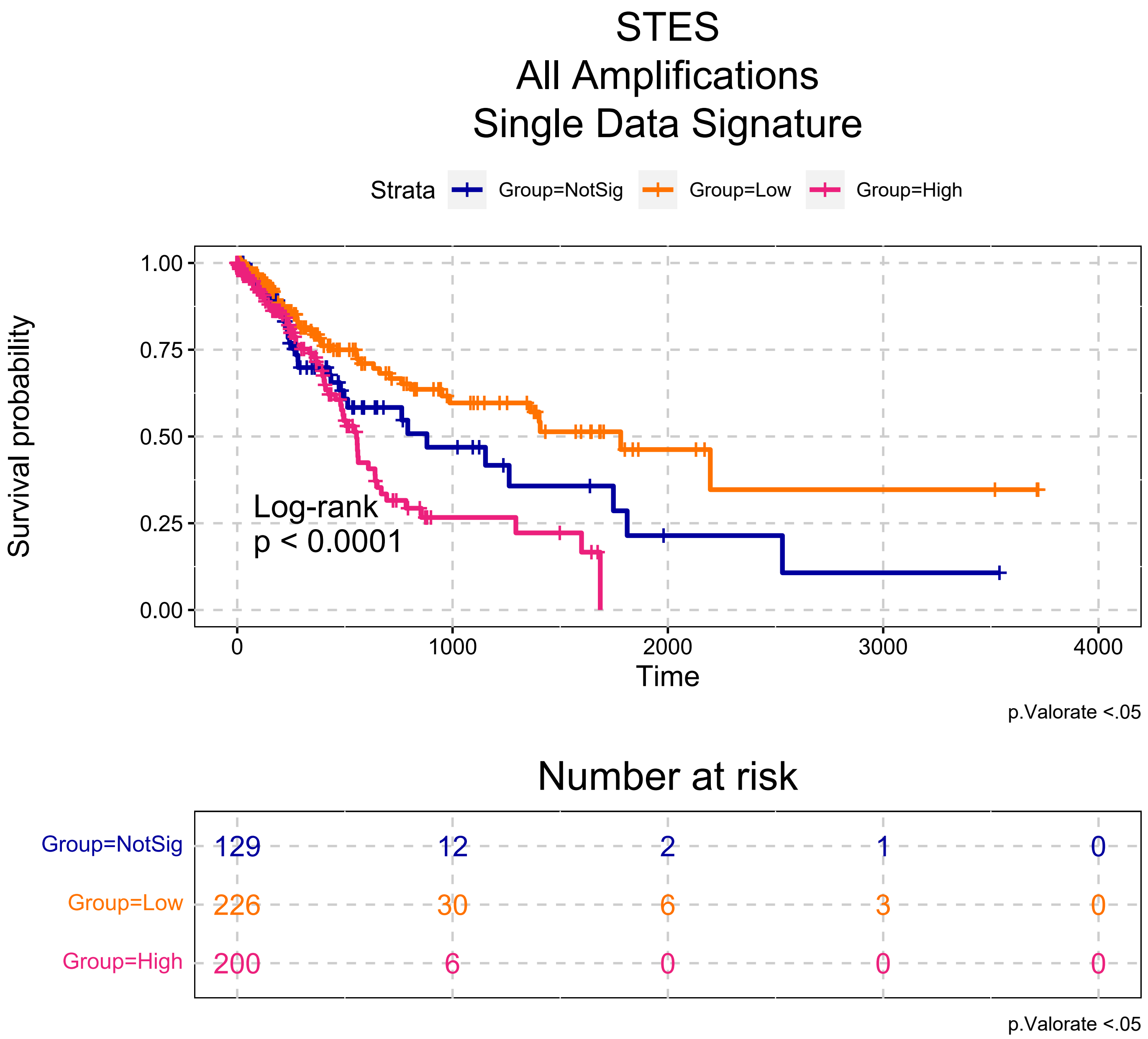

explanatory	beta	HR	L95	U95	p
Low	-0.48	0.62	0.40	0.97	0.03
High	0.37	1.45	0.95	2.22	0.09

n= 556, number of events =140
Score(logrank) test = p <.0001

STES
All Deletions
Single Data Signature

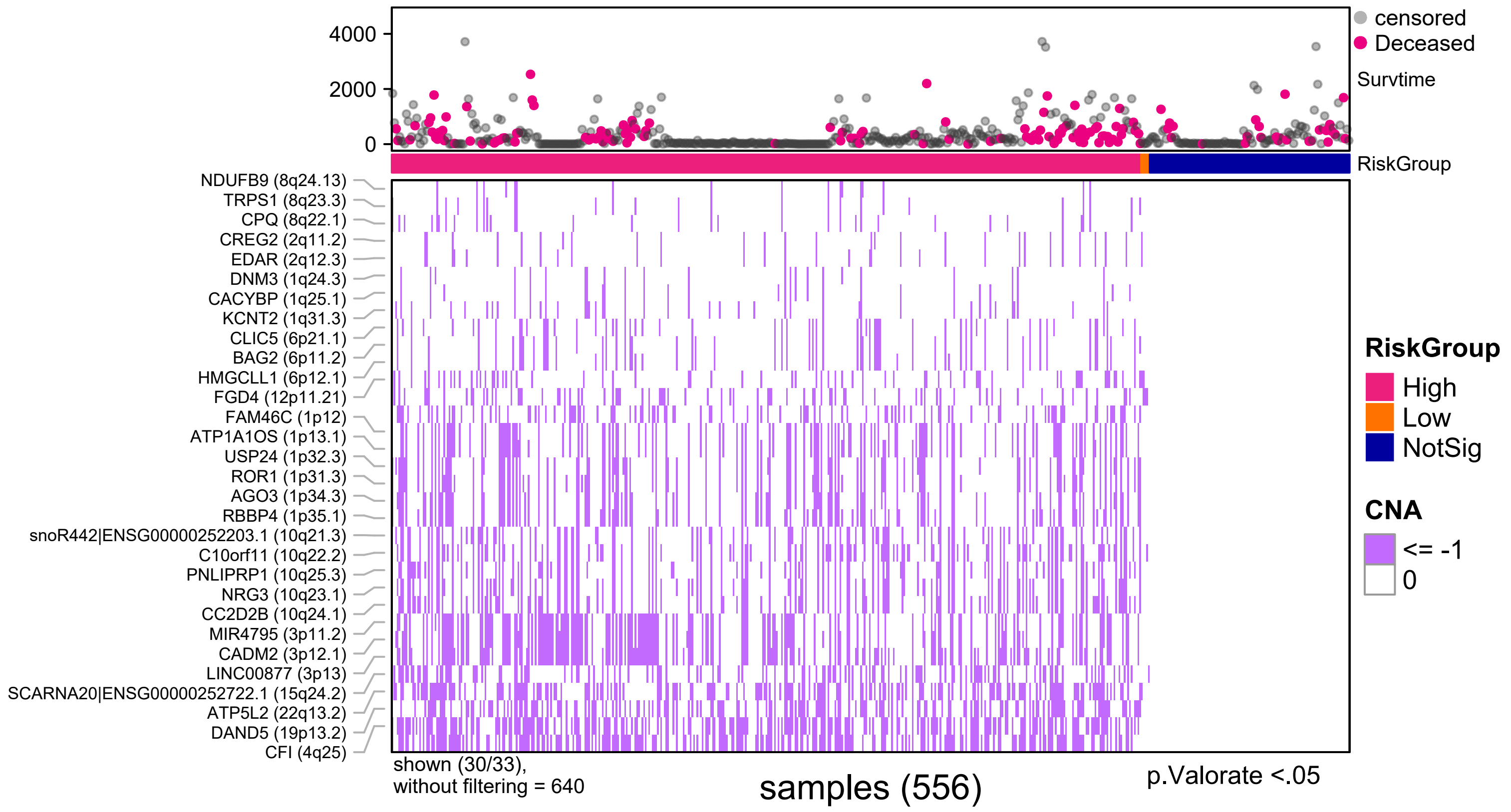

STES
All Deletions
Single Data Signature

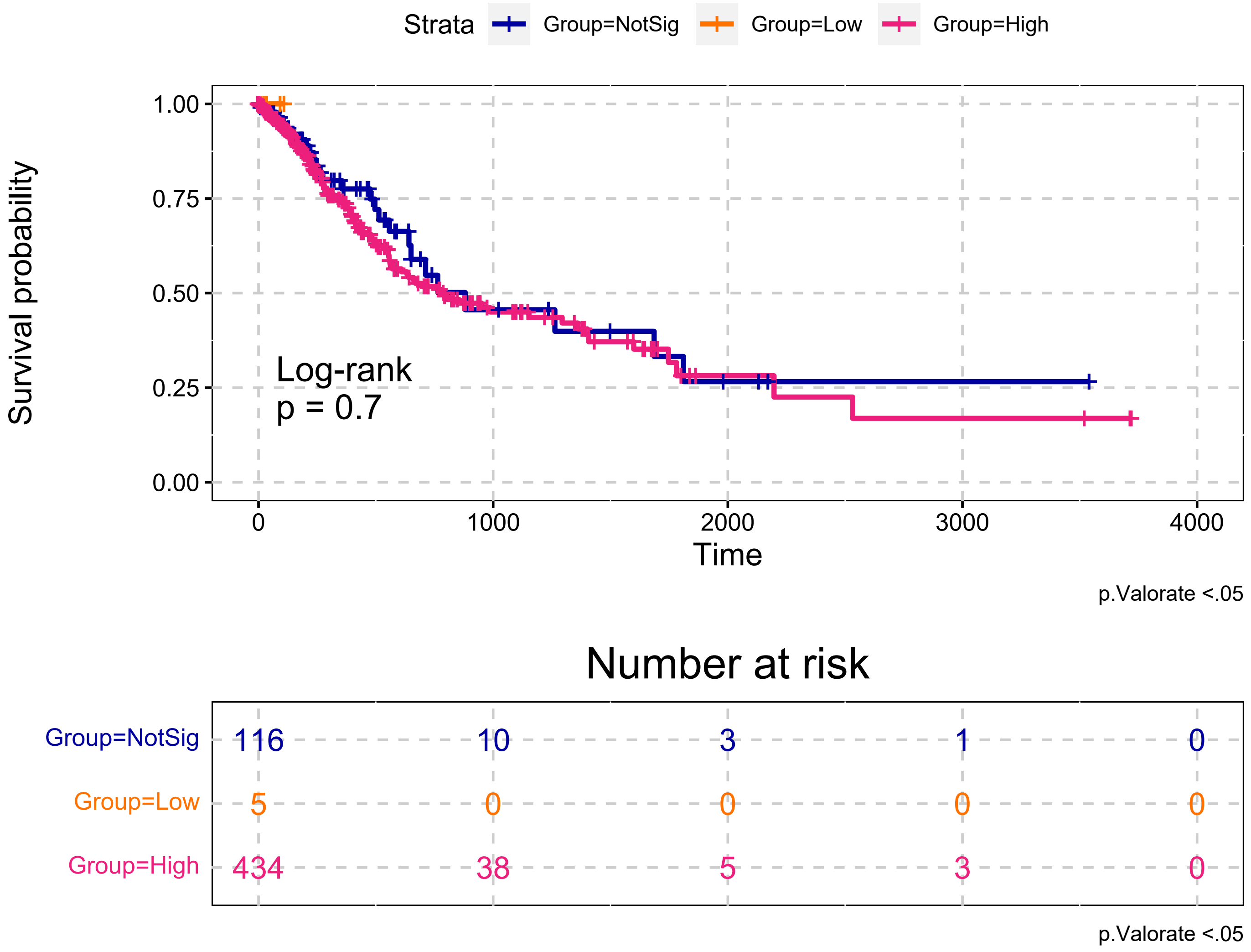

explanatory	beta	HR	L95	U95	p
Low	-12.88	0.00	0.00	Inf	0.99
High	0.16	1.17	0.77	1.80	0.46

n= 556, number of events =140
Score(logrank) test = 0.701

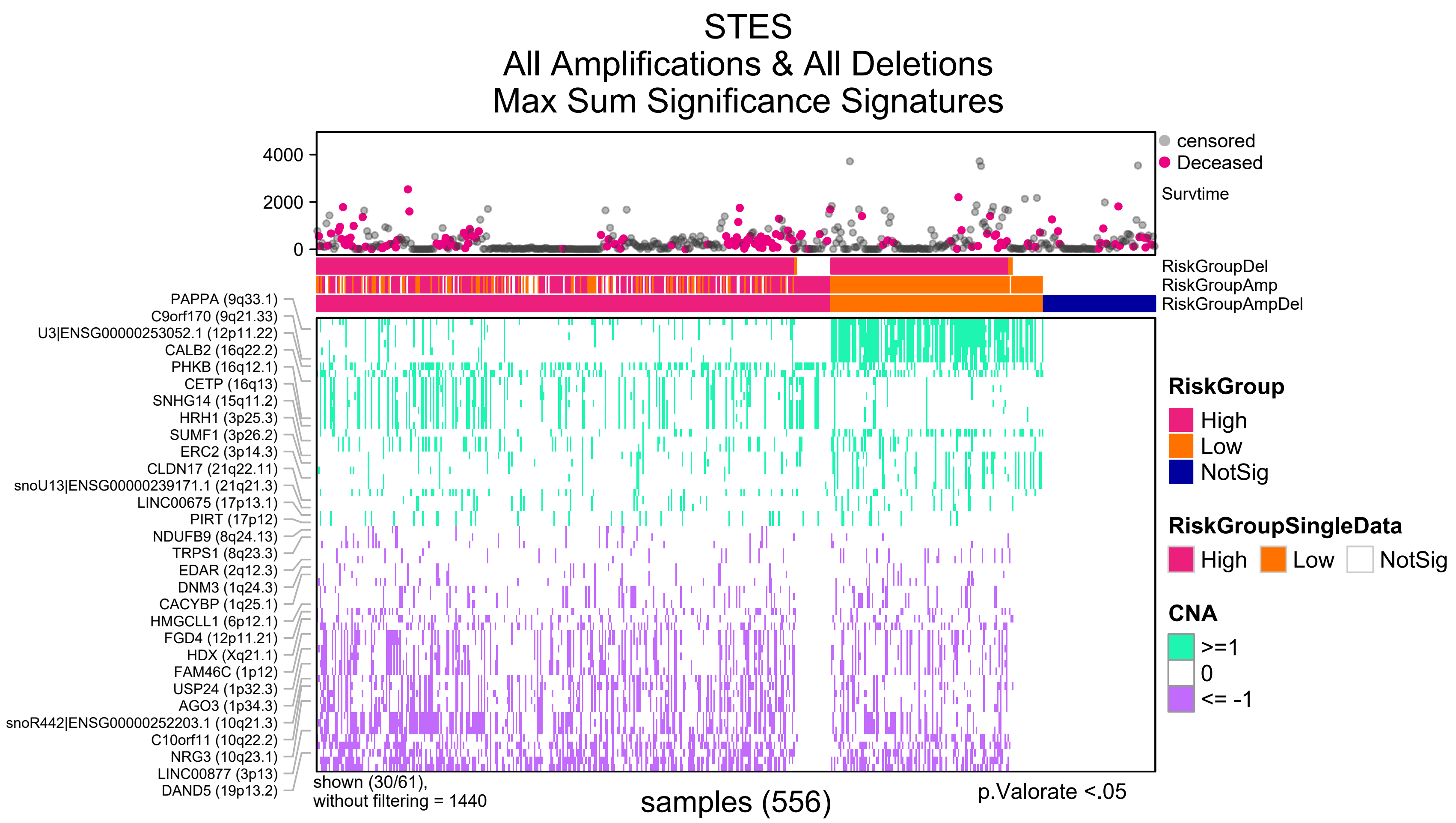
STES
All Amplifications & All Deletions
Max Sum Significance Signatures
4000
2000
0
censored
Deceased
Survtime
RiskGroupDel
RiskGroupAmp
RiskGroupAmpDel
PAPPA (9q33.1)
C9orf170 (9q21.33)
U3|ENSG00000253052.1 (12p11.22)
CALB2 (16q22.2)
PHKB (16q12.1)
CETP (16q13)
SNHG14 (15q11.2)
HRH1 (3p25.3)
SUMF1 (3p26.2)
ERC2 (3p14.3)
CLDN17 (21q22.11)
snoU13|ENSG00000239171.1 (21q21.3)
LINC00675 (17p13.1)
PIRT (17p12)
NDUFB9 (8q24.13)
TRPS1 (8q23.3)
EDAR (2q12.3)
DNM3 (1q24.3)
CACYBP (1q25.1)
HMGCLL1 (6p12.1)
FGD4 (12p11.21)
HDX (Xq21.1)
FAM46C (1p12)
USP24 (1p32.3)
AGO3 (1p34.3)
snoR442|ENSG00000252203.1 (10q21.3)
C10orf11 (10q22.2)
NRG3 (10q23.1)
LINC00877 (3p13)
DAND5 (19p13.2)
RiskGroup
High
Low
NotSig
RiskGroupSingleData
High
Low
NotSig
CNA
>=1
0
<= -1
shown (30/61),
without filtering = 1440
samples (556)
p.Valorate <.05

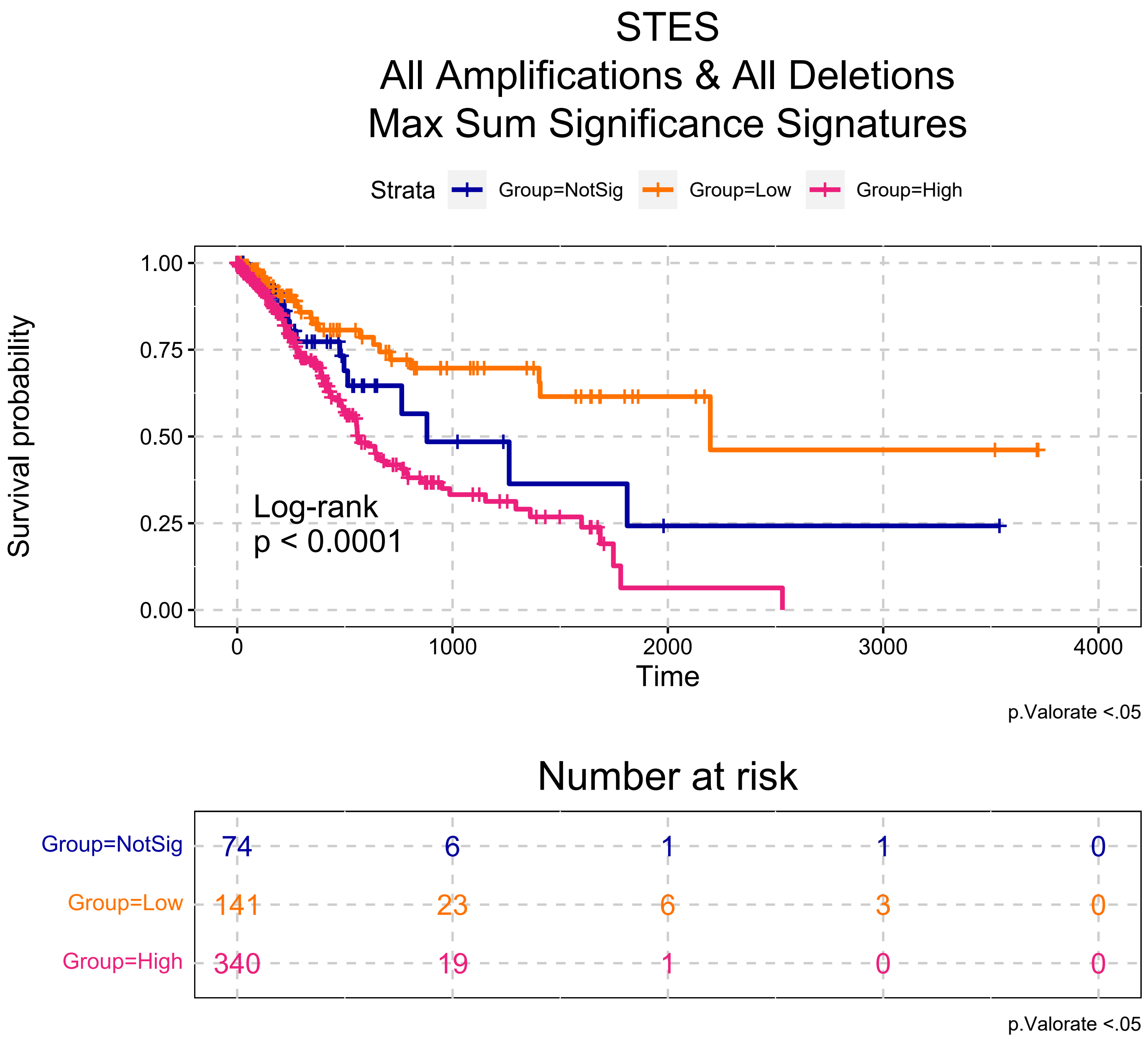

explanatory	beta	HR	L95	U95	p
Low	-0.61	0.54	0.28	1.03	0.06
High	0.47	1.60	0.94	2.73	0.08

n= 556, number of events =140
Score(logrank) test = p <.0001

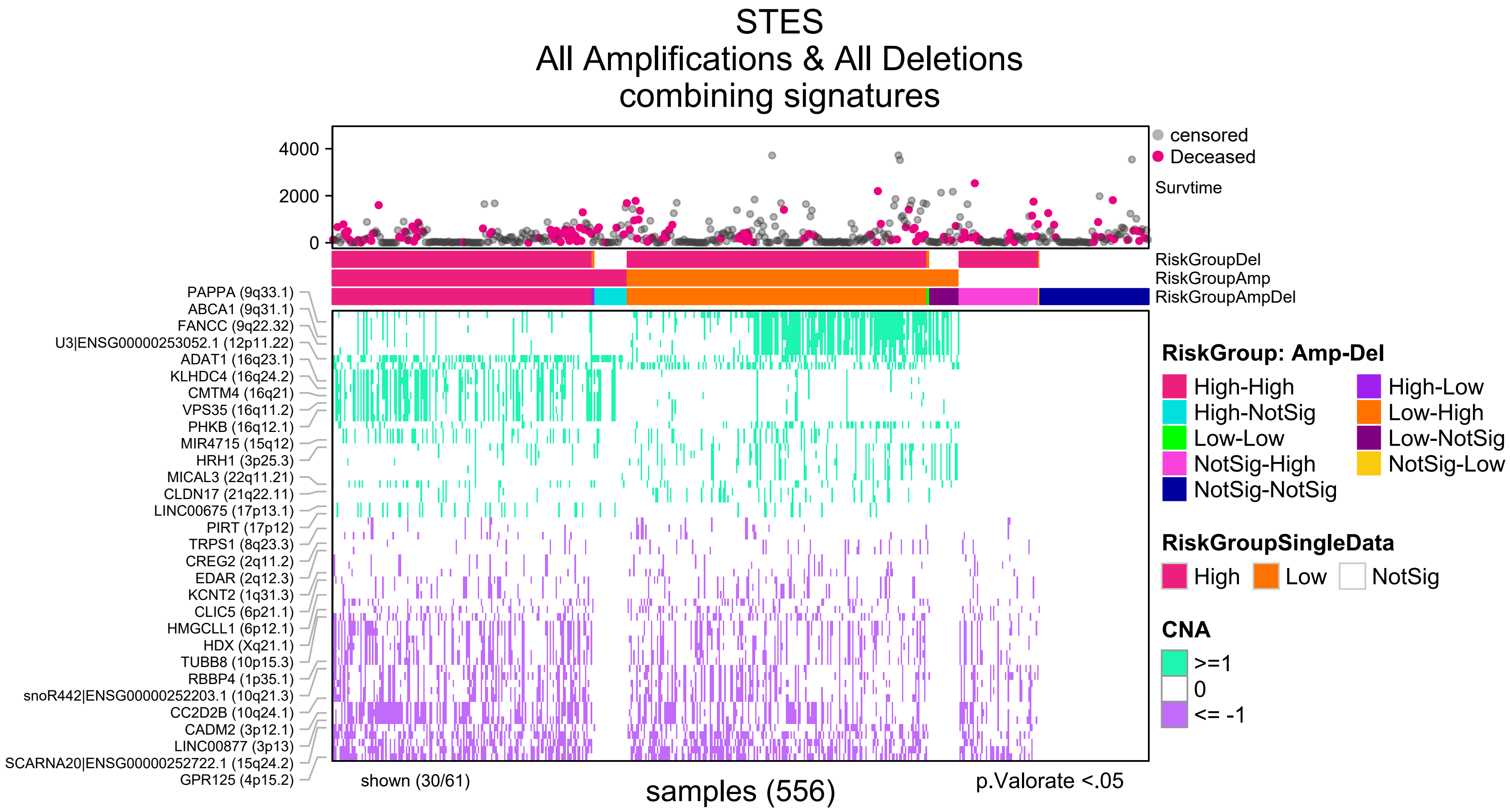

STES
All Amplifications & All Deletions
combining signatures
4000
2000
0
censored
Deceased
Survtime
RiskGroupDel
RiskGroupAmp
RiskGroupAmpDel
PAPPA (9q33.1)
ABCA1 (9q31.1)
FANCC (9q22.32)
U3|ENSG00000253052.1 (12p11.22)
ADAT1 (16q23.1)
KLHDC4 (16q24.2)
CMTM4 (16q21)
VPS35 (16q11.2)
PHKB (16q12.1)
MIR4715 (15q12)
HRH1 (3p25.3)
MICAL3 (22q11.21)
CLDN17 (21q22.11)
LINC00675 (17p13.1)
PIRT (17p12)
TRPS1 (8q23.3)
CREG2 (2q11.2)
EDAR (2q12.3)
KCNT2 (1q31.3)
CLIC5 (6p21.1)
HMGCLL1 (6p12.1)
HDX (Xq21.1)
TUBB8 (10p15.3)
RBBP4 (1p35.1)
snoR442|ENSG00000252203.1 (10q21.3)
CC2D2B (10q24.1)
CADM2 (3p12.1)
LINC00877 (3p13)
SCARNA20|ENSG00000252722.1 (15q24.2)
GPR125 (4p15.2)
RiskGroup: Amp-Del
High-High
High-NotSig
Low-Low
NotSig-High
NotSig-NotSig
High-Low
Low-High
Low-NotSig
NotSig-Low
RiskGroupSingleData
High
Low
NotSig
CNA
>=1
0
<= -1
shown (30/61)
samples (556)
p.Valorate <.05

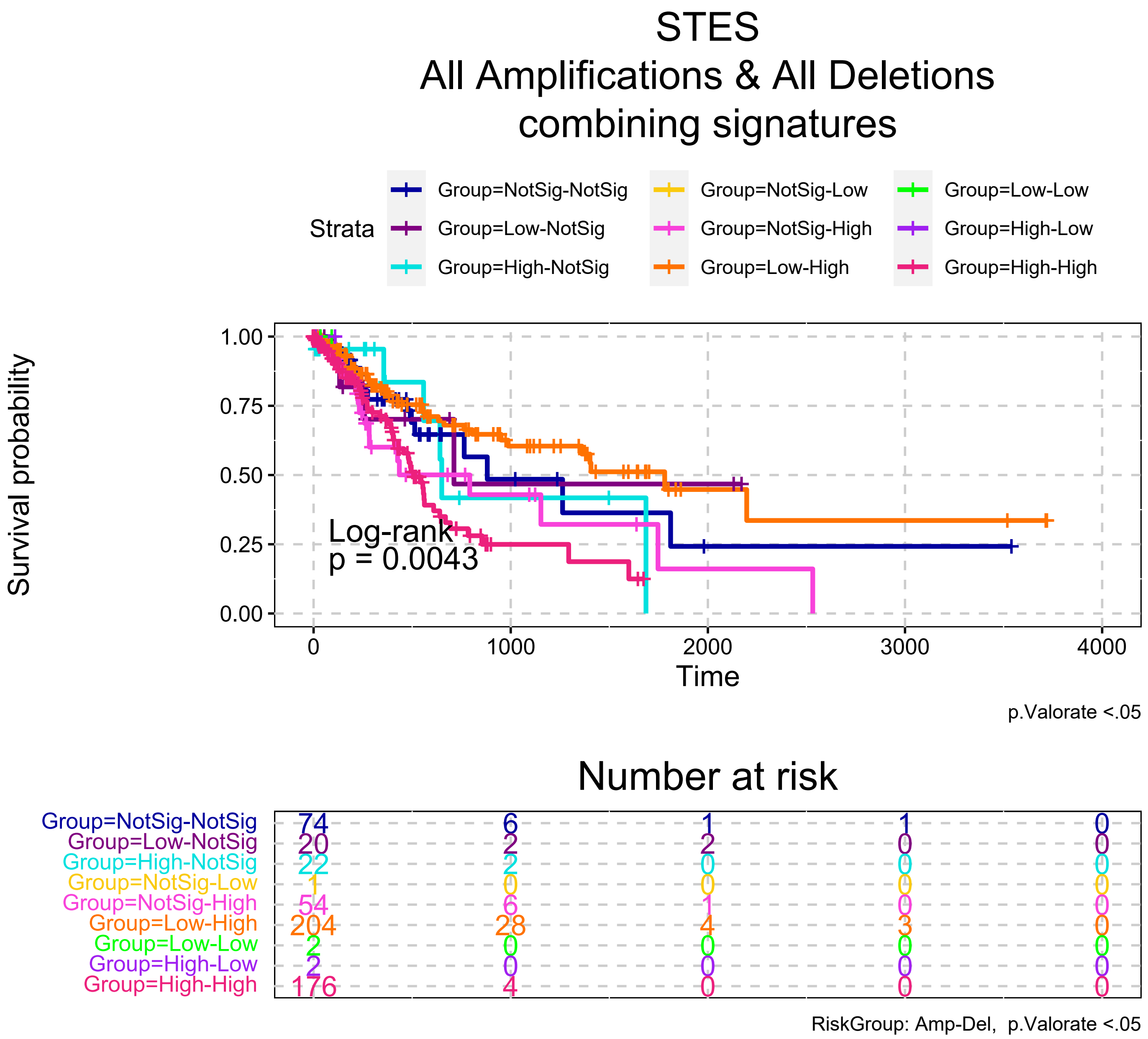

explanatory	beta	HR	L95	U95	p
Low-NotSig	-0.14	0.87	0.29	2.62	0.81
High-NotSig	0.19	1.20	0.47	3.08	0.70
NotSig-Low	-12.88	0.00	0.00	Inf	1.00
NotSig-High	0.45	1.56	0.80	3.07	0.19
Low-High	-0.28	0.75	0.42	1.35	0.34
Low-Low	-12.88	0.00	0.00	Inf	1.00
High-Low	-12.88	0.00	0.00	Inf	1.00
High-High	0.64	1.89	1.08	3.32	0.03

n= 556, number of events =140
Score(logrank) test = 0.004

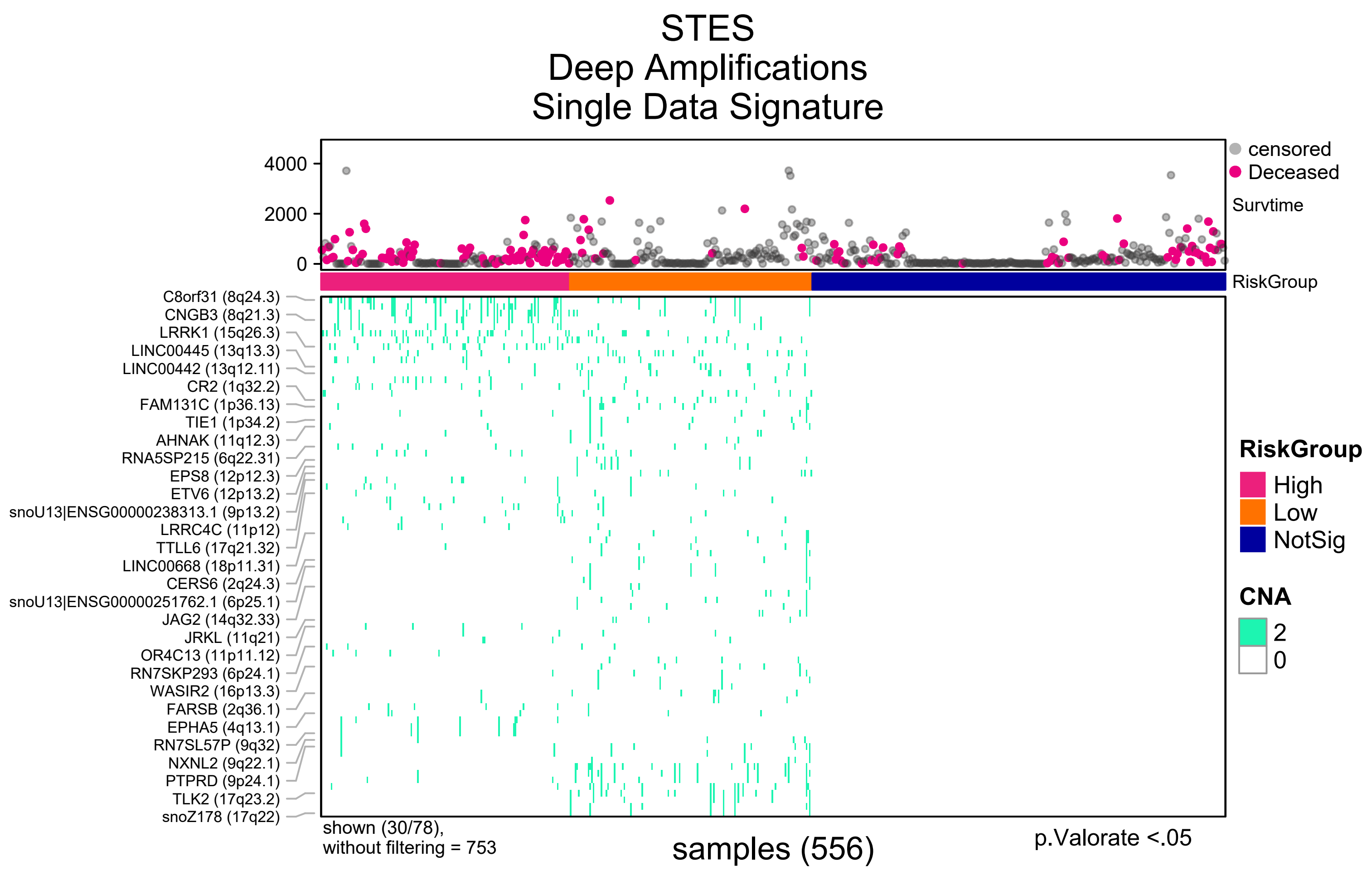
STES
Deep Amplifications
Single Data Signature
4000
2000
0
censored
Deceased
Survtime
RiskGroup
C8orf31 (8q24.3)
CNGB3 (8q21.3)
LRRK1 (15q26.3)
LINC00445 (13q13.3)
LINC00442 (13q12.11)
CR2 (1q32.2)
FAM131C (1p36.13)
TIE1 (1p34.2)
AHNAK (11q12.3)
RNA5SP215 (6q22.31)
EPS8 (12p12.3)
ETV6 (12p13.2)
snoU13|ENSG00000238313.1 (9p13.2)
LRRC4C (11p12)
TTLL6 (17q21.32)
LINC00668 (18p11.31)
CERS6 (2q24.3)
snoU13|ENSG00000251762.1 (6p25.1)
JAG2 (14q32.33)
JRKL (11q21)
OR4C13 (11p11.12)
RN7SKP293 (6p24.1)
WASIR2 (16p13.3)
FARSB (2q36.1)
EPHA5 (4q13.1)
RN7SL57P (9q32)
NXNL2 (9q22.1)
PTPRD (9p24.1)
TLK2 (17q23.2)
snoZ178 (17q22)
RiskGroup
High
Low
NotSig
CNA
2
0
shown (30/78),
without filtering = 753
samples (556)
p.Valorate <.05

STES
Deep Amplifications
Single Data Signature

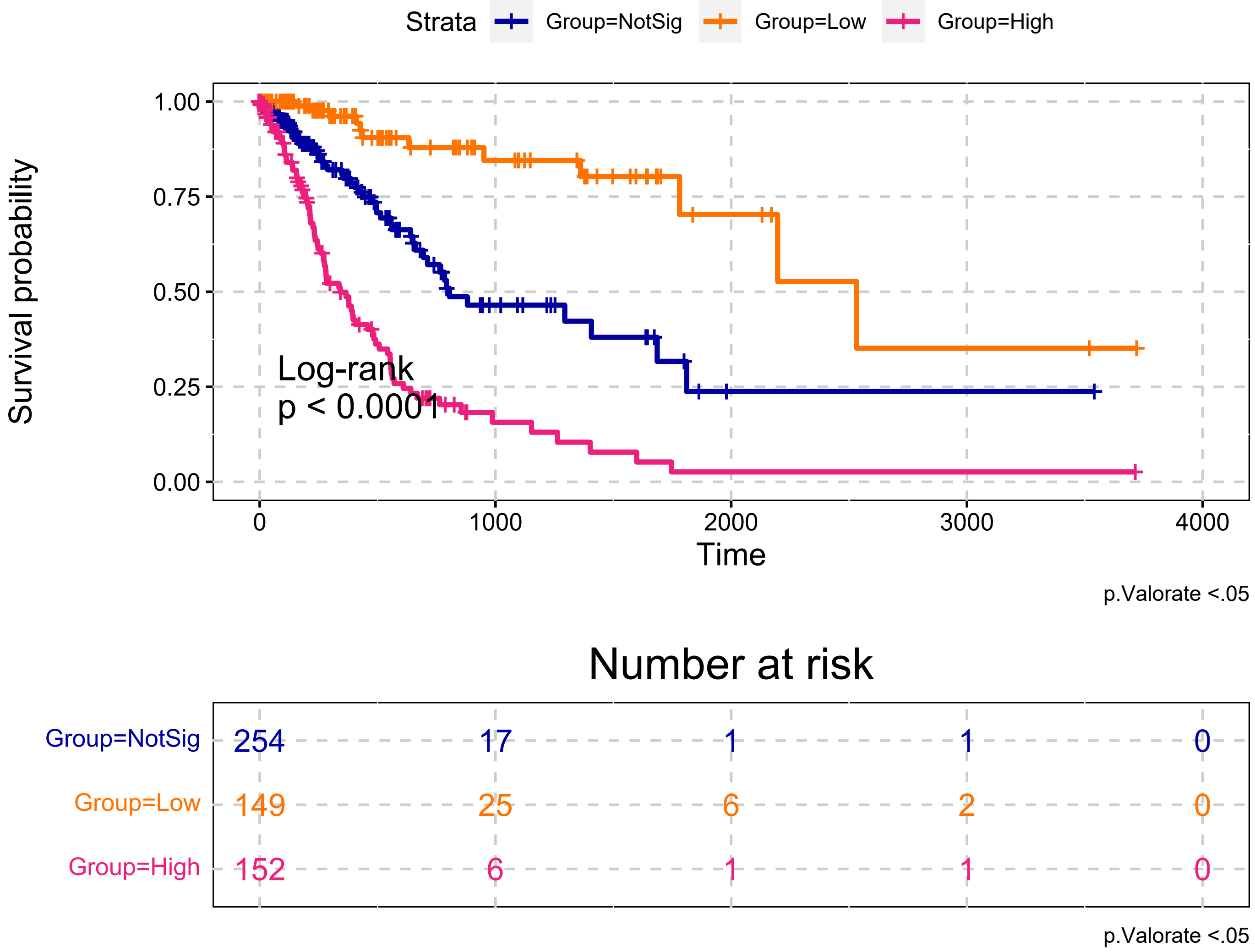

explanatory	beta	HR	L95	U95	p
Low	-1.36	0.26	0.14	0.49	0.00
High	1.02	2.78	1.94	3.97	0.00

n= 556, number of events =140
Score(logrank) test = p <.0001

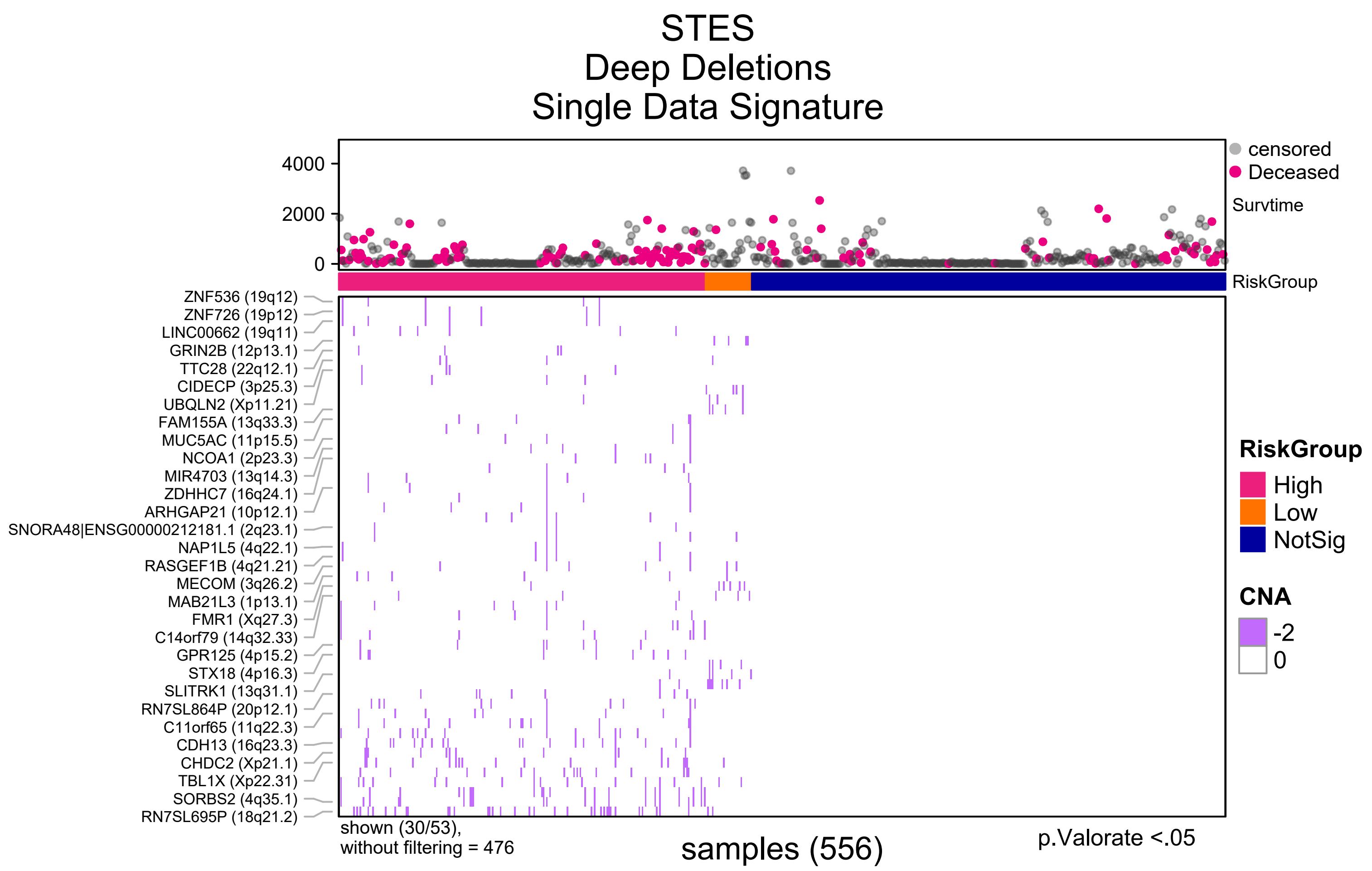

STES
Deep Deletions
Single Data Signature
4000
2000
0
censored
Deceased
Survtime
RiskGroup
ZNF536 (19q12)
ZNF726 (19p12)
LINC00662 (19q11)
GRIN2B (12p13.1)
TTC28 (22q12.1)
CIDECP (3p25.3)
UBQLN2 (Xp11.21)
FAM155A (13q33.3)
MUC5AC (11p15.5)
NCOA1 (2p23.3)
MIR4703 (13q14.3)
ZDHHC7 (16q24.1)
ARHGAP21 (10p12.1)
SNORA48|ENSG00000212181.1 (2q23.1)
NAP1L5 (4q22.1)
RASGEF1B (4q21.21)
MECOM (3q26.2)
MAB21L3 (1p13.1)
FMR1 (Xq27.3)
C14orf79 (14q32.33)
GPR125 (4p15.2)
STX18 (4p16.3)
SLITRK1 (13q31.1)
RN7SL864P (20p12.1)
C11orf65 (11q22.3)
CDH13 (16q23.3)
CHDC2 (Xp21.1)
TBL1X (Xp22.31)
SORBS2 (4q35.1)
RN7SL695P (18q21.2)
shown (30/53),
without filtering = 476
samples (556)
p.Valorate <.05
RiskGroup
High
Low
NotSig
CNA
-2
0

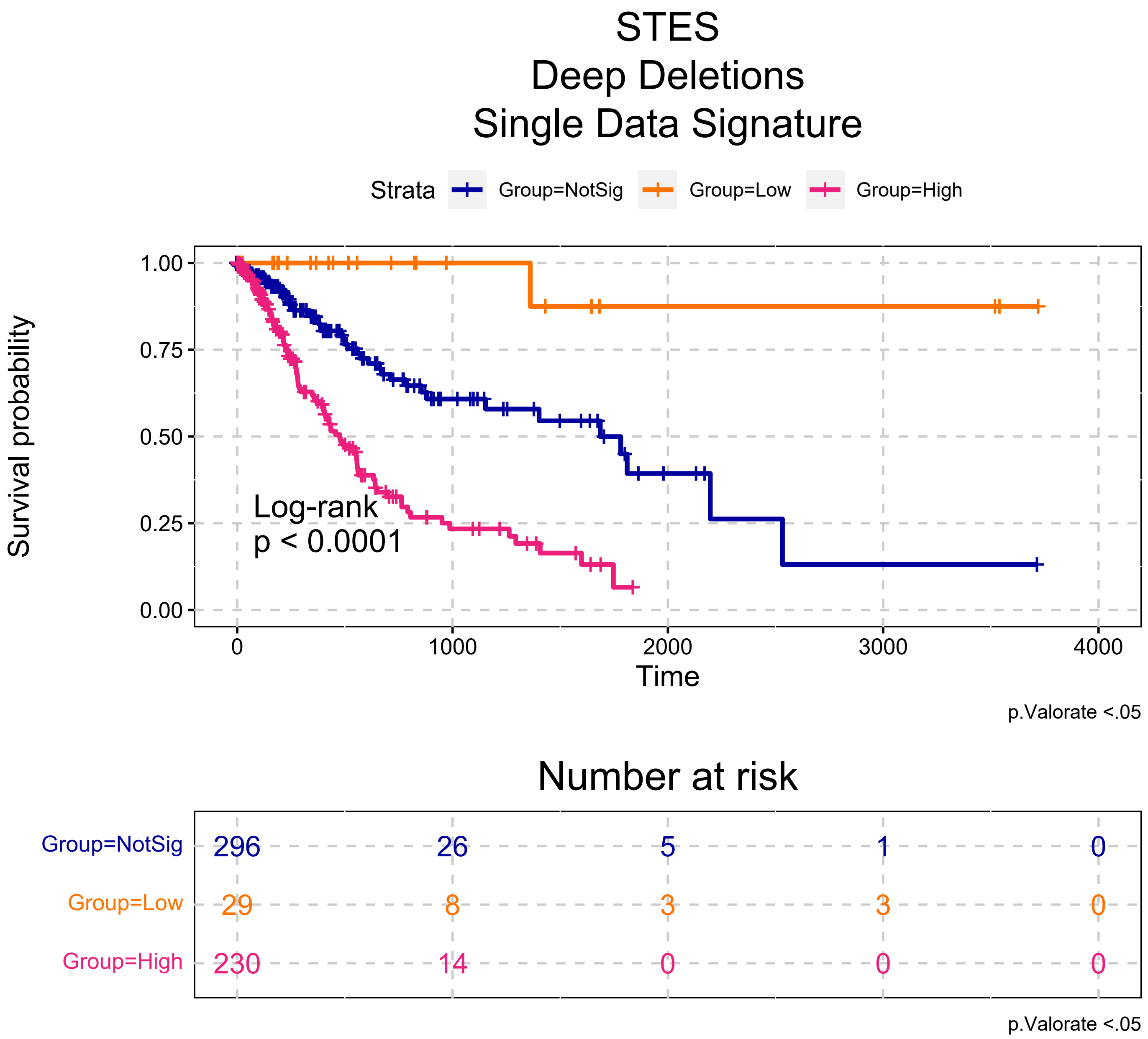

explanatory	beta	HR	L95	U95	p
Low	-2.49	0.08	0.01	0.61	0.01
High	1.02	2.78	1.94	3.97	0.00

n= 556, number of events =140
Score(logrank) test = p <.0001

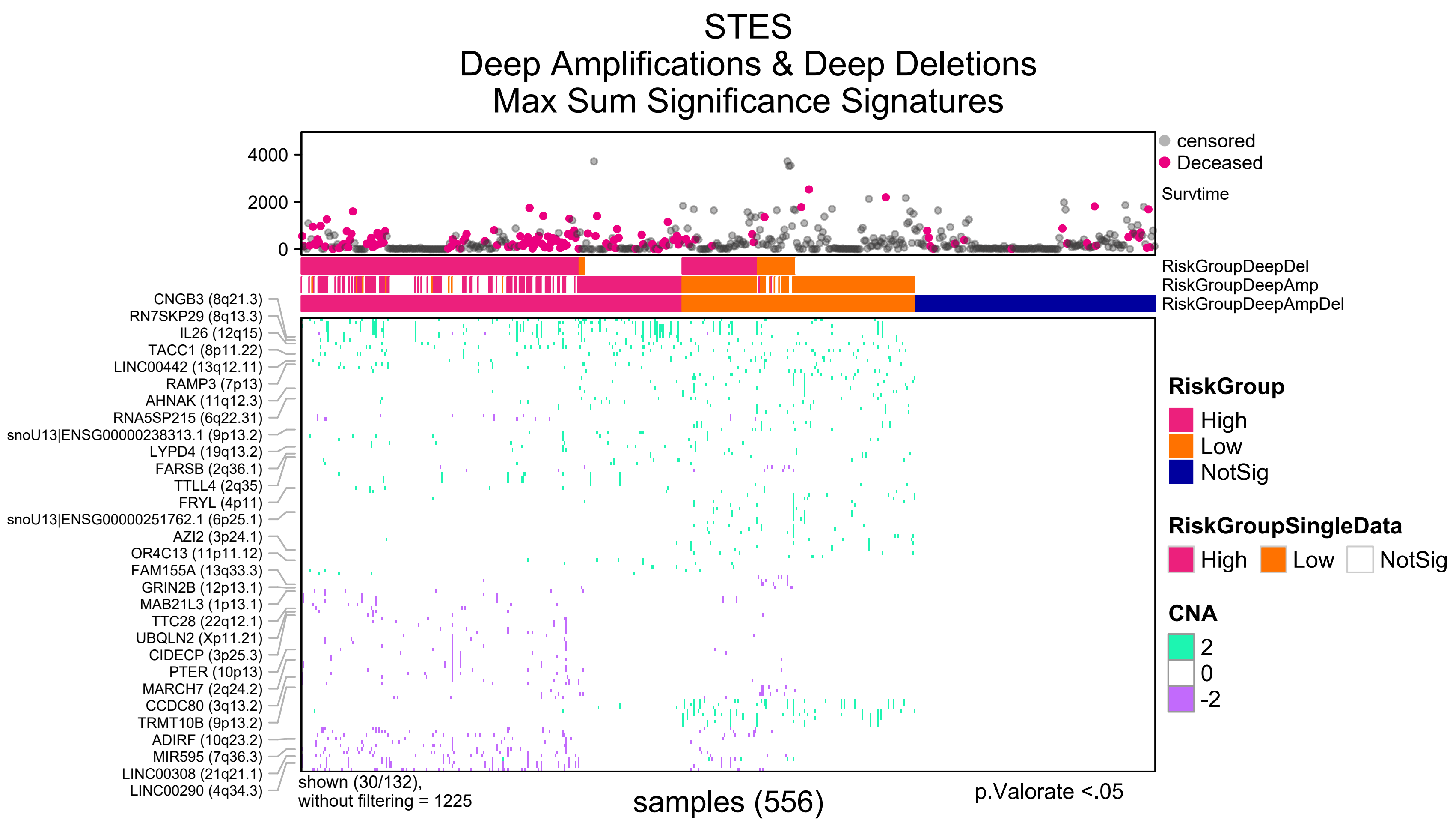
STES
Deep Amplifications & Deep Deletions
Max Sum Significance Signatures
4000
2000
0
censored
Deceased
Survtime
RiskGroupDeepDel
RiskGroupDeepAmp
RiskGroupDeepAmpDel
CNGB3 (8q21.3)
RN7SKP29 (8q13.3)
IL26 (12q15)
TACC1 (8p11.22)
LINC00442 (13q12.11)
RAMP3 (7p13)
AHNAK (11q12.3)
RNA5SP215 (6q22.31)
snoU13|ENSG00000238313.1 (9p13.2)
LYPD4 (19q13.2)
FARSB (2q36.1)
TTLL4 (2q35)
FRYL (4p11)
snoU13|ENSG00000251762.1 (6p25.1)
AZI2 (3p24.1)
OR4C13 (11p11.12)
FAM155A (13q33.3)
GRIN2B (12p13.1)
MAB21L3 (1p13.1)
TTC28 (22q12.1)
UBQLN2 (Xp11.21)
CIDECP (3p25.3)
PTER (10p13)
MARCH7 (2q24.2)
CCDC80 (3q13.2)
TRMT10B (9p13.2)
ADIRF (10q23.2)
MIR595 (7q36.3)
LINC00308 (21q21.1)
LINC00290 (4q34.3)
RiskGroup
High
Low
NotSig
RiskGroupSingleData
High
Low
NotSig
CNA
2
0
-2
shown (30/132),
without filtering = 1225
samples (556)
p.Valorate <.05

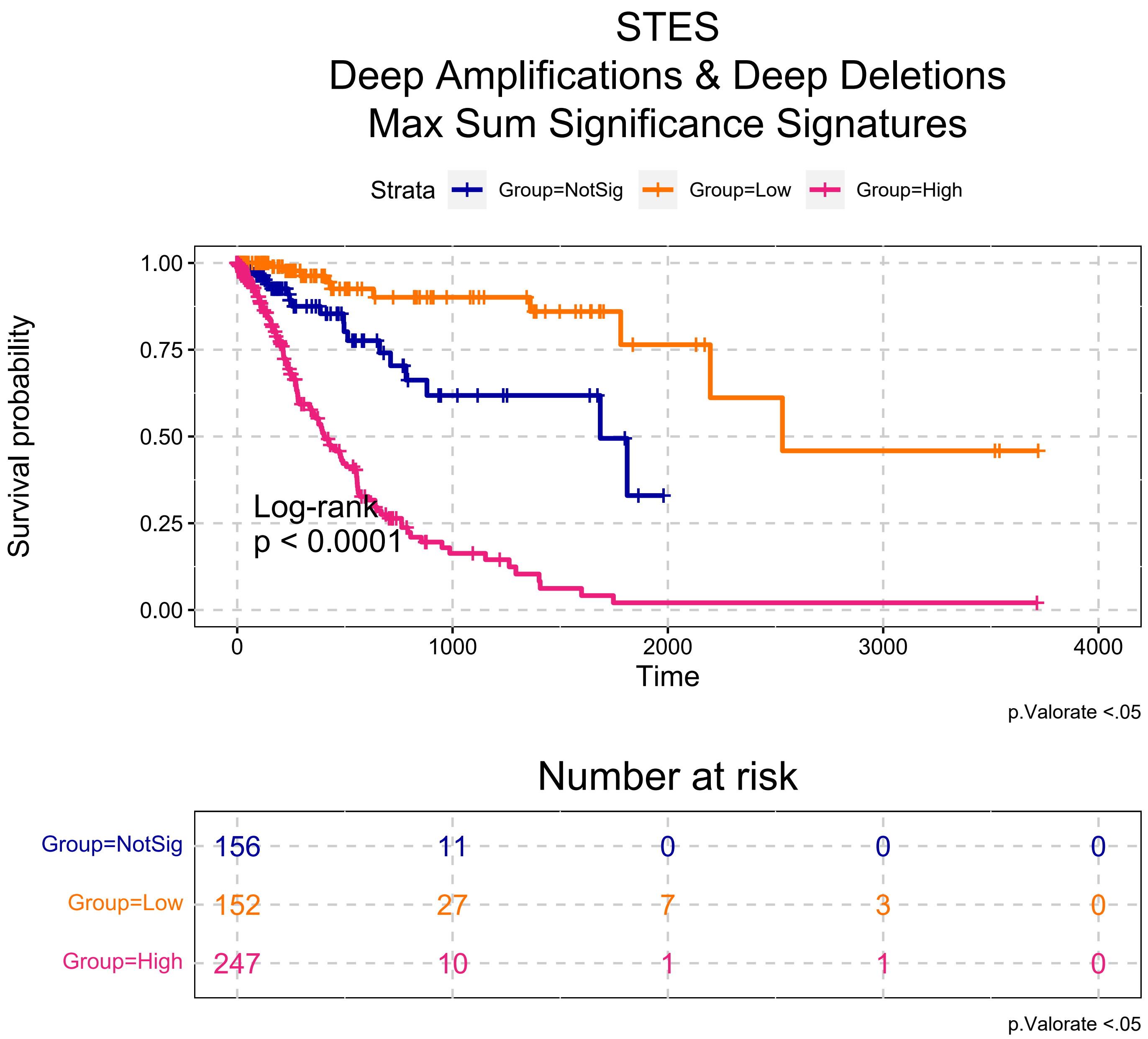

explanatory	beta	HR	L95	U95	p
Low	-1.21	0.30	0.14	0.64	0.00
High	1.36	3.89	2.40	6.29	0.00

n= 556, number of events =140
Score(logrank) test = p <.0001

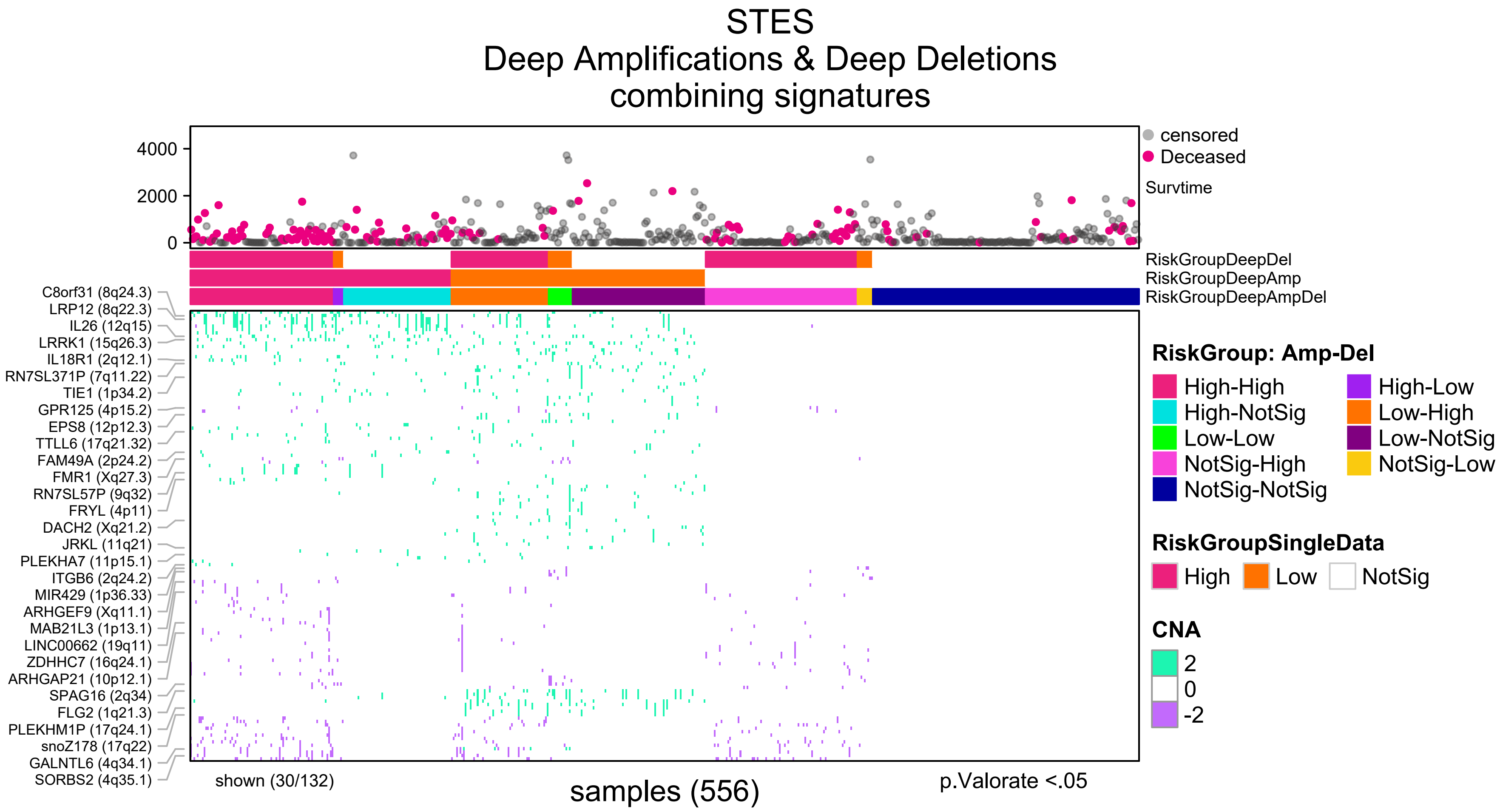
STES
Deep Amplifications & Deep Deletions
combining signatures
4000
2000
0
censored
Deceased
Survtime
RiskGroupDeepDel
RiskGroupDeepAmp
RiskGroupDeepAmpDel
C8orf31 (8q24.3)
LRP12 (8q22.3)
IL26 (12q15)
LRRK1 (15q26.3)
IL18R1 (2q12.1)
RN7SL371P (7q11.22)
TIE1 (1p34.2)
GPR125 (4p15.2)
EPS8 (12p12.3)
TTLL6 (17q21.32)
FAM49A (2p24.2)
FMR1 (Xq27.3)
RN7SL57P (9q32)
FRYL (4p11)
DACH2 (Xq21.2)
JRKL (11q21)
PLEKHA7 (11p15.1)
ITGB6 (2q24.2)
MIR429 (1p36.33)
ARHGEF9 (Xq11.1)
MAB21L3 (1p13.1)
LINC00662 (19q11)
ZDHHC7 (16q24.1)
ARHGAP21 (10p12.1)
SPAG16 (2q34)
FLG2 (1q21.3)
PLEKHM1P (17q24.1)
snoZ178 (17q22)
GALNTL6 (4q34.1)
SORBS2 (4q35.1)
RiskGroup: Amp-Del
High-High
High-Low
High-NotSig
Low-High
Low-Low
Low-NotSig
NotSig-High
NotSig-Low
NotSig-NotSig
RiskGroupSingleData
High
Low
NotSig
CNA
2
0
-2
shown (30/132)
samples (556)
p.Valorate <.05

STES
Deep Amplifications & Deep Deletions combining signatures

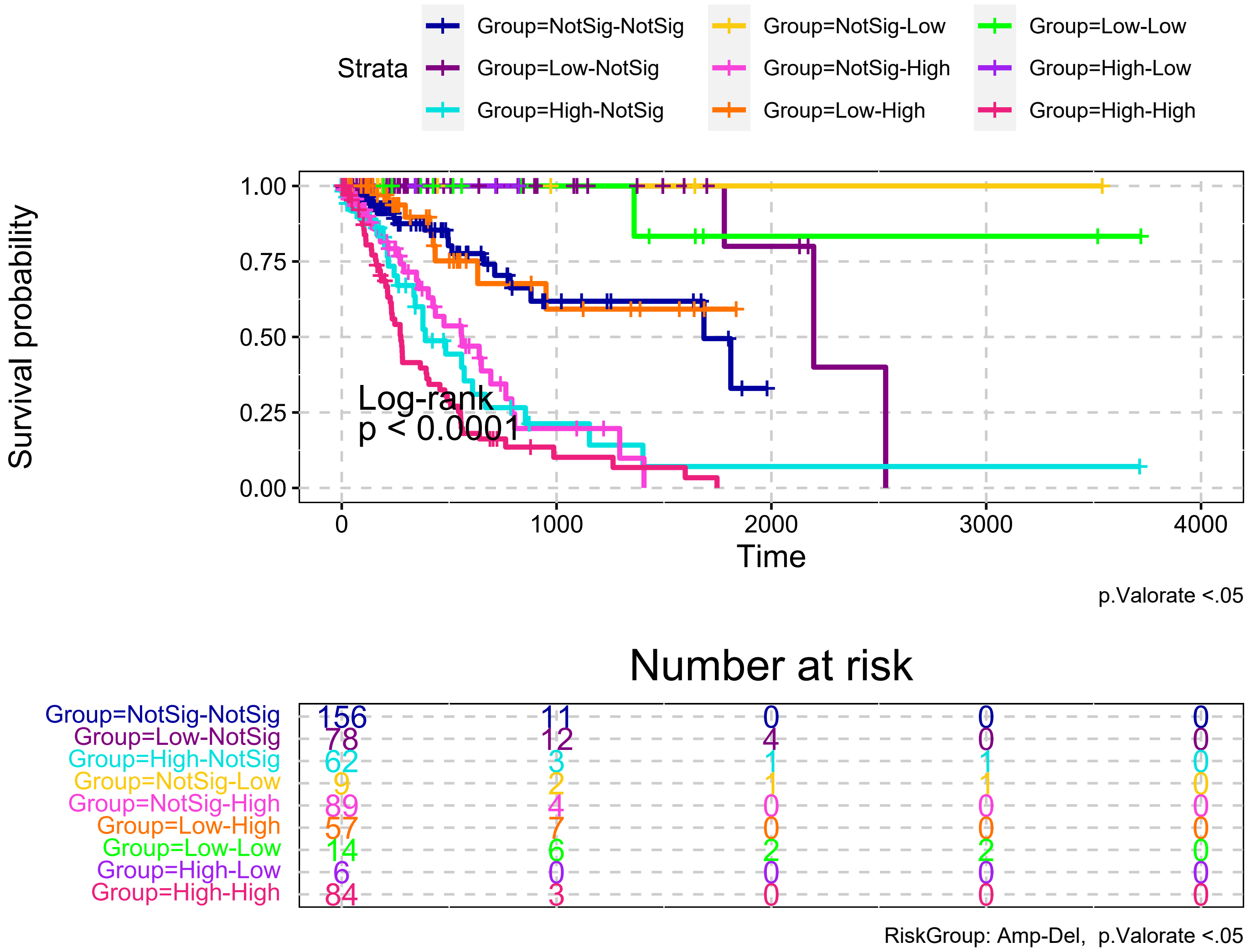

explanatory	beta	HR	L95	U95	p
Low-NotSig	-1.64	0.19	0.06	0.65	0.01
High-NotSig	1.19	3.29	1.81	5.98	0.00
NotSig-Low	-16.23	0.00	0.00	Inf	0.99
NotSig-High	1.14	3.12	1.76	5.53	0.00
Low-High	-0.16	0.85	0.37	1.93	0.70
Low-Low	-1.93	0.14	0.02	1.09	0.06
High-Low	-16.04	0.00	0.00	Inf	1.00
High-High	1.71	5.50	3.27	9.25	0.00

n= 556, number of events =140
Score(logrank) test = p <.0001
