# Supplementary material for: Systematic Modeling of Risk-Associated Copy Number Alterations in Cancer
Source: Int J Mol Sci. 2024 Sep 27;25(19):10455. doi: 10.3390/ijms251910455 (PMC11477427; doi:10.3390/ijms251910455)
Supplement: Supplementary file 1 [file ijms-25-10455-s001.zip › TGCTSignatureV12-sinSombreado.pdf]

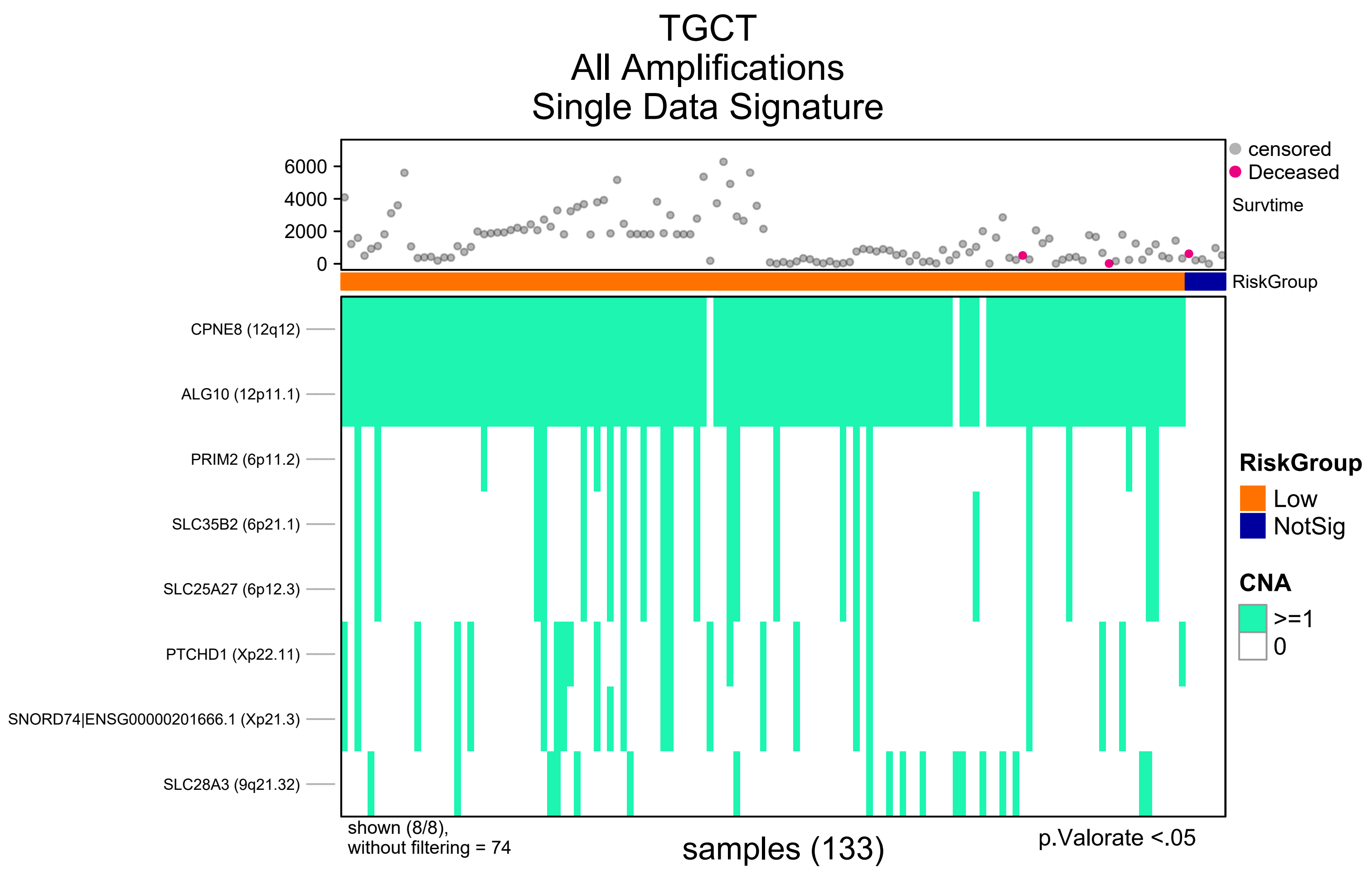

TCGT
All Amplifications
Single Data Signature
6000
4000
2000
0
censored
Deceased
Survtime
RiskGroup
CPNE8 (12q12)
ALG10 (12p11.1)
PRIM2 (6p11.2)
SLC35B2 (6p21.1)
SLC25A27 (6p12.3)
PTCHD1 (Xp22.11)
SNORD74|ENSG00000201666.1 (Xp21.3)
SLC28A3 (9q21.32)
RiskGroup
Low
NotSig
CNA
>=1
0
shown (8/8),
without filtering = 74
samples (133)
p.Valorate <.05

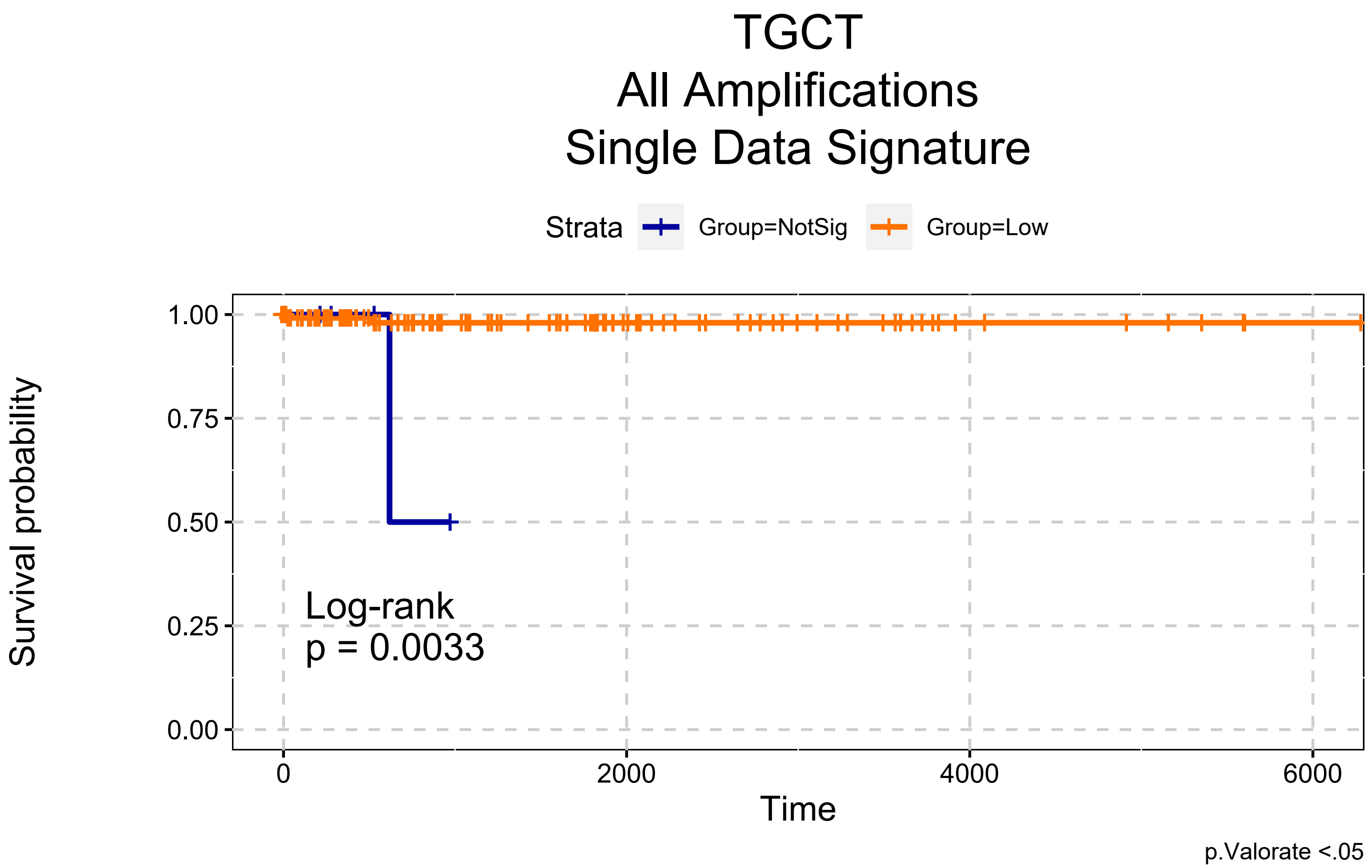

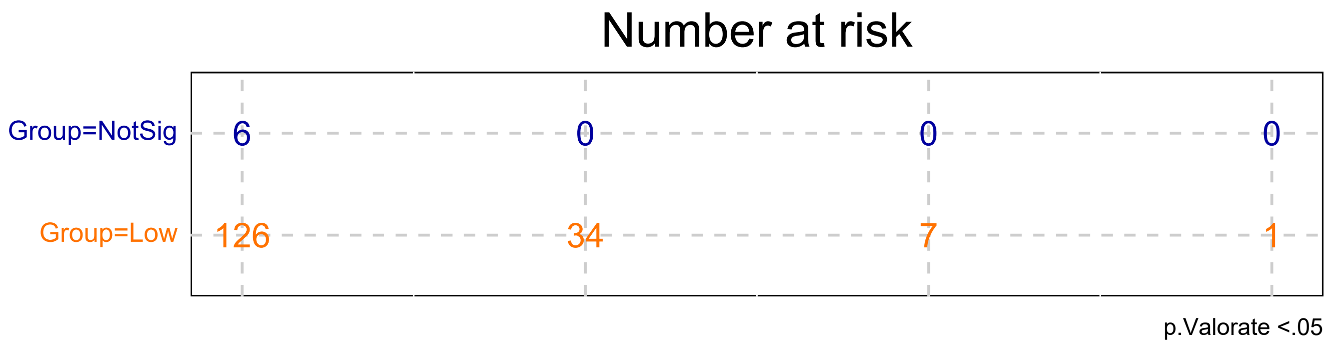

explanatory	beta	HR	L95	U95	p
Low	-2.71	0.07	0.01	0.74	0.03

n= 133, number of events =3
Score(logrank) test = 0.003

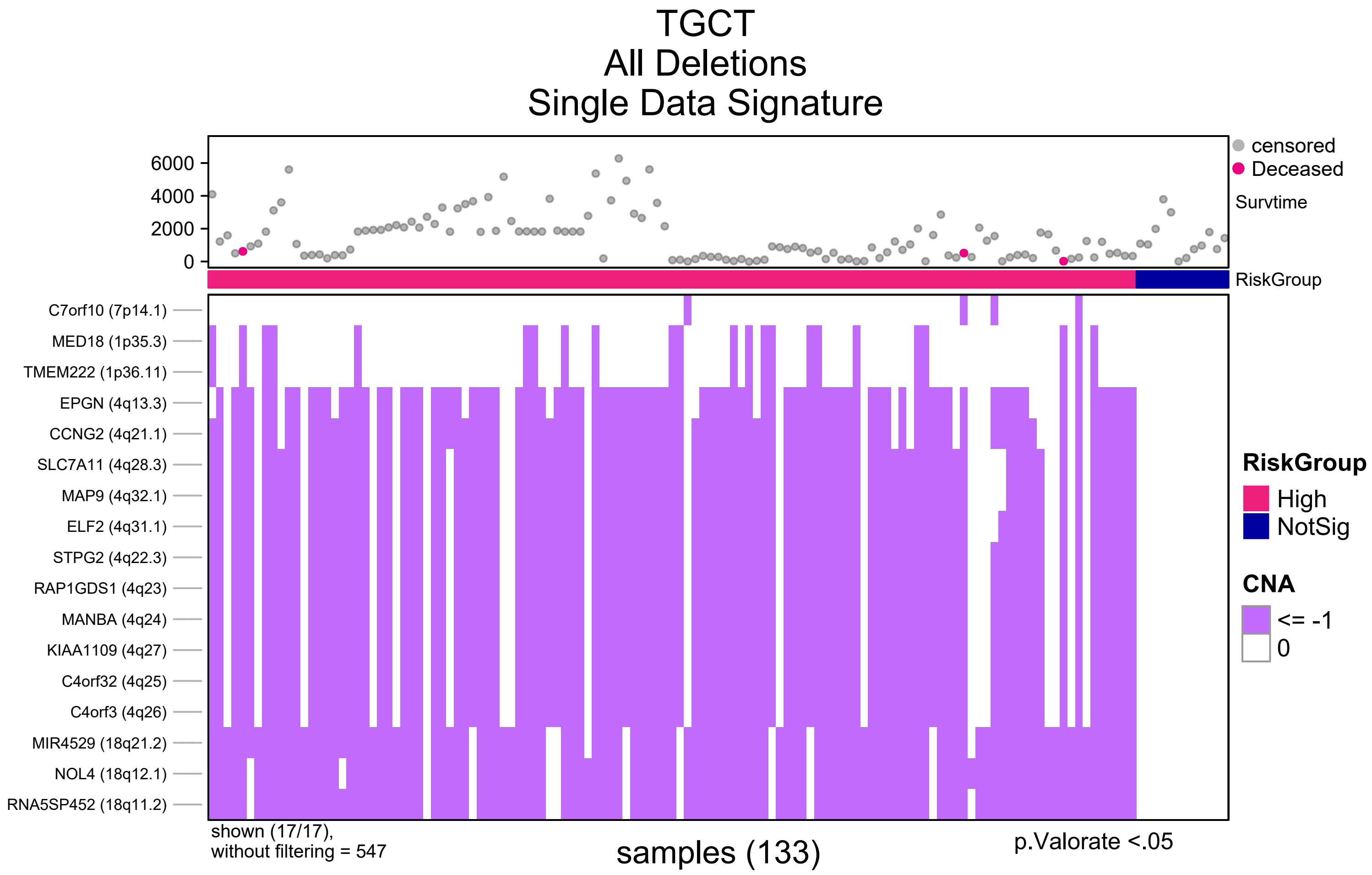

TGCT
All Deletions
Single Data Signature
6000
4000
2000
0
censored
Deceased
Survtime
RiskGroup
C7orf10 (7p14.1)
MED18 (1p35.3)
TMEM222 (1p36.11)
EPGN (4q13.3)
CCNG2 (4q21.1)
SLC7A11 (4q28.3)
MAP9 (4q32.1)
ELF2 (4q31.1)
STPG2 (4q22.3)
RAP1GDS1 (4q23)
MANBA (4q24)
KIAA1109 (4q27)
C4orf32 (4q25)
C4orf3 (4q26)
MIR4529 (18q21.2)
NOL4 (18q12.1)
RNA5SP452 (18q11.2)
RiskGroup
High
NotSig
CNA
<= -1
0
shown (17/17),
without filtering = 547
samples (133)
p.Valorate <.05

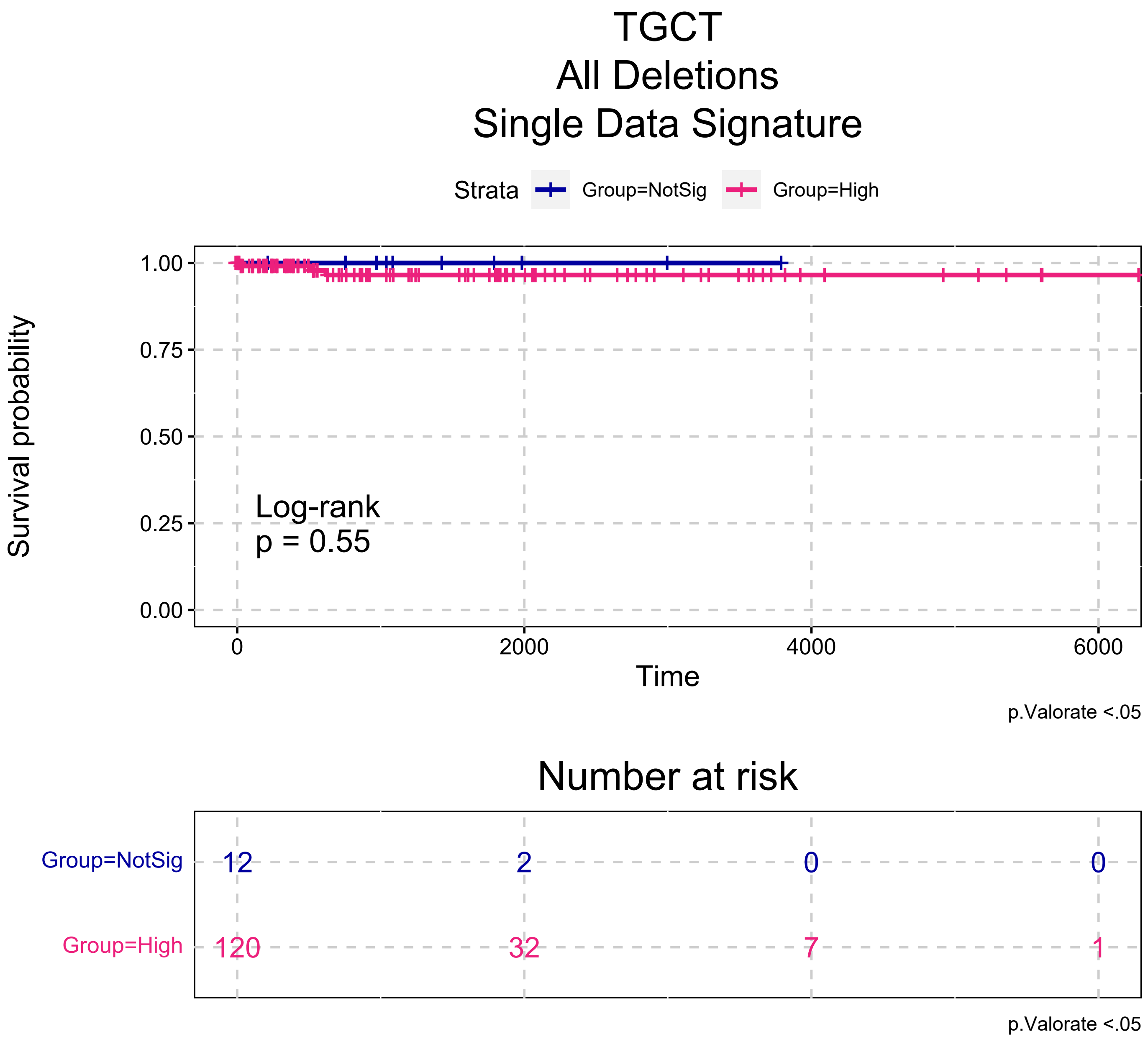

explanatory	beta	HR	L95	U95	p
High	18.18	78950482.04	0.00	Inf	1.00

n= 133, number of events =3
Score(logrank) test = 0.549

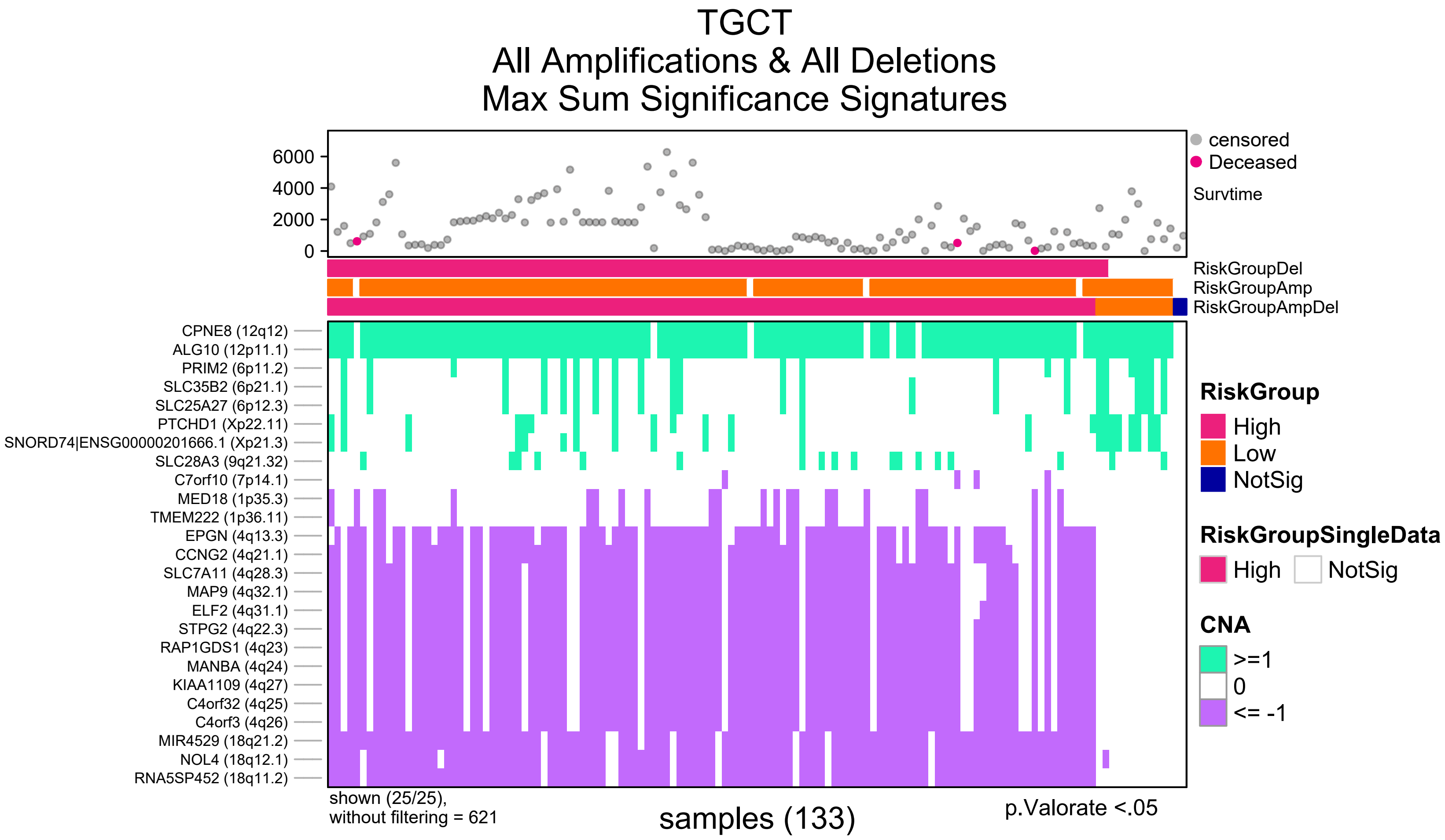

TGCT
All Amplifications & All Deletions
Max Sum Significance Signatures
6000
4000
2000
0
censored
Deceased
Survtime
RiskGroupDel
RiskGroupAmp
RiskGroupAmpDel
CPNE8 (12q12)
ALG10 (12p11.1)
PRIM2 (6p11.2)
SLC35B2 (6p21.1)
SLC25A27 (6p12.3)
PTCHD1 (Xp22.11)
SNORD74|ENSG00000201666.1 (Xp21.3)
SLC28A3 (9q21.32)
C7orf10 (7p14.1)
MED18 (1p35.3)
TMEM222 (1p36.11)
EPGN (4q13.3)
CCNG2 (4q21.1)
SLC7A11 (4q28.3)
MAP9 (4q32.1)
ELF2 (4q31.1)
STPG2 (4q22.3)
RAP1GDS1 (4q23)
MANBA (4q24)
KIAA1109 (4q27)
C4orf32 (4q25)
C4orf3 (4q26)
MIR4529 (18q21.2)
NOL4 (18q12.1)
RNA5SP452 (18q11.2)
RiskGroup
High
Low
NotSig
RiskGroupSingleData
High
NotSig
CNA
>=1
0
<= -1
shown (25/25),
without filtering = 621
samples (133)
p.Valorate <.05

TGCT
All Amplifications & All Deletions
Max Sum Significance Signatures

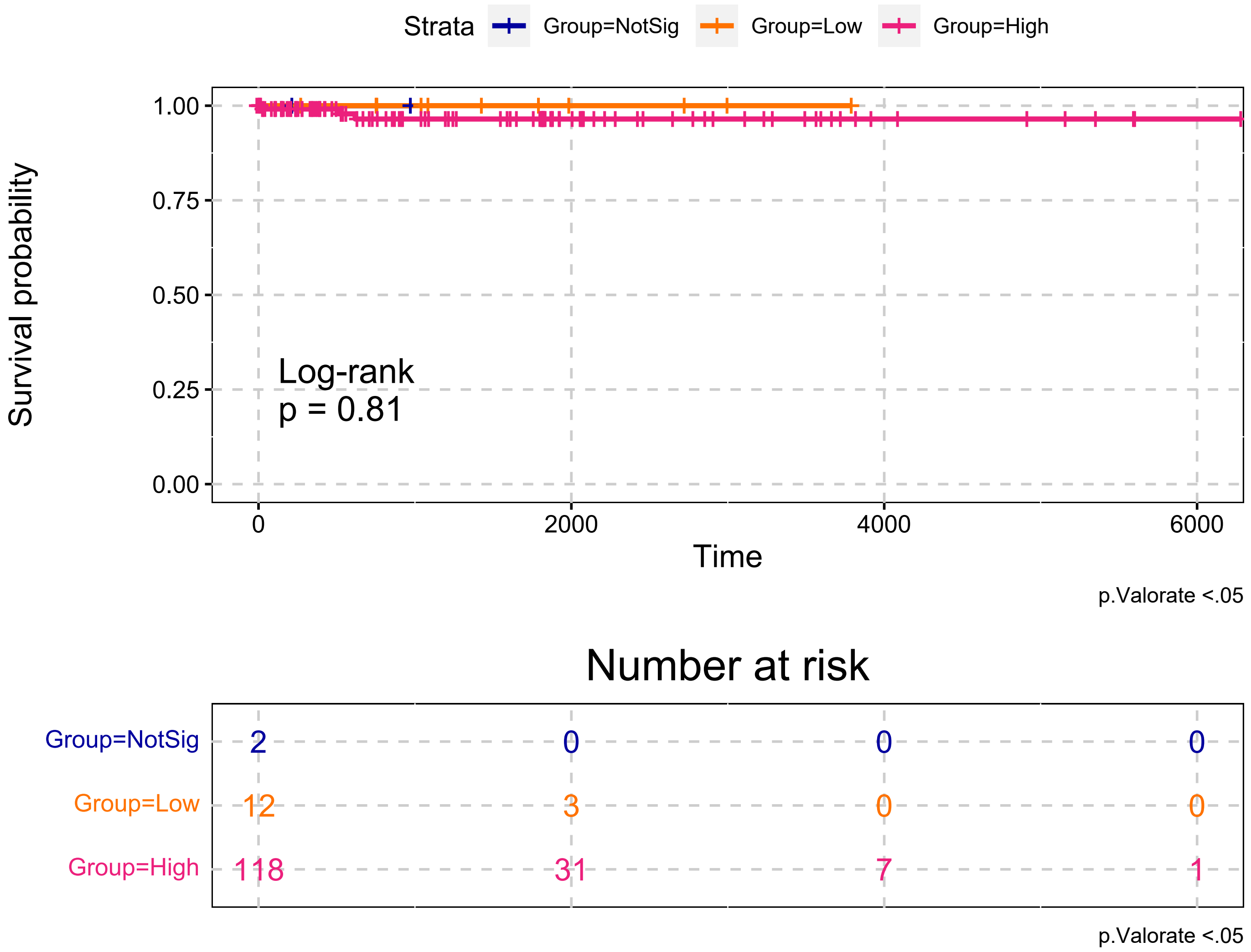

explanatory	beta	HR	L95	U95	p
Low	-0.01	0.99	0.00	Inf	1.00
High	18.20	80317879.55	0.00	Inf	1.00

n= 133, number of events =3
Score(logrank) test = 0.815

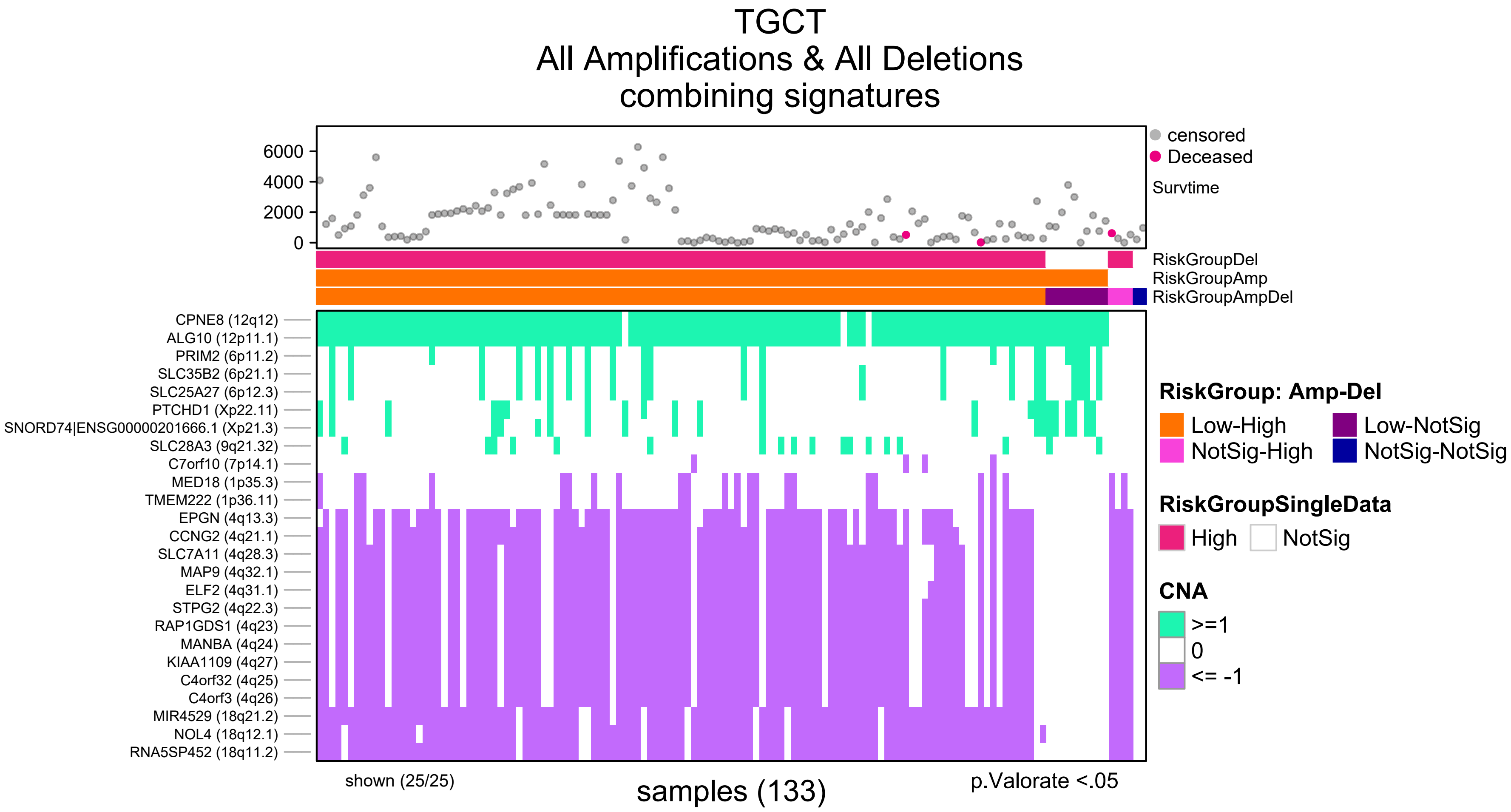

TGCT
All Amplifications & All Deletions
combining signatures
6000
4000
2000
0
censored
Deceased
Survtime
RiskGroupDel
RiskGroupAmp
RiskGroupAmpDel
CPNE8 (12q12)
ALG10 (12p11.1)
PRIM2 (6p11.2)
SLC35B2 (6p21.1)
SLC25A27 (6p12.3)
PTCHD1 (Xp22.11)
SNORD74|ENSG00000201666.1 (Xp21.3)
SLC28A3 (9q21.32)
C7orf10 (7p14.1)
MED18 (1p35.3)
TMEM222 (1p36.11)
EPGN (4q13.3)
CCNG2 (4q21.1)
SLC7A11 (4q28.3)
MAP9 (4q32.1)
ELF2 (4q31.1)
STPG2 (4q22.3)
RAP1GDS1 (4q23)
MANBA (4q24)
KIAA1109 (4q27)
C4orf32 (4q25)
C4orf3 (4q26)
MIR4529 (18q21.2)
NOL4 (18q12.1)
RNA5SP452 (18q11.2)
RiskGroup: Amp-Del
Low-High
Low-NotSig
NotSig-High
NotSig-NotSig
RiskGroupSingleData
High
NotSig
CNA
>=1
0
<= -1
shown (25/25)
samples (133)
p.Valorate <.05

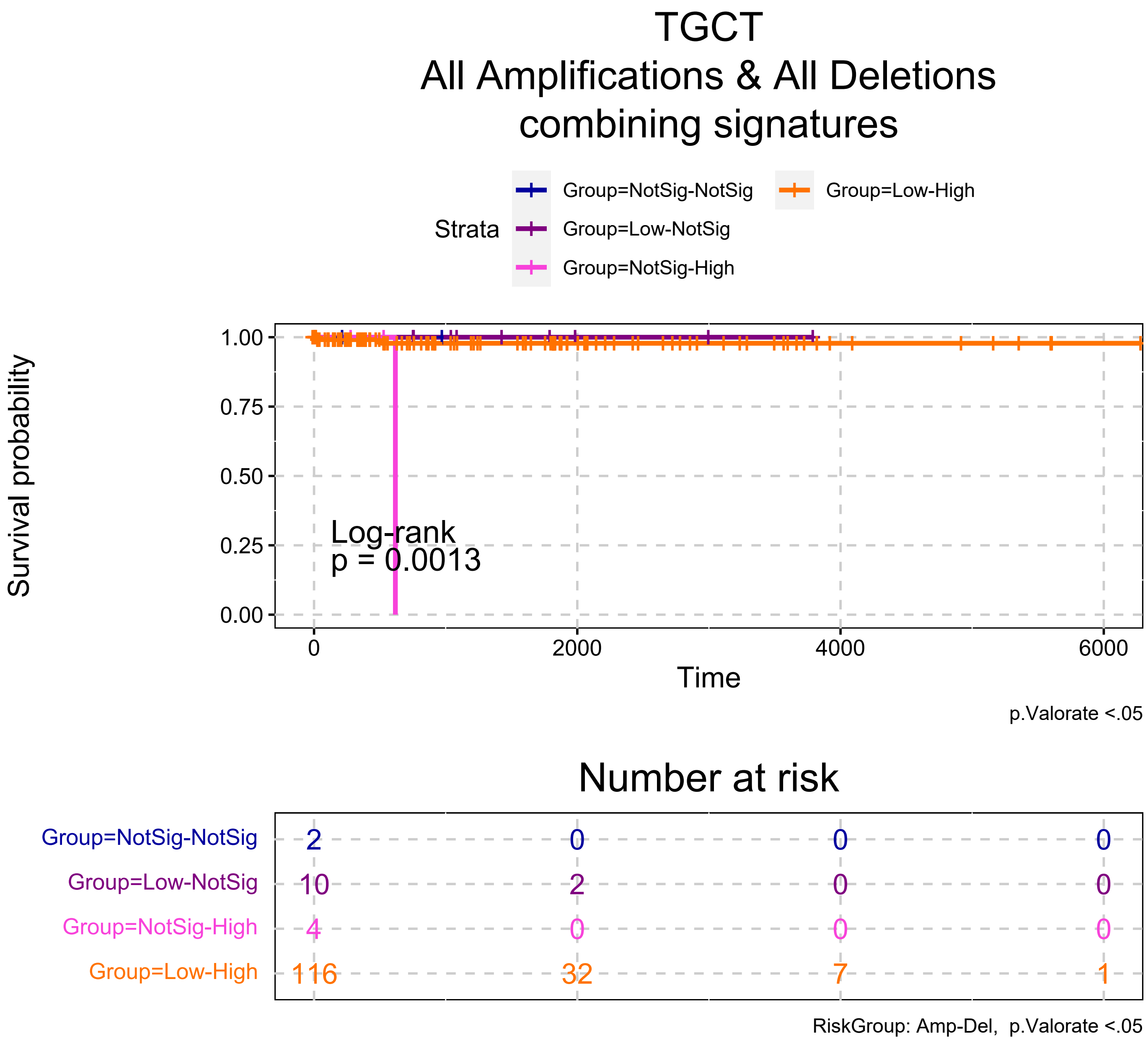

explanatory	beta	HR	L95	U95
Low-NotSig	-0.01	0.99	0.00	Inf
NotSig-High	20.29	649166942.68	57551431.10	7322454[illegible]
Low-High	17.15	28150320.15	2495646.52	3175291[illegible]

n= 133, number of events =3
Score(logrank) test = 0.001

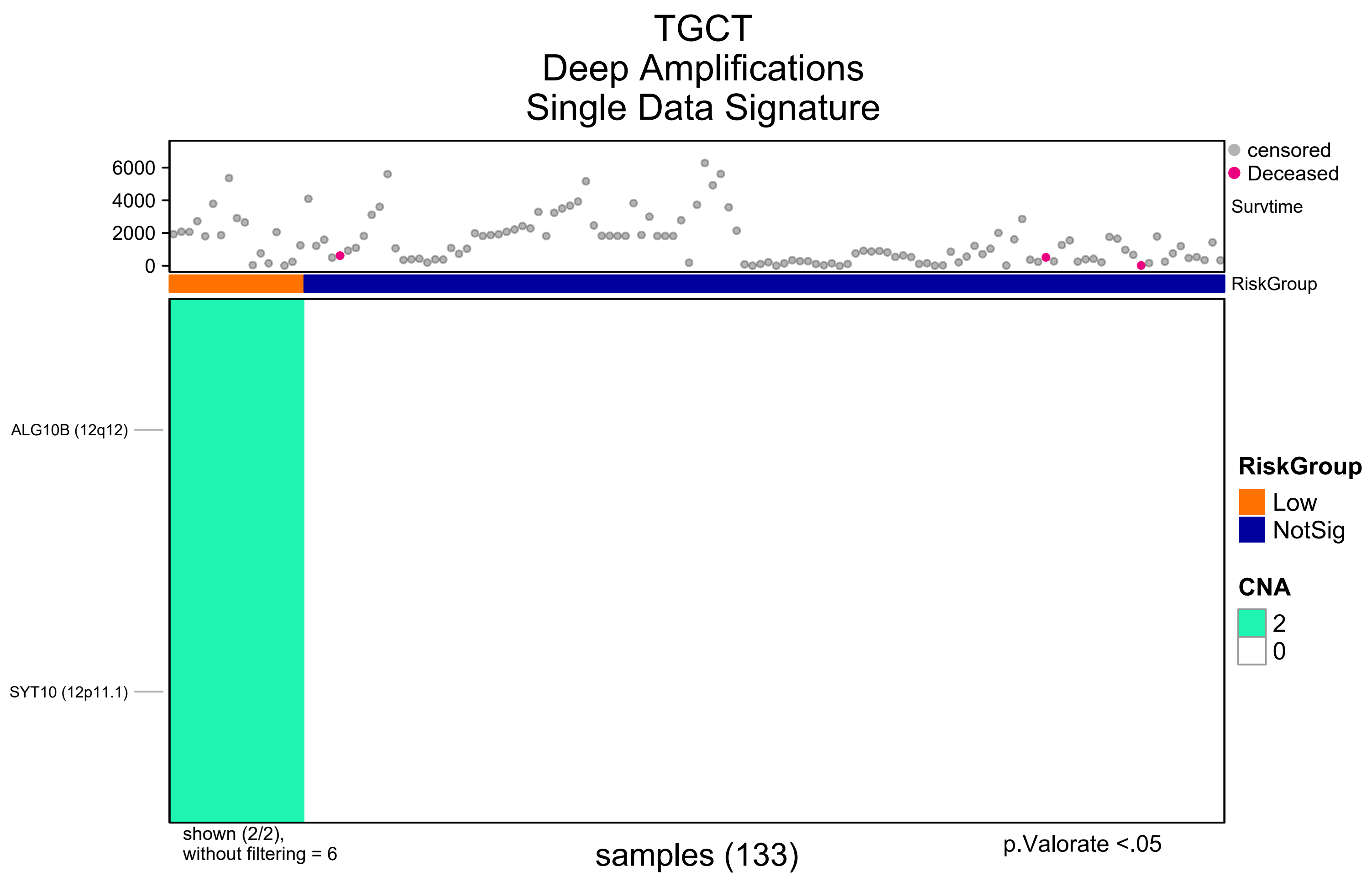
TGCT
Deep Amplifications
Single Data Signature
6000
4000
2000
0
censored
Deceased
Survtime
RiskGroup
ALG10B (12q12)
SYT10 (12p11.1)
RiskGroup
Low
NotSig
CNA
2
0
shown (2/2),
without filtering = 6
samples (133)
p.Valorate <.05

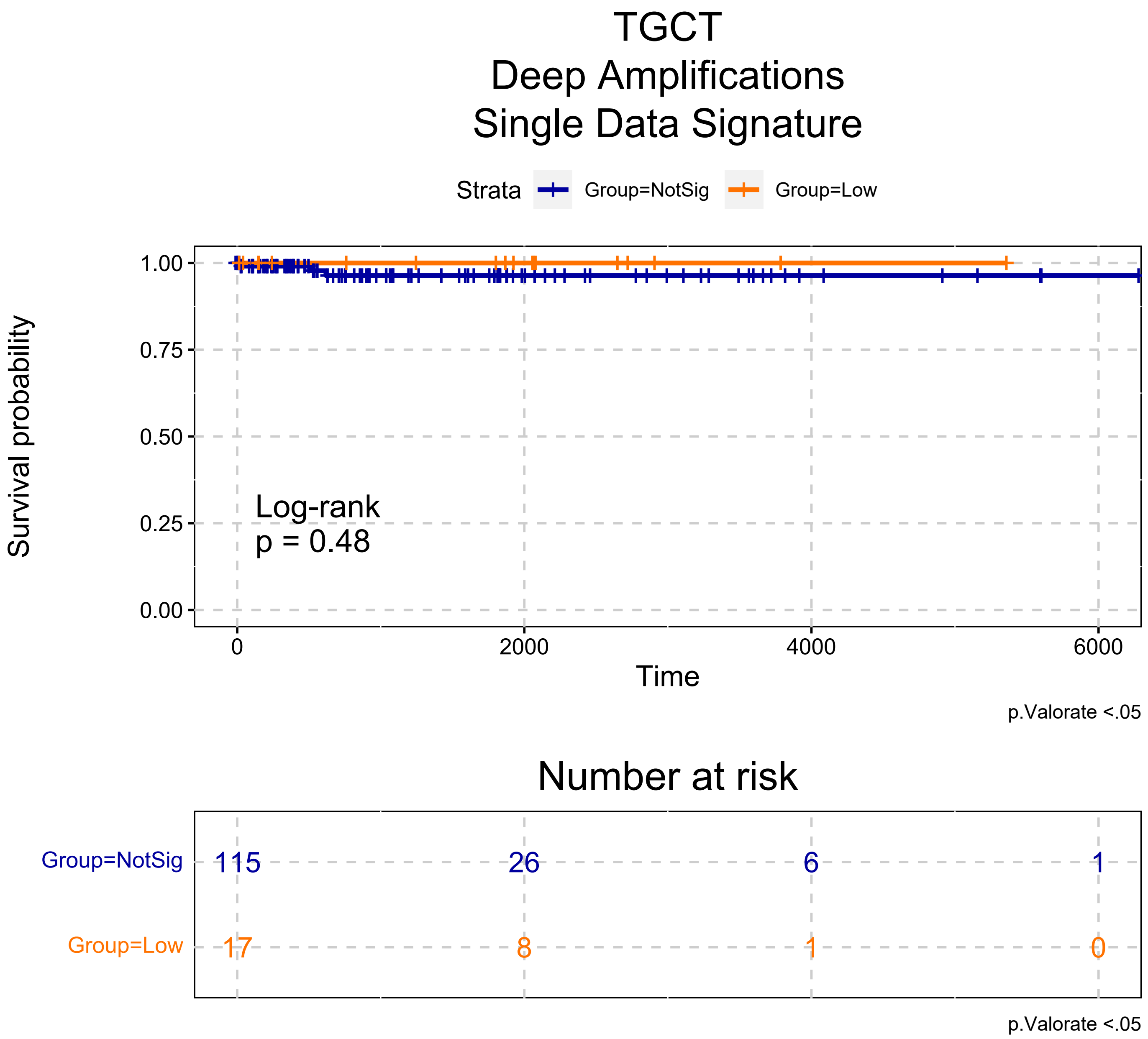

explanatory	beta	HR	L95	U95	p
Low	-18.25	0.00	0.00	Inf	1.00

n= 133, number of events =3
Score(logrank) test = 0.478

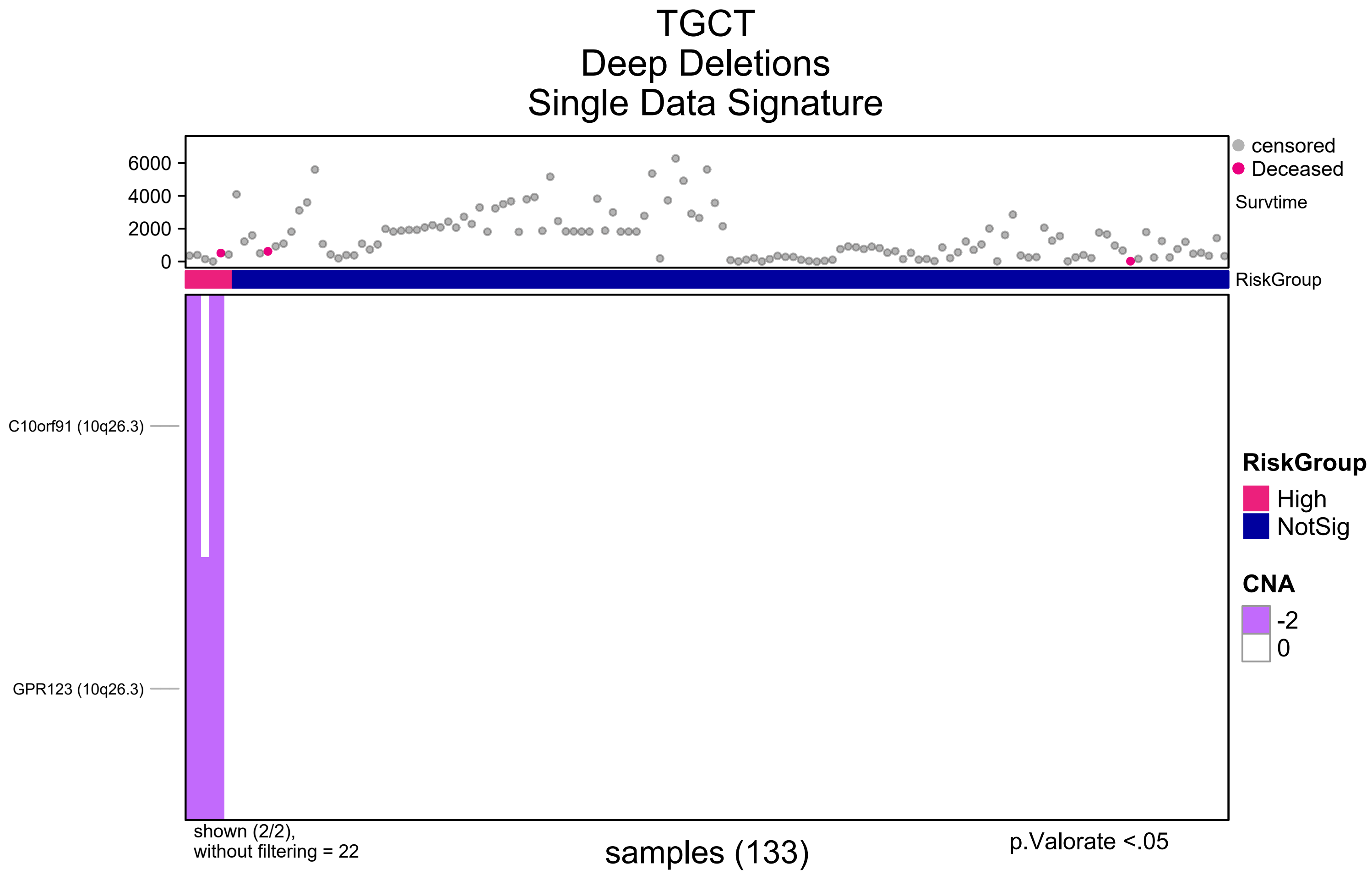

TGCT
Deep Deletions
Single Data Signature
6000
4000
2000
0
censored
Deceased
Survtime
RiskGroup
C10orf91 (10q26.3)
GPR123 (10q26.3)
RiskGroup
High
NotSig
CNA
-2
0
shown (2/2),
without filtering = 22
samples (133)
p.Valorate <.05

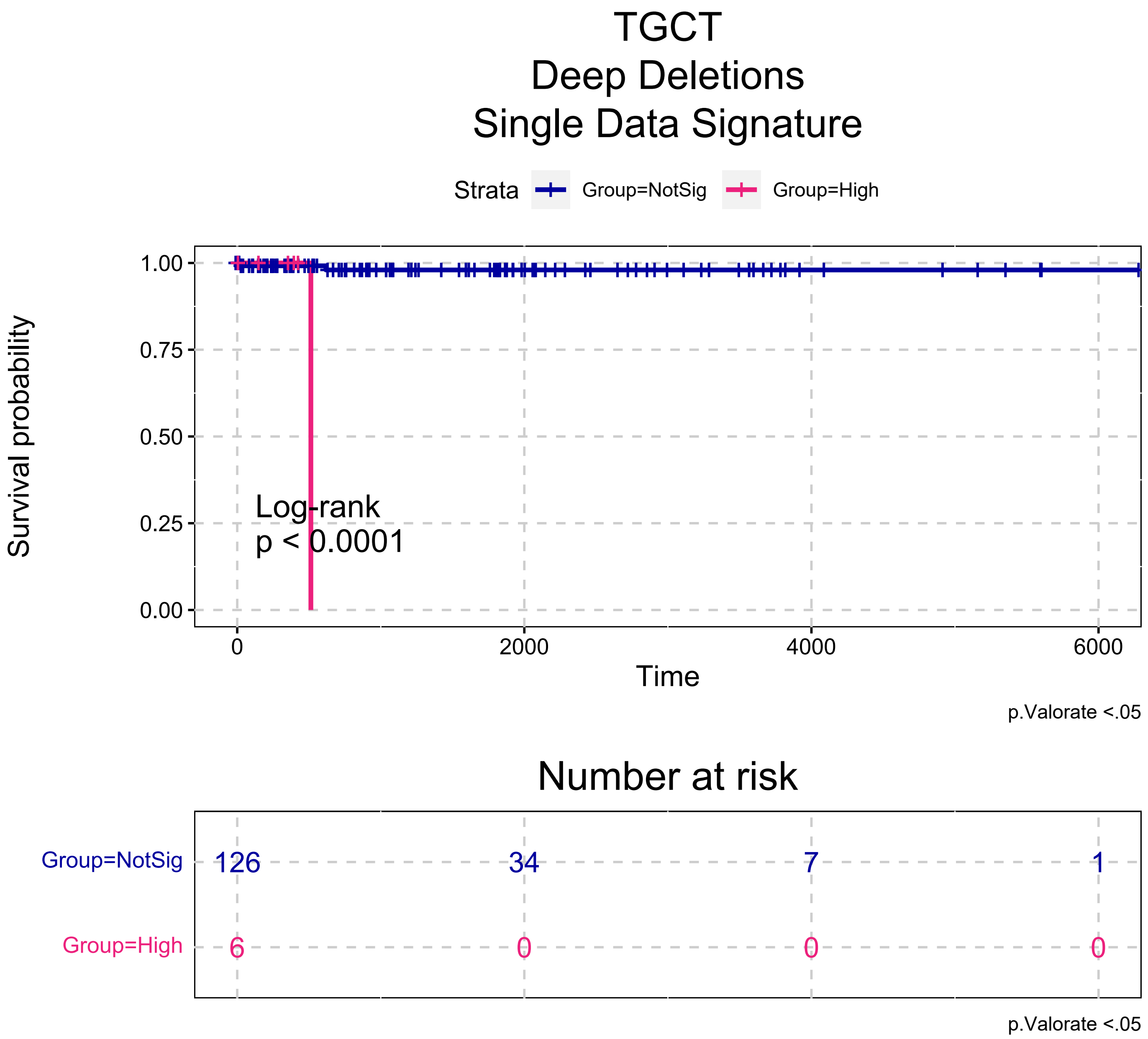

explanatory	beta	HR	L95	U95	p
High	3.83	46.07	2.50	848.30	0.01

n= 133, number of events =3
Score(logrank) test = p <.0001

TGCT
Deep Amplifications & Deep Deletions
Max Sum Significance Signatures

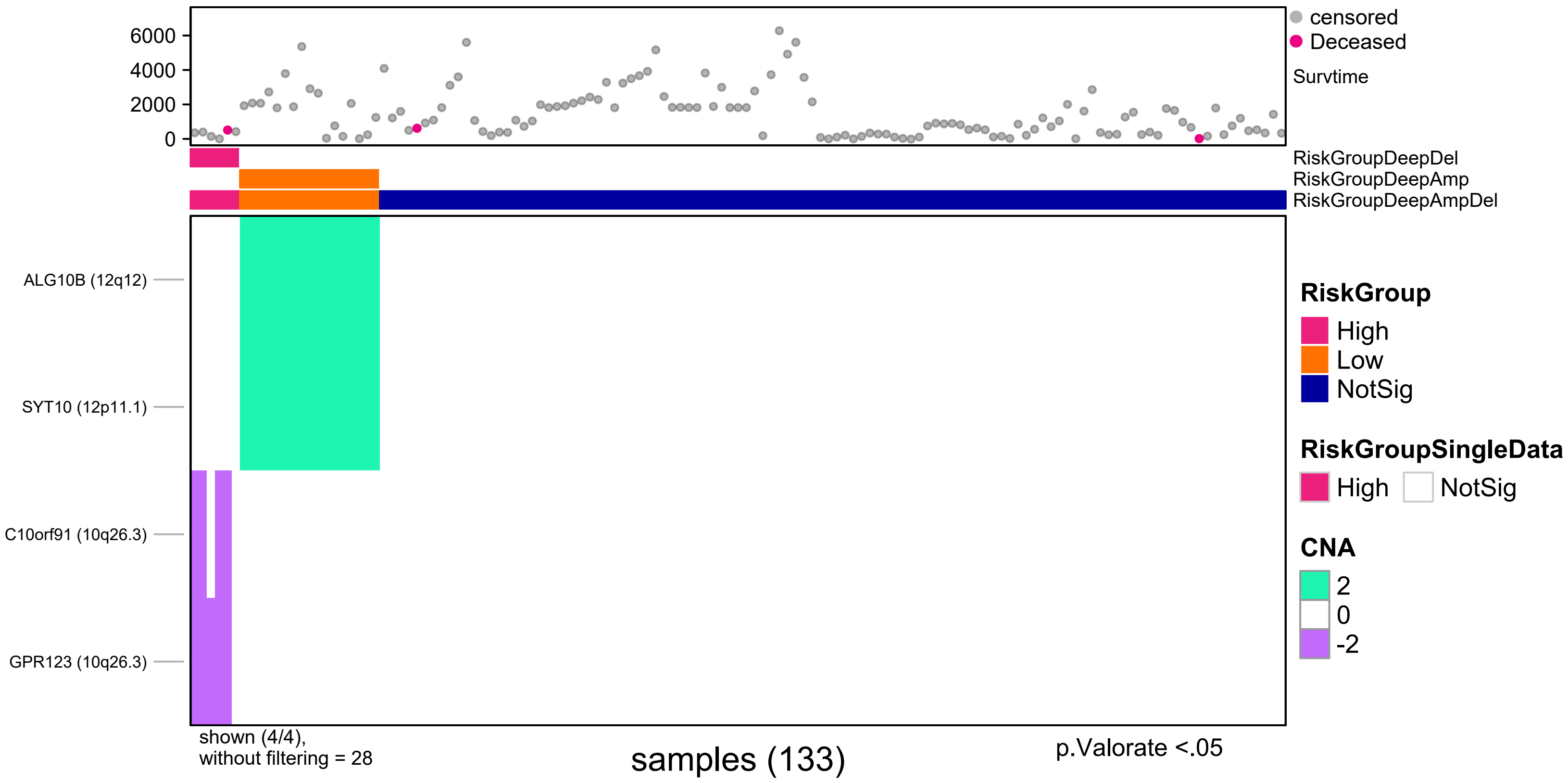

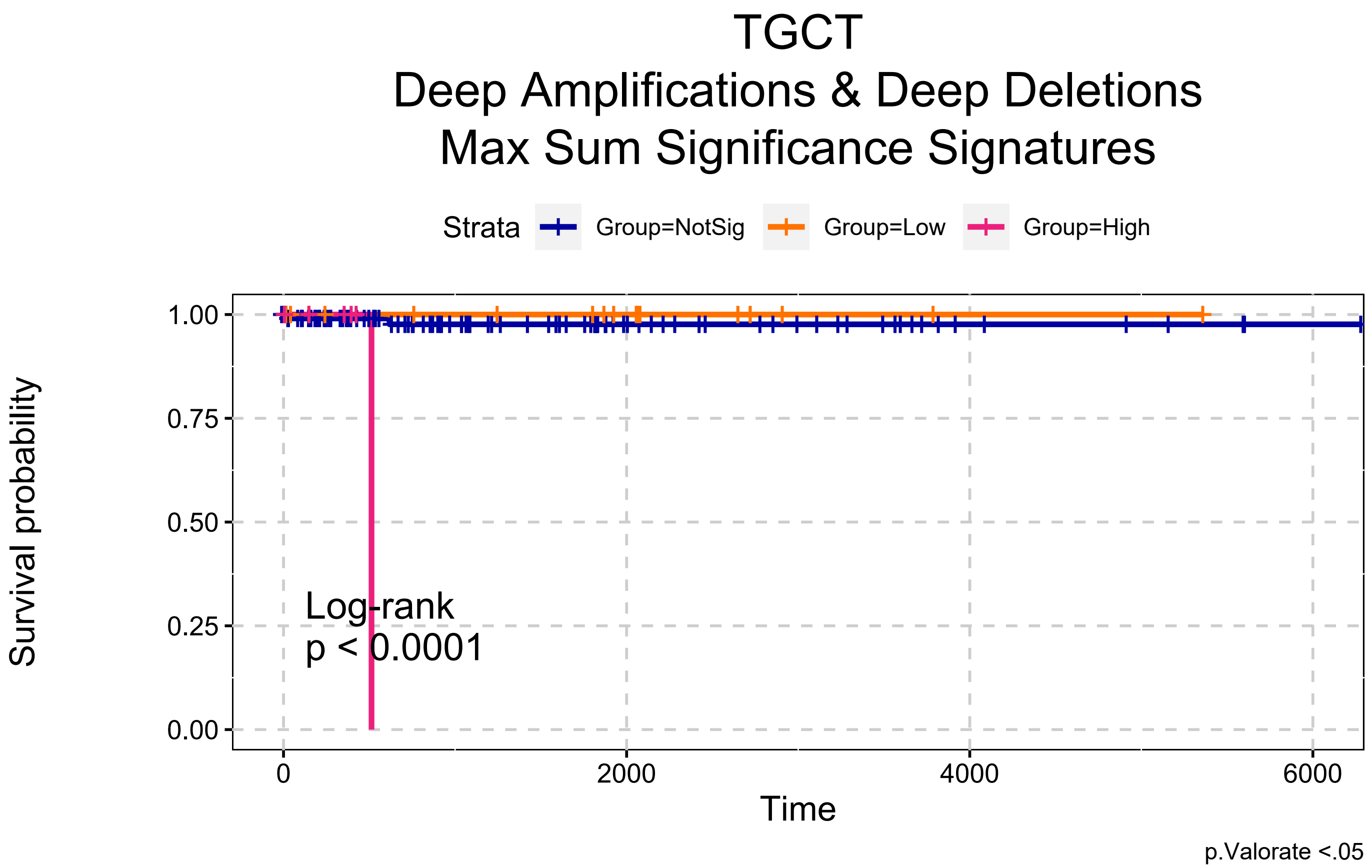

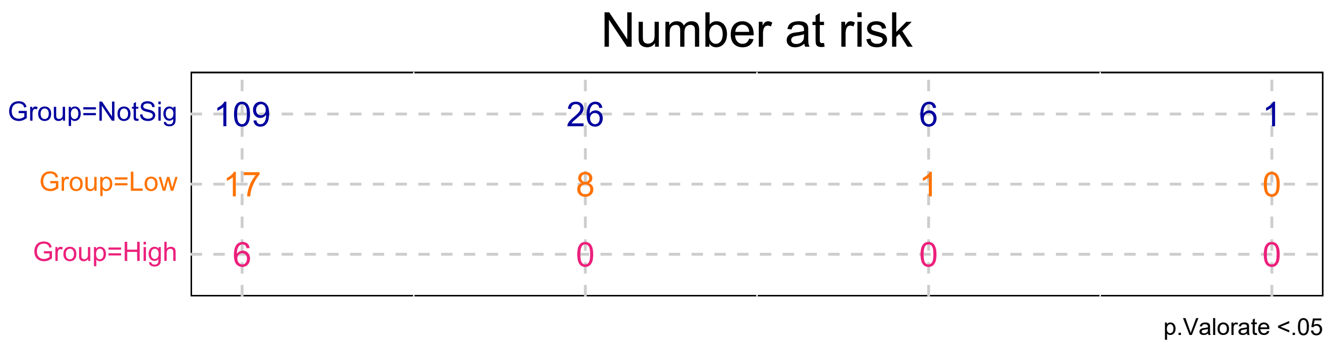

explanatory	beta	HR	L95	U95	p
Low	-17.24	0.00	0.00	Inf	1.00
High	3.68	39.61	2.16	725.82	0.01

n= 133, number of events =3
Score(logrank) test = p <.0001

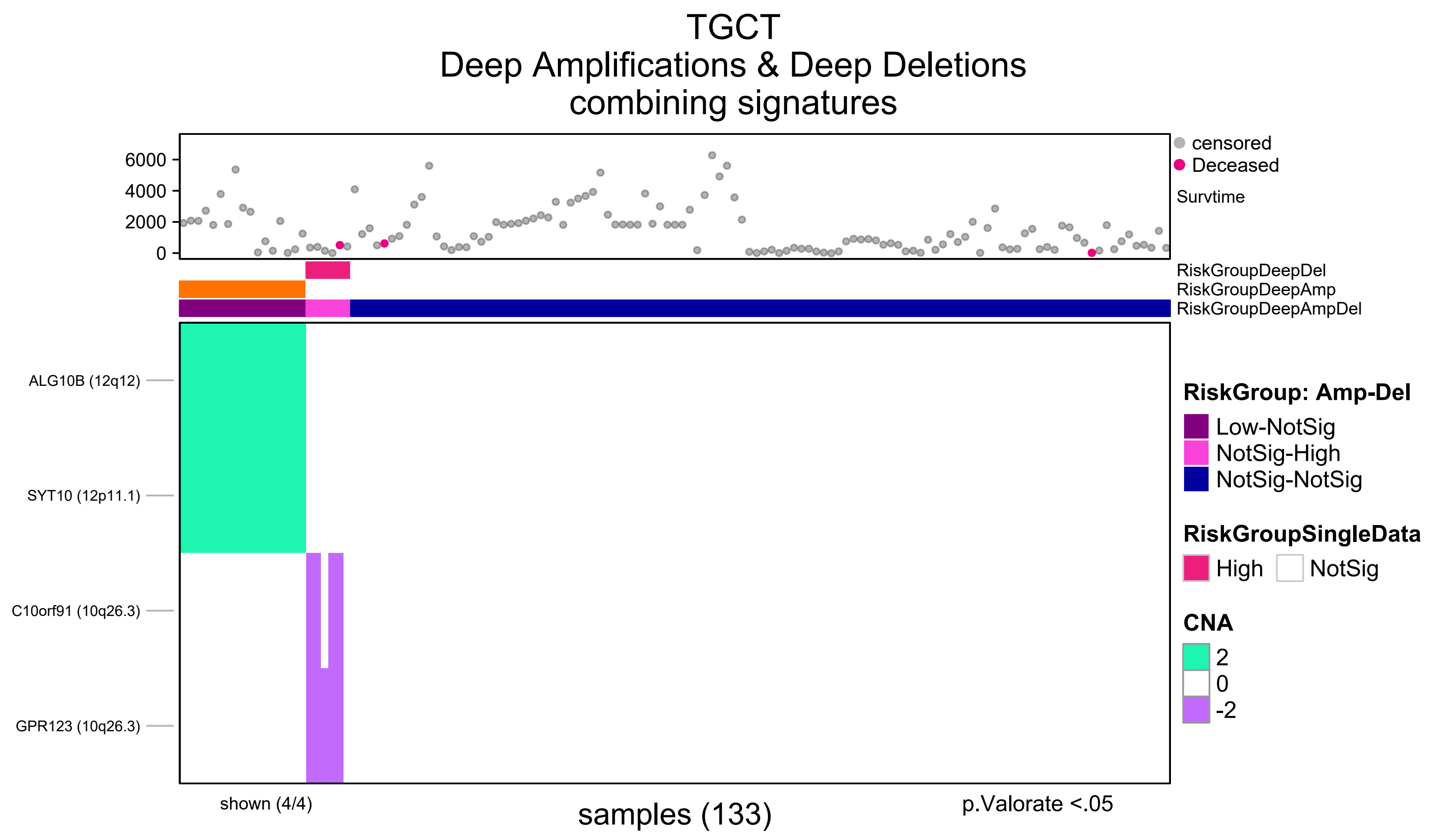
TGCT
Deep Amplifications & Deep Deletions
combining signatures
6000
4000
2000
0
censored
Deceased
Survtime
RiskGroupDeepDel
RiskGroupDeepAmp
RiskGroupDeepAmpDel
ALG10B (12q12)
SYT10 (12p11.1)
C10orf91 (10q26.3)
GPR123 (10q26.3)
RiskGroup: Amp-Del
Low-NotSig
NotSig-High
NotSig-NotSig
RiskGroupSingleData
High
NotSig
CNA
2
0
-2
shown (4/4)
samples (133)
p.Valorate <.05

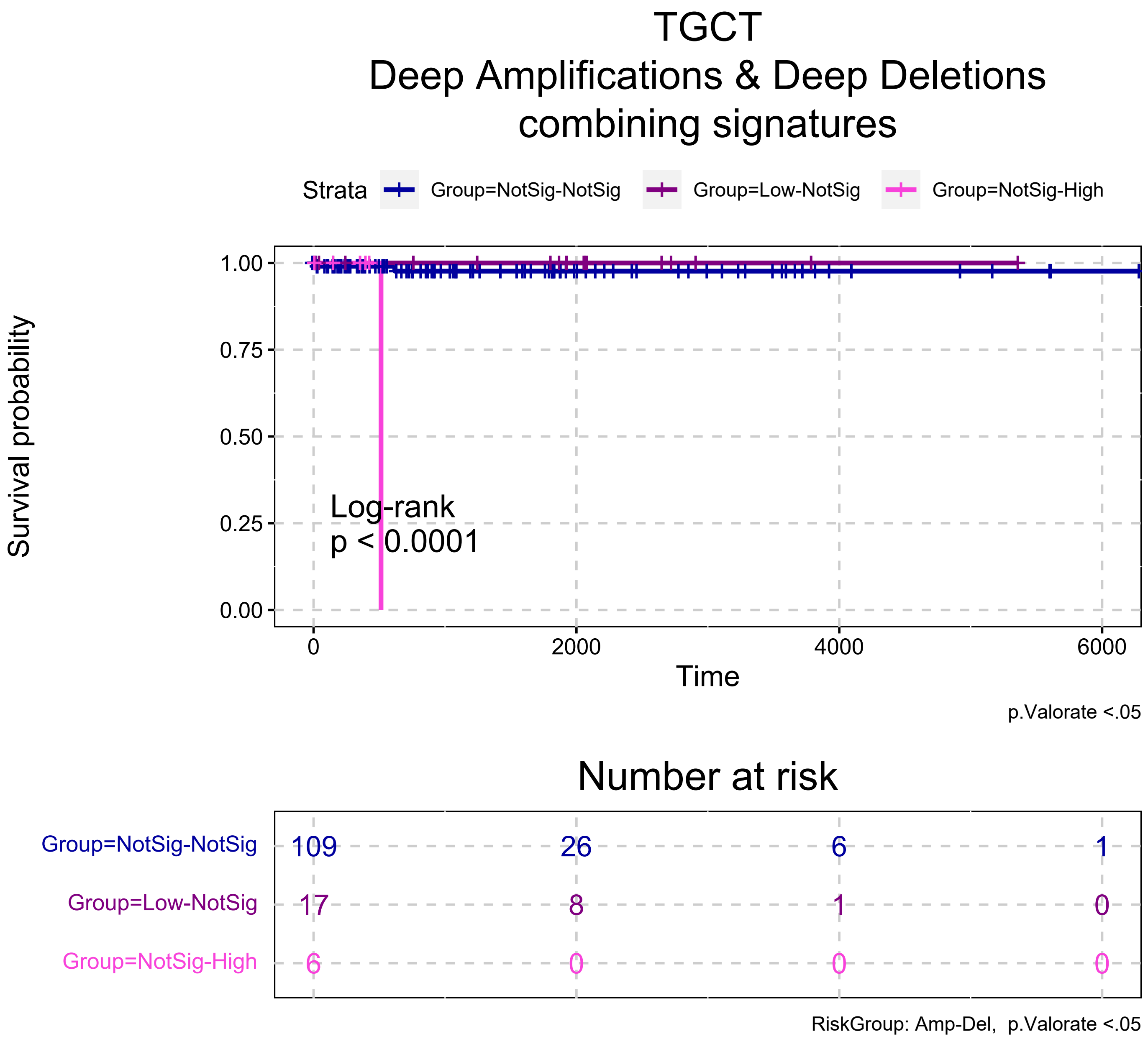

explanatory	beta	HR	L95	U95	p
Low-NotSig	-17.24	0.00	0.00	Inf	1.00
NotSig-High	3.68	39.61	2.16	725.82	0.01

n= 133, number of events =3
Score(logrank) test = p <.0001
