# Supplementary material for: Systematic Modeling of Risk-Associated Copy Number Alterations in Cancer
Source: Int J Mol Sci. 2024 Sep 27;25(19):10455. doi: 10.3390/ijms251910455 (PMC11477427; doi:10.3390/ijms251910455)
Supplement: Supplementary file 1 [file ijms-25-10455-s001.zip › THCASignatureV12-sinSombreado.pdf]

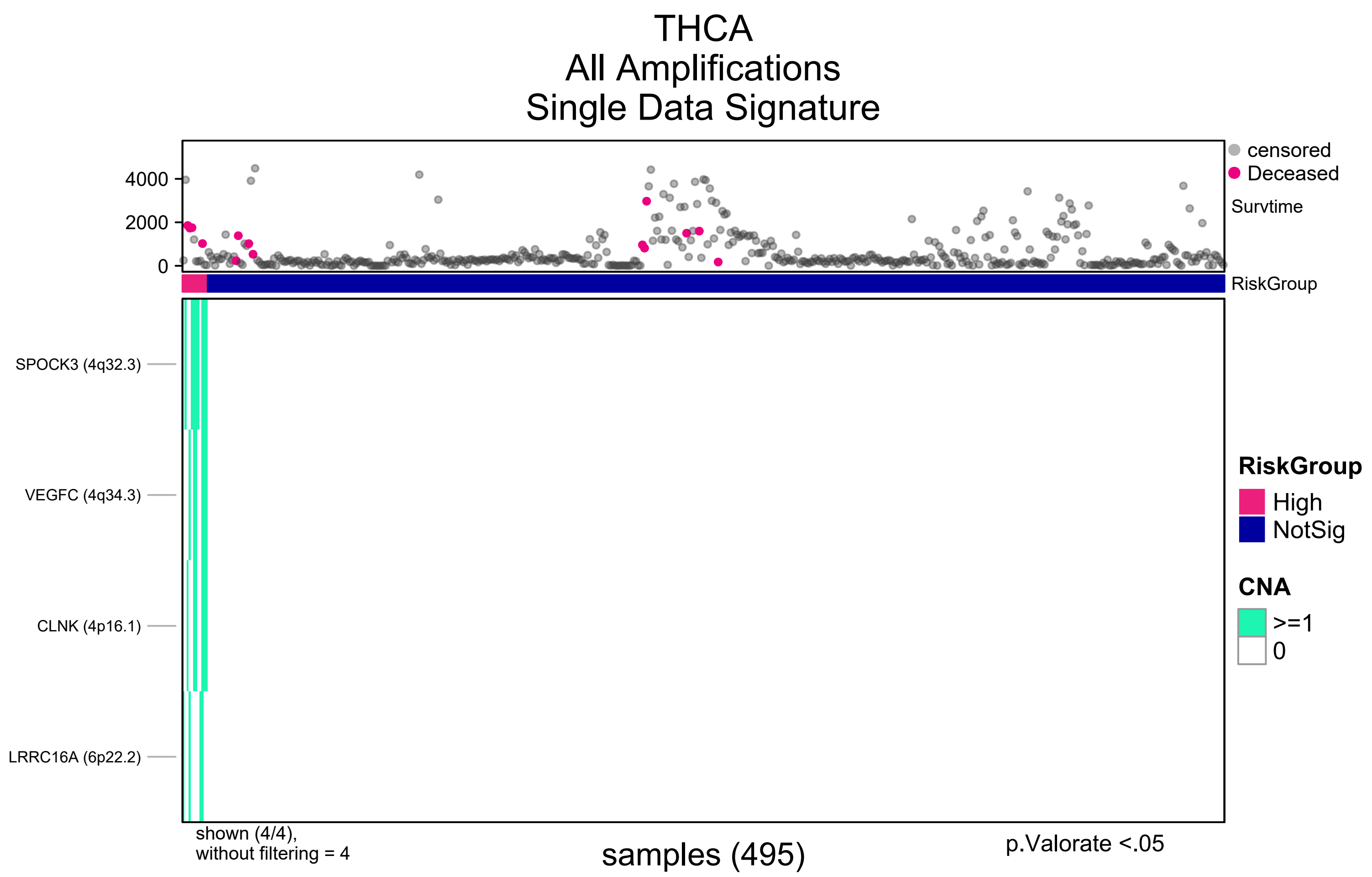
THCA
All Amplifications
Single Data Signature
4000
2000
0
censored
Deceased
Survtime
RiskGroup
SPOCK3 (4q32.3)
VEGFC (4q34.3)
CLNK (4p16.1)
LRRC16A (6p22.2)
RiskGroup
High
NotSig
CNA
>=1
0
shown (4/4),
without filtering = 4
samples (495)
p.Valorate <.05

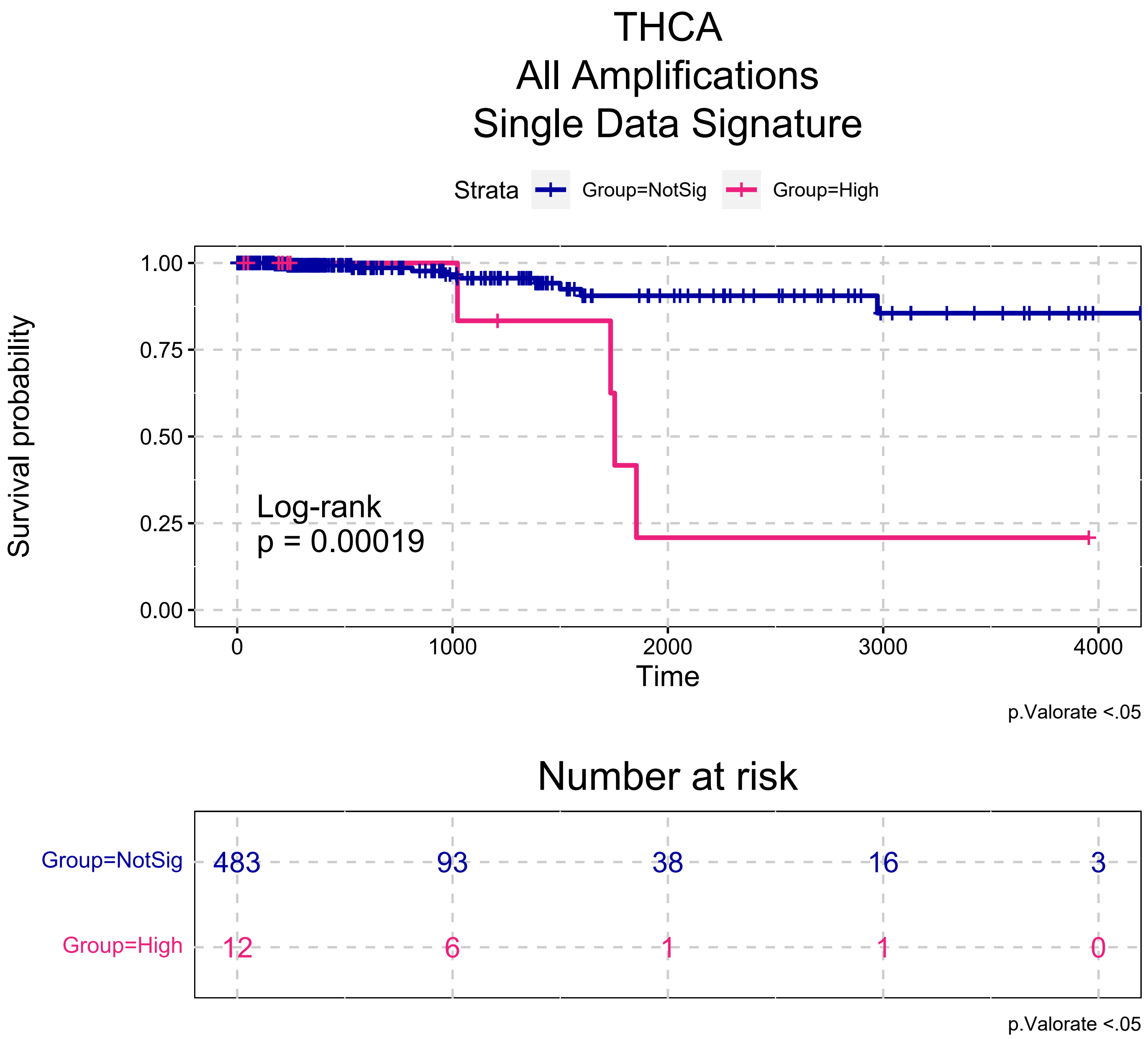

explanatory	beta	HR	L95	U95	p
High	1.92	6.85	2.12	22.09	0.00

n= 495, number of events =14
Score(logrank) test = 0

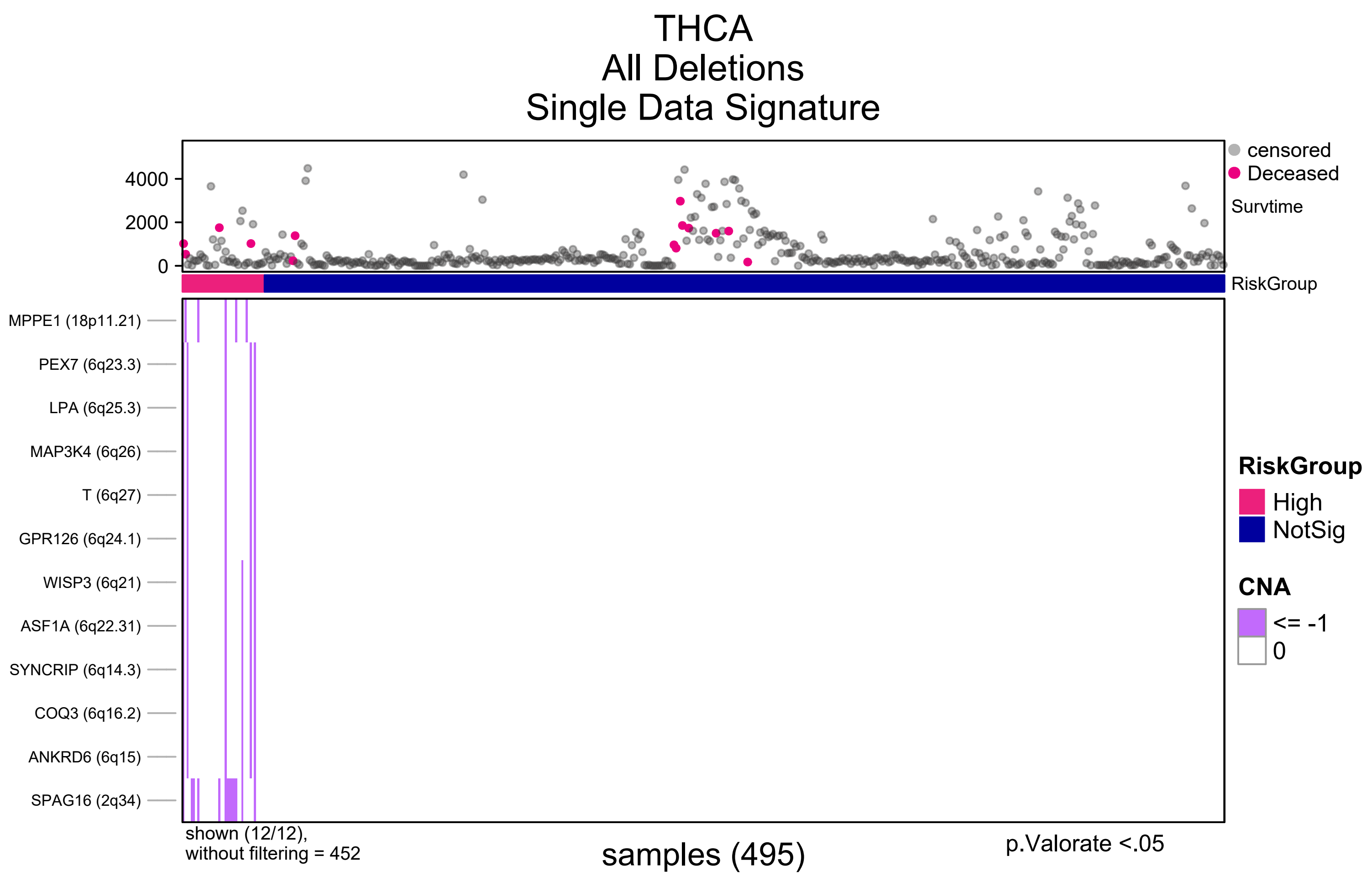

THCA
All Deletions
Single Data Signature
4000
2000
0
censored
Deceased
Survtime
RiskGroup
MPPE1 (18p11.21)
PEX7 (6q23.3)
LPA (6q25.3)
MAP3K4 (6q26)
T (6q27)
GPR126 (6q24.1)
WISP3 (6q21)
ASF1A (6q22.31)
SYNCRIP (6q14.3)
COQ3 (6q16.2)
ANKRD6 (6q15)
SPAG16 (2q34)
RiskGroup
High
NotSig
CNA
<= -1
0
shown (12/12),
without filtering = 452
samples (495)
p.Valorate <.05

THCA
All Deletions
Single Data Signature

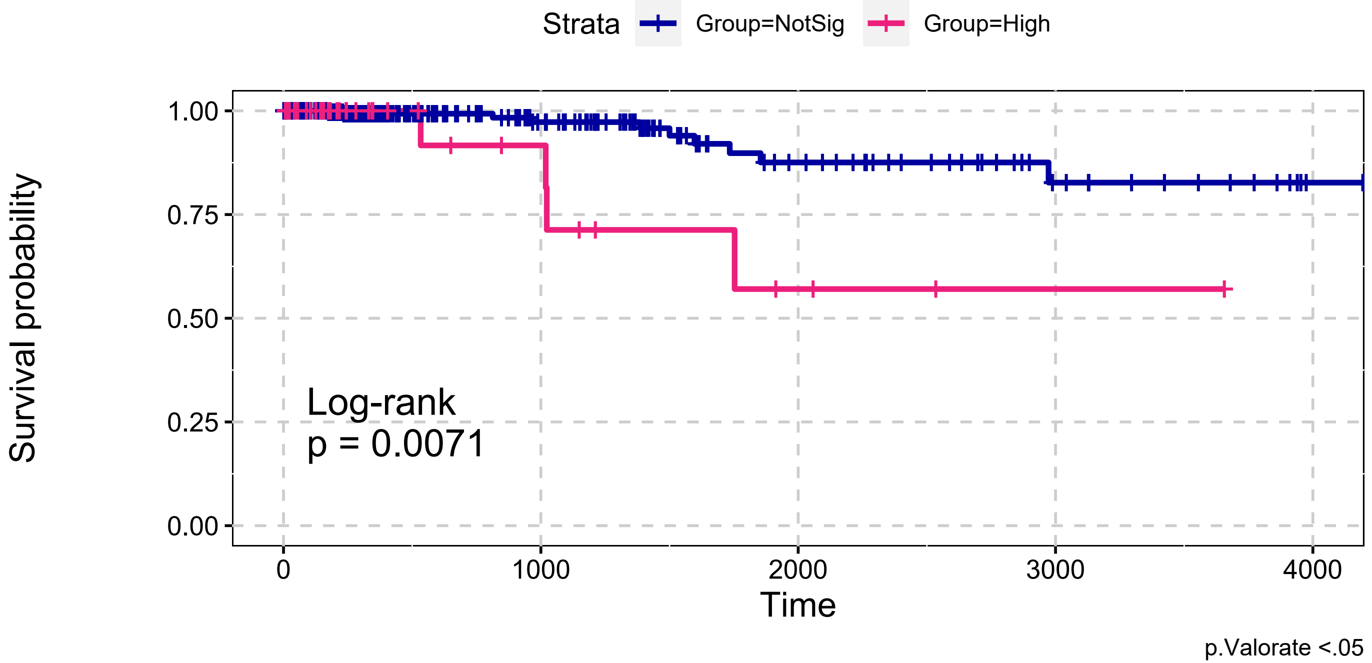

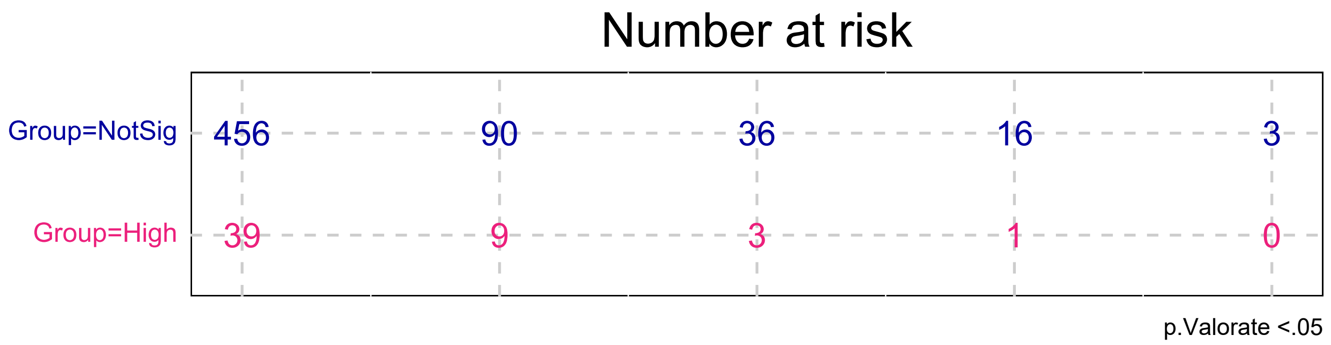

explanatory	beta	HR	L95	U95	p
High	1.47	4.33	1.35	13.88	0.01

n= 495, number of events =14
Score(logrank) test = 0.007

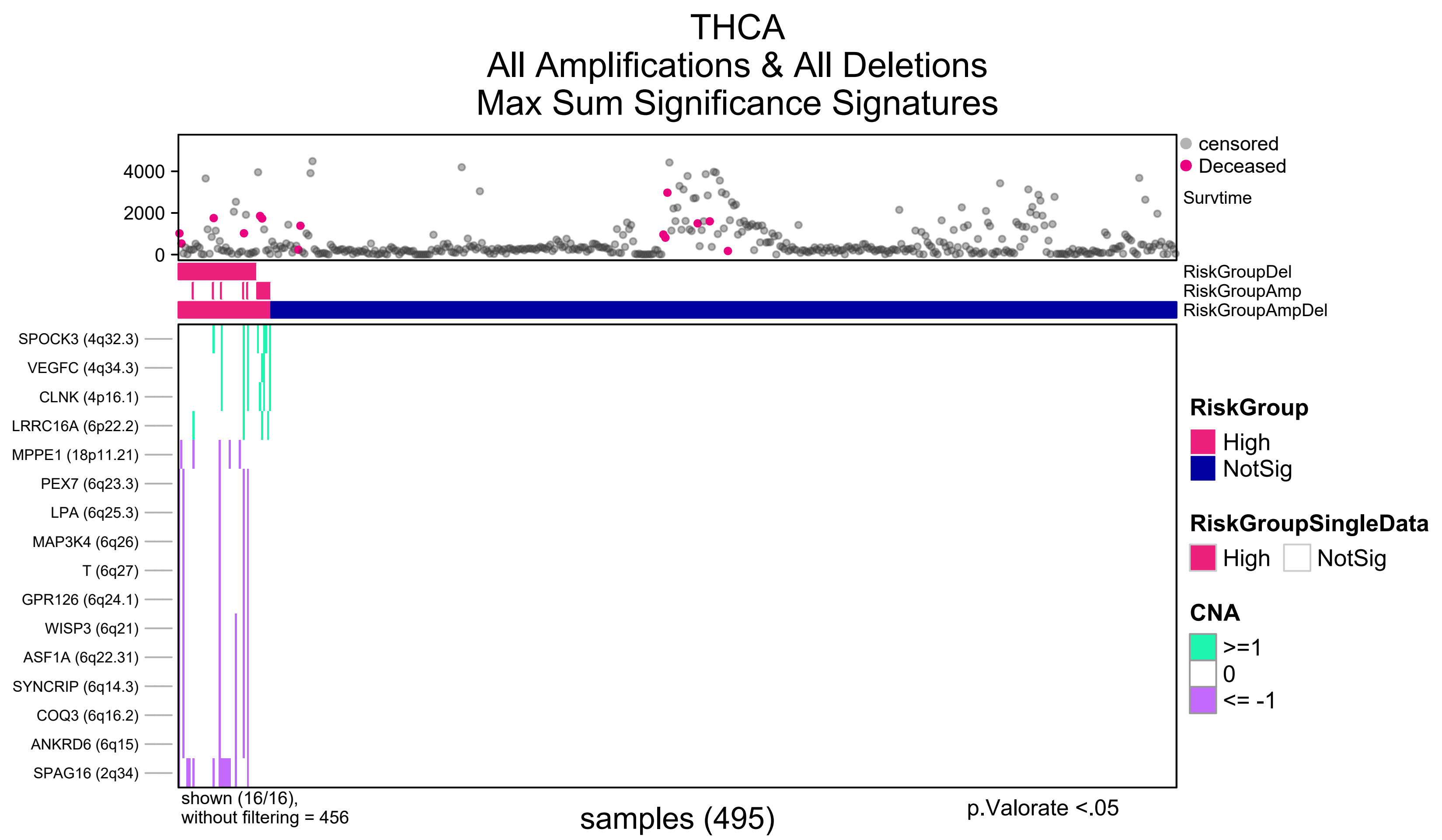

THCA
All Amplifications & All Deletions
Max Sum Significance Signatures
4000
2000
0
censored
Deceased
Survtime
RiskGroupDel
RiskGroupAmp
RiskGroupAmpDel
SPOCK3 (4q32.3)
VEGFC (4q34.3)
CLNK (4p16.1)
LRRC16A (6p22.2)
MPPE1 (18p11.21)
PEX7 (6q23.3)
LPA (6q25.3)
MAP3K4 (6q26)
T (6q27)
GPR126 (6q24.1)
WISP3 (6q21)
ASF1A (6q22.31)
SYNCRIP (6q14.3)
COQ3 (6q16.2)
ANKRD6 (6q15)
SPAG16 (2q34)
RiskGroup
High
NotSig
RiskGroupSingleData
High
NotSig
CNA
>=1
0
<= -1
shown (16/16),
without filtering = 456
samples (495)
p.Valorate <.05

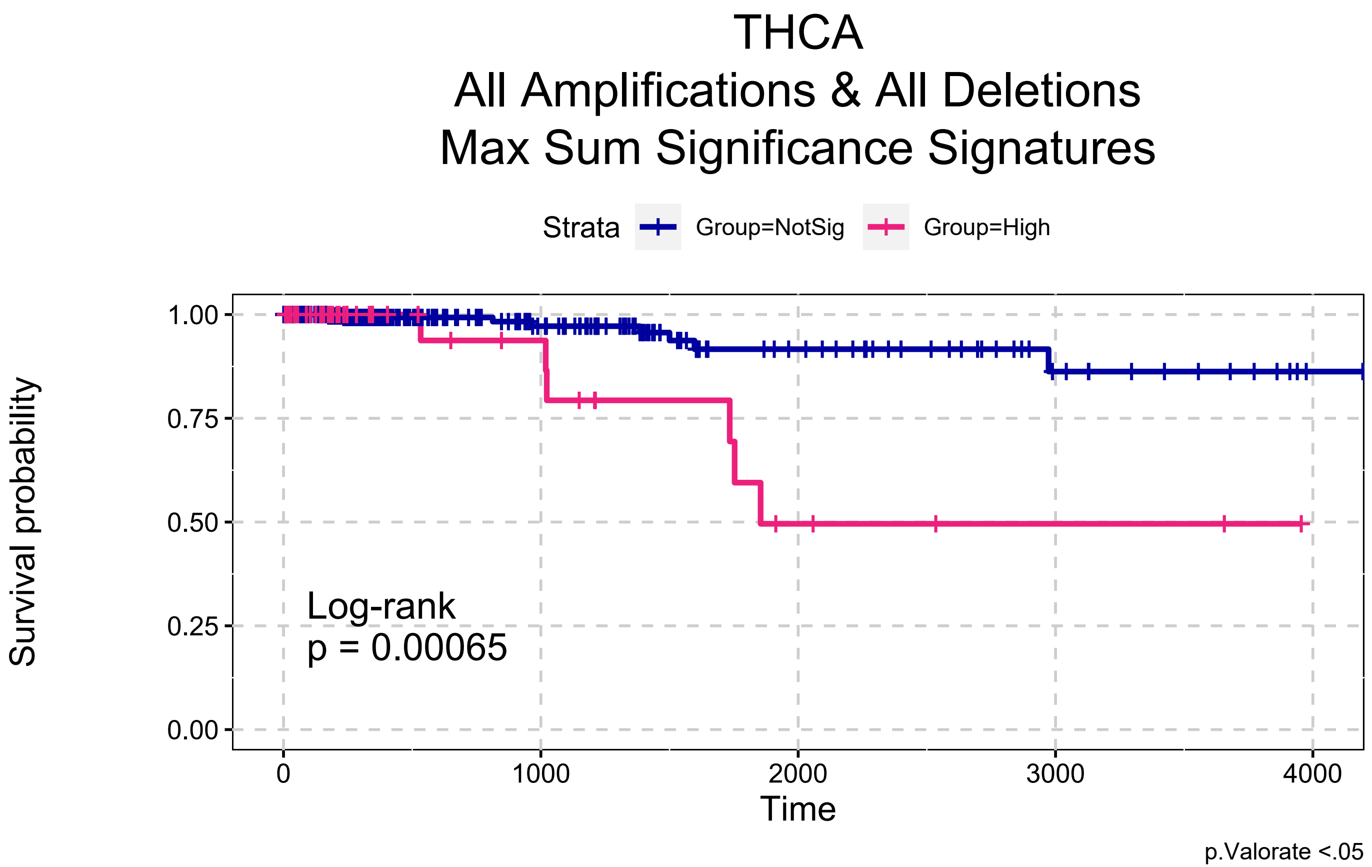

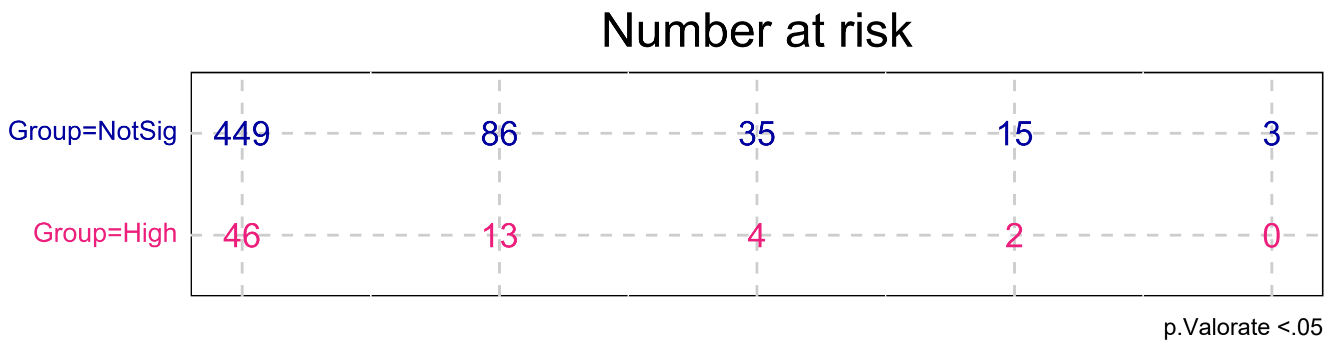

explanatory	beta	HR	L95	U95	p
High	1.66	5.25	1.81	15.23	0.00

n= 495, number of events =14
Score(logrank) test = 0.001

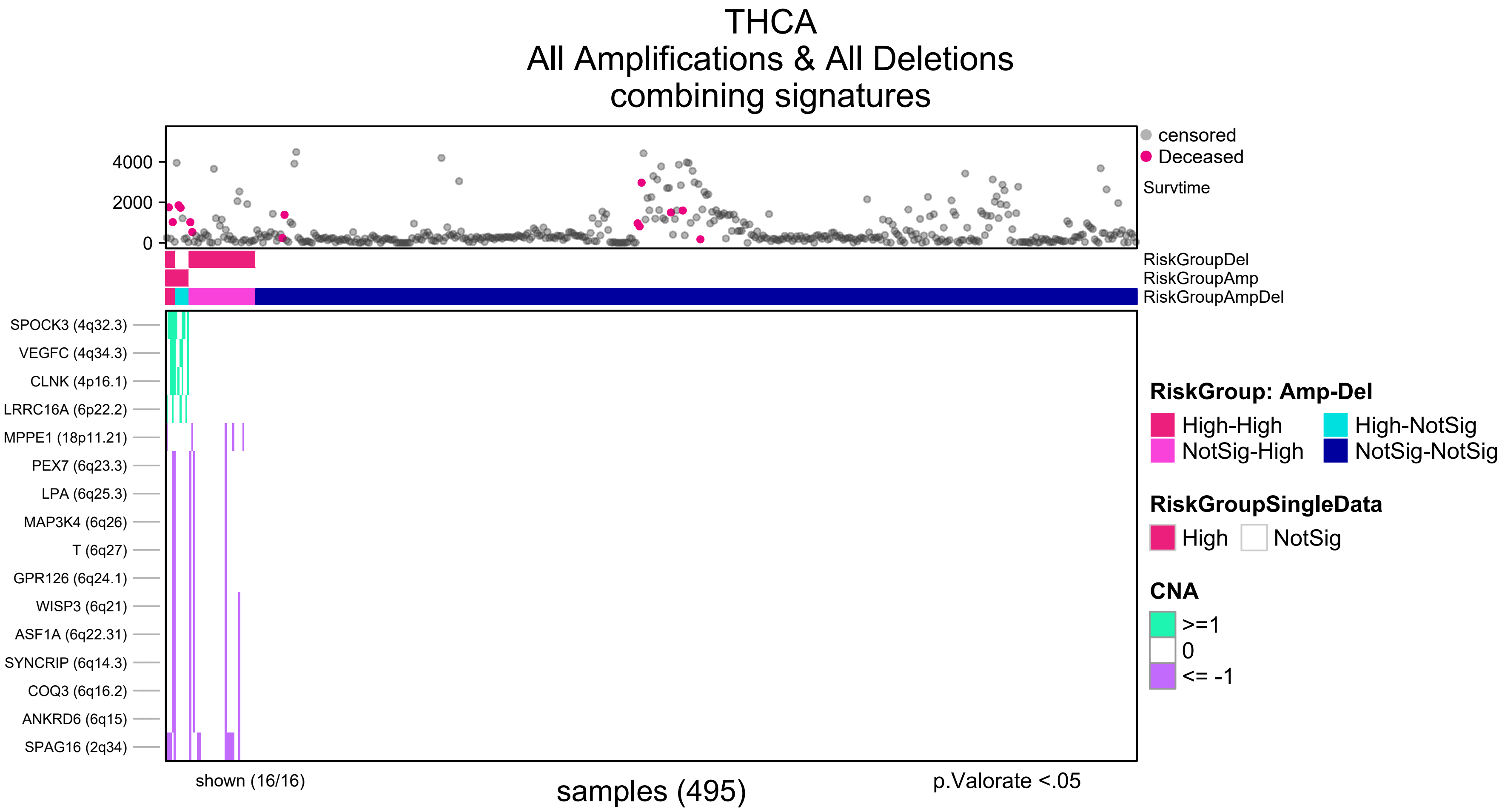
THCA
All Amplifications & All Deletions
combining signatures
4000
2000
0
censored
Deceased
Survtime
RiskGroupDel
RiskGroupAmp
RiskGroupAmpDel
SPOCK3 (4q32.3)
VEGFC (4q34.3)
CLNK (4p16.1)
LRRC16A (6p22.2)
MPPE1 (18p11.21)
PEX7 (6q23.3)
LPA (6q25.3)
MAP3K4 (6q26)
T (6q27)
GPR126 (6q24.1)
WISP3 (6q21)
ASF1A (6q22.31)
SYNCRIP (6q14.3)
COQ3 (6q16.2)
ANKRD6 (6q15)
SPAG16 (2q34)
RiskGroup: Amp-Del
High-High
High-NotSig
NotSig-High
NotSig-NotSig
RiskGroupSingleData
High
NotSig
CNA
>=1
0
<= -1
shown (16/16)
samples (495)
p.Valorate <.05

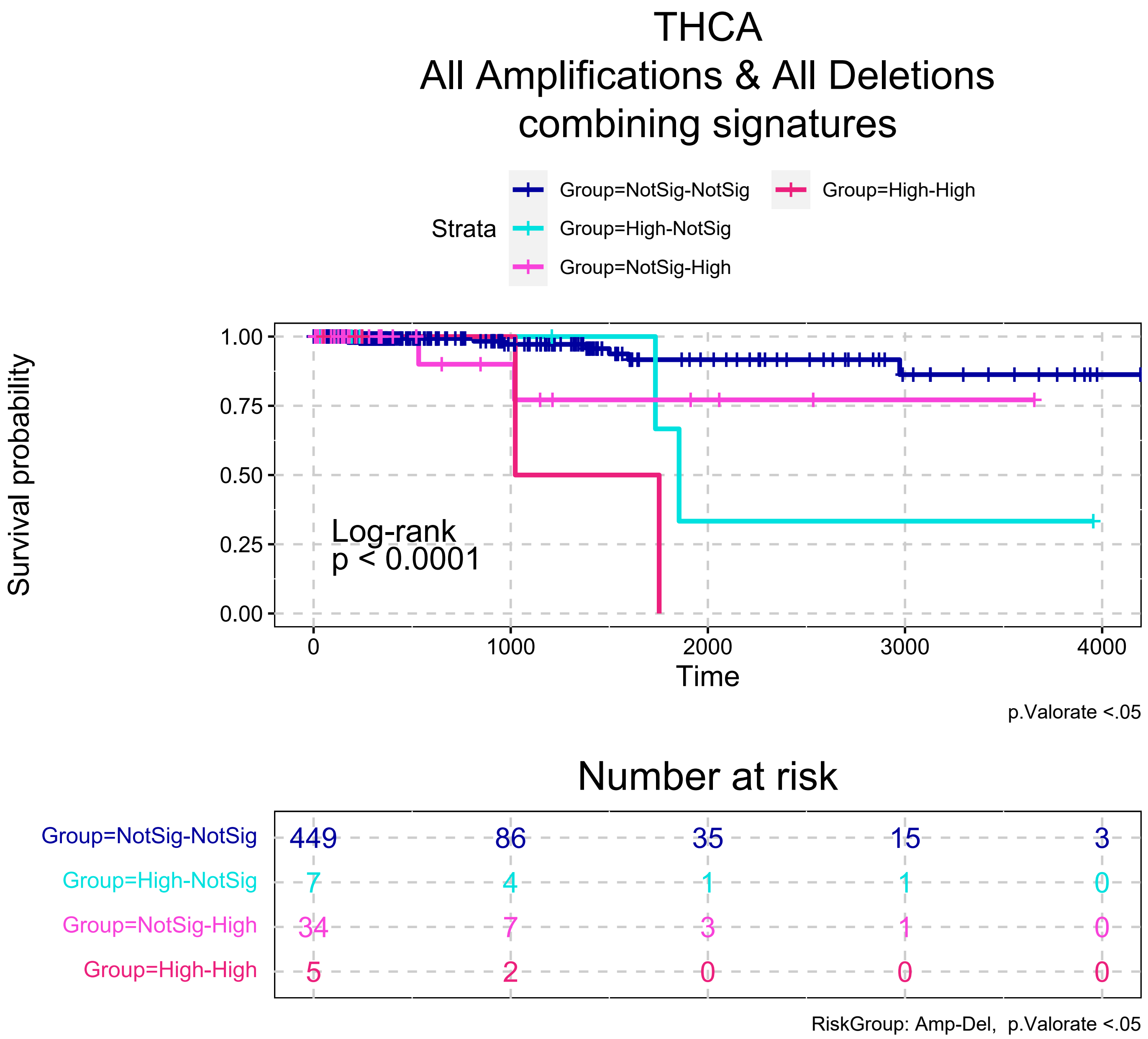

explanatory	beta	HR	L95	U95	p
High-NotSig	1.68	5.38	1.13	25.63	0.03
NotSig-High	1.15	3.14	0.67	14.85	0.15
High-High	2.72	15.20	3.15	73.41	0.00

n= 495, number of events =14
Score(logrank) test = p <.0001
