# Supplementary material for: Systematic Modeling of Risk-Associated Copy Number Alterations in Cancer
Source: Int J Mol Sci. 2024 Sep 27;25(19):10455. doi: 10.3390/ijms251910455 (PMC11477427; doi:10.3390/ijms251910455)
Supplement: Supplementary file 1 [file ijms-25-10455-s001.zip › THYMSignatureV12-sinSombreado.pdf]

THYM
All Amplifications
Single Data Signature

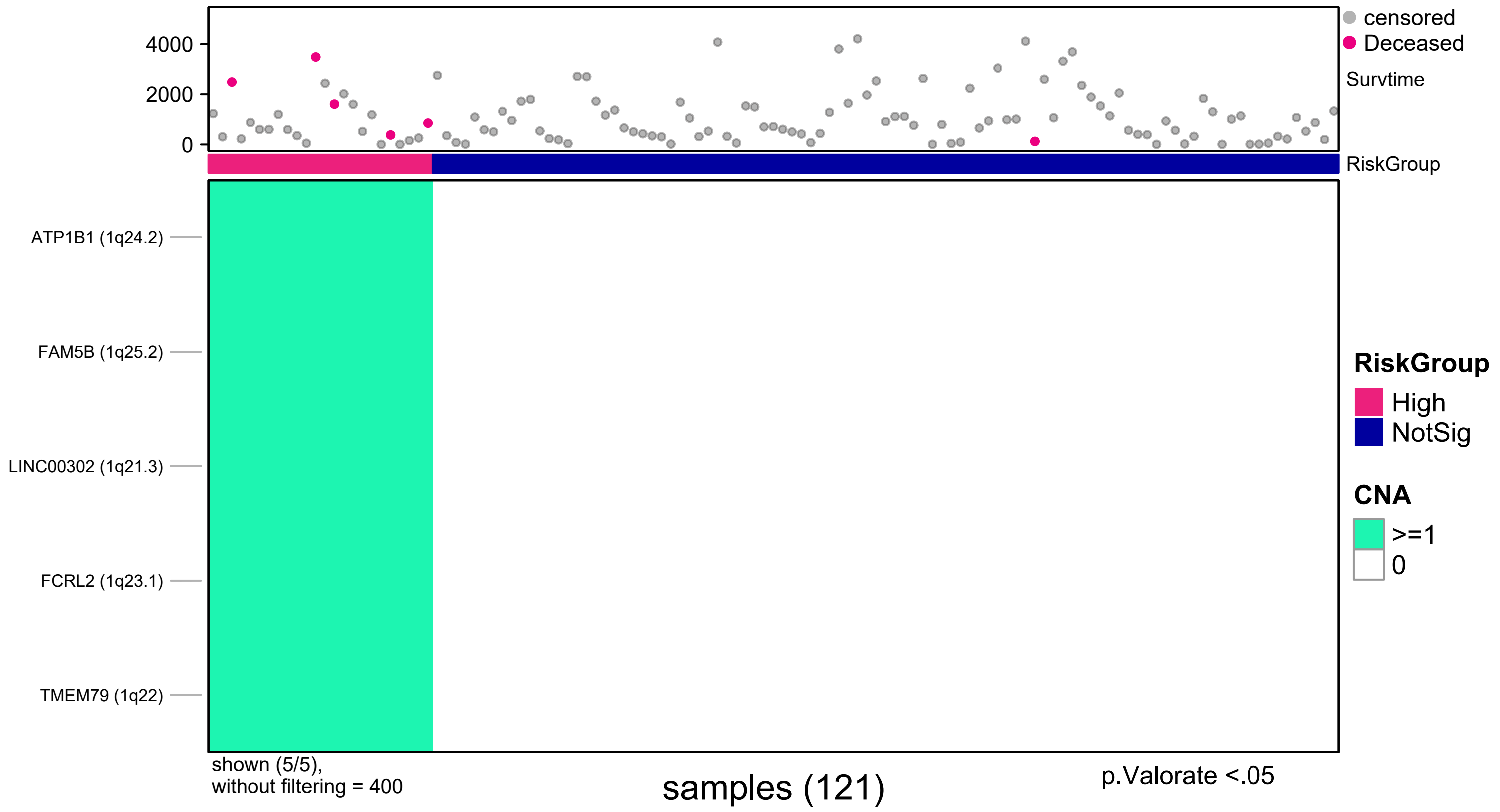

THYM
All Amplifications
Single Data Signature

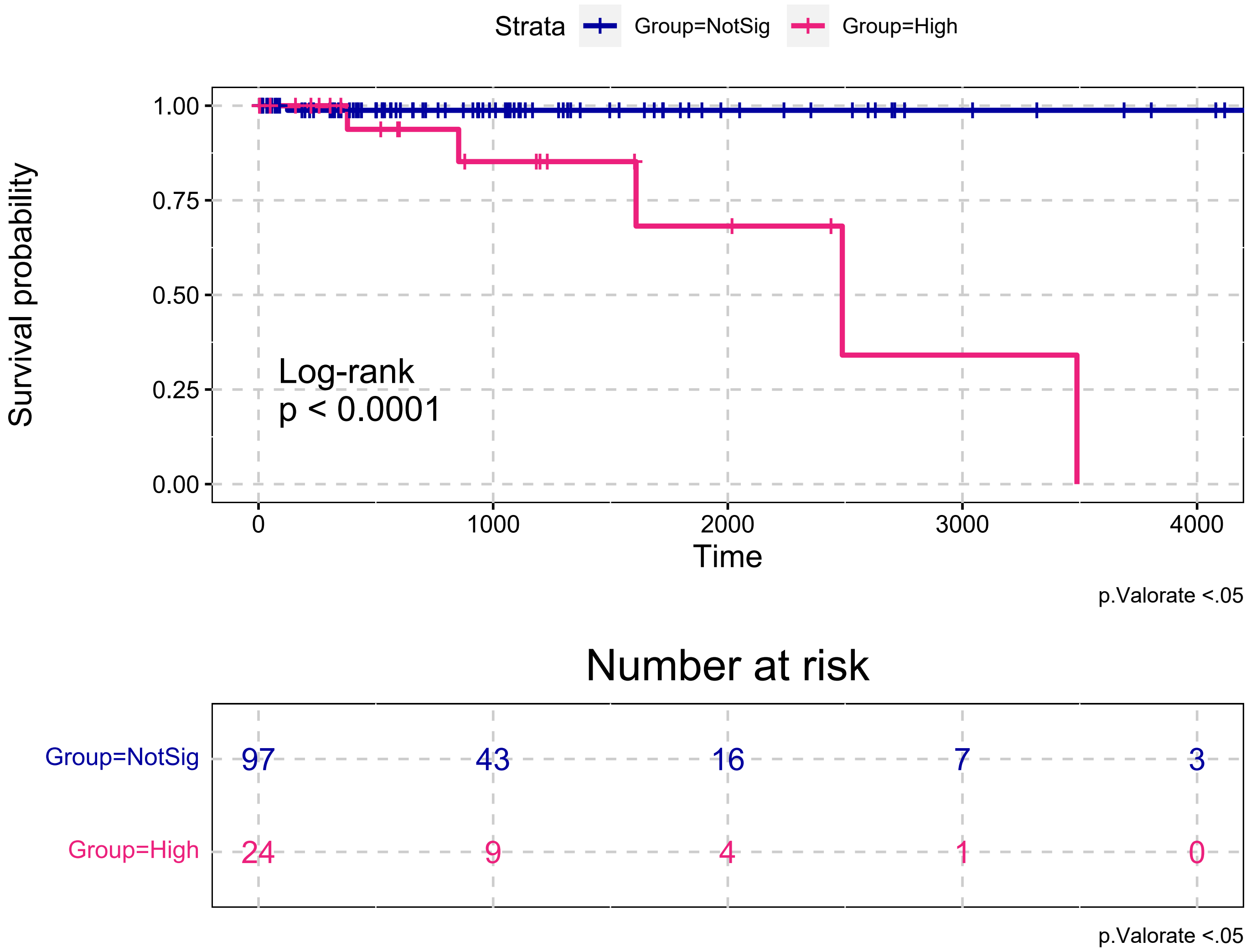

explanatory	beta	HR	L95	U95	p
High	3.18	24.08	2.80	206.97	0.00

n= 121, number of events =6
Score(logrank) test = p <.0001

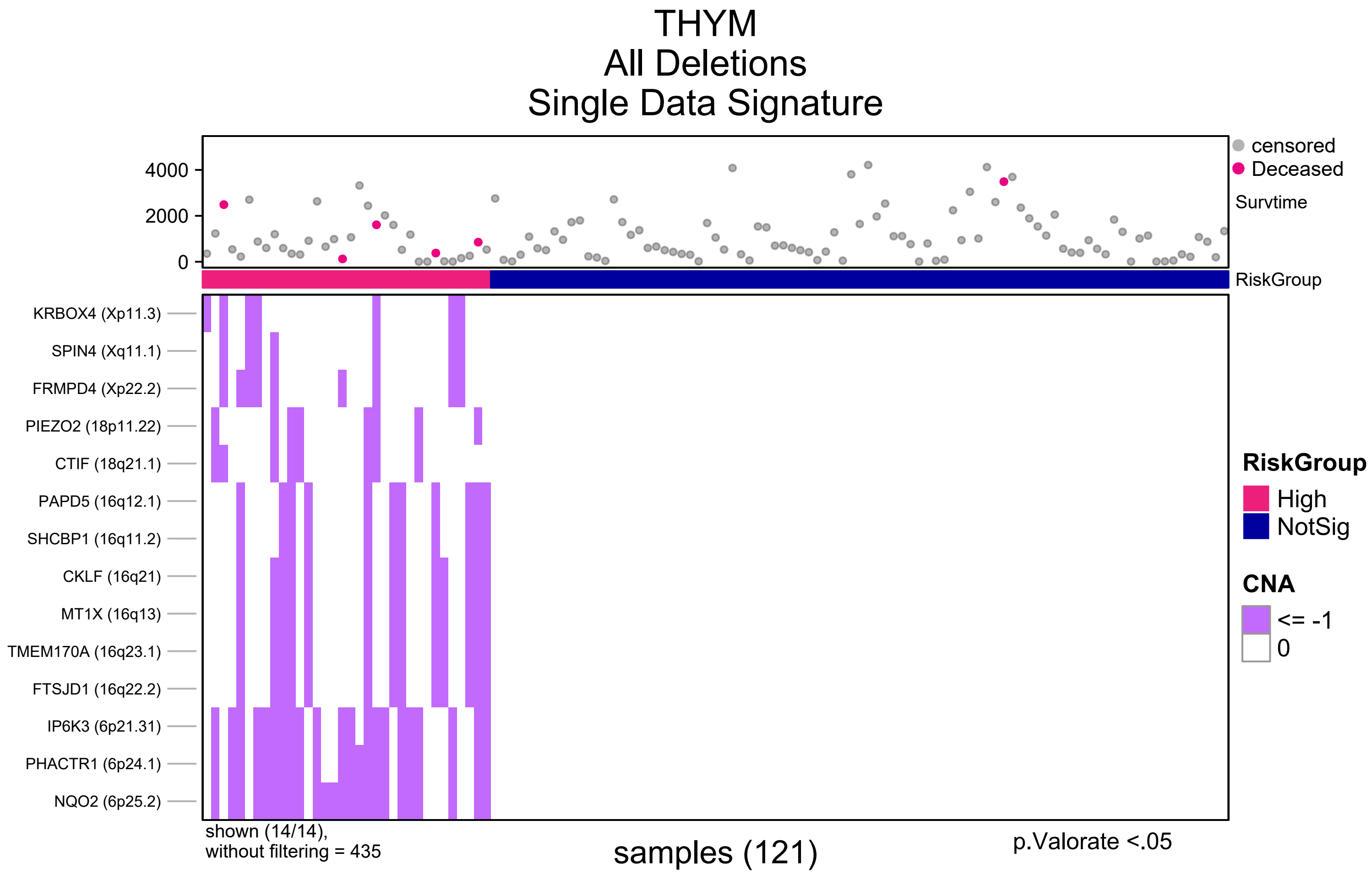

THYM
All Deletions
Single Data Signature
4000
2000
0
censored
Deceased
Survtime
RiskGroup
KRBOX4 (Xp11.3)
SPIN4 (Xq11.1)
FRMPD4 (Xp22.2)
PIEZO2 (18p11.22)
CTIF (18q21.1)
PAPD5 (16q12.1)
SHCBP1 (16q11.2)
CKLF (16q21)
MT1X (16q13)
TMEM170A (16q23.1)
FTSJD1 (16q22.2)
IP6K3 (6p21.31)
PHACTR1 (6p24.1)
NQO2 (6p25.2)
RiskGroup
High
NotSig
CNA
<= -1
0
shown (14/14),
without filtering = 435
samples (121)
p.Valorate <.05

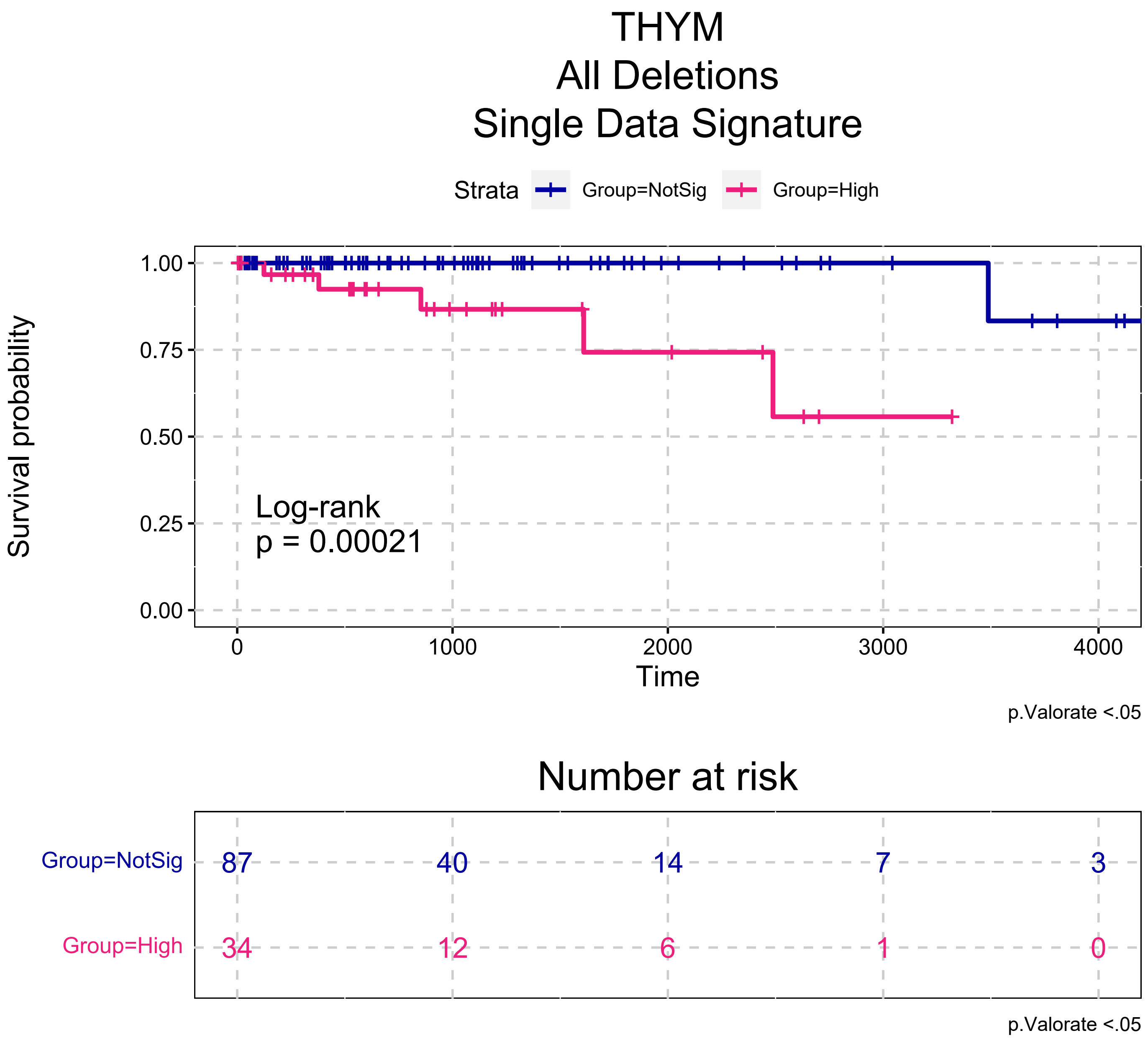

explanatory	beta	HR	L95	U95	p
High	21.85	3100339420.59	0.00	Inf	1.00

n= 121, number of events =6
Score(logrank) test = 0

THYM
All Amplifications & All Deletions
Max Sum Significance Signatures

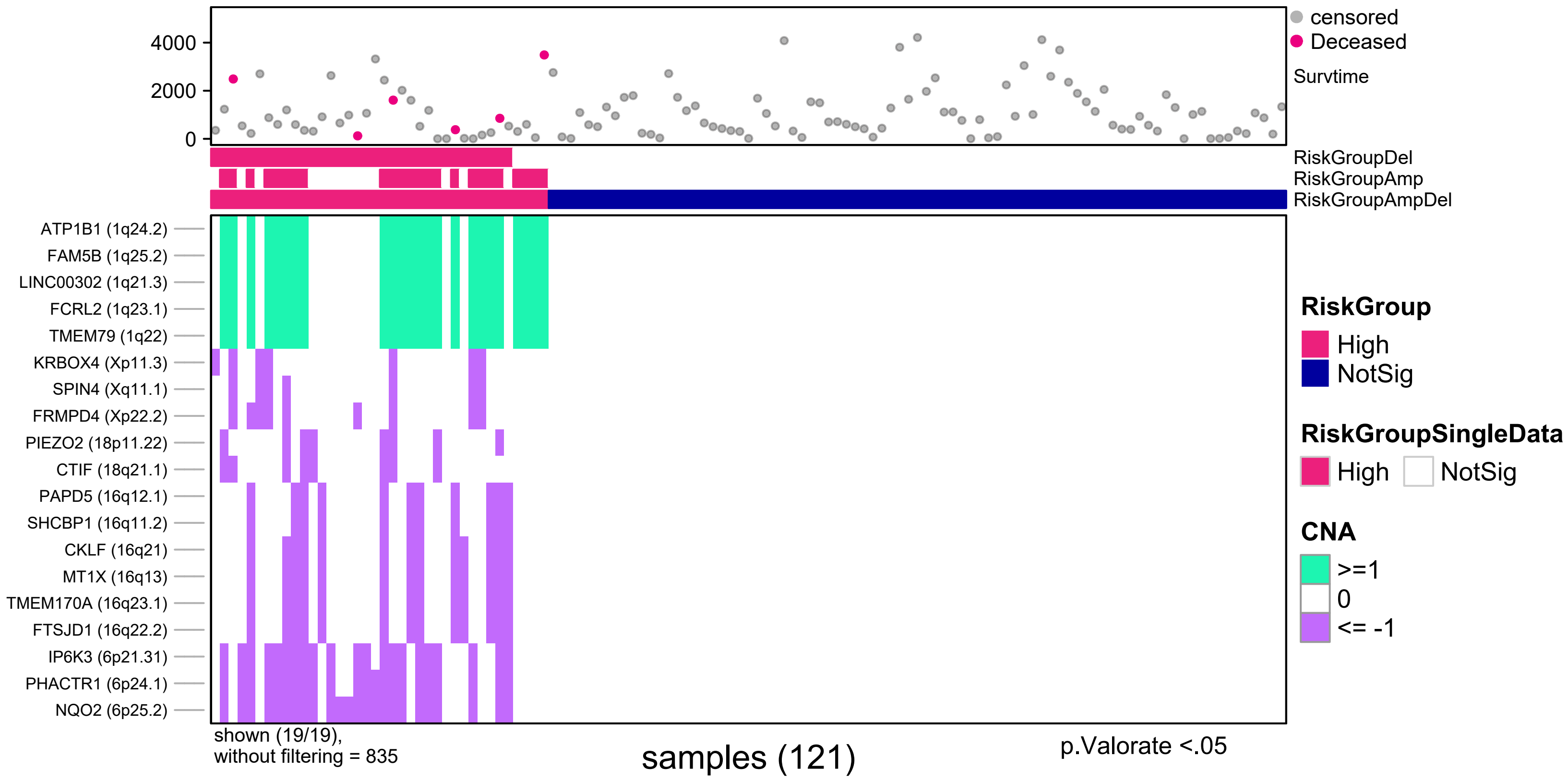

THYM
All Amplifications & All Deletions
Max Sum Significance Signatures

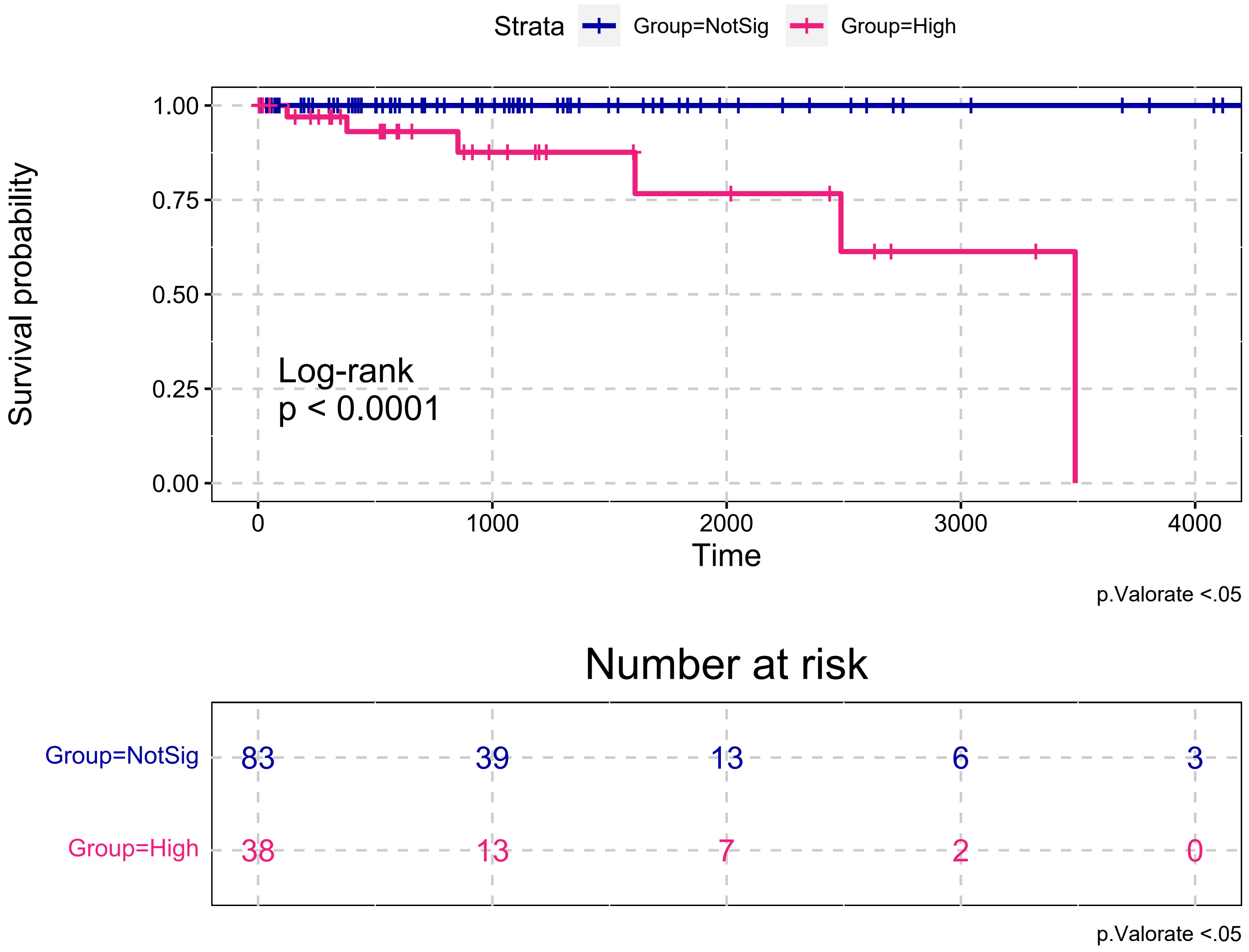

explanatory	beta	HR	L95	U95	p
High	21.77	2845650636.21	0.00	Inf	1.00

n= 121, number of events =6
Score(logrank) test = p <.0001

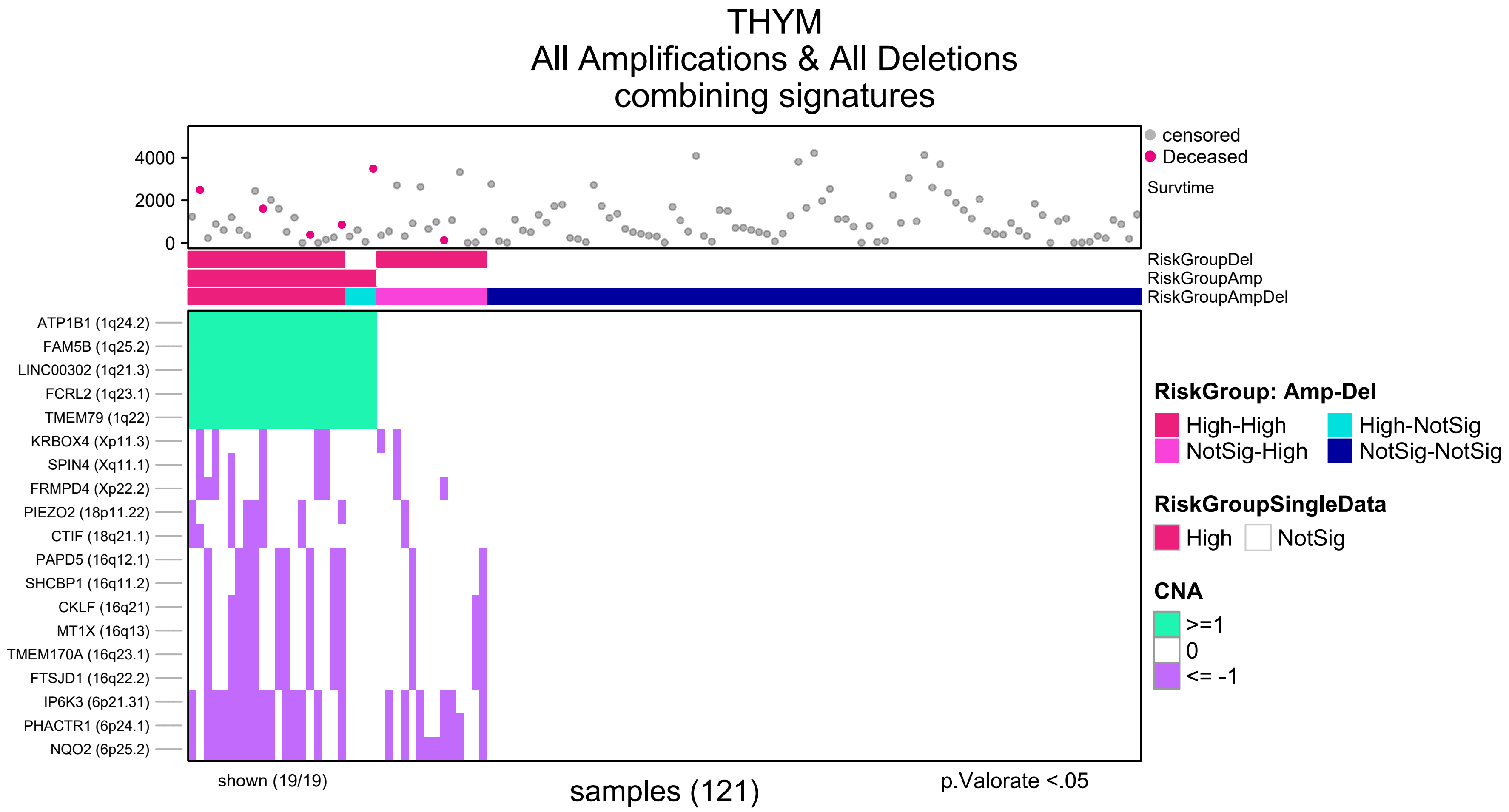
THYM
All Amplifications & All Deletions
combining signatures
4000
2000
0
censored
Deceased
Survtime
RiskGroupDel
RiskGroupAmp
RiskGroupAmpDel
ATP1B1 (1q24.2)
FAM5B (1q25.2)
LINC00302 (1q21.3)
FCRL2 (1q23.1)
TMEM79 (1q22)
KRBOX4 (Xp11.3)
SPIN4 (Xq11.1)
FRMPD4 (Xp22.2)
PIEZO2 (18p11.22)
CTIF (18q21.1)
PAPD5 (16q12.1)
SHCBP1 (16q11.2)
CKLF (16q21)
MT1X (16q13)
TMEM170A (16q23.1)
FTSJD1 (16q22.2)
IP6K3 (6p21.31)
PHACTR1 (6p24.1)
NQO2 (6p25.2)
RiskGroup: Amp-Del
High-High
High-NotSig
NotSig-High
NotSig-NotSig
RiskGroupSingleData
High
NotSig
CNA
>=1
0
<= -1
shown (19/19)
samples (121)
p.Valorate <.05

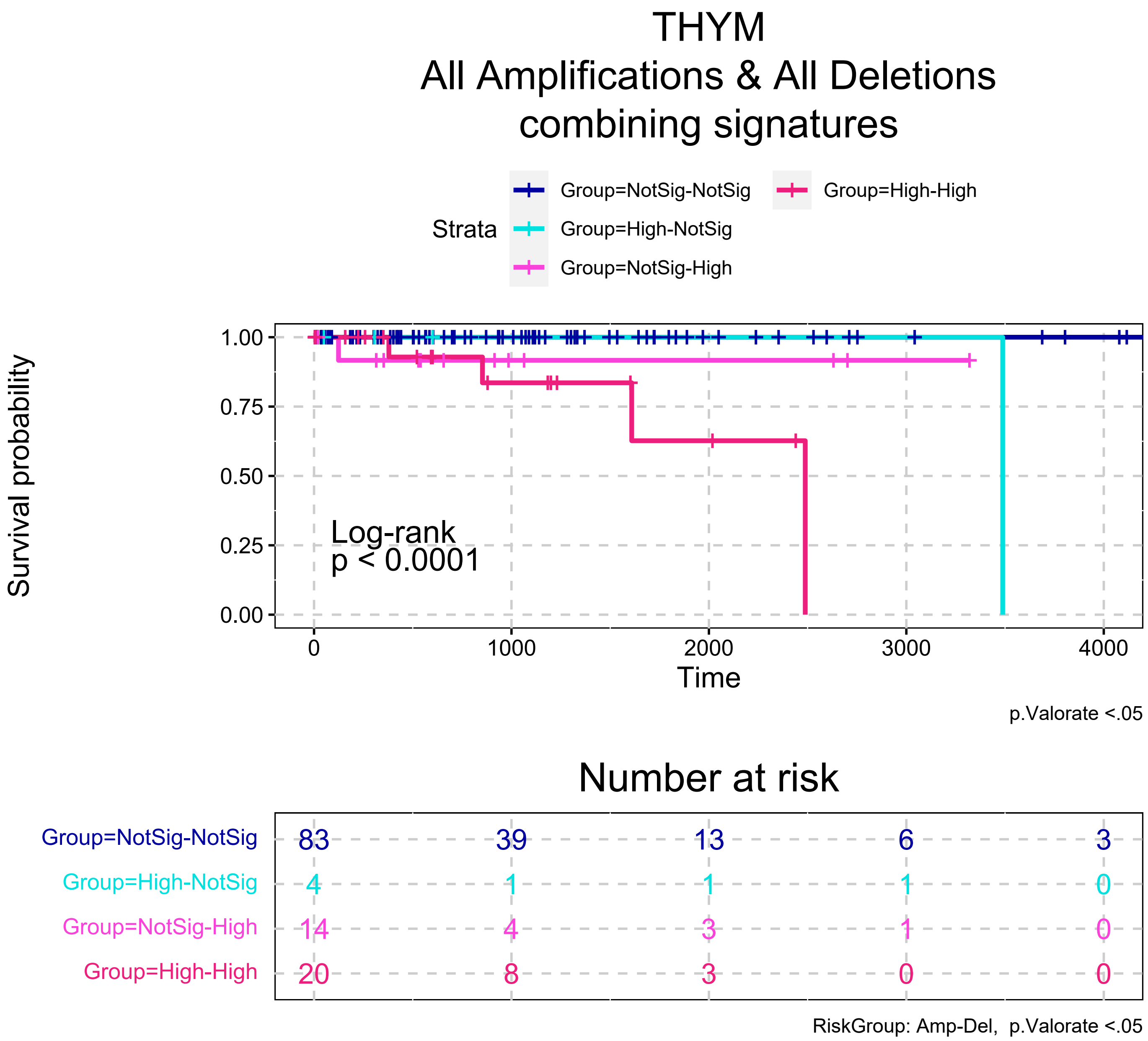

explanatory	beta	HR	L95
High-NotSig	18.95	170478841.91	0.00
NotSig-High	35.70	3207699749477615.50	325555012949
High-High	37.09	12799692994037456.00	129906306243

n= 121, number of events =6
Score(logrank) test = p <.0001

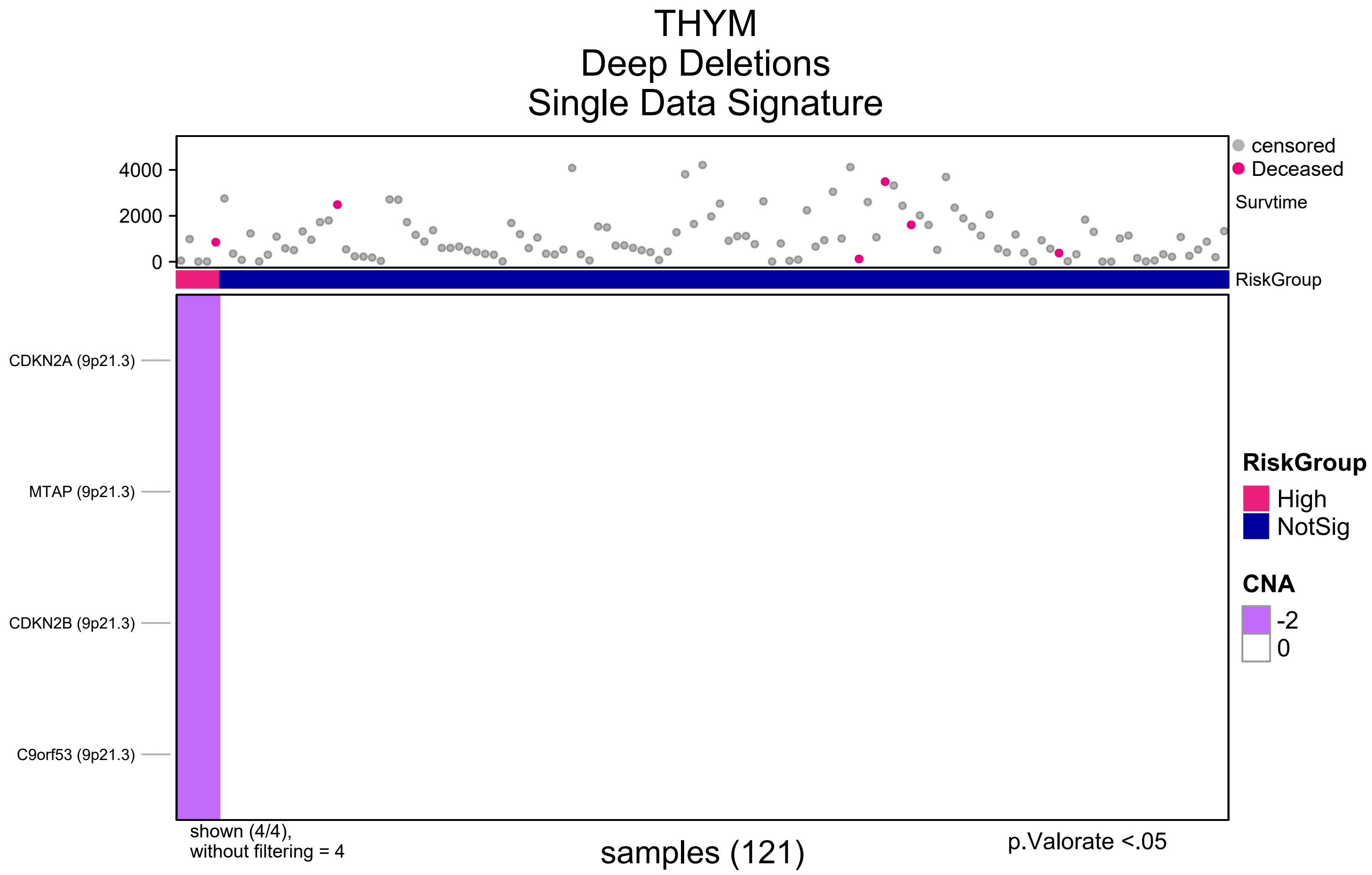
THYM
Deep Deletions
Single Data Signature
4000
2000
0
censored
Deceased
Survtime
RiskGroup
CDKN2A (9p21.3)
MTAP (9p21.3)
CDKN2B (9p21.3)
C9orf53 (9p21.3)
RiskGroup
High
NotSig
CNA
-2
0
shown (4/4),
without filtering = 4
samples (121)
p.Valorate <.05

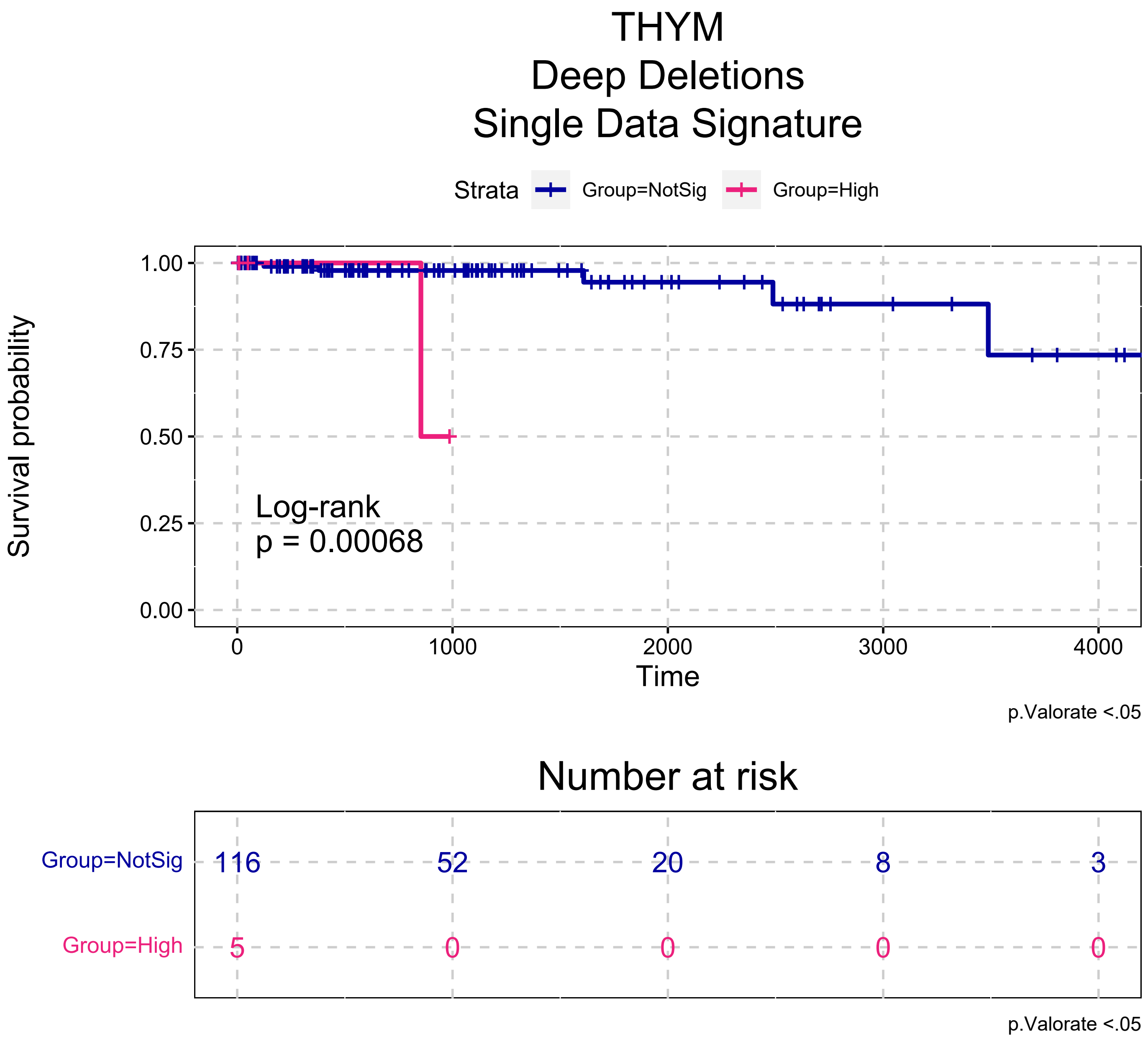

explanatory	beta	HR	L95	U95	p
High	2.97	19.57	1.75	218.96	0.02

n= 121, number of events =6
Score(logrank) test = 0.001
