# Supplementary material for: Systematic Modeling of Risk-Associated Copy Number Alterations in Cancer
Source: Int J Mol Sci. 2024 Sep 27;25(19):10455. doi: 10.3390/ijms251910455 (PMC11477427; doi:10.3390/ijms251910455)
Supplement: Supplementary file 1 [file ijms-25-10455-s001.zip › UCECSignatureV12-sinSombreado.pdf]

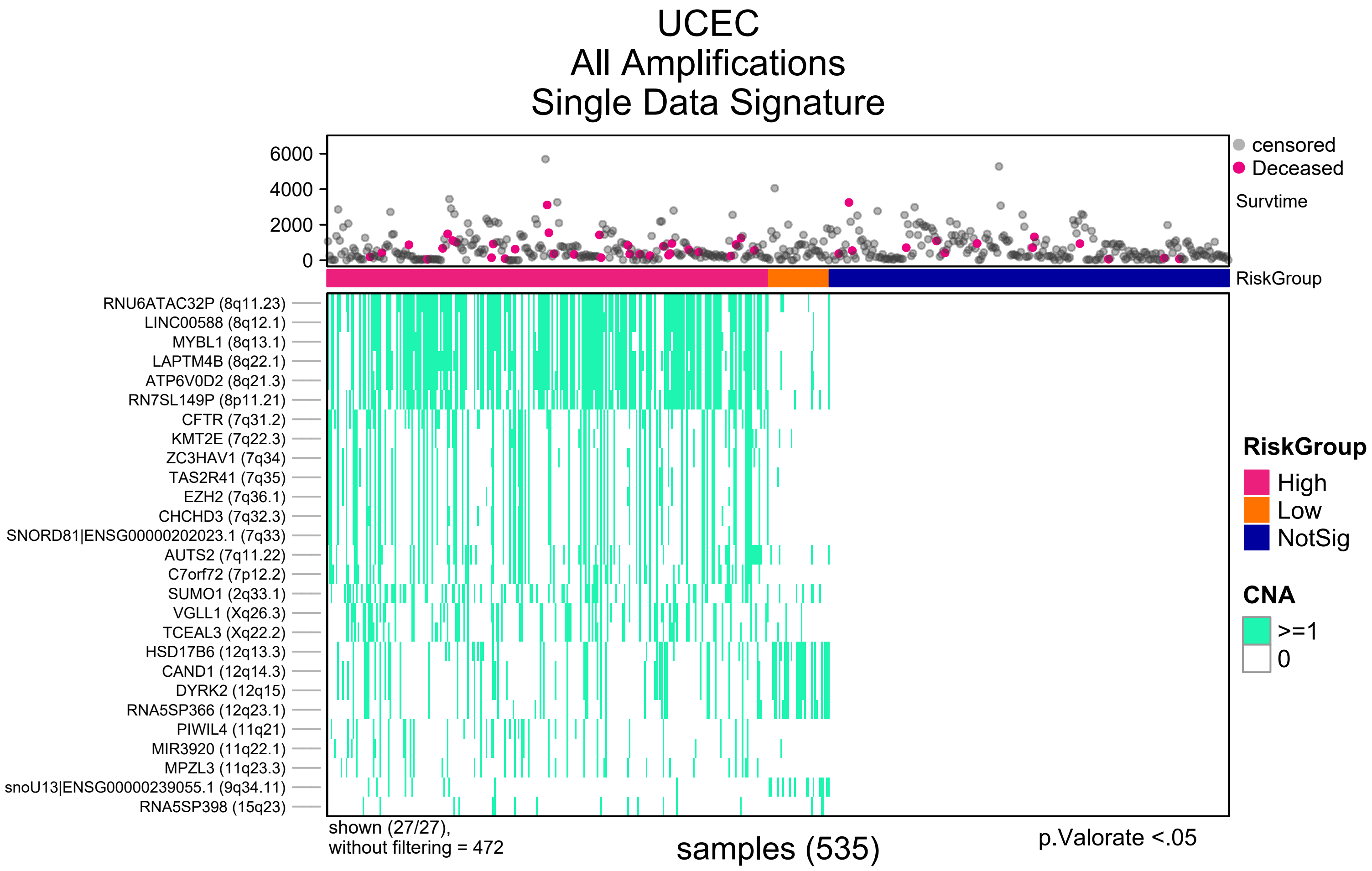
UCEC
All Amplifications
Single Data Signature
6000
4000
2000
0
censored
Deceased
Survtime
RiskGroup
RNU6ATAC32P (8q11.23)
LINC00588 (8q12.1)
MYBL1 (8q13.1)
LAPTM4B (8q22.1)
ATP6V0D2 (8q21.3)
RN7SL149P (8p11.21)
CFTR (7q31.2)
KMT2E (7q22.3)
ZC3HAV1 (7q34)
TAS2R41 (7q35)
EZH2 (7q36.1)
CHCHD3 (7q32.3)
SNORD81|ENSG00000202023.1 (7q33)
AUTS2 (7q11.22)
C7orf72 (7p12.2)
SUMO1 (2q33.1)
VGLL1 (Xq26.3)
TCEAL3 (Xq22.2)
HSD17B6 (12q13.3)
CAND1 (12q14.3)
DYRK2 (12q15)
RNA5SP366 (12q23.1)
PIWIL4 (11q21)
MIR3920 (11q22.1)
MPZL3 (11q23.3)
snoU13|ENSG00000239055.1 (9q34.11)
RNA5SP398 (15q23)
RiskGroup
High
Low
NotSig
CNA
>=1
0
shown (27/27),
without filtering = 472
samples (535)
p.Valorate <.05

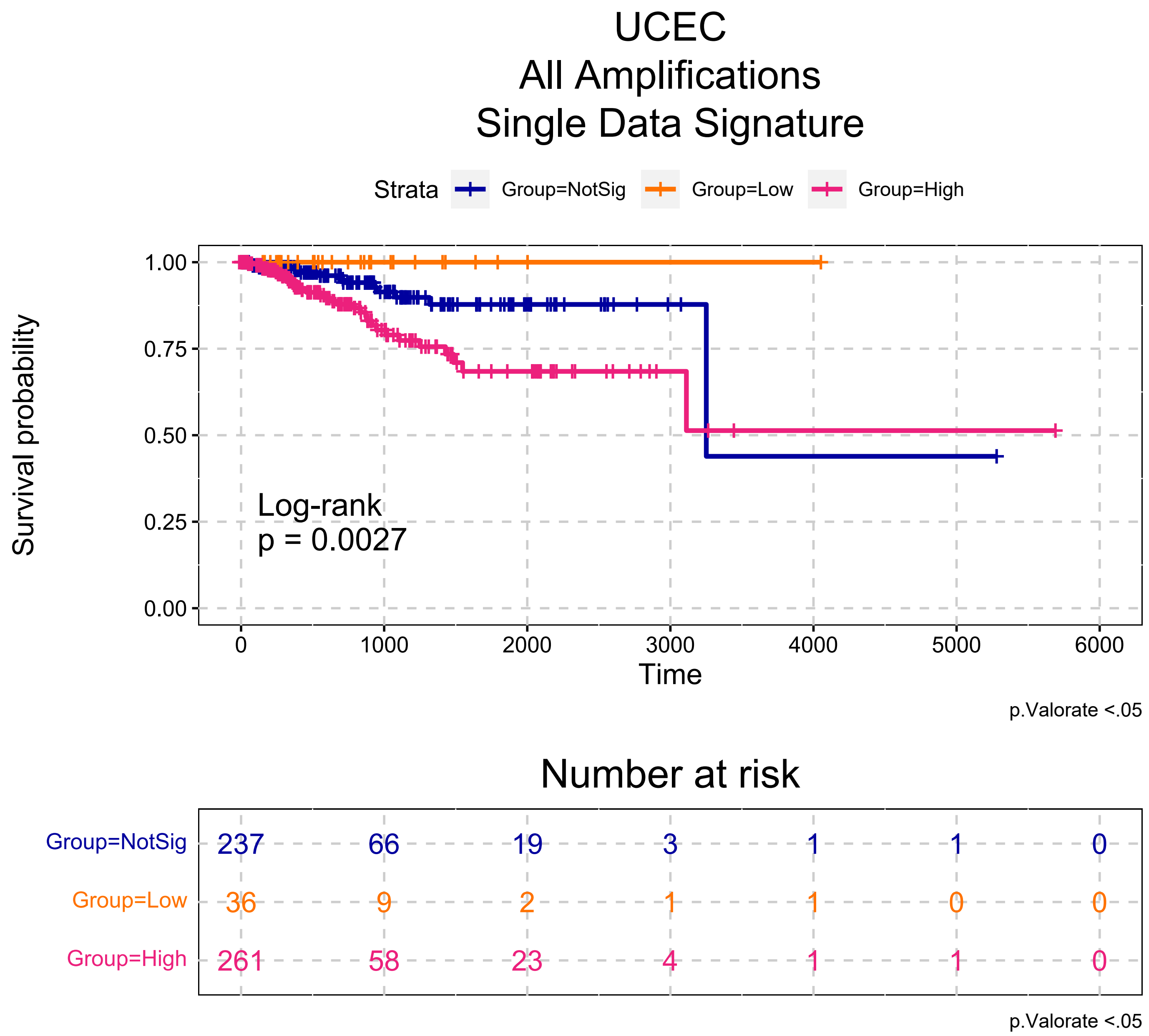

explanatory	beta	HR	L95	U95	p
Low	-16.67	0.00	0.00	Inf	1.00
High	0.89	2.43	1.27	4.63	0.01

n= 535, number of events =45
Score(logrank) test = 0.003

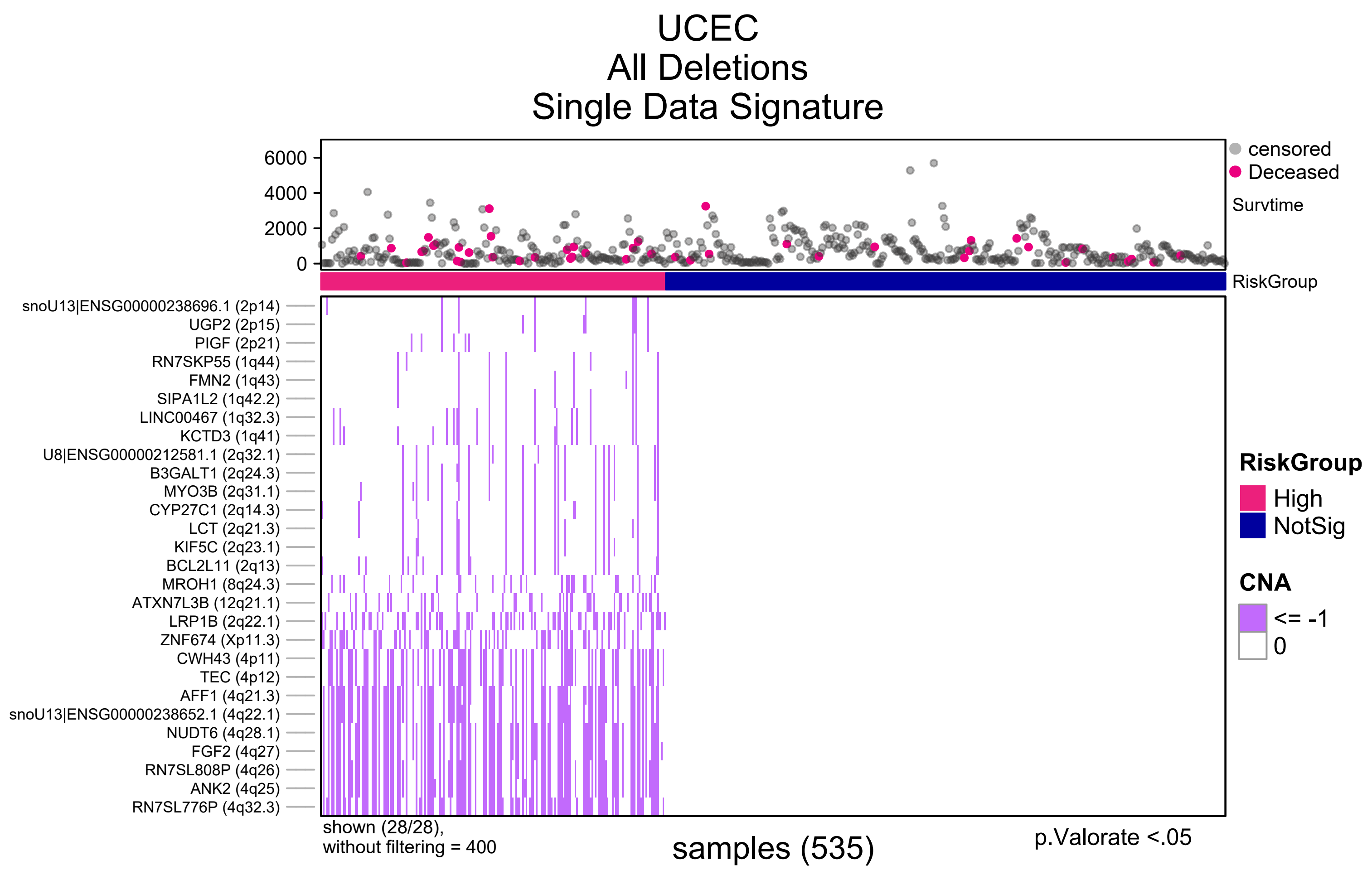
UCEC
All Deletions
Single Data Signature
6000
4000
2000
0
censored
Deceased
Survtime
RiskGroup
snoU13|ENSG00000238696.1 (2p14)
UGP2 (2p15)
PIGF (2p21)
RN7SKP55 (1q44)
FMN2 (1q43)
SIPA1L2 (1q42.2)
LINC00467 (1q32.3)
KCTD3 (1q41)
U8|ENSG00000212581.1 (2q32.1)
B3GALT1 (2q24.3)
MYO3B (2q31.1)
CYP27C1 (2q14.3)
LCT (2q21.3)
KIF5C (2q23.1)
BCL2L11 (2q13)
MROH1 (8q24.3)
ATXN7L3B (12q21.1)
LRP1B (2q22.1)
ZNF674 (Xp11.3)
CWH43 (4p11)
TEC (4p12)
AFF1 (4q21.3)
snoU13|ENSG00000238652.1 (4q22.1)
NUDT6 (4q28.1)
FGF2 (4q27)
RN7SL808P (4q26)
ANK2 (4q25)
RN7SL776P (4q32.3)
RiskGroup
High
NotSig
CNA
<= -1
0
shown (28/28),
without filtering = 400
samples (535)
p.Valorate <.05

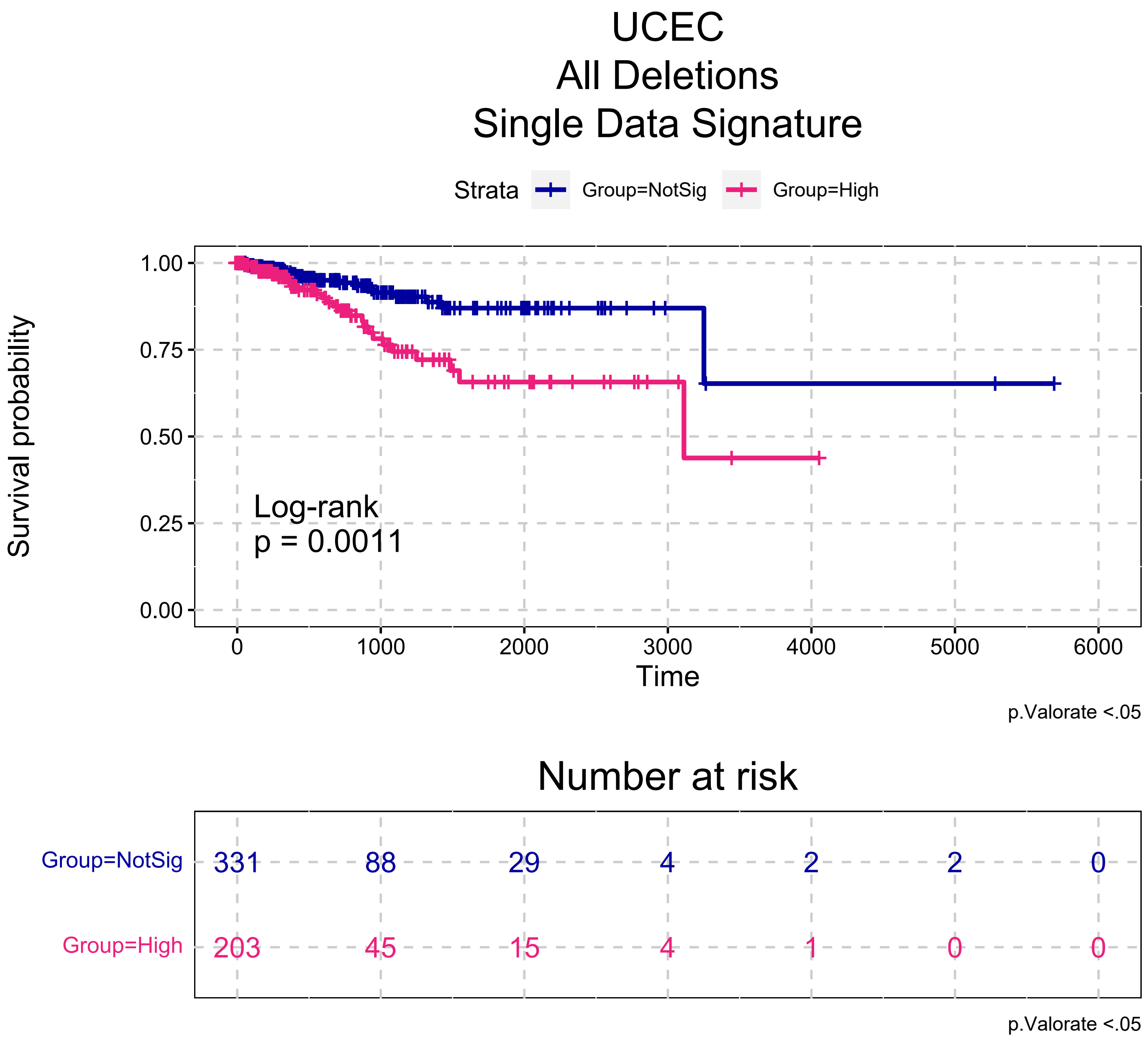

explanatory	beta	HR	L95	U95	p
High	0.95	2.59	1.43	4.68	0.00

n= 535, number of events =45
Score(logrank) test = 0.001

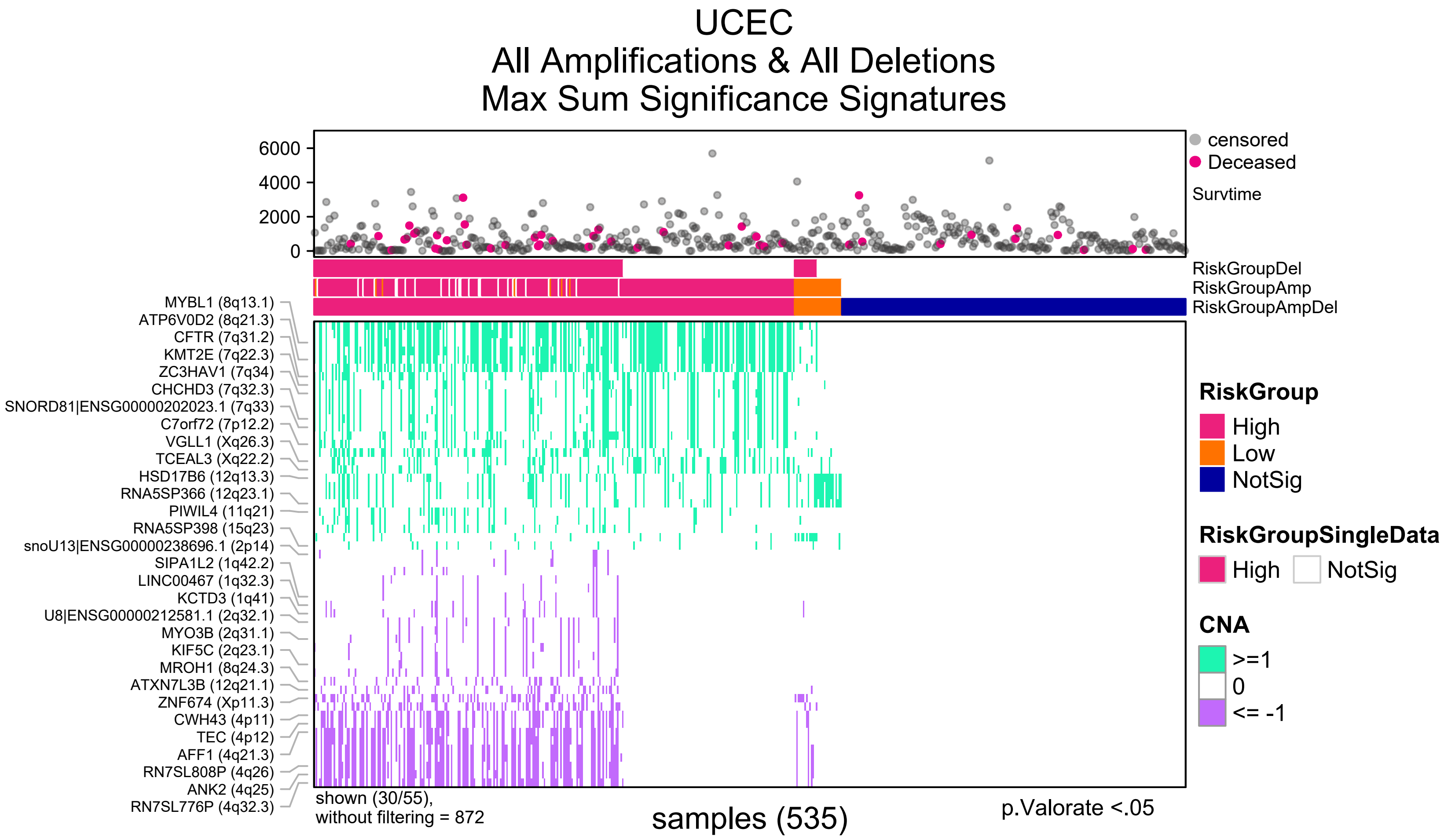
UCEC
All Amplifications & All Deletions
Max Sum Significance Signatures
6000
4000
2000
0
censored
Deceased
Survtime
RiskGroupDel
RiskGroupAmp
RiskGroupAmpDel
MYBL1 (8q13.1)
ATP6V0D2 (8q21.3)
CFTR (7q31.2)
KMT2E (7q22.3)
ZC3HAV1 (7q34)
CHCHD3 (7q32.3)
SNORD81|ENSG00000202023.1 (7q33)
C7orf72 (7p12.2)
VGLL1 (Xq26.3)
TCEAL3 (Xq22.2)
HSD17B6 (12q13.3)
RNA5SP366 (12q23.1)
PIWIL4 (11q21)
RNA5SP398 (15q23)
snoU13|ENSG00000238696.1 (2p14)
SIPA1L2 (1q42.2)
LINC00467 (1q32.3)
KCTD3 (1q41)
U8|ENSG00000212581.1 (2q32.1)
MYO3B (2q31.1)
KIF5C (2q23.1)
MROH1 (8q24.3)
ATXN7L3B (12q21.1)
ZNF674 (Xp11.3)
CWH43 (4p11)
TEC (4p12)
AFF1 (4q21.3)
RN7SL808P (4q26)
ANK2 (4q25)
RN7SL776P (4q32.3)
RiskGroup
High
Low
NotSig
RiskGroupSingleData
High
NotSig
CNA
>=1
0
<= -1
shown (30/55),
without filtering = 872
samples (535)
p.Valorate <.05

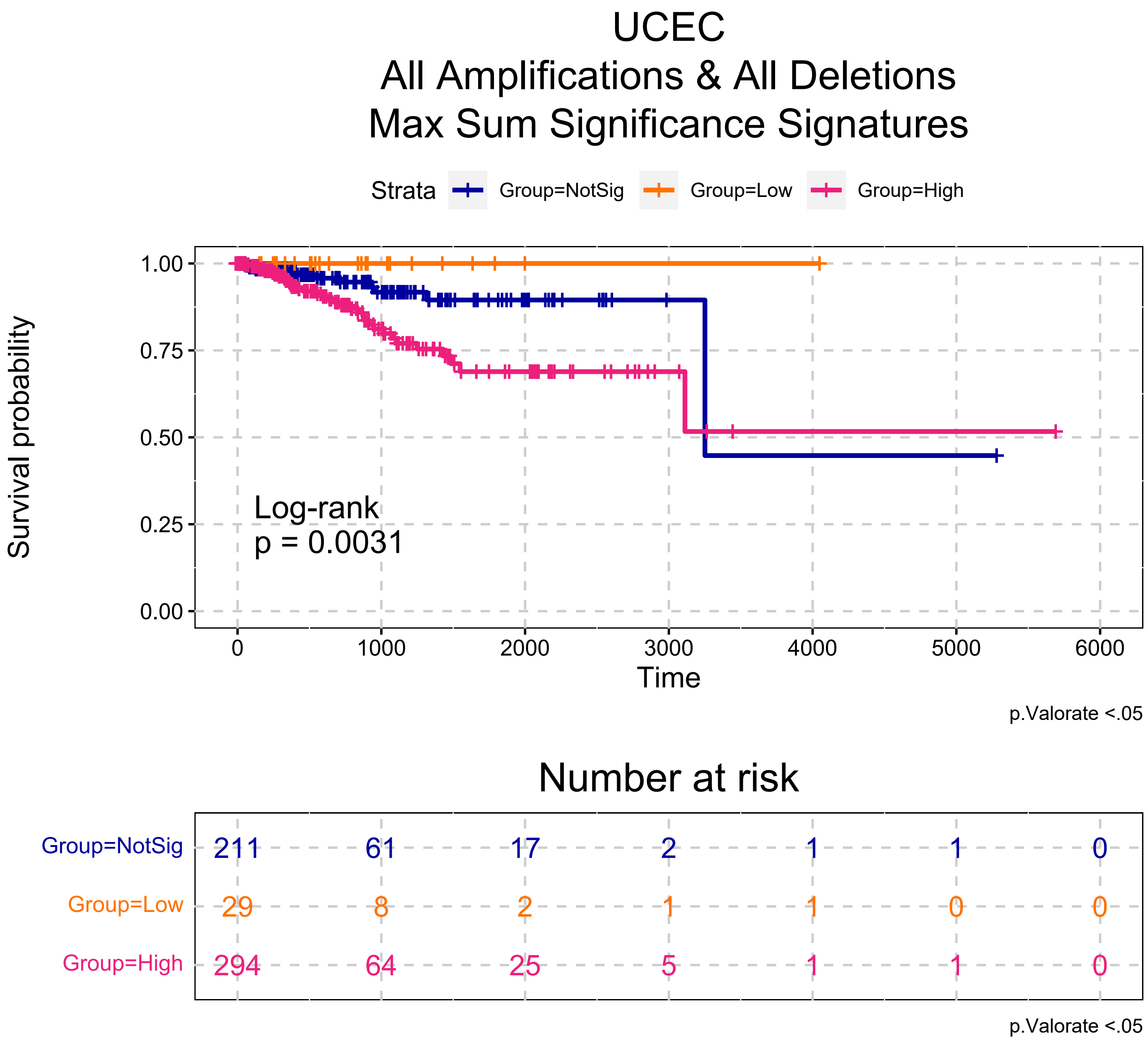

explanatory	beta	HR	L95	U95	p
Low	-16.58	0.00	0.00	Inf	1.00
High	0.94	2.57	1.30	5.08	0.01

n= 535, number of events =45
Score(logrank) test = 0.003

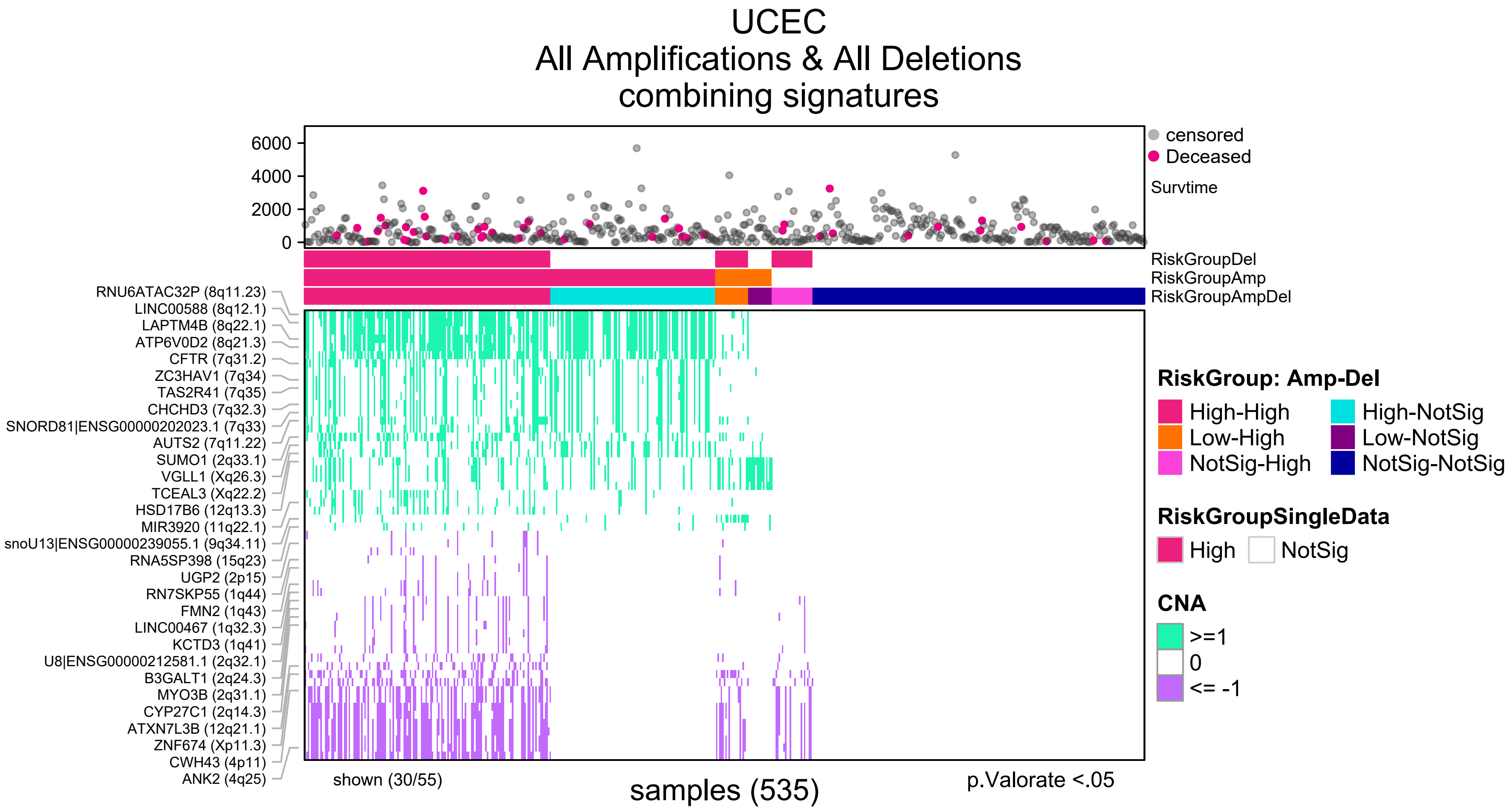
UCEC
All Amplifications & All Deletions
combining signatures
6000
4000
2000
0
censored
Deceased
Survtime
RiskGroupDel
RiskGroupAmp
RiskGroupAmpDel
RNU6ATAC32P (8q11.23)
LINC00588 (8q12.1)
LAPTM4B (8q22.1)
ATP6V0D2 (8q21.3)
CFTR (7q31.2)
ZC3HAV1 (7q34)
TAS2R41 (7q35)
CHCHD3 (7q32.3)
SNORD81|ENSG00000202023.1 (7q33)
AUTS2 (7q11.22)
SUMO1 (2q33.1)
VGLL1 (Xq26.3)
TCEAL3 (Xq22.2)
HSD17B6 (12q13.3)
MIR3920 (11q22.1)
snoU13|ENSG00000239055.1 (9q34.11)
RNA5SP398 (15q23)
UGP2 (2p15)
RN7SKP55 (1q44)
FMN2 (1q43)
LINC00467 (1q32.3)
KCTD3 (1q41)
U8|ENSG00000212581.1 (2q32.1)
B3GALT1 (2q24.3)
MYO3B (2q31.1)
CYP27C1 (2q14.3)
ATXN7L3B (12q21.1)
ZNF674 (Xp11.3)
CWH43 (4p11)
ANK2 (4q25)
RiskGroup: Amp-Del
High-High
High-NotSig
Low-High
Low-NotSig
NotSig-High
NotSig-NotSig
RiskGroupSingleData
High
NotSig
CNA
>=1
0
<= -1
shown (30/55)
samples (535)
p.Valorate <.05

UCEC
All Amplifications & All Deletions combining signatures

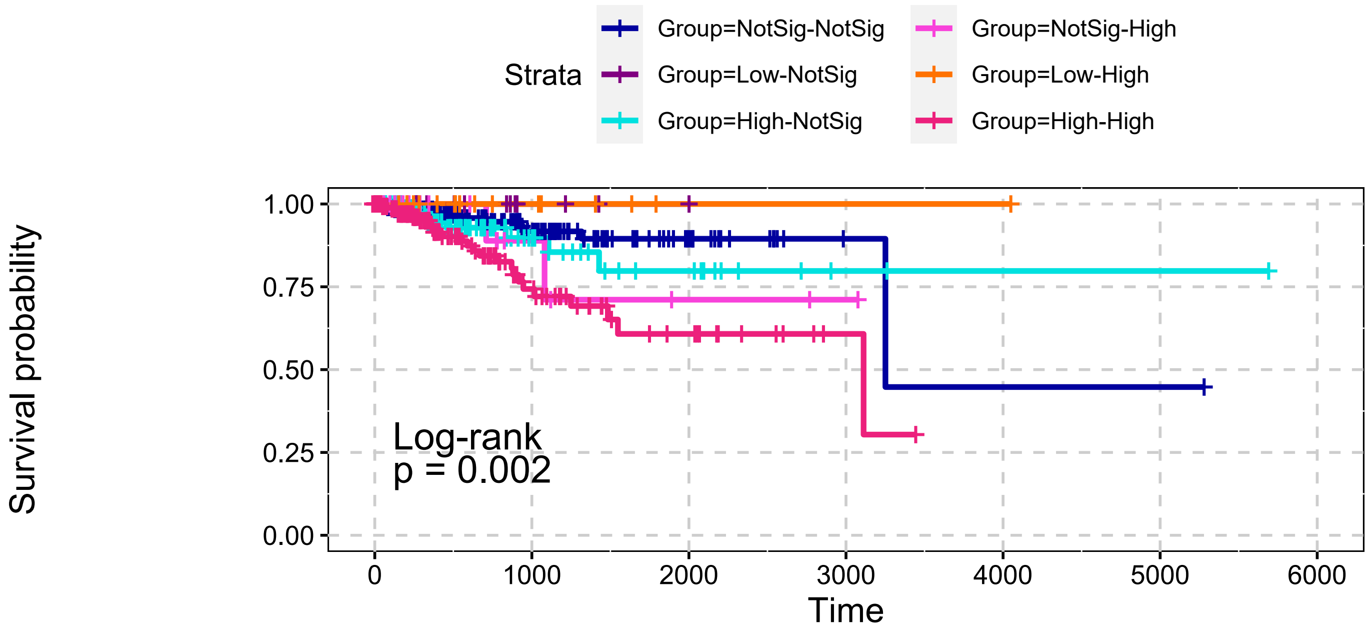

p.Valorate <.05

Number at risk

	0	1000	2000	3000	4000	5000	6000
Group=NotSig-NotSig	211	61	17	2	1	1	0
Group=Low-NotSig	15	3	1	0	0	0	0
Group=High-NotSig	105	24	11	2	1	1	0
Group=NotSig-High	26	5	2	1	0	0	0
Group=Low-High	21	6	1	1	1	0	0
Group=High-High	156	34	12	2	0	0	0

RiskGroup: Amp-Del, p.Valorate <.05

**explanatory**	**beta**	**HR**	**L95**	**U95**	**p**
Low-NotSig	-16.69	0.00	0.00	Inf	1.00
High-NotSig	0.43	1.53	0.61	3.81	0.36
NotSig-High	0.78	2.18	0.48	9.84	0.31
Low-High	-16.71	0.00	0.00	Inf	1.00
High-High	1.25	3.48	1.71	7.12	0.00

n= 535, number of events =45
Score(logrank) test = 0.002

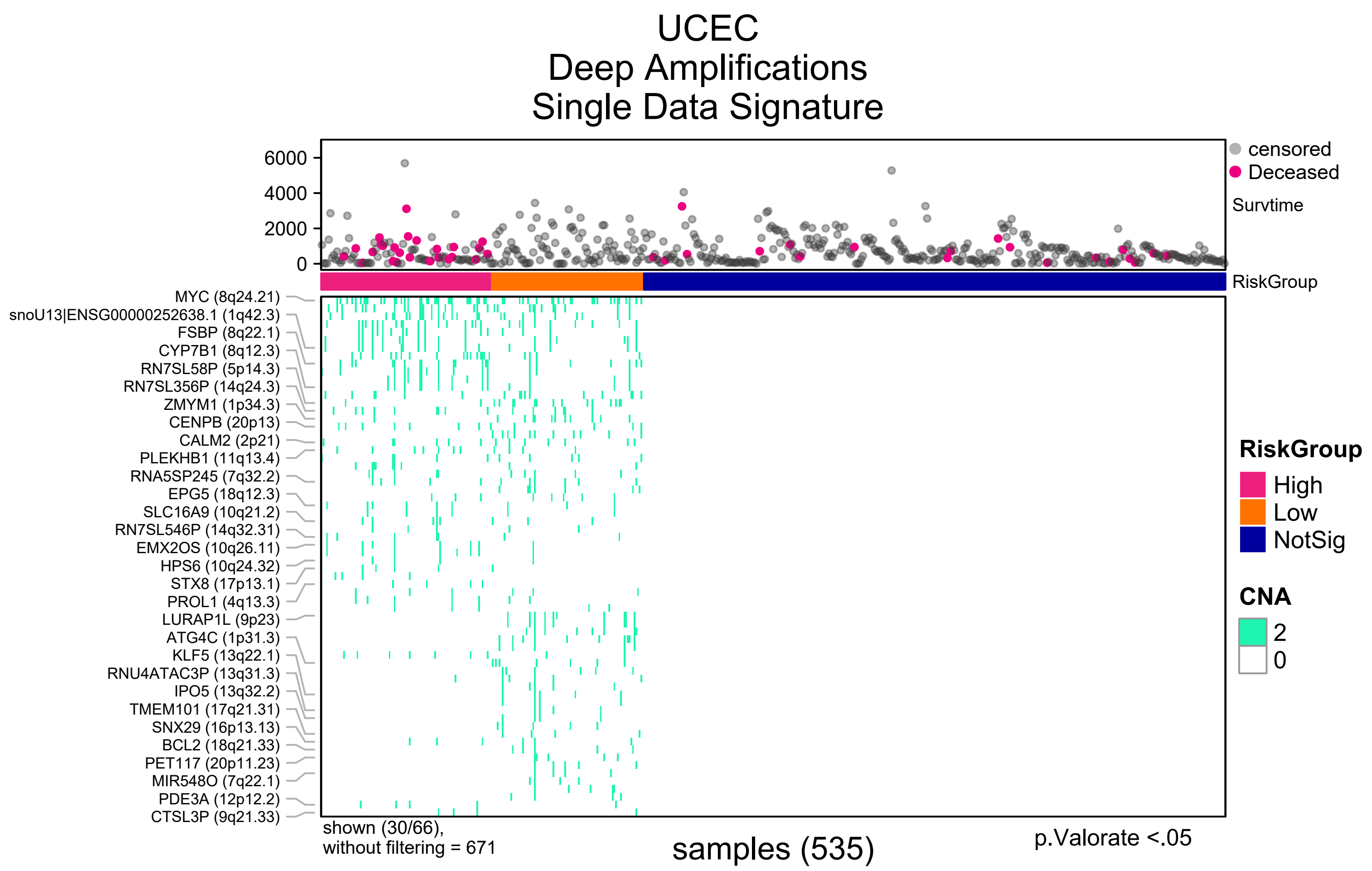

UCEC
Deep Amplifications
Single Data Signature
6000
4000
2000
0
censored
Deceased
Survtime
RiskGroup
MYC (8q24.21)
snoU13|ENSG00000252638.1 (1q42.3)
FSBP (8q22.1)
CYP7B1 (8q12.3)
RN7SL58P (5p14.3)
RN7SL356P (14q24.3)
ZMYM1 (1p34.3)
CENPB (20p13)
CALM2 (2p21)
PLEKHB1 (11q13.4)
RNA5SP245 (7q32.2)
EPG5 (18q12.3)
SLC16A9 (10q21.2)
RN7SL546P (14q32.31)
EMX2OS (10q26.11)
HPS6 (10q24.32)
STX8 (17p13.1)
PROL1 (4q13.3)
LURAP1L (9p23)
ATG4C (1p31.3)
KLF5 (13q22.1)
RNU4ATAC3P (13q31.3)
IPO5 (13q32.2)
TMEM101 (17q21.31)
SNX29 (16p13.13)
BCL2 (18q21.33)
PET117 (20p11.23)
MIR548O (7q22.1)
PDE3A (12p12.2)
CTSL3P (9q21.33)
RiskGroup
High
Low
NotSig
CNA
2
0
shown (30/66),
without filtering = 671
samples (535)
p.Valorate <.05

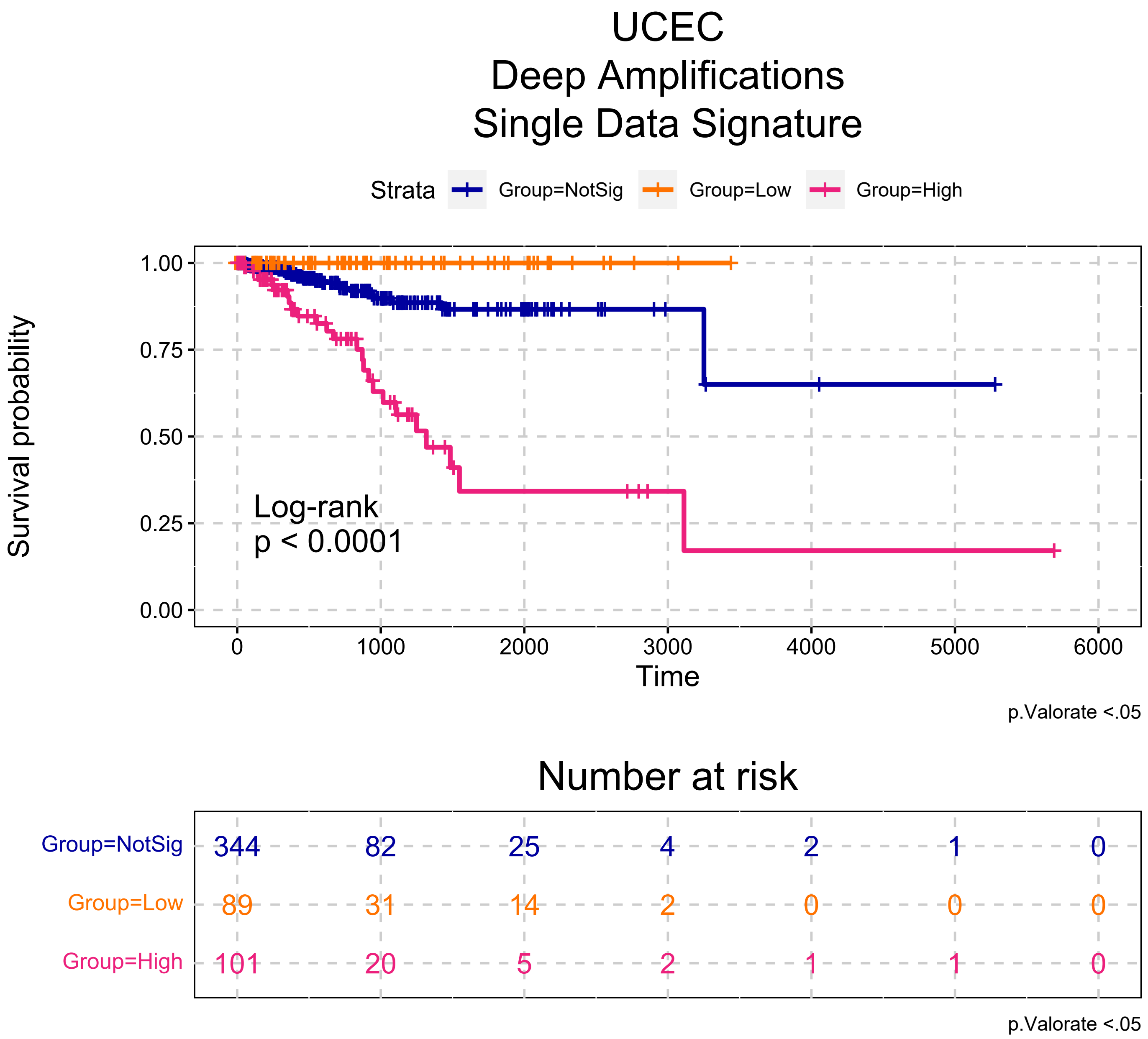

explanatory	beta	HR	L95	U95	p
Low	-18.78	0.00	0.00	Inf	1.00
High	1.59	4.90	2.71	8.83	0.00

n= 535, number of events =45
Score(logrank) test = p <.0001

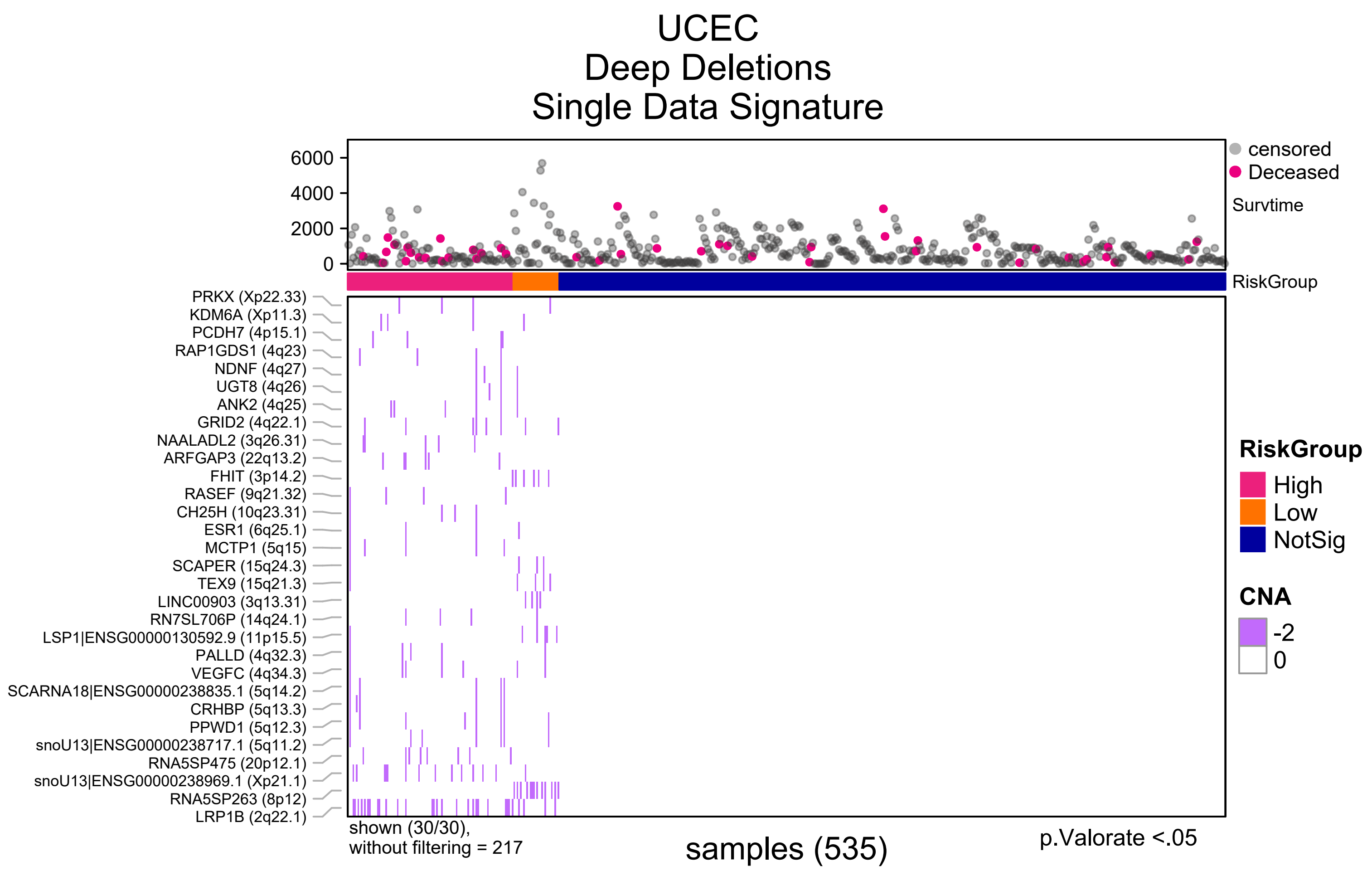
UCEC
Deep Deletions
Single Data Signature
6000
4000
2000
0
censored
Deceased
Survtime
RiskGroup
PRKX (Xp22.33)
KDM6A (Xp11.3)
PCDH7 (4p15.1)
RAP1GDS1 (4q23)
NDNF (4q27)
UGT8 (4q26)
ANK2 (4q25)
GRID2 (4q22.1)
NAALADL2 (3q26.31)
ARFGAP3 (22q13.2)
FHIT (3p14.2)
RASEF (9q21.32)
CH25H (10q23.31)
ESR1 (6q25.1)
MCTP1 (5q15)
SCAPER (15q24.3)
TEX9 (15q21.3)
LINC00903 (3q13.31)
RN7SL706P (14q24.1)
LSP1|ENSG00000130592.9 (11p15.5)
PALLD (4q32.3)
VEGFC (4q34.3)
SCARNA18|ENSG00000238835.1 (5q14.2)
CRHBP (5q13.3)
PPWD1 (5q12.3)
snoU13|ENSG00000238717.1 (5q11.2)
RNA5SP475 (20p12.1)
snoU13|ENSG00000238969.1 (Xp21.1)
RNA5SP263 (8p12)
LRP1B (2q22.1)
RiskGroup
High
Low
NotSig
CNA
-2
0
shown (30/30),
without filtering = 217
samples (535)
p.Valorate <.05

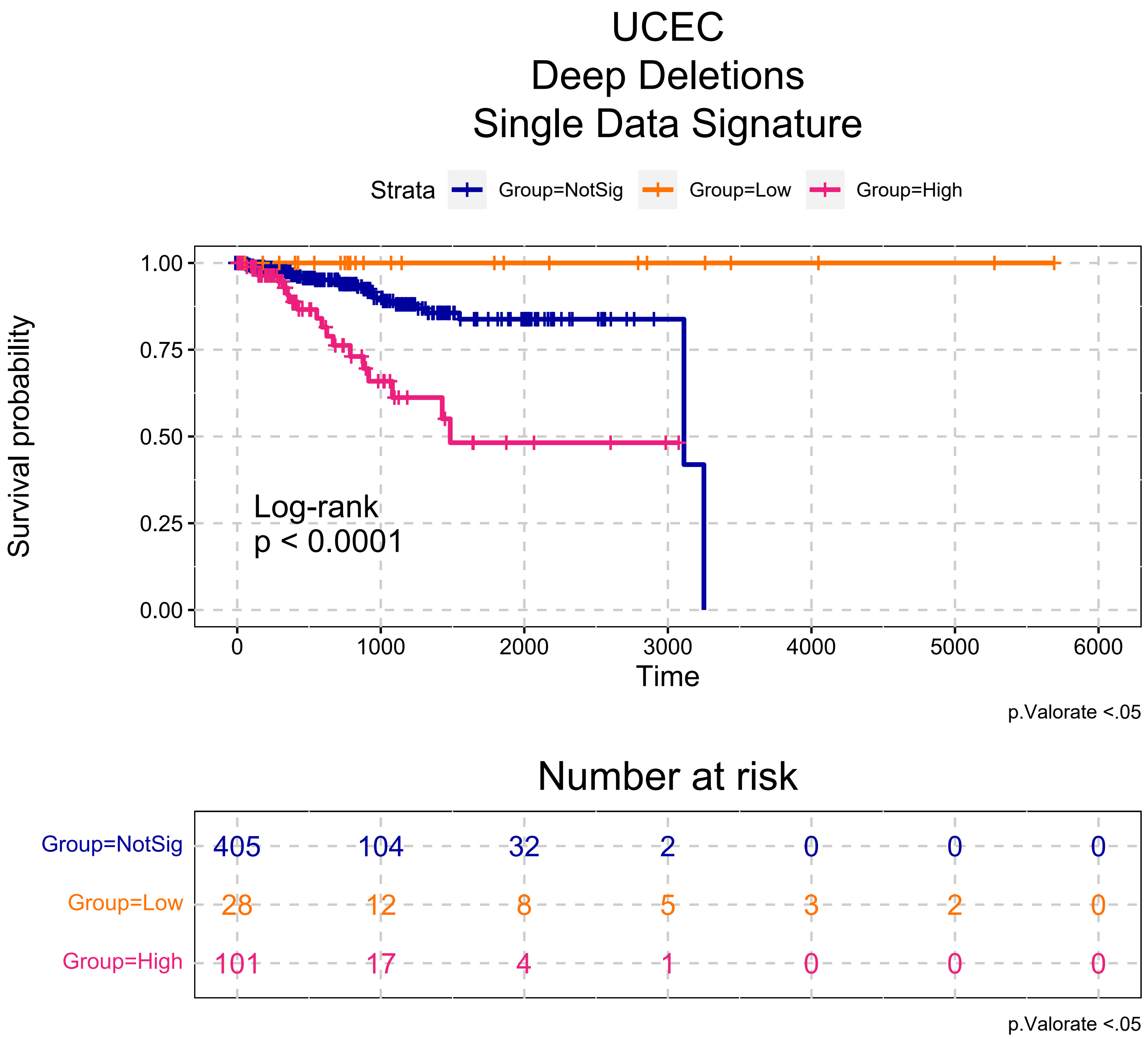

explanatory	beta	HR	L95	U95	p
Low	-19.16	0.00	0.00	Inf	1.00
High	1.34	3.82	2.08	7.02	0.00

n= 535, number of events =45
Score(logrank) test = p <.0001

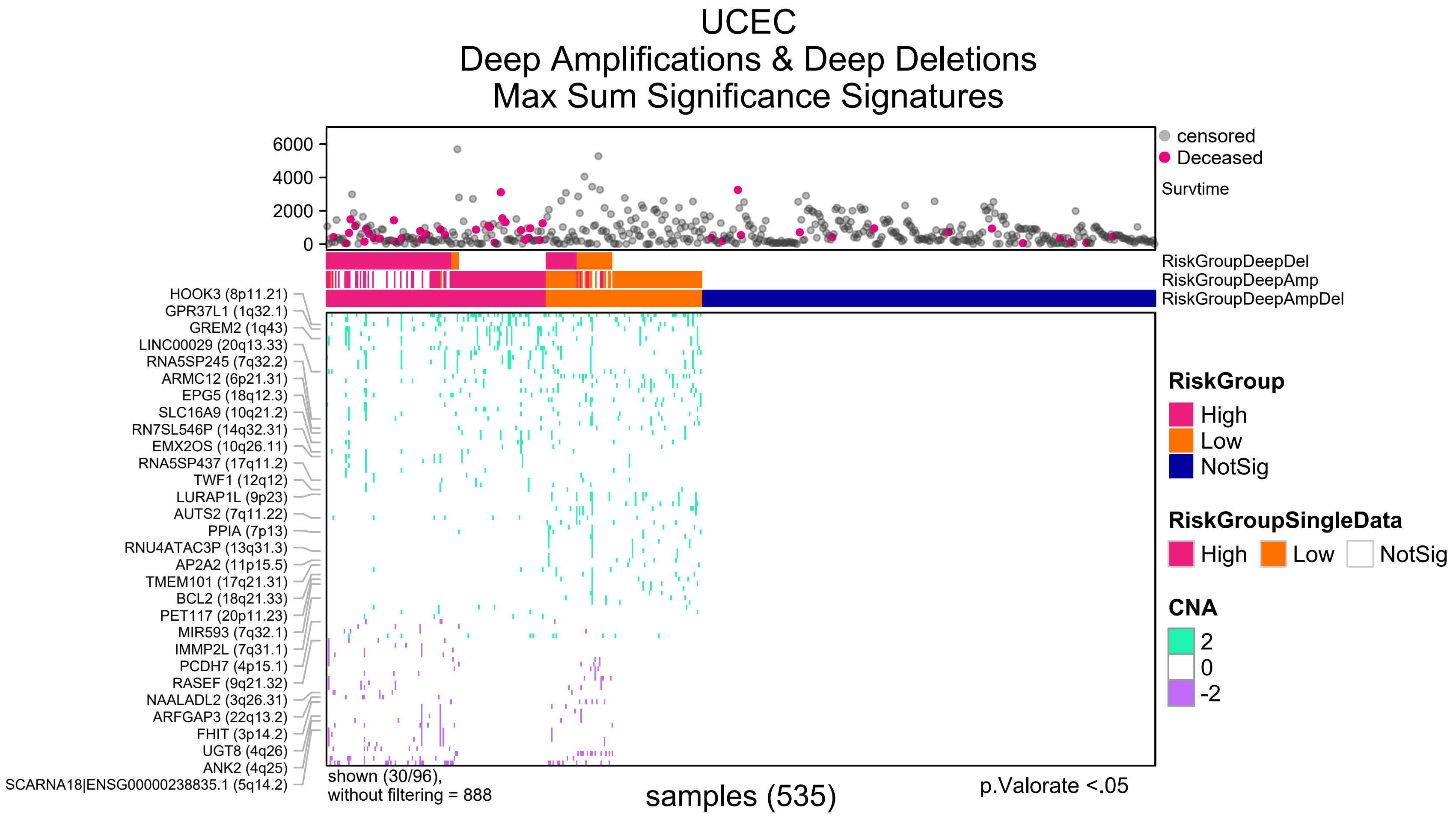
UCEC
Deep Amplifications & Deep Deletions
Max Sum Significance Signatures
6000
4000
2000
0
censored
Deceased
Survtime
RiskGroupDeepDel
RiskGroupDeepAmp
RiskGroupDeepAmpDel
HOOK3 (8p11.21)
GPR37L1 (1q32.1)
GREM2 (1q43)
LINC00029 (20q13.33)
RNA5SP245 (7q32.2)
ARMC12 (6p21.31)
EPG5 (18q12.3)
SLC16A9 (10q21.2)
RN7SL546P (14q32.31)
EMX2OS (10q26.11)
RNA5SP437 (17q11.2)
TWF1 (12q12)
LURAP1L (9p23)
AUTS2 (7q11.22)
PPIA (7p13)
RNU4ATAC3P (13q31.3)
AP2A2 (11p15.5)
TMEM101 (17q21.31)
BCL2 (18q21.33)
PET117 (20p11.23)
MIR593 (7q32.1)
IMMP2L (7q31.1)
PCDH7 (4p15.1)
RASEF (9q21.32)
NAALADL2 (3q26.31)
ARFGAP3 (22q13.2)
FHIT (3p14.2)
UGT8 (4q26)
ANK2 (4q25)
SCARNA18|ENSG00000238835.1 (5q14.2)
RiskGroup
High
Low
NotSig
RiskGroupSingleData
High
Low
NotSig
CNA
2
0
-2
shown (30/96),
without filtering = 888
samples (535)
p.Valorate <.05

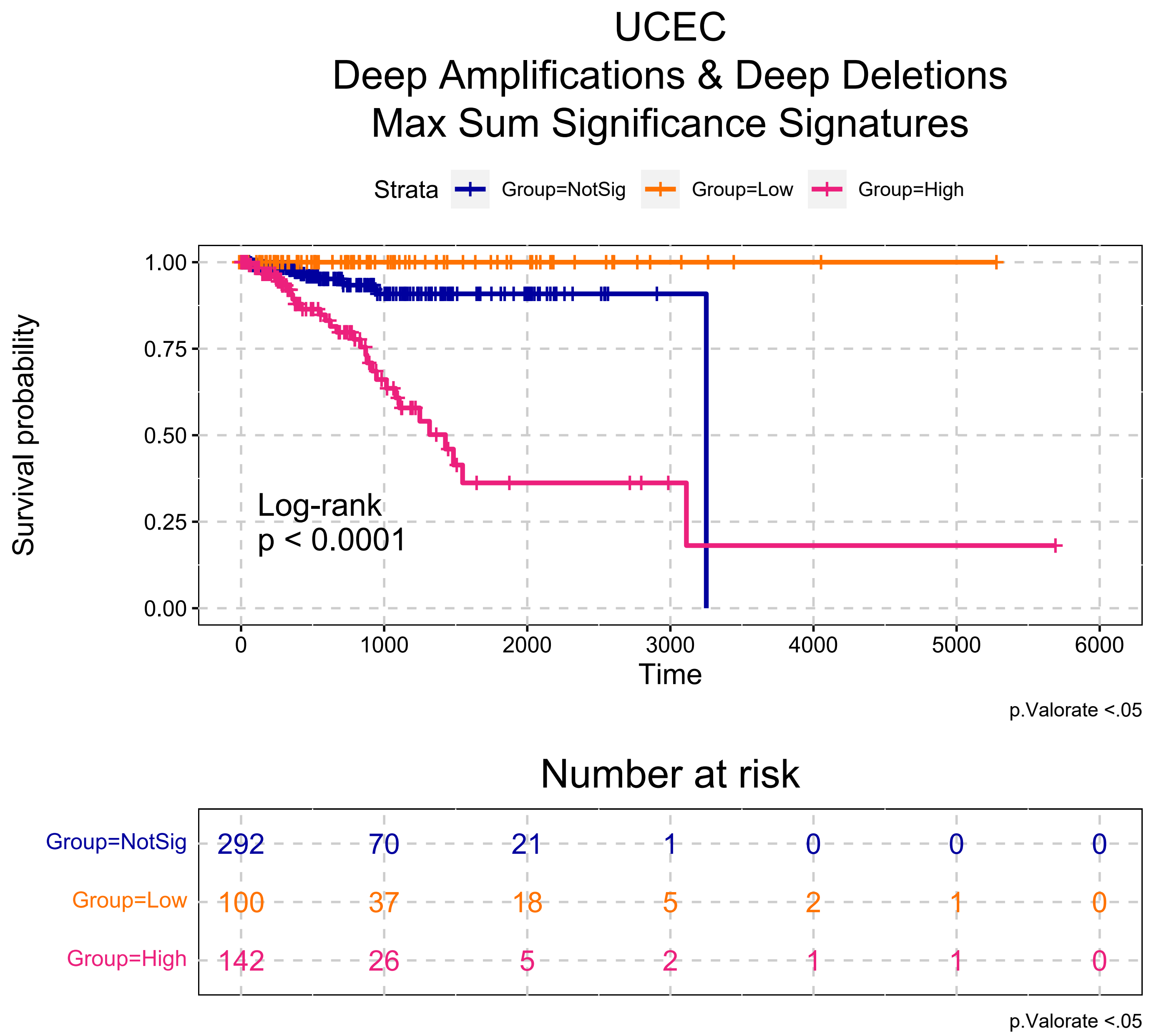

explanatory	beta	HR	L95	U95	p
Low	-18.55	0.00	0.00	Inf	1.00
High	1.68	5.34	2.83	10.08	0.00

n= 535, number of events =45
Score(logrank) test = p <.0001

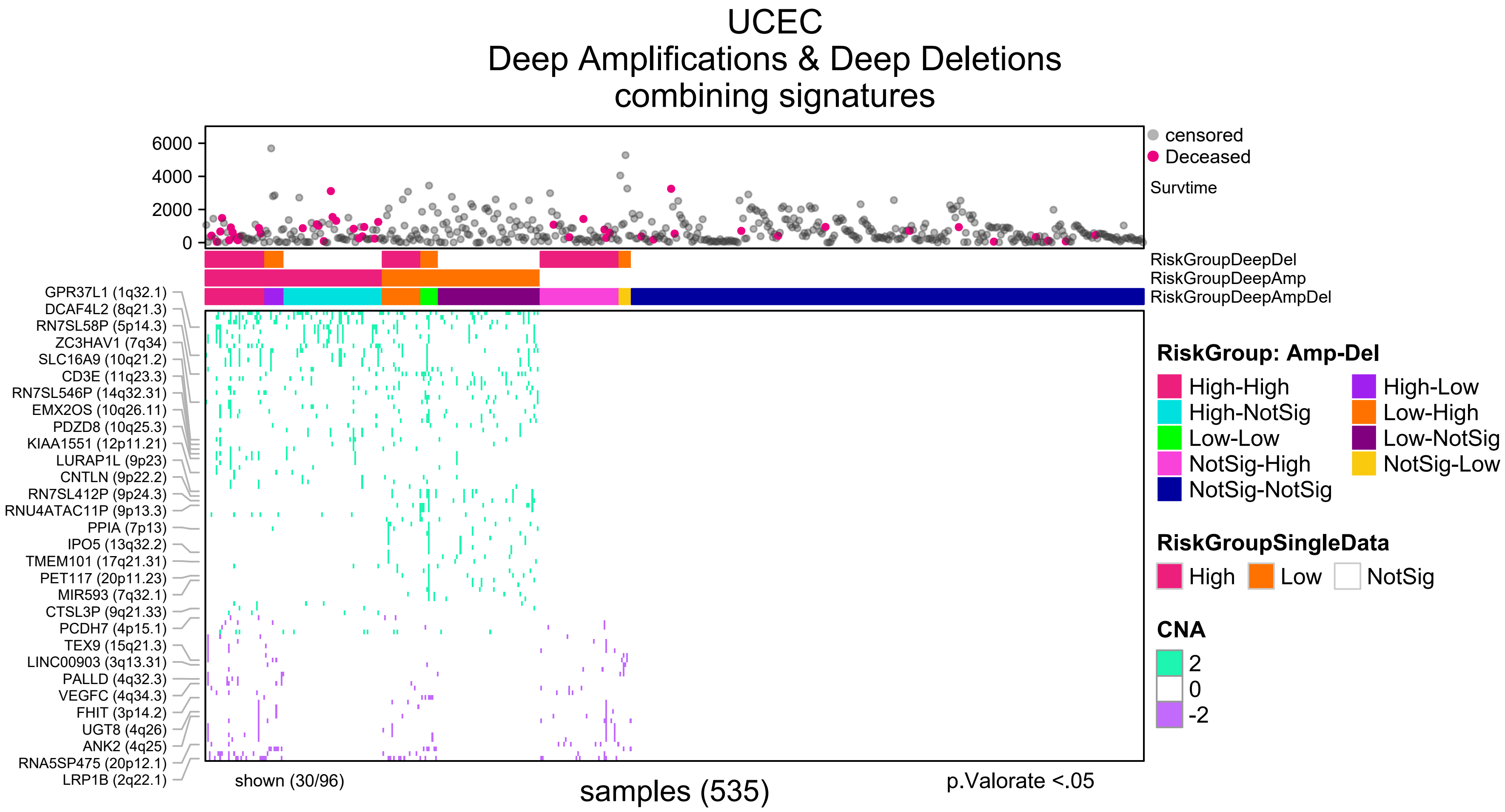

UCEC
Deep Amplifications & Deep Deletions
combining signatures
6000
4000
2000
0
censored
Deceased
Survtime
RiskGroupDeepDel
RiskGroupDeepAmp
RiskGroupDeepAmpDel
GPR37L1 (1q32.1)
DCAF4L2 (8q21.3)
RN7SL58P (5p14.3)
ZC3HAV1 (7q34)
SLC16A9 (10q21.2)
CD3E (11q23.3)
RN7SL546P (14q32.31)
EMX2OS (10q26.11)
PDZD8 (10q25.3)
KIAA1551 (12p11.21)
LURAP1L (9p23)
CNTLN (9p22.2)
RN7SL412P (9p24.3)
RNU4ATAC11P (9p13.3)
PPIA (7p13)
IPO5 (13q32.2)
TMEM101 (17q21.31)
PET117 (20p11.23)
MIR593 (7q32.1)
CTSL3P (9q21.33)
PCDH7 (4p15.1)
TEX9 (15q21.3)
LINC00903 (3q13.31)
PALLD (4q32.3)
VEGFC (4q34.3)
FHIT (3p14.2)
UGT8 (4q26)
ANK2 (4q25)
RNA5SP475 (20p12.1)
LRP1B (2q22.1)
RiskGroup: Amp-Del
High-High
High-Low
High-NotSig
Low-High
Low-Low
Low-NotSig
NotSig-High
NotSig-Low
NotSig-NotSig
RiskGroupSingleData
High
Low
NotSig
CNA
2
0
-2
shown (30/96)
samples (535)
p.Valorate <.05

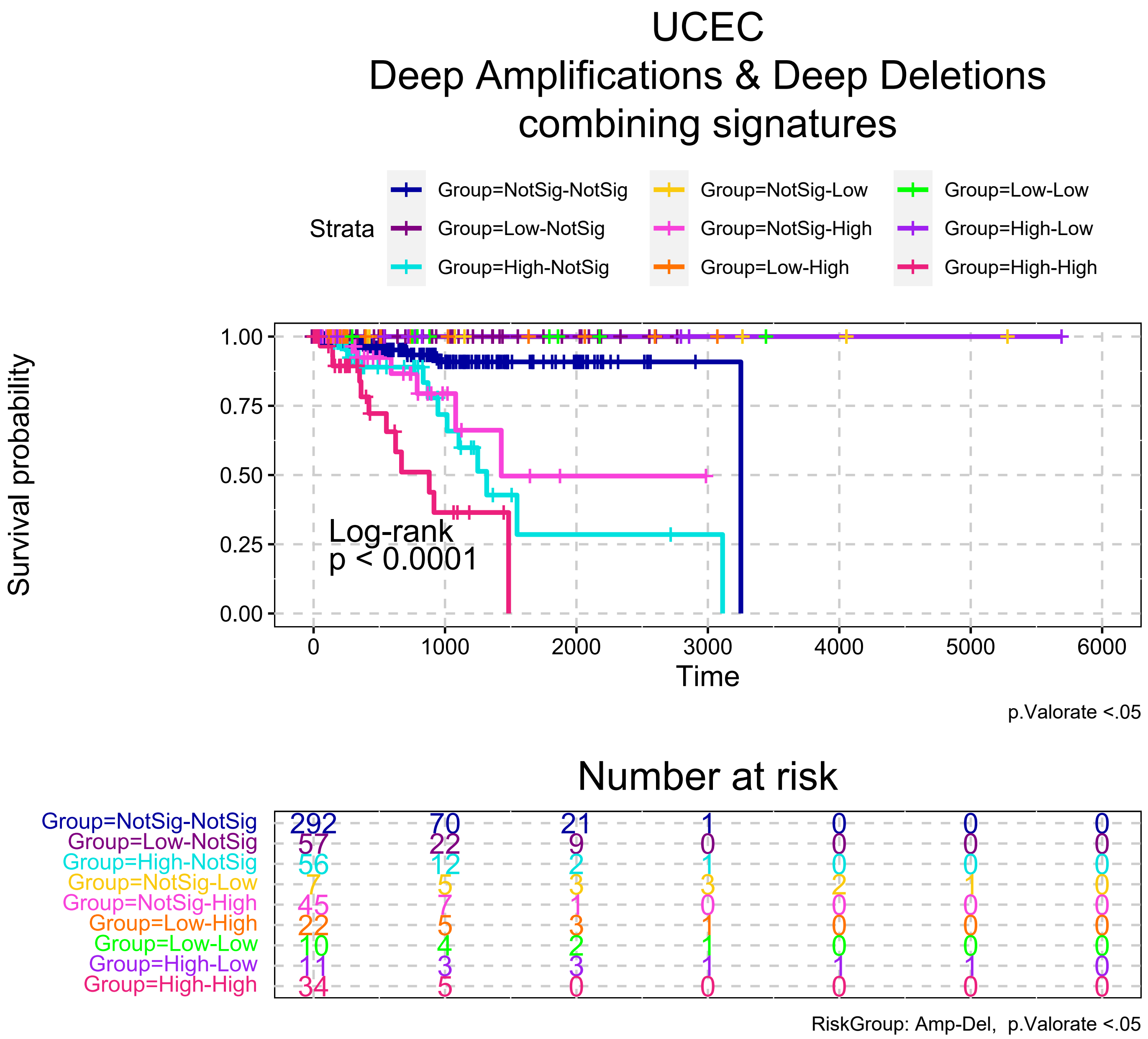

explanatory	beta	HR	L95	U95	p
Low-NotSig	-18.56	0.00	0.00	Inf	1.00
High-NotSig	1.73	5.66	2.60	12.32	0.00
NotSig-Low	-19.74	0.00	0.00	Inf	1.00
NotSig-High	1.35	3.87	1.47	10.20	0.01
Low-High	-18.56	0.00	0.00	Inf	1.00
Low-Low	-19.25	0.00	0.00	Inf	1.00
High-Low	-19.32	0.00	0.00	Inf	1.00
High-High	2.50	12.13	5.49	26.84	0.00

n= 535, number of events =45
Score(logrank) test = p <.0001
