# Supplementary material for: Systematic Modeling of Risk-Associated Copy Number Alterations in Cancer
Source: Int J Mol Sci. 2024 Sep 27;25(19):10455. doi: 10.3390/ijms251910455 (PMC11477427; doi:10.3390/ijms251910455)
Supplement: Supplementary file 1 [file ijms-25-10455-s001.zip › UCSSignatureV12-sinSombreado.pdf]

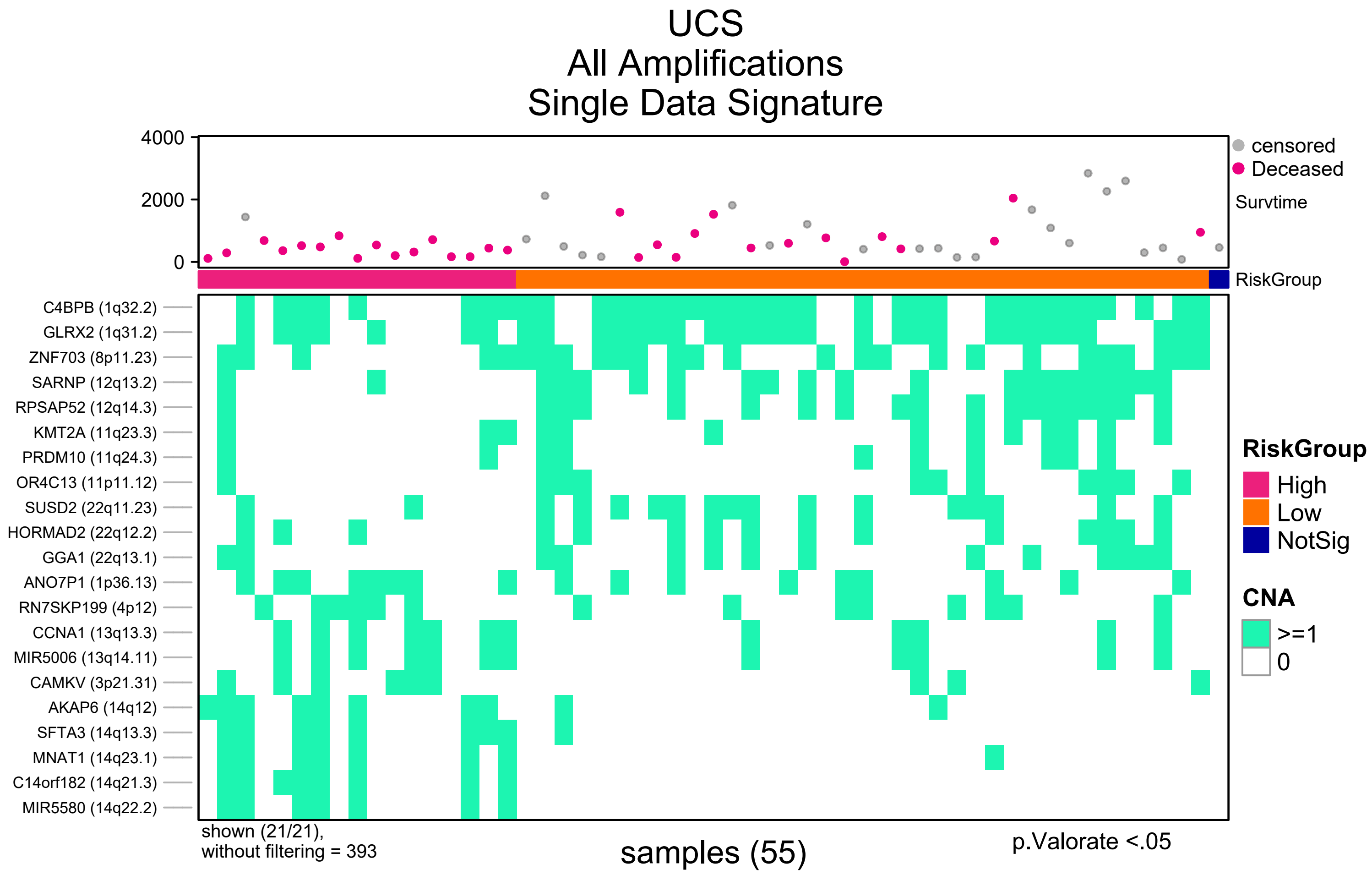
UCS
All Amplifications
Single Data Signature
4000
2000
0
censored
Deceased
Survtime
RiskGroup
C4BPB (1q32.2)
GLRX2 (1q31.2)
ZNF703 (8p11.23)
SARNP (12q13.2)
RPSAP52 (12q14.3)
KMT2A (11q23.3)
PRDM10 (11q24.3)
OR4C13 (11p11.12)
SUSD2 (22q11.23)
HORMAD2 (22q12.2)
GGA1 (22q13.1)
ANO7P1 (1p36.13)
RN7SKP199 (4p12)
CCNA1 (13q13.3)
MIR5006 (13q14.11)
CAMKV (3p21.31)
AKAP6 (14q12)
SFTA3 (14q13.3)
MNAT1 (14q23.1)
C14orf182 (14q21.3)
MIR5580 (14q22.2)
RiskGroup
High
Low
NotSig
CNA
>=1
0
shown (21/21),
without filtering = 393
samples (55)
p.Valorate <.05

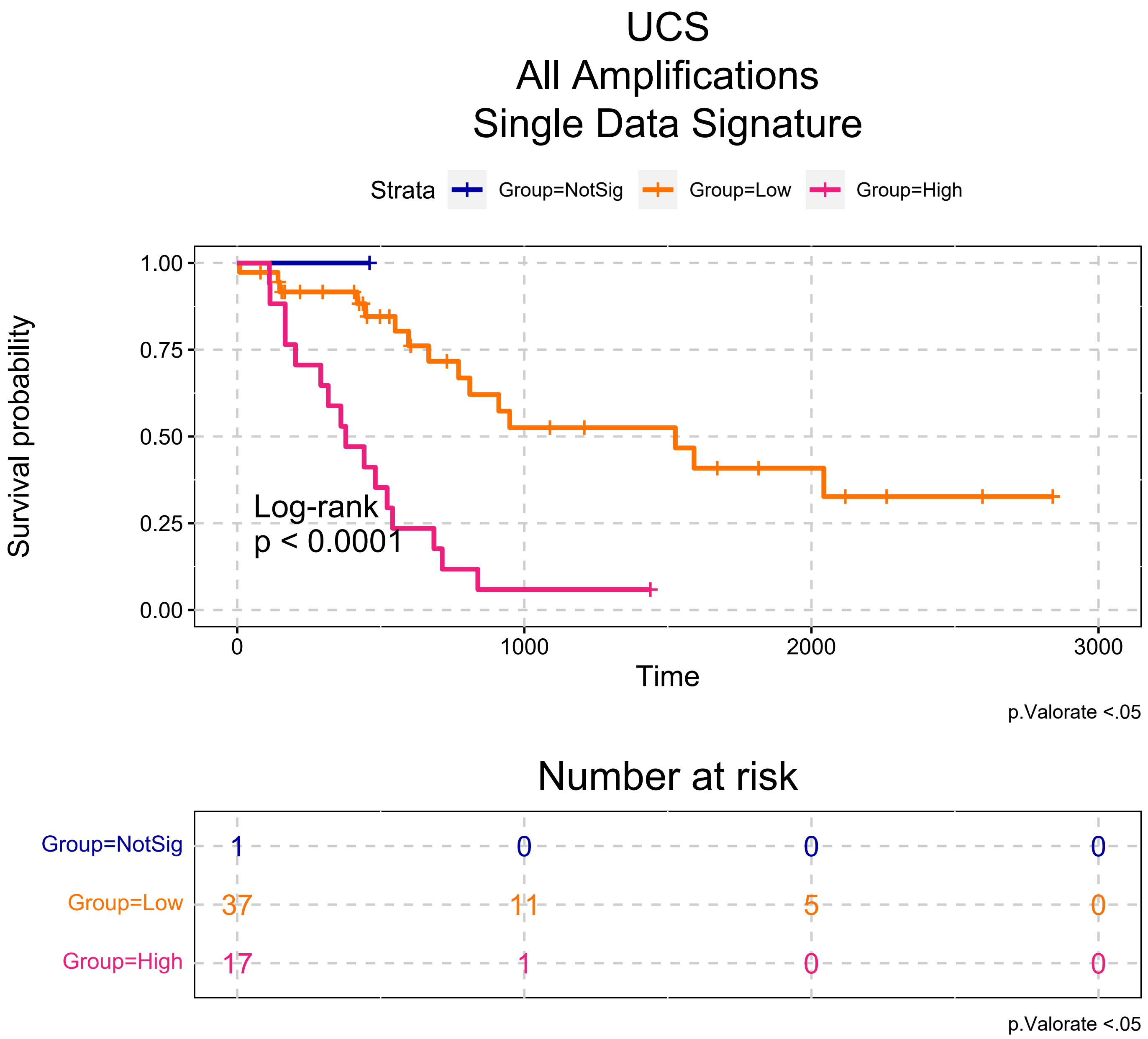


| explanatory | beta | HR | L95 | U95 | p |
|---|---|---|---|---|---|
| Low | 15.81 | 7316813.77 | 0.00 | Inf | 1.00 |
| High | 17.39 | 35691521.46 | 0.00 | Inf | 1.00 |

n= 55, number of events =31
Score(logrank) test = p <.0001

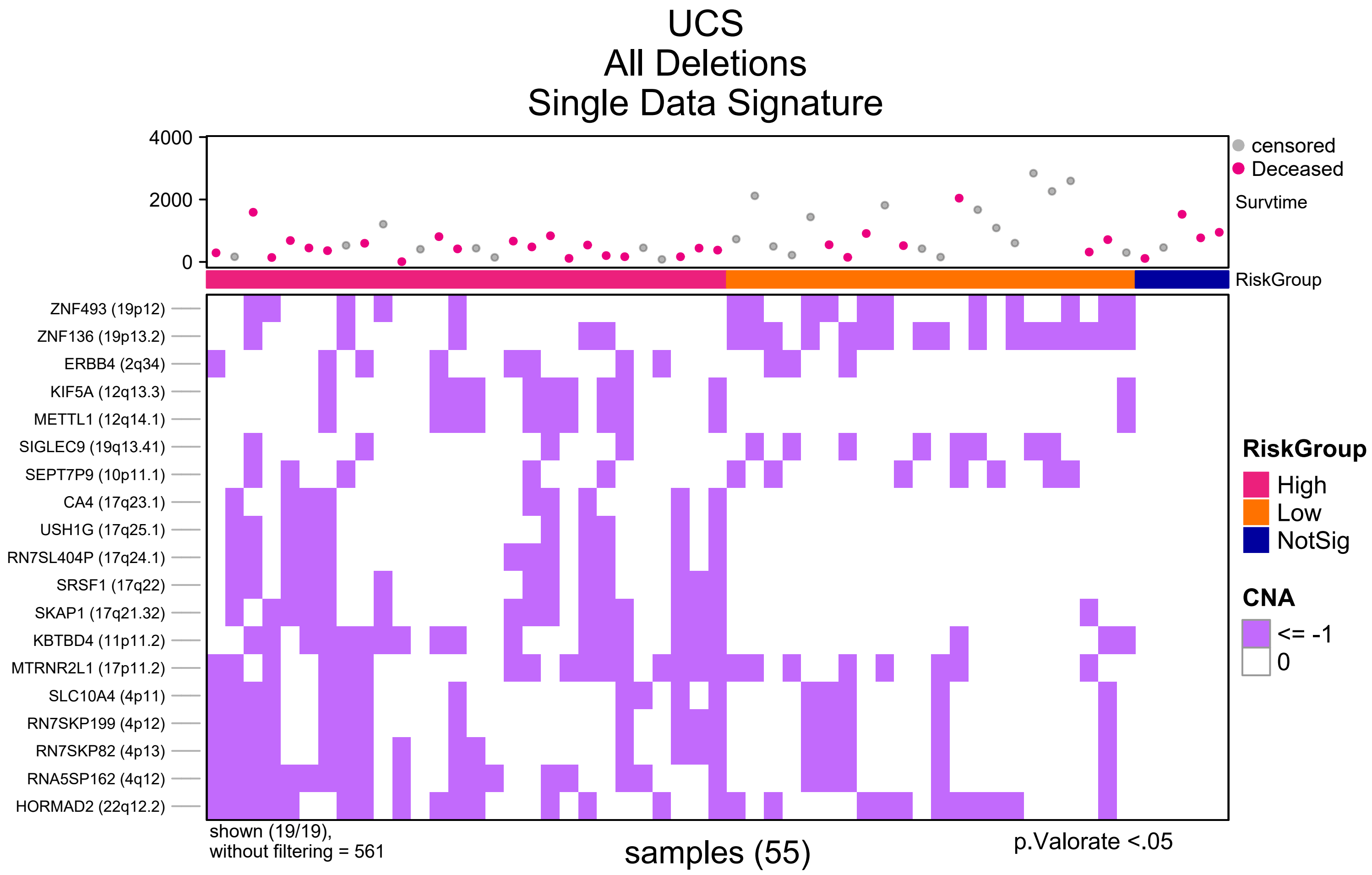

UCS
All Deletions
Single Data Signature
4000
2000
0
censored
Deceased
Survtime
RiskGroup
ZNF493 (19p12)
ZNF136 (19p13.2)
ERBB4 (2q34)
KIF5A (12q13.3)
METTL1 (12q14.1)
SIGLEC9 (19q13.41)
SEPT7P9 (10p11.1)
CA4 (17q23.1)
USH1G (17q25.1)
RN7SL404P (17q24.1)
SRSF1 (17q22)
SKAP1 (17q21.32)
KBTBD4 (11p11.2)
MTRNR2L1 (17p11.2)
SLC10A4 (4p11)
RN7SKP199 (4p12)
RN7SKP82 (4p13)
RNA5SP162 (4q12)
HORMAD2 (22q12.2)
RiskGroup
High
Low
NotSig
CNA
<= -1
0
shown (19/19),
without filtering = 561
samples (55)
p.Valorate <.05

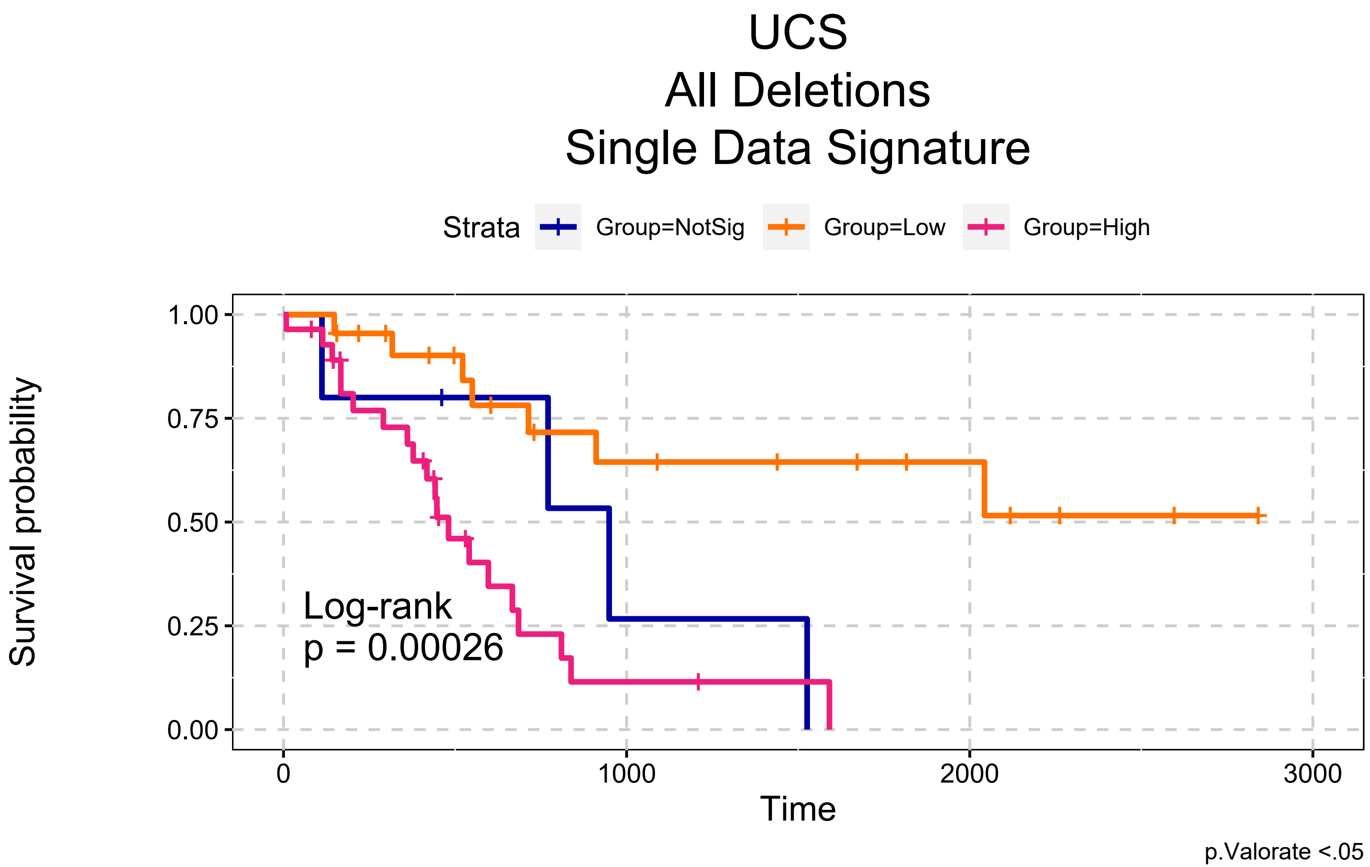


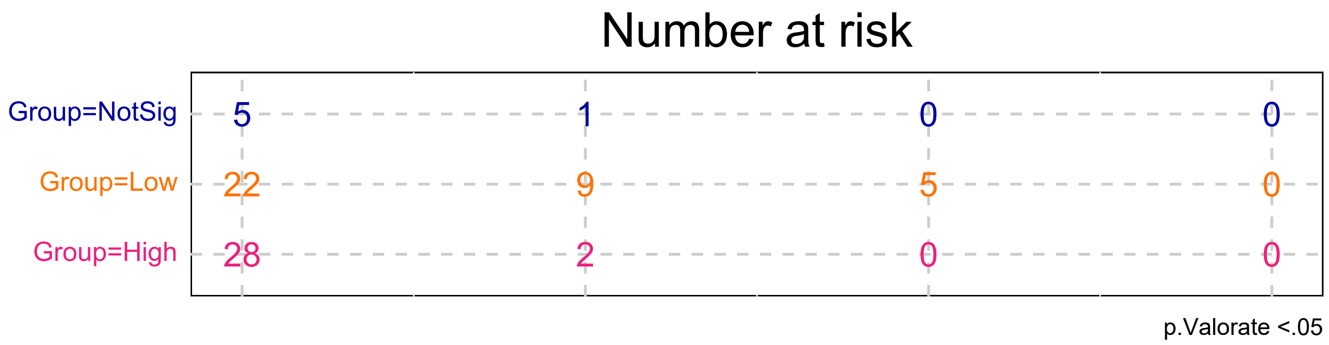


| explanatory | beta | HR | L95 | U95 | p |
|---|---|---|---|---|---|
| Low | -1.23 | 0.29 | 0.08 | 1.06 | 0.06 |
| High | 0.56 | 1.75 | 0.59 | 5.25 | 0.32 |

n= 55, number of events =31
Score(logrank) test = 0

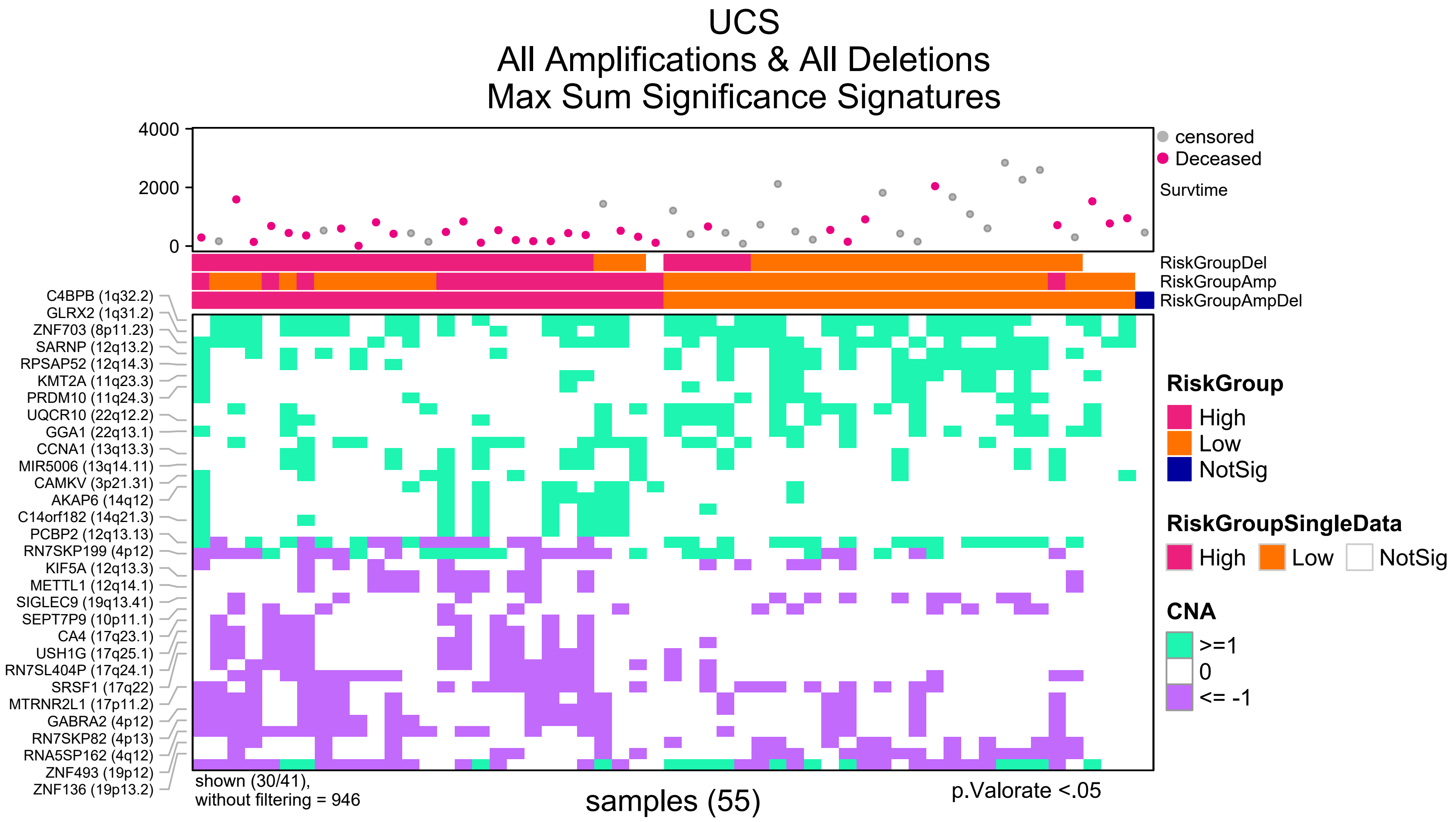
UCS
All Amplifications & All Deletions
Max Sum Significance Signatures
4000
2000
0
censored
Deceased
Survtime
RiskGroupDel
RiskGroupAmp
RiskGroupAmpDel
C4BPB (1q32.2)
GLRX2 (1q31.2)
ZNF703 (8p11.23)
SARNP (12q13.2)
RPSAP52 (12q14.3)
KMT2A (11q23.3)
PRDM10 (11q24.3)
UQCR10 (22q12.2)
GGA1 (22q13.1)
CCNA1 (13q13.3)
MIR5006 (13q14.11)
CAMKV (3p21.31)
AKAP6 (14q12)
C14orf182 (14q21.3)
PCBP2 (12q13.13)
RN7SKP199 (4p12)
KIF5A (12q13.3)
METTL1 (12q14.1)
SIGLEC9 (19q13.41)
SEPT7P9 (10p11.1)
CA4 (17q23.1)
USH1G (17q25.1)
RN7SL404P (17q24.1)
SRSF1 (17q22)
MTRNR2L1 (17p11.2)
GABRA2 (4p12)
RN7SKP82 (4p13)
RNA5SP162 (4q12)
ZNF493 (19p12)
ZNF136 (19p13.2)
RiskGroup
High
Low
NotSig
RiskGroupSingleData
High
Low
NotSig
CNA
>=1
0
<= -1
shown (30/41),
without filtering = 946
samples (55)
p.Valorate <.05

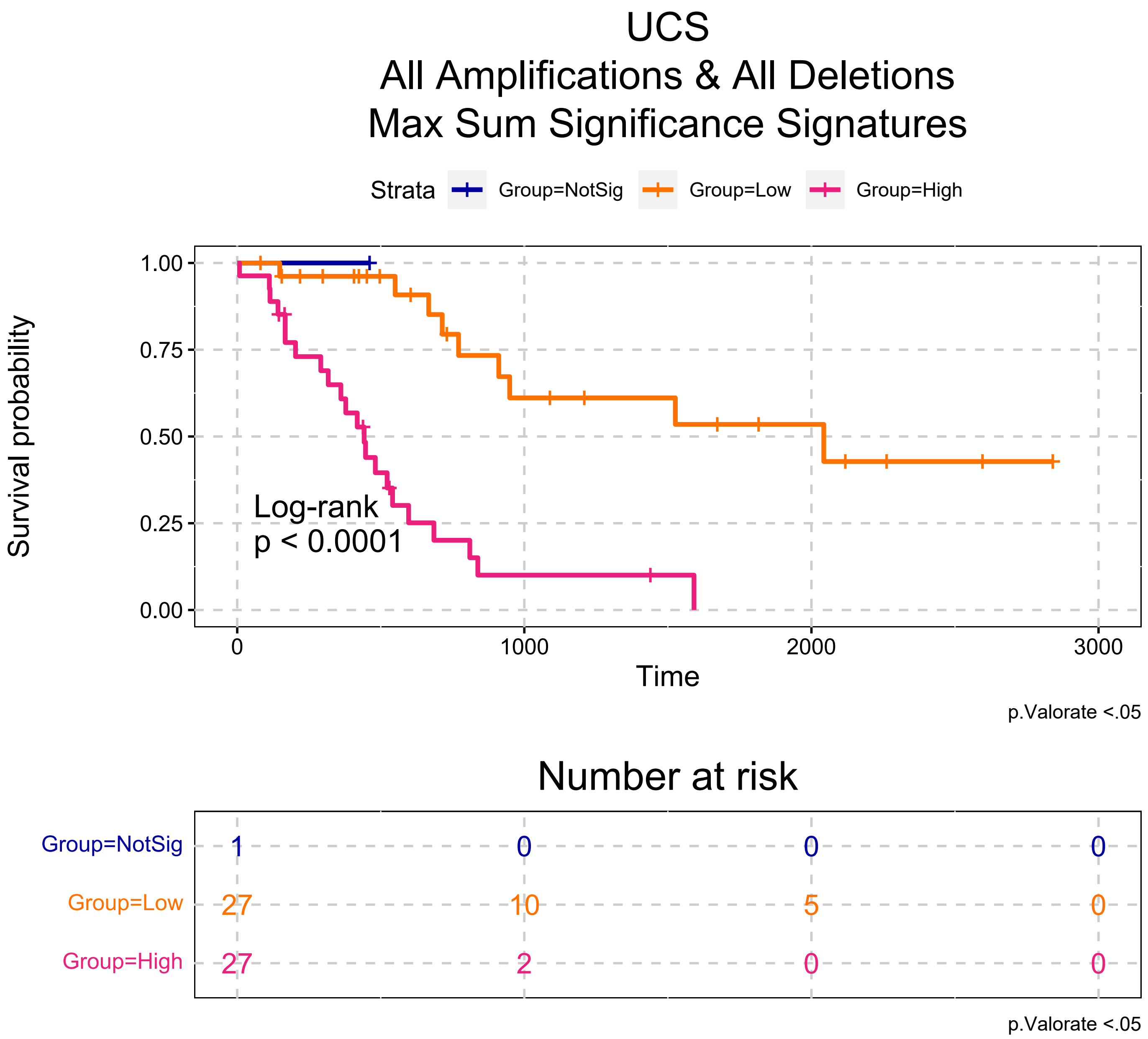


| explanatory | beta | HR | L95 | U95 | p |
|---|---|---|---|---|---|
| Low | 15.27 | 4290827.20 | 0.00 | Inf | 1.00 |
| High | 17.12 | 27293093.57 | 0.00 | Inf | 1.00 |

n= 55, number of events =31
Score(logrank) test = p <.0001

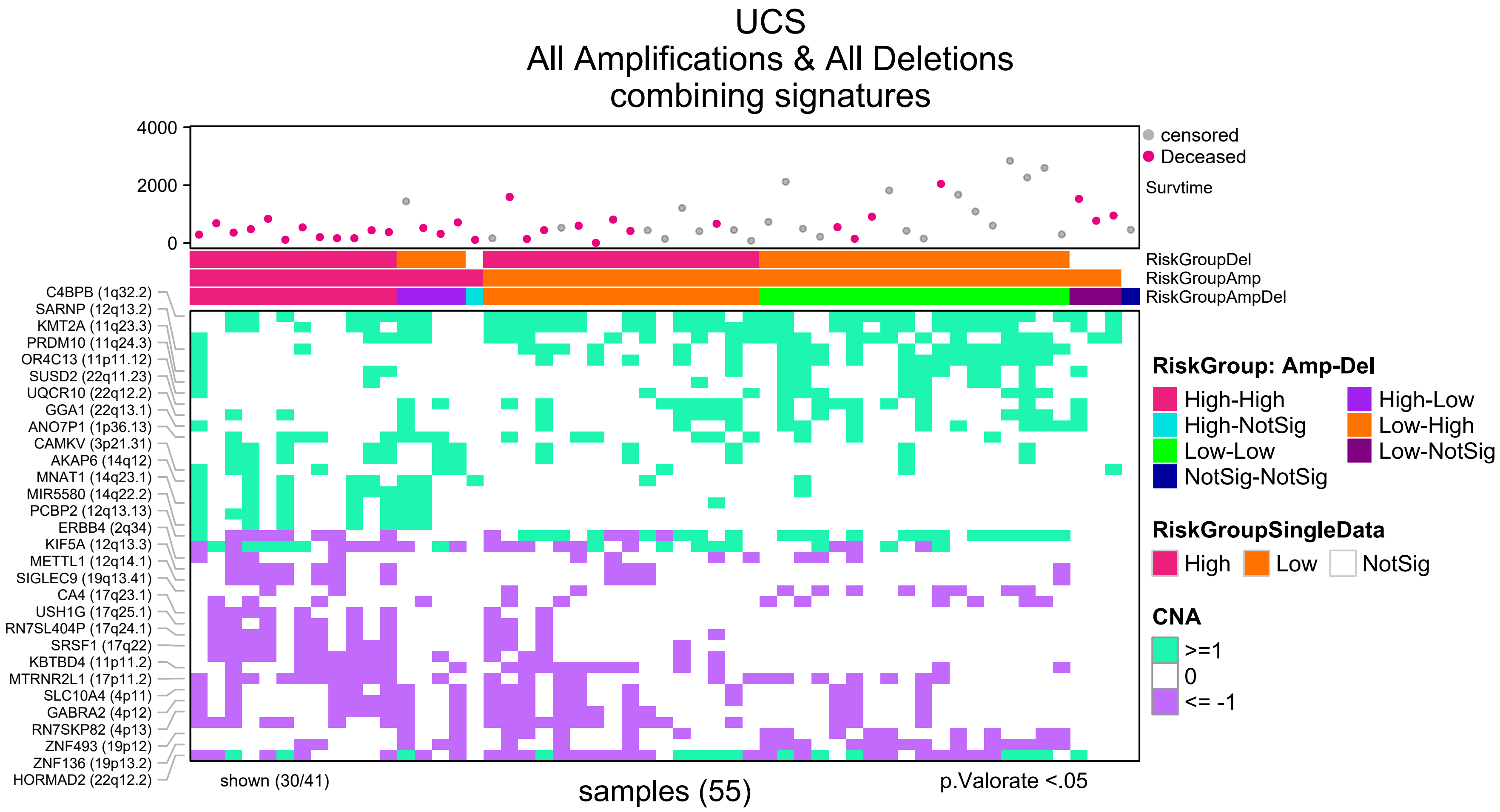

UCS
All Amplifications & All Deletions
combining signatures
4000
2000
0
censored
Deceased
Survtime
RiskGroupDel
RiskGroupAmp
RiskGroupAmpDel
C4BPB (1q32.2)
SARNP (12q13.2)
KMT2A (11q23.3)
PRDM10 (11q24.3)
OR4C13 (11p11.12)
SUSD2 (22q11.23)
UQCR10 (22q12.2)
GGA1 (22q13.1)
ANO7P1 (1p36.13)
CAMKV (3p21.31)
AKAP6 (14q12)
MNAT1 (14q23.1)
MIR5580 (14q22.2)
PCBP2 (12q13.13)
ERBB4 (2q34)
KIF5A (12q13.3)
METTL1 (12q14.1)
SIGLEC9 (19q13.41)
CA4 (17q23.1)
USH1G (17q25.1)
RN7SL404P (17q24.1)
SRSF1 (17q22)
KBTBD4 (11p11.2)
MTRNR2L1 (17p11.2)
SLC10A4 (4p11)
GABRA2 (4p12)
RN7SKP82 (4p13)
ZNF493 (19p12)
ZNF136 (19p13.2)
HORMAD2 (22q12.2)
shown (30/41)
samples (55)
p.Valorate <.05
RiskGroup: Amp-Del
High-High
High-NotSig
Low-Low
NotSig-NotSig
High-Low
Low-High
Low-NotSig
RiskGroupSingleData
High
Low
NotSig
CNA
>=1
0
<= -1

# UCS
## All Amplifications & All Deletions combining signatures

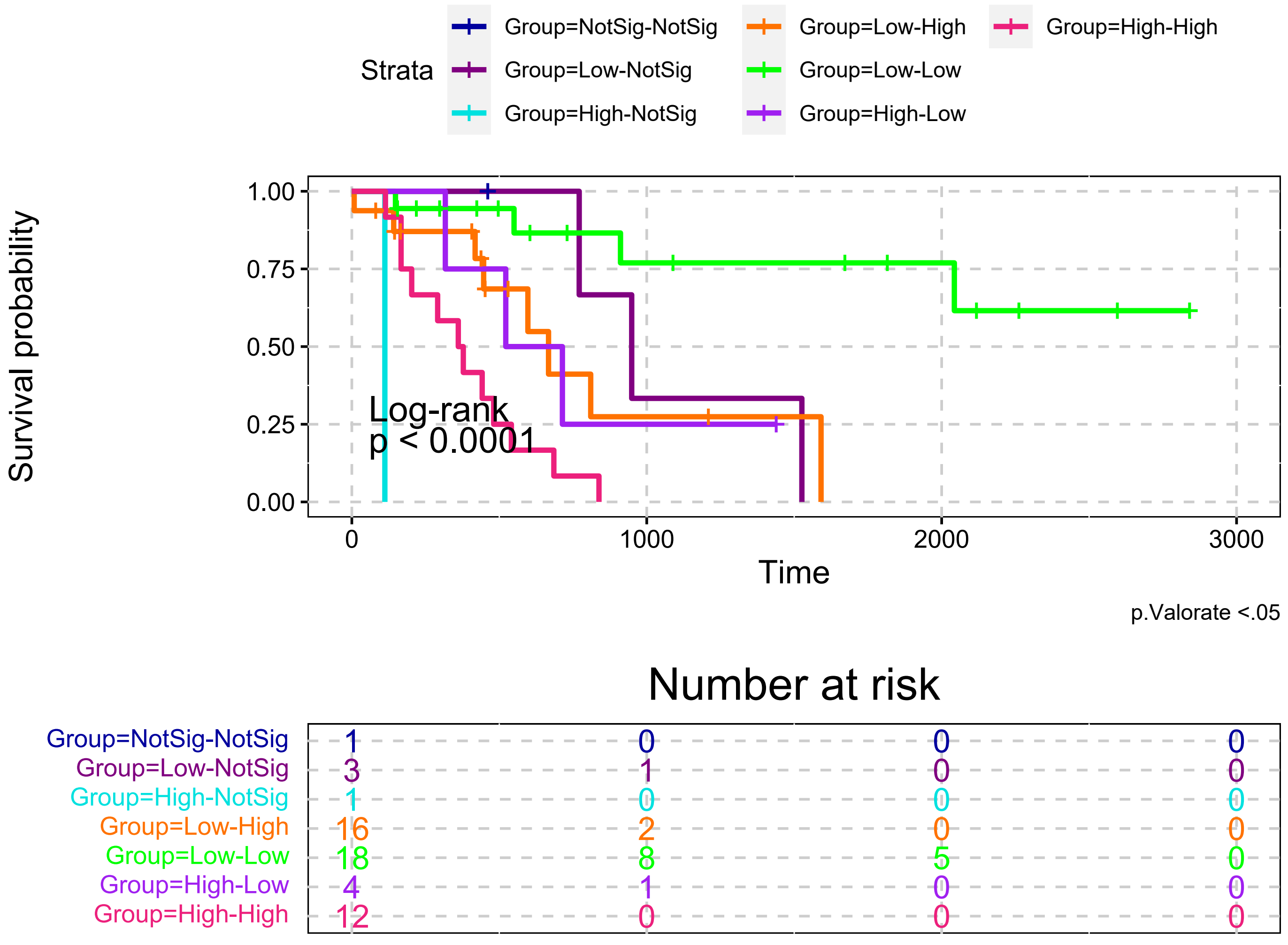


RiskGroup: Amp-Del, p.Valorate <.05

| explanatory | beta | HR | L95 | U95 |
|---|---|---|---|---|
| Low-NotSig | 10.85 | 51488.52 | 15046.96 | 176186.26 |
| High-NotSig | 15.29 | 4381598.13 | 274038.46 | 70057327.72 |
| Low-High | 11.18 | 71672.07 | 31564.31 | 162743.47 |
| Low-Low | 9.18 | 9742.78 | 2755.80 | 34444.38 |
| High-Low | 11.24 | 75832.61 | 22692.17 | 253417.08 |
| High-High | 12.31 | 222828.11 | 100898.39 | 492102.66 |

n= 55, number of events =31
Score(logrank) test = p <.0001

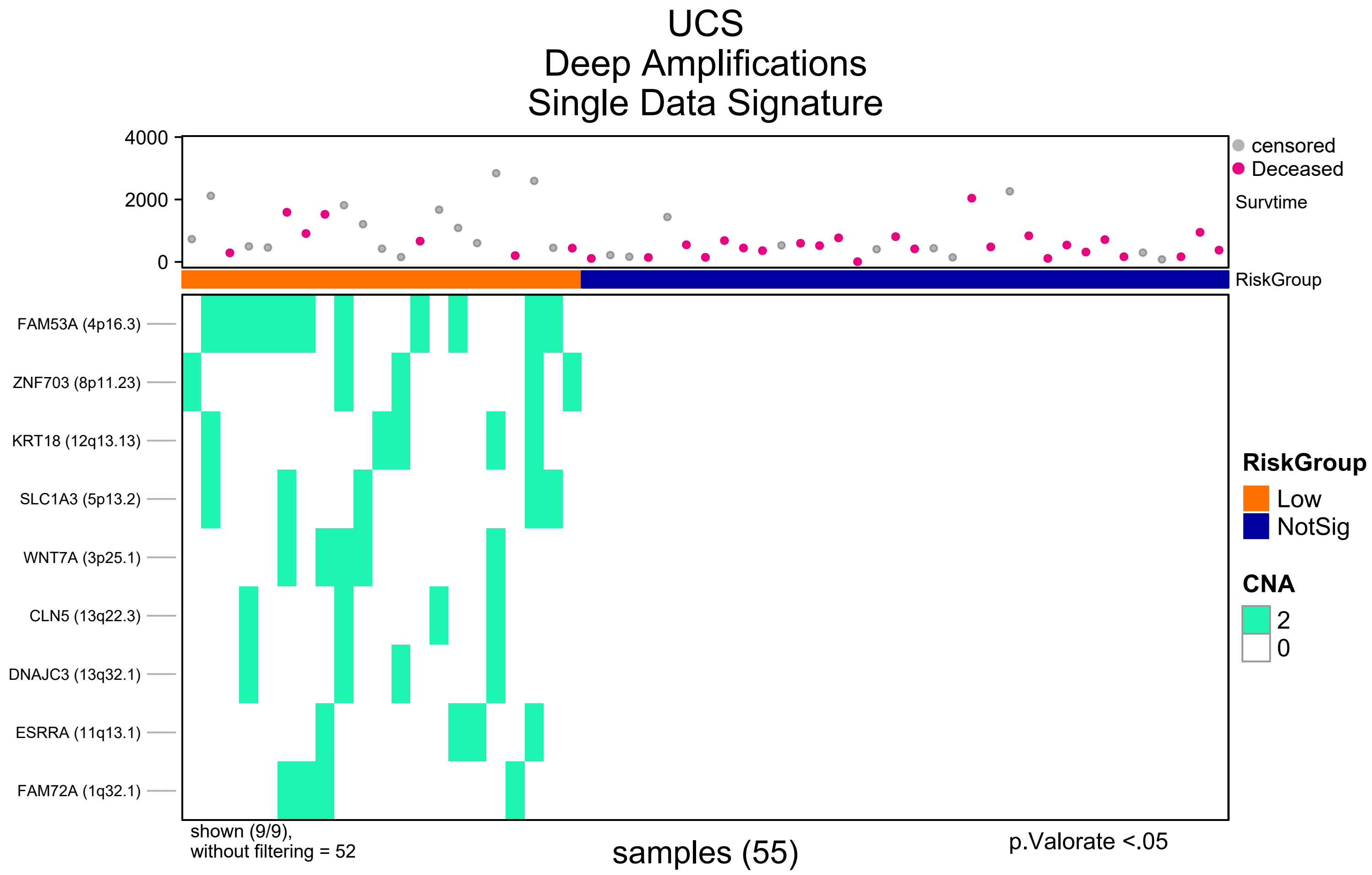
UCS
Deep Amplifications
Single Data Signature
4000
2000
0
censored
Deceased
Survtime
RiskGroup
FAM53A (4p16.3)
ZNF703 (8p11.23)
KRT18 (12q13.13)
SLC1A3 (5p13.2)
WNT7A (3p25.1)
CLN5 (13q22.3)
DNAJC3 (13q32.1)
ESRRA (11q13.1)
FAM72A (1q32.1)
RiskGroup
Low
NotSig
CNA
2
0
shown (9/9),
without filtering = 52
samples (55)
p.Valorate <.05

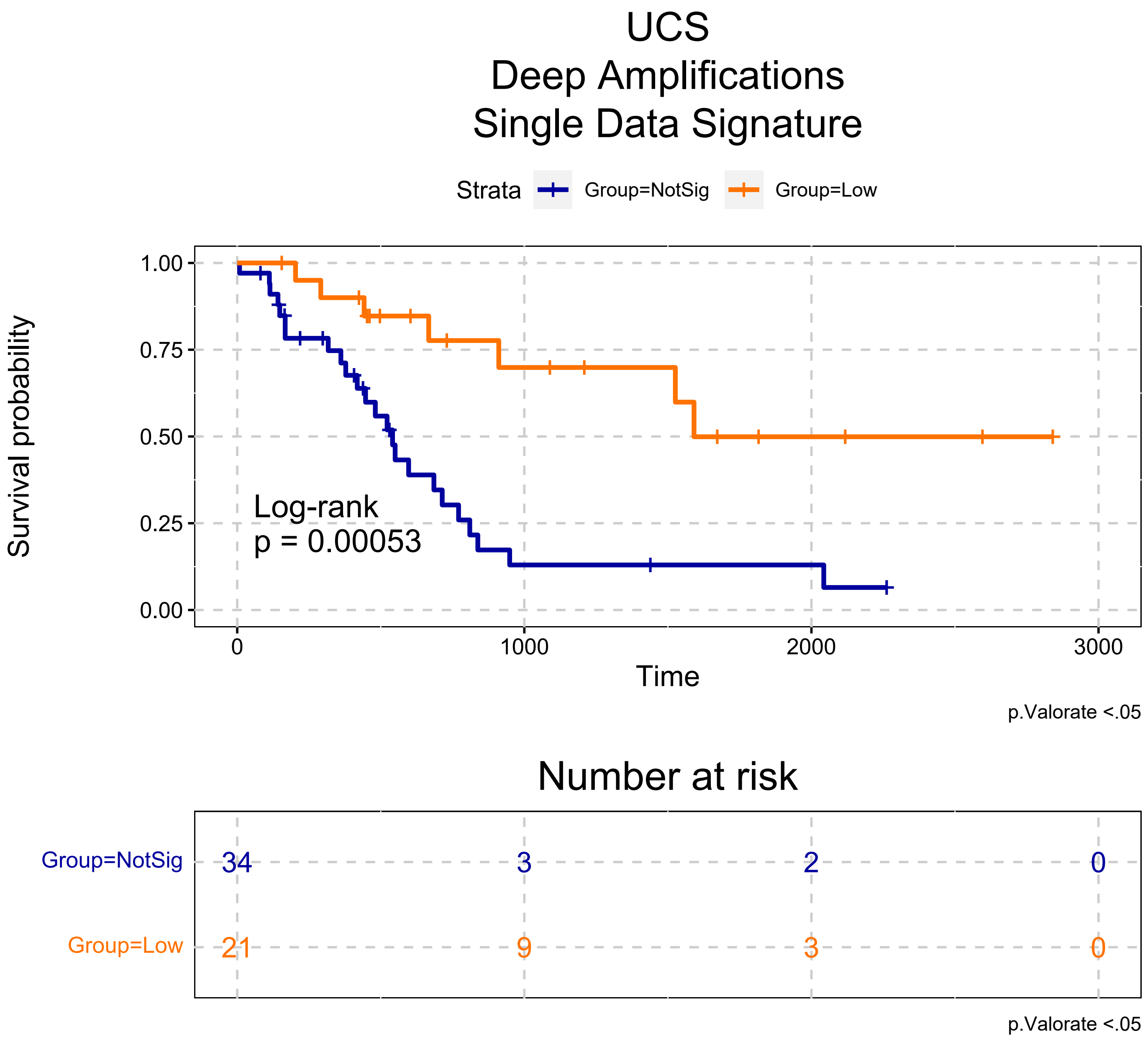


| explanatory | beta | HR | L95 | U95 | p |
|---|---|---|---|---|---|
| Low | -1.41 | 0.24 | 0.10 | 0.57 | 0.00 |

n= 55, number of events =31
Score(logrank) test = 0.001
