# Supplementary material for: Systematic Modeling of Risk-Associated Copy Number Alterations in Cancer
Source: Int J Mol Sci. 2024 Sep 27;25(19):10455. doi: 10.3390/ijms251910455 (PMC11477427; doi:10.3390/ijms251910455)
Supplement: Supplementary file 1 [file ijms-25-10455-s001.zip › UVMSignatureV12-sinSombreado.pdf]

UVM
All Amplifications
Single Data Signature

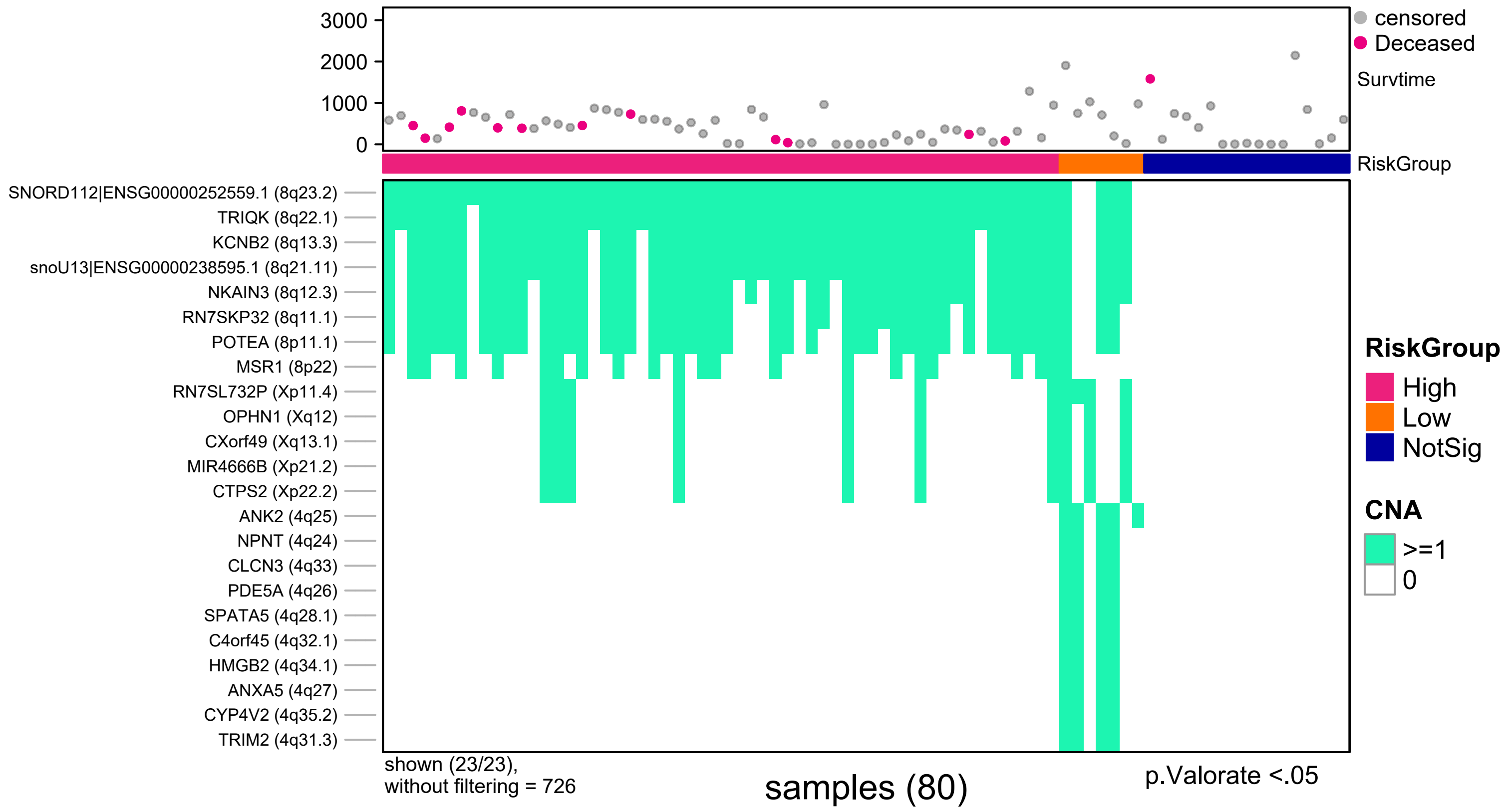

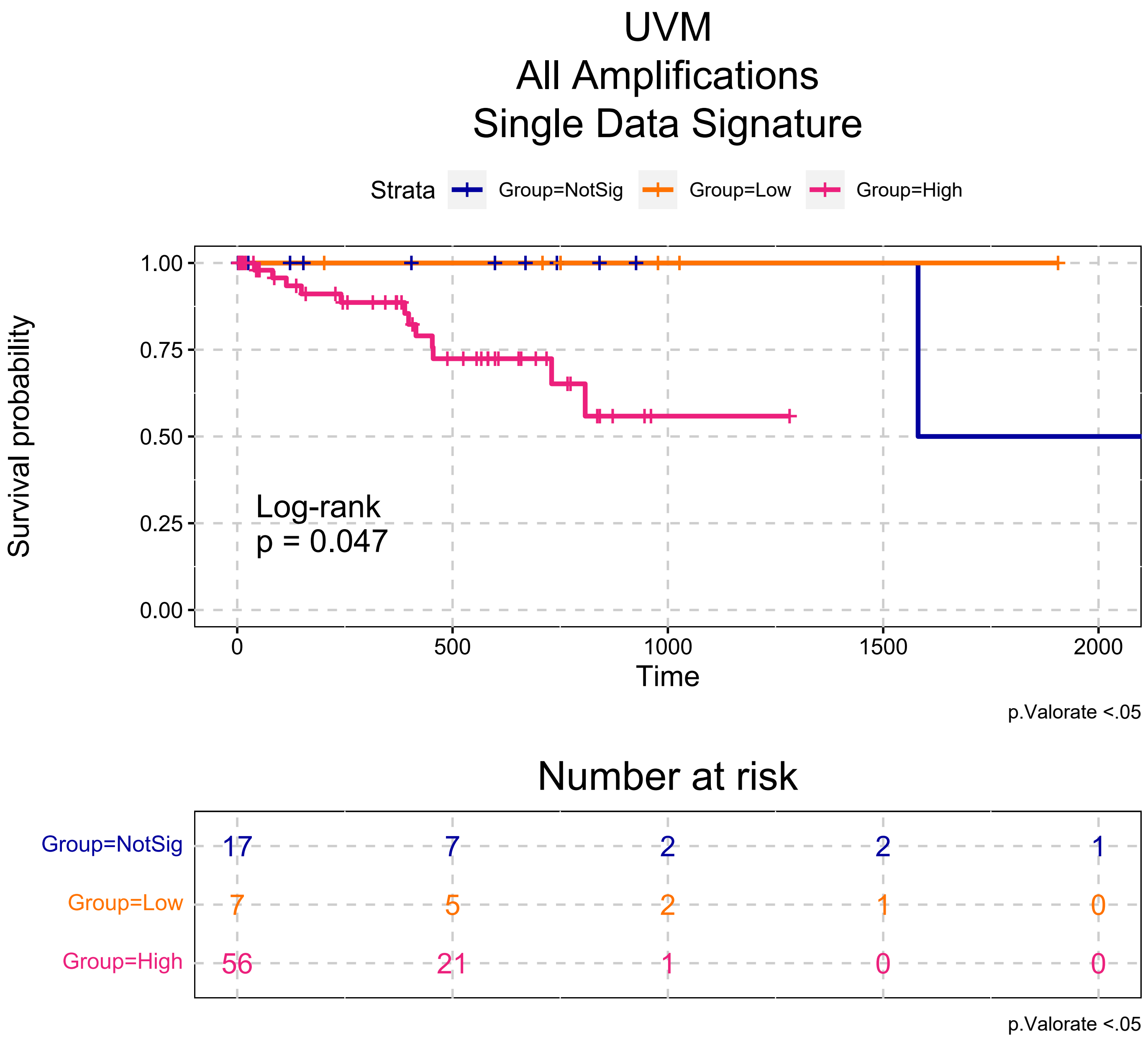

explanatory	beta	HR	L95	U95	p
Low	-18.51	0.00	0.00	Inf	1.00
High	20.05	509686428.12	0.00	Inf	1.00

n= 80, number of events =13
Score(logrank) test = 0.047

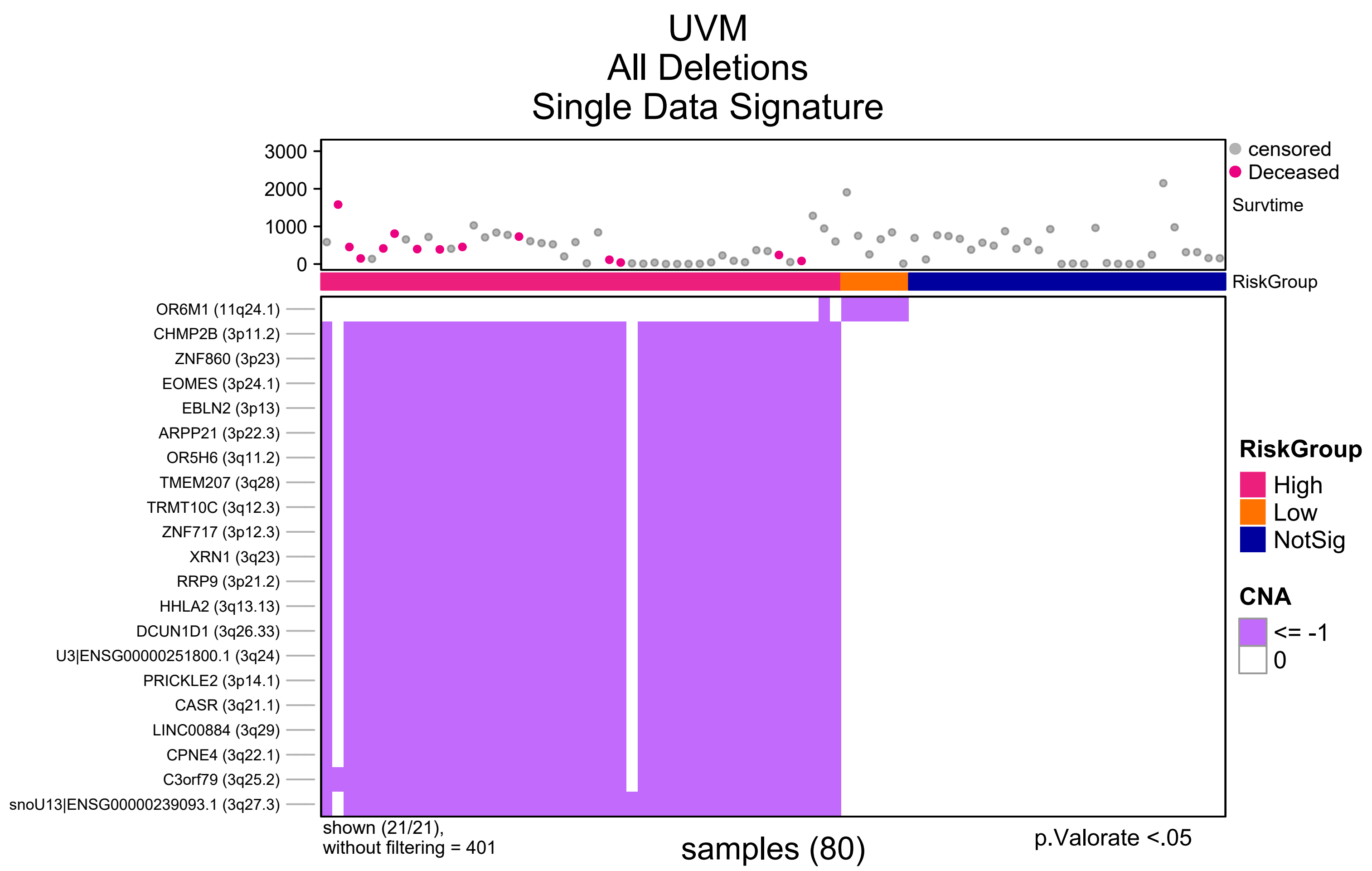

UVM
All Deletions
Single Data Signature
3000
2000
1000
0
censored
Deceased
Survtime
RiskGroup
OR6M1 (11q24.1)
CHMP2B (3p11.2)
ZNF860 (3p23)
EOMES (3p24.1)
EBLN2 (3p13)
ARPP21 (3p22.3)
OR5H6 (3q11.2)
TMEM207 (3q28)
TRMT10C (3q12.3)
ZNF717 (3p12.3)
XRN1 (3q23)
RRP9 (3p21.2)
HHLA2 (3q13.13)
DCUN1D1 (3q26.33)
U3|ENSG00000251800.1 (3q24)
PRICKLE2 (3p14.1)
CASR (3q21.1)
LINC00884 (3q29)
CPNE4 (3q22.1)
C3orf79 (3q25.2)
snoU13|ENSG00000239093.1 (3q27.3)
RiskGroup
High
Low
NotSig
CNA
<= -1
0
shown (21/21),
without filtering = 401
samples (80)
p.Valorate <.05

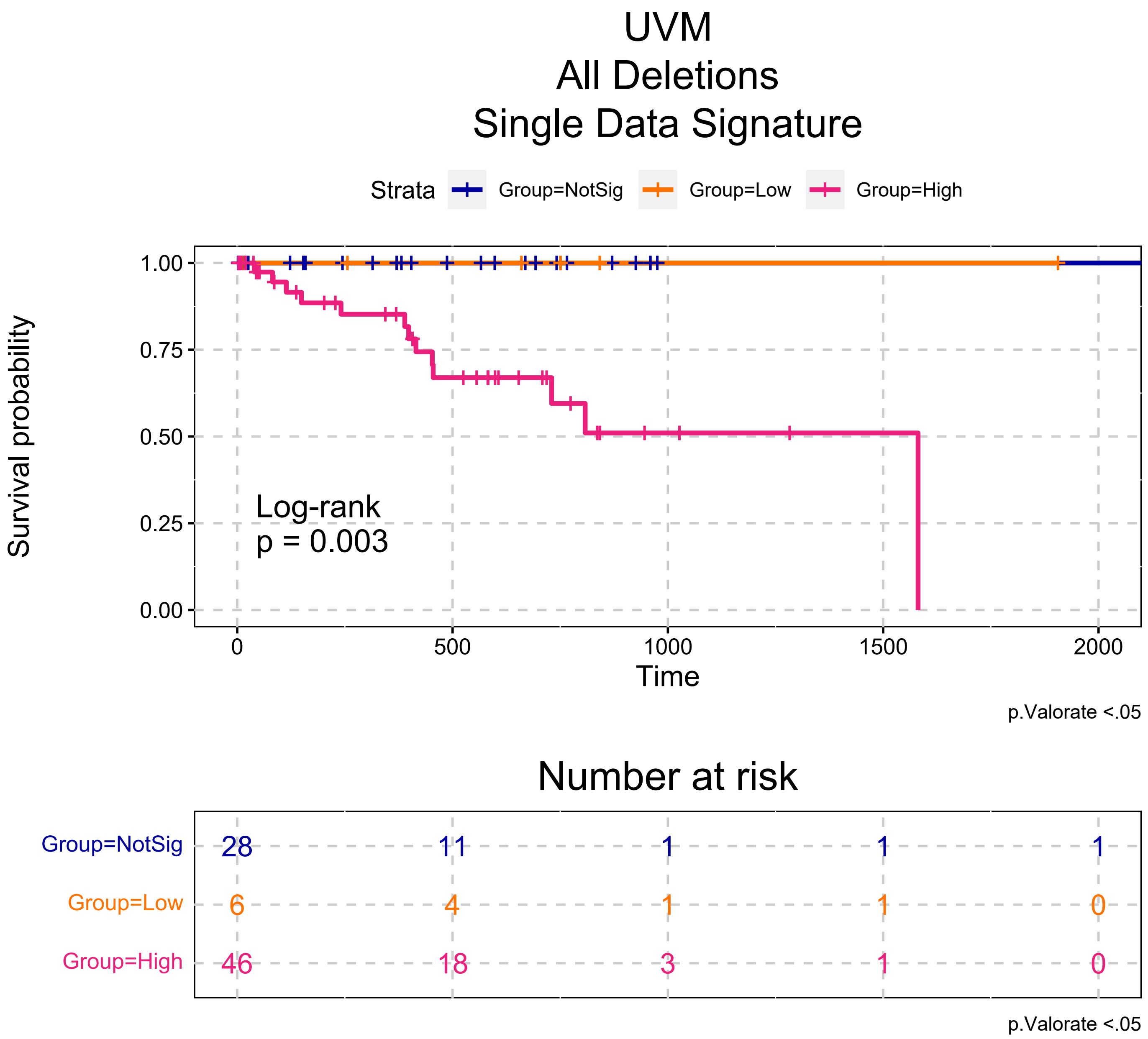

explanatory	beta	HR	L95	U95	p
Low	-0.10	0.90	0.00	Inf	1.00
High	21.10	1462043357.74	0.00	Inf	1.00

n= 80, number of events =13
Score(logrank) test = 0.003

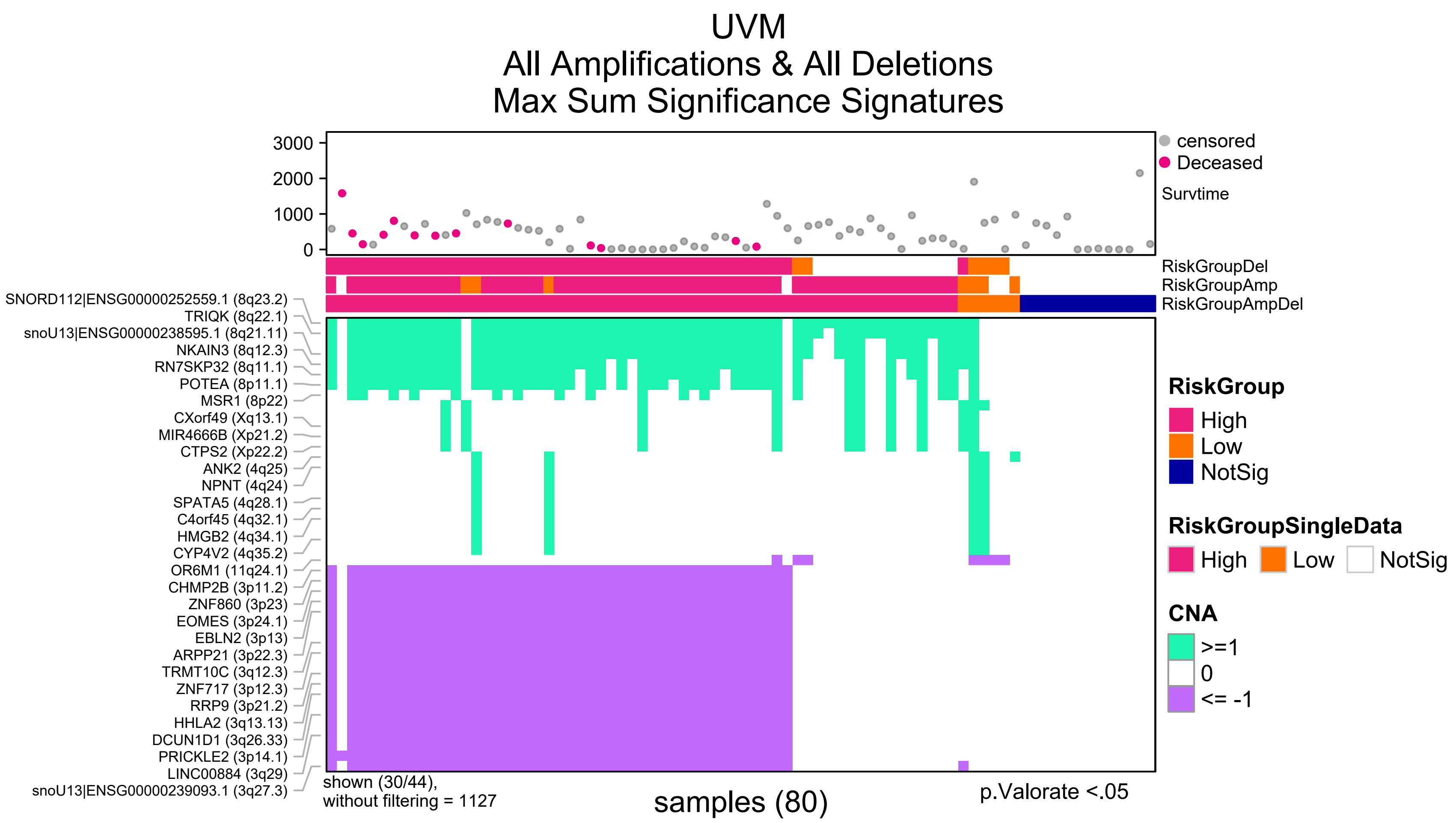
UVM
All Amplifications & All Deletions
Max Sum Significance Signatures
3000
2000
1000
0
censored
Deceased
Survtime
RiskGroupDel
RiskGroupAmp
RiskGroupAmpDel
SNORD112|ENSG00000252559.1 (8q23.2)
TRIQK (8q22.1)
snoU13|ENSG00000238595.1 (8q21.11)
NKAIN3 (8q12.3)
RN7SKP32 (8q11.1)
POTEA (8p11.1)
MSR1 (8p22)
CXorf49 (Xq13.1)
MIR4666B (Xp21.2)
CTPS2 (Xp22.2)
ANK2 (4q25)
NPNT (4q24)
SPATA5 (4q28.1)
C4orf45 (4q32.1)
HMGB2 (4q34.1)
CYP4V2 (4q35.2)
OR6M1 (11q24.1)
CHMP2B (3p11.2)
ZNF860 (3p23)
EOMES (3p24.1)
EBLN2 (3p13)
ARPP21 (3p22.3)
TRMT10C (3q12.3)
ZNF717 (3p12.3)
RRP9 (3p21.2)
HHLA2 (3q13.13)
DCUN1D1 (3q26.33)
PRICKLE2 (3p14.1)
LINC00884 (3q29)
snoU13|ENSG00000239093.1 (3q27.3)
RiskGroup
High
Low
NotSig
RiskGroupSingleData
High
Low
NotSig
CNA
>=1
0
<= -1
shown (30/44),
without filtering = 1127
samples (80)
p.Valorate <.05

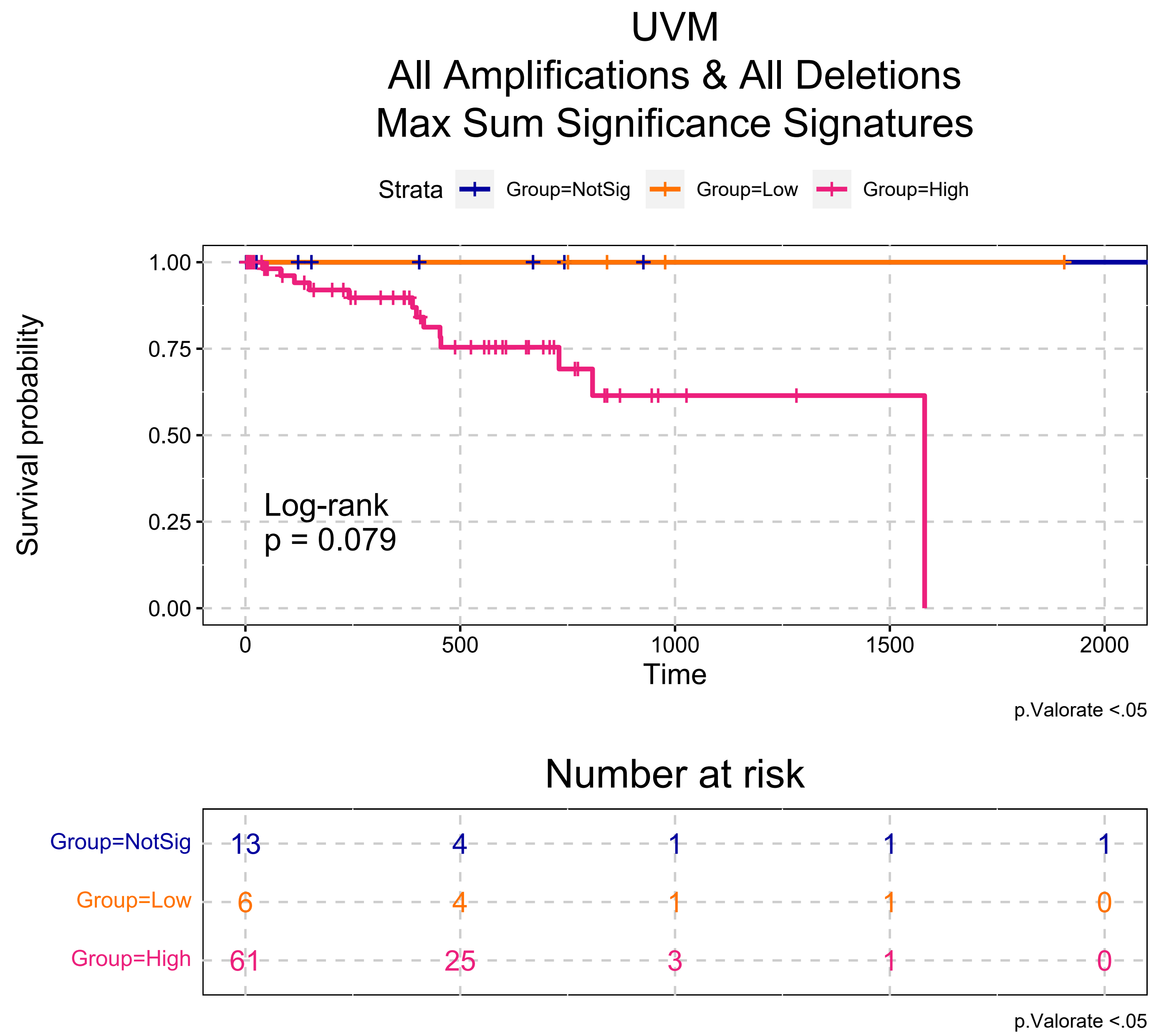

explanatory	beta	HR	L95	U95	p
Low	-0.05	0.95	0.00	Inf	1.00
High	19.72	367967760.10	0.00	Inf	1.00

n= 80, number of events =13
Score(logrank) test = 0.079

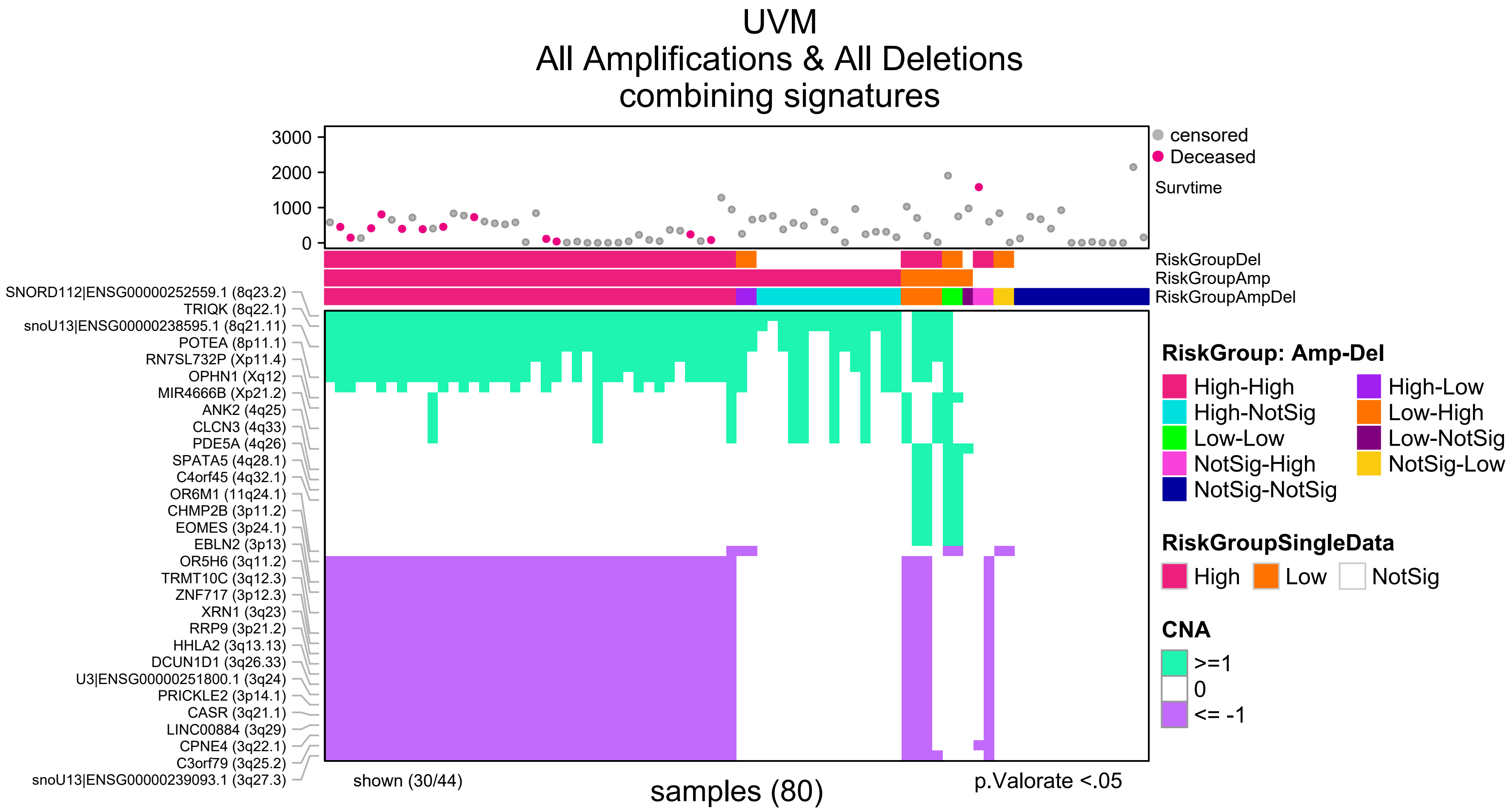
UVM
All Amplifications & All Deletions
combining signatures
3000
2000
1000
0
censored
Deceased
Survtime
RiskGroupDel
RiskGroupAmp
RiskGroupAmpDel
SNORD112|ENSG00000252559.1 (8q23.2)
TRIQK (8q22.1)
snoU13|ENSG00000238595.1 (8q21.11)
POTEA (8p11.1)
RN7SL732P (Xp11.4)
OPHN1 (Xq12)
MIR4666B (Xp21.2)
ANK2 (4q25)
CLCN3 (4q33)
PDE5A (4q26)
SPATA5 (4q28.1)
C4orf45 (4q32.1)
OR6M1 (11q24.1)
CHMP2B (3p11.2)
EOMES (3p24.1)
EBLN2 (3p13)
OR5H6 (3q11.2)
TRMT10C (3q12.3)
ZNF717 (3p12.3)
XRN1 (3q23)
RRP9 (3p21.2)
HHLA2 (3q13.13)
DCUN1D1 (3q26.33)
U3|ENSG00000251800.1 (3q24)
PRICKLE2 (3p14.1)
CASR (3q21.1)
LINC00884 (3q29)
CPNE4 (3q22.1)
C3orf79 (3q25.2)
snoU13|ENSG00000239093.1 (3q27.3)
shown (30/44)
samples (80)
p.Valorate <.05
RiskGroup: Amp-Del
High-High
High-NotSig
Low-Low
NotSig-High
NotSig-NotSig
High-Low
Low-High
Low-NotSig
NotSig-Low
RiskGroupSingleData
High
Low
NotSig
CNA
>=1
0
<= -1

UVM
All Amplifications & All Deletions combining signatures

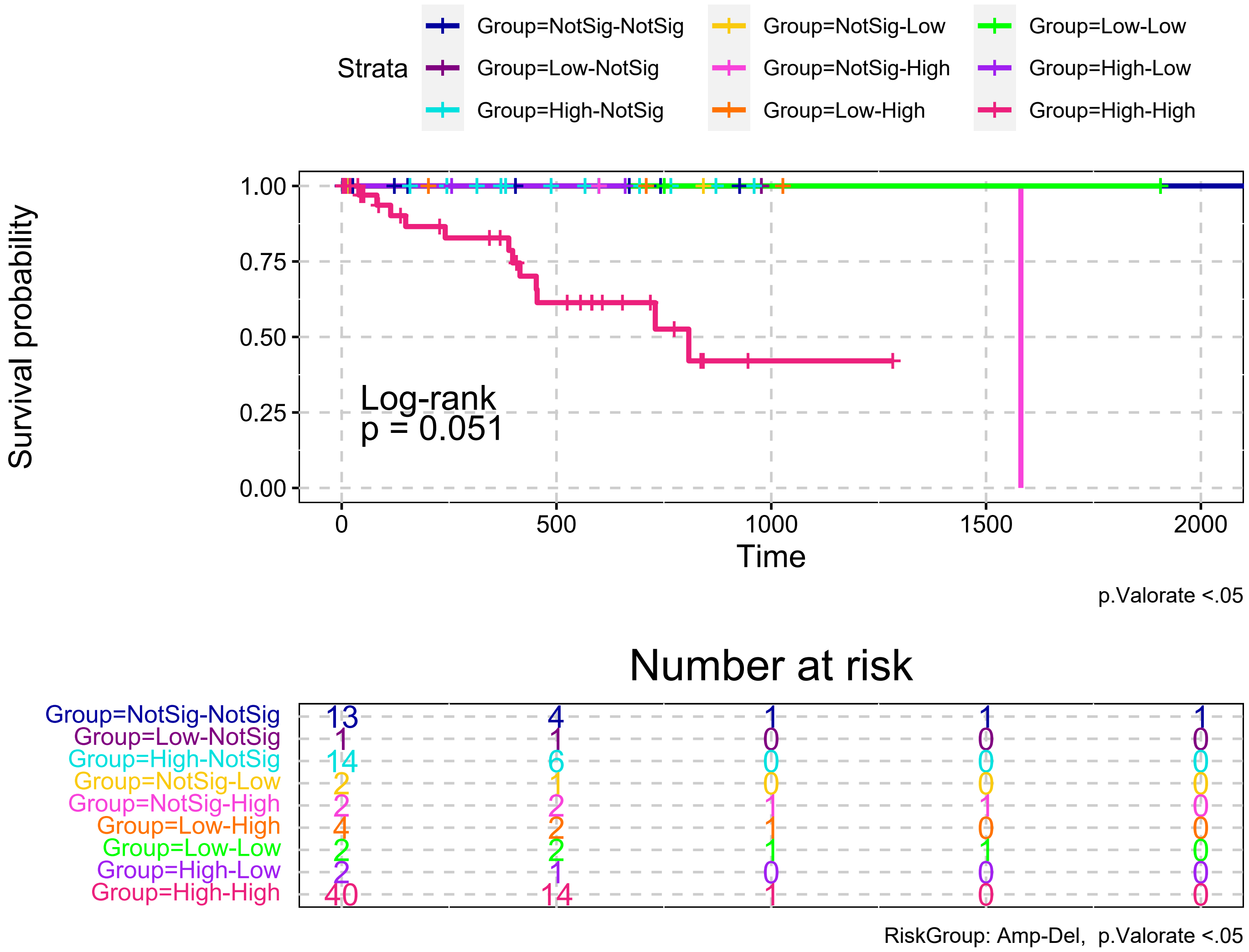

explanatory	beta	HR	L95	U95
Low-NotSig	17.68	47747684.11	0.00	Inf
High-NotSig	17.77	52028142.90	0.00	Inf
NotSig-Low	17.68	47747684.11	0.00	Inf
NotSig-High	19.66	347030283.48	0.00	Inf
Low-High	17.74	50693132.99	0.00	Inf
Low-Low	-0.63	0.53	0.00	Inf
High-Low	17.81	54488343.01	0.00	Inf
High-High	39.20	106008727185800560.00	0.00	Inf

n= 80, number of events =13
Score(logrank) test = 0.051

UVM
Deep Amplifications
Single Data Signature

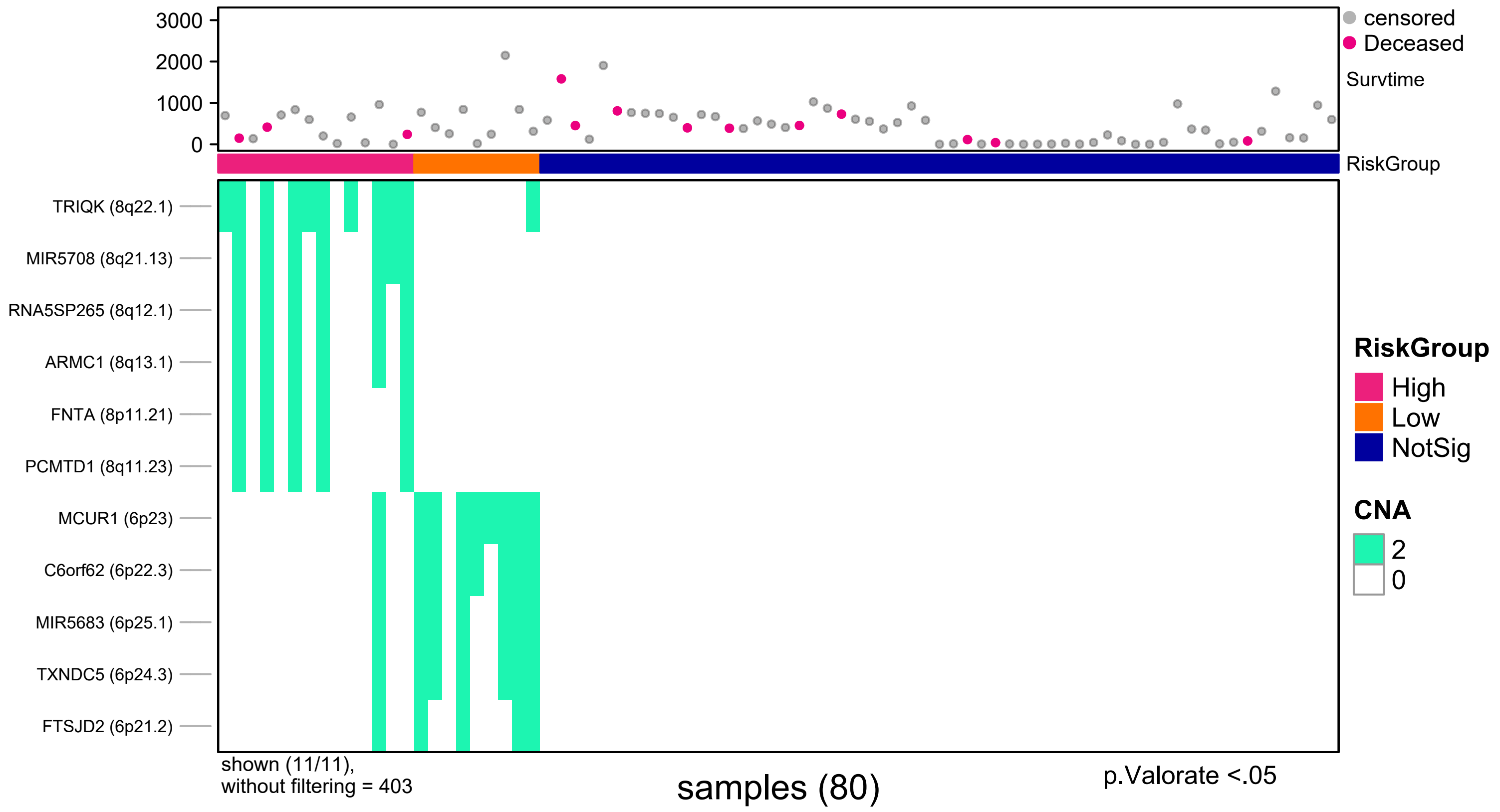

UVM
Deep Amplifications
Single Data Signature

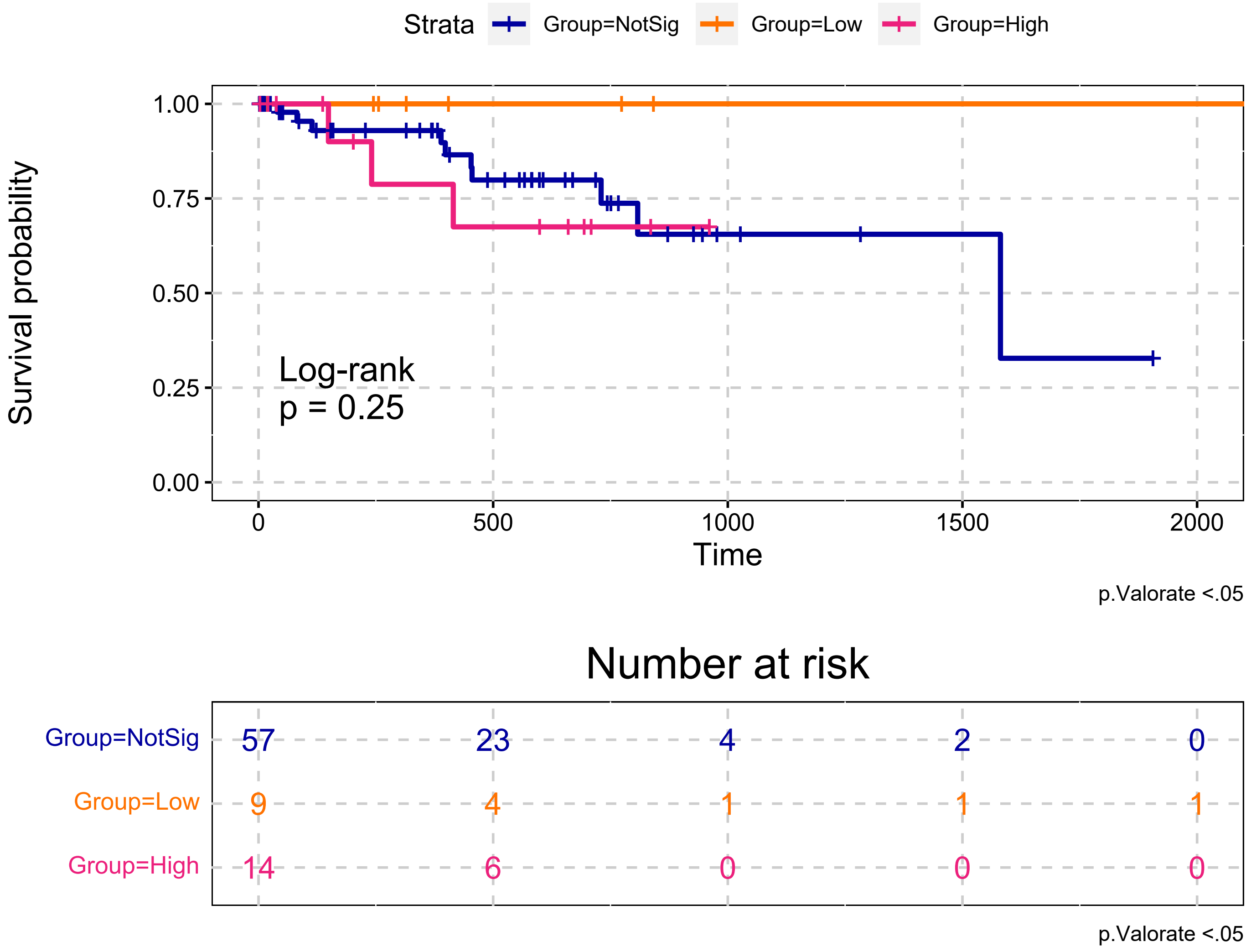

explanatory	beta	HR	L95	U95	p
Low	-19.28	0.00	0.00	Inf	1.00
High	0.32	1.38	0.37	5.12	0.63

n= 80, number of events =13
Score(logrank) test = 0.253
