# Supplementary material for: Systematic Modeling of Risk-Associated Copy Number Alterations in Cancer
Source: Int J Mol Sci. 2024 Sep 27;25(19):10455. doi: 10.3390/ijms251910455 (PMC11477427; doi:10.3390/ijms251910455)
Supplement: Supplementary file 1 [file ijms-25-10455-s001.zip › ACCSignatureV12-sinSombreado.pdf]

ACC
All Amplifications
Single Data Signature

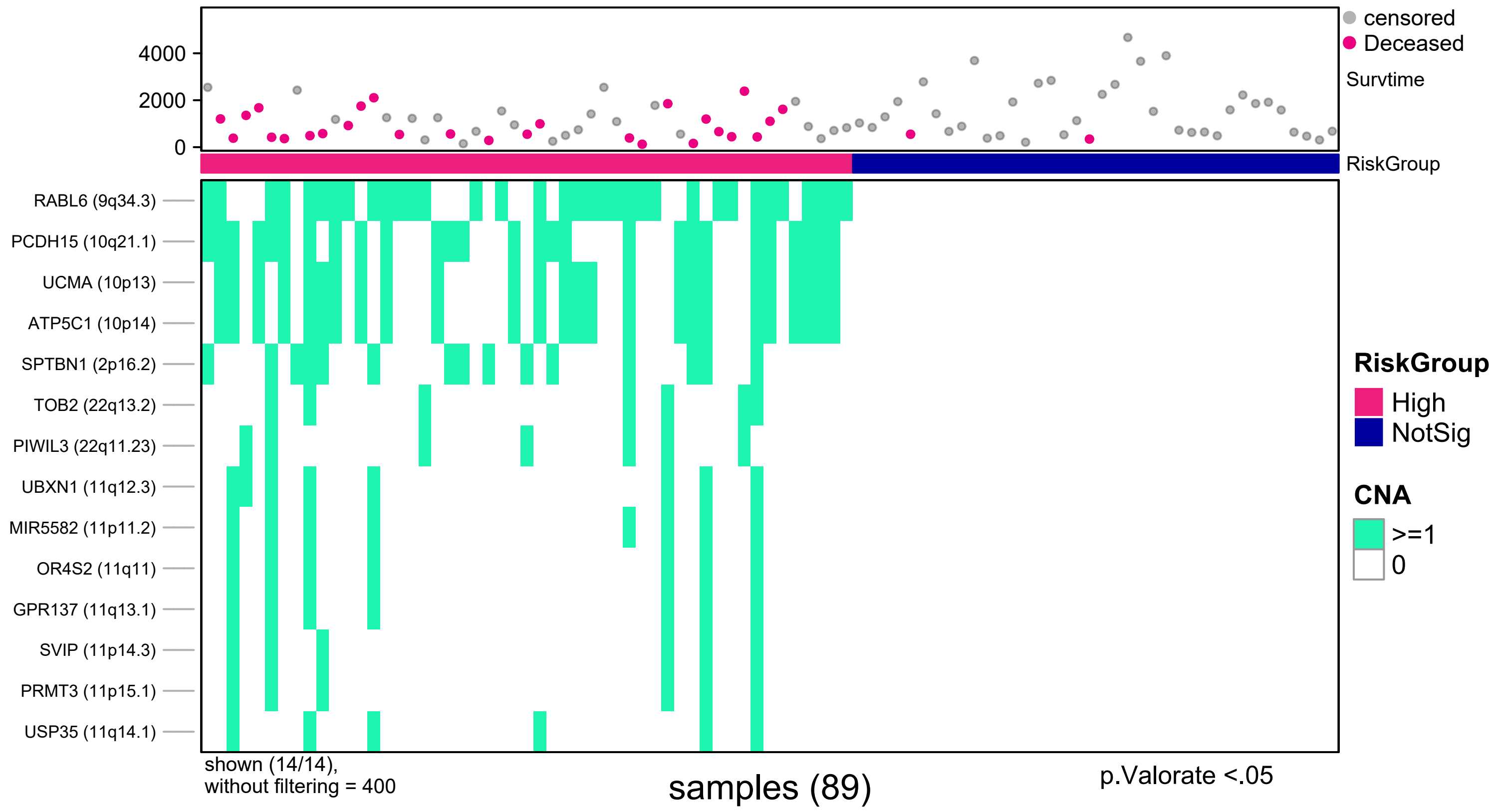

ACC
All Amplifications
Single Data Signature

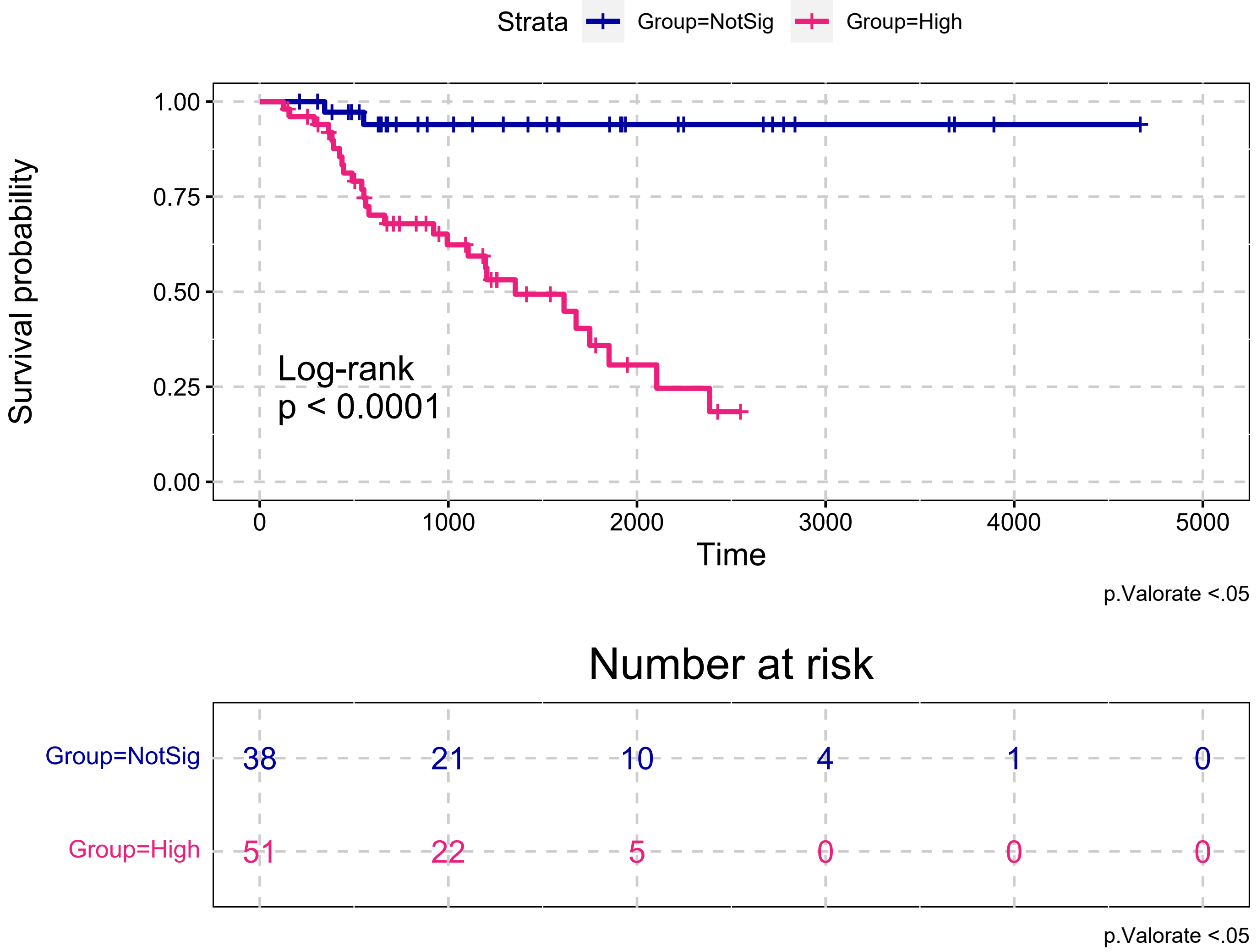

explanatory	beta	HR	L95	U95	p
High	2.64	13.98	3.31	59.12	0.00

n= 89, number of events =29
Score(logrank) test = p <.0001

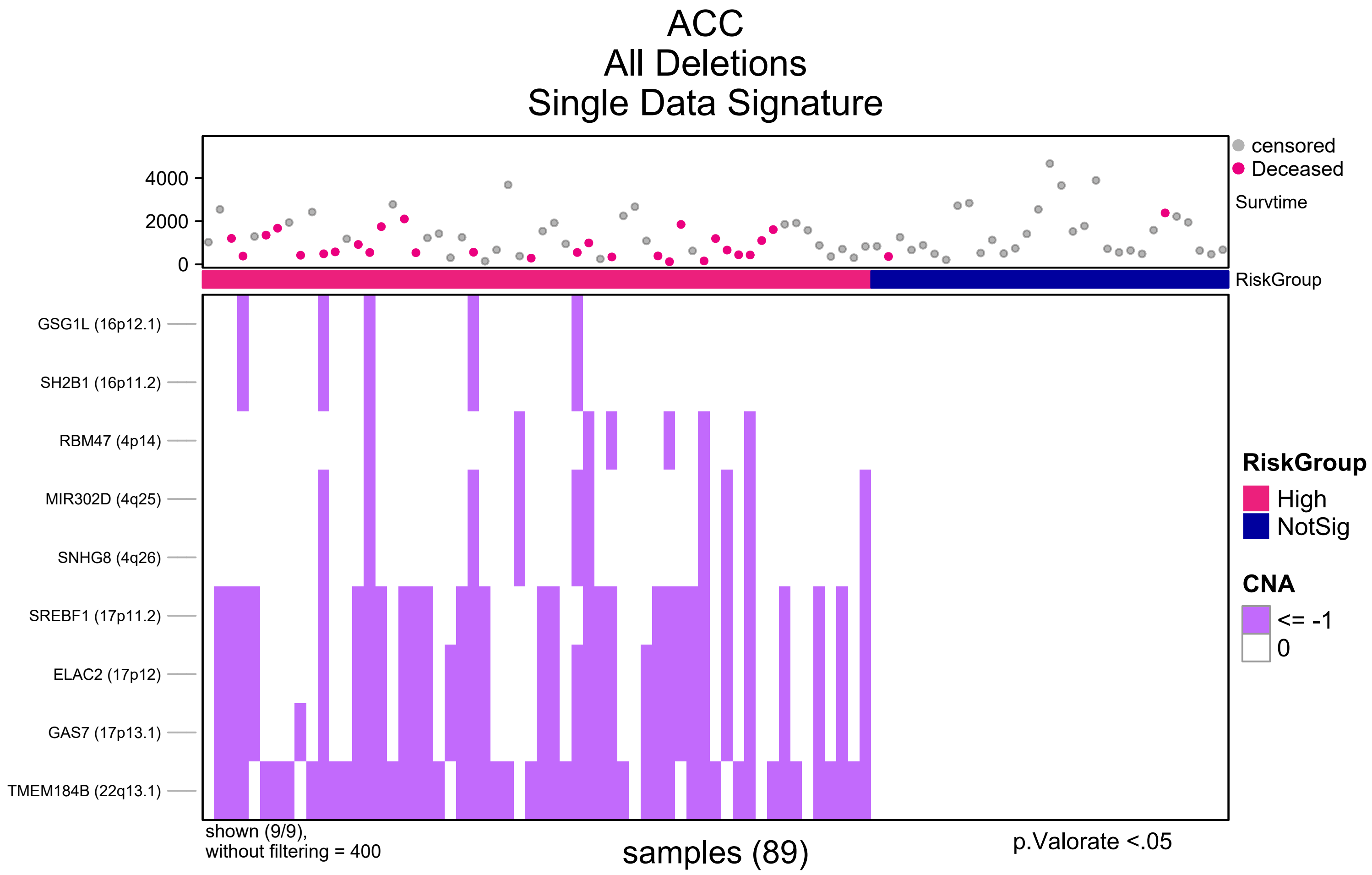
ACC
All Deletions
Single Data Signature
censored
Deceased
Survtime
4000
2000
0
RiskGroup
GSG1L (16p12.1)
SH2B1 (16p11.2)
RBM47 (4p14)
MIR302D (4q25)
SNHG8 (4q26)
SREBF1 (17p11.2)
ELAC2 (17p12)
GAS7 (17p13.1)
TMEM184B (22q13.1)
RiskGroup
High
NotSig
CNA
<= -1
0
shown (9/9),
without filtering = 400
samples (89)
p.Valorate <.05

ACC
All Deletions
Single Data Signature

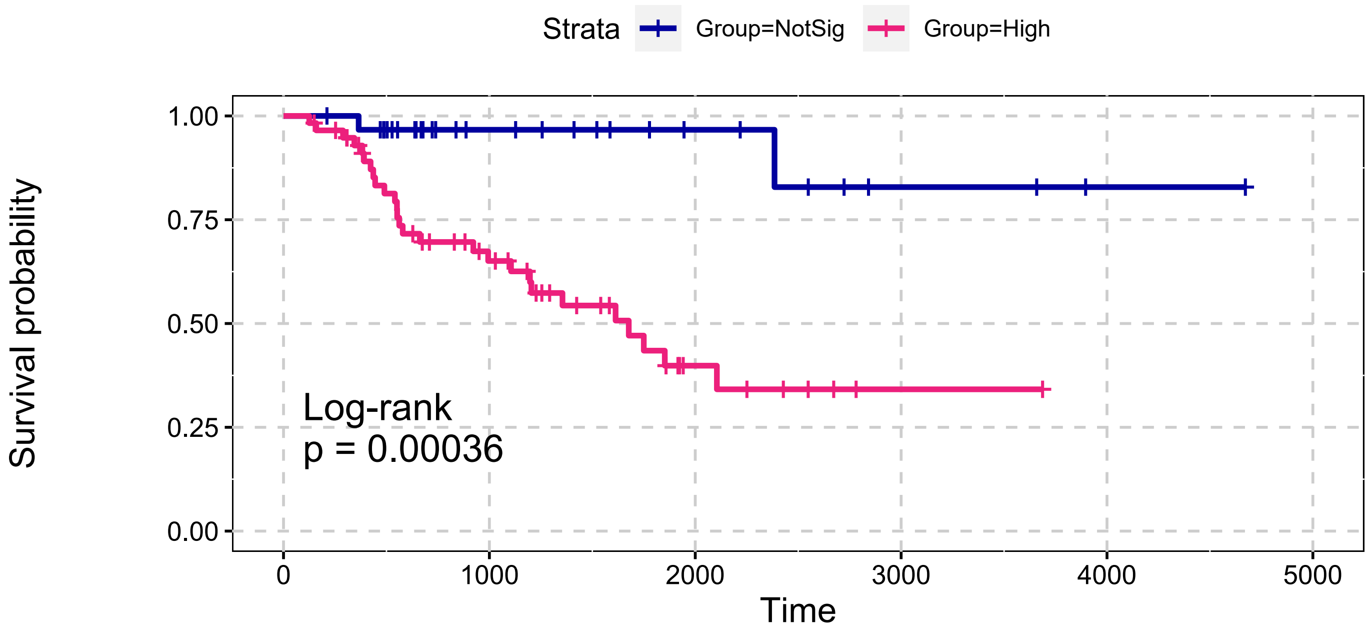

p.Valorate <.05

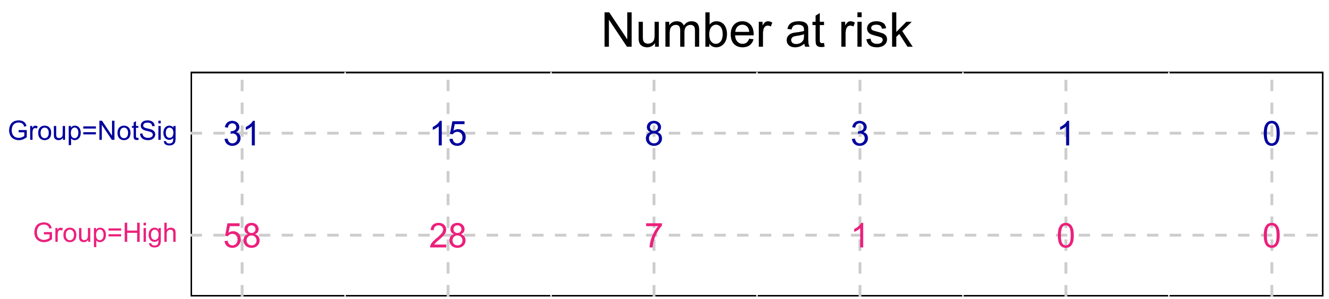

p.Valorate <.05

explanatory	beta	HR	L95	U95	p
High	2.18	8.81	2.09	37.15	0.00

n= 89, number of events =29
Score(logrank) test = 0

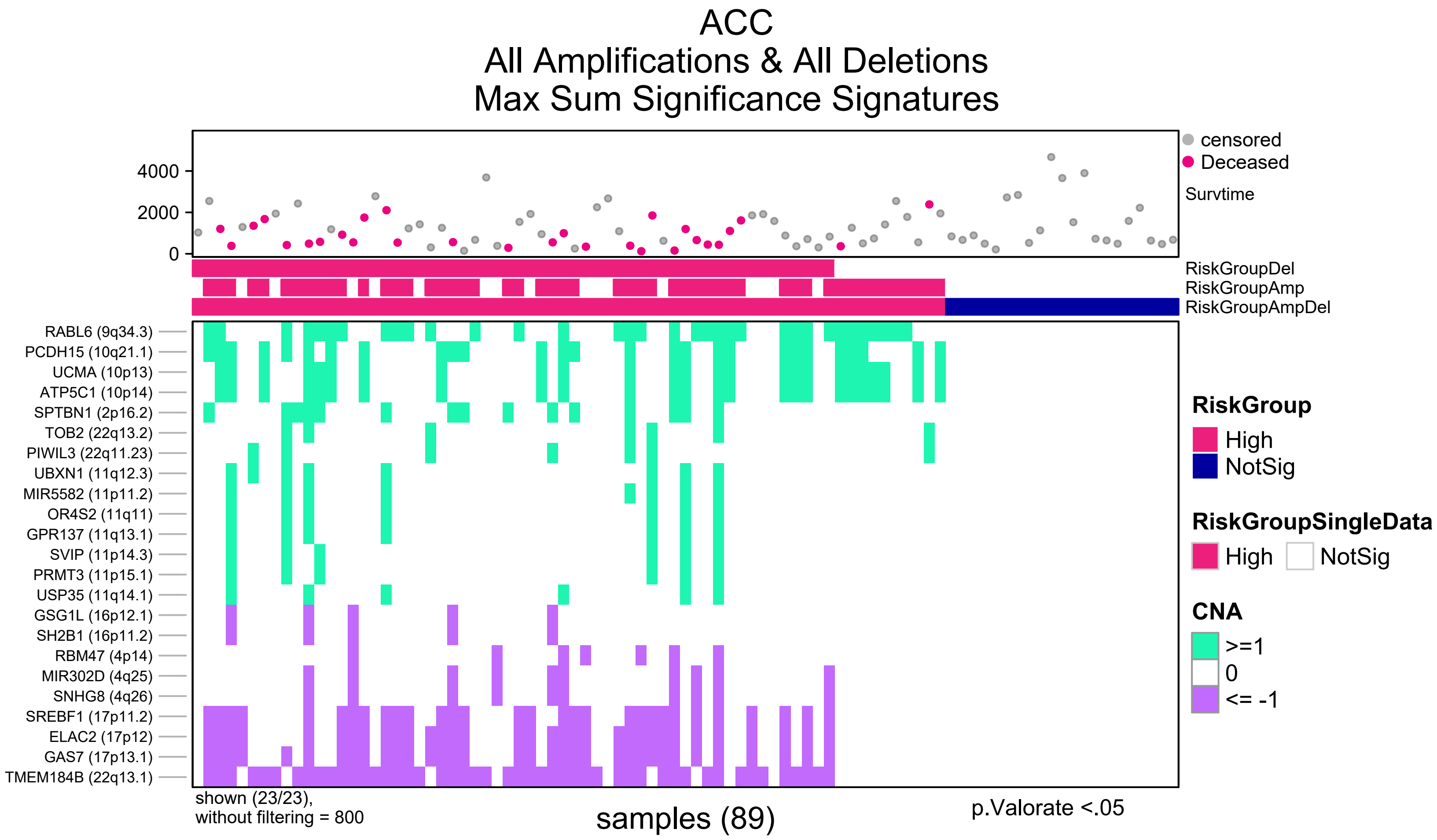

ACC
All Amplifications & All Deletions
Max Sum Significance Signatures
4000
2000
0
censored
Deceased
Survtime
RiskGroupDel
RiskGroupAmp
RiskGroupAmpDel
RABL6 (9q34.3)
PCDH15 (10q21.1)
UCMA (10p13)
ATP5C1 (10p14)
SPTBN1 (2p16.2)
TOB2 (22q13.2)
PIWIL3 (22q11.23)
UBXN1 (11q12.3)
MIR5582 (11p11.2)
OR4S2 (11q11)
GPR137 (11q13.1)
SVIP (11p14.3)
PRMT3 (11p15.1)
USP35 (11q14.1)
GSG1L (16p12.1)
SH2B1 (16p11.2)
RBM47 (4p14)
MIR302D (4q25)
SNHG8 (4q26)
SREBF1 (17p11.2)
ELAC2 (17p12)
GAS7 (17p13.1)
TMEM184B (22q13.1)
RiskGroup
High
NotSig
RiskGroupSingleData
High
NotSig
CNA
>=1
0
<= -1
shown (23/23),
without filtering = 800
samples (89)
p.Valorate <.05

ACC
All Amplifications & All Deletions
Max Sum Significance Signatures

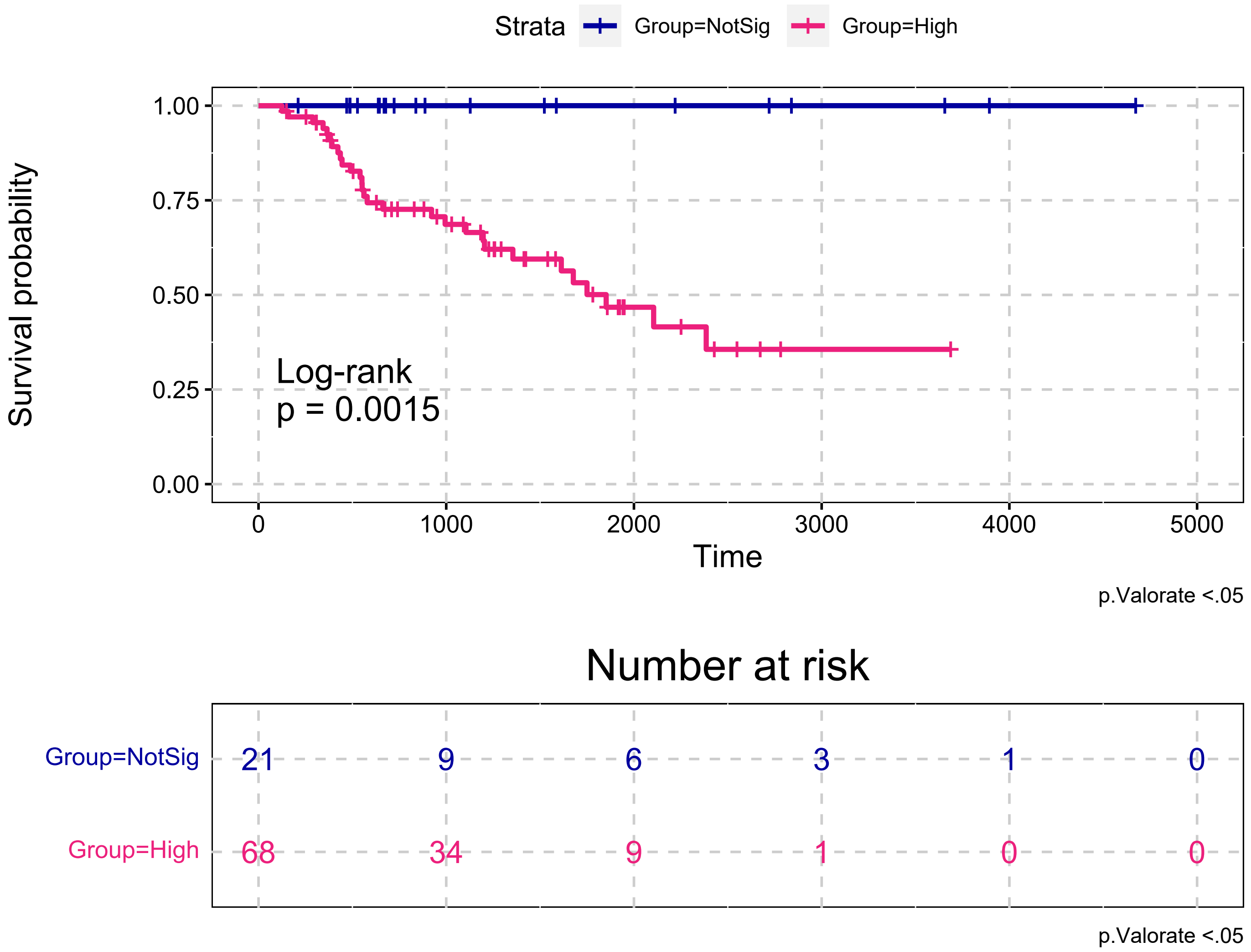

explanatory	beta	HR	L95	U95	p
High	19.51	296148920.35	0.00	Inf	1.00

n= 89, number of events =29
Score(logrank) test = 0.001

ACC
All Amplifications & All Deletions combining signatures

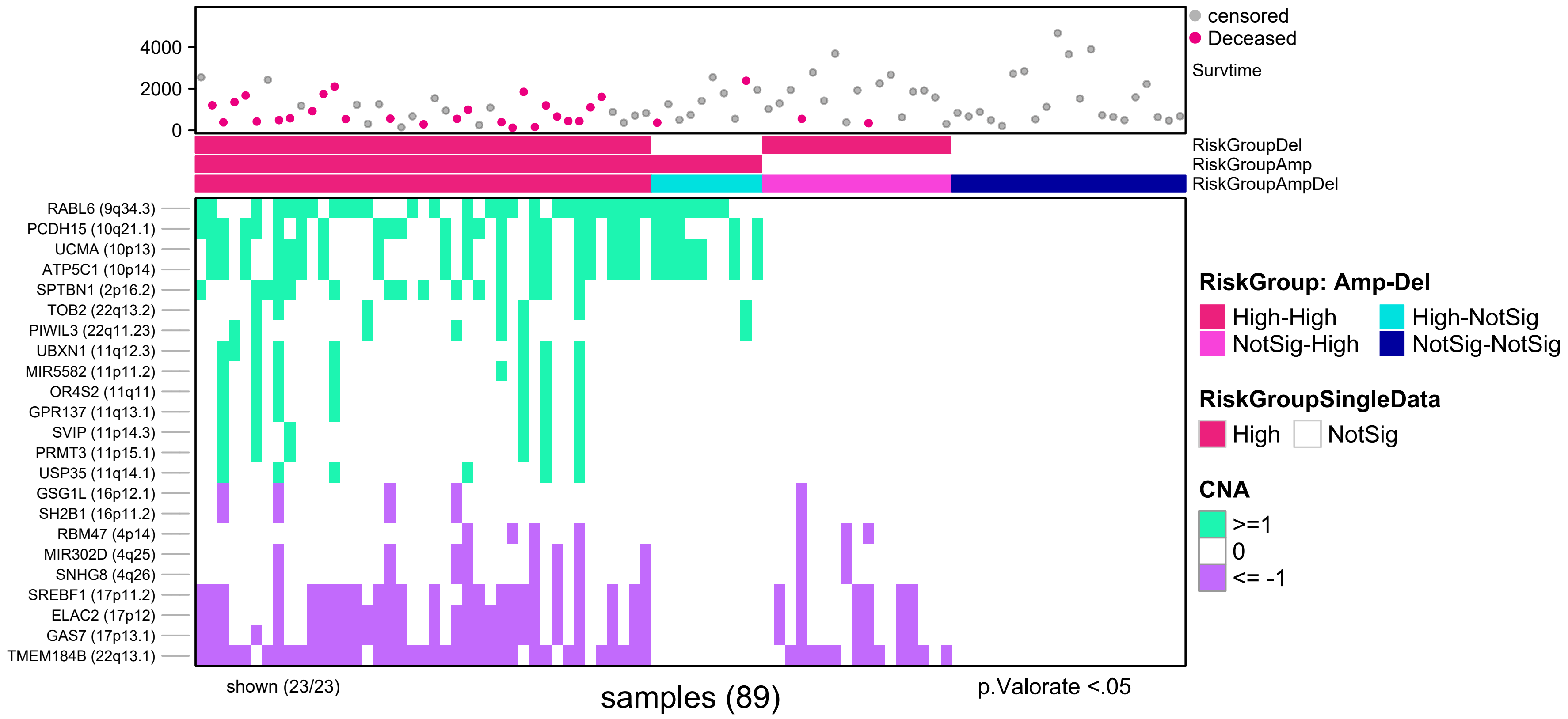

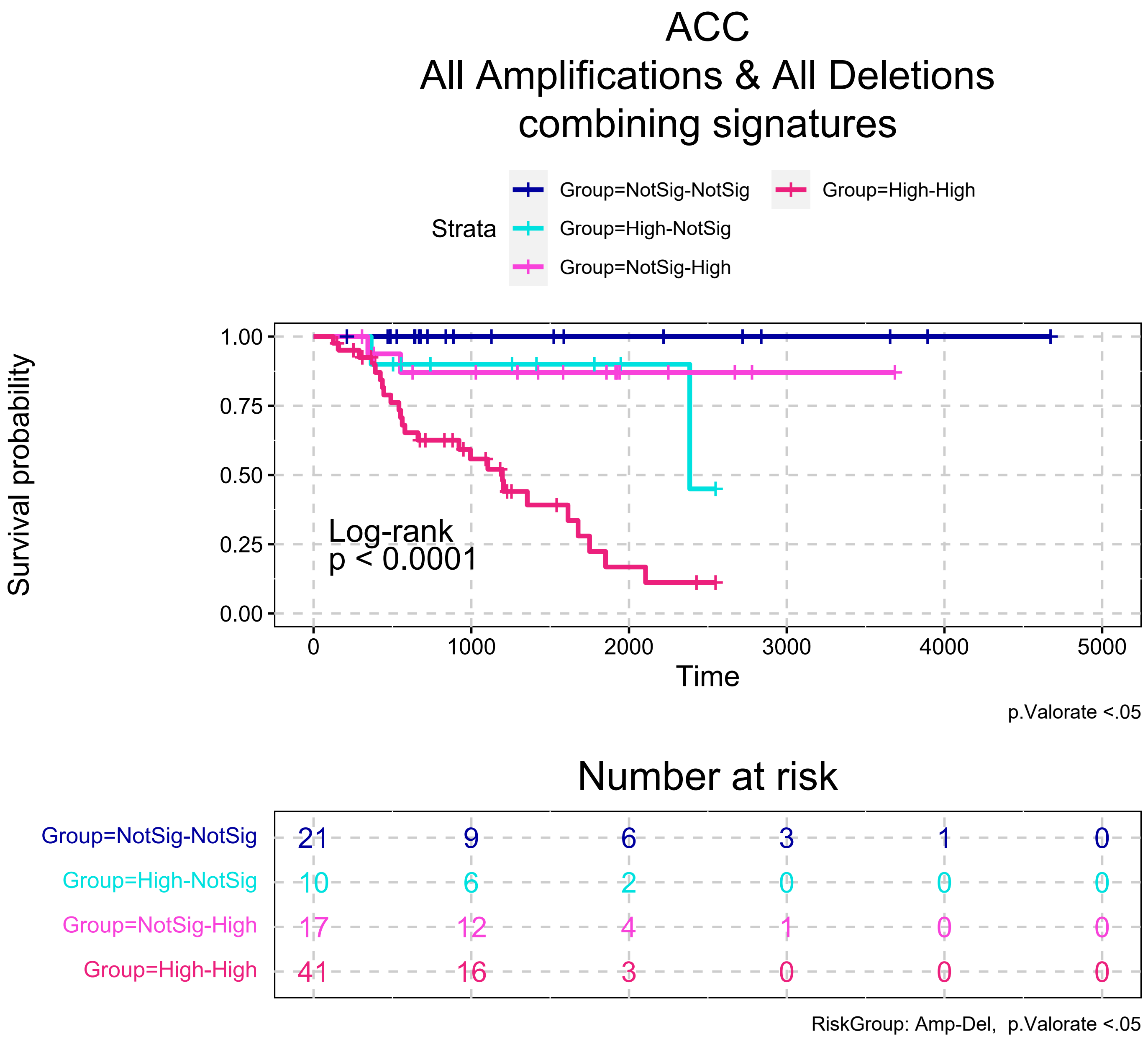

explanatory	beta	HR	L95	U95	p
High-NotSig	18.91	162837376.89	0.00	Inf	1.00
NotSig-High	18.30	88961800.34	0.00	Inf	1.00
High-High	20.50	803472774.56	0.00	Inf	1.00

n= 89, number of events =29
Score(logrank) test = p <.0001

ACC
Deep Amplifications
Single Data Signature

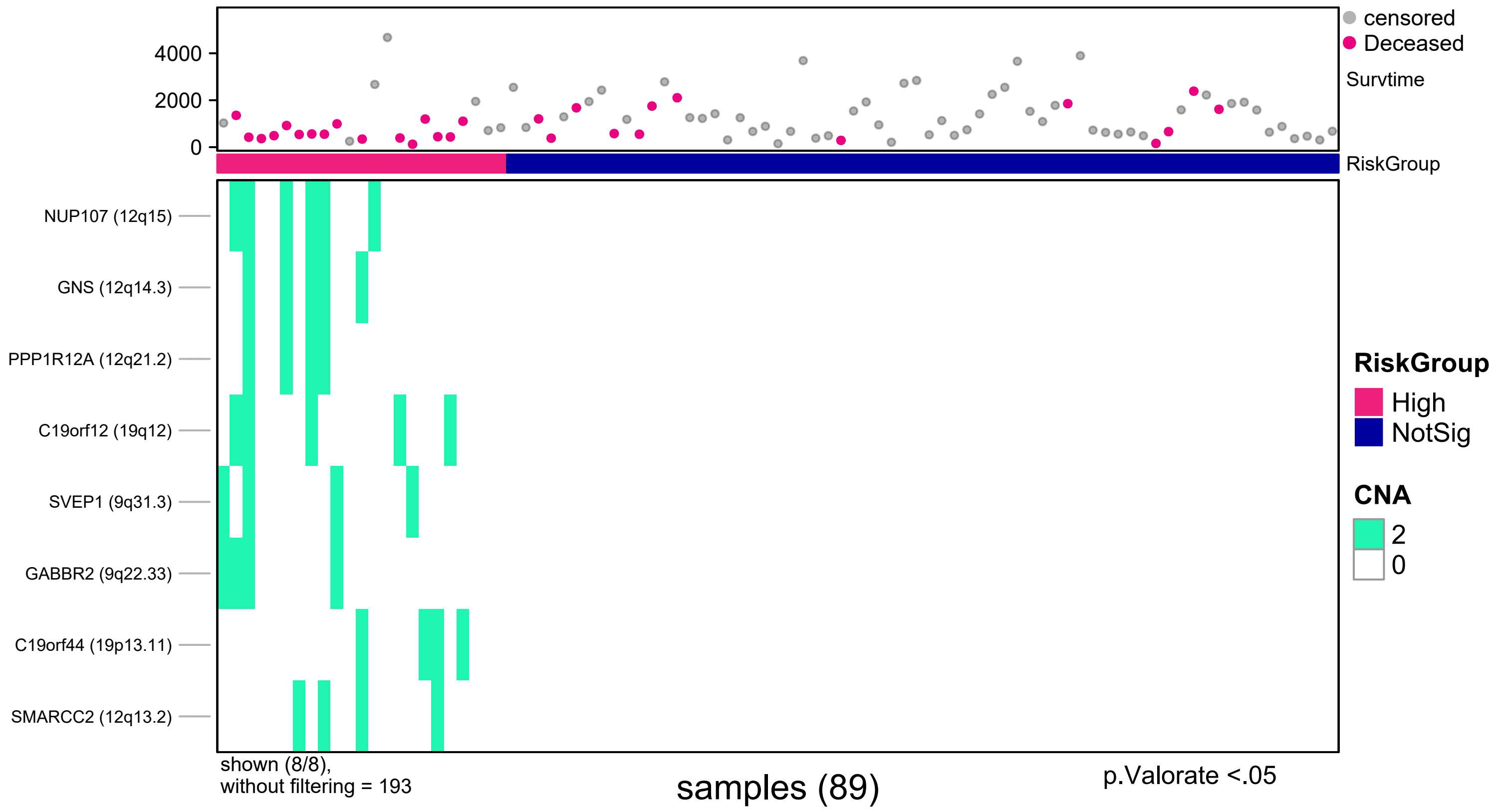

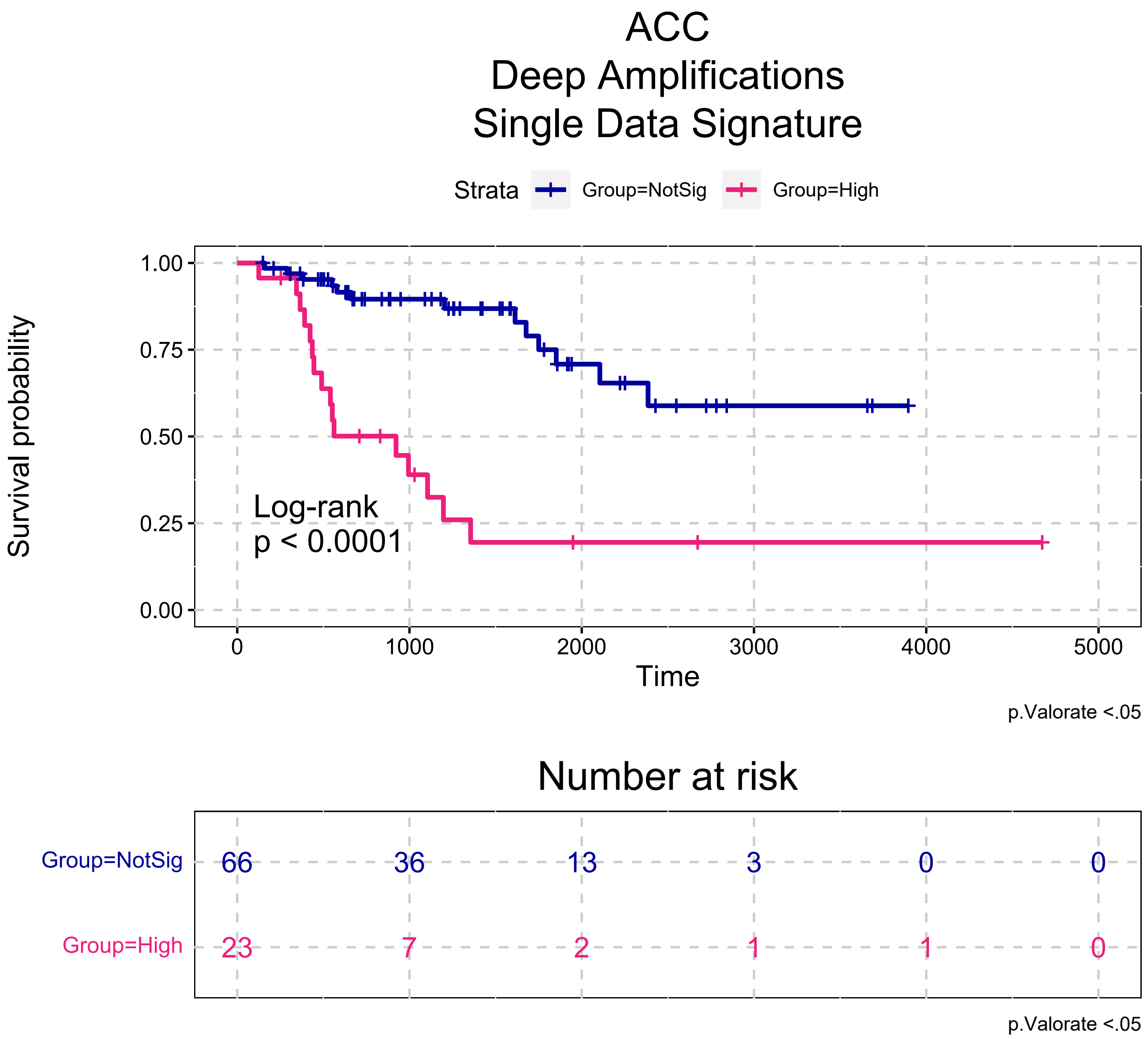

explanatory	beta	HR	L95	U95	p
High	1.68	5.39	2.57	11.32	0.00

n= 89, number of events =29
Score(logrank) test = p <.0001

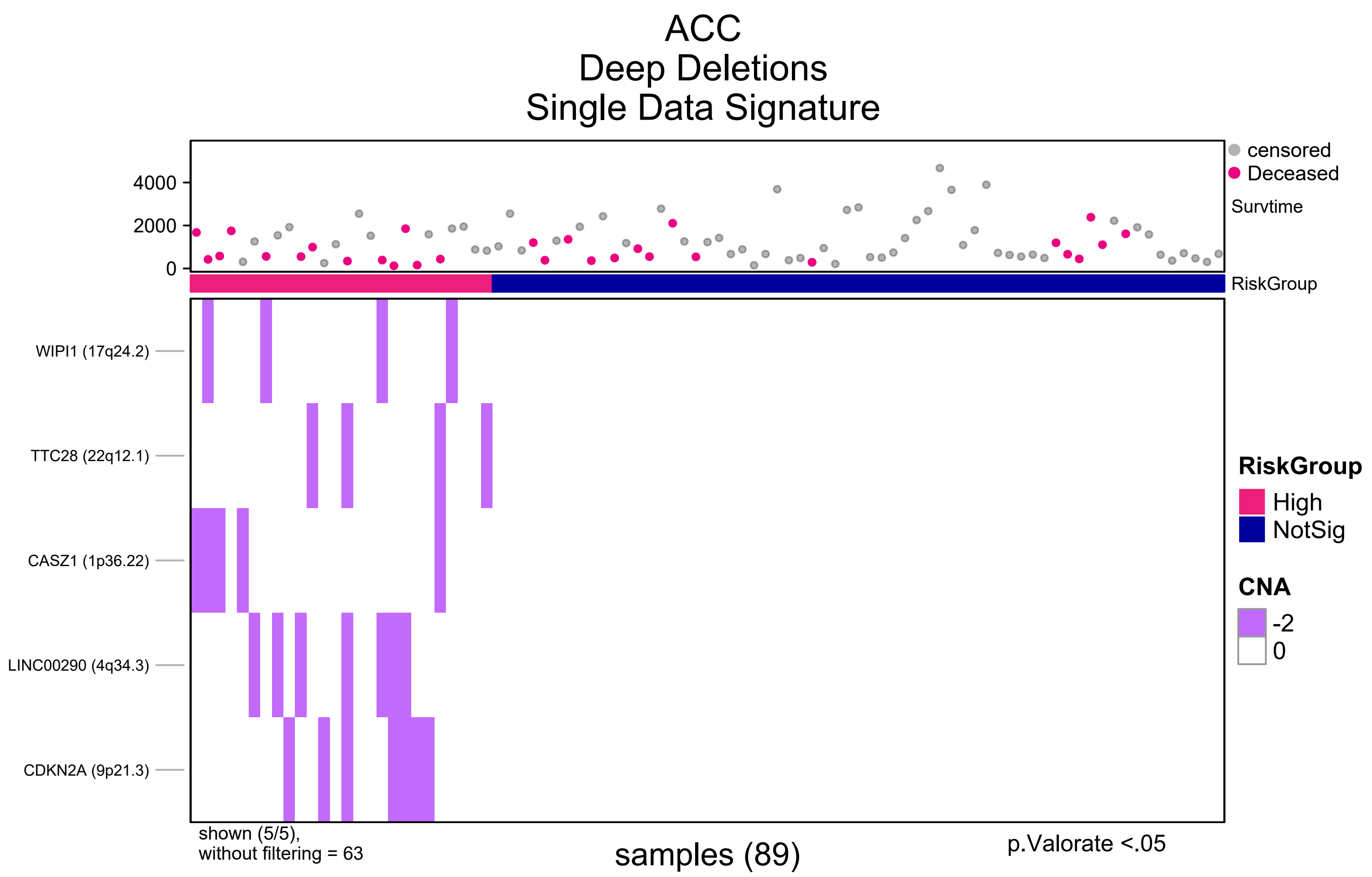

ACC
Deep Deletions
Single Data Signature
4000
2000
0
censored
Deceased
Survtime
RiskGroup
WIPI1 (17q24.2)
TTC28 (22q12.1)
CASZ1 (1p36.22)
LINC00290 (4q34.3)
CDKN2A (9p21.3)
RiskGroup
High
NotSig
CNA
-2
0
shown (5/5),
without filtering = 63
samples (89)
p.Valorate <.05

ACC
Deep Deletions
Single Data Signature

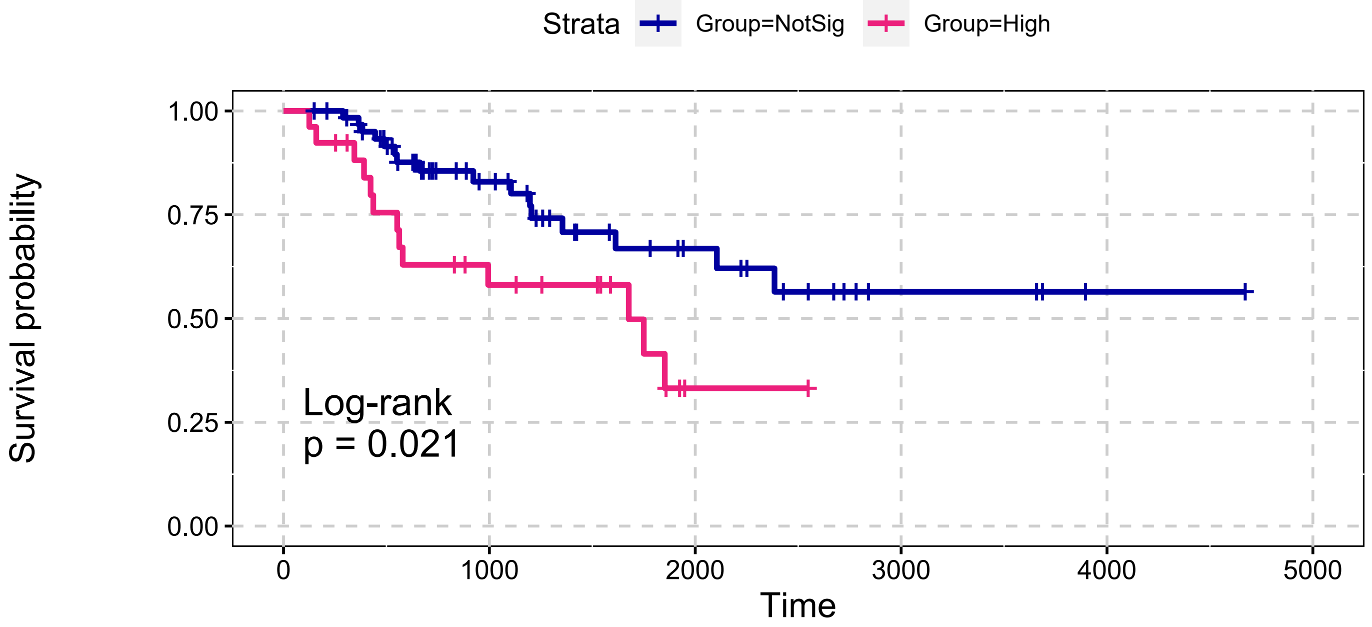

p.Valorate <.05

Number at risk

	0	1000	2000	3000	4000	5000
Group=NotSig	63	31	14	4	1	0
Group=High	26	12	1	0	0	0

p.Valorate <.05

**explanatory**	**beta**	**HR**	**L95**	**U95**	**p**
High	0.85	2.33	1.11	4.90	0.03

n= 89, number of events =29
Score(logrank) test = 0.021

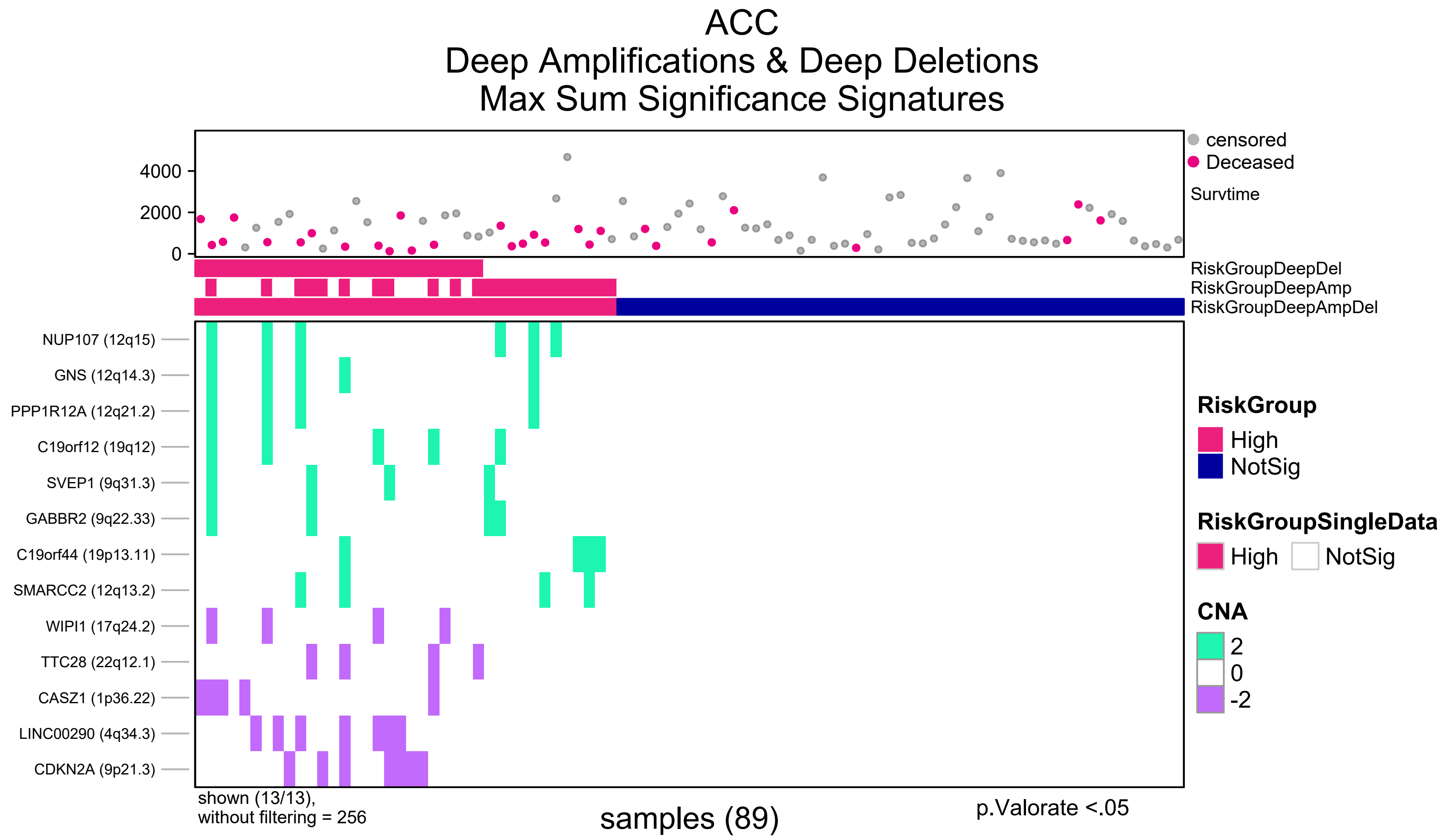
ACC
Deep Amplifications & Deep Deletions
Max Sum Significance Signatures
4000
2000
0
censored
Deceased
Survtime
RiskGroupDeepDel
RiskGroupDeepAmp
RiskGroupDeepAmpDel
NUP107 (12q15)
GNS (12q14.3)
PPP1R12A (12q21.2)
C19orf12 (19q12)
SVEP1 (9q31.3)
GABBR2 (9q22.33)
C19orf44 (19p13.11)
SMARCC2 (12q13.2)
WIPI1 (17q24.2)
TTC28 (22q12.1)
CASZ1 (1p36.22)
LINC00290 (4q34.3)
CDKN2A (9p21.3)
RiskGroup
High
NotSig
RiskGroupSingleData
High
NotSig
CNA
2
0
-2
shown (13/13),
without filtering = 256
samples (89)
p.Valorate <.05

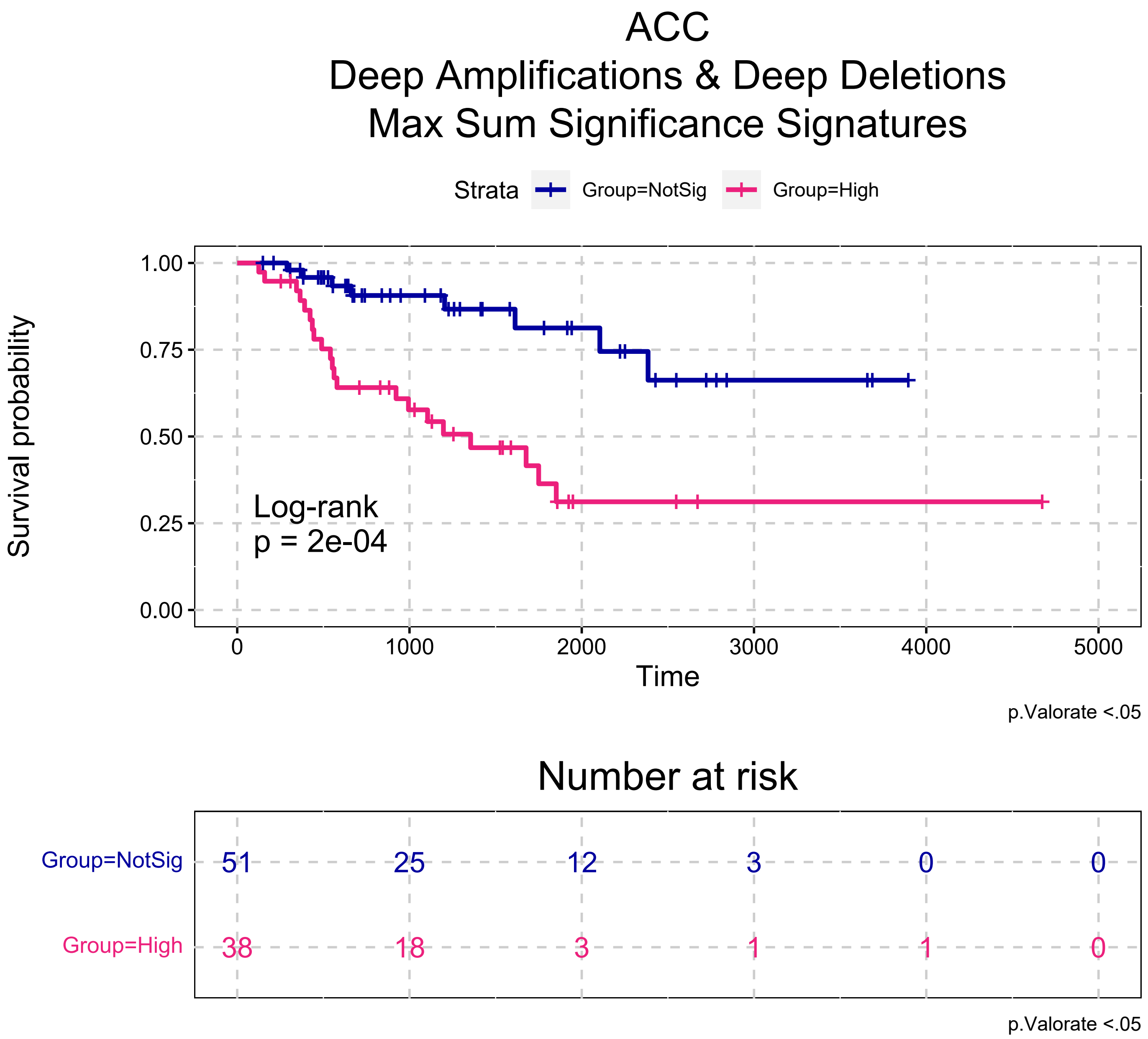

explanatory	beta	HR	L95	U95	p
High	1.43	4.19	1.85	9.52	0.00

n= 89, number of events =29
Score(logrank) test = 0

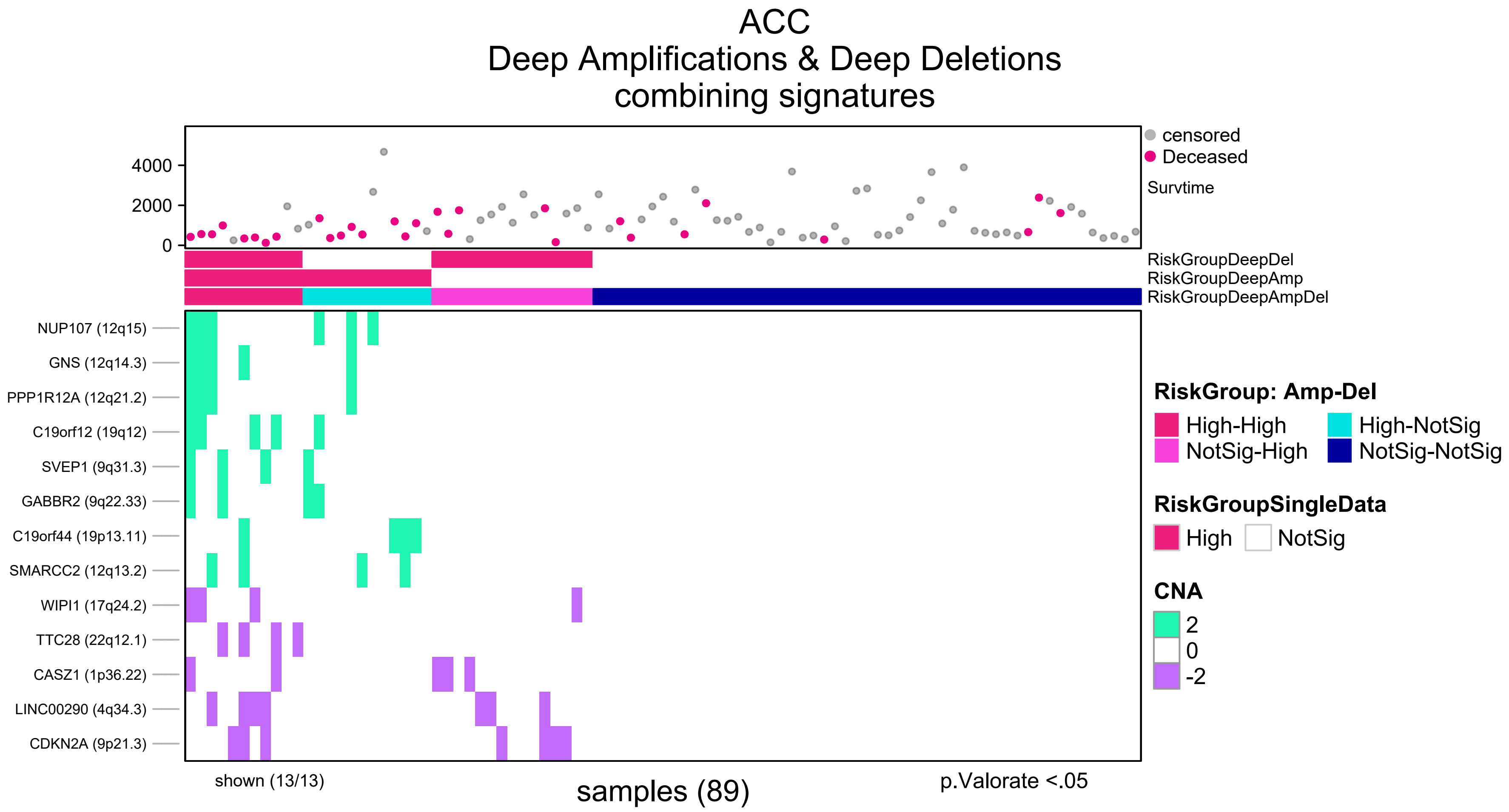

ACC
Deep Amplifications & Deep Deletions
combining signatures
4000
2000
0
censored
Deceased
Survtime
RiskGroupDeepDel
RiskGroupDeepAmp
RiskGroupDeepAmpDel
NUP107 (12q15)
GNS (12q14.3)
PPP1R12A (12q21.2)
C19orf12 (19q12)
SVEP1 (9q31.3)
GABBR2 (9q22.33)
C19orf44 (19p13.11)
SMARCC2 (12q13.2)
WIPI1 (17q24.2)
TTC28 (22q12.1)
CASZ1 (1p36.22)
LINC00290 (4q34.3)
CDKN2A (9p21.3)
RiskGroup: Amp-Del
High-High
High-NotSig
NotSig-High
NotSig-NotSig
RiskGroupSingleData
High
NotSig
CNA
2
0
-2
shown (13/13)
samples (89)
p.Valorate <.05

ACC
Deep Amplifications & Deep Deletions combining signatures

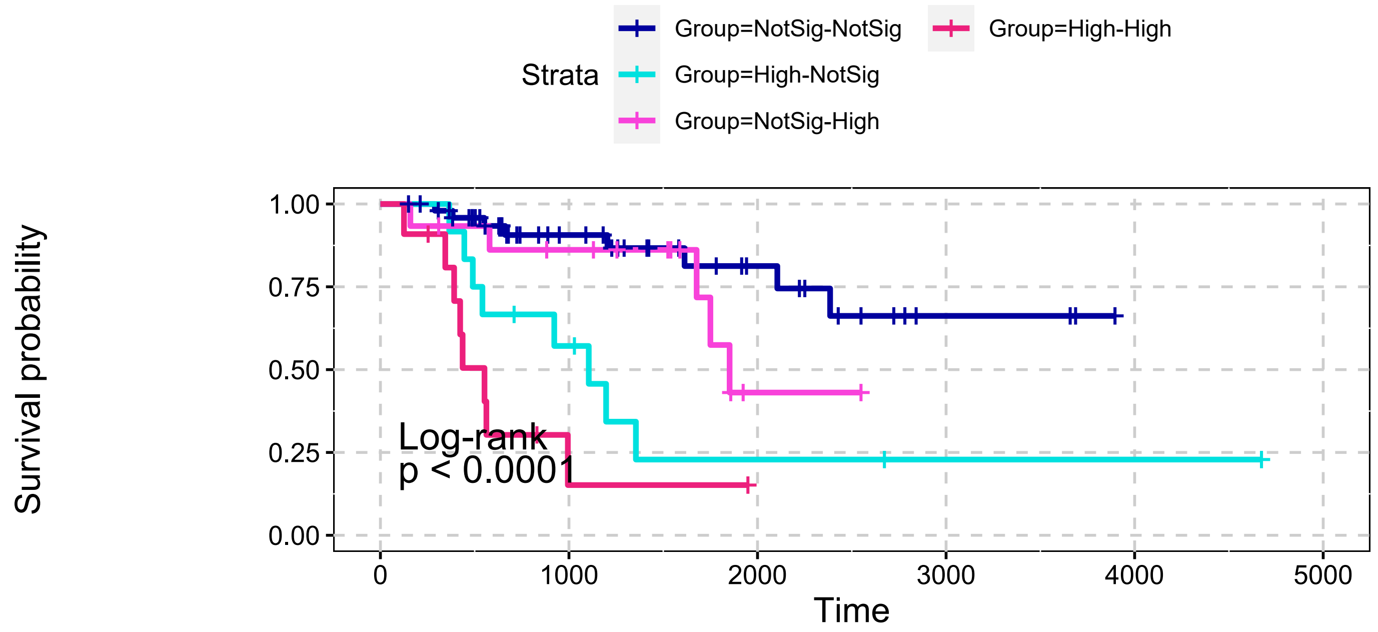

p.Valorate <.05

Number at risk

	0	1000	2000	3000	4000	5000
Group=NotSig-NotSig	51	25	12	3	0	0
Group=High-NotSig	12	6	2	1	1	0
Group=NotSig-High	15	11	1	0	0	0
Group=High-High	11	1	0	0	0	0

RiskGroup: Amp-Del, p.Valorate <.05

**explanatory**	**beta**	**HR**	**L95**	**U95**	**p**
High-NotSig	1.59	4.90	1.83	13.10	0.00
NotSig-High	0.66	1.94	0.63	5.98	0.25
High-High	2.36	10.56	3.84	29.04	0.00

n= 89, number of events =29
Score(logrank) test = p <.0001
