# Supplementary material for: Systematic Modeling of Risk-Associated Copy Number Alterations in Cancer
Source: Int J Mol Sci. 2024 Sep 27;25(19):10455. doi: 10.3390/ijms251910455 (PMC11477427; doi:10.3390/ijms251910455)
Supplement: Supplementary file 1 [file ijms-25-10455-s001.zip › BLCASignatureV12-sinSombreado.pdf]

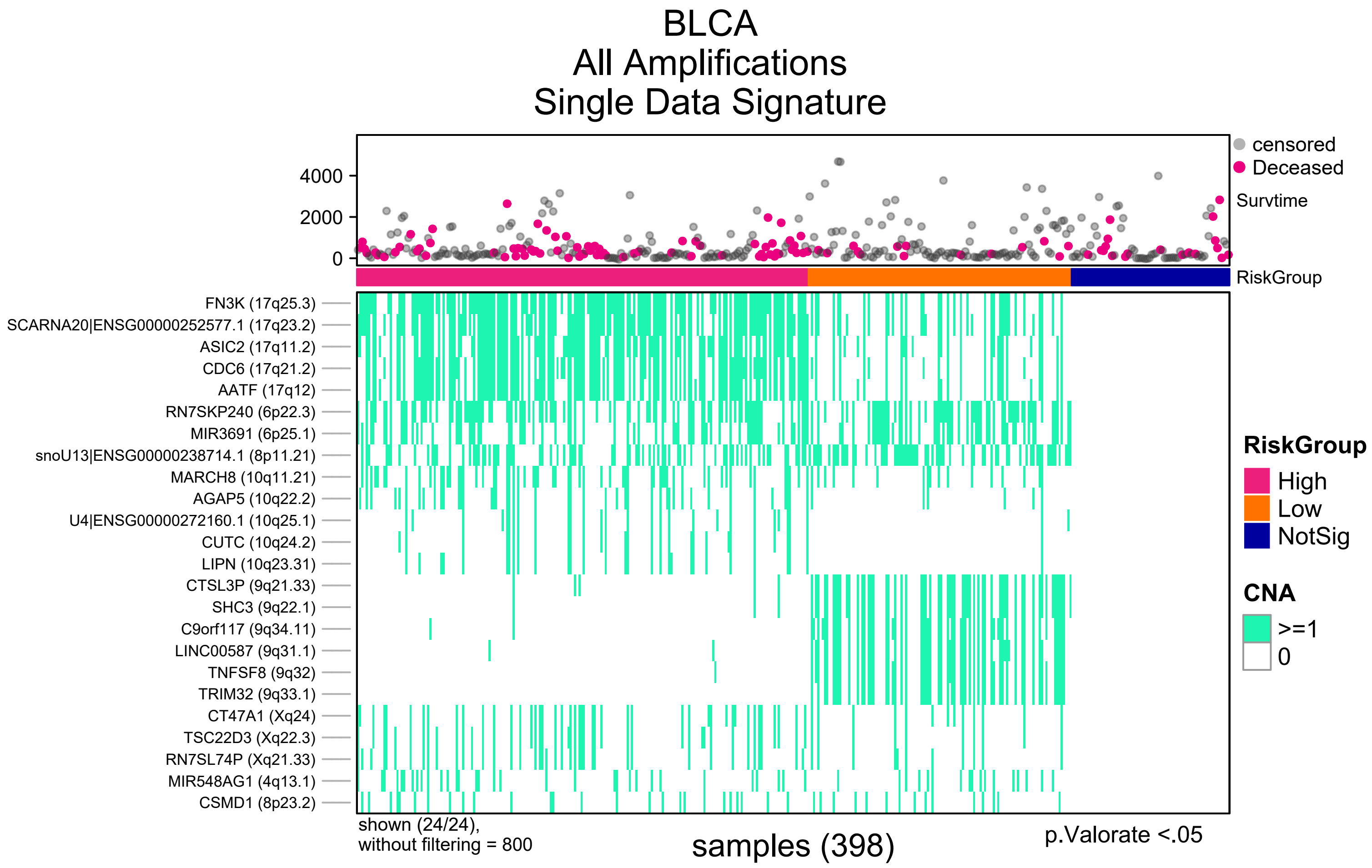
BLCA
All Amplifications
Single Data Signature
4000
2000
0
censored
Deceased
Survtime
RiskGroup
FN3K (17q25.3)
SCARNA20|ENSG00000252577.1 (17q23.2)
ASIC2 (17q11.2)
CDC6 (17q21.2)
AATF (17q12)
RN7SKP240 (6p22.3)
MIR3691 (6p25.1)
snoU13|ENSG00000238714.1 (8p11.21)
MARCH8 (10q11.21)
AGAP5 (10q22.2)
U4|ENSG00000272160.1 (10q25.1)
CUTC (10q24.2)
LIPN (10q23.31)
CTSL3P (9q21.33)
SHC3 (9q22.1)
C9orf117 (9q34.11)
LINC00587 (9q31.1)
TNFSF8 (9q32)
TRIM32 (9q33.1)
CT47A1 (Xq24)
TSC22D3 (Xq22.3)
RN7SL74P (Xq21.33)
MIR548AG1 (4q13.1)
CSMD1 (8p23.2)
RiskGroup
High
Low
NotSig
CNA
>=1
0
shown (24/24),
without filtering = 800
samples (398)
p.Valorate <.05

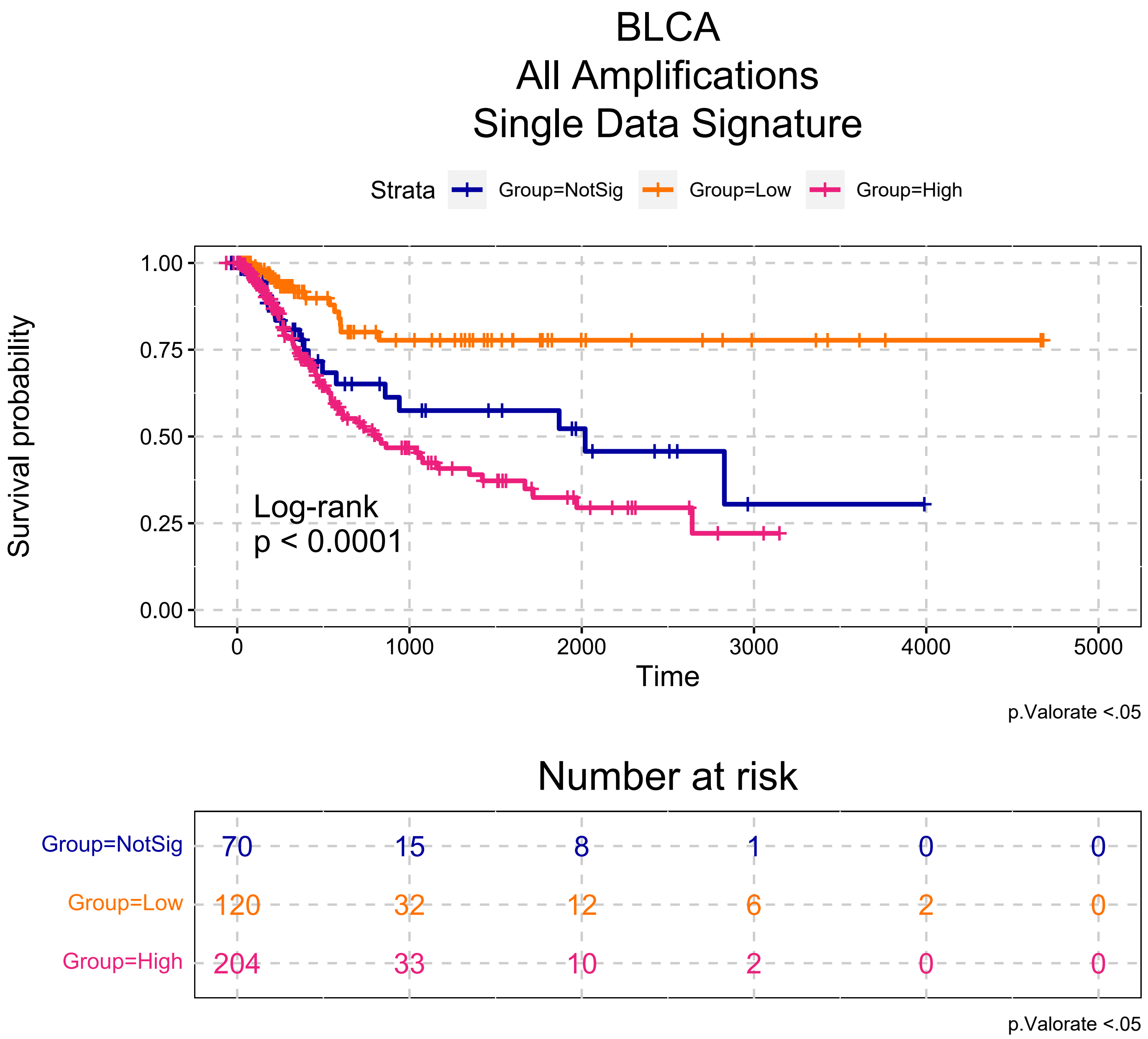

explanatory	beta	HR	L95	U95	p
Low	-1.01	0.36	0.18	0.73	0.00
High	0.34	1.40	0.84	2.32	0.19

n= 398, number of events =107
Score(logrank) test = p <.0001

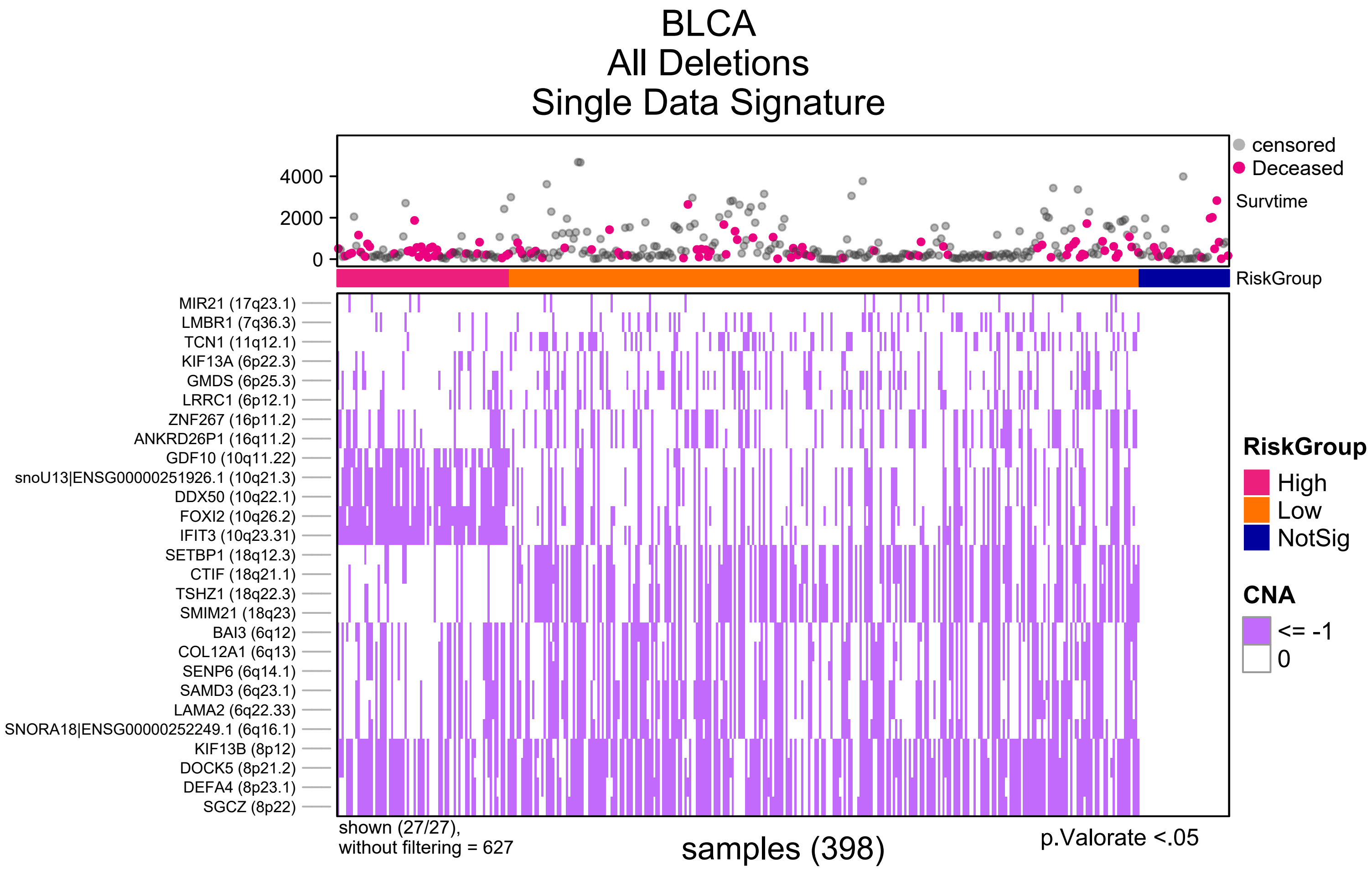
BLCA
All Deletions
Single Data Signature
4000
2000
0
censored
Deceased
Survtime
RiskGroup
MIR21 (17q23.1)
LMBR1 (7q36.3)
TCN1 (11q12.1)
KIF13A (6p22.3)
GMDS (6p25.3)
LRRC1 (6p12.1)
ZNF267 (16p11.2)
ANKRD26P1 (16q11.2)
GDF10 (10q11.22)
snoU13|ENSG00000251926.1 (10q21.3)
DDX50 (10q22.1)
FOXI2 (10q26.2)
IFIT3 (10q23.31)
SETBP1 (18q12.3)
CTIF (18q21.1)
TSHZ1 (18q22.3)
SMIM21 (18q23)
BAI3 (6q12)
COL12A1 (6q13)
SENP6 (6q14.1)
SAMD3 (6q23.1)
LAMA2 (6q22.33)
SNORA18|ENSG00000252249.1 (6q16.1)
KIF13B (8p12)
DOCK5 (8p21.2)
DEFA4 (8p23.1)
SGCZ (8p22)
RiskGroup
High
Low
NotSig
CNA
<= -1
0
shown (27/27),
without filtering = 627
samples (398)
p.Valorate <.05

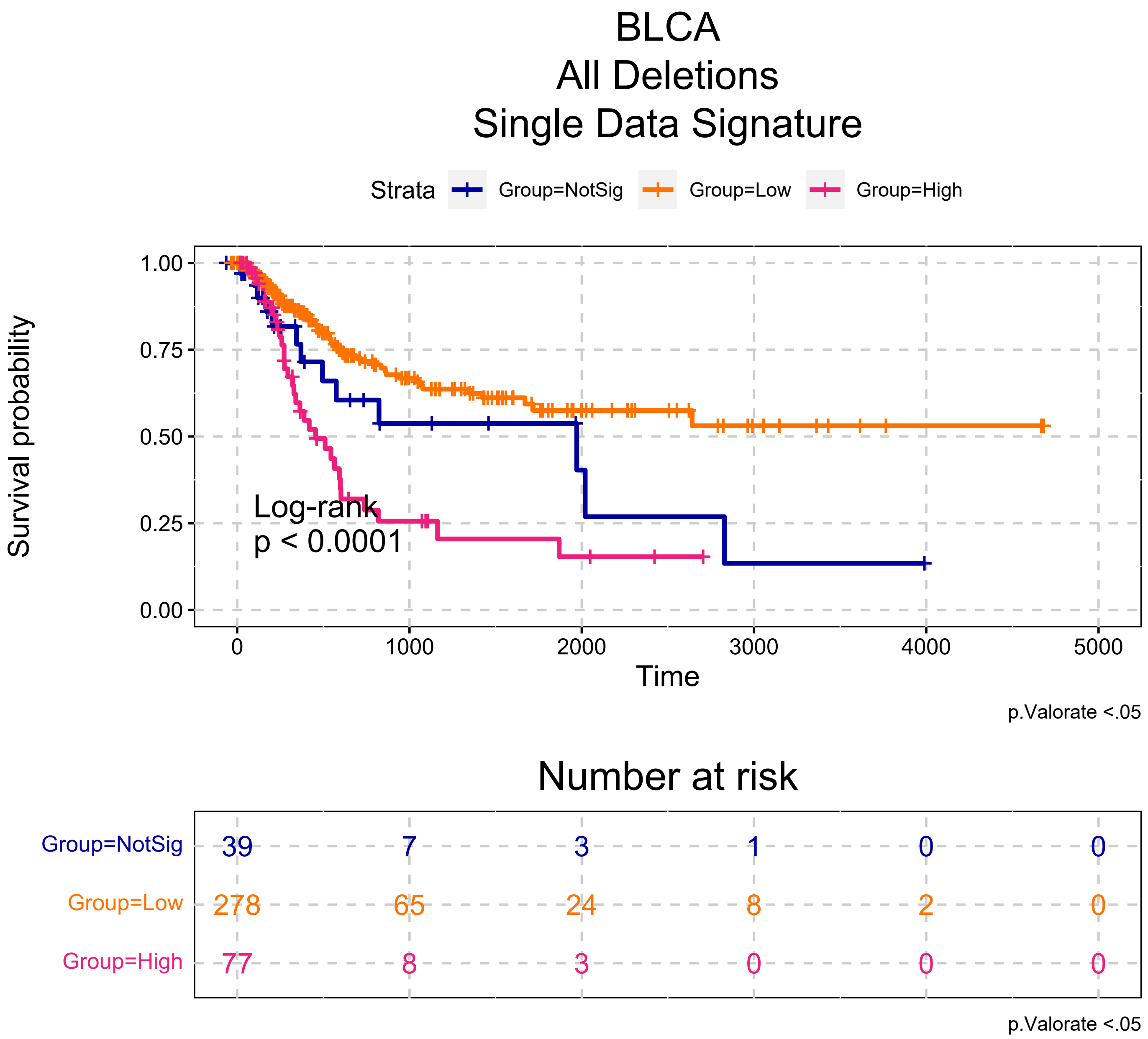

explanatory	beta	HR	L95	U95	p
Low	-0.60	0.55	0.30	1.00	0.05
High	0.51	1.66	0.87	3.17	0.12

n= 398, number of events =107
Score(logrank) test = p <.0001

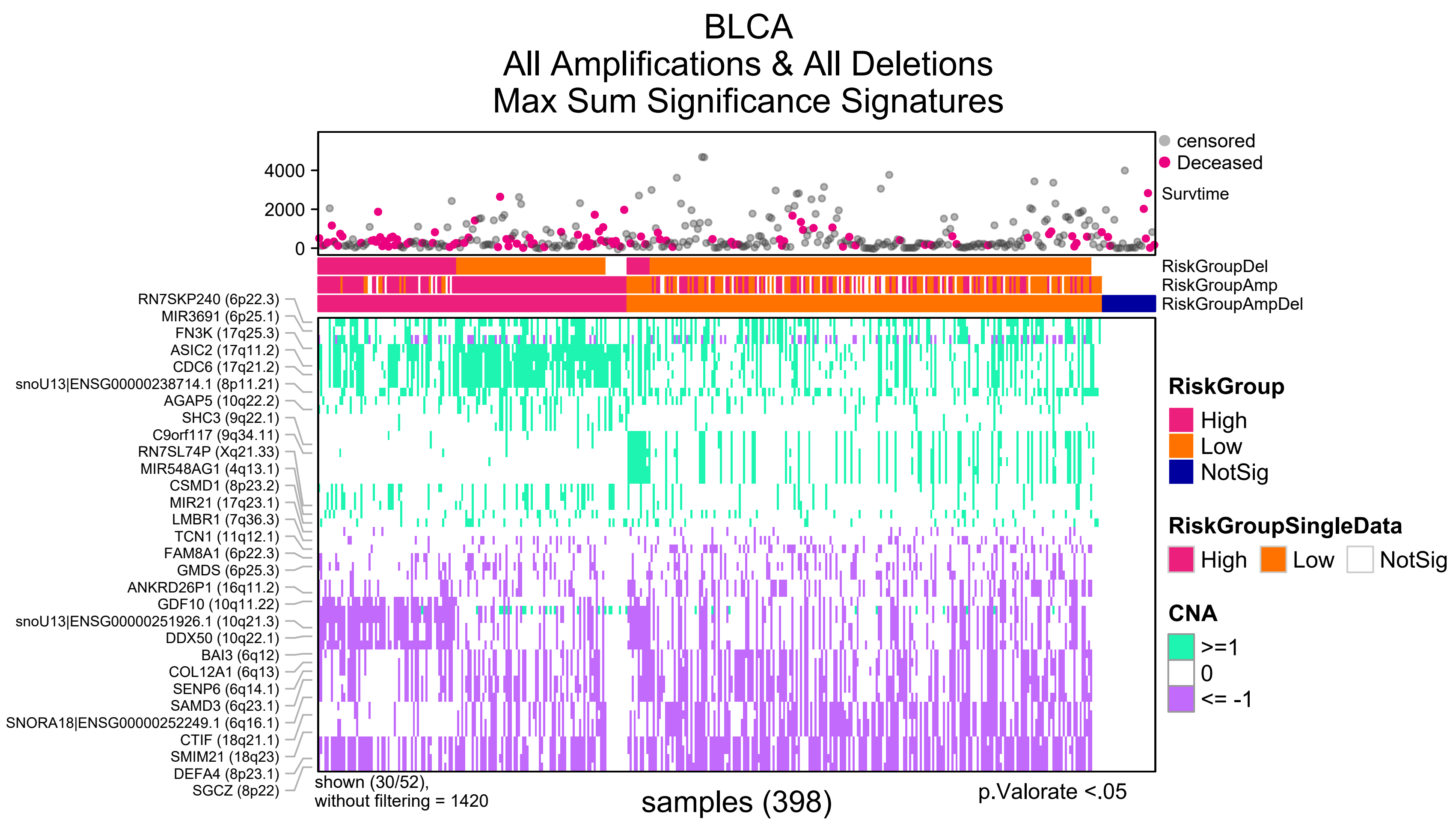
BLCA
All Amplifications & All Deletions
Max Sum Significance Signatures
4000
2000
0
censored
Deceased
Survtime
RiskGroupDel
RiskGroupAmp
RiskGroupAmpDel
RN7SKP240 (6p22.3)
MIR3691 (6p25.1)
FN3K (17q25.3)
ASIC2 (17q11.2)
CDC6 (17q21.2)
snoU13|ENSG00000238714.1 (8p11.21)
AGAP5 (10q22.2)
SHC3 (9q22.1)
C9orf117 (9q34.11)
RN7SL74P (Xq21.33)
MIR548AG1 (4q13.1)
CSMD1 (8p23.2)
MIR21 (17q23.1)
LMBR1 (7q36.3)
TCN1 (11q12.1)
FAM8A1 (6p22.3)
GMDS (6p25.3)
ANKRD26P1 (16q11.2)
GDF10 (10q11.22)
snoU13|ENSG00000251926.1 (10q21.3)
DDX50 (10q22.1)
BAI3 (6q12)
COL12A1 (6q13)
SENP6 (6q14.1)
SAMD3 (6q23.1)
SNORA18|ENSG00000252249.1 (6q16.1)
CTIF (18q21.1)
SMIM21 (18q23)
DEFA4 (8p23.1)
SGCZ (8p22)
RiskGroup
High
Low
NotSig
RiskGroupSingleData
High
Low
NotSig
CNA
>=1
0
<= -1
shown (30/52),
without filtering = 1420
samples (398)
p.Valorate <.05

BLCA
All Amplifications & All Deletions
Max Sum Significance Signatures

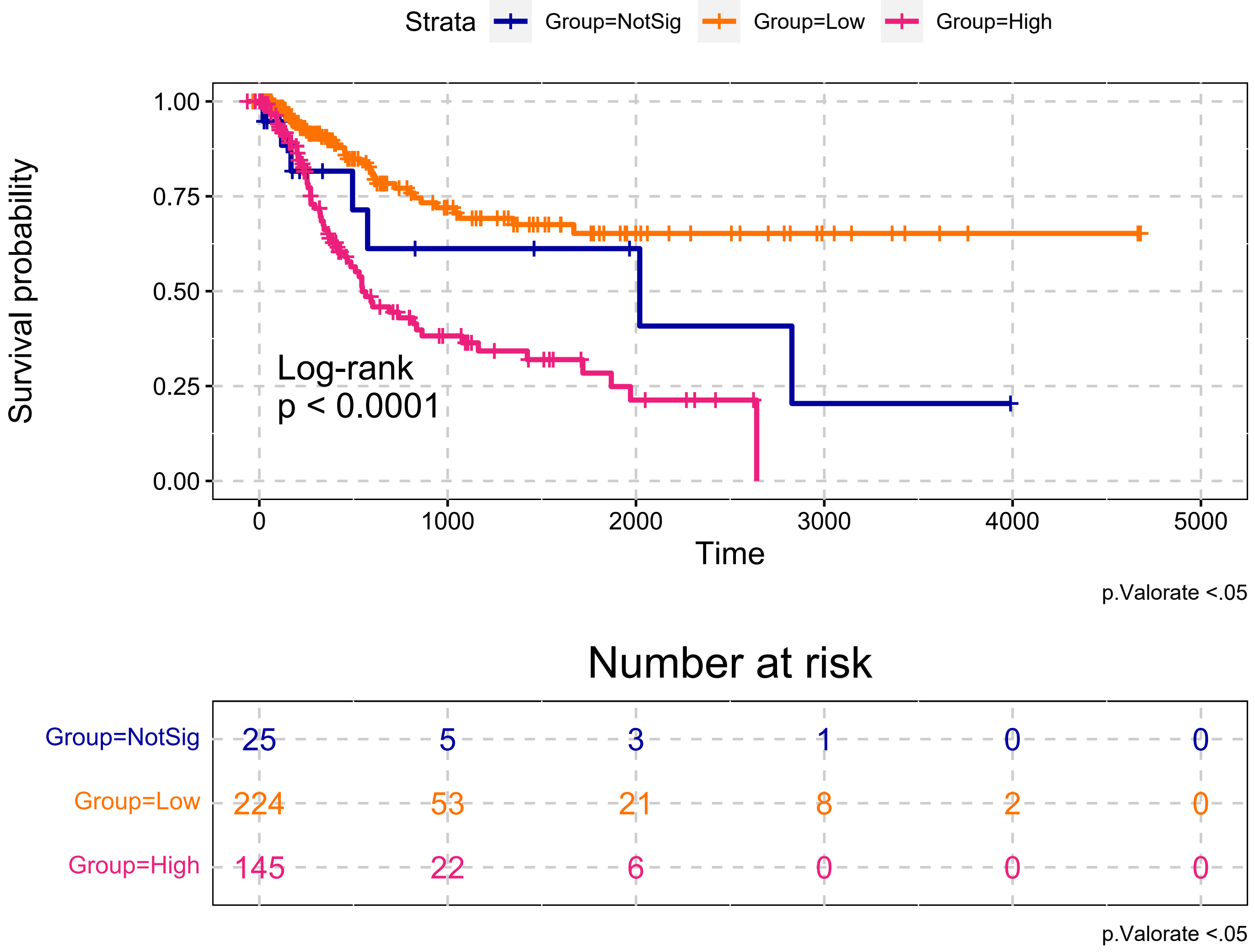

explanatory	beta	HR	L95	U95	p
Low	-0.74	0.48	0.21	1.07	0.07
High	0.47	1.60	0.73	3.53	0.24

n= 398, number of events =107
Score(logrank) test = p <.0001

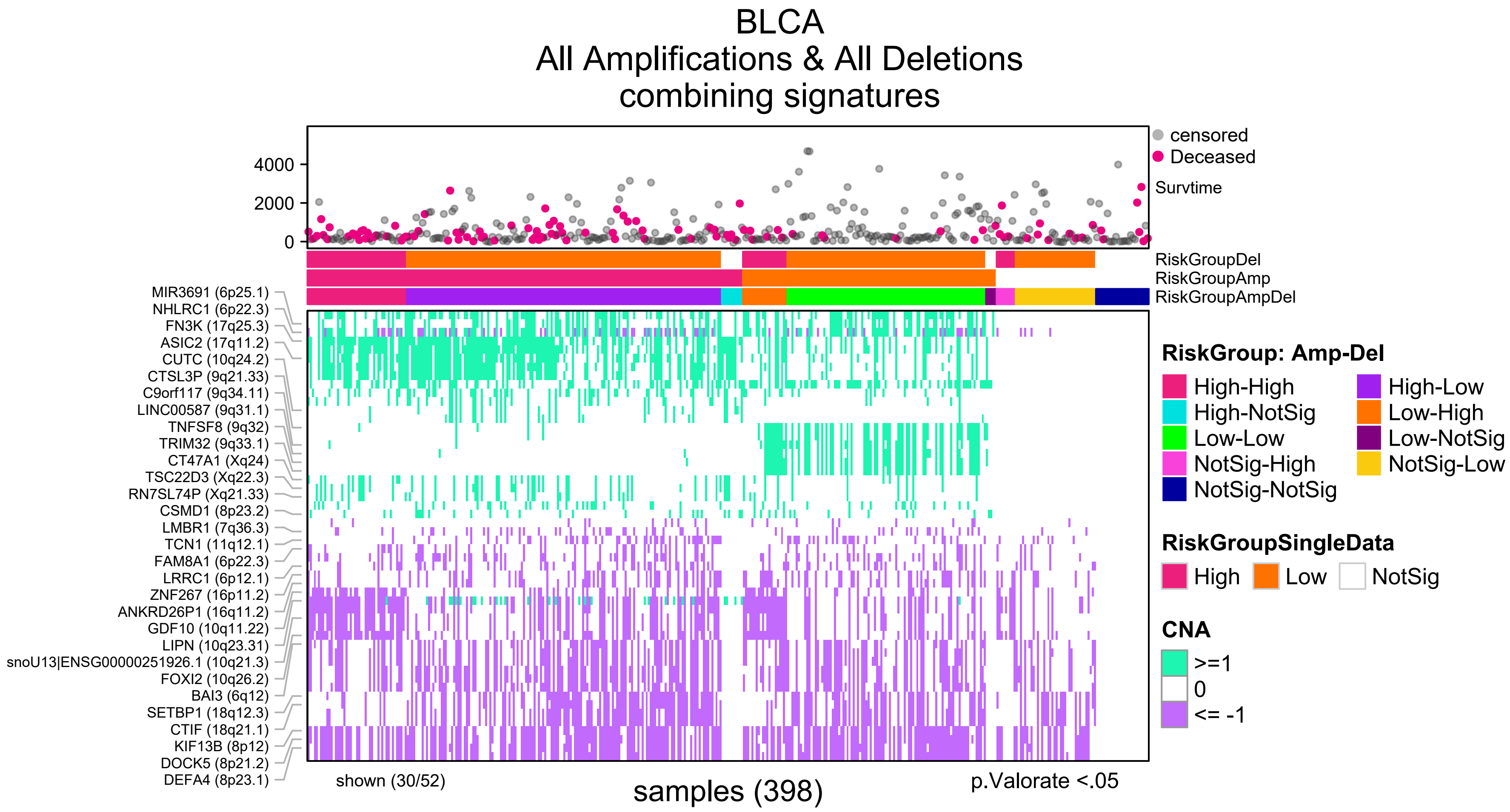
BLCA
All Amplifications & All Deletions
combining signatures
4000
2000
0
censored
Deceased
Survtime
RiskGroupDel
RiskGroupAmp
RiskGroupAmpDel
MIR3691 (6p25.1)
NHLRC1 (6p22.3)
FN3K (17q25.3)
ASIC2 (17q11.2)
CUTC (10q24.2)
CTSL3P (9q21.33)
C9orf117 (9q34.11)
LINC00587 (9q31.1)
TNFSF8 (9q32)
TRIM32 (9q33.1)
CT47A1 (Xq24)
TSC22D3 (Xq22.3)
RN7SL74P (Xq21.33)
CSMD1 (8p23.2)
LMBR1 (7q36.3)
TCN1 (11q12.1)
FAM8A1 (6p22.3)
LRRC1 (6p12.1)
ZNF267 (16p11.2)
ANKRD26P1 (16q11.2)
GDF10 (10q11.22)
LIPN (10q23.31)
snoU13|ENSG00000251926.1 (10q21.3)
FOXI2 (10q26.2)
BAI3 (6q12)
SETBP1 (18q12.3)
CTIF (18q21.1)
KIF13B (8p12)
DOCK5 (8p21.2)
DEFA4 (8p23.1)
RiskGroup: Amp-Del
High-High
High-NotSig
Low-Low
NotSig-High
NotSig-NotSig
High-Low
Low-High
Low-NotSig
NotSig-Low
RiskGroupSingleData
High
Low
NotSig
CNA
>=1
0
<= -1
shown (30/52)
samples (398)
p.Valorate <.05

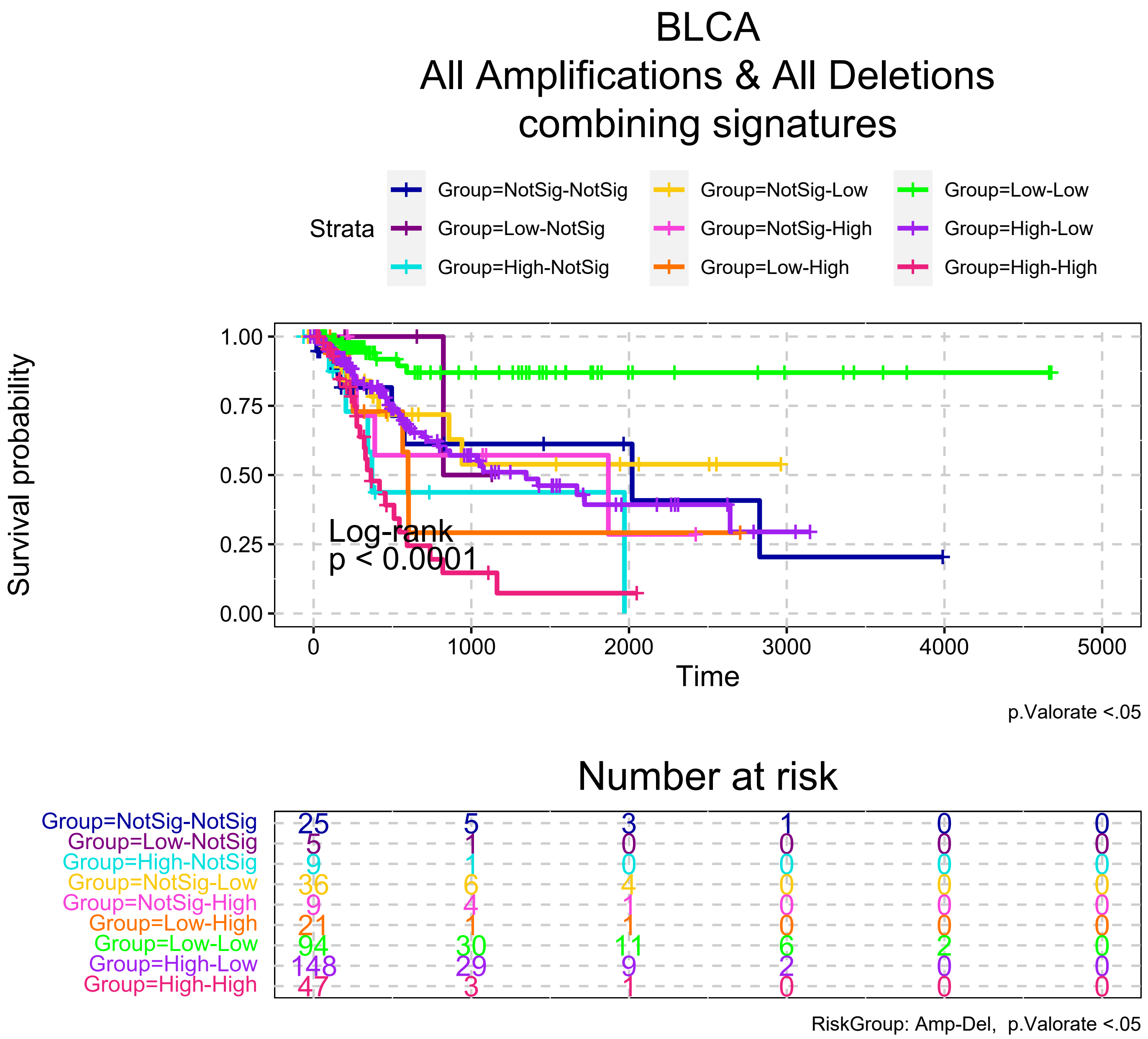

explanatory	beta	HR	L95	U95	p
Low-NotSig	-0.60	0.55	0.07	4.49	0.58
High-NotSig	0.78	2.17	0.68	6.91	0.19
NotSig-Low	-0.25	0.78	0.28	2.15	0.63
NotSig-High	0.06	1.06	0.31	3.66	0.92
Low-High	0.37	1.45	0.48	4.35	0.51
Low-Low	-1.65	0.19	0.07	0.55	0.00
High-Low	-0.03	0.97	0.44	2.18	0.95
High-High	0.97	2.64	1.12	6.23	0.03

n= 398, number of events =107
Score(logrank) test = p <.0001

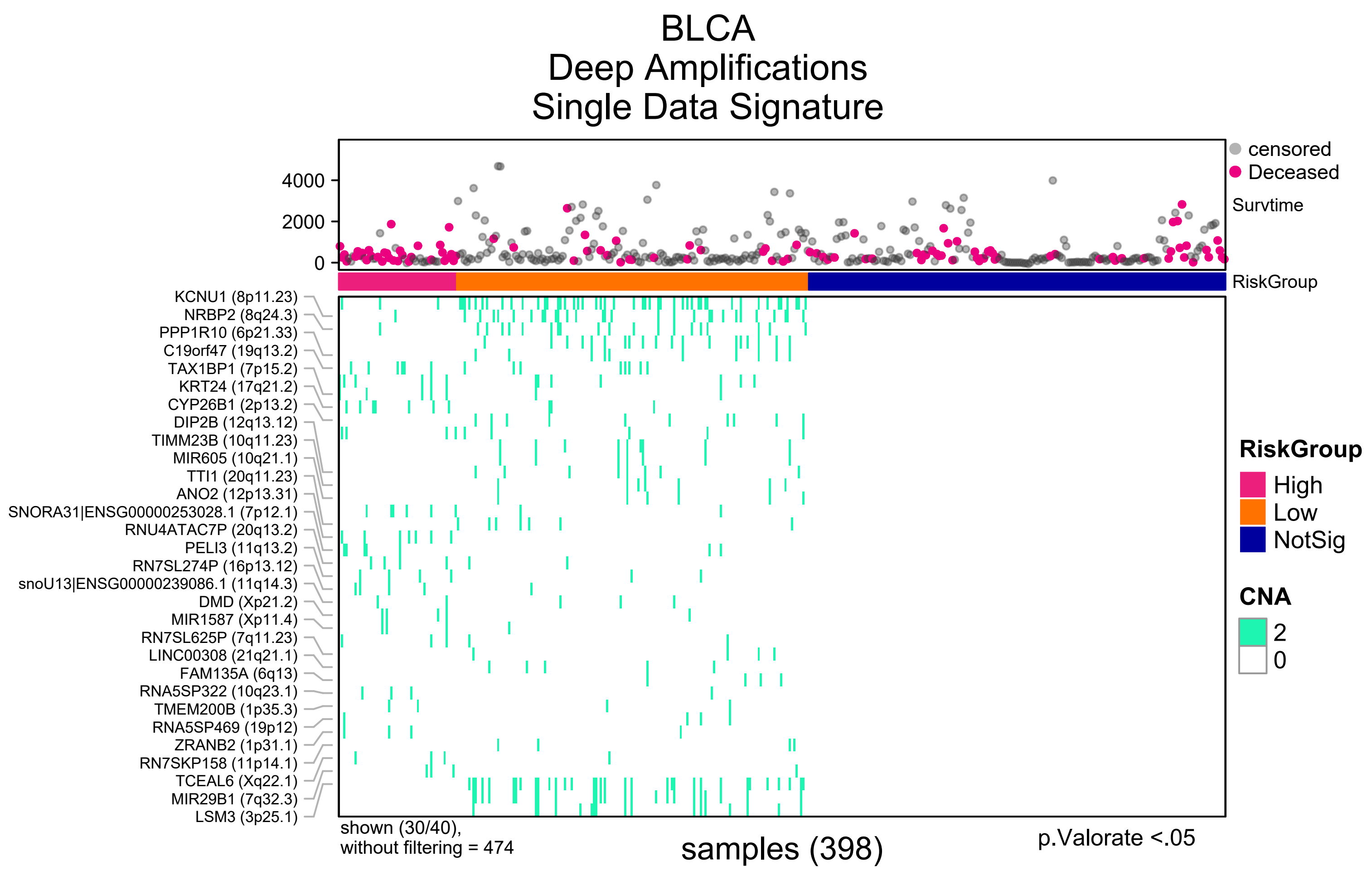
BLCA
Deep Amplifications
Single Data Signature
4000
2000
0
censored
Deceased
Survtime
RiskGroup
KCNU1 (8p11.23)
NRBP2 (8q24.3)
PPP1R10 (6p21.33)
C19orf47 (19q13.2)
TAX1BP1 (7p15.2)
KRT24 (17q21.2)
CYP26B1 (2p13.2)
DIP2B (12q13.12)
TIMM23B (10q11.23)
MIR605 (10q21.1)
TTI1 (20q11.23)
ANO2 (12p13.31)
SNORA31|ENSG00000253028.1 (7p12.1)
RNU4ATAC7P (20q13.2)
PELI3 (11q13.2)
RN7SL274P (16p13.12)
snoU13|ENSG00000239086.1 (11q14.3)
DMD (Xp21.2)
MIR1587 (Xp11.4)
RN7SL625P (7q11.23)
LINC00308 (21q21.1)
FAM135A (6q13)
RNA5SP322 (10q23.1)
TMEM200B (1p35.3)
RNA5SP469 (19p12)
ZRANB2 (1p31.1)
RN7SKP158 (11p14.1)
TCEAL6 (Xq22.1)
MIR29B1 (7q32.3)
LSM3 (3p25.1)
RiskGroup
High
Low
NotSig
CNA
2
0
shown (30/40),
without filtering = 474
samples (398)
p.Valorate <.05

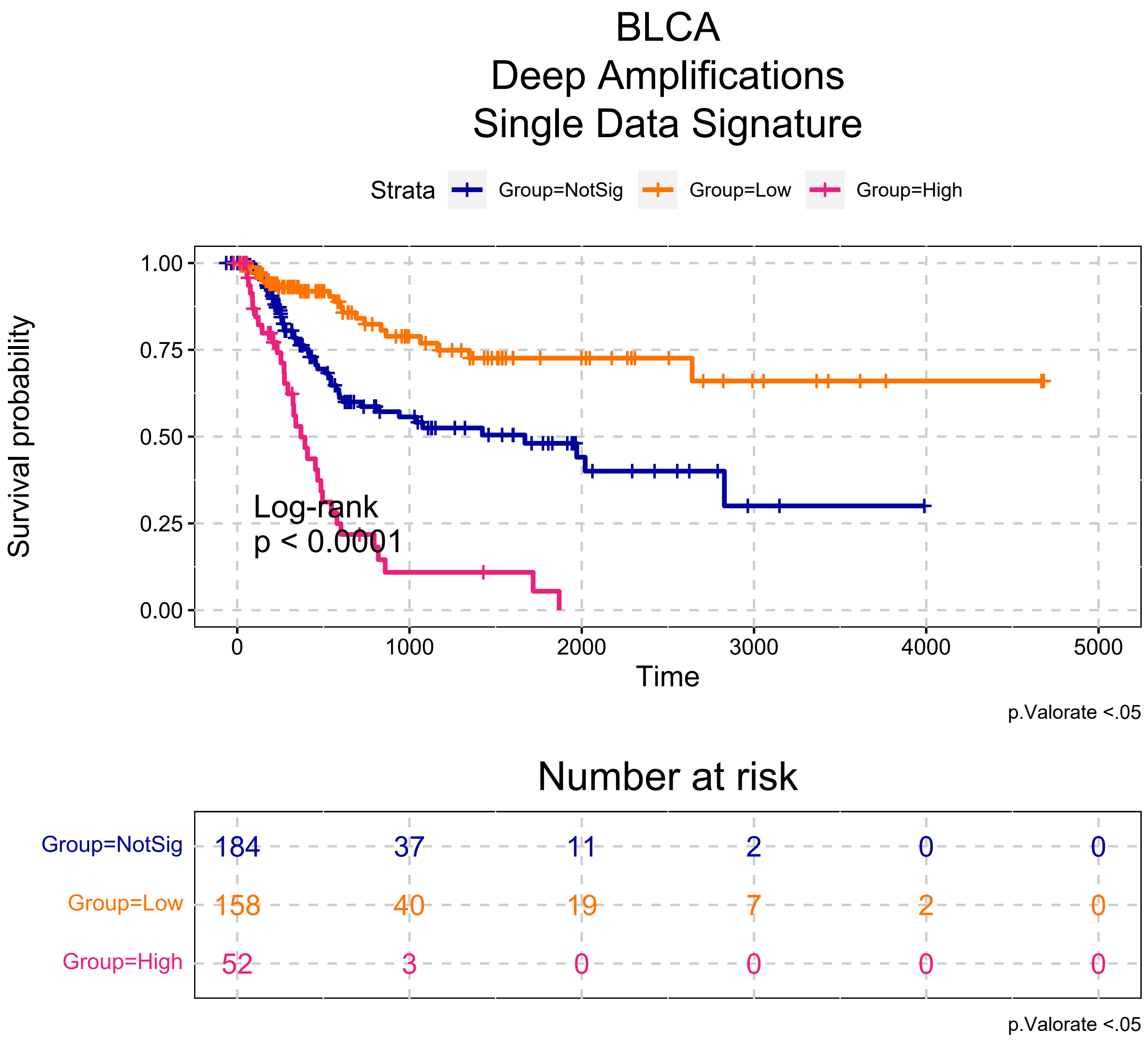

explanatory	beta	HR	L95	U95	p
Low	-0.94	0.39	0.24	0.64	0.00
High	1.15	3.16	2.03	4.93	0.00

n= 398, number of events =107
Score(logrank) test = p <.0001

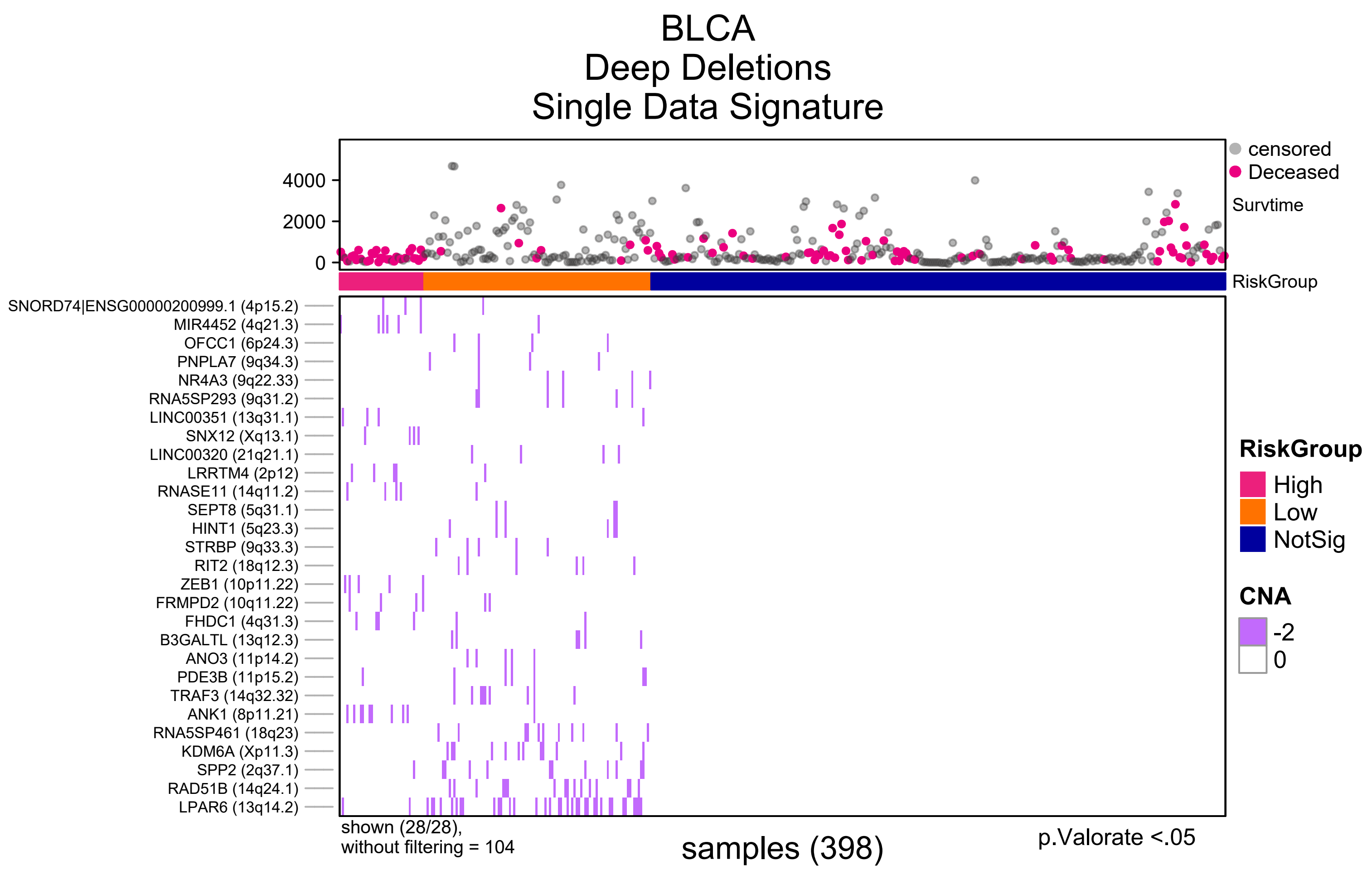
BLCA
Deep Deletions
Single Data Signature
4000
2000
0
censored
Deceased
Survtime
RiskGroup
SNORD74|ENSG00000200999.1 (4p15.2)
MIR4452 (4q21.3)
OFCC1 (6p24.3)
PNPLA7 (9q34.3)
NR4A3 (9q22.33)
RNA5SP293 (9q31.2)
LINC00351 (13q31.1)
SNX12 (Xq13.1)
LINC00320 (21q21.1)
LRRTM4 (2p12)
RNASE11 (14q11.2)
SEPT8 (5q31.1)
HINT1 (5q23.3)
STRBP (9q33.3)
RIT2 (18q12.3)
ZEB1 (10p11.22)
FRMPD2 (10q11.22)
FHDC1 (4q31.3)
B3GALTL (13q12.3)
ANO3 (11p14.2)
PDE3B (11p15.2)
TRAF3 (14q32.32)
ANK1 (8p11.21)
RNA5SP461 (18q23)
KDM6A (Xp11.3)
SPP2 (2q37.1)
RAD51B (14q24.1)
LPAR6 (13q14.2)
RiskGroup
High
Low
NotSig
CNA
-2
0
shown (28/28),
without filtering = 104
samples (398)
p.Valorate <.05

BLCA
Deep Deletions
Single Data Signature

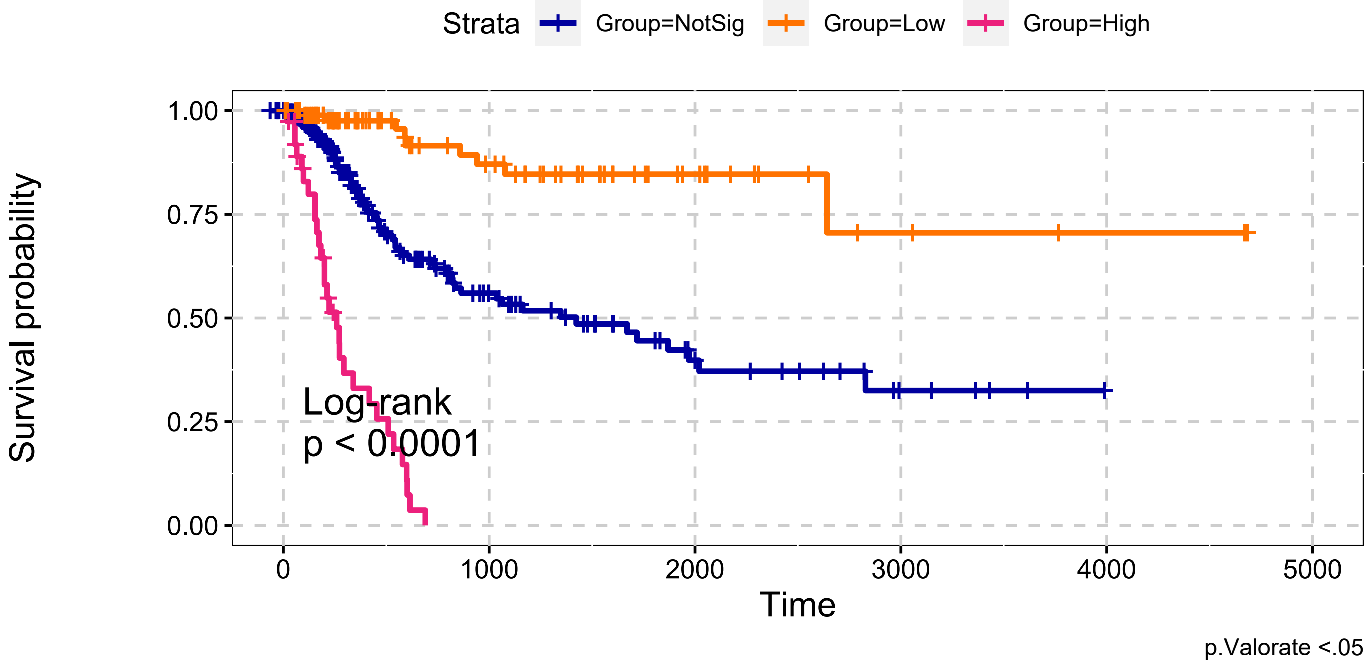

explanatory	beta	HR	L95	U95	p
Low	-1.55	0.21	0.11	0.42	0.00
High	1.77	5.84	3.70	9.22	0.00

n= 398, number of events =107
Score(logrank) test = p <.0001

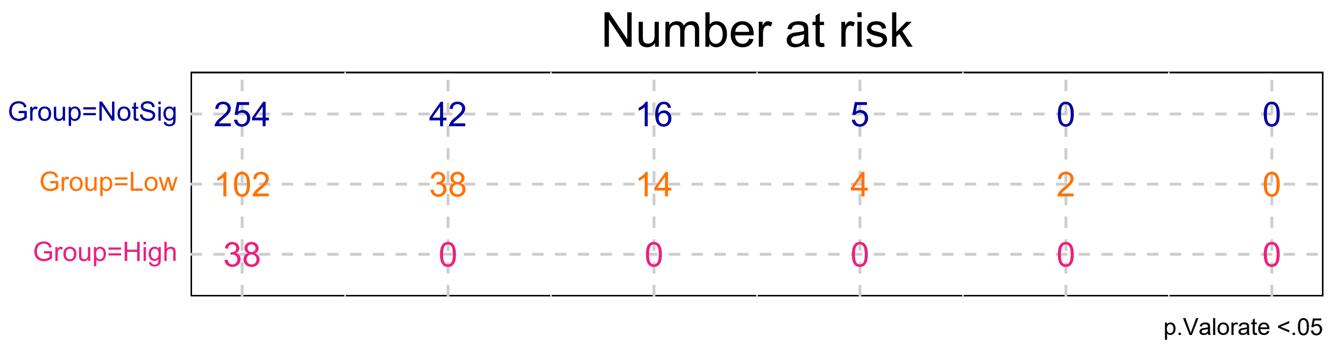

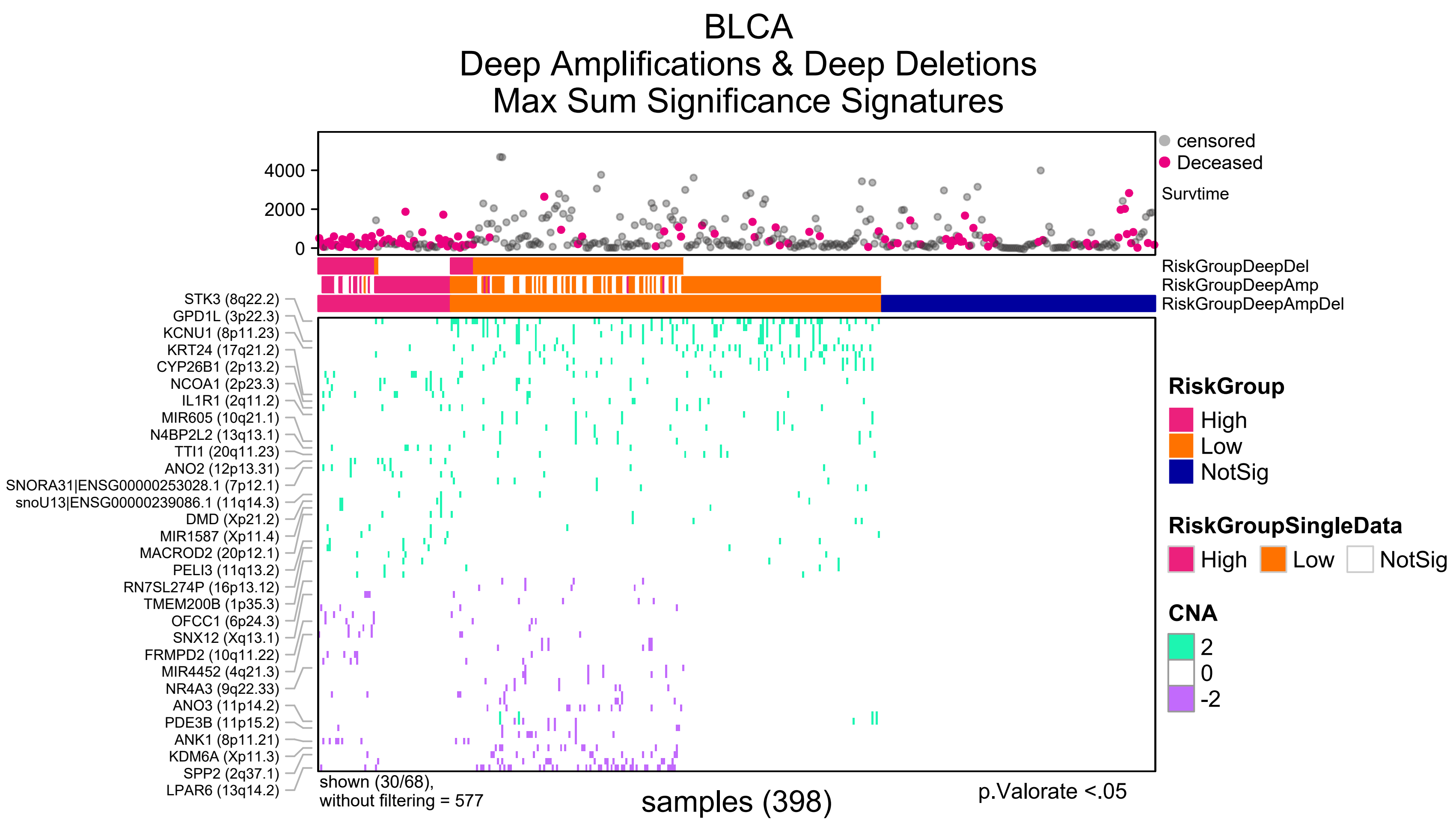
BLCA
Deep Amplifications & Deep Deletions
Max Sum Significance Signatures
censored
Deceased
Survtime
4000
2000
0
RiskGroupDeepDel
RiskGroupDeepAmp
RiskGroupDeepAmpDel
STK3 (8q22.2)
GPD1L (3p22.3)
KCNU1 (8p11.23)
KRT24 (17q21.2)
CYP26B1 (2p13.2)
NCOA1 (2p23.3)
IL1R1 (2q11.2)
MIR605 (10q21.1)
N4BP2L2 (13q13.1)
TTI1 (20q11.23)
ANO2 (12p13.31)
SNORA31|ENSG00000253028.1 (7p12.1)
snoU13|ENSG00000239086.1 (11q14.3)
DMD (Xp21.2)
MIR1587 (Xp11.4)
MACROD2 (20p12.1)
PELI3 (11q13.2)
RN7SL274P (16p13.12)
TMEM200B (1p35.3)
OFCC1 (6p24.3)
SNX12 (Xq13.1)
FRMPD2 (10q11.22)
MIR4452 (4q21.3)
NR4A3 (9q22.33)
ANO3 (11p14.2)
PDE3B (11p15.2)
ANK1 (8p11.21)
KDM6A (Xp11.3)
SPP2 (2q37.1)
LPAR6 (13q14.2)
RiskGroup
High
Low
NotSig
RiskGroupSingleData
High
Low
NotSig
CNA
2
0
-2
shown (30/68),
without filtering = 577
samples (398)
p.Valorate <.05

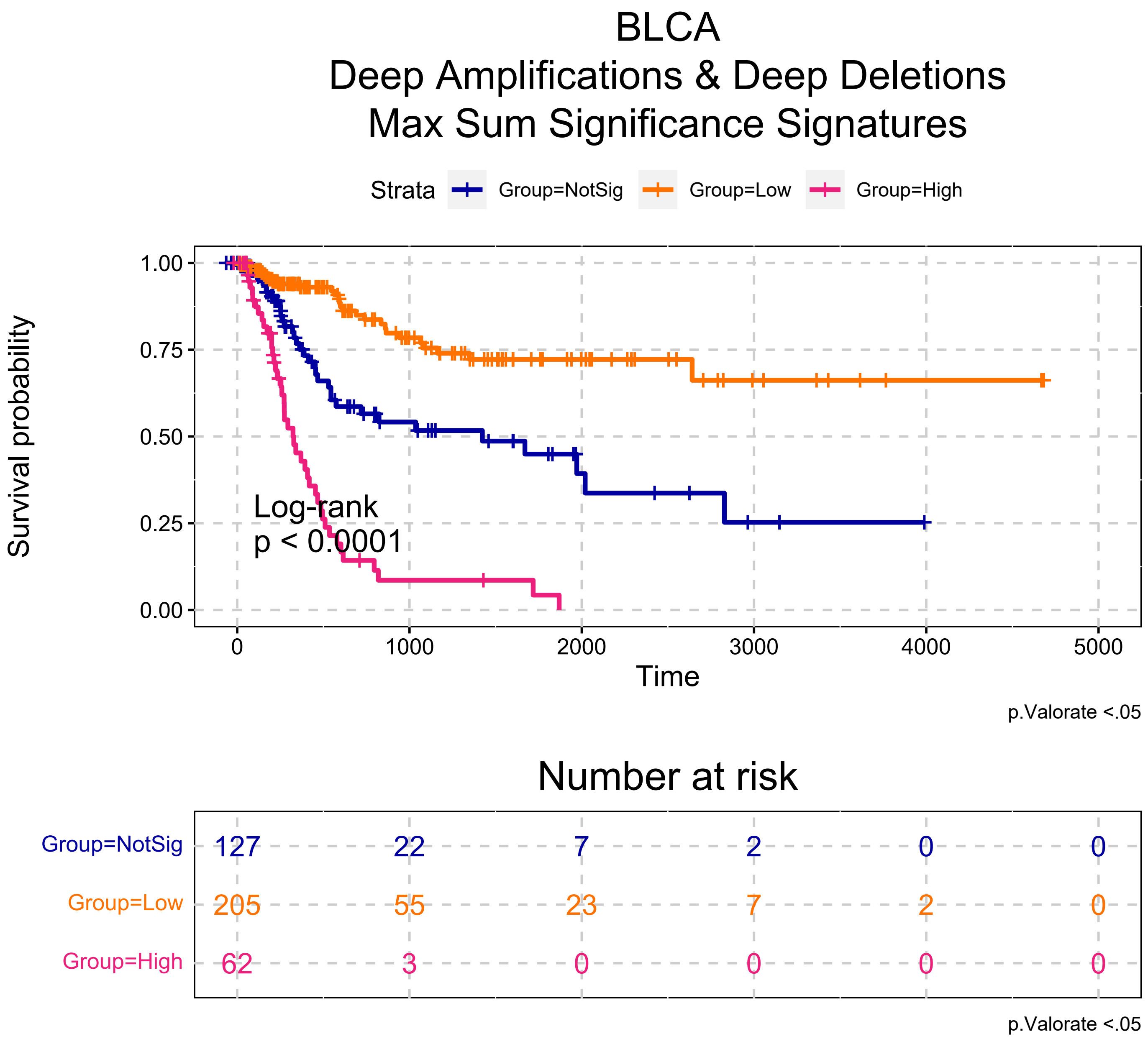

explanatory	beta	HR	L95	U95	p
Low	-1.07	0.34	0.21	0.56	0.00
High	1.24	3.47	2.20	5.46	0.00

n= 398, number of events =107
Score(logrank) test = p <.0001

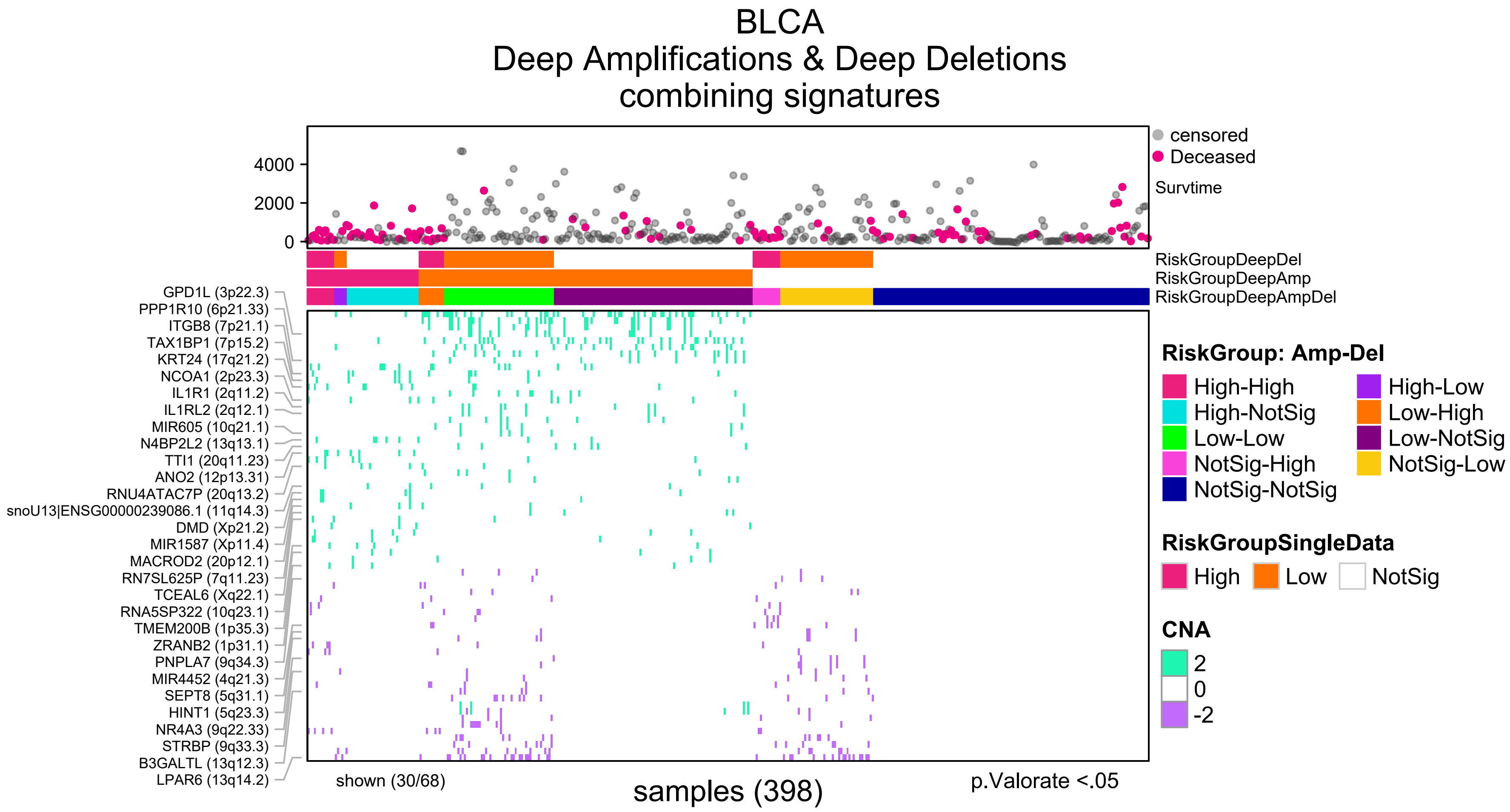

BLCA
Deep Amplifications & Deep Deletions
combining signatures
4000
2000
0
censored
Deceased
Survtime
RiskGroupDeepDel
RiskGroupDeepAmp
RiskGroupDeepAmpDel
GPD1L (3p22.3)
PPP1R10 (6p21.33)
ITGB8 (7p21.1)
TAX1BP1 (7p15.2)
KRT24 (17q21.2)
NCOA1 (2p23.3)
IL1R1 (2q11.2)
IL1RL2 (2q12.1)
MIR605 (10q21.1)
N4BP2L2 (13q13.1)
TTI1 (20q11.23)
ANO2 (12p13.31)
RNU4ATAC7P (20q13.2)
snoU13|ENSG00000239086.1 (11q14.3)
DMD (Xp21.2)
MIR1587 (Xp11.4)
MACROD2 (20p12.1)
RN7SL625P (7q11.23)
TCEAL6 (Xq22.1)
RNA5SP322 (10q23.1)
TMEM200B (1p35.3)
ZRANB2 (1p31.1)
PNPLA7 (9q34.3)
MIR4452 (4q21.3)
SEPT8 (5q31.1)
HINT1 (5q23.3)
NR4A3 (9q22.33)
STRBP (9q33.3)
B3GALTL (13q12.3)
LPAR6 (13q14.2)
RiskGroup: Amp-Del
High-High
High-NotSig
Low-Low
NotSig-High
NotSig-NotSig
High-Low
Low-High
Low-NotSig
NotSig-Low
RiskGroupSingleData
High
Low
NotSig
CNA
2
0
-2
shown (30/68)
samples (398)
p.Valorate <.05

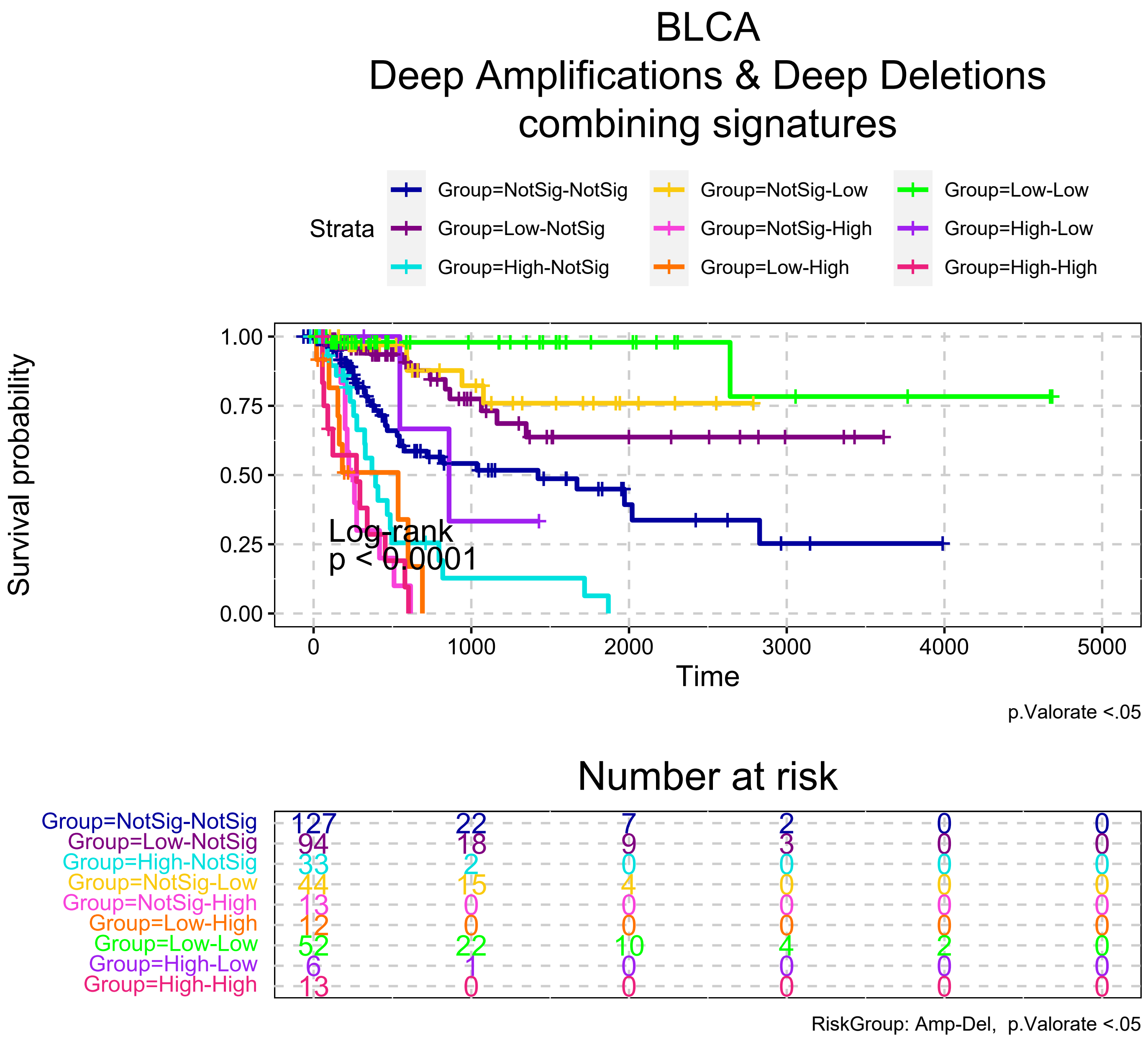

explanatory	beta	HR	L95	U95	p
Low-NotSig	-1.03	0.36	0.19	0.68	0.00
High-NotSig	1.09	2.98	1.71	5.20	0.00
NotSig-Low	-1.33	0.26	0.10	0.68	0.01
NotSig-High	1.73	5.61	2.78	11.34	0.00
Low-High	1.56	4.76	2.17	10.44	0.00
Low-Low	-2.63	0.07	0.02	0.30	0.00
High-Low	-0.02	0.98	0.24	4.09	0.98
High-High	1.84	6.27	3.12	12.62	0.00

n= 398, number of events =107
Score(logrank) test = p <.0001
