# Supplementary material for: Systematic Modeling of Risk-Associated Copy Number Alterations in Cancer
Source: Int J Mol Sci. 2024 Sep 27;25(19):10455. doi: 10.3390/ijms251910455 (PMC11477427; doi:10.3390/ijms251910455)
Supplement: Supplementary file 1 [file ijms-25-10455-s001.zip › BRCASignatureV12-sinSombreado.pdf]

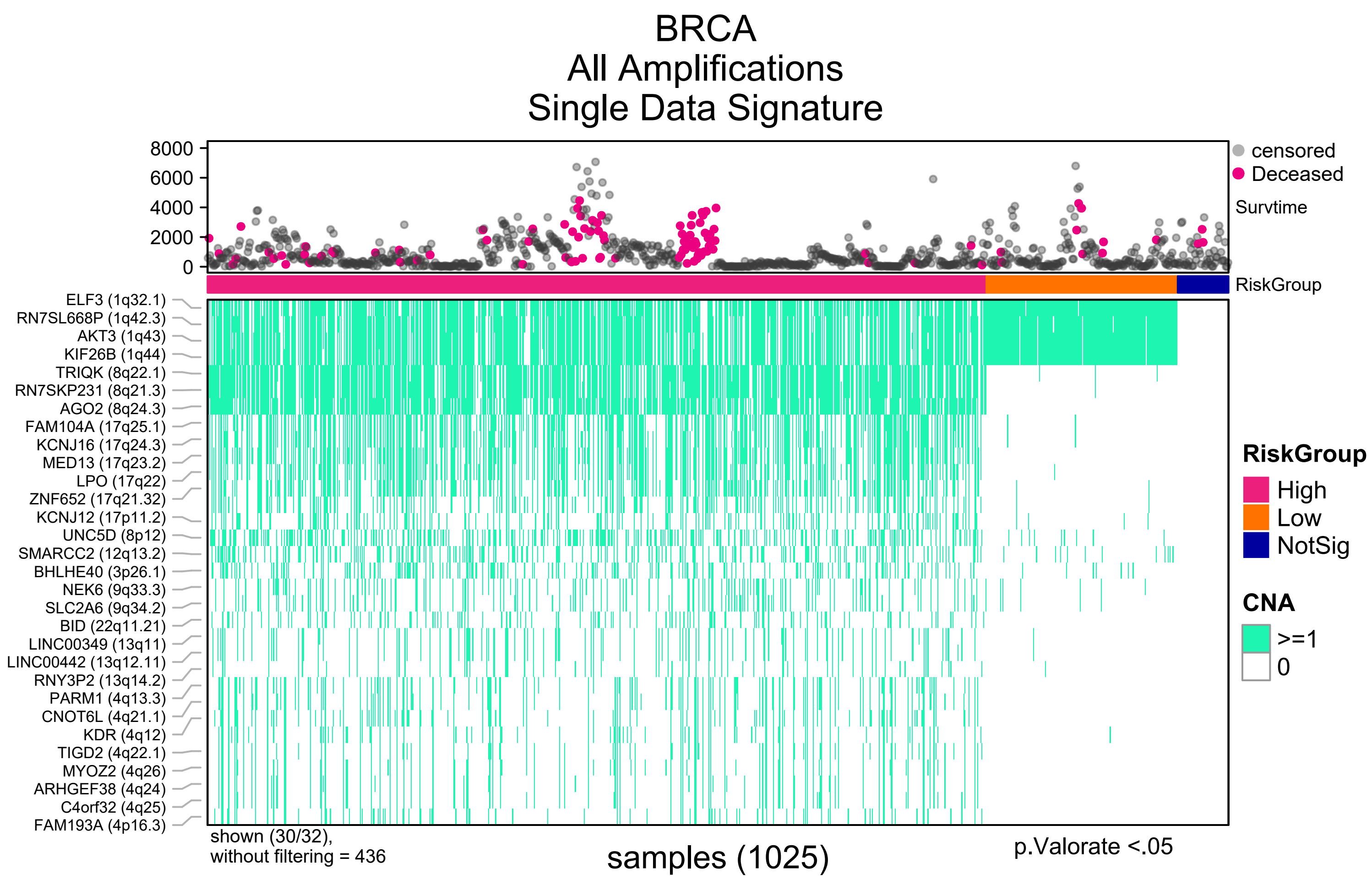

BRCA
All Amplifications
Single Data Signature
8000
6000
4000
2000
0
censored
Deceased
Survtime
RiskGroup
ELF3 (1q32.1)
RN7SL668P (1q42.3)
AKT3 (1q43)
KIF26B (1q44)
TRIQK (8q22.1)
RN7SKP231 (8q21.3)
AGO2 (8q24.3)
FAM104A (17q25.1)
KCNJ16 (17q24.3)
MED13 (17q23.2)
LPO (17q22)
ZNF652 (17q21.32)
KCNJ12 (17p11.2)
UNC5D (8p12)
SMARCC2 (12q13.2)
BHLHE40 (3p26.1)
NEK6 (9q33.3)
SLC2A6 (9q34.2)
BID (22q11.21)
LINC00349 (13q11)
LINC00442 (13q12.11)
RNY3P2 (13q14.2)
PARM1 (4q13.3)
CNOT6L (4q21.1)
KDR (4q12)
TIGD2 (4q22.1)
MYOZ2 (4q26)
ARHGEF38 (4q24)
C4orf32 (4q25)
FAM193A (4p16.3)
RiskGroup
High
Low
NotSig
CNA
>=1
0
shown (30/32),
without filtering = 436
samples (1025)
p.Valorate <.05

BRCA
All Amplifications
Single Data Signature

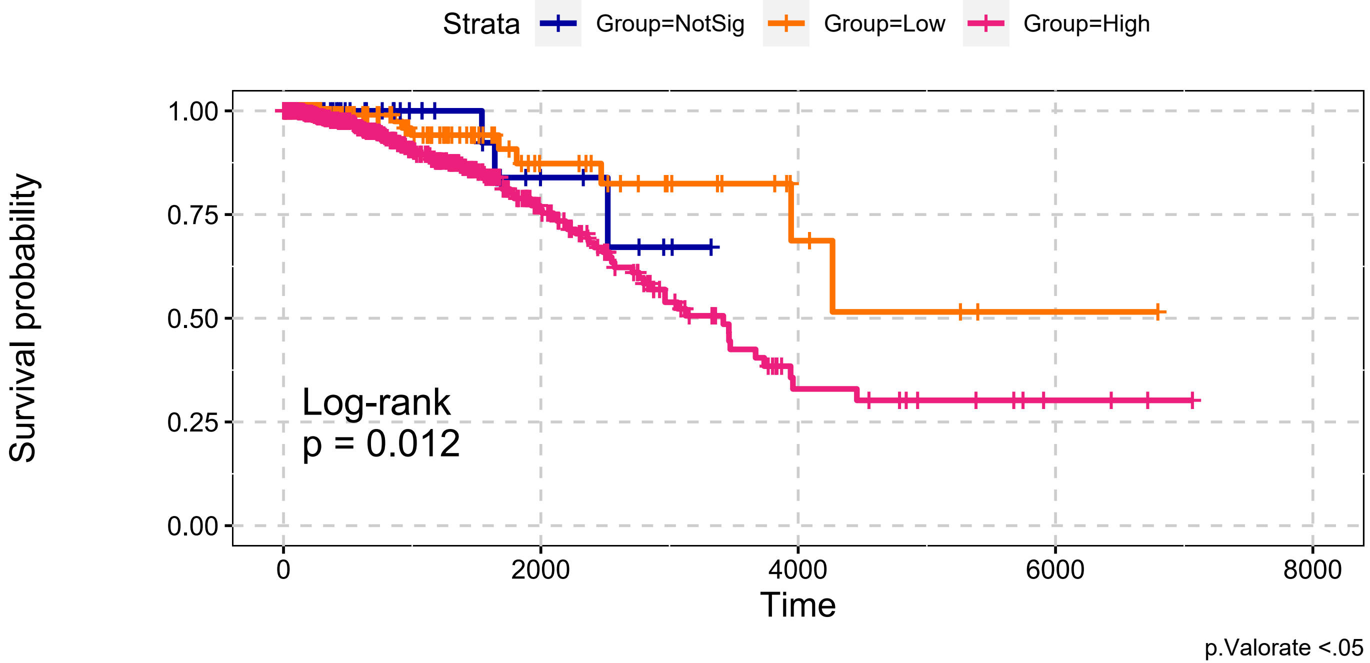

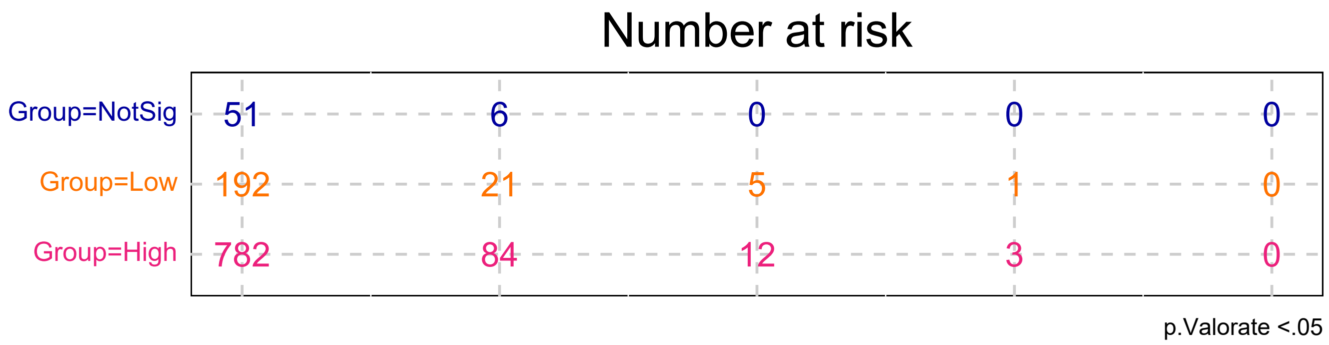

explanatory	beta	HR	L95	U95	p
Low	-0.32	0.72	0.19	2.68	0.63
High	0.63	1.87	0.59	5.92	0.29

n= 1025, number of events =101
Score(logrank) test = 0.012

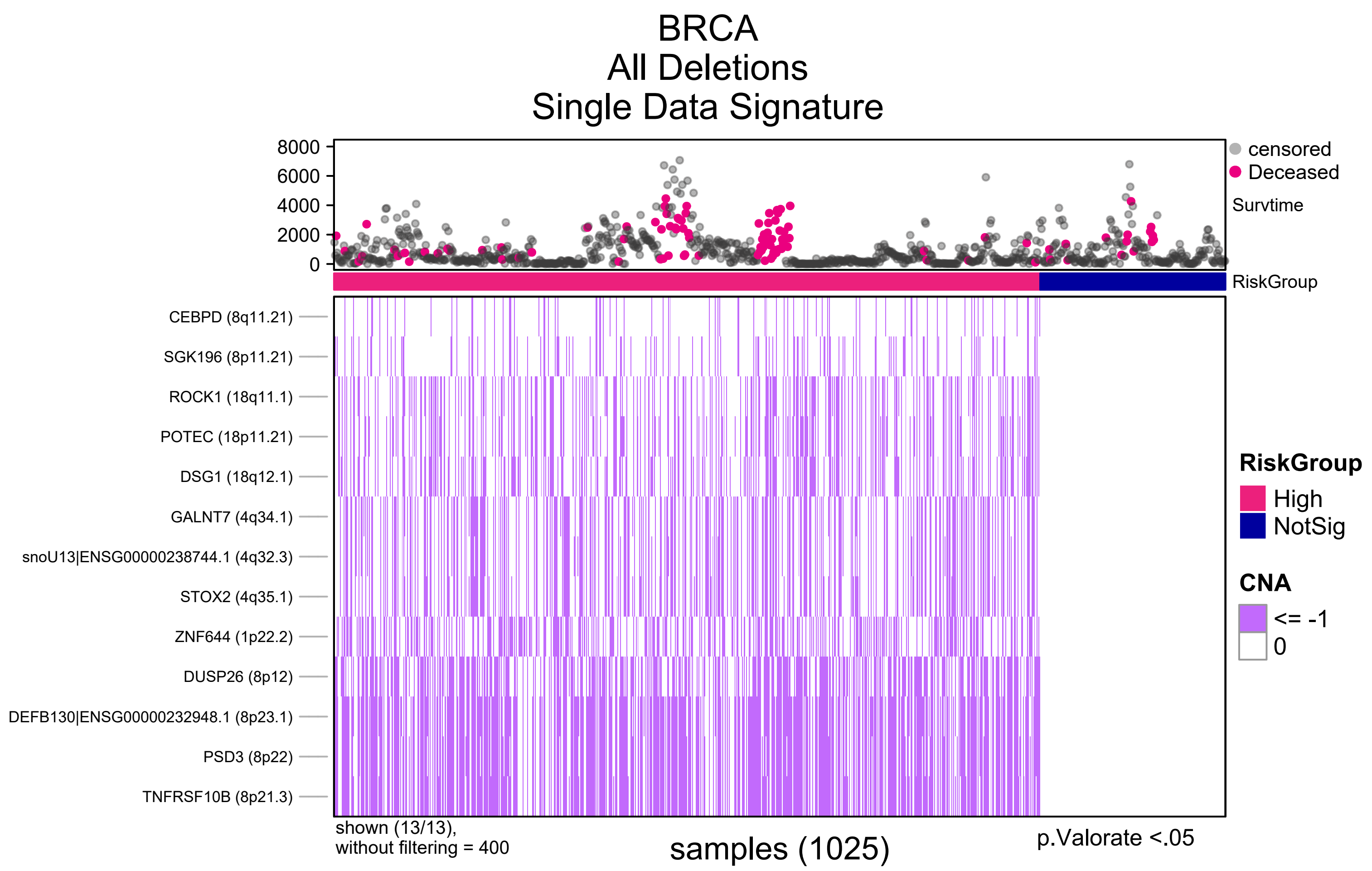

BRCA
All Deletions
Single Data Signature
8000
6000
4000
2000
0
censored
Deceased
Survtime
RiskGroup
CEBPD (8q11.21)
SGK196 (8p11.21)
ROCK1 (18q11.1)
POTEC (18p11.21)
DSG1 (18q12.1)
GALNT7 (4q34.1)
snoU13|ENSG00000238744.1 (4q32.3)
STOX2 (4q35.1)
ZNF644 (1p22.2)
DUSP26 (8p12)
DEFB130|ENSG00000232948.1 (8p23.1)
PSD3 (8p22)
TNFRSF10B (8p21.3)
RiskGroup
High
NotSig
CNA
<= -1
0
shown (13/13),
without filtering = 400
samples (1025)
p.Valorate <.05

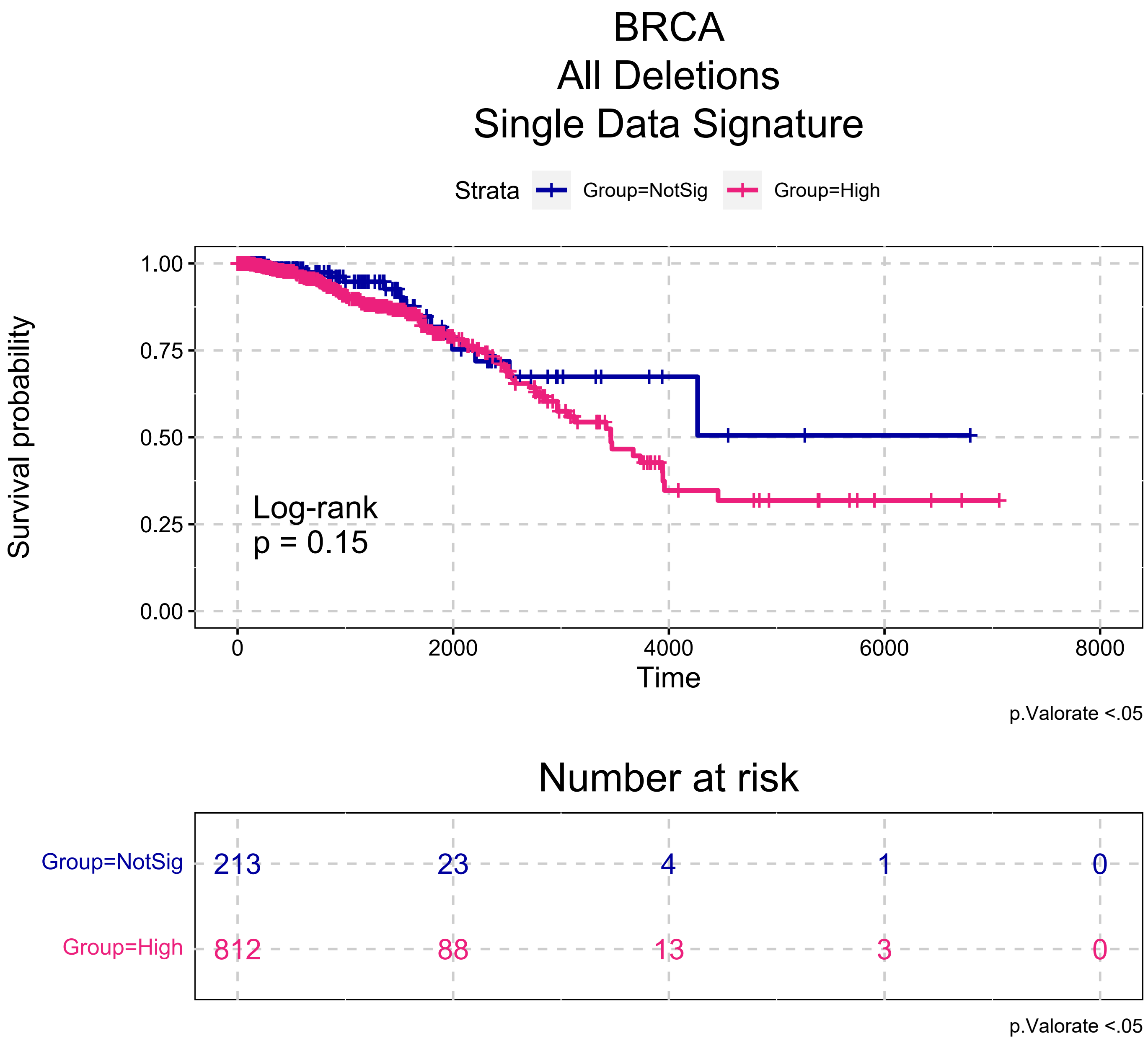

explanatory	beta	HR	L95	U95	p
High	0.40	1.49	0.86	2.58	0.15

n= 1025, number of events =101
Score(logrank) test = 0.152

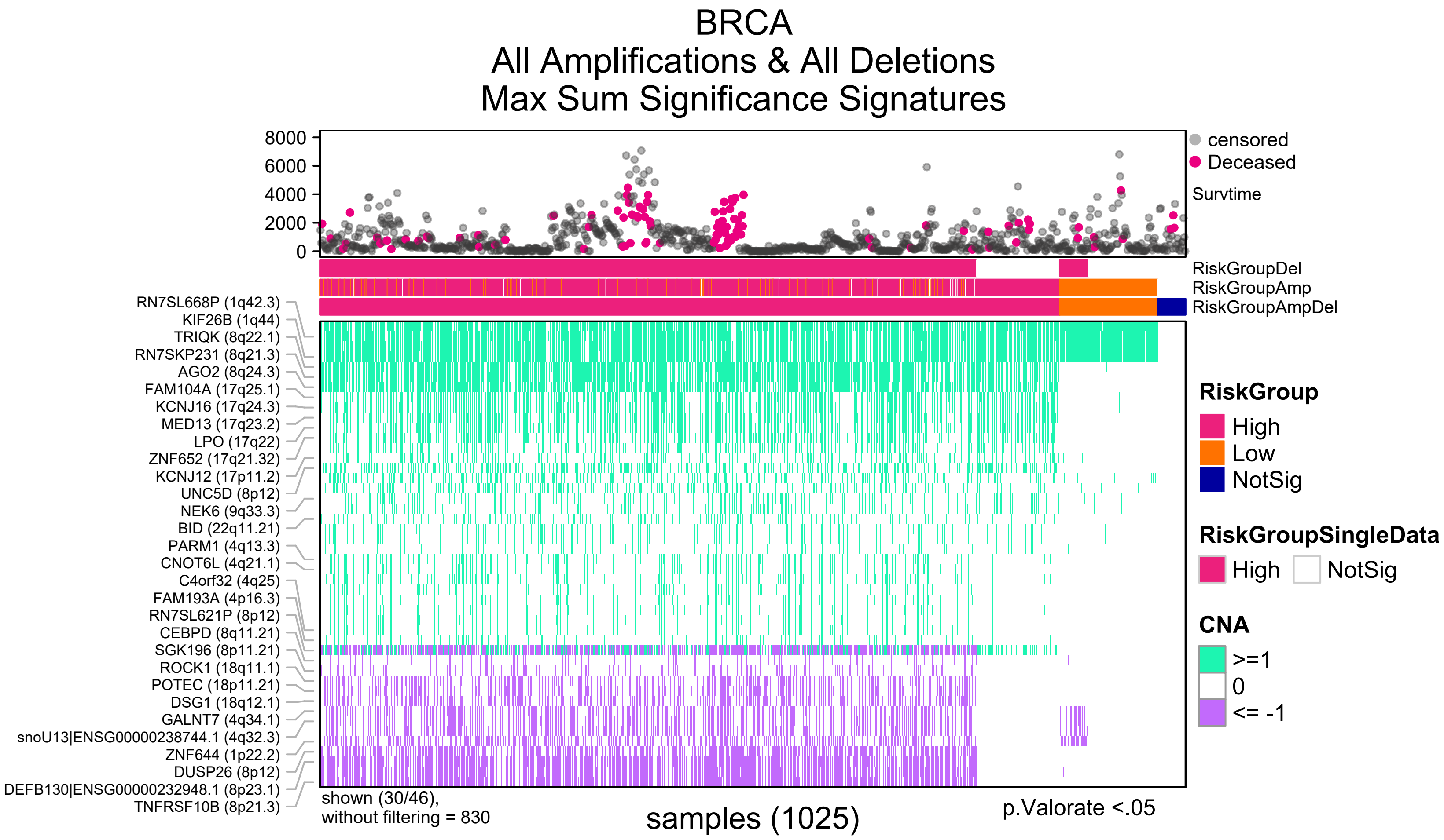
BRCA
All Amplifications & All Deletions
Max Sum Significance Signatures
8000
6000
4000
2000
0
censored
Deceased
Survtime
RiskGroupDel
RiskGroupAmp
RiskGroupAmpDel
RN7SL668P (1q42.3)
KIF26B (1q44)
TRIQK (8q22.1)
RN7SKP231 (8q21.3)
AGO2 (8q24.3)
FAM104A (17q25.1)
KCNJ16 (17q24.3)
MED13 (17q23.2)
LPO (17q22)
ZNF652 (17q21.32)
KCNJ12 (17p11.2)
UNC5D (8p12)
NEK6 (9q33.3)
BID (22q11.21)
PARM1 (4q13.3)
CNOT6L (4q21.1)
C4orf32 (4q25)
FAM193A (4p16.3)
RN7SL621P (8p12)
CEBPD (8q11.21)
SGK196 (8p11.21)
ROCK1 (18q11.1)
POTEC (18p11.21)
DSG1 (18q12.1)
GALNT7 (4q34.1)
snoU13|ENSG00000238744.1 (4q32.3)
ZNF644 (1p22.2)
DUSP26 (8p12)
DEFB130|ENSG00000232948.1 (8p23.1)
TNFRSF10B (8p21.3)
RiskGroup
High
Low
NotSig
RiskGroupSingleData
High
NotSig
CNA
>=1
0
<= -1
shown (30/46),
without filtering = 830
samples (1025)
p.Valorate <.05

BRCA
All Amplifications & All Deletions
Max Sum Significance Signatures

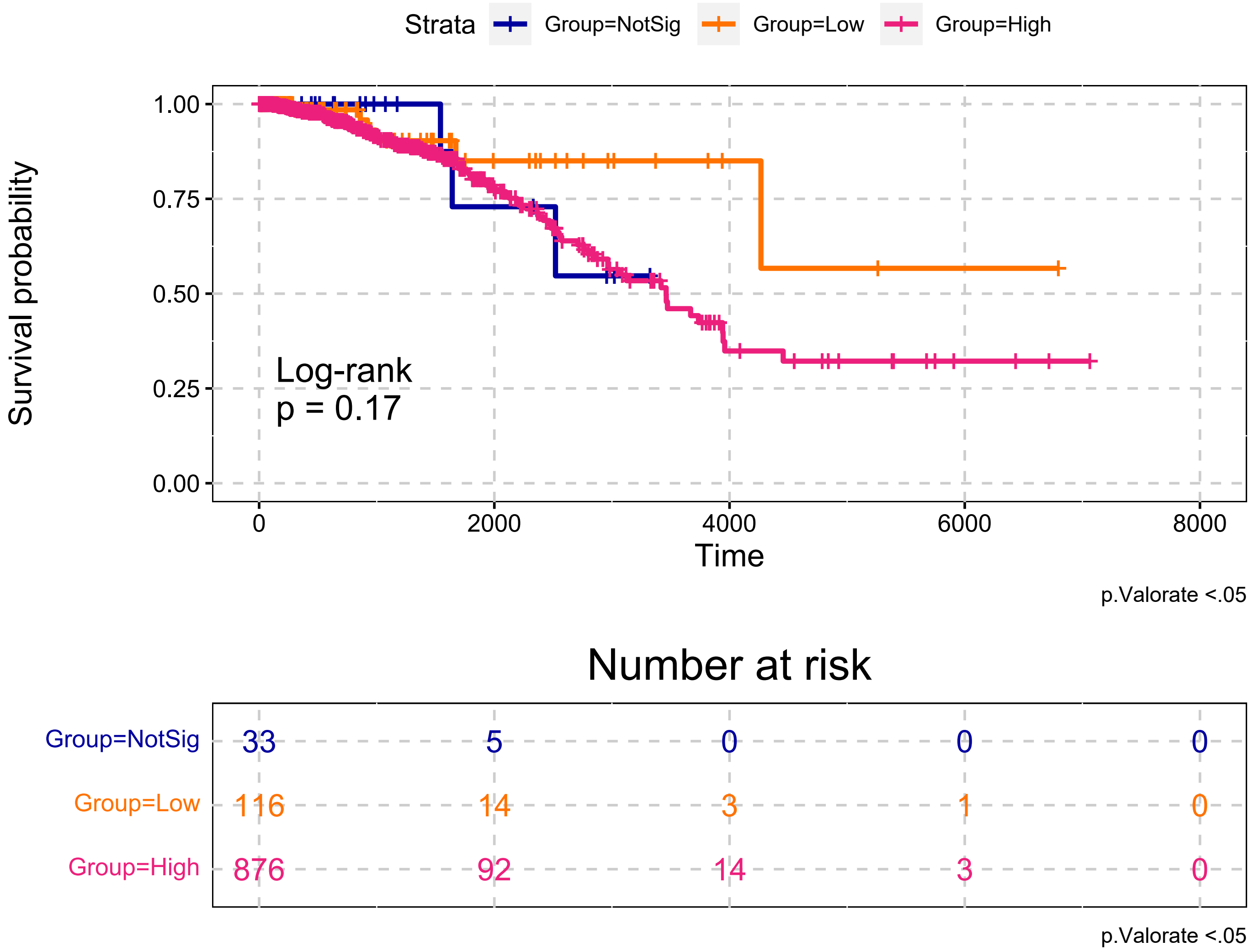

explanatory	beta	HR	L95	U95	p
Low	-0.60	0.55	0.14	2.20	0.40
High	0.16	1.18	0.37	3.73	0.78

n= 1025, number of events =101
Score(logrank) test = 0.173

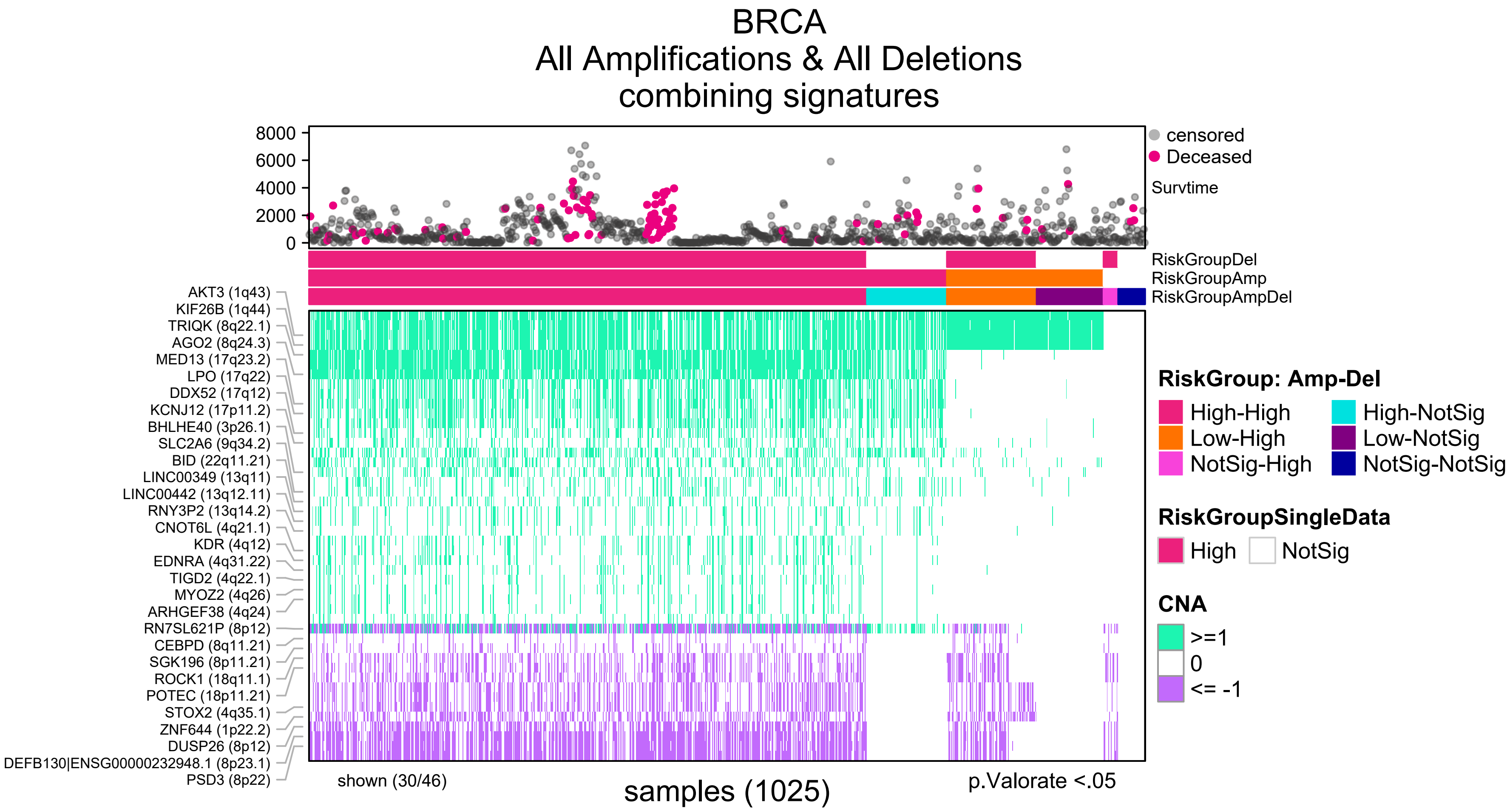
BRCA
All Amplifications & All Deletions
combining signatures
8000
6000
4000
2000
0
censored
Deceased
Survtime
RiskGroupDel
RiskGroupAmp
RiskGroupAmpDel
AKT3 (1q43)
KIF26B (1q44)
TRIQK (8q22.1)
AGO2 (8q24.3)
MED13 (17q23.2)
LPO (17q22)
DDX52 (17q12)
KCNJ12 (17p11.2)
BHLHE40 (3p26.1)
SLC2A6 (9q34.2)
BID (22q11.21)
LINC00349 (13q11)
LINC00442 (13q12.11)
RNY3P2 (13q14.2)
CNOT6L (4q21.1)
KDR (4q12)
EDNRA (4q31.22)
TIGD2 (4q22.1)
MYOZ2 (4q26)
ARHGEF38 (4q24)
RN7SL621P (8p12)
CEBPD (8q11.21)
SGK196 (8p11.21)
ROCK1 (18q11.1)
POTEC (18p11.21)
STOX2 (4q35.1)
ZNF644 (1p22.2)
DUSP26 (8p12)
DEFB130|ENSG00000232948.1 (8p23.1)
PSD3 (8p22)
shown (30/46)
samples (1025)
p.Valorate <.05
RiskGroup: Amp-Del
High-High
Low-High
NotSig-High
High-NotSig
Low-NotSig
NotSig-NotSig
RiskGroupSingleData
High
NotSig
CNA
>=1
0
<= -1

BRCA
All Amplifications & All Deletions combining signatures

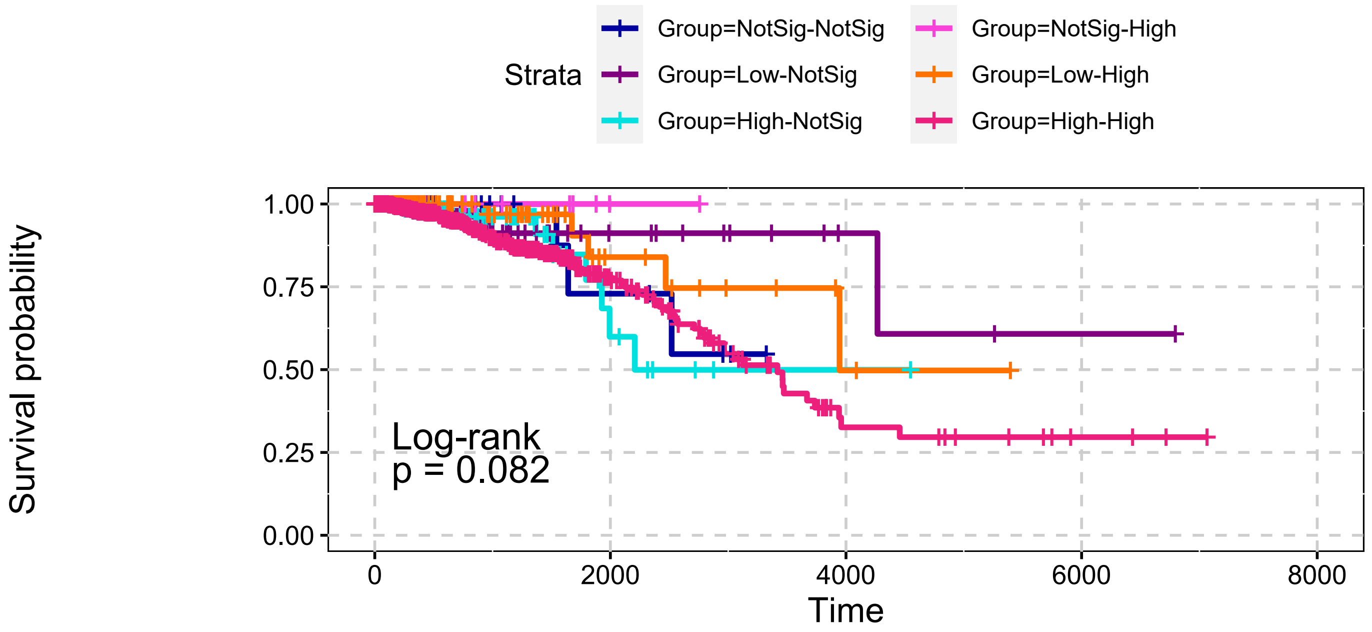

p.Valorate <.05

Number at risk

Group=NotSig-NotSig	33	5	0	0	0
Group=Low-NotSig	82	11	3	1	0
Group=High-NotSig	98	7	1	0	0
Group=NotSig-High	18	1	0	0	0
Group=Low-High	110	10	2	0	0
Group=High-High	684	77	11	3	0

RiskGroup: Amp-Del, p.Valorate <.05

explanatory	beta	HR	L95	U95	p
Low-NotSig	-0.83	0.43	0.10	1.95	0.28
High-NotSig	0.16	1.18	0.31	4.45	0.81
NotSig-High	-15.95	0.00	0.00	Inf	0.99
Low-High	-0.59	0.56	0.13	2.34	0.42
High-High	0.25	1.29	0.41	4.09	0.67

n= 1025, number of events =101
Score(logrank) test = 0.082

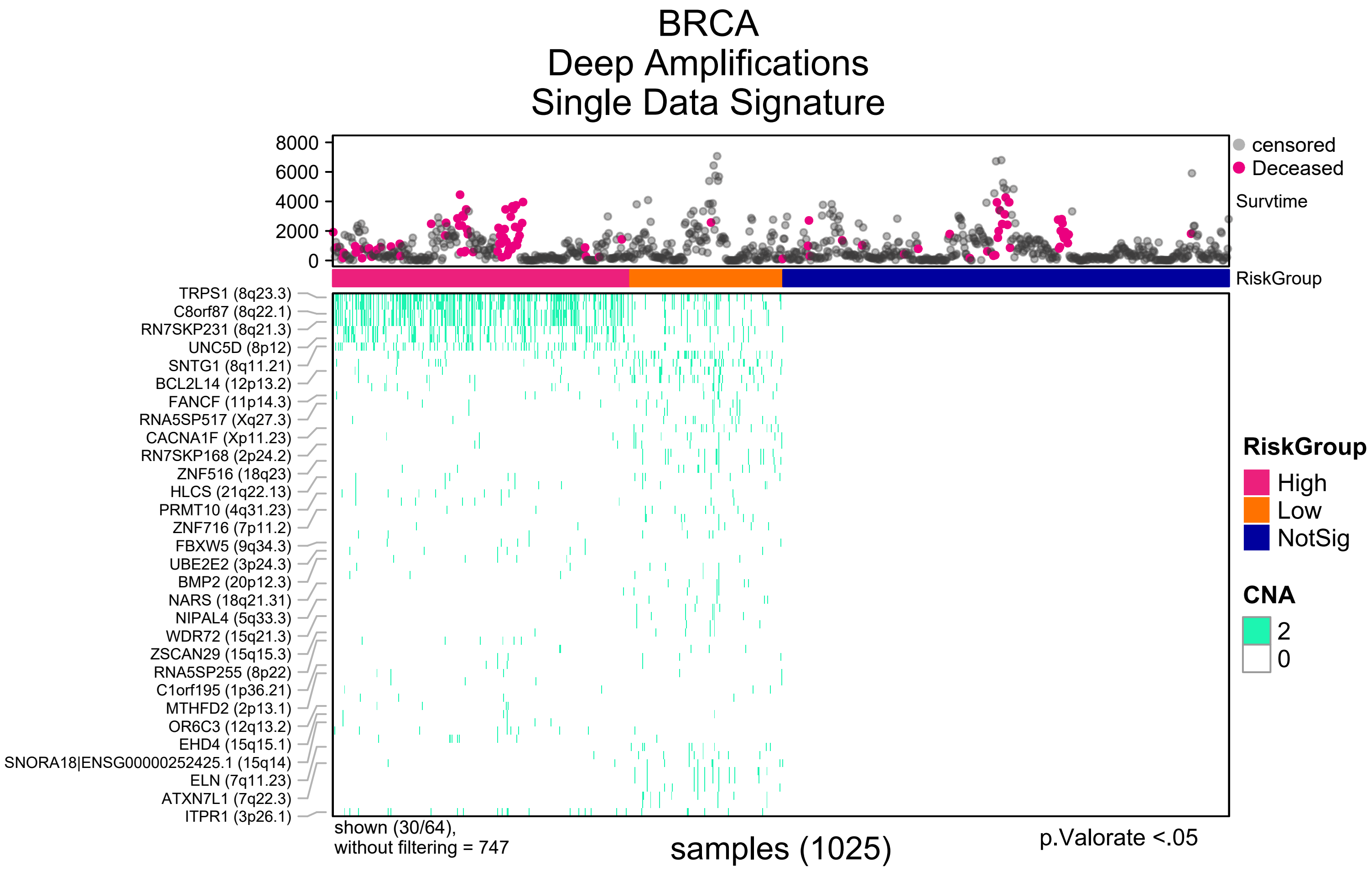

BRCA
Deep Amplifications
Single Data Signature
8000
6000
4000
2000
0
censored
Deceased
Survtime
RiskGroup
TRPS1 (8q23.3)
C8orf87 (8q22.1)
RN7SKP231 (8q21.3)
UNC5D (8p12)
SNTG1 (8q11.21)
BCL2L14 (12p13.2)
FANCF (11p14.3)
RNA5SP517 (Xq27.3)
CACNA1F (Xp11.23)
RN7SKP168 (2p24.2)
ZNF516 (18q23)
HLCS (21q22.13)
PRMT10 (4q31.23)
ZNF716 (7p11.2)
FBXW5 (9q34.3)
UBE2E2 (3p24.3)
BMP2 (20p12.3)
NARS (18q21.31)
NIPAL4 (5q33.3)
WDR72 (15q21.3)
ZSCAN29 (15q15.3)
RNA5SP255 (8p22)
C1orf195 (1p36.21)
MTHFD2 (2p13.1)
OR6C3 (12q13.2)
EHD4 (15q15.1)
SNORA18|ENSG00000252425.1 (15q14)
ELN (7q11.23)
ATXN7L1 (7q22.3)
ITPR1 (3p26.1)
RiskGroup
High
Low
NotSig
CNA
2
0
shown (30/64),
without filtering = 747
samples (1025)
p.Valorate <.05

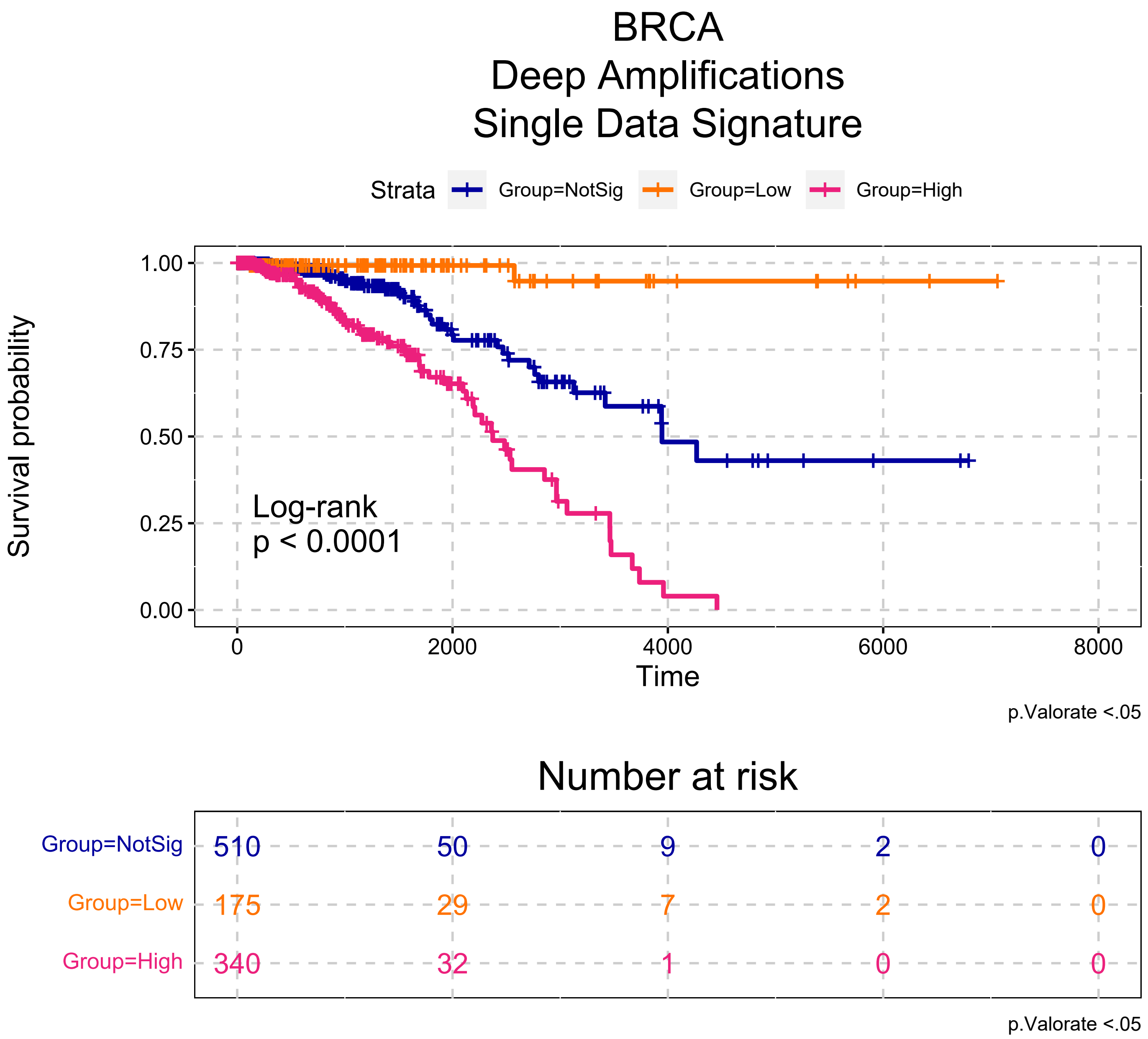

explanatory	beta	HR	L95	U95	p
Low	-2.25	0.11	0.03	0.44	0.00
High	1.12	3.08	2.03	4.67	0.00

n= 1025, number of events =101
Score(logrank) test = p <.0001

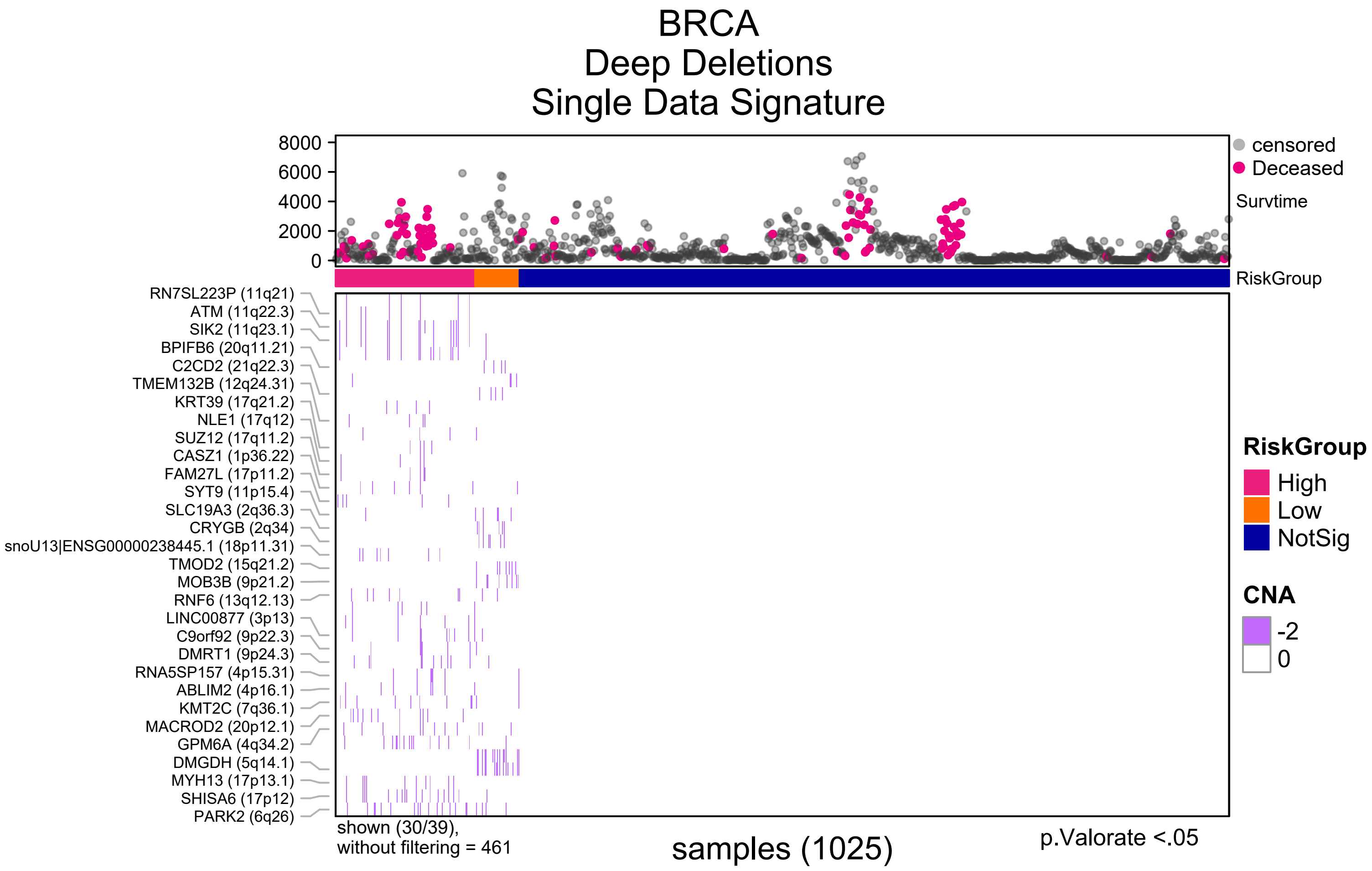
BRCA
Deep Deletions
Single Data Signature
8000
6000
4000
2000
0
censored
Deceased
Survtime
RiskGroup
RN7SL223P (11q21)
ATM (11q22.3)
SIK2 (11q23.1)
BPIFB6 (20q11.21)
C2CD2 (21q22.3)
TMEM132B (12q24.31)
KRT39 (17q21.2)
NLE1 (17q12)
SUZ12 (17q11.2)
CASZ1 (1p36.22)
FAM27L (17p11.2)
SYT9 (11p15.4)
SLC19A3 (2q36.3)
CRYGB (2q34)
snoU13|ENSG00000238445.1 (18p11.31)
TMOD2 (15q21.2)
MOB3B (9p21.2)
RNF6 (13q12.13)
LINC00877 (3p13)
C9orf92 (9p22.3)
DMRT1 (9p24.3)
RNA5SP157 (4p15.31)
ABLIM2 (4p16.1)
KMT2C (7q36.1)
MACROD2 (20p12.1)
GPM6A (4q34.2)
DMGDH (5q14.1)
MYH13 (17p13.1)
SHISA6 (17p12)
PARK2 (6q26)
RiskGroup
High
Low
NotSig
CNA
-2
0
shown (30/39),
without filtering = 461
samples (1025)
p.Valorate <.05

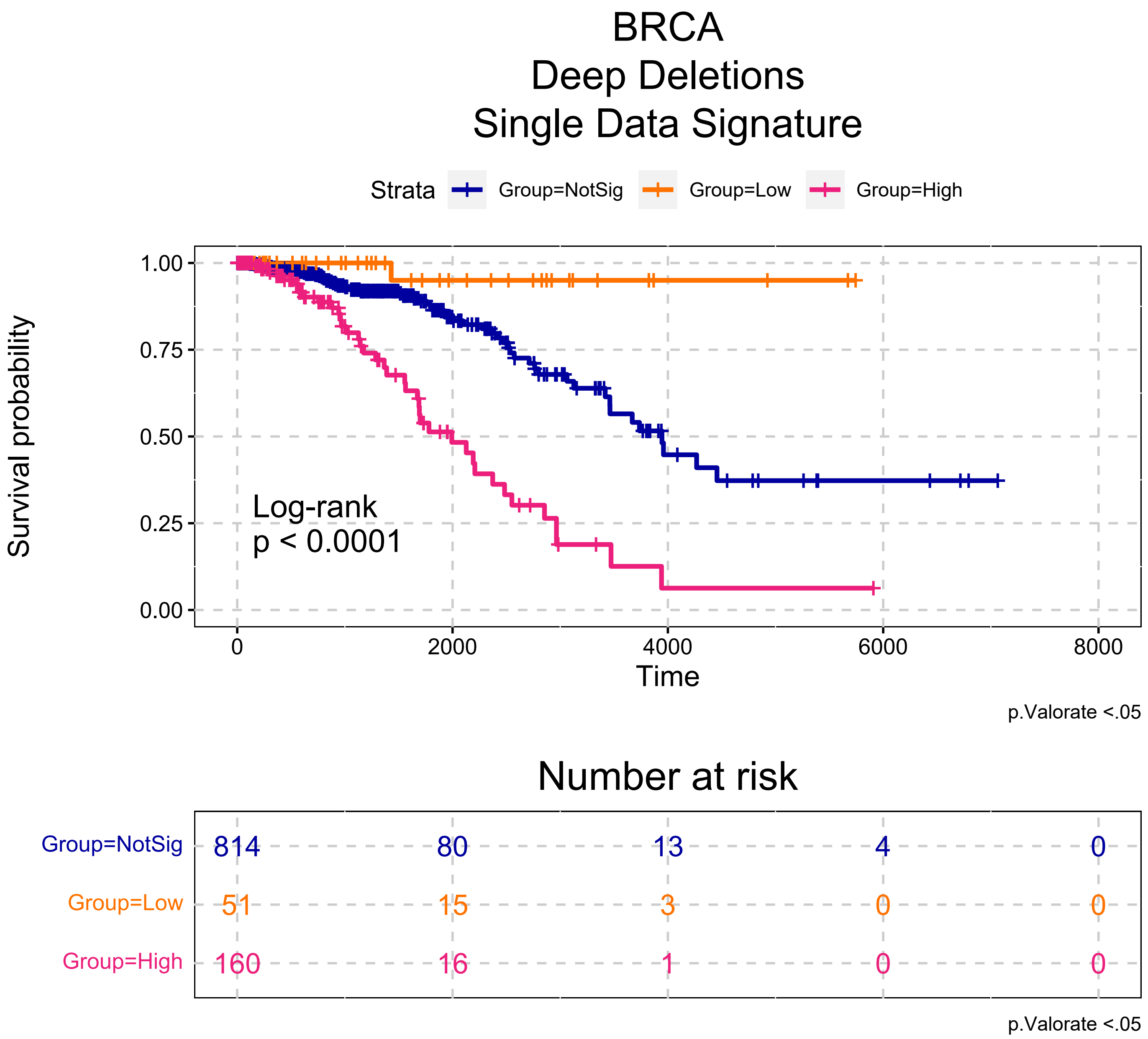

explanatory	beta	HR	L95	U95	p
Low	-2.19	0.11	0.02	0.81	0.03
High	1.27	3.56	2.38	5.32	0.00

n= 1025, number of events =101
Score(logrank) test = p <.0001

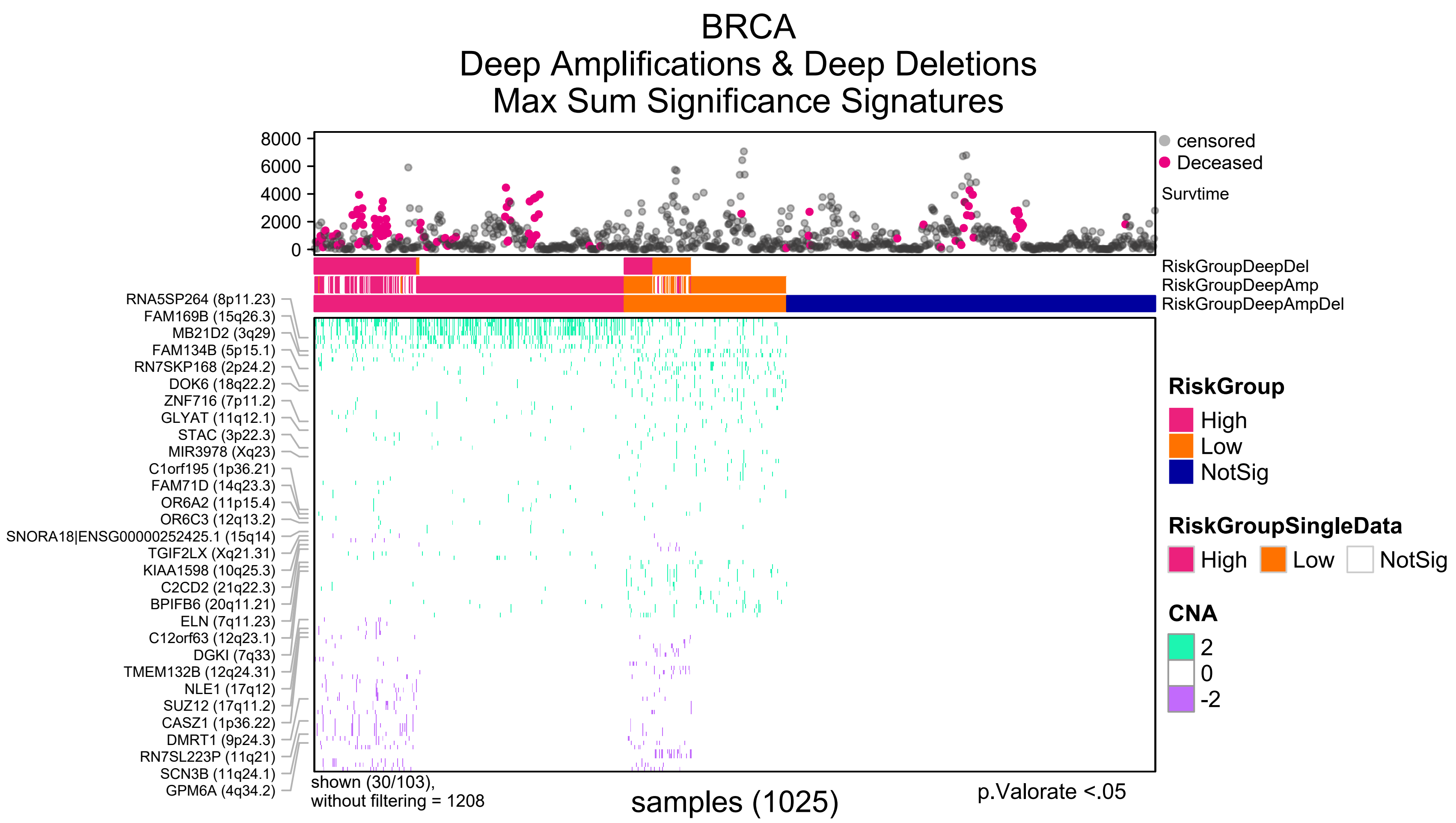
BRCA
Deep Amplifications & Deep Deletions
Max Sum Significance Signatures
8000
6000
4000
2000
0
censored
Deceased
Survtime
RiskGroupDeepDel
RiskGroupDeepAmp
RiskGroupDeepAmpDel
RNA5SP264 (8p11.23)
FAM169B (15q26.3)
MB21D2 (3q29)
FAM134B (5p15.1)
RN7SKP168 (2p24.2)
DOK6 (18q22.2)
ZNF716 (7p11.2)
GLYAT (11q12.1)
STAC (3p22.3)
MIR3978 (Xq23)
C1orf195 (1p36.21)
FAM71D (14q23.3)
OR6A2 (11p15.4)
OR6C3 (12q13.2)
SNORA18|ENSG00000252425.1 (15q14)
TGIF2LX (Xq21.31)
KIAA1598 (10q25.3)
C2CD2 (21q22.3)
BPIFB6 (20q11.21)
ELN (7q11.23)
C12orf63 (12q23.1)
DGKI (7q33)
TMEM132B (12q24.31)
NLE1 (17q12)
SUZ12 (17q11.2)
CASZ1 (1p36.22)
DMRT1 (9p24.3)
RN7SL223P (11q21)
SCN3B (11q24.1)
GPM6A (4q34.2)
RiskGroup
High
Low
NotSig
RiskGroupSingleData
High
Low
NotSig
CNA
2
0
-2
shown (30/103),
without filtering = 1208
samples (1025)
p.Valorate <.05

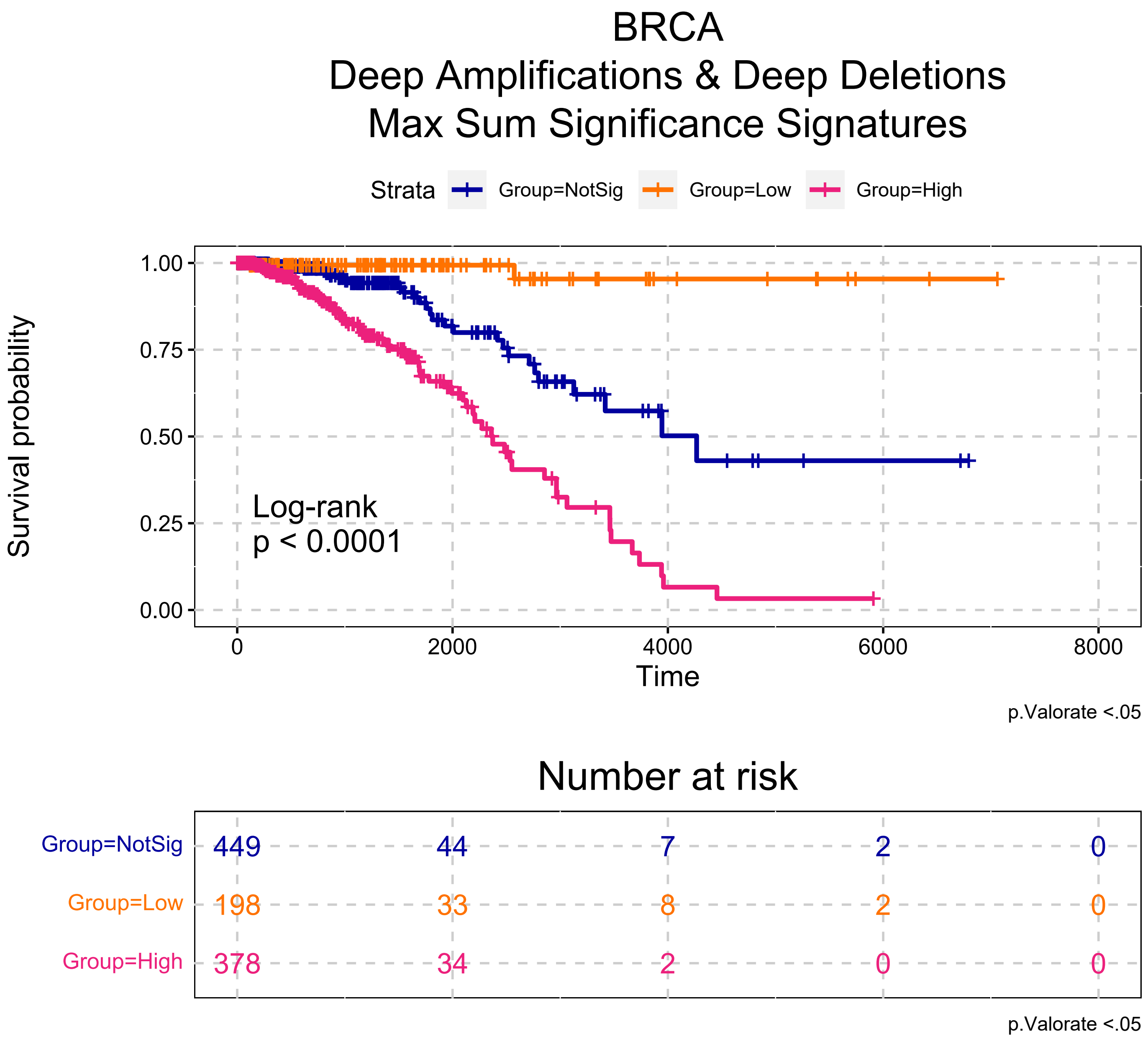

explanatory	beta	HR	L95	U95	p
Low	-2.31	0.10	0.02	0.42	0.00
High	1.17	3.21	2.08	4.97	0.00

n= 1025, number of events =101
Score(logrank) test = p <.0001

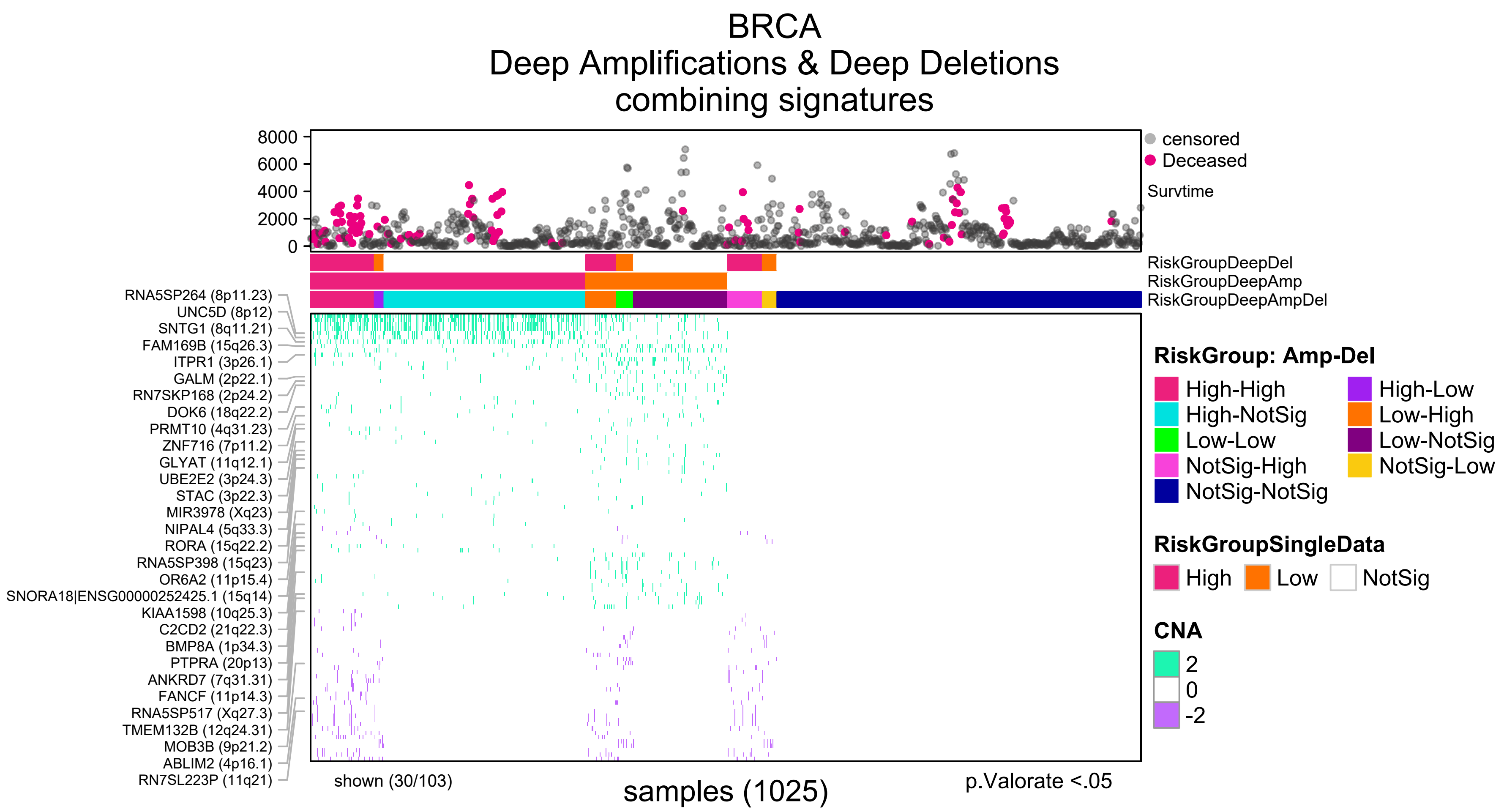

BRCA
Deep Amplifications & Deep Deletions
combining signatures
8000
6000
4000
2000
0
censored
Deceased
Survtime
RiskGroupDeepDel
RiskGroupDeepAmp
RiskGroupDeepAmpDel
RNA5SP264 (8p11.23)
UNC5D (8p12)
SNTG1 (8q11.21)
FAM169B (15q26.3)
ITPR1 (3p26.1)
GALM (2p22.1)
RN7SKP168 (2p24.2)
DOK6 (18q22.2)
PRMT10 (4q31.23)
ZNF716 (7p11.2)
GLYAT (11q12.1)
UBE2E2 (3p24.3)
STAC (3p22.3)
MIR3978 (Xq23)
NIPAL4 (5q33.3)
RORA (15q22.2)
RNA5SP398 (15q23)
OR6A2 (11p15.4)
SNORA18|ENSG00000252425.1 (15q14)
KIAA1598 (10q25.3)
C2CD2 (21q22.3)
BMP8A (1p34.3)
PTPRA (20p13)
ANKRD7 (7q31.31)
FANCF (11p14.3)
RNA5SP517 (Xq27.3)
TMEM132B (12q24.31)
MOB3B (9p21.2)
ABLIM2 (4p16.1)
RN7SL223P (11q21)
RiskGroup: Amp-Del
High-High
High-Low
High-NotSig
Low-High
Low-Low
Low-NotSig
NotSig-High
NotSig-Low
NotSig-NotSig
RiskGroupSingleData
High
Low
NotSig
CNA
2
0
-2
shown (30/103)
samples (1025)
p.Valorate <.05

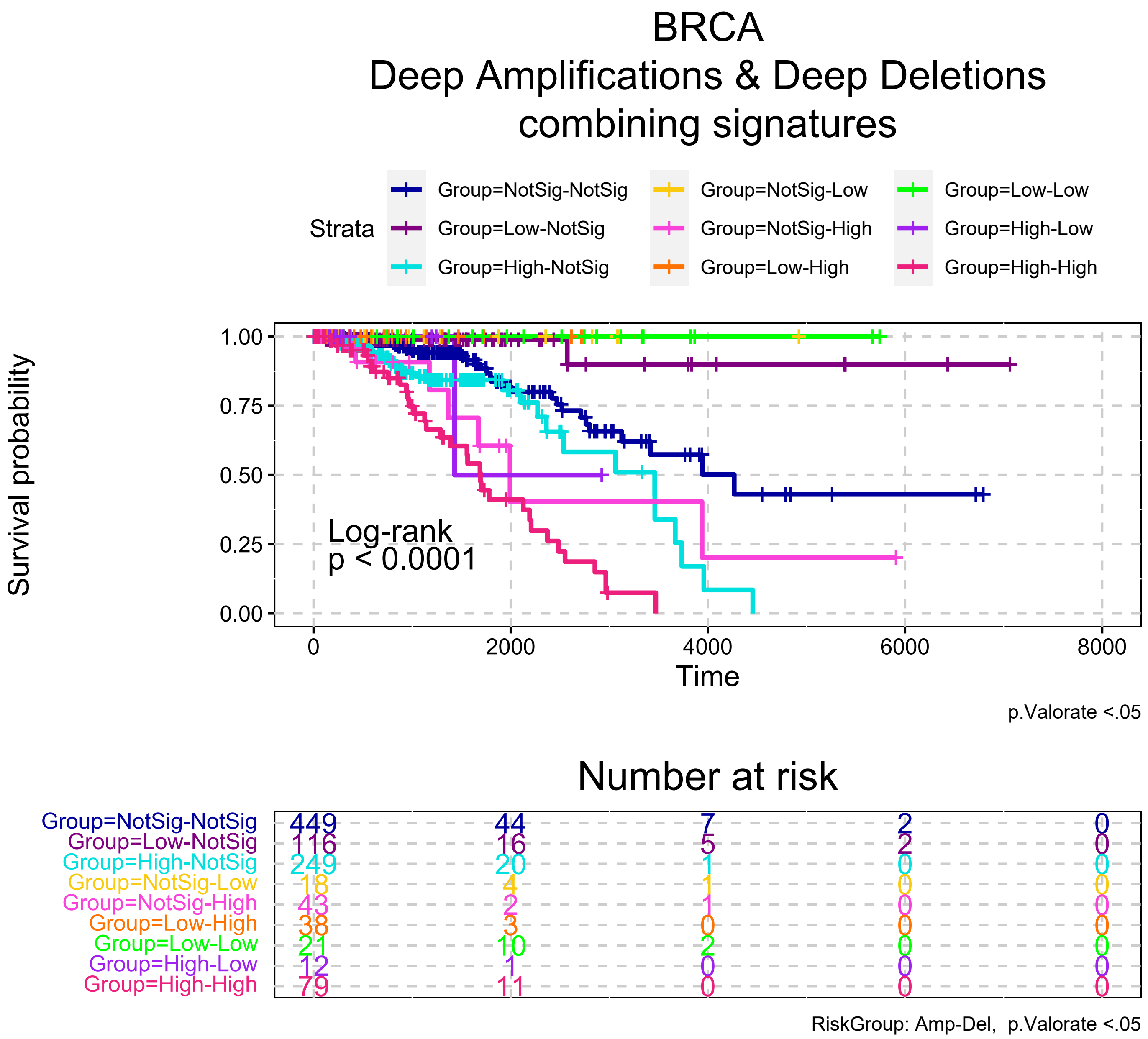

explanatory	beta	HR	L95	U95	p
Low-NotSig	-1.70	0.18	0.04	0.77	0.02
High-NotSig	0.80	2.22	1.32	3.73	0.00
NotSig-Low	-17.68	0.00	0.00	Inf	1.00
NotSig-High	0.96	2.62	1.14	6.01	0.02
Low-High	-17.85	0.00	0.00	Inf	1.00
Low-Low	-17.69	0.00	0.00	Inf	1.00
High-Low	0.54	1.72	0.23	12.70	0.59
High-High	1.81	6.08	3.65	10.13	0.00

n= 1025, number of events =101
Score(logrank) test = p <.0001
