# Supplementary material for: Systematic Modeling of Risk-Associated Copy Number Alterations in Cancer
Source: Int J Mol Sci. 2024 Sep 27;25(19):10455. doi: 10.3390/ijms251910455 (PMC11477427; doi:10.3390/ijms251910455)
Supplement: Supplementary file 1 [file ijms-25-10455-s001.zip › CESCSignatureV12-sinSombreado.pdf]

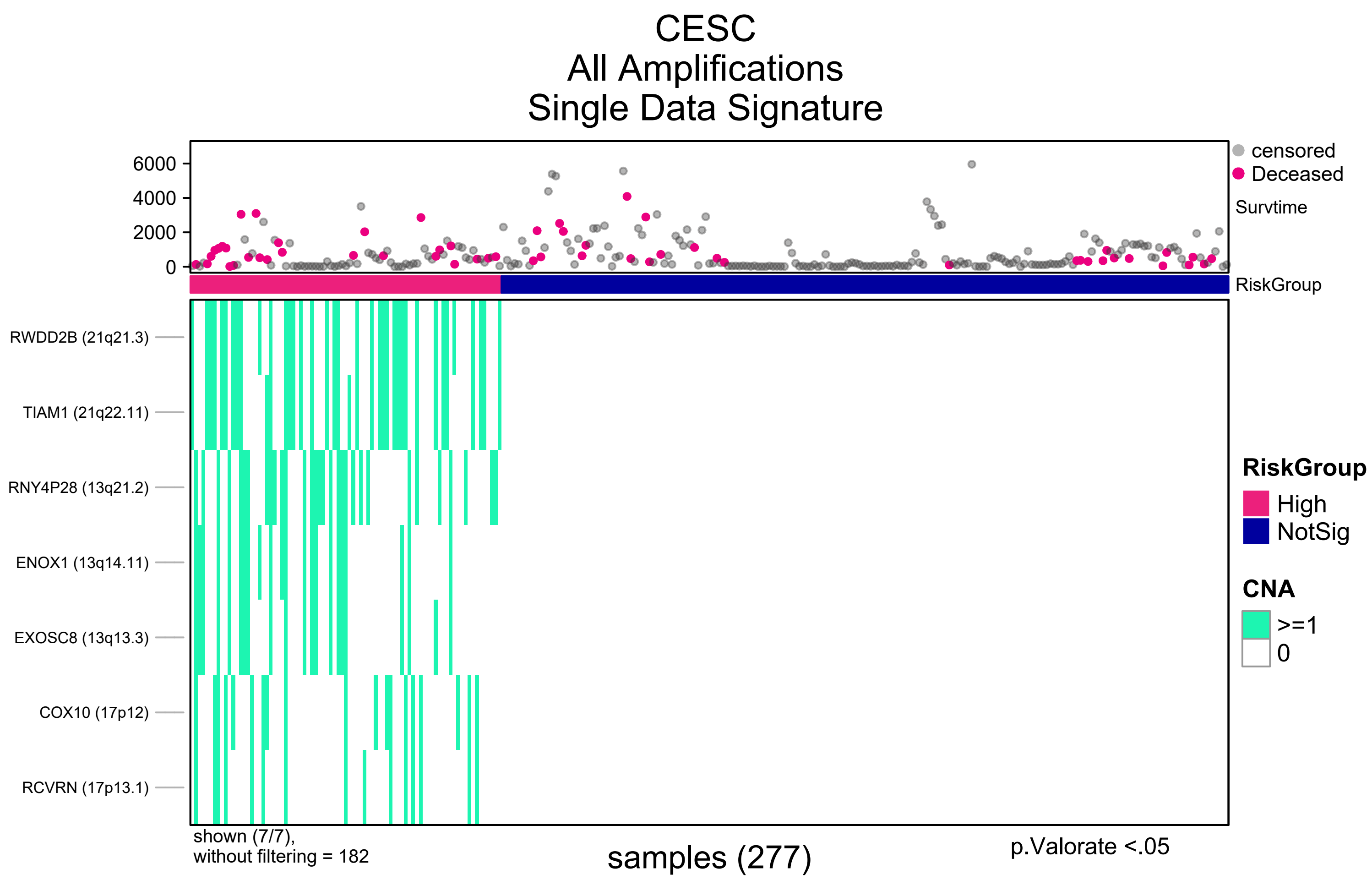

CESC
All Amplifications
Single Data Signature
6000
4000
2000
0
censored
Deceased
Survtime
RiskGroup
RWDD2B (21q21.3)
TIAM1 (21q22.11)
RNY4P28 (13q21.2)
ENOX1 (13q14.11)
EXOSC8 (13q13.3)
COX10 (17p12)
RCVRN (17p13.1)
RiskGroup
High
NotSig
CNA
>=1
0
shown (7/7),
without filtering = 182
samples (277)
p.Valorate <.05

CESC
All Amplifications
Single Data Signature

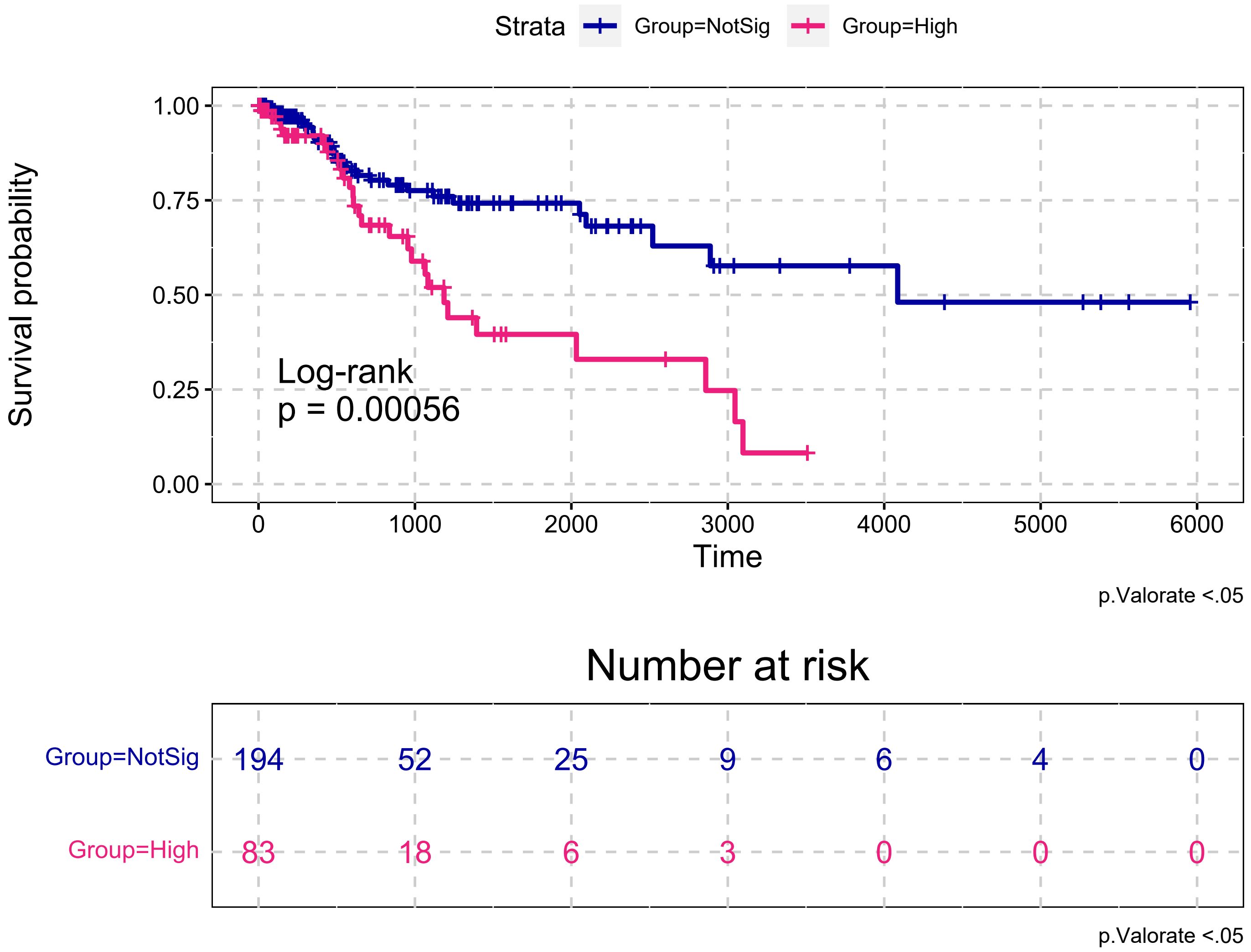

explanatory	beta	HR	L95	U95	p
High	0.91	2.48	1.45	4.21	0.00

n= 277, number of events =56
Score(logrank) test = 0.001

CESC
All Deletions
Single Data Signature

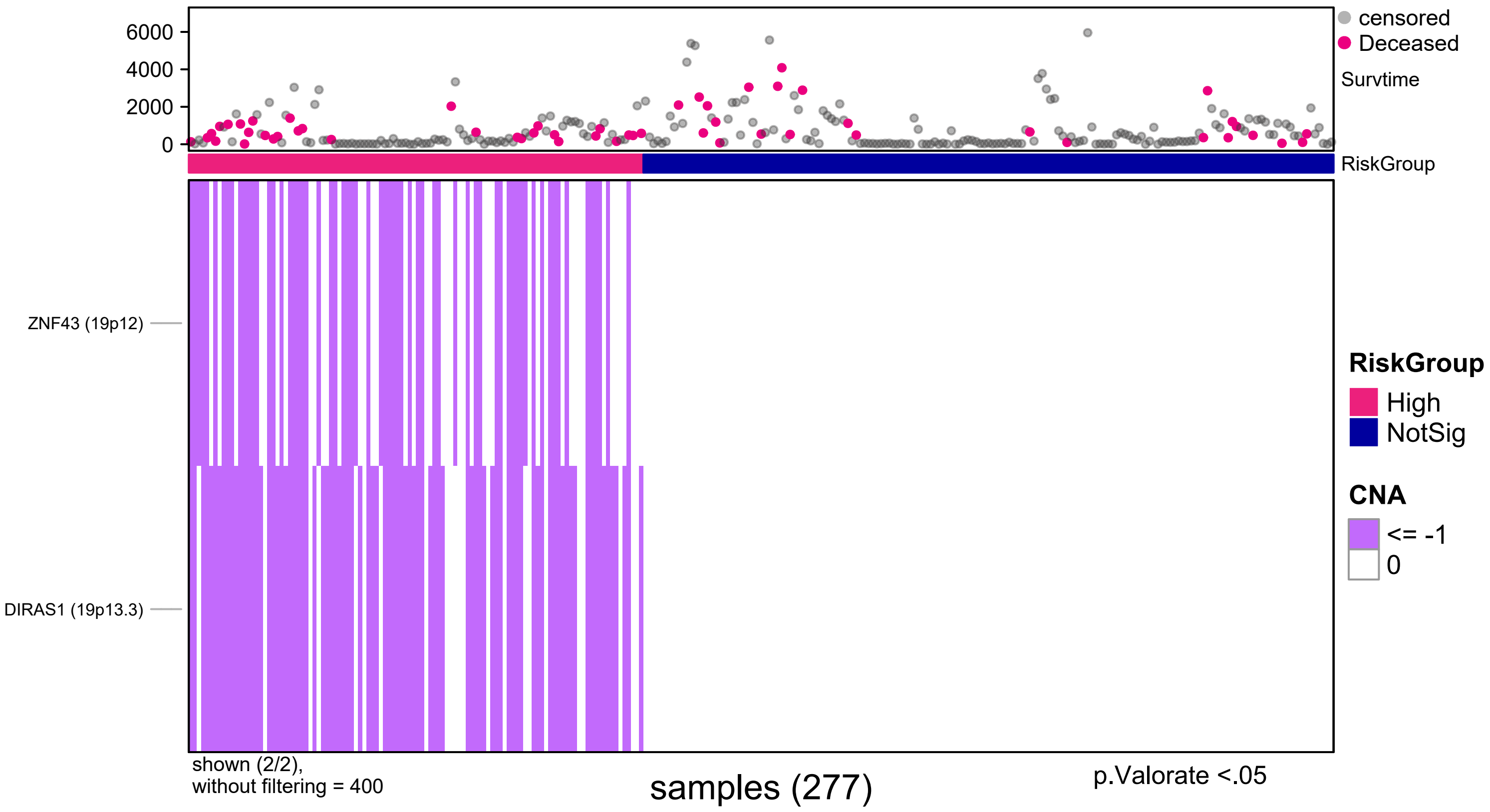

CESC
All Deletions
Single Data Signature

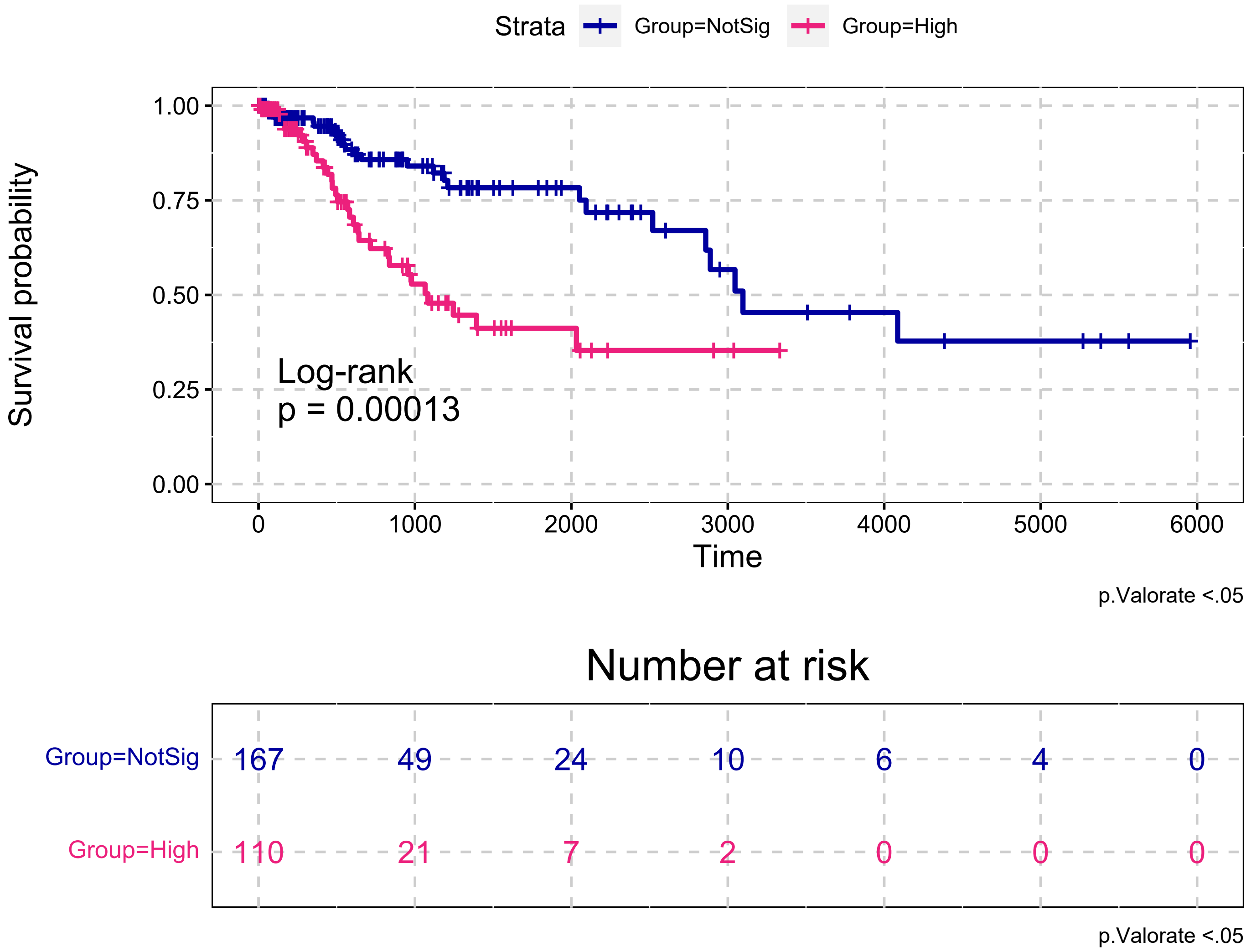

explanatory	beta	HR	L95	U95	p
High	1.02	2.78	1.61	4.78	0.00

n= 277, number of events =56
Score(logrank) test = 0

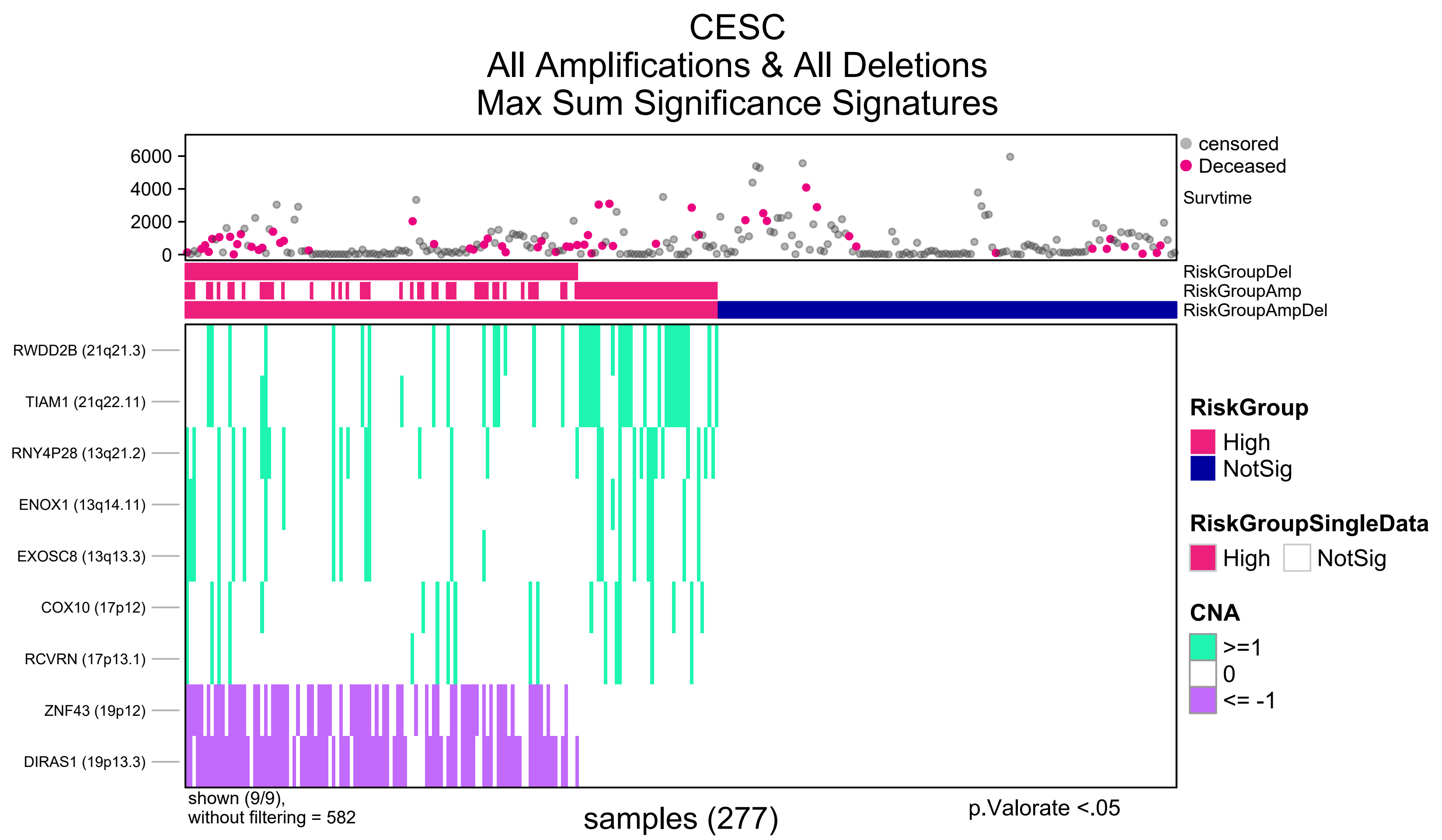
CESC
All Amplifications & All Deletions
Max Sum Significance Signatures
6000
4000
2000
0
censored
Deceased
Survtime
RiskGroupDel
RiskGroupAmp
RiskGroupAmpDel
RWDD2B (21q21.3)
TIAM1 (21q22.11)
RNY4P28 (13q21.2)
ENOX1 (13q14.11)
EXOSC8 (13q13.3)
COX10 (17p12)
RCVRN (17p13.1)
ZNF43 (19p12)
DIRAS1 (19p13.3)
RiskGroup
High
NotSig
RiskGroupSingleData
High
NotSig
CNA
>=1
0
<= -1
shown (9/9),
without filtering = 582
samples (277)
p.Valorate <.05

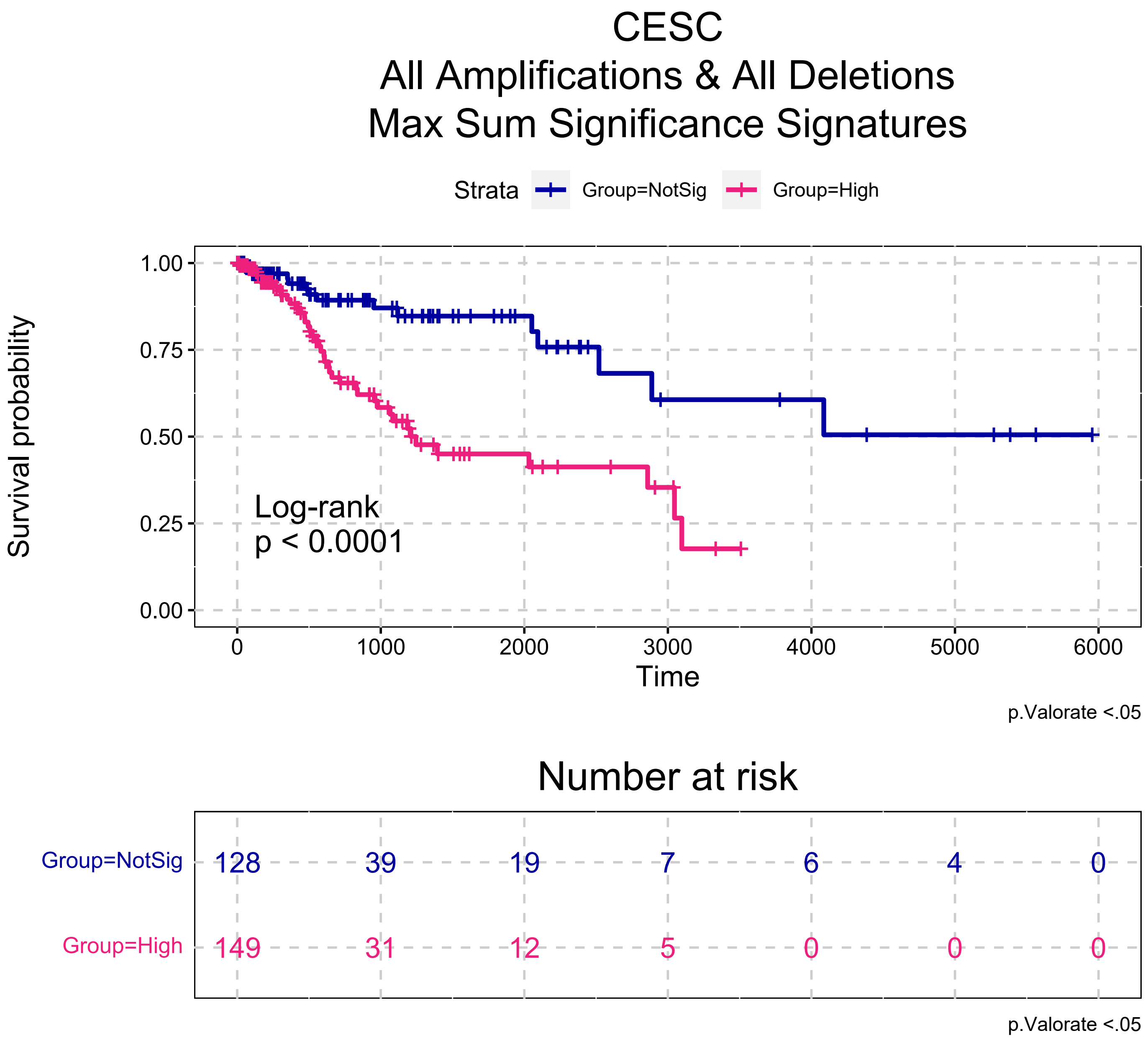

explanatory	beta	HR	L95	U95	p
High	1.19	3.28	1.78	6.03	0.00

n= 277, number of events =56
Score(logrank) test = p <.0001

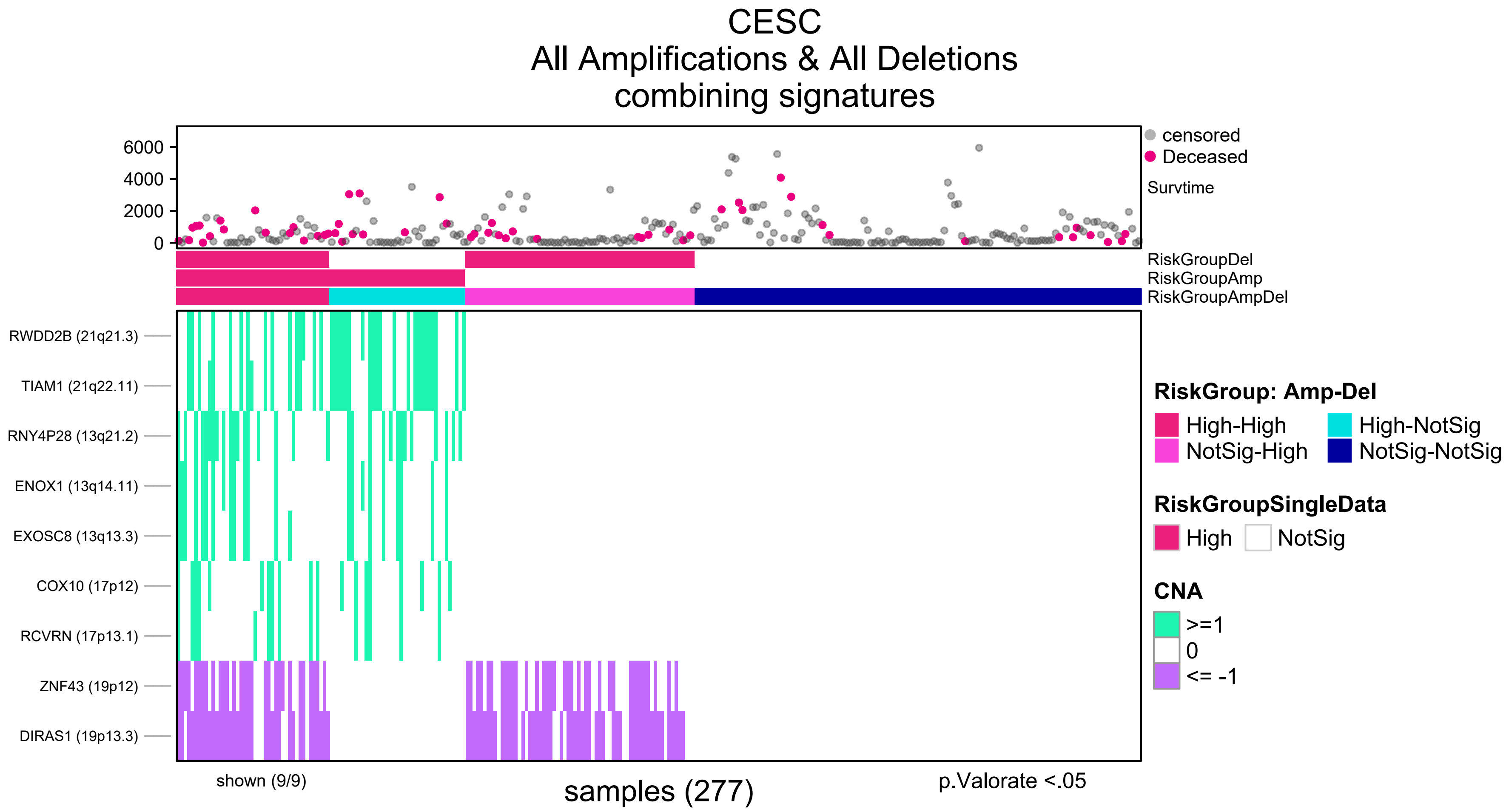

CESC
All Amplifications & All Deletions
combining signatures
6000
4000
2000
0
censored
Deceased
Survtime
RiskGroupDel
RiskGroupAmp
RiskGroupAmpDel
RWDD2B (21q21.3)
TIAM1 (21q22.11)
RNY4P28 (13q21.2)
ENOX1 (13q14.11)
EXOSC8 (13q13.3)
COX10 (17p12)
RCVRN (17p13.1)
ZNF43 (19p12)
DIRAS1 (19p13.3)
RiskGroup: Amp-Del
High-High
High-NotSig
NotSig-High
NotSig-NotSig
RiskGroupSingleData
High
NotSig
CNA
>=1
0
<= -1
shown (9/9)
samples (277)
p.Valorate <.05

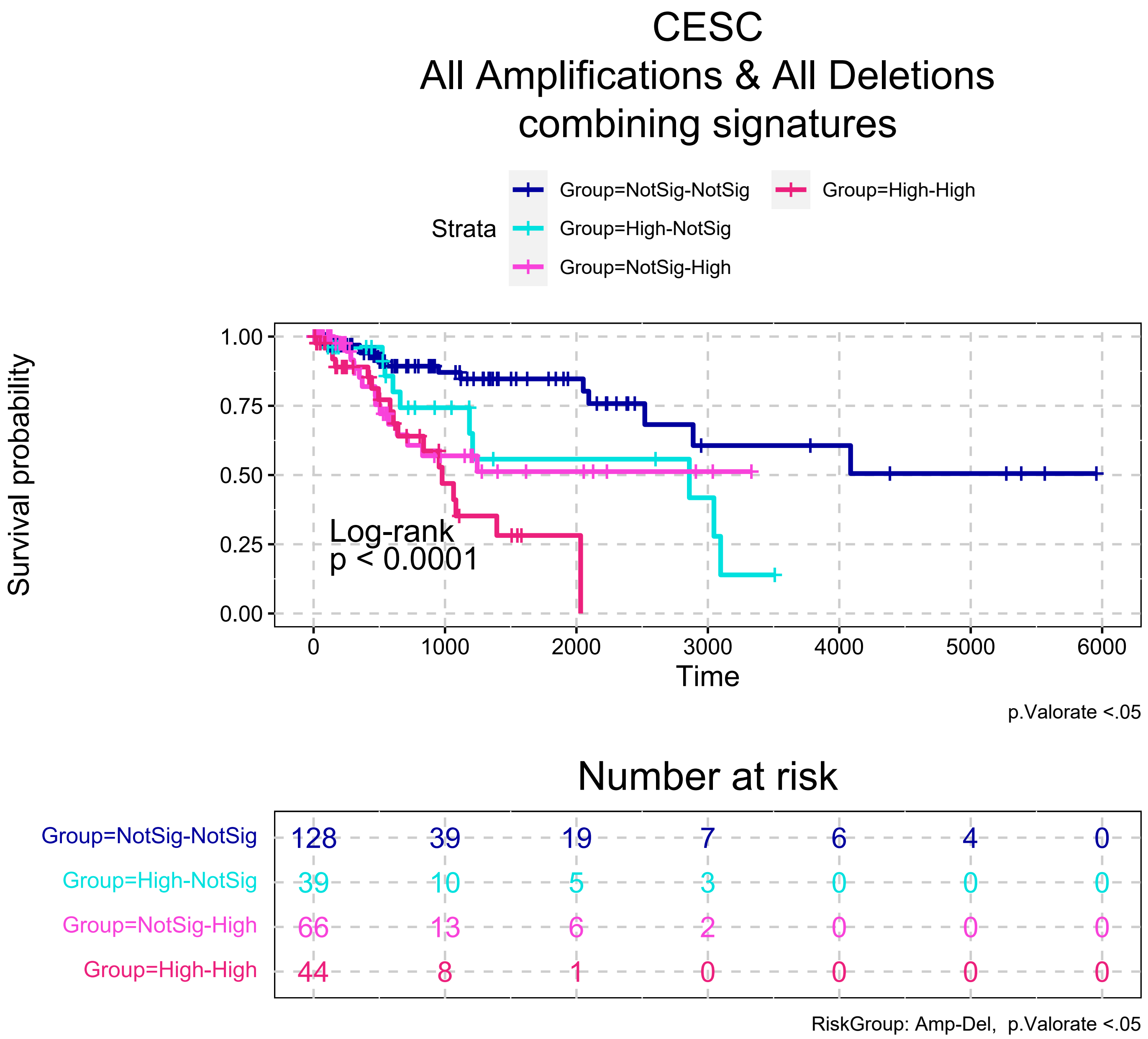

explanatory	beta	HR	L95	U95	p
High-NotSig	0.91	2.47	1.10	5.58	0.03
NotSig-High	1.02	2.78	1.32	5.86	0.01
High-High	1.65	5.21	2.51	10.81	0.00

n= 277, number of events =56
Score(logrank) test = p <.0001

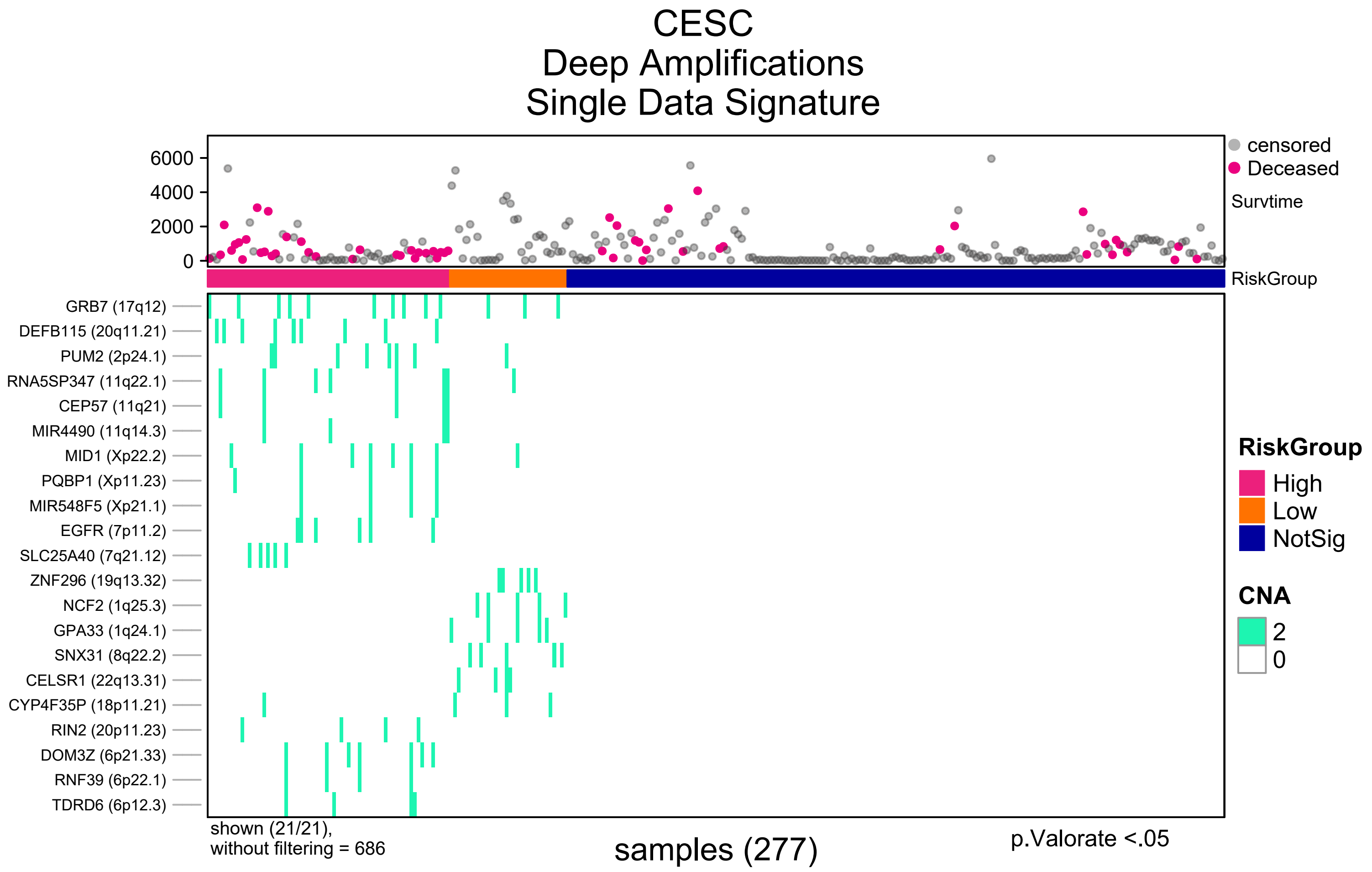

CESC
Deep Amplifications
Single Data Signature
6000
4000
2000
0
censored
Deceased
Survtime
RiskGroup
GRB7 (17q12)
DEFB115 (20q11.21)
PUM2 (2p24.1)
RNA5SP347 (11q22.1)
CEP57 (11q21)
MIR4490 (11q14.3)
MID1 (Xp22.2)
PQBP1 (Xp11.23)
MIR548F5 (Xp21.1)
EGFR (7p11.2)
SLC25A40 (7q21.12)
ZNF296 (19q13.32)
NCF2 (1q25.3)
GPA33 (1q24.1)
SNX31 (8q22.2)
CELSR1 (22q13.31)
CYP4F35P (18p11.21)
RIN2 (20p11.23)
DOM3Z (6p21.33)
RNF39 (6p22.1)
TDRD6 (6p12.3)
RiskGroup
High
Low
NotSig
CNA
2
0
shown (21/21),
without filtering = 686
samples (277)
p.Valorate <.05

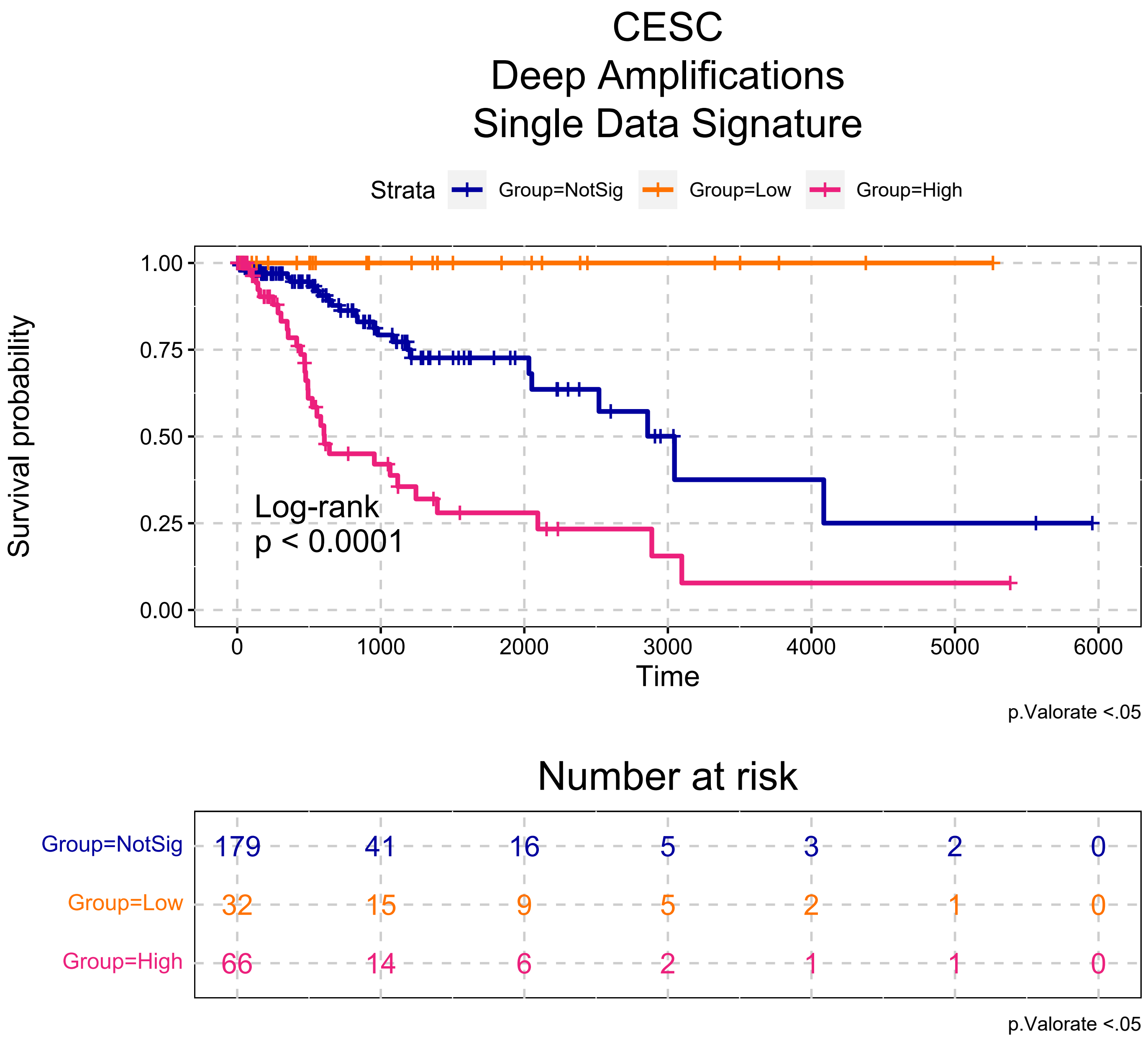

explanatory	beta	HR	L95	U95	p
Low	-18.34	0.00	0.00	Inf	1.00
High	1.24	3.45	2.03	5.86	0.00

n= 277, number of events =56
Score(logrank) test = p <.0001

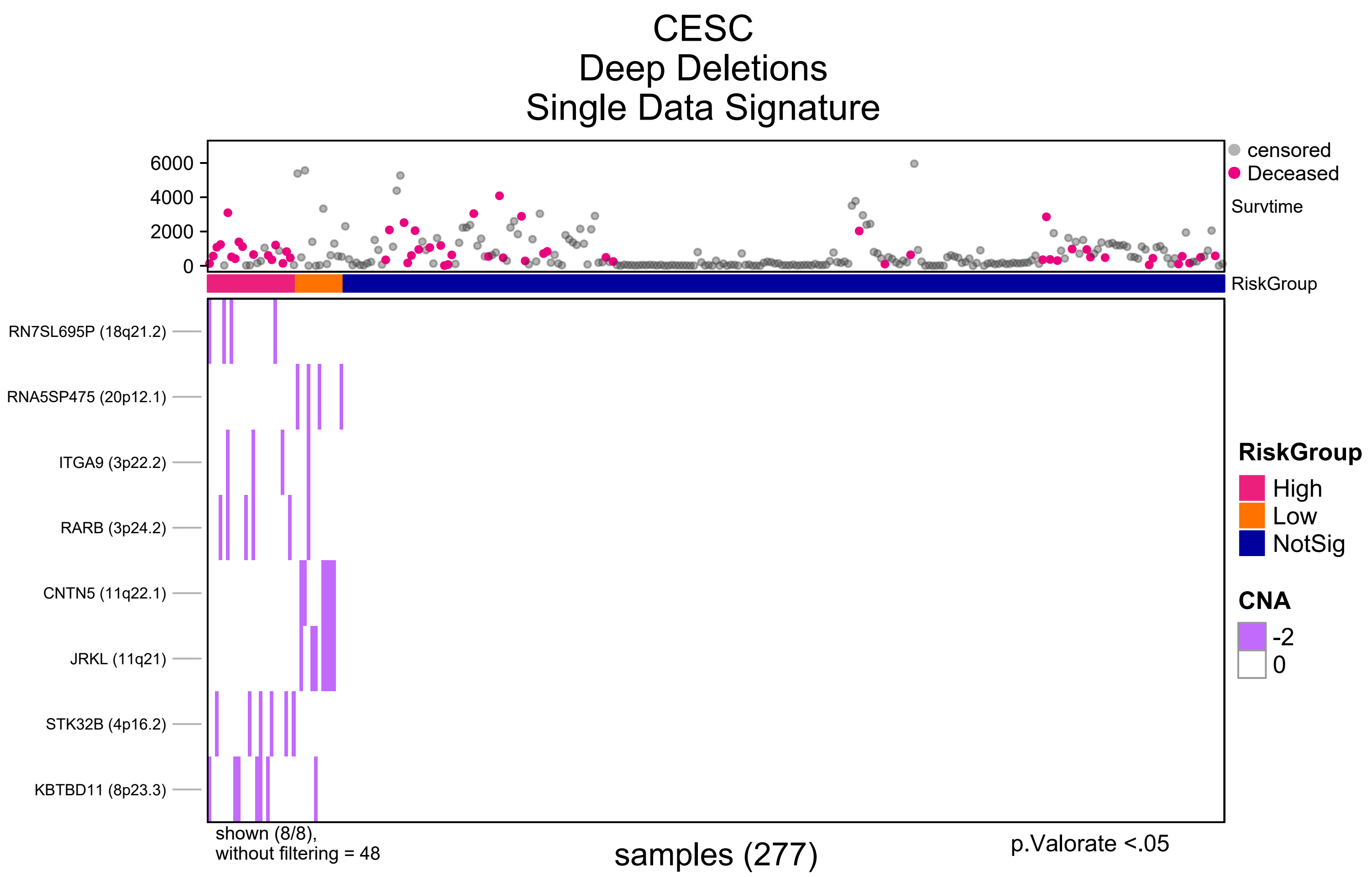

CESC
Deep Deletions
Single Data Signature
6000
4000
2000
0
censored
Deceased
Survtime
RiskGroup
RN7SL695P (18q21.2)
RNA5SP475 (20p12.1)
ITGA9 (3p22.2)
RARB (3p24.2)
CNTN5 (11q22.1)
JRKL (11q21)
STK32B (4p16.2)
KBTBD11 (8p23.3)
RiskGroup
High
Low
NotSig
CNA
-2
0
shown (8/8),
without filtering = 48
samples (277)
p.Valorate <.05

CESC
Deep Deletions
Single Data Signature

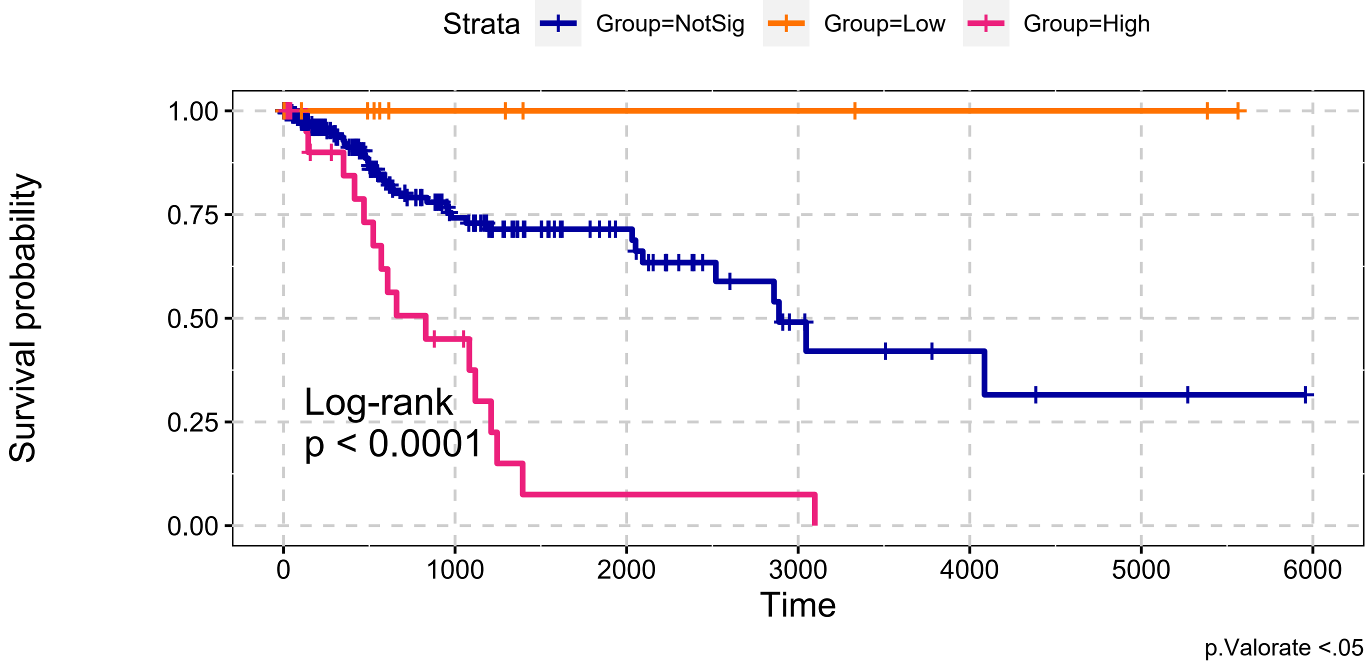

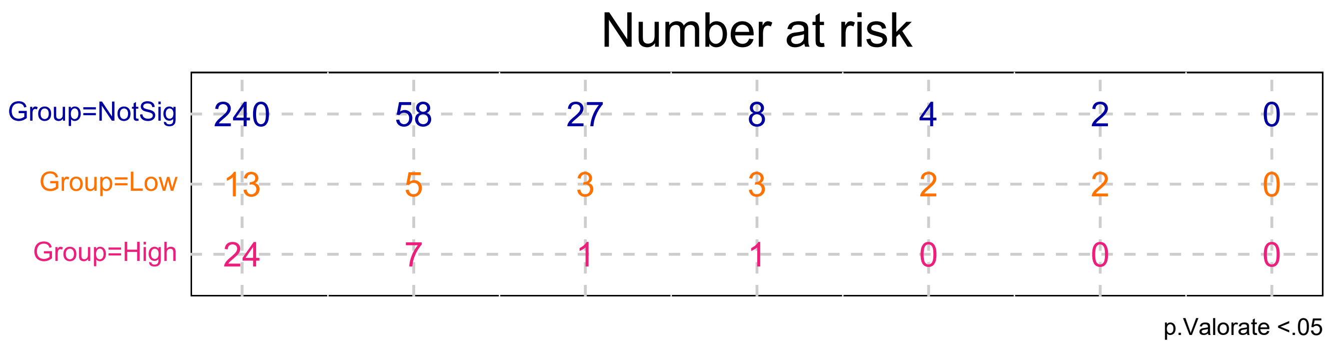

explanatory	beta	HR	L95	U95	p
Low	-17.41	0.00	0.00	Inf	1.00
High	1.30	3.66	2.04	6.58	0.00

n= 277, number of events =56
Score(logrank) test = p <.0001

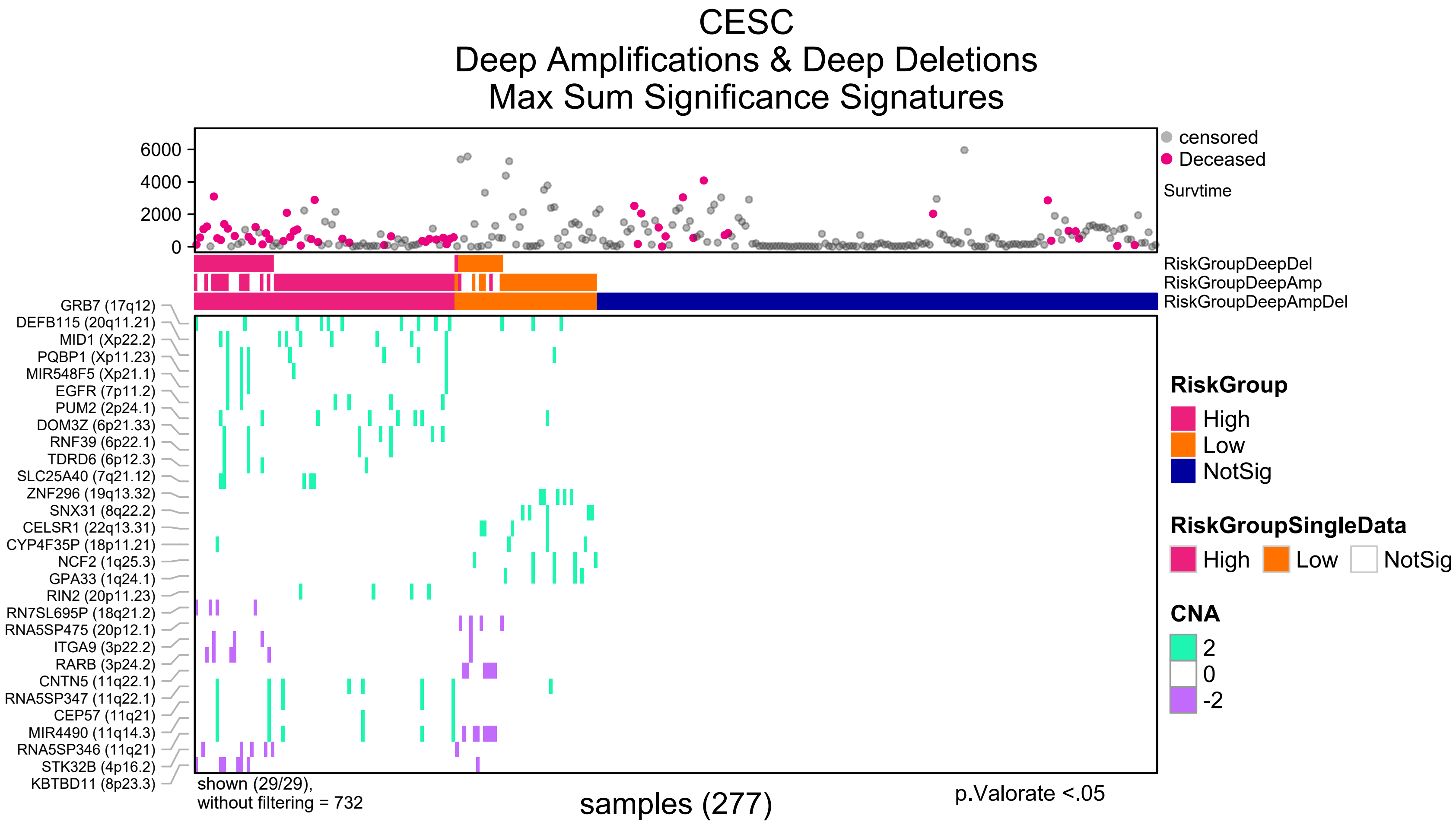
CESC
Deep Amplifications & Deep Deletions
Max Sum Significance Signatures
6000
4000
2000
0
censored
Deceased
Survtime
RiskGroupDeepDel
RiskGroupDeepAmp
RiskGroupDeepAmpDel
GRB7 (17q12)
DEFB115 (20q11.21)
MID1 (Xp22.2)
PQBP1 (Xp11.23)
MIR548F5 (Xp21.1)
EGFR (7p11.2)
PUM2 (2p24.1)
DOM3Z (6p21.33)
RNF39 (6p22.1)
TDRD6 (6p12.3)
SLC25A40 (7q21.12)
ZNF296 (19q13.32)
SNX31 (8q22.2)
CELSR1 (22q13.31)
CYP4F35P (18p11.21)
NCF2 (1q25.3)
GPA33 (1q24.1)
RIN2 (20p11.23)
RN7SL695P (18q21.2)
RNA5SP475 (20p12.1)
ITGA9 (3p22.2)
RARB (3p24.2)
CNTN5 (11q22.1)
RNA5SP347 (11q22.1)
CEP57 (11q21)
MIR4490 (11q14.3)
RNA5SP346 (11q21)
STK32B (4p16.2)
KBTBD11 (8p23.3)
RiskGroup
High
Low
NotSig
RiskGroupSingleData
High
Low
NotSig
CNA
2
0
-2
shown (29/29),
without filtering = 732
samples (277)
p.Valorate <.05

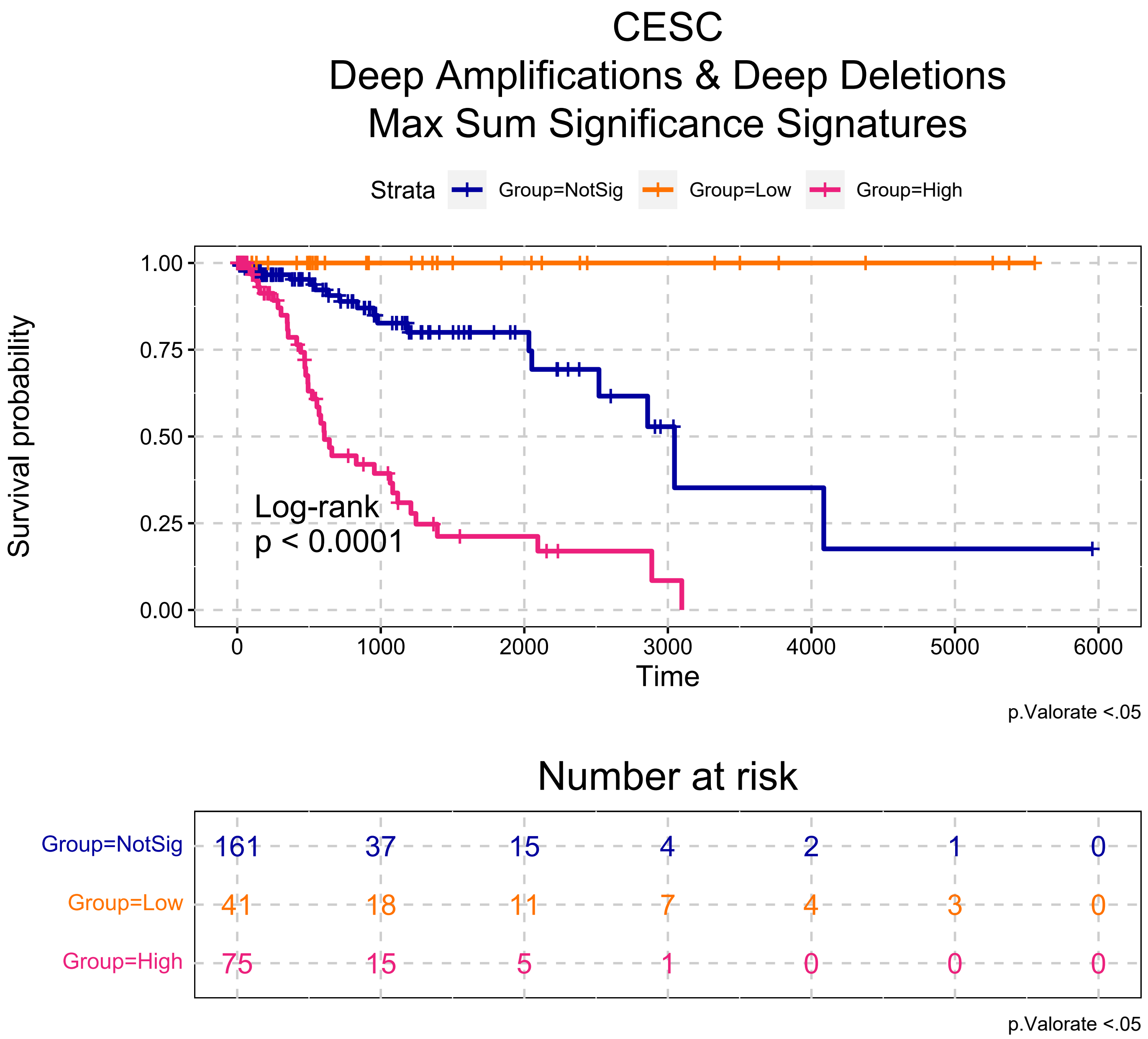

explanatory	beta	HR	L95	U95	p
Low	-18.61	0.00	0.00	Inf	1.00
High	1.59	4.89	2.77	8.61	0.00

n= 277, number of events =56
Score(logrank) test = p <.0001

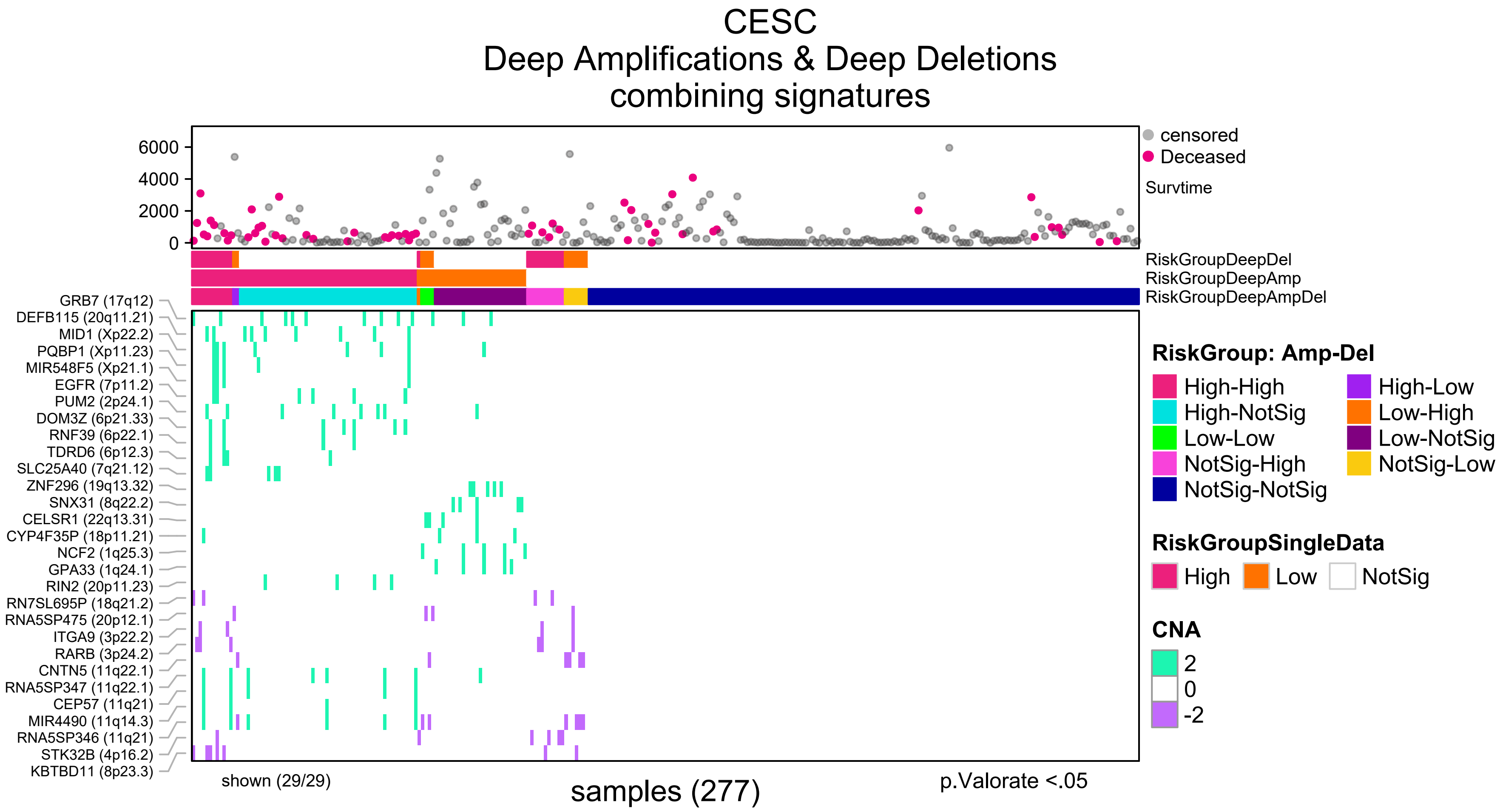

CESC
Deep Amplifications & Deep Deletions
combining signatures
6000
4000
2000
0
censored
Deceased
Survtime
RiskGroupDeepDel
RiskGroupDeepAmp
RiskGroupDeepAmpDel
GRB7 (17q12)
DEFB115 (20q11.21)
MID1 (Xp22.2)
PQBP1 (Xp11.23)
MIR548F5 (Xp21.1)
EGFR (7p11.2)
PUM2 (2p24.1)
DOM3Z (6p21.33)
RNF39 (6p22.1)
TDRD6 (6p12.3)
SLC25A40 (7q21.12)
ZNF296 (19q13.32)
SNX31 (8q22.2)
CELSR1 (22q13.31)
CYP4F35P (18p11.21)
NCF2 (1q25.3)
GPA33 (1q24.1)
RIN2 (20p11.23)
RN7SL695P (18q21.2)
RNA5SP475 (20p12.1)
ITGA9 (3p22.2)
RARB (3p24.2)
CNTN5 (11q22.1)
RNA5SP347 (11q22.1)
CEP57 (11q21)
MIR4490 (11q14.3)
RNA5SP346 (11q21)
STK32B (4p16.2)
KBTBD11 (8p23.3)
RiskGroup: Amp-Del
High-High
High-NotSig
Low-Low
NotSig-High
NotSig-NotSig
High-Low
Low-High
Low-NotSig
NotSig-Low
RiskGroupSingleData
High
Low
NotSig
CNA
2
0
-2
shown (29/29)
samples (277)
p.Valorate <.05

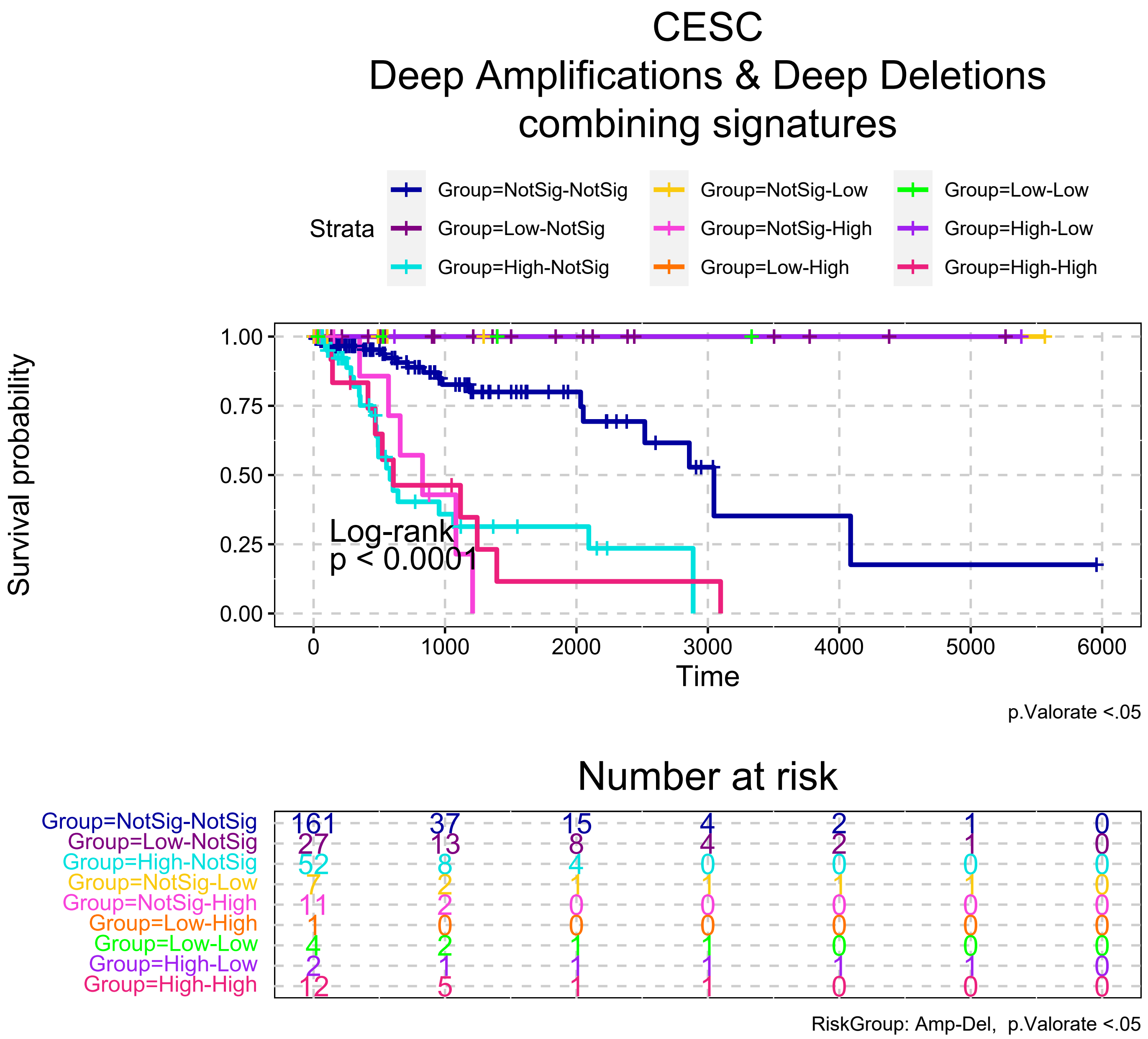

explanatory	beta	HR	L95	U95	p
Low-NotSig	-18.58	0.00	0.00	Inf	1.00
High-NotSig	1.58	4.85	2.57	9.16	0.00
NotSig-Low	-18.69	0.00	0.00	Inf	1.00
NotSig-High	1.71	5.52	2.16	14.12	0.00
Low-High	-18.17	0.00	0.00	Inf	1.00
Low-Low	-18.59	0.00	0.00	Inf	1.00
High-Low	-18.82	0.00	0.00	Inf	1.00
High-High	1.54	4.65	2.12	10.22	0.00

n= 277, number of events =56
Score(logrank) test = p <.0001
