# Supplementary material for: Systematic Modeling of Risk-Associated Copy Number Alterations in Cancer
Source: Int J Mol Sci. 2024 Sep 27;25(19):10455. doi: 10.3390/ijms251910455 (PMC11477427; doi:10.3390/ijms251910455)
Supplement: Supplementary file 1 [file ijms-25-10455-s001.zip › CHOLSignatureV12-sinSombreado.pdf]

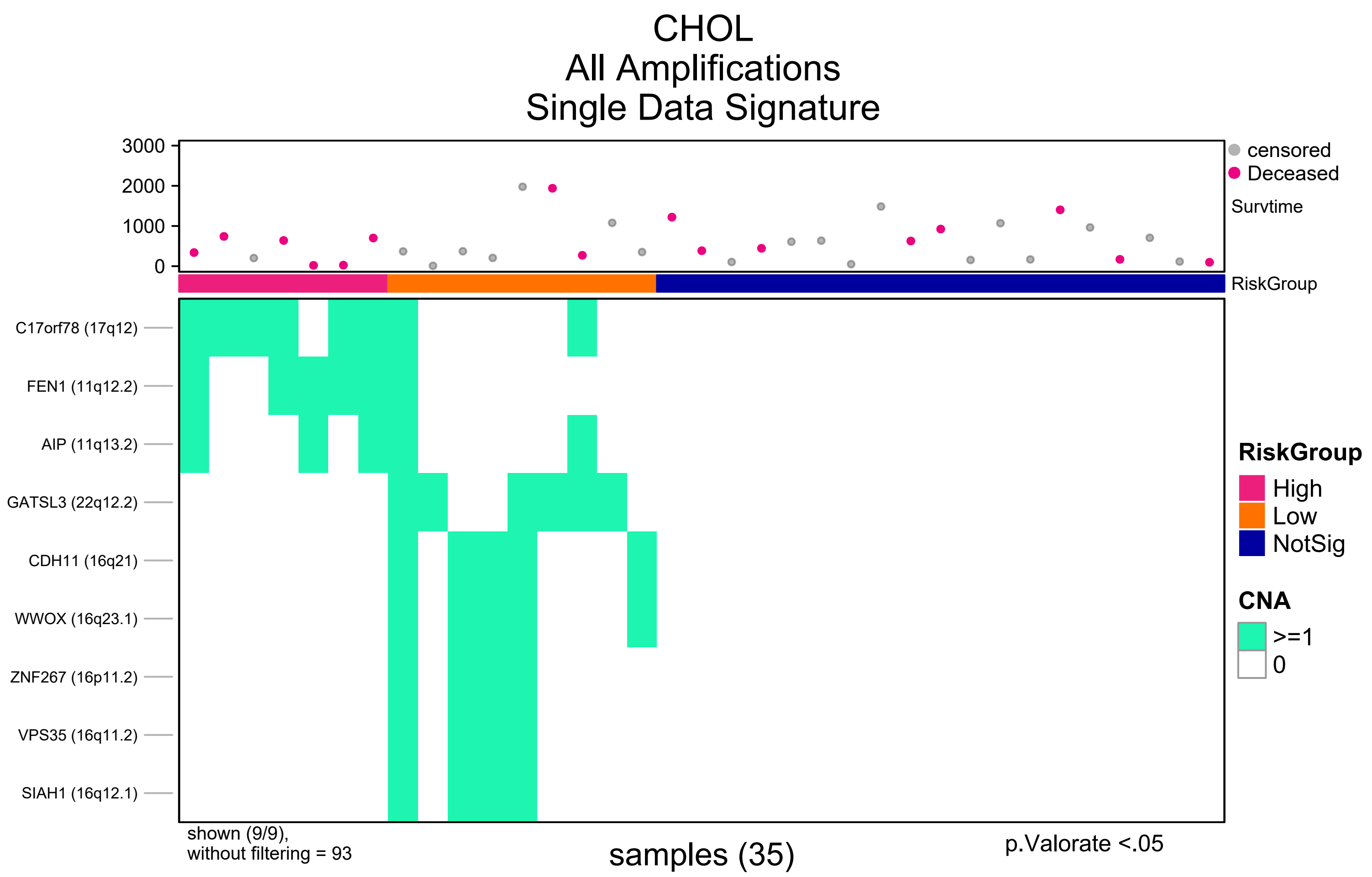
CHOL
All Amplifications
Single Data Signature
3000
2000
1000
0
censored
Deceased
Survtime
RiskGroup
C17orf78 (17q12)
FEN1 (11q12.2)
AIP (11q13.2)
GATSL3 (22q12.2)
CDH11 (16q21)
WWOX (16q23.1)
ZNF267 (16p11.2)
VPS35 (16q11.2)
SIAH1 (16q12.1)
RiskGroup
High
Low
NotSig
CNA
>=1
0
shown (9/9),
without filtering = 93
samples (35)
p.Valorate <.05

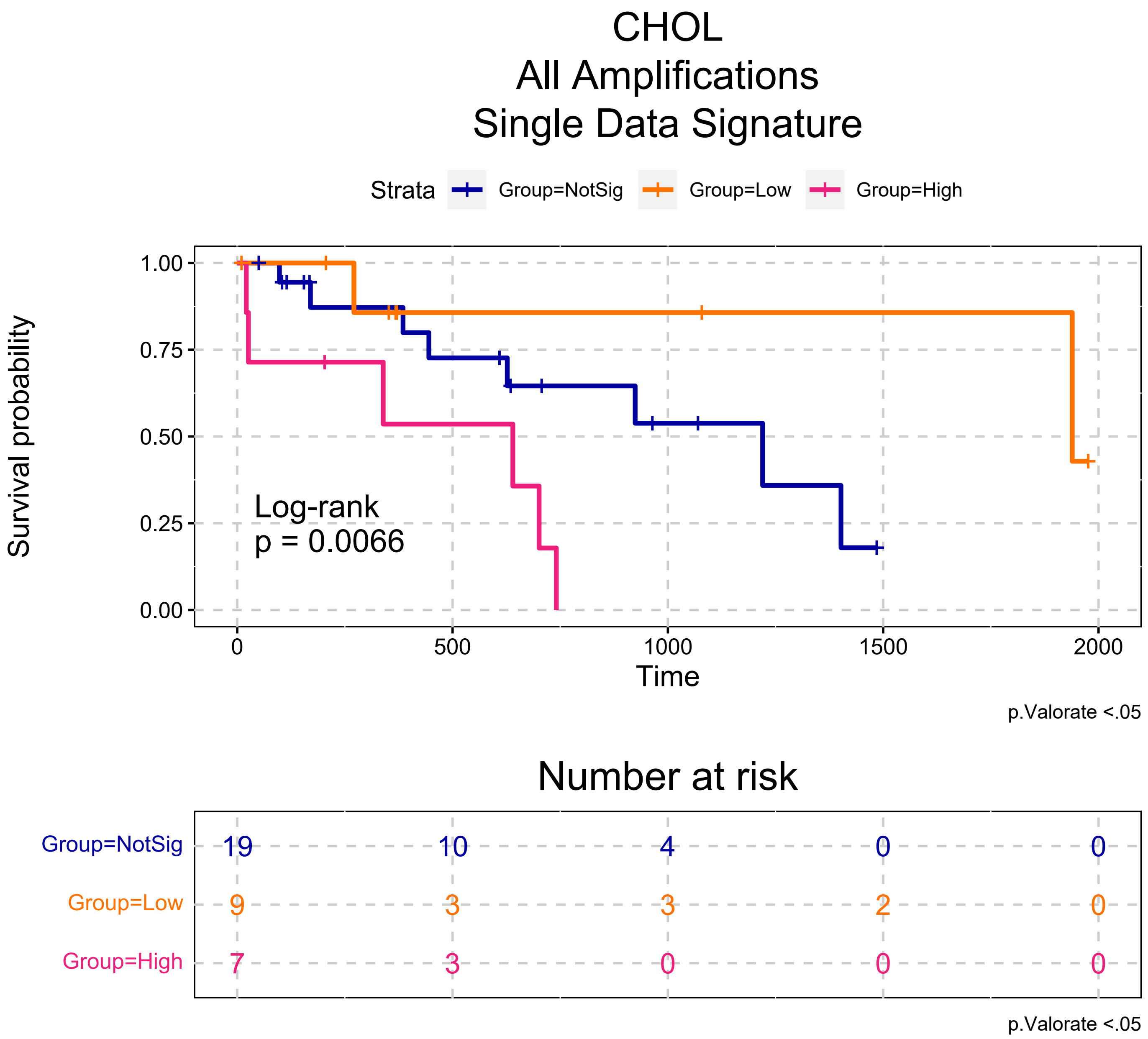

explanatory	beta	HR	L95	U95	p
Low	-1.43	0.24	0.03	1.95	0.18
High	1.29	3.62	1.14	11.49	0.03

n= 35, number of events =16
Score(logrank) test = 0.007

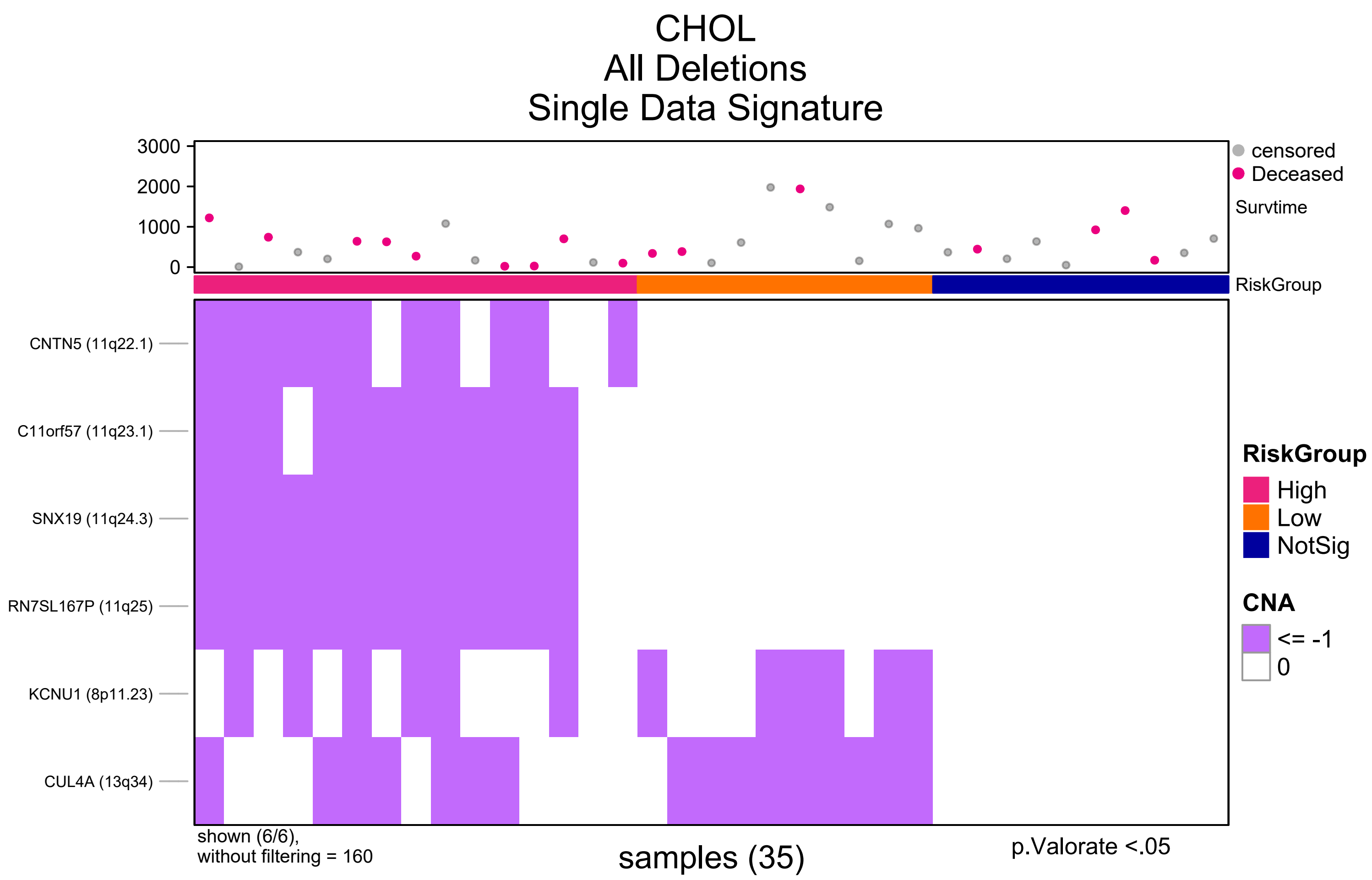
CHOL
All Deletions
Single Data Signature
3000
2000
1000
0
censored
Deceased
Survtime
RiskGroup
CNTN5 (11q22.1)
C11orf57 (11q23.1)
SNX19 (11q24.3)
RN7SL167P (11q25)
KCNU1 (8p11.23)
CUL4A (13q34)
RiskGroup
High
Low
NotSig
CNA
<= -1
0
shown (6/6),
without filtering = 160
samples (35)
p.Valorate <.05

CHOL
All Deletions
Single Data Signature

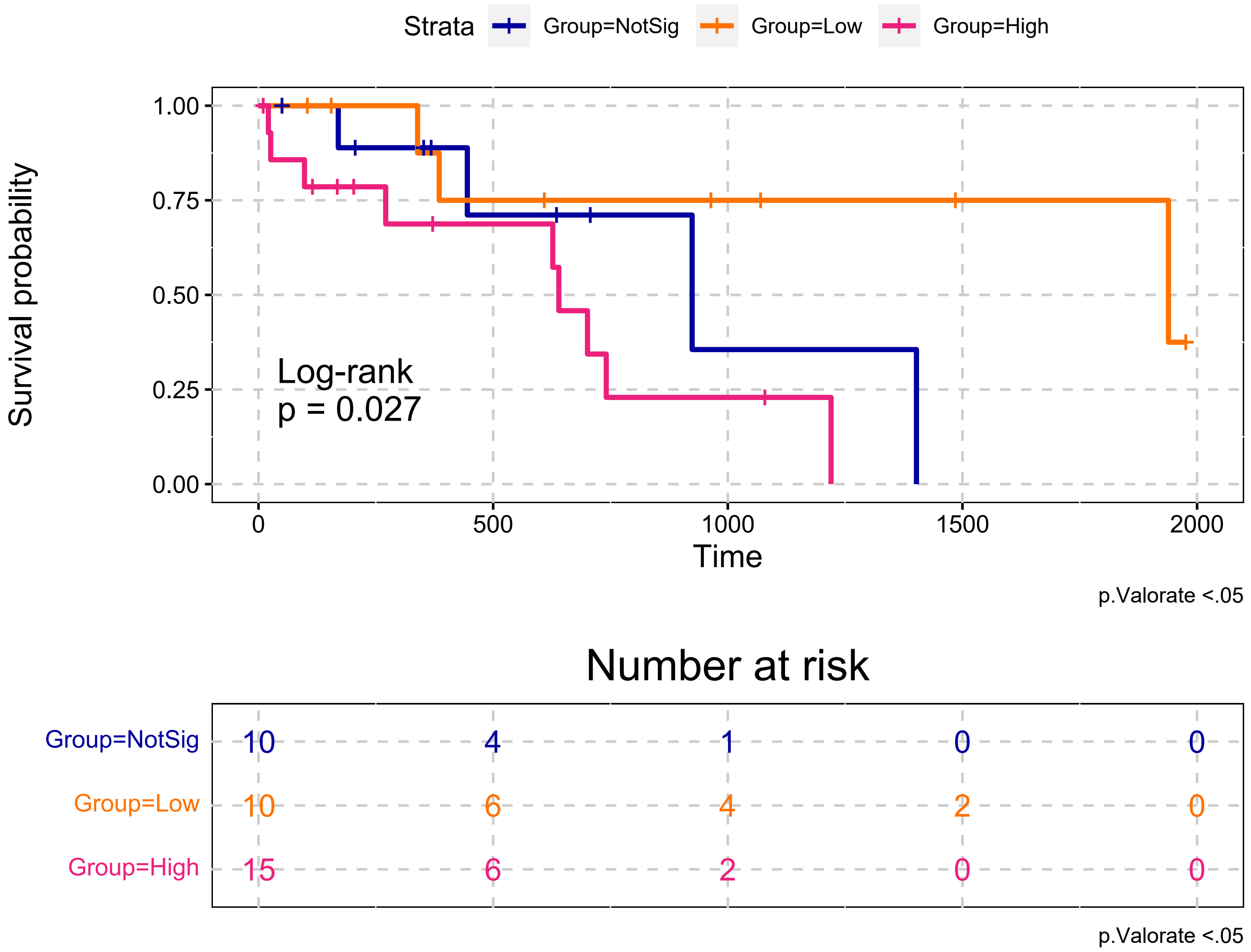

explanatory	beta	HR	L95	U95	p
Low	-1.23	0.29	0.05	1.64	0.16
High	0.74	2.10	0.62	7.09	0.23

n= 35, number of events =16
Score(logrank) test = 0.027

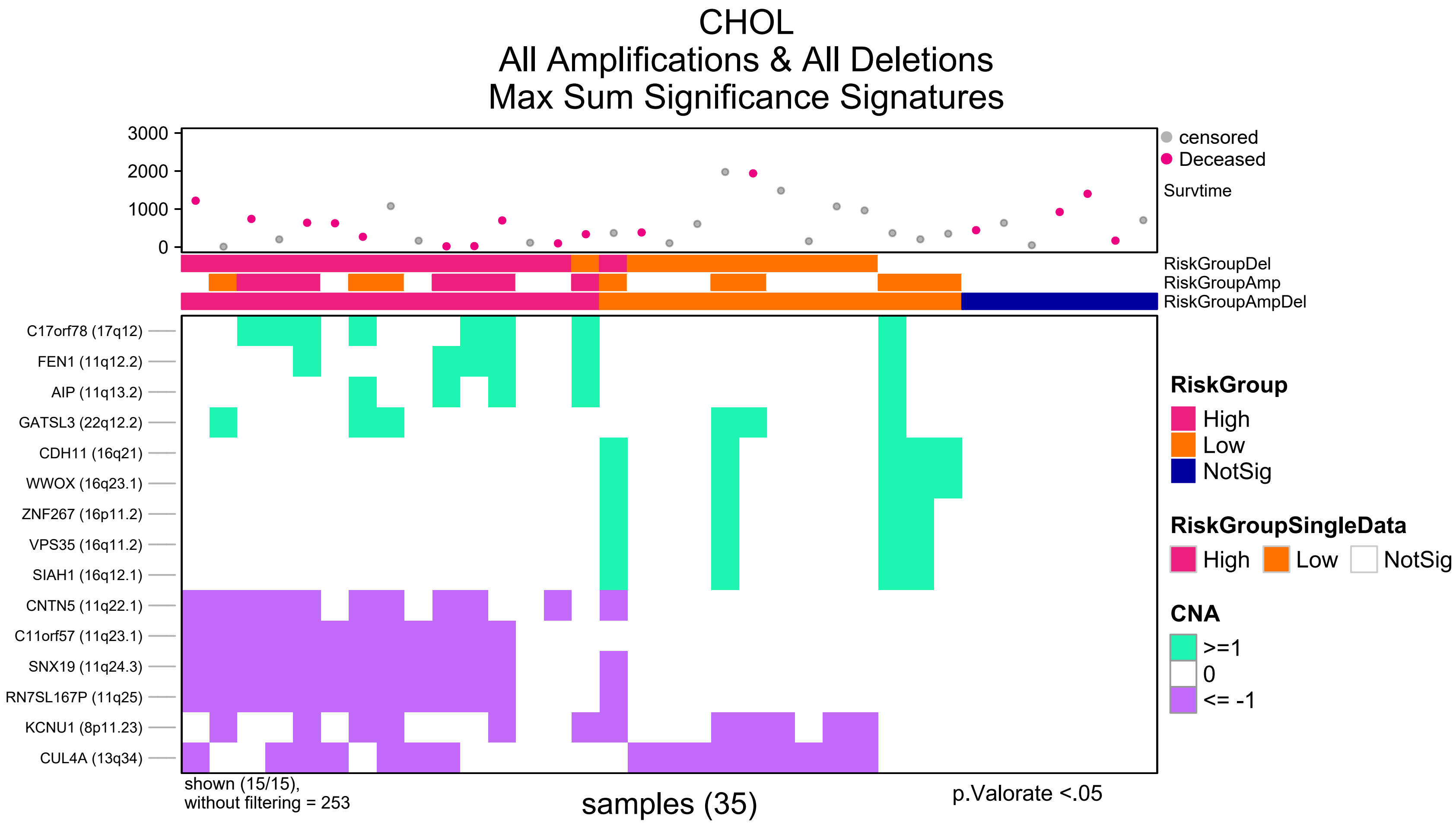
CHOL
All Amplifications & All Deletions
Max Sum Significance Signatures
3000
2000
1000
0
censored
Deceased
Survtime
RiskGroupDel
RiskGroupAmp
RiskGroupAmpDel
C17orf78 (17q12)
FEN1 (11q12.2)
AIP (11q13.2)
GATSL3 (22q12.2)
CDH11 (16q21)
WWOX (16q23.1)
ZNF267 (16p11.2)
VPS35 (16q11.2)
SIAH1 (16q12.1)
CNTN5 (11q22.1)
C11orf57 (11q23.1)
SNX19 (11q24.3)
RN7SL167P (11q25)
KCNU1 (8p11.23)
CUL4A (13q34)
RiskGroup
High
Low
NotSig
RiskGroupSingleData
High
Low
NotSig
CNA
>=1
0
<= -1
shown (15/15),
without filtering = 253
samples (35)
p.Valorate <.05

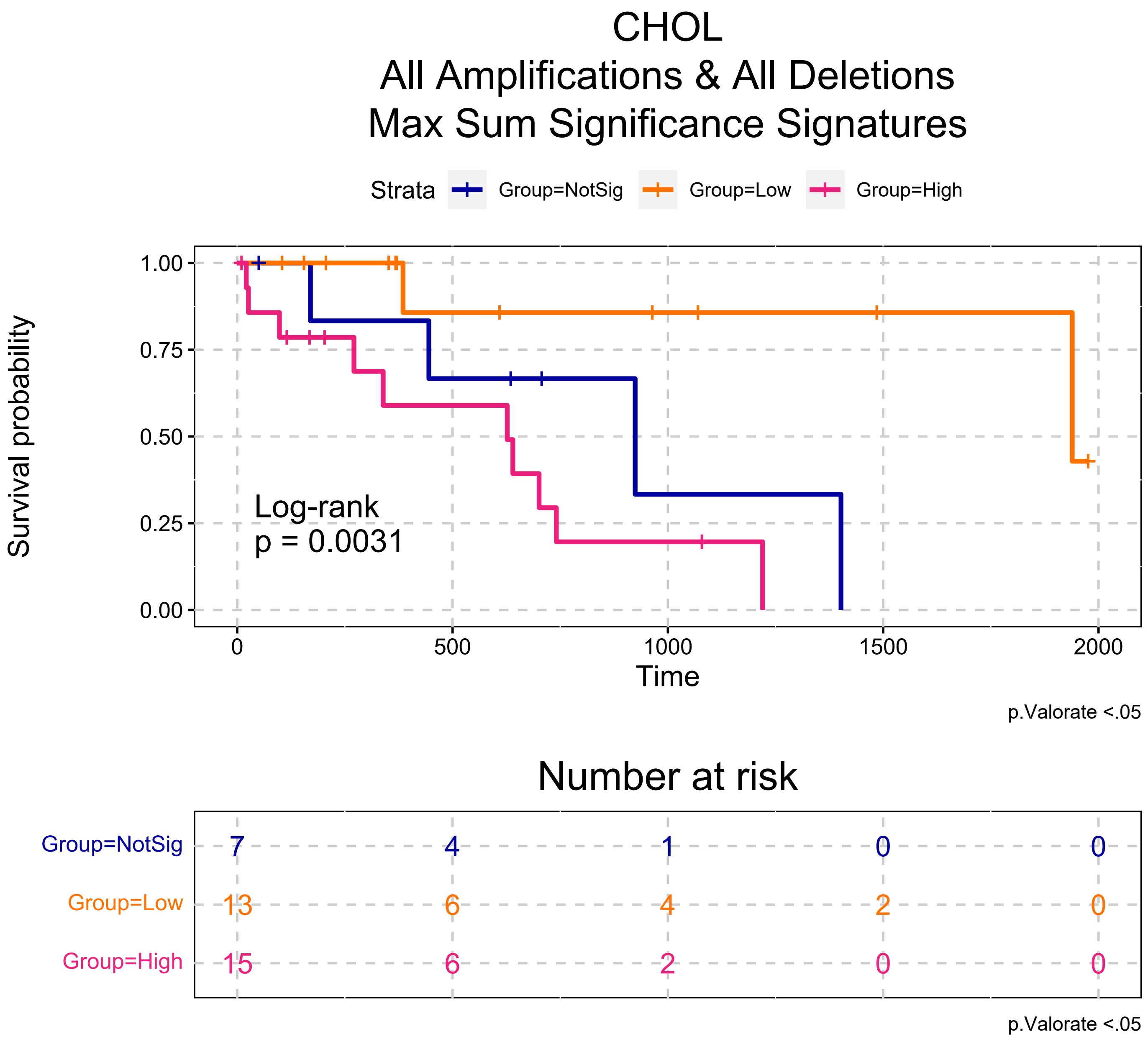

explanatory	beta	HR	L95	U95	p
Low	-2.14	0.12	0.01	1.06	0.06
High	0.76	2.14	0.62	7.34	0.23

n= 35, number of events =16
Score(logrank) test = 0.003

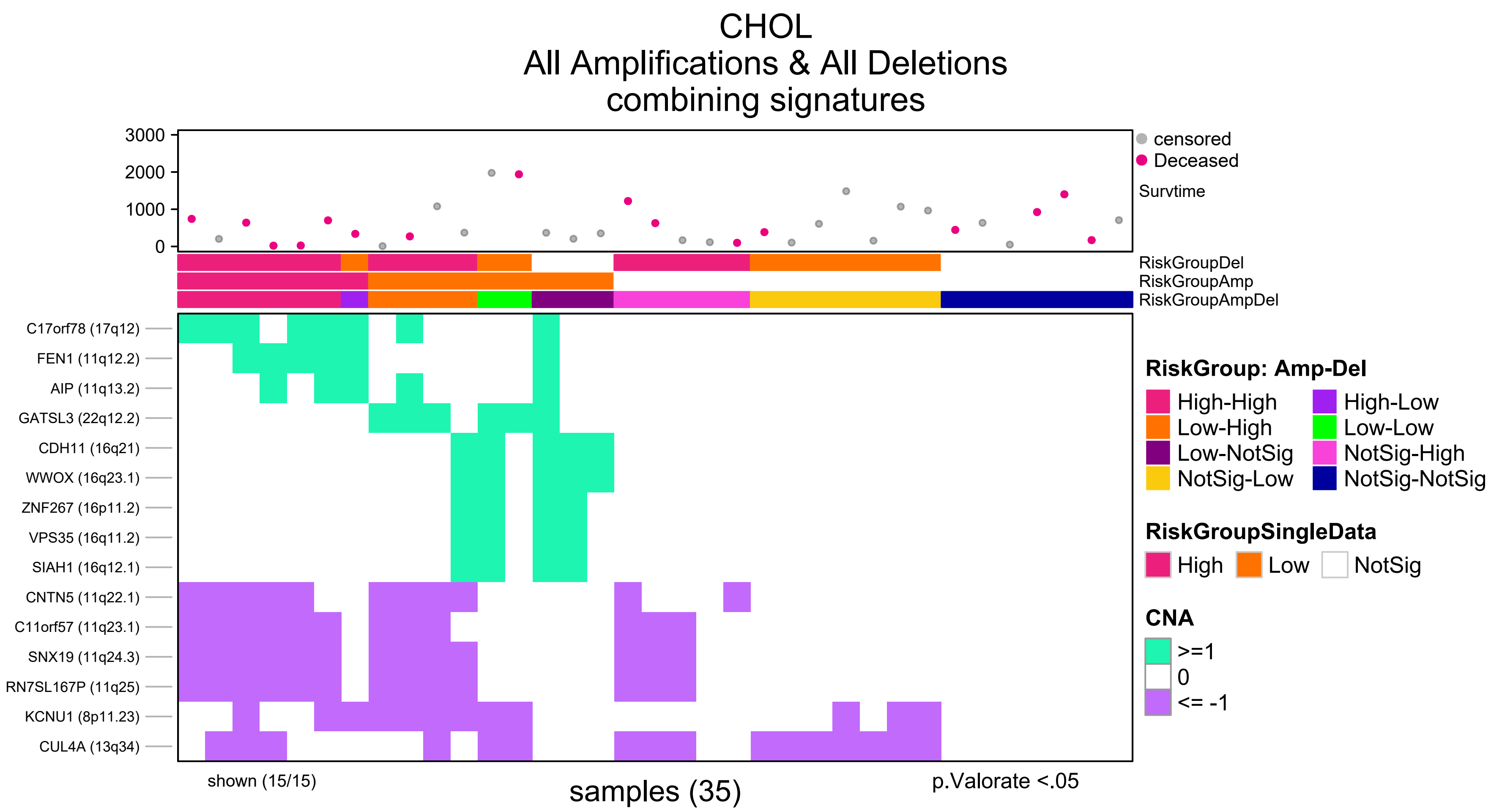
CHOL
All Amplifications & All Deletions
combining signatures
3000
2000
1000
0
censored
Deceased
Survtime
RiskGroupDel
RiskGroupAmp
RiskGroupAmpDel
C17orf78 (17q12)
FEN1 (11q12.2)
AIP (11q13.2)
GATSL3 (22q12.2)
CDH11 (16q21)
WWOX (16q23.1)
ZNF267 (16p11.2)
VPS35 (16q11.2)
SIAH1 (16q12.1)
CNTN5 (11q22.1)
C11orf57 (11q23.1)
SNX19 (11q24.3)
RN7SL167P (11q25)
KCNU1 (8p11.23)
CUL4A (13q34)
RiskGroup: Amp-Del
High-High
High-Low
Low-High
Low-Low
Low-NotSig
NotSig-High
NotSig-Low
NotSig-NotSig
RiskGroupSingleData
High
Low
NotSig
CNA
>=1
0
<= -1
shown (15/15)
samples (35)
p.Valorate <.05

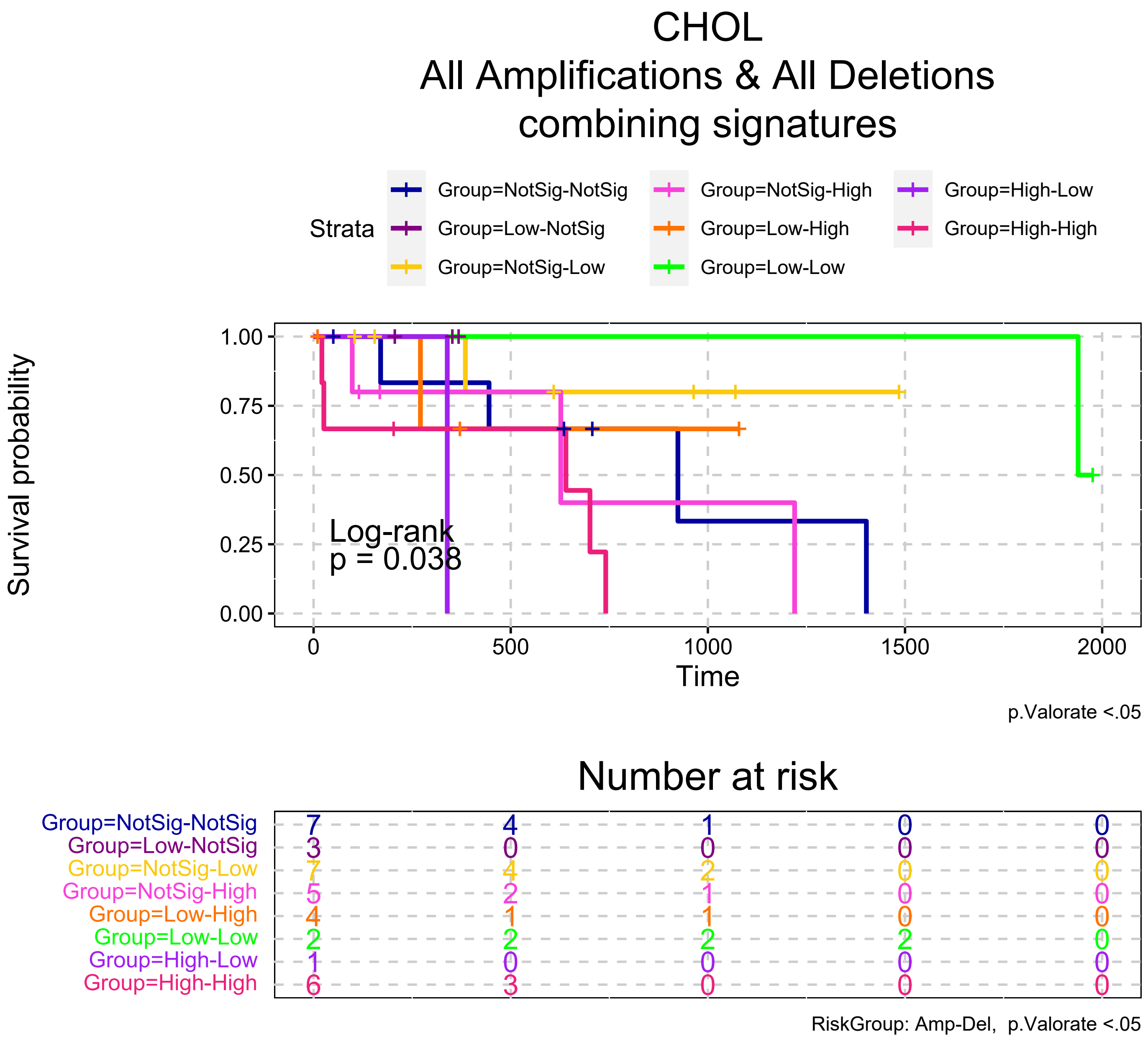

explanatory	beta	HR	L95	U95	p
Low-NotSig	-20.04	0.00	0.00	Inf	1.00
NotSig-Low	-1.43	0.24	0.03	2.14	0.20
NotSig-High	0.57	1.77	0.37	8.59	0.48
Low-High	-0.12	0.89	0.09	8.48	0.92
Low-Low	-19.89	0.00	0.00	Inf	1.00
High-Low	1.70	5.45	0.51	58.04	0.16
High-High	1.09	2.97	0.71	12.51	0.14

n= 35, number of events =16
Score(logrank) test = 0.038
