# Supplementary material for: Systematic Modeling of Risk-Associated Copy Number Alterations in Cancer
Source: Int J Mol Sci. 2024 Sep 27;25(19):10455. doi: 10.3390/ijms251910455 (PMC11477427; doi:10.3390/ijms251910455)
Supplement: Supplementary file 1 [file ijms-25-10455-s001.zip › COADREADSignatureV12-sinSombreado.pdf]

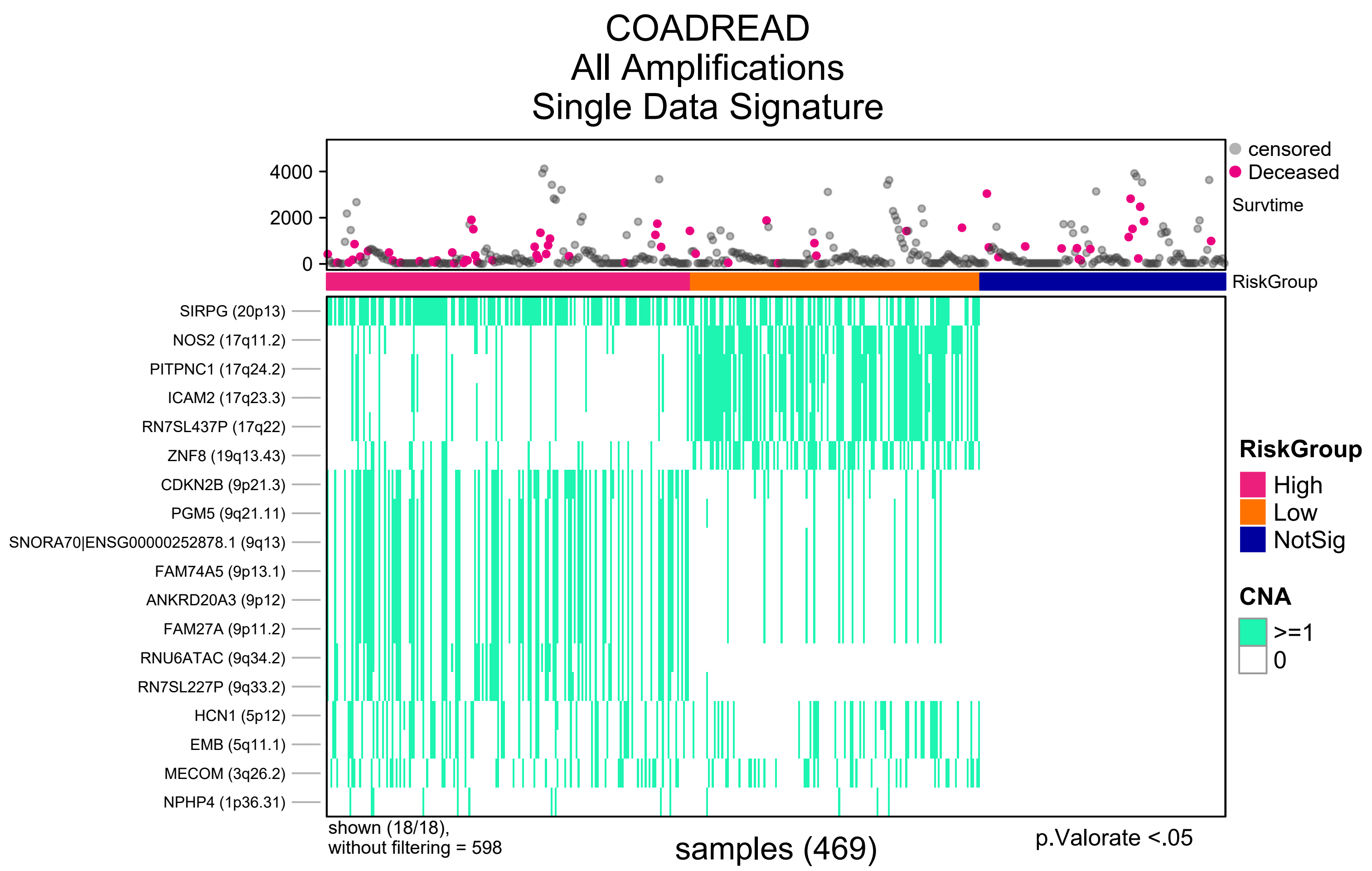

COADREAD
All Amplifications
Single Data Signature
4000
2000
0
censored
Deceased
Survtime
RiskGroup
SIRPG (20p13)
NOS2 (17q11.2)
PITPNC1 (17q24.2)
ICAM2 (17q23.3)
RN7SL437P (17q22)
ZNF8 (19q13.43)
CDKN2B (9p21.3)
PGM5 (9q21.11)
SNORA70|ENSG00000252878.1 (9q13)
FAM74A5 (9p13.1)
ANKRD20A3 (9p12)
FAM27A (9p11.2)
RNU6ATAC (9q34.2)
RN7SL227P (9q33.2)
HCN1 (5p12)
EMB (5q11.1)
MECOM (3q26.2)
NPHP4 (1p36.31)
RiskGroup
High
Low
NotSig
CNA
>=1
0
shown (18/18),
without filtering = 598
samples (469)
p.Valorate <.05

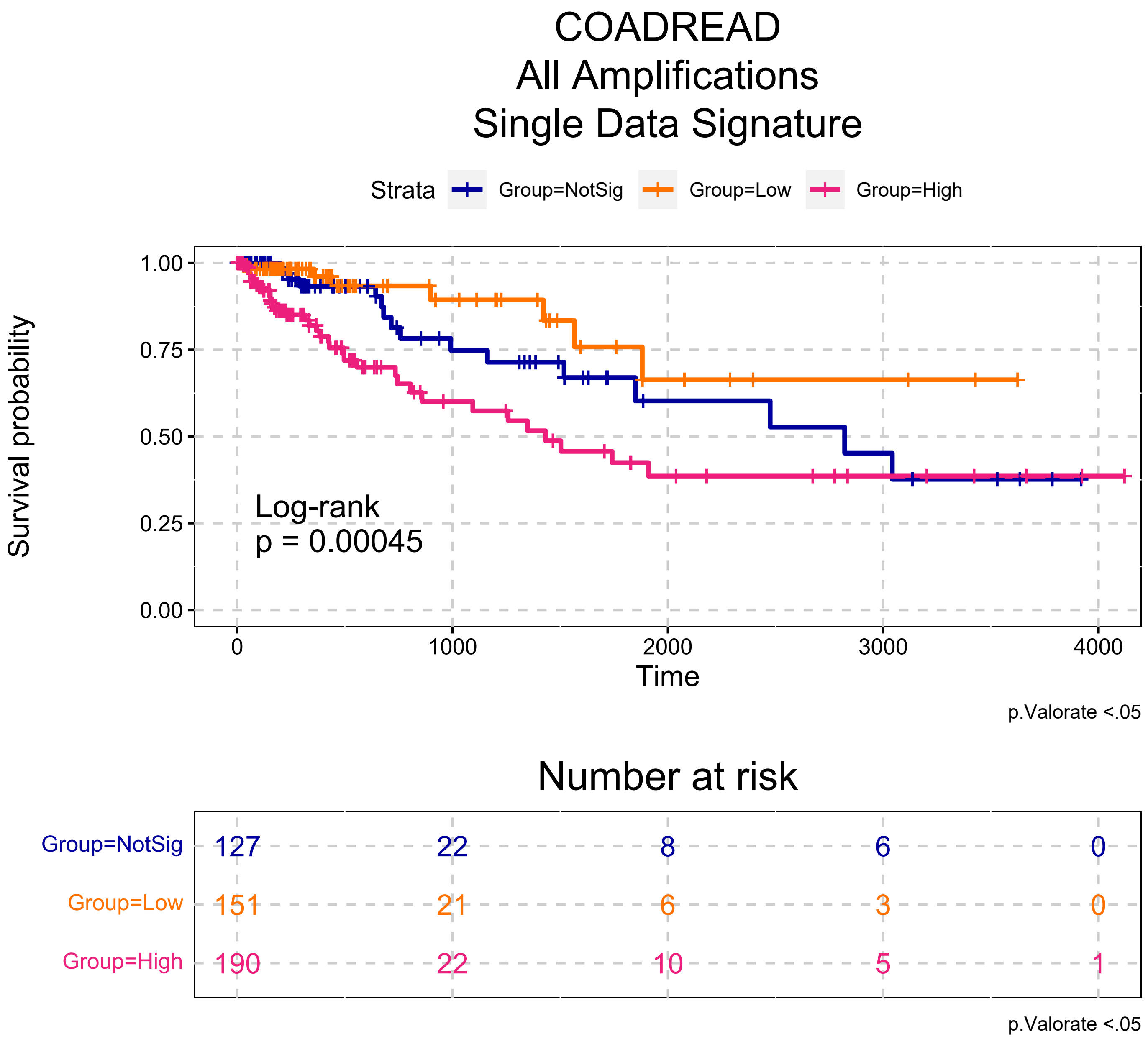

explanatory	beta	HR	L95	U95	p
Low	-0.63	0.54	0.23	1.25	0.15
High	0.69	2.00	1.11	3.60	0.02

n= 469, number of events =61
Score(logrank) test = 0

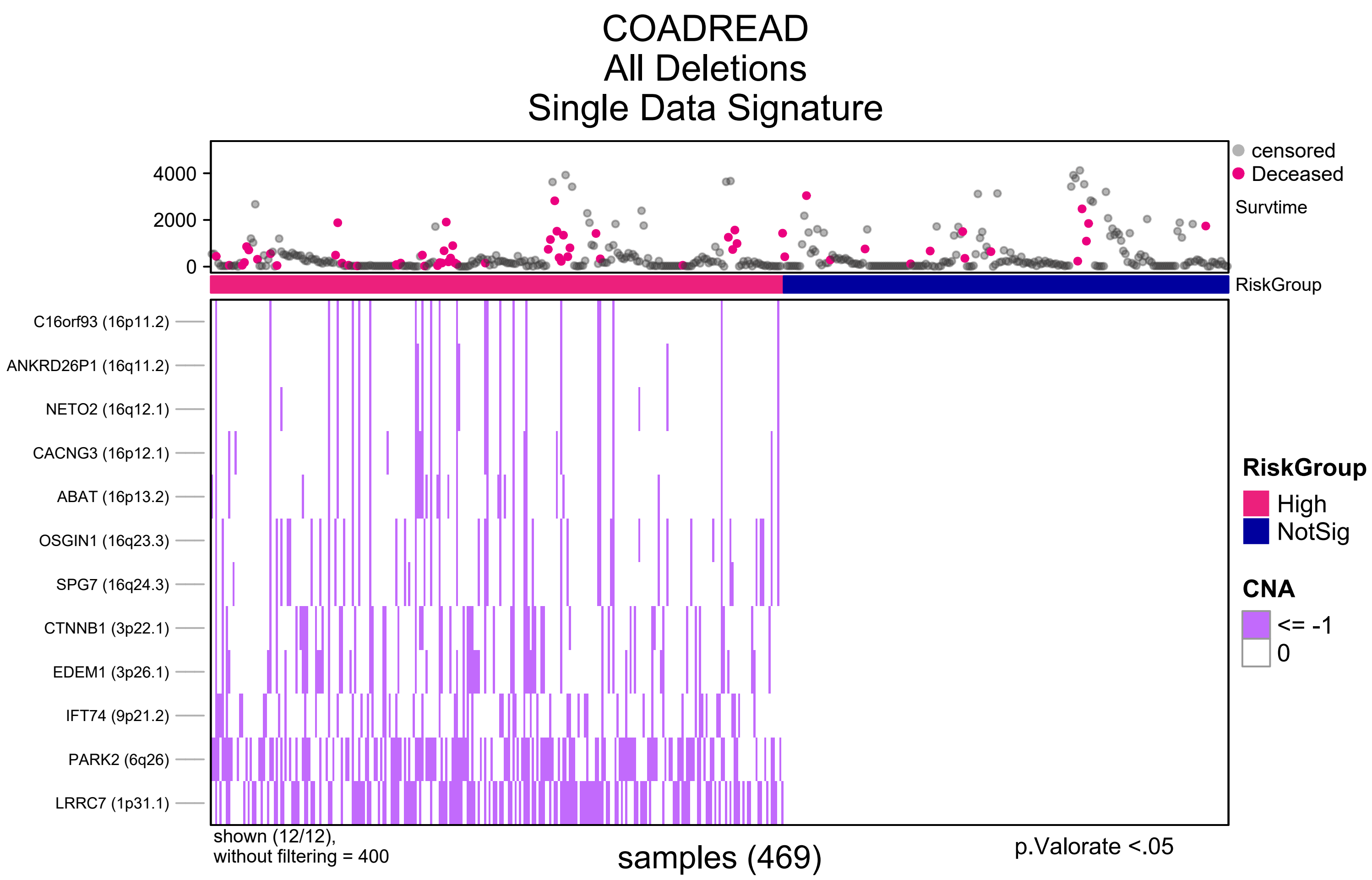
COADREAD
All Deletions
Single Data Signature
4000
2000
0
censored
Deceased
Survtime
RiskGroup
C16orf93 (16p11.2)
ANKRD26P1 (16q11.2)
NETO2 (16q12.1)
CACNG3 (16p12.1)
ABAT (16p13.2)
OSGIN1 (16q23.3)
SPG7 (16q24.3)
CTNNB1 (3p22.1)
EDEM1 (3p26.1)
IFT74 (9p21.2)
PARK2 (6q26)
LRRC7 (1p31.1)
RiskGroup
High
NotSig
CNA
<= -1
0
shown (12/12),
without filtering = 400
samples (469)
p.Valorate <.05

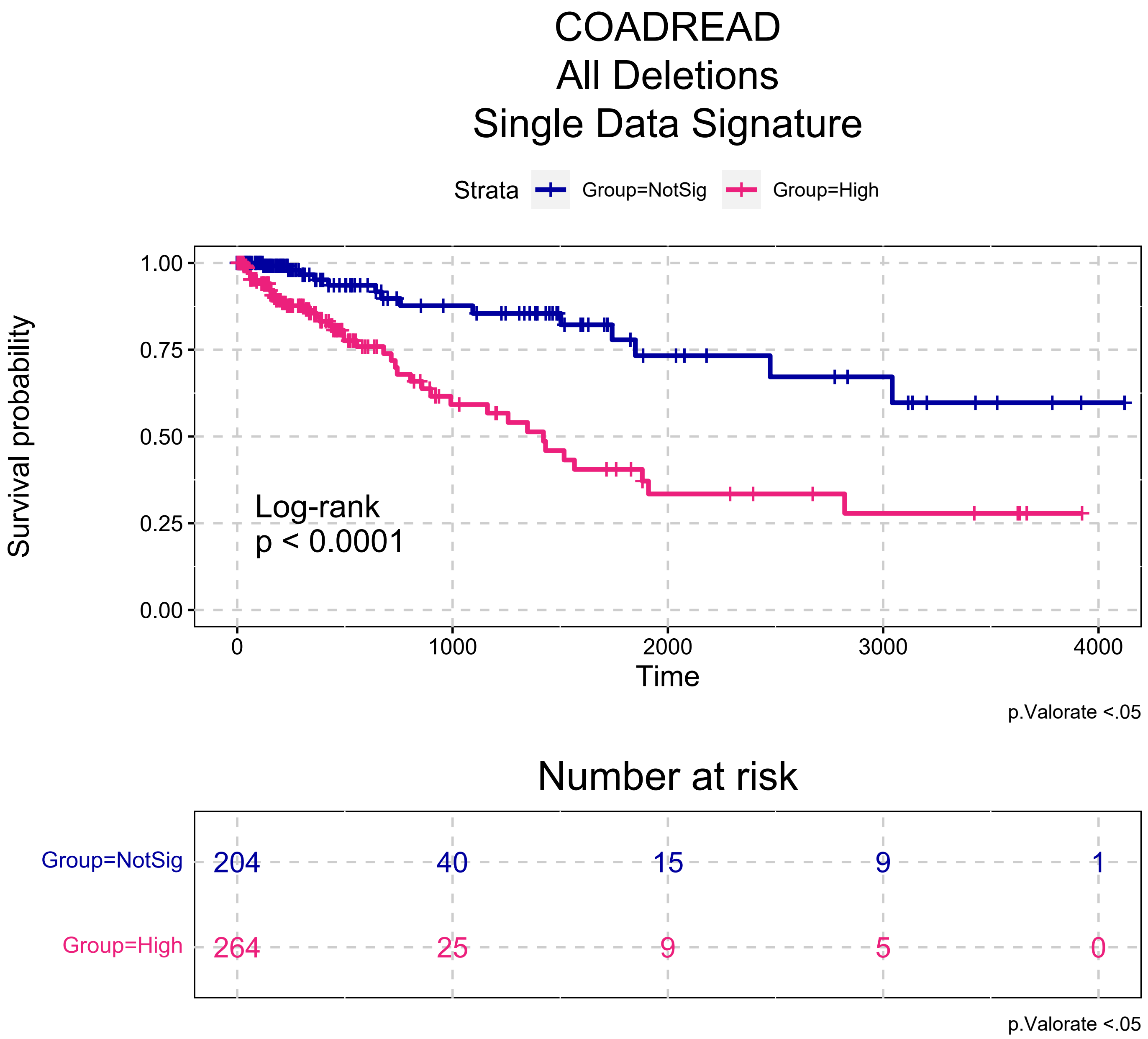

explanatory	beta	HR	L95	U95	p
High	1.30	3.66	2.00	6.67	0.00

n= 469, number of events =61
Score(logrank) test = p <.0001

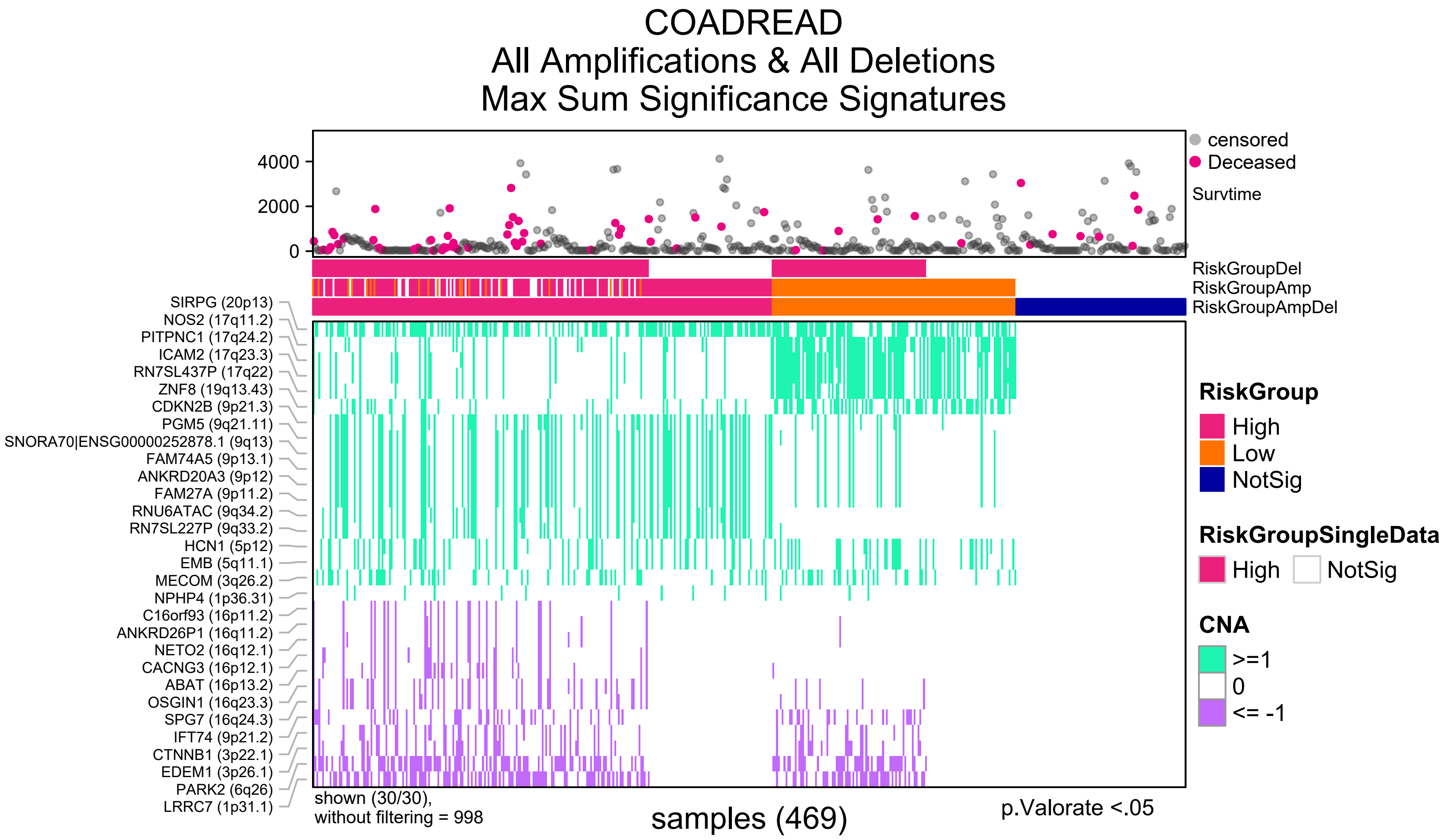

COADREAD
All Amplifications & All Deletions
Max Sum Significance Signatures
4000
2000
0
censored
Deceased
Survtime
RiskGroupDel
RiskGroupAmp
RiskGroupAmpDel
SIRPG (20p13)
NOS2 (17q11.2)
PITPNC1 (17q24.2)
ICAM2 (17q23.3)
RN7SL437P (17q22)
ZNF8 (19q13.43)
CDKN2B (9p21.3)
PGM5 (9q21.11)
SNORA70|ENSG00000252878.1 (9q13)
FAM74A5 (9p13.1)
ANKRD20A3 (9p12)
FAM27A (9p11.2)
RNU6ATAC (9q34.2)
RN7SL227P (9q33.2)
HCN1 (5p12)
EMB (5q11.1)
MECOM (3q26.2)
NPHP4 (1p36.31)
C16orf93 (16p11.2)
ANKRD26P1 (16q11.2)
NETO2 (16q12.1)
CACNG3 (16p12.1)
ABAT (16p13.2)
OSGIN1 (16q23.3)
SPG7 (16q24.3)
IFT74 (9p21.2)
CTNNB1 (3p22.1)
EDEM1 (3p26.1)
PARK2 (6q26)
LRRC7 (1p31.1)
RiskGroup
High
Low
NotSig
RiskGroupSingleData
High
NotSig
CNA
>=1
0
<= -1
shown (30/30),
without filtering = 998
samples (469)
p.Valorate <.05

COADREAD
All Amplifications & All Deletions
Max Sum Significance Signatures

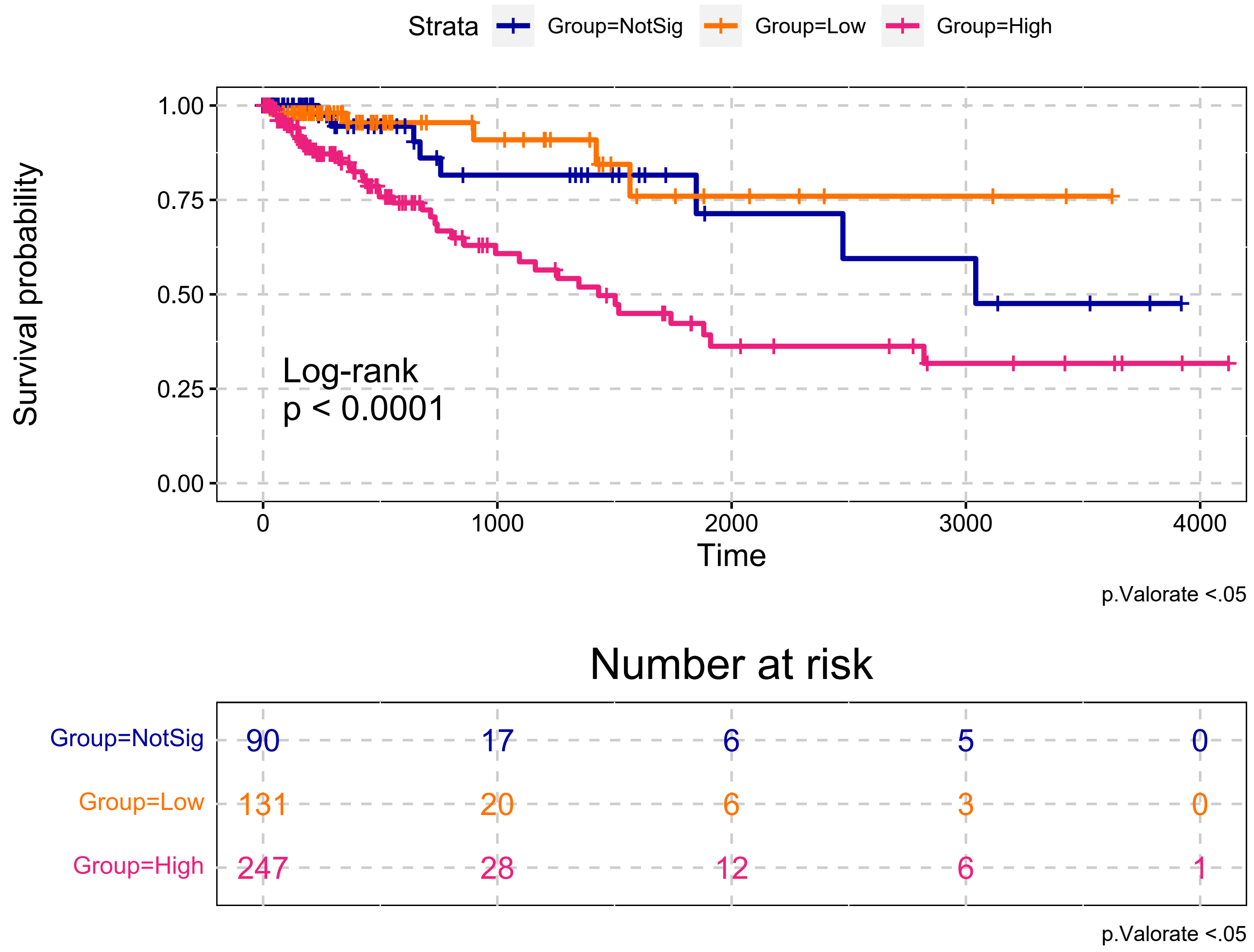

explanatory	beta	HR	L95	U95	p
Low	-0.43	0.65	0.23	1.89	0.43
High	1.02	2.78	1.31	5.88	0.01

n= 469, number of events =61
Score(logrank) test = p <.0001

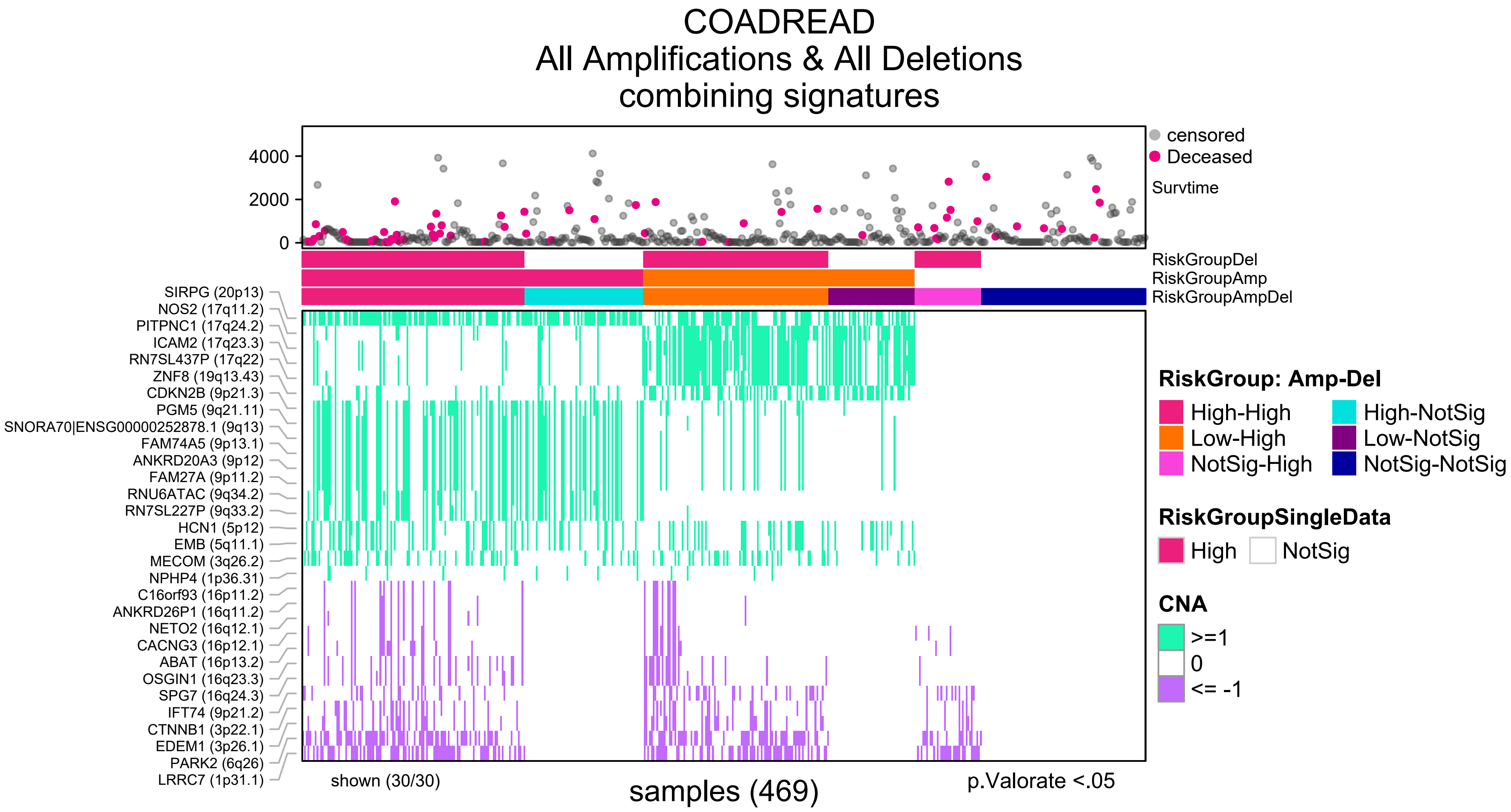

COADREAD
All Amplifications & All Deletions
combining signatures
4000
2000
0
censored
Deceased
Survtime
RiskGroupDel
RiskGroupAmp
RiskGroupAmpDel
SIRPG (20p13)
NOS2 (17q11.2)
PITPNC1 (17q24.2)
ICAM2 (17q23.3)
RN7SL437P (17q22)
ZNF8 (19q13.43)
CDKN2B (9p21.3)
PGM5 (9q21.11)
SNORA70|ENSG00000252878.1 (9q13)
FAM74A5 (9p13.1)
ANKRD20A3 (9p12)
FAM27A (9p11.2)
RNU6ATAC (9q34.2)
RN7SL227P (9q33.2)
HCN1 (5p12)
EMB (5q11.1)
MECOM (3q26.2)
NPHP4 (1p36.31)
C16orf93 (16p11.2)
ANKRD26P1 (16q11.2)
NETO2 (16q12.1)
CACNG3 (16p12.1)
ABAT (16p13.2)
OSGIN1 (16q23.3)
SPG7 (16q24.3)
IFT74 (9p21.2)
CTNNB1 (3p22.1)
EDEM1 (3p26.1)
PARK2 (6q26)
LRRC7 (1p31.1)
RiskGroup: Amp-Del
High-High
High-NotSig
Low-High
Low-NotSig
NotSig-High
NotSig-NotSig
RiskGroupSingleData
High
NotSig
CNA
>=1
0
<= -1
shown (30/30)
samples (469)
p.Valorate <.05

COADREAD
All Amplifications & All Deletions combining signatures

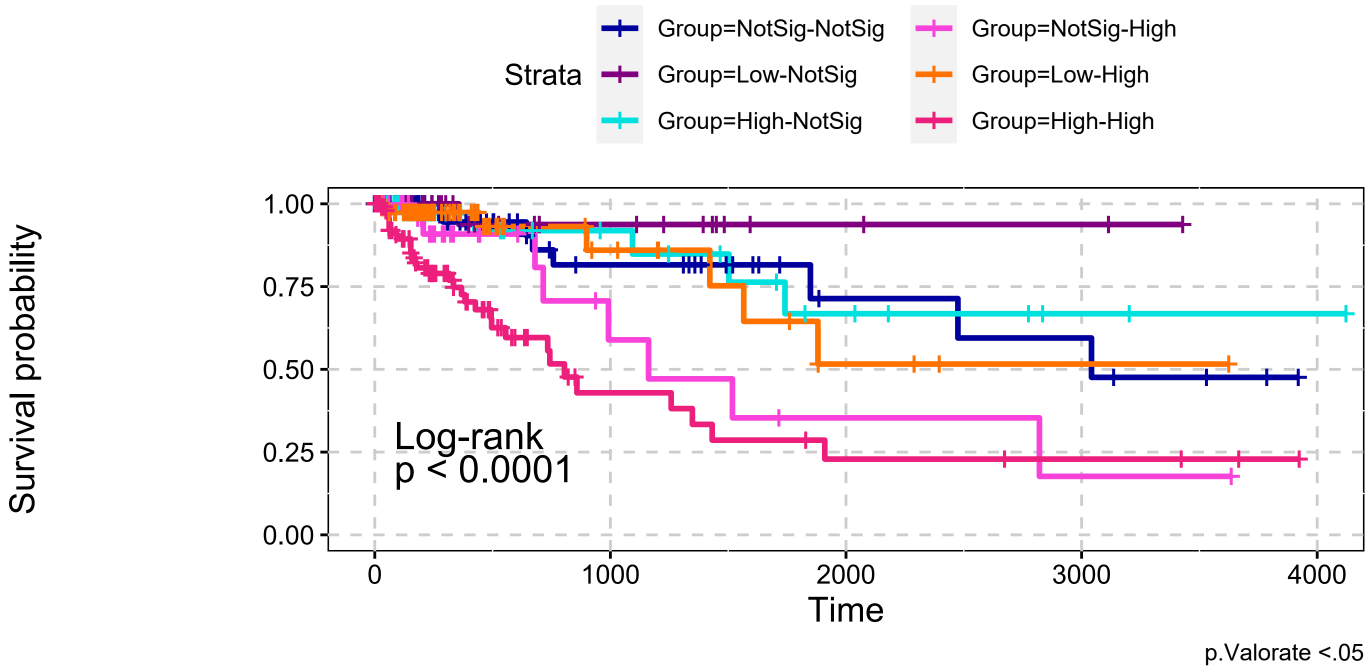

Number at risk

	0	1000	2000	3000	4000
Group=NotSig-NotSig	90	17	6	5	0
Group=Low-NotSig	48	10	3	2	0
Group=High-NotSig	66	13	6	2	1
Group=NotSig-High	37	5	2	1	0
Group=Low-High	103	11	3	1	0
Group=High-High	124	9	4	3	0

RiskGroup: Amp-Del, p.Valorate <.05

explanatory	beta	HR	L95	U95	p
Low-NotSig	-1.39	0.25	0.03	1.99	0.19
High-NotSig	-0.12	0.89	0.29	2.72	0.84
NotSig-High	0.97	2.63	0.99	7.03	0.05
Low-High	0.13	1.14	0.41	3.15	0.80
High-High	1.52	4.57	2.10	9.97	0.00

n= 469, number of events =61
Score(logrank) test = p <.0001

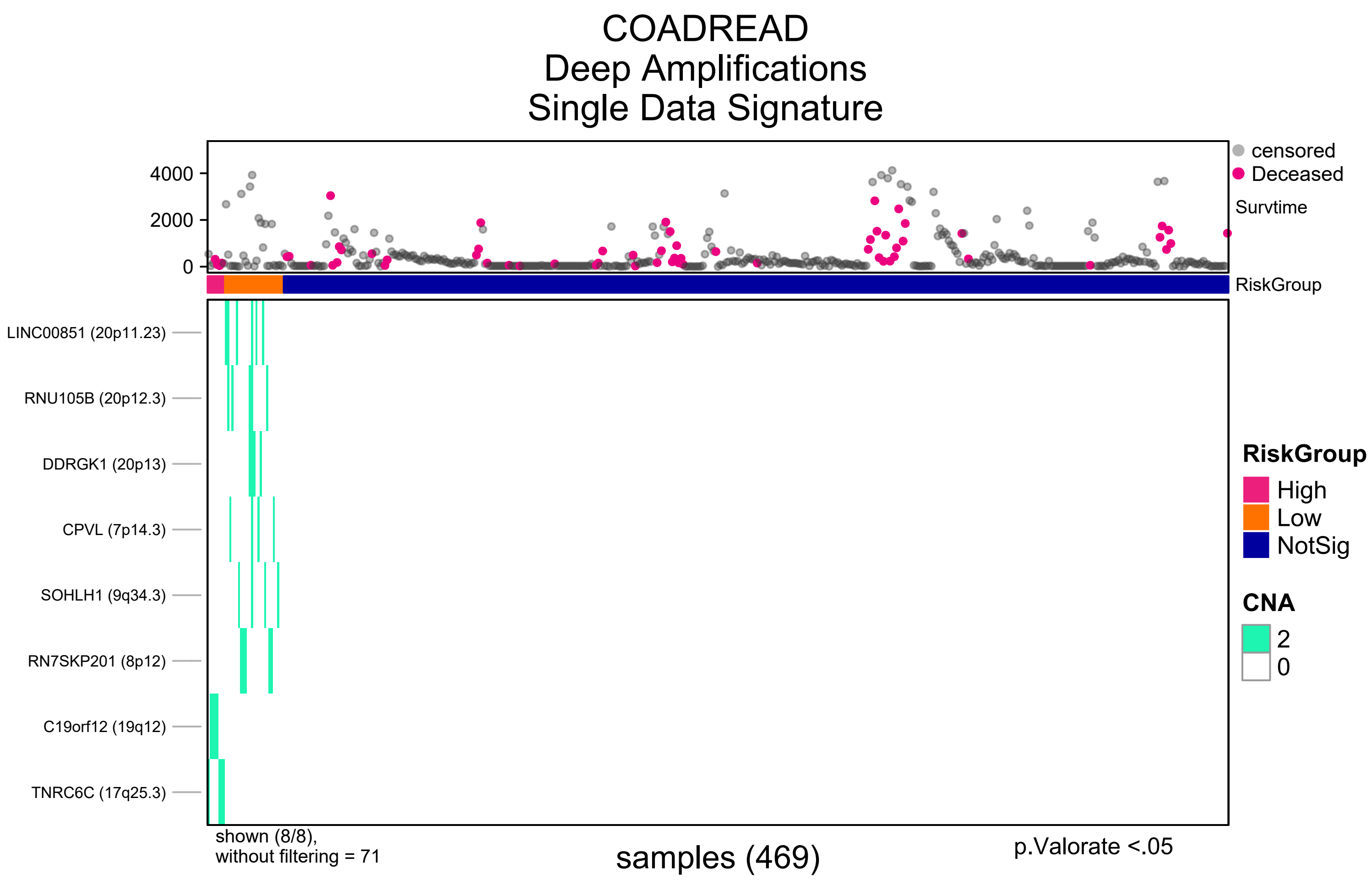
COADREAD
Deep Amplifications
Single Data Signature
4000
2000
0
censored
Deceased
Survtime
RiskGroup
LINC00851 (20p11.23)
RNU105B (20p12.3)
DDRGK1 (20p13)
CPVL (7p14.3)
SOHLH1 (9q34.3)
RN7SKP201 (8p12)
C19orf12 (19q12)
TNRC6C (17q25.3)
RiskGroup
High
Low
NotSig
CNA
2
0
shown (8/8),
without filtering = 71
samples (469)
p.Valorate <.05

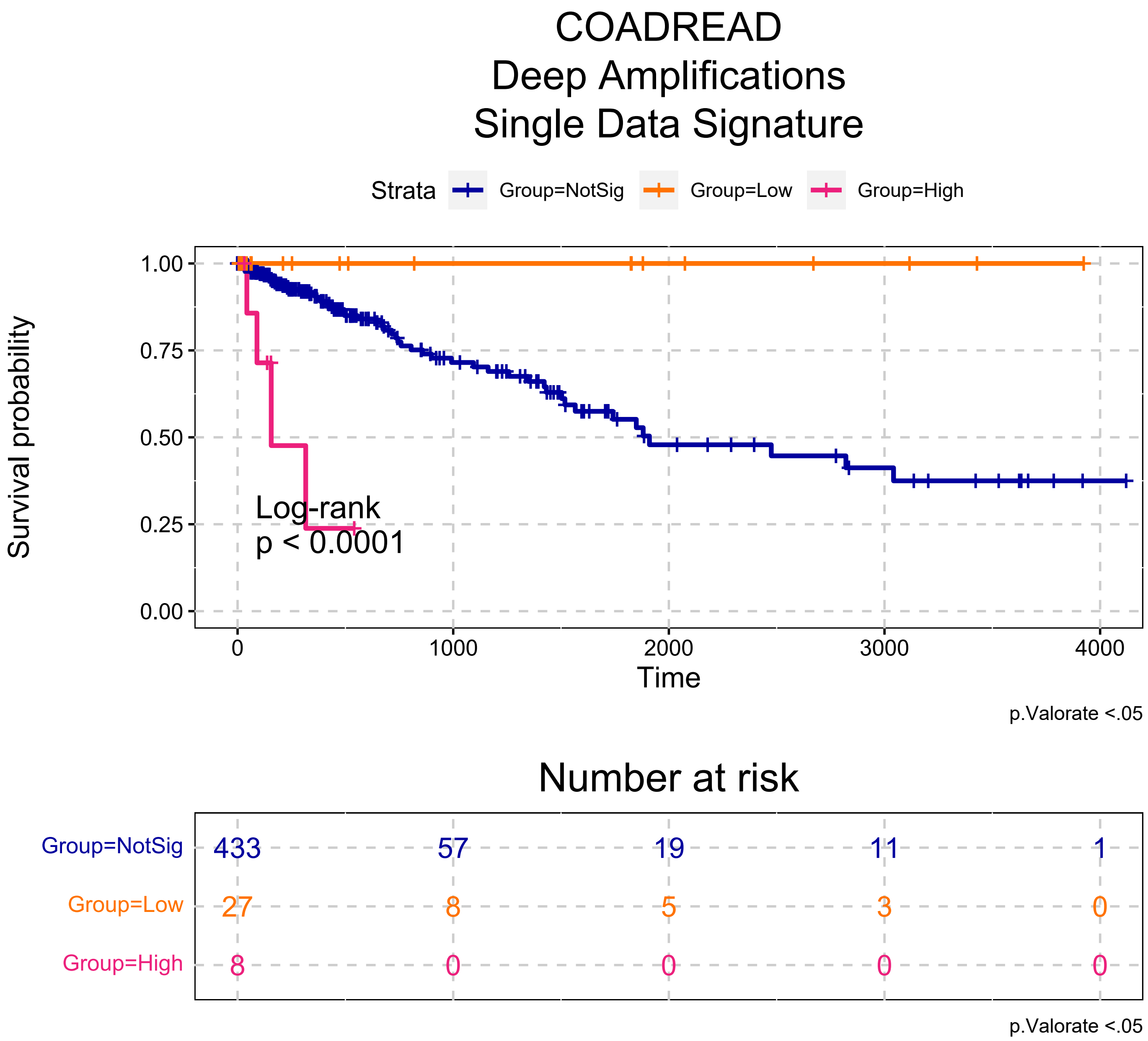

explanatory	beta	HR	L95	U95	p
Low	-17.84	0.00	0.00	Inf	0.99
High	2.26	9.61	3.35	27.54	0.00

n= 469, number of events =61
Score(logrank) test = p <.0001

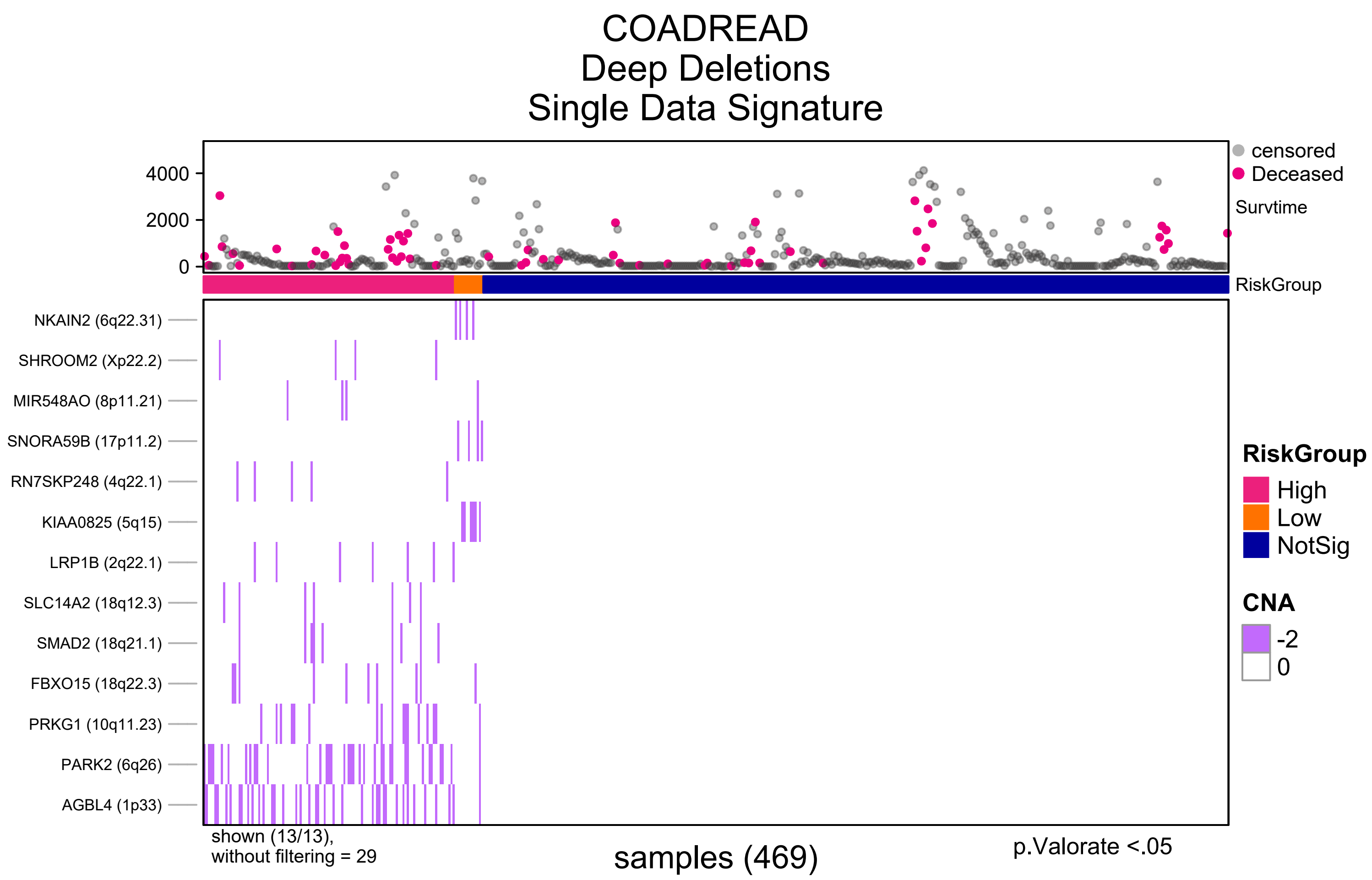

COADREAD
Deep Deletions
Single Data Signature
4000
2000
0
censored
Deceased
Survtime
RiskGroup
NKAIN2 (6q22.31)
SHROOM2 (Xp22.2)
MIR548AO (8p11.21)
SNORA59B (17p11.2)
RN7SKP248 (4q22.1)
KIAA0825 (5q15)
LRP1B (2q22.1)
SLC14A2 (18q12.3)
SMAD2 (18q21.1)
FBXO15 (18q22.3)
PRKG1 (10q11.23)
PARK2 (6q26)
AGBL4 (1p33)
RiskGroup
High
Low
NotSig
CNA
-2
0
shown (13/13),
without filtering = 29
samples (469)
p.Valorate <.05

COADREAD
Deep Deletions
Single Data Signature

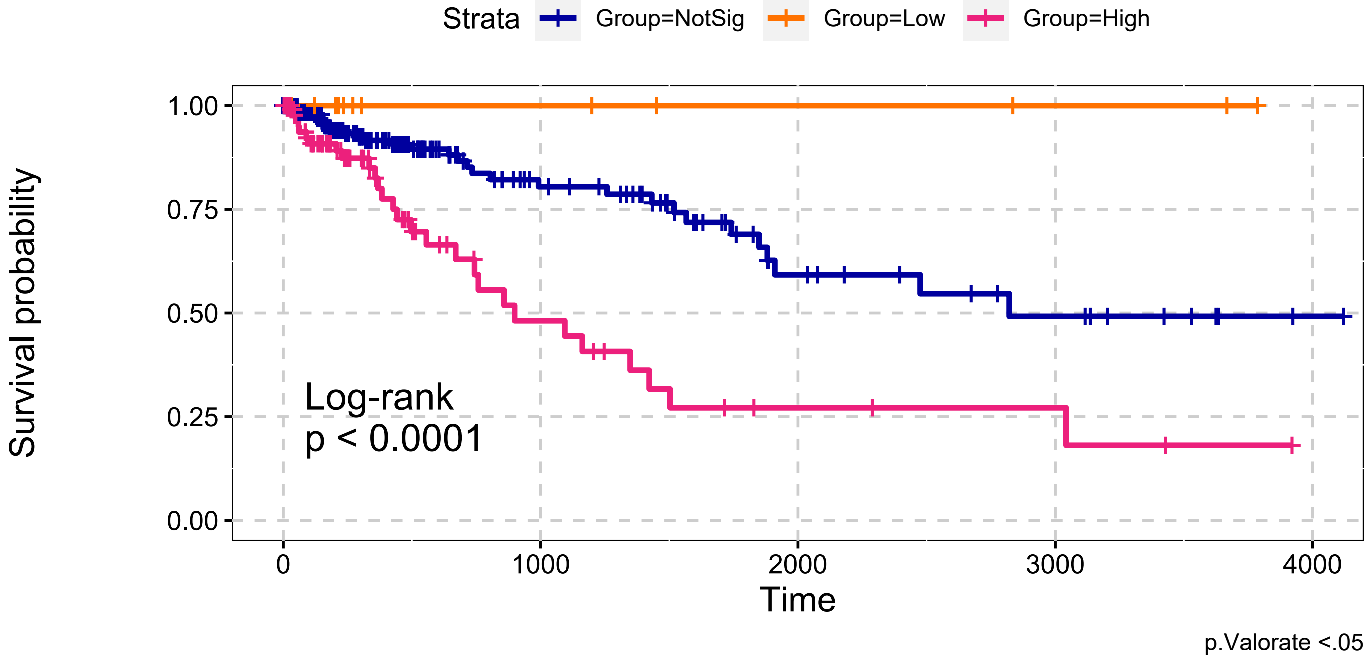

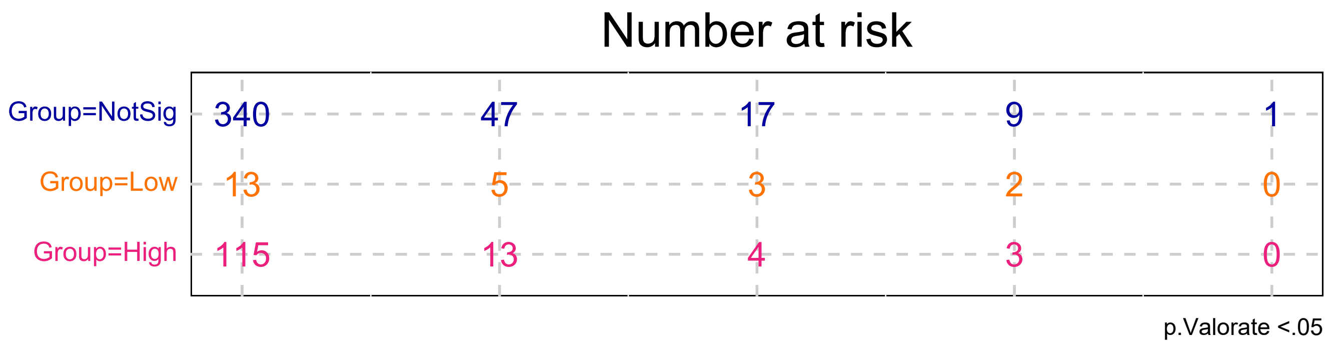

explanatory	beta	HR	L95	U95	p
Low	-16.99	0.00	0.00	Inf	1.00
High	1.07	2.91	1.75	4.82	0.00

n= 469, number of events =61
Score(logrank) test = p <.0001

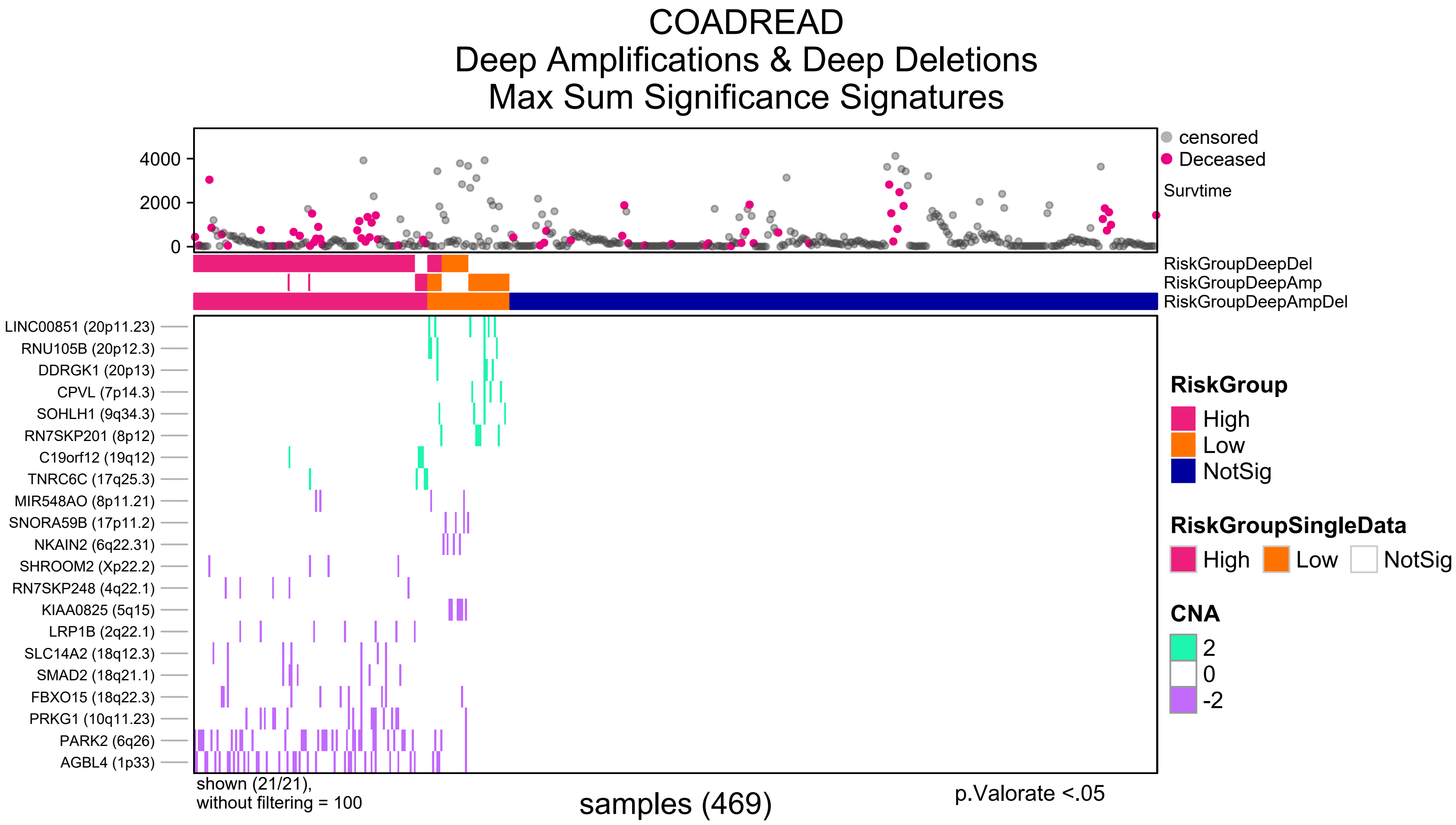
COADREAD
Deep Amplifications & Deep Deletions
Max Sum Significance Signatures
4000
2000
0
censored
Deceased
Survtime
RiskGroupDeepDel
RiskGroupDeepAmp
RiskGroupDeepAmpDel
LINC00851 (20p11.23)
RNU105B (20p12.3)
DDRGK1 (20p13)
CPVL (7p14.3)
SOHLH1 (9q34.3)
RN7SKP201 (8p12)
C19orf12 (19q12)
TNRC6C (17q25.3)
MIR548AO (8p11.21)
SNORA59B (17p11.2)
NKAIN2 (6q22.31)
SHROOM2 (Xp22.2)
RN7SKP248 (4q22.1)
KIAA0825 (5q15)
LRP1B (2q22.1)
SLC14A2 (18q12.3)
SMAD2 (18q21.1)
FBXO15 (18q22.3)
PRKG1 (10q11.23)
PARK2 (6q26)
AGBL4 (1p33)
RiskGroup
High
Low
NotSig
RiskGroupSingleData
High
Low
NotSig
CNA
2
0
-2
shown (21/21),
without filtering = 100
samples (469)
p.Valorate <.05

COADREAD
Deep Amplifications & Deep Deletions
Max Sum Significance Signatures

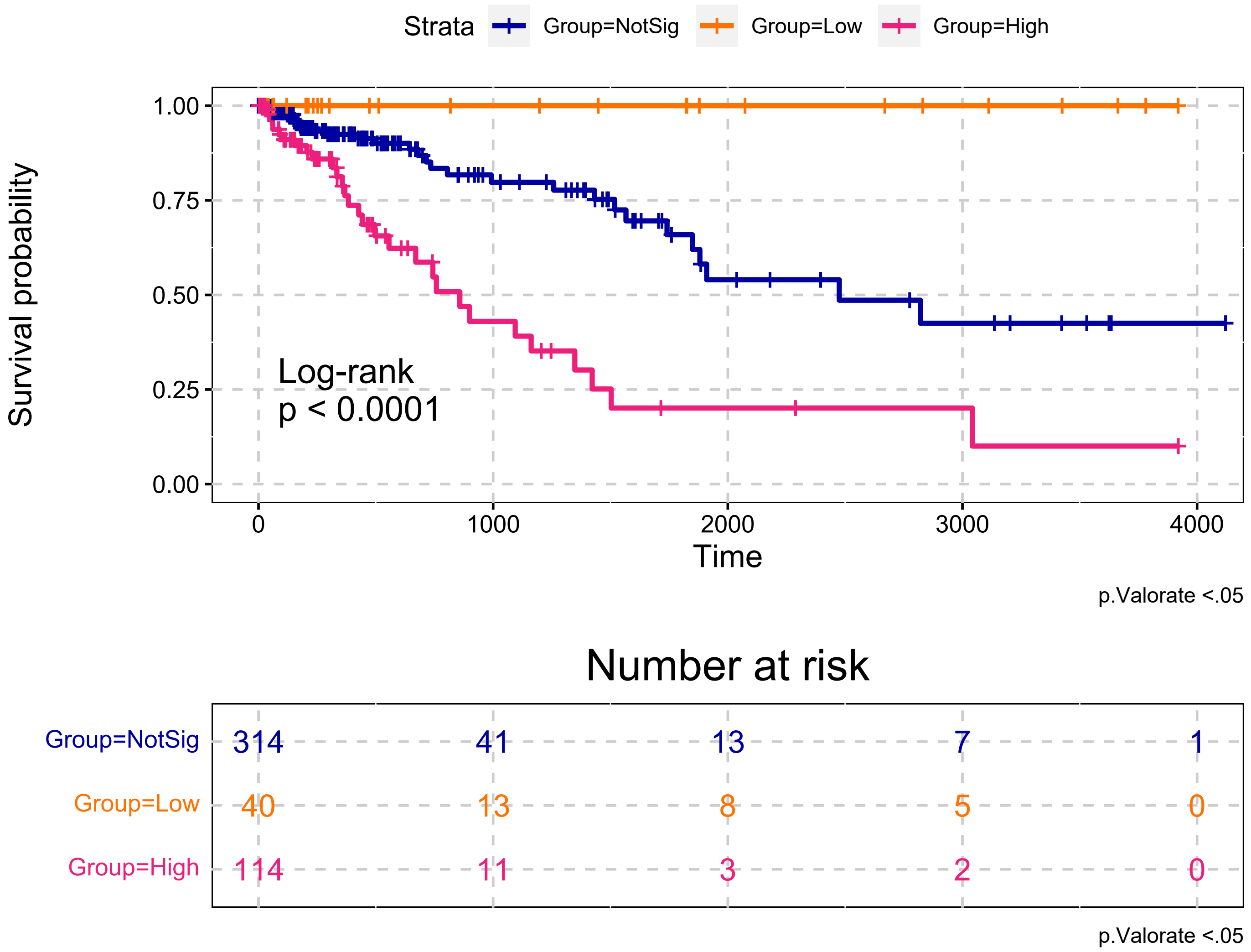

explanatory	beta	HR	L95	U95	p
Low	-18.32	0.00	0.00	Inf	1.00
High	1.19	3.29	1.98	5.46	0.00

n= 469, number of events =61
Score(logrank) test = p <.0001

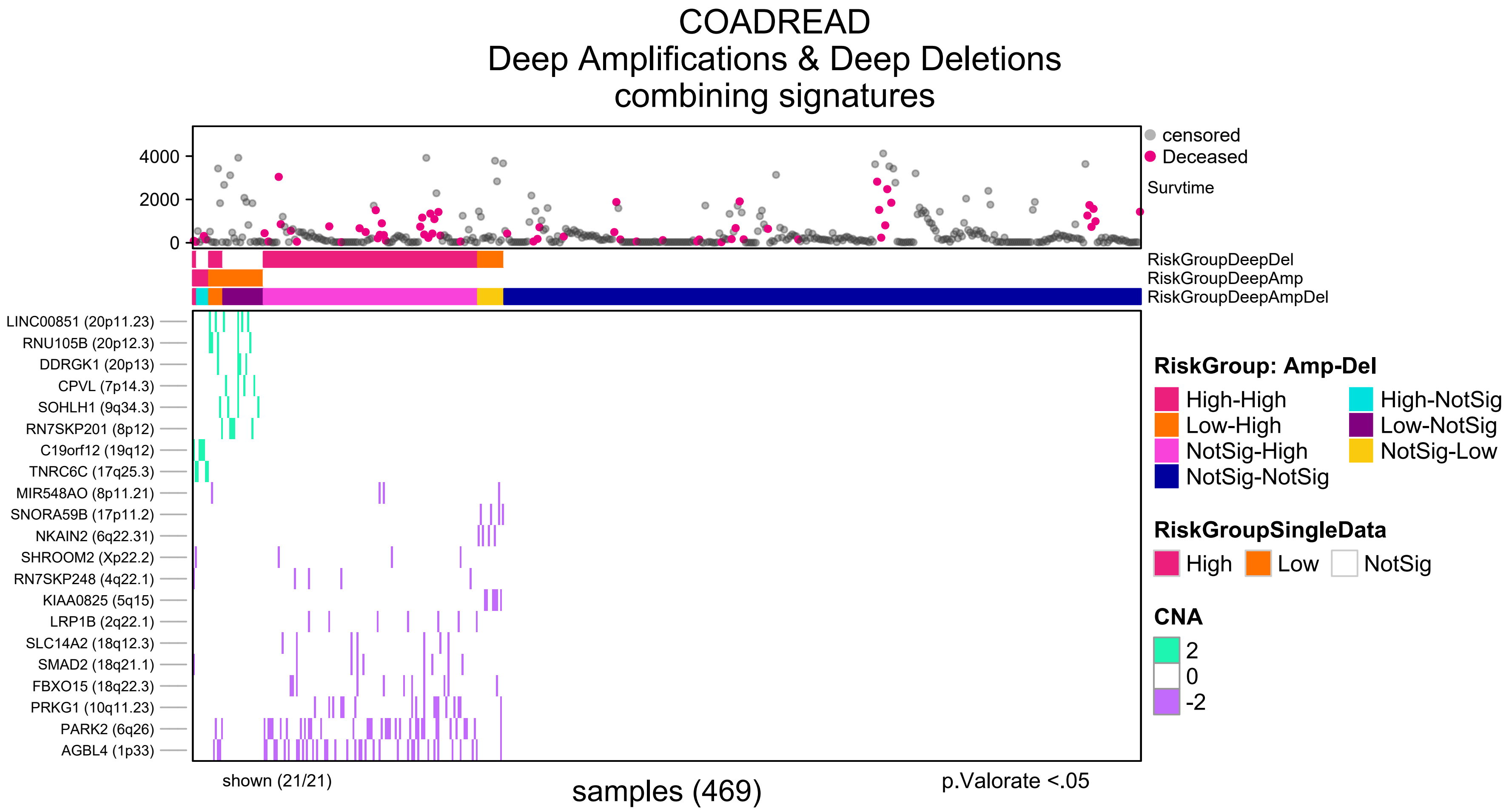

COADREAD
Deep Amplifications & Deep Deletions
combining signatures
4000
2000
0
censored
Deceased
Survtime
RiskGroupDeepDel
RiskGroupDeepAmp
RiskGroupDeepAmpDel
LINC00851 (20p11.23)
RNU105B (20p12.3)
DDRGK1 (20p13)
CPVL (7p14.3)
SOHLH1 (9q34.3)
RN7SKP201 (8p12)
C19orf12 (19q12)
TNRC6C (17q25.3)
MIR548AO (8p11.21)
SNORA59B (17p11.2)
NKAIN2 (6q22.31)
SHROOM2 (Xp22.2)
RN7SKP248 (4q22.1)
KIAA0825 (5q15)
LRP1B (2q22.1)
SLC14A2 (18q12.3)
SMAD2 (18q21.1)
FBXO15 (18q22.3)
PRKG1 (10q11.23)
PARK2 (6q26)
AGBL4 (1p33)
RiskGroup: Amp-Del
High-High
Low-High
NotSig-High
NotSig-NotSig
High-NotSig
Low-NotSig
NotSig-Low
RiskGroupSingleData
High
Low
NotSig
CNA
2
0
-2
shown (21/21)
samples (469)
p.Valorate <.05

COADREAD
Deep Amplifications & Deep Deletions combining signatures

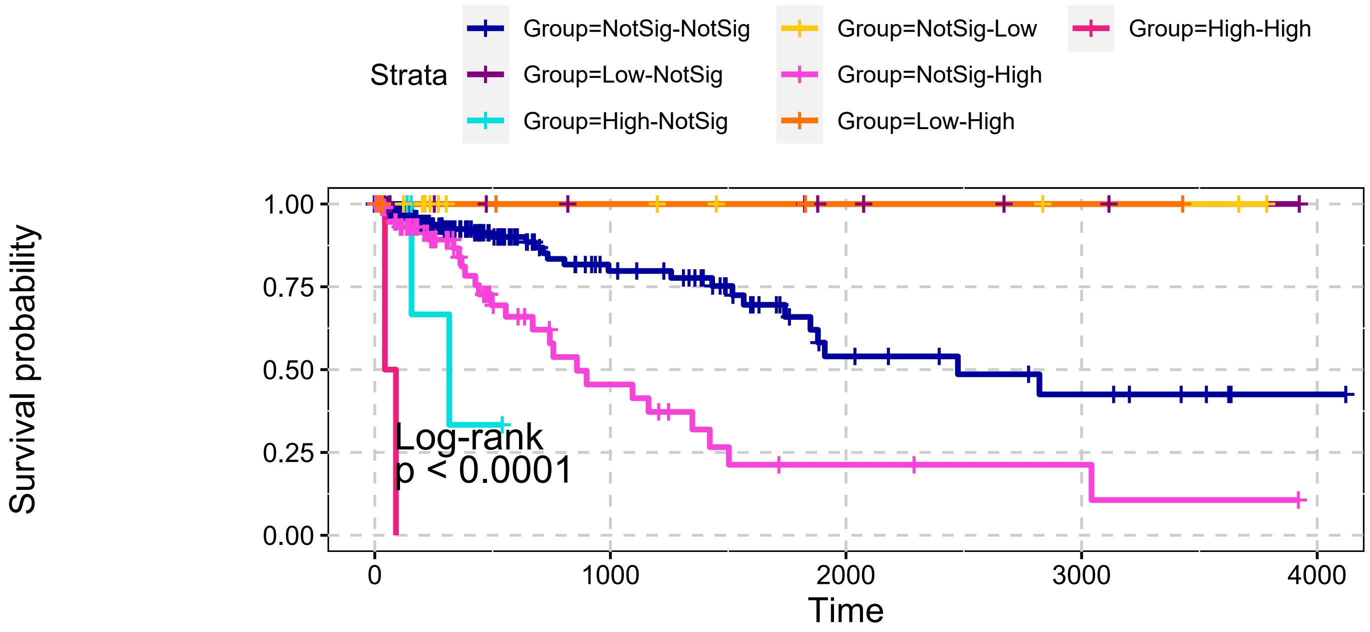

p.Valorate <.05

explanatory	beta	HR	L95	U95	p
Low-NotSig	-14.84	0.00	0.00	Inf	0.99
High-NotSig	2.02	7.56	1.80	31.69	0.01
NotSig-Low	-14.84	0.00	0.00	Inf	0.99
NotSig-High	1.09	2.97	1.78	4.95	0.00
Low-High	-14.84	0.00	0.00	Inf	0.99
High-High	4.55	94.30	19.88	447.36	0.00

n= 469, number of events =61
Score(logrank) test = p <.0001

Number at risk

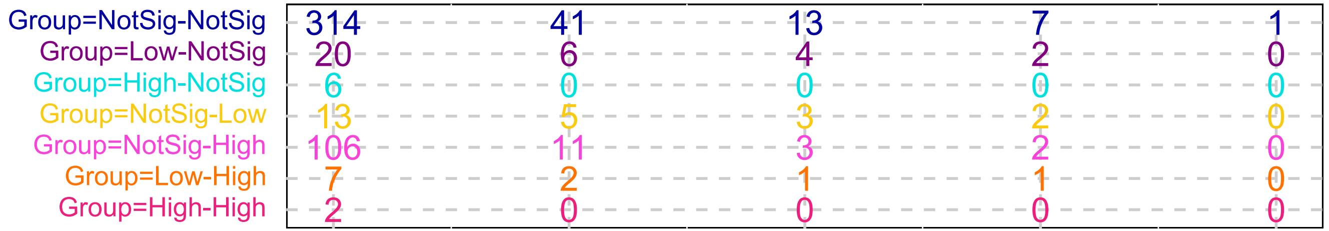

RiskGroup: Amp-Del, p.Valorate <.05
