# Supplementary material for: Systematic Modeling of Risk-Associated Copy Number Alterations in Cancer
Source: Int J Mol Sci. 2024 Sep 27;25(19):10455. doi: 10.3390/ijms251910455 (PMC11477427; doi:10.3390/ijms251910455)
Supplement: Supplementary file 1 [file ijms-25-10455-s001.zip › COADSignatureV12-sinSombreado.pdf]

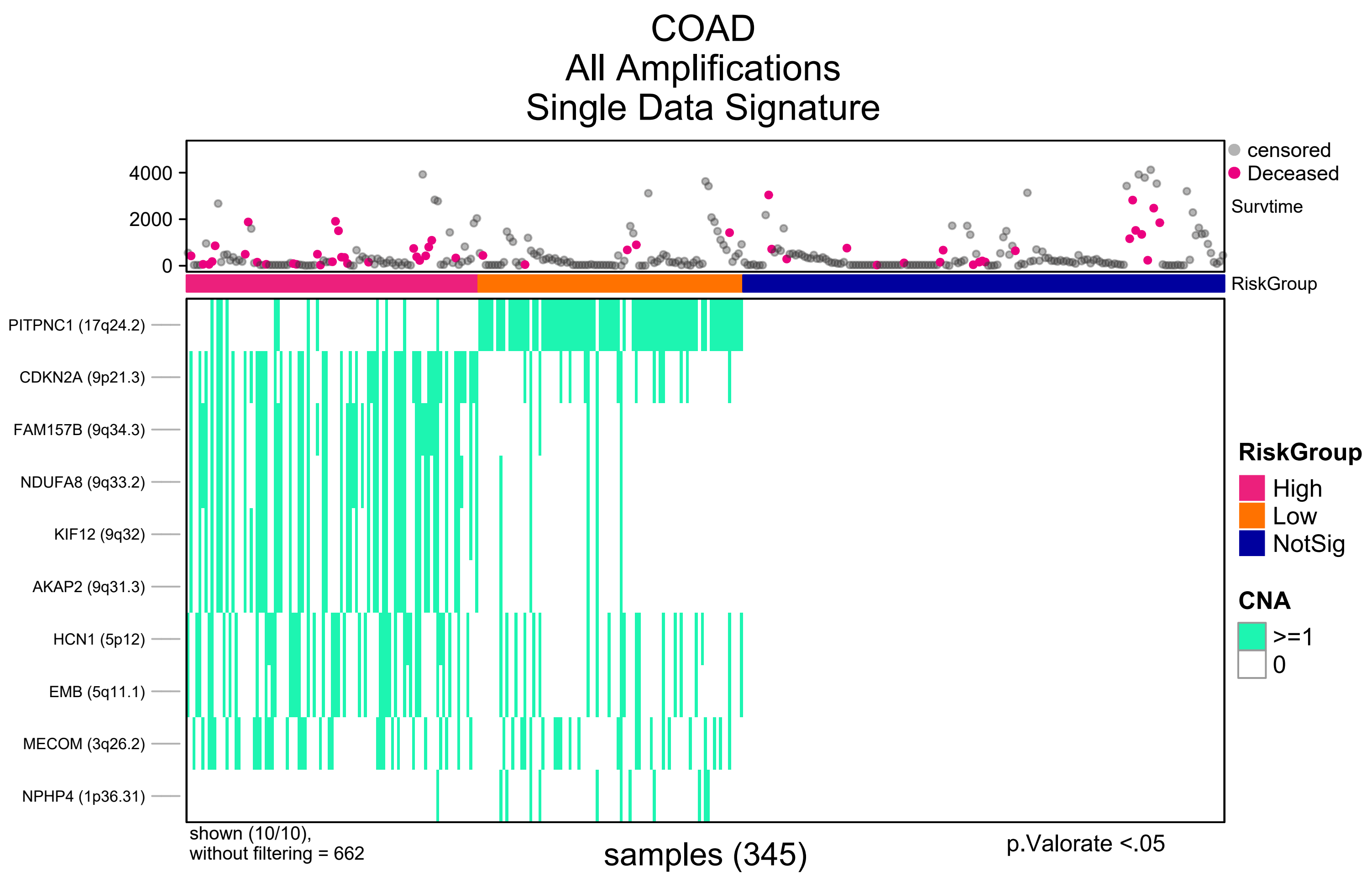

COAD
All Amplifications
Single Data Signature
4000
2000
0
censored
Deceased
Survtime
RiskGroup
PITPNC1 (17q24.2)
CDKN2A (9p21.3)
FAM157B (9q34.3)
NDUFA8 (9q33.2)
KIF12 (9q32)
AKAP2 (9q31.3)
HCN1 (5p12)
EMB (5q11.1)
MECOM (3q26.2)
NPHP4 (1p36.31)
RiskGroup
High
Low
NotSig
CNA
>=1
0
shown (10/10),
without filtering = 662
samples (345)
p.Valorate <.05

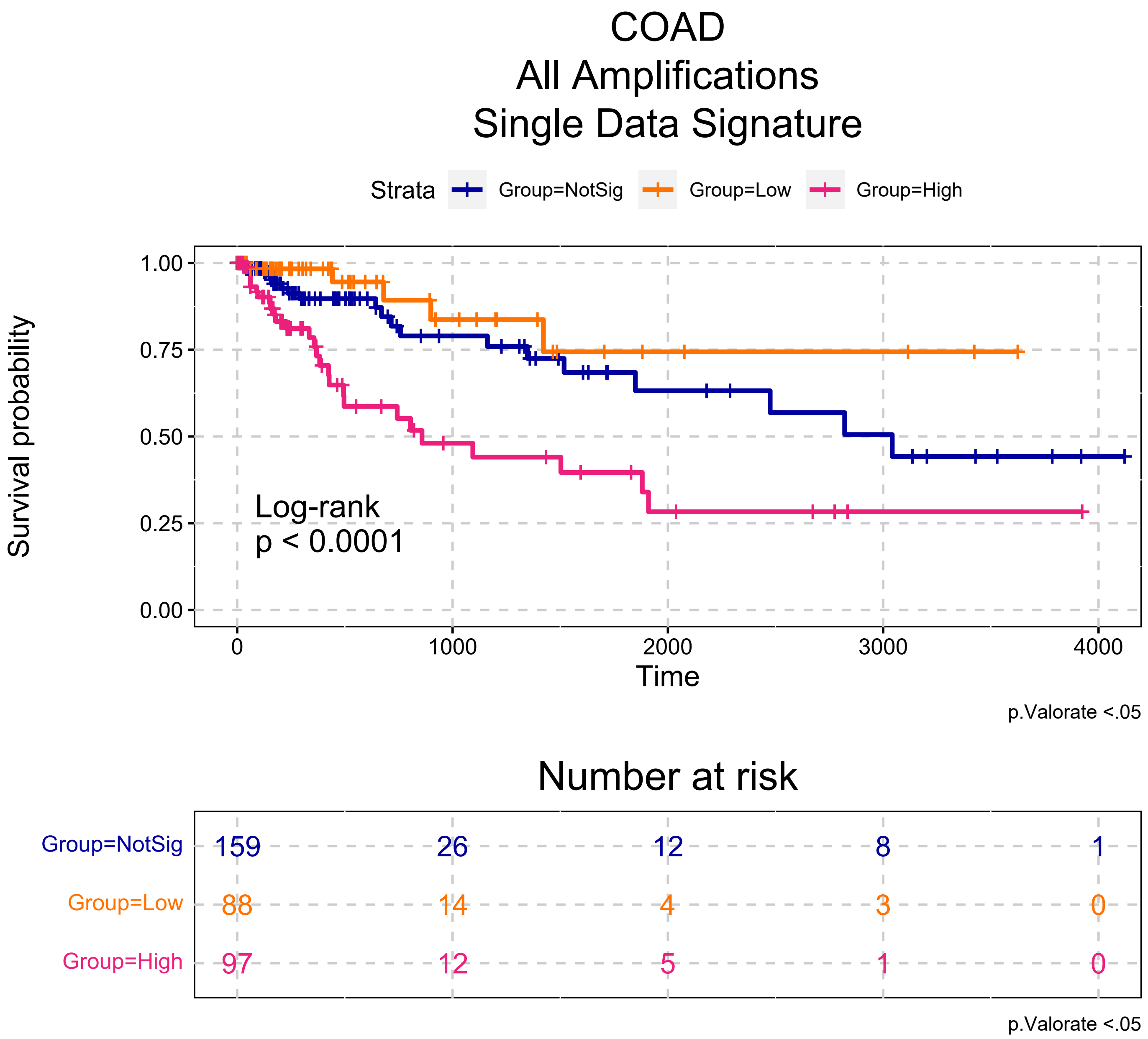

explanatory	beta	HR	L95	U95	p
Low	-0.73	0.48	0.18	1.28	0.14
High	0.93	2.54	1.42	4.54	0.00

n= 345, number of events =52
Score(logrank) test = p <.0001

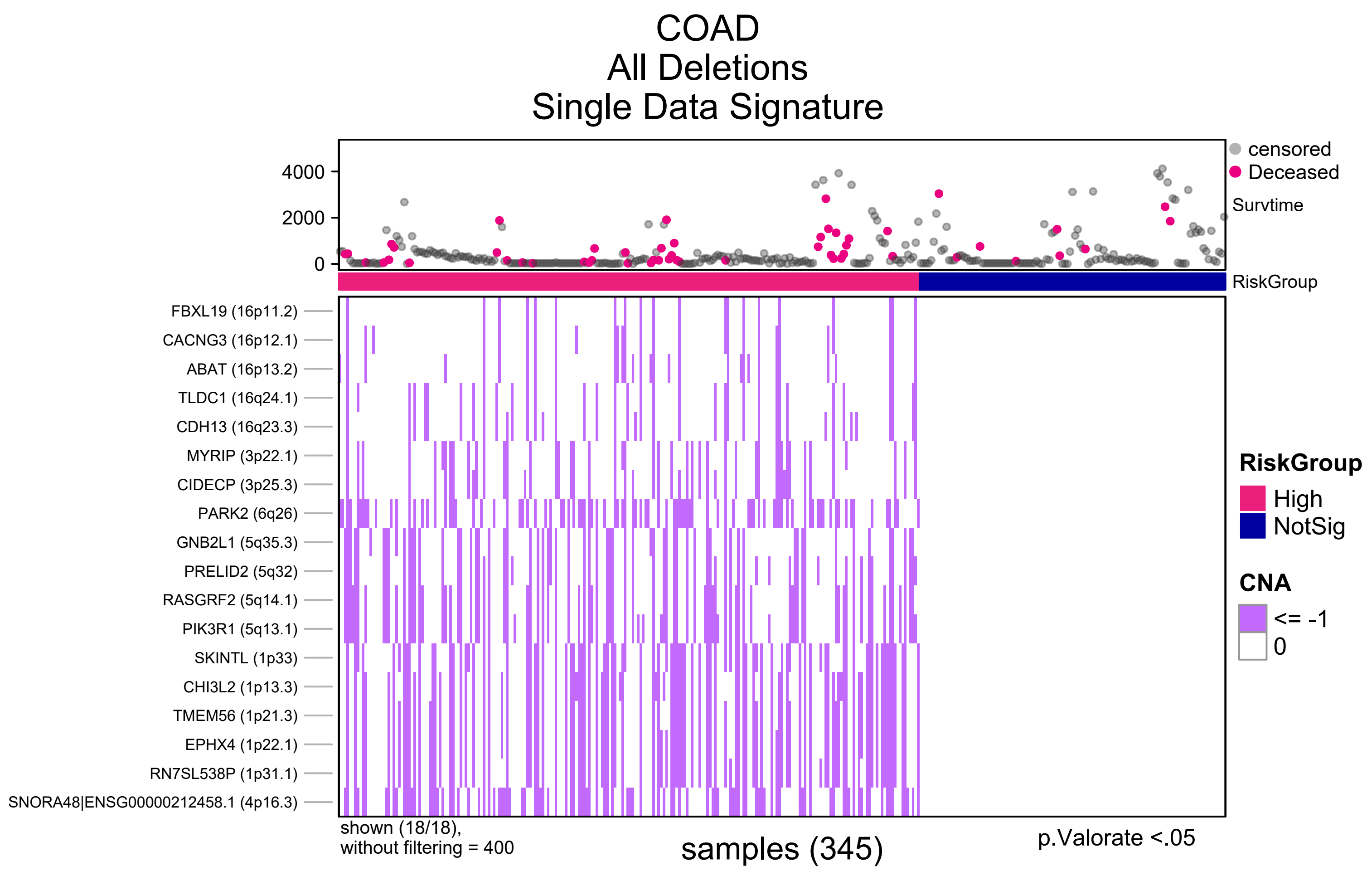
COAD
All Deletions
Single Data Signature
4000
2000
0
censored
Deceased
Survtime
RiskGroup
FBXL19 (16p11.2)
CACNG3 (16p12.1)
ABAT (16p13.2)
TLDC1 (16q24.1)
CDH13 (16q23.3)
MYRIP (3p22.1)
CIDECP (3p25.3)
PARK2 (6q26)
GNB2L1 (5q35.3)
PRELID2 (5q32)
RASGRF2 (5q14.1)
PIK3R1 (5q13.1)
SKINTL (1p33)
CHI3L2 (1p13.3)
TMEM56 (1p21.3)
EPHX4 (1p22.1)
RN7SL538P (1p31.1)
SNORA48|ENSG00000212458.1 (4p16.3)
RiskGroup
High
NotSig
CNA
<= -1
0
shown (18/18),
without filtering = 400
samples (345)
p.Valorate <.05

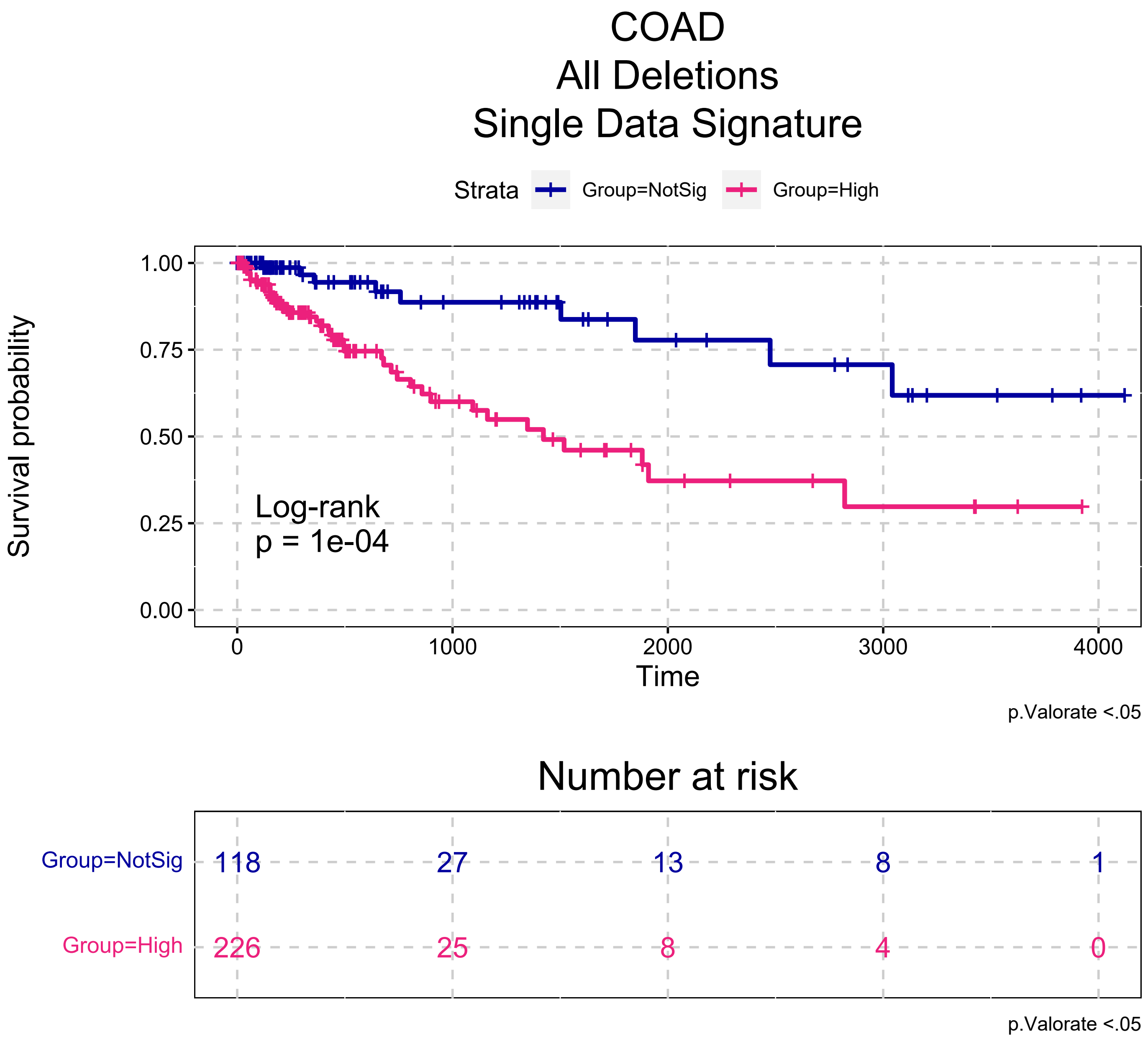

explanatory	beta	HR	L95	U95	p
High	1.35	3.84	1.86	7.95	0.00

n= 345, number of events =52
Score(logrank) test = 0

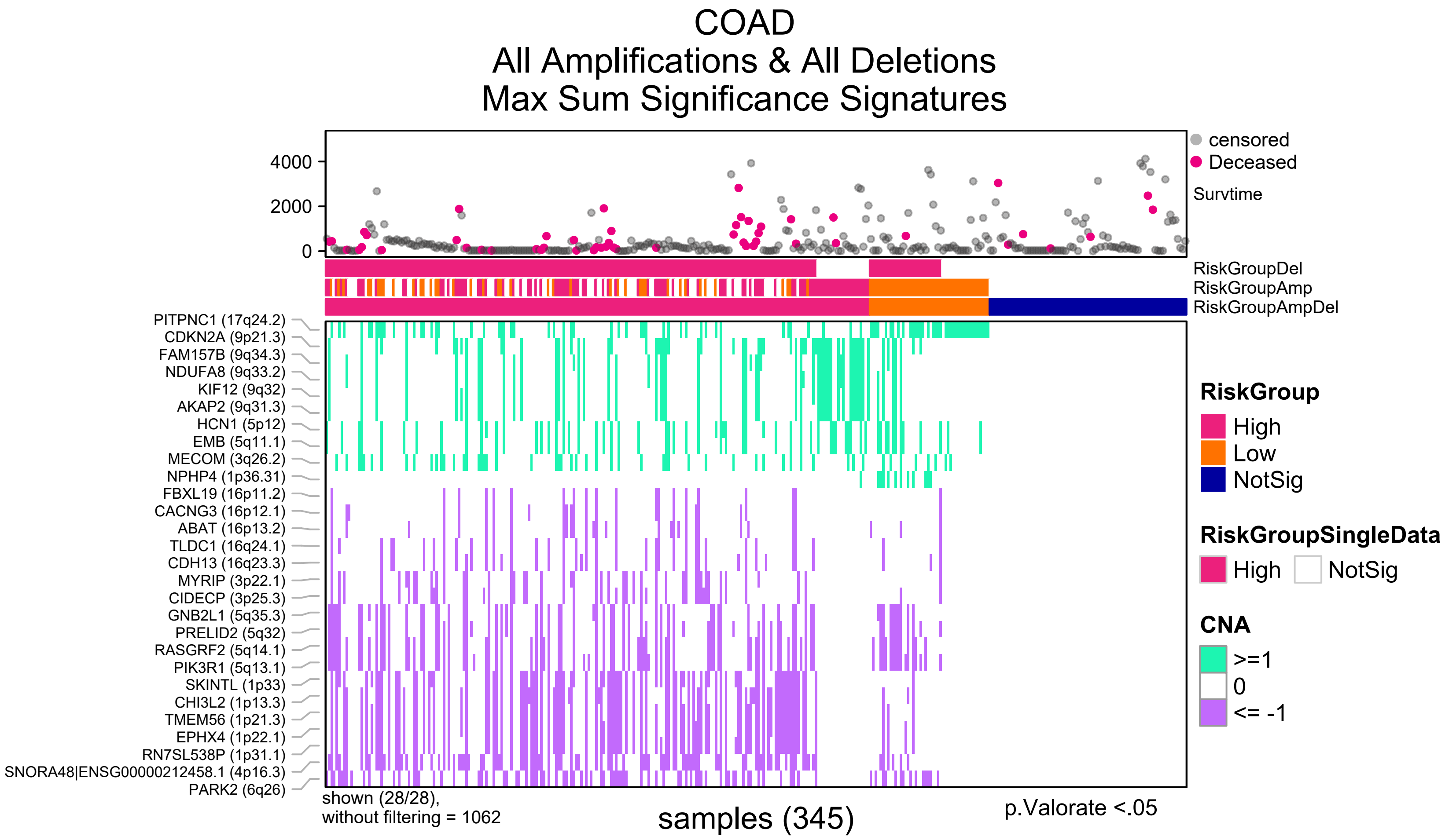

COAD
All Amplifications & All Deletions
Max Sum Significance Signatures
4000
2000
0
censored
Deceased
Survtime
RiskGroupDel
RiskGroupAmp
RiskGroupAmpDel
PITPNC1 (17q24.2)
CDKN2A (9p21.3)
FAM157B (9q34.3)
NDUFA8 (9q33.2)
KIF12 (9q32)
AKAP2 (9q31.3)
HCN1 (5p12)
EMB (5q11.1)
MECOM (3q26.2)
NPHP4 (1p36.31)
FBXL19 (16p11.2)
CACNG3 (16p12.1)
ABAT (16p13.2)
TLDC1 (16q24.1)
CDH13 (16q23.3)
MYRIP (3p22.1)
CIDECP (3p25.3)
GNB2L1 (5q35.3)
PRELID2 (5q32)
RASGRF2 (5q14.1)
PIK3R1 (5q13.1)
SKINTL (1p33)
CHI3L2 (1p13.3)
TMEM56 (1p21.3)
EPHX4 (1p22.1)
RN7SL538P (1p31.1)
SNORA48|ENSG00000212458.1 (4p16.3)
PARK2 (6q26)
RiskGroup
High
Low
NotSig
RiskGroupSingleData
High
NotSig
CNA
>=1
0
<= -1
shown (28/28),
without filtering = 1062
samples (345)
p.Valorate <.05

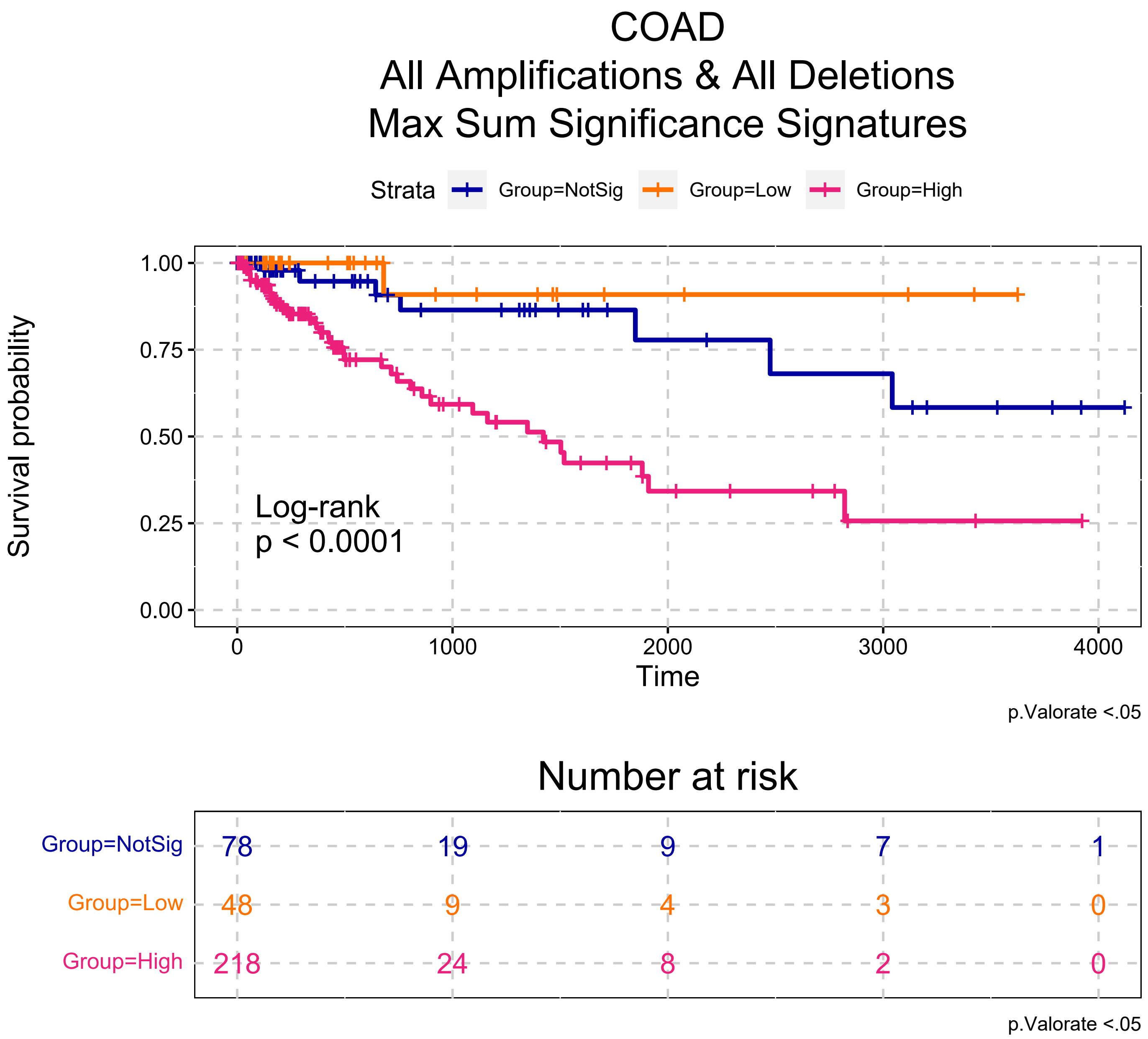

explanatory	beta	HR	L95	U95	p
Low	-1.30	0.27	0.03	2.22	0.22
High	1.32	3.75	1.66	8.45	0.00

n= 345, number of events =52
Score(logrank) test = p <.0001

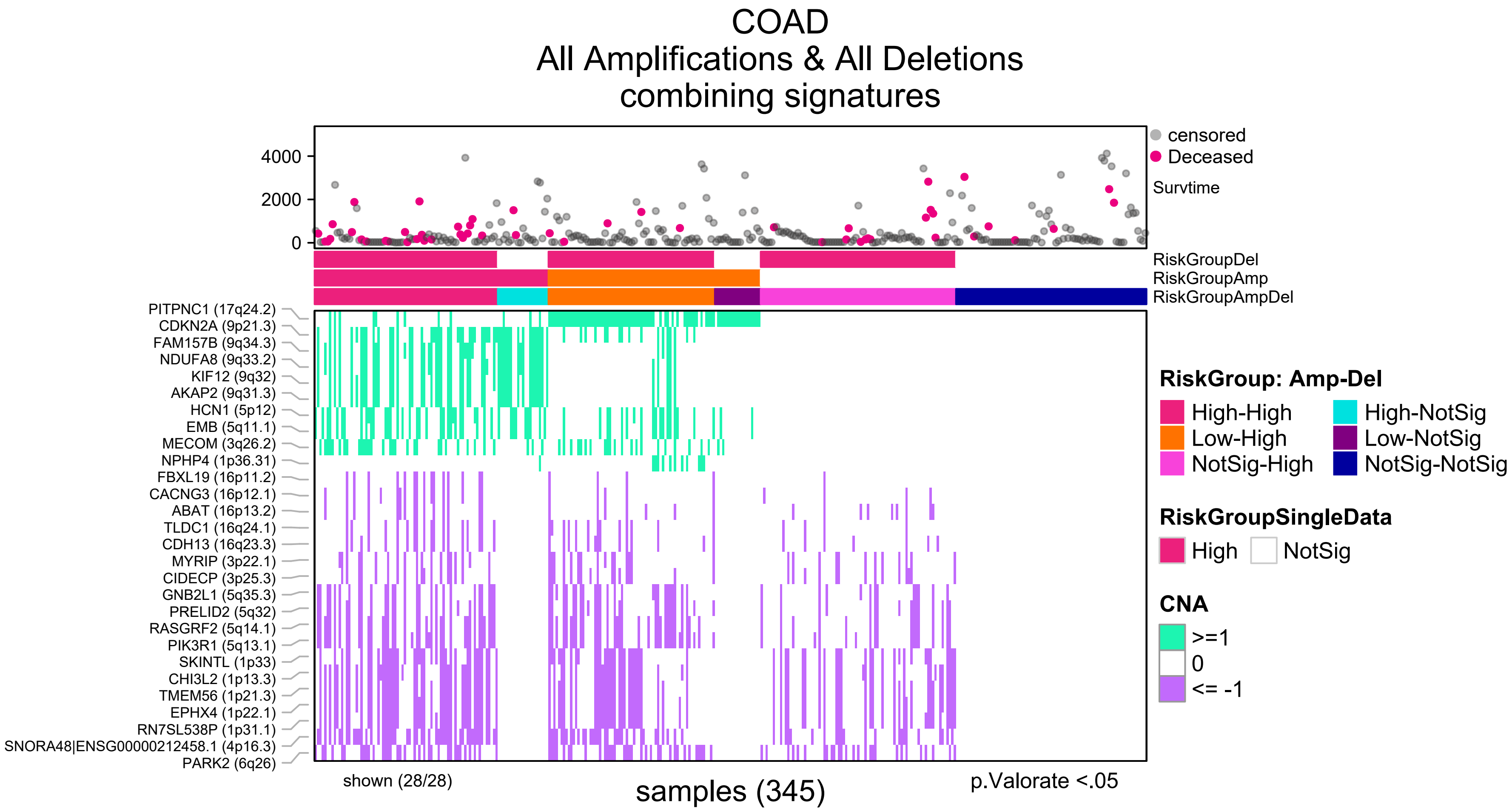

COAD
All Amplifications & All Deletions
combining signatures
4000
2000
0
censored
Deceased
Survtime
RiskGroupDel
RiskGroupAmp
RiskGroupAmpDel
PITPNC1 (17q24.2)
CDKN2A (9p21.3)
FAM157B (9q34.3)
NDUFA8 (9q33.2)
KIF12 (9q32)
AKAP2 (9q31.3)
HCN1 (5p12)
EMB (5q11.1)
MECOM (3q26.2)
NPHP4 (1p36.31)
FBXL19 (16p11.2)
CACNG3 (16p12.1)
ABAT (16p13.2)
TLDC1 (16q24.1)
CDH13 (16q23.3)
MYRIP (3p22.1)
CIDECP (3p25.3)
GNB2L1 (5q35.3)
PRELID2 (5q32)
RASGRF2 (5q14.1)
PIK3R1 (5q13.1)
SKINTL (1p33)
CHI3L2 (1p13.3)
TMEM56 (1p21.3)
EPHX4 (1p22.1)
RN7SL538P (1p31.1)
SNORA48|ENSG00000212458.1 (4p16.3)
PARK2 (6q26)
RiskGroup: Amp-Del
High-High
High-NotSig
Low-High
Low-NotSig
NotSig-High
NotSig-NotSig
RiskGroupSingleData
High
NotSig
CNA
>=1
0
<= -1
shown (28/28)
samples (345)
p.Valorate <.05

COAD
All Amplifications & All Deletions combining signatures

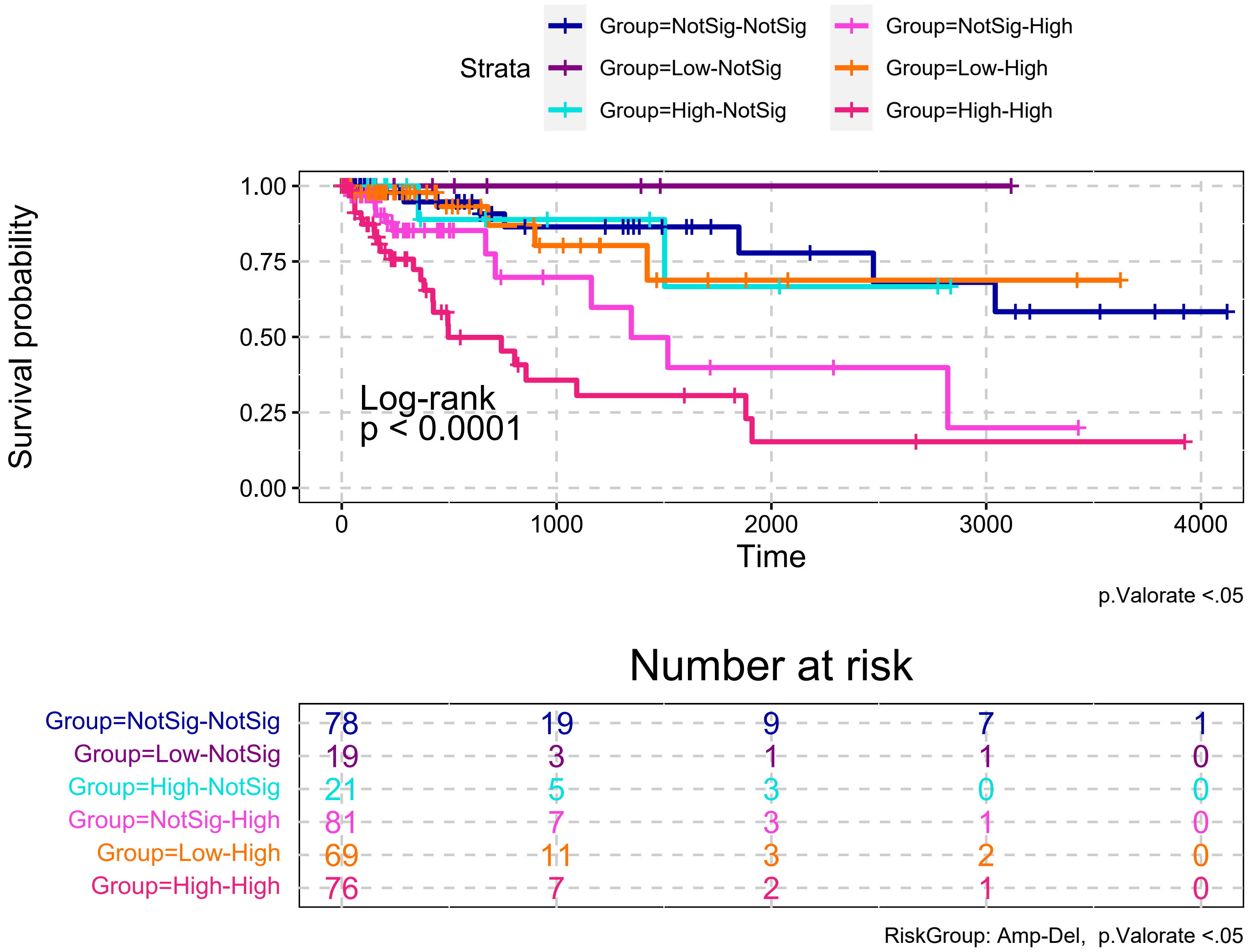

explanatory	beta	HR	L95	U95	p
Low-NotSig	-16.67	0.00	0.00	Inf	1.00
High-NotSig	0.07	1.07	0.22	5.19	0.93
NotSig-High	1.17	3.21	1.26	8.14	0.01
Low-High	0.11	1.12	0.35	3.55	0.85
High-High	1.85	6.37	2.72	14.92	0.00

n= 345, number of events =52
Score(logrank) test = p <.0001

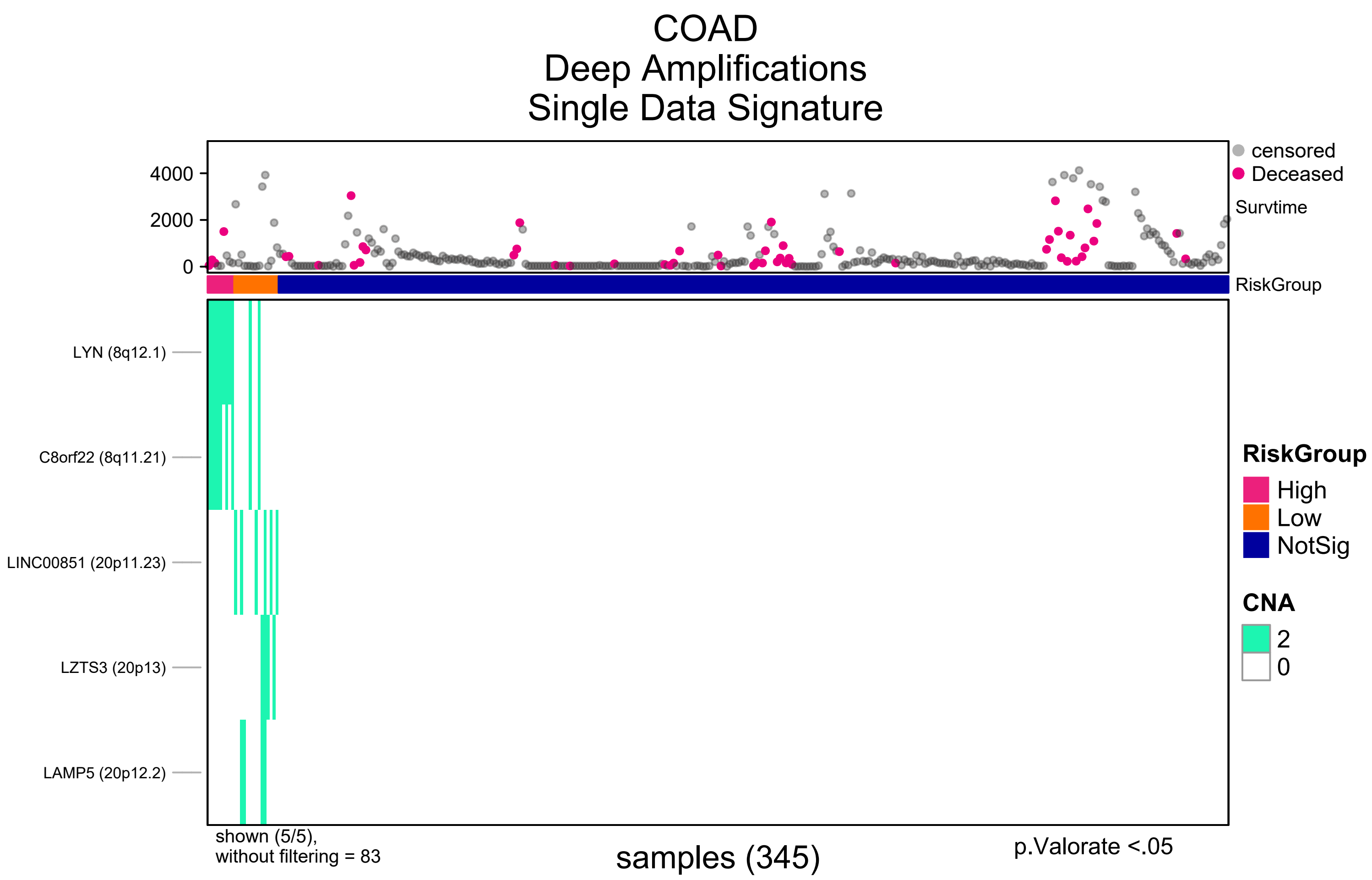
COAD
Deep Amplifications
Single Data Signature
4000
2000
0
censored
Deceased
Survtime
RiskGroup
LYN (8q12.1)
C8orf22 (8q11.21)
LINC00851 (20p11.23)
LZTS3 (20p13)
LAMP5 (20p12.2)
RiskGroup
High
Low
NotSig
CNA
2
0
shown (5/5),
without filtering = 83
samples (345)
p.Valorate <.05

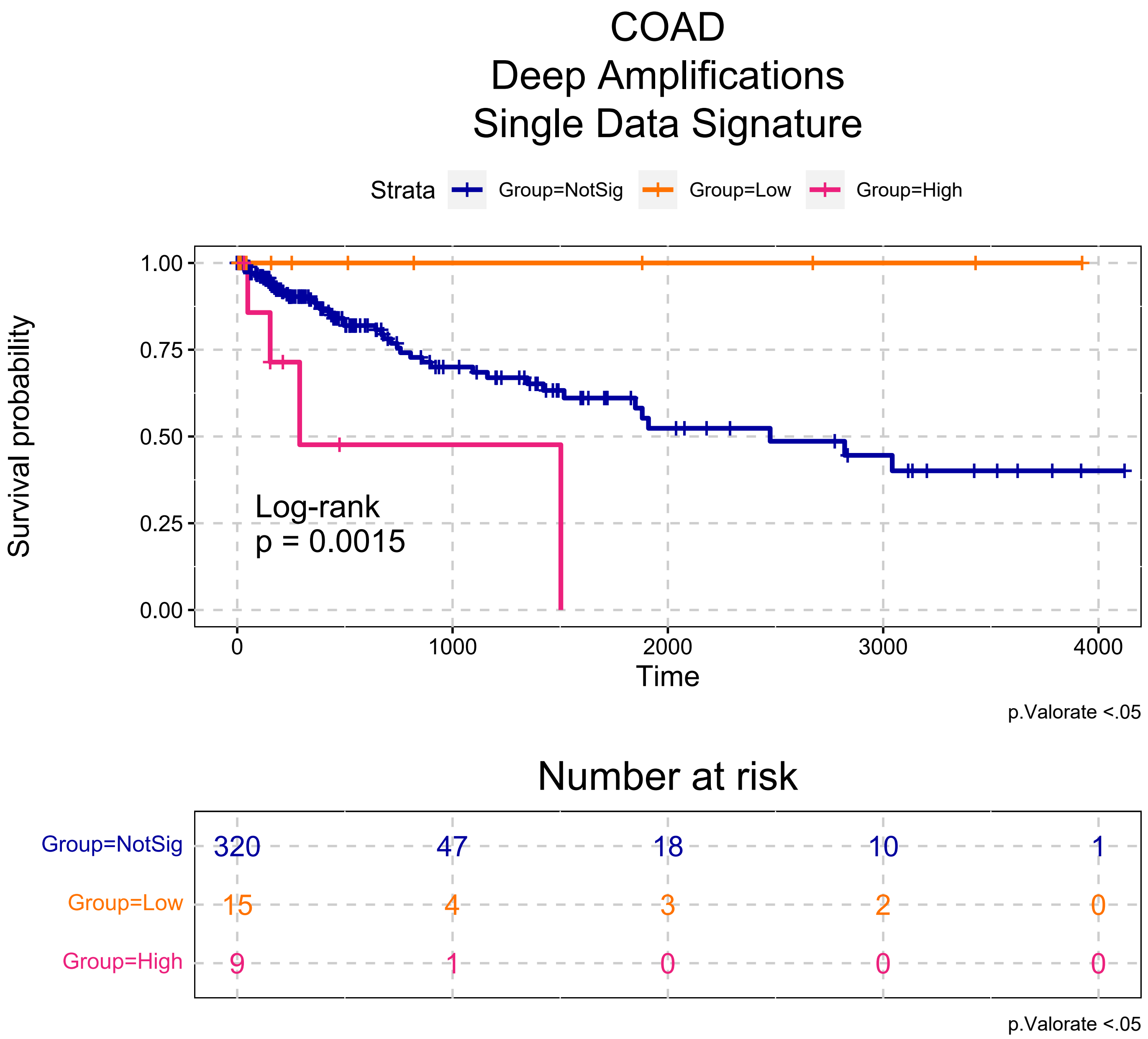

explanatory	beta	HR	L95	U95	p
Low	-17.63	0.00	0.00	Inf	1.00
High	1.41	4.11	1.47	11.52	0.01

n= 345, number of events =52
Score(logrank) test = 0.001

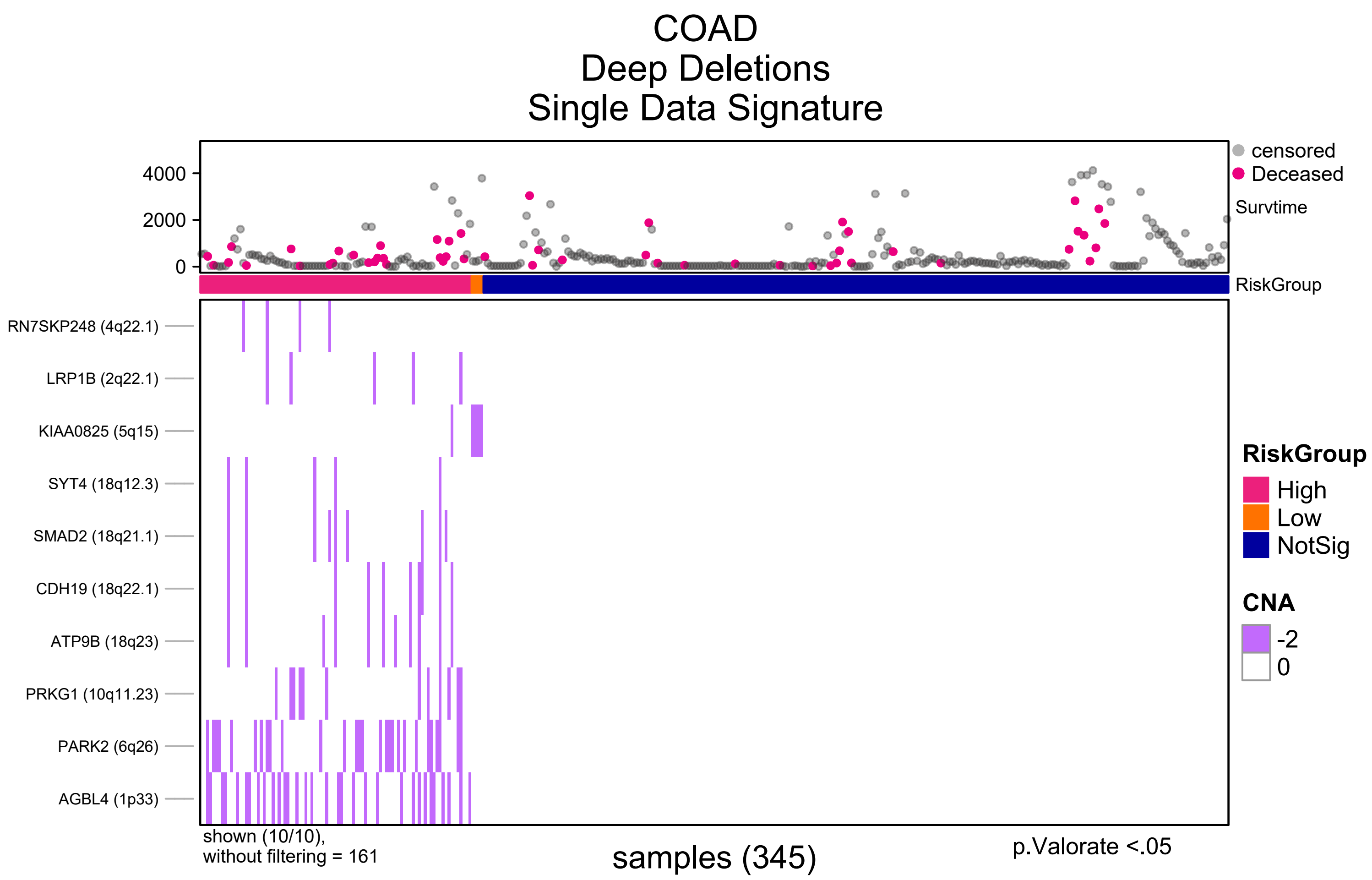

COAD
Deep Deletions
Single Data Signature
4000
2000
0
censored
Deceased
Survtime
RiskGroup
RN7SKP248 (4q22.1)
LRP1B (2q22.1)
KIAA0825 (5q15)
SYT4 (18q12.3)
SMAD2 (18q21.1)
CDH19 (18q22.1)
ATP9B (18q23)
PRKG1 (10q11.23)
PARK2 (6q26)
AGBL4 (1p33)
RiskGroup
High
Low
NotSig
CNA
-2
0
shown (10/10),
without filtering = 161
samples (345)
p.Valorate <.05

COAD
Deep Deletions
Single Data Signature

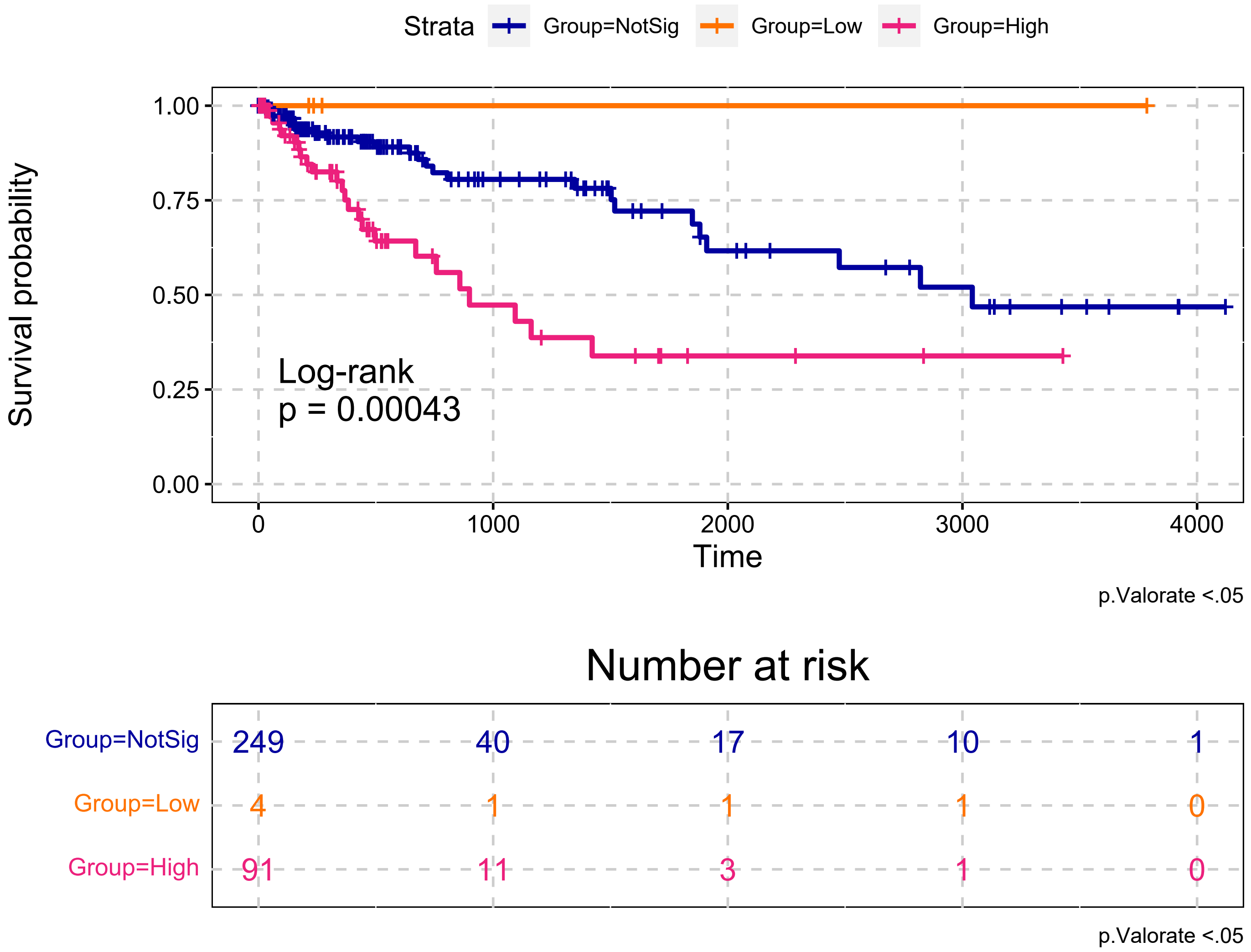

explanatory	beta	HR	L95	U95	p
Low	-15.89	0.00	0.00	Inf	1.00
High	1.01	2.75	1.58	4.77	0.00

n= 345, number of events =52
Score(logrank) test = 0

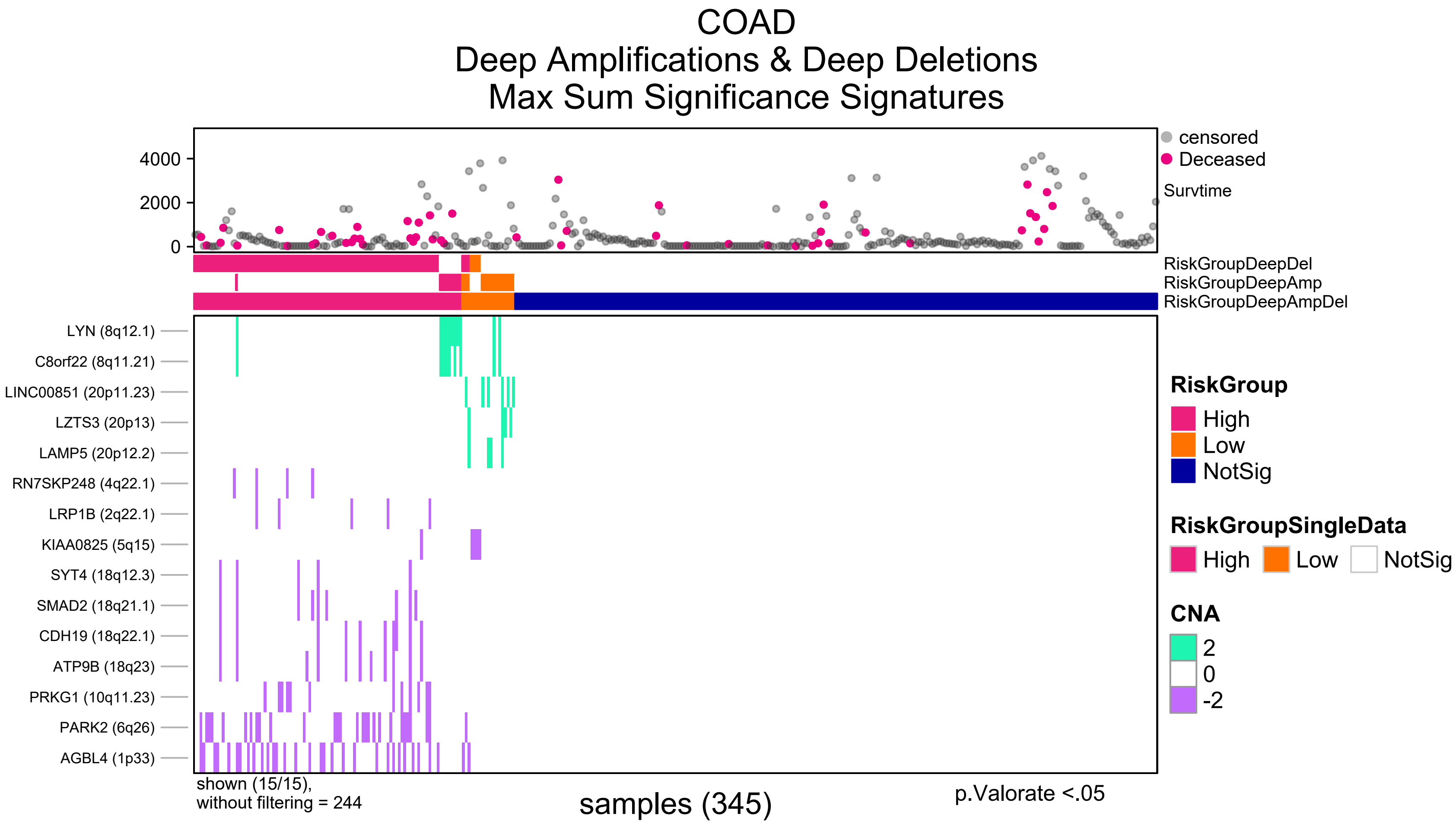
COAD
Deep Amplifications & Deep Deletions
Max Sum Significance Signatures
4000
2000
0
censored
Deceased
Survtime
RiskGroupDeepDel
RiskGroupDeepAmp
RiskGroupDeepAmpDel
LYN (8q12.1)
C8orf22 (8q11.21)
LINC00851 (20p11.23)
LZTS3 (20p13)
LAMP5 (20p12.2)
RN7SKP248 (4q22.1)
LRP1B (2q22.1)
KIAA0825 (5q15)
SYT4 (18q12.3)
SMAD2 (18q21.1)
CDH19 (18q22.1)
ATP9B (18q23)
PRKG1 (10q11.23)
PARK2 (6q26)
AGBL4 (1p33)
RiskGroup
High
Low
NotSig
RiskGroupSingleData
High
Low
NotSig
CNA
2
0
-2
shown (15/15),
without filtering = 244
samples (345)
p.Valorate <.05

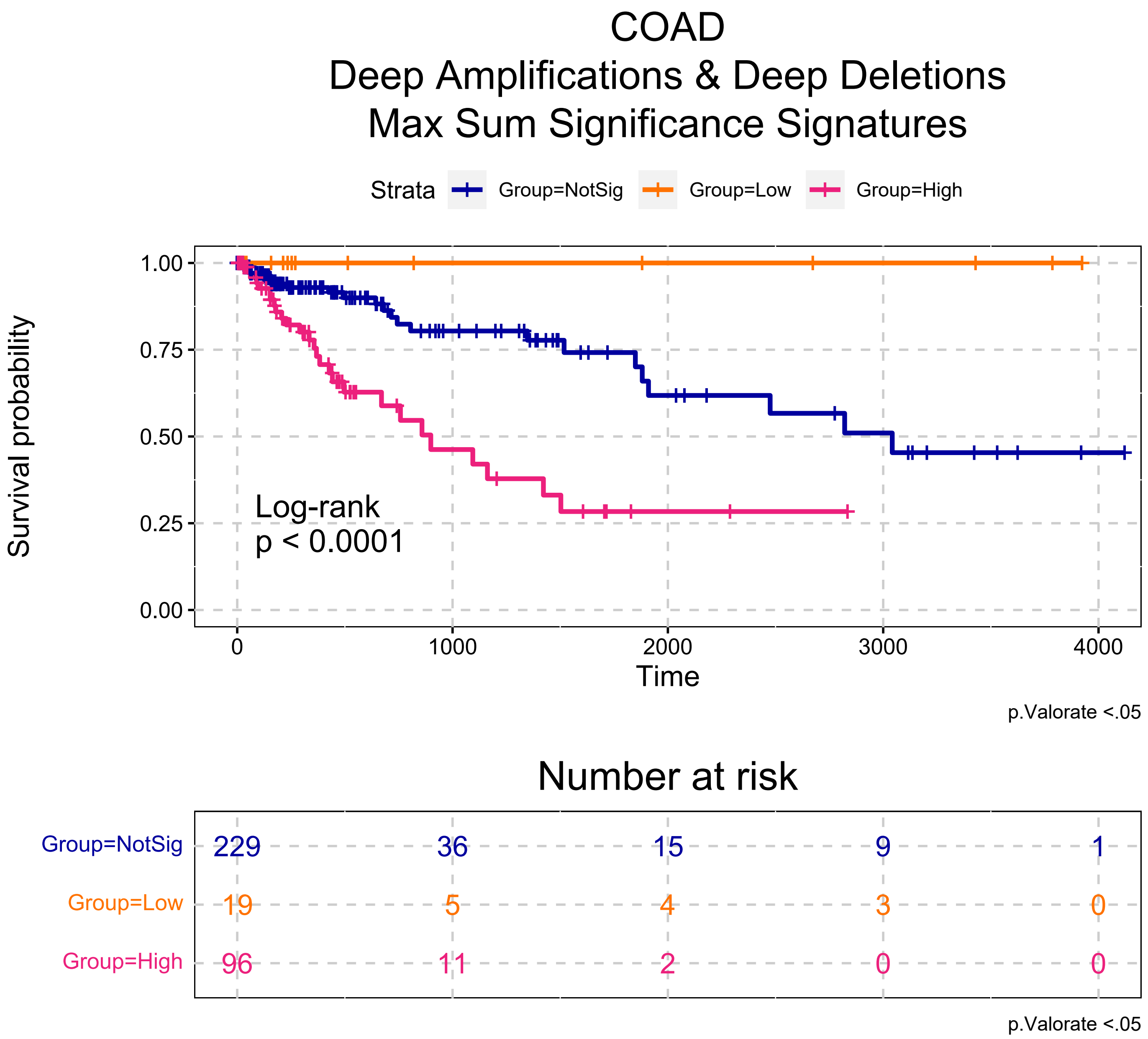

explanatory	beta	HR	L95	U95	p
Low	-18.03	0.00	0.00	Inf	1.00
High	1.18	3.25	1.85	5.69	0.00

n= 345, number of events =52
Score(logrank) test = p <.0001

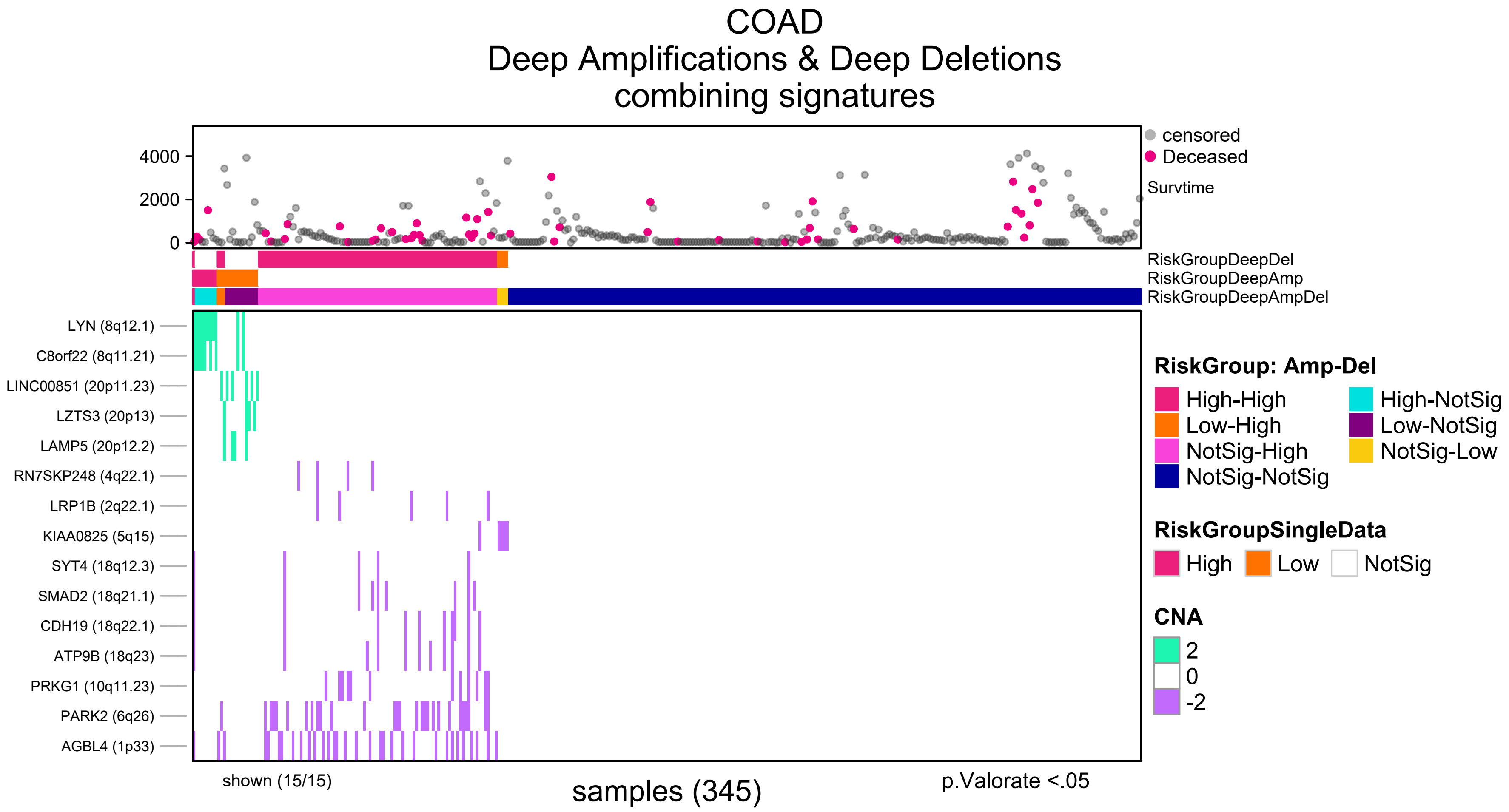
COAD
Deep Amplifications & Deep Deletions
combining signatures
4000
2000
0
censored
Deceased
Survtime
RiskGroupDeepDel
RiskGroupDeepAmp
RiskGroupDeepAmpDel
LYN (8q12.1)
C8orf22 (8q11.21)
LINC00851 (20p11.23)
LZTS3 (20p13)
LAMP5 (20p12.2)
RN7SKP248 (4q22.1)
LRP1B (2q22.1)
KIAA0825 (5q15)
SYT4 (18q12.3)
SMAD2 (18q21.1)
CDH19 (18q22.1)
ATP9B (18q23)
PRKG1 (10q11.23)
PARK2 (6q26)
AGBL4 (1p33)
RiskGroup: Amp-Del
High-High
Low-High
NotSig-High
NotSig-NotSig
High-NotSig
Low-NotSig
NotSig-Low
RiskGroupSingleData
High
Low
NotSig
CNA
2
0
-2
shown (15/15)
samples (345)
p.Valorate <.05

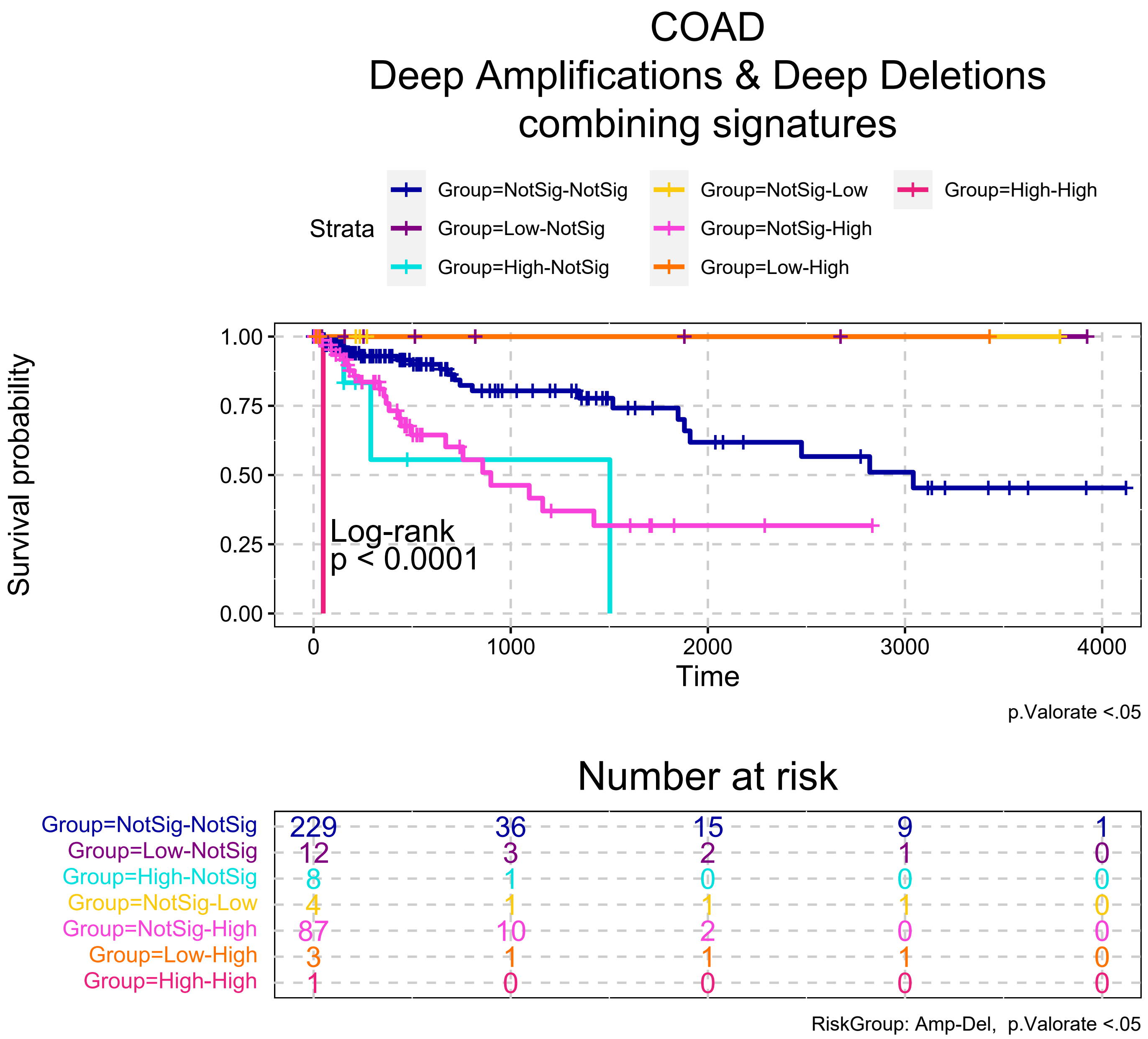

explanatory	beta	HR	L95	U95	p
Low-NotSig	-16.04	0.00	0.00	Inf	0.99
High-NotSig	1.55	4.72	1.46	15.28	0.01
NotSig-Low	-16.07	0.00	0.00	Inf	1.00
NotSig-High	1.10	3.01	1.72	5.27	0.00
Low-High	-16.13	0.00	0.00	Inf	1.00
High-High	4.93	138.77	14.38	1338.96	0.00

n= 345, number of events =52
Score(logrank) test = p <.0001
