# Supplementary material for: Systematic Modeling of Risk-Associated Copy Number Alterations in Cancer
Source: Int J Mol Sci. 2024 Sep 27;25(19):10455. doi: 10.3390/ijms251910455 (PMC11477427; doi:10.3390/ijms251910455)
Supplement: Supplementary file 1 [file ijms-25-10455-s001.zip › DLBCSignatureV12-sinSombreado.pdf]

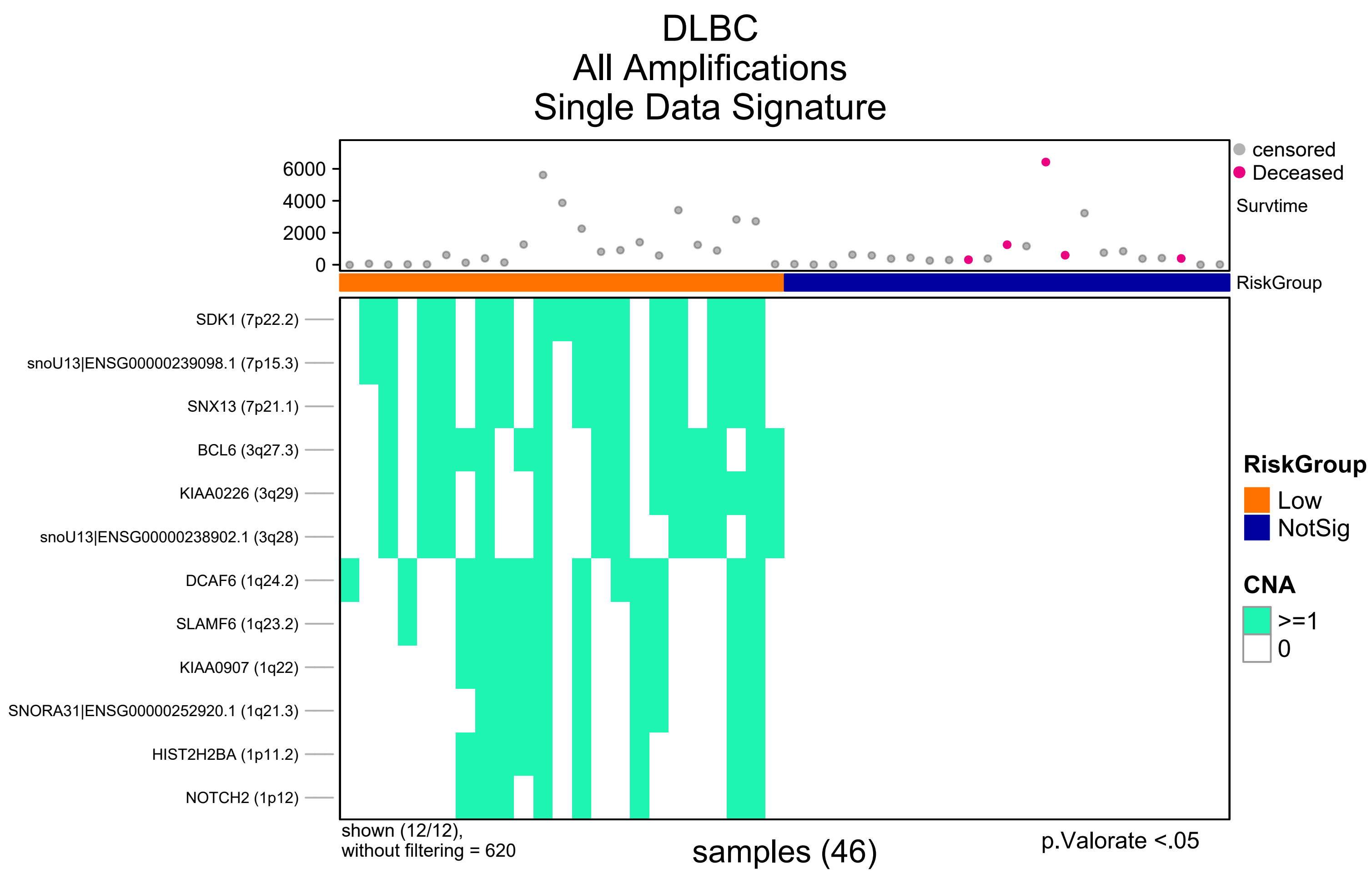

DLBC
All Amplifications
Single Data Signature
6000
4000
2000
0
censored
Deceased
Survtime
RiskGroup
SDK1 (7p22.2)
snoU13|ENSG00000239098.1 (7p15.3)
SNX13 (7p21.1)
BCL6 (3q27.3)
KIAA0226 (3q29)
snoU13|ENSG00000238902.1 (3q28)
DCAF6 (1q24.2)
SLAMF6 (1q23.2)
KIAA0907 (1q22)
SNORA31|ENSG00000252920.1 (1q21.3)
HIST2H2BA (1p11.2)
NOTCH2 (1p12)
RiskGroup
Low
NotSig
CNA
>=1
0
shown (12/12),
without filtering = 620
samples (46)
p.Valorate <.05

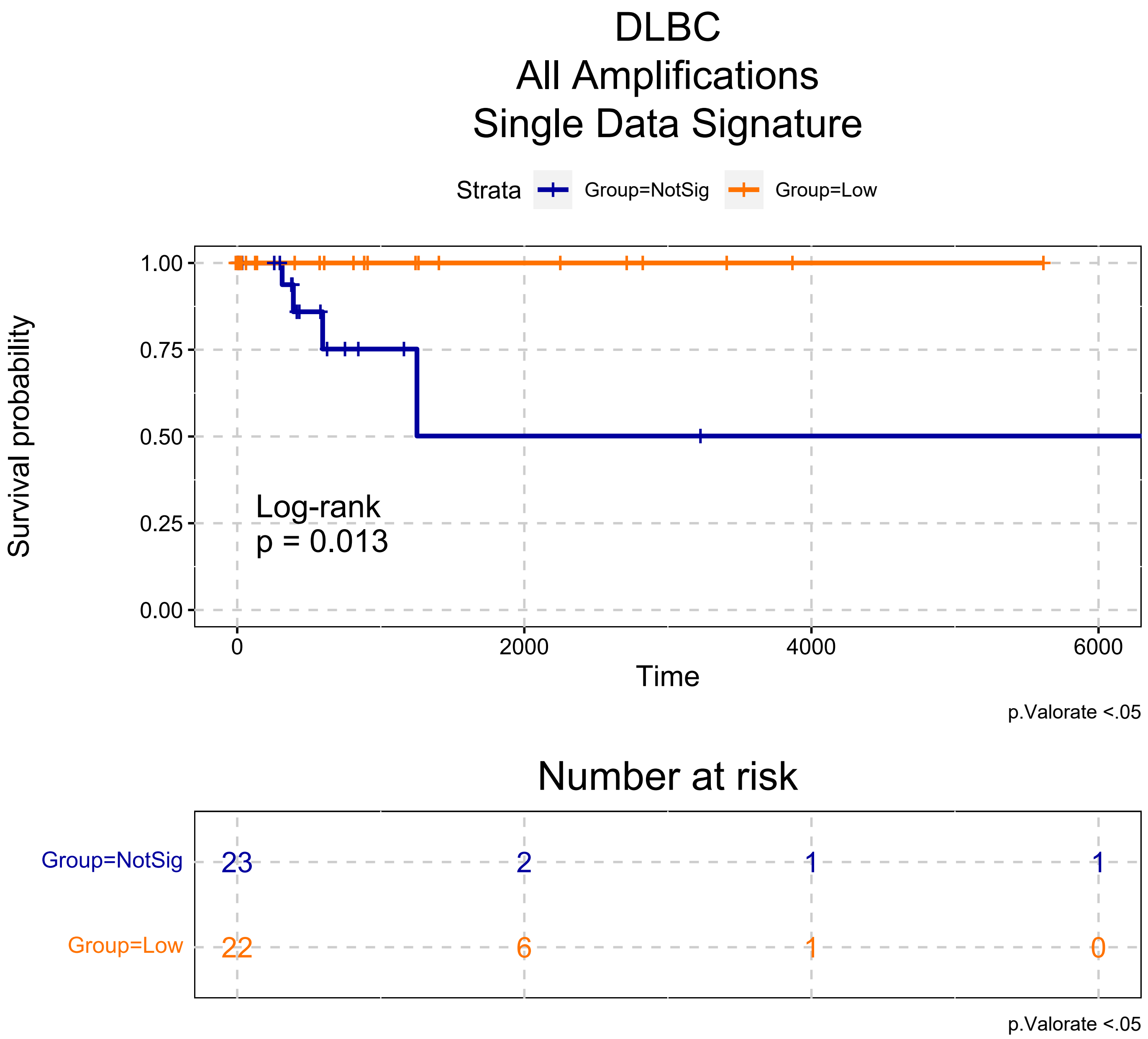

explanatory	beta	HR	L95	U95	p
Low	-21.77	0.00	0.00	Inf	1.00

n= 46, number of events =5
Score(logrank) test = 0.013

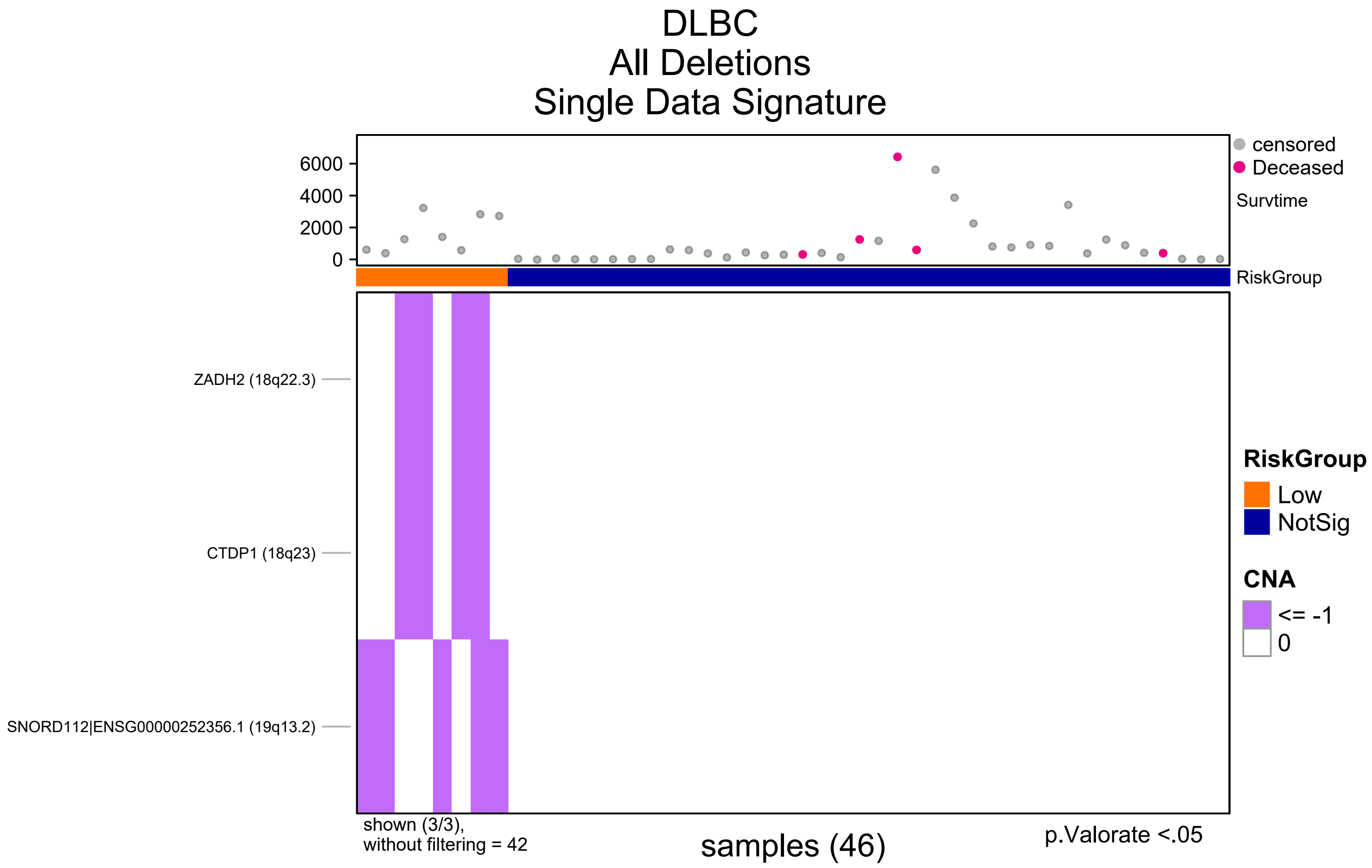

DLBC
All Deletions
Single Data Signature
6000
4000
2000
0
censored
Deceased
Survtime
RiskGroup
ZADH2 (18q22.3)
CTDP1 (18q23)
SNORD112|ENSG00000252356.1 (19q13.2)
RiskGroup
Low
NotSig
CNA
<= -1
0
shown (3/3),
without filtering = 42
samples (46)
p.Valorate <.05

DLBC
All Deletions
Single Data Signature

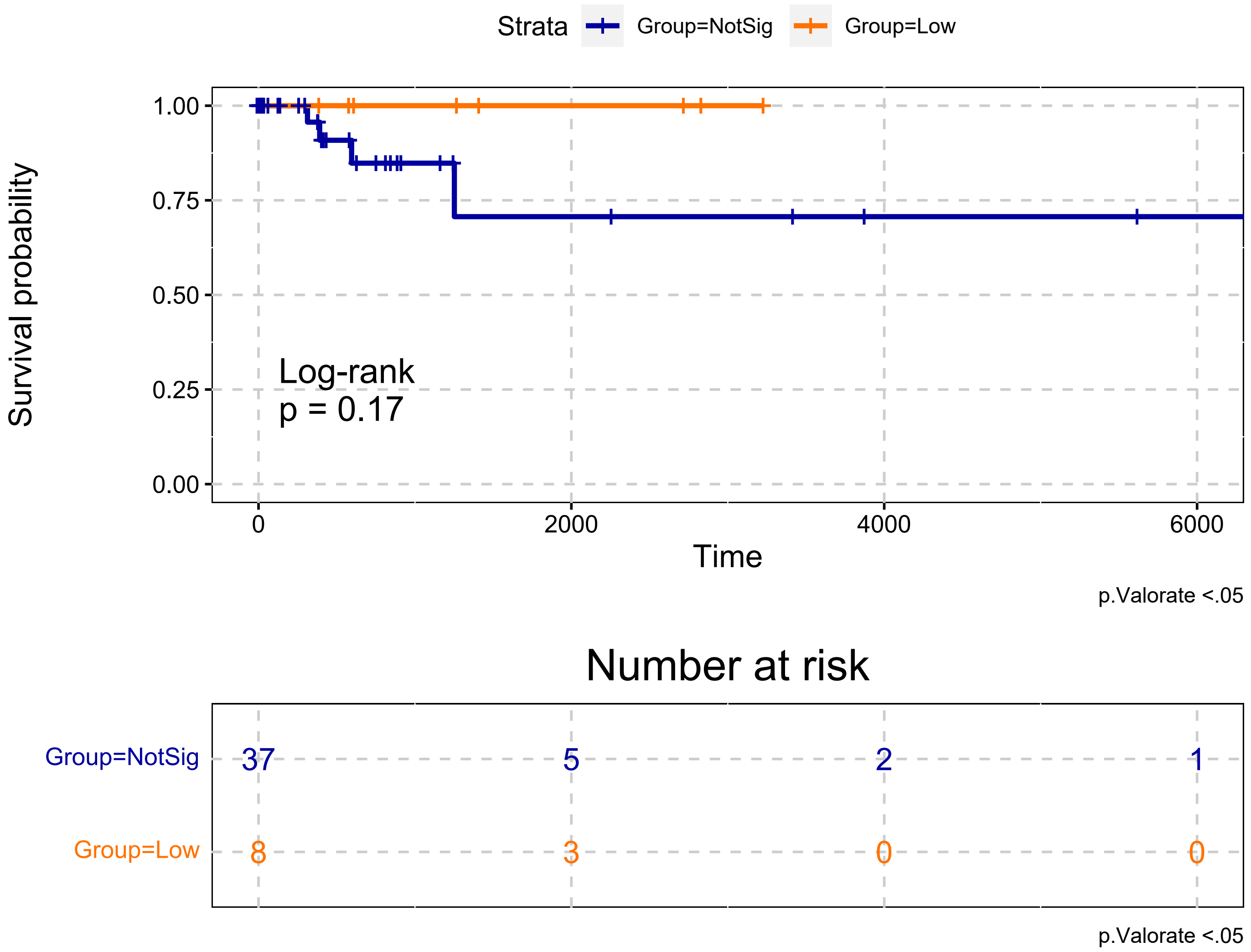

explanatory	beta	HR	L95	U95	p
Low	-19.69	0.00	0.00	Inf	1.00

n= 46, number of events =5
Score(logrank) test = 0.169

DLBC
All Amplifications & All Deletions
Max Sum Significance Signatures

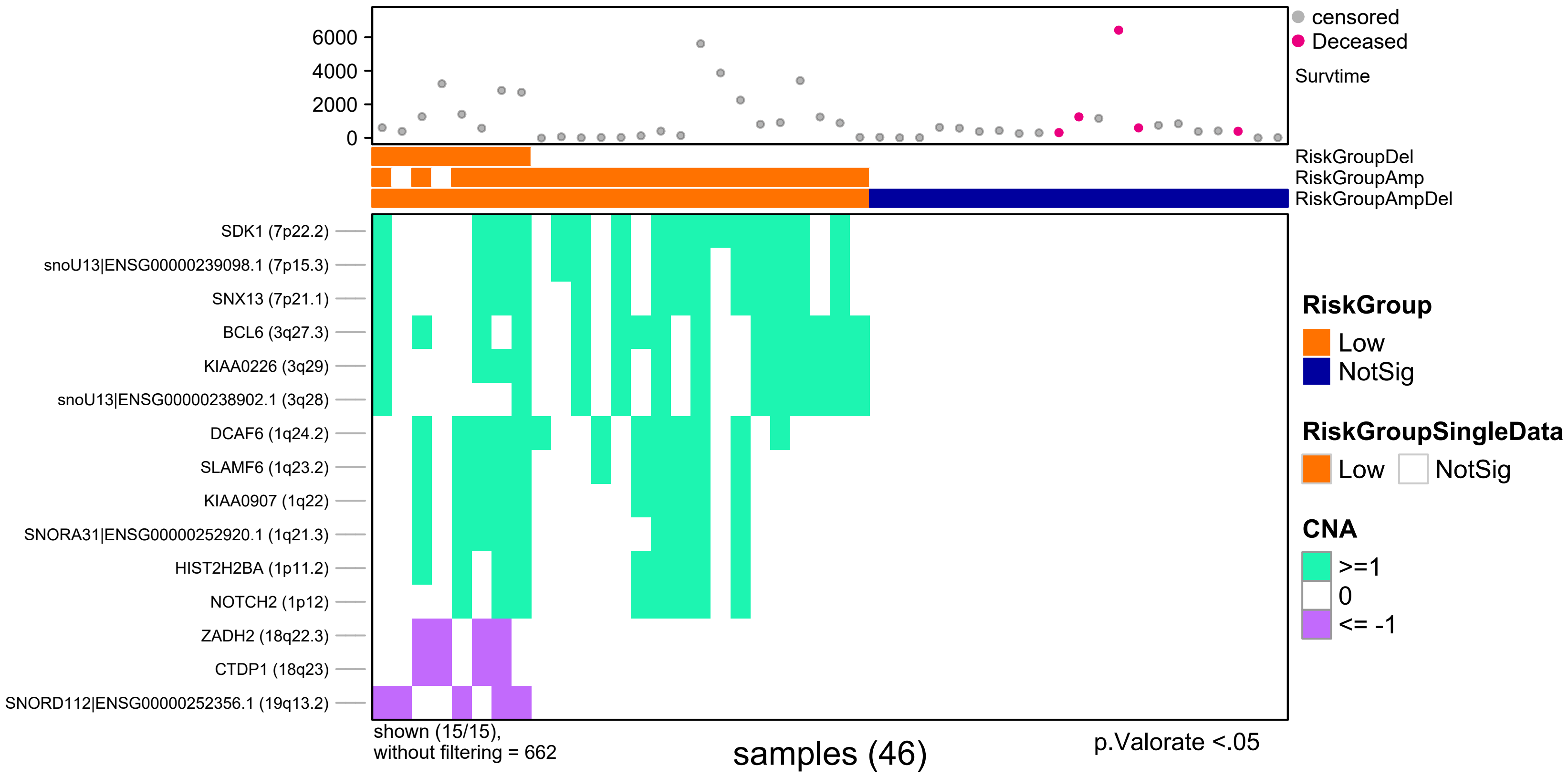

DLBC
All Amplifications & All Deletions
Max Sum Significance Signatures

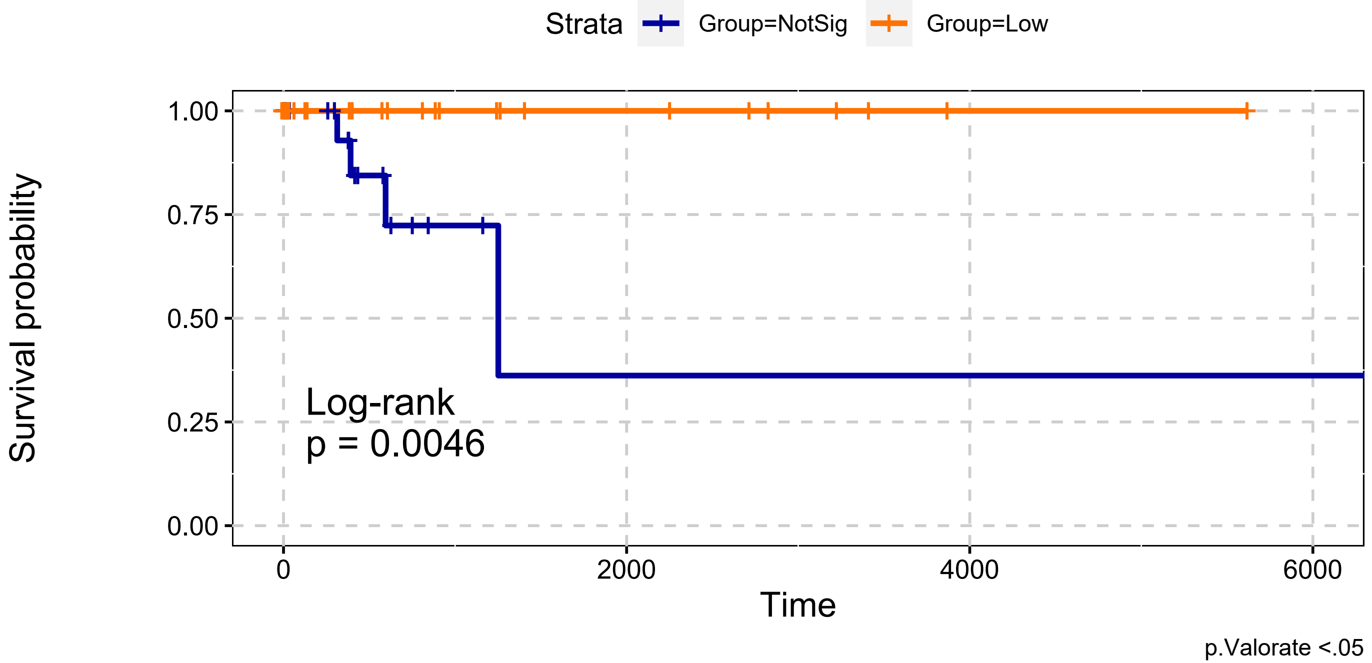

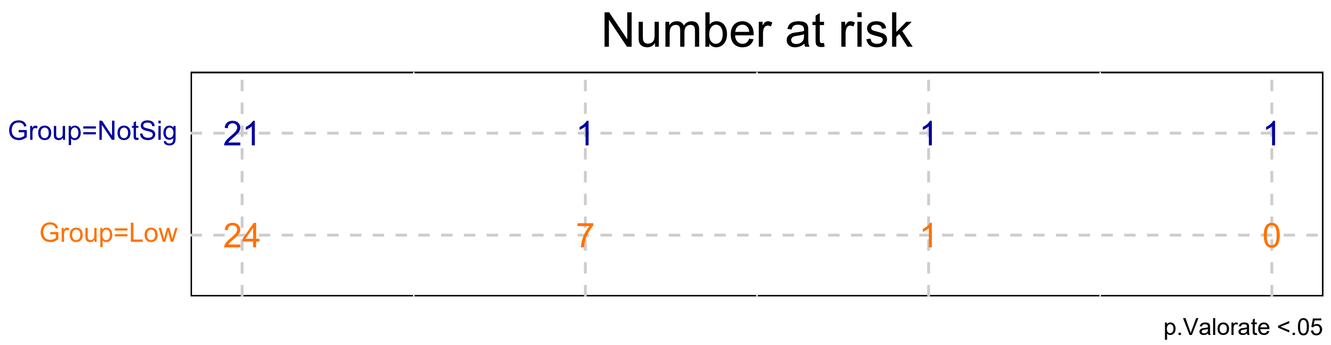

explanatory	beta	HR	L95	U95	p
Low	-22.26	0.00	0.00	Inf	1.00

n= 46, number of events =5
Score(logrank) test = 0.005

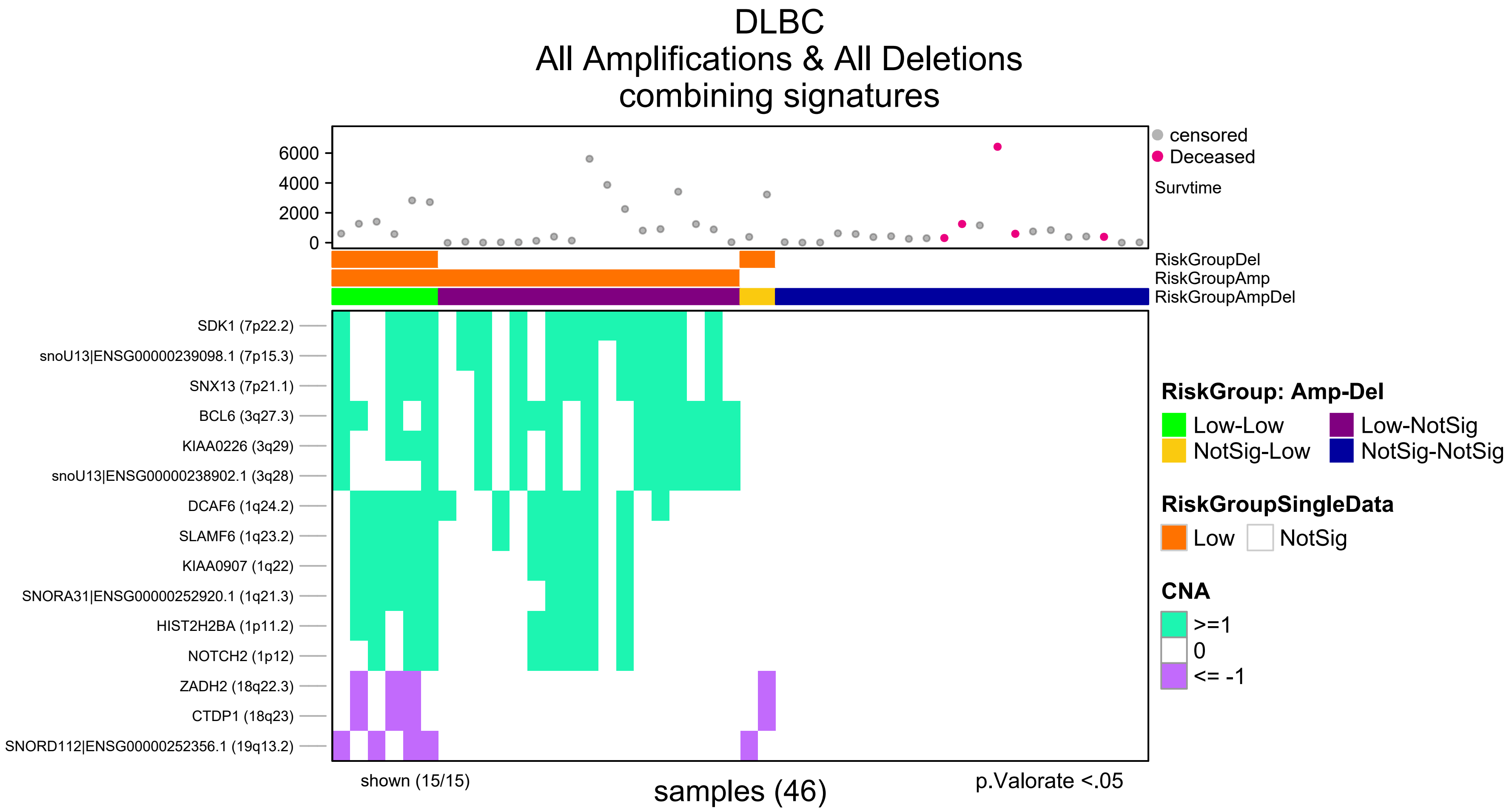
DLBC
All Amplifications & All Deletions
combining signatures
6000
4000
2000
0
censored
Deceased
Survtime
RiskGroupDel
RiskGroupAmp
RiskGroupAmpDel
SDK1 (7p22.2)
snoU13|ENSG00000239098.1 (7p15.3)
SNX13 (7p21.1)
BCL6 (3q27.3)
KIAA0226 (3q29)
snoU13|ENSG00000238902.1 (3q28)
DCAF6 (1q24.2)
SLAMF6 (1q23.2)
KIAA0907 (1q22)
SNORA31|ENSG00000252920.1 (1q21.3)
HIST2H2BA (1p11.2)
NOTCH2 (1p12)
ZADH2 (18q22.3)
CTDP1 (18q23)
SNORD112|ENSG00000252356.1 (19q13.2)
RiskGroup: Amp-Del
Low-Low
Low-NotSig
NotSig-Low
NotSig-NotSig
RiskGroupSingleData
Low
NotSig
CNA
>=1
0
<= -1
shown (15/15)
samples (46)
p.Valorate <.05

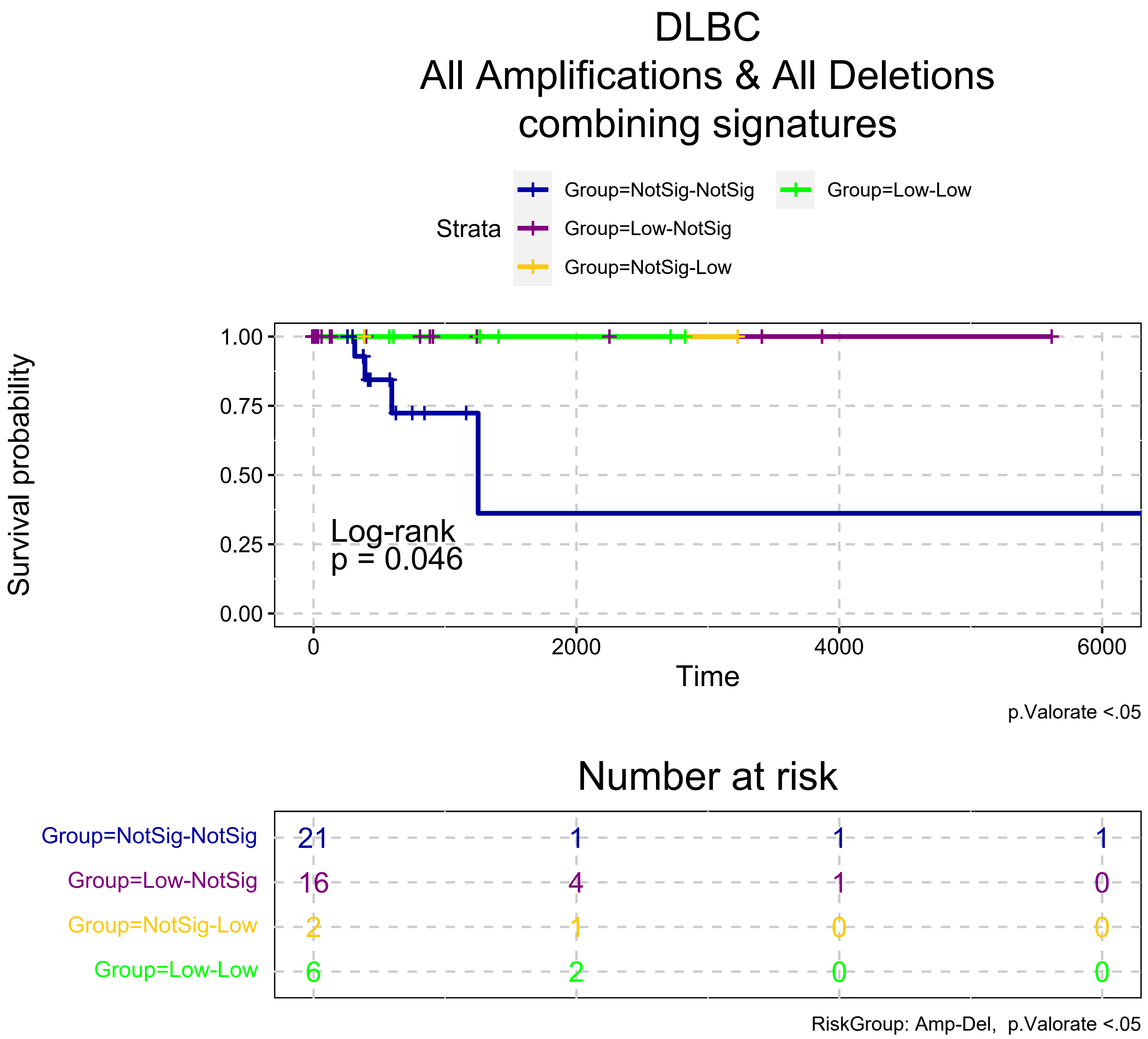

explanatory	beta	HR	L95	U95	p
Low-NotSig	-22.22	0.00	0.00	Inf	1.00
NotSig-Low	-22.30	0.00	0.00	Inf	1.00
Low-Low	-22.30	0.00	0.00	Inf	1.00

n= 46, number of events =5
Score(logrank) test = 0.046
