# Supplementary material for: Systematic Modeling of Risk-Associated Copy Number Alterations in Cancer
Source: Int J Mol Sci. 2024 Sep 27;25(19):10455. doi: 10.3390/ijms251910455 (PMC11477427; doi:10.3390/ijms251910455)
Supplement: Supplementary file 1 [file ijms-25-10455-s001.zip › ESCASignatureV12-sinSombreado.pdf]

ESCA
All Amplifications
Single Data Signature

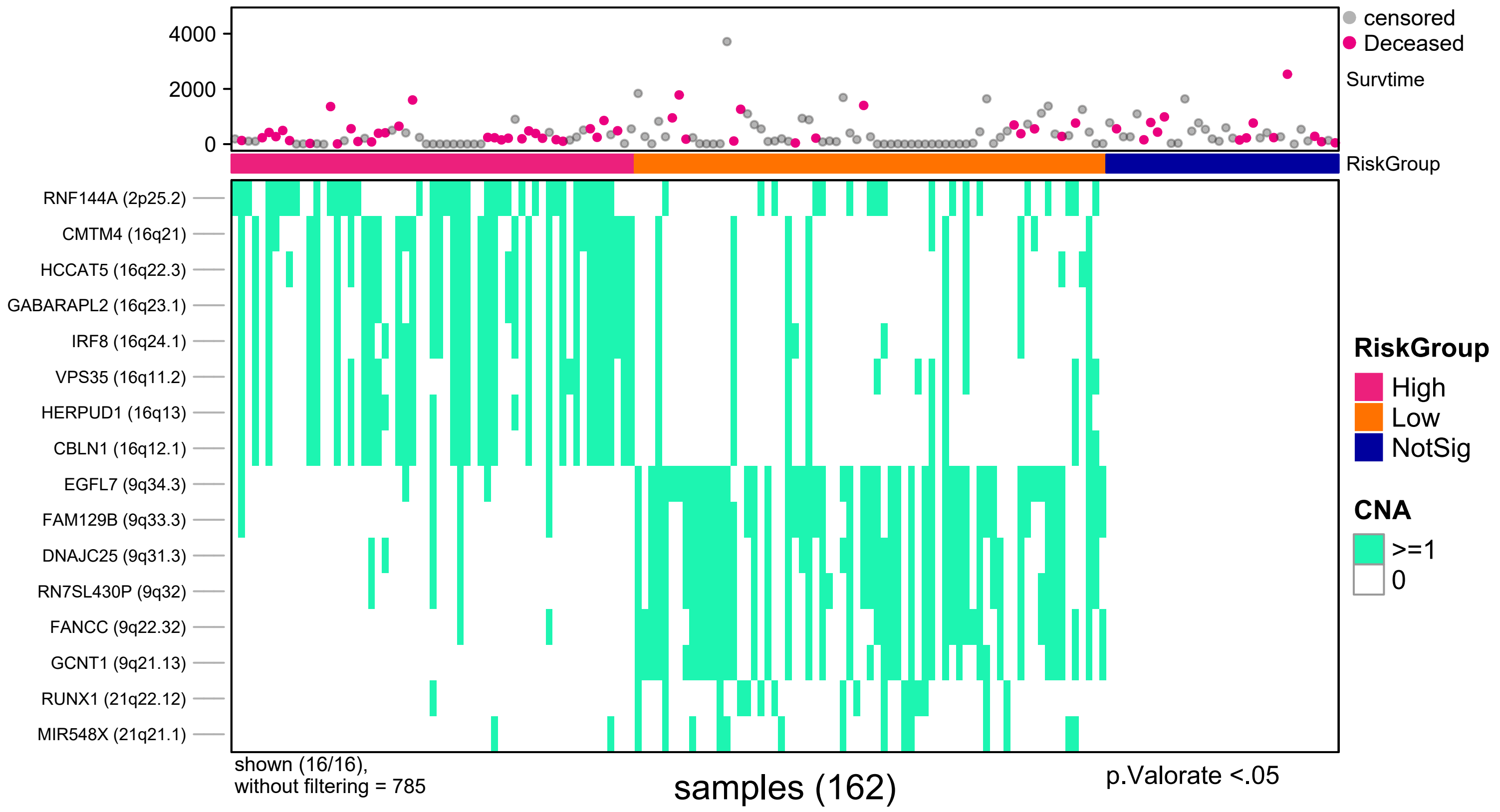

ESCA
All Amplifications
Single Data Signature

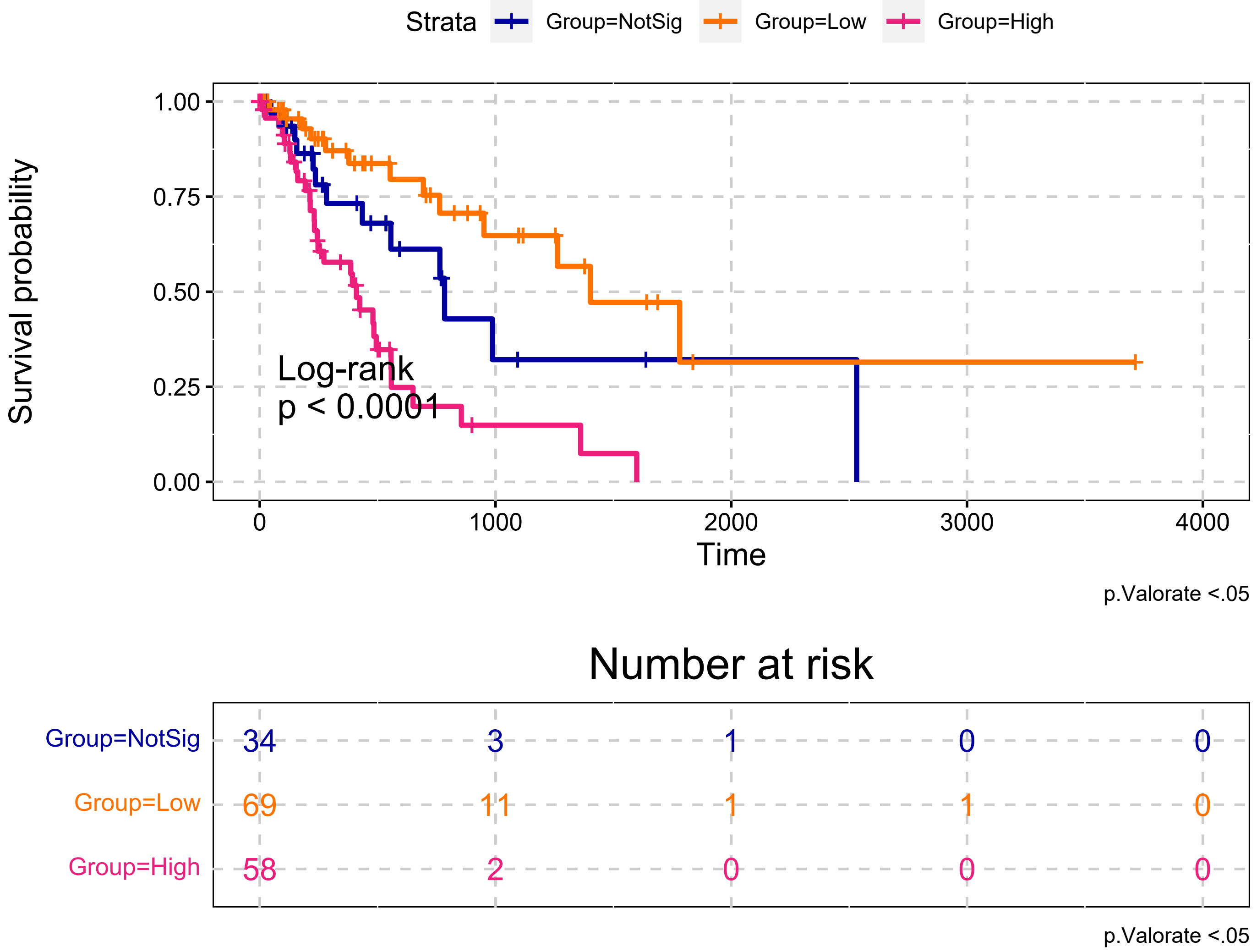

explanatory	beta	HR	L95	U95	p
Low	-0.65	0.52	0.24	1.13	0.10
High	0.87	2.39	1.23	4.66	0.01

n= 162, number of events =56
Score(logrank) test = p <.0001

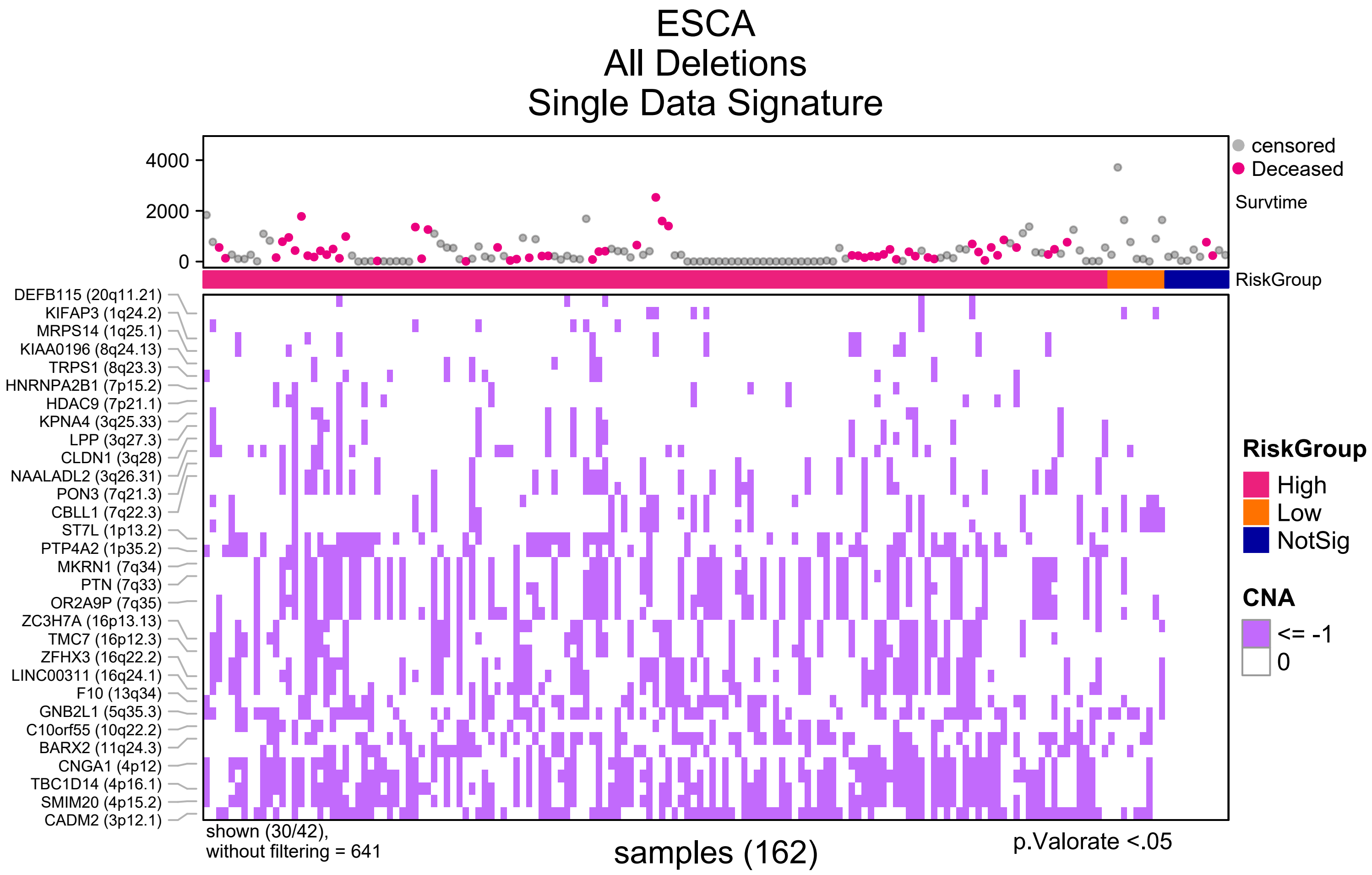
ESCA
All Deletions
Single Data Signature
4000
2000
0
censored
Deceased
Survtime
RiskGroup
DEFB115 (20q11.21)
KIFAP3 (1q24.2)
MRPS14 (1q25.1)
KIAA0196 (8q24.13)
TRPS1 (8q23.3)
HNRNPA2B1 (7p15.2)
HDAC9 (7p21.1)
KPNA4 (3q25.33)
LPP (3q27.3)
CLDN1 (3q28)
NAALADL2 (3q26.31)
PON3 (7q21.3)
CBLL1 (7q22.3)
ST7L (1p13.2)
PTP4A2 (1p35.2)
MKRN1 (7q34)
PTN (7q33)
OR2A9P (7q35)
ZC3H7A (16p13.13)
TMC7 (16p12.3)
ZFHX3 (16q22.2)
LINC00311 (16q24.1)
F10 (13q34)
GNB2L1 (5q35.3)
C10orf55 (10q22.2)
BARX2 (11q24.3)
CNGA1 (4p12)
TBC1D14 (4p16.1)
SMIM20 (4p15.2)
CADM2 (3p12.1)
RiskGroup
High
Low
NotSig
CNA
<= -1
0
shown (30/42),
without filtering = 641
samples (162)
p.Valorate <.05

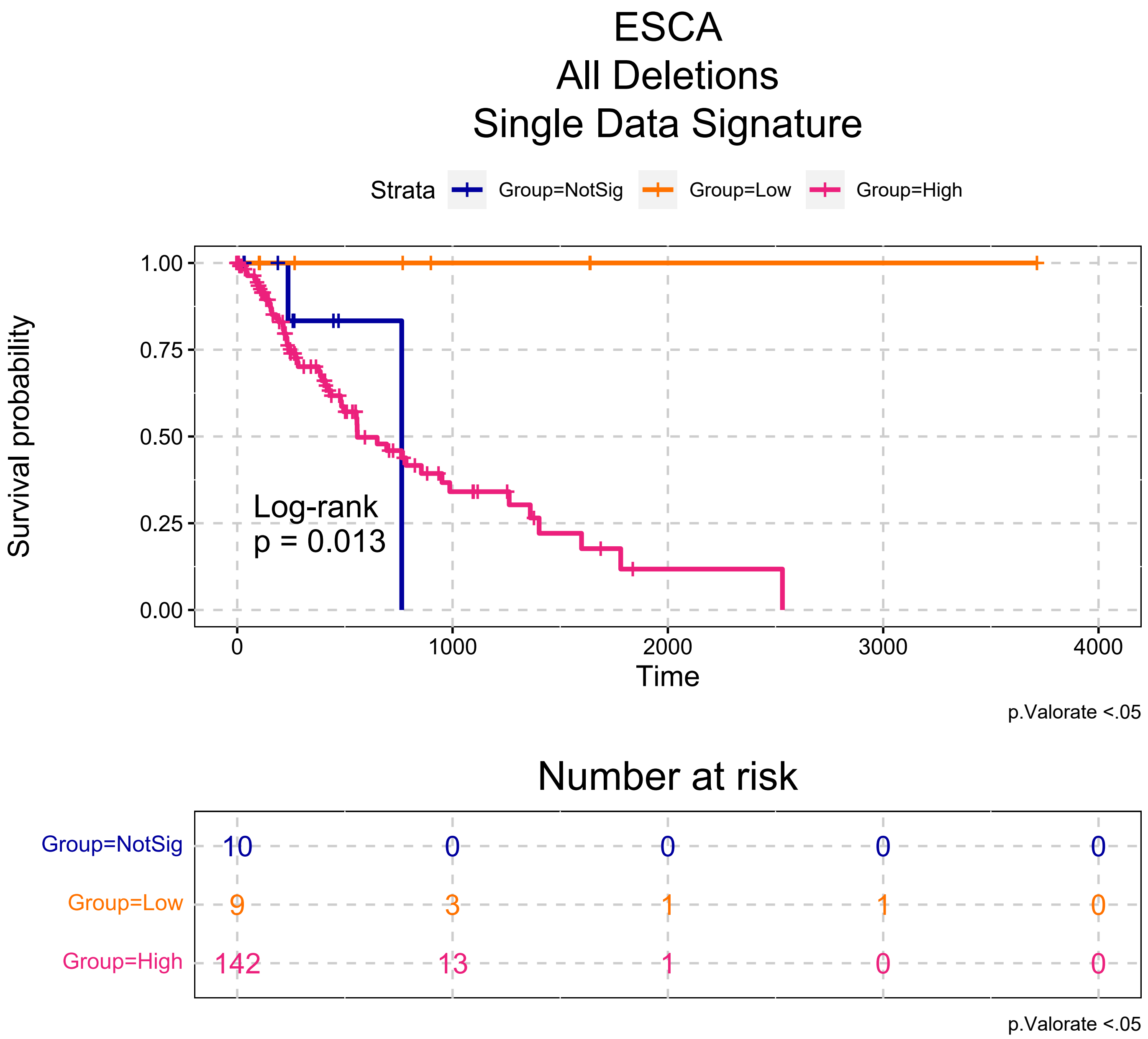

explanatory	beta	HR	L95	U95	p
Low	-17.96	0.00	0.00	Inf	1.00
High	0.41	1.50	0.36	6.23	0.57

n= 162, number of events =56
Score(logrank) test = 0.013

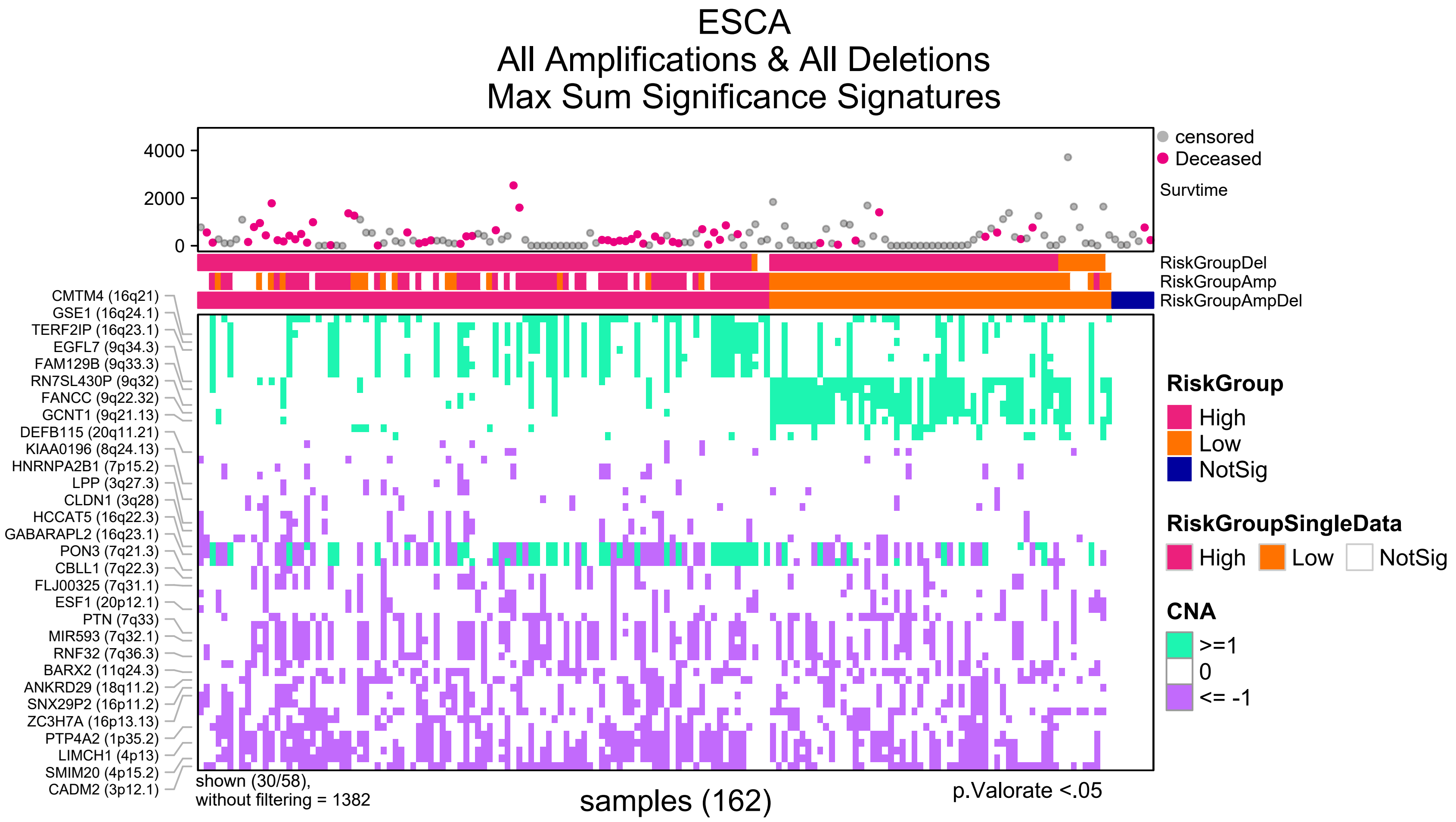

ESCA
All Amplifications & All Deletions
Max Sum Significance Signatures
4000
2000
0
censored
Deceased
Survtime
RiskGroupDel
RiskGroupAmp
RiskGroupAmpDel
CMTM4 (16q21)
GSE1 (16q24.1)
TERF2IP (16q23.1)
EGFL7 (9q34.3)
FAM129B (9q33.3)
RN7SL430P (9q32)
FANCC (9q22.32)
GCNT1 (9q21.13)
DEFB115 (20q11.21)
KIAA0196 (8q24.13)
HNRNPA2B1 (7p15.2)
LPP (3q27.3)
CLDN1 (3q28)
HCCAT5 (16q22.3)
GABARAPL2 (16q23.1)
PON3 (7q21.3)
CBLL1 (7q22.3)
FLJ00325 (7q31.1)
ESF1 (20p12.1)
PTN (7q33)
MIR593 (7q32.1)
RNF32 (7q36.3)
BARX2 (11q24.3)
ANKRD29 (18q11.2)
SNX29P2 (16p11.2)
ZC3H7A (16p13.13)
PTP4A2 (1p35.2)
LIMCH1 (4p13)
SMIM20 (4p15.2)
CADM2 (3p12.1)
RiskGroup
High
Low
NotSig
RiskGroupSingleData
High
Low
NotSig
CNA
>=1
0
<= -1
shown (30/58),
without filtering = 1382
samples (162)
p.Valorate <.05

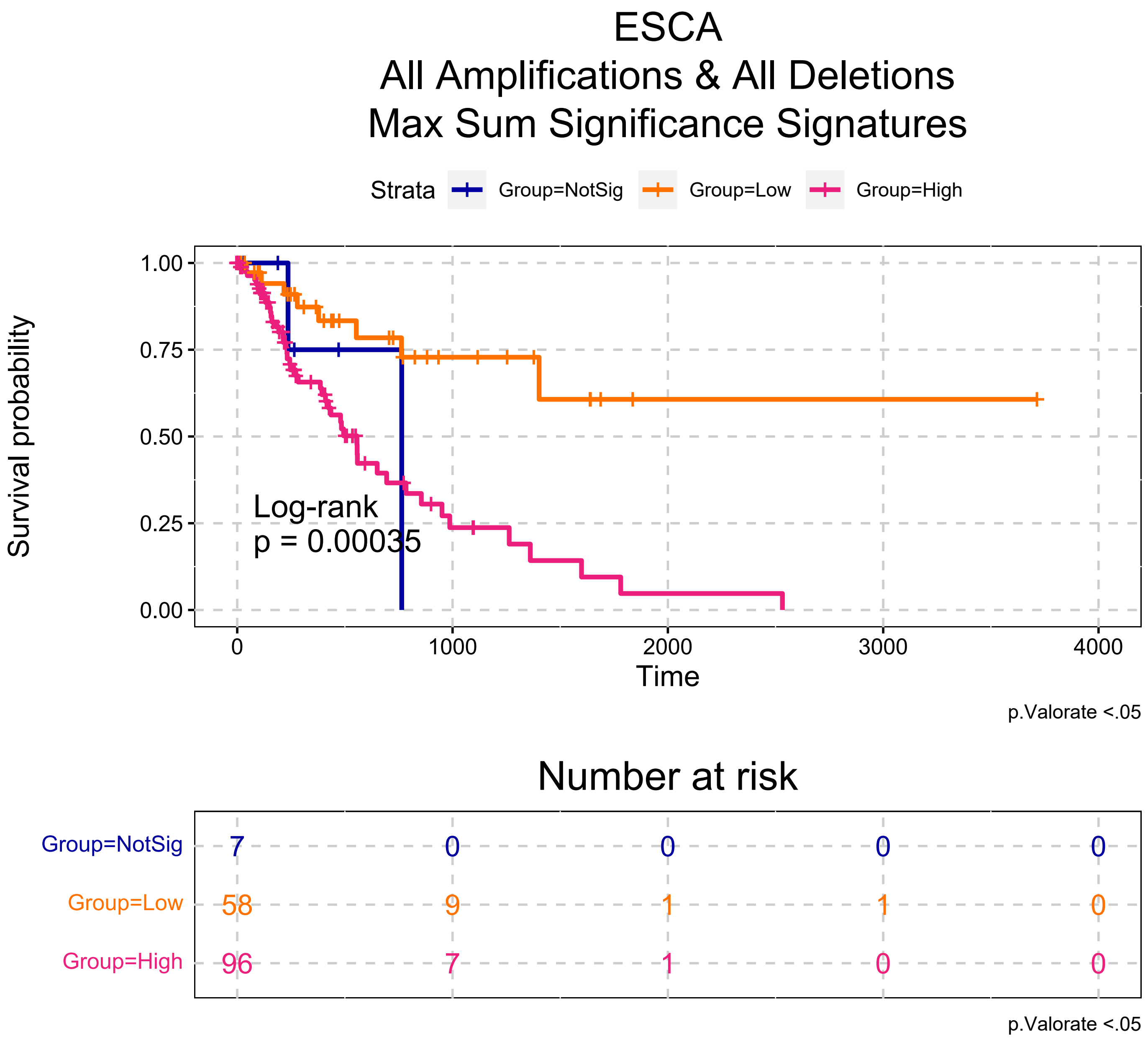

explanatory	beta	HR	L95	U95	p
Low	-1.17	0.31	0.06	1.48	0.14
High	0.26	1.29	0.31	5.36	0.72

n= 162, number of events =56
Score(logrank) test = 0

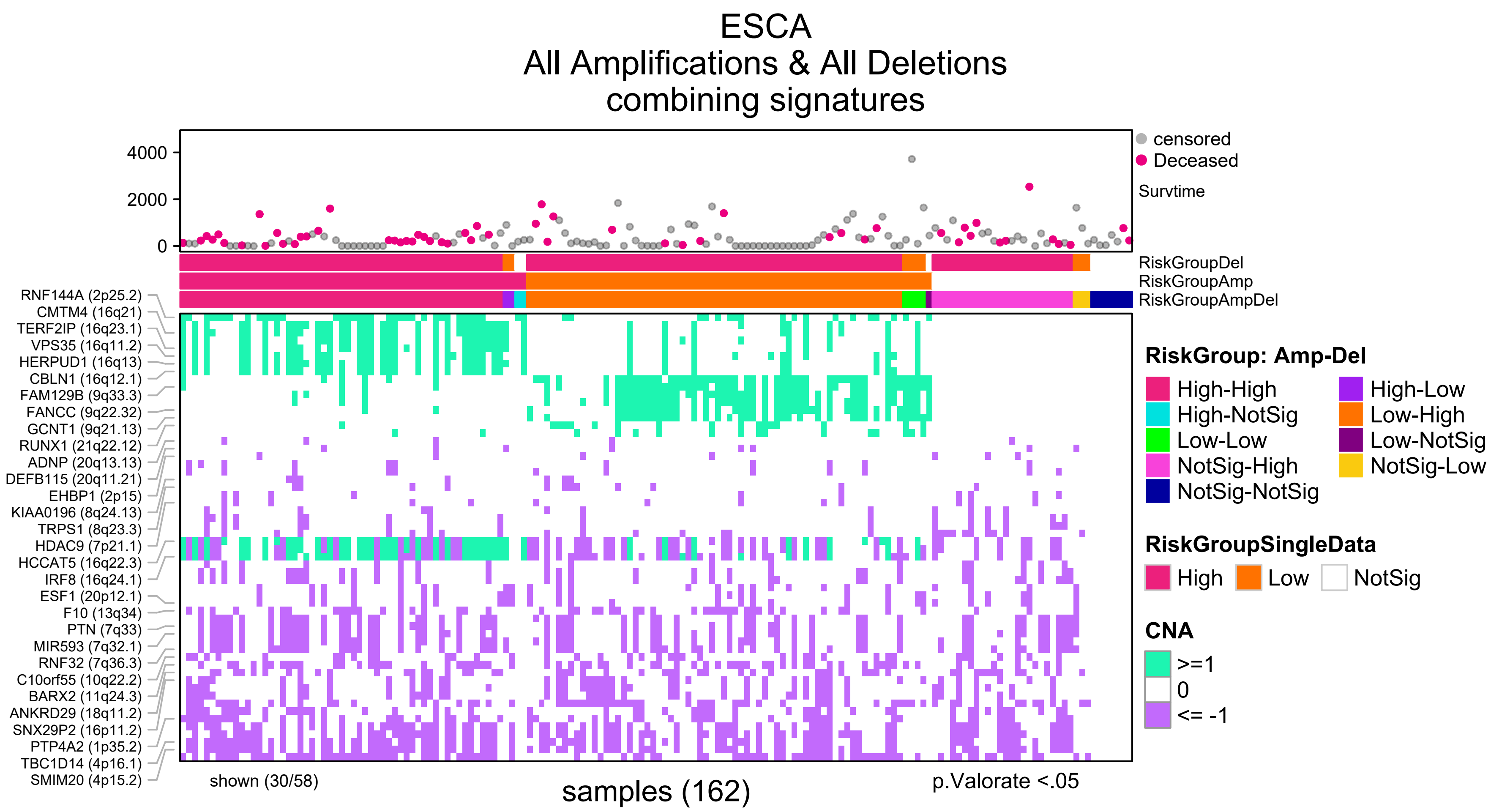
ESCA
All Amplifications & All Deletions
combining signatures
4000
2000
0
censored
Deceased
Survtime
RiskGroupDel
RiskGroupAmp
RiskGroupAmpDel
RNF144A (2p25.2)
CMTM4 (16q21)
TERF2IP (16q23.1)
VPS35 (16q11.2)
HERPUD1 (16q13)
CBLN1 (16q12.1)
FAM129B (9q33.3)
FANCC (9q22.32)
GCNT1 (9q21.13)
RUNX1 (21q22.12)
ADNP (20q13.13)
DEFB115 (20q11.21)
EHBP1 (2p15)
KIAA0196 (8q24.13)
TRPS1 (8q23.3)
HDAC9 (7p21.1)
HCCAT5 (16q22.3)
IRF8 (16q24.1)
ESF1 (20p12.1)
F10 (13q34)
PTN (7q33)
MIR593 (7q32.1)
RNF32 (7q36.3)
C10orf55 (10q22.2)
BARX2 (11q24.3)
ANKRD29 (18q11.2)
SNX29P2 (16p11.2)
PTP4A2 (1p35.2)
TBC1D14 (4p16.1)
SMIM20 (4p15.2)
RiskGroup: Amp-Del
High-High
High-NotSig
Low-Low
NotSig-High
NotSig-NotSig
High-Low
Low-High
Low-NotSig
NotSig-Low
RiskGroupSingleData
High
Low
NotSig
CNA
>=1
0
<= -1
shown (30/58)
samples (162)
p.Valorate <.05

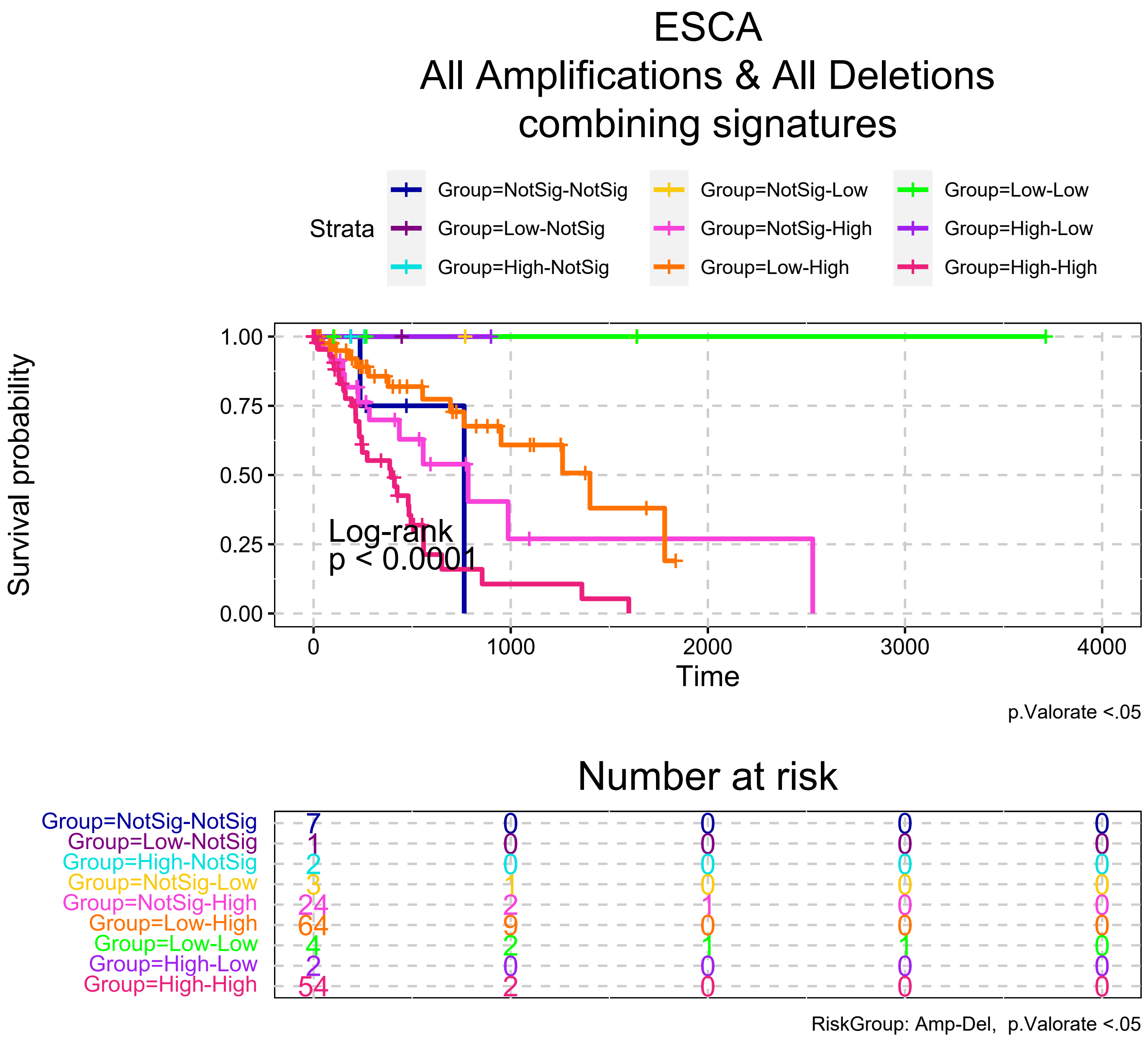

explanatory	beta	HR	L95	U95	p
Low-NotSig	-18.38	0.00	0.00	Inf	1.00
High-NotSig	-18.35	0.00	0.00	Inf	1.00
NotSig-Low	-18.62	0.00	0.00	Inf	1.00
NotSig-High	-0.16	0.86	0.19	3.94	0.84
Low-High	-0.76	0.47	0.10	2.11	0.32
Low-Low	-18.75	0.00	0.00	Inf	1.00
High-Low	-18.53	0.00	0.00	Inf	1.00
High-High	0.69	1.99	0.47	8.36	0.35

n= 162, number of events =56
Score(logrank) test = p <.0001

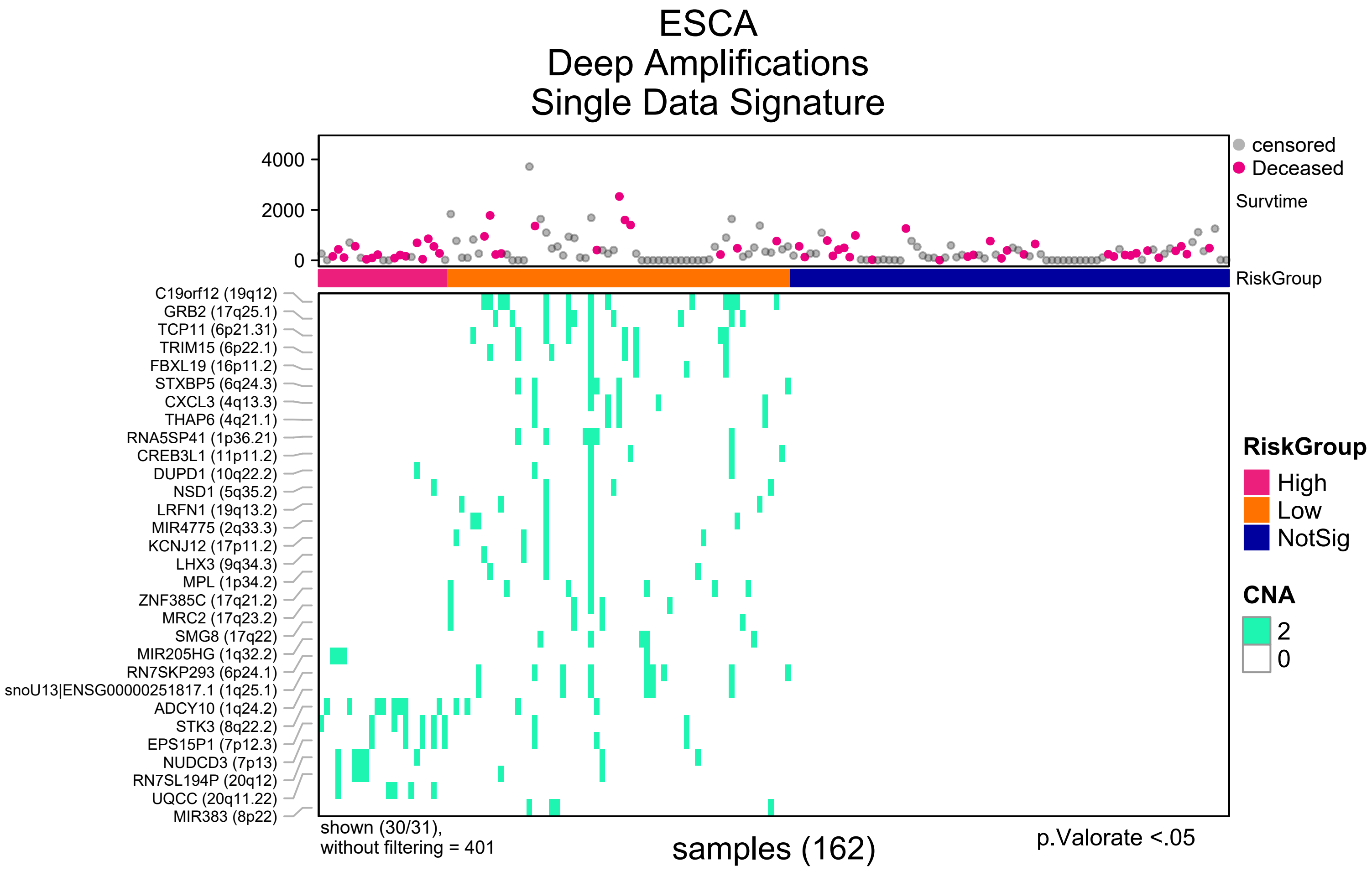

ESCA
Deep Amplifications
Single Data Signature
4000
2000
0
censored
Deceased
Survtime
RiskGroup
C19orf12 (19q12)
GRB2 (17q25.1)
TCP11 (6p21.31)
TRIM15 (6p22.1)
FBXL19 (16p11.2)
STXBP5 (6q24.3)
CXCL3 (4q13.3)
THAP6 (4q21.1)
RNA5SP41 (1p36.21)
CREB3L1 (11p11.2)
DUPD1 (10q22.2)
NSD1 (5q35.2)
LRFN1 (19q13.2)
MIR4775 (2q33.3)
KCNJ12 (17p11.2)
LHX3 (9q34.3)
MPL (1p34.2)
ZNF385C (17q21.2)
MRC2 (17q23.2)
SMG8 (17q22)
MIR205HG (1q32.2)
RN7SKP293 (6p24.1)
snoU13|ENSG00000251817.1 (1q25.1)
ADCY10 (1q24.2)
STK3 (8q22.2)
EPS15P1 (7p12.3)
NUDCD3 (7p13)
RN7SL194P (20q12)
UQCC (20q11.22)
MIR383 (8p22)
RiskGroup
High
Low
NotSig
CNA
2
0
shown (30/31),
without filtering = 401
samples (162)
p.Valorate <.05

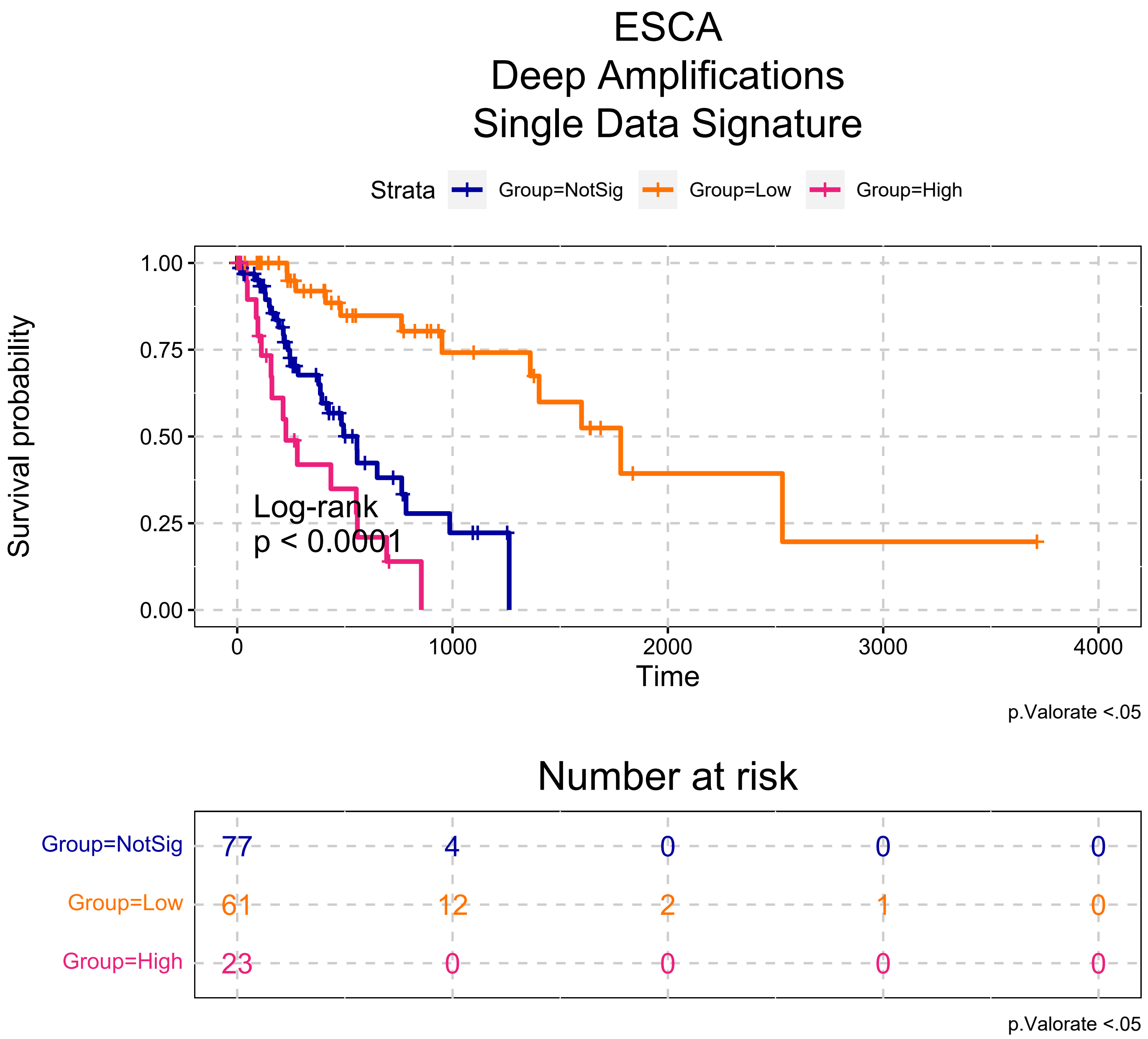

explanatory	beta	HR	L95	U95	p
Low	-1.77	0.17	0.07	0.40	0.00
High	0.72	2.06	1.10	3.86	0.02

n= 162, number of events =56
Score(logrank) test = p <.0001

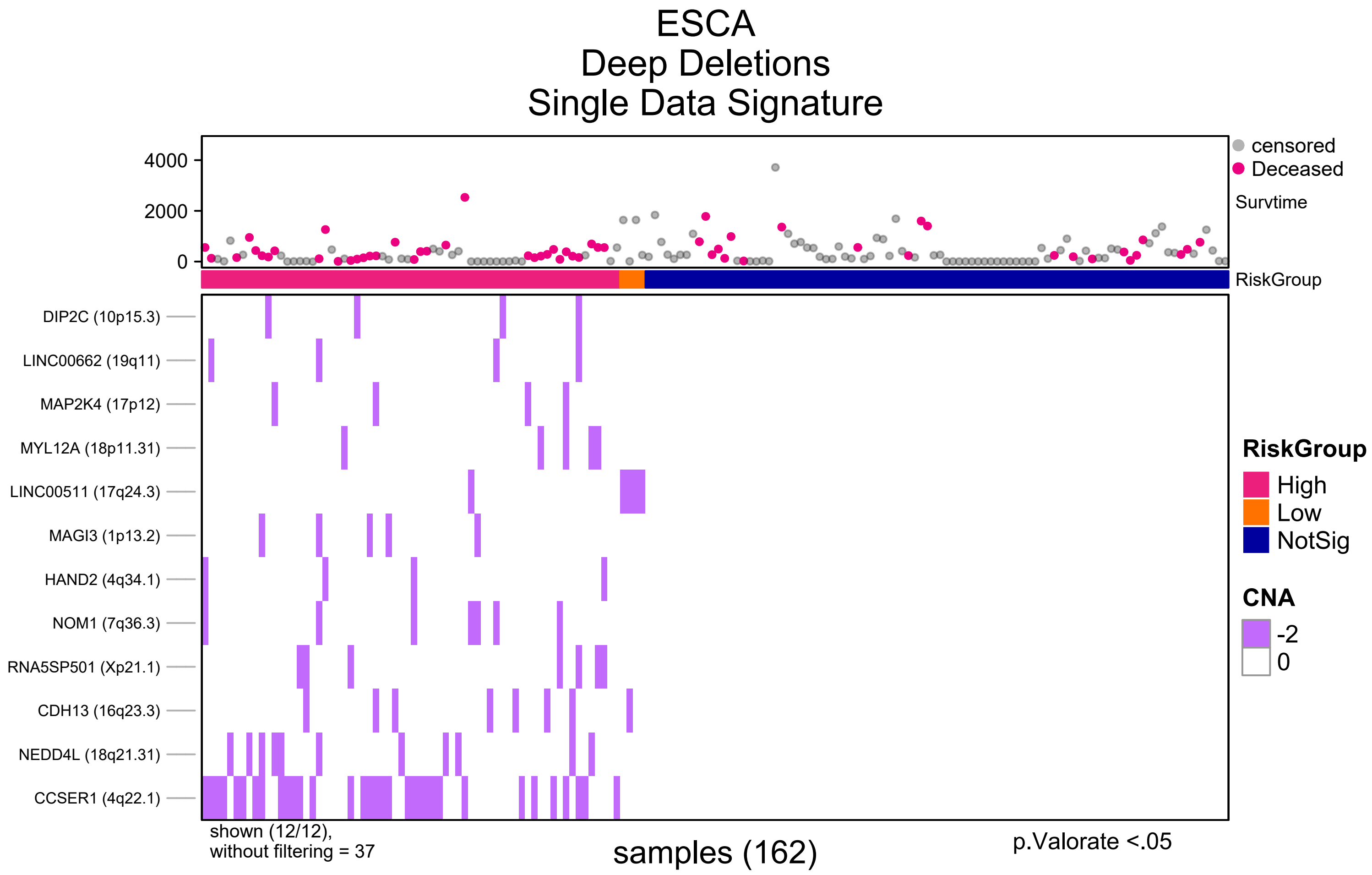

ESCA
Deep Deletions
Single Data Signature
4000
2000
0
censored
Deceased
Survtime
RiskGroup
DIP2C (10p15.3)
LINC00662 (19q11)
MAP2K4 (17p12)
MYL12A (18p11.31)
LINC00511 (17q24.3)
MAGI3 (1p13.2)
HAND2 (4q34.1)
NOM1 (7q36.3)
RNA5SP501 (Xp21.1)
CDH13 (16q23.3)
NEDD4L (18q21.31)
CCSER1 (4q22.1)
RiskGroup
High
Low
NotSig
CNA
-2
0
shown (12/12),
without filtering = 37
samples (162)
p.Valorate <.05

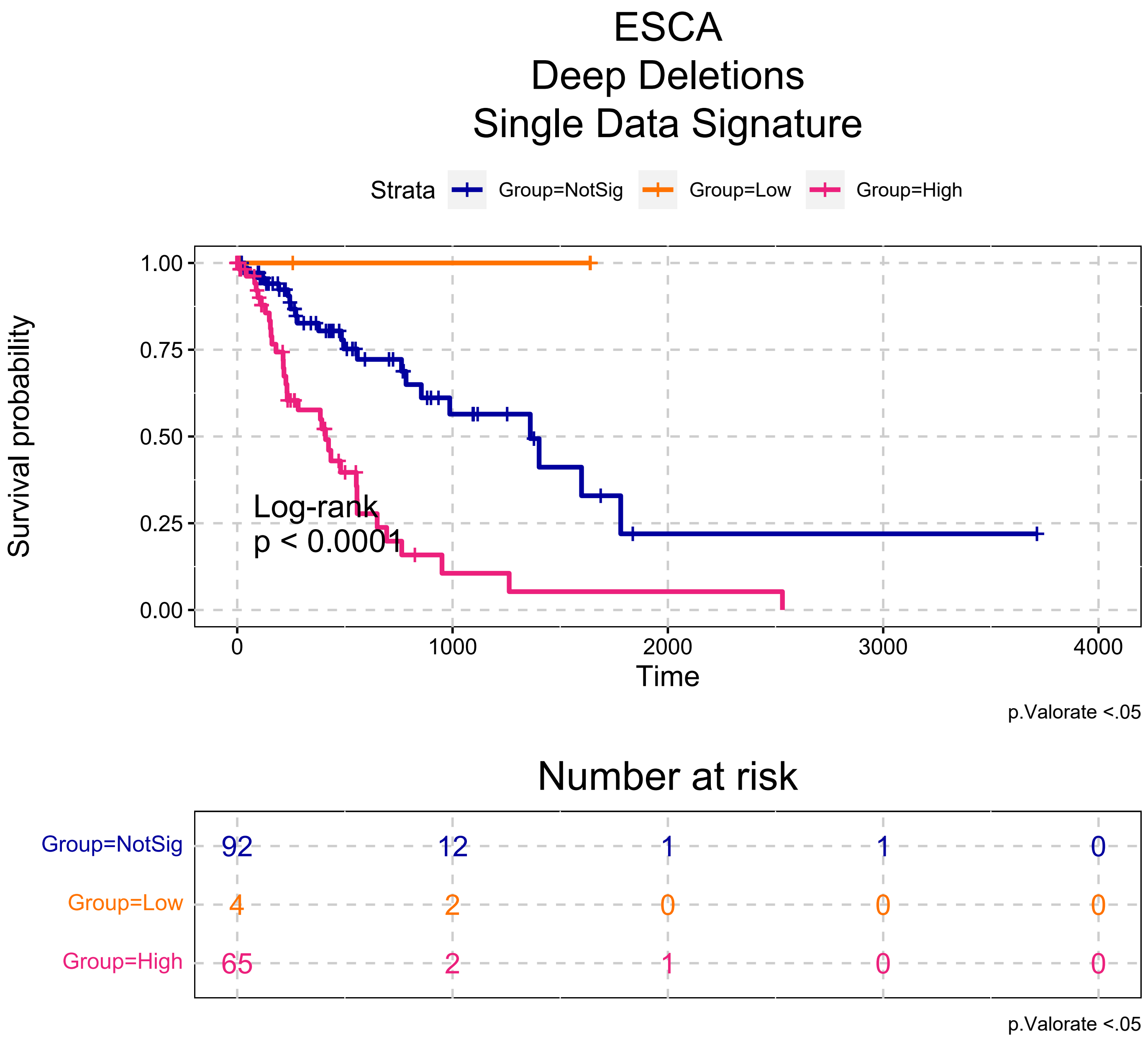

explanatory	beta	HR	L95	U95	p
Low	-16.90	0.00	0.00	Inf	1.00
High	1.26	3.52	2.03	6.10	0.00

n= 162, number of events =56
Score(logrank) test = p <.0001

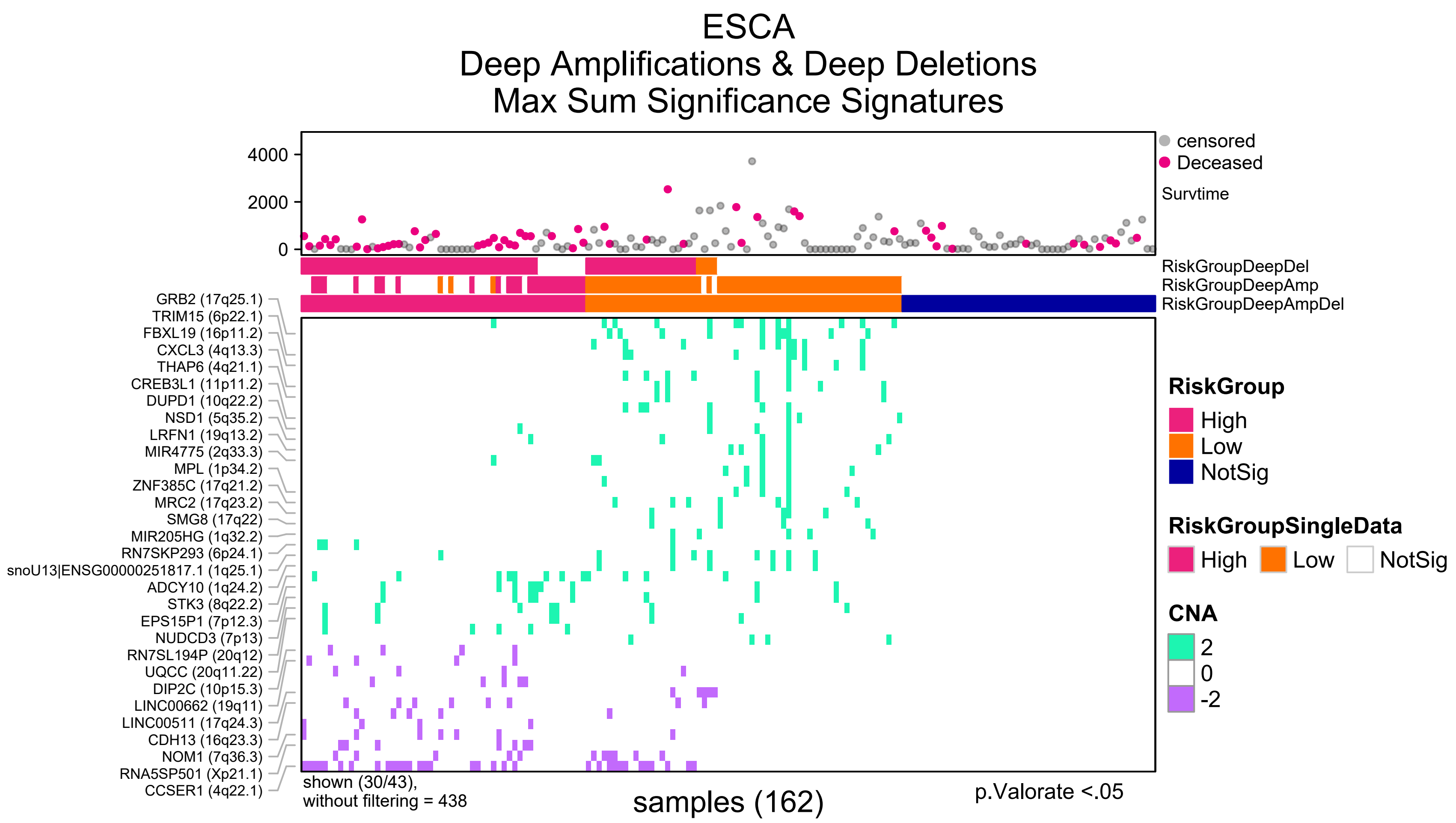
ESCA
Deep Amplifications & Deep Deletions
Max Sum Significance Signatures
4000
2000
0
censored
Deceased
Survtime
RiskGroupDeepDel
RiskGroupDeepAmp
RiskGroupDeepAmpDel
GRB2 (17q25.1)
TRIM15 (6p22.1)
FBXL19 (16p11.2)
CXCL3 (4q13.3)
THAP6 (4q21.1)
CREB3L1 (11p11.2)
DUPD1 (10q22.2)
NSD1 (5q35.2)
LRFN1 (19q13.2)
MIR4775 (2q33.3)
MPL (1p34.2)
ZNF385C (17q21.2)
MRC2 (17q23.2)
SMG8 (17q22)
MIR205HG (1q32.2)
RN7SKP293 (6p24.1)
snoU13|ENSG00000251817.1 (1q25.1)
ADCY10 (1q24.2)
STK3 (8q22.2)
EPS15P1 (7p12.3)
NUDCD3 (7p13)
RN7SL194P (20q12)
UQCC (20q11.22)
DIP2C (10p15.3)
LINC00662 (19q11)
LINC00511 (17q24.3)
CDH13 (16q23.3)
NOM1 (7q36.3)
RNA5SP501 (Xp21.1)
CCSER1 (4q22.1)
shown (30/43),
without filtering = 438
samples (162)
p.Valorate <.05
RiskGroup
High
Low
NotSig
RiskGroupSingleData
High
Low
NotSig
CNA
2
0
-2

ESCA
Deep Amplifications & Deep Deletions
Max Sum Significance Signatures

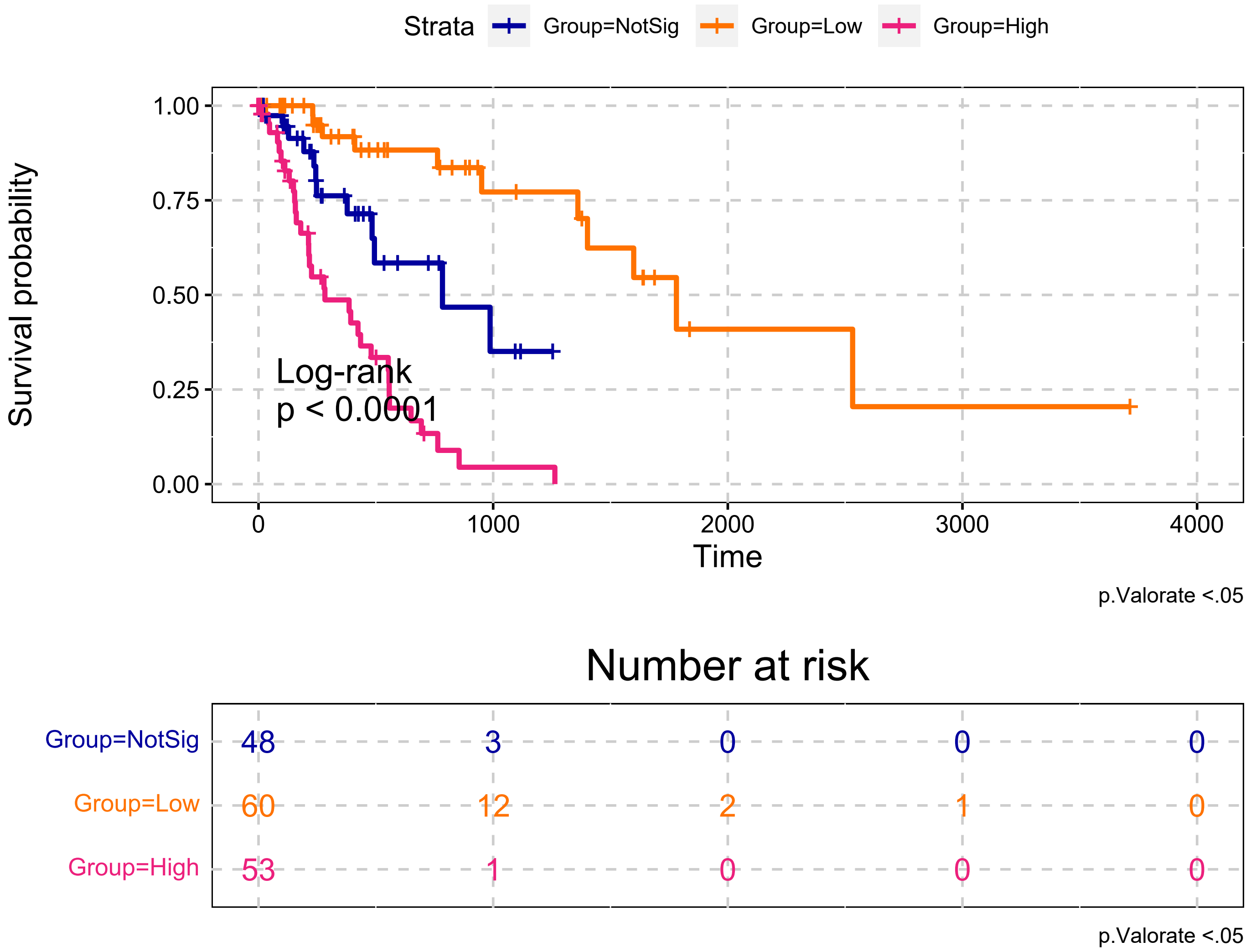

explanatory	beta	HR	L95	U95	p
Low	-1.45	0.24	0.09	0.64	0.00
High	1.13	3.10	1.59	6.05	0.00

n= 162, number of events =56
Score(logrank) test = p <.0001

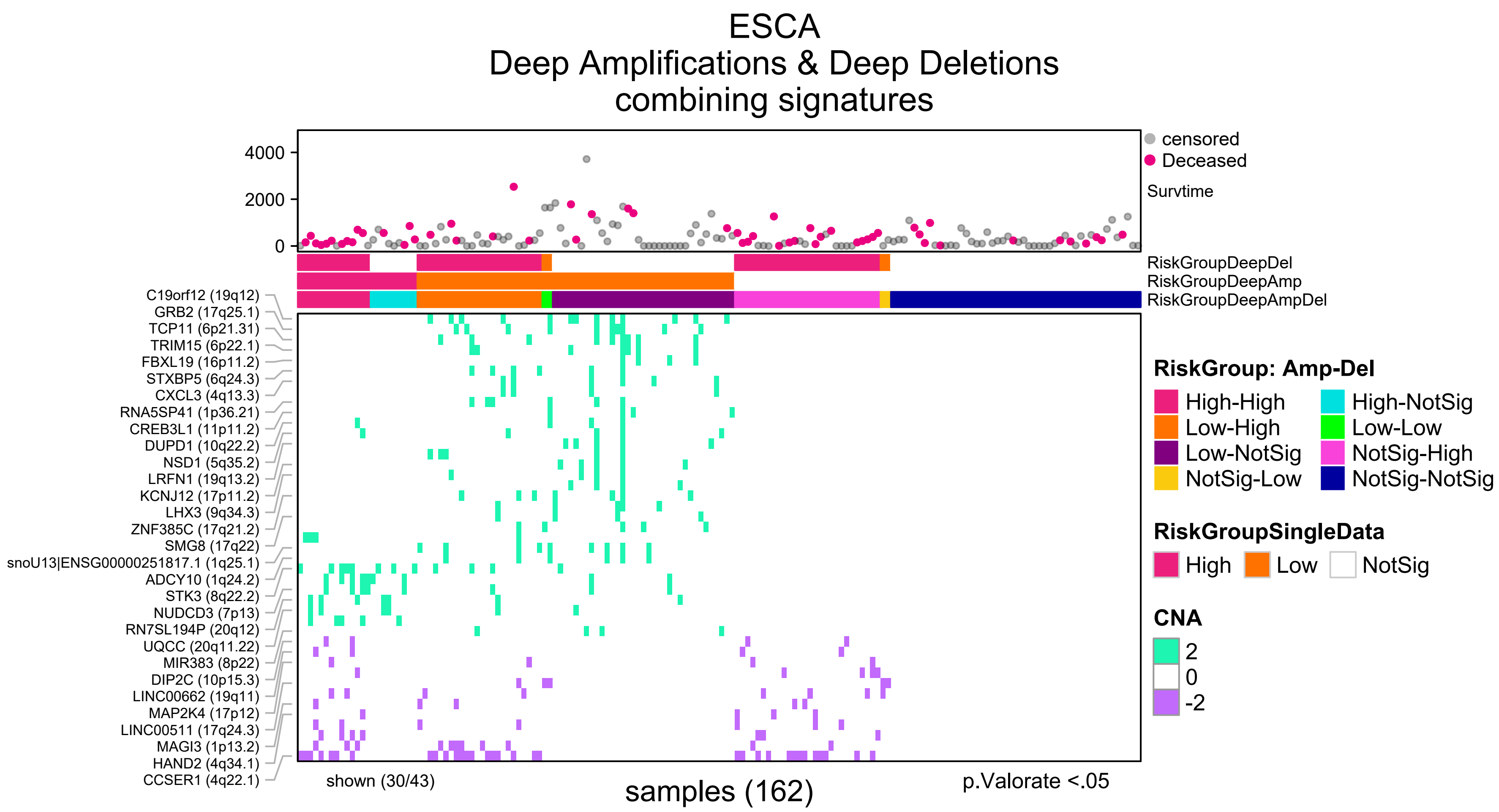

ESCA
Deep Amplifications & Deep Deletions
combining signatures
4000
2000
0
censored
Deceased
Survtime
RiskGroupDeepDel
RiskGroupDeepAmp
RiskGroupDeepAmpDel
C19orf12 (19q12)
GRB2 (17q25.1)
TCP11 (6p21.31)
TRIM15 (6p22.1)
FBXL19 (16p11.2)
STXBP5 (6q24.3)
CXCL3 (4q13.3)
RNA5SP41 (1p36.21)
CREB3L1 (11p11.2)
DUPD1 (10q22.2)
NSD1 (5q35.2)
LRFN1 (19q13.2)
KCNJ12 (17p11.2)
LHX3 (9q34.3)
ZNF385C (17q21.2)
SMG8 (17q22)
snoU13|ENSG00000251817.1 (1q25.1)
ADCY10 (1q24.2)
STK3 (8q22.2)
NUDCD3 (7p13)
RN7SL194P (20q12)
UQCC (20q11.22)
MIR383 (8p22)
DIP2C (10p15.3)
LINC00662 (19q11)
MAP2K4 (17p12)
LINC00511 (17q24.3)
MAGI3 (1p13.2)
HAND2 (4q34.1)
CCSER1 (4q22.1)
RiskGroup: Amp-Del
High-High
High-NotSig
Low-High
Low-Low
Low-NotSig
NotSig-High
NotSig-Low
NotSig-NotSig
RiskGroupSingleData
High
Low
NotSig
CNA
2
0
-2
shown (30/43)
samples (162)
p.Valorate <.05

ESCA
Deep Amplifications & Deep Deletions combining signatures

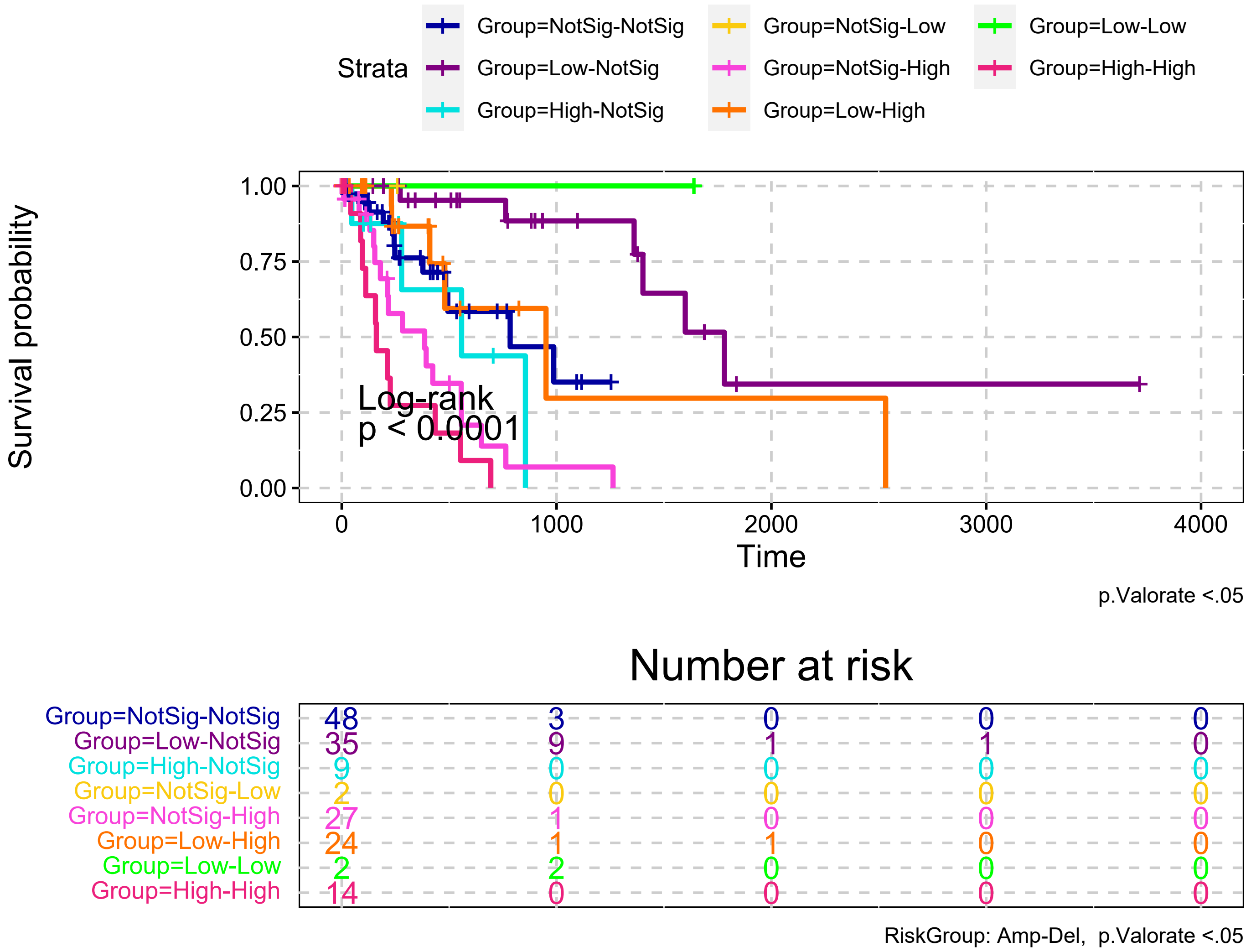

explanatory	beta	HR	L95	U95	p
Low-NotSig	-1.68	0.19	0.06	0.58	0.00
High-NotSig	0.47	1.60	0.51	5.00	0.42
NotSig-Low	-18.19	0.00	0.00	Inf	1.00
NotSig-High	1.08	2.95	1.40	6.23	0.00
Low-High	-0.52	0.60	0.20	1.77	0.35
Low-Low	-18.23	0.00	0.00	Inf	1.00
High-High	1.74	5.67	2.45	13.12	0.00

n= 162, number of events =56
Score(logrank) test = p <.0001
