# Supplementary material for: Systematic Modeling of Risk-Associated Copy Number Alterations in Cancer
Source: Int J Mol Sci. 2024 Sep 27;25(19):10455. doi: 10.3390/ijms251910455 (PMC11477427; doi:10.3390/ijms251910455)
Supplement: Supplementary file 1 [file ijms-25-10455-s001.zip › GBMLGGSignatureV12-sinSombreado.pdf]

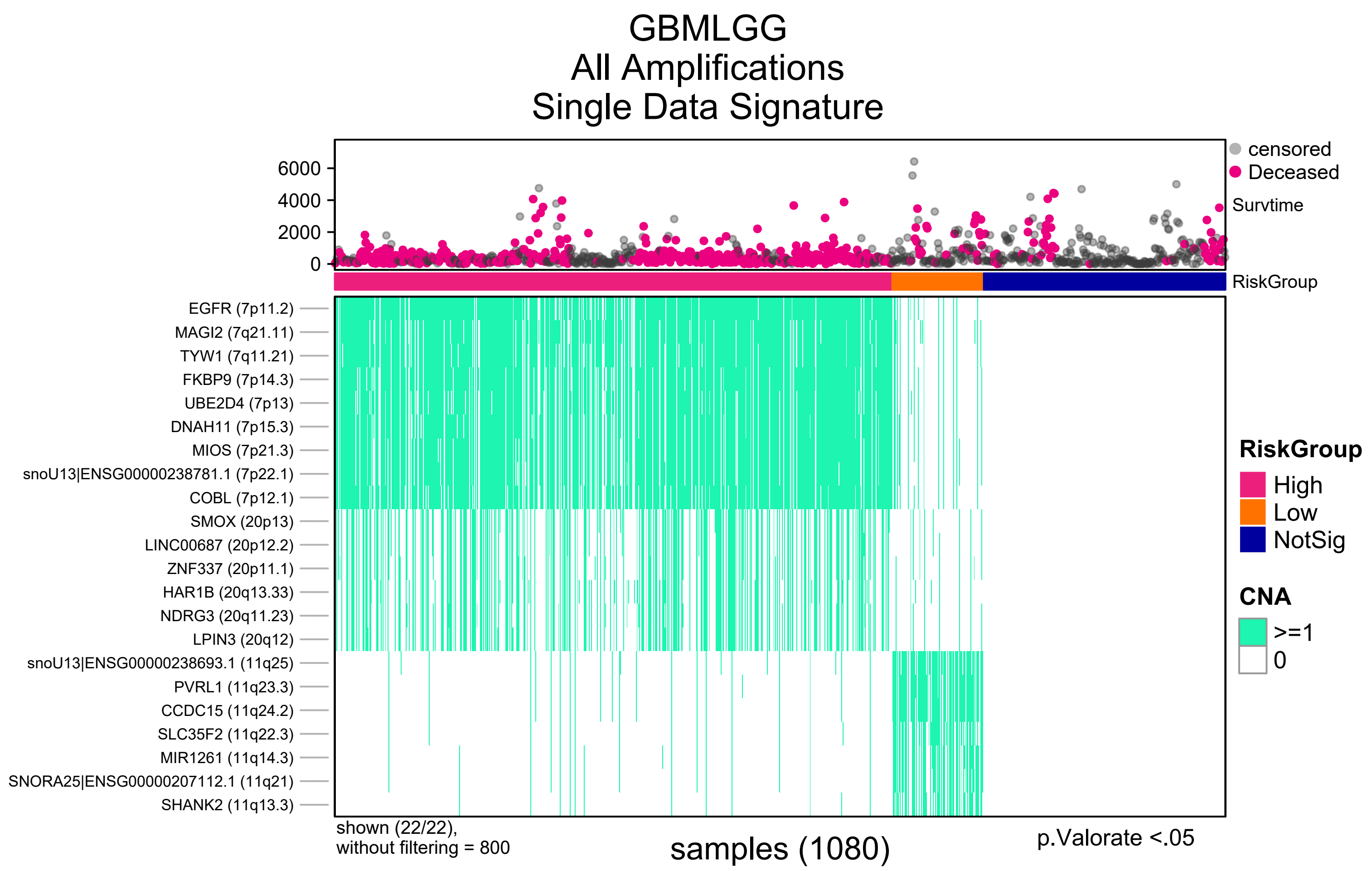
GBMLGG
All Amplifications
Single Data Signature
6000
4000
2000
0
censored
Deceased
Survtime
RiskGroup
EGFR (7p11.2)
MAGI2 (7q21.11)
TYW1 (7q11.21)
FKBP9 (7p14.3)
UBE2D4 (7p13)
DNAH11 (7p15.3)
MIOS (7p21.3)
snoU13|ENSG00000238781.1 (7p22.1)
COBL (7p12.1)
SMOX (20p13)
LINC00687 (20p12.2)
ZNF337 (20p11.1)
HAR1B (20q13.33)
NDRG3 (20q11.23)
LPIN3 (20q12)
snoU13|ENSG00000238693.1 (11q25)
PVRL1 (11q23.3)
CCDC15 (11q24.2)
SLC35F2 (11q22.3)
MIR1261 (11q14.3)
SNORA25|ENSG00000207112.1 (11q21)
SHANK2 (11q13.3)
RiskGroup
High
Low
NotSig
CNA
>=1
0
shown (22/22),
without filtering = 800
samples (1080)
p.Valorate <.05

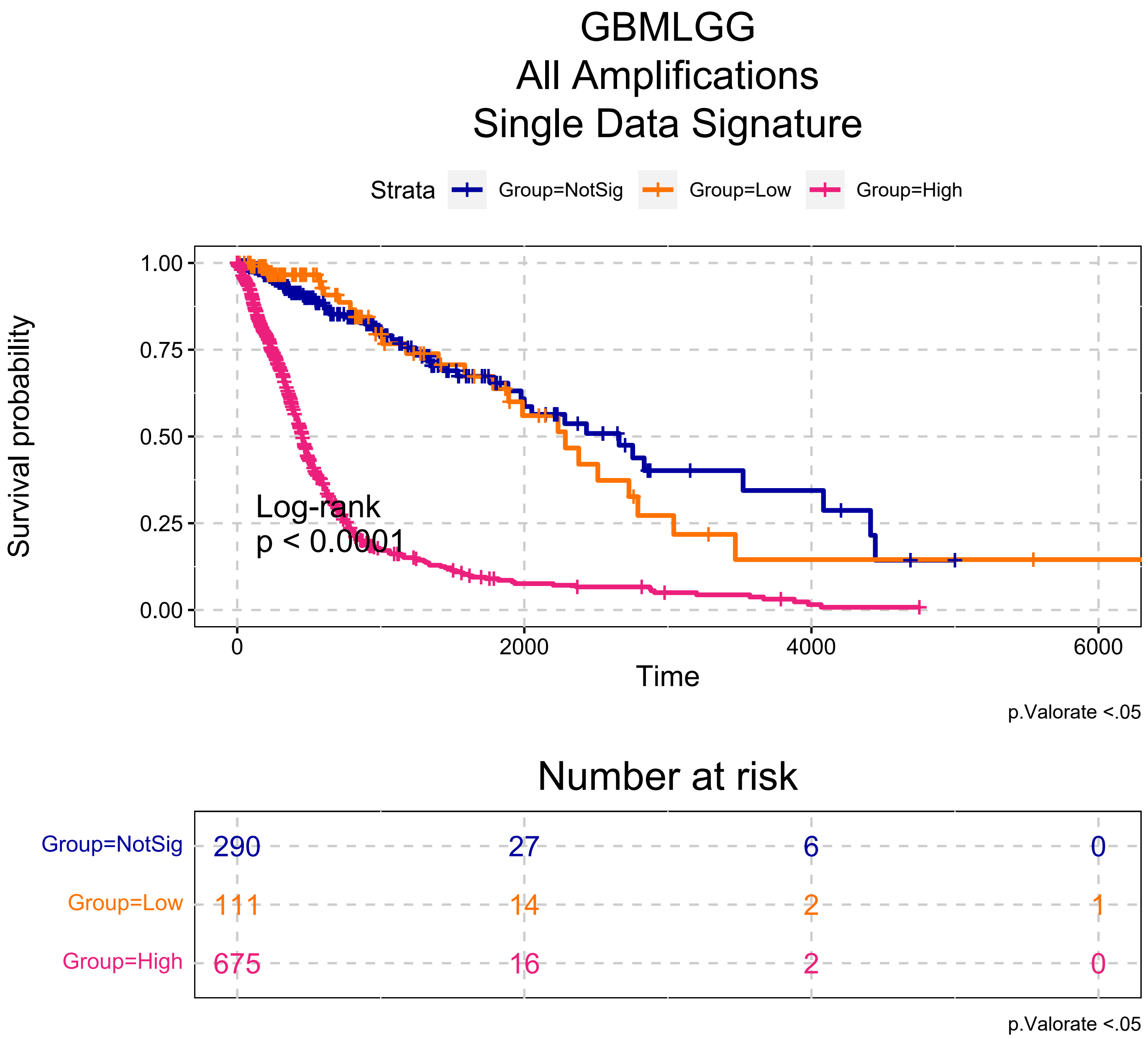

explanatory	beta	HR	L95	U95	p
Low	0.10	1.11	0.69	1.77	0.67
High	1.73	5.63	4.23	7.49	0.00

n= 1080, number of events =522
Score(logrank) test = p <.0001

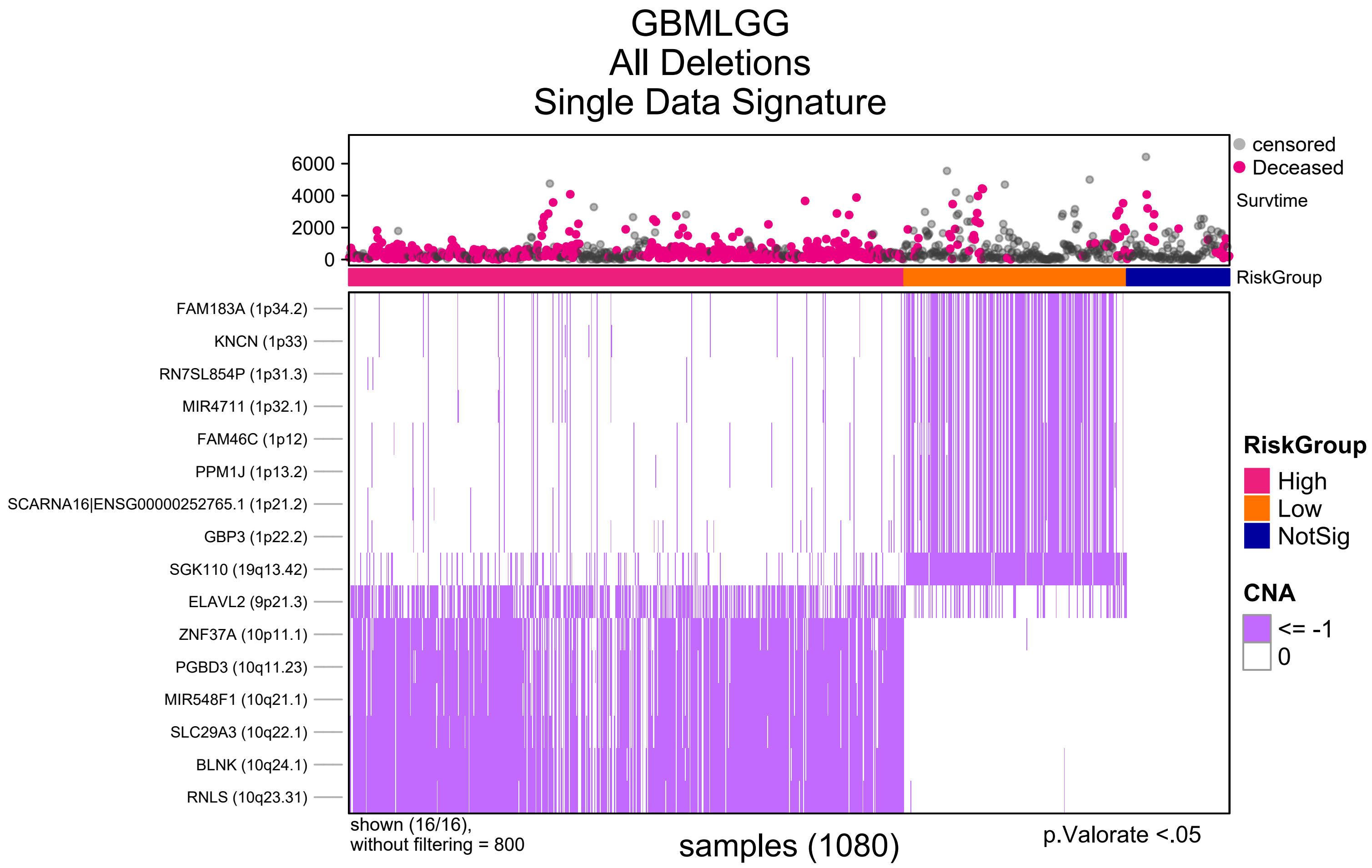
GBMLGG
All Deletions
Single Data Signature
6000
4000
2000
0
censored
Deceased
Survtime
RiskGroup
FAM183A (1p34.2)
KNCN (1p33)
RN7SL854P (1p31.3)
MIR4711 (1p32.1)
FAM46C (1p12)
PPM1J (1p13.2)
SCARNA16|ENSG00000252765.1 (1p21.2)
GBP3 (1p22.2)
SGK110 (19q13.42)
ELAVL2 (9p21.3)
ZNF37A (10p11.1)
PGBD3 (10q11.23)
MIR548F1 (10q21.1)
SLC29A3 (10q22.1)
BLNK (10q24.1)
RNLS (10q23.31)
RiskGroup
High
Low
NotSig
CNA
<= -1
0
shown (16/16),
without filtering = 800
samples (1080)
p.Valorate <.05

GBMLGG
All Deletions
Single Data Signature

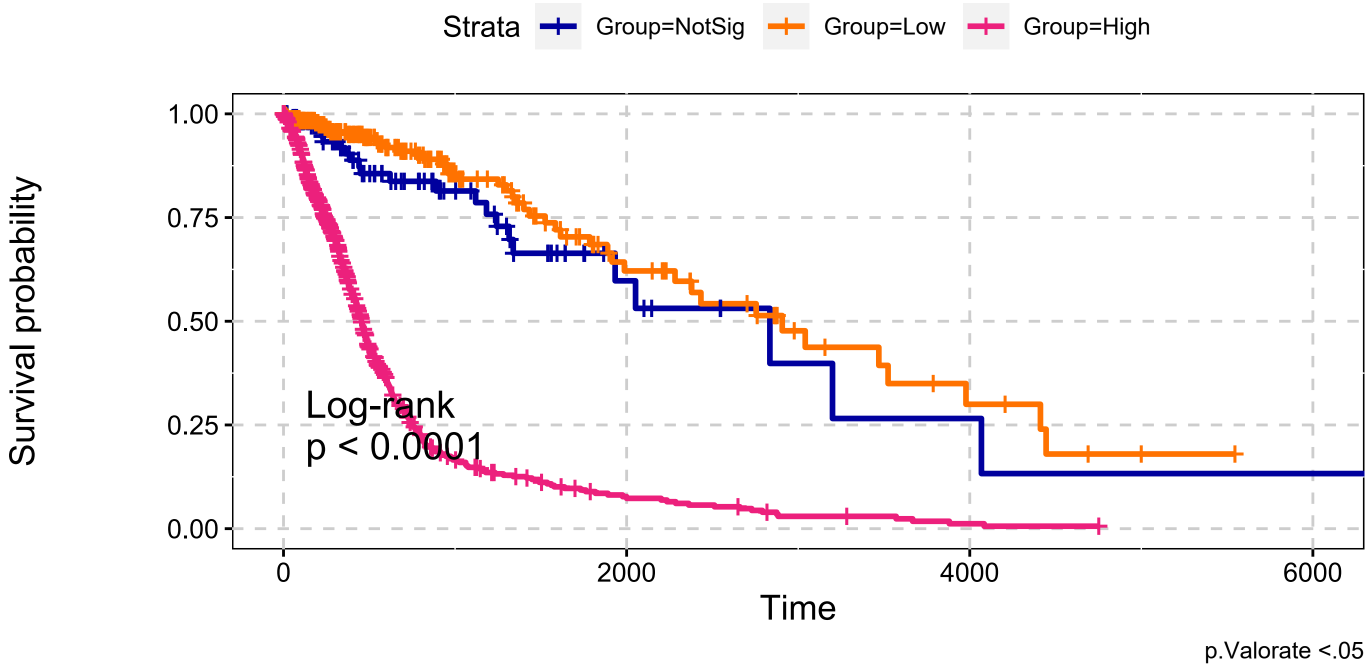

Number at risk

	0	2000	4000	6000
Group=NotSig	125	9	2	1
Group=Low	271	29	6	0
Group=High	680	19	2	0

p.Valorate <.05

explanatory	beta	HR	L95	U95	p
Low	-0.26	0.77	0.46	1.27	0.30
High	1.69	5.43	3.56	8.27	0.00

n= 1080, number of events =522
Score(logrank) test = p <.0001

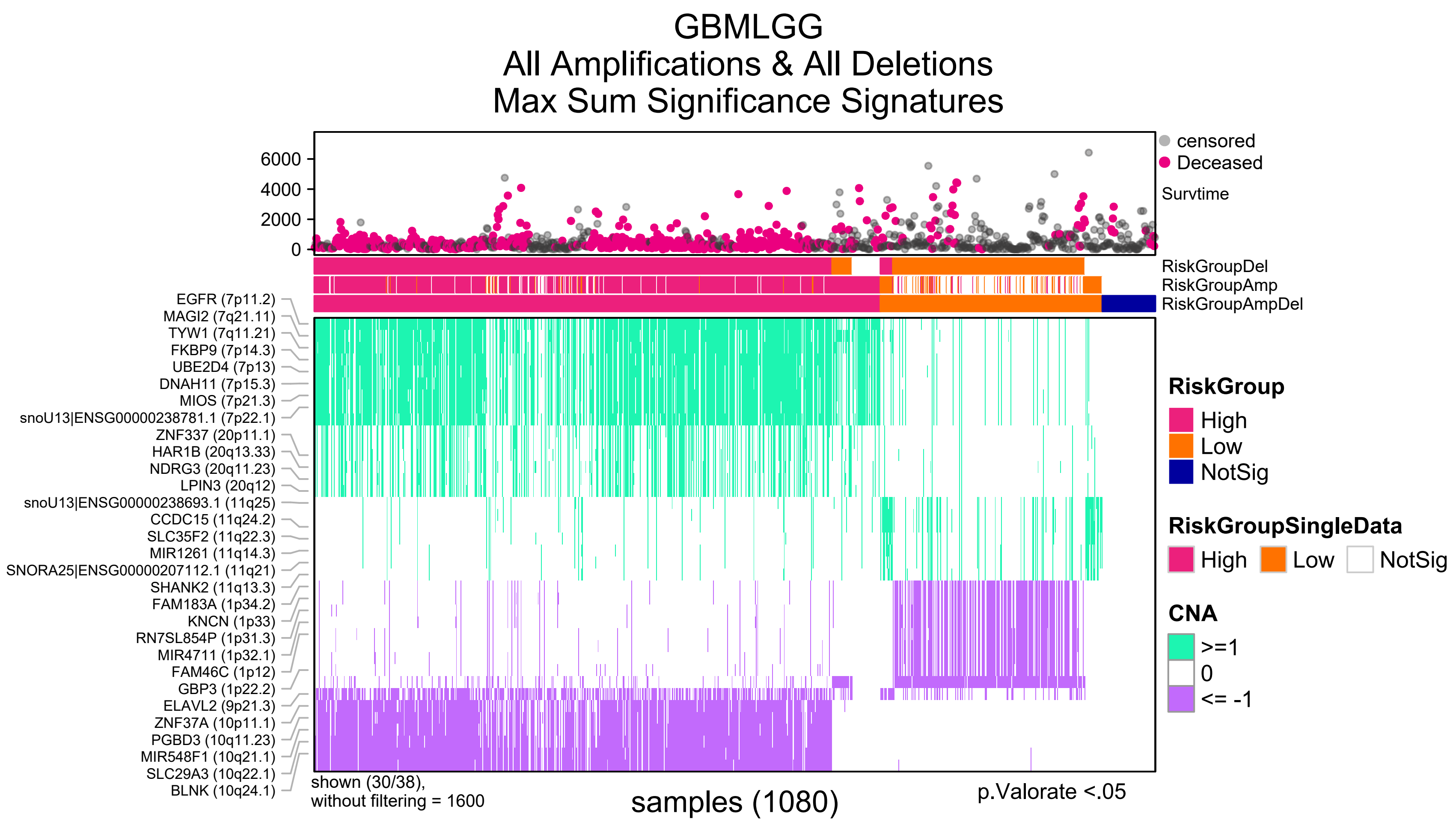
GBMLGG
All Amplifications & All Deletions
Max Sum Significance Signatures
6000
4000
2000
0
censored
Deceased
Survtime
RiskGroupDel
RiskGroupAmp
RiskGroupAmpDel
EGFR (7p11.2)
MAGI2 (7q21.11)
TYW1 (7q11.21)
FKBP9 (7p14.3)
UBE2D4 (7p13)
DNAH11 (7p15.3)
MIOS (7p21.3)
snoU13|ENSG00000238781.1 (7p22.1)
ZNF337 (20p11.1)
HAR1B (20q13.33)
NDRG3 (20q11.23)
LPIN3 (20q12)
snoU13|ENSG00000238693.1 (11q25)
CCDC15 (11q24.2)
SLC35F2 (11q22.3)
MIR1261 (11q14.3)
SNORA25|ENSG00000207112.1 (11q21)
SHANK2 (11q13.3)
FAM183A (1p34.2)
KNCN (1p33)
RN7SL854P (1p31.3)
MIR4711 (1p32.1)
FAM46C (1p12)
GBP3 (1p22.2)
ELAVL2 (9p21.3)
ZNF37A (10p11.1)
PGBD3 (10q11.23)
MIR548F1 (10q21.1)
SLC29A3 (10q22.1)
BLNK (10q24.1)
RiskGroup
High
Low
NotSig
RiskGroupSingleData
High
Low
NotSig
CNA
>=1
0
<= -1
shown (30/38),
without filtering = 1600
samples (1080)
p.Valorate <.05

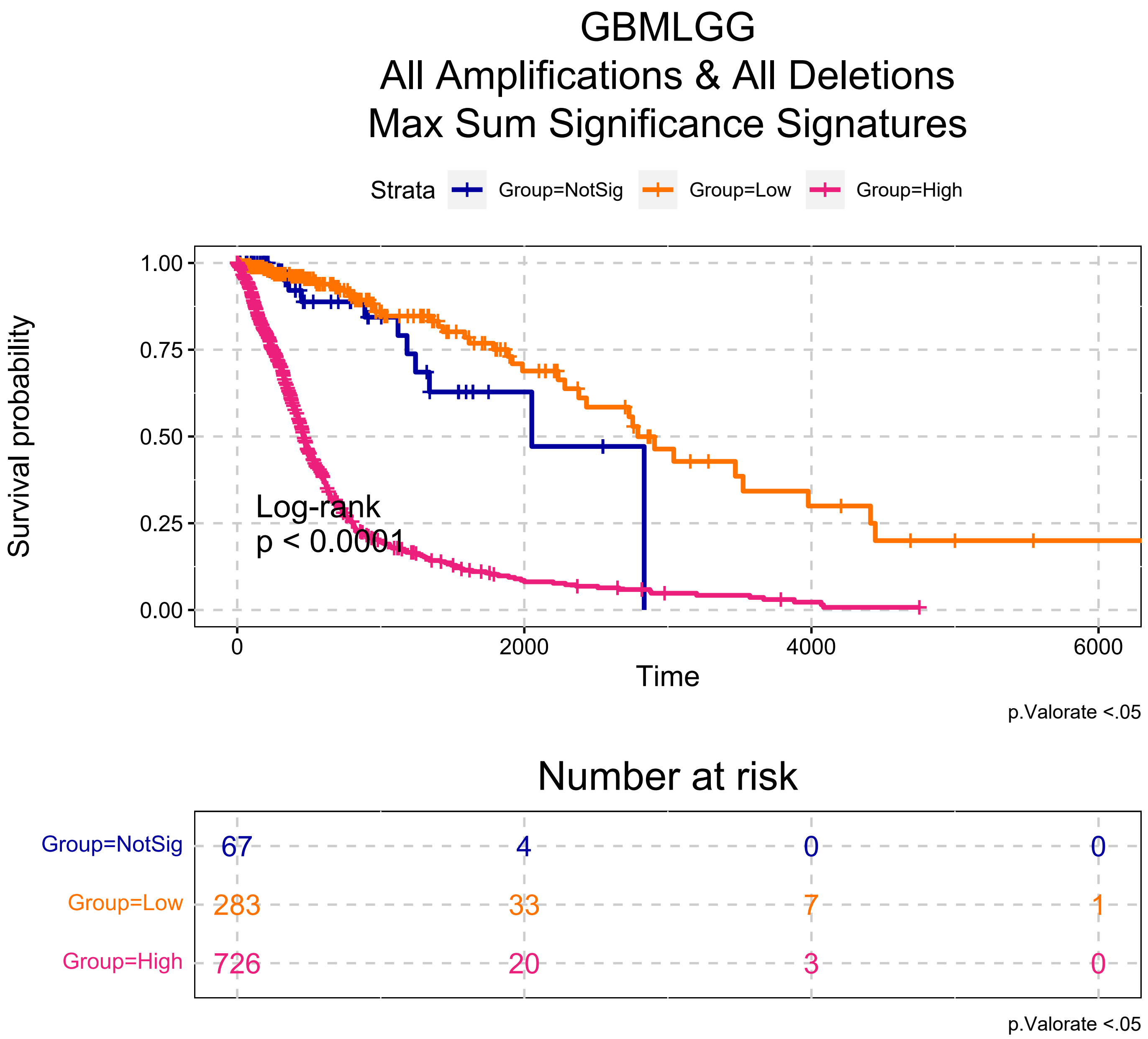

explanatory	beta	HR	L95	U95	p
Low	-0.32	0.73	0.38	1.42	0.35
High	1.67	5.29	2.91	9.63	0.00

n= 1080, number of events =522
Score(logrank) test = p <.0001

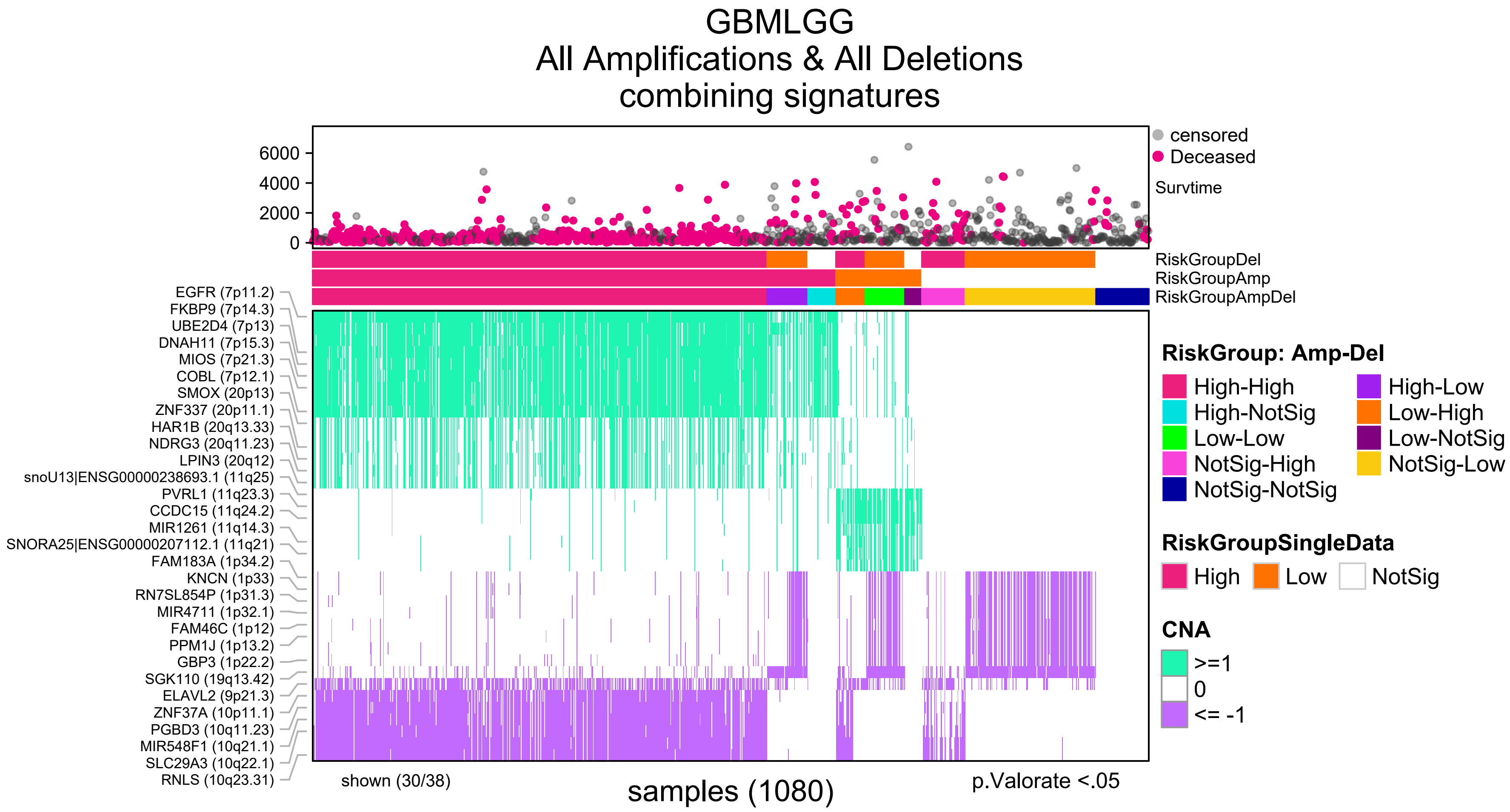
GBMLGG
All Amplifications & All Deletions
combining signatures
6000
4000
2000
0
censored
Deceased
Survtime
RiskGroupDel
RiskGroupAmp
RiskGroupAmpDel
EGFR (7p11.2)
FKBP9 (7p14.3)
UBE2D4 (7p13)
DNAH11 (7p15.3)
MIOS (7p21.3)
COBL (7p12.1)
SMOX (20p13)
ZNF337 (20p11.1)
HAR1B (20q13.33)
NDRG3 (20q11.23)
LPIN3 (20q12)
snoU13|ENSG00000238693.1 (11q25)
PVRL1 (11q23.3)
CCDC15 (11q24.2)
MIR1261 (11q14.3)
SNORA25|ENSG00000207112.1 (11q21)
FAM183A (1p34.2)
KNCN (1p33)
RN7SL854P (1p31.3)
MIR4711 (1p32.1)
FAM46C (1p12)
PPM1J (1p13.2)
GBP3 (1p22.2)
SGK110 (19q13.42)
ELAVL2 (9p21.3)
ZNF37A (10p11.1)
PGBD3 (10q11.23)
MIR548F1 (10q21.1)
SLC29A3 (10q22.1)
RNLS (10q23.31)
shown (30/38)
samples (1080)
p.Valorate <.05
RiskGroup: Amp-Del
High-High
High-NotSig
Low-Low
NotSig-High
NotSig-NotSig
High-Low
Low-High
Low-NotSig
NotSig-Low
RiskGroupSingleData
High
Low
NotSig
CNA
>=1
0
<= -1

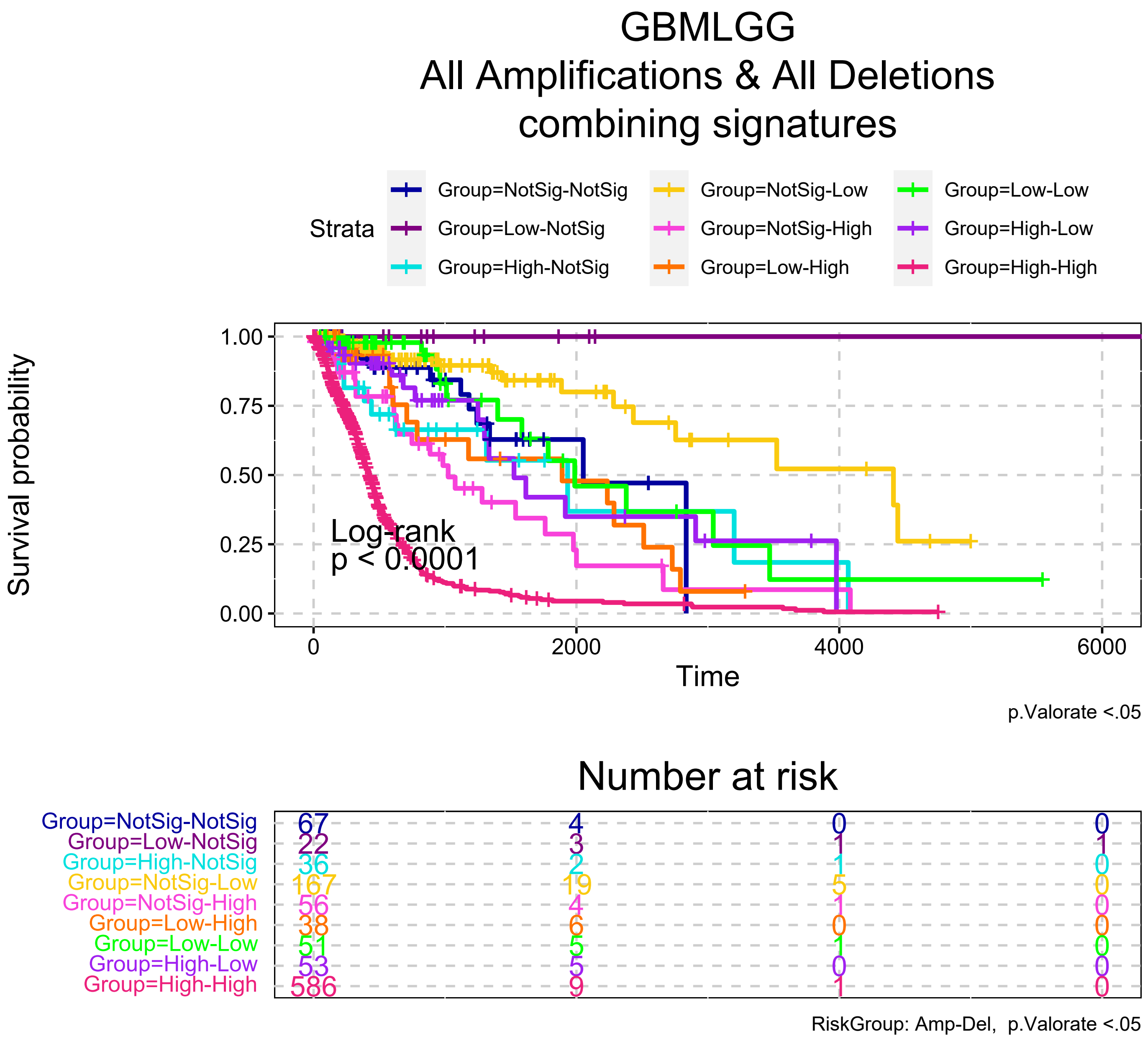

explanatory	beta	HR	L95	U95	p
Low-NotSig	-15.21	0.00	0.00	Inf	0.99
High-NotSig	0.65	1.91	0.84	4.33	0.12
NotSig-Low	-0.65	0.52	0.24	1.10	0.09
NotSig-High	0.93	2.53	1.24	5.15	0.01
Low-High	0.63	1.88	0.85	4.15	0.12
Low-Low	0.01	1.01	0.44	2.28	0.99
High-Low	0.37	1.45	0.67	3.16	0.35
High-High	1.92	6.85	3.75	12.49	0.00

n= 1080, number of events =522
Score(logrank) test = p <.0001

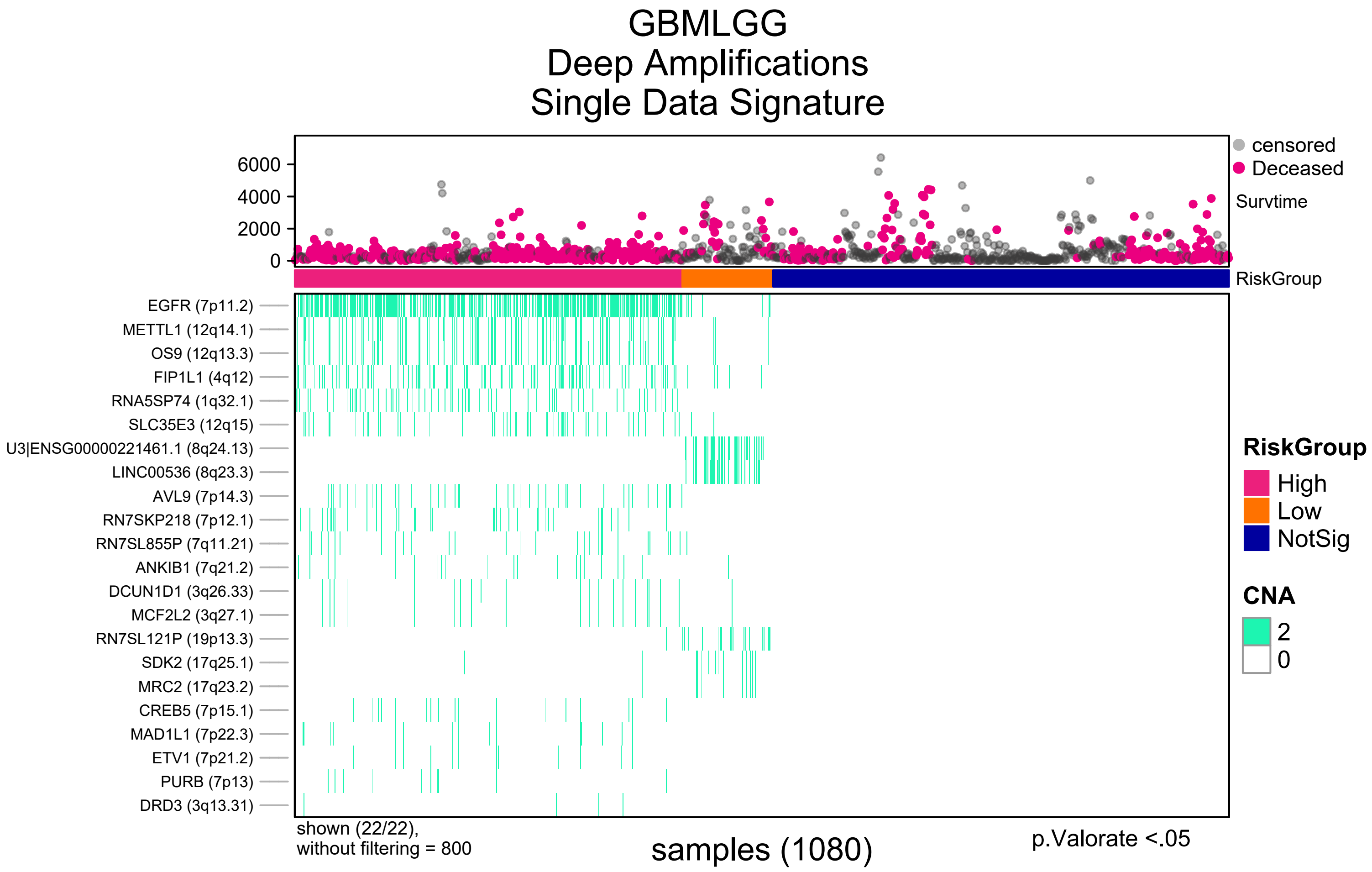
GBMLGG
Deep Amplifications
Single Data Signature
6000
4000
2000
0
censored
Deceased
Survtime
RiskGroup
EGFR (7p11.2)
METTL1 (12q14.1)
OS9 (12q13.3)
FIP1L1 (4q12)
RNA5SP74 (1q32.1)
SLC35E3 (12q15)
U3|ENSG00000221461.1 (8q24.13)
LINC00536 (8q23.3)
AVL9 (7p14.3)
RN7SKP218 (7p12.1)
RN7SL855P (7q11.21)
ANKIB1 (7q21.2)
DCUN1D1 (3q26.33)
MCF2L2 (3q27.1)
RN7SL121P (19p13.3)
SDK2 (17q25.1)
MRC2 (17q23.2)
CREB5 (7p15.1)
MAD1L1 (7p22.3)
ETV1 (7p21.2)
PURB (7p13)
DRD3 (3q13.31)
RiskGroup
High
Low
NotSig
CNA
2
0
shown (22/22),
without filtering = 800
samples (1080)
p.Valorate <.05

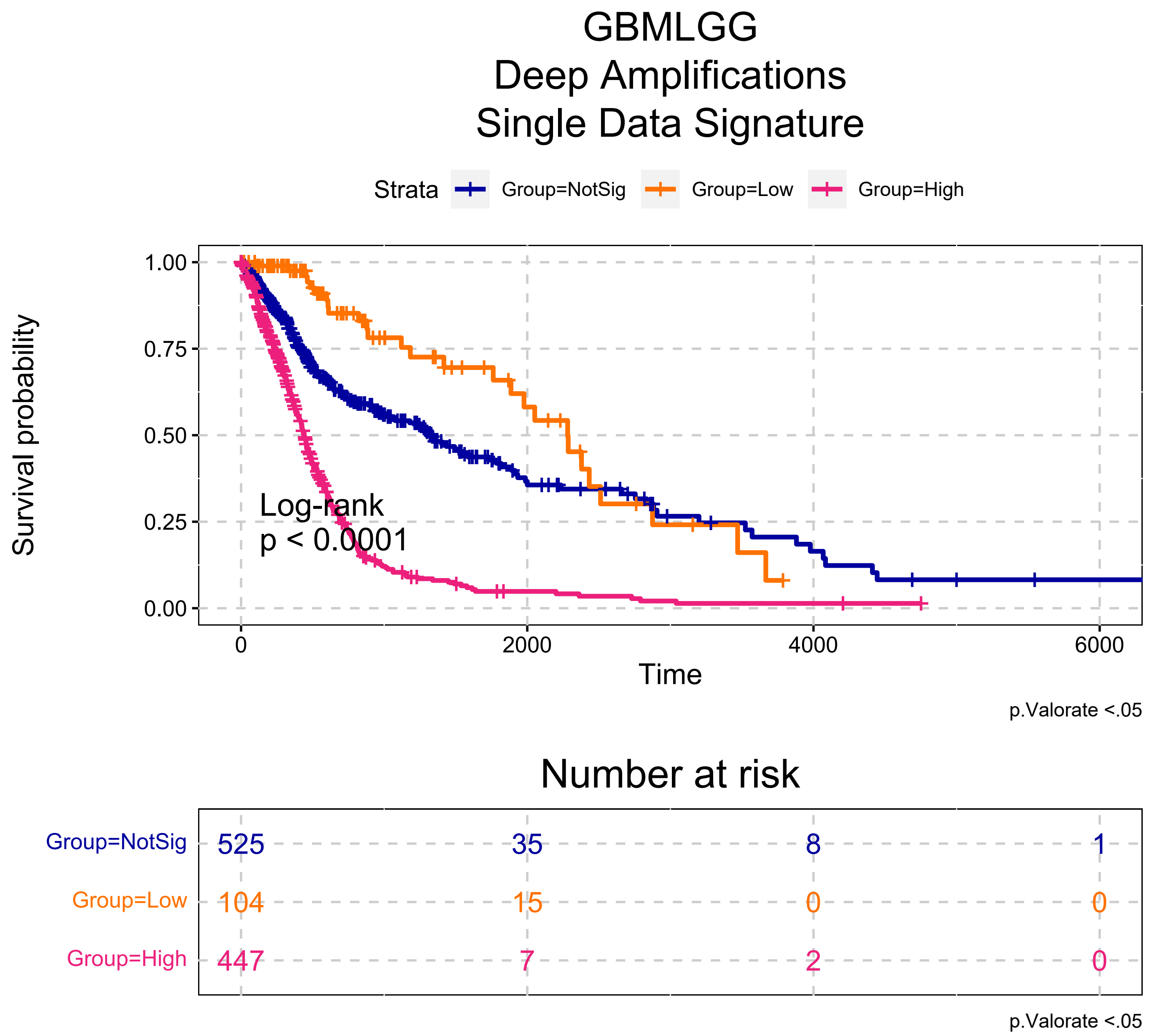

explanatory	beta	HR	L95	U95	p
Low	-0.62	0.54	0.36	0.81	0.00
High	1.05	2.86	2.37	3.45	0.00

n= 1080, number of events =522
Score(logrank) test = p <.0001

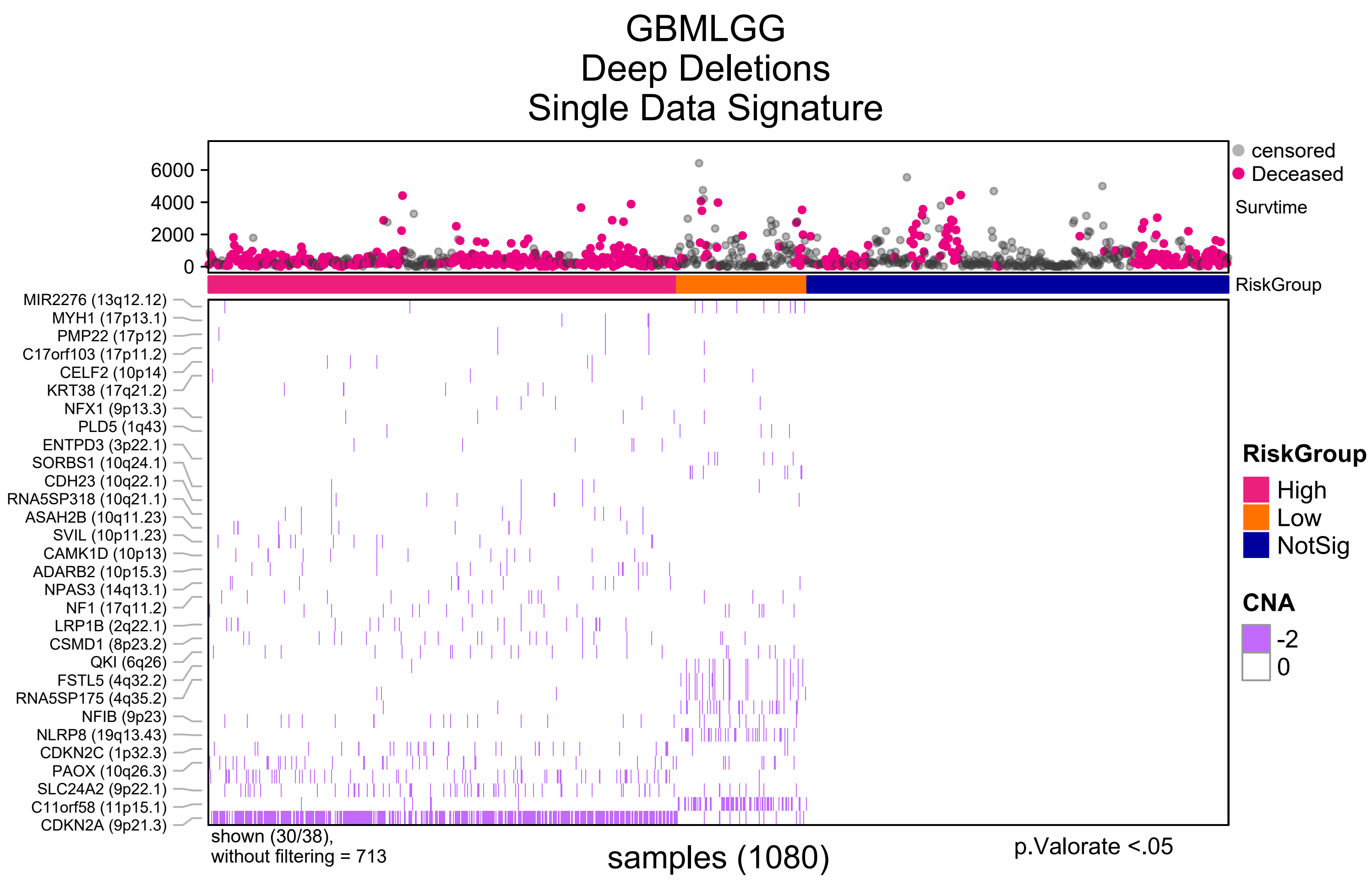
GBMLGG
Deep Deletions
Single Data Signature
6000
4000
2000
0
censored
Deceased
Survtime
RiskGroup
MIR2276 (13q12.12)
MYH1 (17p13.1)
PMP22 (17p12)
C17orf103 (17p11.2)
CELF2 (10p14)
KRT38 (17q21.2)
NFX1 (9p13.3)
PLD5 (1q43)
ENTPD3 (3p22.1)
SORBS1 (10q24.1)
CDH23 (10q22.1)
RNA5SP318 (10q21.1)
ASAH2B (10q11.23)
SVIL (10p11.23)
CAMK1D (10p13)
ADARB2 (10p15.3)
NPAS3 (14q13.1)
NF1 (17q11.2)
LRP1B (2q22.1)
CSMD1 (8p23.2)
QKI (6q26)
FSTL5 (4q32.2)
RNA5SP175 (4q35.2)
NFIB (9p23)
NLRP8 (19q13.43)
CDKN2C (1p32.3)
PAOX (10q26.3)
SLC24A2 (9p22.1)
C11orf58 (11p15.1)
CDKN2A (9p21.3)
RiskGroup
High
Low
NotSig
CNA
-2
0
shown (30/38),
without filtering = 713
samples (1080)
p.Valorate <.05

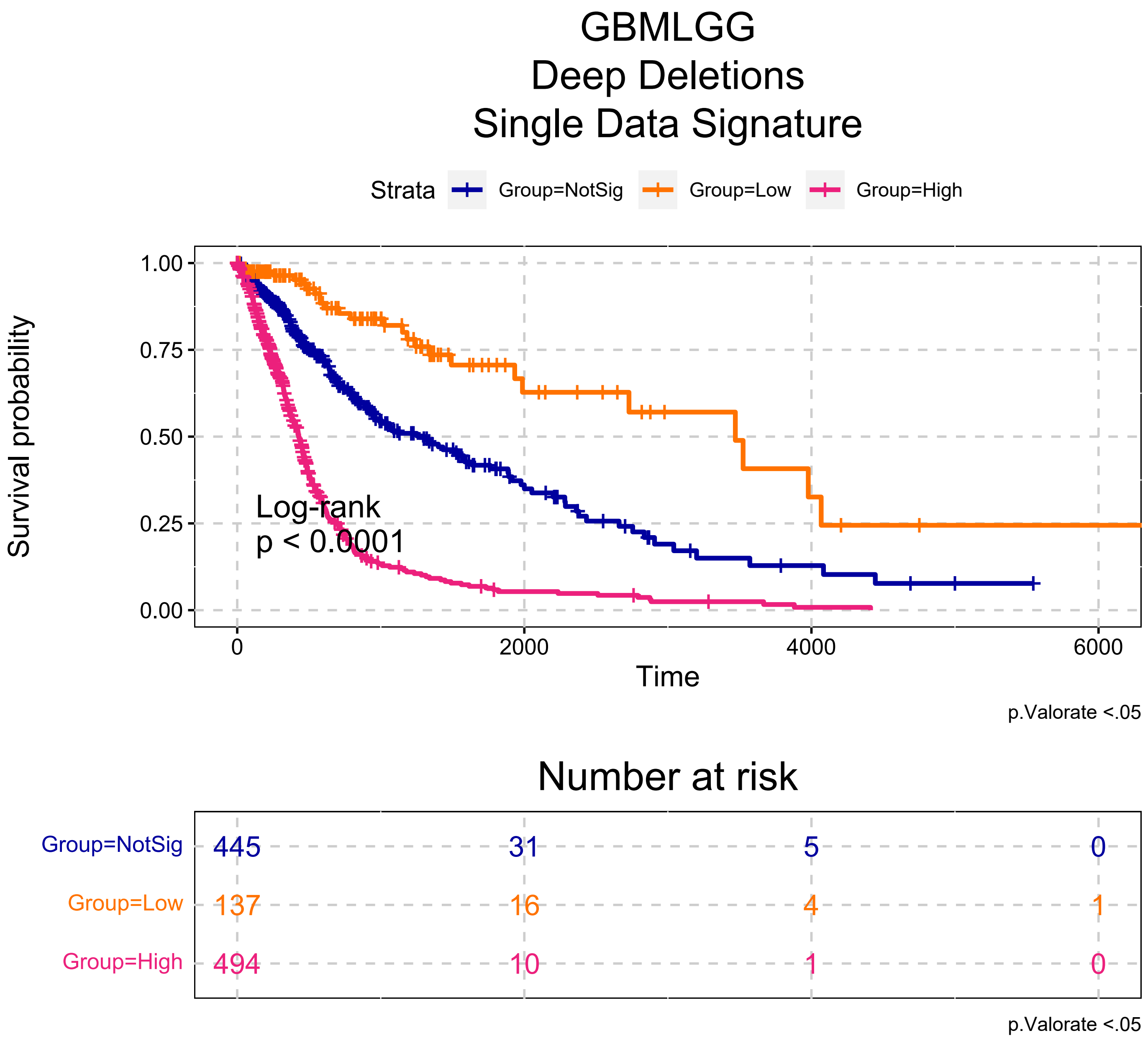

explanatory	beta	HR	L95	U95	p
Low	-0.92	0.40	0.26	0.61	0.00
High	1.10	3.01	2.47	3.65	0.00

n= 1080, number of events =522
Score(logrank) test = p <.0001

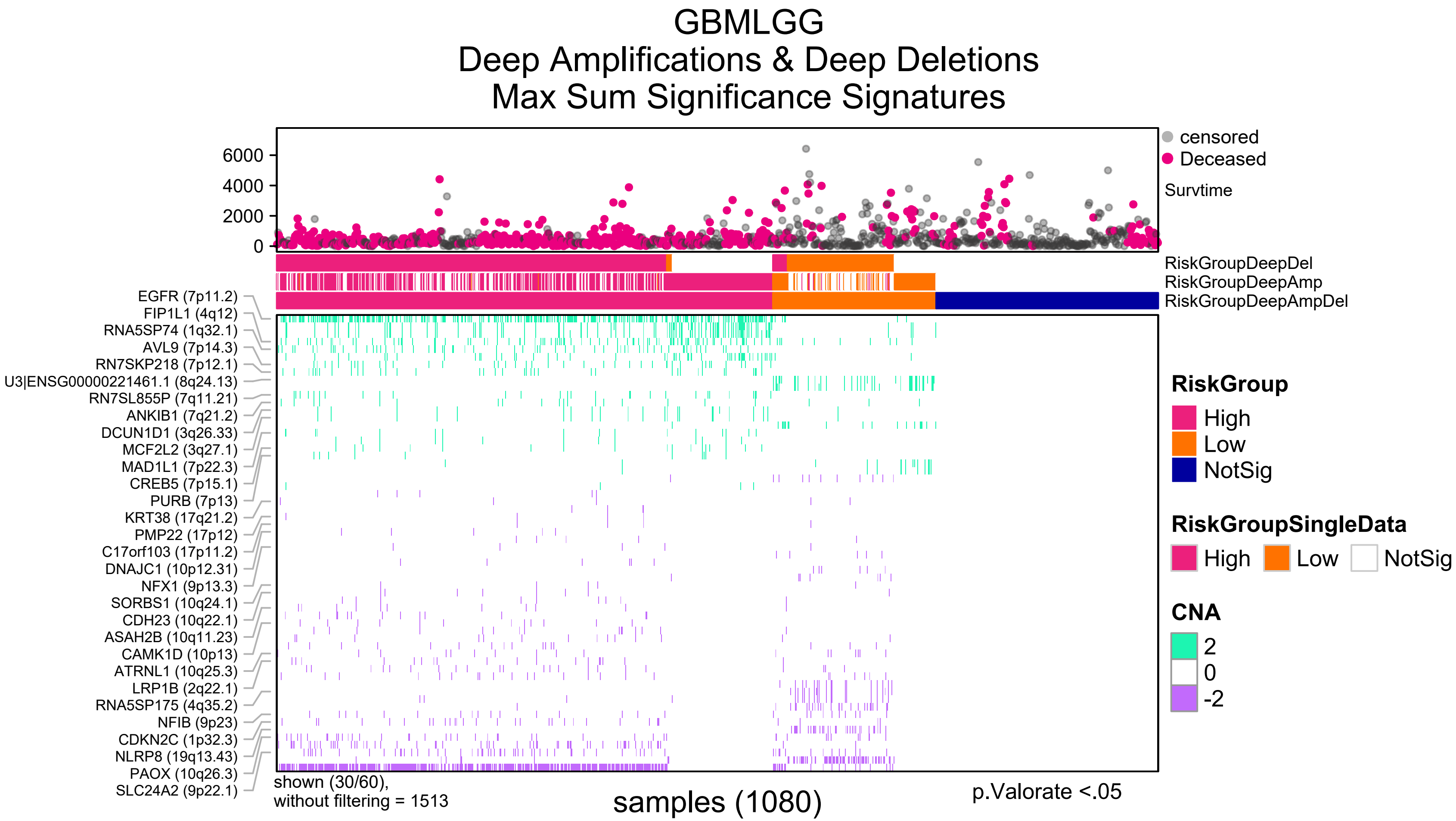
GBMLGG
Deep Amplifications & Deep Deletions
Max Sum Significance Signatures
6000
4000
2000
0
censored
Deceased
Survtime
RiskGroupDeepDel
RiskGroupDeepAmp
RiskGroupDeepAmpDel
EGFR (7p11.2)
FIP1L1 (4q12)
RNA5SP74 (1q32.1)
AVL9 (7p14.3)
RN7SKP218 (7p12.1)
U3|ENSG00000221461.1 (8q24.13)
RN7SL855P (7q11.21)
ANKIB1 (7q21.2)
DCUN1D1 (3q26.33)
MCF2L2 (3q27.1)
MAD1L1 (7p22.3)
CREB5 (7p15.1)
PURB (7p13)
KRT38 (17q21.2)
PMP22 (17p12)
C17orf103 (17p11.2)
DNAJC1 (10p12.31)
NFX1 (9p13.3)
SORBS1 (10q24.1)
CDH23 (10q22.1)
ASAH2B (10q11.23)
CAMK1D (10p13)
ATRNL1 (10q25.3)
LRP1B (2q22.1)
RNA5SP175 (4q35.2)
NFIB (9p23)
CDKN2C (1p32.3)
NLRP8 (19q13.43)
PAOX (10q26.3)
SLC24A2 (9p22.1)
RiskGroup
High
Low
NotSig
RiskGroupSingleData
High
Low
NotSig
CNA
2
0
-2
shown (30/60),
without filtering = 1513
samples (1080)
p.Valorate <.05

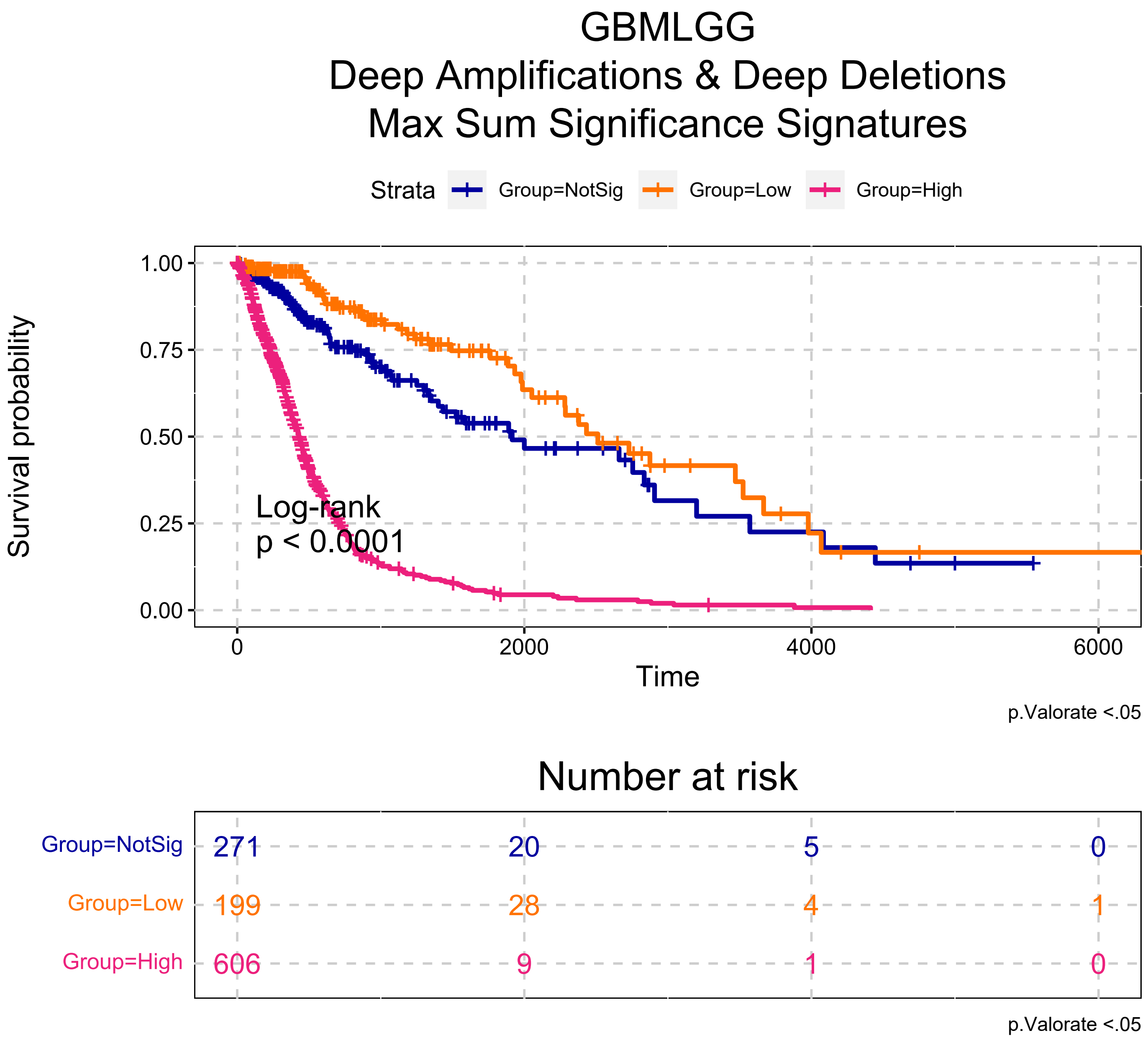

explanatory	beta	HR	L95	U95	p
Low	-0.44	0.64	0.44	0.95	0.03
High	1.57	4.78	3.66	6.26	0.00

n= 1080, number of events =522
Score(logrank) test = p <.0001

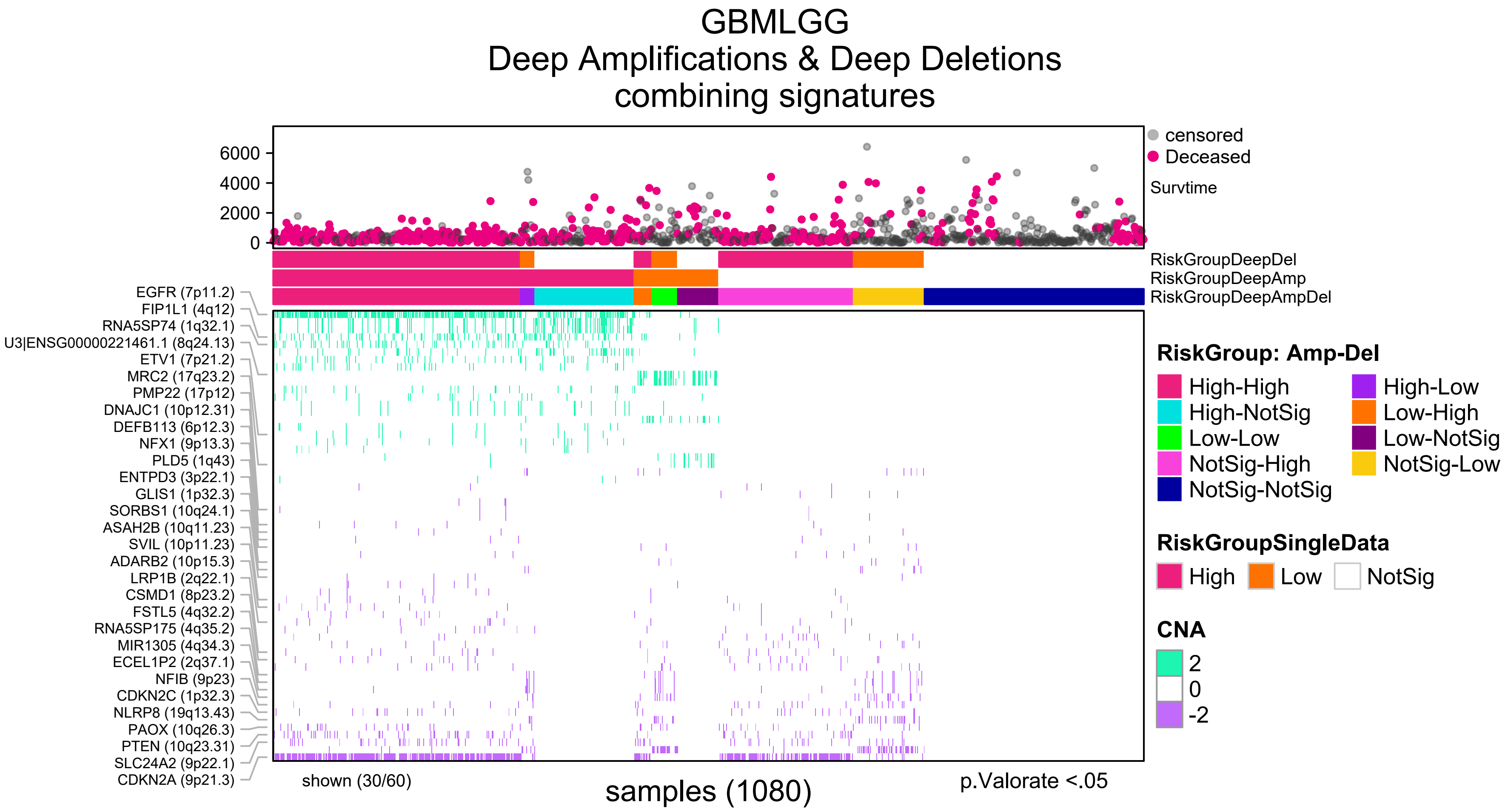

GBMLGG
Deep Amplifications & Deep Deletions
combining signatures
6000
4000
2000
0
censored
Deceased
Survtime
RiskGroupDeepDel
RiskGroupDeepAmp
RiskGroupDeepAmpDel
EGFR (7p11.2)
FIP1L1 (4q12)
RNA5SP74 (1q32.1)
U3|ENSG00000221461.1 (8q24.13)
ETV1 (7p21.2)
MRC2 (17q23.2)
PMP22 (17p12)
DNAJC1 (10p12.31)
DEFB113 (6p12.3)
NFX1 (9p13.3)
PLD5 (1q43)
ENTPD3 (3p22.1)
GLIS1 (1p32.3)
SORBS1 (10q24.1)
ASAH2B (10q11.23)
SVIL (10p11.23)
ADARB2 (10p15.3)
LRP1B (2q22.1)
CSMD1 (8p23.2)
FSTL5 (4q32.2)
RNA5SP175 (4q35.2)
MIR1305 (4q34.3)
ECEL1P2 (2q37.1)
NFIB (9p23)
CDKN2C (1p32.3)
NLRP8 (19q13.43)
PAOX (10q26.3)
PTEN (10q23.31)
SLC24A2 (9p22.1)
CDKN2A (9p21.3)
shown (30/60)
samples (1080)
p.Valorate <.05
RiskGroup: Amp-Del
High-High
High-Low
High-NotSig
Low-High
Low-Low
Low-NotSig
NotSig-High
NotSig-Low
NotSig-NotSig
RiskGroupSingleData
High
Low
NotSig
CNA
2
0
-2

GBMLGG
Deep Amplifications & Deep Deletions combining signatures

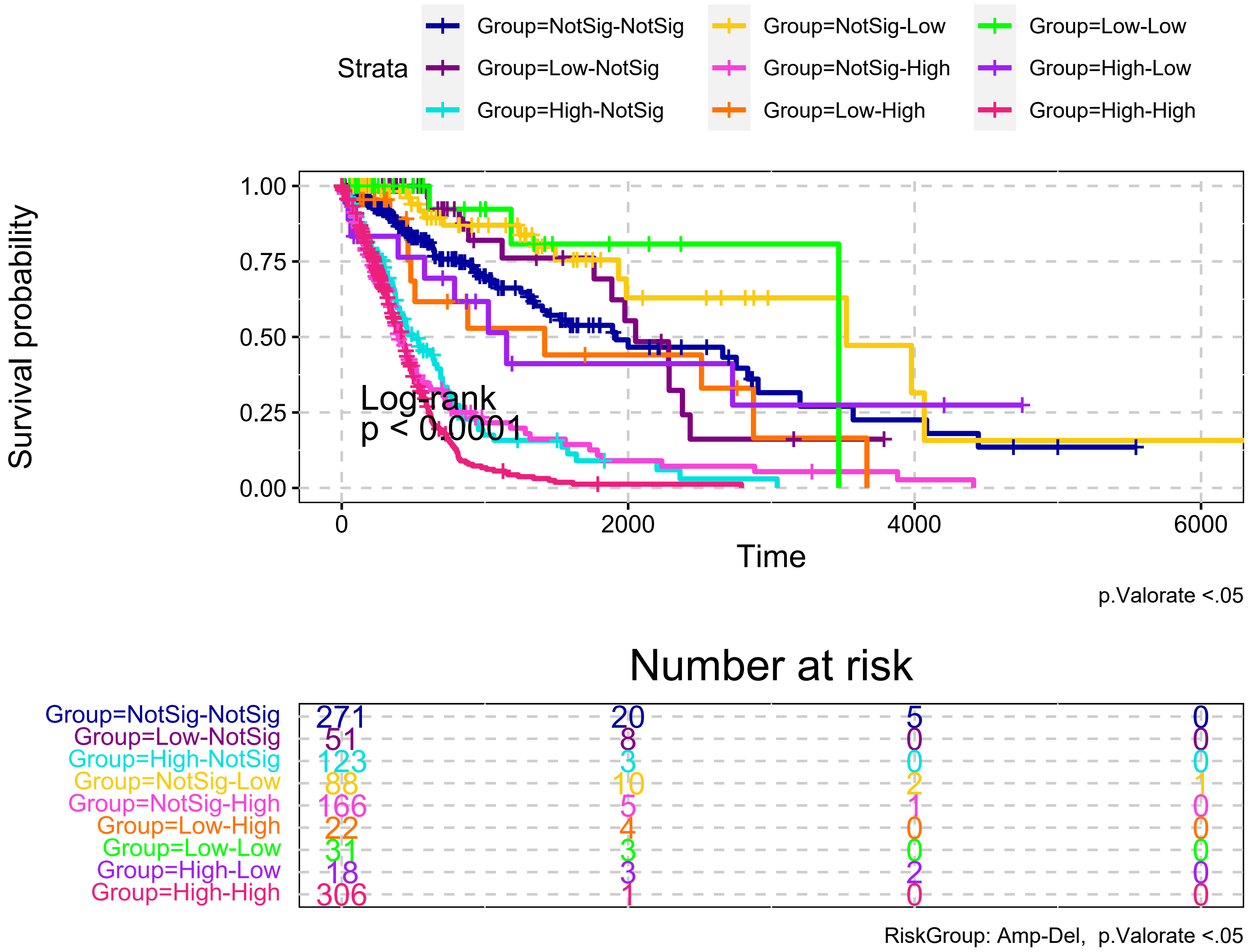

explanatory	beta	HR	L95	U95	p
Low-NotSig	-0.25	0.78	0.43	1.42	0.42
High-NotSig	1.36	3.91	2.79	5.47	0.00
NotSig-Low	-0.70	0.50	0.28	0.88	0.02
NotSig-High	1.42	4.15	3.04	5.68	0.00
Low-High	0.38	1.47	0.77	2.78	0.24
Low-Low	-1.02	0.36	0.11	1.15	0.08
High-Low	0.18	1.20	0.60	2.42	0.61
High-High	1.77	5.85	4.39	7.79	0.00

n= 1080, number of events =522
Score(logrank) test = p <.0001
