# Supplementary material for: Systematic Modeling of Risk-Associated Copy Number Alterations in Cancer
Source: Int J Mol Sci. 2024 Sep 27;25(19):10455. doi: 10.3390/ijms251910455 (PMC11477427; doi:10.3390/ijms251910455)
Supplement: Supplementary file 1 [file ijms-25-10455-s001.zip › GBMSignatureV12-sinSombreado.pdf]

GBM
All Amplifications
Single Data Signature

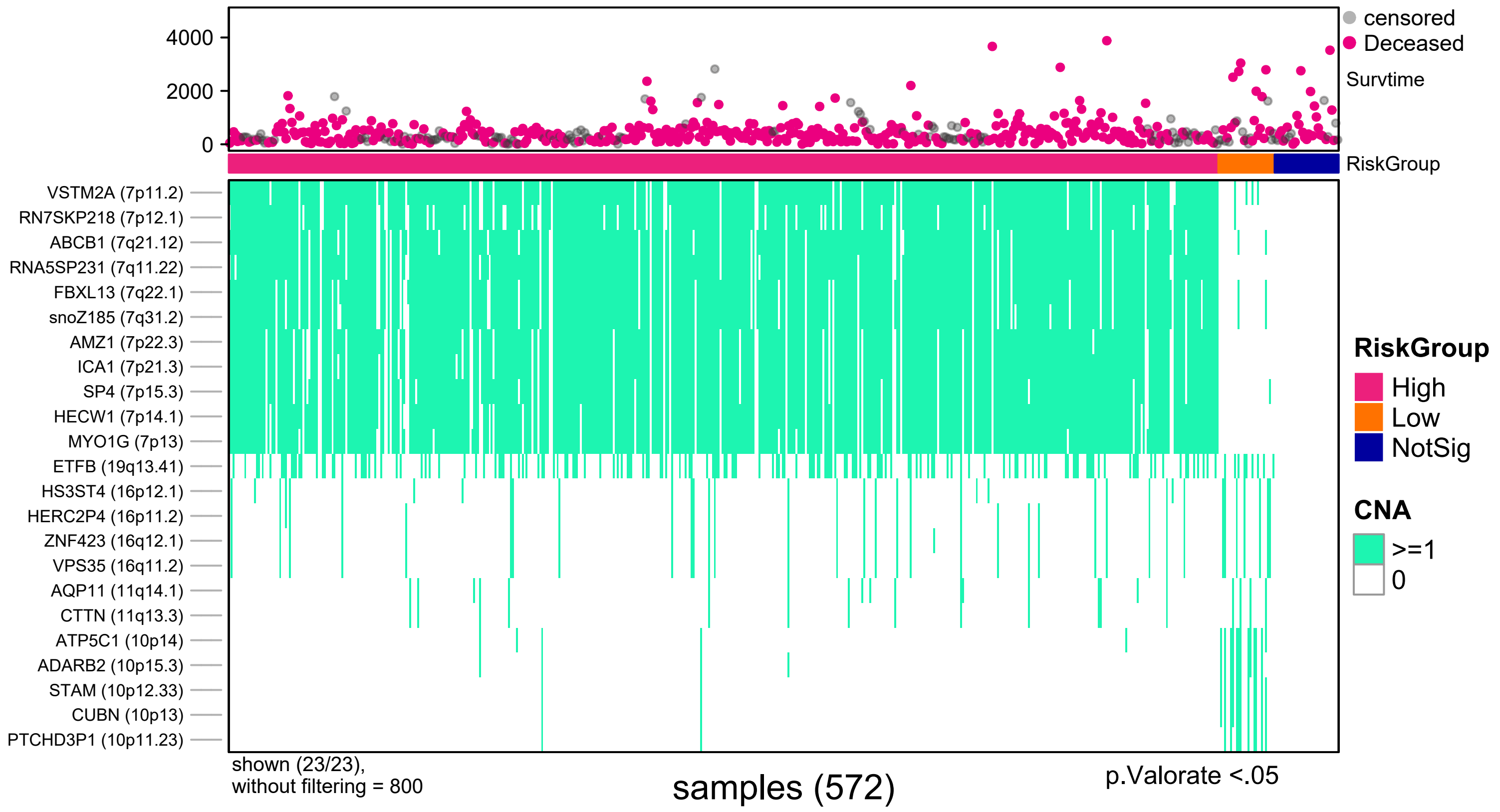

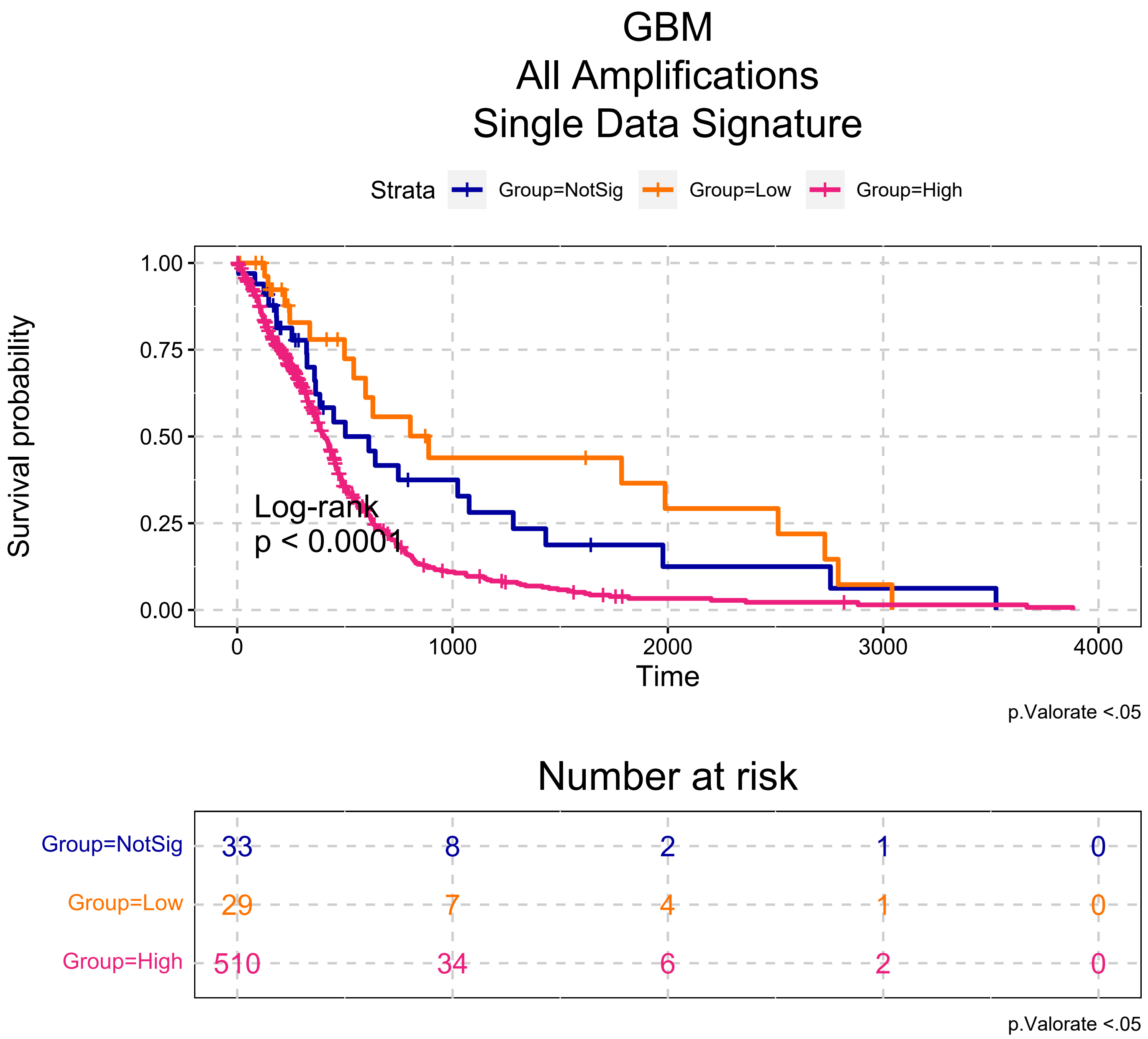

explanatory	beta	HR	L95	U95	p
Low	-0.39	0.68	0.36	1.26	0.22
High	0.51	1.67	1.10	2.53	0.02

n= 572, number of events =430
Score(logrank) test = p <.0001

GBM
All Deletions
Single Data Signature

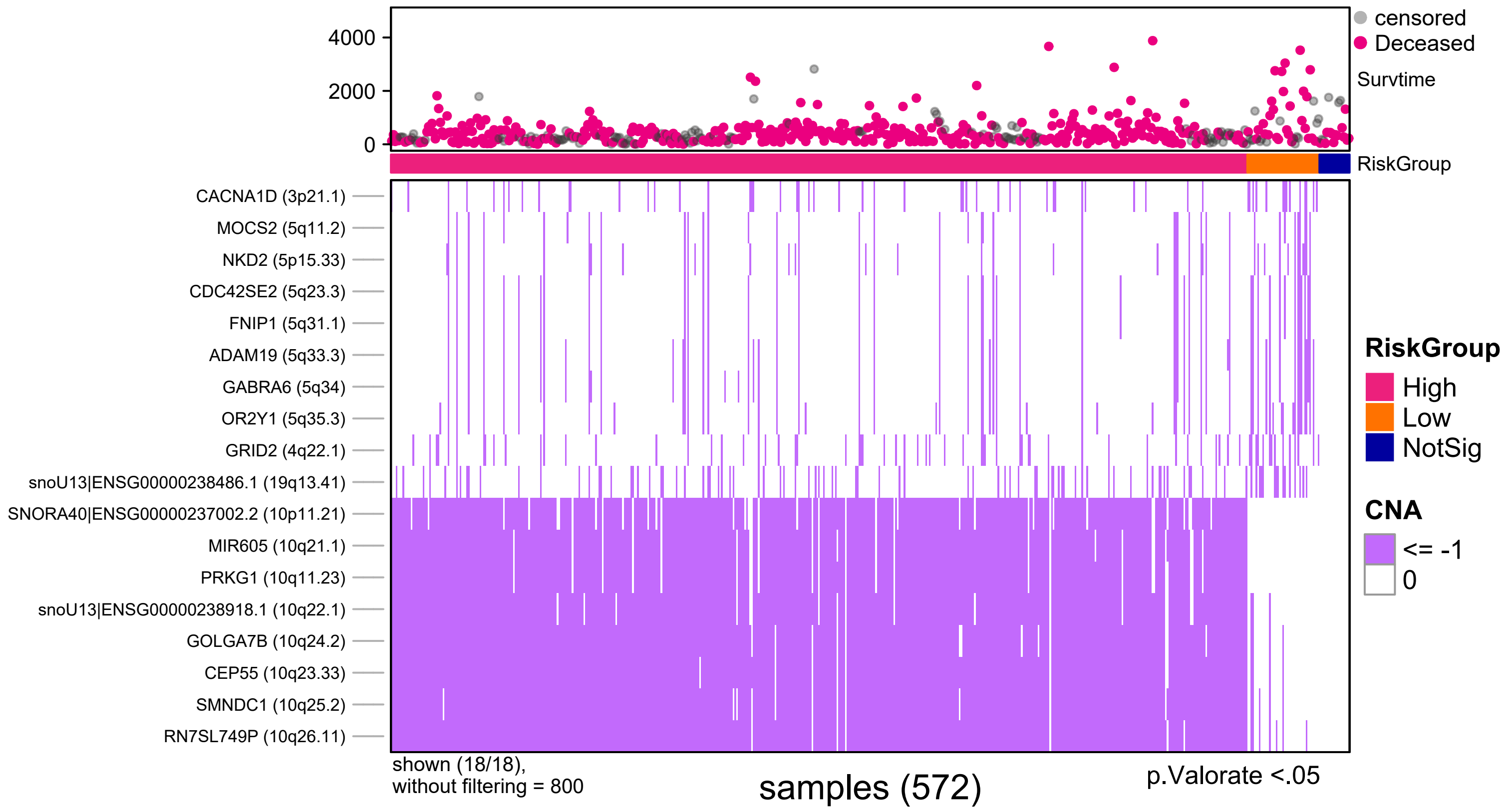

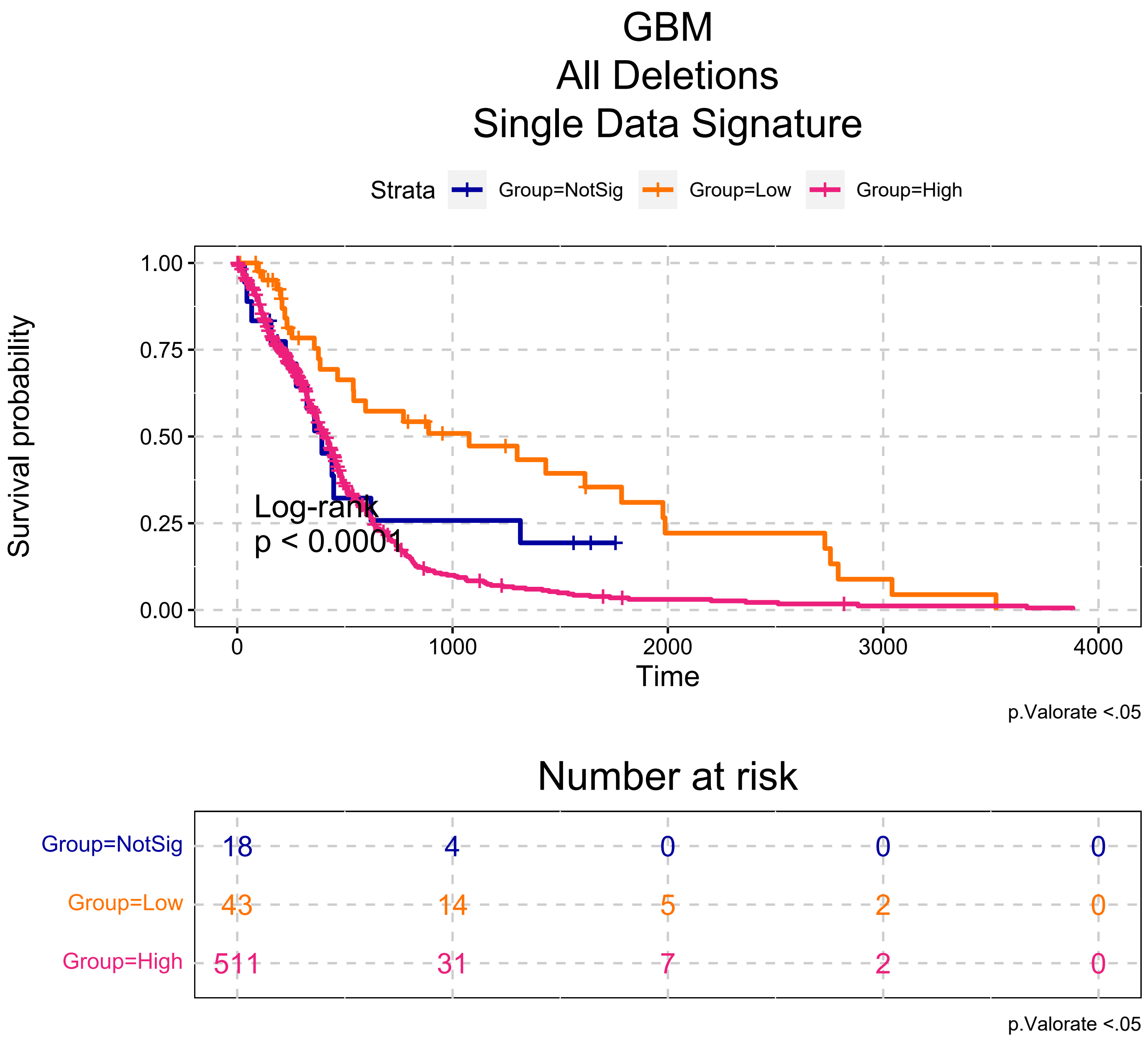

explanatory	beta	HR	L95	U95	p
Low	-0.55	0.58	0.30	1.12	0.10
High	0.33	1.39	0.80	2.42	0.25

n= 572, number of events =430
Score(logrank) test = p <.0001

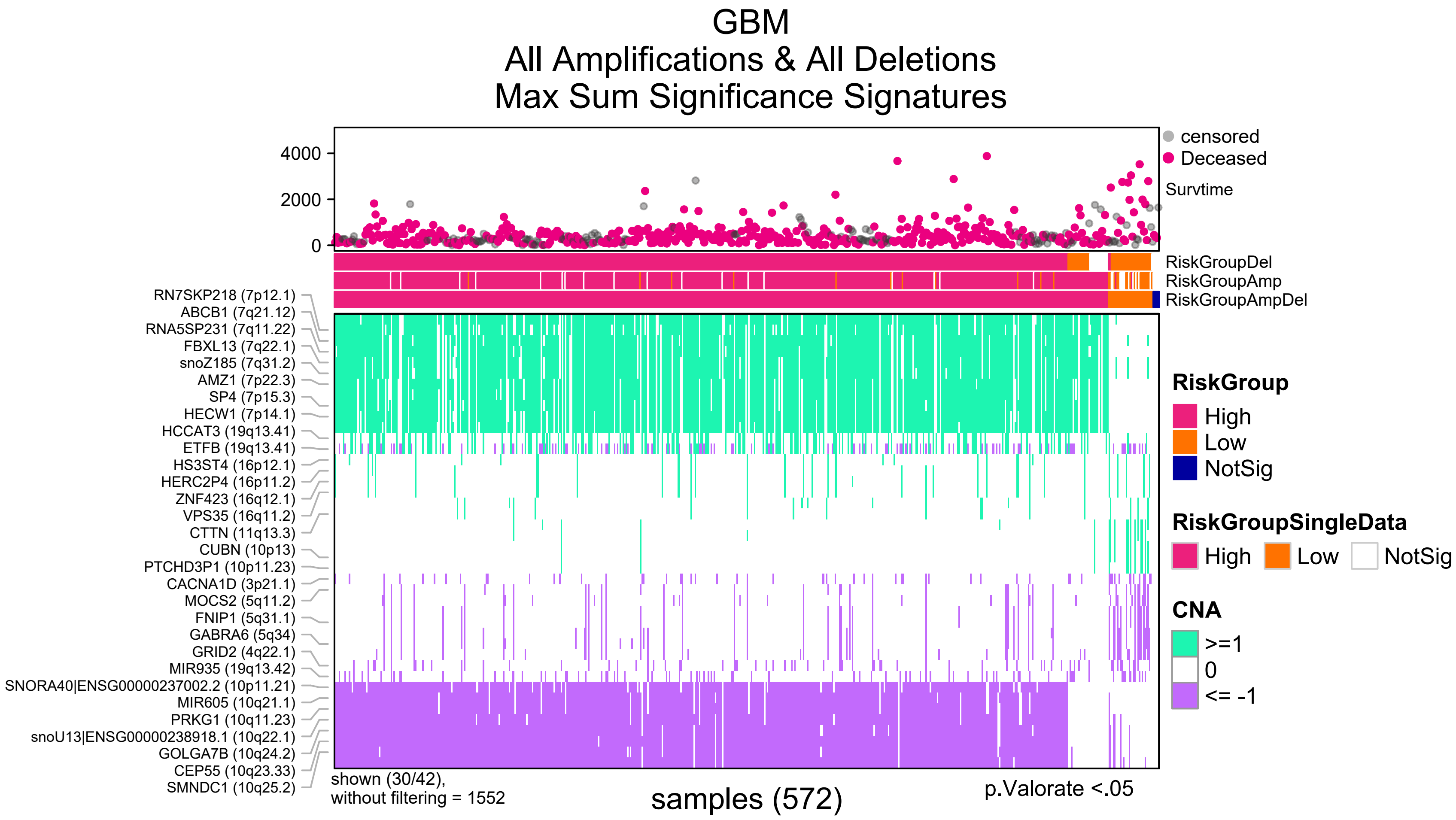

GBM
All Amplifications & All Deletions
Max Sum Significance Signatures
4000
2000
0
censored
Deceased
Survtime
RiskGroupDel
RiskGroupAmp
RiskGroupAmpDel
RN7SKP218 (7p12.1)
ABCB1 (7q21.12)
RNA5SP231 (7q11.22)
FBXL13 (7q22.1)
snoZ185 (7q31.2)
AMZ1 (7p22.3)
SP4 (7p15.3)
HECW1 (7p14.1)
HCCAT3 (19q13.41)
ETFB (19q13.41)
HS3ST4 (16p12.1)
HERC2P4 (16p11.2)
ZNF423 (16q12.1)
VPS35 (16q11.2)
CTTN (11q13.3)
CUBN (10p13)
PTCHD3P1 (10p11.23)
CACNA1D (3p21.1)
MOCS2 (5q11.2)
FNIP1 (5q31.1)
GABRA6 (5q34)
GRID2 (4q22.1)
MIR935 (19q13.42)
SNORA40|ENSG00000237002.2 (10p11.21)
MIR605 (10q21.1)
PRKG1 (10q11.23)
snoU13|ENSG00000238918.1 (10q22.1)
GOLGA7B (10q24.2)
CEP55 (10q23.33)
SMNDC1 (10q25.2)
RiskGroup
High
Low
NotSig
RiskGroupSingleData
High
Low
NotSig
CNA
>=1
0
<= -1
shown (30/42),
without filtering = 1552
samples (572)
p.Valorate <.05

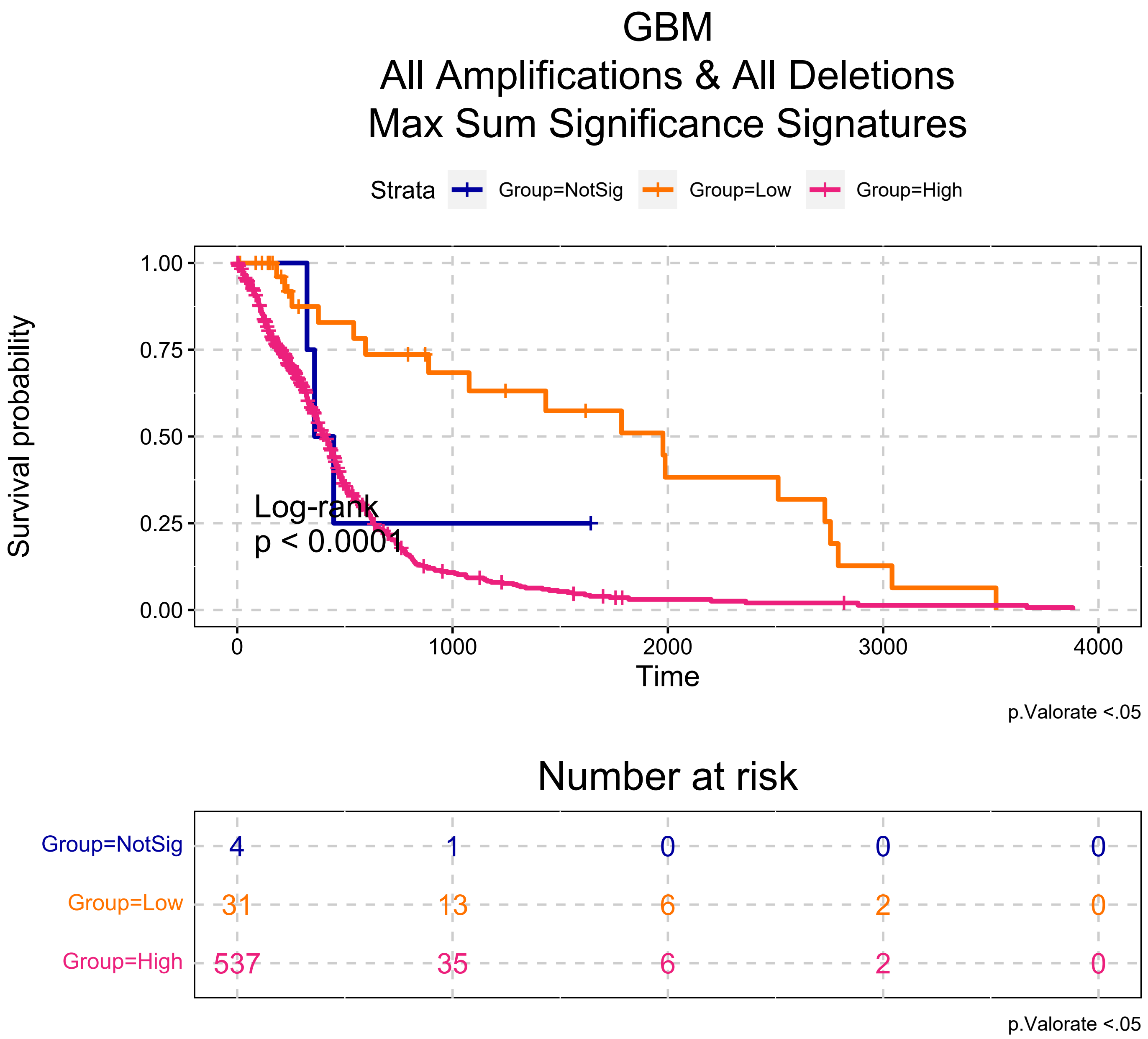

explanatory	beta	HR	L95	U95	p
Low	-0.72	0.49	0.14	1.66	0.25
High	0.50	1.64	0.53	5.13	0.39

n= 572, number of events =430
Score(logrank) test = p <.0001

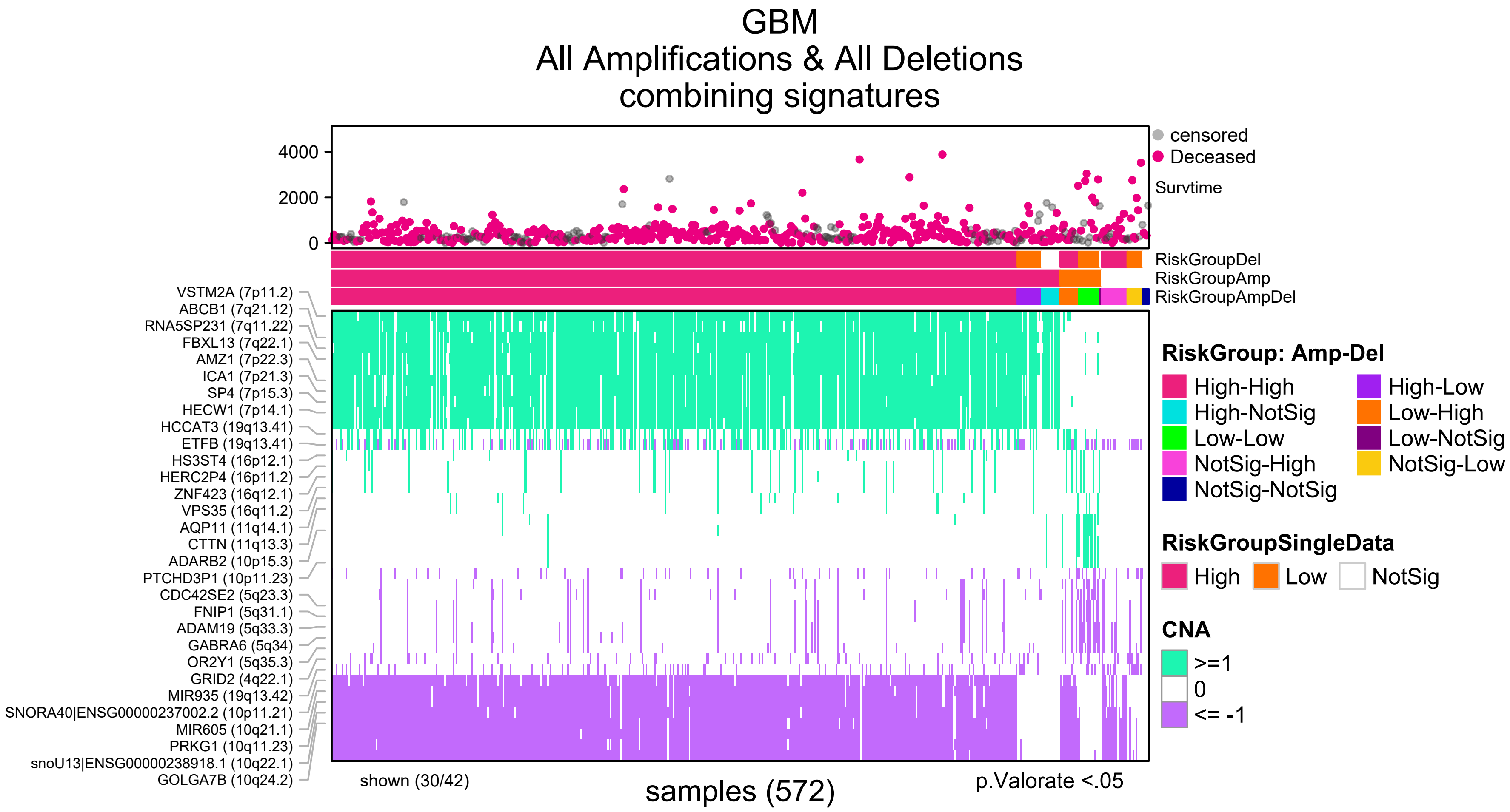
GBM
All Amplifications & All Deletions
combining signatures
4000
2000
0
censored
Deceased
Survtime
RiskGroupDel
RiskGroupAmp
RiskGroupAmpDel
VSTM2A (7p11.2)
ABCB1 (7q21.12)
RNA5SP231 (7q11.22)
FBXL13 (7q22.1)
AMZ1 (7p22.3)
ICA1 (7p21.3)
SP4 (7p15.3)
HECW1 (7p14.1)
HCCAT3 (19q13.41)
ETFB (19q13.41)
HS3ST4 (16p12.1)
HERC2P4 (16p11.2)
ZNF423 (16q12.1)
VPS35 (16q11.2)
AQP11 (11q14.1)
CTTN (11q13.3)
ADARB2 (10p15.3)
PTCHD3P1 (10p11.23)
CDC42SE2 (5q23.3)
FNIP1 (5q31.1)
ADAM19 (5q33.3)
GABRA6 (5q34)
OR2Y1 (5q35.3)
GRID2 (4q22.1)
MIR935 (19q13.42)
SNORA40|ENSG00000237002.2 (10p11.21)
MIR605 (10q21.1)
PRKG1 (10q11.23)
snoU13|ENSG00000238918.1 (10q22.1)
GOLGA7B (10q24.2)
shown (30/42)
samples (572)
p.Valorate <.05
RiskGroup: Amp-Del
High-High
High-NotSig
Low-Low
NotSig-High
NotSig-NotSig
High-Low
Low-High
Low-NotSig
NotSig-Low
RiskGroupSingleData
High
Low
NotSig
CNA
>=1
0
<= -1

GBM
All Amplifications & All Deletions combining signatures

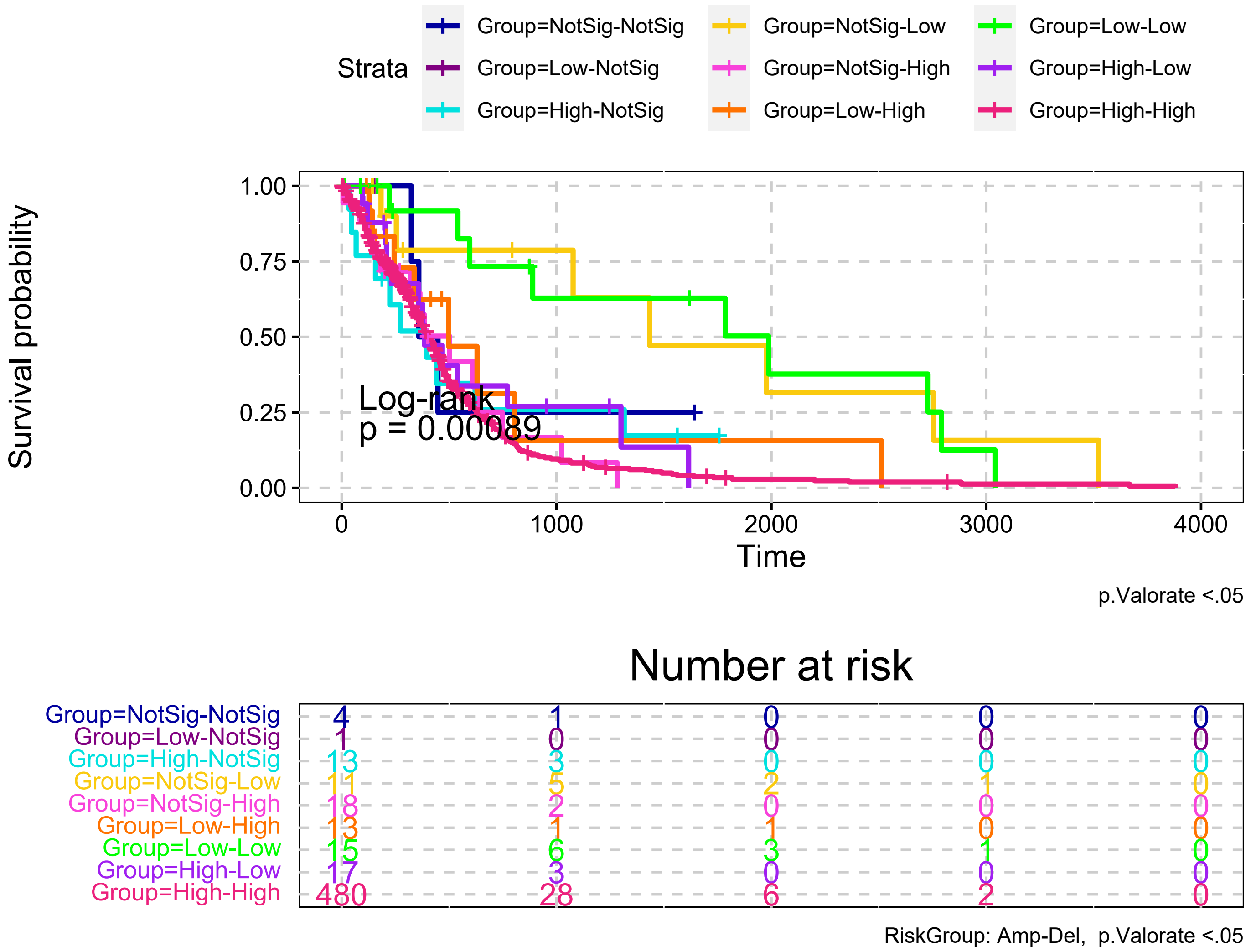

explanatory	beta	HR	L95	U95	p
Low-NotSig	-11.57	0.00	0.00	Inf	0.99
High-NotSig	0.25	1.28	0.35	4.66	0.71
NotSig-Low	-0.64	0.53	0.14	2.05	0.36
NotSig-High	0.49	1.63	0.47	5.67	0.45
Low-High	0.11	1.12	0.30	4.24	0.87
Low-Low	-0.74	0.48	0.13	1.77	0.27
High-Low	0.21	1.23	0.35	4.33	0.74
High-High	0.51	1.67	0.54	5.22	0.38

n= 572, number of events =430
Score(logrank) test = 0.001

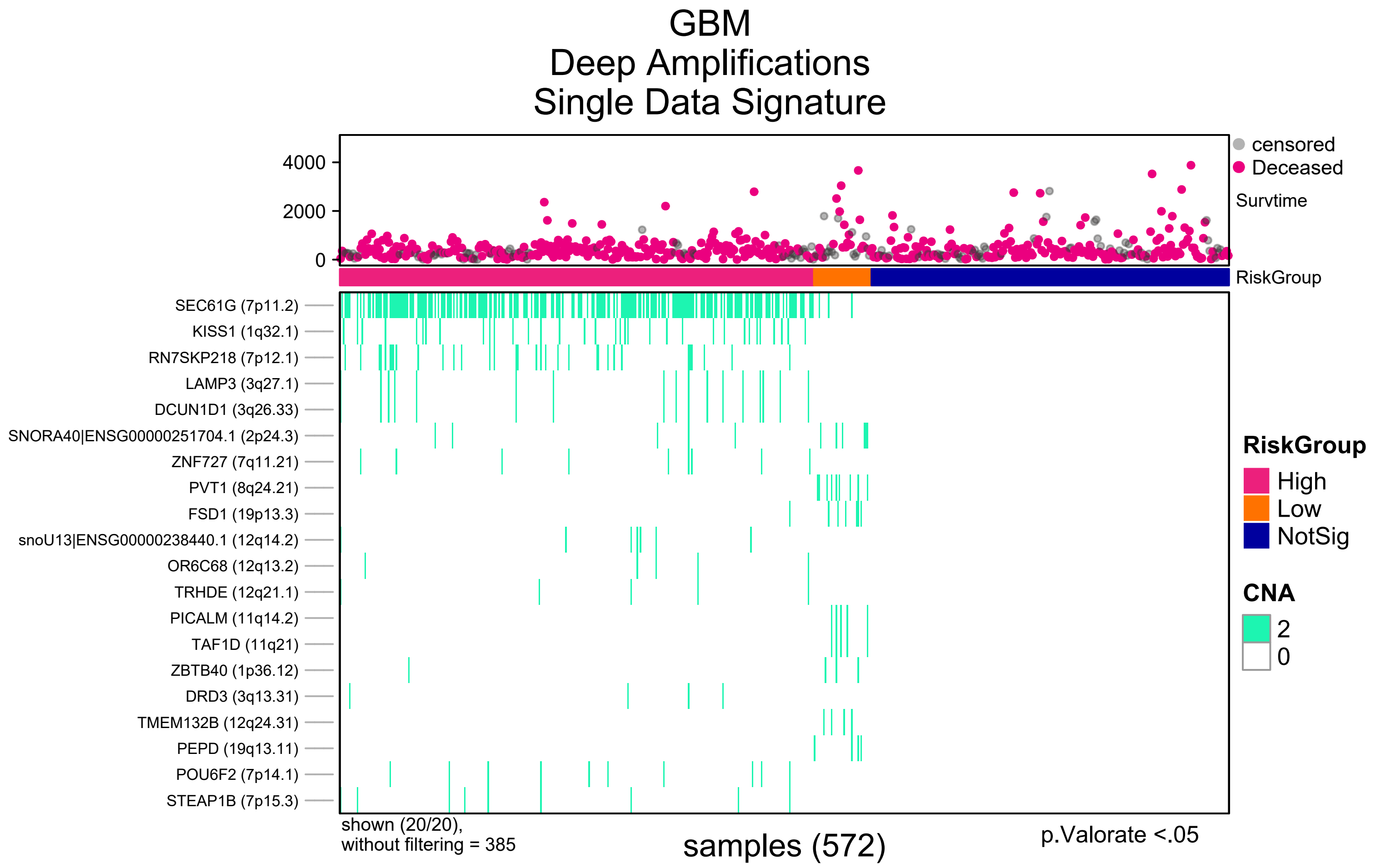
GBM
Deep Amplifications
Single Data Signature
4000
2000
0
censored
Deceased
Survtime
RiskGroup
SEC61G (7p11.2)
KISS1 (1q32.1)
RN7SKP218 (7p12.1)
LAMP3 (3q27.1)
DCUN1D1 (3q26.33)
SNORA40|ENSG00000251704.1 (2p24.3)
ZNF727 (7q11.21)
PVT1 (8q24.21)
FSD1 (19p13.3)
snoU13|ENSG00000238440.1 (12q14.2)
OR6C68 (12q13.2)
TRHDE (12q21.1)
PICALM (11q14.2)
TAF1D (11q21)
ZBTB40 (1p36.12)
DRD3 (3q13.31)
TMEM132B (12q24.31)
PEPD (19q13.11)
POU6F2 (7p14.1)
STEAP1B (7p15.3)
RiskGroup
High
Low
NotSig
CNA
2
0
shown (20/20),
without filtering = 385
samples (572)
p.Valorate <.05

GBM
Deep Amplifications
Single Data Signature

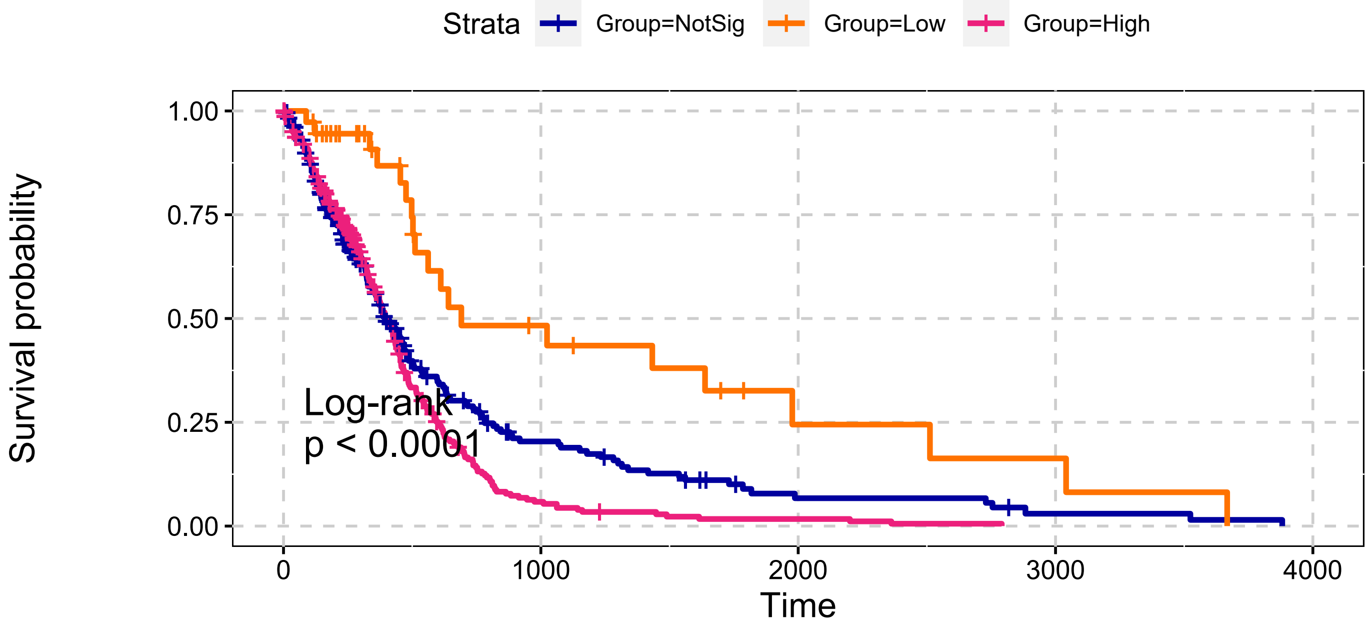

p.Valorate <.05

Number at risk

	0	1000	2000	3000	4000
Group=NotSig	230	27	6	2	0
Group=Low	37	10	3	2	0
Group=High	305	12	3	0	0

p.Valorate <.05

explanatory	beta	HR	L95	U95	p
Low	-0.81	0.44	0.28	0.71	0.00
High	0.30	1.35	1.10	1.65	0.00

n= 572, number of events =430
Score(logrank) test = p <.0001

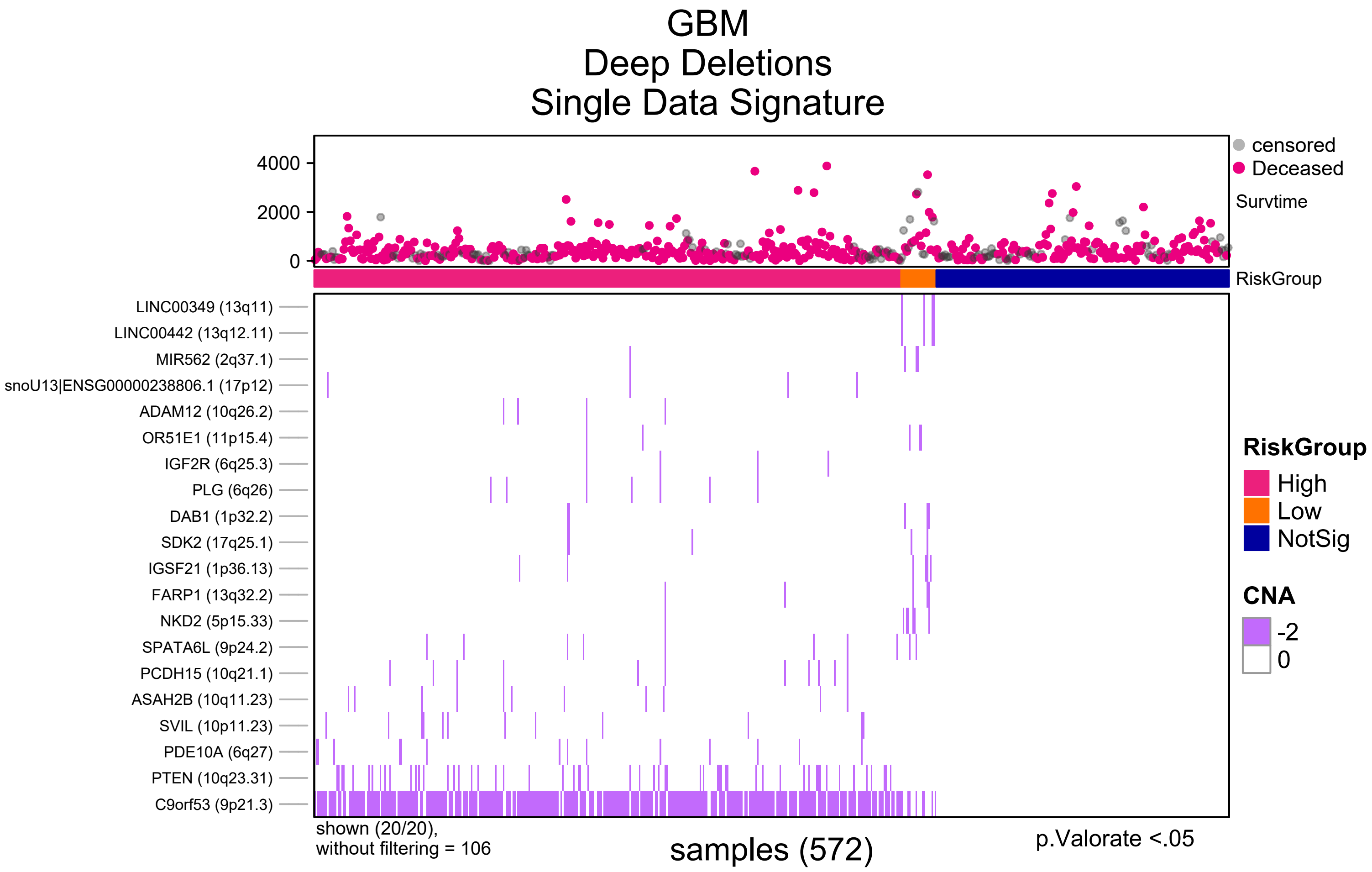

GBM
Deep Deletions
Single Data Signature
4000
2000
0
censored
Deceased
Survtime
RiskGroup
LINC00349 (13q11)
LINC00442 (13q12.11)
MIR562 (2q37.1)
snoU13|ENSG00000238806.1 (17p12)
ADAM12 (10q26.2)
OR51E1 (11p15.4)
IGF2R (6q25.3)
PLG (6q26)
DAB1 (1p32.2)
SDK2 (17q25.1)
IGSF21 (1p36.13)
FARP1 (13q32.2)
NKD2 (5p15.33)
SPATA6L (9p24.2)
PCDH15 (10q21.1)
ASAH2B (10q11.23)
SVIL (10p11.23)
PDE10A (6q27)
PTEN (10q23.31)
C9orf53 (9p21.3)
RiskGroup
High
Low
NotSig
CNA
-2
0
shown (20/20),
without filtering = 106
samples (572)
p.Valorate <.05

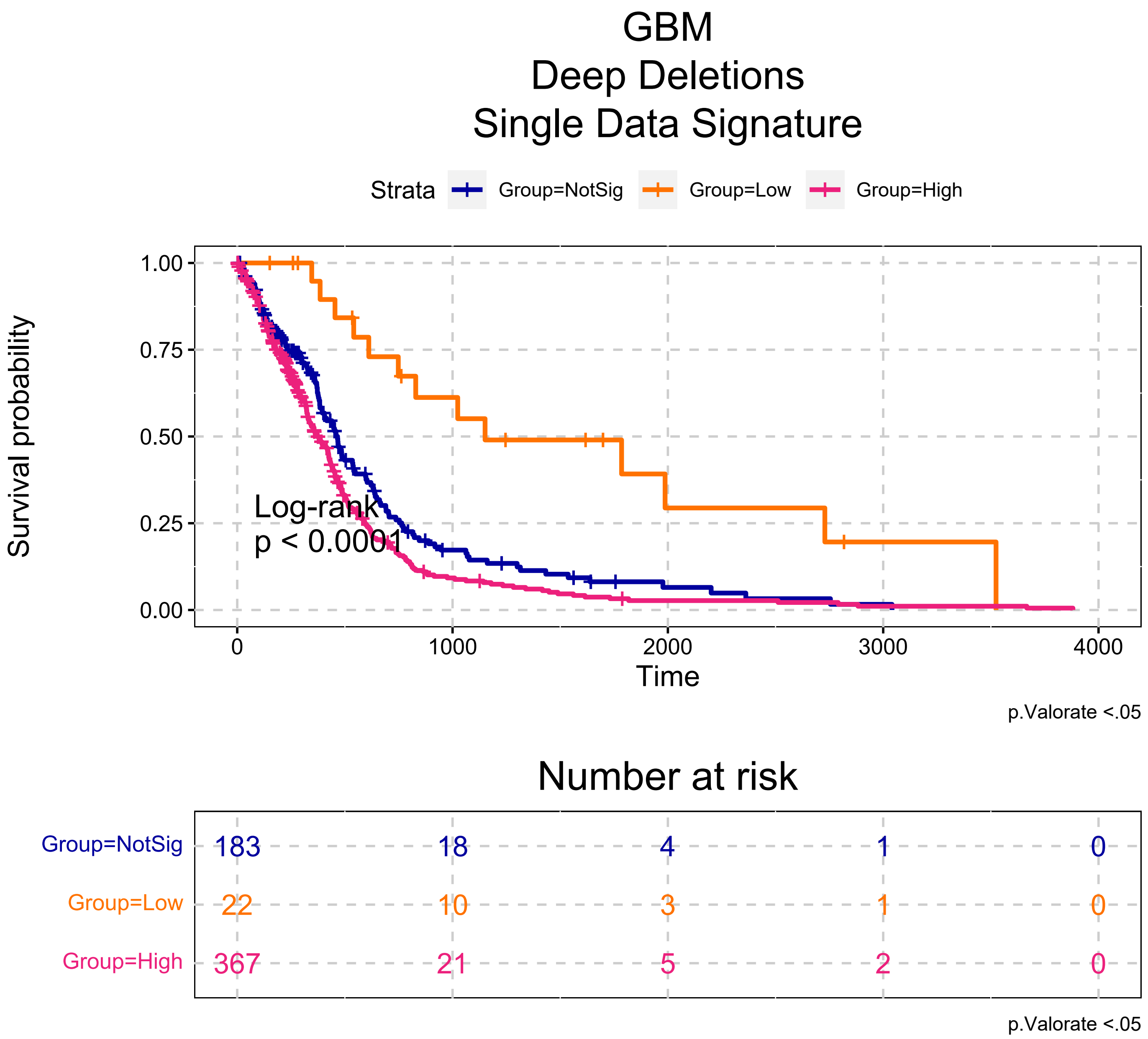

explanatory	beta	HR	L95	U95	p
Low	-1.03	0.36	0.20	0.63	0.00
High	0.28	1.33	1.08	1.63	0.01

n= 572, number of events =430
Score(logrank) test = p <.0001

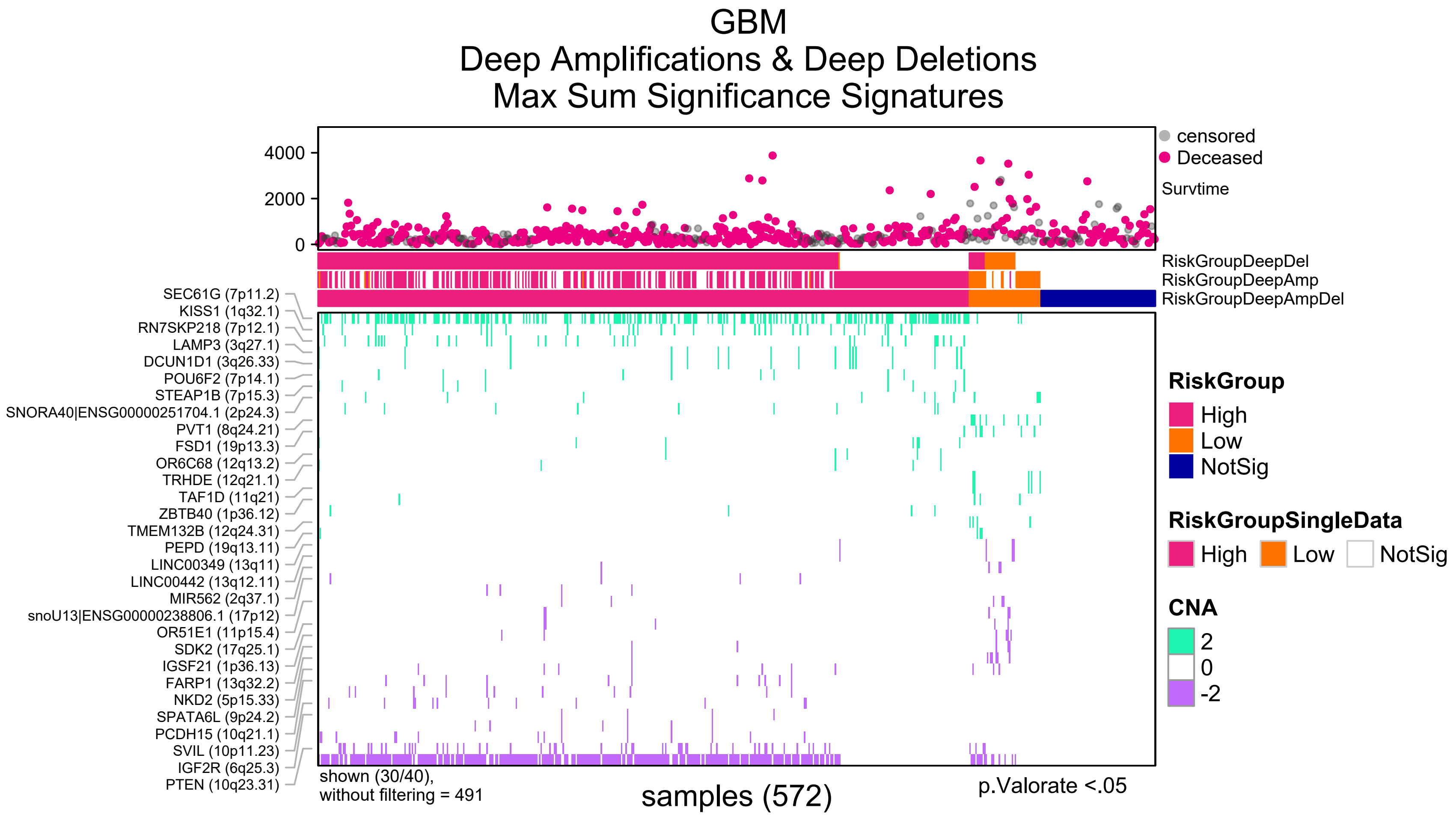

GBM
Deep Amplifications & Deep Deletions
Max Sum Significance Signatures
4000
2000
0
censored
Deceased
Survtime
RiskGroupDeepDel
RiskGroupDeepAmp
RiskGroupDeepAmpDel
SEC61G (7p11.2)
KISS1 (1q32.1)
RN7SKP218 (7p12.1)
LAMP3 (3q27.1)
DCUN1D1 (3q26.33)
POU6F2 (7p14.1)
STEAP1B (7p15.3)
SNORA40|ENSG00000251704.1 (2p24.3)
PVT1 (8q24.21)
FSD1 (19p13.3)
OR6C68 (12q13.2)
TRHDE (12q21.1)
TAF1D (11q21)
ZBTB40 (1p36.12)
TMEM132B (12q24.31)
PEPD (19q13.11)
LINC00349 (13q11)
LINC00442 (13q12.11)
MIR562 (2q37.1)
snoU13|ENSG00000238806.1 (17p12)
OR51E1 (11p15.4)
SDK2 (17q25.1)
IGSF21 (1p36.13)
FARP1 (13q32.2)
NKD2 (5p15.33)
SPATA6L (9p24.2)
PCDH15 (10q21.1)
SVIL (10p11.23)
IGF2R (6q25.3)
PTEN (10q23.31)
RiskGroup
High
Low
NotSig
RiskGroupSingleData
High
Low
NotSig
CNA
2
0
-2
shown (30/40),
without filtering = 491
samples (572)
p.Valorate <.05

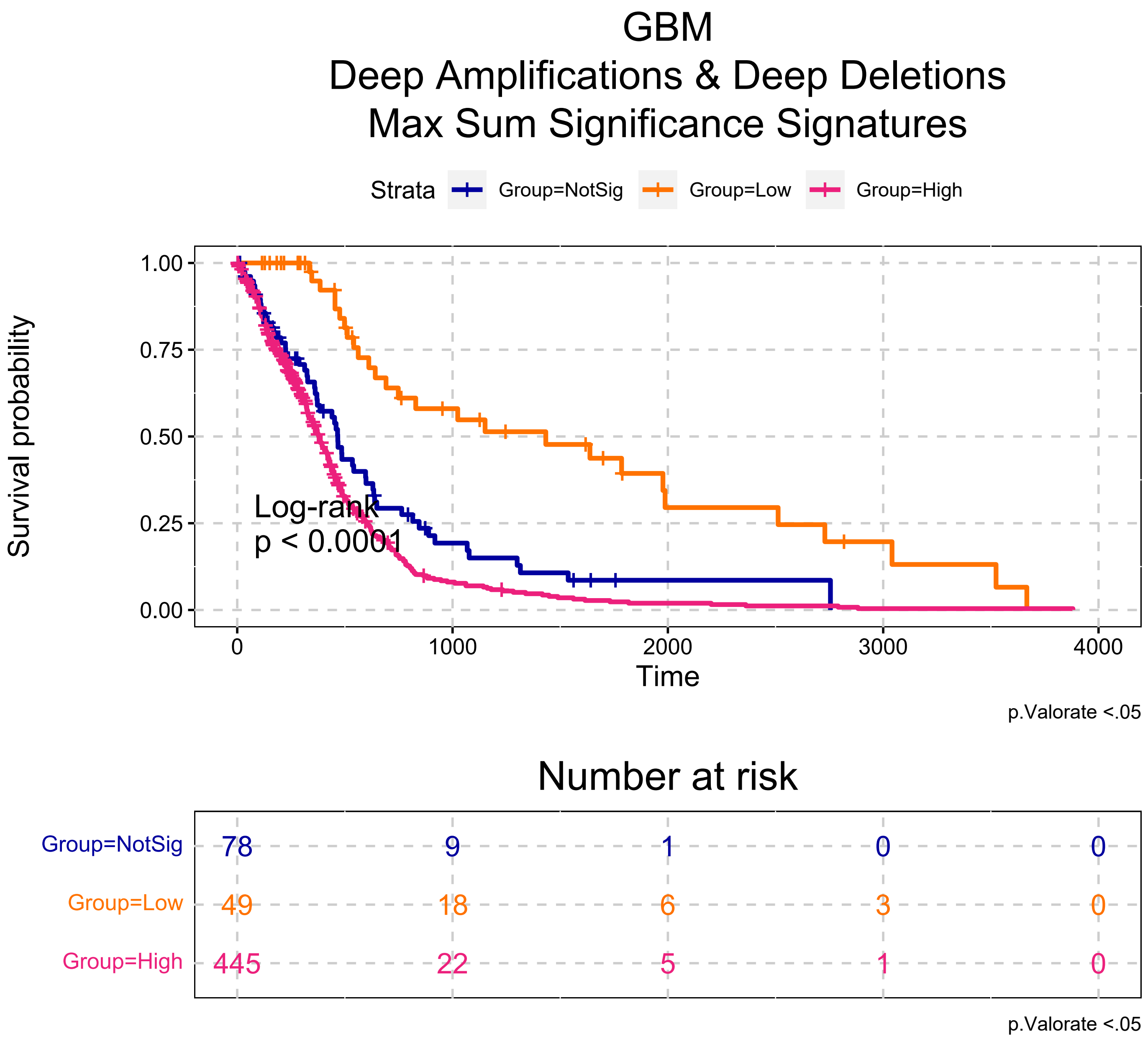

explanatory	beta	HR	L95	U95	p
Low	-1.01	0.37	0.23	0.58	0.00
High	0.35	1.42	1.07	1.89	0.02

n= 572, number of events =430
Score(logrank) test = p <.0001

GBM
Deep Amplifications & Deep Deletions combining signatures

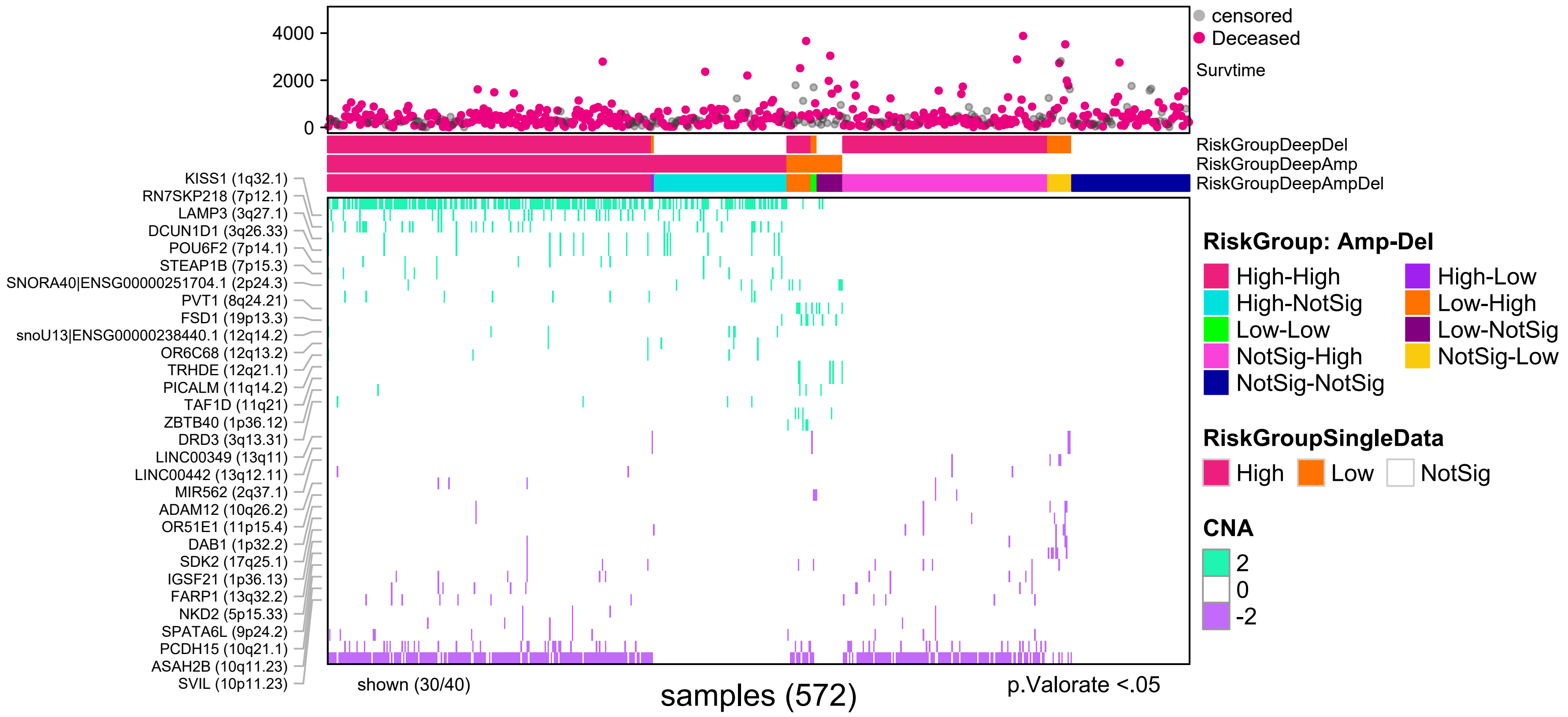

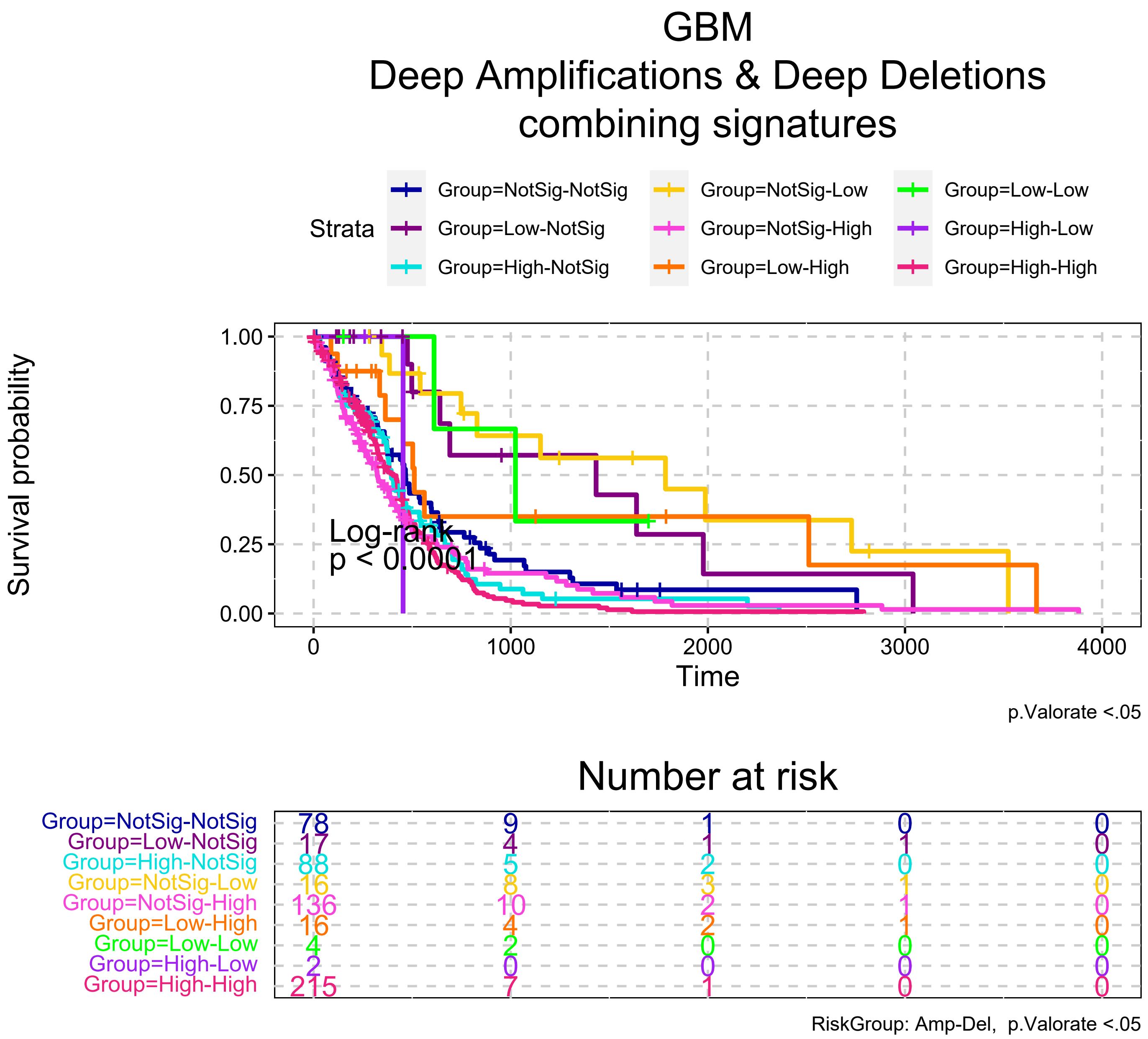

explanatory	beta	HR	L95	U95	p
Low-NotSig	-0.92	0.40	0.19	0.84	0.02
High-NotSig	0.26	1.30	0.91	1.86	0.15
NotSig-Low	-1.12	0.33	0.16	0.64	0.00
NotSig-High	0.32	1.37	0.99	1.90	0.06
Low-High	-0.60	0.55	0.28	1.08	0.08
Low-Low	-1.00	0.37	0.09	1.52	0.17
High-Low	0.07	1.07	0.15	7.74	0.95
High-High	0.40	1.50	1.10	2.03	0.01

n= 572, number of events =430
Score(logrank) test = p <.0001
