# Supplementary material for: Systematic Modeling of Risk-Associated Copy Number Alterations in Cancer
Source: Int J Mol Sci. 2024 Sep 27;25(19):10455. doi: 10.3390/ijms251910455 (PMC11477427; doi:10.3390/ijms251910455)
Supplement: Supplementary file 1 [file ijms-25-10455-s001.zip › HNSCSignatureV12-sinSombreado.pdf]

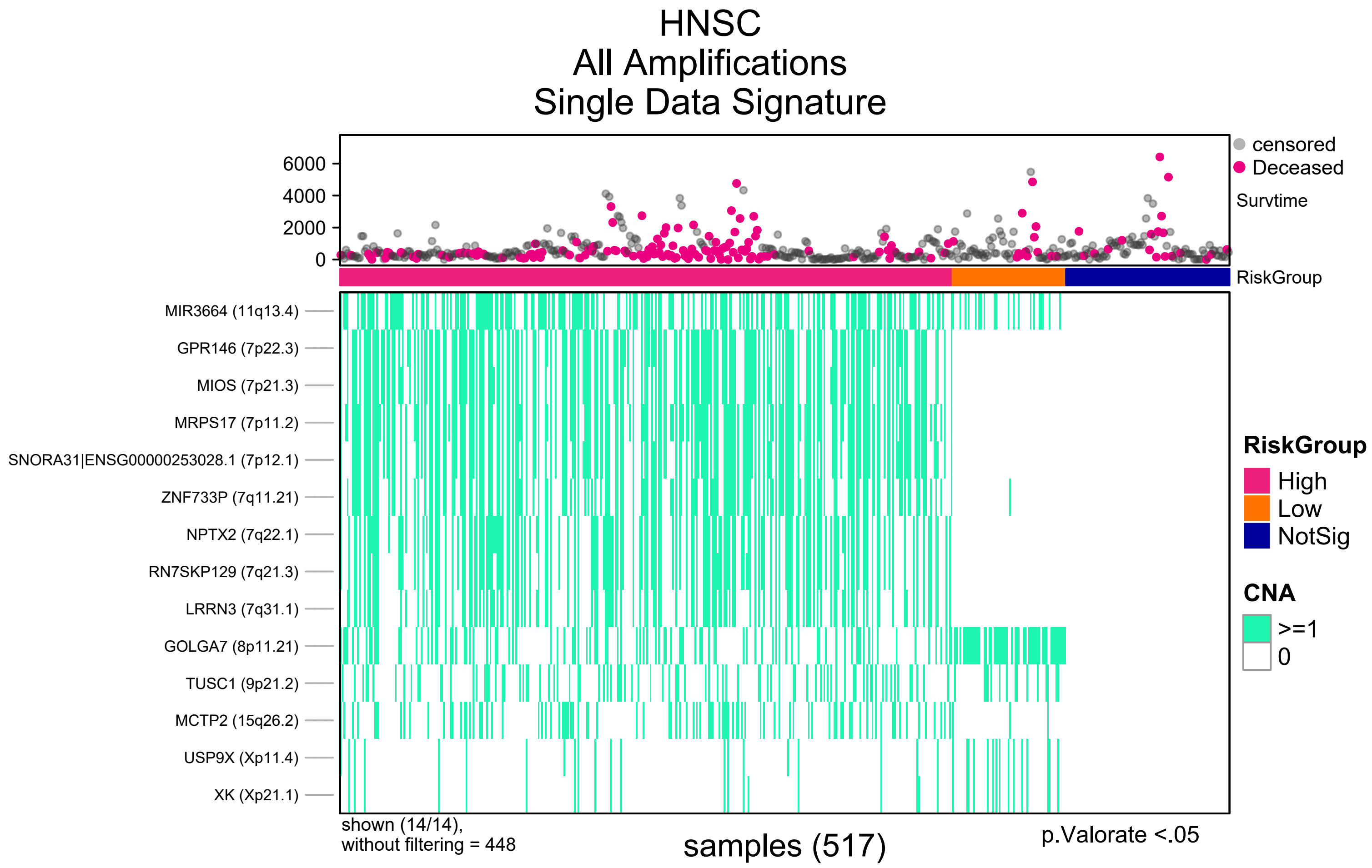
HNSC
All Amplifications
Single Data Signature
6000
4000
2000
0
censored
Deceased
Survtime
RiskGroup
MIR3664 (11q13.4)
GPR146 (7p22.3)
MIOS (7p21.3)
MRPS17 (7p11.2)
SNORA31|ENSG00000253028.1 (7p12.1)
ZNF733P (7q11.21)
NPTX2 (7q22.1)
RN7SKP129 (7q21.3)
LRRN3 (7q31.1)
GOLGA7 (8p11.21)
TUSC1 (9p21.2)
MCTP2 (15q26.2)
USP9X (Xp11.4)
XK (Xp21.1)
RiskGroup
High
Low
NotSig
CNA
>=1
0
shown (14/14),
without filtering = 448
samples (517)
p.Valorate <.05

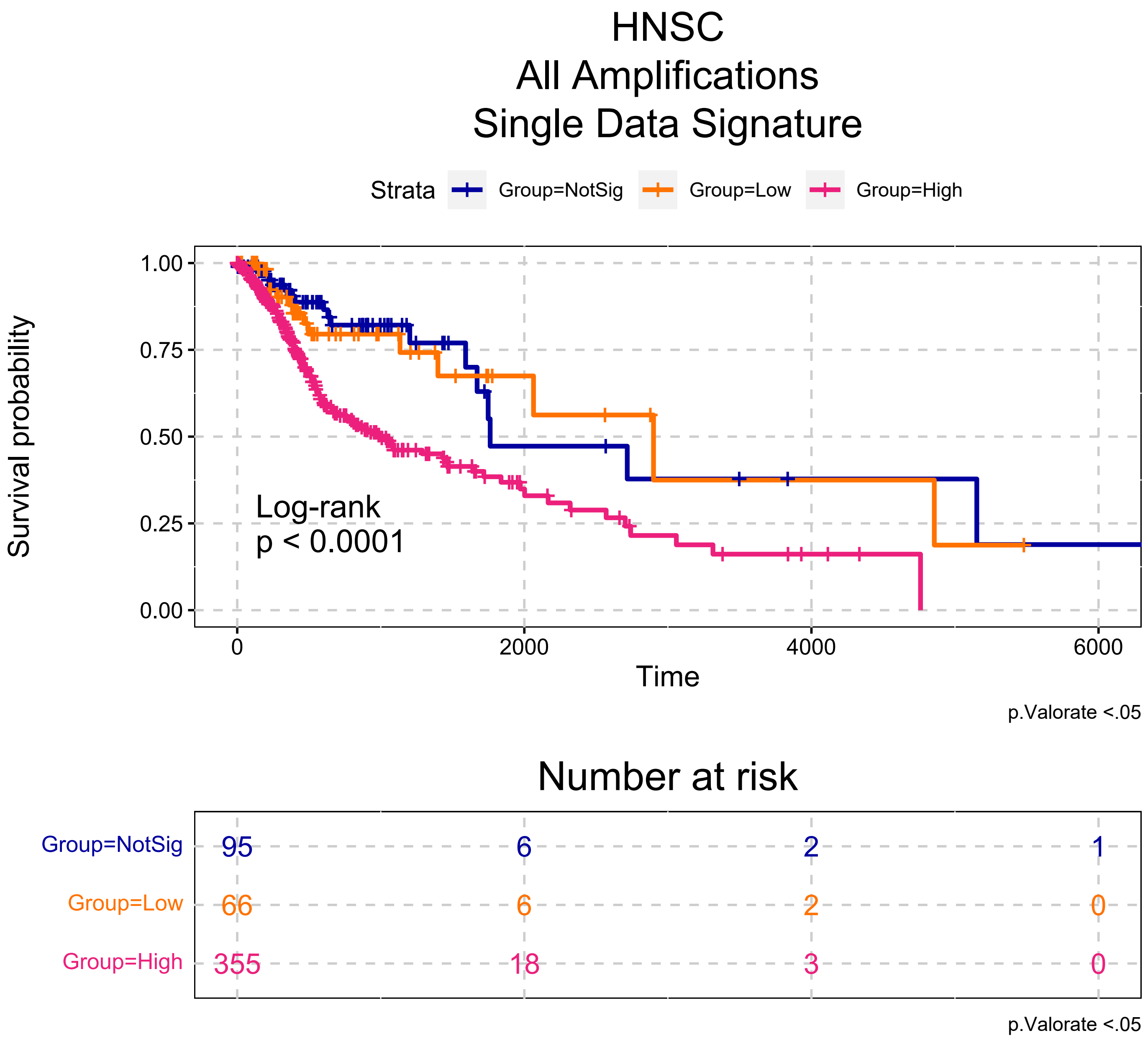

explanatory	beta	HR	L95	U95	p
Low	0.13	1.14	0.56	2.29	0.72
High	0.98	2.66	1.61	4.39	0.00

n= 517, number of events =167
Score(logrank) test = p <.0001

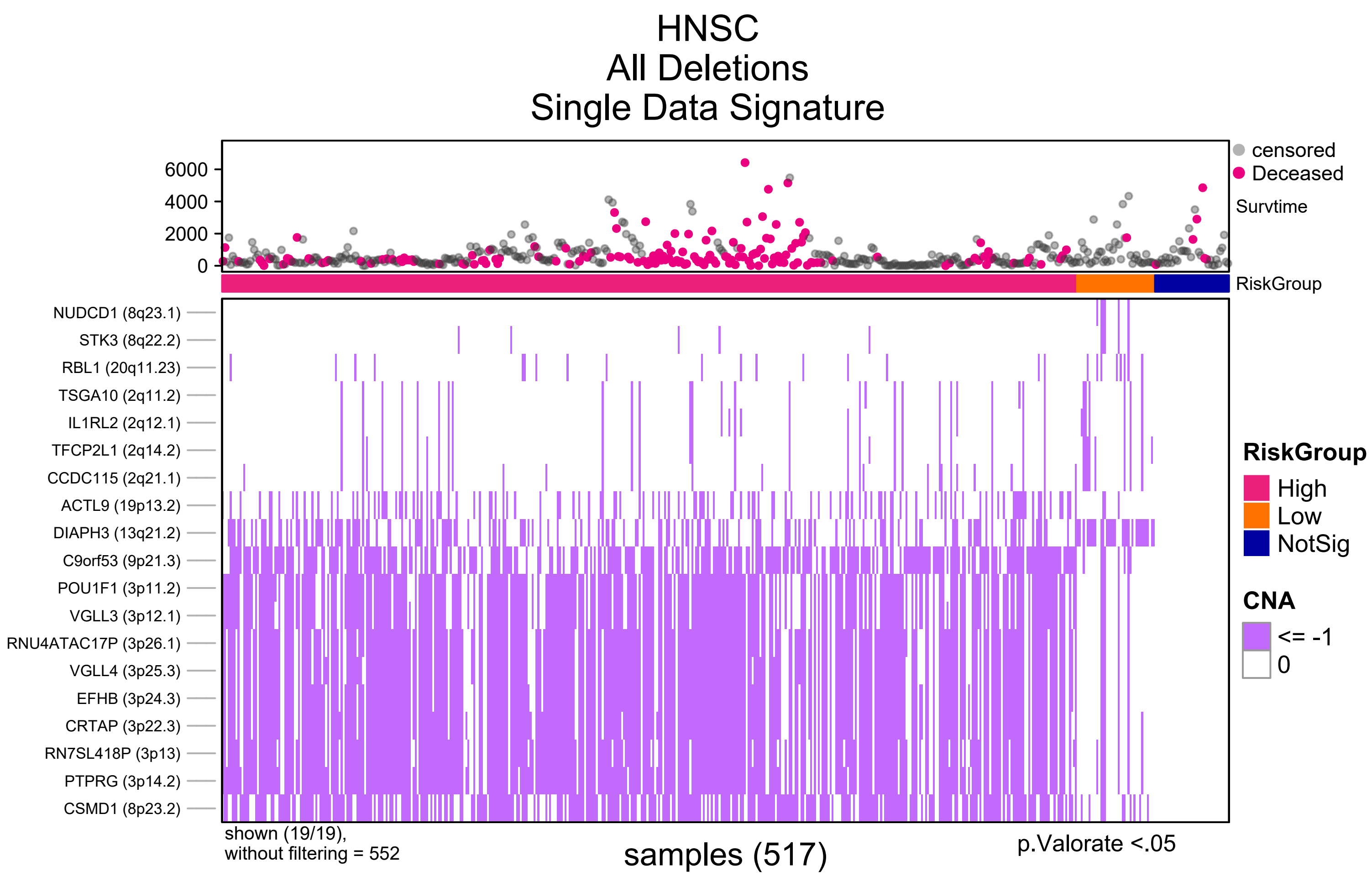
HNSC
All Deletions
Single Data Signature
6000
4000
2000
0
censored
Deceased
Survtime
RiskGroup
NUDCD1 (8q23.1)
STK3 (8q22.2)
RBL1 (20q11.23)
TSGA10 (2q11.2)
IL1RL2 (2q12.1)
TFCP2L1 (2q14.2)
CCDC115 (2q21.1)
ACTL9 (19p13.2)
DIAPH3 (13q21.2)
C9orf53 (9p21.3)
POU1F1 (3p11.2)
VGLL3 (3p12.1)
RNU4ATAC17P (3p26.1)
VGLL4 (3p25.3)
EFHB (3p24.3)
CRTAP (3p22.3)
RN7SL418P (3p13)
PTPRG (3p14.2)
CSMD1 (8p23.2)
RiskGroup
High
Low
NotSig
CNA
<= -1
0
shown (19/19),
without filtering = 552
samples (517)
p.Valorate <.05

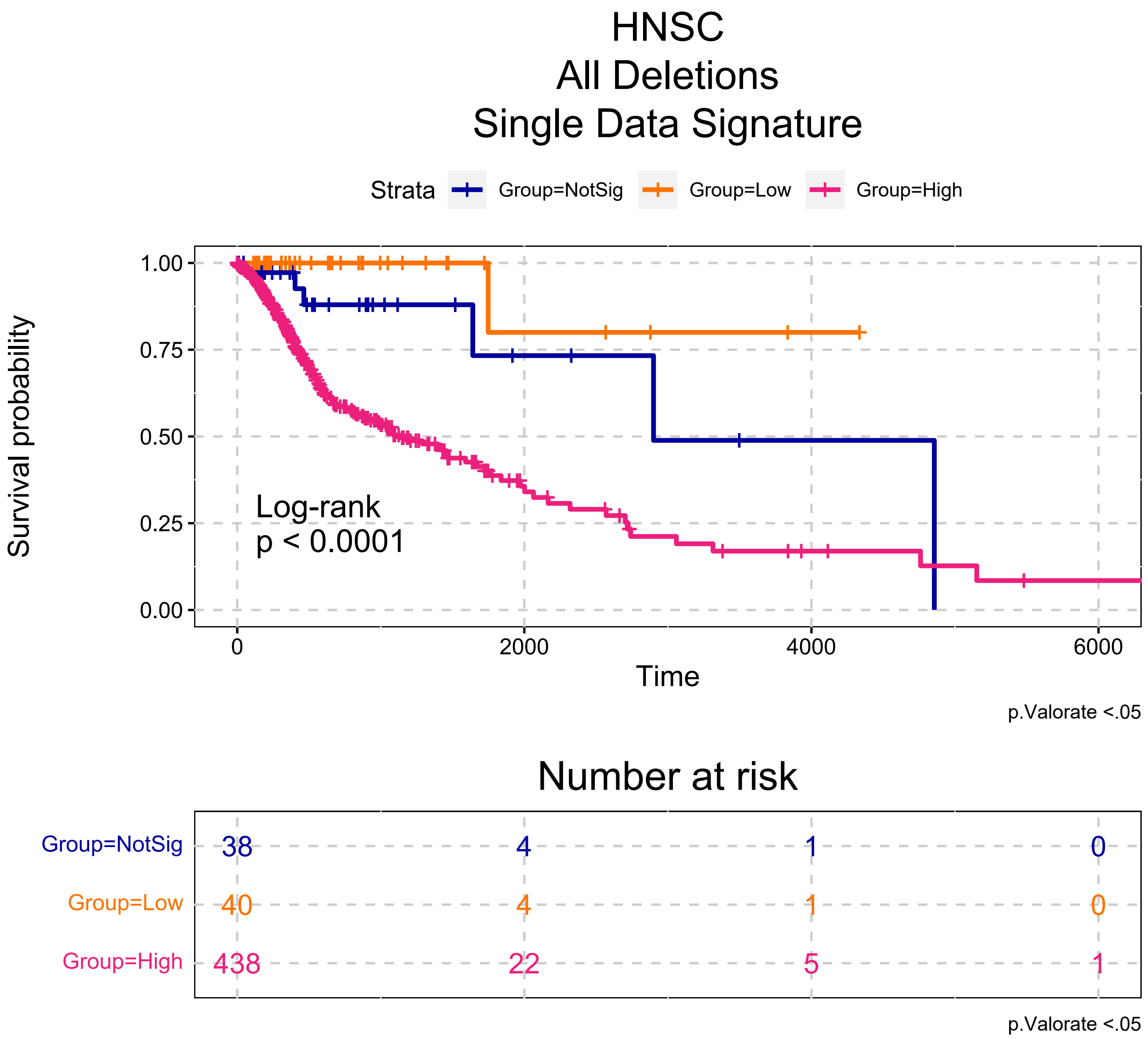

explanatory	beta	HR	L95	U95	p
Low	-1.84	0.16	0.02	1.32	0.09
High	1.12	3.07	1.36	6.96	0.01

n= 517, number of events =167
Score(logrank) test = p <.0001

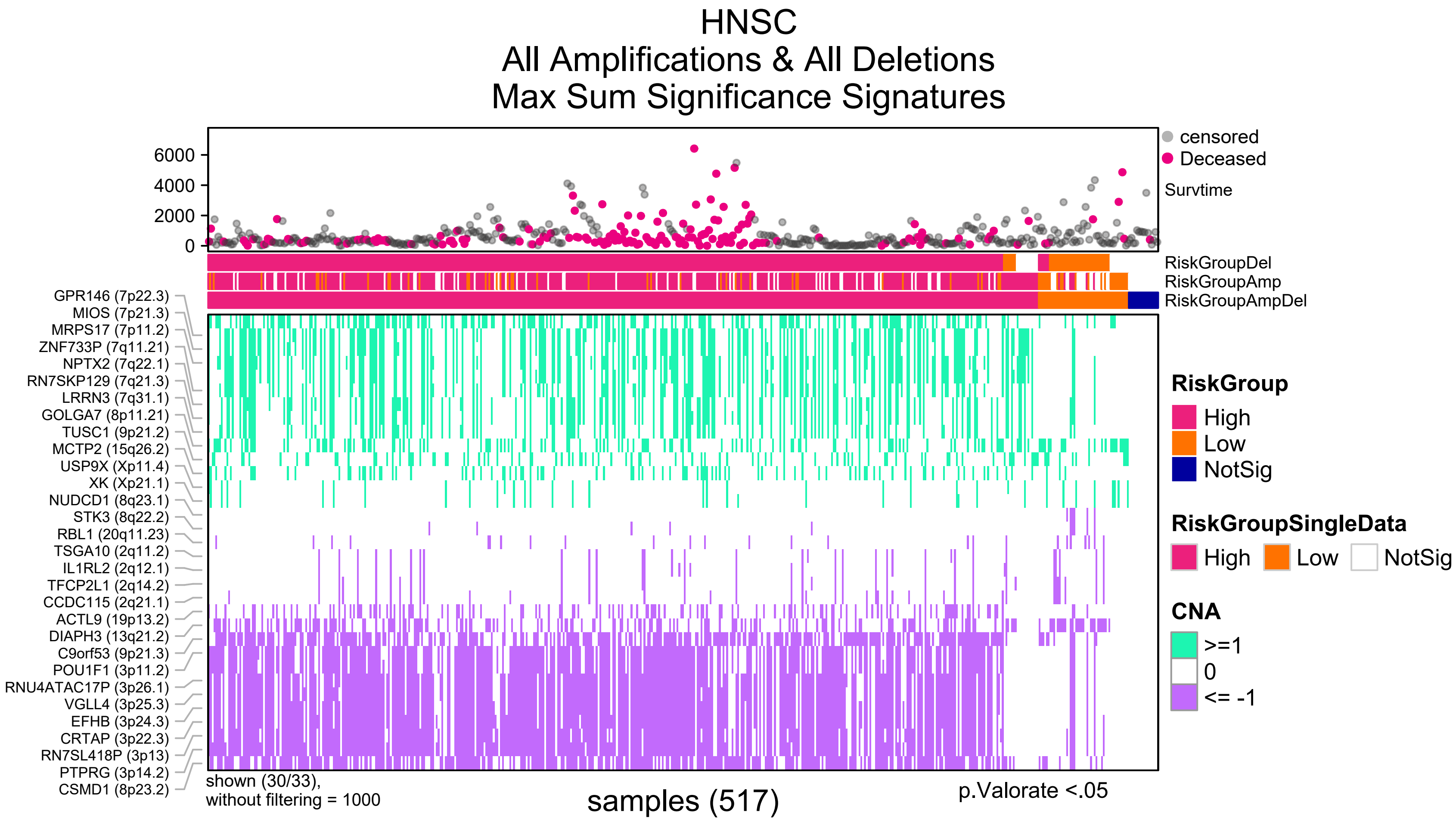
HNSC
All Amplifications & All Deletions
Max Sum Significance Signatures
6000
4000
2000
0
censored
Deceased
Survtime
RiskGroupDel
RiskGroupAmp
RiskGroupAmpDel
GPR146 (7p22.3)
MIOS (7p21.3)
MRPS17 (7p11.2)
ZNF733P (7q11.21)
NPTX2 (7q22.1)
RN7SKP129 (7q21.3)
LRRN3 (7q31.1)
GOLGA7 (8p11.21)
TUSC1 (9p21.2)
MCTP2 (15q26.2)
USP9X (Xp11.4)
XK (Xp21.1)
NUDCD1 (8q23.1)
STK3 (8q22.2)
RBL1 (20q11.23)
TSGA10 (2q11.2)
IL1RL2 (2q12.1)
TFCP2L1 (2q14.2)
CCDC115 (2q21.1)
ACTL9 (19p13.2)
DIAPH3 (13q21.2)
C9orf53 (9p21.3)
POU1F1 (3p11.2)
RNU4ATAC17P (3p26.1)
VGLL4 (3p25.3)
EFHB (3p24.3)
CRTAP (3p22.3)
RN7SL418P (3p13)
PTPRG (3p14.2)
CSMD1 (8p23.2)
RiskGroup
High
Low
NotSig
RiskGroupSingleData
High
Low
NotSig
CNA
>=1
0
<= -1
shown (30/33),
without filtering = 1000
samples (517)
p.Valorate <.05

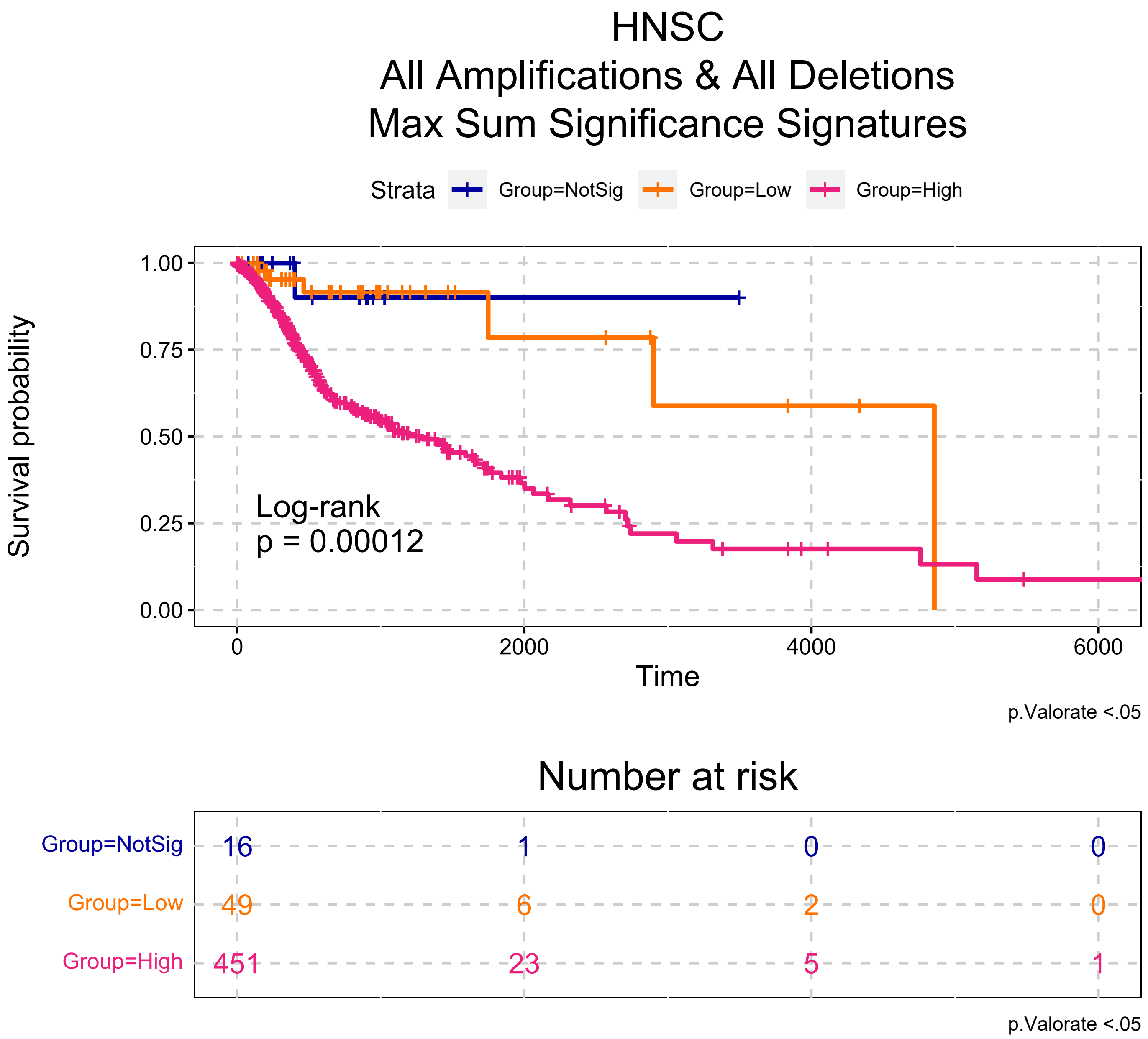

explanatory	beta	HR	L95	U95	p
Low	0.57	1.76	0.21	14.66	0.60
High	1.96	7.12	1.00	50.88	0.05

n= 517, number of events =167
Score(logrank) test = 0

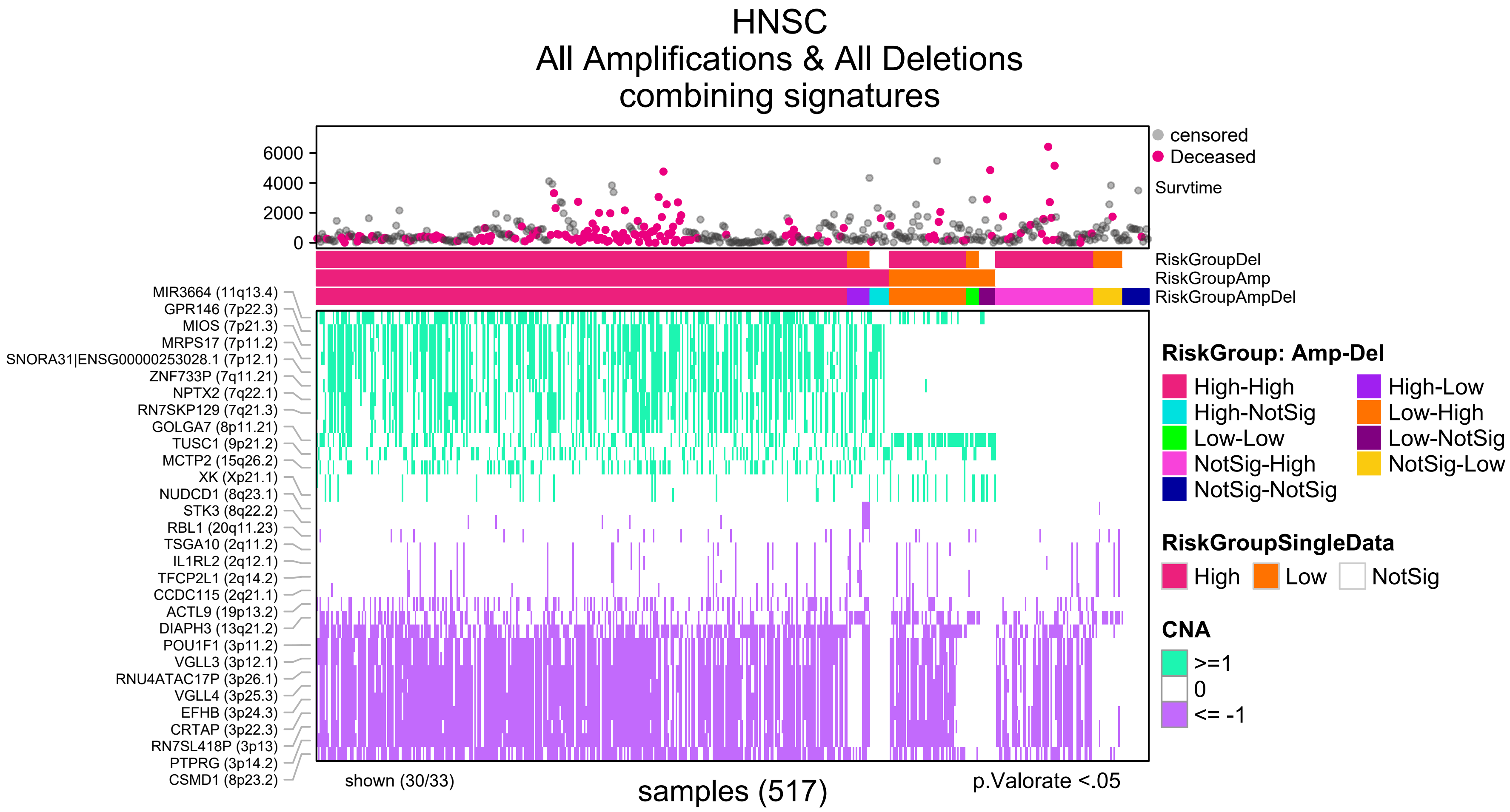
HNSC
All Amplifications & All Deletions
combining signatures
6000
4000
2000
0
censored
Deceased
Survtime
RiskGroupDel
RiskGroupAmp
RiskGroupAmpDel
MIR3664 (11q13.4)
GPR146 (7p22.3)
MIOS (7p21.3)
MRPS17 (7p11.2)
SNORA31|ENSG00000253028.1 (7p12.1)
ZNF733P (7q11.21)
NPTX2 (7q22.1)
RN7SKP129 (7q21.3)
GOLGA7 (8p11.21)
TUSC1 (9p21.2)
MCTP2 (15q26.2)
XK (Xp21.1)
NUDCD1 (8q23.1)
STK3 (8q22.2)
RBL1 (20q11.23)
TSGA10 (2q11.2)
IL1RL2 (2q12.1)
TFCP2L1 (2q14.2)
CCDC115 (2q21.1)
ACTL9 (19p13.2)
DIAPH3 (13q21.2)
POU1F1 (3p11.2)
VGLL3 (3p12.1)
RNU4ATAC17P (3p26.1)
VGLL4 (3p25.3)
EFHB (3p24.3)
CRTAP (3p22.3)
RN7SL418P (3p13)
PTPRG (3p14.2)
CSMD1 (8p23.2)
shown (30/33)
samples (517)
p.Valorate <.05
RiskGroup: Amp-Del
High-High
High-NotSig
Low-Low
NotSig-High
NotSig-NotSig
High-Low
Low-High
Low-NotSig
NotSig-Low
RiskGroupSingleData
High
Low
NotSig
CNA
>=1
0
<= -1

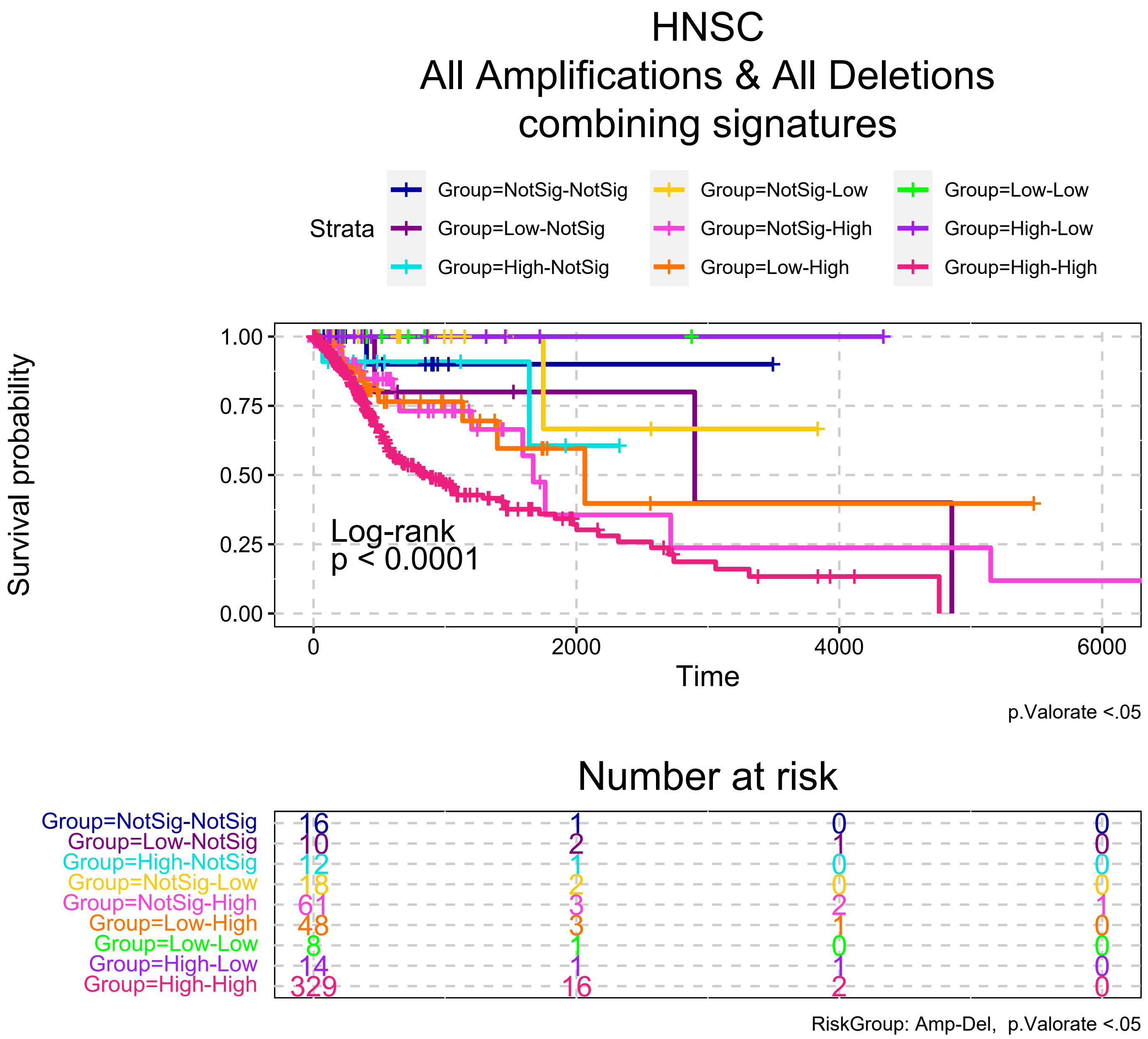

explanatory	beta	HR	L95	U95	p
Low-NotSig	1.26	3.53	0.36	34.24	0.28
High-NotSig	1.06	2.88	0.26	31.82	0.39
NotSig-Low	-0.28	0.75	0.05	12.06	0.84
NotSig-High	1.48	4.39	0.58	33.22	0.15
Low-High	1.39	4.00	0.51	31.05	0.19
Low-Low	-15.30	0.00	0.00	Inf	1.00
High-Low	-15.31	0.00	0.00	Inf	0.99
High-High	2.14	8.54	1.19	61.09	0.03

n= 517, number of events =167
Score(logrank) test = p <.0001

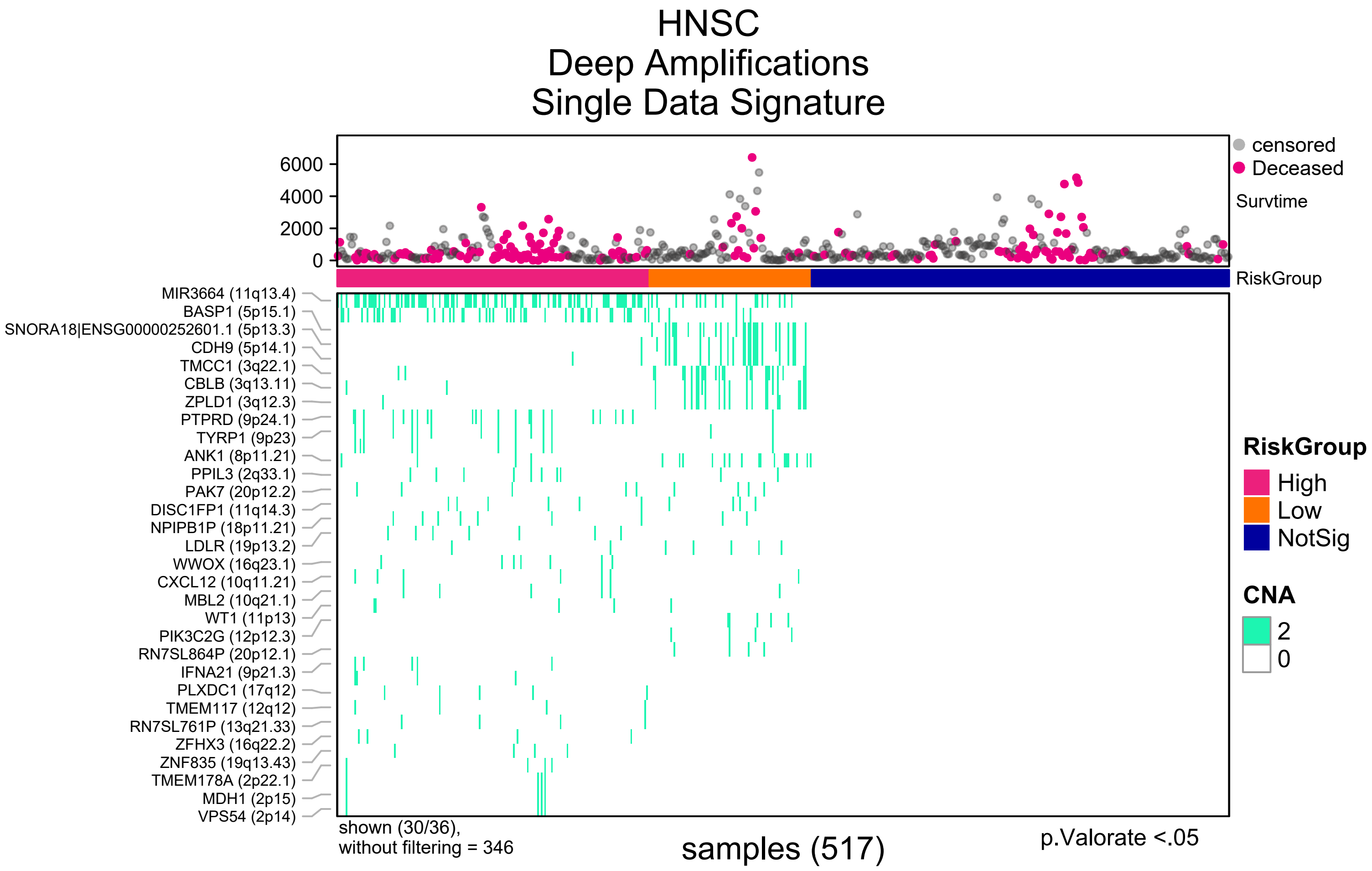
HNSC
Deep Amplifications
Single Data Signature
6000
4000
2000
0
censored
Deceased
Survtime
RiskGroup
MIR3664 (11q13.4)
BASP1 (5p15.1)
SNORA18|ENSG00000252601.1 (5p13.3)
CDH9 (5p14.1)
TMCC1 (3q22.1)
CBLB (3q13.11)
ZPLD1 (3q12.3)
PTPRD (9p24.1)
TYRP1 (9p23)
ANK1 (8p11.21)
PPIL3 (2q33.1)
PAK7 (20p12.2)
DISC1FP1 (11q14.3)
NPIPB1P (18p11.21)
LDLR (19p13.2)
WWOX (16q23.1)
CXCL12 (10q11.21)
MBL2 (10q21.1)
WT1 (11p13)
PIK3C2G (12p12.3)
RN7SL864P (20p12.1)
IFNA21 (9p21.3)
PLXDC1 (17q12)
TMEM117 (12q12)
RN7SL761P (13q21.33)
ZFHX3 (16q22.2)
ZNF835 (19q13.43)
TMEM178A (2p22.1)
MDH1 (2p15)
VPS54 (2p14)
RiskGroup
High
Low
NotSig
CNA
2
0
shown (30/36),
without filtering = 346
samples (517)
p.Valorate <.05

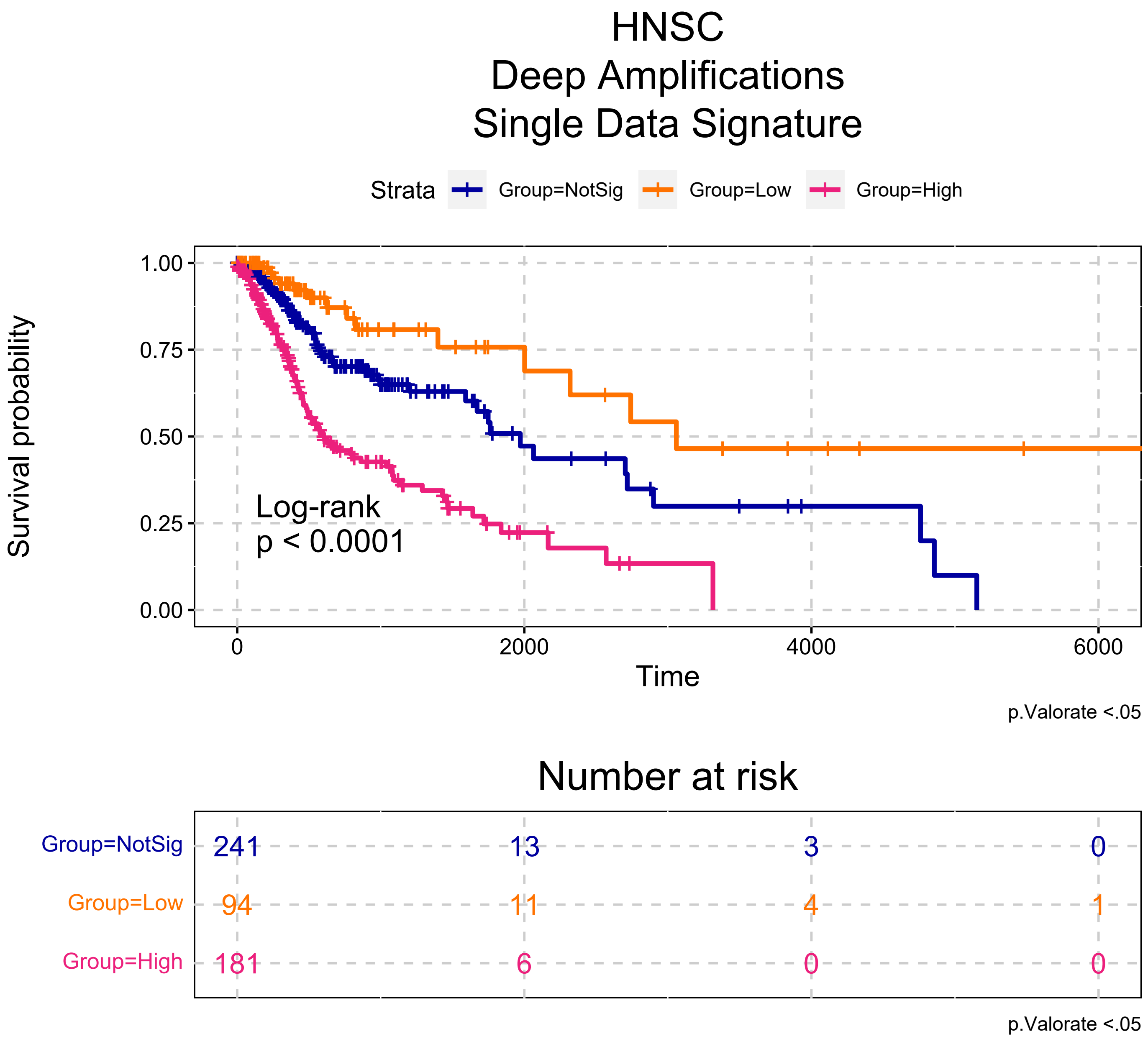

explanatory	beta	HR	L95	U95	p
Low	-0.77	0.46	0.26	0.83	0.01
High	0.84	2.31	1.66	3.20	0.00

n= 517, number of events =167
Score(logrank) test = p <.0001

HNSC
Deep Deletions
Single Data Signature

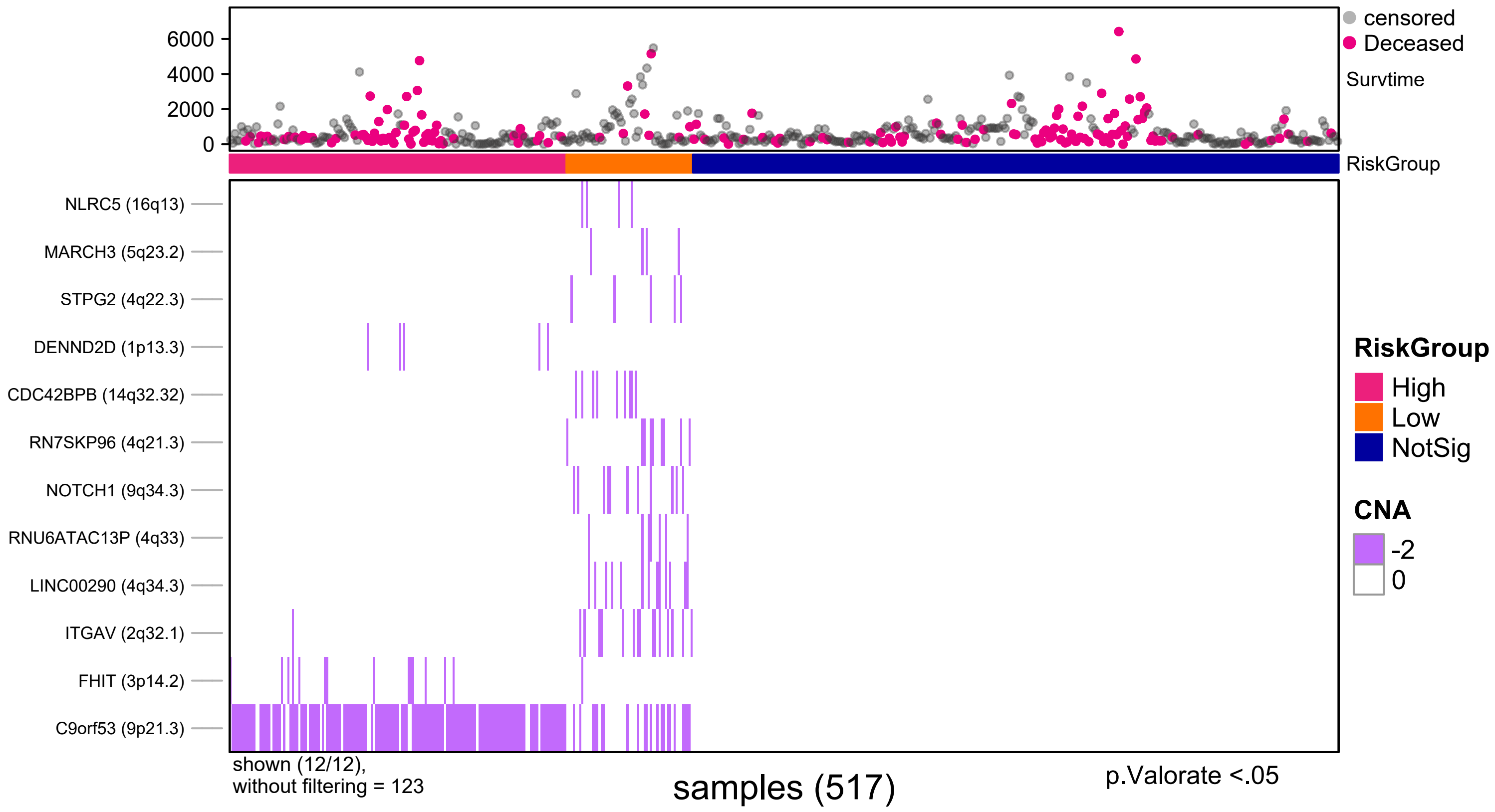

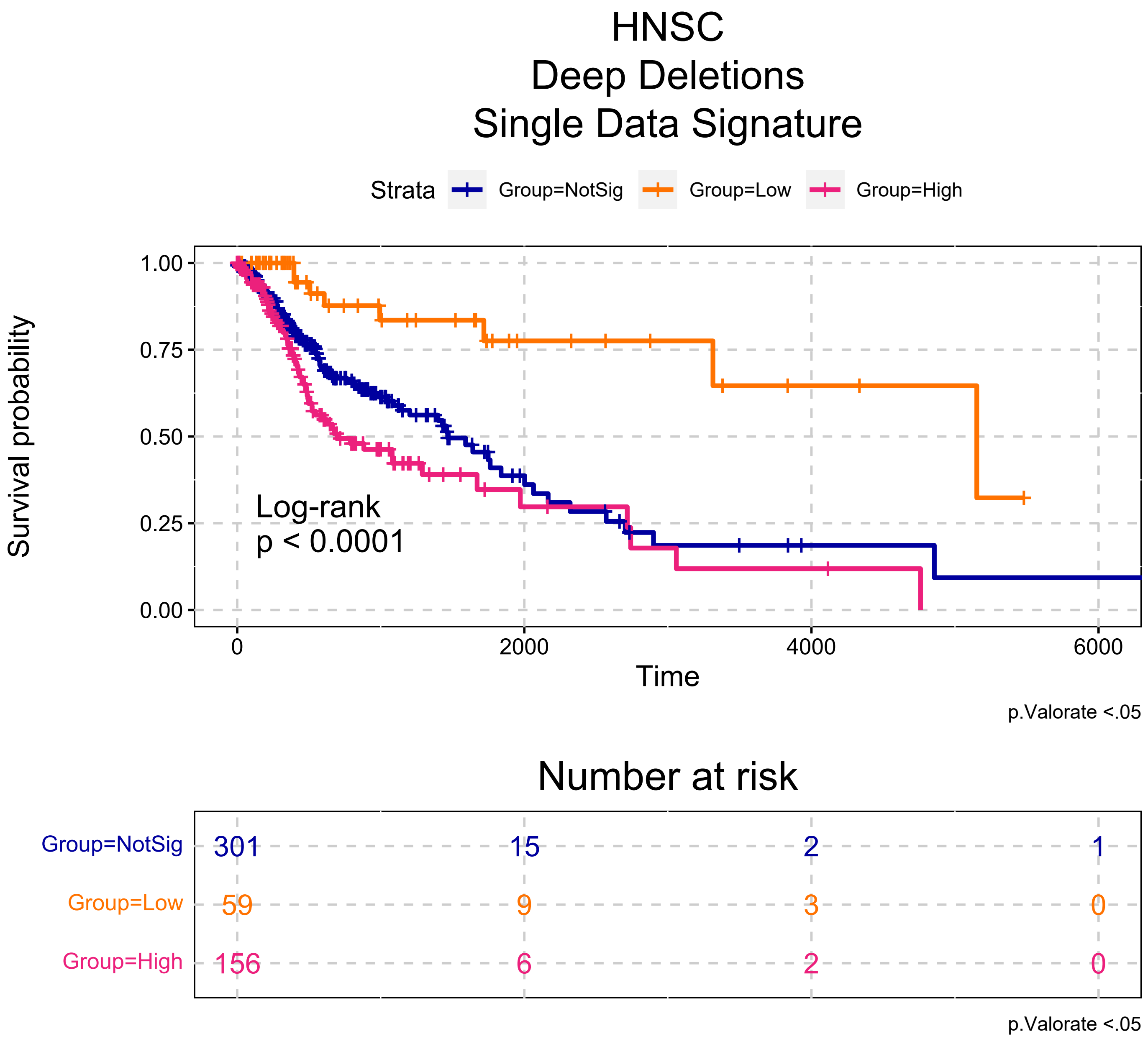

explanatory	beta	HR	L95	U95	p
Low	-1.35	0.26	0.12	0.54	0.00
High	0.39	1.47	1.07	2.03	0.02

n= 517, number of events =167
Score(logrank) test = p <.0001

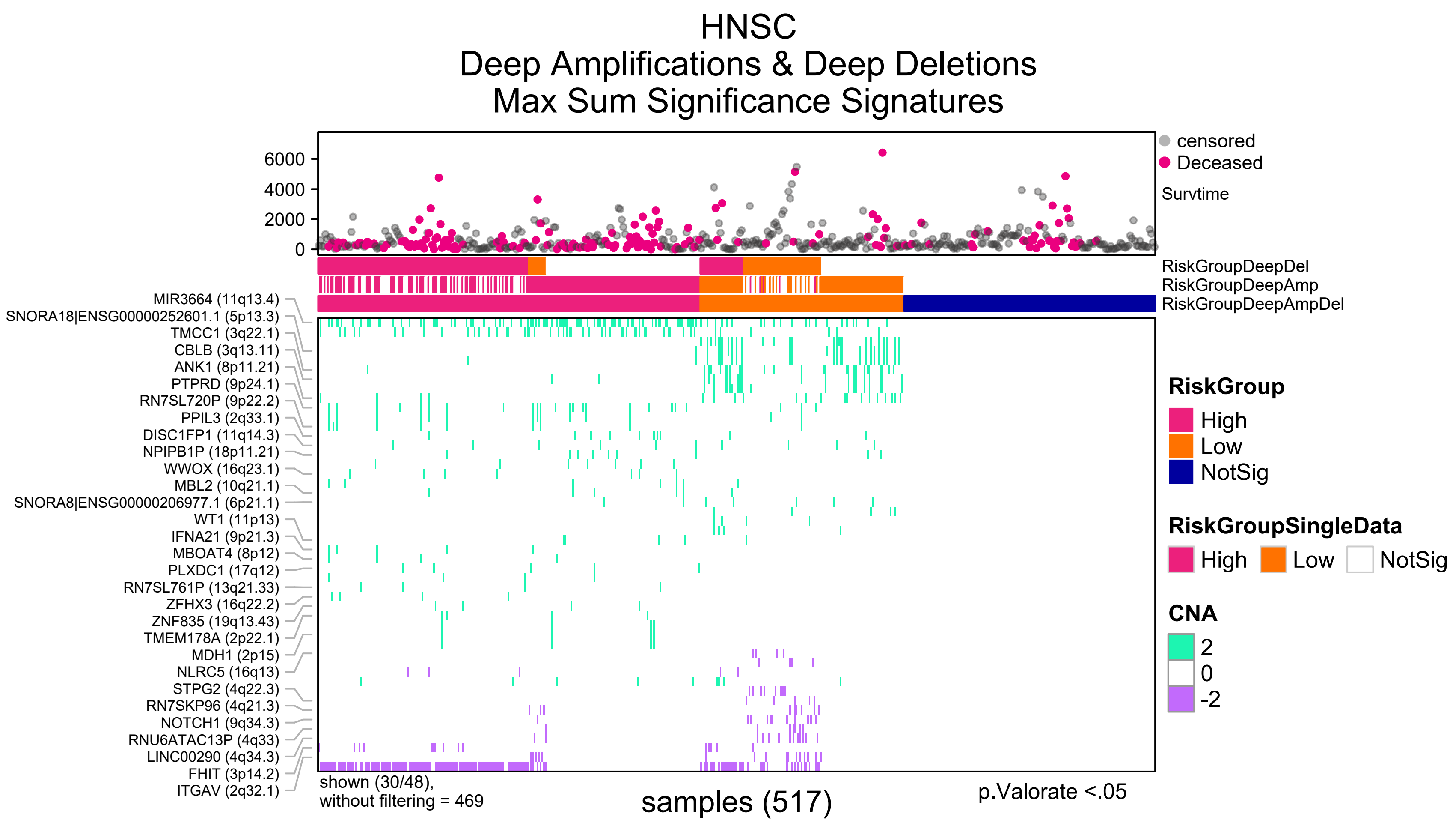

HNSC
Deep Amplifications & Deep Deletions
Max Sum Significance Signatures
6000
4000
2000
0
censored
Deceased
Survtime
RiskGroupDeepDel
RiskGroupDeepAmp
RiskGroupDeepAmpDel
MIR3664 (11q13.4)
SNORA18|ENSG00000252601.1 (5p13.3)
TMCC1 (3q22.1)
CBLB (3q13.11)
ANK1 (8p11.21)
PTPRD (9p24.1)
RN7SL720P (9p22.2)
PPIL3 (2q33.1)
DISC1FP1 (11q14.3)
NPIPB1P (18p11.21)
WWOX (16q23.1)
MBL2 (10q21.1)
SNORA8|ENSG00000206977.1 (6p21.1)
WT1 (11p13)
IFNA21 (9p21.3)
MBOAT4 (8p12)
PLXDC1 (17q12)
RN7SL761P (13q21.33)
ZFHX3 (16q22.2)
ZNF835 (19q13.43)
TMEM178A (2p22.1)
MDH1 (2p15)
NLRC5 (16q13)
STPG2 (4q22.3)
RN7SKP96 (4q21.3)
NOTCH1 (9q34.3)
RNU6ATAC13P (4q33)
LINC00290 (4q34.3)
FHIT (3p14.2)
ITGAV (2q32.1)
RiskGroup
High
Low
NotSig
RiskGroupSingleData
High
Low
NotSig
CNA
2
0
-2
shown (30/48),
without filtering = 469
samples (517)
p.Valorate <.05

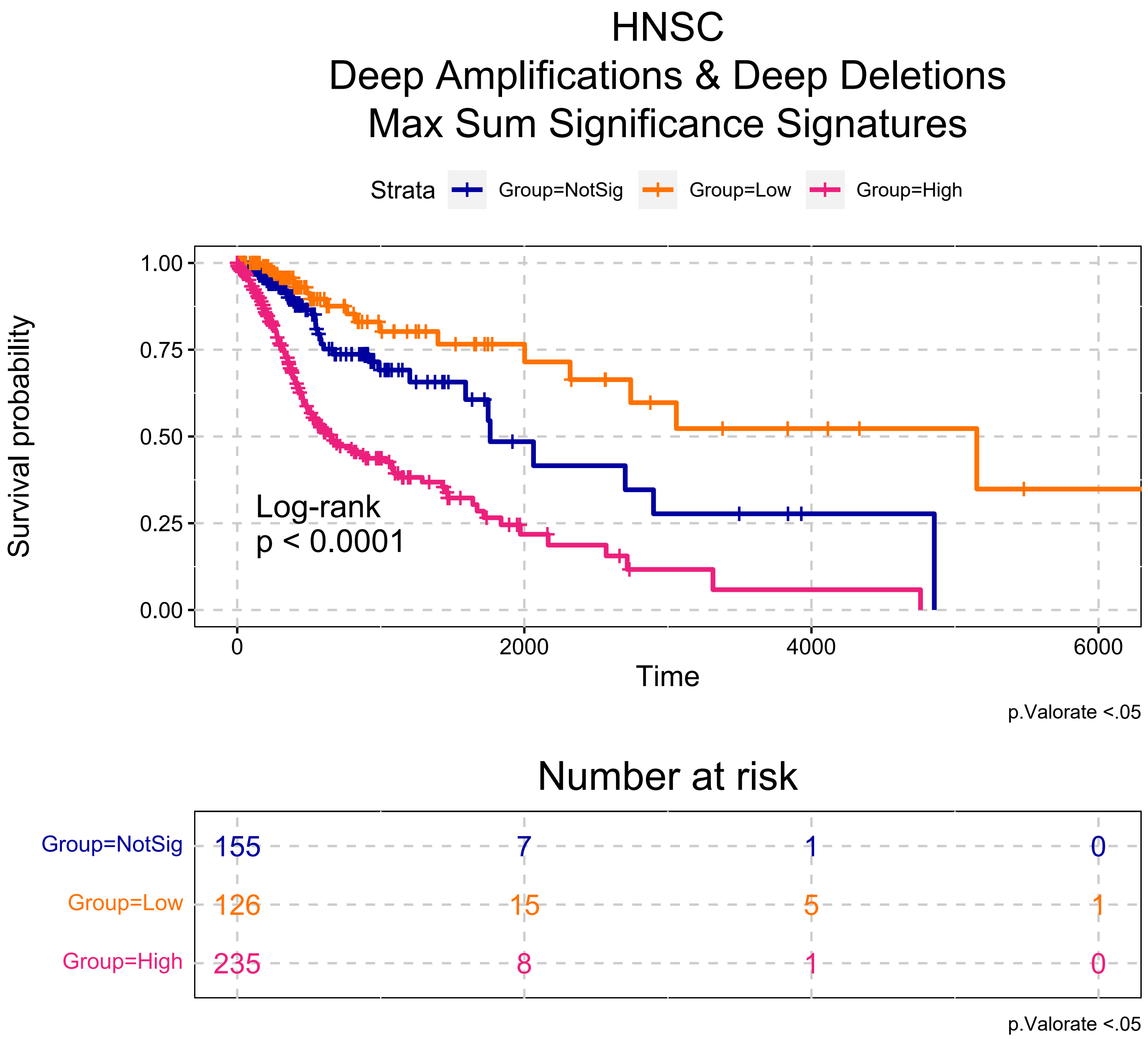

explanatory	beta	HR	L95	U95	p
Low	-0.72	0.48	0.27	0.87	0.02
High	0.94	2.56	1.74	3.76	0.00

n= 517, number of events =167
Score(logrank) test = p <.0001

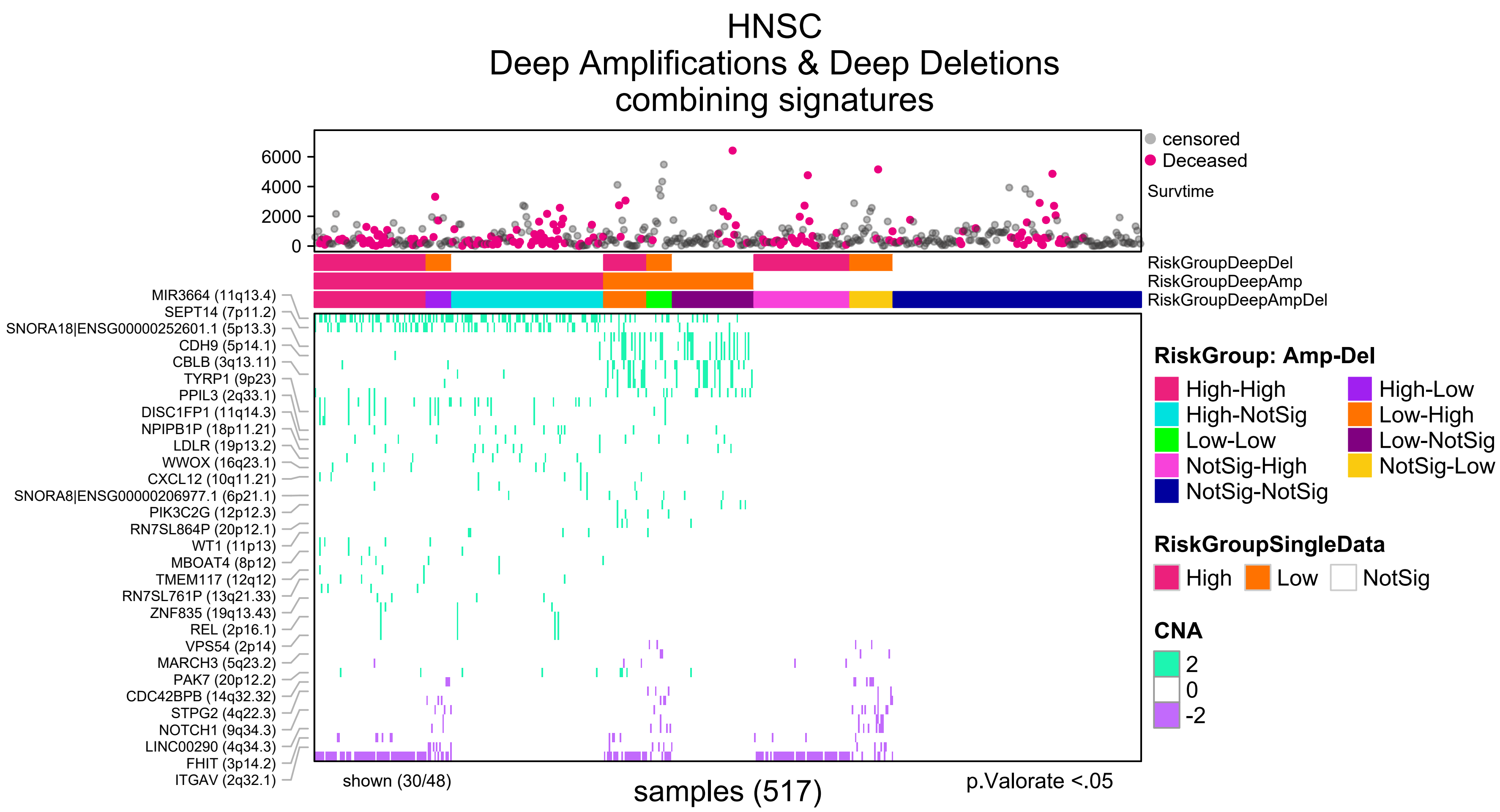

HNSC
Deep Amplifications & Deep Deletions
combining signatures
6000
4000
2000
0
censored
Deceased
Survtime
RiskGroupDeepDel
RiskGroupDeepAmp
RiskGroupDeepAmpDel
MIR3664 (11q13.4)
SEPT14 (7p11.2)
SNORA18|ENSG00000252601.1 (5p13.3)
CDH9 (5p14.1)
CBLB (3q13.11)
TYRP1 (9p23)
PPIL3 (2q33.1)
DISC1FP1 (11q14.3)
NPIPB1P (18p11.21)
LDLR (19p13.2)
WWOX (16q23.1)
CXCL12 (10q11.21)
SNORA8|ENSG00000206977.1 (6p21.1)
PIK3C2G (12p12.3)
RN7SL864P (20p12.1)
WT1 (11p13)
MBOAT4 (8p12)
TMEM117 (12q12)
RN7SL761P (13q21.33)
ZNF835 (19q13.43)
REL (2p16.1)
VPS54 (2p14)
MARCH3 (5q23.2)
PAK7 (20p12.2)
CDC42BPB (14q32.32)
STPG2 (4q22.3)
NOTCH1 (9q34.3)
LINC00290 (4q34.3)
FHIT (3p14.2)
ITGAV (2q32.1)
RiskGroup: Amp-Del
High-High
High-NotSig
Low-Low
NotSig-High
NotSig-NotSig
High-Low
Low-High
Low-NotSig
NotSig-Low
RiskGroupSingleData
High
Low
NotSig
CNA
2
0
-2
shown (30/48)
samples (517)
p.Valorate <.05

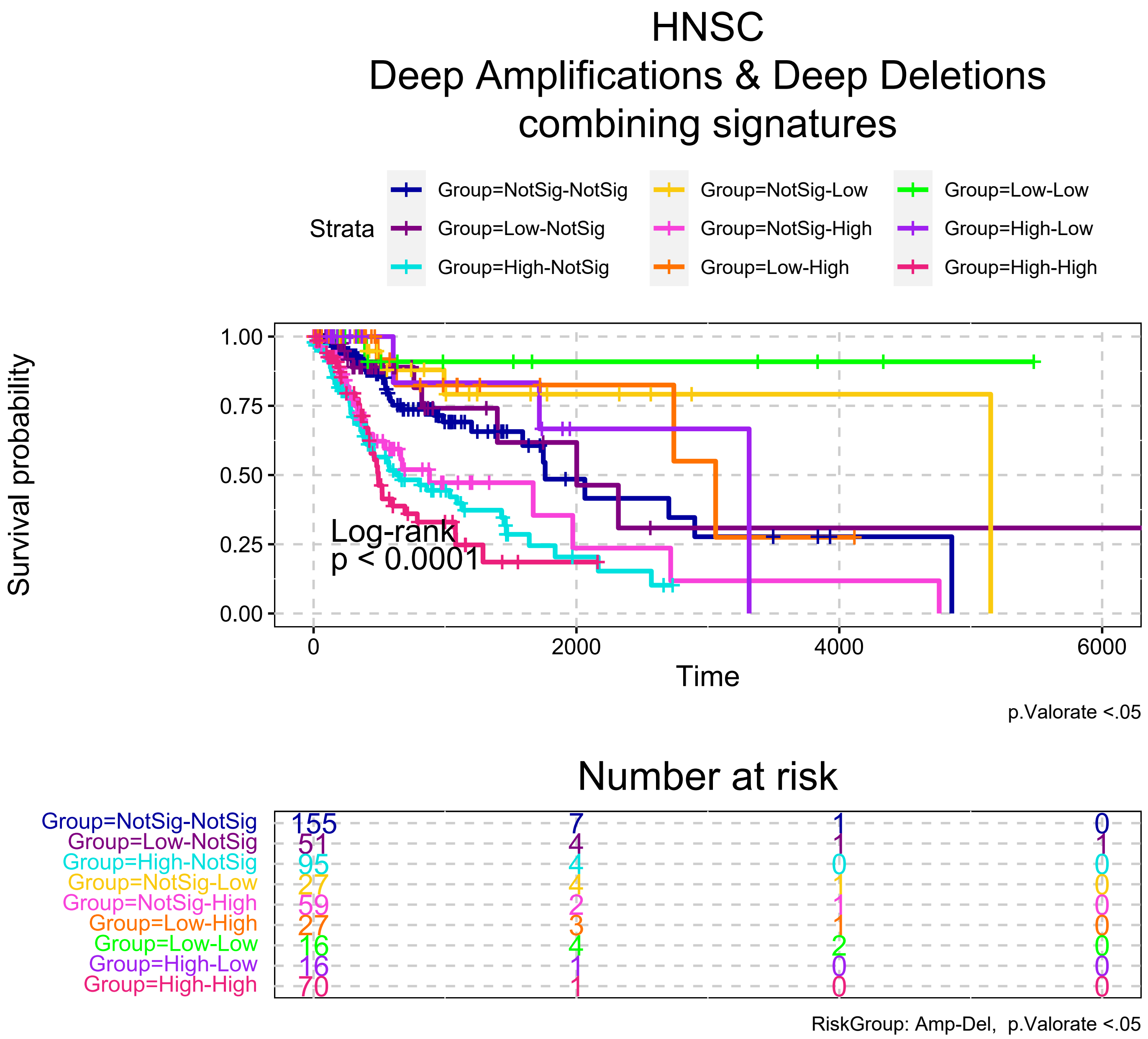

explanatory	beta	HR	L95	U95	p
Low-NotSig	-0.26	0.77	0.36	1.63	0.49
High-NotSig	1.03	2.80	1.80	4.34	0.00
NotSig-Low	-0.92	0.40	0.14	1.15	0.09
NotSig-High	0.79	2.20	1.31	3.69	0.00
Low-High	-0.62	0.54	0.19	1.53	0.24
Low-Low	-2.19	0.11	0.02	0.83	0.03
High-Low	-0.36	0.70	0.21	2.29	0.55
High-High	1.21	3.34	2.07	5.40	0.00

n= 517, number of events =167
Score(logrank) test = p <.0001
