# Supplementary material for: Systematic Modeling of Risk-Associated Copy Number Alterations in Cancer
Source: Int J Mol Sci. 2024 Sep 27;25(19):10455. doi: 10.3390/ijms251910455 (PMC11477427; doi:10.3390/ijms251910455)
Supplement: Supplementary file 1 [file ijms-25-10455-s001.zip › KICHSignatureV12-sinSombreado.pdf]

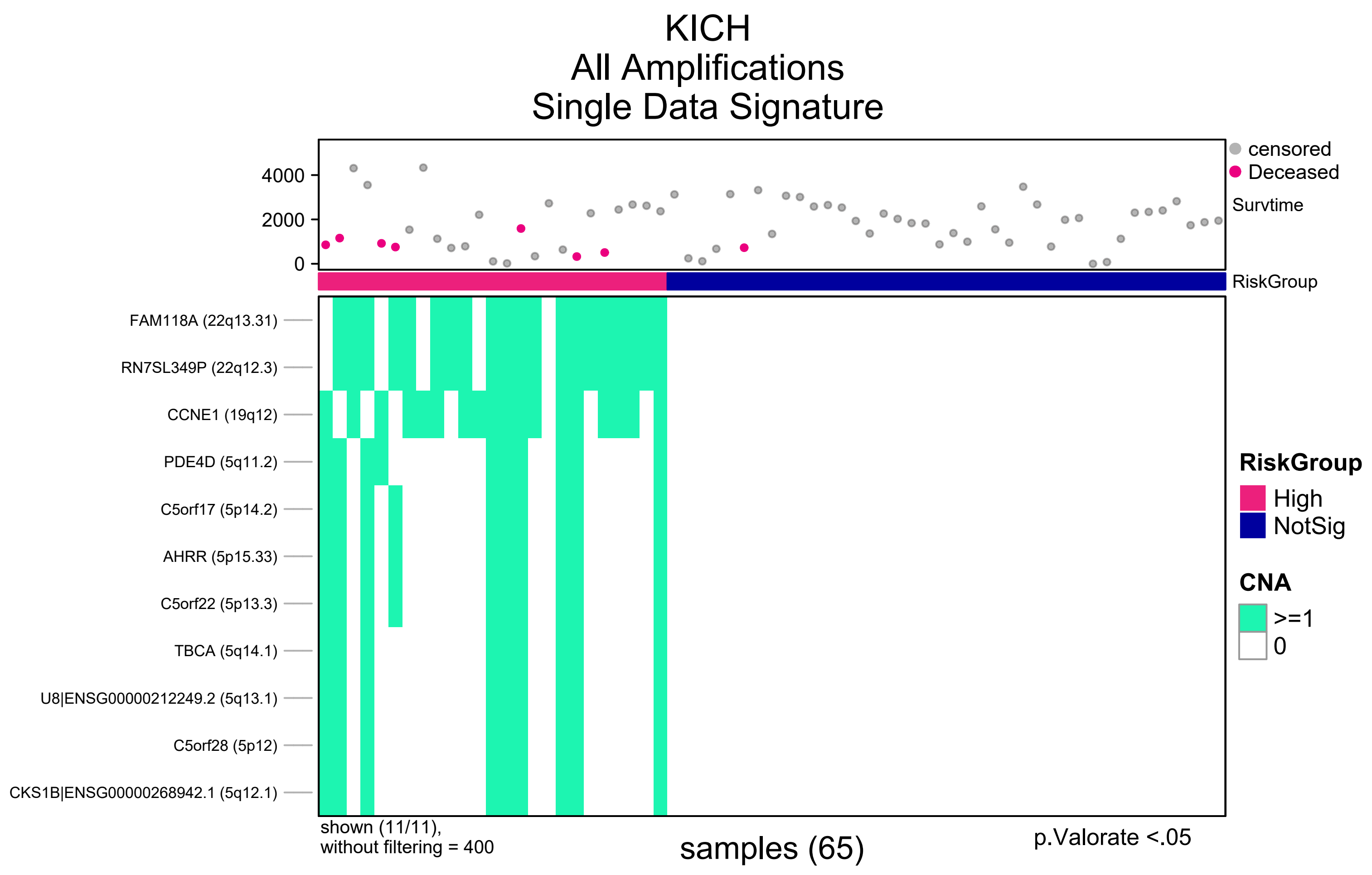
KICH
All Amplifications
Single Data Signature
4000
2000
0
censored
Deceased
Survtime
RiskGroup
FAM118A (22q13.31)
RN7SL349P (22q12.3)
CCNE1 (19q12)
PDE4D (5q11.2)
C5orf17 (5p14.2)
AHRR (5p15.33)
C5orf22 (5p13.3)
TBCA (5q14.1)
U8|ENSG00000212249.2 (5q13.1)
C5orf28 (5p12)
CKS1B|ENSG00000268942.1 (5q12.1)
RiskGroup
High
NotSig
CNA
>=1
0
shown (11/11),
without filtering = 400
samples (65)
p.Valorate <.05

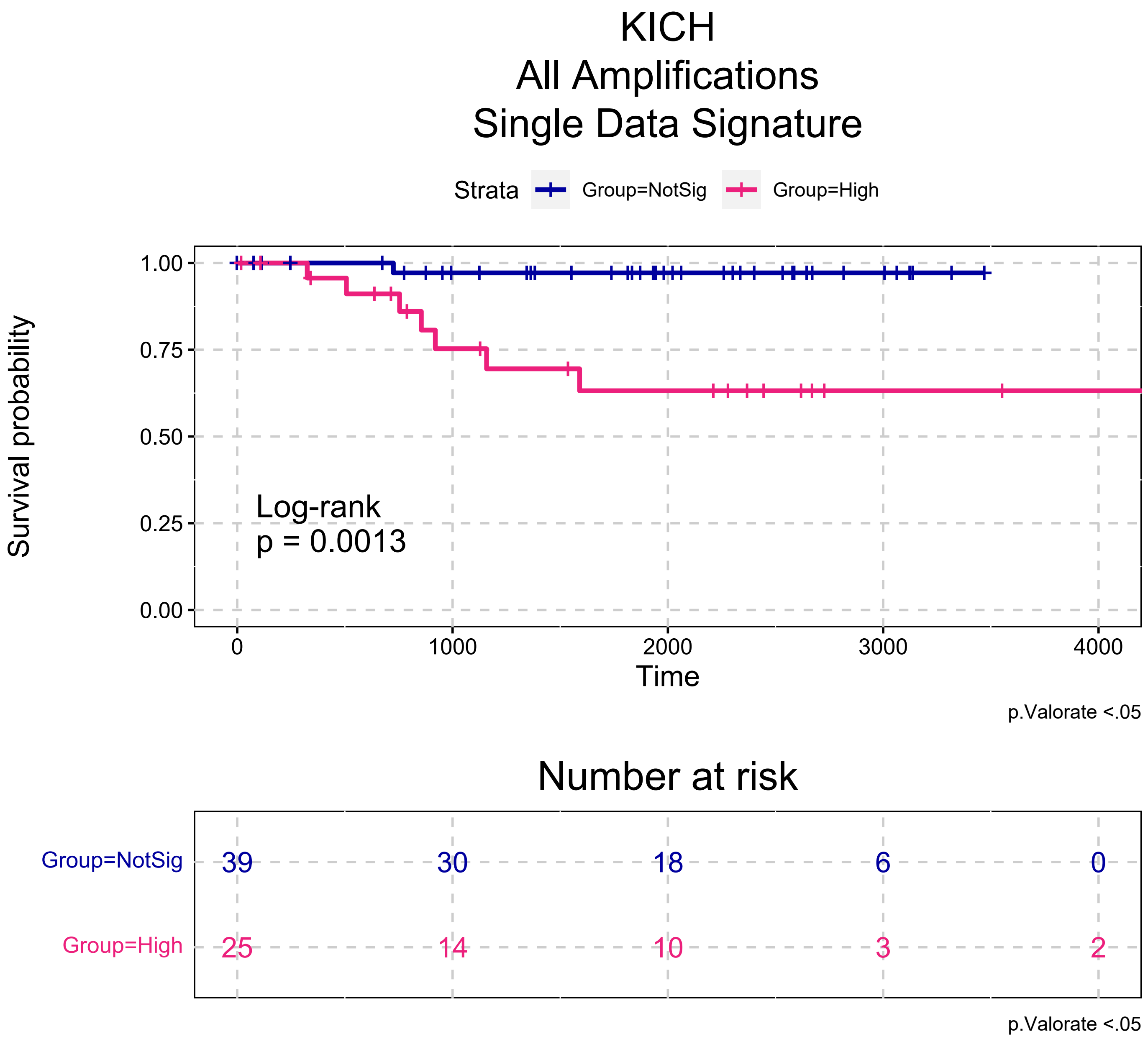

explanatory	beta	HR	L95	U95	p
High	2.63	13.81	1.70	112.44	0.01

n= 65, number of events =8
Score(logrank) test = 0.001

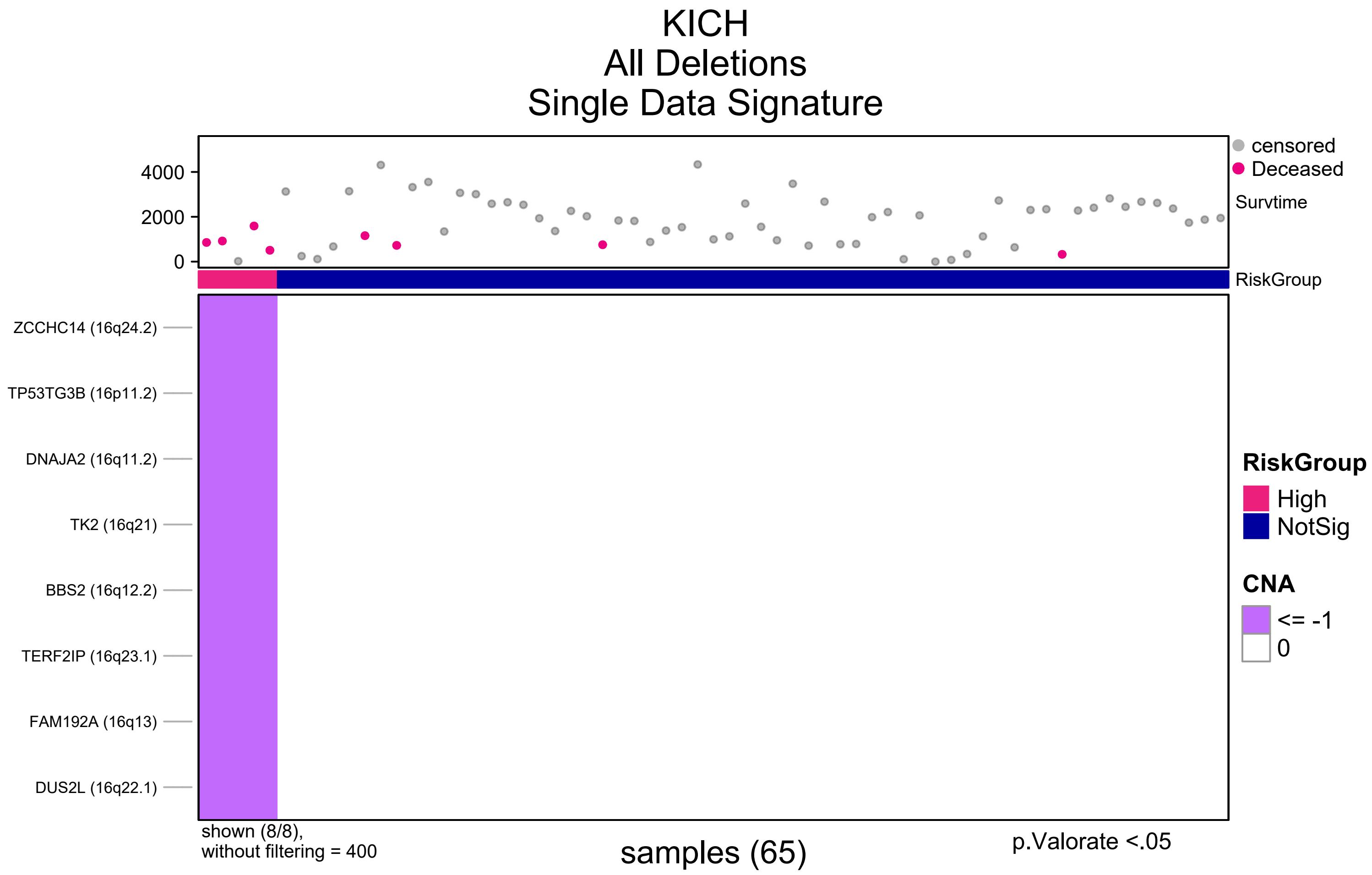
KICH
All Deletions
Single Data Signature
censored
Deceased
Survtime
4000
2000
0
RiskGroup
ZCCHC14 (16q24.2)
TP53TG3B (16p11.2)
DNAJA2 (16q11.2)
TK2 (16q21)
BBS2 (16q12.2)
TERF2IP (16q23.1)
FAM192A (16q13)
DUS2L (16q22.1)
RiskGroup
High
NotSig
CNA
<= -1
0
shown (8/8),
without filtering = 400
samples (65)
p.Valorate <.05

KICH
All Deletions
Single Data Signature

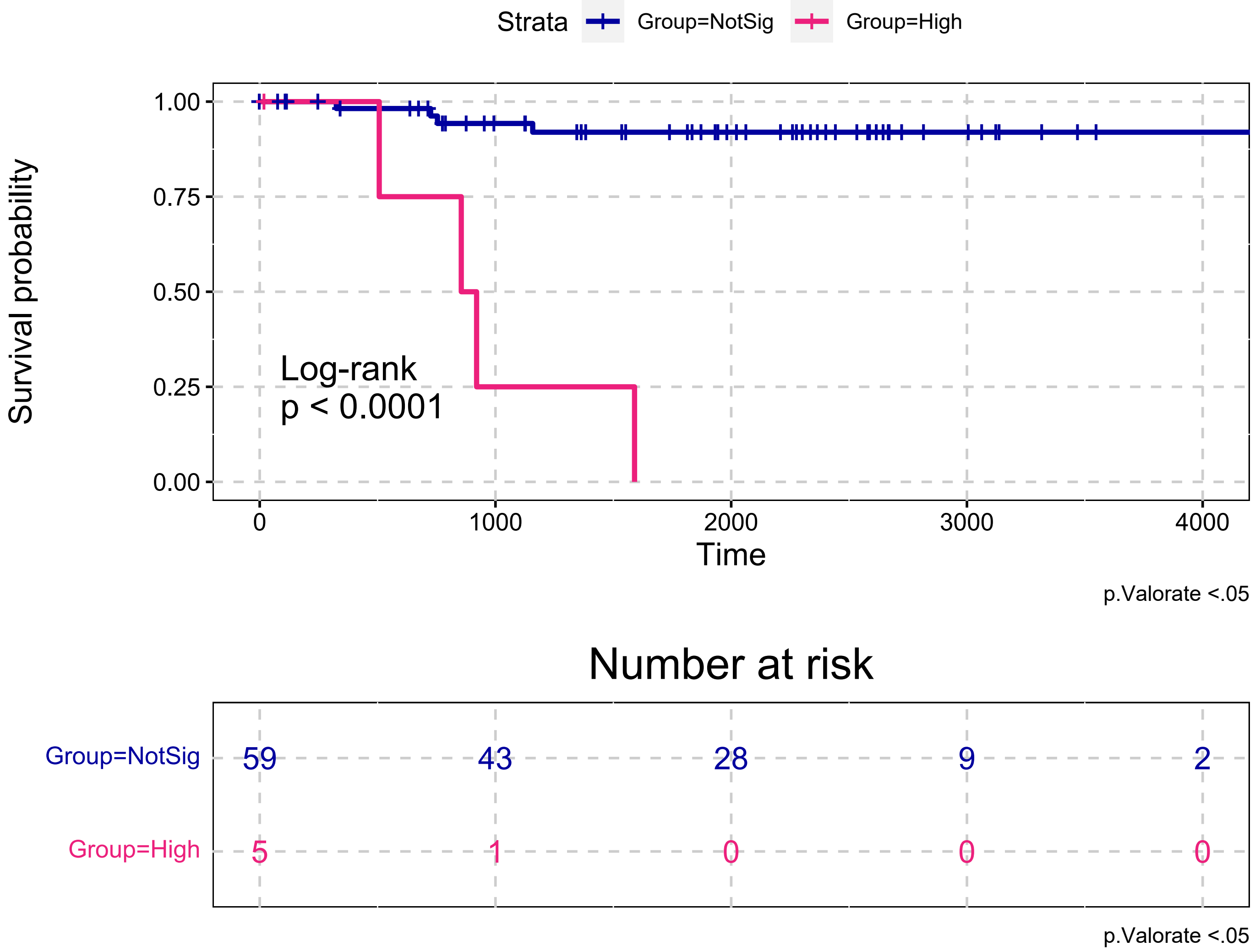

explanatory	beta	HR	L95	U95	p
High	2.99	19.81	4.82	81.42	0.00

n= 65, number of events =8
Score(logrank) test = p <.0001

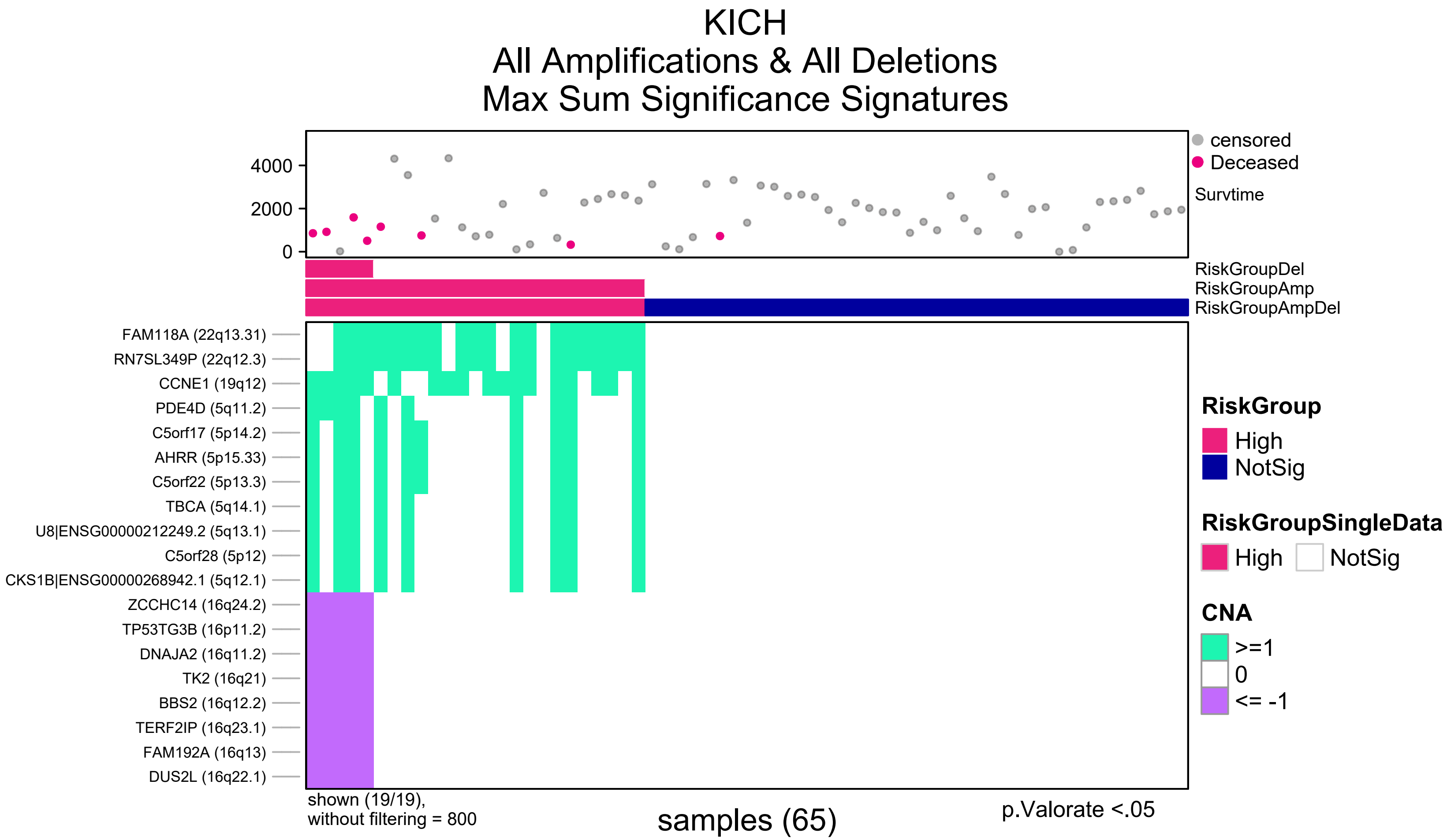
KICH
All Amplifications & All Deletions
Max Sum Significance Signatures
4000
2000
0
censored
Deceased
Survtime
RiskGroupDel
RiskGroupAmp
RiskGroupAmpDel
FAM118A (22q13.31)
RN7SL349P (22q12.3)
CCNE1 (19q12)
PDE4D (5q11.2)
C5orf17 (5p14.2)
AHRR (5p15.33)
C5orf22 (5p13.3)
TBCA (5q14.1)
U8|ENSG00000212249.2 (5q13.1)
C5orf28 (5p12)
CKS1B|ENSG00000268942.1 (5q12.1)
ZCCHC14 (16q24.2)
TP53TG3B (16p11.2)
DNAJA2 (16q11.2)
TK2 (16q21)
BBS2 (16q12.2)
TERF2IP (16q23.1)
FAM192A (16q13)
DUS2L (16q22.1)
RiskGroup
High
NotSig
RiskGroupSingleData
High
NotSig
CNA
>=1
0
<= -1
shown (19/19),
without filtering = 800
samples (65)
p.Valorate <.05

KICH
All Amplifications & All Deletions
Max Sum Significance Signatures

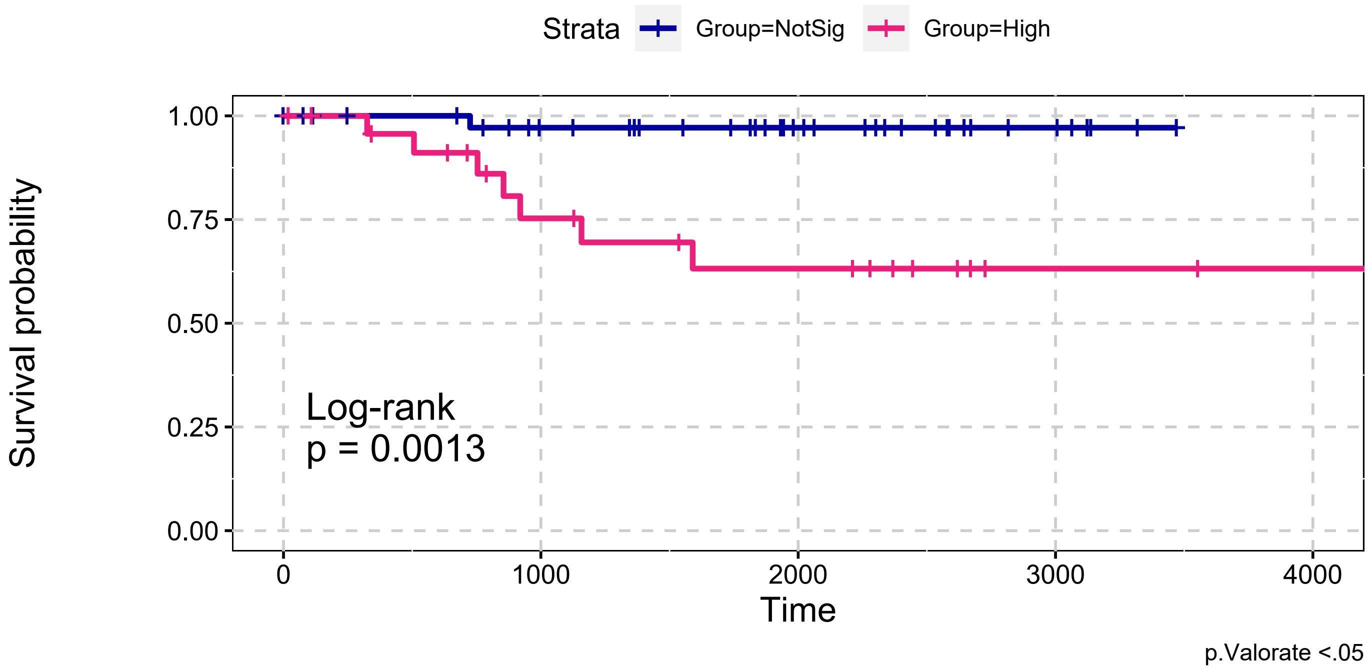

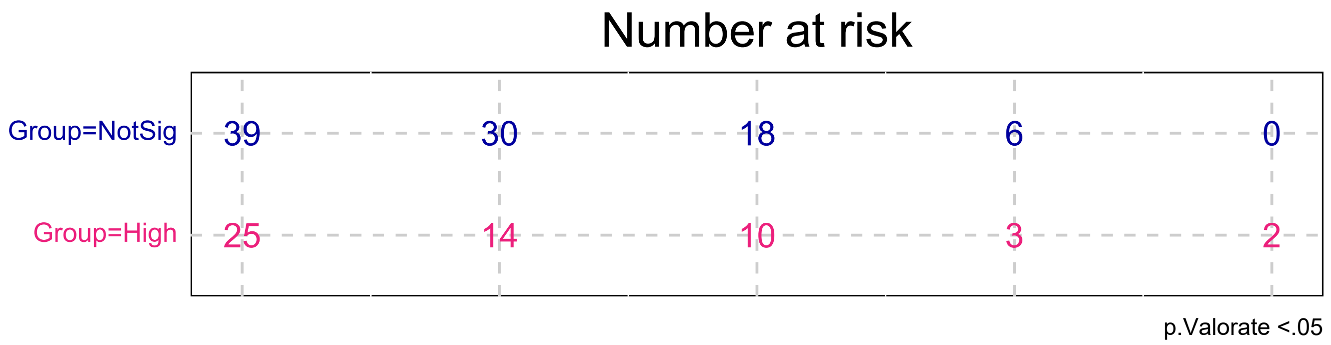

explanatory	beta	HR	L95	U95	p
High	2.63	13.81	1.70	112.44	0.01

n= 65, number of events =8
Score(logrank) test = 0.001

KICH
All Amplifications & All Deletions combining signatures

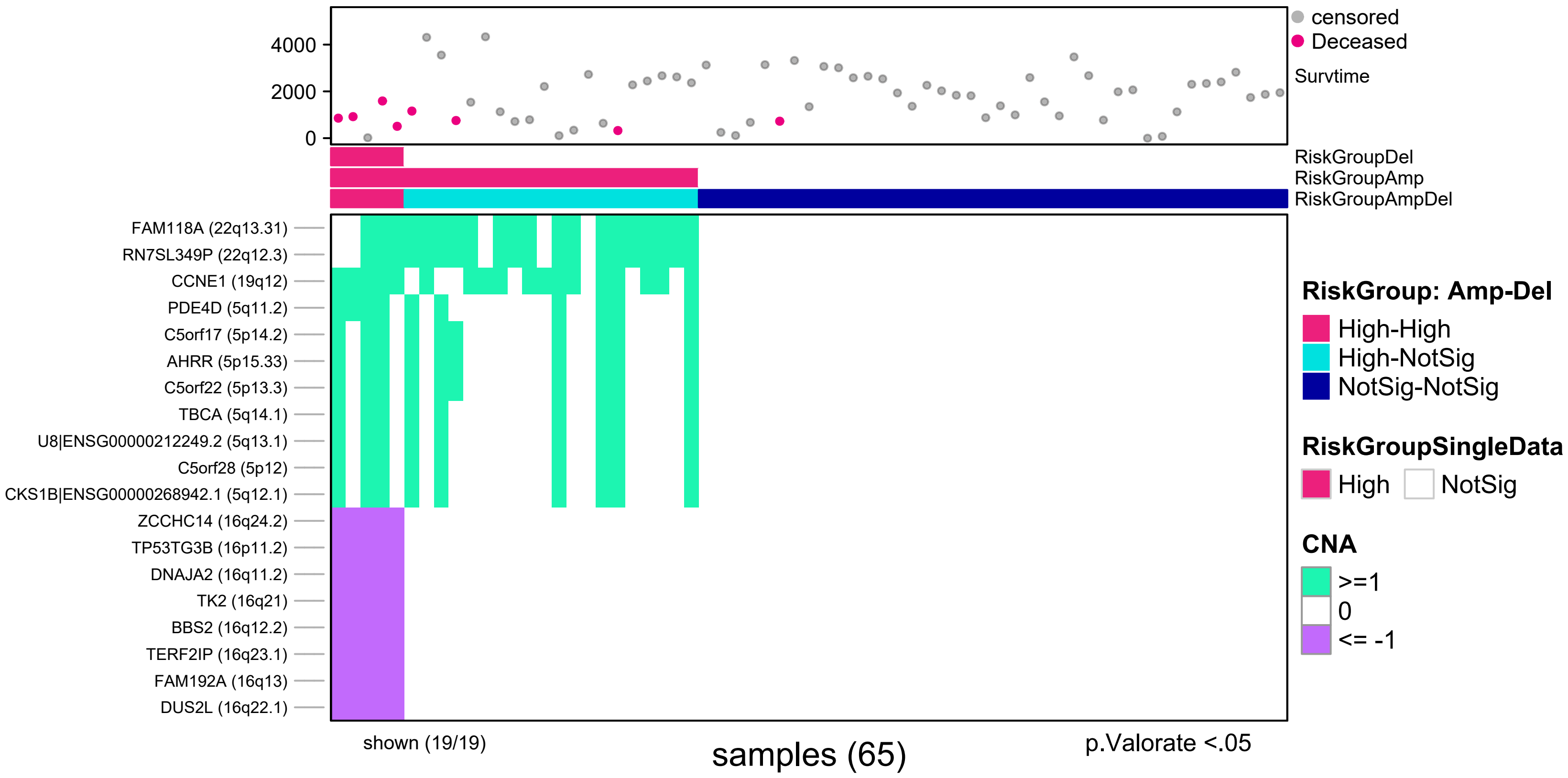

KICH
All Amplifications & All Deletions combining signatures

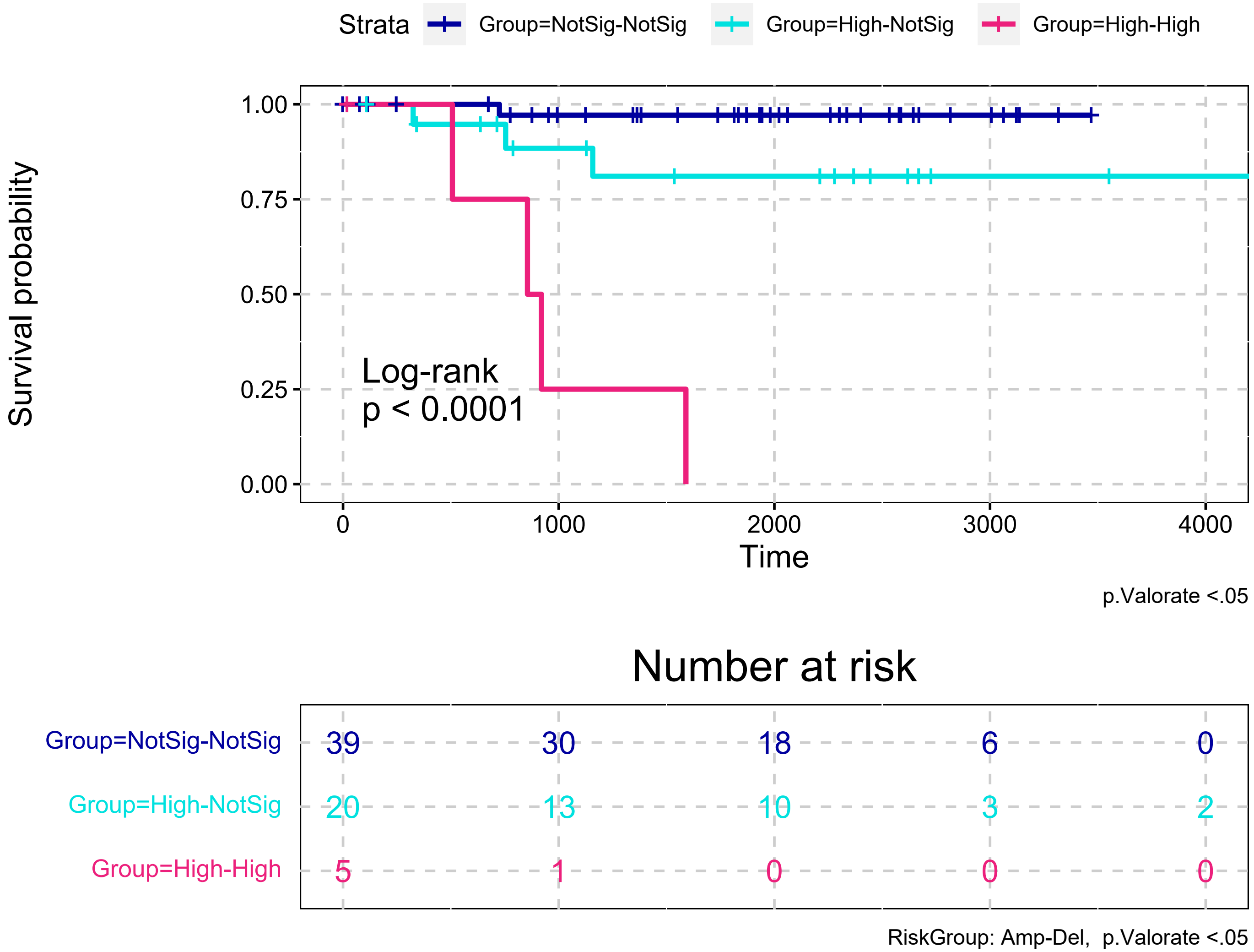

explanatory	beta	HR	L95	U95	p
High-NotSig	1.94	6.99	0.73	67.28	0.09
High-High	4.02	55.73	6.11	508.18	0.00

n= 65, number of events =8
Score(logrank) test = p <.0001
