# Supplementary material for: Systematic Modeling of Risk-Associated Copy Number Alterations in Cancer
Source: Int J Mol Sci. 2024 Sep 27;25(19):10455. doi: 10.3390/ijms251910455 (PMC11477427; doi:10.3390/ijms251910455)
Supplement: Supplementary file 1 [file ijms-25-10455-s001.zip › KIPANSignatureV12-sinSombreado.pdf]

KIPAN
All Amplifications
Single Data Signature

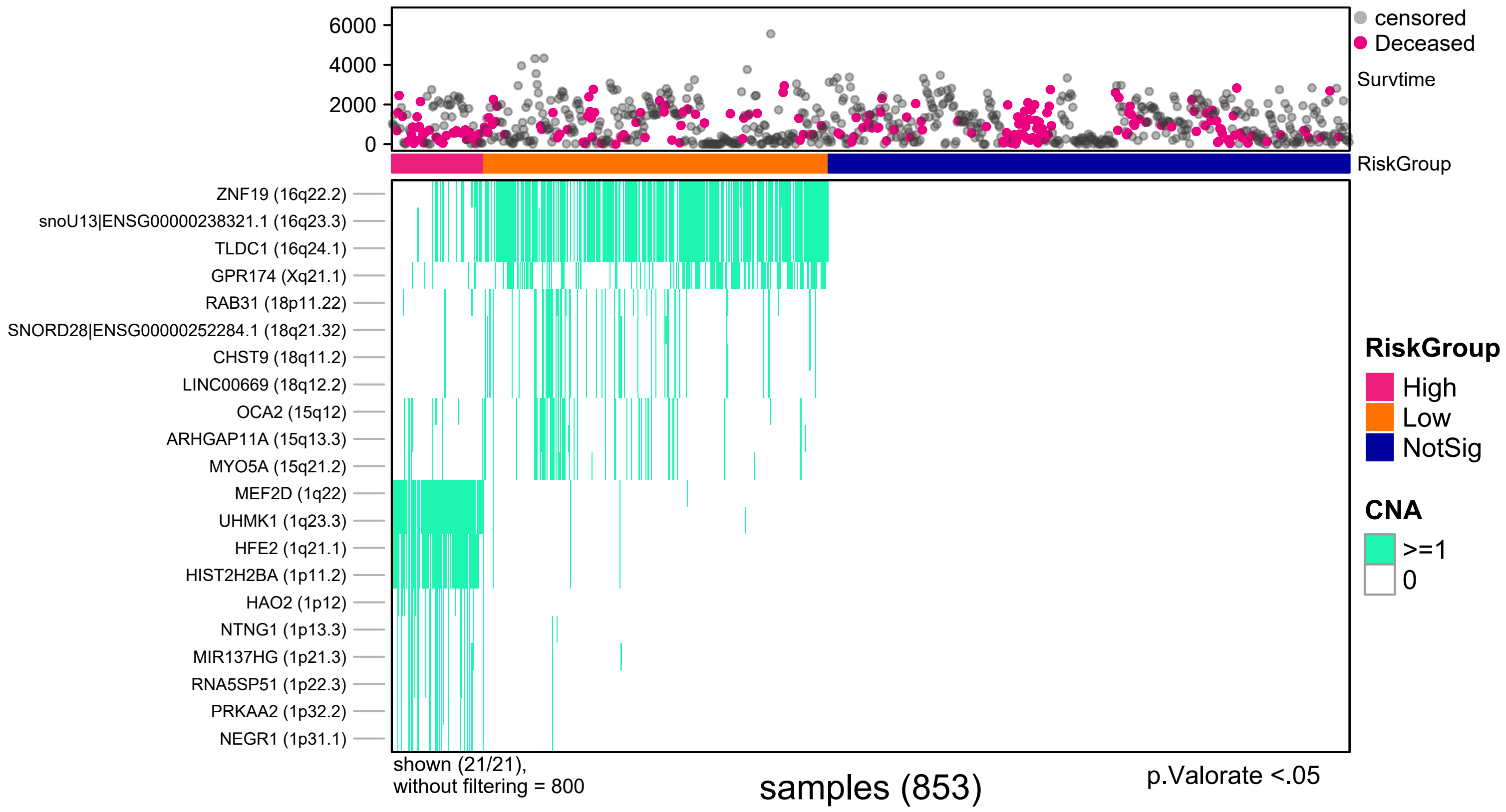

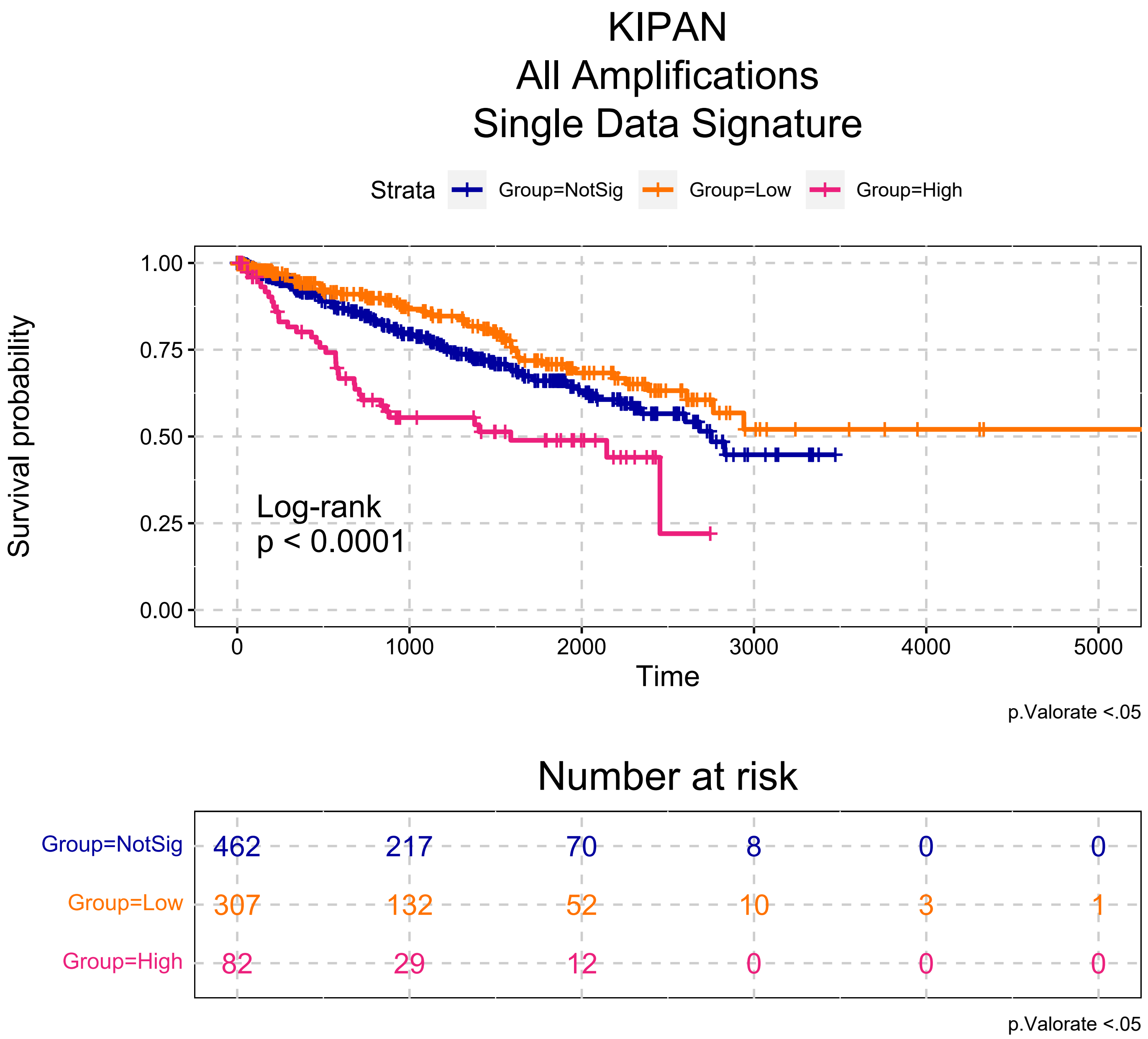

explanatory	beta	HR	L95	U95	p
Low	-0.30	0.74	0.53	1.02	0.07
High	0.69	2.00	1.37	2.93	0.00

n= 853, number of events =200
Score(logrank) test = p <.0001

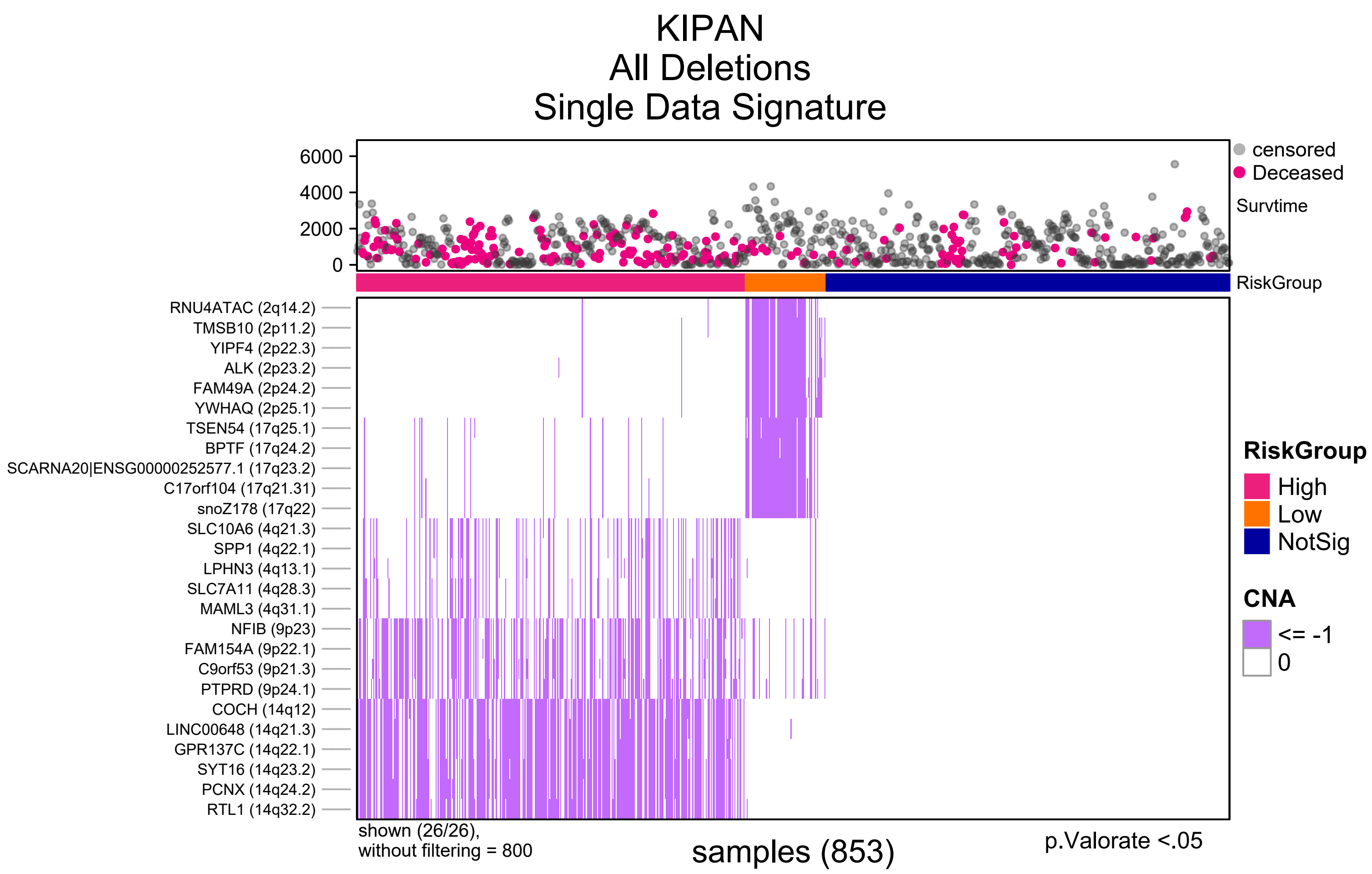
KIPAN
All Deletions
Single Data Signature
6000
4000
2000
0
censored
Deceased
Survtime
RiskGroup
RNU4ATAC (2q14.2)
TMSB10 (2p11.2)
YIPF4 (2p22.3)
ALK (2p23.2)
FAM49A (2p24.2)
YWHAQ (2p25.1)
TSEN54 (17q25.1)
BPTF (17q24.2)
SCARNA20|ENSG00000252577.1 (17q23.2)
C17orf104 (17q21.31)
snoZ178 (17q22)
SLC10A6 (4q21.3)
SPP1 (4q22.1)
LPHN3 (4q13.1)
SLC7A11 (4q28.3)
MAML3 (4q31.1)
NFIB (9p23)
FAM154A (9p22.1)
C9orf53 (9p21.3)
PTPRD (9p24.1)
COCH (14q12)
LINC00648 (14q21.3)
GPR137C (14q22.1)
SYT16 (14q23.2)
PCNX (14q24.2)
RTL1 (14q32.2)
RiskGroup
High
Low
NotSig
CNA
<= -1
0
shown (26/26),
without filtering = 800
samples (853)
p.Valorate <.05

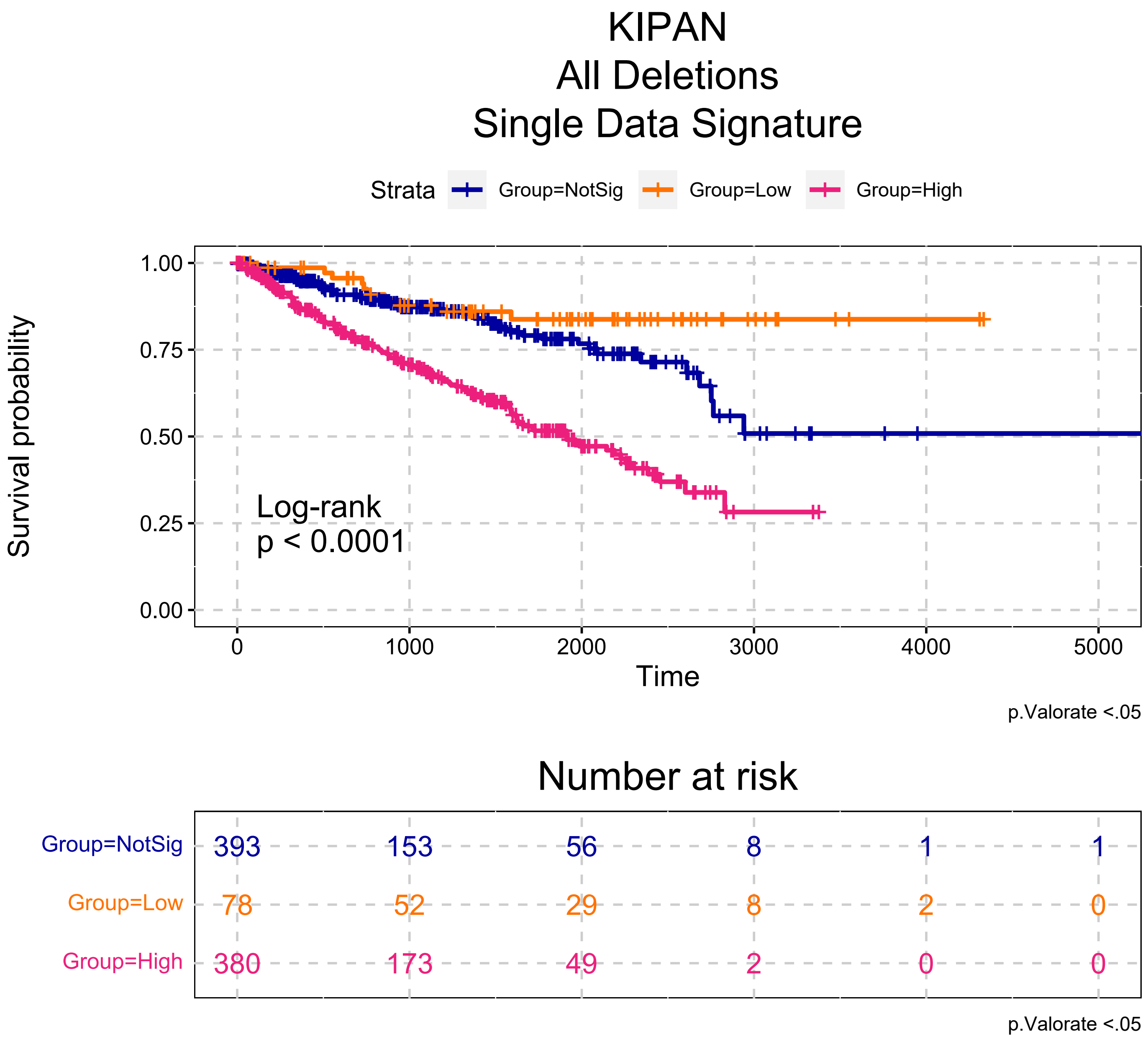

explanatory	beta	HR	L95	U95	p
Low	-0.62	0.54	0.27	1.06	0.07
High	0.94	2.55	1.85	3.50	0.00

n= 853, number of events =200
Score(logrank) test = p <.0001

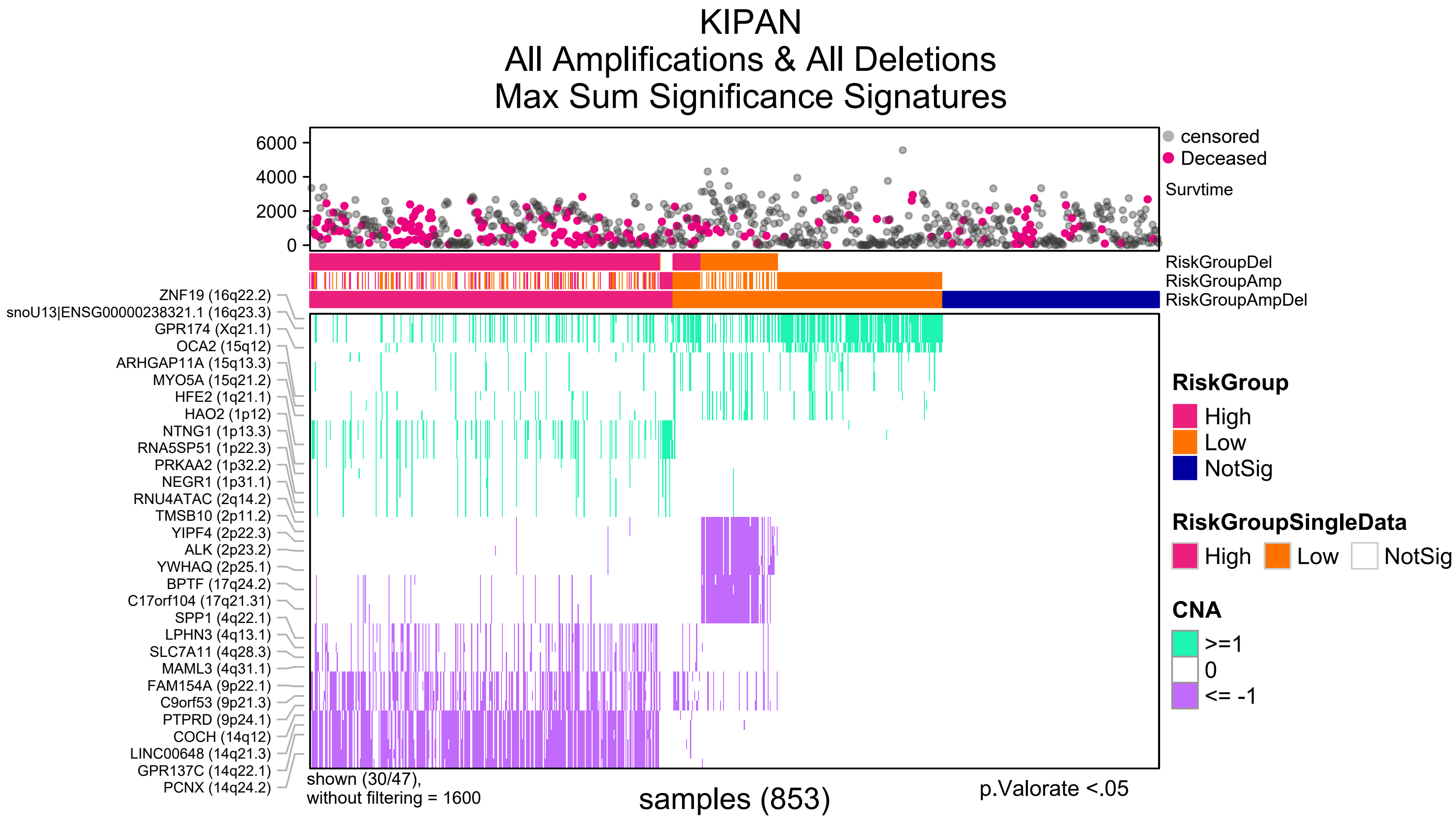
KIPAN
All Amplifications & All Deletions
Max Sum Significance Signatures
6000
4000
2000
0
censored
Deceased
Survtime
RiskGroupDel
RiskGroupAmp
RiskGroupAmpDel
ZNF19 (16q22.2)
snoU13|ENSG00000238321.1 (16q23.3)
GPR174 (Xq21.1)
OCA2 (15q12)
ARHGAP11A (15q13.3)
MYO5A (15q21.2)
HFE2 (1q21.1)
HAO2 (1p12)
NTNG1 (1p13.3)
RNA5SP51 (1p22.3)
PRKAA2 (1p32.2)
NEGR1 (1p31.1)
RNU4ATAC (2q14.2)
TMSB10 (2p11.2)
YIPF4 (2p22.3)
ALK (2p23.2)
YWHAQ (2p25.1)
BPTF (17q24.2)
C17orf104 (17q21.31)
SPP1 (4q22.1)
LPHN3 (4q13.1)
SLC7A11 (4q28.3)
MAML3 (4q31.1)
FAM154A (9p22.1)
C9orf53 (9p21.3)
PTPRD (9p24.1)
COCH (14q12)
LINC00648 (14q21.3)
GPR137C (14q22.1)
PCNX (14q24.2)
RiskGroup
High
Low
NotSig
RiskGroupSingleData
High
Low
NotSig
CNA
>=1
0
<= -1
shown (30/47),
without filtering = 1600
samples (853)
p.Valorate <.05

KIPAN
All Amplifications & All Deletions
Max Sum Significance Signatures

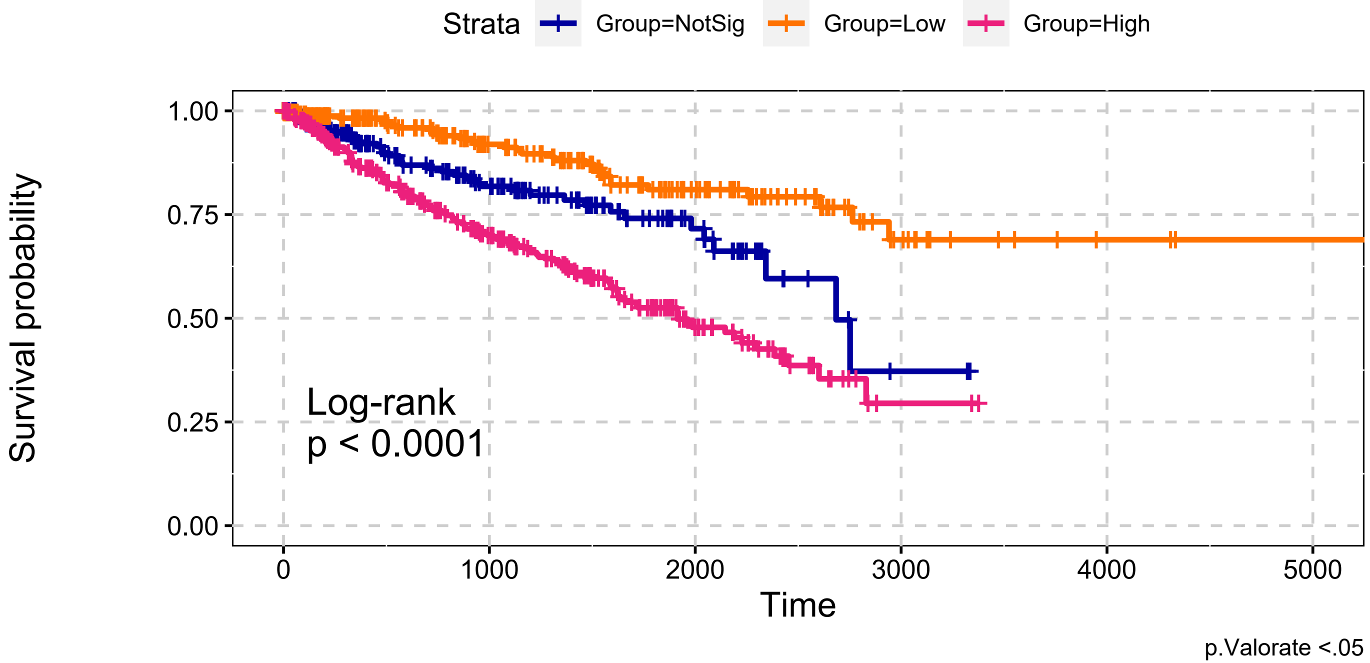

Number at risk

	0	1000	2000	3000	4000	5000
Group=NotSig	216	89	29	2	0	0
Group=Low	270	124	58	14	3	1
Group=High	365	165	47	2	0	0

p.Valorate <.05

**explanatory**	**beta**	**HR**	**L95**	**U95**	**p**
Low	-0.72	0.49	0.30	0.79	0.00
High	0.61	1.85	1.29	2.64	0.00

n= 853, number of events =200
Score(logrank) test = p <.0001

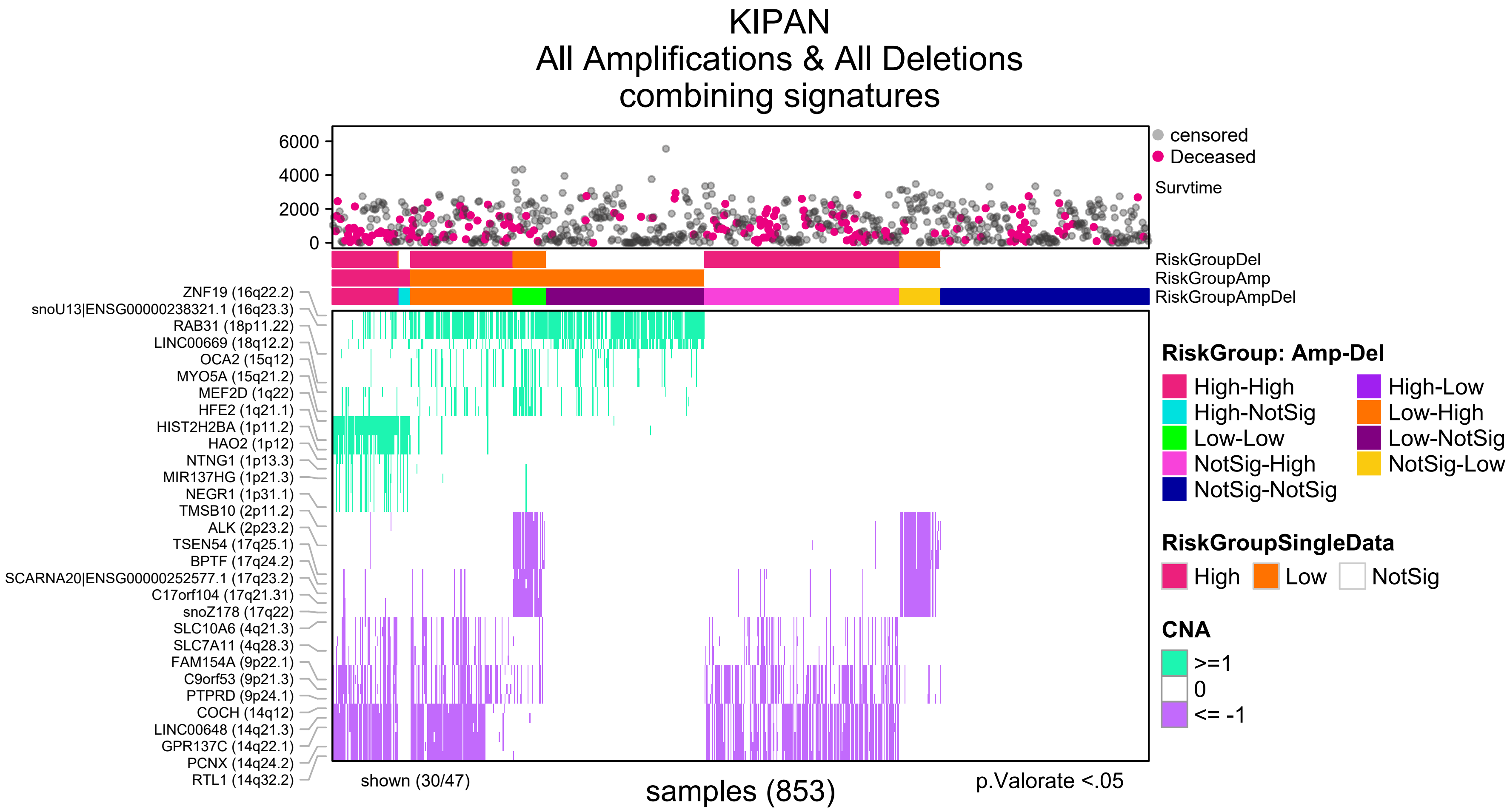
KIPAN
All Amplifications & All Deletions
combining signatures
6000
4000
2000
0
censored
Deceased
Survtime
RiskGroupDel
RiskGroupAmp
RiskGroupAmpDel
ZNF19 (16q22.2)
snoU13|ENSG00000238321.1 (16q23.3)
RAB31 (18p11.22)
LINC00669 (18q12.2)
OCA2 (15q12)
MYO5A (15q21.2)
MEF2D (1q22)
HFE2 (1q21.1)
HIST2H2BA (1p11.2)
HAO2 (1p12)
NTNG1 (1p13.3)
MIR137HG (1p21.3)
NEGR1 (1p31.1)
TMSB10 (2p11.2)
ALK (2p23.2)
TSEN54 (17q25.1)
BPTF (17q24.2)
SCARNA20|ENSG00000252577.1 (17q23.2)
C17orf104 (17q21.31)
snoZ178 (17q22)
SLC10A6 (4q21.3)
SLC7A11 (4q28.3)
FAM154A (9p22.1)
C9orf53 (9p21.3)
PTPRD (9p24.1)
COCH (14q12)
LINC00648 (14q21.3)
GPR137C (14q22.1)
PCNX (14q24.2)
RTL1 (14q32.2)
RiskGroup: Amp-Del
High-High
High-NotSig
Low-Low
NotSig-High
NotSig-NotSig
High-Low
Low-High
Low-NotSig
NotSig-Low
RiskGroupSingleData
High
Low
NotSig
CNA
>=1
0
<= -1
shown (30/47)
samples (853)
p.Valorate <.05

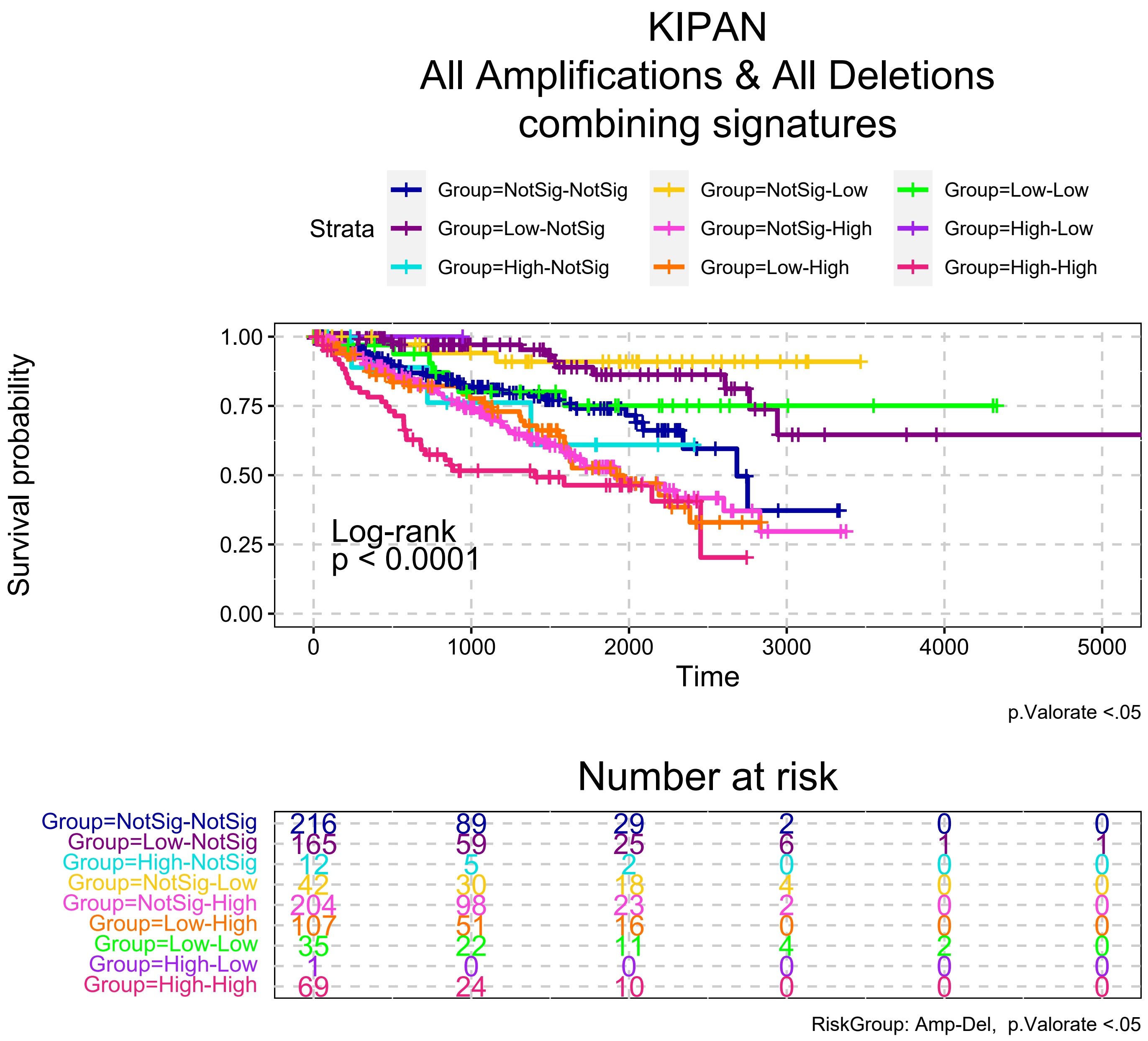

explanatory	beta	HR	L95	U95	p
Low-NotSig	-1.05	0.35	0.18	0.68	0.00
High-NotSig	0.26	1.29	0.40	4.18	0.67
NotSig-Low	-1.59	0.20	0.06	0.66	0.01
NotSig-High	0.53	1.69	1.14	2.51	0.01
Low-High	0.56	1.76	1.12	2.77	0.01
Low-Low	-0.45	0.64	0.28	1.43	0.27
High-Low	-12.99	0.00	0.00	Inf	0.99
High-High	0.97	2.63	1.65	4.20	0.00

n= 853, number of events =200
Score(logrank) test = p <.0001

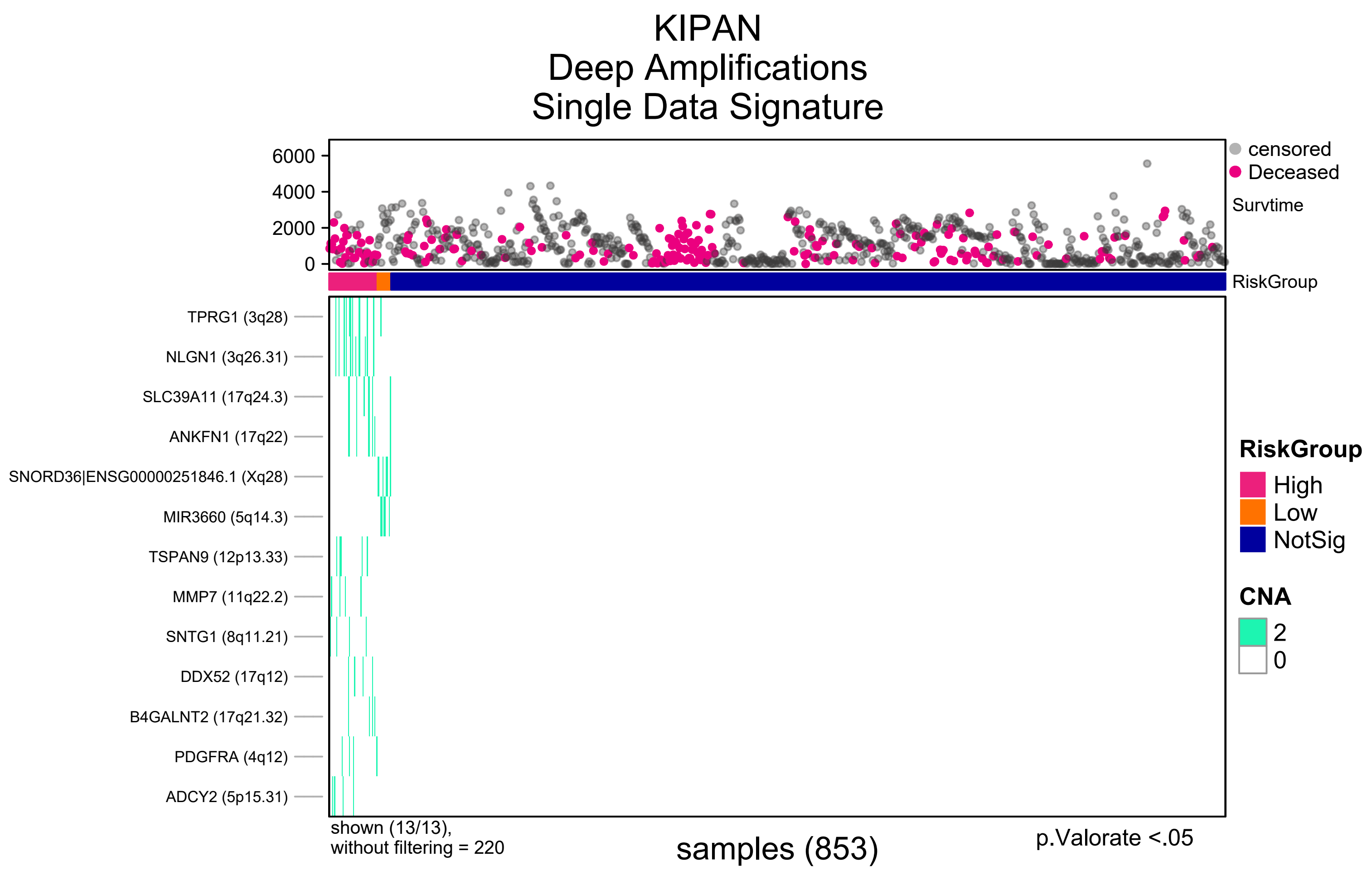
KIPAN
Deep Amplifications
Single Data Signature
6000
4000
2000
0
censored
Deceased
Survtime
RiskGroup
TPRG1 (3q28)
NLGN1 (3q26.31)
SLC39A11 (17q24.3)
ANKFN1 (17q22)
SNORD36|ENSG00000251846.1 (Xq28)
MIR3660 (5q14.3)
TSPAN9 (12p13.33)
MMP7 (11q22.2)
SNTG1 (8q11.21)
DDX52 (17q12)
B4GALNT2 (17q21.32)
PDGFRA (4q12)
ADCY2 (5p15.31)
RiskGroup
High
Low
NotSig
CNA
2
0
shown (13/13),
without filtering = 220
samples (853)
p.Valorate <.05

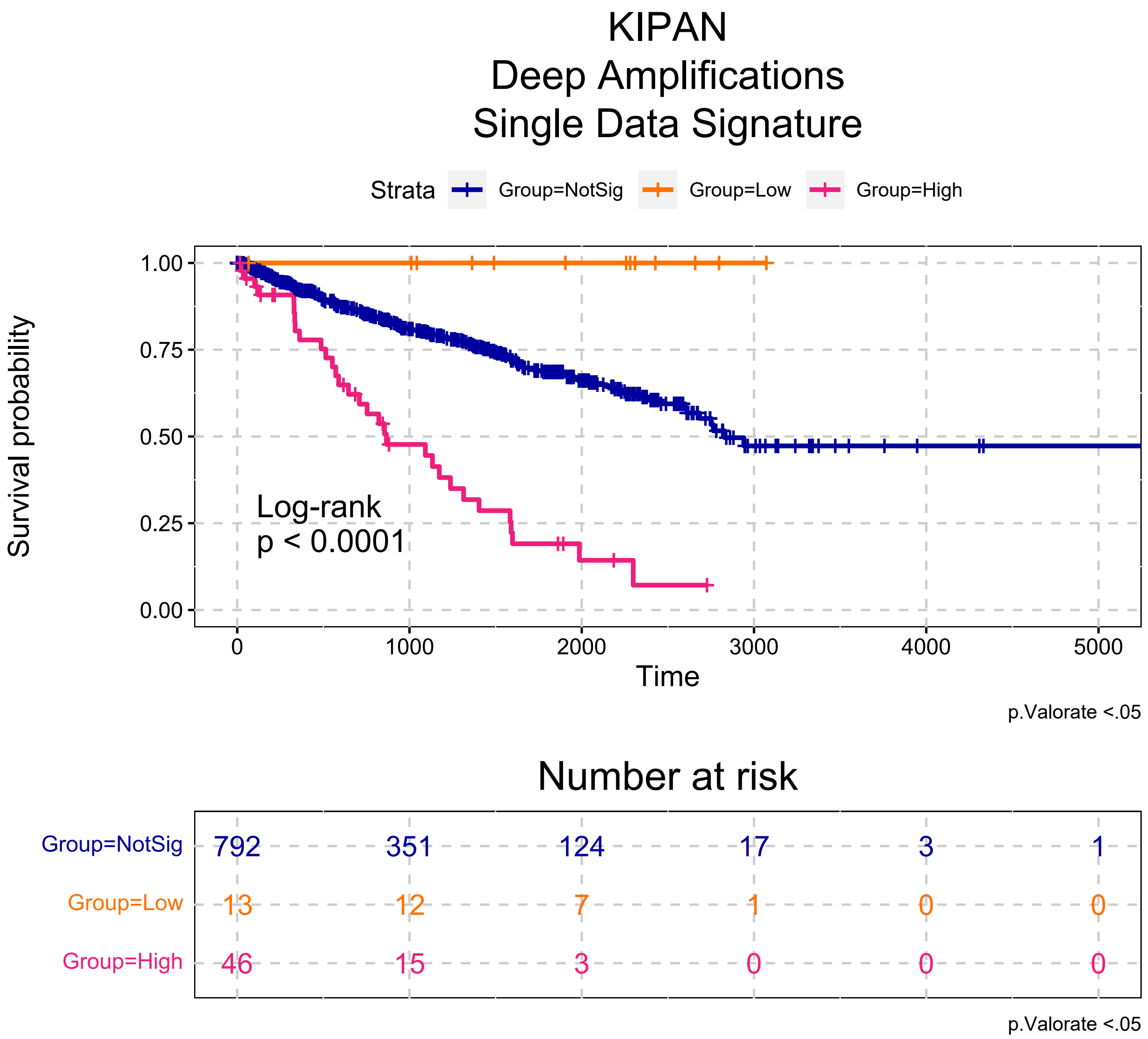

explanatory	beta	HR	L95	U95	p
Low	-16.49	0.00	0.00	Inf	0.99
High	1.37	3.94	2.68	5.80	0.00

n= 853, number of events =200
Score(logrank) test = p <.0001

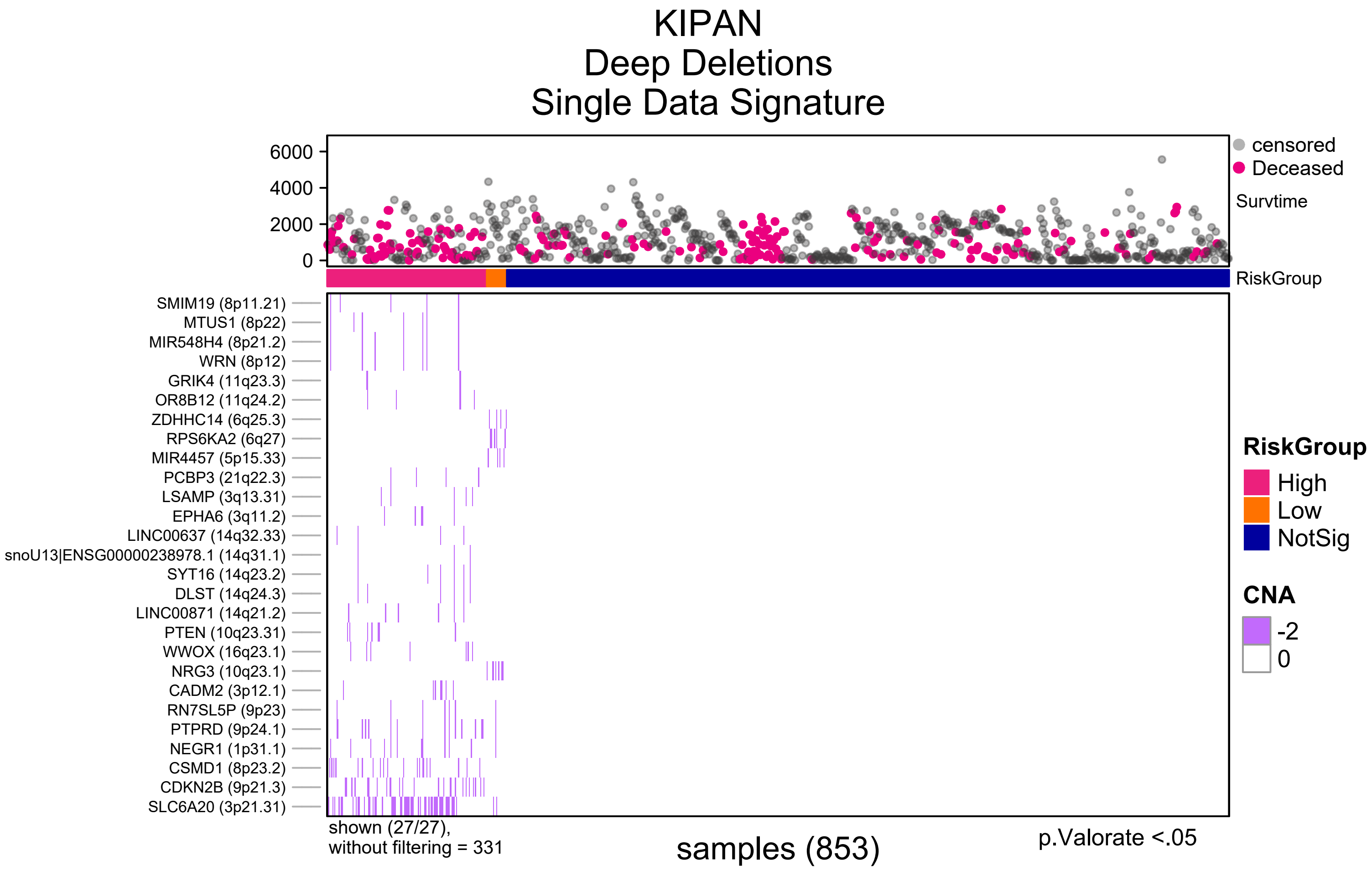

KIPAN
Deep Deletions
Single Data Signature
6000
4000
2000
0
censored
Deceased
Survtime
RiskGroup
SMIM19 (8p11.21)
MTUS1 (8p22)
MIR548H4 (8p21.2)
WRN (8p12)
GRIK4 (11q23.3)
OR8B12 (11q24.2)
ZDHHC14 (6q25.3)
RPS6KA2 (6q27)
MIR4457 (5p15.33)
PCBP3 (21q22.3)
LSAMP (3q13.31)
EPHA6 (3q11.2)
LINC00637 (14q32.33)
snoU13|ENSG00000238978.1 (14q31.1)
SYT16 (14q23.2)
DLST (14q24.3)
LINC00871 (14q21.2)
PTEN (10q23.31)
WWOX (16q23.1)
NRG3 (10q23.1)
CADM2 (3p12.1)
RN7SL5P (9p23)
PTPRD (9p24.1)
NEGR1 (1p31.1)
CSMD1 (8p23.2)
CDKN2B (9p21.3)
SLC6A20 (3p21.31)
RiskGroup
High
Low
NotSig
CNA
-2
0
shown (27/27),
without filtering = 331
samples (853)
p.Valorate <.05

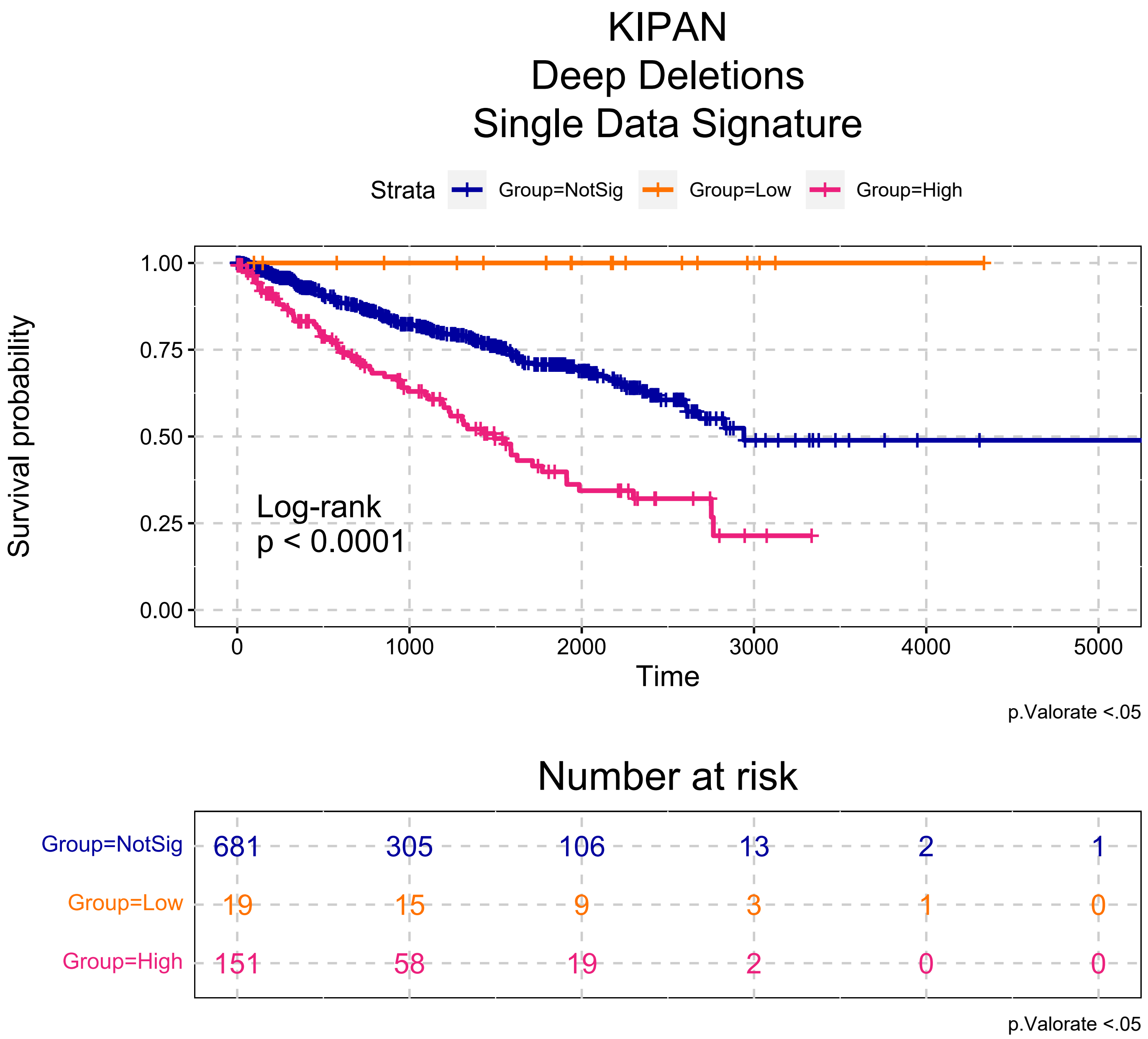

explanatory	beta	HR	L95	U95	p
Low	-17.01	0.00	0.00	Inf	0.99
High	0.90	2.45	1.82	3.29	0.00

n= 853, number of events =200
Score(logrank) test = p <.0001

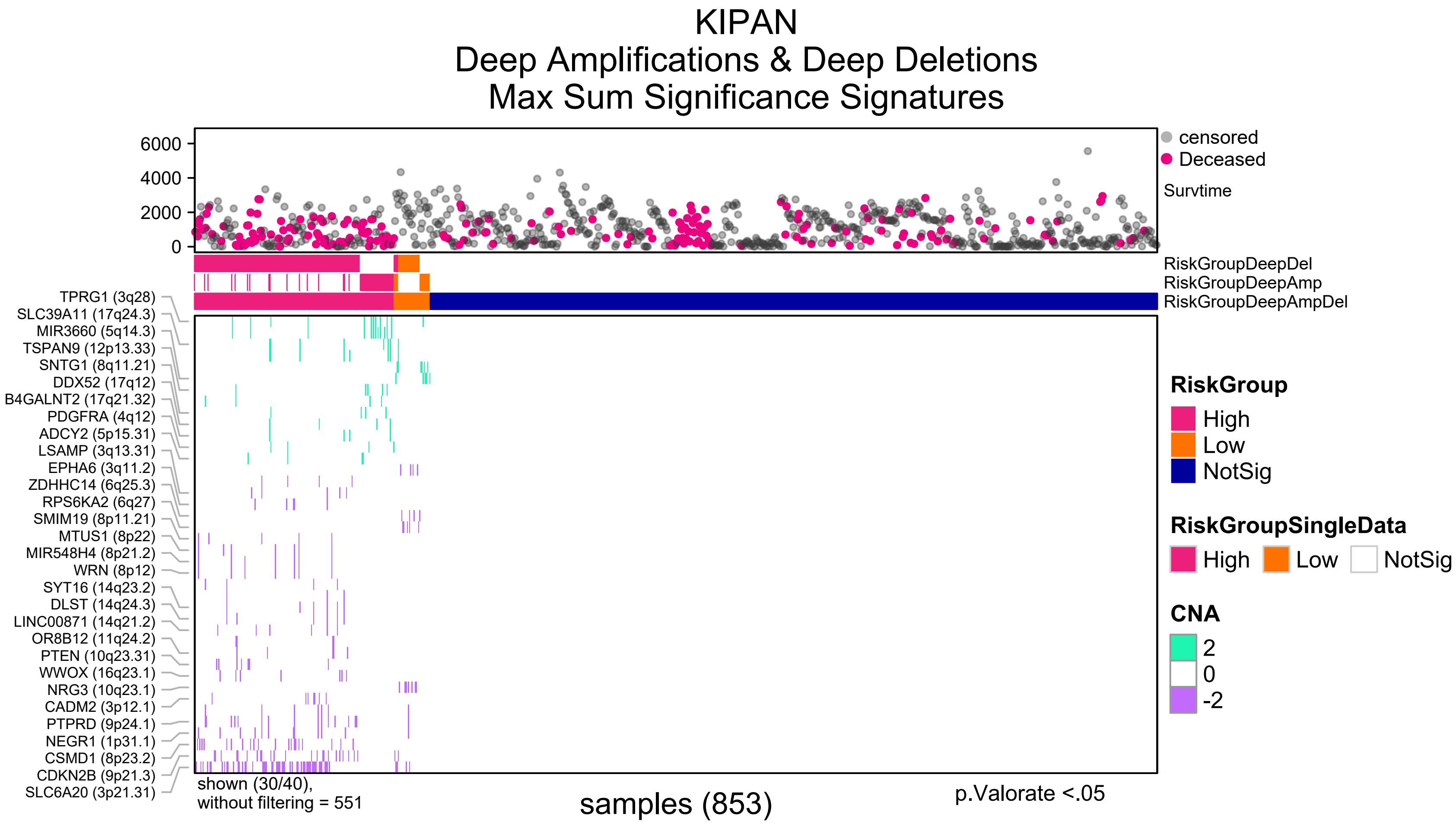
KIPAN
Deep Amplifications & Deep Deletions
Max Sum Significance Signatures
6000
4000
2000
0
censored
Deceased
Survtime
RiskGroupDeepDel
RiskGroupDeepAmp
RiskGroupDeepAmpDel
TPRG1 (3q28)
SLC39A11 (17q24.3)
MIR3660 (5q14.3)
TSPAN9 (12p13.33)
SNTG1 (8q11.21)
DDX52 (17q12)
B4GALNT2 (17q21.32)
PDGFRA (4q12)
ADCY2 (5p15.31)
LSAMP (3q13.31)
EPHA6 (3q11.2)
ZDHHC14 (6q25.3)
RPS6KA2 (6q27)
SMIM19 (8p11.21)
MTUS1 (8p22)
MIR548H4 (8p21.2)
WRN (8p12)
SYT16 (14q23.2)
DLST (14q24.3)
LINC00871 (14q21.2)
OR8B12 (11q24.2)
PTEN (10q23.31)
WWOX (16q23.1)
NRG3 (10q23.1)
CADM2 (3p12.1)
PTPRD (9p24.1)
NEGR1 (1p31.1)
CSMD1 (8p23.2)
CDKN2B (9p21.3)
SLC6A20 (3p21.31)
RiskGroup
High
Low
NotSig
RiskGroupSingleData
High
Low
NotSig
CNA
2
0
-2
shown (30/40),
without filtering = 551
samples (853)
p.Valorate <.05

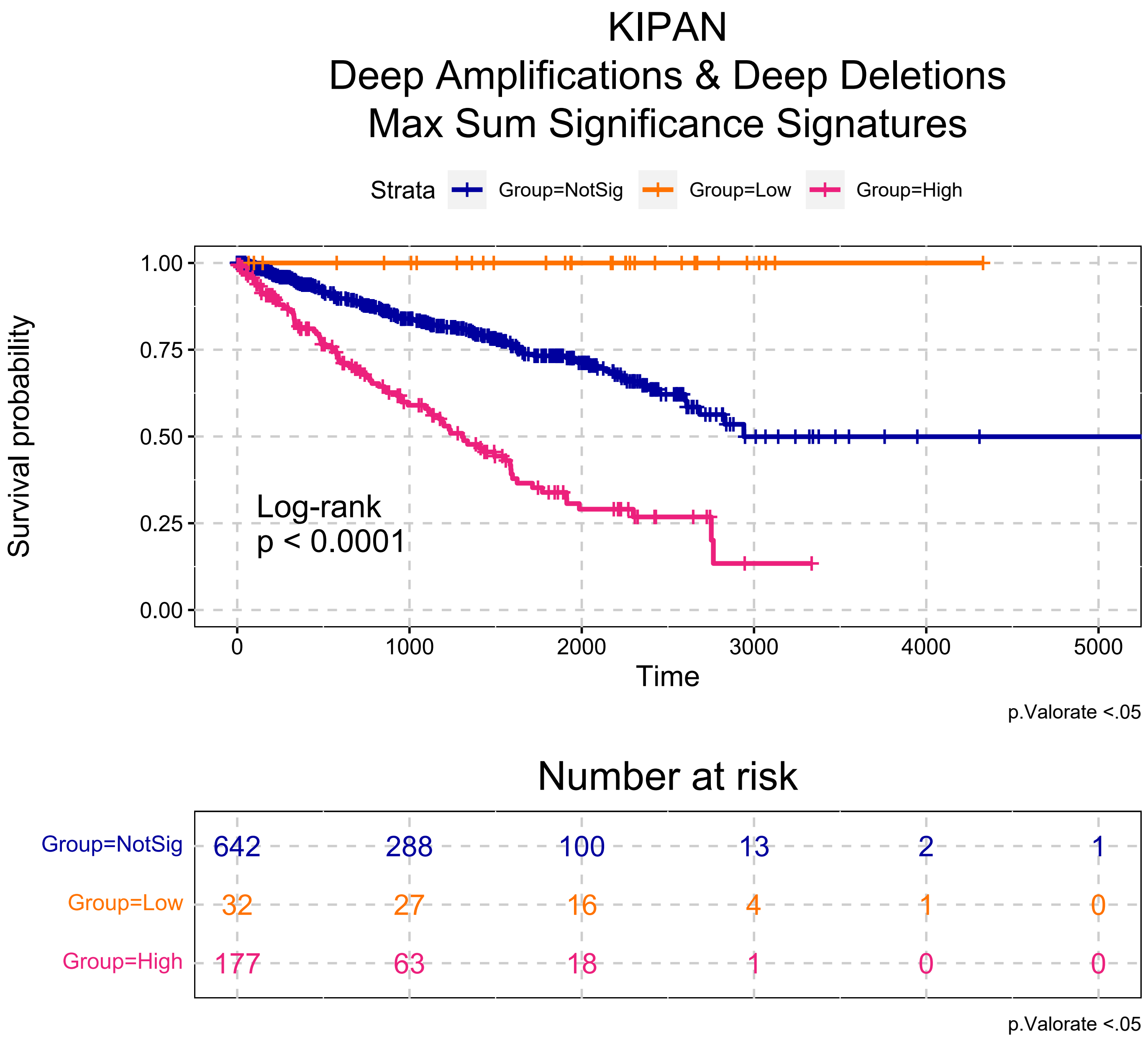

explanatory	beta	HR	L95	U95	p
Low	-17.09	0.00	0.00	Inf	0.99
High	1.14	3.12	2.35	4.13	0.00

n= 853, number of events =200
Score(logrank) test = p <.0001

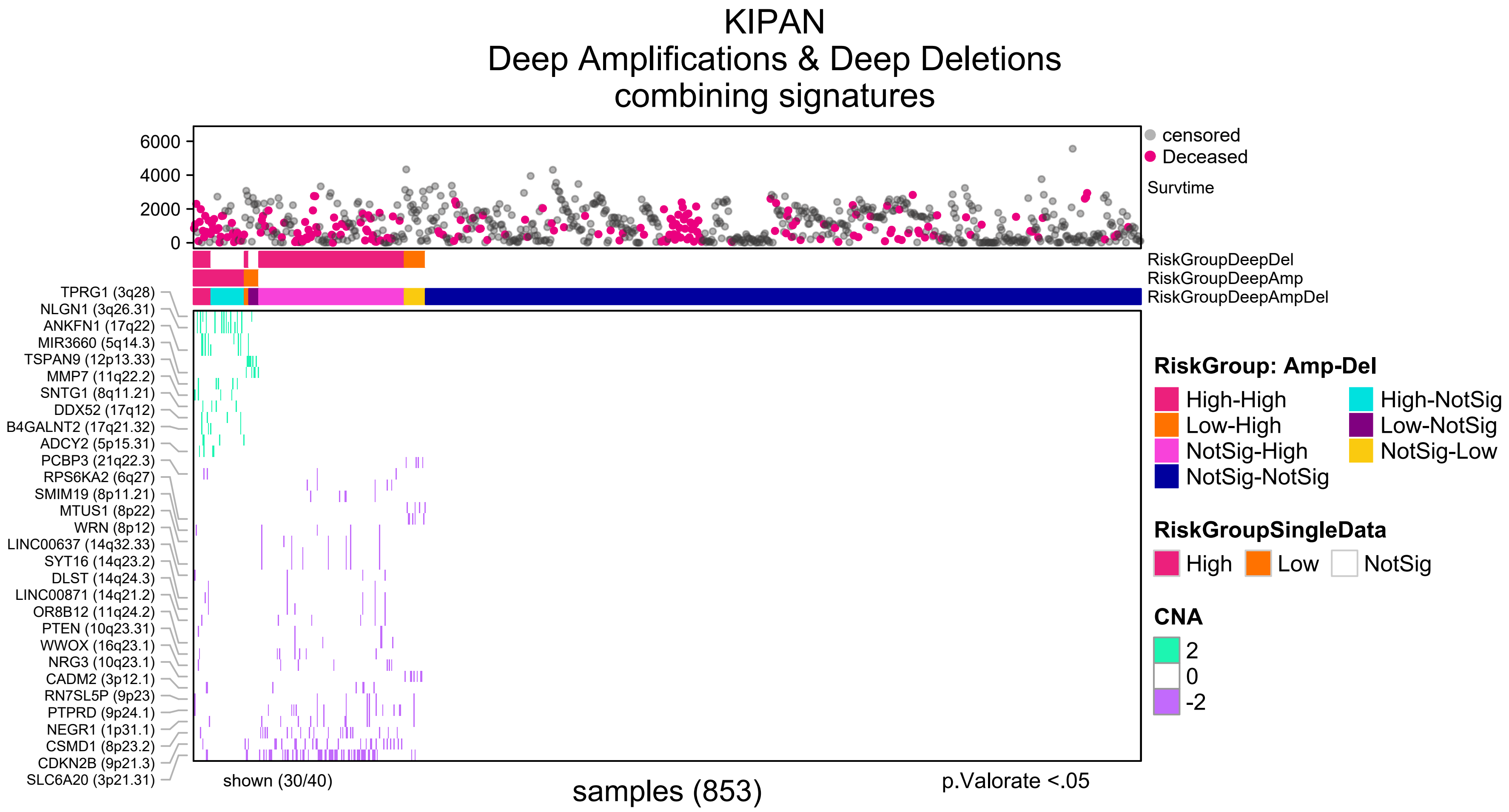
KIPAN
Deep Amplifications & Deep Deletions
combining signatures
6000
4000
2000
0
censored
Deceased
Survtime
RiskGroupDeepDel
RiskGroupDeepAmp
RiskGroupDeepAmpDel
TPRG1 (3q28)
NLGN1 (3q26.31)
ANKFN1 (17q22)
MIR3660 (5q14.3)
TSPAN9 (12p13.33)
MMP7 (11q22.2)
SNTG1 (8q11.21)
DDX52 (17q12)
B4GALNT2 (17q21.32)
ADCY2 (5p15.31)
PCBP3 (21q22.3)
RPS6KA2 (6q27)
SMIM19 (8p11.21)
MTUS1 (8p22)
WRN (8p12)
LINC00637 (14q32.33)
SYT16 (14q23.2)
DLST (14q24.3)
LINC00871 (14q21.2)
OR8B12 (11q24.2)
PTEN (10q23.31)
WWOX (16q23.1)
NRG3 (10q23.1)
CADM2 (3p12.1)
RN7SL5P (9p23)
PTPRD (9p24.1)
NEGR1 (1p31.1)
CSMD1 (8p23.2)
CDKN2B (9p21.3)
SLC6A20 (3p21.31)
RiskGroup: Amp-Del
High-High
Low-High
NotSig-High
NotSig-NotSig
High-NotSig
Low-NotSig
NotSig-Low
RiskGroupSingleData
High
Low
NotSig
CNA
2
0
-2
shown (30/40)
samples (853)
p.Valorate <.05

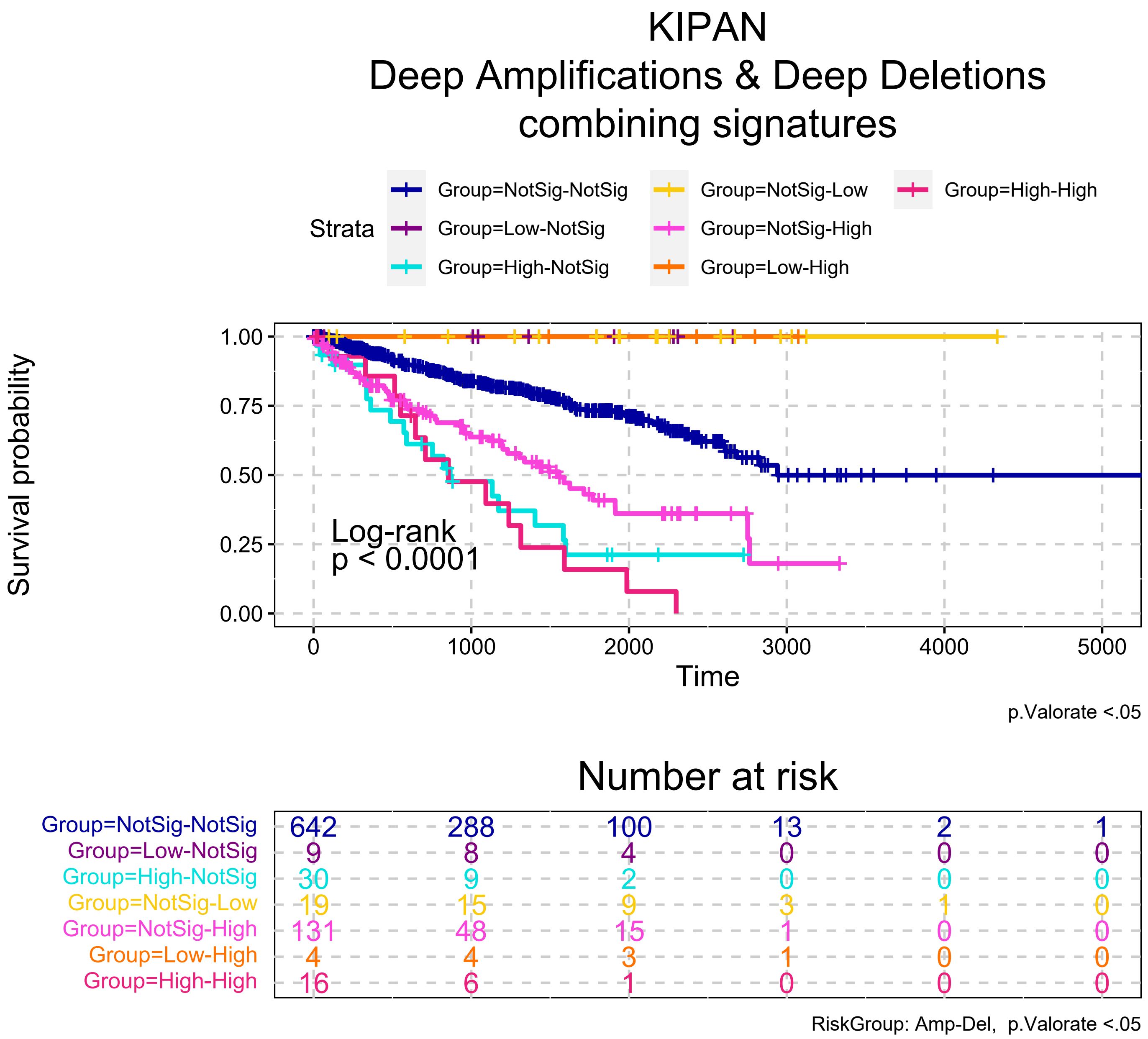

explanatory	beta	HR	L95	U95	p
Low-NotSig	-17.33	0.00	0.00	Inf	1.00
High-NotSig	1.46	4.30	2.61	7.08	0.00
NotSig-Low	-17.31	0.00	0.00	Inf	0.99
NotSig-High	0.96	2.61	1.88	3.61	0.00
Low-High	-17.30	0.00	0.00	Inf	1.00
High-High	1.69	5.44	3.06	9.67	0.00

n= 853, number of events =200
Score(logrank) test = p <.0001
