# Supplementary material for: Systematic Modeling of Risk-Associated Copy Number Alterations in Cancer
Source: Int J Mol Sci. 2024 Sep 27;25(19):10455. doi: 10.3390/ijms251910455 (PMC11477427; doi:10.3390/ijms251910455)
Supplement: Supplementary file 1 [file ijms-25-10455-s001.zip › KIRCSignatureV12-sinSombreado.pdf]

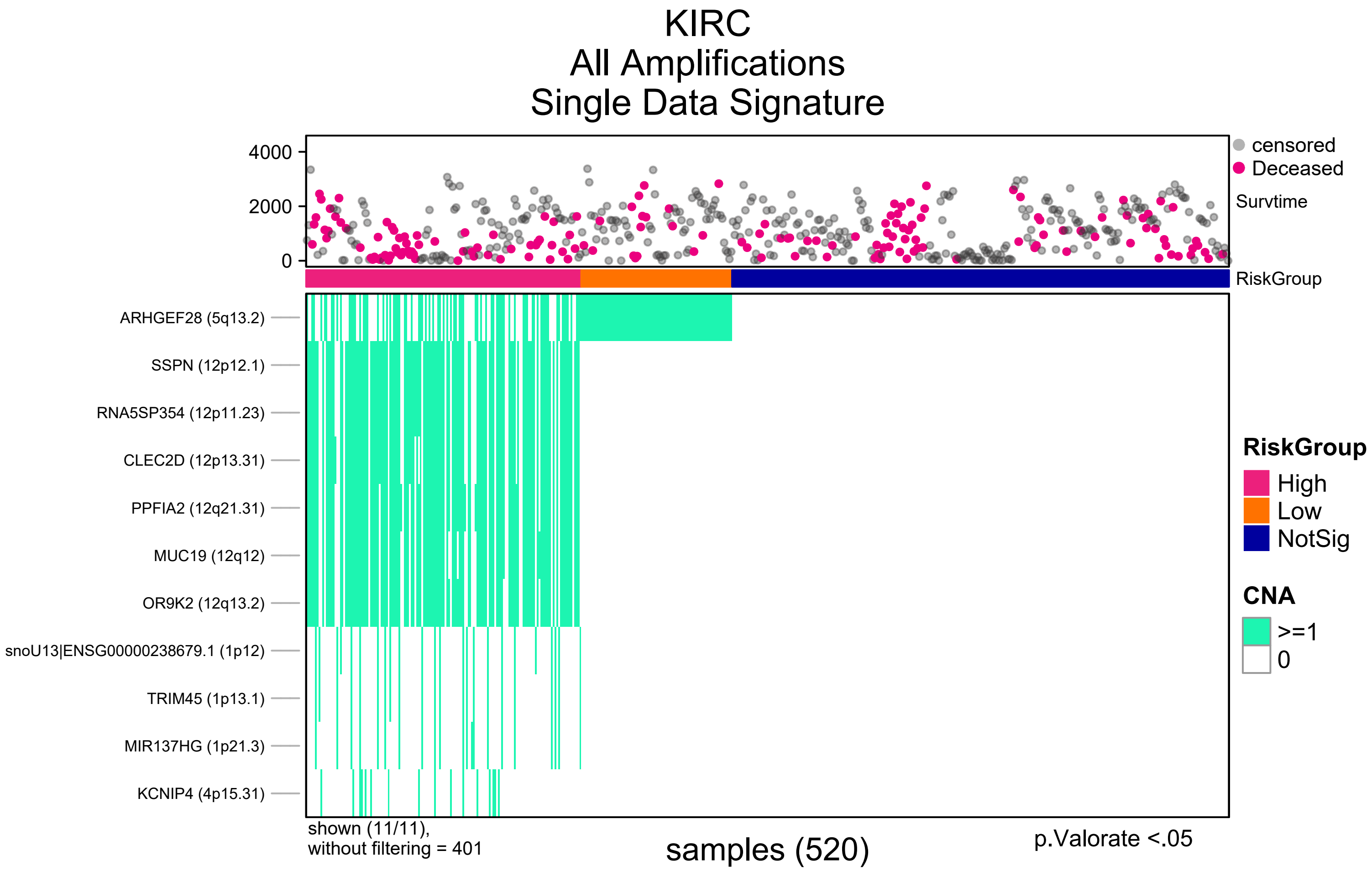
KIRC
All Amplifications
Single Data Signature
4000
2000
0
censored
Deceased
Survtime
RiskGroup
ARHGEF28 (5q13.2)
SSPN (12p12.1)
RNA5SP354 (12p11.23)
CLEC2D (12p13.31)
PPFIA2 (12q21.31)
MUC19 (12q12)
OR9K2 (12q13.2)
snoU13|ENSG00000238679.1 (1p12)
TRIM45 (1p13.1)
MIR137HG (1p21.3)
KCNIP4 (4p15.31)
RiskGroup
High
Low
NotSig
CNA
>=1
0
shown (11/11),
without filtering = 401
samples (520)
p.Valorate <.05

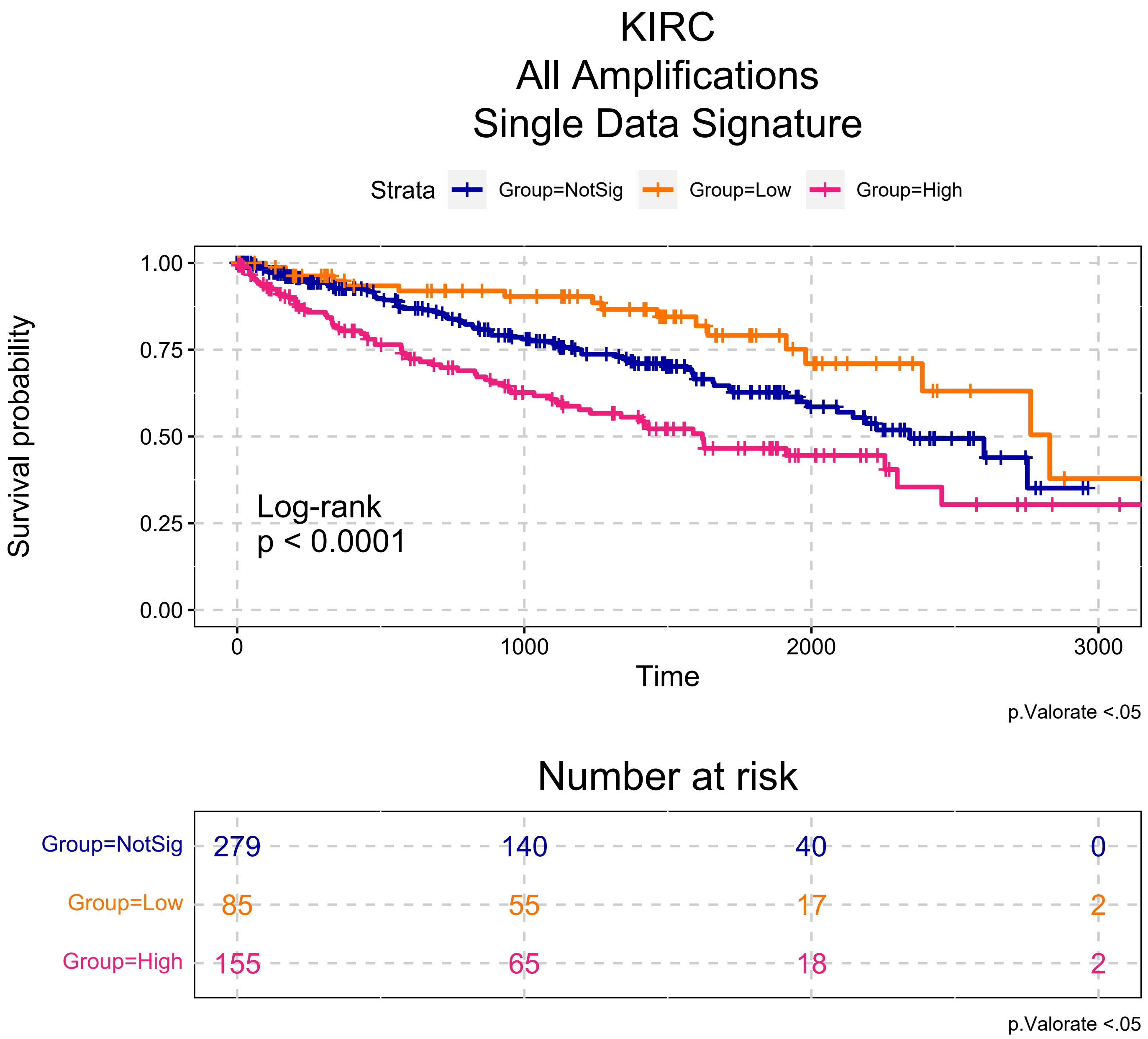

explanatory	beta	HR	L95	U95	p
Low	-0.54	0.58	0.34	0.99	0.04
High	0.54	1.72	1.24	2.39	0.00

n= 520, number of events =160
Score(logrank) test = p <.0001

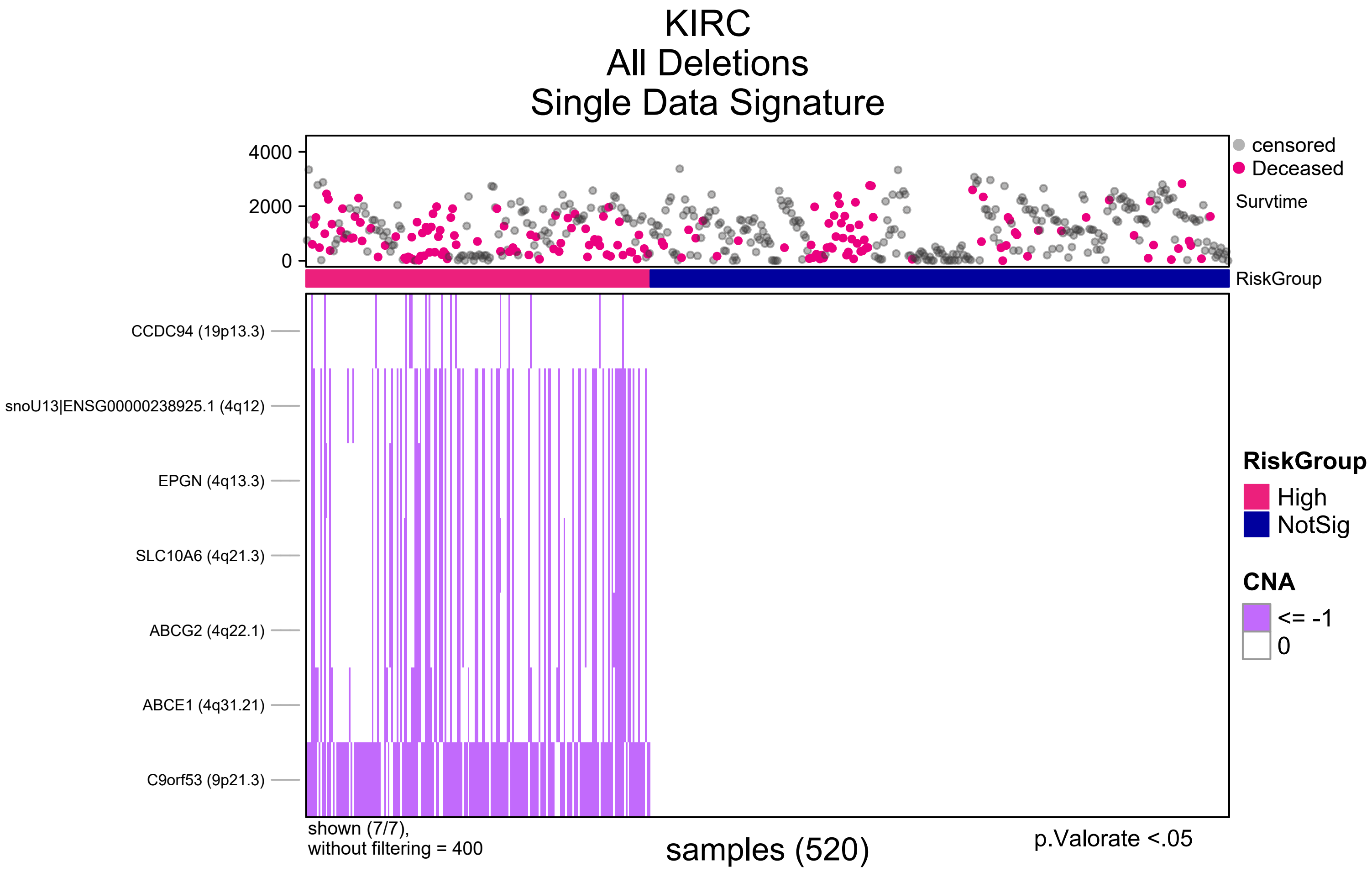
KIRC
All Deletions
Single Data Signature
4000
2000
0
censored
Deceased
Survtime
RiskGroup
CCDC94 (19p13.3)
snoU13|ENSG00000238925.1 (4q12)
EPGN (4q13.3)
SLC10A6 (4q21.3)
ABCG2 (4q22.1)
ABCE1 (4q31.21)
C9orf53 (9p21.3)
RiskGroup
High
NotSig
CNA
<= -1
0
shown (7/7),
without filtering = 400
samples (520)
p.Valorate <.05

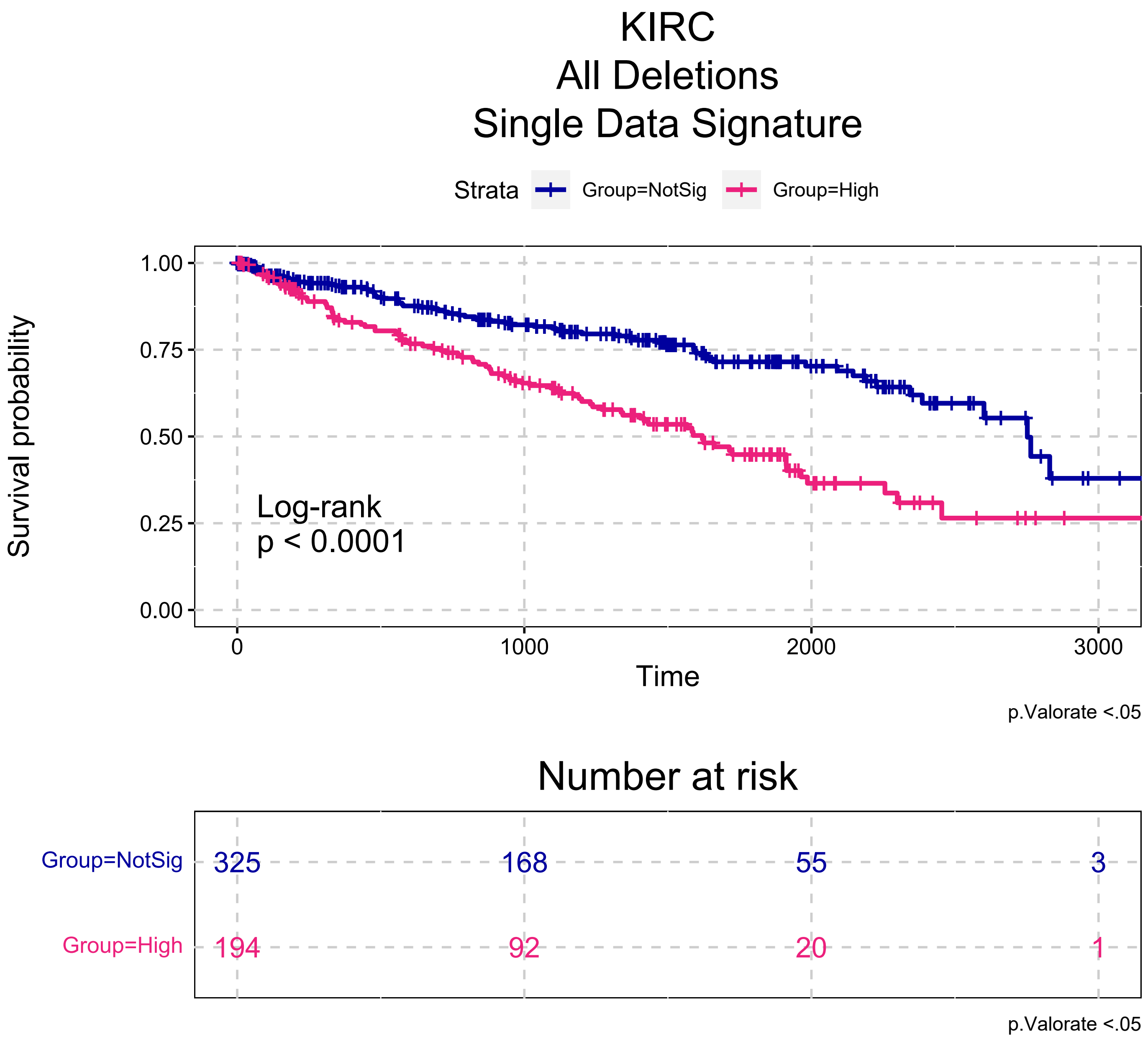

explanatory	beta	HR	L95	U95	p
High	0.81	2.25	1.64	3.07	0.00

n= 520, number of events =160
Score(logrank) test = p <.0001

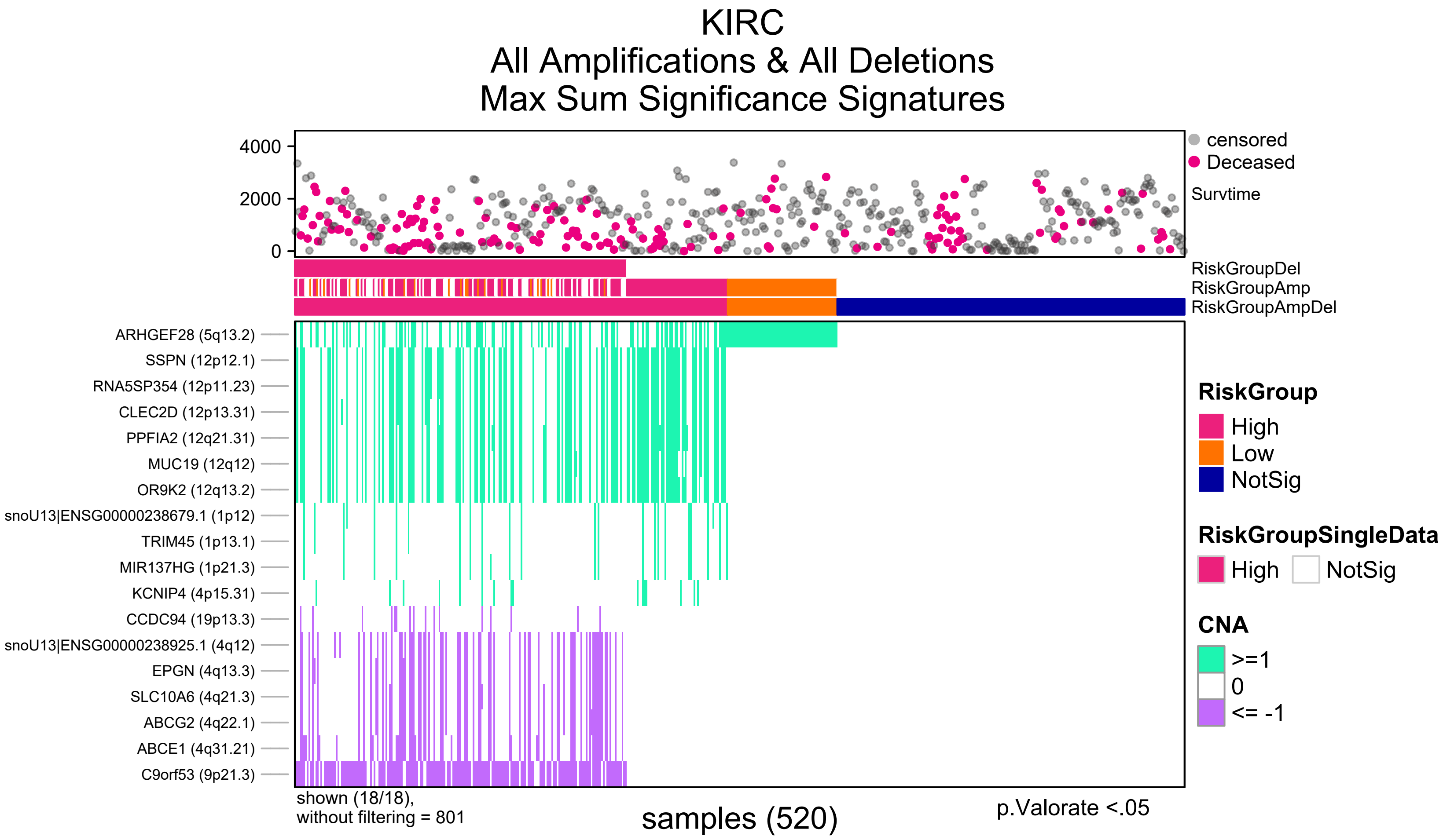
KIRC
All Amplifications & All Deletions
Max Sum Significance Signatures
4000
2000
0
censored
Deceased
Survtime
RiskGroupDel
RiskGroupAmp
RiskGroupAmpDel
ARHGEF28 (5q13.2)
SSPN (12p12.1)
RNA5SP354 (12p11.23)
CLEC2D (12p13.31)
PPFIA2 (12q21.31)
MUC19 (12q12)
OR9K2 (12q13.2)
snoU13|ENSG00000238679.1 (1p12)
TRIM45 (1p13.1)
MIR137HG (1p21.3)
KCNIP4 (4p15.31)
CCDC94 (19p13.3)
snoU13|ENSG00000238925.1 (4q12)
EPGN (4q13.3)
SLC10A6 (4q21.3)
ABCG2 (4q22.1)
ABCE1 (4q31.21)
C9orf53 (9p21.3)
RiskGroup
High
Low
NotSig
RiskGroupSingleData
High
NotSig
CNA
>=1
0
<= -1
shown (18/18),
without filtering = 801
samples (520)
p.Valorate <.05

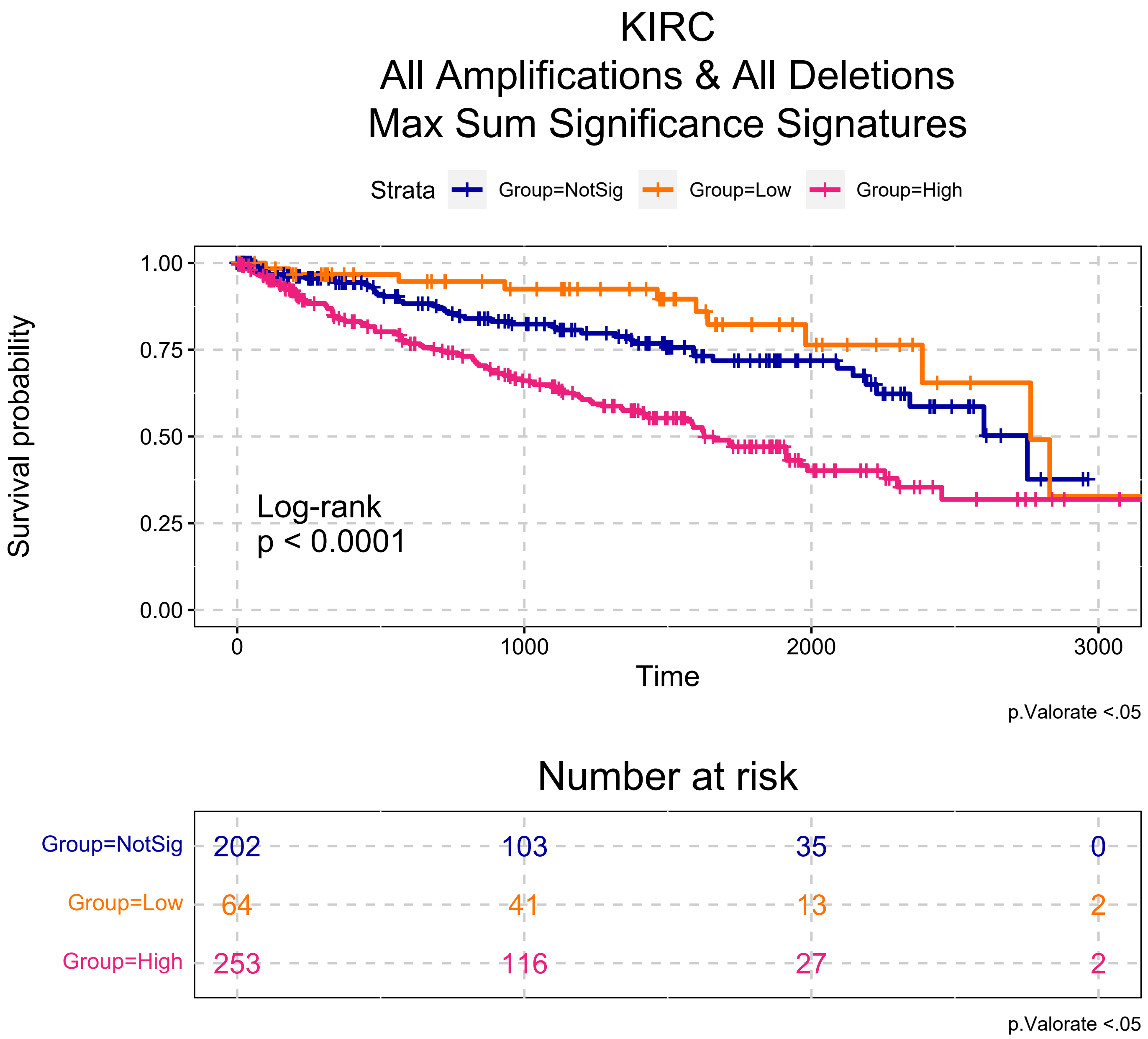

explanatory	beta	HR	L95	U95	p
Low	-0.44	0.65	0.33	1.25	0.20
High	0.75	2.11	1.48	3.01	0.00

n= 520, number of events =160
Score(logrank) test = p <.0001

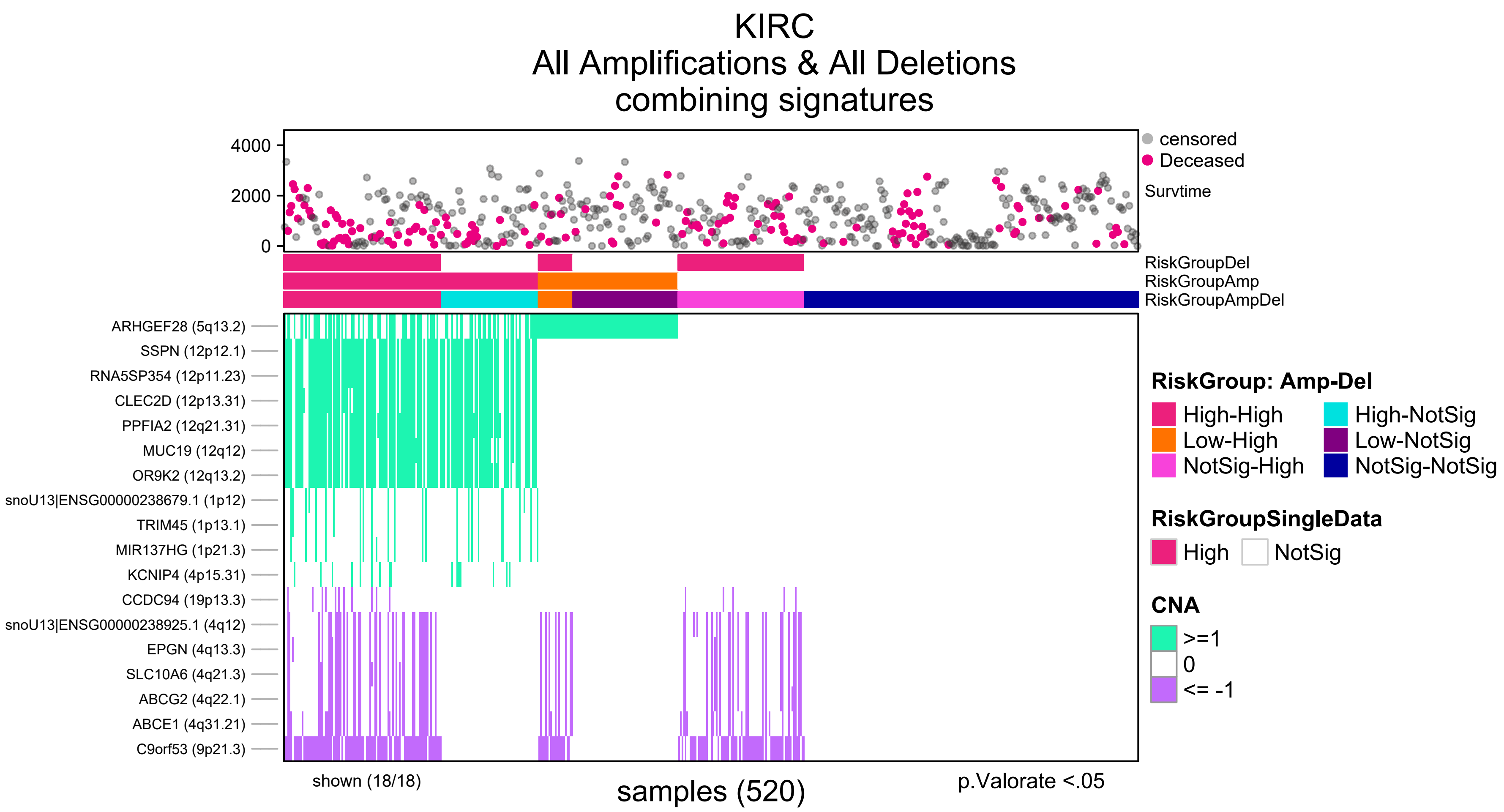
KIRC
All Amplifications & All Deletions
combining signatures
4000
2000
0
censored
Deceased
Survtime
RiskGroupDel
RiskGroupAmp
RiskGroupAmpDel
ARHGEF28 (5q13.2)
SSPN (12p12.1)
RNA5SP354 (12p11.23)
CLEC2D (12p13.31)
PPFIA2 (12q21.31)
MUC19 (12q12)
OR9K2 (12q13.2)
snoU13|ENSG00000238679.1 (1p12)
TRIM45 (1p13.1)
MIR137HG (1p21.3)
KCNIP4 (4p15.31)
CCDC94 (19p13.3)
snoU13|ENSG00000238925.1 (4q12)
EPGN (4q13.3)
SLC10A6 (4q21.3)
ABCG2 (4q22.1)
ABCE1 (4q31.21)
C9orf53 (9p21.3)
RiskGroup: Amp-Del
High-High
Low-High
NotSig-High
High-NotSig
Low-NotSig
NotSig-NotSig
RiskGroupSingleData
High
NotSig
CNA
>=1
0
<= -1
shown (18/18)
samples (520)
p.Valorate <.05

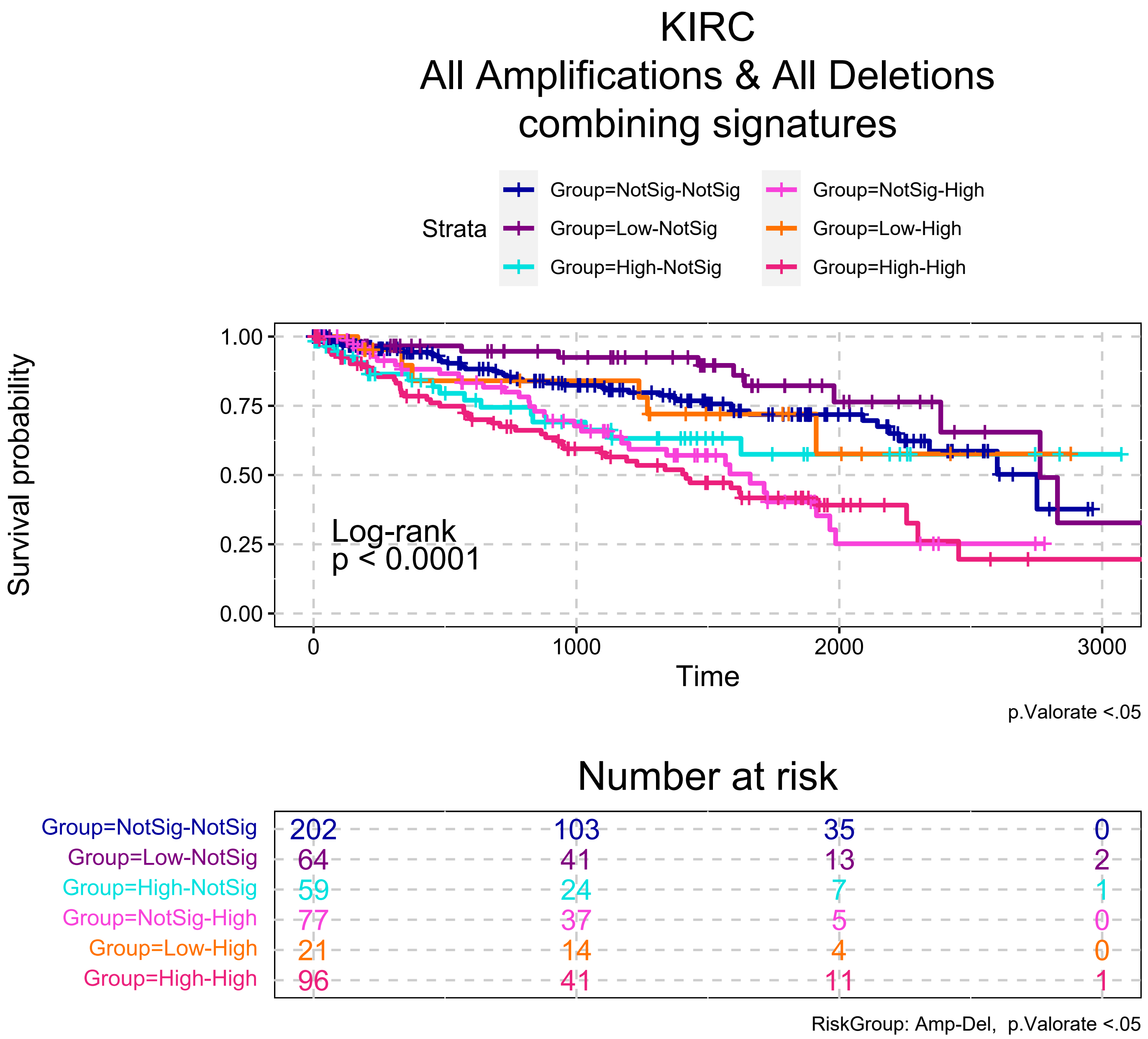

explanatory	beta	HR	L95	U95	p
Low-NotSig	-0.44	0.64	0.33	1.25	0.19
High-NotSig	0.46	1.58	0.90	2.78	0.11
NotSig-High	0.79	2.21	1.41	3.48	0.00
Low-High	0.11	1.11	0.47	2.62	0.80
High-High	0.97	2.63	1.75	3.95	0.00

n= 520, number of events =160
Score(logrank) test = p <.0001

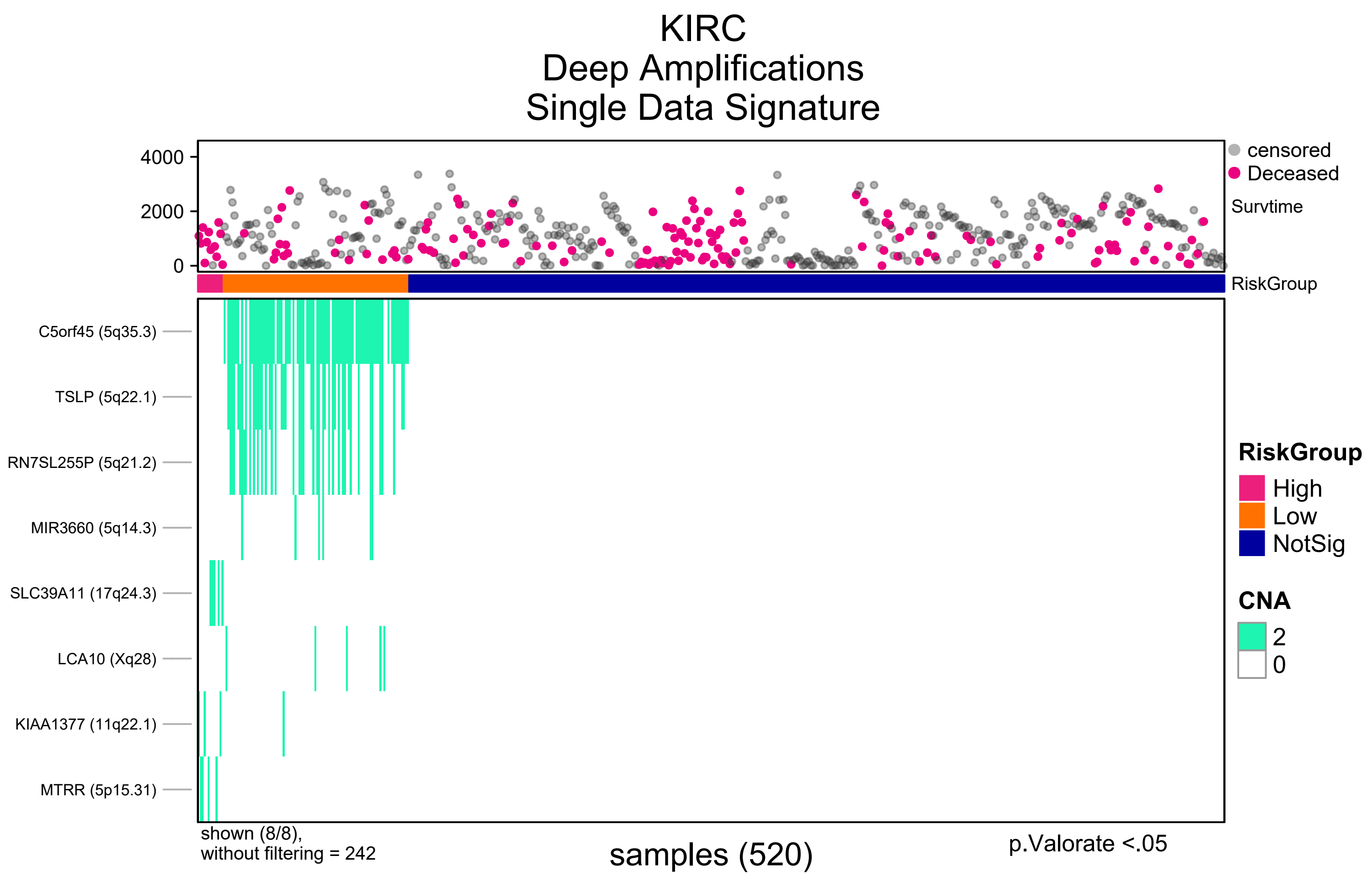
KIRC
Deep Amplifications
Single Data Signature
4000
2000
0
censored
Deceased
Survtime
RiskGroup
C5orf45 (5q35.3)
TSLP (5q22.1)
RN7SL255P (5q21.2)
MIR3660 (5q14.3)
SLC39A11 (17q24.3)
LCA10 (Xq28)
KIAA1377 (11q22.1)
MTRR (5p15.31)
RiskGroup
High
Low
NotSig
CNA
2
0
shown (8/8),
without filtering = 242
samples (520)
p.Valorate <.05

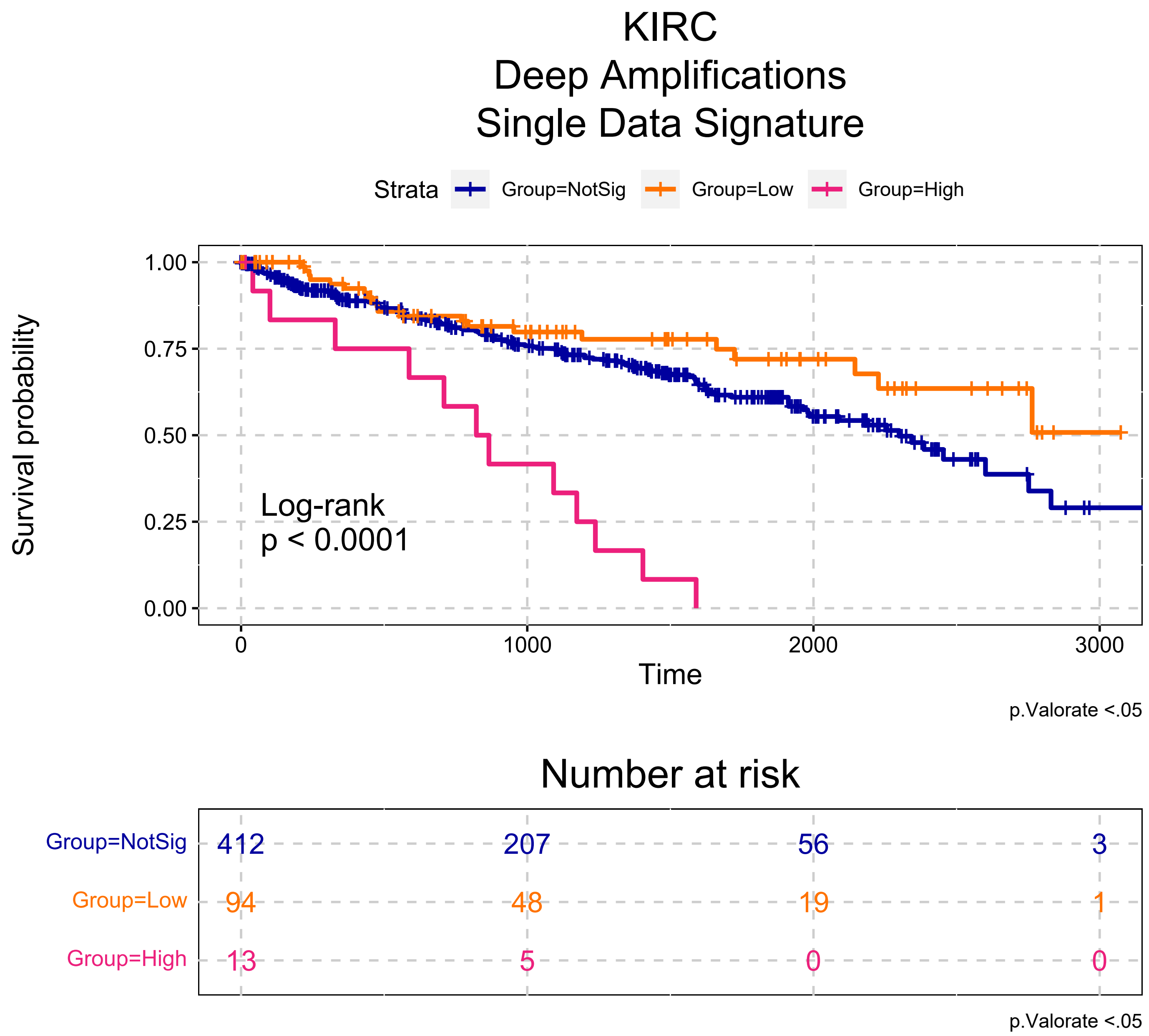

explanatory	beta	HR	L95	U95	p
Low	-0.44	0.64	0.41	1.03	0.06
High	1.48	4.41	2.42	8.03	0.00

n= 520, number of events =160
Score(logrank) test = p <.0001

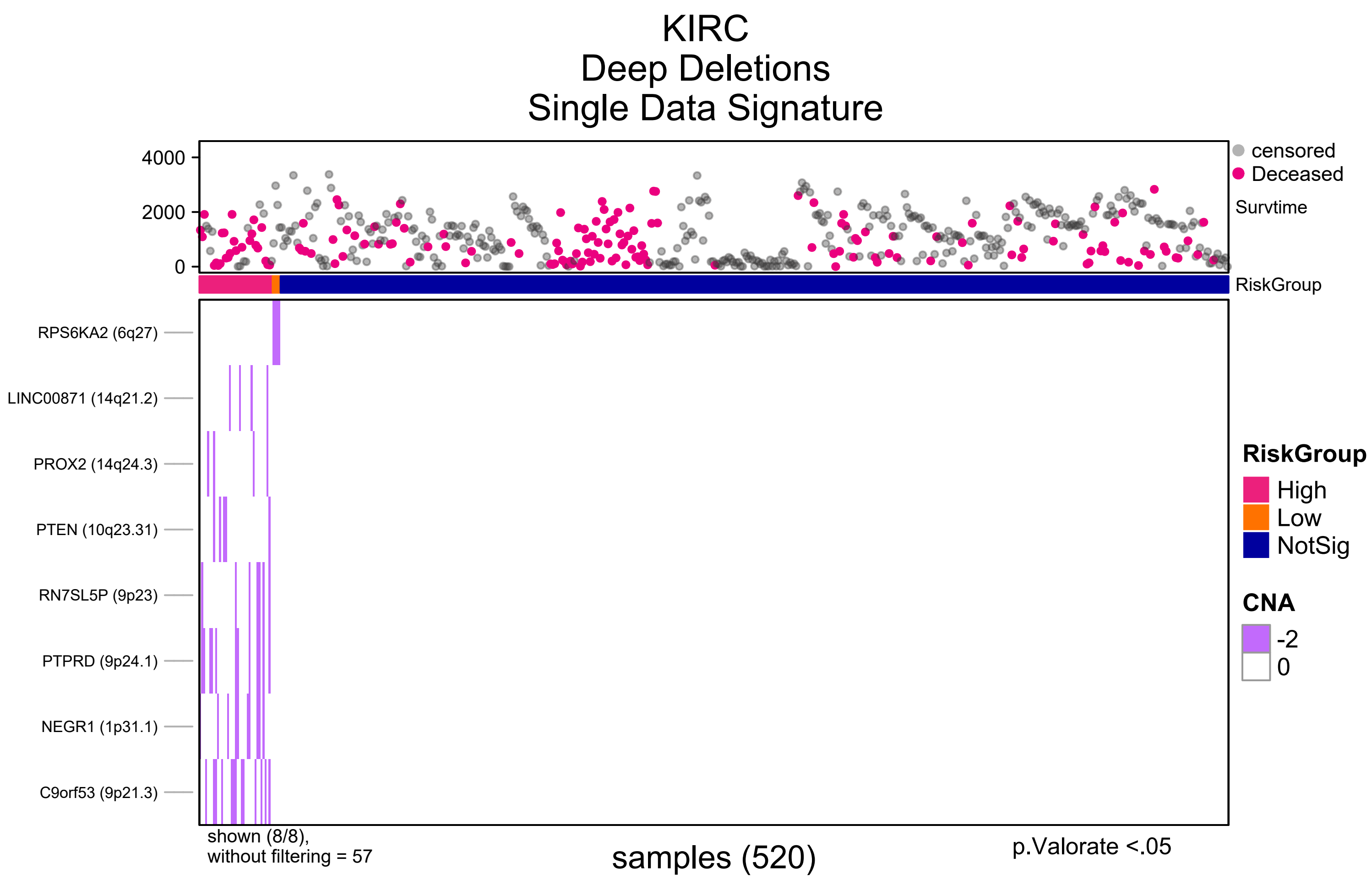

KIRC
Deep Deletions
Single Data Signature
4000
2000
0
censored
Deceased
Survtime
RiskGroup
RPS6KA2 (6q27)
LINC00871 (14q21.2)
PROX2 (14q24.3)
PTEN (10q23.31)
RN7SL5P (9p23)
PTPRD (9p24.1)
NEGR1 (1p31.1)
C9orf53 (9p21.3)
RiskGroup
High
Low
NotSig
CNA
-2
0
shown (8/8),
without filtering = 57
samples (520)
p.Valorate <.05

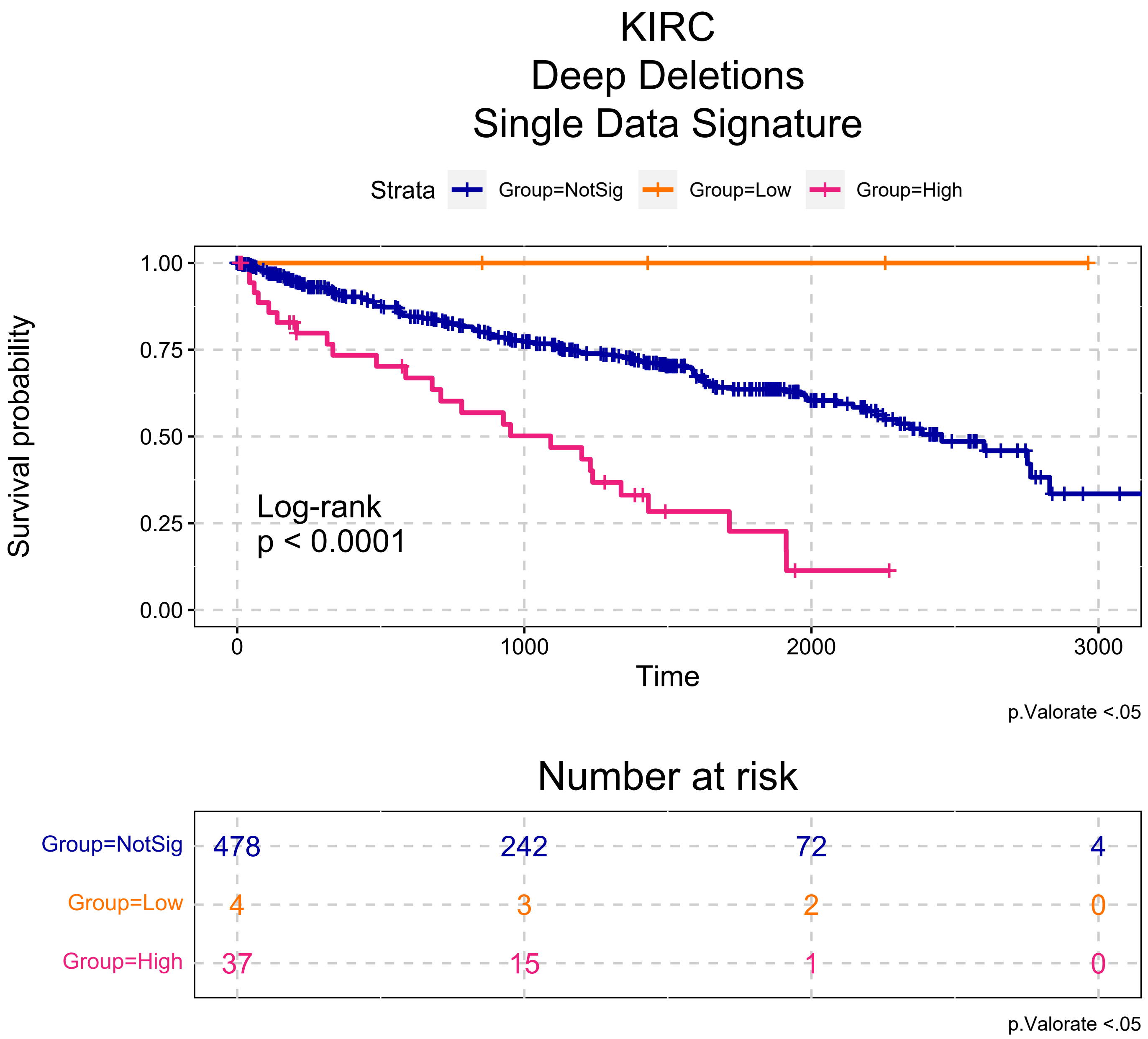

explanatory	beta	HR	L95	U95	p
Low	-16.13	0.00	0.00	Inf	0.99
High	1.20	3.31	2.15	5.09	0.00

n= 520, number of events =160
Score(logrank) test = p <.0001

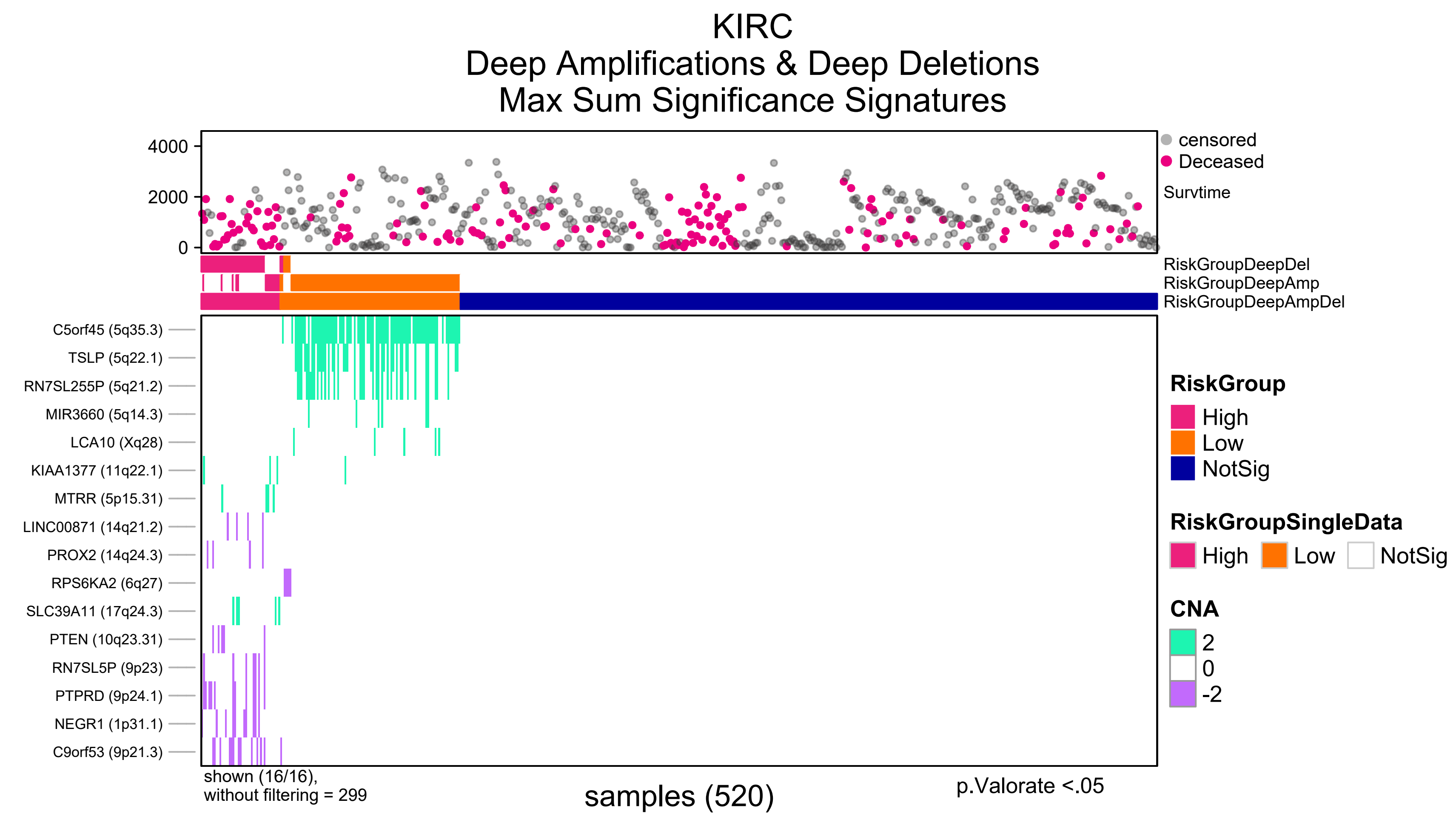
KIRC
Deep Amplifications & Deep Deletions
Max Sum Significance Signatures
4000
2000
0
censored
Deceased
Survtime
RiskGroupDeepDel
RiskGroupDeepAmp
RiskGroupDeepAmpDel
C5orf45 (5q35.3)
TSLP (5q22.1)
RN7SL255P (5q21.2)
MIR3660 (5q14.3)
LCA10 (Xq28)
KIAA1377 (11q22.1)
MTRR (5p15.31)
LINC00871 (14q21.2)
PROX2 (14q24.3)
RPS6KA2 (6q27)
SLC39A11 (17q24.3)
PTEN (10q23.31)
RN7SL5P (9p23)
PTPRD (9p24.1)
NEGR1 (1p31.1)
C9orf53 (9p21.3)
RiskGroup
High
Low
NotSig
RiskGroupSingleData
High
Low
NotSig
CNA
2
0
-2
shown (16/16),
without filtering = 299
samples (520)
p.Valorate <.05

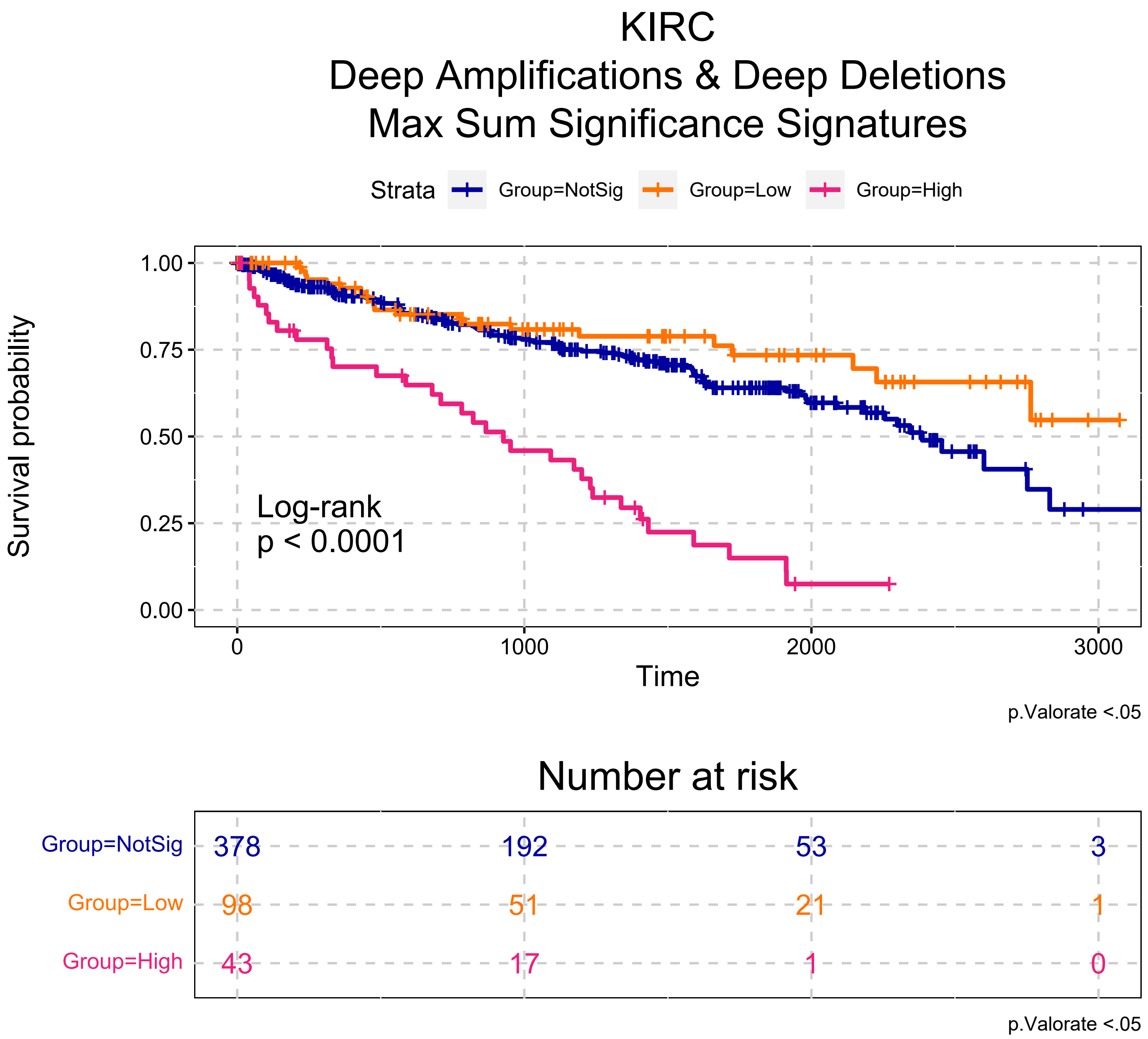

explanatory	beta	HR	L95	U95	p
Low	-0.42	0.66	0.41	1.05	0.08
High	1.33	3.79	2.55	5.63	0.00

n= 520, number of events =160
Score(logrank) test = p <.0001

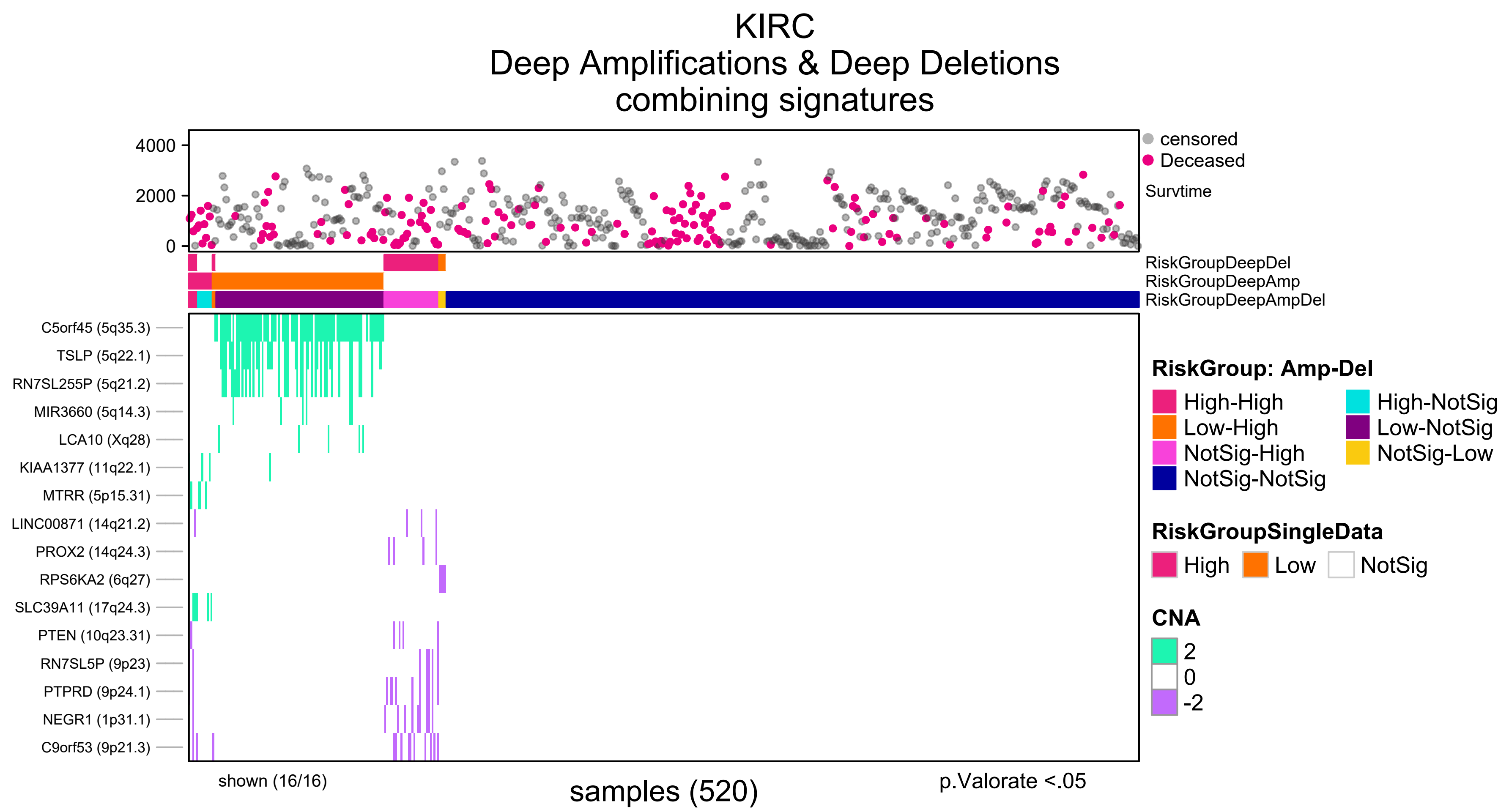
KIRC
Deep Amplifications & Deep Deletions
combining signatures
4000
2000
0
censored
Deceased
Survtime
RiskGroupDeepDel
RiskGroupDeepAmp
RiskGroupDeepAmpDel
C5orf45 (5q35.3)
TSLP (5q22.1)
RN7SL255P (5q21.2)
MIR3660 (5q14.3)
LCA10 (Xq28)
KIAA1377 (11q22.1)
MTRR (5p15.31)
LINC00871 (14q21.2)
PROX2 (14q24.3)
RPS6KA2 (6q27)
SLC39A11 (17q24.3)
PTEN (10q23.31)
RN7SL5P (9p23)
PTPRD (9p24.1)
NEGR1 (1p31.1)
C9orf53 (9p21.3)
RiskGroup: Amp-Del
High-High
Low-High
NotSig-High
NotSig-NotSig
High-NotSig
Low-NotSig
NotSig-Low
RiskGroupSingleData
High
Low
NotSig
CNA
2
0
-2
shown (16/16)
samples (520)
p.Valorate <.05

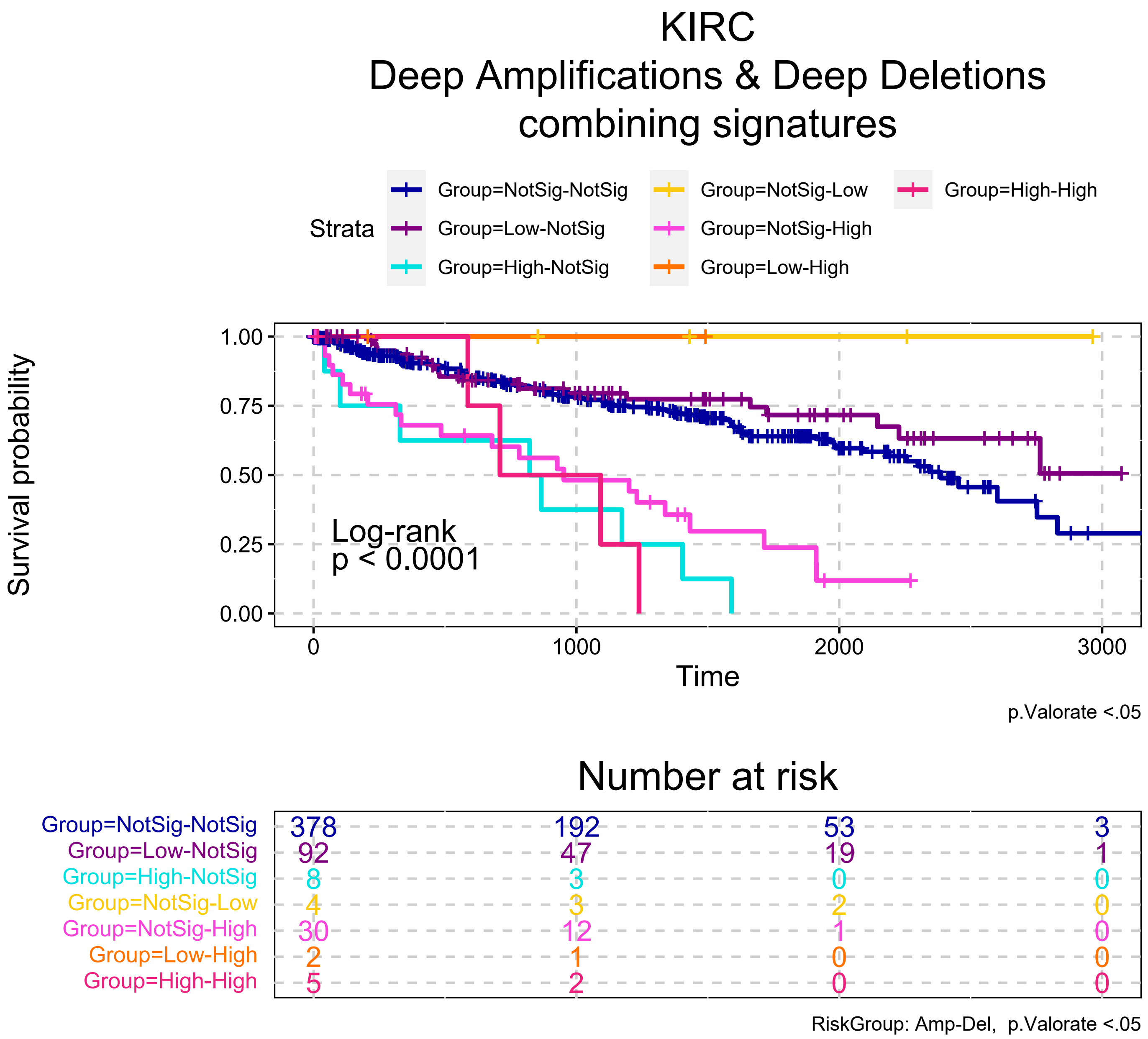

explanatory	beta	HR	L95	U95	p
Low-NotSig	-0.33	0.72	0.45	1.15	0.17
High-NotSig	1.66	5.24	2.54	10.82	0.00
NotSig-Low	-15.55	0.00	0.00	Inf	0.99
NotSig-High	1.21	3.35	2.09	5.37	0.00
Low-High	-15.48	0.00	0.00	Inf	1.00
High-High	1.51	4.51	1.65	12.33	0.00

n= 520, number of events =160
Score(logrank) test = p <.0001
