# Supplementary material for: Systematic Modeling of Risk-Associated Copy Number Alterations in Cancer
Source: Int J Mol Sci. 2024 Sep 27;25(19):10455. doi: 10.3390/ijms251910455 (PMC11477427; doi:10.3390/ijms251910455)
Supplement: Supplementary file 1 [file ijms-25-10455-s001.zip › KIRPSignatureV12-sinSombreado.pdf]

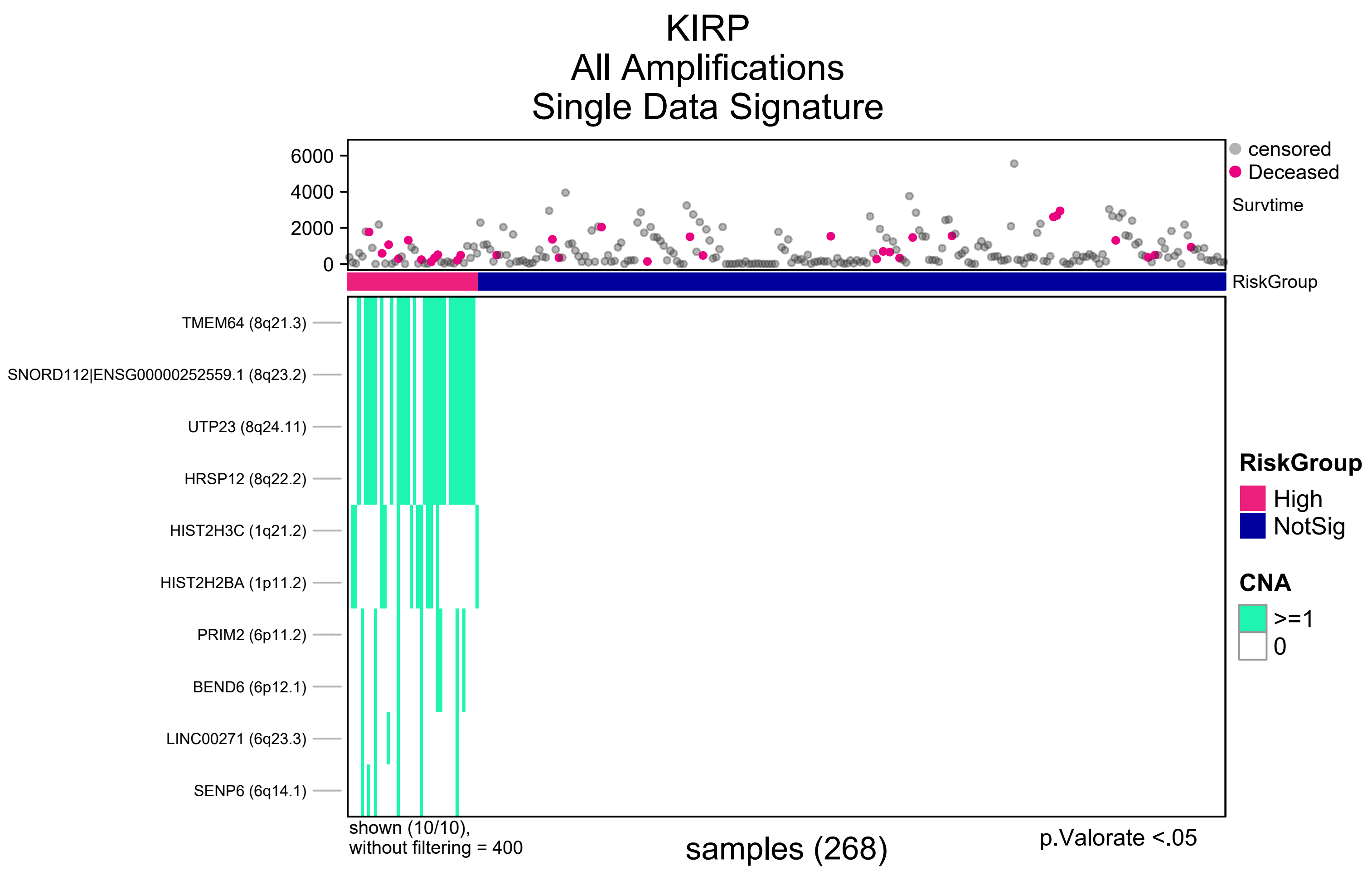

KIRP
All Amplifications
Single Data Signature
6000
4000
2000
0
censored
Deceased
Survtime
RiskGroup
TMEM64 (8q21.3)
SNORD112|ENSG00000252559.1 (8q23.2)
UTP23 (8q24.11)
HRSP12 (8q22.2)
HIST2H3C (1q21.2)
HIST2H2BA (1p11.2)
PRIM2 (6p11.2)
BEND6 (6p12.1)
LINC00271 (6q23.3)
SENP6 (6q14.1)
RiskGroup
High
NotSig
CNA
>=1
0
shown (10/10),
without filtering = 400
samples (268)
p.Valorate <.05

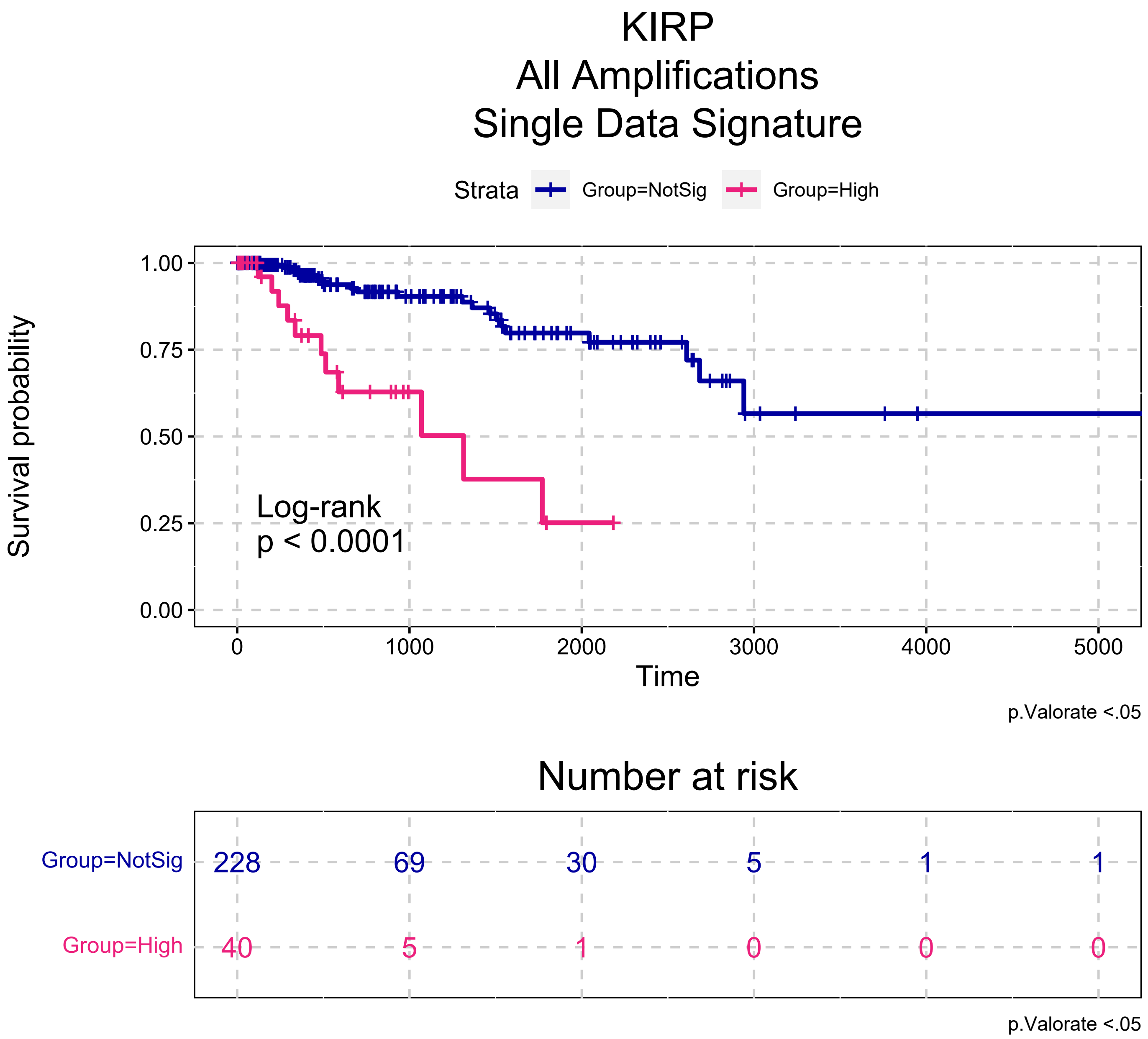

explanatory	beta	HR	L95	U95	p
High	1.74	5.69	2.66	12.20	0.00

n= 268, number of events =32
Score(logrank) test = p <.0001

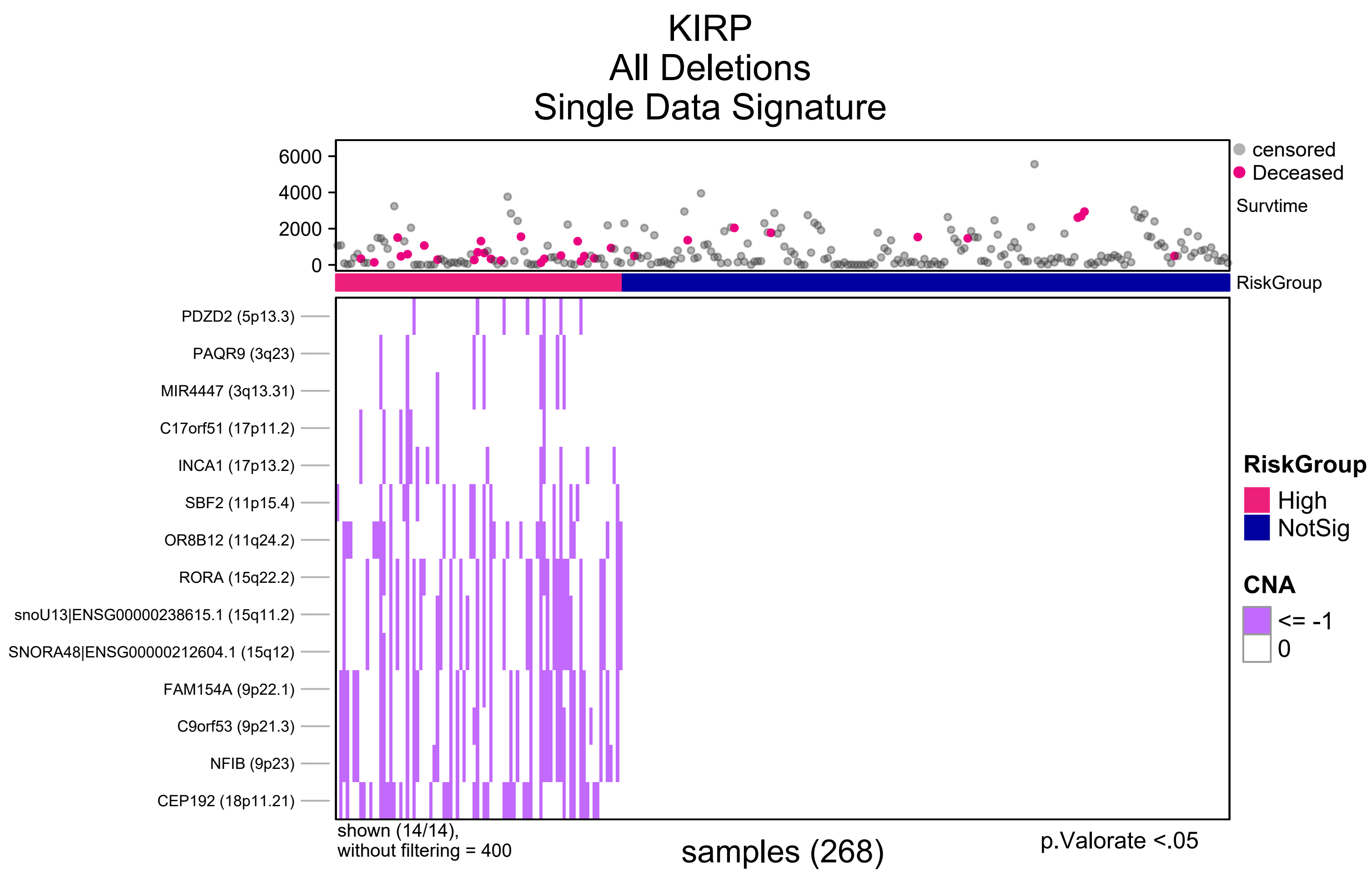
KIRP
All Deletions
Single Data Signature
6000
4000
2000
0
censored
Deceased
Survtime
RiskGroup
PDZD2 (5p13.3)
PAQR9 (3q23)
MIR4447 (3q13.31)
C17orf51 (17p11.2)
INCA1 (17p13.2)
SBF2 (11p15.4)
OR8B12 (11q24.2)
RORA (15q22.2)
snoU13|ENSG00000238615.1 (15q11.2)
SNORA48|ENSG00000212604.1 (15q12)
FAM154A (9p22.1)
C9orf53 (9p21.3)
NFIB (9p23)
CEP192 (18p11.21)
RiskGroup
High
NotSig
CNA
<= -1
0
shown (14/14),
without filtering = 400
samples (268)
p.Valorate <.05

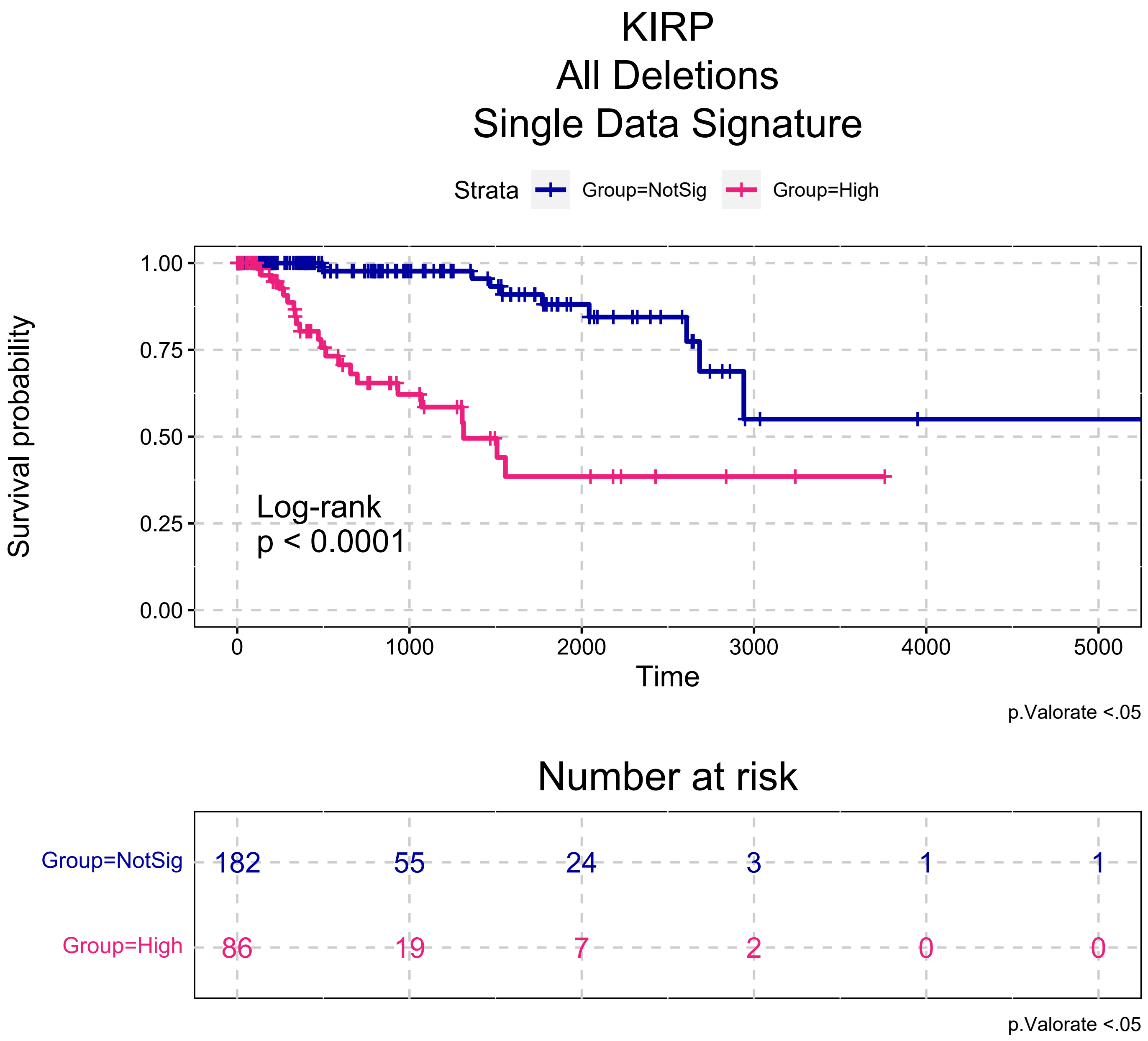

explanatory	beta	HR	L95	U95	p
High	1.91	6.75	3.18	14.32	0.00

n= 268, number of events =32
Score(logrank) test = p <.0001

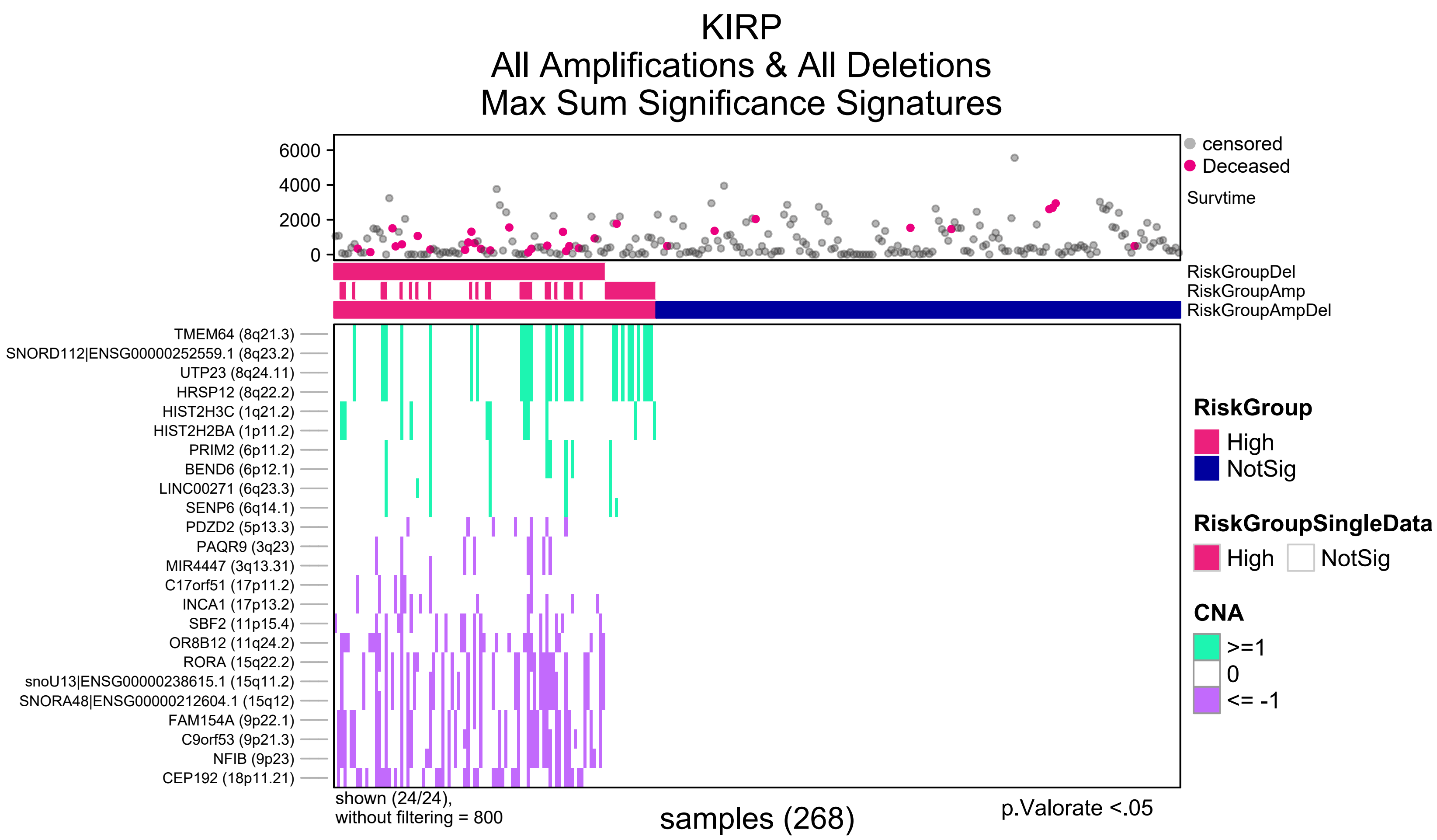

KIRP
All Amplifications & All Deletions
Max Sum Significance Signatures
6000
4000
2000
0
censored
Deceased
Survtime
RiskGroupDel
RiskGroupAmp
RiskGroupAmpDel
TMEM64 (8q21.3)
SNORD112|ENSG00000252559.1 (8q23.2)
UTP23 (8q24.11)
HRSP12 (8q22.2)
HIST2H3C (1q21.2)
HIST2H2BA (1p11.2)
PRIM2 (6p11.2)
BEND6 (6p12.1)
LINC00271 (6q23.3)
SENP6 (6q14.1)
PDZD2 (5p13.3)
PAQR9 (3q23)
MIR4447 (3q13.31)
C17orf51 (17p11.2)
INCA1 (17p13.2)
SBF2 (11p15.4)
OR8B12 (11q24.2)
RORA (15q22.2)
snoU13|ENSG00000238615.1 (15q11.2)
SNORA48|ENSG00000212604.1 (15q12)
FAM154A (9p22.1)
C9orf53 (9p21.3)
NFIB (9p23)
CEP192 (18p11.21)
RiskGroup
High
NotSig
RiskGroupSingleData
High
NotSig
CNA
>=1
0
<= -1
shown (24/24),
without filtering = 800
samples (268)
p.Valorate <.05

KIRP
All Amplifications & All Deletions
Max Sum Significance Signatures

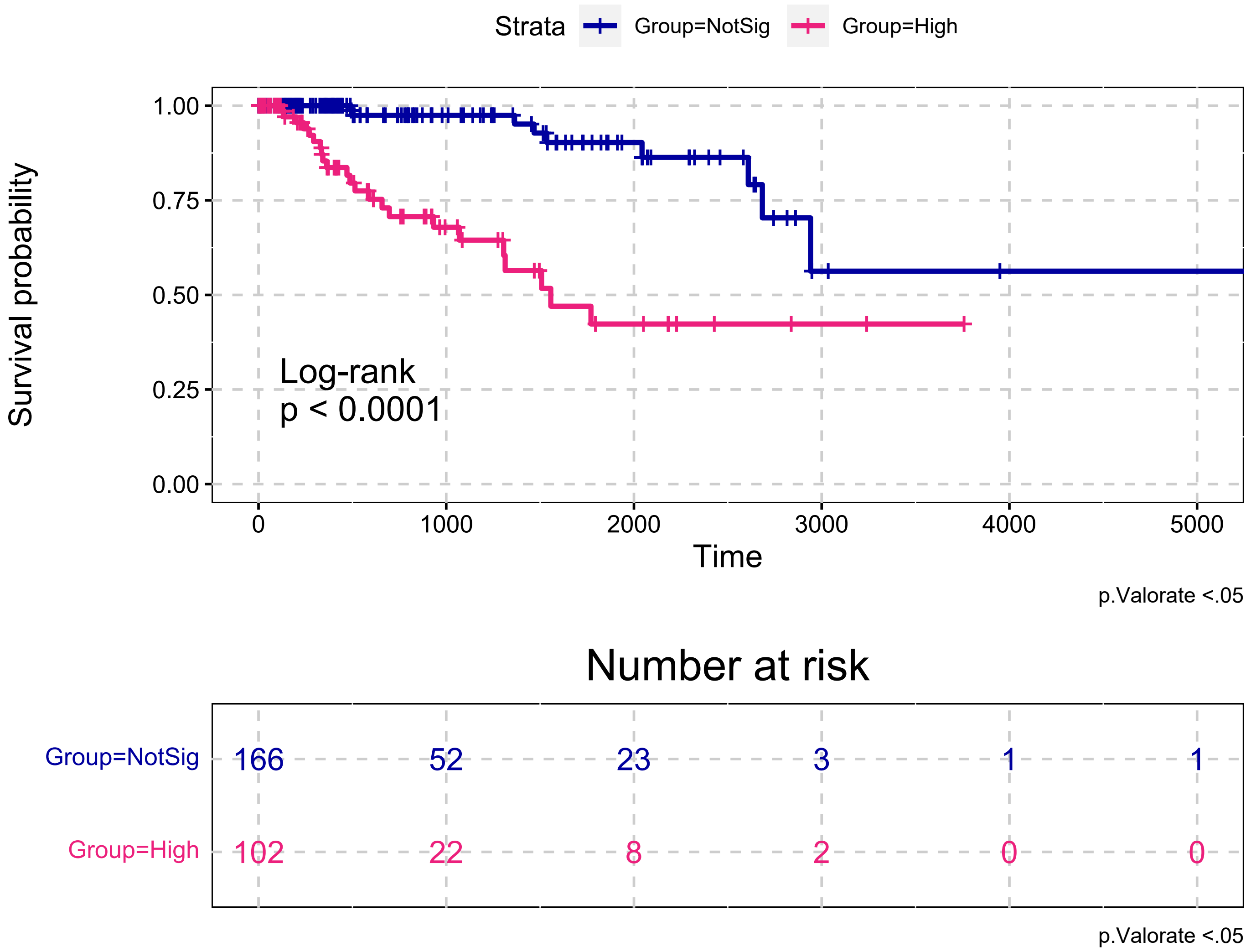

explanatory	beta	HR	L95	U95	p
High	1.79	5.99	2.76	13.00	0.00

n= 268, number of events =32
Score(logrank) test = p <.0001

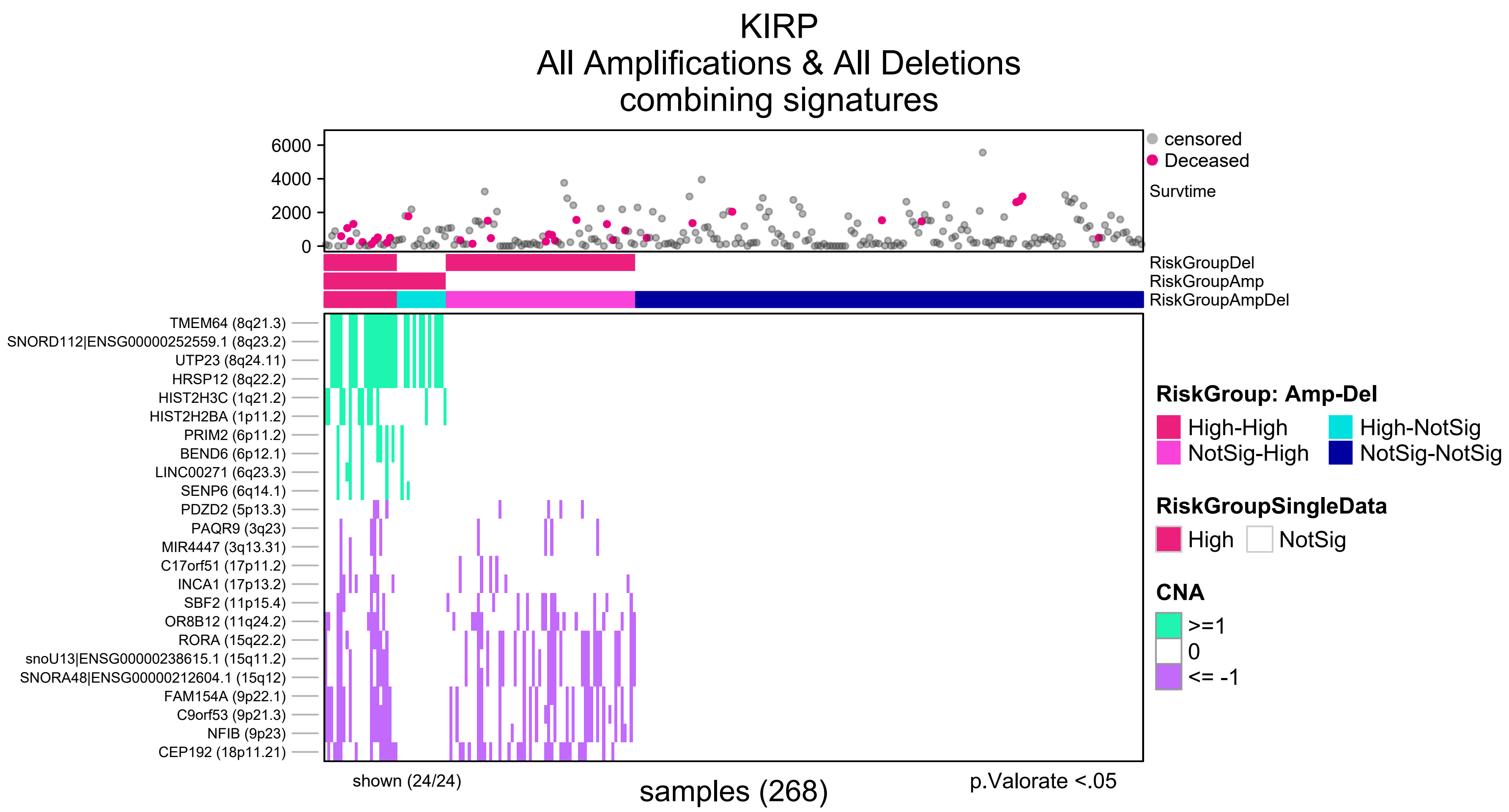
KIRP
All Amplifications & All Deletions
combining signatures
6000
4000
2000
0
censored
Deceased
Survtime
RiskGroupDel
RiskGroupAmp
RiskGroupAmpDel
TMEM64 (8q21.3)
SNORD112|ENSG00000252559.1 (8q23.2)
UTP23 (8q24.11)
HRSP12 (8q22.2)
HIST2H3C (1q21.2)
HIST2H2BA (1p11.2)
PRIM2 (6p11.2)
BEND6 (6p12.1)
LINC00271 (6q23.3)
SENP6 (6q14.1)
PDZD2 (5p13.3)
PAQR9 (3q23)
MIR4447 (3q13.31)
C17orf51 (17p11.2)
INCA1 (17p13.2)
SBF2 (11p15.4)
OR8B12 (11q24.2)
RORA (15q22.2)
snoU13|ENSG00000238615.1 (15q11.2)
SNORA48|ENSG00000212604.1 (15q12)
FAM154A (9p22.1)
C9orf53 (9p21.3)
NFIB (9p23)
CEP192 (18p11.21)
RiskGroup: Amp-Del
High-High
High-NotSig
NotSig-High
NotSig-NotSig
RiskGroupSingleData
High
NotSig
CNA
>=1
0
<= -1
shown (24/24)
samples (268)
p.Valorate <.05

KIRP
All Amplifications & All Deletions combining signatures

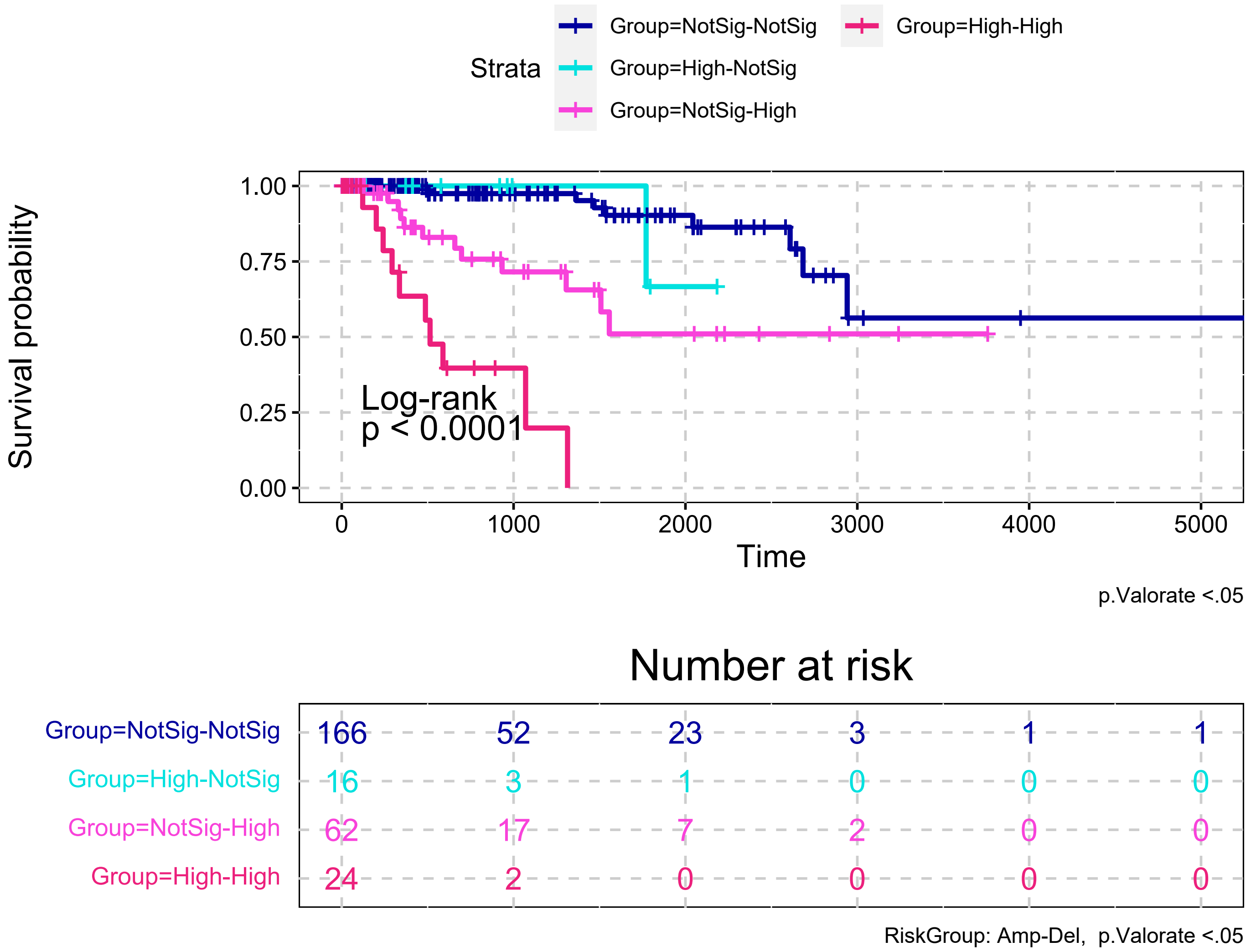

explanatory	beta	HR	L95	U95	p
High-NotSig	0.44	1.55	0.19	12.31	0.68
NotSig-High	1.51	4.52	1.90	10.75	0.00
High-High	3.34	28.36	10.38	77.46	0.00

n= 268, number of events =32
Score(logrank) test = p <.0001

KIRP
Deep Deletions
Single Data Signature

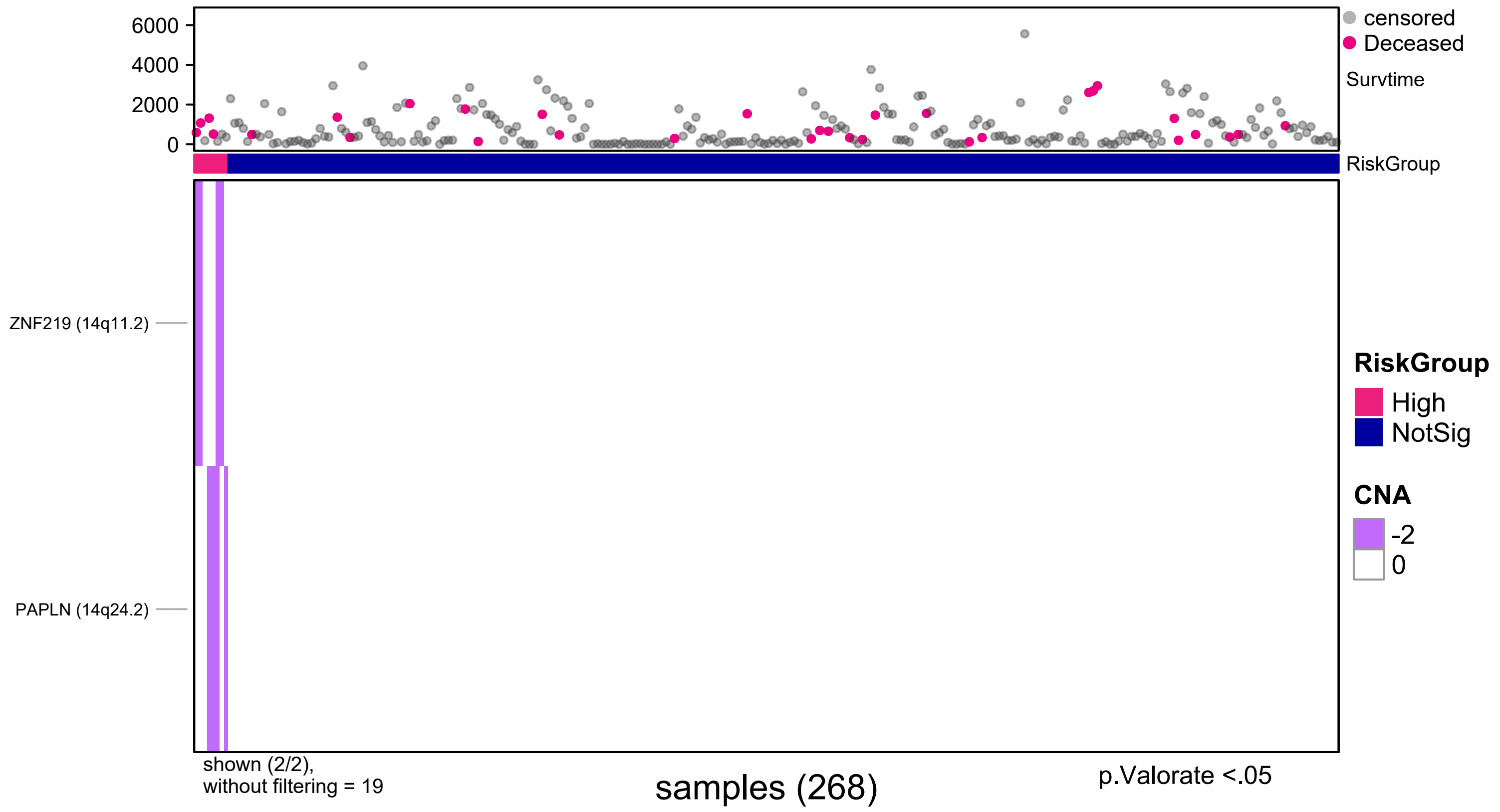

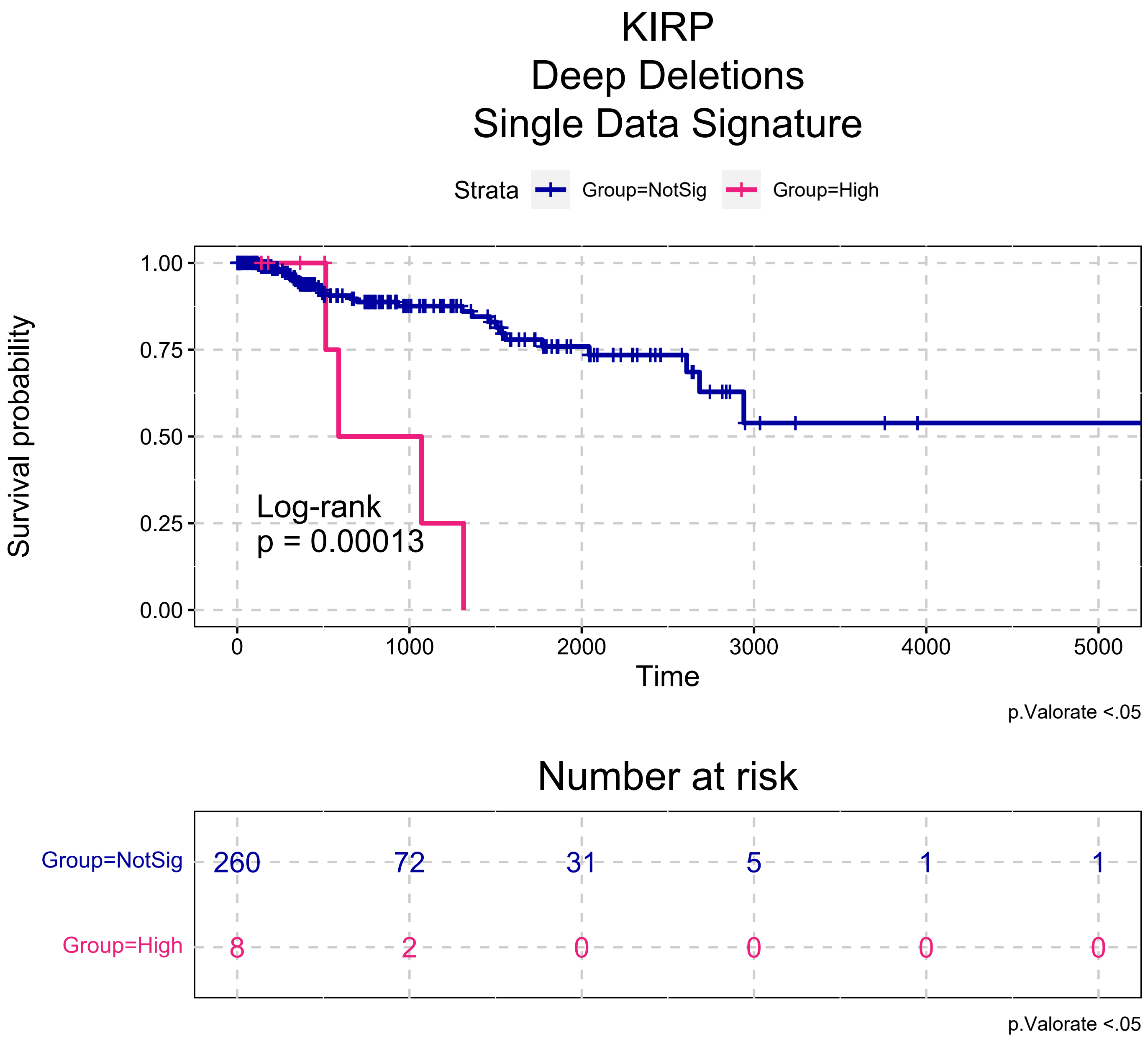

explanatory	beta	HR	L95	U95	p
High	1.85	6.39	2.15	18.99	0.00

n= 268, number of events =32
Score(logrank) test = 0
