# Supplementary material for: Systematic Modeling of Risk-Associated Copy Number Alterations in Cancer
Source: Int J Mol Sci. 2024 Sep 27;25(19):10455. doi: 10.3390/ijms251910455 (PMC11477427; doi:10.3390/ijms251910455)
Supplement: Supplementary file 1 [file ijms-25-10455-s001.zip › LAMLSignatureV12-sinSombreado.pdf]

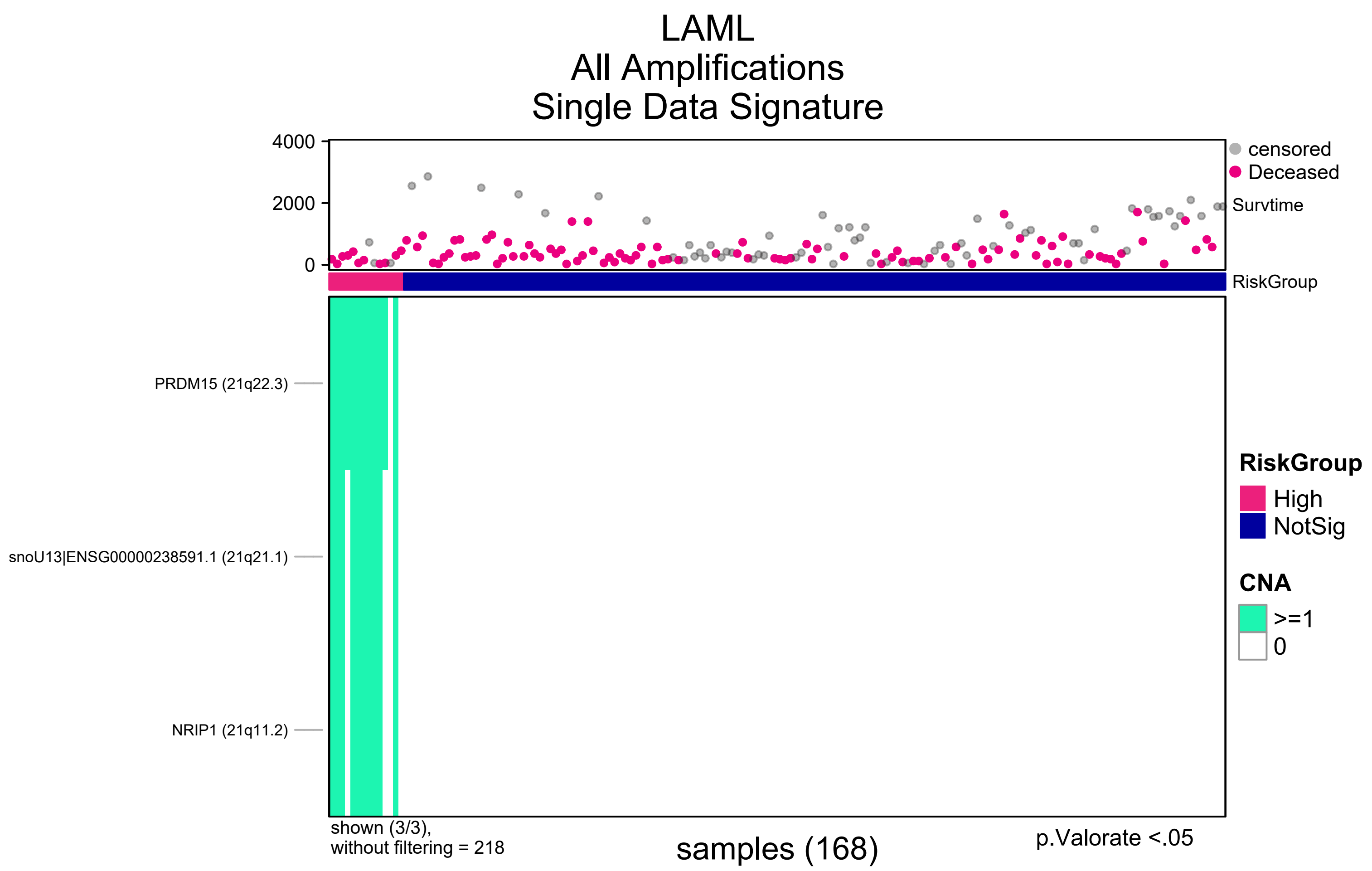

LAML
All Amplifications
Single Data Signature
4000
2000
0
censored
Deceased
Survtime
RiskGroup
PRDM15 (21q22.3)
snoU13|ENSG00000238591.1 (21q21.1)
NRIP1 (21q11.2)
RiskGroup
High
NotSig
CNA
>=1
0
shown (3/3),
without filtering = 218
samples (168)
p.Valorate <.05

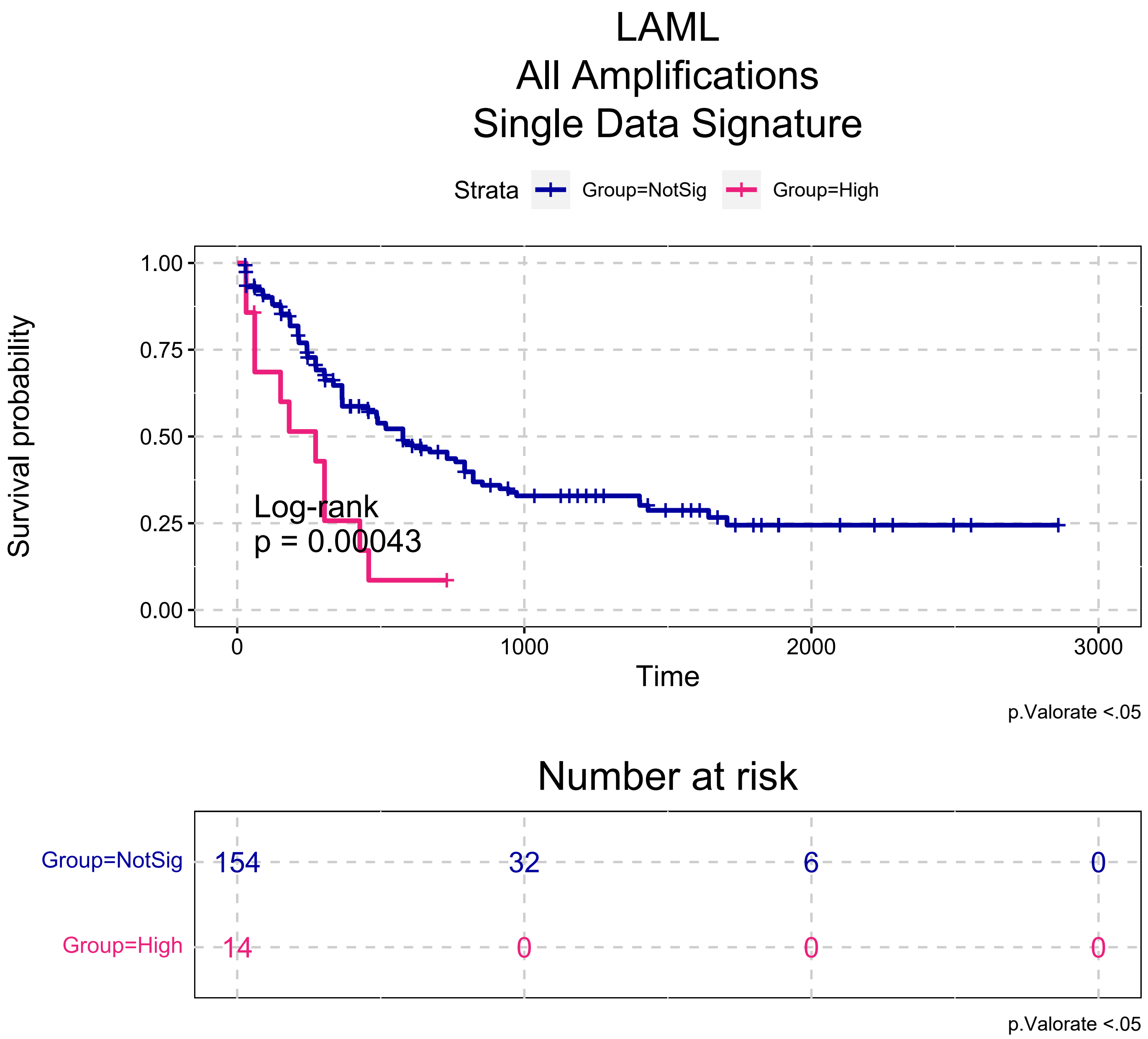

explanatory	beta	HR	L95	U95	p
High	1.10	3.01	1.59	5.71	0.00

n= 168, number of events =104
Score(logrank) test = 0

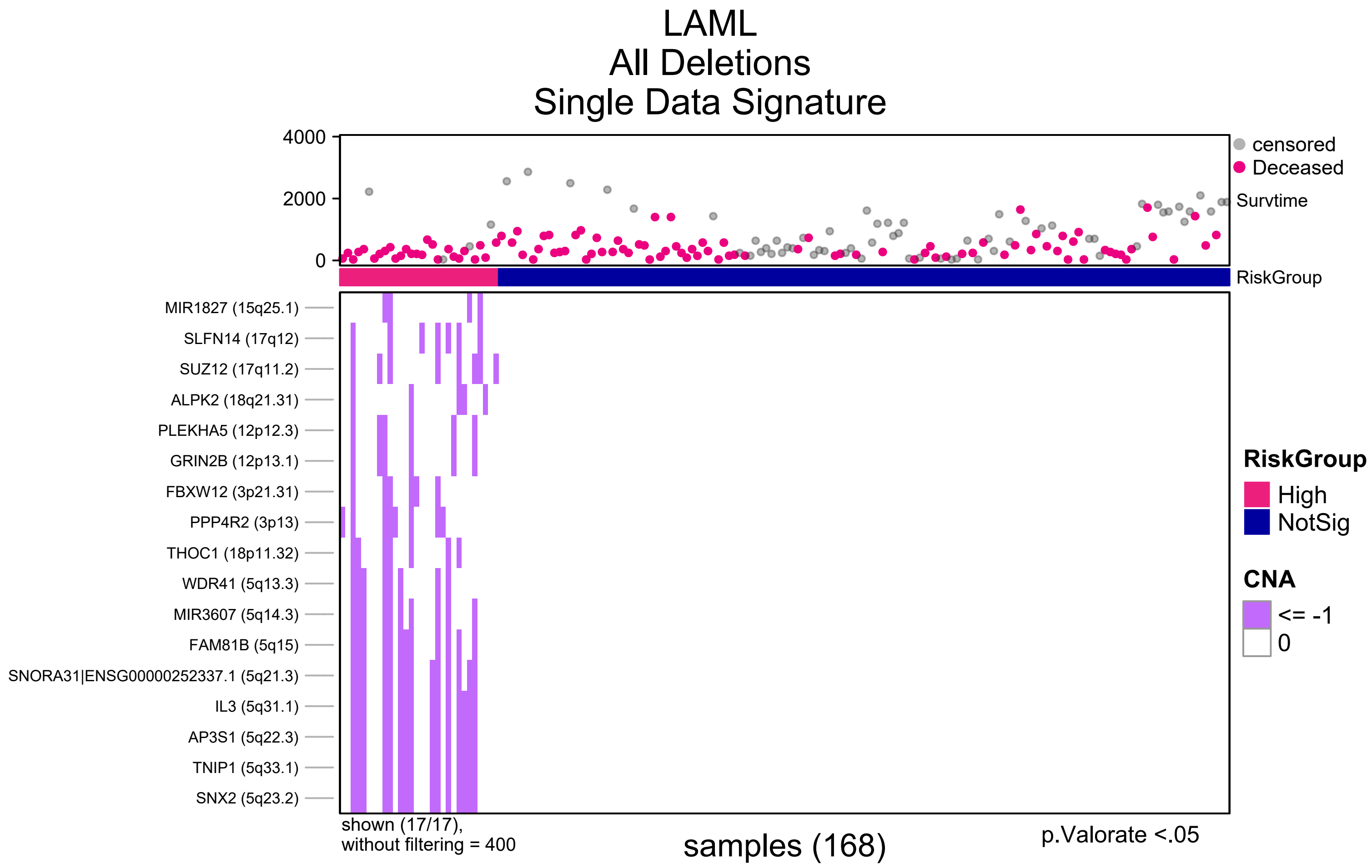

LAML
All Deletions
Single Data Signature
4000
2000
0
censored
Deceased
Survtime
RiskGroup
MIR1827 (15q25.1)
SLFN14 (17q12)
SUZ12 (17q11.2)
ALPK2 (18q21.31)
PLEKHA5 (12p12.3)
GRIN2B (12p13.1)
FBXW12 (3p21.31)
PPP4R2 (3p13)
THOC1 (18p11.32)
WDR41 (5q13.3)
MIR3607 (5q14.3)
FAM81B (5q15)
SNORA31|ENSG00000252337.1 (5q21.3)
IL3 (5q31.1)
AP3S1 (5q22.3)
TNIP1 (5q33.1)
SNX2 (5q23.2)
RiskGroup
High
NotSig
CNA
<= -1
0
shown (17/17),
without filtering = 400
samples (168)
p.Valorate <.05

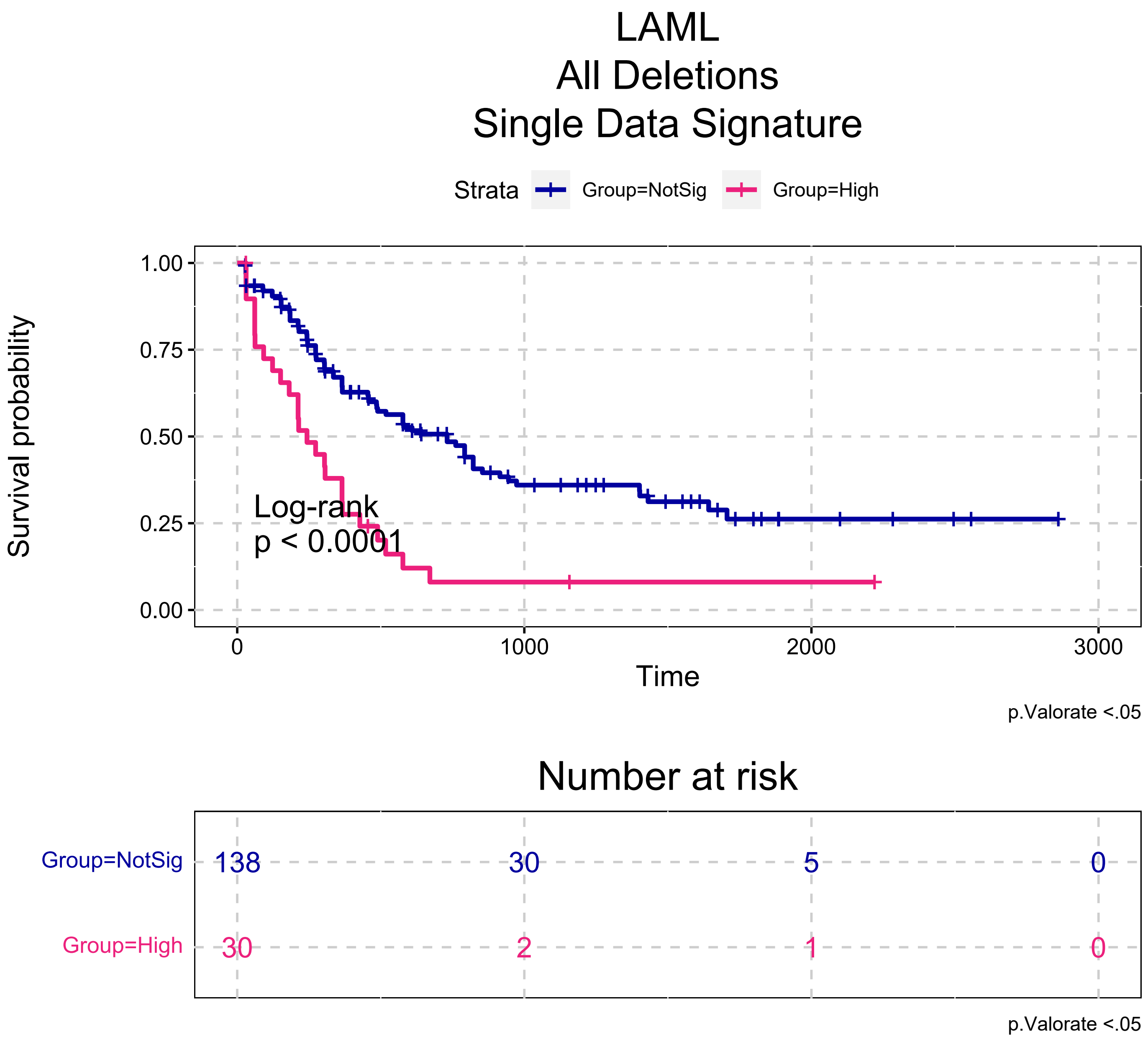

explanatory	beta	HR	L95	U95	p
High	0.98	2.65	1.69	4.17	0.00

n= 168, number of events =104
Score(logrank) test = p <.0001

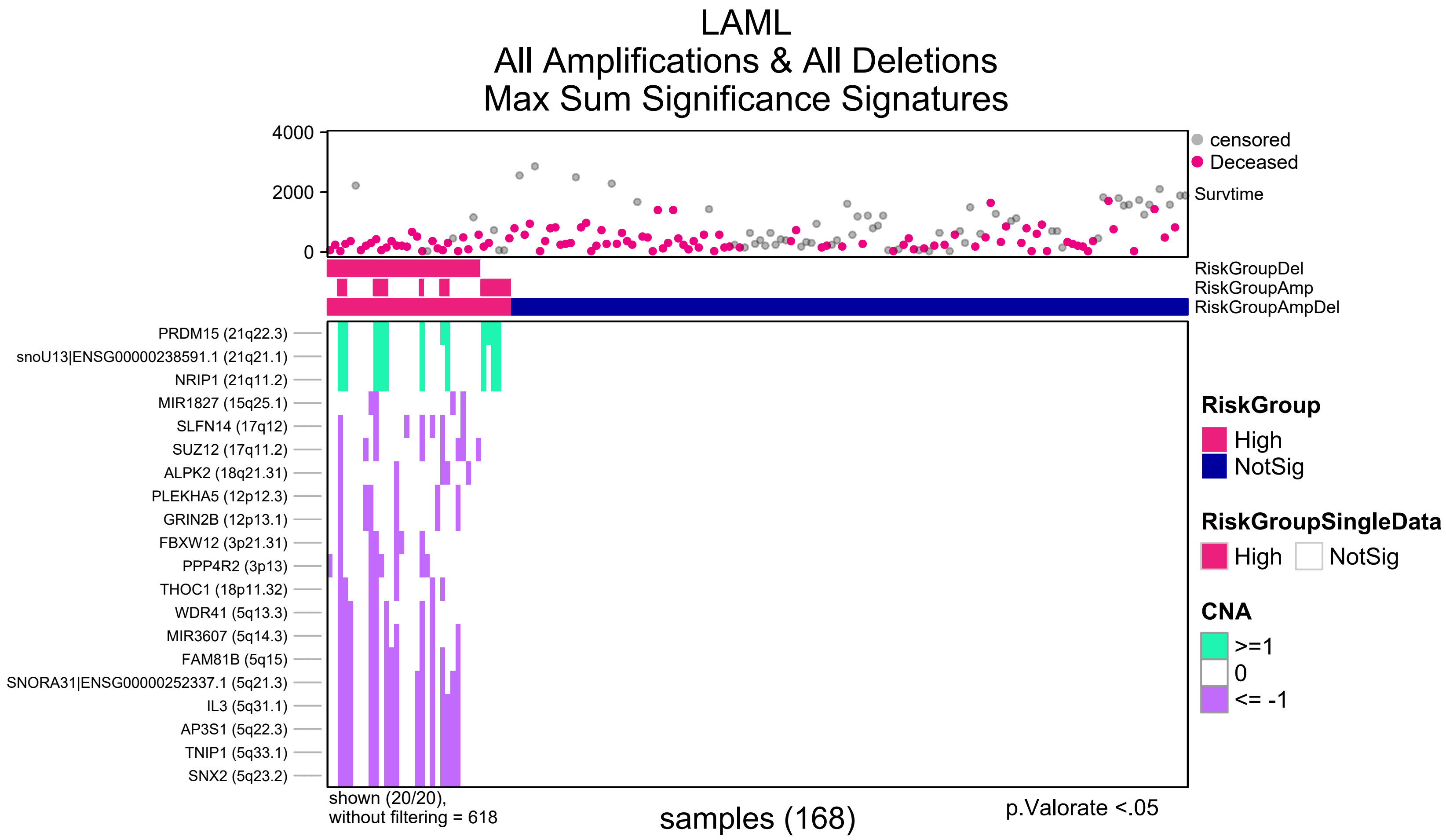
LAML
All Amplifications & All Deletions
Max Sum Significance Signatures
4000
2000
0
censored
Deceased
Survtime
RiskGroupDel
RiskGroupAmp
RiskGroupAmpDel
PRDM15 (21q22.3)
snoU13|ENSG00000238591.1 (21q21.1)
NRIP1 (21q11.2)
MIR1827 (15q25.1)
SLFN14 (17q12)
SUZ12 (17q11.2)
ALPK2 (18q21.31)
PLEKHA5 (12p12.3)
GRIN2B (12p13.1)
FBXW12 (3p21.31)
PPP4R2 (3p13)
THOC1 (18p11.32)
WDR41 (5q13.3)
MIR3607 (5q14.3)
FAM81B (5q15)
SNORA31|ENSG00000252337.1 (5q21.3)
IL3 (5q31.1)
AP3S1 (5q22.3)
TNIP1 (5q33.1)
SNX2 (5q23.2)
RiskGroup
High
NotSig
RiskGroupSingleData
High
NotSig
CNA
>=1
0
<= -1
shown (20/20),
without filtering = 618
samples (168)
p.Valorate <.05

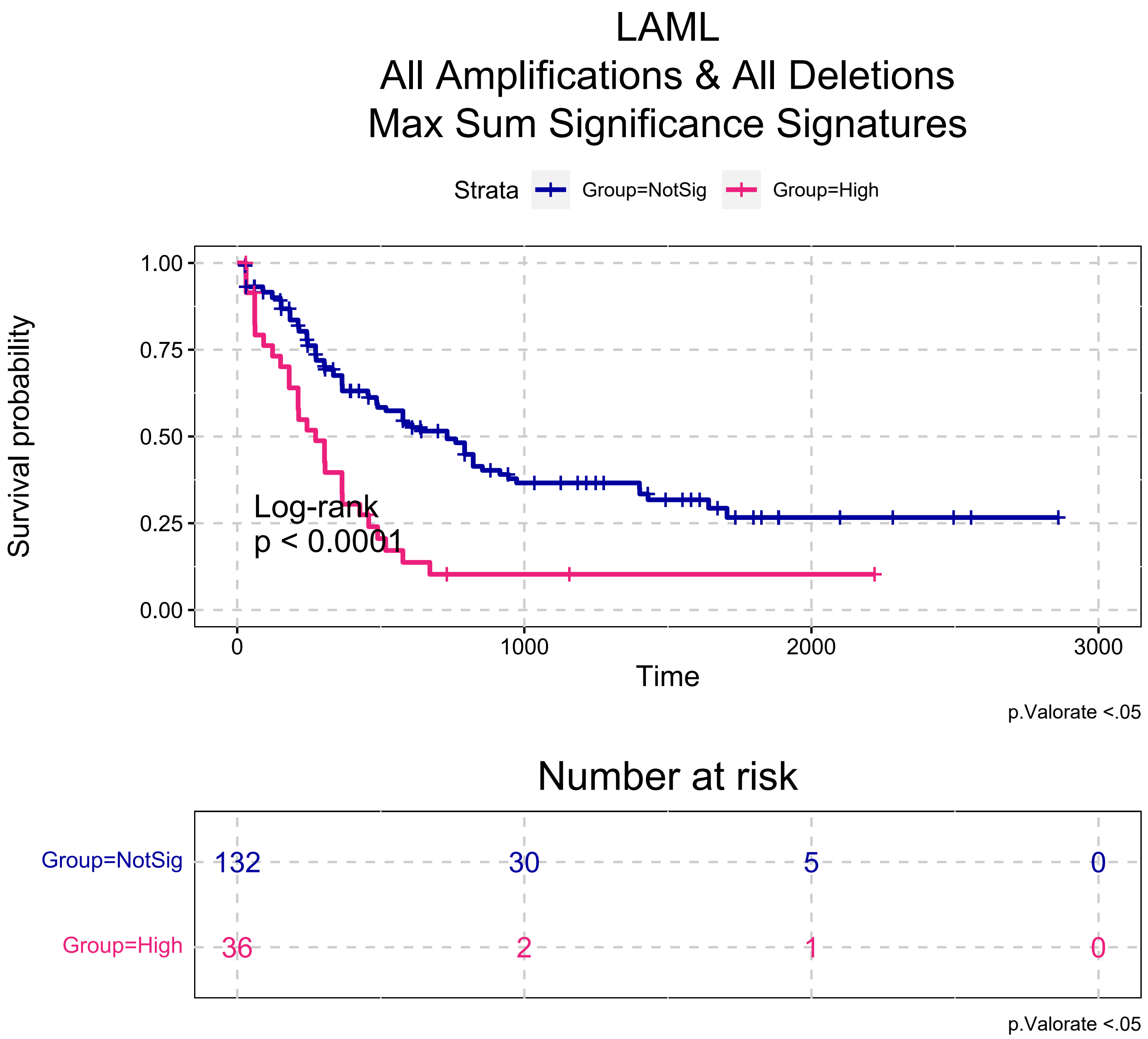

explanatory	beta	HR	L95	U95	p
High	0.93	2.54	1.64	3.94	0.00

n= 168, number of events =104
Score(logrank) test = p <.0001

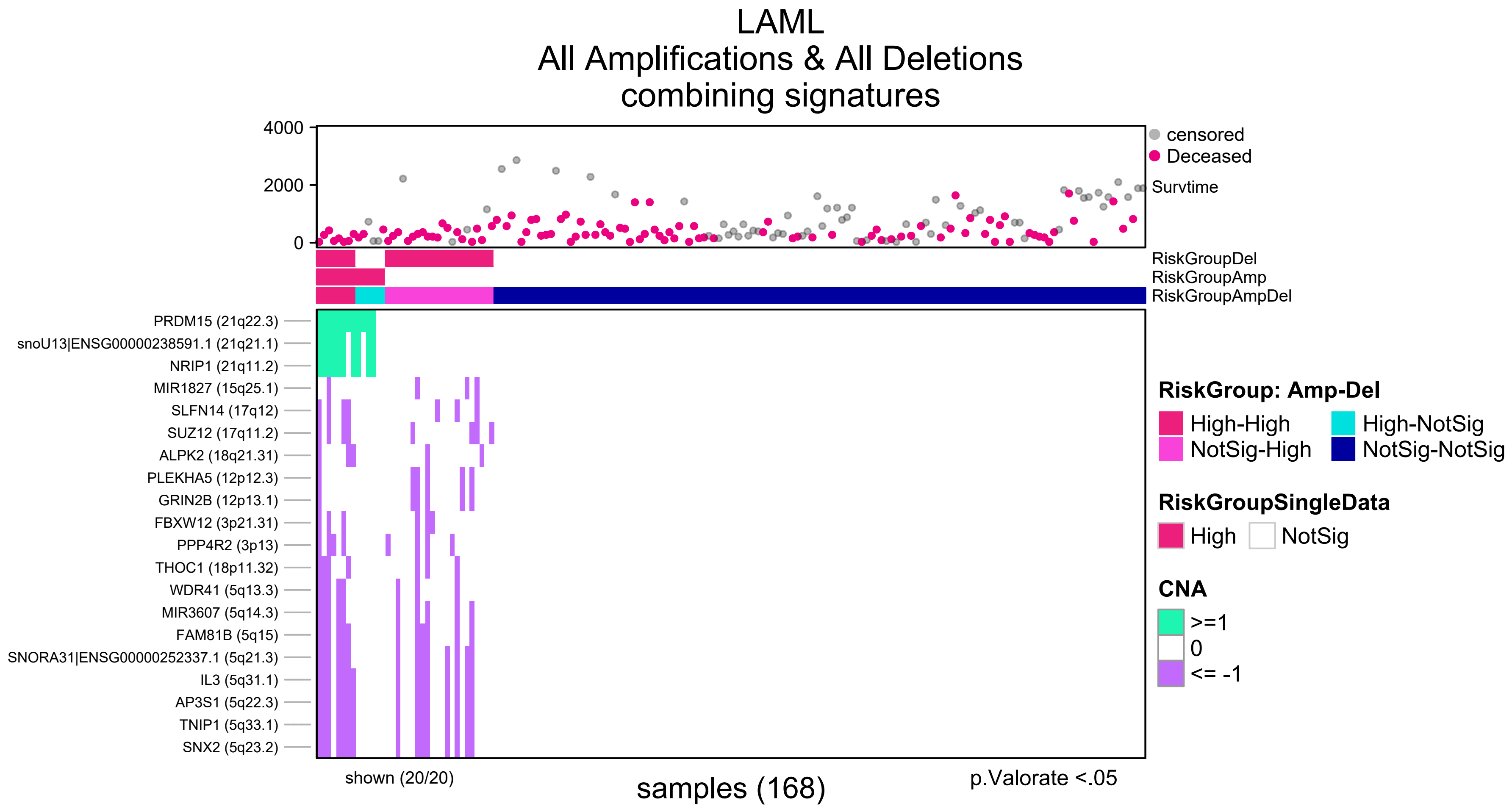
LAML
All Amplifications & All Deletions
combining signatures
4000
2000
0
censored
Deceased
Survtime
RiskGroupDel
RiskGroupAmp
RiskGroupAmpDel
PRDM15 (21q22.3)
snoU13|ENSG00000238591.1 (21q21.1)
NRIP1 (21q11.2)
MIR1827 (15q25.1)
SLFN14 (17q12)
SUZ12 (17q11.2)
ALPK2 (18q21.31)
PLEKHA5 (12p12.3)
GRIN2B (12p13.1)
FBXW12 (3p21.31)
PPP4R2 (3p13)
THOC1 (18p11.32)
WDR41 (5q13.3)
MIR3607 (5q14.3)
FAM81B (5q15)
SNORA31|ENSG00000252337.1 (5q21.3)
IL3 (5q31.1)
AP3S1 (5q22.3)
TNIP1 (5q33.1)
SNX2 (5q23.2)
RiskGroup: Amp-Del
High-High
High-NotSig
NotSig-High
NotSig-NotSig
RiskGroupSingleData
High
NotSig
CNA
>=1
0
<= -1
shown (20/20)
samples (168)
p.Valorate <.05

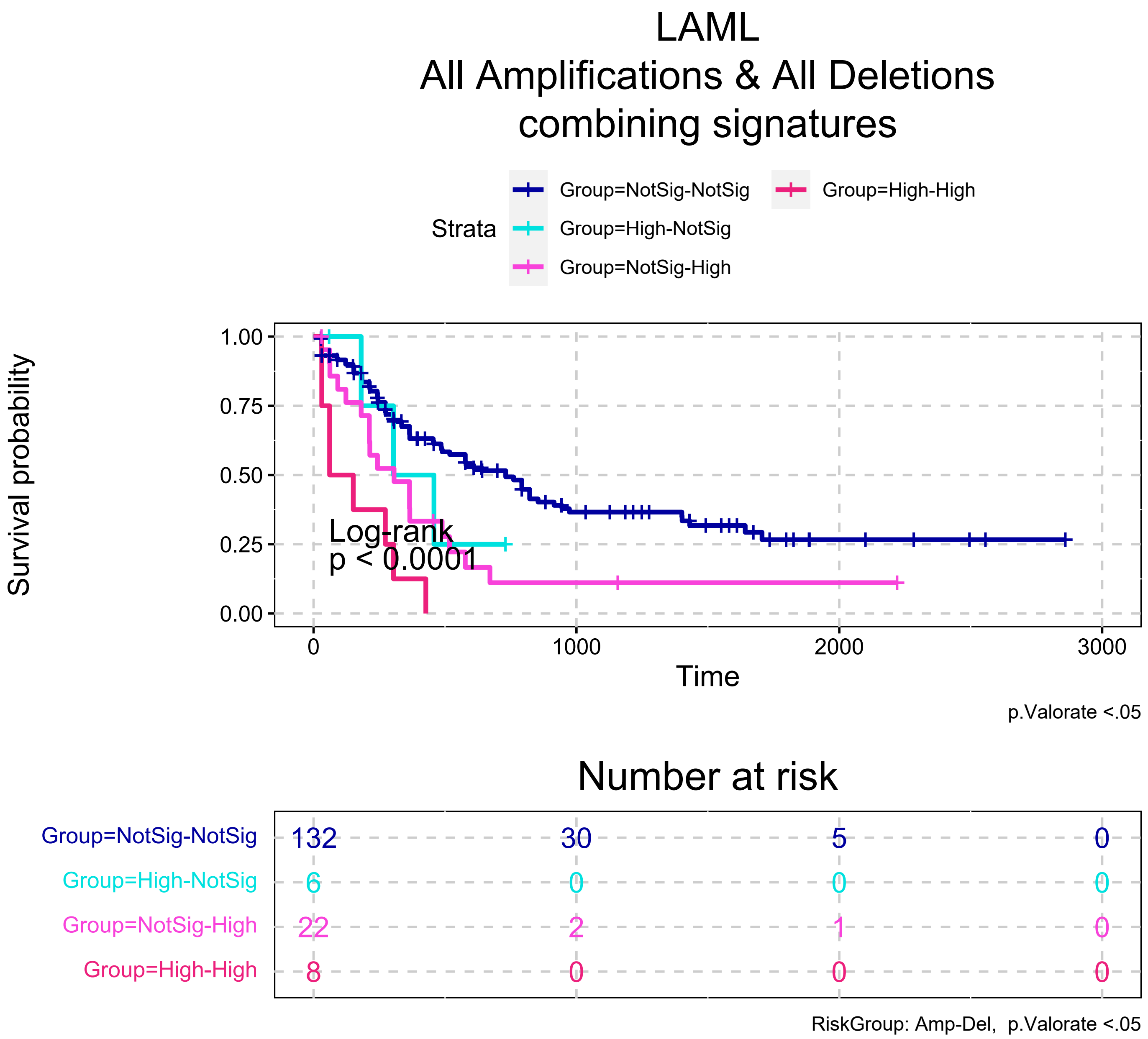

explanatory	beta	HR	L95	U95	p
High-NotSig	0.52	1.68	0.53	5.38	0.38
NotSig-High	0.80	2.21	1.32	3.73	0.00
High-High	1.75	5.77	2.71	12.28	0.00

n= 168, number of events =104
Score(logrank) test = p <.0001

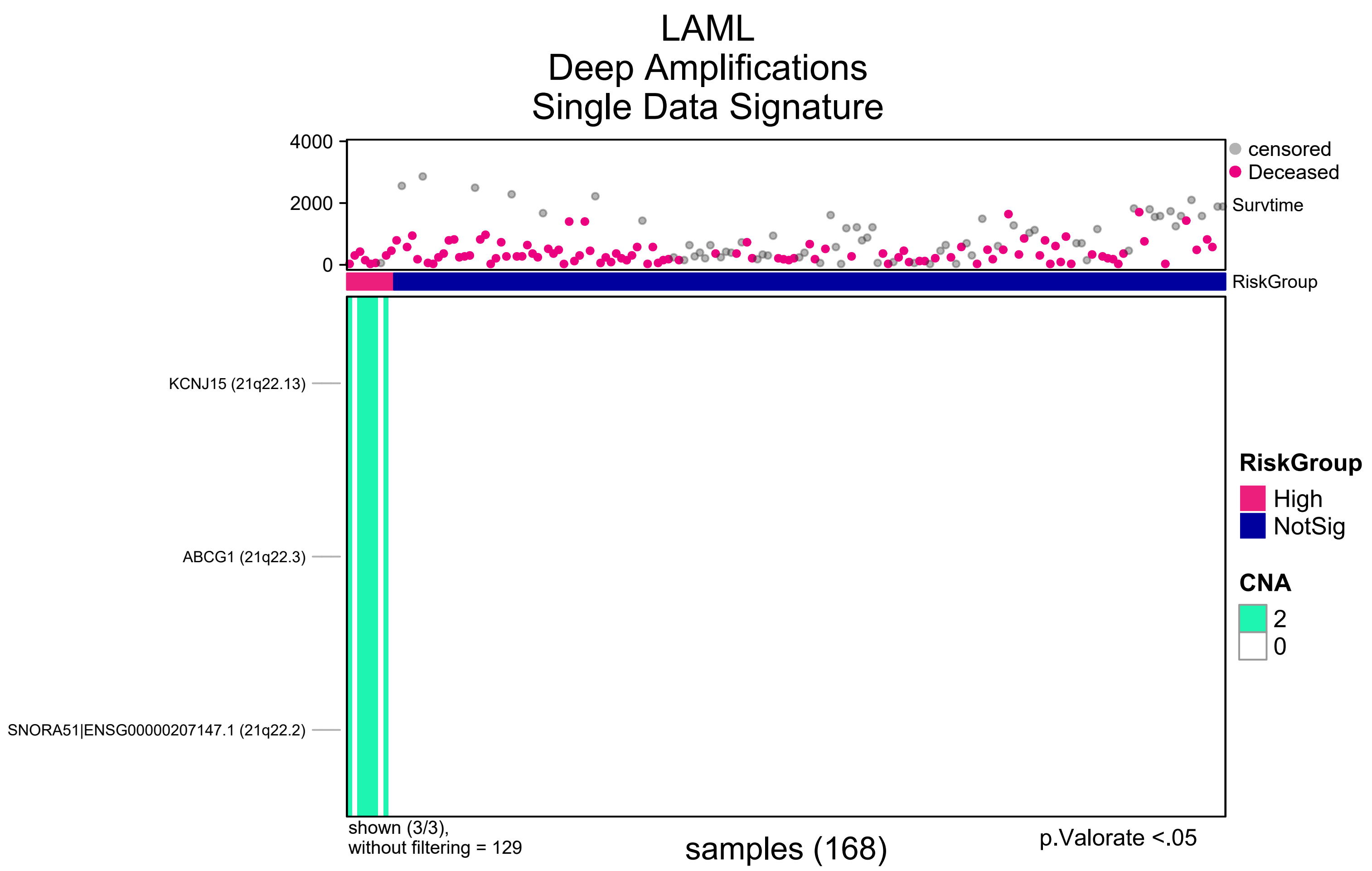
LAML
Deep Amplifications
Single Data Signature
4000
2000
0
censored
Deceased
Survtime
RiskGroup
KCNJ15 (21q22.13)
ABCG1 (21q22.3)
SNORA51|ENSG00000207147.1 (21q22.2)
RiskGroup
High
NotSig
CNA
2
0
shown (3/3),
without filtering = 129
samples (168)
p.Valorate <.05

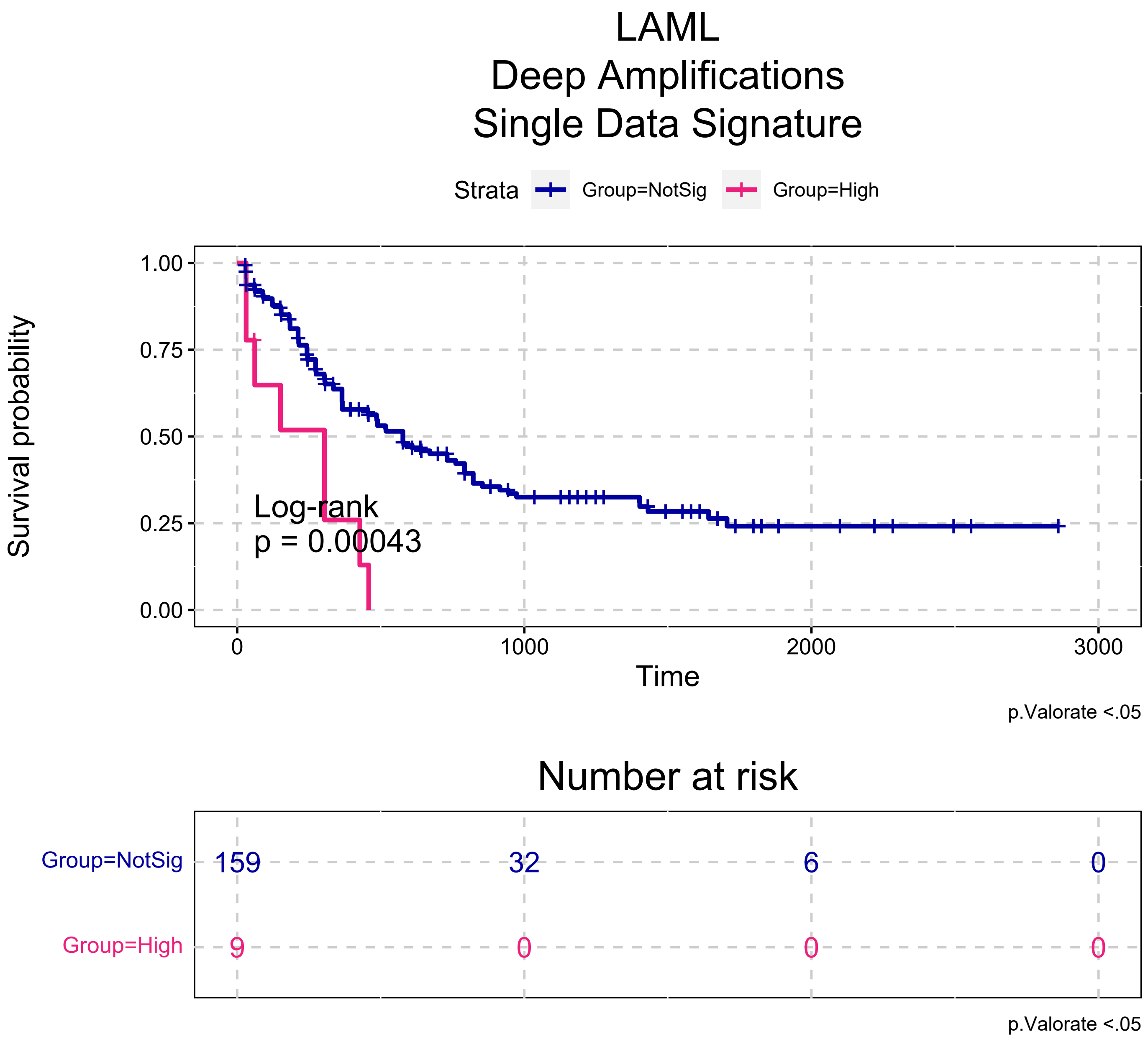

explanatory	beta	HR	L95	U95	p
High	1.25	3.51	1.68	7.34	0.00

n= 168, number of events =104
Score(logrank) test = 0
