# Supplementary material for: Systematic Modeling of Risk-Associated Copy Number Alterations in Cancer
Source: Int J Mol Sci. 2024 Sep 27;25(19):10455. doi: 10.3390/ijms251910455 (PMC11477427; doi:10.3390/ijms251910455)
Supplement: Supplementary file 1 [file ijms-25-10455-s001.zip › LGGSignatureV12-sinSombreado.pdf]

# LGG
# All Amplifications
# Single Data Signature

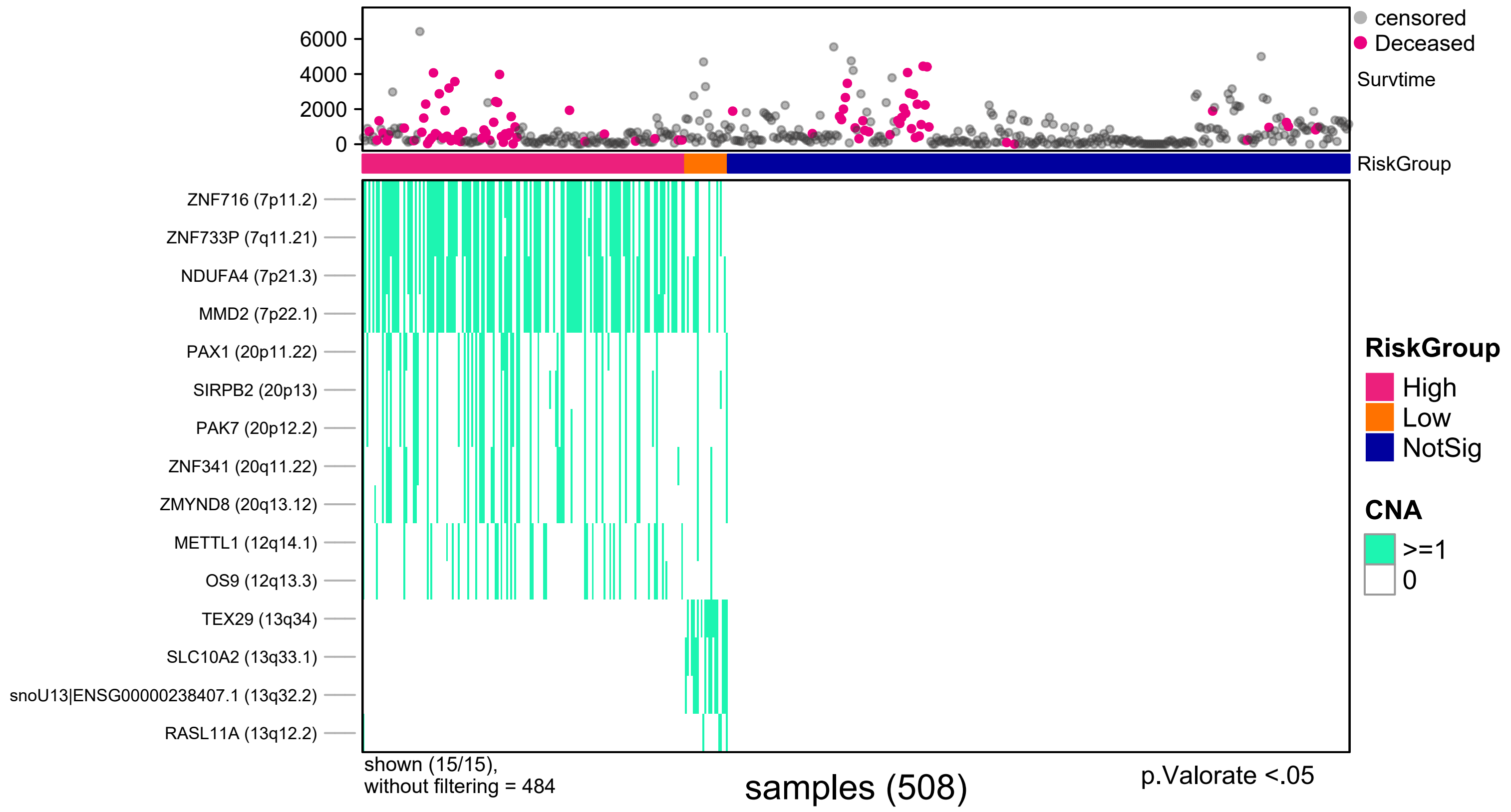

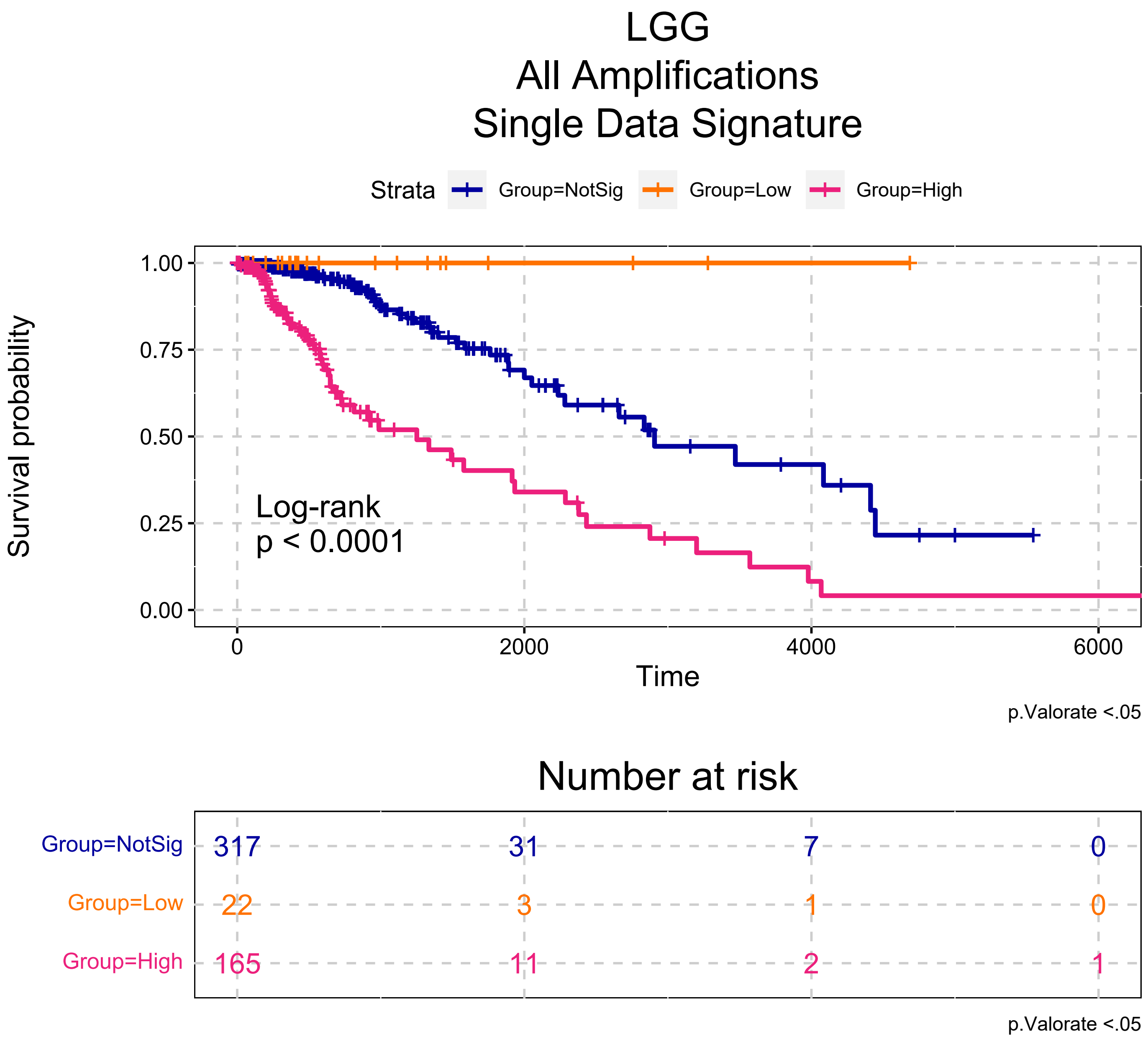


| explanatory | beta | HR | L95 | U95 | p |
|---|---|---|---|---|---|
| Low | -16.88 | 0.00 | 0.00 | Inf | 0.99 |
| High | 1.28 | 3.58 | 2.36 | 5.44 | 0.00 |

n= 508, number of events =92
Score(logrank) test = p <.0001

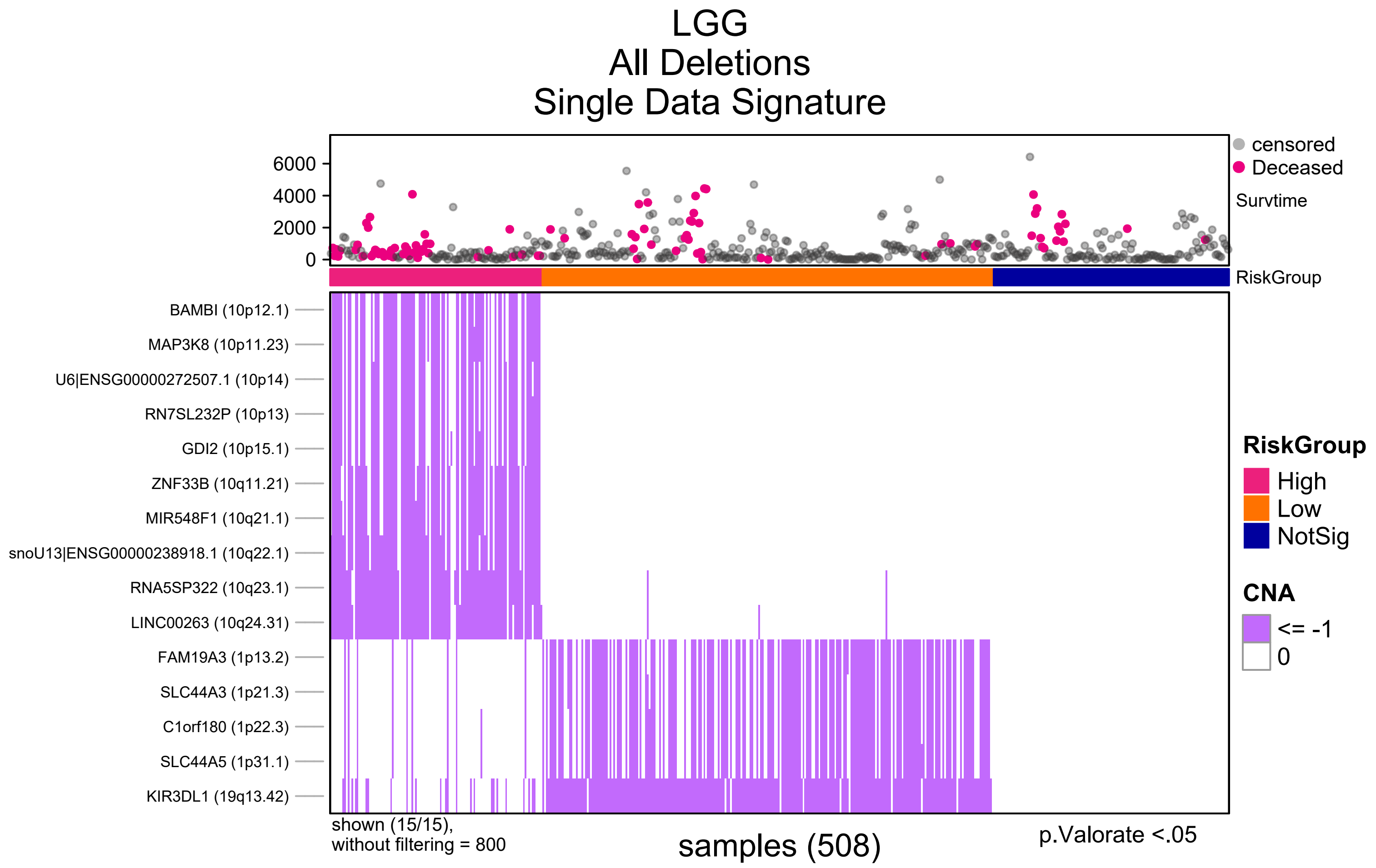

LGG
All Deletions
Single Data Signature
6000
4000
2000
0
censored
Deceased
Survtime
RiskGroup
BAMBI (10p12.1)
MAP3K8 (10p11.23)
U6|ENSG00000272507.1 (10p14)
RN7SL232P (10p13)
GDI2 (10p15.1)
ZNF33B (10q11.21)
MIR548F1 (10q21.1)
snoU13|ENSG00000238918.1 (10q22.1)
RNA5SP322 (10q23.1)
LINC00263 (10q24.31)
FAM19A3 (1p13.2)
SLC44A3 (1p21.3)
C1orf180 (1p22.3)
SLC44A5 (1p31.1)
KIR3DL1 (19q13.42)
RiskGroup
High
Low
NotSig
CNA
<= -1
0
shown (15/15),
without filtering = 800
samples (508)
p.Valorate <.05

# LGG
# All Deletions
# Single Data Signature

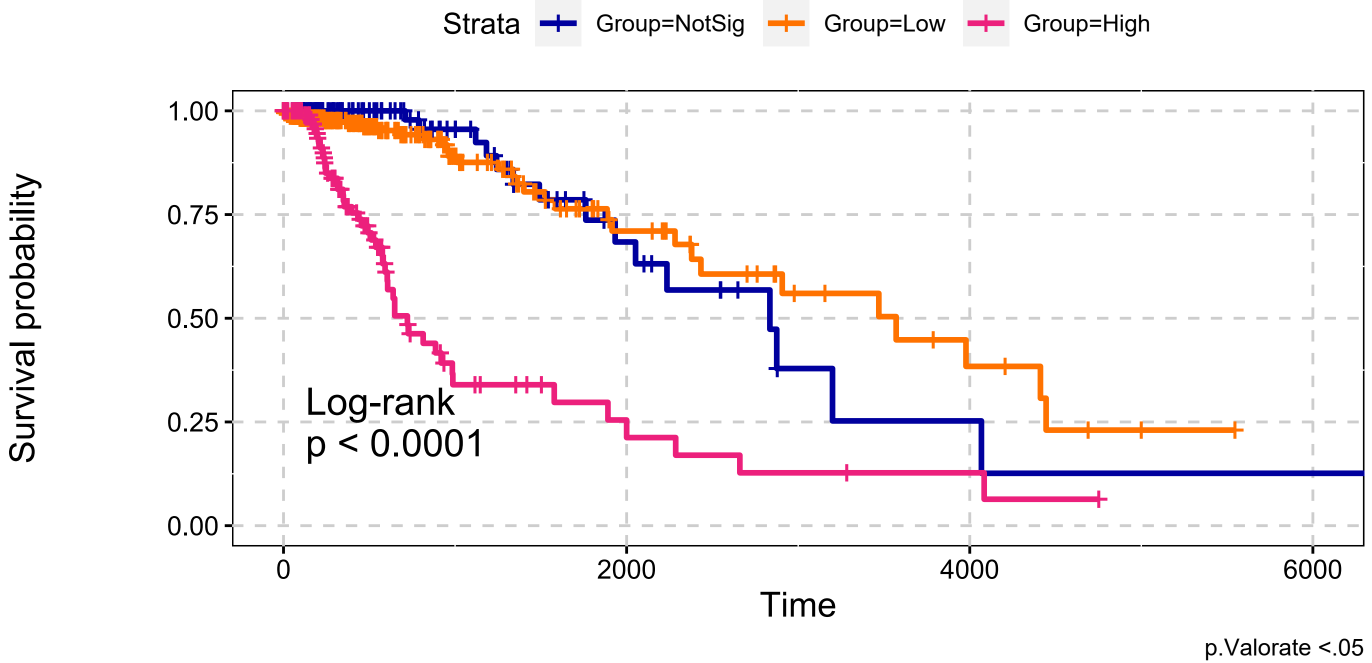


## Number at risk

| | 0 | 2000 | 4000 | 6000 |
|---|---|---|---|---|
| Group=NotSig | 132 | 13 | 2 | 1 |
| Group=Low | 253 | 26 | 6 | 0 |
| Group=High | 119 | 6 | 2 | 0 |

p.Valorate <.05

| explanatory | beta | HR | L95 | U95 | p |
|---|---|---|---|---|---|
| Low | -0.06 | 0.95 | 0.51 | 1.75 | 0.86 |
| High | 1.56 | 4.78 | 2.66 | 8.59 | 0.00 |

n= 508, number of events =92
Score(logrank) test = p <.0001

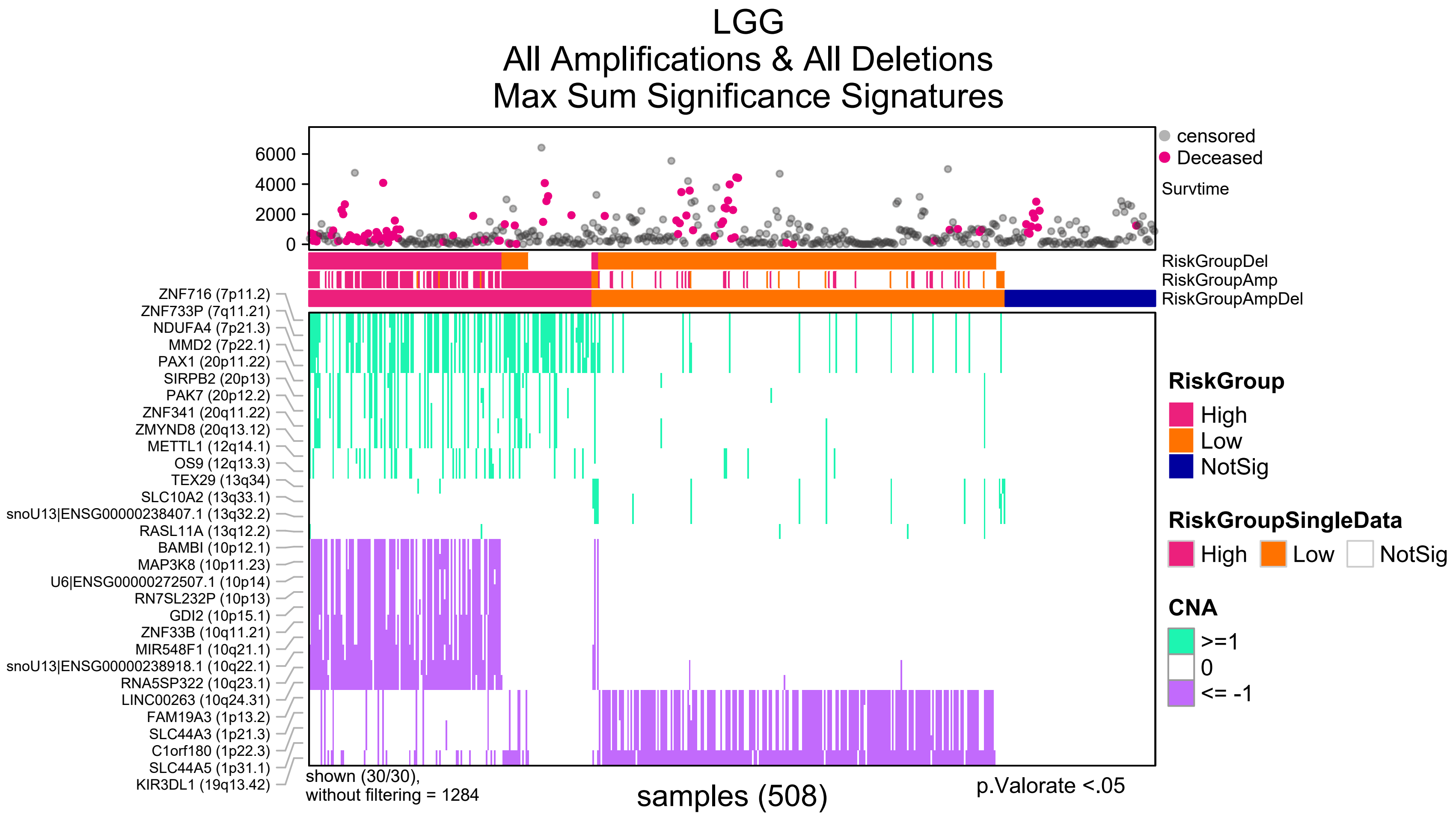

LGG
All Amplifications & All Deletions
Max Sum Significance Signatures
6000
4000
2000
0
censored
Deceased
Survtime
RiskGroupDel
RiskGroupAmp
RiskGroupAmpDel
ZNF716 (7p11.2)
ZNF733P (7q11.21)
NDUFA4 (7p21.3)
MMD2 (7p22.1)
PAX1 (20p11.22)
SIRPB2 (20p13)
PAK7 (20p12.2)
ZNF341 (20q11.22)
ZMYND8 (20q13.12)
METTL1 (12q14.1)
OS9 (12q13.3)
TEX29 (13q34)
SLC10A2 (13q33.1)
snoU13|ENSG00000238407.1 (13q32.2)
RASL11A (13q12.2)
BAMBI (10p12.1)
MAP3K8 (10p11.23)
U6|ENSG00000272507.1 (10p14)
RN7SL232P (10p13)
GDI2 (10p15.1)
ZNF33B (10q11.21)
MIR548F1 (10q21.1)
snoU13|ENSG00000238918.1 (10q22.1)
RNA5SP322 (10q23.1)
LINC00263 (10q24.31)
FAM19A3 (1p13.2)
SLC44A3 (1p21.3)
C1orf180 (1p22.3)
SLC44A5 (1p31.1)
KIR3DL1 (19q13.42)
RiskGroup
High
Low
NotSig
RiskGroupSingleData
High
Low
NotSig
CNA
>=1
0
<= -1
shown (30/30),
without filtering = 1284
samples (508)
p.Valorate <.05

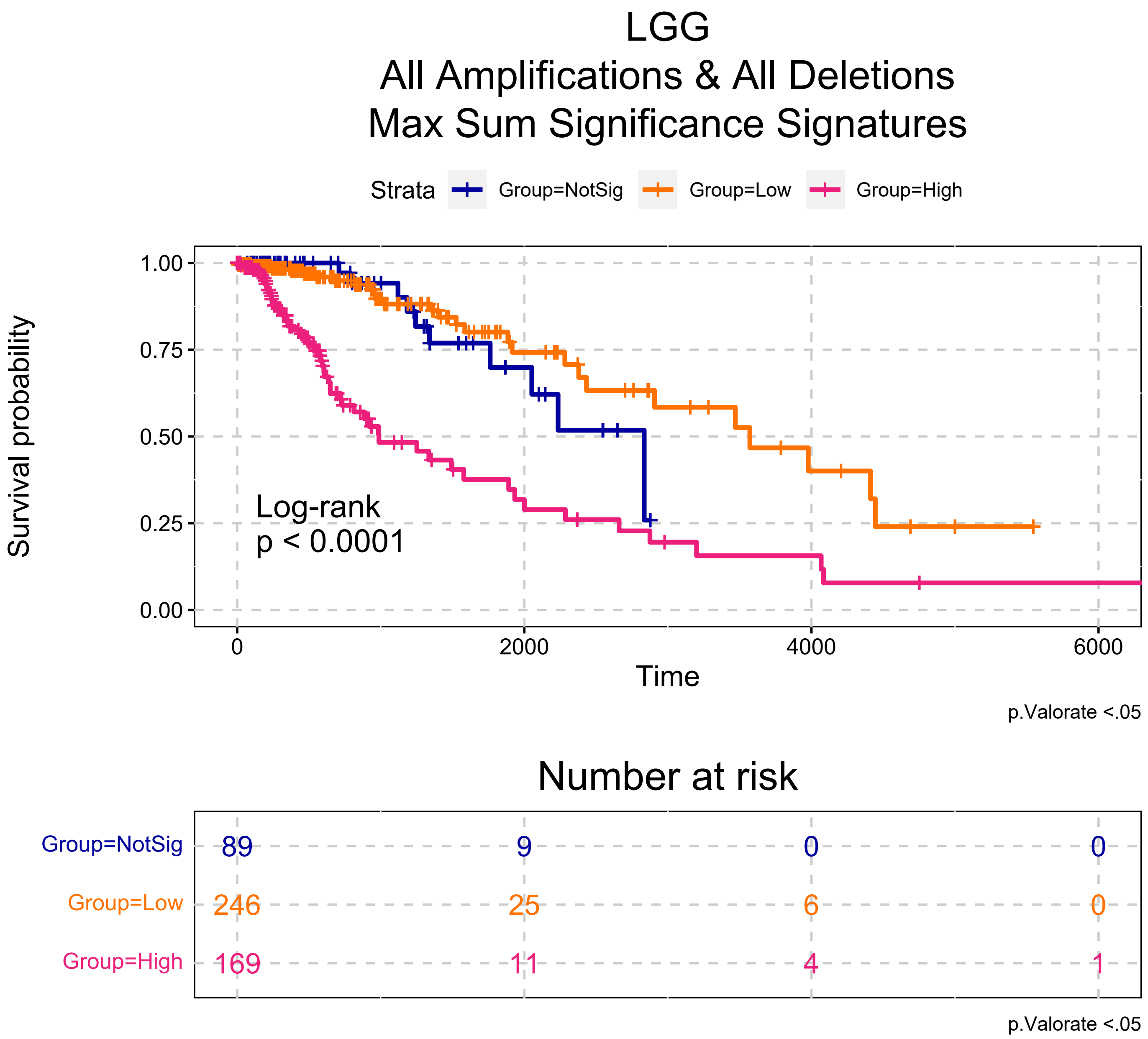


| explanatory | beta | HR | L95 | U95 | p |
|---|---|---|---|---|---|
| Low | -0.19 | 0.82 | 0.40 | 1.71 | 0.60 |
| High | 1.24 | 3.47 | 1.75 | 6.86 | 0.00 |

n= 508, number of events =92
Score(logrank) test = p <.0001

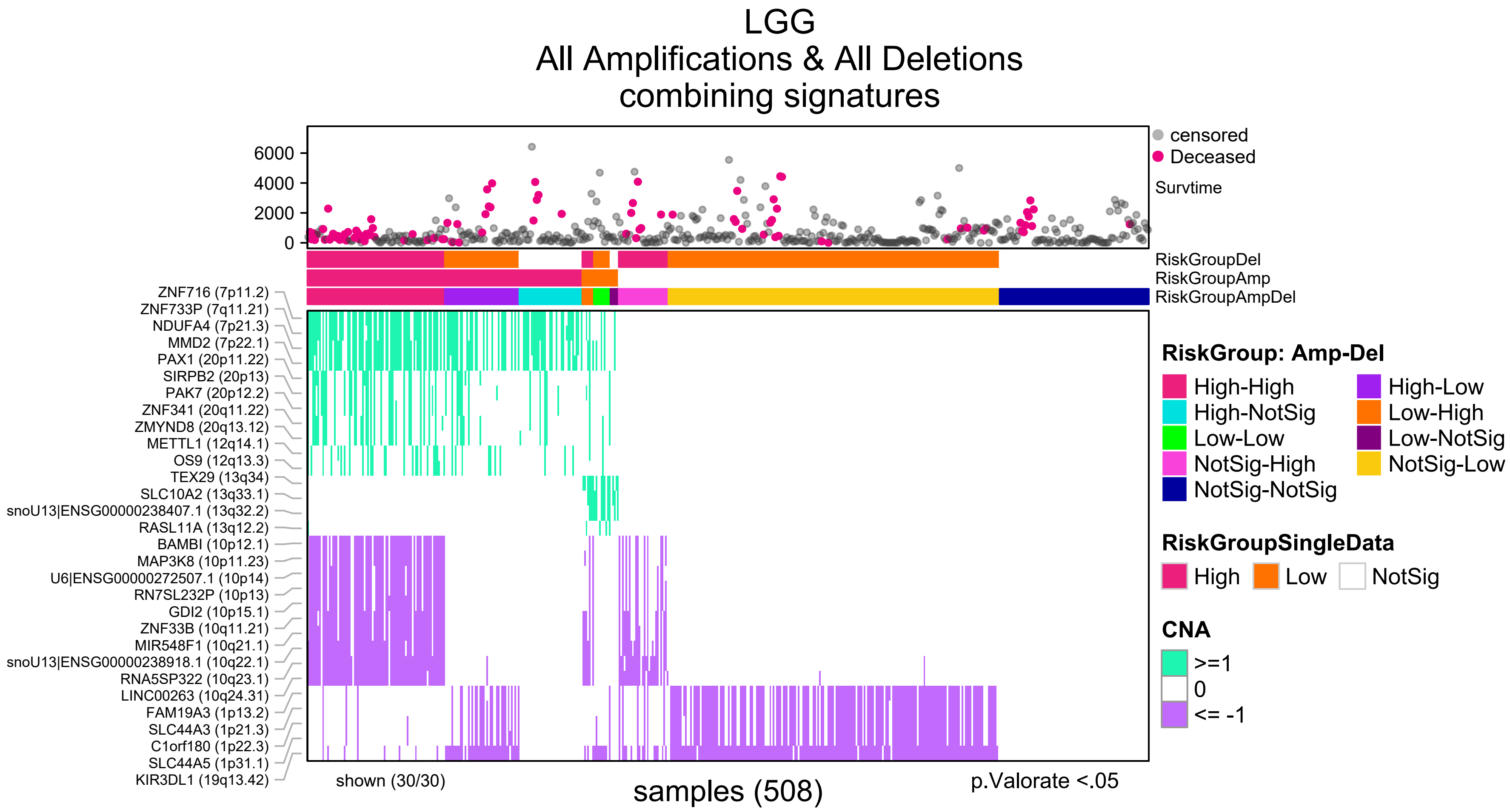
LGG
All Amplifications & All Deletions
combining signatures
6000
4000
2000
0
censored
Deceased
Survtime
RiskGroupDel
RiskGroupAmp
RiskGroupAmpDel
ZNF716 (7p11.2)
ZNF733P (7q11.21)
NDUFA4 (7p21.3)
MMD2 (7p22.1)
PAX1 (20p11.22)
SIRPB2 (20p13)
PAK7 (20p12.2)
ZNF341 (20q11.22)
ZMYND8 (20q13.12)
METTL1 (12q14.1)
OS9 (12q13.3)
TEX29 (13q34)
SLC10A2 (13q33.1)
snoU13|ENSG00000238407.1 (13q32.2)
RASL11A (13q12.2)
BAMBI (10p12.1)
MAP3K8 (10p11.23)
U6|ENSG00000272507.1 (10p14)
RN7SL232P (10p13)
GDI2 (10p15.1)
ZNF33B (10q11.21)
MIR548F1 (10q21.1)
snoU13|ENSG00000238918.1 (10q22.1)
RNA5SP322 (10q23.1)
LINC00263 (10q24.31)
FAM19A3 (1p13.2)
SLC44A3 (1p21.3)
C1orf180 (1p22.3)
SLC44A5 (1p31.1)
KIR3DL1 (19q13.42)
shown (30/30)
samples (508)
p.Valorate <.05
RiskGroup: Amp-Del
High-High
High-NotSig
Low-Low
NotSig-High
NotSig-NotSig
High-Low
Low-High
Low-NotSig
NotSig-Low
RiskGroupSingleData
High
Low
NotSig
CNA
>=1
0
<= -1

LGG
All Amplifications & All Deletions
combining signatures

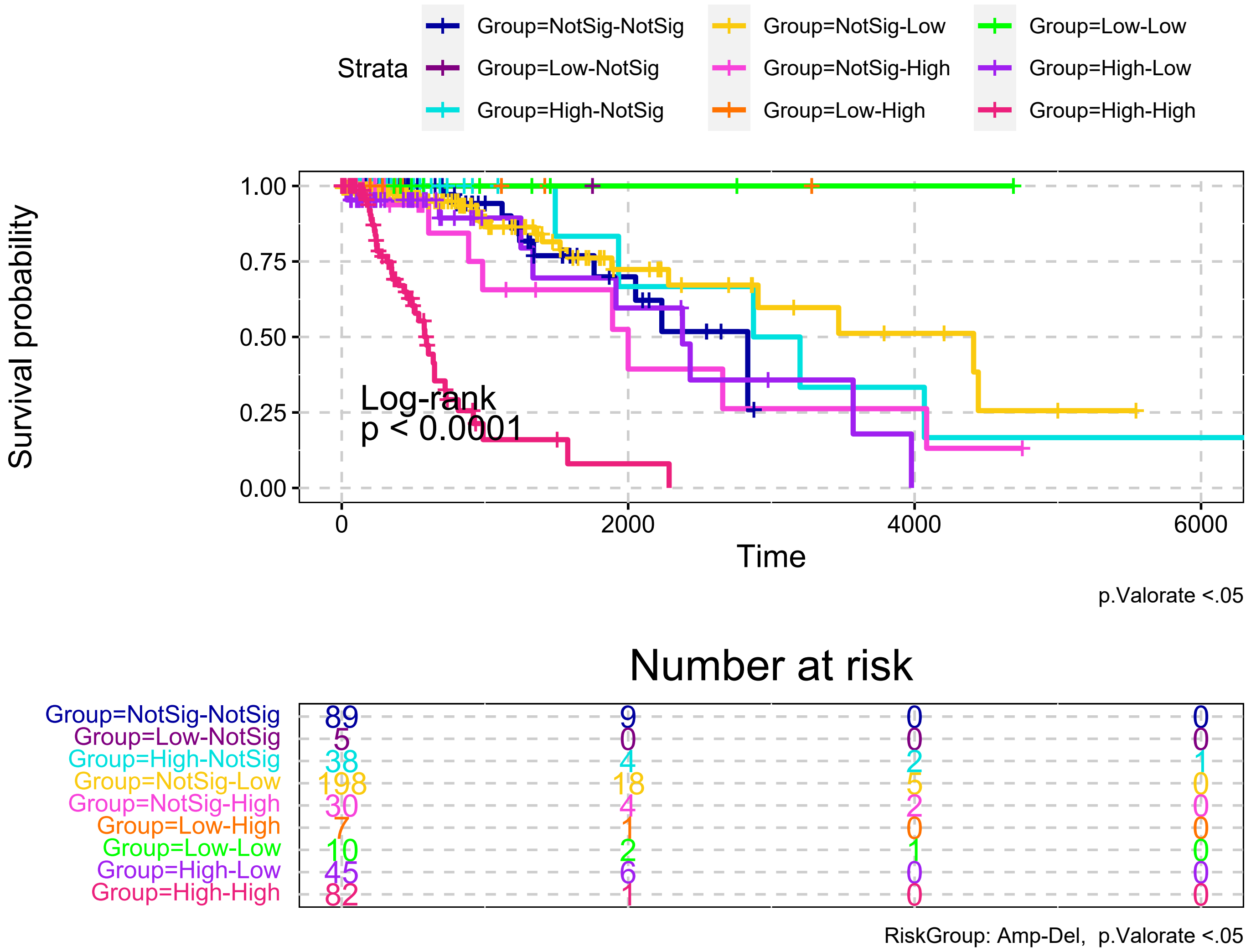


| explanatory | beta | HR | L95 | U95 | p |
|---|---|---|---|---|---|
| Low-NotSig | -21.29 | 0.00 | 0.00 | Inf | 1.00 |
| High-NotSig | -0.21 | 0.81 | 0.27 | 2.45 | 0.71 |
| NotSig-Low | -0.29 | 0.75 | 0.35 | 1.61 | 0.46 |
| NotSig-High | 0.38 | 1.46 | 0.56 | 3.82 | 0.44 |
| Low-High | -17.85 | 0.00 | 0.00 | Inf | 1.00 |
| Low-Low | -17.02 | 0.00 | 0.00 | Inf | 1.00 |
| High-Low | 0.44 | 1.55 | 0.64 | 3.77 | 0.33 |
| High-High | 2.37 | 10.68 | 5.19 | 21.98 | 0.00 |

n= 508, number of events =92
Score(logrank) test = p <.0001

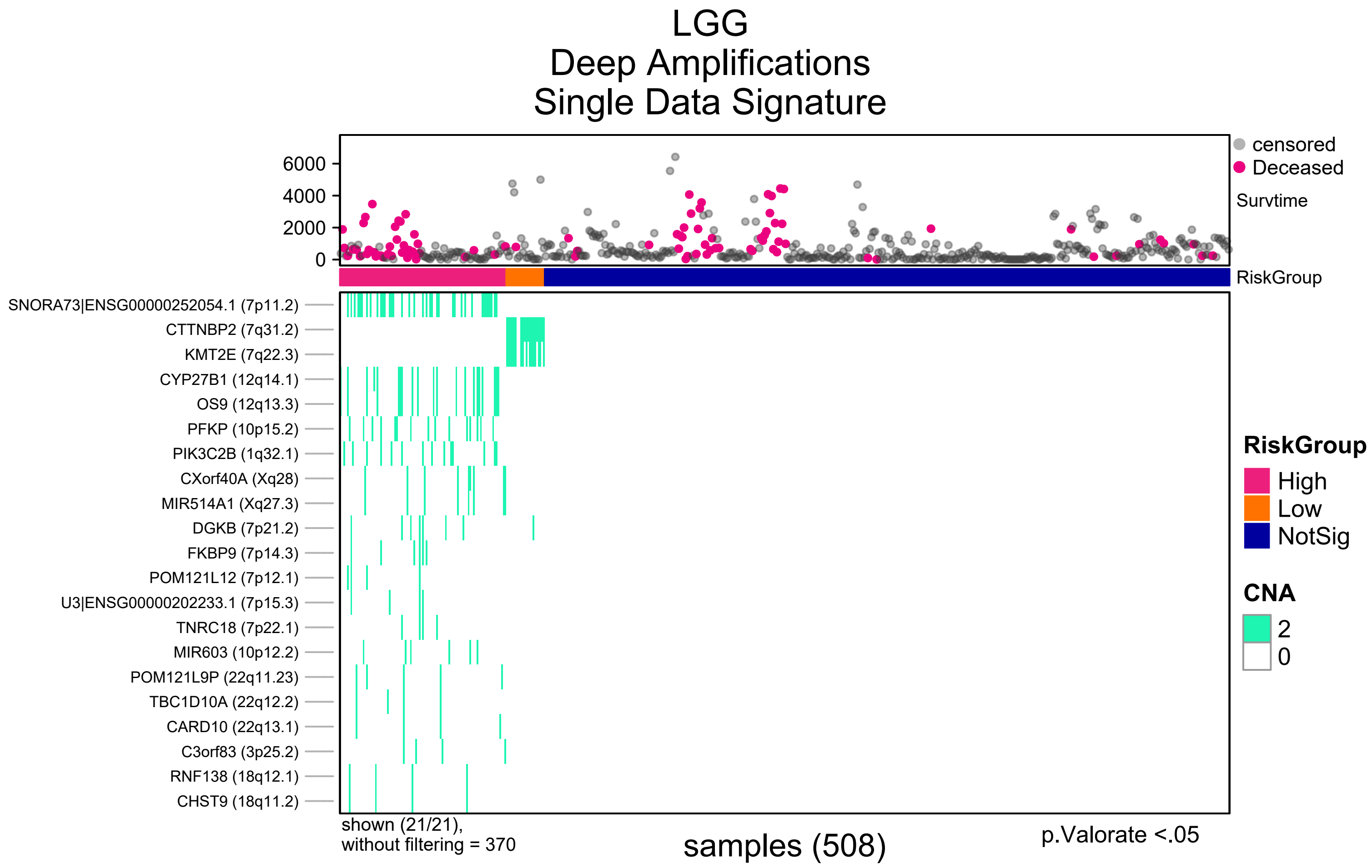
LGG
Deep Amplifications
Single Data Signature
6000
4000
2000
0
censored
Deceased
Survtime
RiskGroup
SNORA73|ENSG00000252054.1 (7p11.2)
CTTNBP2 (7q31.2)
KMT2E (7q22.3)
CYP27B1 (12q14.1)
OS9 (12q13.3)
PFKP (10p15.2)
PIK3C2B (1q32.1)
CXorf40A (Xq28)
MIR514A1 (Xq27.3)
DGKB (7p21.2)
FKBP9 (7p14.3)
POM121L12 (7p12.1)
U3|ENSG00000202233.1 (7p15.3)
TNRC18 (7p22.1)
MIR603 (10p12.2)
POM121L9P (22q11.23)
TBC1D10A (22q12.2)
CARD10 (22q13.1)
C3orf83 (3p25.2)
RNF138 (18q12.1)
CHST9 (18q11.2)
RiskGroup
High
Low
NotSig
CNA
2
0
shown (21/21),
without filtering = 370
samples (508)
p.Valorate <.05

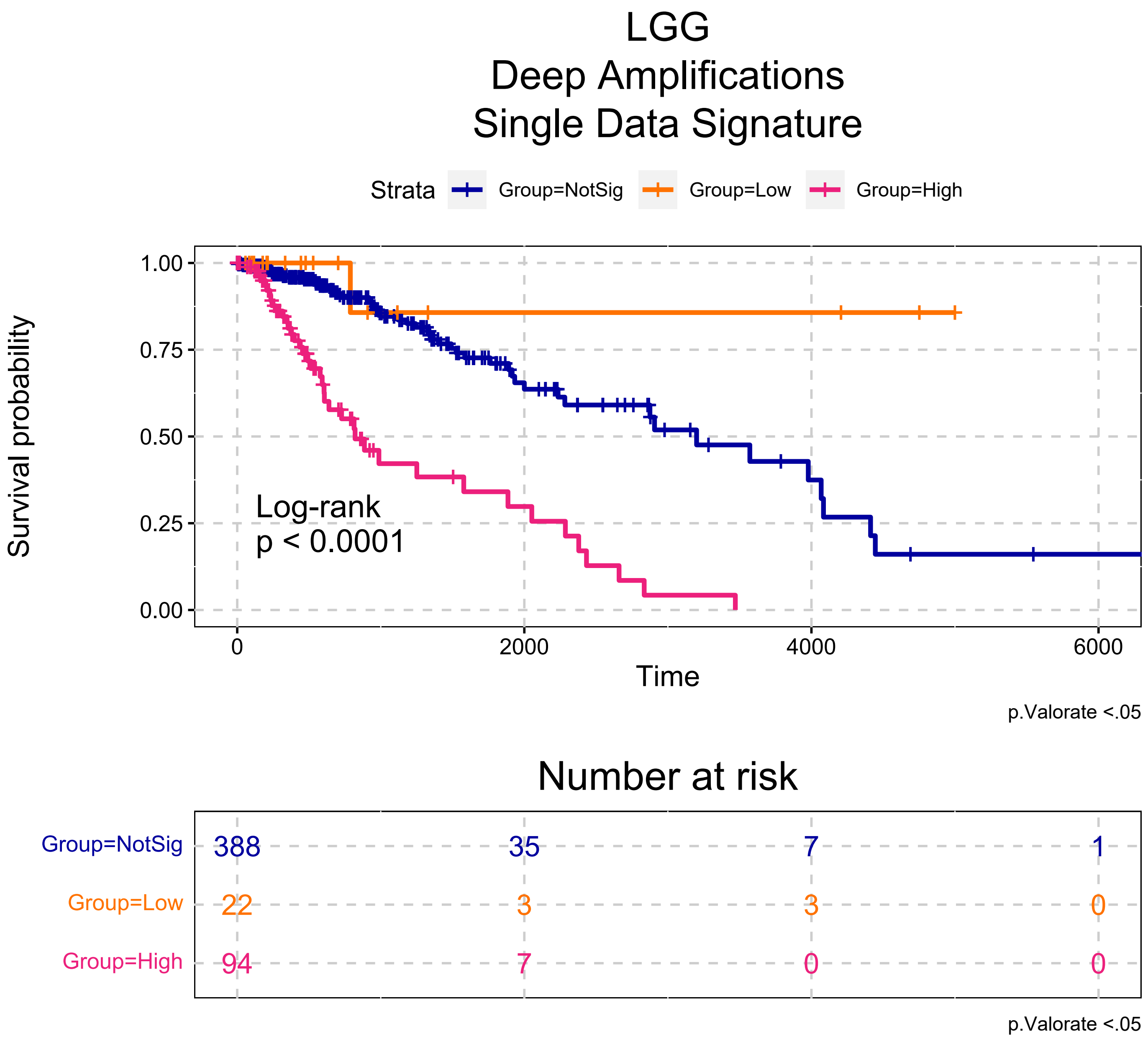


| explanatory | beta | HR | L95 | U95 | p |
|---|---|---|---|---|---|
| Low | -1.74 | 0.18 | 0.02 | 1.30 | 0.09 |
| High | 1.52 | 4.55 | 2.96 | 7.00 | 0.00 |

n= 508, number of events =92
Score(logrank) test = p <.0001

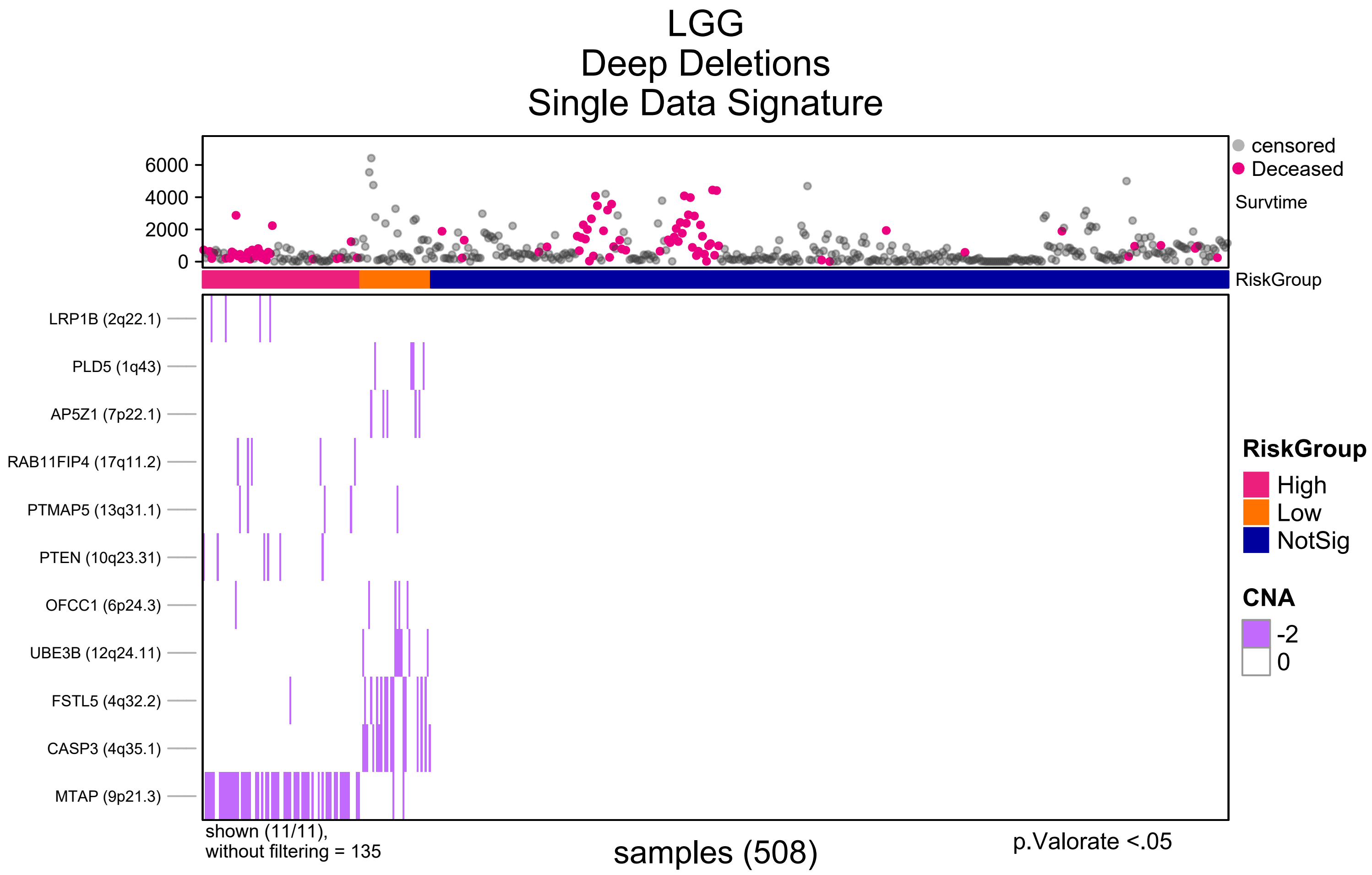
LGG
Deep Deletions
Single Data Signature
6000
4000
2000
0
censored
Deceased
Survtime
RiskGroup
LRP1B (2q22.1)
PLD5 (1q43)
AP5Z1 (7p22.1)
RAB11FIP4 (17q11.2)
PTMAP5 (13q31.1)
PTEN (10q23.31)
OFCC1 (6p24.3)
UBE3B (12q24.11)
FSTL5 (4q32.2)
CASP3 (4q35.1)
MTAP (9p21.3)
RiskGroup
High
Low
NotSig
CNA
-2
0
shown (11/11),
without filtering = 135
samples (508)
p.Valorate <.05

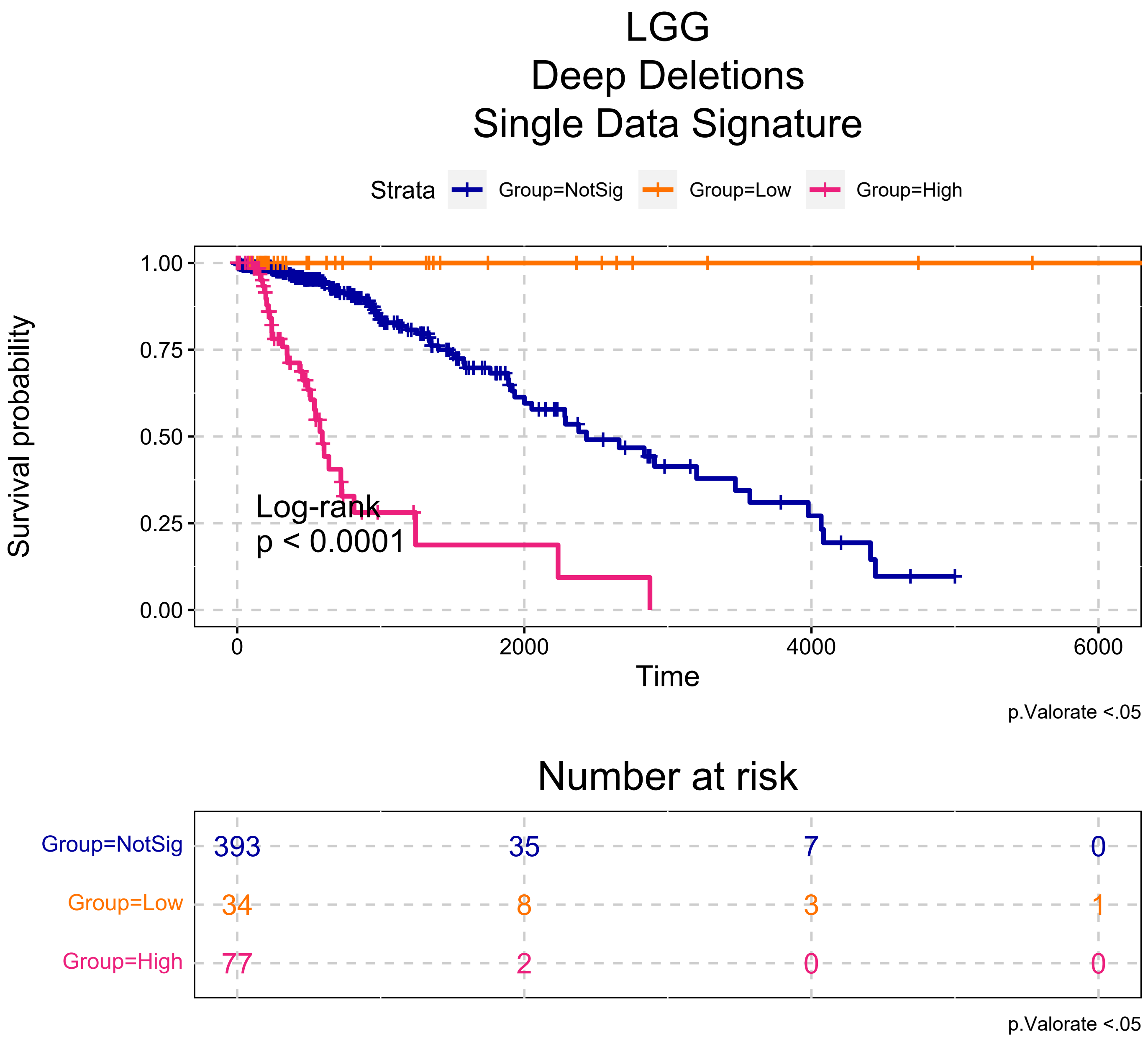


| explanatory | beta | HR | L95 | U95 | p |
|---|---|---|---|---|---|
| Low | -18.72 | 0.00 | 0.00 | Inf | 1.00 |
| High | 1.93 | 6.88 | 4.32 | 10.96 | 0.00 |

n= 508, number of events =92
Score(logrank) test = p <.0001

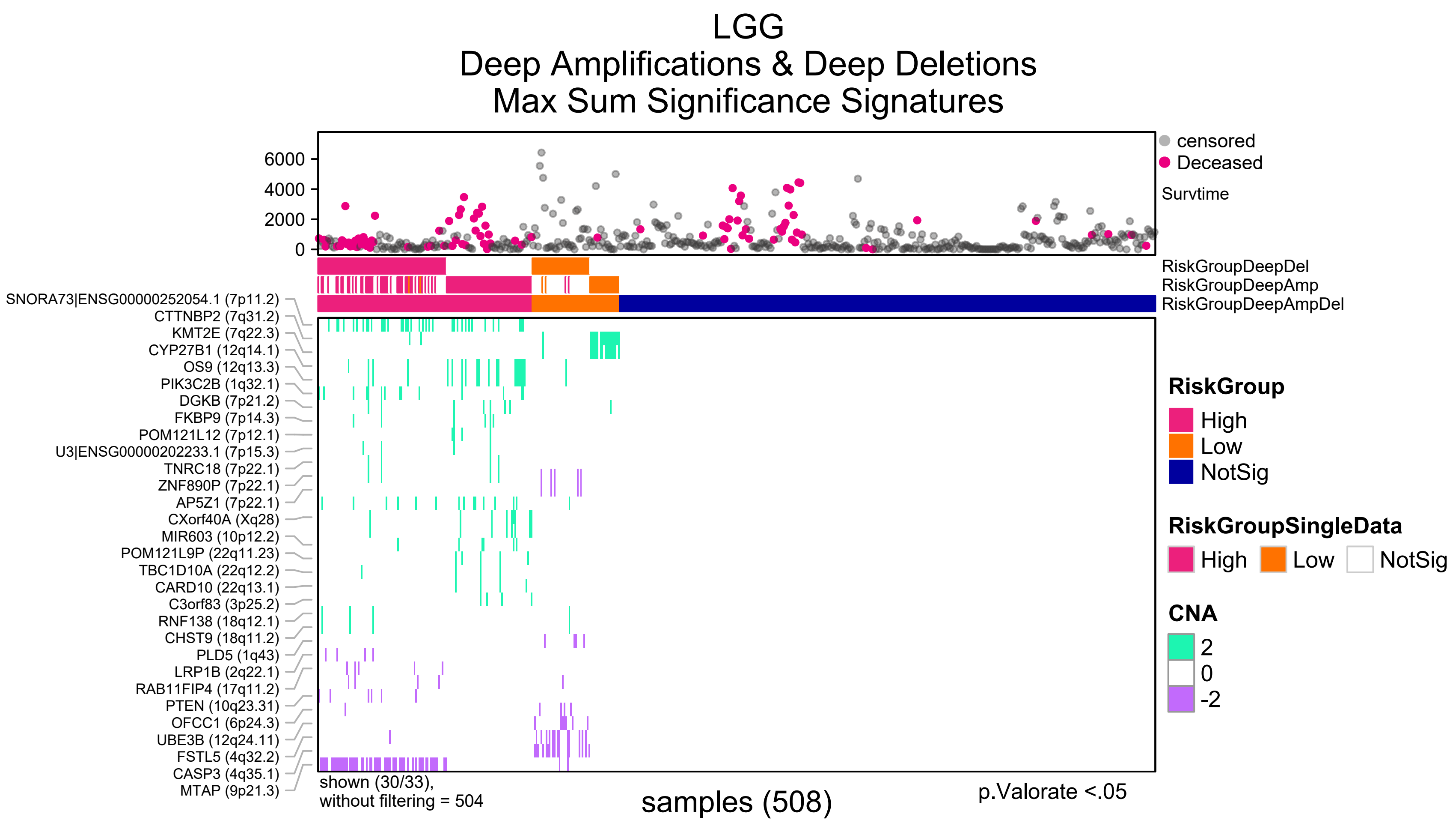
LGG
Deep Amplifications & Deep Deletions
Max Sum Significance Signatures
6000
4000
2000
0
censored
Deceased
Survtime
RiskGroupDeepDel
RiskGroupDeepAmp
RiskGroupDeepAmpDel
SNORA73|ENSG00000252054.1 (7p11.2)
CTTNBP2 (7q31.2)
KMT2E (7q22.3)
CYP27B1 (12q14.1)
OS9 (12q13.3)
PIK3C2B (1q32.1)
DGKB (7p21.2)
FKBP9 (7p14.3)
POM121L12 (7p12.1)
U3|ENSG00000202233.1 (7p15.3)
TNRC18 (7p22.1)
ZNF890P (7p22.1)
AP5Z1 (7p22.1)
CXorf40A (Xq28)
MIR603 (10p12.2)
POM121L9P (22q11.23)
TBC1D10A (22q12.2)
CARD10 (22q13.1)
C3orf83 (3p25.2)
RNF138 (18q12.1)
CHST9 (18q11.2)
PLD5 (1q43)
LRP1B (2q22.1)
RAB11FIP4 (17q11.2)
PTEN (10q23.31)
OFCC1 (6p24.3)
UBE3B (12q24.11)
FSTL5 (4q32.2)
CASP3 (4q35.1)
MTAP (9p21.3)
RiskGroup
High
Low
NotSig
RiskGroupSingleData
High
Low
NotSig
CNA
2
0
-2
shown (30/33),
without filtering = 504
samples (508)
p.Valorate <.05

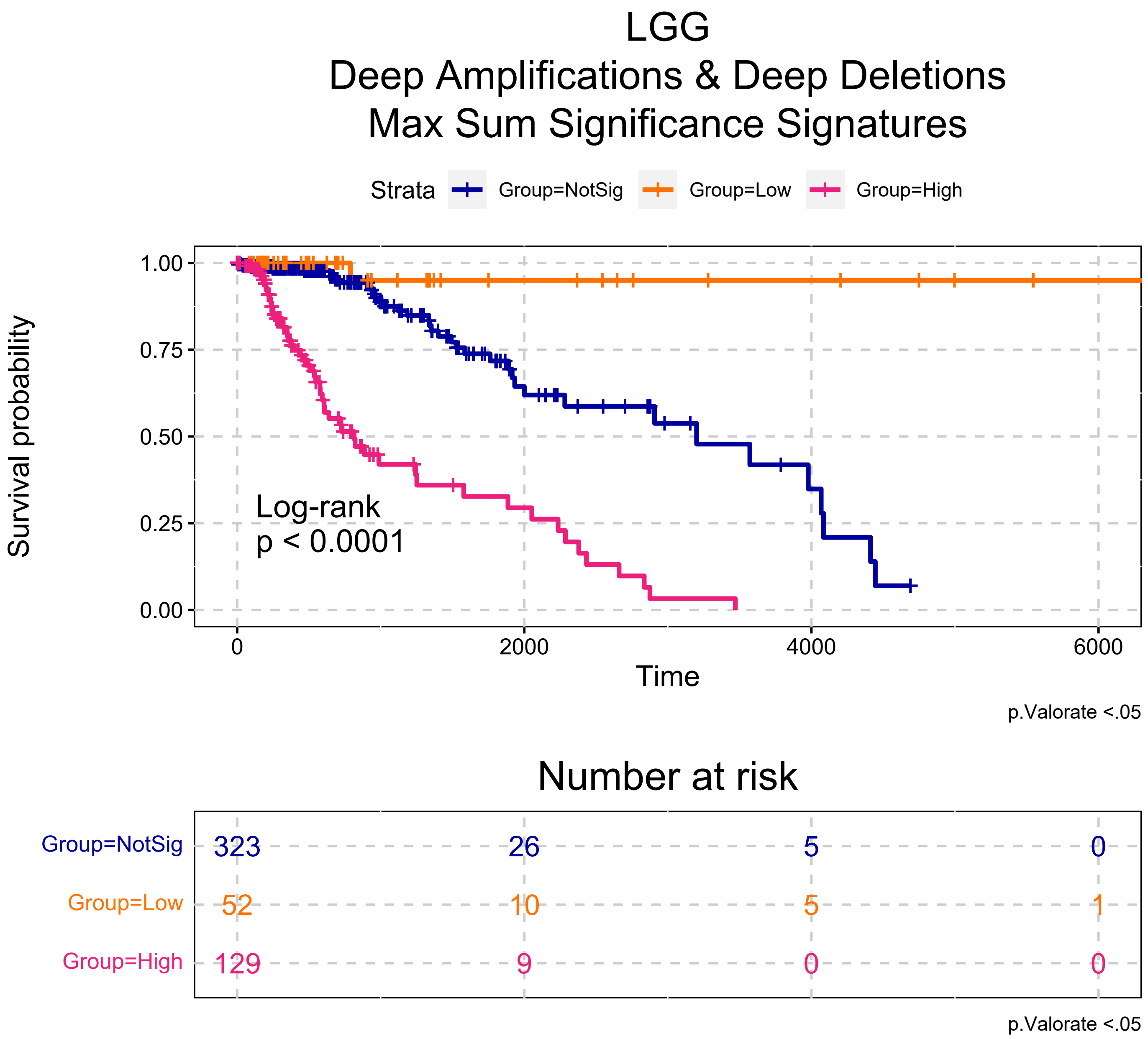


| explanatory | beta | HR | L95 | U95 | p |
|---|---|---|---|---|---|
| Low | -2.70 | 0.07 | 0.01 | 0.50 | 0.01 |
| High | 1.74 | 5.70 | 3.67 | 8.86 | 0.00 |

n= 508, number of events =92
Score(logrank) test = p <.0001

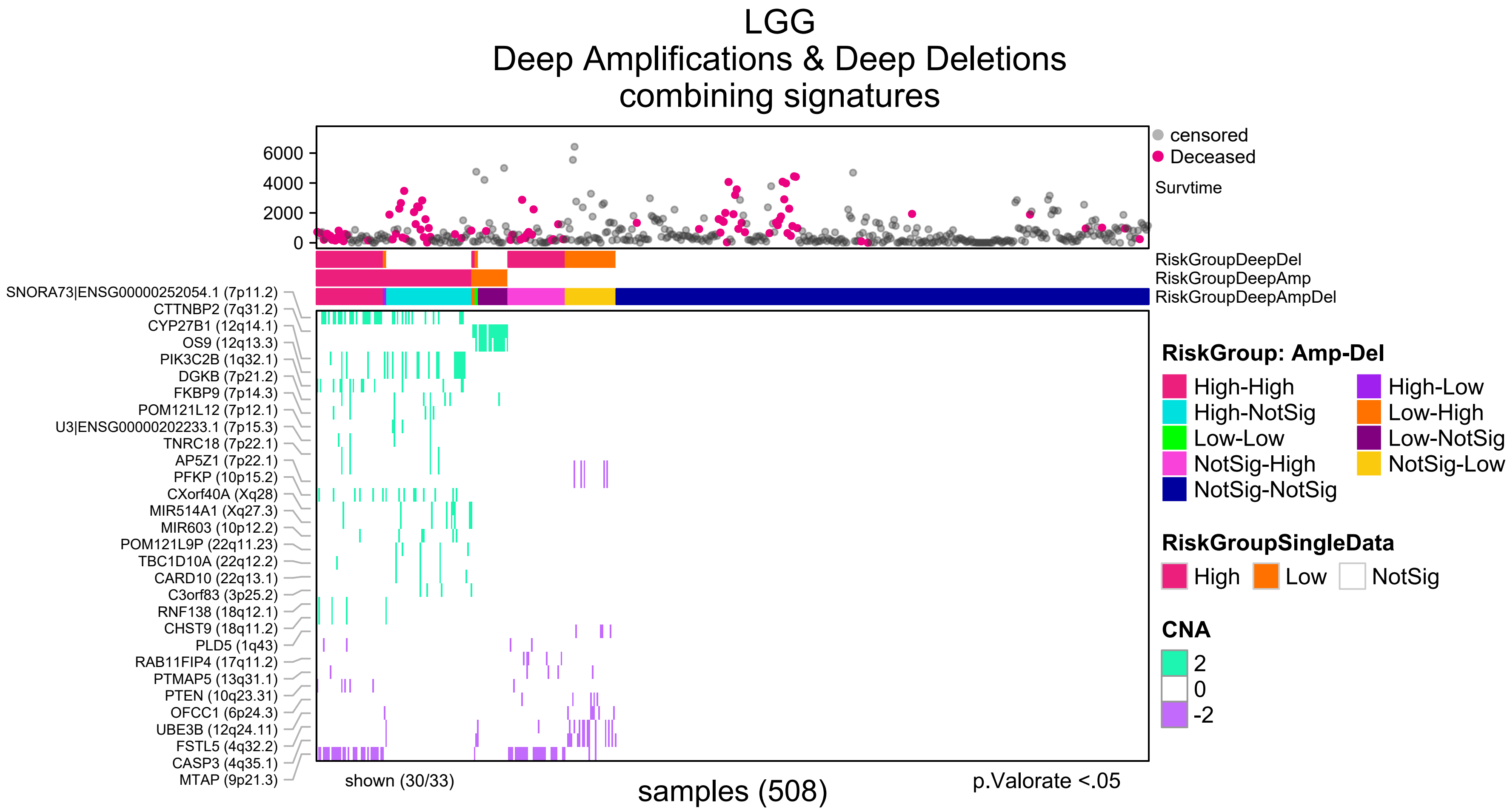
LGG
Deep Amplifications & Deep Deletions
combining signatures
6000
4000
2000
0
censored
Deceased
Survtime
RiskGroupDeepDel
RiskGroupDeepAmp
RiskGroupDeepAmpDel
SNORA73|ENSG00000252054.1 (7p11.2)
CTTNBP2 (7q31.2)
CYP27B1 (12q14.1)
OS9 (12q13.3)
PIK3C2B (1q32.1)
DGKB (7p21.2)
FKBP9 (7p14.3)
POM121L12 (7p12.1)
U3|ENSG00000202233.1 (7p15.3)
TNRC18 (7p22.1)
AP5Z1 (7p22.1)
PFKP (10p15.2)
CXorf40A (Xq28)
MIR514A1 (Xq27.3)
MIR603 (10p12.2)
POM121L9P (22q11.23)
TBC1D10A (22q12.2)
CARD10 (22q13.1)
C3orf83 (3p25.2)
RNF138 (18q12.1)
CHST9 (18q11.2)
PLD5 (1q43)
RAB11FIP4 (17q11.2)
PTMAP5 (13q31.1)
PTEN (10q23.31)
OFCC1 (6p24.3)
UBE3B (12q24.11)
FSTL5 (4q32.2)
CASP3 (4q35.1)
MTAP (9p21.3)
RiskGroup: Amp-Del
High-High
High-NotSig
Low-Low
NotSig-High
NotSig-NotSig
High-Low
Low-High
Low-NotSig
NotSig-Low
RiskGroupSingleData
High
Low
NotSig
CNA
2
0
-2
shown (30/33)
samples (508)
p.Valorate <.05

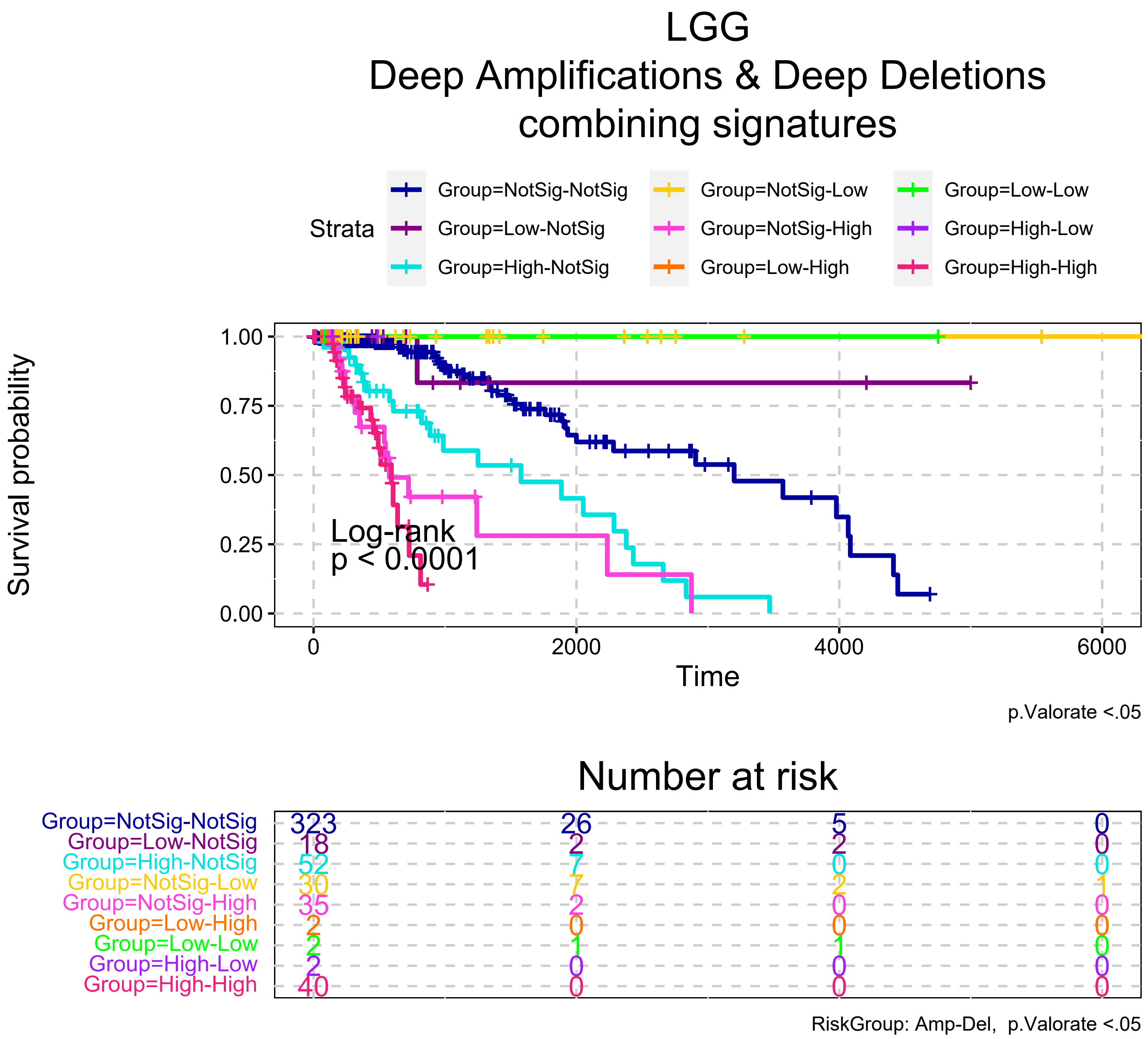


| explanatory | beta | HR | L95 | U95 | p |
|---|---|---|---|---|---|
| Low-NotSig | -1.49 | 0.22 | 0.03 | 1.68 | 0.15 |
| High-NotSig | 1.32 | 3.76 | 2.18 | 6.47 | 0.00 |
| NotSig-Low | -18.61 | 0.00 | 0.00 | Inf | 1.00 |
| NotSig-High | 1.94 | 6.99 | 3.71 | 13.18 | 0.00 |
| Low-High | NA | NA | NA | NA | NA |
| Low-Low | -18.72 | 0.00 | 0.00 | Inf | 1.00 |
| High-Low | NA | NA | NA | NA | NA |
| High-High | 2.69 | 14.66 | 7.56 | 28.45 | 0.00 |

n= 508, number of events =92
Score(logrank) test = p <.0001
