# Supplementary material for: Systematic Modeling of Risk-Associated Copy Number Alterations in Cancer
Source: Int J Mol Sci. 2024 Sep 27;25(19):10455. doi: 10.3390/ijms251910455 (PMC11477427; doi:10.3390/ijms251910455)
Supplement: Supplementary file 1 [file ijms-25-10455-s001.zip › LIHCSignatureV12-sinSombreado.pdf]

LIHC
All Amplifications
Single Data Signature

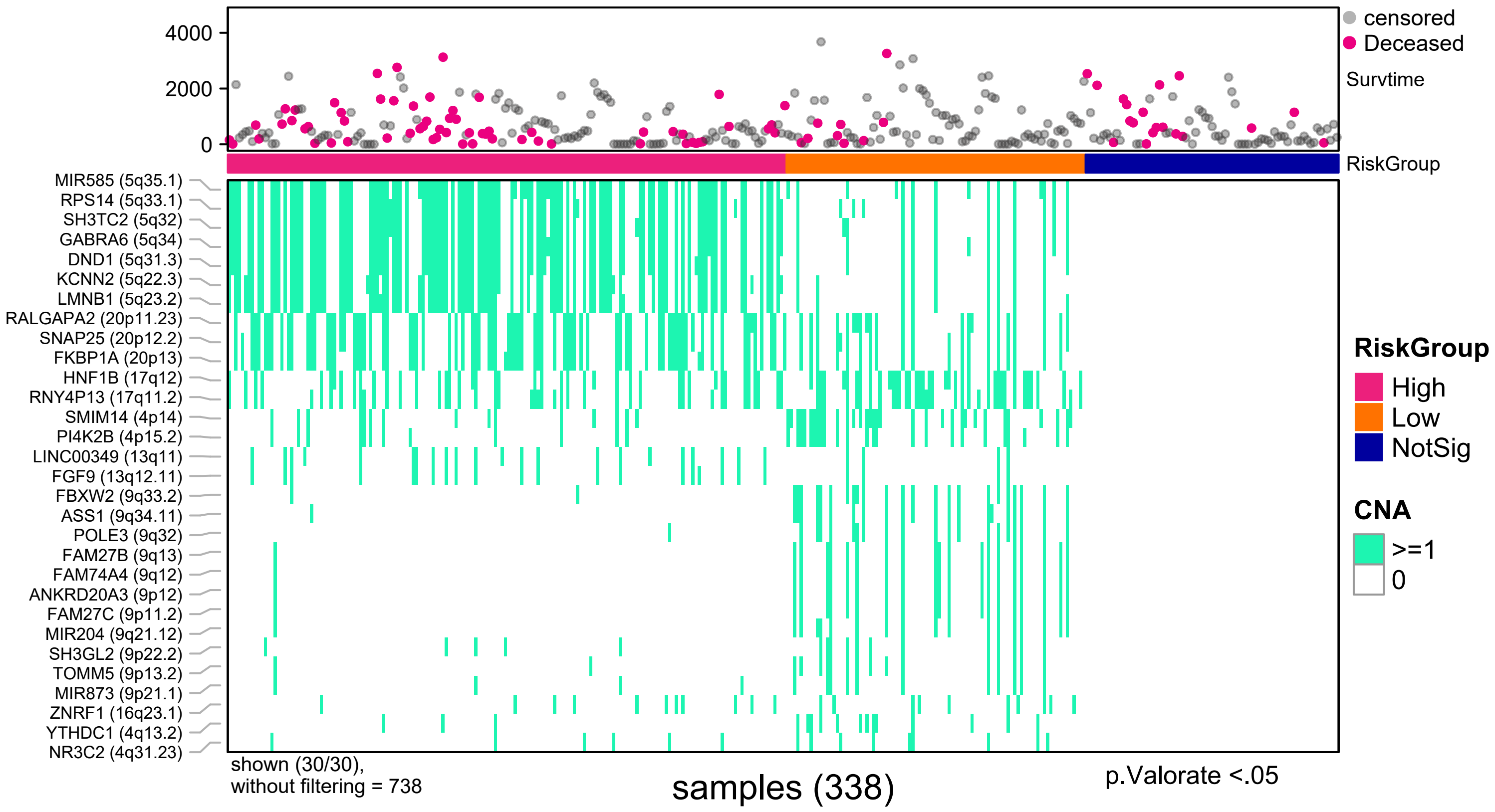

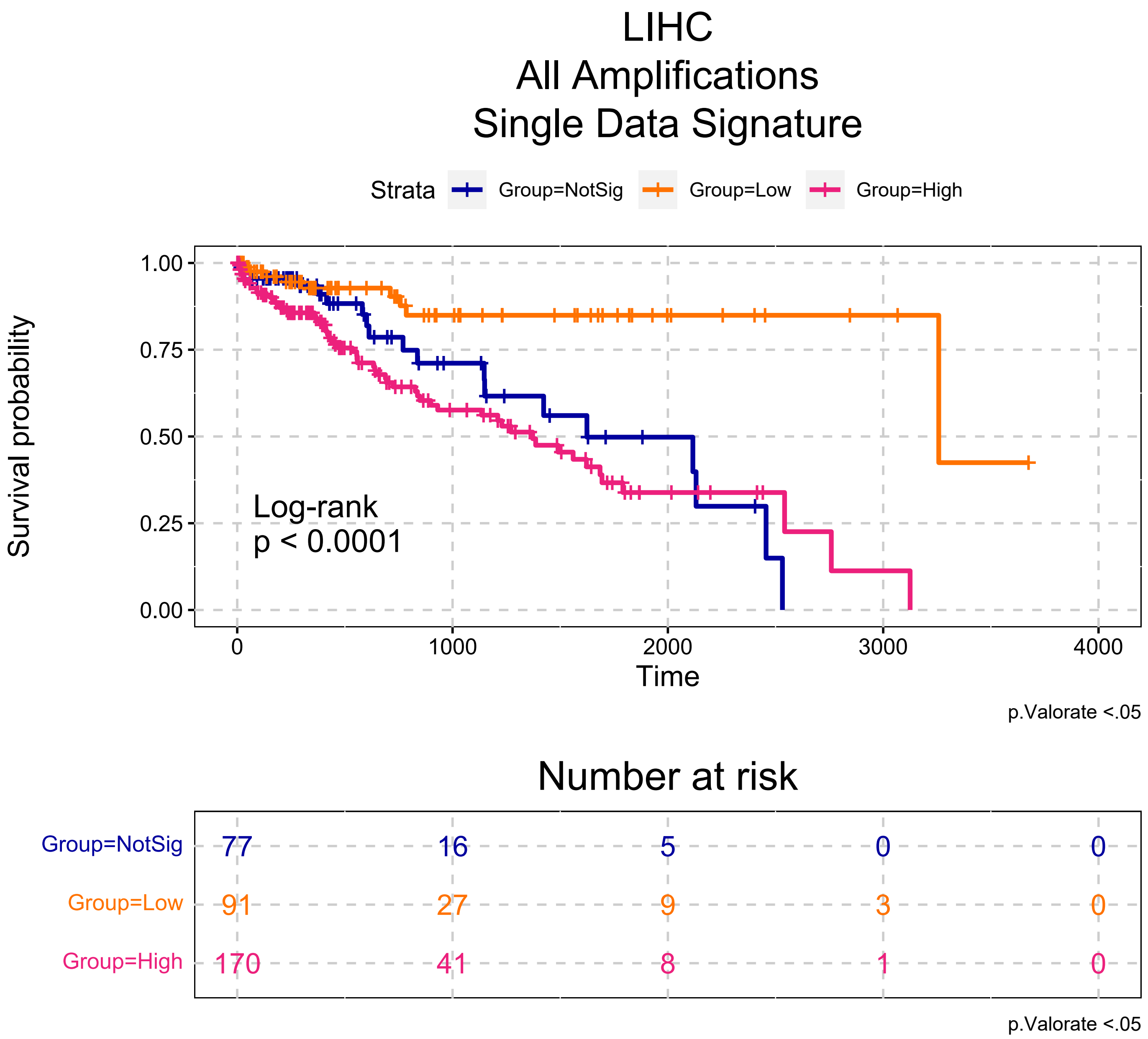

explanatory	beta	HR	L95	U95	p
Low	-1.35	0.26	0.11	0.60	0.00
High	0.28	1.32	0.79	2.22	0.29

n= 338, number of events =90
Score(logrank) test = p <.0001

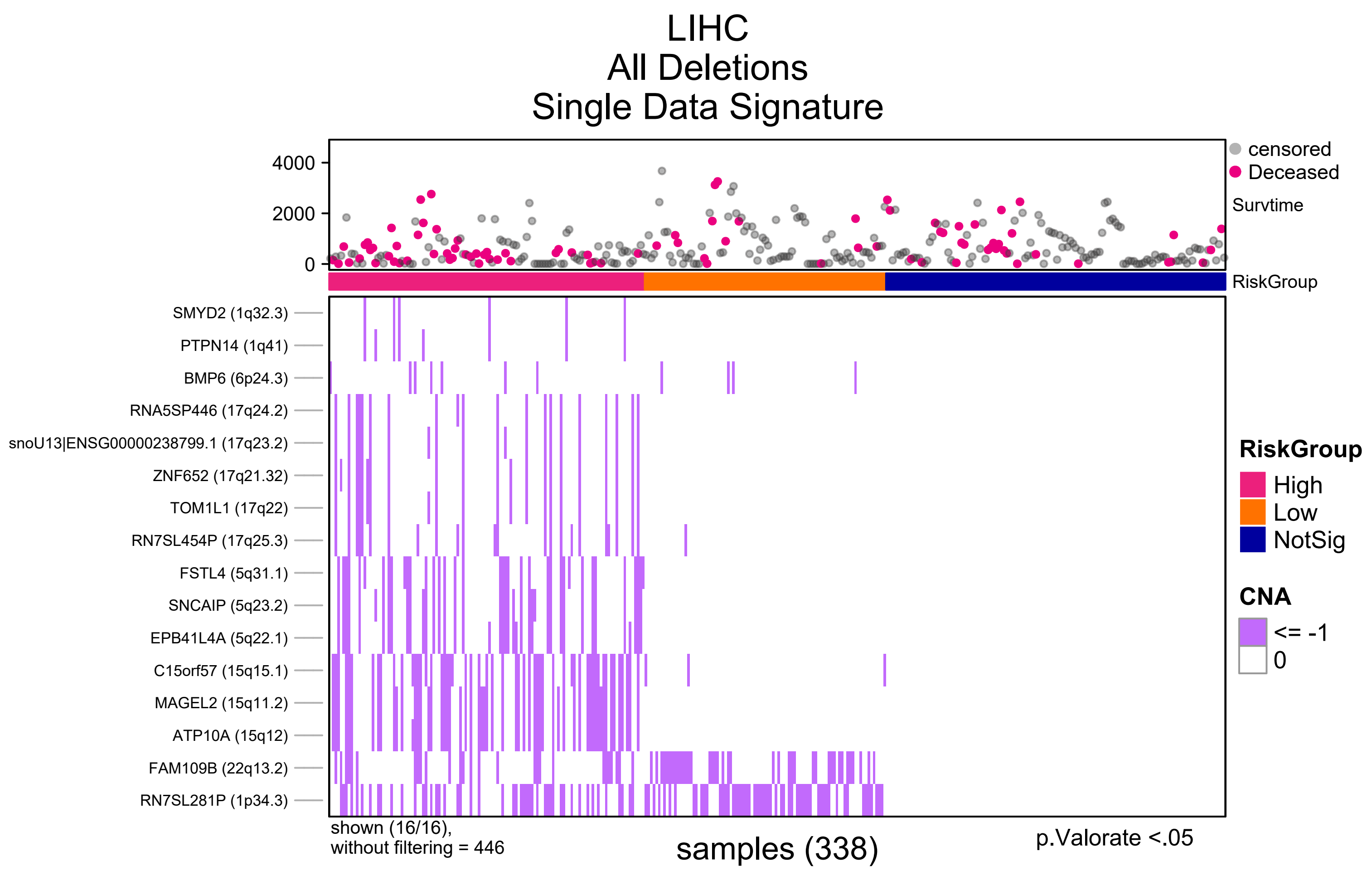
LIHC
All Deletions
Single Data Signature
4000
2000
0
censored
Deceased
Survtime
RiskGroup
SMYD2 (1q32.3)
PTPN14 (1q41)
BMP6 (6p24.3)
RNA5SP446 (17q24.2)
snoU13|ENSG00000238799.1 (17q23.2)
ZNF652 (17q21.32)
TOM1L1 (17q22)
RN7SL454P (17q25.3)
FSTL4 (5q31.1)
SNCAIP (5q23.2)
EPB41L4A (5q22.1)
C15orf57 (15q15.1)
MAGEL2 (15q11.2)
ATP10A (15q12)
FAM109B (22q13.2)
RN7SL281P (1p34.3)
RiskGroup
High
Low
NotSig
CNA
<= -1
0
shown (16/16),
without filtering = 446
samples (338)
p.Valorate <.05

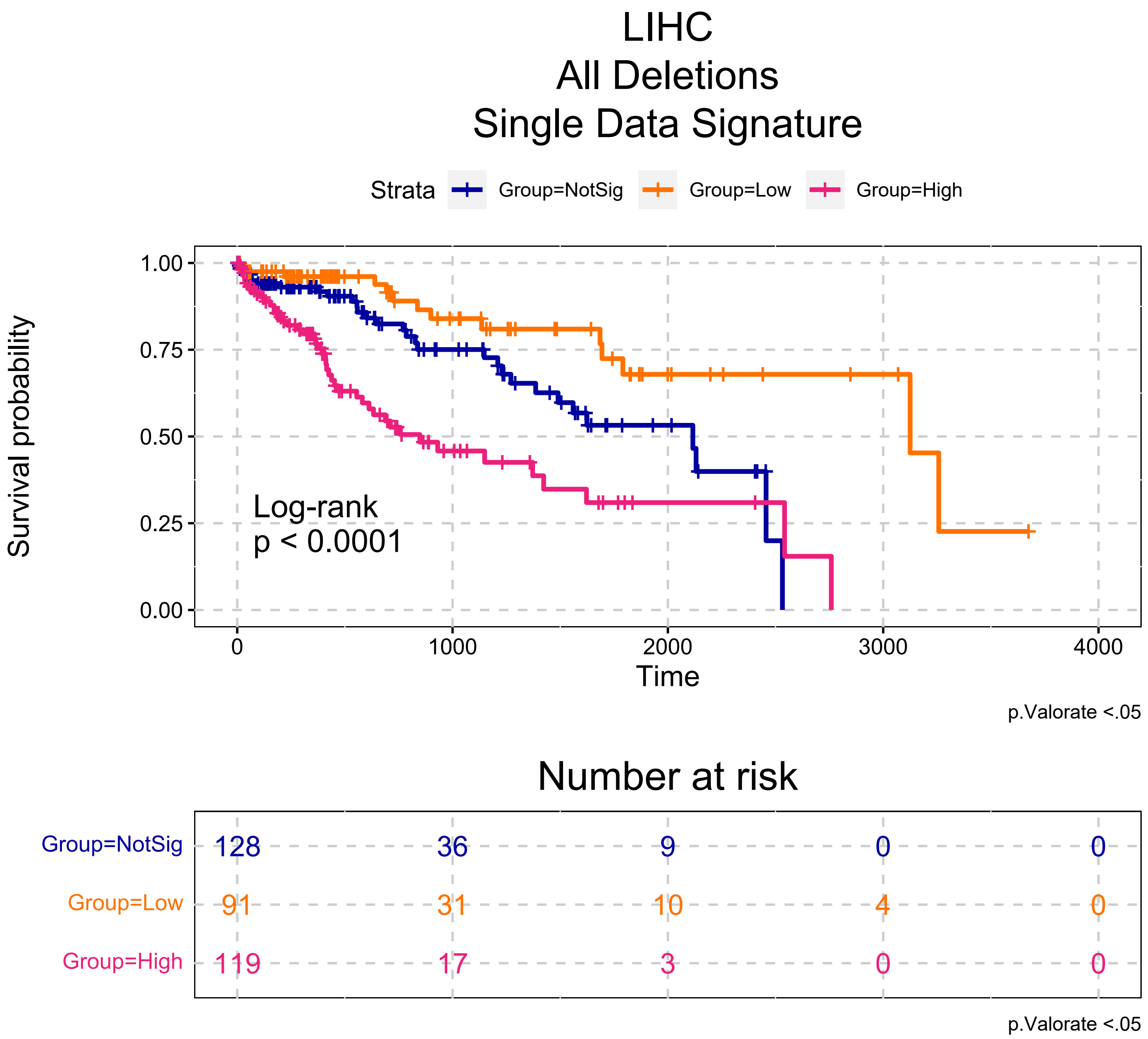

explanatory	beta	HR	L95	U95	p
Low	-0.86	0.42	0.21	0.83	0.01
High	0.76	2.13	1.34	3.38	0.00

n= 338, number of events =90
Score(logrank) test = p <.0001

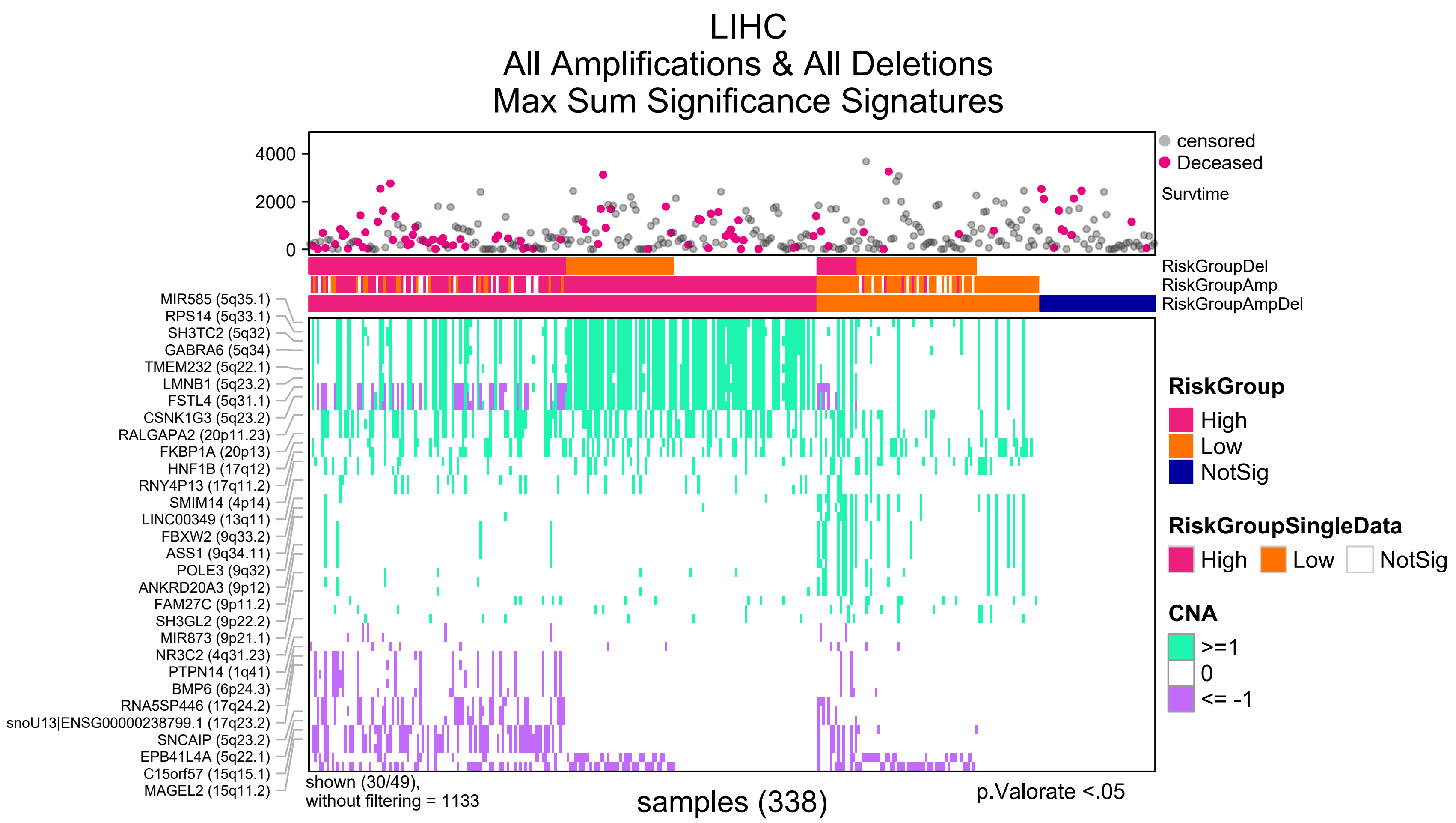

LIHC
All Amplifications & All Deletions
Max Sum Significance Signatures
4000
2000
0
censored
Deceased
Survtime
RiskGroupDel
RiskGroupAmp
RiskGroupAmpDel
MIR585 (5q35.1)
RPS14 (5q33.1)
SH3TC2 (5q32)
GABRA6 (5q34)
TMEM232 (5q22.1)
LMNB1 (5q23.2)
FSTL4 (5q31.1)
CSNK1G3 (5q23.2)
RALGAPA2 (20p11.23)
FKBP1A (20p13)
HNF1B (17q12)
RNY4P13 (17q11.2)
SMIM14 (4p14)
LINC00349 (13q11)
FBXW2 (9q33.2)
ASS1 (9q34.11)
POLE3 (9q32)
ANKRD20A3 (9p12)
FAM27C (9p11.2)
SH3GL2 (9p22.2)
MIR873 (9p21.1)
NR3C2 (4q31.23)
PTPN14 (1q41)
BMP6 (6p24.3)
RNA5SP446 (17q24.2)
snoU13|ENSG00000238799.1 (17q23.2)
SNCAIP (5q23.2)
EPB41L4A (5q22.1)
C15orf57 (15q15.1)
MAGEL2 (15q11.2)
RiskGroup
High
Low
NotSig
RiskGroupSingleData
High
Low
NotSig
CNA
>=1
0
<= -1
shown (30/49),
without filtering = 1133
samples (338)
p.Valorate <.05

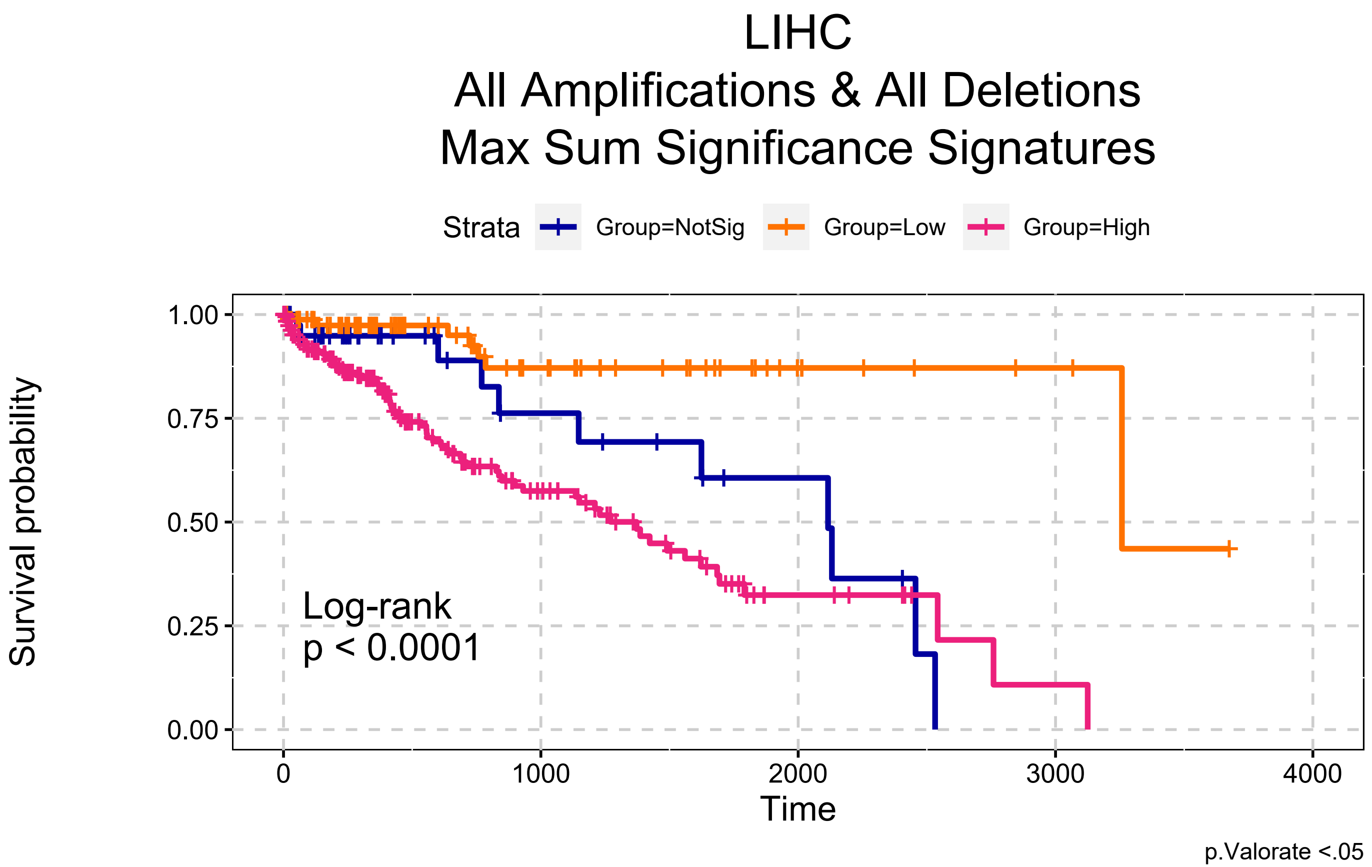

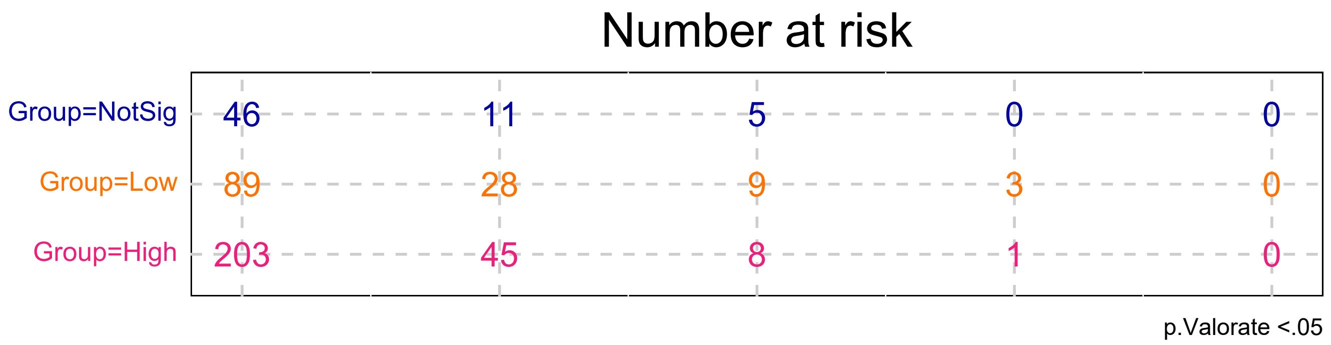

explanatory	beta	HR	L95	U95	p
Low	-1.54	0.21	0.08	0.58	0.00
High	0.44	1.56	0.82	2.95	0.17

n= 338, number of events =90
Score(logrank) test = p <.0001

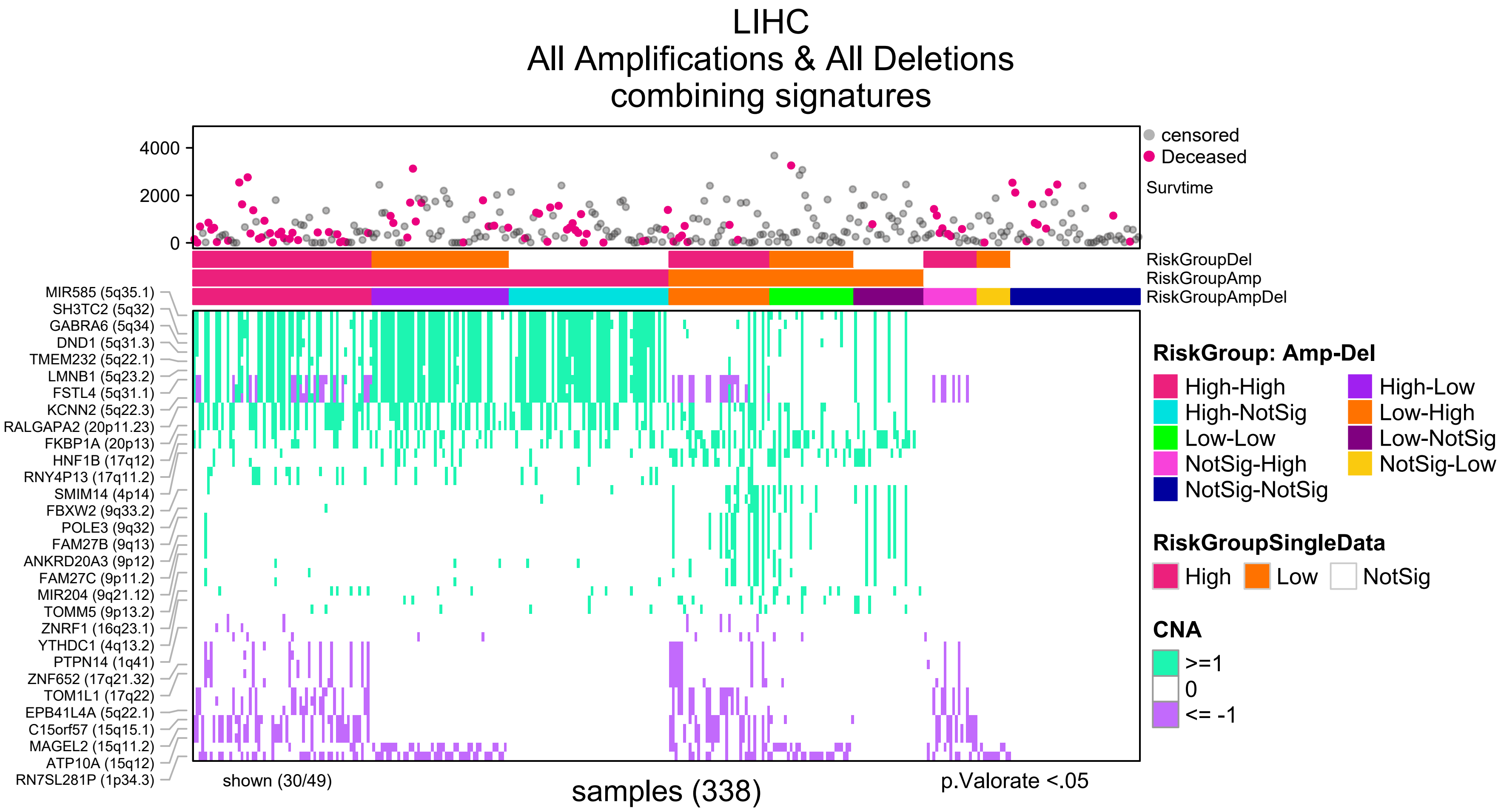

LIHC
All Amplifications & All Deletions
combining signatures
4000
2000
0
censored
Deceased
Survtime
RiskGroupDel
RiskGroupAmp
RiskGroupAmpDel
MIR585 (5q35.1)
SH3TC2 (5q32)
GABRA6 (5q34)
DND1 (5q31.3)
TMEM232 (5q22.1)
LMNB1 (5q23.2)
FSTL4 (5q31.1)
KCNN2 (5q22.3)
RALGAPA2 (20p11.23)
FKBP1A (20p13)
HNF1B (17q12)
RNY4P13 (17q11.2)
SMIM14 (4p14)
FBXW2 (9q33.2)
POLE3 (9q32)
FAM27B (9q13)
ANKRD20A3 (9p12)
FAM27C (9p11.2)
MIR204 (9q21.12)
TOMM5 (9p13.2)
ZNRF1 (16q23.1)
YTHDC1 (4q13.2)
PTPN14 (1q41)
ZNF652 (17q21.32)
TOM1L1 (17q22)
EPB41L4A (5q22.1)
C15orf57 (15q15.1)
MAGEL2 (15q11.2)
ATP10A (15q12)
RN7SL281P (1p34.3)
shown (30/49)
samples (338)
p.Valorate <.05
RiskGroup: Amp-Del
High-High
High-Low
High-NotSig
Low-High
Low-Low
Low-NotSig
NotSig-High
NotSig-Low
NotSig-NotSig
RiskGroupSingleData
High
Low
NotSig
CNA
>=1
0
<= -1

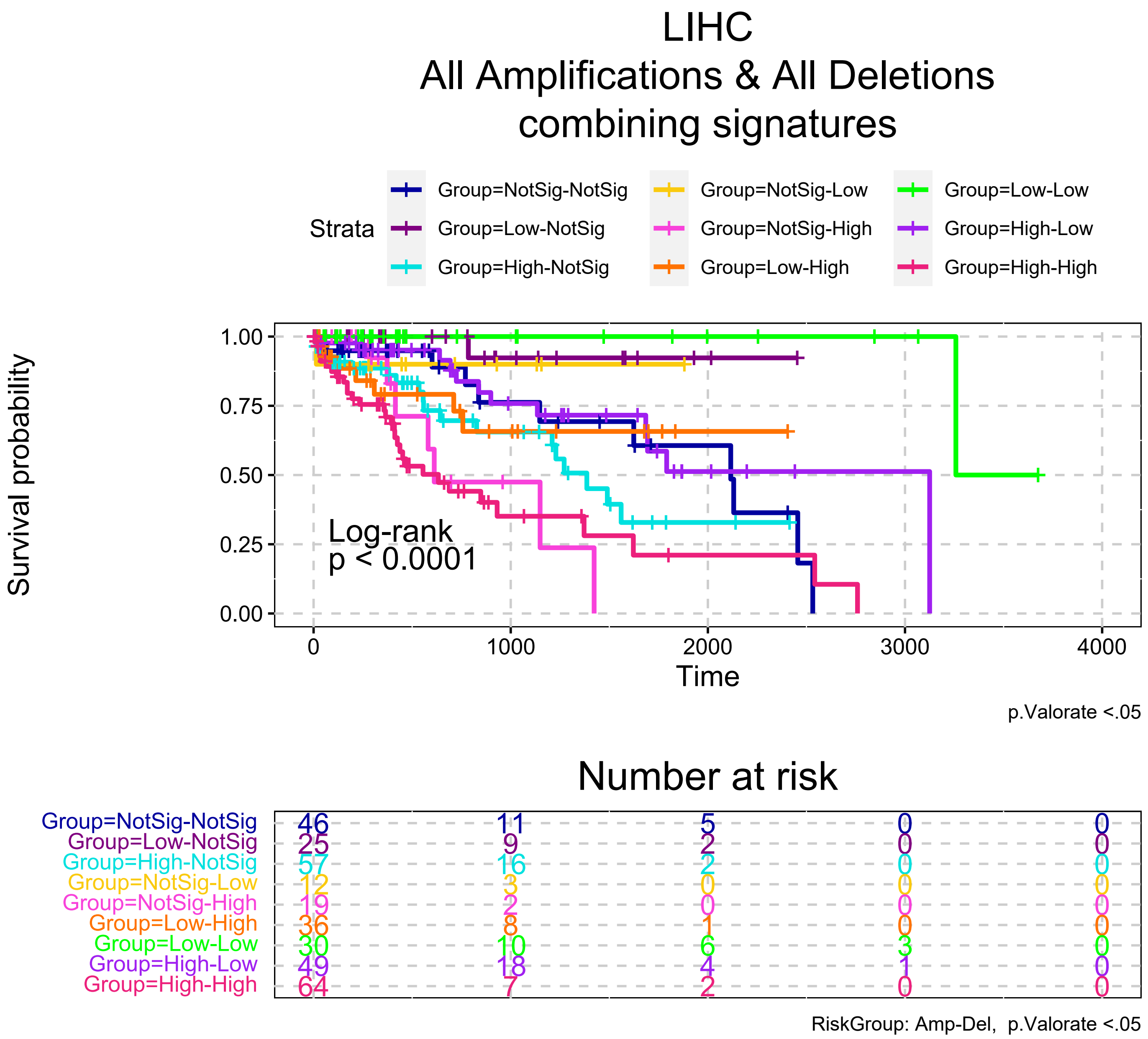

explanatory	beta	HR	L95	U95	p
Low-NotSig	-2.06	0.13	0.02	0.99	0.05
High-NotSig	0.38	1.47	0.69	3.10	0.31
NotSig-Low	-0.93	0.39	0.05	3.06	0.37
NotSig-High	0.88	2.40	0.92	6.28	0.07
Low-High	-0.01	0.99	0.38	2.55	0.98
Low-Low	-18.61	0.00	0.00	Inf	0.99
High-Low	-0.36	0.70	0.30	1.62	0.40
High-High	0.98	2.67	1.33	5.37	0.01

n= 338, number of events =90
Score(logrank) test = p <.0001

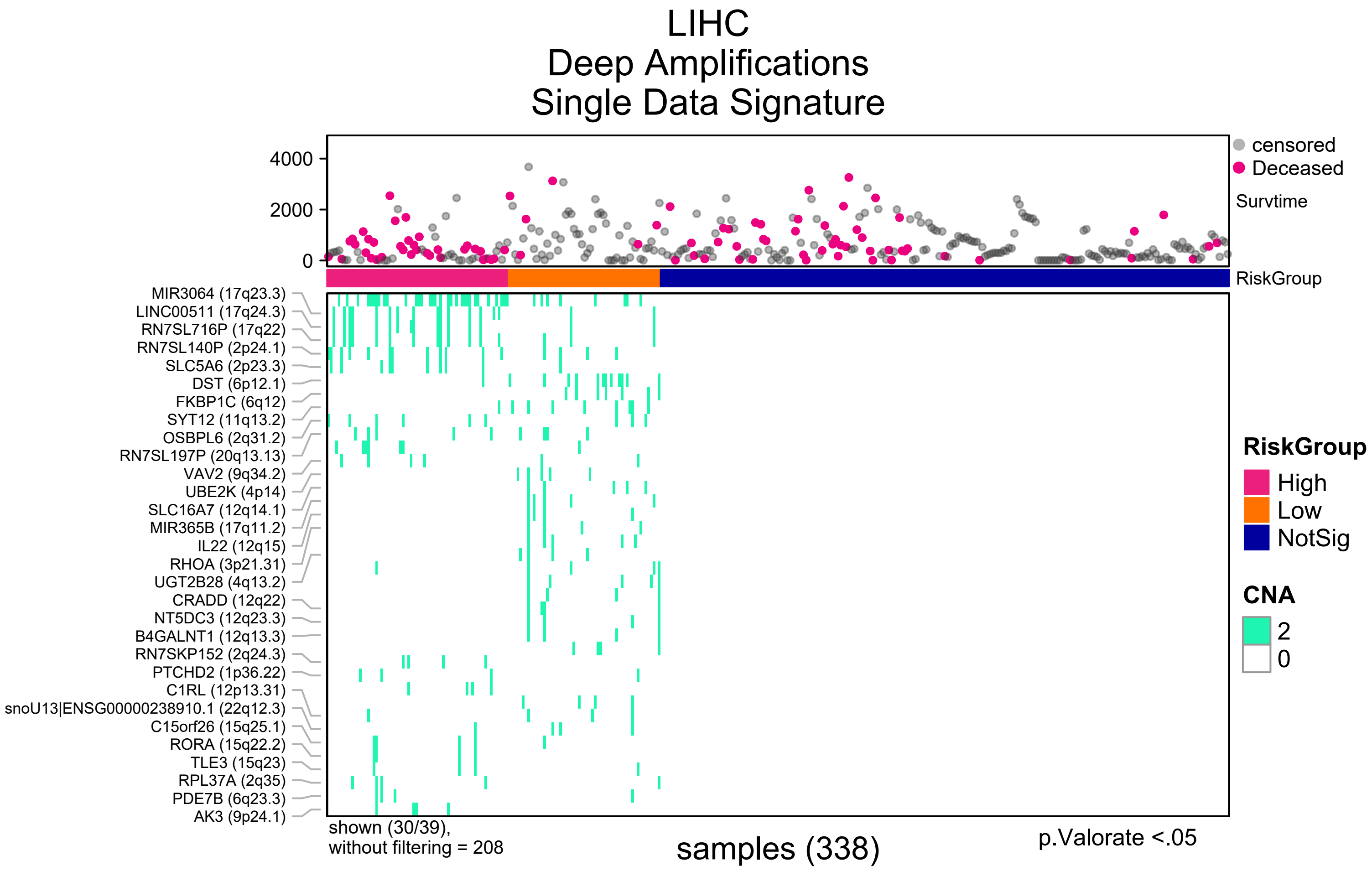

LIHC
Deep Amplifications
Single Data Signature
4000
2000
0
censored
Deceased
Survtime
RiskGroup
MIR3064 (17q23.3)
LINC00511 (17q24.3)
RN7SL716P (17q22)
RN7SL140P (2p24.1)
SLC5A6 (2p23.3)
DST (6p12.1)
FKBP1C (6q12)
SYT12 (11q13.2)
OSBPL6 (2q31.2)
RN7SL197P (20q13.13)
VAV2 (9q34.2)
UBE2K (4p14)
SLC16A7 (12q14.1)
MIR365B (17q11.2)
IL22 (12q15)
RHOA (3p21.31)
UGT2B28 (4q13.2)
CRADD (12q22)
NT5DC3 (12q23.3)
B4GALNT1 (12q13.3)
RN7SKP152 (2q24.3)
PTCHD2 (1p36.22)
C1RL (12p13.31)
snoU13|ENSG00000238910.1 (22q12.3)
C15orf26 (15q25.1)
RORA (15q22.2)
TLE3 (15q23)
RPL37A (2q35)
PDE7B (6q23.3)
AK3 (9p24.1)
RiskGroup
High
Low
NotSig
CNA
2
0
shown (30/39),
without filtering = 208
samples (338)
p.Valorate <.05

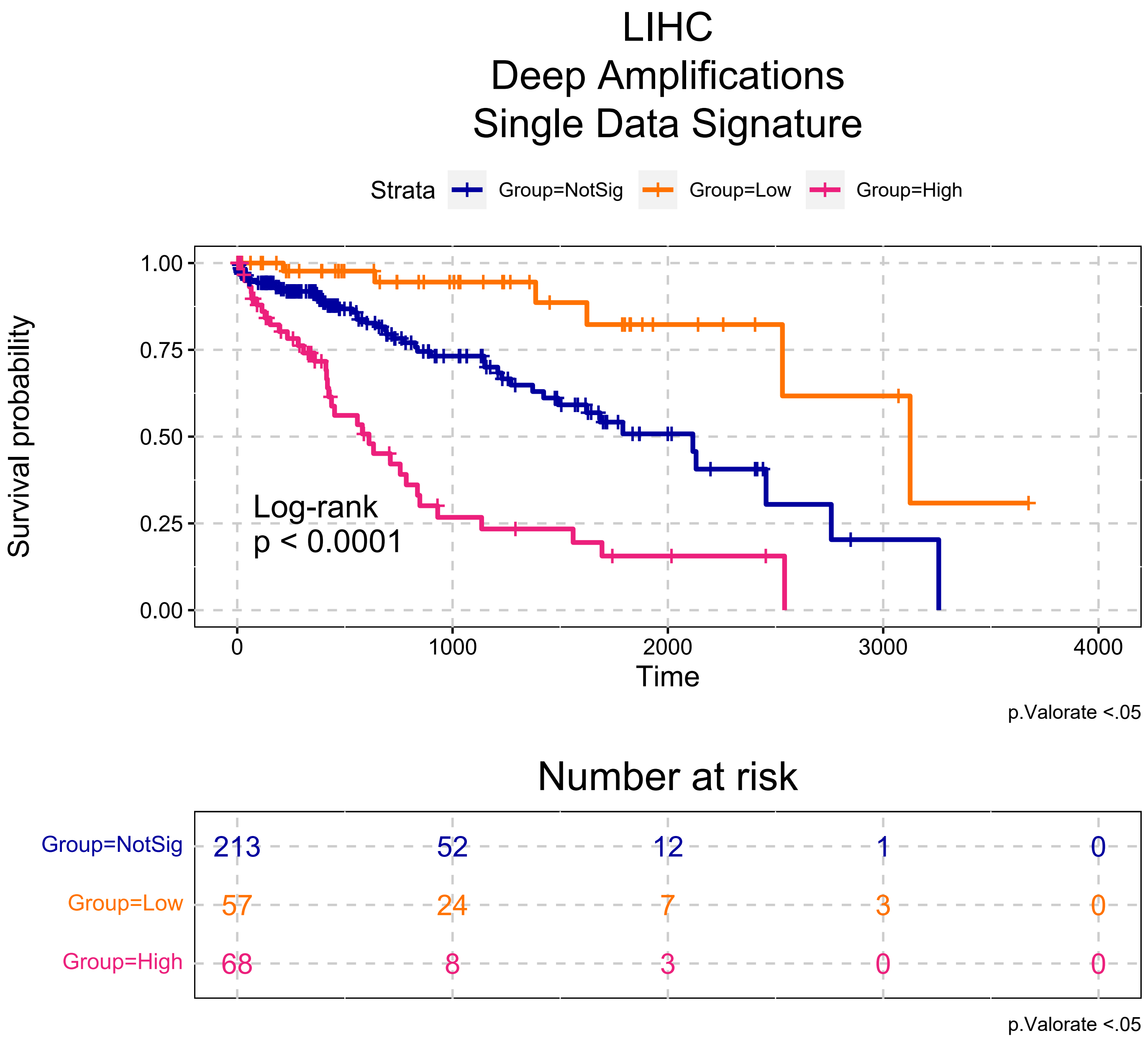

explanatory	beta	HR	L95	U95	p
Low	-1.29	0.27	0.12	0.65	0.00
High	1.13	3.08	1.99	4.78	0.00

n= 338, number of events =90
Score(logrank) test = p <.0001

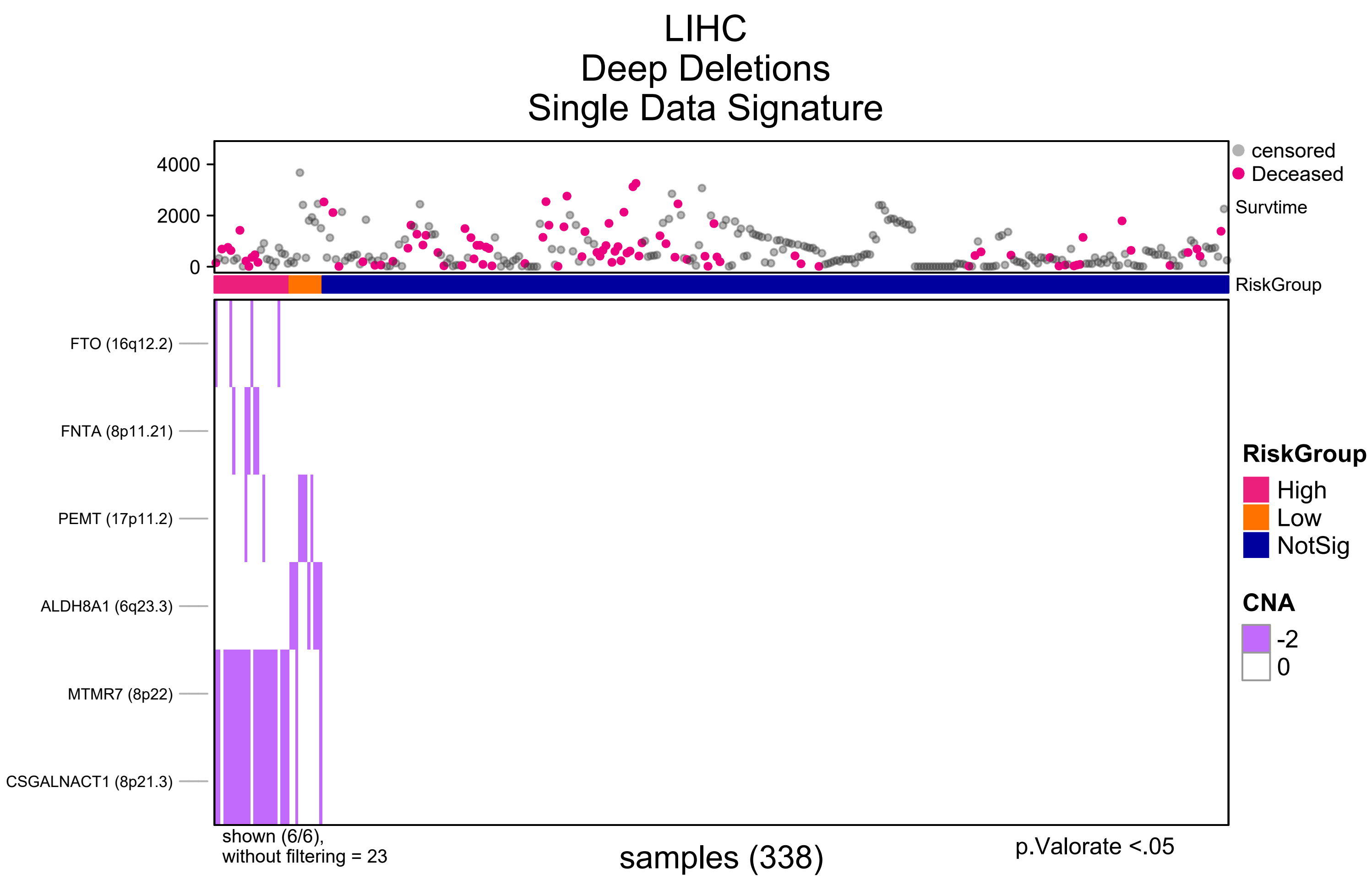

LIHC
Deep Deletions
Single Data Signature
4000
2000
0
censored
Deceased
Survtime
RiskGroup
FTO (16q12.2)
FNTA (8p11.21)
PEMT (17p11.2)
ALDH8A1 (6q23.3)
MTMR7 (8p22)
CSGALNACT1 (8p21.3)
RiskGroup
High
Low
NotSig
CNA
-2
0
shown (6/6),
without filtering = 23
samples (338)
p.Valorate <.05

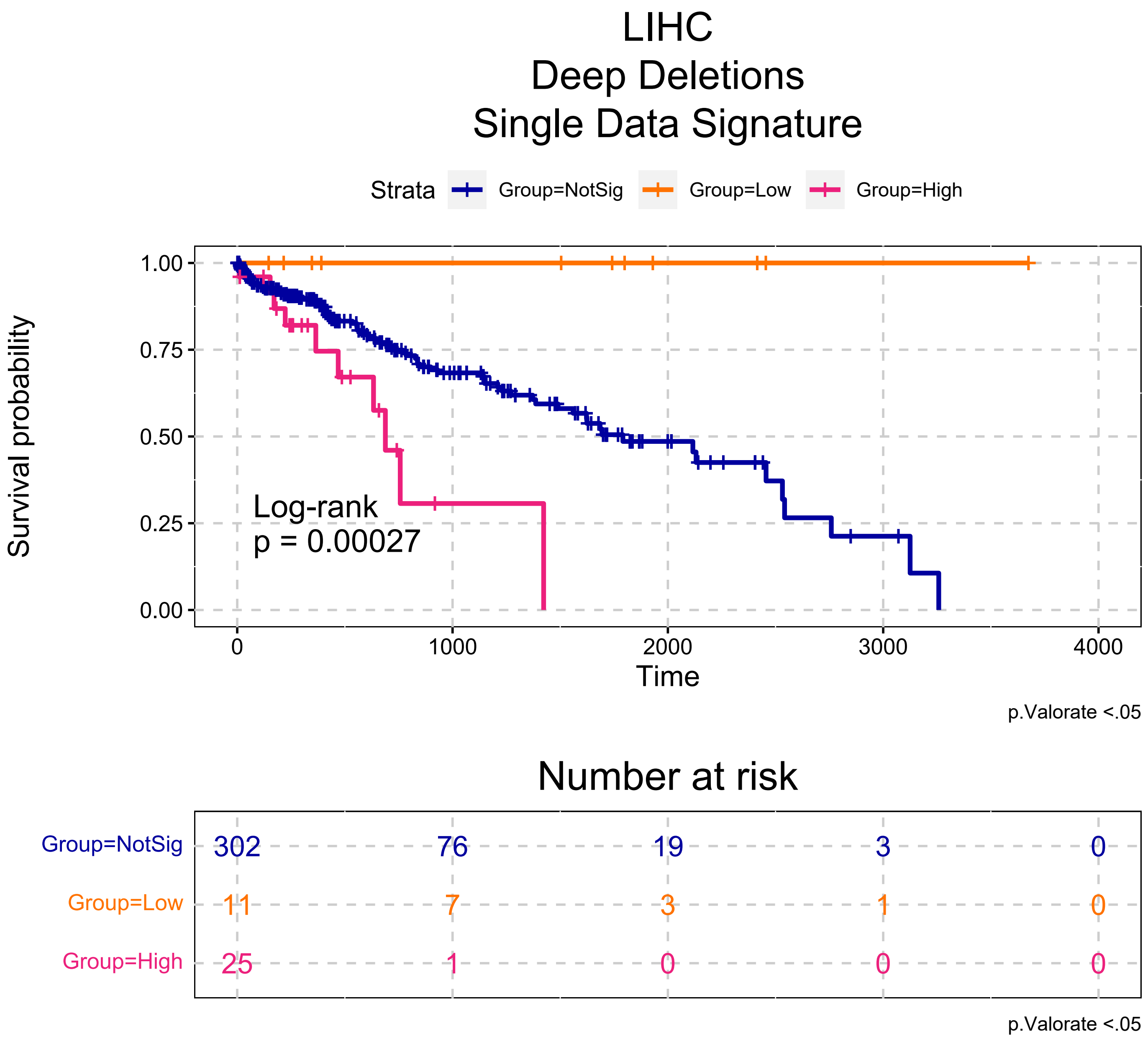

explanatory	beta	HR	L95	U95	p
Low	-18.26	0.00	0.00	Inf	1.00
High	0.95	2.58	1.31	5.06	0.01

n= 338, number of events =90
Score(logrank) test = 0

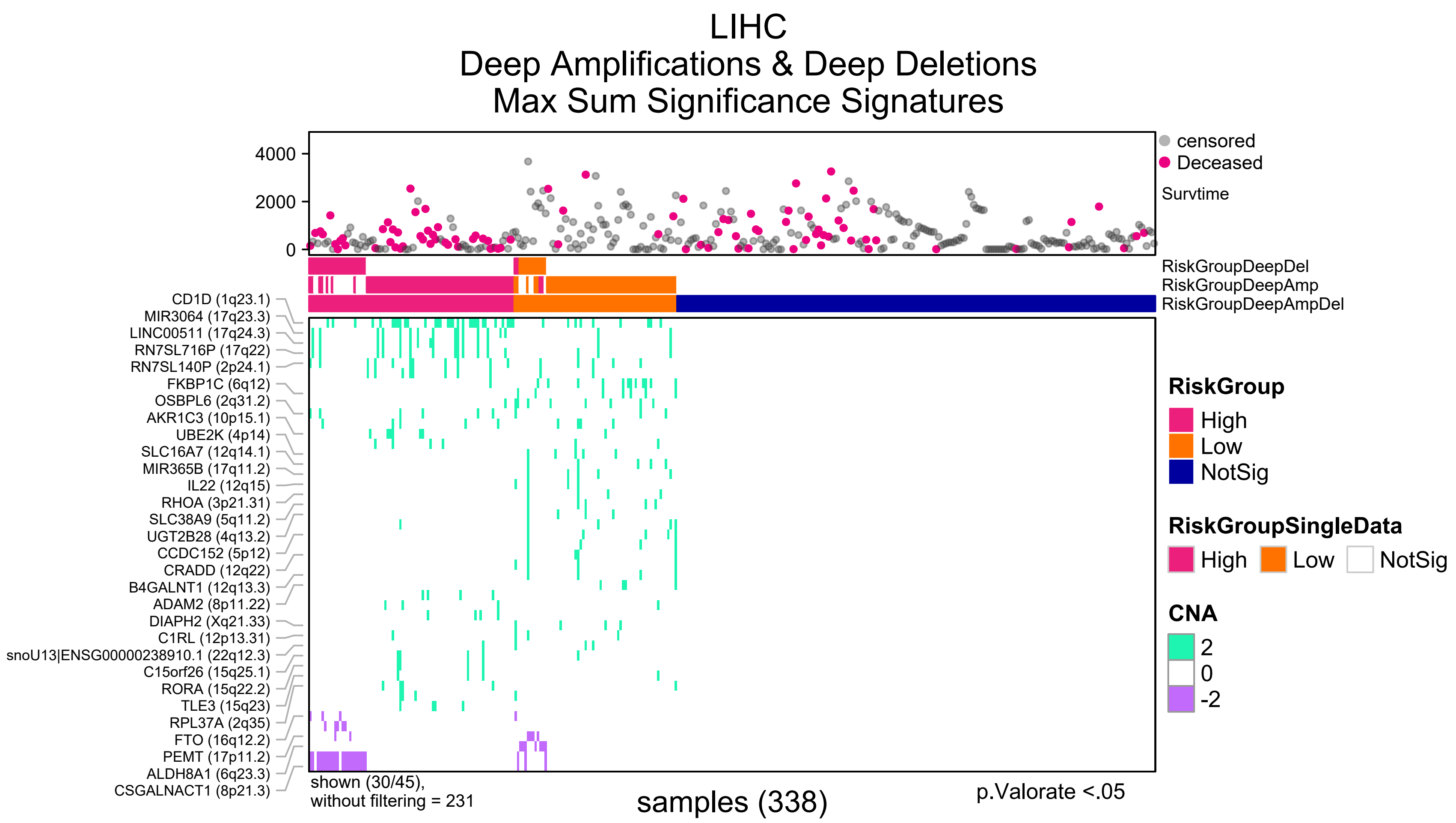
LIHC
Deep Amplifications & Deep Deletions
Max Sum Significance Signatures
4000
2000
0
censored
Deceased
Survtime
RiskGroupDeepDel
RiskGroupDeepAmp
RiskGroupDeepAmpDel
CD1D (1q23.1)
MIR3064 (17q23.3)
LINC00511 (17q24.3)
RN7SL716P (17q22)
RN7SL140P (2p24.1)
FKBP1C (6q12)
OSBPL6 (2q31.2)
AKR1C3 (10p15.1)
UBE2K (4p14)
SLC16A7 (12q14.1)
MIR365B (17q11.2)
IL22 (12q15)
RHOA (3p21.31)
SLC38A9 (5q11.2)
UGT2B28 (4q13.2)
CCDC152 (5p12)
CRADD (12q22)
B4GALNT1 (12q13.3)
ADAM2 (8p11.22)
DIAPH2 (Xq21.33)
C1RL (12p13.31)
snoU13|ENSG00000238910.1 (22q12.3)
C15orf26 (15q25.1)
RORA (15q22.2)
TLE3 (15q23)
RPL37A (2q35)
FTO (16q12.2)
PEMT (17p11.2)
ALDH8A1 (6q23.3)
CSGALNACT1 (8p21.3)
RiskGroup
High
Low
NotSig
RiskGroupSingleData
High
Low
NotSig
CNA
2
0
-2
shown (30/45),
without filtering = 231
samples (338)
p.Valorate <.05

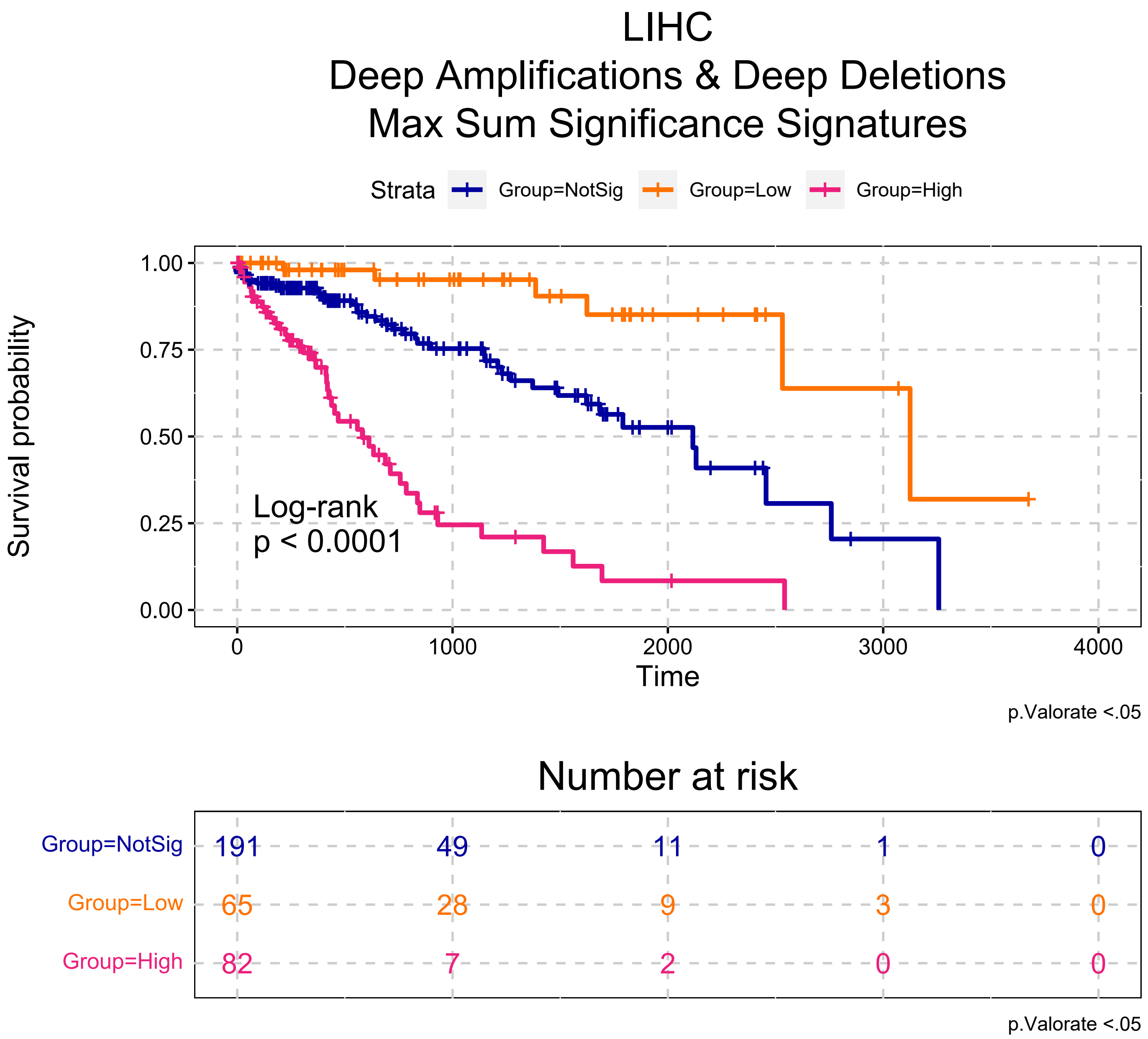

explanatory	beta	HR	L95	U95	p
Low	-1.38	0.25	0.11	0.60	0.00
High	1.35	3.85	2.48	5.98	0.00

n= 338, number of events =90
Score(logrank) test = p <.0001

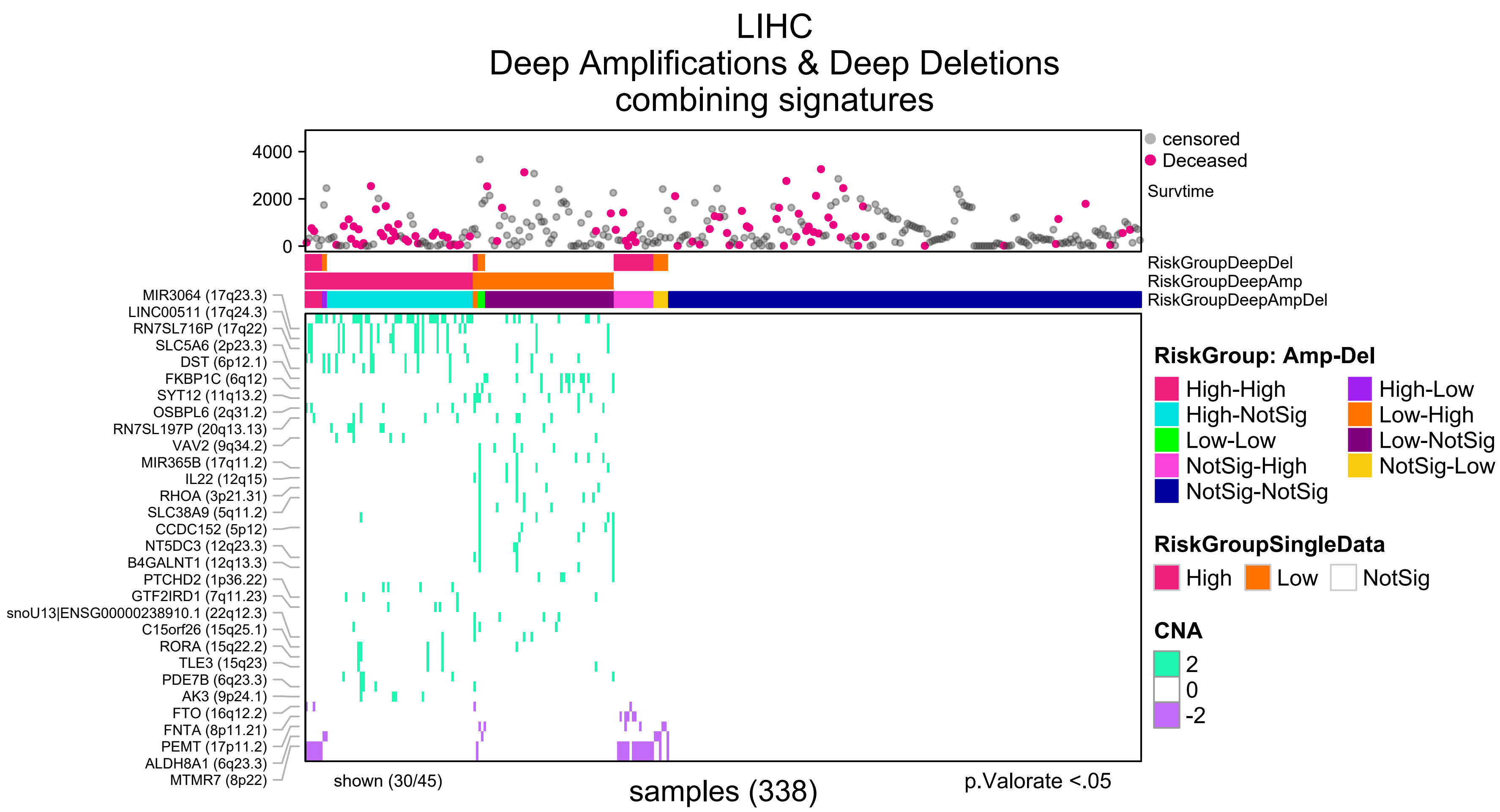

LIHC
Deep Amplifications & Deep Deletions
combining signatures
4000
2000
0
censored
Deceased
Survtime
RiskGroupDeepDel
RiskGroupDeepAmp
RiskGroupDeepAmpDel
MIR3064 (17q23.3)
LINC00511 (17q24.3)
RN7SL716P (17q22)
SLC5A6 (2p23.3)
DST (6p12.1)
FKBP1C (6q12)
SYT12 (11q13.2)
OSBPL6 (2q31.2)
RN7SL197P (20q13.13)
VAV2 (9q34.2)
MIR365B (17q11.2)
IL22 (12q15)
RHOA (3p21.31)
SLC38A9 (5q11.2)
CCDC152 (5p12)
NT5DC3 (12q23.3)
B4GALNT1 (12q13.3)
PTCHD2 (1p36.22)
GTF2IRD1 (7q11.23)
snoU13|ENSG00000238910.1 (22q12.3)
C15orf26 (15q25.1)
RORA (15q22.2)
TLE3 (15q23)
PDE7B (6q23.3)
AK3 (9p24.1)
FTO (16q12.2)
FNTA (8p11.21)
PEMT (17p11.2)
ALDH8A1 (6q23.3)
MTMR7 (8p22)
RiskGroup: Amp-Del
High-High
High-NotSig
Low-Low
NotSig-High
NotSig-NotSig
High-Low
Low-High
Low-NotSig
NotSig-Low
RiskGroupSingleData
High
Low
NotSig
CNA
2
0
-2
shown (30/45)
samples (338)
p.Valorate <.05

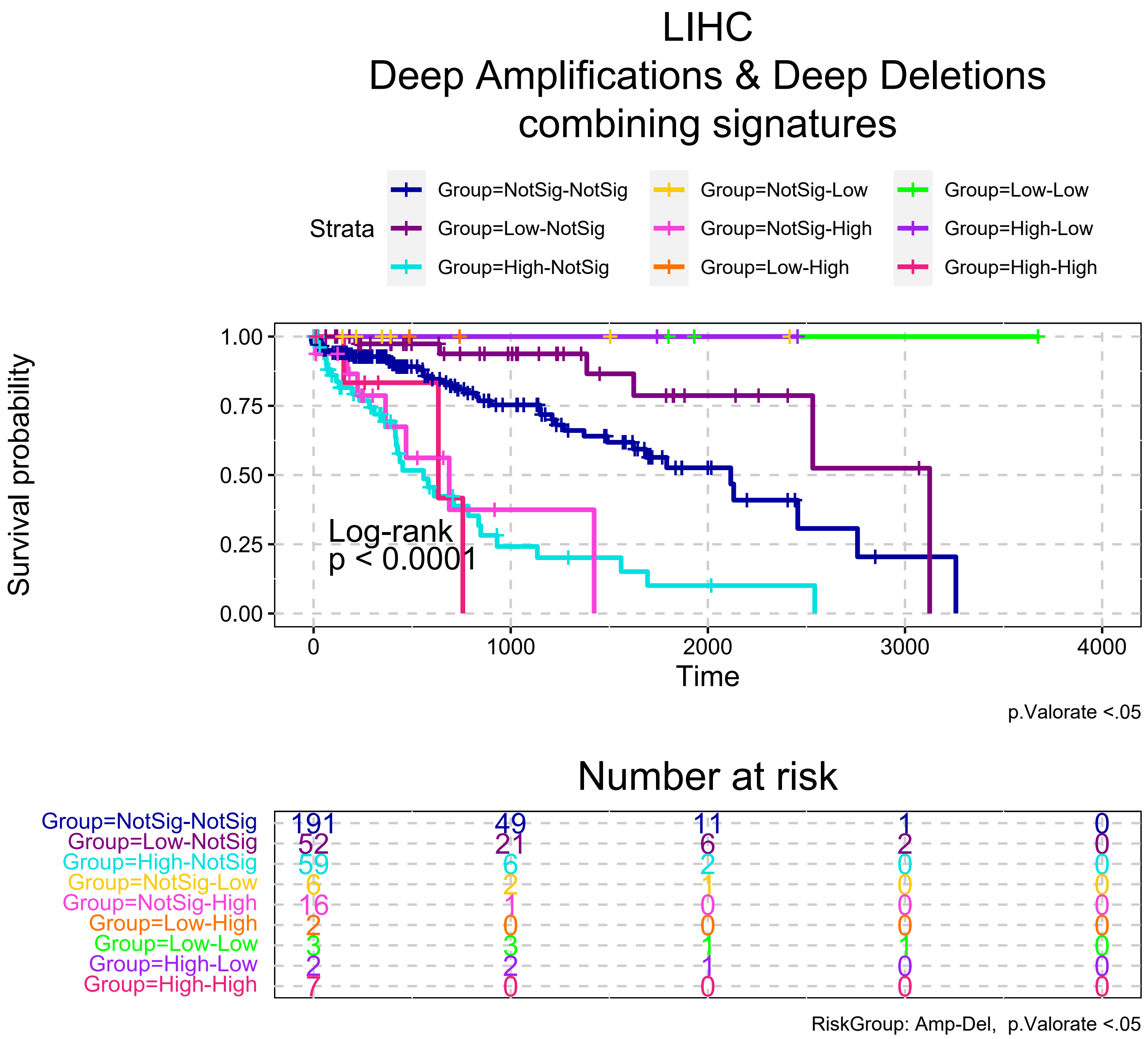

explanatory	beta	HR	L95	U95	p
Low-NotSig	-1.01	0.37	0.15	0.87	0.02
High-NotSig	1.36	3.90	2.43	6.23	0.00
NotSig-Low	-17.46	0.00	0.00	Inf	1.00
NotSig-High	1.30	3.66	1.62	8.28	0.00
Low-High	-17.38	0.00	0.00	Inf	1.00
Low-Low	-17.63	0.00	0.00	Inf	1.00
High-Low	-17.53	0.00	0.00	Inf	1.00
High-High	1.48	4.40	1.34	14.45	0.01

n= 338, number of events =90
Score(logrank) test = p <.0001
