# Supplementary material for: Systematic Modeling of Risk-Associated Copy Number Alterations in Cancer
Source: Int J Mol Sci. 2024 Sep 27;25(19):10455. doi: 10.3390/ijms251910455 (PMC11477427; doi:10.3390/ijms251910455)
Supplement: Supplementary file 1 [file ijms-25-10455-s001.zip › LUADSignatureV12-sinSombreado.pdf]

LUAD
All Amplifications
Single Data Signature

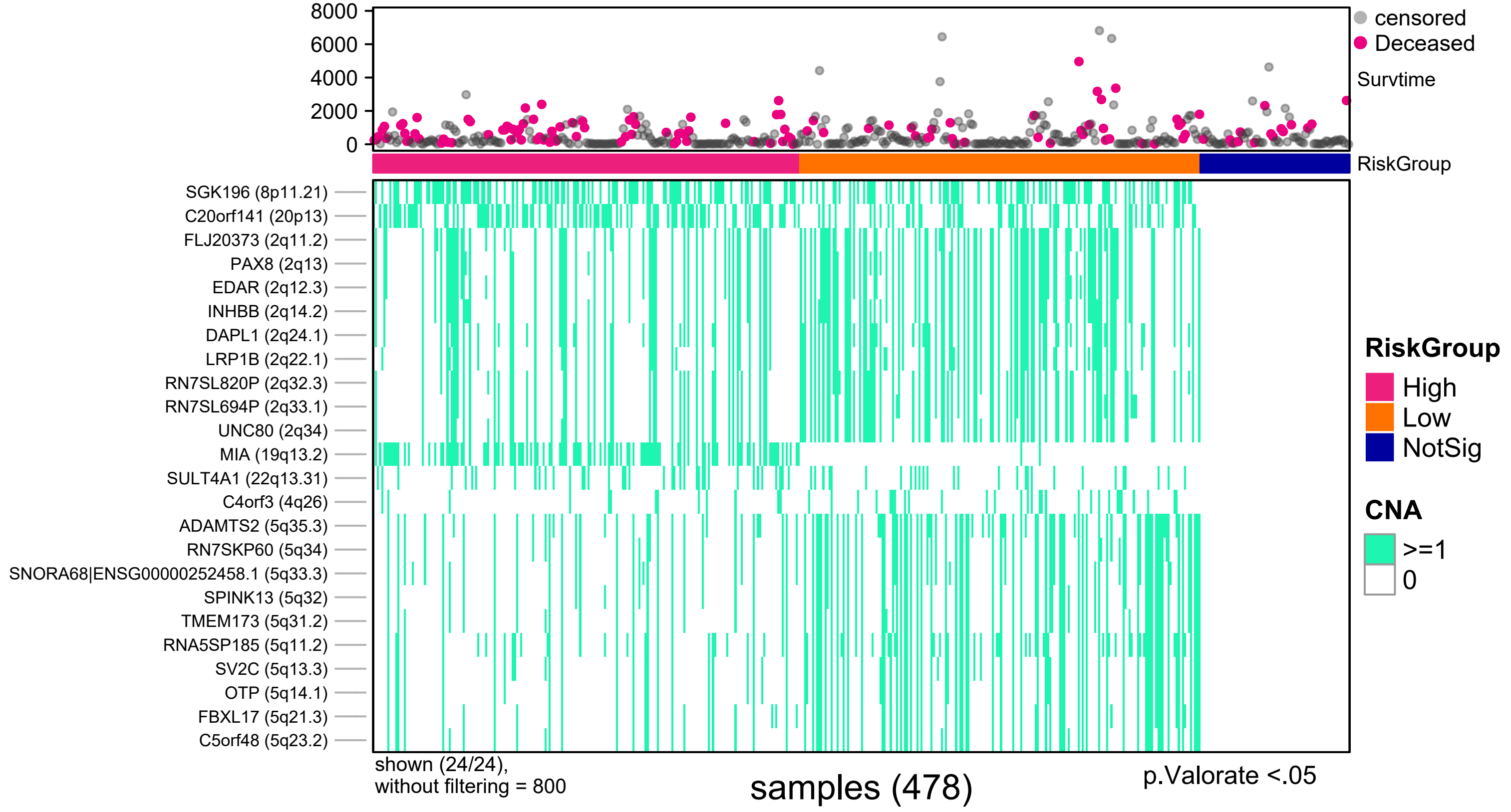

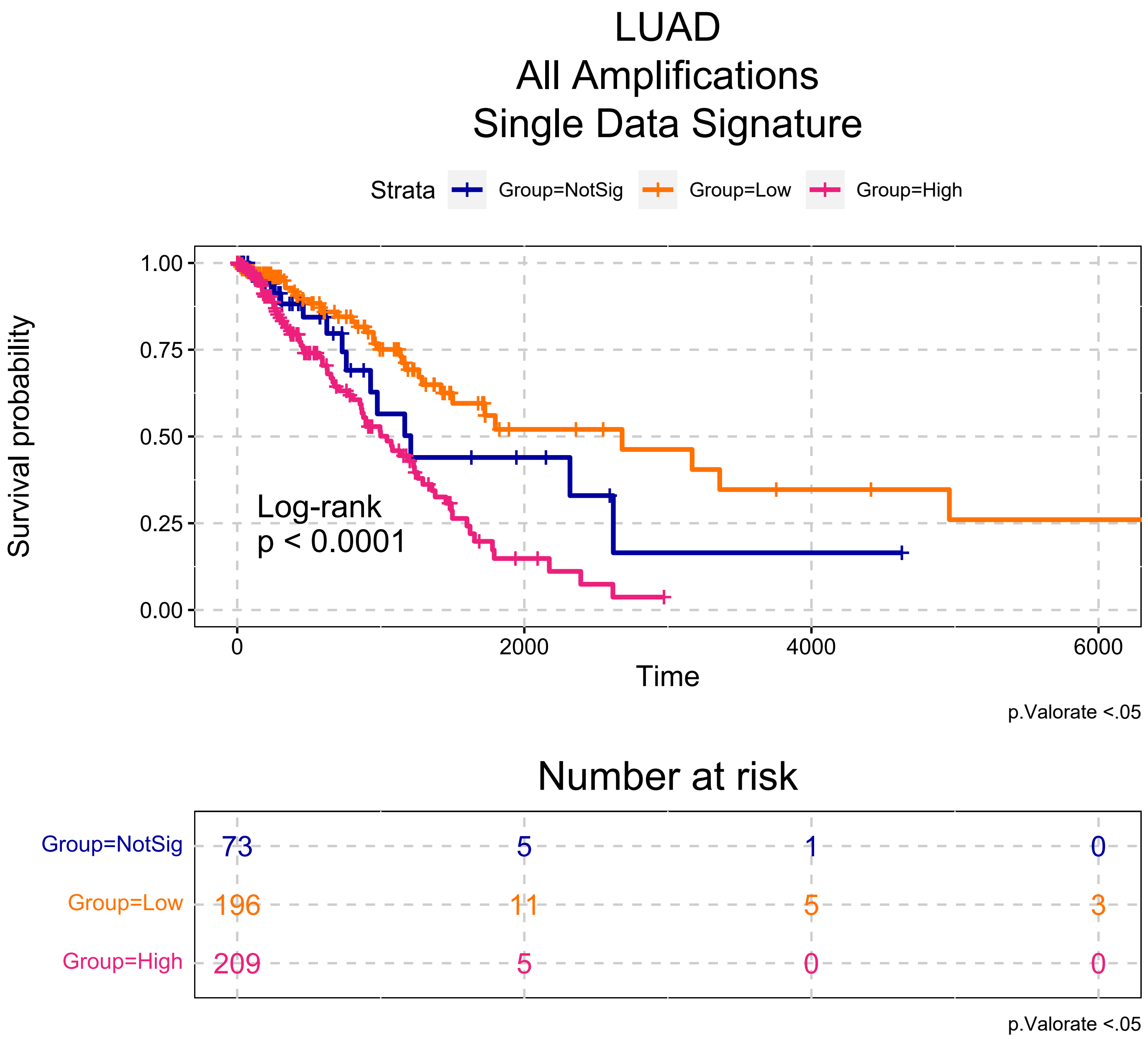

explanatory	beta	HR	L95	U95	p
Low	-0.46	0.63	0.34	1.16	0.14
High	0.57	1.76	1.00	3.09	0.05

n= 478, number of events =122
Score(logrank) test = p <.0001

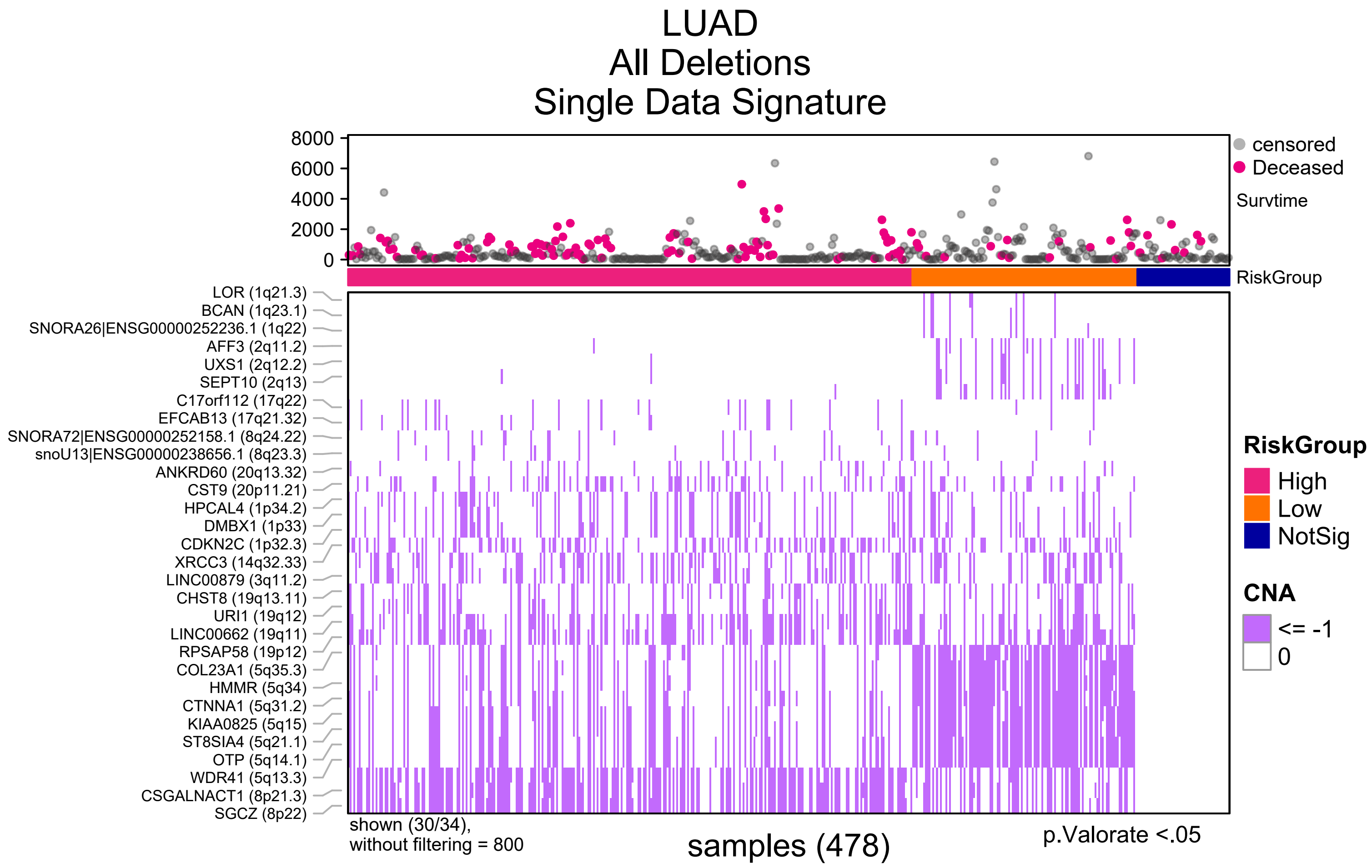
LUAD
All Deletions
Single Data Signature
8000
6000
4000
2000
0
censored
Deceased
Survtime
RiskGroup
LOR (1q21.3)
BCAN (1q23.1)
SNORA26|ENSG00000252236.1 (1q22)
AFF3 (2q11.2)
UXS1 (2q12.2)
SEPT10 (2q13)
C17orf112 (17q22)
EFCAB13 (17q21.32)
SNORA72|ENSG00000252158.1 (8q24.22)
snoU13|ENSG00000238656.1 (8q23.3)
ANKRD60 (20q13.32)
CST9 (20p11.21)
HPCAL4 (1p34.2)
DMBX1 (1p33)
CDKN2C (1p32.3)
XRCC3 (14q32.33)
LINC00879 (3q11.2)
CHST8 (19q13.11)
URI1 (19q12)
LINC00662 (19q11)
RPSAP58 (19p12)
COL23A1 (5q35.3)
HMMR (5q34)
CTNNA1 (5q31.2)
KIAA0825 (5q15)
ST8SIA4 (5q21.1)
OTP (5q14.1)
WDR41 (5q13.3)
CSGALNACT1 (8p21.3)
SGCZ (8p22)
RiskGroup
High
Low
NotSig
CNA
<= -1
0
shown (30/34),
without filtering = 800
samples (478)
p.Valorate <.05

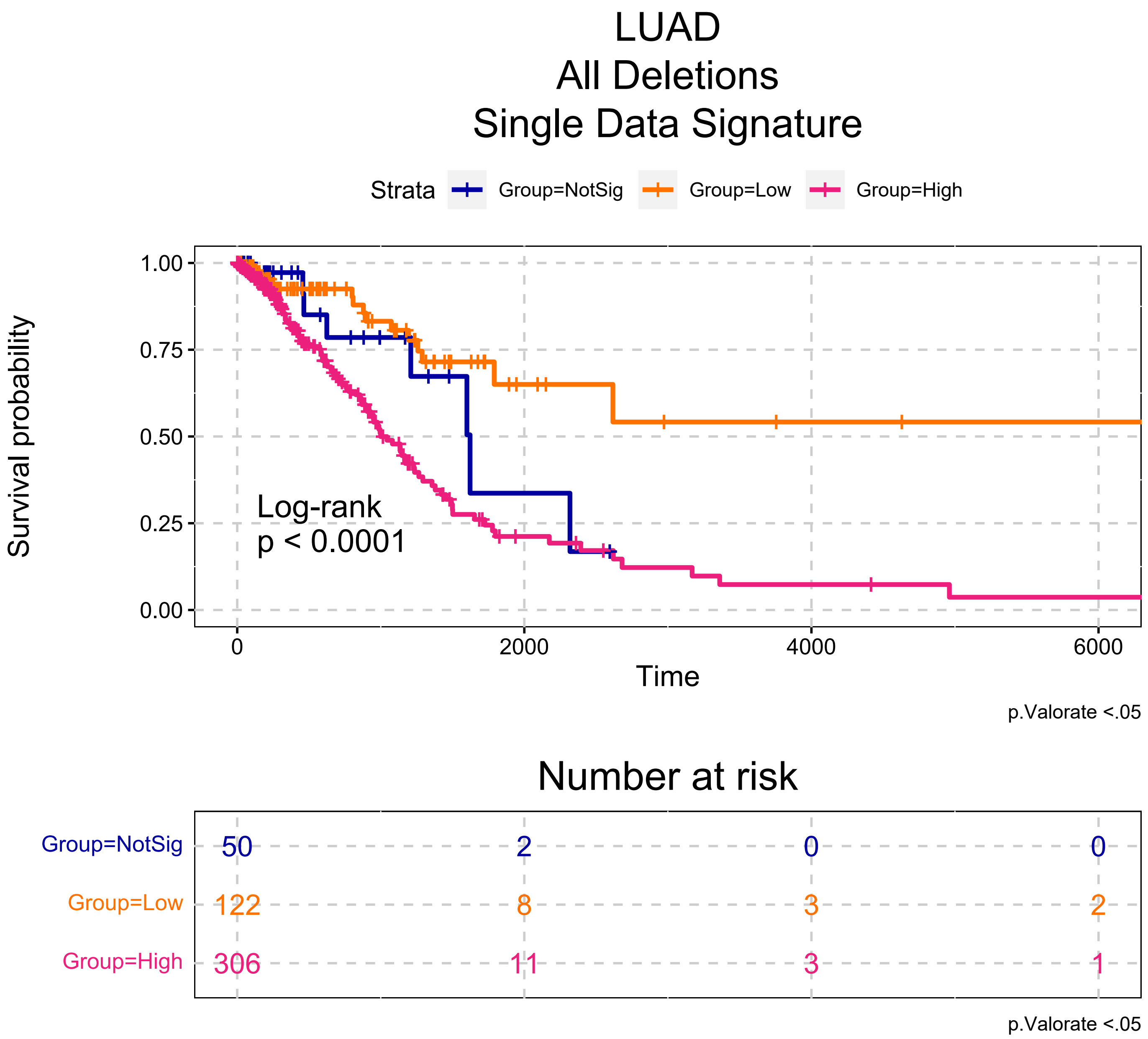

explanatory	beta	HR	L95	U95	p
Low	-0.64	0.53	0.22	1.23	0.14
High	0.62	1.87	0.91	3.85	0.09

n= 478, number of events =122
Score(logrank) test = p <.0001

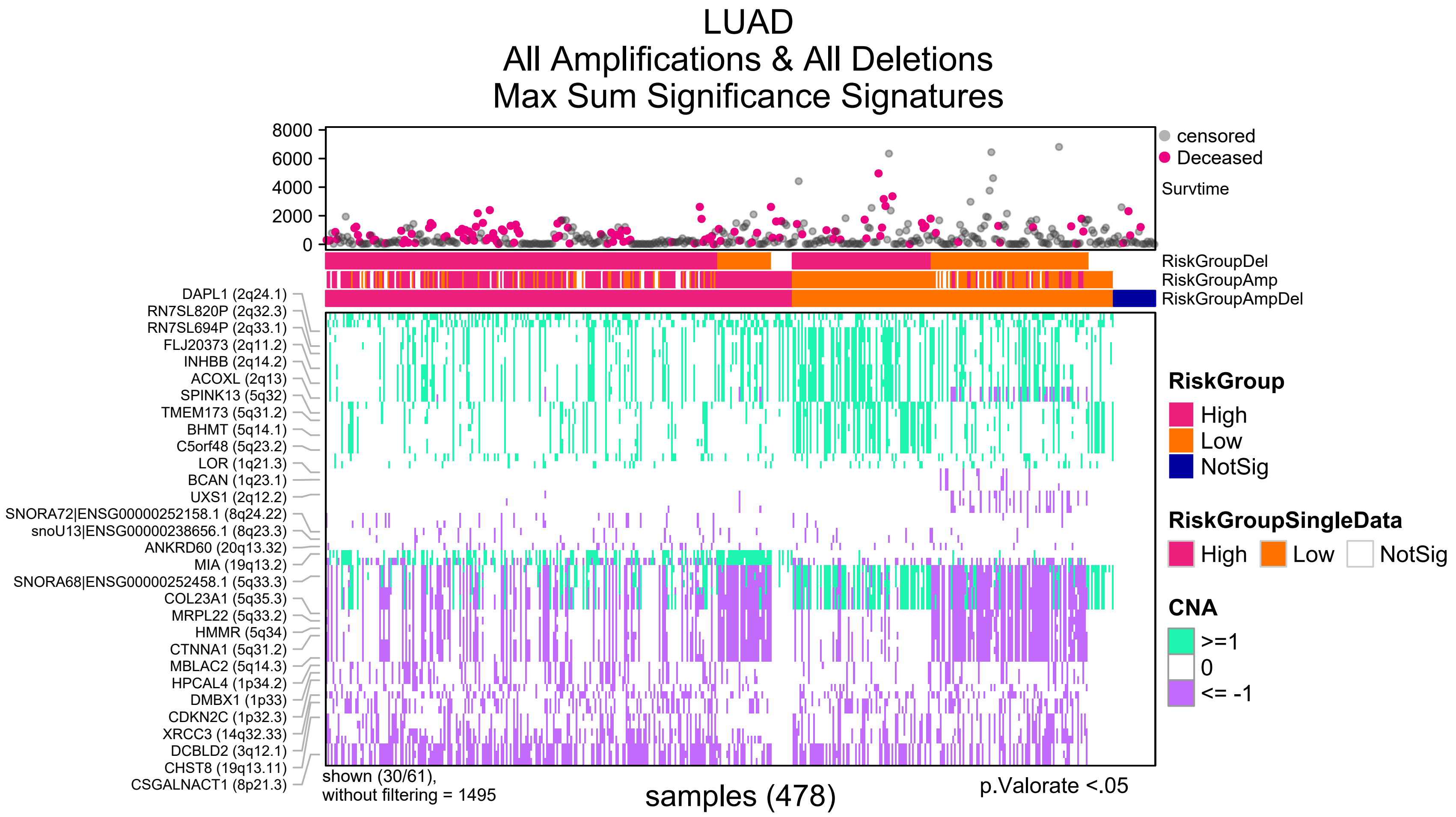

LUAD
All Amplifications & All Deletions
Max Sum Significance Signatures
8000
6000
4000
2000
0
censored
Deceased
Survtime
RiskGroupDel
RiskGroupAmp
RiskGroupAmpDel
DAPL1 (2q24.1)
RN7SL820P (2q32.3)
RN7SL694P (2q33.1)
FLJ20373 (2q11.2)
INHBB (2q14.2)
ACOXL (2q13)
SPINK13 (5q32)
TMEM173 (5q31.2)
BHMT (5q14.1)
C5orf48 (5q23.2)
LOR (1q21.3)
BCAN (1q23.1)
UXS1 (2q12.2)
SNORA72|ENSG00000252158.1 (8q24.22)
snoU13|ENSG00000238656.1 (8q23.3)
ANKRD60 (20q13.32)
MIA (19q13.2)
SNORA68|ENSG00000252458.1 (5q33.3)
COL23A1 (5q35.3)
MRPL22 (5q33.2)
HMMR (5q34)
CTNNA1 (5q31.2)
MBLAC2 (5q14.3)
HPCAL4 (1p34.2)
DMBX1 (1p33)
CDKN2C (1p32.3)
XRCC3 (14q32.33)
DCBLD2 (3q12.1)
CHST8 (19q13.11)
CSGALNACT1 (8p21.3)
RiskGroup
High
Low
NotSig
RiskGroupSingleData
High
Low
NotSig
CNA
>=1
0
<= -1
shown (30/61),
without filtering = 1495
samples (478)
p.Valorate <.05

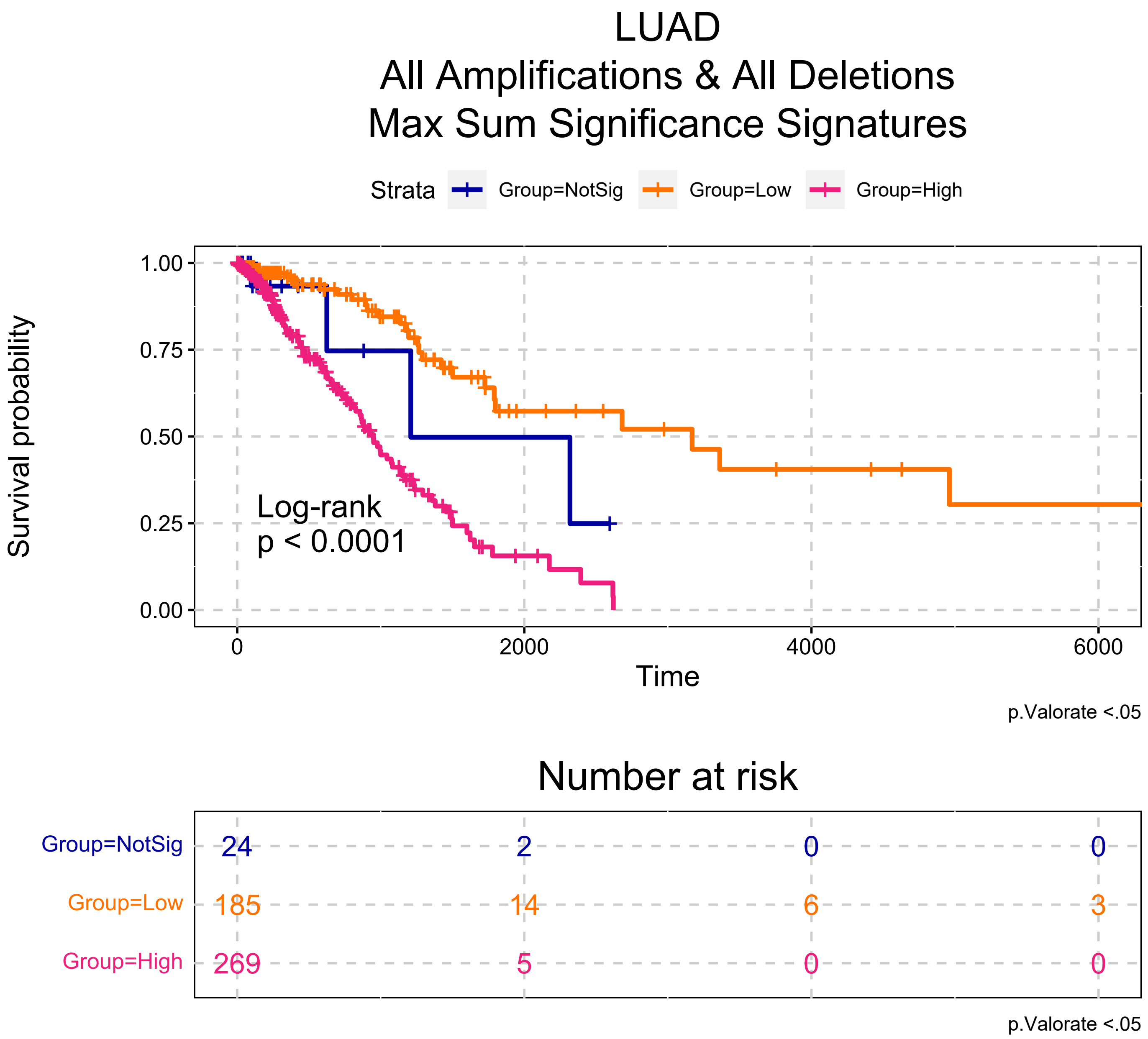

explanatory	beta	HR	L95	U95	p
Low	-0.78	0.46	0.16	1.33	0.15
High	0.70	2.02	0.74	5.53	0.17

n= 478, number of events =122
Score(logrank) test = p <.0001

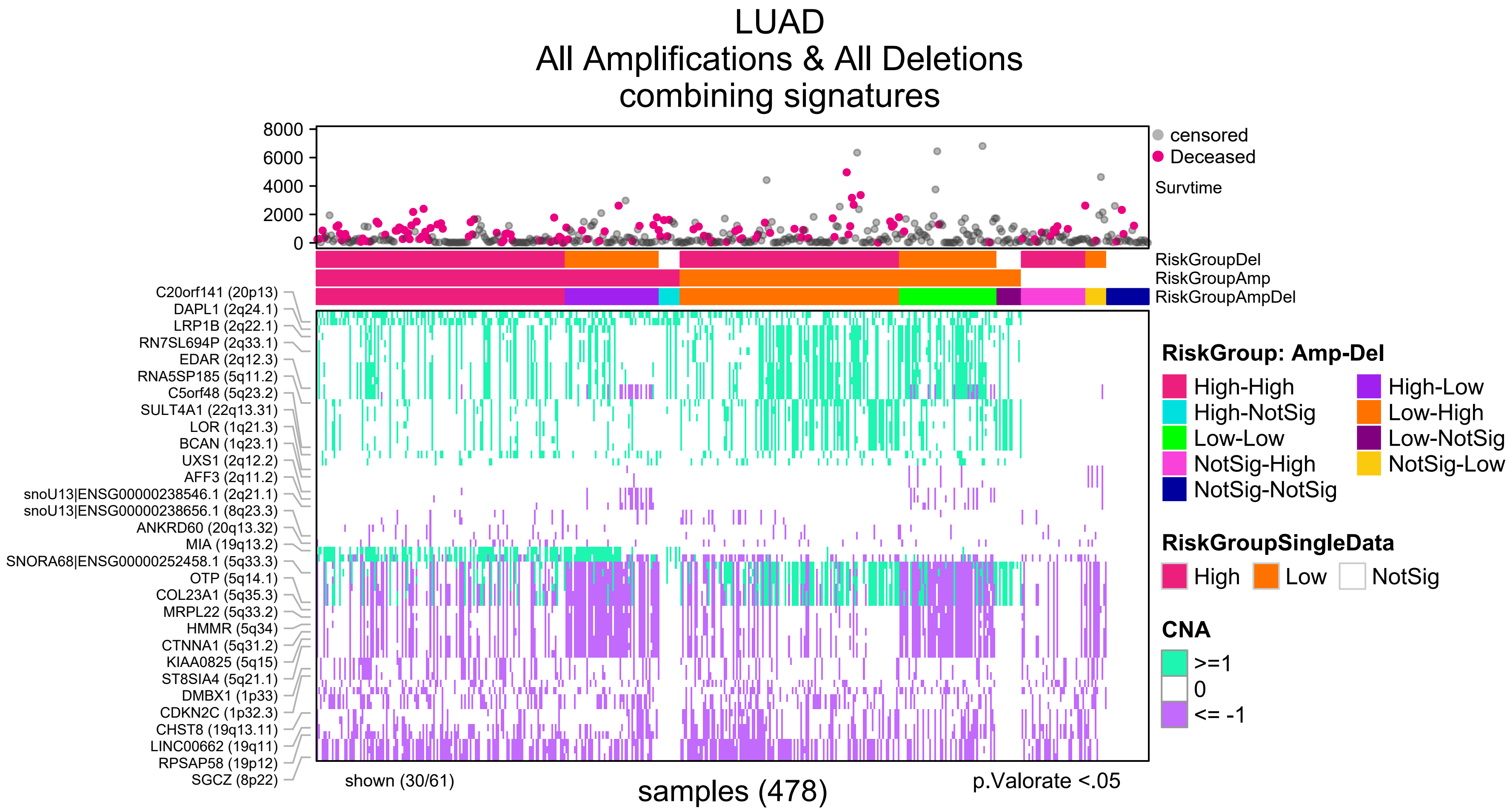

LUAD
All Amplifications & All Deletions
combining signatures
8000
6000
4000
2000
0
censored
Deceased
Survtime
RiskGroupDel
RiskGroupAmp
RiskGroupAmpDel
C20orf141 (20p13)
DAPL1 (2q24.1)
LRP1B (2q22.1)
RN7SL694P (2q33.1)
EDAR (2q12.3)
RNA5SP185 (5q11.2)
C5orf48 (5q23.2)
SULT4A1 (22q13.31)
LOR (1q21.3)
BCAN (1q23.1)
UXS1 (2q12.2)
AFF3 (2q11.2)
snoU13|ENSG00000238546.1 (2q21.1)
snoU13|ENSG00000238656.1 (8q23.3)
ANKRD60 (20q13.32)
MIA (19q13.2)
SNORA68|ENSG00000252458.1 (5q33.3)
OTP (5q14.1)
COL23A1 (5q35.3)
MRPL22 (5q33.2)
HMMR (5q34)
CTNNA1 (5q31.2)
KIAA0825 (5q15)
ST8SIA4 (5q21.1)
DMBX1 (1p33)
CDKN2C (1p32.3)
CHST8 (19q13.11)
LINC00662 (19q11)
RPSAP58 (19p12)
SGCZ (8p22)
RiskGroup: Amp-Del
High-High
High-NotSig
Low-Low
NotSig-High
NotSig-NotSig
High-Low
Low-High
Low-NotSig
NotSig-Low
RiskGroupSingleData
High
Low
NotSig
CNA
>=1
0
<= -1
shown (30/61)
samples (478)
p.Valorate <.05

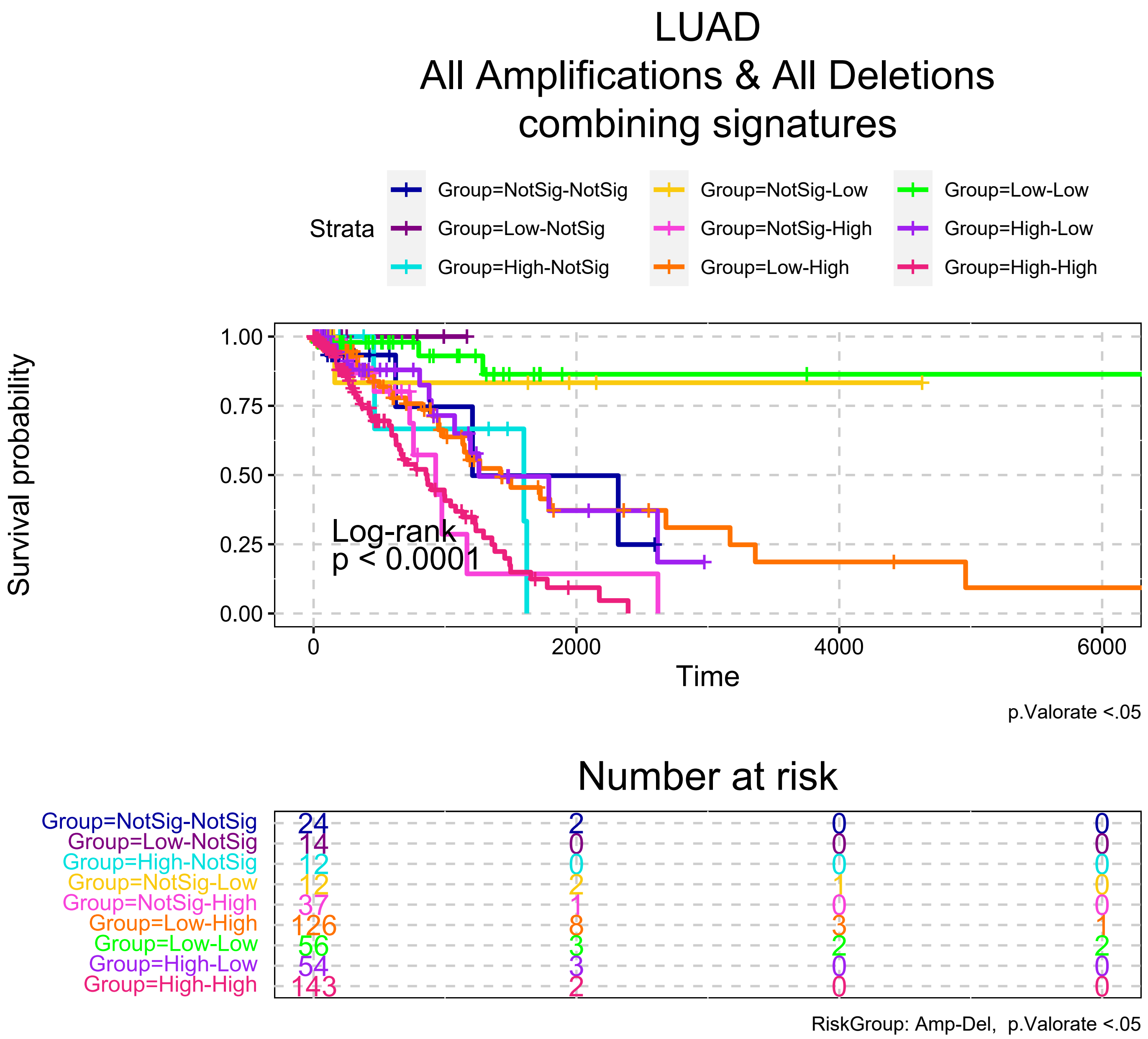

explanatory	beta	HR	L95	U95	p
Low-NotSig	-16.05	0.00	0.00	Inf	0.99
High-NotSig	0.16	1.17	0.29	4.70	0.83
NotSig-Low	-1.59	0.20	0.02	1.84	0.16
NotSig-High	0.57	1.78	0.55	5.70	0.33
Low-High	-0.03	0.97	0.34	2.77	0.95
Low-Low	-1.89	0.15	0.03	0.68	0.01
High-Low	-0.01	0.99	0.32	3.07	0.98
High-High	0.87	2.38	0.86	6.59	0.10

n= 478, number of events =122
Score(logrank) test = p <.0001

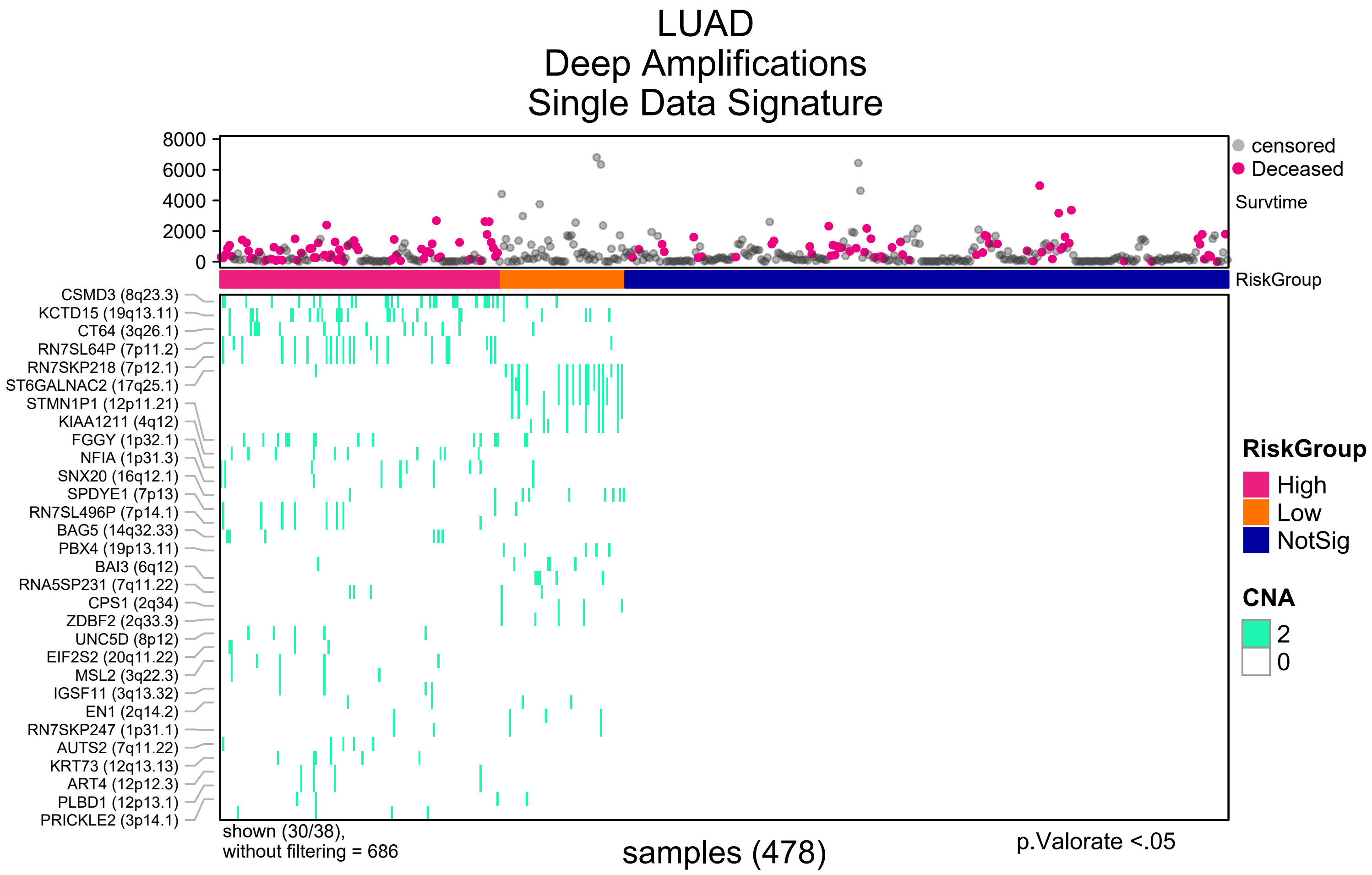
LUAD
Deep Amplifications
Single Data Signature
8000
6000
4000
2000
0
censored
Deceased
Survtime
RiskGroup
CSMD3 (8q23.3)
KCTD15 (19q13.11)
CT64 (3q26.1)
RN7SL64P (7p11.2)
RN7SKP218 (7p12.1)
ST6GALNAC2 (17q25.1)
STMN1P1 (12p11.21)
KIAA1211 (4q12)
FGGY (1p32.1)
NFIA (1p31.3)
SNX20 (16q12.1)
SPDYE1 (7p13)
RN7SL496P (7p14.1)
BAG5 (14q32.33)
PBX4 (19p13.11)
BAI3 (6q12)
RNA5SP231 (7q11.22)
CPS1 (2q34)
ZDBF2 (2q33.3)
UNC5D (8p12)
EIF2S2 (20q11.22)
MSL2 (3q22.3)
IGSF11 (3q13.32)
EN1 (2q14.2)
RN7SKP247 (1p31.1)
AUTS2 (7q11.22)
KRT73 (12q13.13)
ART4 (12p12.3)
PLBD1 (12p13.1)
PRICKLE2 (3p14.1)
RiskGroup
High
Low
NotSig
CNA
2
0
shown (30/38),
without filtering = 686
samples (478)
p.Valorate <.05

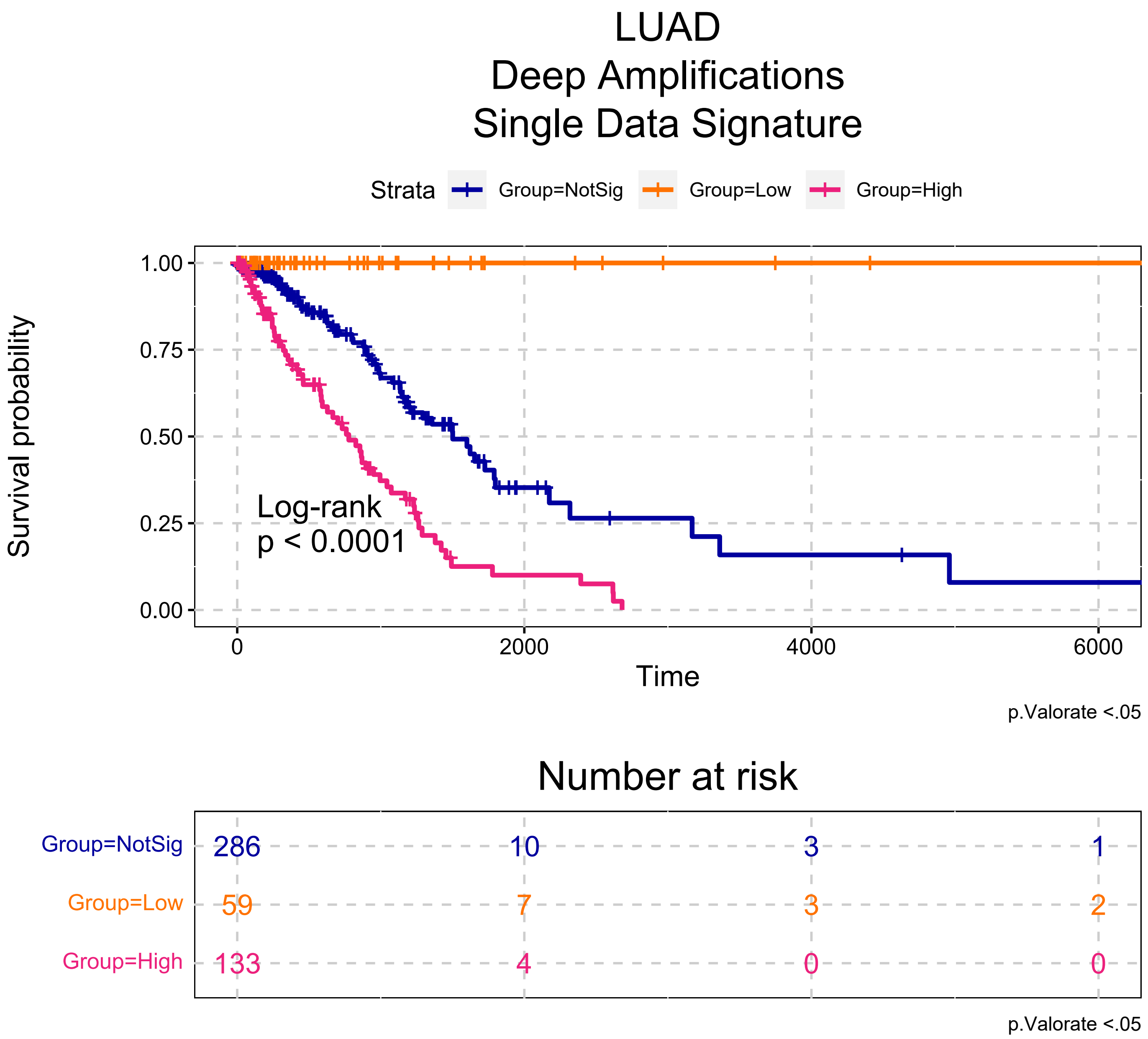

explanatory	beta	HR	L95	U95	p
Low	-18.26	0.00	0.00	Inf	0.99
High	1.00	2.71	1.89	3.90	0.00

n= 478, number of events =122
Score(logrank) test = p <.0001

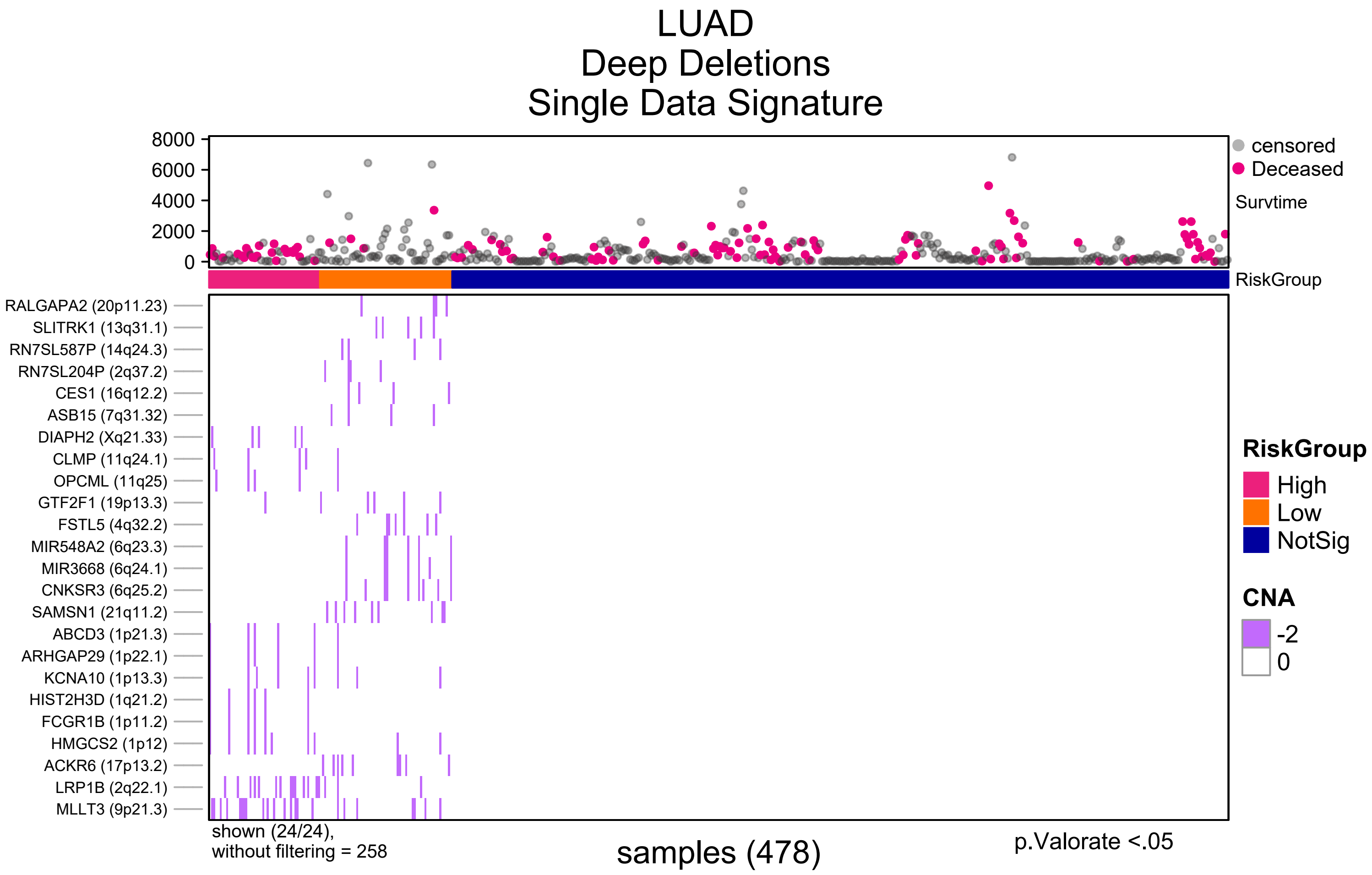

LUAD
Deep Deletions
Single Data Signature
8000
6000
4000
2000
0
censored
Deceased
Survtime
RiskGroup
RALGAPA2 (20p11.23)
SLITRK1 (13q31.1)
RN7SL587P (14q24.3)
RN7SL204P (2q37.2)
CES1 (16q12.2)
ASB15 (7q31.32)
DIAPH2 (Xq21.33)
CLMP (11q24.1)
OPCML (11q25)
GTF2F1 (19p13.3)
FSTL5 (4q32.2)
MIR548A2 (6q23.3)
MIR3668 (6q24.1)
CNKSR3 (6q25.2)
SAMSN1 (21q11.2)
ABCD3 (1p21.3)
ARHGAP29 (1p22.1)
KCNA10 (1p13.3)
HIST2H3D (1q21.2)
FCGR1B (1p11.2)
HMGCS2 (1p12)
ACKR6 (17p13.2)
LRP1B (2q22.1)
MLLT3 (9p21.3)
RiskGroup
High
Low
NotSig
CNA
-2
0
shown (24/24),
without filtering = 258
samples (478)
p.Valorate <.05

LUAD
Deep Deletions
Single Data Signature

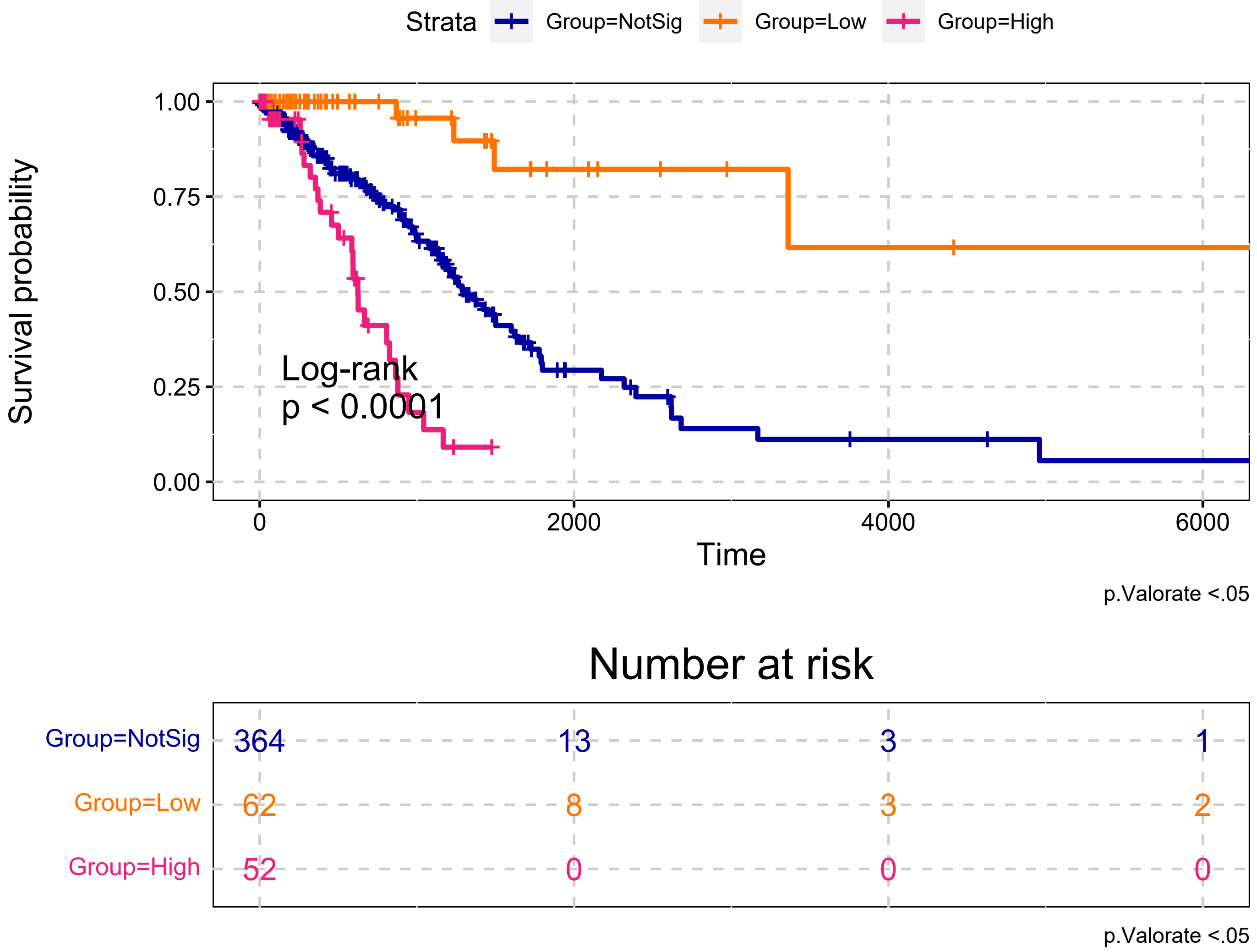

explanatory	beta	HR	L95	U95	p
Low	-1.99	0.14	0.05	0.37	0.00
High	1.08	2.93	1.84	4.66	0.00

n= 478, number of events =122
Score(logrank) test = p <.0001

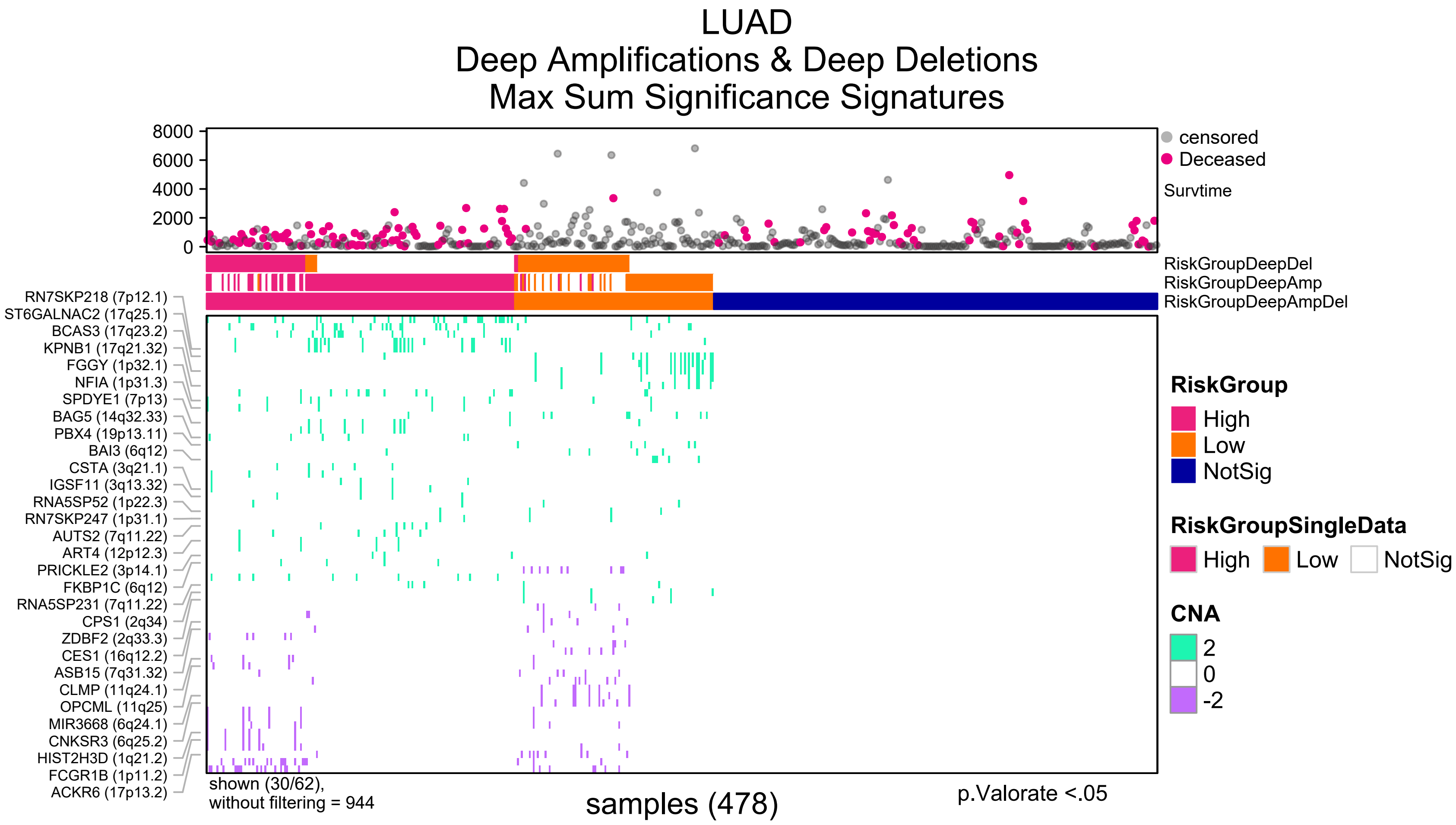
LUAD
Deep Amplifications & Deep Deletions
Max Sum Significance Signatures
8000
6000
4000
2000
0
censored
Deceased
Survtime
RiskGroupDeepDel
RiskGroupDeepAmp
RiskGroupDeepAmpDel
RN7SKP218 (7p12.1)
ST6GALNAC2 (17q25.1)
BCAS3 (17q23.2)
KPNB1 (17q21.32)
FGGY (1p32.1)
NFIA (1p31.3)
SPDYE1 (7p13)
BAG5 (14q32.33)
PBX4 (19p13.11)
BAI3 (6q12)
CSTA (3q21.1)
IGSF11 (3q13.32)
RNA5SP52 (1p22.3)
RN7SKP247 (1p31.1)
AUTS2 (7q11.22)
ART4 (12p12.3)
PRICKLE2 (3p14.1)
FKBP1C (6q12)
RNA5SP231 (7q11.22)
CPS1 (2q34)
ZDBF2 (2q33.3)
CES1 (16q12.2)
ASB15 (7q31.32)
CLMP (11q24.1)
OPCML (11q25)
MIR3668 (6q24.1)
CNKSR3 (6q25.2)
HIST2H3D (1q21.2)
FCGR1B (1p11.2)
ACKR6 (17p13.2)
RiskGroup
High
Low
NotSig
RiskGroupSingleData
High
Low
NotSig
CNA
2
0
-2
shown (30/62),
without filtering = 944
samples (478)
p.Valorate <.05

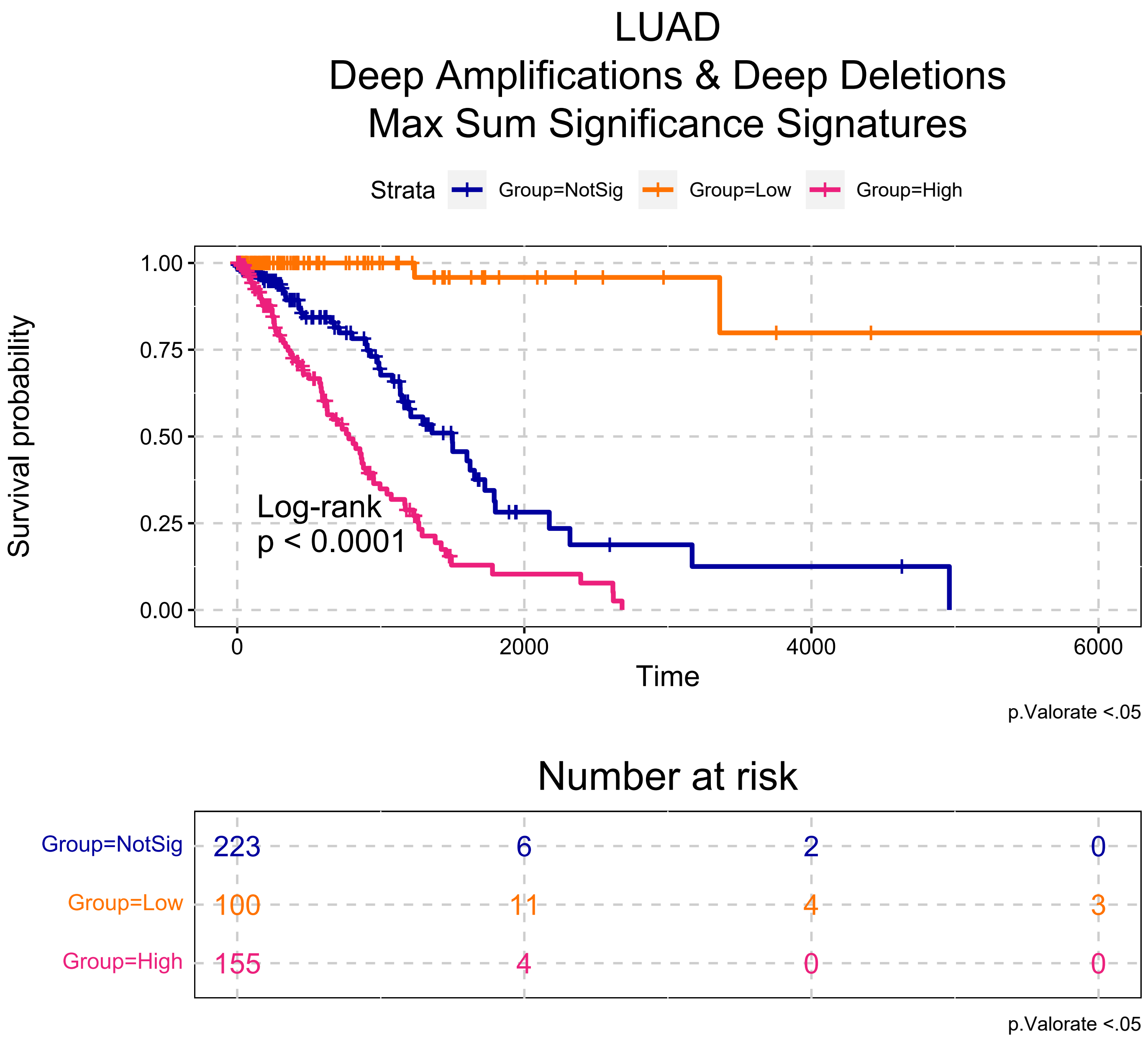

explanatory	beta	HR	L95	U95	p
Low	-3.05	0.05	0.01	0.20	0.00
High	0.90	2.46	1.69	3.59	0.00

n= 478, number of events =122
Score(logrank) test = p <.0001

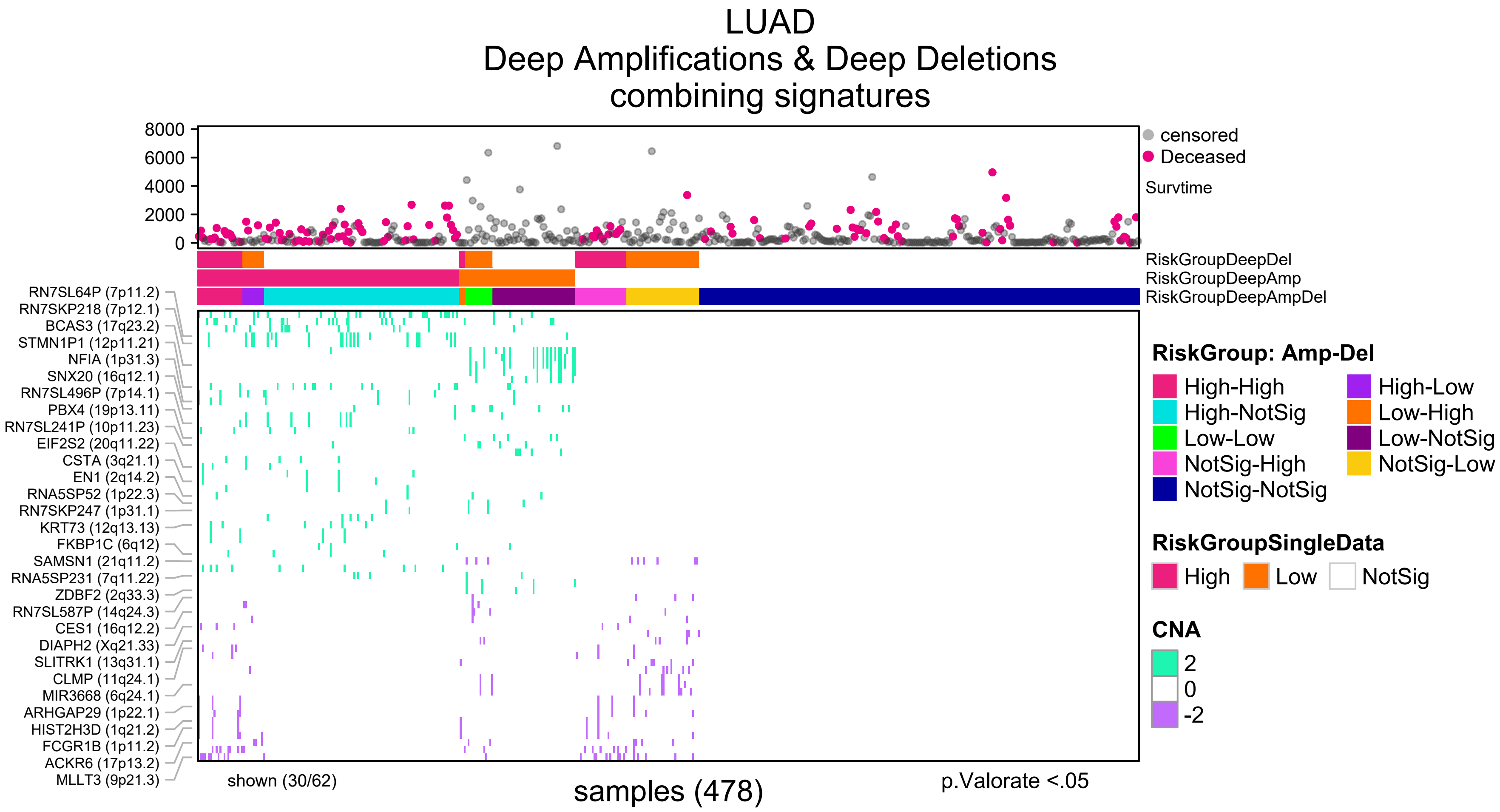

LUAD
Deep Amplifications & Deep Deletions
combining signatures
8000
6000
4000
2000
0
censored
Deceased
Survtime
RiskGroupDeepDel
RiskGroupDeepAmp
RiskGroupDeepAmpDel
RN7SL64P (7p11.2)
RN7SKP218 (7p12.1)
BCAS3 (17q23.2)
STMN1P1 (12p11.21)
NFIA (1p31.3)
SNX20 (16q12.1)
RN7SL496P (7p14.1)
PBX4 (19p13.11)
RN7SL241P (10p11.23)
EIF2S2 (20q11.22)
CSTA (3q21.1)
EN1 (2q14.2)
RNA5SP52 (1p22.3)
RN7SKP247 (1p31.1)
KRT73 (12q13.13)
FKBP1C (6q12)
SAMSN1 (21q11.2)
RNA5SP231 (7q11.22)
ZDBF2 (2q33.3)
RN7SL587P (14q24.3)
CES1 (16q12.2)
DIAPH2 (Xq21.33)
SLITRK1 (13q31.1)
CLMP (11q24.1)
MIR3668 (6q24.1)
ARHGAP29 (1p22.1)
HIST2H3D (1q21.2)
FCGR1B (1p11.2)
ACKR6 (17p13.2)
MLLT3 (9p21.3)
RiskGroup: Amp-Del
High-High
High-NotSig
Low-Low
NotSig-High
NotSig-NotSig
High-Low
Low-High
Low-NotSig
NotSig-Low
RiskGroupSingleData
High
Low
NotSig
CNA
2
0
-2
shown (30/62)
samples (478)
p.Valorate <.05

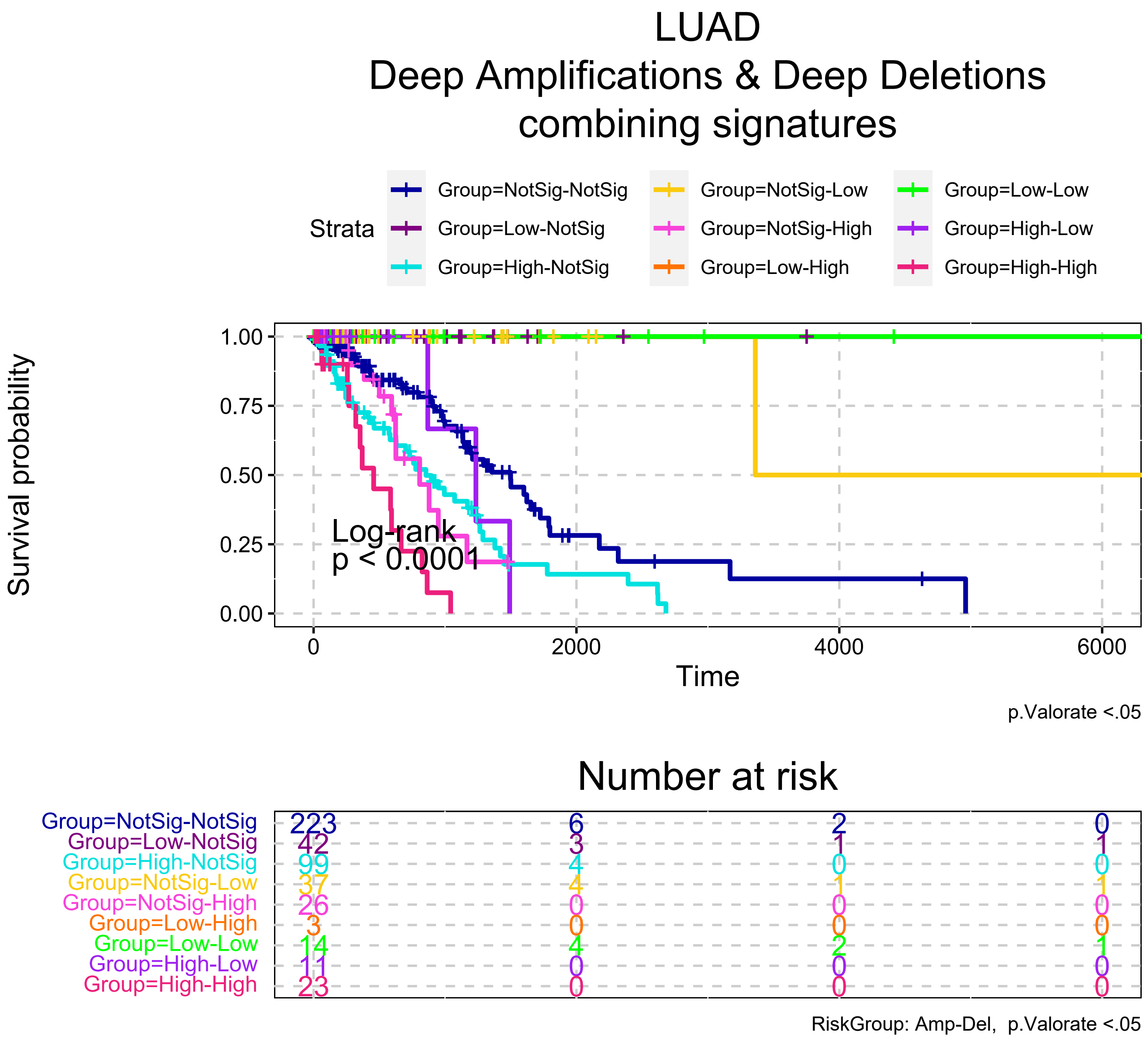

explanatory	beta	HR	L95	U95	p
Low-NotSig	-18.76	0.00	0.00	Inf	1.00
High-NotSig	0.78	2.19	1.45	3.31	0.00
NotSig-Low	-2.74	0.06	0.01	0.47	0.01
NotSig-High	0.84	2.31	1.18	4.53	0.01
Low-High	NA	NA	NA	NA	NA
Low-Low	-18.80	0.00	0.00	Inf	1.00
High-Low	0.43	1.53	0.47	4.96	0.48
High-High	1.83	6.22	3.32	11.65	0.00

n= 478, number of events =122
Score(logrank) test = p <.0001
